# Supplementary material for: mRNA Multipeptide-HLA Class II Immunotherapy for Melanoma
Source: Cells. 2025 Sep 12;14(18):1430. doi: 10.3390/cells14181430 (PMC12469133; doi:10.3390/cells14181430)
Supplement: Supplementary file 1 [file cells-14-01430-s001.zip › cells-3834690-supplementary.pdf]

Table S1. Amino acid sequences of the 15 melanoma antigens analyzed (Table 1).

Protein ID: O75767 (TRP2)
MSPLWWGFLLSCLGCKILPGAQGQFPRVCMTVDSLVNKECCPRLGAESANVCGSQQGRGQ
CTEVRADTRPWSGPYILRNQDDRELWPRKFFHRTCKCTGNFAGYNCGDCKFGWTGPNCER
KKPPVIRQNIHSLSPQEREQFLGALDLAKKRVHPDYVITTQHWVGLLGPNGTQPQFANCS
VYDFFVWLHYYSVRDTLLGGFFPWLKVYYYRFVIGLRVWQWEVISCKLIKRATTRQP

Protein ID: P04271 (S100)
MSELEKAMVALIDVFHQYSGREGDKHKLKKSELKELINNELSHFLEEIKEQEVVDKVMET
LDNDGDGECDFQEFMAFVAMVTTACHEFFEHE

Protein ID: P0DTW1 (GAGE1)
MSWRGRSTYYWPRPRRYVQPPEMIGPMRPEQFSDEVEPATPEEGEPATQRQDPAAAQEGE
DEGASAGQGPKPEADSQEQGHPQTGCECEDGPDGQEMDPPNPEEVKTPEEGEGQSQC

Protein ID: P14679 (Tyrosinase)
MLLAVLYCLLWSFQTSAGHFPRACVSSKNLMEKECCPPWSGDRSPCGQLSGRGSCQNILL
SNAPLGPQFPFTGVDDRESWPSVFYNRTCQCSGNFMGFNCGNCKFGFWGPNCTERRLLVR
RNIFDLSAPEKDKFFAYLTLAKHTISSDYVIPIGTYGQMKNGSTPMFNDINIYDLFVWMH
YYVSMDALLGGSEIWRDIDFAHEAPAFLPWHRLFLLRWEQEIQKLTGDENFTIPYWDWRD
AEKCDICTDEYMGGQHPTNPNLLSPASFFSSWQIVCSRLEEYNSHQSLCNGTPEGPLRRN
PGNHDKSRTPRLPSSADVEFCLSLTQYESGSMDKAANFSFRNTLEGFASPLTGIADASQS
SMHNALHIYMNGTMSQVQGSANDPIFLLHHAFVDSIFEQWLRRHRPLQEVYPEANAPIGH
NRESYMVPFIPLYRNGDFFISSKDLGYDYSYLQDSDPDSFQDYIKSYLEQASRIWSWLLG
AAMVGAVLTALLAGLVSLLCRHKRKQLPEEKQPLLMEKEDYHSLYQSHL

Protein ID: 17643 (TRP1)
MSAPKLLSLGCIFFPLLLFQQARAQFPRQCATVEALRSGMCCPDLSPVSGPGTDRCGSSS
GRGRCEAVTADSRPHSPQYPHDGRDDREVWPLRFFNRTCHCNGNFSGHNCGTCRPGWRGA
ACDQRVLIVRRNLLDLSKEEKNHFVRALDMAKRTTHPLFVIATRRSEEILGPDGNTPQFE
NISIYNYFVWTHYYSVKKTFLGVGQESFGEVDFSHEGPAFLTWHRYHLLRLEKDMQEMLQ
EPSFSLPYWNFATGKNVCDICTDDLMGSRSNFDSTLISPNSVFSQWRVVCDSLEDYDTLG
TLCNSTEDGPIRRNPAGNVARPMVQRLPEPQDVAQCLEVGLFDTPPFYSNSTNSFRNTVE
GYSDPTGKYDPAVRSLHNLAHLFLNGTGGQTHLSPNDPIFVLLHTFTDAVFDEWLRRYNA
DISTFPLENAPIGHNRQYNMVPFWPPVTNTEMFVTAPDNLGYTYEIQWPSREFSVPEIIA
IAVVGALLLVALIFGTASYLIRARRSMDEANQPLLTDQYQCYAEEYEKLQNPNQSVV

Protein ID: P40967 (PMEL17/gd100)
MDLVLKRCLLHLAVIGALLAVGATKVPRNQDWLGVSRQLRTKAWNRQLYPEWTEAQRLDC
WRGGQVSLKVSNDGPTLIGANASFSIALNFPGSQKVLPDGQVIWVNNTIINGSQVWGGQP
VYPQETDDACIFPDGGPCPSGSWSQKRSFVYVWKTWGQYWQVLGGPVSGLSIGTGRAMLG
THTMEVTVYHRRGSRSYVPLAHSSSAFTITDQVPFSVSVSQLRALDGGNKHFLRNQPLTF
ALQLHDPSGYLAEADLSYTWDFGDSSGTLISRALVVTHTYLEPGPVTAQVVLQAAIPLTS
CGSSPVPGTTDGHRPTAEAPNTTAGQVPTTEVVGTTPGQAPTAEPSGTTSVQVPTTEVIS
TAPVQMPTAESTGMTPEKVPVSEVMGTTLAEMSTPEATGMTPAEVSIVVLSGTTAAQVTT
TEWVETTARELPIPEPEGPDASSIMSTESITGSLGPLLDGTATLRLVKRQVPLDCVLYRY
GSFSVTLDIVQGIESAEILQAVPSGEGDAFELTVSCQGGLPKEACMEISSPGCQPPAQRL
CQPVLPSPACQLVLHQILKGGSGTYCLNVSLADTNSLAVVSTQLIMPGQEAGLGQVPLIV
GILLVLMAVVLASLIYRRRLMKQDFSVPQLPHSSSHWLRLPRIFCSCPIGENSPLLSGQQ

Protein ID: P43355 (MAGE1)
MSLEQRSLHCKPEEALEAQQEALGLVCVQAATSSSSPLVLGTLEEVPTAGSTDPPQSPQG
ASAFPTTINFTRQRQPSEGSSSREEEGPSTSCILESLFRAVITKKVADLVGFLLLKYRAR
EPVTKAEMLESVIKNYKHCFPEIFGKASESLQLVFGIDVKEADPTGHSYVLVTCLGLSYD
GLLGDNQIMPKTGFLIIVLVMIAMEGGHAPEEEIWEELSVMEVYDGREHSAYGEPRKLLT
QDLVQEKYLEYRQVPDSDPARYEFLWGPRALAETSYVKVLEYVIKVSARVRFFFPSLREA
ALREEEEGV

Protein ID: P43358 (MAGE4)
MSSEQKSQHCKPEEGVEAQEEALGLVGAQAPTTEEQEAAVSSSSPLVPGTLEEVPAAESA
GPPQSPQGASALPTTISFTCWRQPNEGSSSQEEEGPSTSPDAESLFREALSNKVDELAHF
LLRKYRAKELVTKAEMLERVIKNYKRCFPVIFGKASESLKMIFGIDVKEVDPASNTYTLV
TCLGLSYDGLLGNNQIFPKTGLLIIVLGTIAMEGDSASEEEIWEELGVMGVYDGREHTVY
GEPRKLLTQDWVQENYLEYRQVPGSNPARYEFLWGPRALAETSYVKVLEHVVRVNARVRI
AYPSLREAALLEEEEGV

Protein ID: P78358 (NY-ESO-1)
MQAEGRGTGGSTGDADGPGGPGIPDGPGGNAGGPGEAGATGGRGPRGAGAARASGPGGGA
PRGPHGGAASGLNGCCRCGARGPESRLLEFYLAMPFATPMEAELARRSLAQDAPPLPVPG
VLLKEFTVSGNILTIRLTAADHRQLQLSISSCLQQLSLLMWITQCFLPVFLAQPPSGQRR

Protein ID: Q13066 (GAGE2B)
MSWRGRSTYRPRPRRYVEPPEMIGPMRPEQFSDEVEPATPEEGEPATQRQDPAAAQEGED
EGASAGQGPKPEAHSQEQGHPQTGCECEDGPDGQEMDPPNPEEVKTPEEGEKQSQC

Protein ID: Q13072 (BAGE)
MAARAVFLALSAQLLQARLMKEESPVVSWRLEPEDGTALCFIF

Protein ID: Q16385 (SSX2)
MNGDDAFARRPTVGAQIPEKIQKAFDDIAKYFSKEEWEKMKASEKIFYVYMKRKYEAMTK
LGFKATLPPFMCNKRAEDFQGNDLDNDPNRGNQVERPQMTFGRLQGISPKIMPKKPAEEG
NDSEEVPEASGPQNDGKELCPPGKPTTSEKIHERSGPKRGEHAWTHRLRERKQLVIYEEI
SDPEEDDE

Protein ID: Q16655 (MELAN-A/MART-1)
MPREDAHFIYGYPKKGHGHSYTTAEEAAGIGILTVILGVLLLIGCWYCRRRNGYRALMDK
SLHVGTQCALTRRCPQEGFDHRDSKVSLQEKNCEPVVPNAPPAYEKLSAEQSPPPYSP

Protein ID: Q6NT46 (GAGE2A)
MSWRGRSTYRPRPRRYVEPPEMIGPMRPEQFSDEVEPATPEEGEPATQRQDPAAAQEGQD
EGASAGQGPKPEAHSQEQGHPQTGCECEDGPDGQEMDPPNPEEVKTPEEGEKQSQC

Protein ID: Q9UEU5 (GAGE 2D)
MSWRGRSTYRPRPRRYVEPPEMIGPMRPEQFSDEVEPATPEEGEPATQRQDPAAAQEGED
EGASAGQGPKPEADSQEQGHPQTGCECEDGPDGQEMDPPNPEEVKTPEEGEKQSQC

Table S2. The 192 HLA-II alleles used.

	DPB1 gene		DQB1 gene		DRB1 gene
1	DPB1*01:01	1	DQB1*02:02	1	DRB1*01:01
2	DPB1*02:01	2	DQB1*03:01	2	DRB1*01:02
3	DPB1*02:02	3	DQB1*03:02	3	DRB1*01:03
4	DPB1*03:01	4	DQB1*03:03	4	DRB1*01:11
5	DPB1*04:01	5	DQB1*03:04	5	DRB1*01:18
6	DPB1*04:02	6	DQB1*03:05	6	DRB1*01:20
7	DPB1*05:01	7	DQB1*03:10	7	DRB1*01:24
8	DPB1*06:01	8	DQB1*03:14	8	DRB1*01:29
9	DPB1*09:01	9	DQB1*03:17	9	DRB1*03:01
10	DPB1*10:01	10	DQB1*03:19	10	DRB1*03:02
11	DPB1*104:01	11	DQB1*03:23	11	DRB1*03:04
12	DPB1*105:01	12	DQB1*03:25	12	DRB1*03:05
13	DPB1*11:01	13	DQB1*04:01	13	DRB1*03:11
14	DPB1*124:01	14	DQB1*04:02	14	DRB1*03:13
15	DPB1*126:01	15	DQB1*04:03	15	DRB1*03:15
16	DPB1*13:01	16	DQB1*05:01	16	DRB1*03:41
17	DPB1*14:01	17	DQB1*05:02	17	DRB1*04:01
18	DPB1*15:01	18	DQB1*05:03	18	DRB1*04:02
19	DPB1*16:01	19	DQB1*05:06	19	DRB1*04:03
20	DPB1*17:01	20	DQB1*05:11	20	DRB1*04:04
21	DPB1*19:01	21	DQB1*06:01	21	DRB1*04:05
22	DPB1*20:01	22	DQB1*06:02	22	DRB1*04:06
23	DPB1*23:01	23	DQB1*06:03	23	DRB1*04:07
24	DPB1*26:01	24	DQB1*06:04	24	DRB1*04:08
25	DPB1*28:01	25	DQB1*06:07	25	DRB1*04:10
26	DPB1*30:01	26	DQB1*06:08	26	DRB1*04:11
27	DPB1*33:01	27	DQB1*06:09	27	DRB1*04:17
28	DPB1*34:01	28	DQB1*06:11	28	DRB1*04:44
29	DPB1*35:01	29	DQB1*06:14	29	DRB1*04:53
30	DPB1*39:01	30	DQB1*06:15	30	DRB1*04:56
31	DPB1*40:01	31	DQB1*06:18	31	DRB1*04:72
32	DPB1*41:01	32	DQB1*06:19	32	DRB1*07:01
33	DPB1*46:01	33	DQB1*06:22	33	DRB1*08:01
34	DPB1*47:01	34	DQB1*06:27	34	DRB1*08:02
35	DPB1*49:01	35	DQB1*06:32	35	DRB1*08:03
36	DPB1*55:01			36	DRB1*08:04

37	DPB1*71:01		37	DRB1*08:24
38	DPB1*72:01		38	DRB1*08:30
39	DPB1*81:01		39	DRB1*08:36
40	DPB1*85:01		40	DRB1*09:01
41	DPB1*91:01		41	DRB1*09:02
			42	DRB1*10:01
			43	DRB1*11:01
			44	DRB1*11:02
			45	DRB1*11:03
			46	DRB1*11:04
			47	DRB1*11:06
			48	DRB1*11:07
			49	DRB1*11:08
			50	DRB1*11:10
			51	DRB1*11:11
			52	DRB1*11:12
			53	DRB1*11:13
			54	DRB1*11:14
			55	DRB1*11:19
			56	DRB1*11:27
			57	DRB1*11:28
			58	DRB1*11:29
			59	DRB1*11:37
			60	DRB1*11:42
			61	DRB1*11:46
			62	DRB1*11:49
			63	DRB1*11:54
			64	DRB1*11:58
			65	DRB1*11:62
			66	DRB1*11:65
			67	DRB1*11:74
			68	DRB1*11:84
			69	DRB1*12:01
			70	DRB1*12:02
			71	DRB1*12:03
			72	DRB1*12:16
			73	DRB1*13:01
			74	DRB1*13:02
			75	DRB1*13:03
			76	DRB1*13:05

		77	DRB1*13:07
		78	DRB1*13:11
		79	DRB1*13:12
		80	DRB1*13:14
		81	DRB1*13:21
		82	DRB1*13:23
		83	DRB1*13:26
		84	DRB1*13:33
		85	DRB1*13:50
		86	DRB1*13:61
		87	DRB1*13:66
		88	DRB1*13:96
		89	DRB1*13:97
		90	DRB1*14:01
		91	DRB1*14:02
		92	DRB1*14:03
		93	DRB1*14:04
		94	DRB1*14:05
		95	DRB1*14:06
		96	DRB1*14:07
		97	DRB1*14:12
		98	DRB1*14:23
		99	DRB1*14:27
		100	DRB1*14:32
		101	DRB1*14:38
		102	DRB1*14:44
		103	DRB1*14:54
		104	DRB1*14:68
		105	DRB1*15:01
		106	DRB1*15:02
		107	DRB1*15:03
		108	DRB1*15:06
		109	DRB1*15:07
		110	DRB1*15:15
		111	DRB1*15:37
		112	DRB1*16:01
		113	DRB1*16:02
		114	DRB1*16:04
		115	DRB1*16:05
		116	DRB1*16:09

Table S3. All estimated strong binding affinities (IC_{50} < 50 nM) for 15-mer sequences for the 117 HLA-II alleles with strong binding and the 11/15 antigens that had any strong affinity. Start, End denote the location of the peptide in the protein sequence tested. Four antigens (GAGE1, GAGE2B/C, GAGE2A, GAGE2D) did not have any strong affinity (Table 1) and are not shown in this table.

Index	Protein	Antigen	Allele	Start	End	Peptide	ic50
1	O75767	TRP2	DRB1*01:18	206	220	KVYYYRFVIGLRVWQ	5.31
2	O75767	TRP2	DRB1*01:18	208	222	YYYRFVIGLRVWQWE	5.78
3	O75767	TRP2	DRB1*01:18	207	221	VYYYRFVIGLRVWQW	6.24
4	O75767	TRP2	DRB1*01:24	206	220	KVYYYRFVIGLRVWQ	6.44
5	O75767	TRP2	DRB1*01:24	208	222	YYYRFVIGLRVWQWE	6.88
6	O75767	TRP2	DPB1*33:01	181	195	VYDFFVWLHYYSVRD	6.90
7	O75767	TRP2	DPB1*71:01	181	195	VYDFFVWLHYYSVRD	6.90
8	O75767	TRP2	DRB1*01:18	205	219	LKVYYYRFVIGLRVW	6.90
9	O75767	TRP2	DRB1*01:01	206	220	KVYYYRFVIGLRVWQ	6.91
10	O75767	TRP2	DPB1*33:01	182	196	YDFFVWLHYYSVRDT	7.06
11	O75767	TRP2	DPB1*71:01	182	196	YDFFVWLHYYSVRDT	7.06
12	O75767	TRP2	DRB1*01:18	209	223	YYRFVIGLRVWQWEV	7.14
13	O75767	TRP2	DRB1*01:24	207	221	VYYYRFVIGLRVWQW	7.21
14	O75767	TRP2	DRB1*01:18	204	218	WLKVYYYRFVIGLRV	7.51
15	O75767	TRP2	DRB1*01:01	208	222	YYYRFVIGLRVWQWE	7.56
16	O75767	TRP2	DPB1*33:01	180	194	SVYDFFVWLHYYSVR	7.64
17	O75767	TRP2	DPB1*71:01	180	194	SVYDFFVWLHYYSVR	7.64
18	O75767	TRP2	DRB1*01:24	205	219	LKVYYYRFVIGLRVW	8.17
19	O75767	TRP2	DPB1*33:01	183	197	DFFVWLHYYSVRDTL	8.37
20	O75767	TRP2	DPB1*71:01	183	197	DFFVWLHYYSVRDTL	8.37
21	O75767	TRP2	DRB1*01:01	207	221	VYYYRFVIGLRVWQW	8.56
22	O75767	TRP2	DRB1*01:01	209	223	YYRFVIGLRVWQWEV	9.39
23	O75767	TRP2	DRB1*07:01	204	218	WLKVYYYRFVIGLRV	9.46
24	O75767	TRP2	DRB1*01:24	209	223	YYRFVIGLRVWQWEV	9.55
25	O75767	TRP2	DRB1*01:29	206	220	KVYYYRFVIGLRVWQ	9.64
26	O75767	TRP2	DRB1*01:11	206	220	KVYYYRFVIGLRVWQ	9.65
27	O75767	TRP2	DRB1*01:01	205	219	LKVYYYRFVIGLRVW	9.70
28	O75767	TRP2	DRB1*01:01	204	218	WLKVYYYRFVIGLRV	9.93
29	O75767	TRP2	DRB1*01:24	204	218	WLKVYYYRFVIGLRV	10.06
30	O75767	TRP2	DRB1*01:29	208	222	YYYRFVIGLRVWQWE	10.94
31	O75767	TRP2	DRB1*01:11	207	221	VYYYRFVIGLRVWQW	11.19
32	O75767	TRP2	DRB1*01:11	208	222	YYYRFVIGLRVWQWE	11.36
33	O75767	TRP2	DRB1*01:29	207	221	VYYYRFVIGLRVWQW	11.62
34	O75767	TRP2	DPB1*33:01	179	193	CSVYDFFVWLHYYSV	12.36
35	O75767	TRP2	DPB1*71:01	179	193	CSVYDFFVWLHYYSV	12.36

36	O75767	TRP2	DRB1*07:01	206	220	KVYYYRFVIGLRVWQ	12.59
37	O75767	TRP2	DPB1*33:01	184	198	FFVWLHYYSVRDTLL	12.95
38	O75767	TRP2	DPB1*71:01	184	198	FFVWLHYYSVRDTLL	12.95
39	O75767	TRP2	DRB1*01:11	205	219	LKVYYYRFVIGLRVW	13.59
40	O75767	TRP2	DPB1*02:01	181	195	VYDFFVWLHYYSVRD	13.62
41	O75767	TRP2	DPB1*46:01	181	195	VYDFFVWLHYYSVRD	13.62
42	O75767	TRP2	DPB1*81:01	181	195	VYDFFVWLHYYSVRD	13.62
43	O75767	TRP2	DPB1*02:01	182	196	YDFFVWLHYYSVRDT	13.64
44	O75767	TRP2	DPB1*46:01	182	196	YDFFVWLHYYSVRDT	13.64
45	O75767	TRP2	DPB1*81:01	182	196	YDFFVWLHYYSVRDT	13.64
46	O75767	TRP2	DPB1*02:02	181	195	VYDFFVWLHYYSVRD	14.25
47	O75767	TRP2	DPB1*47:01	181	195	VYDFFVWLHYYSVRD	14.25
48	O75767	TRP2	DRB1*01:29	205	219	LKVYYYRFVIGLRVW	14.70
49	O75767	TRP2	DRB1*01:29	209	223	YYRFVIGLRVWQWEV	14.91
50	O75767	TRP2	DPB1*02:02	182	196	YDFFVWLHYYSVRDT	15.00
51	O75767	TRP2	DPB1*47:01	182	196	YDFFVWLHYYSVRDT	15.00
52	O75767	TRP2	DPB1*02:01	180	194	SVYDFFVWLHYYSVR	15.36
53	O75767	TRP2	DPB1*46:01	180	194	SVYDFFVWLHYYSVR	15.36
54	O75767	TRP2	DPB1*81:01	180	194	SVYDFFVWLHYYSVR	15.36
55	O75767	TRP2	DRB1*07:01	205	219	LKVYYYRFVIGLRVW	15.43
56	O75767	TRP2	DRB1*07:01	208	222	YYYRFVIGLRVWQWE	15.65
57	O75767	TRP2	DPB1*02:02	180	194	SVYDFFVWLHYYSVR	15.68
58	O75767	TRP2	DPB1*47:01	180	194	SVYDFFVWLHYYSVR	15.68
59	O75767	TRP2	DRB1*01:29	204	218	WLKVYYYRFVIGLRV	16.28
60	O75767	TRP2	DRB1*01:11	209	223	YYRFVIGLRVWQWEV	16.56
61	O75767	TRP2	DPB1*02:01	183	197	DFFVWLHYYSVRDTL	16.72
62	O75767	TRP2	DPB1*46:01	183	197	DFFVWLHYYSVRDTL	16.72
63	O75767	TRP2	DPB1*81:01	183	197	DFFVWLHYYSVRDTL	16.72
64	O75767	TRP2	DRB1*01:11	204	218	WLKVYYYRFVIGLRV	16.89
65	O75767	TRP2	DPB1*04:01	181	195	VYDFFVWLHYYSVRD	17.12
66	O75767	TRP2	DPB1*126:01	181	195	VYDFFVWLHYYSVRD	17.12
67	O75767	TRP2	DPB1*23:01	181	195	VYDFFVWLHYYSVRD	17.12
68	O75767	TRP2	DPB1*39:01	181	195	VYDFFVWLHYYSVRD	17.12
69	O75767	TRP2	DRB1*07:01	207	221	VYYYRFVIGLRVWQW	17.44
70	O75767	TRP2	DPB1*02:02	183	197	DFFVWLHYYSVRDTL	17.81
71	O75767	TRP2	DPB1*47:01	183	197	DFFVWLHYYSVRDTL	17.81
72	O75767	TRP2	DPB1*04:01	182	196	YDFFVWLHYYSVRDT	18.02
73	O75767	TRP2	DPB1*126:01	182	196	YDFFVWLHYYSVRDT	18.02
74	O75767	TRP2	DPB1*23:01	182	196	YDFFVWLHYYSVRDT	18.02
75	O75767	TRP2	DPB1*39:01	182	196	YDFFVWLHYYSVRDT	18.02
76	O75767	TRP2	DRB1*10:01	206	220	KVYYYRFVIGLRVWQ	18.44
77	O75767	TRP2	DPB1*04:01	180	194	SVYDFFVWLHYYSVR	19.26
78	O75767	TRP2	DPB1*126:01	180	194	SVYDFFVWLHYYSVR	19.26

79	O75767	TRP2	DPB1*23:01	180	194	SVYDFFVWLHYYSVR	19.26
80	O75767	TRP2	DPB1*39:01	180	194	SVYDFFVWLHYYSVR	19.26
81	O75767	TRP2	DRB1*11:04	222	236	EVISCKLIKRATTRQ	19.29
82	O75767	TRP2	DRB1*11:46	222	236	EVISCKLIKRATTRQ	19.29
83	O75767	TRP2	DRB1*11:58	222	236	EVISCKLIKRATTRQ	19.29
84	O75767	TRP2	DRB1*13:11	222	236	EVISCKLIKRATTRQ	19.29
85	O75767	TRP2	DRB1*16:09	206	220	KVYYYRFVIGLRVWQ	19.39
86	O75767	TRP2	DPB1*33:01	194	208	RDTLLGGFFPWLKVY	19.47
87	O75767	TRP2	DPB1*71:01	194	208	RDTLLGGFFPWLKVY	19.47
88	O75767	TRP2	DRB1*11:04	223	237	VISCKLIKRATTRQP	20.64
89	O75767	TRP2	DRB1*11:46	223	237	VISCKLIKRATTRQP	20.64
90	O75767	TRP2	DRB1*11:58	223	237	VISCKLIKRATTRQP	20.64
91	O75767	TRP2	DRB1*13:11	223	237	VISCKLIKRATTRQP	20.64
92	O75767	TRP2	DRB1*07:01	209	223	YYRFVIGLRVWQWEV	21.28
93	O75767	TRP2	DRB1*01:18	210	224	YRFVIGLRVWQWEVI	21.41
94	O75767	TRP2	DRB1*01:01	159	173	TTQHWVGLLGPNGTQ	21.60
95	O75767	TRP2	DRB1*10:01	208	222	YYYRFVIGLRVWQWE	21.78
96	O75767	TRP2	DPB1*33:01	193	207	VRDTLLGGFFPWLKV	22.03
97	O75767	TRP2	DPB1*71:01	193	207	VRDTLLGGFFPWLKV	22.03
98	O75767	TRP2	DRB1*11:04	221	235	WEVISCKLIKRATTR	22.06
99	O75767	TRP2	DRB1*11:46	221	235	WEVISCKLIKRATTR	22.06
100	O75767	TRP2	DRB1*11:58	221	235	WEVISCKLIKRATTR	22.06
101	O75767	TRP2	DRB1*13:11	221	235	WEVISCKLIKRATTR	22.06
102	O75767	TRP2	DRB1*16:05	206	220	KVYYYRFVIGLRVWQ	22.24
103	O75767	TRP2	DRB1*11:42	222	236	EVISCKLIKRATTRQ	22.36
104	O75767	TRP2	DRB1*16:02	206	220	KVYYYRFVIGLRVWQ	22.86
105	O75767	TRP2	DRB1*16:09	208	222	YYYRFVIGLRVWQWE	22.89
106	O75767	TRP2	DPB1*72:01	181	195	VYDFFVWLHYYSVRD	22.94
107	O75767	TRP2	DPB1*72:01	182	196	YDFFVWLHYYSVRDT	23.67
108	O75767	TRP2	DRB1*11:42	221	235	WEVISCKLIKRATTR	23.84
109	O75767	TRP2	DRB1*01:01	160	174	TQHWVGLLGPNGTQP	23.93
110	O75767	TRP2	DRB1*15:02	206	220	KVYYYRFVIGLRVWQ	24.09
111	O75767	TRP2	DRB1*10:01	207	221	VYYYRFVIGLRVWQW	24.36
112	O75767	TRP2	DRB1*01:20	208	222	YYYRFVIGLRVWQWE	24.68
113	O75767	TRP2	DRB1*11:42	223	237	VISCKLIKRATTRQP	25.20
114	O75767	TRP2	DPB1*04:01	183	197	DFFVWLHYYSVRDTL	25.56
115	O75767	TRP2	DPB1*126:01	183	197	DFFVWLHYYSVRDTL	25.56
116	O75767	TRP2	DPB1*23:01	183	197	DFFVWLHYYSVRDTL	25.56
117	O75767	TRP2	DPB1*39:01	183	197	DFFVWLHYYSVRDTL	25.56
118	O75767	TRP2	DRB1*16:09	207	221	VYYYRFVIGLRVWQW	25.57
119	O75767	TRP2	DRB1*10:01	204	218	WLKVYYYRFVIGLRV	25.68
120	O75767	TRP2	DRB1*01:20	206	220	KVYYYRFVIGLRVWQ	25.87
121	O75767	TRP2	DRB1*11:03	222	236	EVISCKLIKRATTRQ	25.88

122	O75767	TRP2	DRB1*16:05	207	221	VYYYRFVIGLRVWQW	26.40
123	O75767	TRP2	DRB1*16:09	204	218	WLKVYYYRFVIGLRV	26.84
124	O75767	TRP2	DRB1*10:01	205	219	LKVYYYRFVIGLRVW	26.89
125	O75767	TRP2	DRB1*01:18	182	196	YDFFVWLHYYSVRDT	27.59
126	O75767	TRP2	DRB1*11:03	221	235	WEVISCKLIKRATTR	27.69
127	O75767	TRP2	DPB1*33:01	211	225	RFVIGLRVWQWEVIS	27.72
128	O75767	TRP2	DPB1*71:01	211	225	RFVIGLRVWQWEVIS	27.72
129	O75767	TRP2	DRB1*16:09	205	219	LKVYYYRFVIGLRVW	27.73
130	O75767	TRP2	DRB1*01:18	159	173	TTQHWVGLLGPNGTQ	27.90
131	O75767	TRP2	DRB1*03:11	61	75	CTEVRADTRPWSGPY	28.01
132	O75767	TRP2	DRB1*16:05	208	222	YYYRFVIGLRVWQWE	28.04
133	O75767	TRP2	DPB1*02:01	184	198	FFVWLHYYSVRDTLL	28.16
134	O75767	TRP2	DPB1*46:01	184	198	FFVWLHYYSVRDTLL	28.16
135	O75767	TRP2	DPB1*81:01	184	198	FFVWLHYYSVRDTLL	28.16
136	O75767	TRP2	DRB1*03:11	62	76	TEVRADTRPWSGPYI	28.24
137	O75767	TRP2	DPB1*33:01	213	227	VIGLRVWQWEVISCK	28.38
138	O75767	TRP2	DPB1*71:01	213	227	VIGLRVWQWEVISCK	28.38
139	O75767	TRP2	DPB1*72:01	180	194	SVYDFFVWLHYYSVR	28.48
140	O75767	TRP2	DPB1*02:02	184	198	FFVWLHYYSVRDTLL	28.60
141	O75767	TRP2	DPB1*47:01	184	198	FFVWLHYYSVRDTLL	28.60
142	O75767	TRP2	DRB1*01:01	210	224	YRFVIGLRVWQWEVI	28.72
143	O75767	TRP2	DRB1*16:02	208	222	YYYRFVIGLRVWQWE	28.76
144	O75767	TRP2	DPB1*02:02	179	193	CSVYDFFVWLHYYSV	28.85
145	O75767	TRP2	DPB1*47:01	179	193	CSVYDFFVWLHYYSV	28.85
146	O75767	TRP2	DRB1*16:05	205	219	LKVYYYRFVIGLRVW	28.85
147	O75767	TRP2	DRB1*01:01	158	172	ITTQHWVGLLGPNGT	28.99
148	O75767	TRP2	DRB1*11:03	223	237	VISCKLIKRATTRQP	29.49
149	O75767	TRP2	DRB1*16:01	206	220	KVYYYRFVIGLRVWQ	29.62
150	O75767	TRP2	DRB1*15:02	207	221	VYYYRFVIGLRVWQW	29.64
151	O75767	TRP2	DRB1*16:02	207	221	VYYYRFVIGLRVWQW	29.70
152	O75767	TRP2	DPB1*33:01	195	209	DTLLGGFFPWLKVYY	29.79
153	O75767	TRP2	DPB1*71:01	195	209	DTLLGGFFPWLKVYY	29.79
154	O75767	TRP2	DRB1*01:20	207	221	VYYYRFVIGLRVWQW	29.94
155	O75767	TRP2	DRB1*15:15	206	220	KVYYYRFVIGLRVWQ	30.51
156	O75767	TRP2	DRB1*10:01	209	223	YYRFVIGLRVWQWEV	30.56
157	O75767	TRP2	DRB1*11:04	142	156	LGALDLAKKRVHPDY	30.57
158	O75767	TRP2	DRB1*11:46	142	156	LGALDLAKKRVHPDY	30.57
159	O75767	TRP2	DRB1*11:58	142	156	LGALDLAKKRVHPDY	30.57
160	O75767	TRP2	DRB1*13:11	142	156	LGALDLAKKRVHPDY	30.57
161	O75767	TRP2	DRB1*15:02	205	219	LKVYYYRFVIGLRVW	30.65
162	O75767	TRP2	DRB1*11:01	222	236	EVISCKLIKRATTRQ	30.92
163	O75767	TRP2	DRB1*11:10	222	236	EVISCKLIKRATTRQ	30.92
164	O75767	TRP2	DRB1*11:12	222	236	EVISCKLIKRATTRQ	30.92

165	O75767	TRP2	DRB1*11:28	222	236	EVISCKLIKRATTRQ	30.92
166	O75767	TRP2	DRB1*11:29	222	236	EVISCKLIKRATTRQ	30.92
167	O75767	TRP2	DRB1*11:49	222	236	EVISCKLIKRATTRQ	30.92
168	O75767	TRP2	DRB1*11:62	222	236	EVISCKLIKRATTRQ	30.92
169	O75767	TRP2	DRB1*11:74	222	236	EVISCKLIKRATTRQ	30.92
170	O75767	TRP2	DRB1*13:05	222	236	EVISCKLIKRATTRQ	30.92
171	O75767	TRP2	DRB1*13:14	222	236	EVISCKLIKRATTRQ	30.92
172	O75767	TRP2	DRB1*13:50	222	236	EVISCKLIKRATTRQ	30.92
173	O75767	TRP2	DPB1*33:01	210	224	YRFVIGLRVWQWEVI	30.93
174	O75767	TRP2	DPB1*71:01	210	224	YRFVIGLRVWQWEVI	30.93
175	O75767	TRP2	DRB1*15:02	208	222	YYYRFVIGLRVWQWE	30.94
176	O75767	TRP2	DRB1*16:02	204	218	WLKVYYYRFVIGLRV	31.11
177	O75767	TRP2	DRB1*01:01	161	175	QHWVGLLGPNGTQPQ	31.19
178	O75767	TRP2	DRB1*11:42	220	234	QWEVISCKLIKRATT	31.20
179	O75767	TRP2	DRB1*01:20	209	223	YYRFVIGLRVWQWEV	31.31
180	O75767	TRP2	DPB1*04:01	179	193	CSVYDFFVWLHYYSV	31.35
181	O75767	TRP2	DPB1*126:01	179	193	CSVYDFFVWLHYYSV	31.35
182	O75767	TRP2	DPB1*23:01	179	193	CSVYDFFVWLHYYSV	31.35
183	O75767	TRP2	DPB1*39:01	179	193	CSVYDFFVWLHYYSV	31.35
184	O75767	TRP2	DRB1*01:18	160	174	TQHWVGLLGPNGTQP	31.38
185	O75767	TRP2	DPB1*15:01	181	195	VYDFFVWLHYYSVRD	31.55
186	O75767	TRP2	DPB1*72:01	183	197	DFFVWLHYYSVRDTL	31.85
187	O75767	TRP2	DPB1*15:01	182	196	YDFFVWLHYYSVRDT	31.94
188	O75767	TRP2	DRB1*16:09	209	223	YYRFVIGLRVWQWEV	31.95
189	O75767	TRP2	DRB1*01:18	181	195	VYDFFVWLHYYSVRD	32.05
190	O75767	TRP2	DRB1*16:05	204	218	WLKVYYYRFVIGLRV	32.26
191	O75767	TRP2	DRB1*03:11	59	73	GQCTEVRADTRPWSG	32.62
192	O75767	TRP2	DRB1*16:02	205	219	LKVYYYRFVIGLRVW	32.79
193	O75767	TRP2	DPB1*02:01	179	193	CSVYDFFVWLHYYSV	33.00
194	O75767	TRP2	DPB1*46:01	179	193	CSVYDFFVWLHYYSV	33.00
195	O75767	TRP2	DPB1*81:01	179	193	CSVYDFFVWLHYYSV	33.00
196	O75767	TRP2	DRB1*01:18	183	197	DFFVWLHYYSVRDTL	33.13
197	O75767	TRP2	DRB1*03:11	60	74	QCTEVRADTRPWSGP	33.23
198	O75767	TRP2	DRB1*01:24	210	224	YRFVIGLRVWQWEVI	33.28
199	O75767	TRP2	DRB1*11:01	223	237	VISCKLIKRATTRQP	33.86
200	O75767	TRP2	DRB1*11:10	223	237	VISCKLIKRATTRQP	33.86
201	O75767	TRP2	DRB1*11:12	223	237	VISCKLIKRATTRQP	33.86
202	O75767	TRP2	DRB1*11:28	223	237	VISCKLIKRATTRQP	33.86
203	O75767	TRP2	DRB1*11:29	223	237	VISCKLIKRATTRQP	33.86
204	O75767	TRP2	DRB1*11:49	223	237	VISCKLIKRATTRQP	33.86
205	O75767	TRP2	DRB1*11:62	223	237	VISCKLIKRATTRQP	33.86
206	O75767	TRP2	DRB1*11:74	223	237	VISCKLIKRATTRQP	33.86
207	O75767	TRP2	DRB1*13:05	223	237	VISCKLIKRATTRQP	33.86

208	O75767	TRP2	DRB1*13:14	223	237	VISCKLIKRATTRQP	33.86
209	O75767	TRP2	DRB1*13:50	223	237	VISCKLIKRATTRQP	33.86
210	O75767	TRP2	DRB1*15:02	204	218	WLKVYYYRFVIGLRV	33.96
211	O75767	TRP2	DPB1*33:01	214	228	IGLRVWQWEVISCKL	34.14
212	O75767	TRP2	DPB1*71:01	214	228	IGLRVWQWEVISCKL	34.14
213	O75767	TRP2	DRB1*15:01	184	198	FFVWLHYYSVRDTLL	35.04
214	O75767	TRP2	DRB1*15:06	184	198	FFVWLHYYSVRDTLL	35.04
215	O75767	TRP2	DRB1*11:04	140	154	QFLGALDLAKKRVHP	35.10
216	O75767	TRP2	DRB1*11:46	140	154	QFLGALDLAKKRVHP	35.10
217	O75767	TRP2	DRB1*11:58	140	154	QFLGALDLAKKRVHP	35.10
218	O75767	TRP2	DRB1*13:11	140	154	QFLGALDLAKKRVHP	35.10
219	O75767	TRP2	DPB1*33:01	192	206	SVRDTLLGGFFPWLK	35.17
220	O75767	TRP2	DPB1*71:01	192	206	SVRDTLLGGFFPWLK	35.17
221	O75767	TRP2	DRB1*01:18	184	198	FFVWLHYYSVRDTLL	35.64
222	O75767	TRP2	DRB1*01:18	187	201	WLHYYSVRDTLLGGF	35.91
223	O75767	TRP2	DPB1*15:01	180	194	SVYDFFVWLHYYSVR	36.05
224	O75767	TRP2	DRB1*01:01	187	201	WLHYYSVRDTLLGGF	36.30
225	O75767	TRP2	DRB1*11:04	220	234	QWEVISCKLIKRATT	36.31
226	O75767	TRP2	DRB1*11:46	220	234	QWEVISCKLIKRATT	36.31
227	O75767	TRP2	DRB1*11:58	220	234	QWEVISCKLIKRATT	36.31
228	O75767	TRP2	DRB1*13:11	220	234	QWEVISCKLIKRATT	36.31
229	O75767	TRP2	DRB1*08:04	222	236	EVISCKLIKRATTRQ	36.35
230	O75767	TRP2	DRB1*16:01	208	222	YYYRFVIGLRVWQWE	36.45
231	O75767	TRP2	DRB1*11:01	221	235	WEVISCKLIKRATTR	36.89
232	O75767	TRP2	DRB1*11:10	221	235	WEVISCKLIKRATTR	36.89
233	O75767	TRP2	DRB1*11:12	221	235	WEVISCKLIKRATTR	36.89
234	O75767	TRP2	DRB1*11:28	221	235	WEVISCKLIKRATTR	36.89
235	O75767	TRP2	DRB1*11:29	221	235	WEVISCKLIKRATTR	36.89
236	O75767	TRP2	DRB1*11:49	221	235	WEVISCKLIKRATTR	36.89
237	O75767	TRP2	DRB1*11:62	221	235	WEVISCKLIKRATTR	36.89
238	O75767	TRP2	DRB1*11:74	221	235	WEVISCKLIKRATTR	36.89
239	O75767	TRP2	DRB1*13:05	221	235	WEVISCKLIKRATTR	36.89
240	O75767	TRP2	DRB1*13:14	221	235	WEVISCKLIKRATTR	36.89
241	O75767	TRP2	DRB1*13:50	221	235	WEVISCKLIKRATTR	36.89
242	O75767	TRP2	DPB1*33:01	185	199	FVWLHYYSVRDTLLG	36.94
243	O75767	TRP2	DPB1*71:01	185	199	FVWLHYYSVRDTLLG	36.94
244	O75767	TRP2	DRB1*16:04	206	220	KVYYYRFVIGLRVWQ	37.18
245	O75767	TRP2	DRB1*11:03	142	156	LGALDLAKKRVHPDY	37.56
246	O75767	TRP2	DRB1*15:01	185	199	FVWLHYYSVRDTLLG	37.62
247	O75767	TRP2	DRB1*15:06	185	199	FVWLHYYSVRDTLLG	37.62
248	O75767	TRP2	DRB1*15:01	183	197	DFFVWLHYYSVRDTL	37.90
249	O75767	TRP2	DRB1*15:06	183	197	DFFVWLHYYSVRDTL	37.90
250	O75767	TRP2	DRB1*01:18	158	172	ITTQHWVGLLGPNGT	38.08

251	O75767	TRP2	DRB1*08:04	223	237	VISCKLIKRATTRQP	38.29
252	O75767	TRP2	DRB1*08:04	221	235	WEVISCKLIKRATTR	38.83
253	O75767	TRP2	DPB1*33:01	199	213	GGFFPWLKVYYYRFV	38.84
254	O75767	TRP2	DPB1*71:01	199	213	GGFFPWLKVYYYRFV	38.84
255	O75767	TRP2	DRB1*16:01	207	221	VYYYRFVIGLRVWQW	38.89
256	O75767	TRP2	DRB1*11:42	142	156	LGALDLAKKRVHPDY	38.96
257	O75767	TRP2	DRB1*11:02	221	235	WEVISCKLIKRATTR	39.15
258	O75767	TRP2	DRB1*11:65	221	235	WEVISCKLIKRATTR	39.15
259	O75767	TRP2	DRB1*13:01	221	235	WEVISCKLIKRATTR	39.15
260	O75767	TRP2	DRB1*01:18	180	194	SVYDFFVWLHYYSVR	39.51
261	O75767	TRP2	DRB1*11:01	88	102	RKFFHRTCKCTGNFA	39.55
262	O75767	TRP2	DRB1*11:10	88	102	RKFFHRTCKCTGNFA	39.55
263	O75767	TRP2	DRB1*11:12	88	102	RKFFHRTCKCTGNFA	39.55
264	O75767	TRP2	DRB1*11:28	88	102	RKFFHRTCKCTGNFA	39.55
265	O75767	TRP2	DRB1*11:29	88	102	RKFFHRTCKCTGNFA	39.55
266	O75767	TRP2	DRB1*11:49	88	102	RKFFHRTCKCTGNFA	39.55
267	O75767	TRP2	DRB1*11:62	88	102	RKFFHRTCKCTGNFA	39.55
268	O75767	TRP2	DRB1*11:74	88	102	RKFFHRTCKCTGNFA	39.55
269	O75767	TRP2	DRB1*13:05	88	102	RKFFHRTCKCTGNFA	39.55
270	O75767	TRP2	DRB1*13:14	88	102	RKFFHRTCKCTGNFA	39.55
271	O75767	TRP2	DRB1*13:50	88	102	RKFFHRTCKCTGNFA	39.55
272	O75767	TRP2	DRB1*11:04	141	155	FLGALDLAKKRVHPD	39.69
273	O75767	TRP2	DRB1*11:46	141	155	FLGALDLAKKRVHPD	39.69
274	O75767	TRP2	DRB1*11:58	141	155	FLGALDLAKKRVHPD	39.69
275	O75767	TRP2	DRB1*13:11	141	155	FLGALDLAKKRVHPD	39.69
276	O75767	TRP2	DRB1*11:03	220	234	QWEVISCKLIKRATT	39.71
277	O75767	TRP2	DRB1*11:02	222	236	EVISCKLIKRATTRQ	39.78
278	O75767	TRP2	DRB1*11:65	222	236	EVISCKLIKRATTRQ	39.78
279	O75767	TRP2	DRB1*13:01	222	236	EVISCKLIKRATTRQ	39.78
280	O75767	TRP2	DPB1*33:01	196	210	TLLGGFFPWLKVYYY	39.80
281	O75767	TRP2	DPB1*71:01	196	210	TLLGGFFPWLKVYYY	39.80
282	O75767	TRP2	DRB1*01:18	161	175	QHWVGLLGPNGTQPQ	40.04
283	O75767	TRP2	DRB1*11:03	140	154	QFLGALDLAKKRVHP	40.06
284	O75767	TRP2	DRB1*01:18	185	199	FVWLHYYSVRDTLLG	40.12
285	O75767	TRP2	DRB1*03:11	63	77	EVRADTRPWSGPYIL	40.17
286	O75767	TRP2	DRB1*01:20	204	218	WLKVYYYRFVIGLRV	40.86
287	O75767	TRP2	DRB1*15:15	204	218	WLKVYYYRFVIGLRV	40.86
288	O75767	TRP2	DPB1*33:01	204	218	WLKVYYYRFVIGLRV	41.22
289	O75767	TRP2	DPB1*71:01	204	218	WLKVYYYRFVIGLRV	41.22
290	O75767	TRP2	DRB1*15:15	208	222	YYYRFVIGLRVWQWE	41.32
291	O75767	TRP2	DRB1*01:20	205	219	LKVYYYRFVIGLRVW	41.36
292	O75767	TRP2	DRB1*11:42	140	154	QFLGALDLAKKRVHP	41.77
293	O75767	TRP2	DRB1*01:01	182	196	YDFFVWLHYYSVRDT	42.66

294	O75767	TRP2	DRB1*01:18	188	202	LHYYSVRDTLLGGFF	42.86
295	O75767	TRP2	DRB1*11:03	141	155	FLGALDLAKKRVHPD	43.01
296	O75767	TRP2	DRB1*15:15	205	219	LKVYYYRFVIGLRVW	43.07
297	O75767	TRP2	DRB1*15:15	207	221	VYYYRFVIGLRVWQW	43.09
298	O75767	TRP2	DPB1*33:01	198	212	LGGFFPWLKVYYYRF	43.46
299	O75767	TRP2	DPB1*71:01	198	212	LGGFFPWLKVYYYRF	43.46
300	O75767	TRP2	DRB1*16:04	208	222	YYYRFVIGLRVWQWE	43.50
301	O75767	TRP2	DRB1*01:18	186	200	VWLHYYSVRDTLLGG	43.62
302	O75767	TRP2	DPB1*33:01	212	226	FVIGLRVWQWEVISC	43.80
303	O75767	TRP2	DPB1*71:01	212	226	FVIGLRVWQWEVISC	43.80
304	O75767	TRP2	DPB1*33:01	201	215	FFPWLKVYYYRFVIG	43.88
305	O75767	TRP2	DPB1*71:01	201	215	FFPWLKVYYYRFVIG	43.88
306	O75767	TRP2	DPB1*15:01	183	197	DFFVWLHYYSVRDTL	44.02
307	O75767	TRP2	DRB1*01:01	188	202	LHYYSVRDTLLGGFF	44.37
308	O75767	TRP2	DRB1*01:18	138	152	REQFLGALDLAKKRV	44.68
309	O75767	TRP2	DRB1*11:13	221	235	WEVISCKLIKRATTR	44.69
310	O75767	TRP2	DPB1*33:01	203	217	PWLKVYYYRFVIGLR	44.82
311	O75767	TRP2	DPB1*71:01	203	217	PWLKVYYYRFVIGLR	44.82
312	O75767	TRP2	DPB1*33:01	206	220	KVYYYRFVIGLRVWQ	45.12
313	O75767	TRP2	DPB1*71:01	206	220	KVYYYRFVIGLRVWQ	45.12
314	O75767	TRP2	DRB1*16:01	205	219	LKVYYYRFVIGLRVW	45.20
315	O75767	TRP2	DRB1*16:01	204	218	WLKVYYYRFVIGLRV	45.54
316	O75767	TRP2	DRB1*16:02	209	223	YYRFVIGLRVWQWEV	45.76
317	O75767	TRP2	DRB1*10:01	159	173	TTQHWVGLLGPNGTQ	45.93
318	O75767	TRP2	DPB1*33:01	215	229	GLRVWQWEVISCKLI	45.96
319	O75767	TRP2	DPB1*71:01	215	229	GLRVWQWEVISCKLI	45.96
320	O75767	TRP2	DRB1*11:42	141	155	FLGALDLAKKRVHPD	45.97
321	O75767	TRP2	DRB1*11:13	222	236	EVISCKLIKRATTRQ	46.10
322	O75767	TRP2	DRB1*11:84	222	236	EVISCKLIKRATTRQ	46.41
323	O75767	TRP2	DRB1*01:01	186	200	VWLHYYSVRDTLLGG	46.44
324	O75767	TRP2	DRB1*16:05	209	223	YYRFVIGLRVWQWEV	46.50
325	O75767	TRP2	DRB1*01:01	185	199	FVWLHYYSVRDTLLG	46.81
326	O75767	TRP2	DRB1*01:18	137	151	EREQFLGALDLAKKR	46.86
327	O75767	TRP2	DRB1*11:42	219	233	WQWEVISCKLIKRAT	47.17
328	O75767	TRP2	DRB1*16:04	207	221	VYYYRFVIGLRVWQW	47.58
329	O75767	TRP2	DRB1*11:14	122	136	KPPVIRQNIHSLSPQ	47.66
330	O75767	TRP2	DRB1*13:02	122	136	KPPVIRQNIHSLSPQ	47.66
331	O75767	TRP2	DRB1*13:23	122	136	KPPVIRQNIHSLSPQ	47.66
332	O75767	TRP2	DRB1*13:97	122	136	KPPVIRQNIHSLSPQ	47.66
333	O75767	TRP2	DRB1*11:14	121	135	KKPPVIRQNIHSLSP	47.85
334	O75767	TRP2	DRB1*13:02	121	135	KKPPVIRQNIHSLSP	47.85
335	O75767	TRP2	DRB1*13:23	121	135	KKPPVIRQNIHSLSP	47.85
336	O75767	TRP2	DRB1*13:97	121	135	KKPPVIRQNIHSLSP	47.85

337	O75767	TRP2	DRB1*11:08	206	220	KVYYYRFVIGLRVWQ	48.02
338	O75767	TRP2	DRB1*11:02	223	237	VISCKLIKRATTRQP	48.12
339	O75767	TRP2	DRB1*11:65	223	237	VISCKLIKRATTRQP	48.12
340	O75767	TRP2	DRB1*13:01	223	237	VISCKLIKRATTRQP	48.12
341	O75767	TRP2	DRB1*15:15	183	197	DFFVWLHYYSVRDTL	48.79
342	O75767	TRP2	DRB1*11:14	120	134	RKKPPVIRQNIHSLS	48.91
343	O75767	TRP2	DRB1*13:02	120	134	RKKPPVIRQNIHSLS	48.91
344	O75767	TRP2	DRB1*13:23	120	134	RKKPPVIRQNIHSLS	48.91
345	O75767	TRP2	DRB1*13:97	120	134	RKKPPVIRQNIHSLS	48.91
346	O75767	TRP2	DRB1*14:06	221	235	WEVISCKLIKRATTR	48.94
347	O75767	TRP2	DRB1*01:18	136	150	QEREQFLGALDLAKK	49.00
348	O75767	TRP2	DRB1*03:11	58	72	RGQCTEVRADTRPWS	49.15
349	O75767	TRP2	DPB1*02:02	194	208	RDTLLGGFFPWLKVY	49.46
350	O75767	TRP2	DPB1*47:01	194	208	RDTLLGGFFPWLKVY	49.46
351	O75767	TRP2	DPB1*01:01	181	195	VYDFFVWLHYYSVRD	49.56
352	O75767	TRP2	DRB1*01:20	137	151	EREQFLGALDLAKKR	49.71
353	O75767	TRP2	DRB1*11:01	208	222	YYYRFVIGLRVWQWE	49.95
354	O75767	TRP2	DRB1*11:10	208	222	YYYRFVIGLRVWQWE	49.95
355	O75767	TRP2	DRB1*11:12	208	222	YYYRFVIGLRVWQWE	49.95
356	O75767	TRP2	DRB1*11:28	208	222	YYYRFVIGLRVWQWE	49.95
357	O75767	TRP2	DRB1*11:29	208	222	YYYRFVIGLRVWQWE	49.95
358	O75767	TRP2	DRB1*11:49	208	222	YYYRFVIGLRVWQWE	49.95
359	O75767	TRP2	DRB1*11:62	208	222	YYYRFVIGLRVWQWE	49.95
360	O75767	TRP2	DRB1*11:74	208	222	YYYRFVIGLRVWQWE	49.95
361	O75767	TRP2	DRB1*13:05	208	222	YYYRFVIGLRVWQWE	49.95
362	O75767	TRP2	DRB1*13:14	208	222	YYYRFVIGLRVWQWE	49.95
363	O75767	TRP2	DRB1*13:50	208	222	YYYRFVIGLRVWQWE	49.95
364	O75767	TRP2	DRB1*01:29	210	224	YRFVIGLRVWQWEVI	49.96
365	P04271	S100	DRB1*01:18	71	85	EFMAFVAMVTTACHE	20.92
366	P04271	S100	DRB1*01:18	70	84	QEFMAFVAMVTTACH	21.80
367	P04271	S100	DRB1*01:18	73	87	MAFVAMVTTACHEFF	22.14
368	P04271	S100	DRB1*01:18	68	82	DFQEFMAFVAMVTTA	22.40
369	P04271	S100	DRB1*01:18	72	86	FMAFVAMVTTACHEF	22.40
370	P04271	S100	DRB1*10:01	73	87	MAFVAMVTTACHEFF	22.80
371	P04271	S100	DRB1*01:20	28	42	KSELKELINNELSHF	23.27
372	P04271	S100	DRB1*01:01	71	85	EFMAFVAMVTTACHE	23.60
373	P04271	S100	DRB1*01:01	73	87	MAFVAMVTTACHEFF	24.36
374	P04271	S100	DRB1*10:01	72	86	FMAFVAMVTTACHEF	24.93
375	P04271	S100	DRB1*10:01	71	85	EFMAFVAMVTTACHE	25.83
376	P04271	S100	DRB1*01:01	72	86	FMAFVAMVTTACHEF	26.13
377	P04271	S100	DRB1*01:01	70	84	QEFMAFVAMVTTACH	27.21
378	P04271	S100	DRB1*01:18	28	42	KSELKELINNELSHF	27.66
379	P04271	S100	DRB1*04:04	3	17	EKAMVALIDVFHQYS	27.71

380	P04271	S100	DRB1*04:04	73	87	MAFVAMVTTACHEFF	28.91
381	P04271	S100	DRB1*10:01	74	88	AFVAMVTTACHEFFE	28.91
382	P04271	S100	DRB1*04:04	4	18	KAMVALIDVFHQYSG	29.93
383	P04271	S100	DRB1*01:18	67	81	CDFQEFMAFVAMVTT	30.35
384	P04271	S100	DRB1*01:18	74	88	AFVAMVTTACHEFFE	30.76
385	P04271	S100	DRB1*01:18	69	83	FQEFMAFVAMVTTAC	31.01
386	P04271	S100	DRB1*01:01	74	88	AFVAMVTTACHEFFE	31.14
387	P04271	S100	DRB1*10:01	70	84	QEFMAFVAMVTTACH	32.16
388	P04271	S100	DRB1*01:18	66	80	ECDFQEFMAFVAMVT	32.52
389	P04271	S100	DRB1*01:20	29	43	SELKELINNELSHFL	32.83
390	P04271	S100	DRB1*04:04	72	86	FMAFVAMVTTACHEF	33.27
391	P04271	S100	DRB1*01:20	27	41	KKSELKELINNELSH	33.28
392	P04271	S100	DRB1*01:24	70	84	QEFMAFVAMVTTACH	33.55
393	P04271	S100	DRB1*01:01	28	42	KSELKELINNELSHF	33.60
394	P04271	S100	DRB1*11:14	29	43	SELKELINNELSHFL	34.41
395	P04271	S100	DRB1*13:02	29	43	SELKELINNELSHFL	34.41
396	P04271	S100	DRB1*13:23	29	43	SELKELINNELSHFL	34.41
397	P04271	S100	DRB1*13:97	29	43	SELKELINNELSHFL	34.41
398	P04271	S100	DRB1*01:24	71	85	EFMAFVAMVTTACHE	34.66
399	P04271	S100	DRB1*04:04	2	16	LEKAMVALIDVFHQY	35.24
400	P04271	S100	DRB1*11:14	30	44	ELKELINNELSHFLE	36.11
401	P04271	S100	DRB1*13:02	30	44	ELKELINNELSHFLE	36.11
402	P04271	S100	DRB1*13:23	30	44	ELKELINNELSHFLE	36.11
403	P04271	S100	DRB1*13:97	30	44	ELKELINNELSHFLE	36.11
404	P04271	S100	DRB1*01:24	72	86	FMAFVAMVTTACHEF	36.98
405	P04271	S100	DRB1*01:29	71	85	EFMAFVAMVTTACHE	38.12
406	P04271	S100	DRB1*04:08	73	87	MAFVAMVTTACHEFF	38.29
407	P04271	S100	DRB1*04:10	3	17	EKAMVALIDVFHQYS	39.03
408	P04271	S100	DRB1*01:24	73	87	MAFVAMVTTACHEFF	39.17
409	P04271	S100	DRB1*01:01	68	82	DFQEFMAFVAMVTTA	39.72
410	P04271	S100	DRB1*01:29	70	84	QEFMAFVAMVTTACH	39.96
411	P04271	S100	DRB1*01:18	27	41	KKSELKELINNELSH	40.51
412	P04271	S100	DRB1*01:18	29	43	SELKELINNELSHFL	40.69
413	P04271	S100	DRB1*01:20	30	44	ELKELINNELSHFLE	40.73
414	P04271	S100	DRB1*04:08	72	86	FMAFVAMVTTACHEF	40.92
415	P04271	S100	DRB1*01:20	3	17	EKAMVALIDVFHQYS	41.09
416	P04271	S100	DRB1*10:01	3	17	EKAMVALIDVFHQYS	41.59
417	P04271	S100	DRB1*01:11	71	85	EFMAFVAMVTTACHE	41.61
418	P04271	S100	DRB1*04:10	2	16	LEKAMVALIDVFHQY	42.14
419	P04271	S100	DRB1*01:29	72	86	FMAFVAMVTTACHEF	42.84
420	P04271	S100	DRB1*01:24	68	82	DFQEFMAFVAMVTTA	43.30
421	P04271	S100	DRB1*04:04	74	88	AFVAMVTTACHEFFE	43.34
422	P04271	S100	DRB1*10:01	68	82	DFQEFMAFVAMVTTA	43.75

423	P04271	S100	DRB1*13:96	29	43	SELKELINNELSHFL	44.29
424	P04271	S100	DRB1*01:11	72	86	FMAFVAMVTTACHEF	44.61
425	P04271	S100	DRB1*01:20	73	87	MAFVAMVTTACHEFF	44.69
426	P04271	S100	DRB1*01:20	26	40	LKKSELKELINNELS	45.07
427	P04271	S100	DRB1*01:29	73	87	MAFVAMVTTACHEFF	45.33
428	P04271	S100	DRB1*11:14	31	45	LKELINNELSHFLEE	45.36
429	P04271	S100	DRB1*13:02	31	45	LKELINNELSHFLEE	45.36
430	P04271	S100	DRB1*13:23	31	45	LKELINNELSHFLEE	45.36
431	P04271	S100	DRB1*13:97	31	45	LKELINNELSHFLEE	45.36
432	P04271	S100	DRB1*01:01	69	83	FQEFMAFVAMVTTAC	45.90
433	P04271	S100	DRB1*10:01	2	16	LEKAMVALIDVFHQY	46.06
434	P04271	S100	DRB1*04:08	71	85	EFMAFVAMVTTACHE	46.13
435	P04271	S100	DRB1*01:11	73	87	MAFVAMVTTACHEFF	46.46
436	P04271	S100	DRB1*10:01	4	18	KAMVALIDVFHQYSG	46.68
437	P04271	S100	DRB1*13:96	30	44	ELKELINNELSHFLE	46.72
438	P04271	S100	DRB1*04:04	71	85	EFMAFVAMVTTACHE	46.77
439	P04271	S100	DRB1*01:11	70	84	QEFMAFVAMVTTACH	47.26
440	P04271	S100	DRB1*01:20	4	18	KAMVALIDVFHQYSG	47.40
441	P04271	S100	DRB1*10:01	69	83	FQEFMAFVAMVTTAC	48.37
442	P04271	S100	DRB1*01:20	1	15	ELEKAMVALIDVFHQ	48.56
443	P04271	S100	DRB1*01:20	2	16	LEKAMVALIDVFHQY	48.67
444	P04271	S100	DRB1*04:10	4	18	KAMVALIDVFHQYSG	48.80
445	P04271	S100	DRB1*01:01	27	41	KKSELKELINNELSH	48.94
446	P04271	S100	DRB1*01:18	30	44	ELKELINNELSHFLE	49.73
447	P04271	S100	DRB1*01:20	72	86	FMAFVAMVTTACHEF	49.78
448	P14679	Tyrosinase	DRB1*01:01	178	192	WMHYYVSMDALLGGS	6.14
449	P14679	Tyrosinase	DRB1*01:18	178	192	WMHYYVSMDALLGGS	6.47
450	P14679	Tyrosinase	DRB1*01:01	179	193	MHYYVSMDALLGGSE	6.72
451	P14679	Tyrosinase	DRB1*01:01	177	191	VWMHYYVSMDALLGG	6.98
452	P14679	Tyrosinase	DRB1*01:18	179	193	MHYYVSMDALLGGSE	7.11
453	P14679	Tyrosinase	DRB1*01:18	177	191	VWMHYYVSMDALLGG	7.47
454	P14679	Tyrosinase	DRB1*01:01	180	194	HYYVSMDALLGGSEI	7.66
455	P14679	Tyrosinase	DRB1*01:18	472	486	SRIWSWLLGAAMVGA	7.97
456	P14679	Tyrosinase	DRB1*01:18	180	194	HYYVSMDALLGGSEI	8.30
457	P14679	Tyrosinase	DRB1*10:01	132	146	DKFFAYLTLAKHTIS	8.37
458	P14679	Tyrosinase	DRB1*10:01	131	145	KDKFFAYLTLAKHTI	8.52
459	P14679	Tyrosinase	DRB1*01:01	472	486	SRIWSWLLGAAMVGA	8.69
460	P14679	Tyrosinase	DRB1*10:01	178	192	WMHYYVSMDALLGGS	9.19
461	P14679	Tyrosinase	DRB1*01:01	181	195	YYVSMDALLGGSEIW	9.22
462	P14679	Tyrosinase	DRB1*10:01	133	147	KFFAYLTLAKHTISS	9.36
463	P14679	Tyrosinase	DRB1*10:01	179	193	MHYYVSMDALLGGSE	9.68
464	P14679	Tyrosinase	DRB1*01:18	471	485	ASRIWSWLLGAAMVG	9.89
465	P14679	Tyrosinase	DRB1*01:18	484	498	VGAVLTALLAGLVSL	9.96

466	P14679	Tyrosinase	DRB1*10:01	177	191	VWMHYYVSMDALLGG	10.07
467	P14679	Tyrosinase	DRB1*01:18	181	195	YYVSMDALLGGSEIW	10.14
468	P14679	Tyrosinase	DRB1*10:01	130	144	EKDKFFAYLTLAKHT	10.21
469	P14679	Tyrosinase	DRB1*01:18	473	487	RIWSWLLGAAMVGAV	10.27
470	P14679	Tyrosinase	DRB1*01:20	483	497	MVGAVLTALLAGLVS	10.27
471	P14679	Tyrosinase	DRB1*01:01	483	497	MVGAVLTALLAGLVS	10.30
472	P14679	Tyrosinase	DRB1*01:01	484	498	VGAVLTALLAGLVSL	10.38
473	P14679	Tyrosinase	DRB1*01:18	483	497	MVGAVLTALLAGLVS	10.38
474	P14679	Tyrosinase	DRB1*01:01	471	485	ASRIWSWLLGAAMVG	10.55
475	P14679	Tyrosinase	DRB1*10:01	180	194	HYYVSMDALLGGSEI	10.58
476	P14679	Tyrosinase	DRB1*01:01	176	190	FVWMHYYVSMDALLG	11.07
477	P14679	Tyrosinase	DRB1*01:01	473	487	RIWSWLLGAAMVGAV	11.66
478	P14679	Tyrosinase	DRB1*01:20	484	498	VGAVLTALLAGLVSL	11.67
479	P14679	Tyrosinase	DRB1*01:18	176	190	FVWMHYYVSMDALLG	11.92
480	P14679	Tyrosinase	DPB1*33:01	384	398	PIFLLHHAFVDSIFE	12.00
481	P14679	Tyrosinase	DPB1*71:01	384	398	PIFLLHHAFVDSIFE	12.00
482	P14679	Tyrosinase	DRB1*01:18	485	499	GAVLTALLAGLVSLL	12.16
483	P14679	Tyrosinase	DRB1*01:29	178	192	WMHYYVSMDALLGGS	12.32
484	P14679	Tyrosinase	DRB1*01:18	470	484	QASRIWSWLLGAAMV	12.37
485	P14679	Tyrosinase	DPB1*33:01	383	397	DPIFLLHHAFVDSIF	12.43
486	P14679	Tyrosinase	DPB1*71:01	383	397	DPIFLLHHAFVDSIF	12.43
487	P14679	Tyrosinase	DRB1*10:01	181	195	YYVSMDALLGGSEIW	12.51
488	P14679	Tyrosinase	DRB1*10:01	176	190	FVWMHYYVSMDALLG	12.54
489	P14679	Tyrosinase	DRB1*01:29	179	193	MHYYVSMDALLGGSE	12.68
490	P14679	Tyrosinase	DRB1*11:03	493	507	AGLVSLLCRHKRKQL	12.77
491	P14679	Tyrosinase	DPB1*33:01	389	403	HHAFVDSIFEQWLRR	13.53
492	P14679	Tyrosinase	DPB1*71:01	389	403	HHAFVDSIFEQWLRR	13.53
493	P14679	Tyrosinase	DRB1*01:24	178	192	WMHYYVSMDALLGGS	13.67
494	P14679	Tyrosinase	DPB1*33:01	382	396	NDPIFLLHHAFVDSI	13.88
495	P14679	Tyrosinase	DPB1*71:01	382	396	NDPIFLLHHAFVDSI	13.88
496	P14679	Tyrosinase	DPB1*33:01	386	400	FLLHHAFVDSIFEQW	13.93
497	P14679	Tyrosinase	DPB1*71:01	386	400	FLLHHAFVDSIFEQW	13.93
498	P14679	Tyrosinase	DRB1*01:01	470	484	QASRIWSWLLGAAMV	13.94
499	P14679	Tyrosinase	DRB1*11:03	494	508	GLVSLLCRHKRKQLP	14.00
500	P14679	Tyrosinase	DRB1*01:24	484	498	VGAVLTALLAGLVSL	14.02
501	P14679	Tyrosinase	DRB1*13:21	426	440	MVPFIPLYRNGDFFI	14.15
502	P14679	Tyrosinase	DRB1*01:24	472	486	SRIWSWLLGAAMVGA	14.32
503	P14679	Tyrosinase	DRB1*01:01	485	499	GAVLTALLAGLVSLL	14.36
504	P14679	Tyrosinase	DRB1*01:18	133	147	KFFAYLTLAKHTISS	14.59
505	P14679	Tyrosinase	DRB1*01:18	132	146	DKFFAYLTLAKHTIS	14.72
506	P14679	Tyrosinase	DRB1*01:24	485	499	GAVLTALLAGLVSLL	14.80
507	P14679	Tyrosinase	DRB1*01:18	131	145	KDKFFAYLTLAKHTI	15.02
508	P14679	Tyrosinase	DRB1*13:21	427	441	VPFIPLYRNGDFFIS	15.28

509	P14679	Tyrosinase	DRB1*01:29	177	191	VWMHYYVSMDALLGG	15.40
510	P14679	Tyrosinase	DRB1*01:29	180	194	HYYVSMDALLGGSEI	15.66
511	P14679	Tyrosinase	DRB1*01:29	472	486	SRIWSWLLGAAMVGA	15.72
512	P14679	Tyrosinase	DRB1*01:24	179	193	MHYYVSMDALLGGSE	15.82
513	P14679	Tyrosinase	DRB1*01:24	177	191	VWMHYYVSMDALLGG	15.90
514	P14679	Tyrosinase	DRB1*11:03	492	506	LAGLVSLLCRHKRKQ	16.35
515	P14679	Tyrosinase	DRB1*13:21	425	439	YMVPFIPLYRNGDFF	16.43
516	P14679	Tyrosinase	DRB1*01:20	485	499	GAVLTALLAGLVSLL	16.49
517	P14679	Tyrosinase	DRB1*10:01	129	143	PEKDKFFAYLTLAKH	16.55
518	P14679	Tyrosinase	DPB1*33:01	390	404	HAFVDSIFEQWLRRH	16.76
519	P14679	Tyrosinase	DPB1*71:01	390	404	HAFVDSIFEQWLRRH	16.76
520	P14679	Tyrosinase	DRB1*10:01	134	148	FFAYLTLAKHTISSD	16.85
521	P14679	Tyrosinase	DPB1*04:01	389	403	HHAFVDSIFEQWLRR	16.90
522	P14679	Tyrosinase	DPB1*126:01	389	403	HHAFVDSIFEQWLRR	16.90
523	P14679	Tyrosinase	DPB1*23:01	389	403	HHAFVDSIFEQWLRR	16.90
524	P14679	Tyrosinase	DPB1*39:01	389	403	HHAFVDSIFEQWLRR	16.90
525	P14679	Tyrosinase	DRB1*01:24	473	487	RIWSWLLGAAMVGAV	17.09
526	P14679	Tyrosinase	DPB1*33:01	385	399	IFLLHHAFVDSIFEQ	17.14
527	P14679	Tyrosinase	DPB1*71:01	385	399	IFLLHHAFVDSIFEQ	17.14
528	P14679	Tyrosinase	DRB1*01:20	489	503	TALLAGLVSLLCRHK	17.15
529	P14679	Tyrosinase	DPB1*33:01	173	187	YDLFVWMHYYVSMDA	17.24
530	P14679	Tyrosinase	DPB1*71:01	173	187	YDLFVWMHYYVSMDA	17.24
531	P14679	Tyrosinase	DPB1*33:01	421	435	NRESYMVPFIPLYRN	17.37
532	P14679	Tyrosinase	DPB1*71:01	421	435	NRESYMVPFIPLYRN	17.37
533	P14679	Tyrosinase	DPB1*33:01	420	434	HNRESYMVPFIPLYR	17.46
534	P14679	Tyrosinase	DPB1*71:01	420	434	HNRESYMVPFIPLYR	17.46
535	P14679	Tyrosinase	DRB1*01:11	178	192	WMHYYVSMDALLGGS	17.88
536	P14679	Tyrosinase	DRB1*01:20	55	69	CQNILLSNAPLGPQF	17.94
537	P14679	Tyrosinase	DRB1*01:24	483	497	MVGAVLTALLAGLVS	17.95
538	P14679	Tyrosinase	DRB1*01:01	133	147	KFFAYLTLAKHTISS	18.00
539	P14679	Tyrosinase	DPB1*33:01	381	395	ANDPIFLLHHAFVDS	18.06
540	P14679	Tyrosinase	DPB1*71:01	381	395	ANDPIFLLHHAFVDS	18.06
541	P14679	Tyrosinase	DRB1*01:01	132	146	DKFFAYLTLAKHTIS	18.08
542	P14679	Tyrosinase	DRB1*01:18	486	500	AVLTALLAGLVSLLC	18.24
543	P14679	Tyrosinase	DRB1*01:24	471	485	ASRIWSWLLGAAMVG	18.32
544	P14679	Tyrosinase	DRB1*01:20	56	70	QNILLSNAPLGPQFP	18.33
545	P14679	Tyrosinase	DRB1*01:29	484	498	VGAVLTALLAGLVSL	18.36
546	P14679	Tyrosinase	DRB1*01:29	483	497	MVGAVLTALLAGLVS	18.42
547	P14679	Tyrosinase	DRB1*11:03	495	509	LVSLLCRHKRKQLPE	18.55
548	P14679	Tyrosinase	DPB1*72:01	389	403	HHAFVDSIFEQWLRR	18.99
549	P14679	Tyrosinase	DPB1*33:01	172	186	IYDLFVWMHYYVSMD	19.04
550	P14679	Tyrosinase	DPB1*71:01	172	186	IYDLFVWMHYYVSMD	19.04
551	P14679	Tyrosinase	DRB1*11:03	491	505	LLAGLVSLLCRHKRK	19.40

552	P14679	Tyrosinase	DRB1*01:01	131	145	KDKFFAYLTLAKHTI	19.53
553	P14679	Tyrosinase	DRB1*13:21	428	442	PFIPLYRNGDFFISS	19.53
554	P14679	Tyrosinase	DPB1*33:01	174	188	DLFVWMHYYVSMDAL	19.62
555	P14679	Tyrosinase	DPB1*71:01	174	188	DLFVWMHYYVSMDAL	19.62
556	P14679	Tyrosinase	DRB1*01:29	471	485	ASRIWSWLLGAAMVG	19.70
557	P14679	Tyrosinase	DRB1*11:02	493	507	AGLVSLLCRHKRKQL	19.74
558	P14679	Tyrosinase	DRB1*11:65	493	507	AGLVSLLCRHKRKQL	19.74
559	P14679	Tyrosinase	DRB1*13:01	493	507	AGLVSLLCRHKRKQL	19.74
560	P14679	Tyrosinase	DRB1*01:18	482	496	AMVGAVLTALLAGLV	19.87
561	P14679	Tyrosinase	DRB1*01:29	473	487	RIWSWLLGAAMVGAV	19.89
562	P14679	Tyrosinase	DRB1*01:11	179	193	MHYYVSMDALLGGSE	19.93
563	P14679	Tyrosinase	DRB1*01:24	180	194	HYYVSMDALLGGSEI	20.06
564	P14679	Tyrosinase	DRB1*01:18	130	144	EKDKFFAYLTLAKHT	20.08
565	P14679	Tyrosinase	DRB1*13:21	424	438	SYMVPFIPLYRNGDF	20.22
566	P14679	Tyrosinase	DRB1*01:18	489	503	TALLAGLVSLLCRHK	20.32
567	P14679	Tyrosinase	DRB1*01:18	474	488	IWSWLLGAAMVGAVL	20.43
568	P14679	Tyrosinase	DRB1*03:11	142	156	KHTISSDYVIPIGTY	20.71
569	P14679	Tyrosinase	DRB1*11:04	493	507	AGLVSLLCRHKRKQL	20.85
570	P14679	Tyrosinase	DRB1*11:46	493	507	AGLVSLLCRHKRKQL	20.85
571	P14679	Tyrosinase	DRB1*11:58	493	507	AGLVSLLCRHKRKQL	20.85
572	P14679	Tyrosinase	DRB1*13:11	493	507	AGLVSLLCRHKRKQL	20.85
573	P14679	Tyrosinase	DPB1*33:01	388	402	LHHAFVDSIFEQWLR	20.88
574	P14679	Tyrosinase	DPB1*71:01	388	402	LHHAFVDSIFEQWLR	20.88
575	P14679	Tyrosinase	DPB1*02:01	384	398	PIFLLHHAFVDSIFE	20.90
576	P14679	Tyrosinase	DPB1*46:01	384	398	PIFLLHHAFVDSIFE	20.90
577	P14679	Tyrosinase	DPB1*81:01	384	398	PIFLLHHAFVDSIFE	20.90
578	P14679	Tyrosinase	DRB1*01:18	134	148	FFAYLTLAKHTISSD	21.00
579	P14679	Tyrosinase	DRB1*01:11	177	191	VWMHYYVSMDALLGG	21.13
580	P14679	Tyrosinase	DRB1*11:14	364	378	NALHIYMNGTMSQVQ	21.33
581	P14679	Tyrosinase	DRB1*13:02	364	378	NALHIYMNGTMSQVQ	21.33
582	P14679	Tyrosinase	DRB1*13:23	364	378	NALHIYMNGTMSQVQ	21.33
583	P14679	Tyrosinase	DRB1*13:97	364	378	NALHIYMNGTMSQVQ	21.33
584	P14679	Tyrosinase	DRB1*01:20	482	496	AMVGAVLTALLAGLV	21.40
585	P14679	Tyrosinase	DPB1*02:02	384	398	PIFLLHHAFVDSIFE	21.63
586	P14679	Tyrosinase	DPB1*47:01	384	398	PIFLLHHAFVDSIFE	21.63
587	P14679	Tyrosinase	DRB1*15:01	363	377	HNALHIYMNGTMSQV	21.63
588	P14679	Tyrosinase	DRB1*15:06	363	377	HNALHIYMNGTMSQV	21.63
589	P14679	Tyrosinase	DRB1*01:18	469	483	EQASRIWSWLLGAAM	21.71
590	P14679	Tyrosinase	DPB1*33:01	171	185	NIYDLFVWMHYYVSM	21.92
591	P14679	Tyrosinase	DPB1*71:01	171	185	NIYDLFVWMHYYVSM	21.92
592	P14679	Tyrosinase	DRB1*01:18	55	69	CQNILLSNAPLGPQF	21.97
593	P14679	Tyrosinase	DPB1*33:01	129	143	PEKDKFFAYLTLAKH	22.14
594	P14679	Tyrosinase	DPB1*71:01	129	143	PEKDKFFAYLTLAKH	22.14

595	P14679	Tyrosinase	DRB1*01:18	487	501	VLTALLAGLVSLLCR	22.16
596	P14679	Tyrosinase	DRB1*01:11	472	486	SRIWSWLLGAAMVGA	22.17
597	P14679	Tyrosinase	DRB1*01:29	181	195	YYVSMDALLGGSEIW	22.18
598	P14679	Tyrosinase	DRB1*01:24	486	500	AVLTALLAGLVSLLC	22.21
599	P14679	Tyrosinase	DRB1*11:42	493	507	AGLVSLLCRHKRKQL	22.26
600	P14679	Tyrosinase	DPB1*02:01	383	397	DPIFLLHHAFVDSIF	22.28
601	P14679	Tyrosinase	DPB1*46:01	383	397	DPIFLLHHAFVDSIF	22.28
602	P14679	Tyrosinase	DPB1*81:01	383	397	DPIFLLHHAFVDSIF	22.28
603	P14679	Tyrosinase	DRB1*11:01	133	147	KFFAYLTLAKHTISS	22.47
604	P14679	Tyrosinase	DRB1*11:10	133	147	KFFAYLTLAKHTISS	22.47
605	P14679	Tyrosinase	DRB1*11:12	133	147	KFFAYLTLAKHTISS	22.47
606	P14679	Tyrosinase	DRB1*11:28	133	147	KFFAYLTLAKHTISS	22.47
607	P14679	Tyrosinase	DRB1*11:29	133	147	KFFAYLTLAKHTISS	22.47
608	P14679	Tyrosinase	DRB1*11:49	133	147	KFFAYLTLAKHTISS	22.47
609	P14679	Tyrosinase	DRB1*11:62	133	147	KFFAYLTLAKHTISS	22.47
610	P14679	Tyrosinase	DRB1*11:74	133	147	KFFAYLTLAKHTISS	22.47
611	P14679	Tyrosinase	DRB1*13:05	133	147	KFFAYLTLAKHTISS	22.47
612	P14679	Tyrosinase	DRB1*13:14	133	147	KFFAYLTLAKHTISS	22.47
613	P14679	Tyrosinase	DRB1*13:50	133	147	KFFAYLTLAKHTISS	22.47
614	P14679	Tyrosinase	DPB1*33:01	422	436	RESYMVPFIPLYRNG	22.51
615	P14679	Tyrosinase	DPB1*71:01	422	436	RESYMVPFIPLYRNG	22.51
616	P14679	Tyrosinase	DRB1*01:18	56	70	QNILLSNAPLGPQFP	22.56
617	P14679	Tyrosinase	DPB1*33:01	130	144	EKDKFFAYLTLAKHT	22.68
618	P14679	Tyrosinase	DPB1*71:01	130	144	EKDKFFAYLTLAKHT	22.68
619	P14679	Tyrosinase	DRB1*01:29	485	499	GAVLTALLAGLVSLL	22.75
620	P14679	Tyrosinase	DPB1*02:02	383	397	DPIFLLHHAFVDSIF	22.77
621	P14679	Tyrosinase	DPB1*47:01	383	397	DPIFLLHHAFVDSIF	22.77
622	P14679	Tyrosinase	DRB1*01:24	470	484	QASRIWSWLLGAAMV	22.77
623	P14679	Tyrosinase	DRB1*11:02	494	508	GLVSLLCRHKRKQLP	22.97
624	P14679	Tyrosinase	DRB1*11:65	494	508	GLVSLLCRHKRKQLP	22.97
625	P14679	Tyrosinase	DRB1*13:01	494	508	GLVSLLCRHKRKQLP	22.97
626	P14679	Tyrosinase	DRB1*11:14	363	377	HNALHIYMNGTMSQV	23.28
627	P14679	Tyrosinase	DRB1*13:02	363	377	HNALHIYMNGTMSQV	23.28
628	P14679	Tyrosinase	DRB1*13:23	363	377	HNALHIYMNGTMSQV	23.28
629	P14679	Tyrosinase	DRB1*13:97	363	377	HNALHIYMNGTMSQV	23.28
630	P14679	Tyrosinase	DPB1*04:01	390	404	HAFVDSIFEQWLRRH	23.30
631	P14679	Tyrosinase	DPB1*126:01	390	404	HAFVDSIFEQWLRRH	23.30
632	P14679	Tyrosinase	DPB1*23:01	390	404	HAFVDSIFEQWLRRH	23.30
633	P14679	Tyrosinase	DPB1*39:01	390	404	HAFVDSIFEQWLRRH	23.30
634	P14679	Tyrosinase	DRB1*13:21	133	147	KFFAYLTLAKHTISS	23.40
635	P14679	Tyrosinase	DPB1*33:01	131	145	KDKFFAYLTLAKHTI	23.41
636	P14679	Tyrosinase	DPB1*71:01	131	145	KDKFFAYLTLAKHTI	23.41
637	P14679	Tyrosinase	DRB1*01:01	486	500	AVLTALLAGLVSLLC	23.43

638	P14679	Tyrosinase	DPB1*33:01	419	433	GHNRESYMVPFIPLY	23.48
639	P14679	Tyrosinase	DPB1*71:01	419	433	GHNRESYMVPFIPLY	23.48
640	P14679	Tyrosinase	DRB1*01:01	489	503	TALLAGLVSLLCRHK	23.48
641	P14679	Tyrosinase	DRB1*03:11	141	155	AKHTISSDYVIPIGT	23.51
642	P14679	Tyrosinase	DRB1*01:20	488	502	LTALLAGLVSLLCRH	23.56
643	P14679	Tyrosinase	DRB1*01:01	198	212	IDFAHEAPAFLPWHR	23.67
644	P14679	Tyrosinase	DRB1*15:01	364	378	NALHIYMNGTMSQVQ	23.69
645	P14679	Tyrosinase	DRB1*15:06	364	378	NALHIYMNGTMSQVQ	23.69
646	P14679	Tyrosinase	DRB1*01:20	178	192	WMHYYVSMDALLGGS	23.91
647	P14679	Tyrosinase	DRB1*01:20	54	68	SCQNILLSNAPLGPQ	23.93
648	P14679	Tyrosinase	DRB1*01:18	463	477	YIKSYLEQASRIWSW	23.97
649	P14679	Tyrosinase	DRB1*01:20	490	504	ALLAGLVSLLCRHKR	23.97
650	P14679	Tyrosinase	DRB1*01:01	482	496	AMVGAVLTALLAGLV	24.00
651	P14679	Tyrosinase	DRB1*01:18	488	502	LTALLAGLVSLLCRH	24.03
652	P14679	Tyrosinase	DRB1*01:01	55	69	CQNILLSNAPLGPQF	24.12
653	P14679	Tyrosinase	DRB1*01:01	56	70	QNILLSNAPLGPQFP	24.12
654	P14679	Tyrosinase	DRB1*13:21	132	146	DKFFAYLTLAKHTIS	24.12
655	P14679	Tyrosinase	DRB1*01:20	179	193	MHYYVSMDALLGGSE	24.19
656	P14679	Tyrosinase	DRB1*11:14	365	379	ALHIYMNGTMSQVQG	24.22
657	P14679	Tyrosinase	DRB1*13:02	365	379	ALHIYMNGTMSQVQG	24.22
658	P14679	Tyrosinase	DRB1*13:23	365	379	ALHIYMNGTMSQVQG	24.22
659	P14679	Tyrosinase	DRB1*13:97	365	379	ALHIYMNGTMSQVQG	24.22
660	P14679	Tyrosinase	DRB1*01:24	181	195	YYVSMDALLGGSEIW	24.46
661	P14679	Tyrosinase	DRB1*11:42	492	506	LAGLVSLLCRHKRKQ	24.46
662	P14679	Tyrosinase	DPB1*33:01	391	405	AFVDSIFEQWLRRHR	24.56
663	P14679	Tyrosinase	DPB1*71:01	391	405	AFVDSIFEQWLRRHR	24.56
664	P14679	Tyrosinase	DRB1*01:24	176	190	FVWMHYYVSMDALLG	24.65
665	P14679	Tyrosinase	DRB1*11:04	492	506	LAGLVSLLCRHKRKQ	24.86
666	P14679	Tyrosinase	DRB1*11:46	492	506	LAGLVSLLCRHKRKQ	24.86
667	P14679	Tyrosinase	DRB1*11:58	492	506	LAGLVSLLCRHKRKQ	24.86
668	P14679	Tyrosinase	DRB1*13:11	492	506	LAGLVSLLCRHKRKQ	24.86
669	P14679	Tyrosinase	DRB1*13:21	131	145	KDKFFAYLTLAKHTI	25.02
670	P14679	Tyrosinase	DRB1*01:20	486	500	AVLTALLAGLVSLLC	25.23
671	P14679	Tyrosinase	DRB1*01:11	180	194	HYYVSMDALLGGSEI	25.25
672	P14679	Tyrosinase	DRB1*10:01	472	486	SRIWSWLLGAAMVGA	25.36
673	P14679	Tyrosinase	DRB1*01:01	469	483	EQASRIWSWLLGAAM	25.47
674	P14679	Tyrosinase	DRB1*01:18	54	68	SCQNILLSNAPLGPQ	25.90
675	P14679	Tyrosinase	DRB1*01:01	130	144	EKDKFFAYLTLAKHT	25.93
676	P14679	Tyrosinase	DRB1*01:20	133	147	KFFAYLTLAKHTISS	26.01
677	P14679	Tyrosinase	DRB1*11:01	132	146	DKFFAYLTLAKHTIS	26.01
678	P14679	Tyrosinase	DRB1*11:10	132	146	DKFFAYLTLAKHTIS	26.01
679	P14679	Tyrosinase	DRB1*11:12	132	146	DKFFAYLTLAKHTIS	26.01
680	P14679	Tyrosinase	DRB1*11:28	132	146	DKFFAYLTLAKHTIS	26.01

681	P14679	Tyrosinase	DRB1*11:29	132	146	DKFFAYLTLAKHTIS	26.01
682	P14679	Tyrosinase	DRB1*11:49	132	146	DKFFAYLTLAKHTIS	26.01
683	P14679	Tyrosinase	DRB1*11:62	132	146	DKFFAYLTLAKHTIS	26.01
684	P14679	Tyrosinase	DRB1*11:74	132	146	DKFFAYLTLAKHTIS	26.01
685	P14679	Tyrosinase	DRB1*13:05	132	146	DKFFAYLTLAKHTIS	26.01
686	P14679	Tyrosinase	DRB1*13:14	132	146	DKFFAYLTLAKHTIS	26.01
687	P14679	Tyrosinase	DRB1*13:50	132	146	DKFFAYLTLAKHTIS	26.01
688	P14679	Tyrosinase	DRB1*01:18	462	476	DYIKSYLEQASRIWS	26.19
689	P14679	Tyrosinase	DRB1*01:29	176	190	FVWMHYYVSMDALLG	26.26
690	P14679	Tyrosinase	DPB1*02:01	382	396	NDPIFLLHHAFVDSI	26.27
691	P14679	Tyrosinase	DPB1*46:01	382	396	NDPIFLLHHAFVDSI	26.27
692	P14679	Tyrosinase	DPB1*81:01	382	396	NDPIFLLHHAFVDSI	26.27
693	P14679	Tyrosinase	DRB1*11:02	492	506	LAGLVSLLCRHKRKQ	26.28
694	P14679	Tyrosinase	DRB1*11:65	492	506	LAGLVSLLCRHKRKQ	26.28
695	P14679	Tyrosinase	DRB1*13:01	492	506	LAGLVSLLCRHKRKQ	26.28
696	P14679	Tyrosinase	DRB1*11:04	494	508	GLVSLLCRHKRKQLP	26.41
697	P14679	Tyrosinase	DRB1*11:46	494	508	GLVSLLCRHKRKQLP	26.41
698	P14679	Tyrosinase	DRB1*11:58	494	508	GLVSLLCRHKRKQLP	26.41
699	P14679	Tyrosinase	DRB1*13:11	494	508	GLVSLLCRHKRKQLP	26.41
700	P14679	Tyrosinase	DRB1*04:08	178	192	WMHYYVSMDALLGGS	26.42
701	P14679	Tyrosinase	DPB1*04:01	388	402	LHHAFVDSIFEQWLR	26.61
702	P14679	Tyrosinase	DPB1*126:01	388	402	LHHAFVDSIFEQWLR	26.61
703	P14679	Tyrosinase	DPB1*23:01	388	402	LHHAFVDSIFEQWLR	26.61
704	P14679	Tyrosinase	DPB1*39:01	388	402	LHHAFVDSIFEQWLR	26.61
705	P14679	Tyrosinase	DRB1*01:20	472	486	SRIWSWLLGAAMVGA	26.76
706	P14679	Tyrosinase	DRB1*01:29	470	484	QASRIWSWLLGAAMV	26.82
707	P14679	Tyrosinase	DRB1*15:01	362	376	MHNALHIYMNGTMSQ	26.91
708	P14679	Tyrosinase	DRB1*15:06	362	376	MHNALHIYMNGTMSQ	26.91
709	P14679	Tyrosinase	DRB1*01:01	474	488	IWSWLLGAAMVGAVL	26.95
710	P14679	Tyrosinase	DPB1*72:01	390	404	HAFVDSIFEQWLRRH	27.20
711	P14679	Tyrosinase	DPB1*33:01	387	401	LLHHAFVDSIFEQWL	27.29
712	P14679	Tyrosinase	DPB1*71:01	387	401	LLHHAFVDSIFEQWL	27.29
713	P14679	Tyrosinase	DRB1*01:01	463	477	YIKSYLEQASRIWSW	27.34
714	P14679	Tyrosinase	DRB1*01:18	383	397	DPIFLLHHAFVDSIF	27.37
715	P14679	Tyrosinase	DRB1*01:20	487	501	VLTALLAGLVSLLCR	27.38
716	P14679	Tyrosinase	DRB1*11:04	135	149	FAYLTLAKHTISSDY	27.46
717	P14679	Tyrosinase	DRB1*11:46	135	149	FAYLTLAKHTISSDY	27.46
718	P14679	Tyrosinase	DRB1*11:58	135	149	FAYLTLAKHTISSDY	27.46
719	P14679	Tyrosinase	DRB1*13:11	135	149	FAYLTLAKHTISSDY	27.46
720	P14679	Tyrosinase	DRB1*15:15	131	145	KDKFFAYLTLAKHTI	27.49
721	P14679	Tyrosinase	DPB1*02:02	382	396	NDPIFLLHHAFVDSI	27.56
722	P14679	Tyrosinase	DPB1*47:01	382	396	NDPIFLLHHAFVDSI	27.56
723	P14679	Tyrosinase	DRB1*11:42	491	505	LLAGLVSLLCRHKRK	27.59

724	P14679	Tyrosinase	DRB1*11:04	133	147	KFFAYLTLAKHTISS	27.61
725	P14679	Tyrosinase	DRB1*11:46	133	147	KFFAYLTLAKHTISS	27.61
726	P14679	Tyrosinase	DRB1*11:58	133	147	KFFAYLTLAKHTISS	27.61
727	P14679	Tyrosinase	DRB1*13:11	133	147	KFFAYLTLAKHTISS	27.61
728	P14679	Tyrosinase	DRB1*01:01	134	148	FFAYLTLAKHTISSD	27.66
729	P14679	Tyrosinase	DRB1*11:04	491	505	LLAGLVSLLCRHKRK	27.66
730	P14679	Tyrosinase	DRB1*11:46	491	505	LLAGLVSLLCRHKRK	27.66
731	P14679	Tyrosinase	DRB1*11:58	491	505	LLAGLVSLLCRHKRK	27.66
732	P14679	Tyrosinase	DRB1*13:11	491	505	LLAGLVSLLCRHKRK	27.66
733	P14679	Tyrosinase	DRB1*01:01	197	211	DIDFAHEAPAFLPWH	27.73
734	P14679	Tyrosinase	DRB1*01:20	180	194	HYYVSMDALLGGSEI	27.74
735	P14679	Tyrosinase	DRB1*01:20	132	146	DKFFAYLTLAKHTIS	27.98
736	P14679	Tyrosinase	DRB1*01:01	182	196	YVSMDALLGGSEIWR	28.17
737	P14679	Tyrosinase	DRB1*01:18	464	478	IKSYLEQASRIWSWL	28.18
738	P14679	Tyrosinase	DRB1*11:14	55	69	CQNILLSNAPLGPQF	28.20
739	P14679	Tyrosinase	DRB1*13:02	55	69	CQNILLSNAPLGPQF	28.20
740	P14679	Tyrosinase	DRB1*13:23	55	69	CQNILLSNAPLGPQF	28.20
741	P14679	Tyrosinase	DRB1*13:97	55	69	CQNILLSNAPLGPQF	28.20
742	P14679	Tyrosinase	DRB1*01:11	484	498	VGAVLTALLAGLVSL	28.25
743	P14679	Tyrosinase	DRB1*01:11	473	487	RIWSWLLGAAMVGAV	28.36
744	P14679	Tyrosinase	DRB1*11:04	134	148	FFAYLTLAKHTISSD	28.42
745	P14679	Tyrosinase	DRB1*11:46	134	148	FFAYLTLAKHTISSD	28.42
746	P14679	Tyrosinase	DRB1*11:58	134	148	FFAYLTLAKHTISSD	28.42
747	P14679	Tyrosinase	DRB1*13:11	134	148	FFAYLTLAKHTISSD	28.42
748	P14679	Tyrosinase	DRB1*16:09	131	145	KDKFFAYLTLAKHTI	28.43
749	P14679	Tyrosinase	DRB1*13:21	134	148	FFAYLTLAKHTISSD	28.46
750	P14679	Tyrosinase	DRB1*01:18	364	378	NALHIYMNGTMSQVQ	28.56
751	P14679	Tyrosinase	DRB1*01:11	471	485	ASRIWSWLLGAAMVG	28.57
752	P14679	Tyrosinase	DRB1*11:01	134	148	FFAYLTLAKHTISSD	28.57
753	P14679	Tyrosinase	DRB1*11:10	134	148	FFAYLTLAKHTISSD	28.57
754	P14679	Tyrosinase	DRB1*11:12	134	148	FFAYLTLAKHTISSD	28.57
755	P14679	Tyrosinase	DRB1*11:28	134	148	FFAYLTLAKHTISSD	28.57
756	P14679	Tyrosinase	DRB1*11:29	134	148	FFAYLTLAKHTISSD	28.57
757	P14679	Tyrosinase	DRB1*11:49	134	148	FFAYLTLAKHTISSD	28.57
758	P14679	Tyrosinase	DRB1*11:62	134	148	FFAYLTLAKHTISSD	28.57
759	P14679	Tyrosinase	DRB1*11:74	134	148	FFAYLTLAKHTISSD	28.57
760	P14679	Tyrosinase	DRB1*13:05	134	148	FFAYLTLAKHTISSD	28.57
761	P14679	Tyrosinase	DRB1*13:14	134	148	FFAYLTLAKHTISSD	28.57
762	P14679	Tyrosinase	DRB1*13:50	134	148	FFAYLTLAKHTISSD	28.57
763	P14679	Tyrosinase	DRB1*11:42	494	508	GLVSLLCRHKRKQLP	28.60
764	P14679	Tyrosinase	DRB1*01:18	490	504	ALLAGLVSLLCRHKR	28.62
765	P14679	Tyrosinase	DRB1*03:11	140	154	LAKHTISSDYVIPIG	28.63
766	P14679	Tyrosinase	DRB1*10:01	471	485	ASRIWSWLLGAAMVG	28.64

767	P14679	Tyrosinase	DRB1*11:14	56	70	QNILLSNAPLGPQFP	28.71
768	P14679	Tyrosinase	DRB1*13:02	56	70	QNILLSNAPLGPQFP	28.71
769	P14679	Tyrosinase	DRB1*13:23	56	70	QNILLSNAPLGPQFP	28.71
770	P14679	Tyrosinase	DRB1*13:97	56	70	QNILLSNAPLGPQFP	28.71
771	P14679	Tyrosinase	DRB1*01:18	198	212	IDFAHEAPAFLPWHR	29.19
772	P14679	Tyrosinase	DPB1*33:01	175	189	LFVWMHYYVSMDALL	29.20
773	P14679	Tyrosinase	DPB1*71:01	175	189	LFVWMHYYVSMDALL	29.20
774	P14679	Tyrosinase	DRB1*01:18	129	143	PEKDKFFAYLTLAKH	29.20
775	P14679	Tyrosinase	DRB1*04:08	179	193	MHYYVSMDALLGGSE	29.29
776	P14679	Tyrosinase	DRB1*01:01	364	378	NALHIYMNGTMSQVQ	29.38
777	P14679	Tyrosinase	DRB1*01:29	133	147	KFFAYLTLAKHTISS	29.56
778	P14679	Tyrosinase	DRB1*11:02	491	505	LLAGLVSLLCRHKRK	29.59
779	P14679	Tyrosinase	DRB1*11:65	491	505	LLAGLVSLLCRHKRK	29.59
780	P14679	Tyrosinase	DRB1*13:01	491	505	LLAGLVSLLCRHKRK	29.59
781	P14679	Tyrosinase	DRB1*01:20	131	145	KDKFFAYLTLAKHTI	29.67
782	P14679	Tyrosinase	DRB1*04:08	177	191	VWMHYYVSMDALLGG	29.69
783	P14679	Tyrosinase	DRB1*11:14	366	380	LHIYMNGTMSQVQGS	29.98
784	P14679	Tyrosinase	DRB1*13:02	366	380	LHIYMNGTMSQVQGS	29.98
785	P14679	Tyrosinase	DRB1*13:23	366	380	LHIYMNGTMSQVQGS	29.98
786	P14679	Tyrosinase	DRB1*13:97	366	380	LHIYMNGTMSQVQGS	29.98
787	P14679	Tyrosinase	DRB1*01:20	177	191	VWMHYYVSMDALLGG	30.03
788	P14679	Tyrosinase	DRB1*01:01	487	501	VLTALLAGLVSLLCR	30.19
789	P14679	Tyrosinase	DRB1*01:01	488	502	LTALLAGLVSLLCRH	30.25
790	P14679	Tyrosinase	DRB1*01:29	132	146	DKFFAYLTLAKHTIS	30.36
791	P14679	Tyrosinase	DRB1*01:01	462	476	DYIKSYLEQASRIWS	30.54
792	P14679	Tyrosinase	DRB1*01:01	54	68	SCQNILLSNAPLGPQ	30.55
793	P14679	Tyrosinase	DRB1*01:18	382	396	NDPIFLLHHAFVDSI	30.61
794	P14679	Tyrosinase	DRB1*01:18	4	18	AVLYCLLWSFQTSAG	30.66
795	P14679	Tyrosinase	DRB1*15:01	361	375	SMHNALHIYMNGTMS	30.89
796	P14679	Tyrosinase	DRB1*15:06	361	375	SMHNALHIYMNGTMS	30.89
797	P14679	Tyrosinase	DRB1*01:11	485	499	GAVLTALLAGLVSLL	31.00
798	P14679	Tyrosinase	DRB1*11:42	135	149	FAYLTLAKHTISSDY	31.03
799	P14679	Tyrosinase	DRB1*01:01	365	379	ALHIYMNGTMSQVQG	31.54
800	P14679	Tyrosinase	DRB1*11:42	133	147	KFFAYLTLAKHTISS	31.66
801	P14679	Tyrosinase	DRB1*01:01	196	210	RDIDFAHEAPAFLPW	31.68
802	P14679	Tyrosinase	DRB1*01:24	487	501	VLTALLAGLVSLLCR	31.75
803	P14679	Tyrosinase	DRB1*01:18	365	379	ALHIYMNGTMSQVQG	31.88
804	P14679	Tyrosinase	DRB1*15:15	130	144	EKDKFFAYLTLAKHT	31.94
805	P14679	Tyrosinase	DRB1*01:18	135	149	FAYLTLAKHTISSDY	32.03
806	P14679	Tyrosinase	DRB1*01:29	131	145	KDKFFAYLTLAKHTI	32.17
807	P14679	Tyrosinase	DRB1*13:21	423	437	ESYMVPFIPLYRNGD	32.19
808	P14679	Tyrosinase	DPB1*02:01	386	400	FLLHHAFVDSIFEQW	32.20
809	P14679	Tyrosinase	DPB1*46:01	386	400	FLLHHAFVDSIFEQW	32.20

810	P14679	Tyrosinase	DPB1*81:01	386	400	FLLHHAFVDSIFEQW	32.20
811	P14679	Tyrosinase	DRB1*01:18	384	398	PIFLLHHAFVDSIFE	32.20
812	P14679	Tyrosinase	DRB1*01:20	53	67	GSCQNILLSNAPLGP	32.20
813	P14679	Tyrosinase	DRB1*01:02	483	497	MVGAVLTALLAGLVS	32.45
814	P14679	Tyrosinase	DRB1*01:18	197	211	DIDFAHEAPAFLPWH	32.52
815	P14679	Tyrosinase	DRB1*04:01	178	192	WMHYYVSMDALLGGS	32.52
816	P14679	Tyrosinase	DRB1*11:02	495	509	LVSLLCRHKRKQLPE	32.55
817	P14679	Tyrosinase	DRB1*11:65	495	509	LVSLLCRHKRKQLPE	32.55
818	P14679	Tyrosinase	DRB1*13:01	495	509	LVSLLCRHKRKQLPE	32.55
819	P14679	Tyrosinase	DPB1*33:01	128	142	APEKDKFFAYLTLAK	32.56
820	P14679	Tyrosinase	DPB1*71:01	128	142	APEKDKFFAYLTLAK	32.56
821	P14679	Tyrosinase	DRB1*01:18	182	196	YVSMDALLGGSEIWR	32.68
822	P14679	Tyrosinase	DRB1*11:01	426	440	MVPFIPLYRNGDFFI	32.73
823	P14679	Tyrosinase	DRB1*11:10	426	440	MVPFIPLYRNGDFFI	32.73
824	P14679	Tyrosinase	DRB1*11:12	426	440	MVPFIPLYRNGDFFI	32.73
825	P14679	Tyrosinase	DRB1*11:28	426	440	MVPFIPLYRNGDFFI	32.73
826	P14679	Tyrosinase	DRB1*11:29	426	440	MVPFIPLYRNGDFFI	32.73
827	P14679	Tyrosinase	DRB1*11:49	426	440	MVPFIPLYRNGDFFI	32.73
828	P14679	Tyrosinase	DRB1*11:62	426	440	MVPFIPLYRNGDFFI	32.73
829	P14679	Tyrosinase	DRB1*11:74	426	440	MVPFIPLYRNGDFFI	32.73
830	P14679	Tyrosinase	DRB1*13:05	426	440	MVPFIPLYRNGDFFI	32.73
831	P14679	Tyrosinase	DRB1*13:14	426	440	MVPFIPLYRNGDFFI	32.73
832	P14679	Tyrosinase	DRB1*13:50	426	440	MVPFIPLYRNGDFFI	32.73
833	P14679	Tyrosinase	DRB1*10:01	470	484	QASRIWSWLLGAAMV	32.83
834	P14679	Tyrosinase	DPB1*02:02	389	403	HHAFVDSIFEQWLRR	32.91
835	P14679	Tyrosinase	DPB1*47:01	389	403	HHAFVDSIFEQWLRR	32.91
836	P14679	Tyrosinase	DRB1*01:20	134	148	FFAYLTLAKHTISSD	33.11
837	P14679	Tyrosinase	DRB1*11:42	134	148	FFAYLTLAKHTISSD	33.14
838	P14679	Tyrosinase	DRB1*01:20	471	485	ASRIWSWLLGAAMVG	33.16
839	P14679	Tyrosinase	DRB1*01:18	475	489	WSWLLGAAMVGAVLT	33.19
840	P14679	Tyrosinase	DPB1*72:01	388	402	LHHAFVDSIFEQWLR	33.24
841	P14679	Tyrosinase	DRB1*01:18	363	377	HNALHIYMNGTMSQV	33.33
842	P14679	Tyrosinase	DRB1*11:01	131	145	KDKFFAYLTLAKHTI	33.33
843	P14679	Tyrosinase	DRB1*11:10	131	145	KDKFFAYLTLAKHTI	33.33
844	P14679	Tyrosinase	DRB1*11:12	131	145	KDKFFAYLTLAKHTI	33.33
845	P14679	Tyrosinase	DRB1*11:28	131	145	KDKFFAYLTLAKHTI	33.33
846	P14679	Tyrosinase	DRB1*11:29	131	145	KDKFFAYLTLAKHTI	33.33
847	P14679	Tyrosinase	DRB1*11:49	131	145	KDKFFAYLTLAKHTI	33.33
848	P14679	Tyrosinase	DRB1*11:62	131	145	KDKFFAYLTLAKHTI	33.33
849	P14679	Tyrosinase	DRB1*11:74	131	145	KDKFFAYLTLAKHTI	33.33
850	P14679	Tyrosinase	DRB1*13:05	131	145	KDKFFAYLTLAKHTI	33.33
851	P14679	Tyrosinase	DRB1*13:14	131	145	KDKFFAYLTLAKHTI	33.33
852	P14679	Tyrosinase	DRB1*13:50	131	145	KDKFFAYLTLAKHTI	33.33

853	P14679	Tyrosinase	DRB1*03:11	143	157	HTISSDYVIPIGTYG	33.40
854	P14679	Tyrosinase	DPB1*33:01	132	146	DKFFAYLTLAKHTIS	33.50
855	P14679	Tyrosinase	DPB1*71:01	132	146	DKFFAYLTLAKHTIS	33.50
856	P14679	Tyrosinase	DRB1*01:11	483	497	MVGAVLTALLAGLVS	33.51
857	P14679	Tyrosinase	DPB1*02:01	385	399	IFLLHHAFVDSIFEQ	33.72
858	P14679	Tyrosinase	DPB1*46:01	385	399	IFLLHHAFVDSIFEQ	33.72
859	P14679	Tyrosinase	DPB1*81:01	385	399	IFLLHHAFVDSIFEQ	33.72
860	P14679	Tyrosinase	DRB1*01:01	464	478	IKSYLEQASRIWSWL	33.81
861	P14679	Tyrosinase	DRB1*01:24	474	488	IWSWLLGAAMVGAVL	33.81
862	P14679	Tyrosinase	DRB1*10:01	9	23	LLWSFQTSAGHFPRA	33.85
863	P14679	Tyrosinase	DRB1*11:08	133	147	KFFAYLTLAKHTISS	33.88
864	P14679	Tyrosinase	DPB1*02:02	386	400	FLLHHAFVDSIFEQW	33.94
865	P14679	Tyrosinase	DPB1*47:01	386	400	FLLHHAFVDSIFEQW	33.94
866	P14679	Tyrosinase	DRB1*01:11	176	190	FVWMHYYVSMDALLG	34.06
867	P14679	Tyrosinase	DRB1*16:09	132	146	DKFFAYLTLAKHTIS	34.14
868	P14679	Tyrosinase	DRB1*01:18	53	67	GSCQNILLSNAPLGP	34.26
869	P14679	Tyrosinase	DRB1*01:11	181	195	YYVSMDALLGGSEIW	34.27
870	P14679	Tyrosinase	DRB1*04:01	179	193	MHYYVSMDALLGGSE	34.35
871	P14679	Tyrosinase	DRB1*01:20	473	487	RIWSWLLGAAMVGAV	34.41
872	P14679	Tyrosinase	DRB1*15:15	129	143	PEKDKFFAYLTLAKH	34.63
873	P14679	Tyrosinase	DPB1*02:02	385	399	IFLLHHAFVDSIFEQ	34.65
874	P14679	Tyrosinase	DPB1*47:01	385	399	IFLLHHAFVDSIFEQ	34.65
875	P14679	Tyrosinase	DRB1*11:03	496	510	VSLLCRHKRKQLPEE	34.76
876	P14679	Tyrosinase	DRB1*16:02	178	192	WMHYYVSMDALLGGS	34.80
877	P14679	Tyrosinase	DRB1*01:20	135	149	FAYLTLAKHTISSDY	34.87
878	P14679	Tyrosinase	DRB1*01:20	181	195	YYVSMDALLGGSEIW	34.90
879	P14679	Tyrosinase	DRB1*01:18	461	475	QDYIKSYLEQASRIW	34.94
880	P14679	Tyrosinase	DRB1*01:01	4	18	AVLYCLLWSFQTSAG	34.95
881	P14679	Tyrosinase	DRB1*03:01	142	156	KHTISSDYVIPIGTY	35.22
882	P14679	Tyrosinase	DRB1*03:04	142	156	KHTISSDYVIPIGTY	35.22
883	P14679	Tyrosinase	DRB1*03:13	142	156	KHTISSDYVIPIGTY	35.22
884	P14679	Tyrosinase	DRB1*11:01	135	149	FAYLTLAKHTISSDY	35.24
885	P14679	Tyrosinase	DRB1*11:10	135	149	FAYLTLAKHTISSDY	35.24
886	P14679	Tyrosinase	DRB1*11:12	135	149	FAYLTLAKHTISSDY	35.24
887	P14679	Tyrosinase	DRB1*11:28	135	149	FAYLTLAKHTISSDY	35.24
888	P14679	Tyrosinase	DRB1*11:29	135	149	FAYLTLAKHTISSDY	35.24
889	P14679	Tyrosinase	DRB1*11:49	135	149	FAYLTLAKHTISSDY	35.24
890	P14679	Tyrosinase	DRB1*11:62	135	149	FAYLTLAKHTISSDY	35.24
891	P14679	Tyrosinase	DRB1*11:74	135	149	FAYLTLAKHTISSDY	35.24
892	P14679	Tyrosinase	DRB1*13:05	135	149	FAYLTLAKHTISSDY	35.24
893	P14679	Tyrosinase	DRB1*13:14	135	149	FAYLTLAKHTISSDY	35.24
894	P14679	Tyrosinase	DRB1*13:50	135	149	FAYLTLAKHTISSDY	35.24
895	P14679	Tyrosinase	DPB1*15:01	421	435	NRESYMVPFIPLYRN	35.48

896	P14679	Tyrosinase	DRB1*01:20	364	378	NALHIYMNGTMSQVQ	35.55
897	P14679	Tyrosinase	DRB1*01:01	363	377	HNALHIYMNGTMSQV	35.60
898	P14679	Tyrosinase	DRB1*01:20	491	505	LLAGLVSLLCRHKRK	35.80
899	P14679	Tyrosinase	DRB1*01:02	484	498	VGAVLTALLAGLVSL	35.82
900	P14679	Tyrosinase	DPB1*33:01	418	432	IGHNRESYMVPFIPL	35.97
901	P14679	Tyrosinase	DPB1*71:01	418	432	IGHNRESYMVPFIPL	35.97
902	P14679	Tyrosinase	DRB1*10:01	473	487	RIWSWLLGAAMVGAV	36.16
903	P14679	Tyrosinase	DRB1*01:18	5	19	VLYCLLWSFQTSAGH	36.26
904	P14679	Tyrosinase	DRB1*16:09	133	147	KFFAYLTLAKHTISS	36.33
905	P14679	Tyrosinase	DRB1*15:15	132	146	DKFFAYLTLAKHTIS	36.67
906	P14679	Tyrosinase	DRB1*01:01	135	149	FAYLTLAKHTISSDY	36.68
907	P14679	Tyrosinase	DRB1*01:18	196	210	RDIDFAHEAPAFLPW	36.71
908	P14679	Tyrosinase	DRB1*04:08	180	194	HYYVSMDALLGGSEI	36.79
909	P14679	Tyrosinase	DRB1*01:29	482	496	AMVGAVLTALLAGLV	36.80
910	P14679	Tyrosinase	DRB1*15:01	365	379	ALHIYMNGTMSQVQG	37.02
911	P14679	Tyrosinase	DRB1*15:06	365	379	ALHIYMNGTMSQVQG	37.02
912	P14679	Tyrosinase	DRB1*01:11	470	484	QASRIWSWLLGAAMV	37.30
913	P14679	Tyrosinase	DRB1*01:29	486	500	AVLTALLAGLVSLLC	37.34
914	P14679	Tyrosinase	DPB1*15:01	420	434	HNRESYMVPFIPLYR	37.46
915	P14679	Tyrosinase	DRB1*01:20	57	71	NILLSNAPLGPQFPF	37.47
916	P14679	Tyrosinase	DRB1*01:18	476	490	SWLLGAAMVGAVLTA	37.57
917	P14679	Tyrosinase	DRB1*10:01	383	397	DPIFLLHHAFVDSIF	37.62
918	P14679	Tyrosinase	DRB1*01:18	3	17	LAVLYCLLWSFQTSA	37.78
919	P14679	Tyrosinase	DRB1*01:20	130	144	EKDKFFAYLTLAKHT	37.89
920	P14679	Tyrosinase	DRB1*12:16	131	145	KDKFFAYLTLAKHTI	37.91
921	P14679	Tyrosinase	DRB1*11:02	422	436	RESYMVPFIPLYRNG	37.93
922	P14679	Tyrosinase	DRB1*11:65	422	436	RESYMVPFIPLYRNG	37.93
923	P14679	Tyrosinase	DRB1*13:01	422	436	RESYMVPFIPLYRNG	37.93
924	P14679	Tyrosinase	DRB1*04:05	176	190	FVWMHYYVSMDALLG	38.00
925	P14679	Tyrosinase	DRB1*16:09	130	144	EKDKFFAYLTLAKHT	38.06
926	P14679	Tyrosinase	DRB1*04:01	177	191	VWMHYYVSMDALLGG	38.16
927	P14679	Tyrosinase	DRB1*11:02	421	435	NRESYMVPFIPLYRN	38.22
928	P14679	Tyrosinase	DRB1*11:65	421	435	NRESYMVPFIPLYRN	38.22
929	P14679	Tyrosinase	DRB1*13:01	421	435	NRESYMVPFIPLYRN	38.22
930	P14679	Tyrosinase	DRB1*04:05	177	191	VWMHYYVSMDALLGG	38.24
931	P14679	Tyrosinase	DRB1*01:18	381	395	ANDPIFLLHHAFVDS	38.25
932	P14679	Tyrosinase	DRB1*08:04	135	149	FAYLTLAKHTISSDY	38.34
933	P14679	Tyrosinase	DRB1*11:03	490	504	ALLAGLVSLLCRHKR	38.43
934	P14679	Tyrosinase	DRB1*01:24	489	503	TALLAGLVSLLCRHK	38.53
935	P14679	Tyrosinase	DRB1*16:02	179	193	MHYYVSMDALLGGSE	38.58
936	P14679	Tyrosinase	DRB1*13:21	135	149	FAYLTLAKHTISSDY	38.61
937	P14679	Tyrosinase	DRB1*01:24	133	147	KFFAYLTLAKHTISS	38.64
938	P14679	Tyrosinase	DRB1*01:20	463	477	YIKSYLEQASRIWSW	38.80

939	P14679	Tyrosinase	DRB1*12:16	132	146	DKFFAYLTLAKHTIS	38.91
940	P14679	Tyrosinase	DRB1*12:16	133	147	KFFAYLTLAKHTISS	38.94
941	P14679	Tyrosinase	DRB1*01:01	490	504	ALLAGLVSLLCRHKR	38.99
942	P14679	Tyrosinase	DRB1*10:01	384	398	PIFLLHHAFVDSIFE	39.16
943	P14679	Tyrosinase	DRB1*11:08	132	146	DKFFAYLTLAKHTIS	39.26
944	P14679	Tyrosinase	DPB1*02:01	389	403	HHAFVDSIFEQWLRR	39.39
945	P14679	Tyrosinase	DPB1*46:01	389	403	HHAFVDSIFEQWLRR	39.39
946	P14679	Tyrosinase	DPB1*81:01	389	403	HHAFVDSIFEQWLRR	39.39
947	P14679	Tyrosinase	DRB1*01:18	465	479	KSYLEQASRIWSWLL	39.46
948	P14679	Tyrosinase	DRB1*10:01	8	22	CLLWSFQTSAGHFPR	39.46
949	P14679	Tyrosinase	DRB1*13:21	130	144	EKDKFFAYLTLAKHT	39.49
950	P14679	Tyrosinase	DRB1*07:01	383	397	DPIFLLHHAFVDSIF	39.65
951	P14679	Tyrosinase	DRB1*01:20	363	377	HNALHIYMNGTMSQV	39.75
952	P14679	Tyrosinase	DRB1*11:14	362	376	MHNALHIYMNGTMSQ	39.88
953	P14679	Tyrosinase	DRB1*13:02	362	376	MHNALHIYMNGTMSQ	39.88
954	P14679	Tyrosinase	DRB1*13:23	362	376	MHNALHIYMNGTMSQ	39.88
955	P14679	Tyrosinase	DRB1*13:97	362	376	MHNALHIYMNGTMSQ	39.88
956	P14679	Tyrosinase	DPB1*33:01	127	141	SAPEKDKFFAYLTLA	39.98
957	P14679	Tyrosinase	DPB1*71:01	127	141	SAPEKDKFFAYLTLA	39.98
958	P14679	Tyrosinase	DPB1*33:01	170	184	INIYDLFVWMHYYVS	40.06
959	P14679	Tyrosinase	DPB1*71:01	170	184	INIYDLFVWMHYYVS	40.06
960	P14679	Tyrosinase	DRB1*04:05	178	192	WMHYYVSMDALLGGS	40.42
961	P14679	Tyrosinase	DRB1*01:20	470	484	QASRIWSWLLGAAMV	40.45
962	P14679	Tyrosinase	DRB1*01:01	129	143	PEKDKFFAYLTLAKH	40.52
963	P14679	Tyrosinase	DRB1*16:02	131	145	KDKFFAYLTLAKHTI	40.61
964	P14679	Tyrosinase	DRB1*01:01	5	19	VLYCLLWSFQTSAGH	40.64
965	P14679	Tyrosinase	DRB1*01:20	365	379	ALHIYMNGTMSQVQG	40.68
966	P14679	Tyrosinase	DRB1*01:01	53	67	GSCQNILLSNAPLGP	40.72
967	P14679	Tyrosinase	DRB1*11:04	495	509	LVSLLCRHKRKQLPE	40.74
968	P14679	Tyrosinase	DRB1*11:46	495	509	LVSLLCRHKRKQLPE	40.74
969	P14679	Tyrosinase	DRB1*11:58	495	509	LVSLLCRHKRKQLPE	40.74
970	P14679	Tyrosinase	DRB1*13:11	495	509	LVSLLCRHKRKQLPE	40.74
971	P14679	Tyrosinase	DPB1*72:01	421	435	NRESYMVPFIPLYRN	40.81
972	P14679	Tyrosinase	DRB1*10:01	460	474	FQDYIKSYLEQASRI	40.90
973	P14679	Tyrosinase	DPB1*33:01	176	190	FVWMHYYVSMDALLG	41.15
974	P14679	Tyrosinase	DPB1*71:01	176	190	FVWMHYYVSMDALLG	41.15
975	P14679	Tyrosinase	DRB1*16:02	177	191	VWMHYYVSMDALLGG	41.31
976	P14679	Tyrosinase	DRB1*11:01	427	441	VPFIPLYRNGDFFIS	41.52
977	P14679	Tyrosinase	DRB1*11:10	427	441	VPFIPLYRNGDFFIS	41.52
978	P14679	Tyrosinase	DRB1*11:12	427	441	VPFIPLYRNGDFFIS	41.52
979	P14679	Tyrosinase	DRB1*11:28	427	441	VPFIPLYRNGDFFIS	41.52
980	P14679	Tyrosinase	DRB1*11:29	427	441	VPFIPLYRNGDFFIS	41.52
981	P14679	Tyrosinase	DRB1*11:49	427	441	VPFIPLYRNGDFFIS	41.52

982	P14679	Tyrosinase	DRB1*11:62	427	441	VPFIPLYRNGDFFIS	41.52
983	P14679	Tyrosinase	DRB1*11:74	427	441	VPFIPLYRNGDFFIS	41.52
984	P14679	Tyrosinase	DRB1*13:05	427	441	VPFIPLYRNGDFFIS	41.52
985	P14679	Tyrosinase	DRB1*13:14	427	441	VPFIPLYRNGDFFIS	41.52
986	P14679	Tyrosinase	DRB1*13:50	427	441	VPFIPLYRNGDFFIS	41.52
987	P14679	Tyrosinase	DPB1*02:02	130	144	EKDKFFAYLTLAKHT	41.62
988	P14679	Tyrosinase	DPB1*47:01	130	144	EKDKFFAYLTLAKHT	41.62
989	P14679	Tyrosinase	DRB1*11:14	54	68	SCQNILLSNAPLGPQ	41.65
990	P14679	Tyrosinase	DRB1*13:02	54	68	SCQNILLSNAPLGPQ	41.65
991	P14679	Tyrosinase	DRB1*13:23	54	68	SCQNILLSNAPLGPQ	41.65
992	P14679	Tyrosinase	DRB1*13:97	54	68	SCQNILLSNAPLGPQ	41.65
993	P14679	Tyrosinase	DRB1*01:18	6	20	LYCLLWSFQTSAGHF	41.66
994	P14679	Tyrosinase	DPB1*02:02	421	435	NRESYMVPFIPLYRN	41.71
995	P14679	Tyrosinase	DPB1*47:01	421	435	NRESYMVPFIPLYRN	41.71
996	P14679	Tyrosinase	DRB1*11:42	490	504	ALLAGLVSLLCRHKR	41.77
997	P14679	Tyrosinase	DPB1*15:01	389	403	HHAFVDSIFEQWLRR	41.88
998	P14679	Tyrosinase	DRB1*07:01	384	398	PIFLLHHAFVDSIFE	41.90
999	P14679	Tyrosinase	DRB1*01:24	488	502	LTALLAGLVSLLCRH	42.01
1000	P14679	Tyrosinase	DRB1*01:24	482	496	AMVGAVLTALLAGLV	42.14
1001	P14679	Tyrosinase	DRB1*13:96	364	378	NALHIYMNGTMSQVQ	42.15
1002	P14679	Tyrosinase	DRB1*11:01	425	439	YMVPFIPLYRNGDFF	42.20
1003	P14679	Tyrosinase	DRB1*11:10	425	439	YMVPFIPLYRNGDFF	42.20
1004	P14679	Tyrosinase	DRB1*11:12	425	439	YMVPFIPLYRNGDFF	42.20
1005	P14679	Tyrosinase	DRB1*11:28	425	439	YMVPFIPLYRNGDFF	42.20
1006	P14679	Tyrosinase	DRB1*11:29	425	439	YMVPFIPLYRNGDFF	42.20
1007	P14679	Tyrosinase	DRB1*11:49	425	439	YMVPFIPLYRNGDFF	42.20
1008	P14679	Tyrosinase	DRB1*11:62	425	439	YMVPFIPLYRNGDFF	42.20
1009	P14679	Tyrosinase	DRB1*11:74	425	439	YMVPFIPLYRNGDFF	42.20
1010	P14679	Tyrosinase	DRB1*13:05	425	439	YMVPFIPLYRNGDFF	42.20
1011	P14679	Tyrosinase	DRB1*13:14	425	439	YMVPFIPLYRNGDFF	42.20
1012	P14679	Tyrosinase	DRB1*13:50	425	439	YMVPFIPLYRNGDFF	42.20
1013	P14679	Tyrosinase	DRB1*08:01	426	440	MVPFIPLYRNGDFFI	42.22
1014	P14679	Tyrosinase	DRB1*11:08	134	148	FFAYLTLAKHTISSD	42.23
1015	P14679	Tyrosinase	DRB1*01:01	461	475	QDYIKSYLEQASRIW	42.27
1016	P14679	Tyrosinase	DPB1*02:02	390	404	HAFVDSIFEQWLRRH	42.36
1017	P14679	Tyrosinase	DPB1*47:01	390	404	HAFVDSIFEQWLRRH	42.36
1018	P14679	Tyrosinase	DRB1*04:01	180	194	HYYVSMDALLGGSEI	42.50
1019	P14679	Tyrosinase	DRB1*10:01	483	497	MVGAVLTALLAGLVS	42.59
1020	P14679	Tyrosinase	DRB1*15:15	133	147	KFFAYLTLAKHTISS	42.63
1021	P14679	Tyrosinase	DRB1*01:29	489	503	TALLAGLVSLLCRHK	42.73
1022	P14679	Tyrosinase	DRB1*10:01	182	196	YVSMDALLGGSEIWR	42.74
1023	P14679	Tyrosinase	DPB1*02:02	381	395	ANDPIFLLHHAFVDS	42.84
1024	P14679	Tyrosinase	DPB1*47:01	381	395	ANDPIFLLHHAFVDS	42.84

1025	P14679	Tyrosinase	DRB1*01:01	366	380	LHIYMNGTMSQVQGS	42.87
1026	P14679	Tyrosinase	DRB1*03:01	141	155	AKHTISSDYVIPIGT	42.94
1027	P14679	Tyrosinase	DRB1*03:04	141	155	AKHTISSDYVIPIGT	42.94
1028	P14679	Tyrosinase	DRB1*03:13	141	155	AKHTISSDYVIPIGT	42.94
1029	P14679	Tyrosinase	DPB1*72:01	420	434	HNRESYMVPFIPLYR	43.00
1030	P14679	Tyrosinase	DPB1*02:02	131	145	KDKFFAYLTLAKHTI	43.02
1031	P14679	Tyrosinase	DPB1*47:01	131	145	KDKFFAYLTLAKHTI	43.02
1032	P14679	Tyrosinase	DPB1*02:02	420	434	HNRESYMVPFIPLYR	43.13
1033	P14679	Tyrosinase	DPB1*47:01	420	434	HNRESYMVPFIPLYR	43.13
1034	P14679	Tyrosinase	DRB1*10:01	198	212	IDFAHEAPAFLPWHR	43.41
1035	P14679	Tyrosinase	DRB1*04:08	176	190	FVWMHYYVSMDALLG	43.64
1036	P14679	Tyrosinase	DPB1*02:01	381	395	ANDPIFLLHHAFVDS	43.78
1037	P14679	Tyrosinase	DPB1*46:01	381	395	ANDPIFLLHHAFVDS	43.78
1038	P14679	Tyrosinase	DPB1*81:01	381	395	ANDPIFLLHHAFVDS	43.78
1039	P14679	Tyrosinase	DPB1*02:01	421	435	NRESYMVPFIPLYRN	43.82
1040	P14679	Tyrosinase	DPB1*46:01	421	435	NRESYMVPFIPLYRN	43.82
1041	P14679	Tyrosinase	DPB1*81:01	421	435	NRESYMVPFIPLYRN	43.82
1042	P14679	Tyrosinase	DRB1*01:18	366	380	LHIYMNGTMSQVQGS	43.90
1043	P14679	Tyrosinase	DRB1*01:18	9	23	LLWSFQTSAGHFPRA	43.93
1044	P14679	Tyrosinase	DRB1*11:42	422	436	RESYMVPFIPLYRNG	43.98
1045	P14679	Tyrosinase	DRB1*01:29	474	488	IWSWLLGAAMVGAVL	43.99
1046	P14679	Tyrosinase	DRB1*11:42	132	146	DKFFAYLTLAKHTIS	43.99
1047	P14679	Tyrosinase	DRB1*01:01	475	489	WSWLLGAAMVGAVLT	44.08
1048	P14679	Tyrosinase	DPB1*04:01	391	405	AFVDSIFEQWLRRHR	44.17
1049	P14679	Tyrosinase	DPB1*126:01	391	405	AFVDSIFEQWLRRHR	44.17
1050	P14679	Tyrosinase	DPB1*23:01	391	405	AFVDSIFEQWLRRHR	44.17
1051	P14679	Tyrosinase	DPB1*39:01	391	405	AFVDSIFEQWLRRHR	44.17
1052	P14679	Tyrosinase	DRB1*01:01	383	397	DPIFLLHHAFVDSIF	44.27
1053	P14679	Tyrosinase	DRB1*10:01	135	149	FAYLTLAKHTISSDY	44.28
1054	P14679	Tyrosinase	DRB1*11:03	396	410	IFEQWLRRHRPLQEV	44.32
1055	P14679	Tyrosinase	DRB1*01:01	195	209	WRDIDFAHEAPAFLP	44.47
1056	P14679	Tyrosinase	DRB1*16:09	129	143	PEKDKFFAYLTLAKH	44.53
1057	P14679	Tyrosinase	DRB1*11:04	490	504	ALLAGLVSLLCRHKR	44.71
1058	P14679	Tyrosinase	DRB1*11:46	490	504	ALLAGLVSLLCRHKR	44.71
1059	P14679	Tyrosinase	DRB1*11:58	490	504	ALLAGLVSLLCRHKR	44.71
1060	P14679	Tyrosinase	DRB1*13:11	490	504	ALLAGLVSLLCRHKR	44.71
1061	P14679	Tyrosinase	DRB1*11:42	424	438	SYMVPFIPLYRNGDF	44.75
1062	P14679	Tyrosinase	DRB1*11:13	133	147	KFFAYLTLAKHTISS	44.78
1063	P14679	Tyrosinase	DRB1*01:20	462	476	DYIKSYLEQASRIWS	44.80
1064	P14679	Tyrosinase	DRB1*11:84	493	507	AGLVSLLCRHKRKQL	44.98
1065	P14679	Tyrosinase	DRB1*01:24	132	146	DKFFAYLTLAKHTIS	45.00
1066	P14679	Tyrosinase	DRB1*01:01	476	490	SWLLGAAMVGAVLTA	45.12
1067	P14679	Tyrosinase	DRB1*01:18	491	505	LLAGLVSLLCRHKRK	45.21

1068	P14679	Tyrosinase	DPB1*02:02	129	143	PEKDKFFAYLTLAKH	45.23
1069	P14679	Tyrosinase	DPB1*47:01	129	143	PEKDKFFAYLTLAKH	45.23
1070	P14679	Tyrosinase	DPB1*04:01	386	400	FLLHHAFVDSIFEQW	45.32
1071	P14679	Tyrosinase	DPB1*126:01	386	400	FLLHHAFVDSIFEQW	45.32
1072	P14679	Tyrosinase	DPB1*23:01	386	400	FLLHHAFVDSIFEQW	45.32
1073	P14679	Tyrosinase	DPB1*39:01	386	400	FLLHHAFVDSIFEQW	45.32
1074	P14679	Tyrosinase	DRB1*11:13	135	149	FAYLTLAKHTISSDY	45.35
1075	P14679	Tyrosinase	DPB1*02:01	420	434	HNRESYMVPFIPLYR	45.51
1076	P14679	Tyrosinase	DPB1*46:01	420	434	HNRESYMVPFIPLYR	45.51
1077	P14679	Tyrosinase	DPB1*81:01	420	434	HNRESYMVPFIPLYR	45.51
1078	P14679	Tyrosinase	DRB1*04:05	179	193	MHYYVSMDALLGGSE	45.55
1079	P14679	Tyrosinase	DRB1*01:29	134	148	FFAYLTLAKHTISSD	45.71
1080	P14679	Tyrosinase	DRB1*11:42	423	437	ESYMVPFIPLYRNGD	45.74
1081	P14679	Tyrosinase	DPB1*02:02	173	187	YDLFVWMHYYVSMDA	45.78
1082	P14679	Tyrosinase	DPB1*47:01	173	187	YDLFVWMHYYVSMDA	45.78
1083	P14679	Tyrosinase	DRB1*09:01	383	397	DPIFLLHHAFVDSIF	45.80
1084	P14679	Tyrosinase	DRB1*01:01	9	23	LLWSFQTSAGHFPRA	45.88
1085	P14679	Tyrosinase	DRB1*01:18	57	71	NILLSNAPLGPQFPF	45.90
1086	P14679	Tyrosinase	DRB1*15:07	363	377	HNALHIYMNGTMSQV	45.90
1087	P14679	Tyrosinase	DRB1*08:04	133	147	KFFAYLTLAKHTISS	45.91
1088	P14679	Tyrosinase	DRB1*11:37	133	147	KFFAYLTLAKHTISS	45.92
1089	P14679	Tyrosinase	DRB1*13:07	133	147	KFFAYLTLAKHTISS	45.92
1090	P14679	Tyrosinase	DPB1*33:01	210	224	WHRLFLLRWEQEIQK	46.08
1091	P14679	Tyrosinase	DPB1*71:01	210	224	WHRLFLLRWEQEIQK	46.08
1092	P14679	Tyrosinase	DRB1*11:42	495	509	LVSLLCRHKRKQLPE	46.08
1093	P14679	Tyrosinase	DPB1*04:01	387	401	LLHHAFVDSIFEQWL	46.15
1094	P14679	Tyrosinase	DPB1*126:01	387	401	LLHHAFVDSIFEQWL	46.15
1095	P14679	Tyrosinase	DPB1*23:01	387	401	LLHHAFVDSIFEQWL	46.15
1096	P14679	Tyrosinase	DPB1*39:01	387	401	LLHHAFVDSIFEQWL	46.15
1097	P14679	Tyrosinase	DRB1*11:14	142	156	KHTISSDYVIPIGTY	46.16
1098	P14679	Tyrosinase	DRB1*13:02	142	156	KHTISSDYVIPIGTY	46.16
1099	P14679	Tyrosinase	DRB1*13:23	142	156	KHTISSDYVIPIGTY	46.16
1100	P14679	Tyrosinase	DRB1*13:97	142	156	KHTISSDYVIPIGTY	46.16
1101	P14679	Tyrosinase	DRB1*01:01	3	17	LAVLYCLLWSFQTSA	46.23
1102	P14679	Tyrosinase	DRB1*11:04	464	478	IKSYLEQASRIWSWL	46.23
1103	P14679	Tyrosinase	DRB1*11:46	464	478	IKSYLEQASRIWSWL	46.23
1104	P14679	Tyrosinase	DRB1*11:58	464	478	IKSYLEQASRIWSWL	46.23
1105	P14679	Tyrosinase	DRB1*13:11	464	478	IKSYLEQASRIWSWL	46.23
1106	P14679	Tyrosinase	DRB1*16:01	131	145	KDKFFAYLTLAKHTI	46.43
1107	P14679	Tyrosinase	DRB1*01:29	487	501	VLTALLAGLVSLLCR	46.60
1108	P14679	Tyrosinase	DRB1*07:01	382	396	NDPIFLLHHAFVDSI	46.64
1109	P14679	Tyrosinase	DRB1*08:04	134	148	FFAYLTLAKHTISSD	46.68
1110	P14679	Tyrosinase	DPB1*02:01	131	145	KDKFFAYLTLAKHTI	46.71

1111	P14679	Tyrosinase	DPB1*46:01	131	145	KDKFFAYLTLAKHTI	46.71
1112	P14679	Tyrosinase	DPB1*81:01	131	145	KDKFFAYLTLAKHTI	46.71
1113	P14679	Tyrosinase	DRB1*11:04	132	146	DKFFAYLTLAKHTIS	46.72
1114	P14679	Tyrosinase	DRB1*11:13	134	148	FFAYLTLAKHTISSD	46.72
1115	P14679	Tyrosinase	DRB1*11:46	132	146	DKFFAYLTLAKHTIS	46.72
1116	P14679	Tyrosinase	DRB1*11:58	132	146	DKFFAYLTLAKHTIS	46.72
1117	P14679	Tyrosinase	DRB1*13:11	132	146	DKFFAYLTLAKHTIS	46.72
1118	P14679	Tyrosinase	DRB1*16:09	178	192	WMHYYVSMDALLGGS	46.83
1119	P14679	Tyrosinase	DRB1*11:13	493	507	AGLVSLLCRHKRKQL	46.93
1120	P14679	Tyrosinase	DPB1*72:01	391	405	AFVDSIFEQWLRRHR	46.99
1121	P14679	Tyrosinase	DRB1*11:13	422	436	RESYMVPFIPLYRNG	47.02
1122	P14679	Tyrosinase	DRB1*01:29	130	144	EKDKFFAYLTLAKHT	47.03
1123	P14679	Tyrosinase	DRB1*10:01	382	396	NDPIFLLHHAFVDSI	47.07
1124	P14679	Tyrosinase	DRB1*11:03	397	411	FEQWLRRHRPLQEVY	47.11
1125	P14679	Tyrosinase	DRB1*01:24	131	145	KDKFFAYLTLAKHTI	47.12
1126	P14679	Tyrosinase	DRB1*15:02	131	145	KDKFFAYLTLAKHTI	47.17
1127	P14679	Tyrosinase	DRB1*11:14	57	71	NILLSNAPLGPQFPF	47.26
1128	P14679	Tyrosinase	DRB1*13:02	57	71	NILLSNAPLGPQFPF	47.26
1129	P14679	Tyrosinase	DRB1*13:23	57	71	NILLSNAPLGPQFPF	47.26
1130	P14679	Tyrosinase	DRB1*13:97	57	71	NILLSNAPLGPQFPF	47.26
1131	P14679	Tyrosinase	DPB1*33:01	423	437	ESYMVPFIPLYRNGD	47.37
1132	P14679	Tyrosinase	DPB1*71:01	423	437	ESYMVPFIPLYRNGD	47.37
1133	P14679	Tyrosinase	DPB1*33:01	126	140	LSAPEKDKFFAYLTL	47.38
1134	P14679	Tyrosinase	DPB1*71:01	126	140	LSAPEKDKFFAYLTL	47.38
1135	P14679	Tyrosinase	DRB1*10:01	484	498	VGAVLTALLAGLVSL	47.38
1136	P14679	Tyrosinase	DPB1*33:01	203	217	EAPAFLPWHRLFLLR	47.39
1137	P14679	Tyrosinase	DPB1*71:01	203	217	EAPAFLPWHRLFLLR	47.39
1138	P14679	Tyrosinase	DRB1*10:01	469	483	EQASRIWSWLLGAAM	47.39
1139	P14679	Tyrosinase	DRB1*01:11	486	500	AVLTALLAGLVSLLC	47.46
1140	P14679	Tyrosinase	DRB1*01:01	384	398	PIFLLHHAFVDSIFE	47.49
1141	P14679	Tyrosinase	DRB1*11:13	423	437	ESYMVPFIPLYRNGD	47.63
1142	P14679	Tyrosinase	DRB1*01:20	176	190	FVWMHYYVSMDALLG	47.64
1143	P14679	Tyrosinase	DPB1*72:01	386	400	FLLHHAFVDSIFEQW	47.66
1144	P14679	Tyrosinase	DRB1*13:96	363	377	HNALHIYMNGTMSQV	47.70
1145	P14679	Tyrosinase	DRB1*13:96	365	379	ALHIYMNGTMSQVQG	47.79
1146	P14679	Tyrosinase	DRB1*09:01	384	398	PIFLLHHAFVDSIFE	47.97
1147	P14679	Tyrosinase	DPB1*15:01	422	436	RESYMVPFIPLYRNG	48.09
1148	P14679	Tyrosinase	DPB1*02:01	390	404	HAFVDSIFEQWLRRH	48.15
1149	P14679	Tyrosinase	DPB1*46:01	390	404	HAFVDSIFEQWLRRH	48.15
1150	P14679	Tyrosinase	DPB1*81:01	390	404	HAFVDSIFEQWLRRH	48.15
1151	P14679	Tyrosinase	DRB1*08:01	427	441	VPFIPLYRNGDFFIS	48.28
1152	P14679	Tyrosinase	DRB1*11:03	395	409	SIFEQWLRRHRPLQE	48.36
1153	P14679	Tyrosinase	DRB1*01:02	485	499	GAVLTALLAGLVSLL	48.43

1154	P14679	Tyrosinase	DRB1*01:20	422	436	RESYMVPFIPLYRNG	48.43
1155	P14679	Tyrosinase	DRB1*11:04	465	479	KSYLEQASRIWSWLL	48.45
1156	P14679	Tyrosinase	DRB1*11:46	465	479	KSYLEQASRIWSWLL	48.45
1157	P14679	Tyrosinase	DRB1*11:58	465	479	KSYLEQASRIWSWLL	48.45
1158	P14679	Tyrosinase	DRB1*13:11	465	479	KSYLEQASRIWSWLL	48.45
1159	P14679	Tyrosinase	DRB1*01:01	465	479	KSYLEQASRIWSWLL	48.71
1160	P14679	Tyrosinase	DRB1*10:01	364	378	NALHIYMNGTMSQVQ	48.73
1161	P14679	Tyrosinase	DRB1*01:01	6	20	LYCLLWSFQTSAGHF	48.74
1162	P14679	Tyrosinase	DPB1*33:01	261	275	NLLSPASFFSSWQIV	48.78
1163	P14679	Tyrosinase	DPB1*71:01	261	275	NLLSPASFFSSWQIV	48.78
1164	P14679	Tyrosinase	DRB1*10:01	489	503	TALLAGLVSLLCRHK	48.81
1165	P14679	Tyrosinase	DRB1*01:20	421	435	NRESYMVPFIPLYRN	48.82
1166	P14679	Tyrosinase	DPB1*04:01	420	434	HNRESYMVPFIPLYR	48.89
1167	P14679	Tyrosinase	DPB1*126:01	420	434	HNRESYMVPFIPLYR	48.89
1168	P14679	Tyrosinase	DPB1*23:01	420	434	HNRESYMVPFIPLYR	48.89
1169	P14679	Tyrosinase	DPB1*39:01	420	434	HNRESYMVPFIPLYR	48.89
1170	P14679	Tyrosinase	DPB1*33:01	133	147	KFFAYLTLAKHTISS	48.90
1171	P14679	Tyrosinase	DPB1*71:01	133	147	KFFAYLTLAKHTISS	48.90
1172	P14679	Tyrosinase	DPB1*33:01	264	278	SPASFFSSWQIVCSR	49.00
1173	P14679	Tyrosinase	DPB1*71:01	264	278	SPASFFSSWQIVCSR	49.00
1174	P14679	Tyrosinase	DRB1*11:08	131	145	KDKFFAYLTLAKHTI	49.00
1175	P14679	Tyrosinase	DRB1*11:42	465	479	KSYLEQASRIWSWLL	49.02
1176	P14679	Tyrosinase	DRB1*11:42	421	435	NRESYMVPFIPLYRN	49.18
1177	P14679	Tyrosinase	DRB1*16:01	178	192	WMHYYVSMDALLGGS	49.25
1178	P14679	Tyrosinase	DRB1*14:32	382	396	NDPIFLLHHAFVDSI	49.29
1179	P14679	Tyrosinase	DRB1*01:24	469	483	EQASRIWSWLLGAAM	49.40
1180	P14679	Tyrosinase	DPB1*02:01	130	144	EKDKFFAYLTLAKHT	49.43
1181	P14679	Tyrosinase	DPB1*46:01	130	144	EKDKFFAYLTLAKHT	49.43
1182	P14679	Tyrosinase	DPB1*81:01	130	144	EKDKFFAYLTLAKHT	49.43
1183	P14679	Tyrosinase	DRB1*01:01	57	71	NILLSNAPLGPQFPF	49.45
1184	P14679	Tyrosinase	DRB1*11:42	464	478	IKSYLEQASRIWSWL	49.53
1185	P14679	Tyrosinase	DRB1*01:11	133	147	KFFAYLTLAKHTISS	49.54
1186	P14679	Tyrosinase	DPB1*04:01	421	435	NRESYMVPFIPLYRN	49.59
1187	P14679	Tyrosinase	DPB1*126:01	421	435	NRESYMVPFIPLYRN	49.59
1188	P14679	Tyrosinase	DPB1*23:01	421	435	NRESYMVPFIPLYRN	49.59
1189	P14679	Tyrosinase	DPB1*39:01	421	435	NRESYMVPFIPLYRN	49.59
1190	P14679	Tyrosinase	DRB1*11:03	113	127	TERRLLVRRNIFDLS	49.60
1191	P14679	Tyrosinase	DRB1*01:20	52	66	RGSCQNILLSNAPLG	49.63
1192	P14679	Tyrosinase	DRB1*01:18	385	399	IFLLHHAFVDSIFEQ	49.67
1193	P14679	Tyrosinase	DRB1*11:02	490	504	ALLAGLVSLLCRHKR	49.75
1194	P14679	Tyrosinase	DRB1*11:65	490	504	ALLAGLVSLLCRHKR	49.75
1195	P14679	Tyrosinase	DRB1*13:01	490	504	ALLAGLVSLLCRHKR	49.75
1196	P14679	Tyrosinase	DRB1*10:01	7	21	YCLLWSFQTSAGHFP	49.84

1197	P14679	Tyrosinase	DRB1*01:24	4	18	AVLYCLLWSFQTSAG	49.86
1198	P14679	Tyrosinase	DRB1*10:01	266	280	ASFFSSWQIVCSRLE	49.92
1199	P17643	TRP1	DPB1*33:01	184	198	IYNYFVWTHYYSVKK	3.50
1200	P17643	TRP1	DPB1*71:01	184	198	IYNYFVWTHYYSVKK	3.50
1201	P17643	TRP1	DPB1*33:01	185	199	YNYFVWTHYYSVKKT	3.63
1202	P17643	TRP1	DPB1*71:01	185	199	YNYFVWTHYYSVKKT	3.63
1203	P17643	TRP1	DPB1*33:01	183	197	SIYNYFVWTHYYSVK	3.78
1204	P17643	TRP1	DPB1*71:01	183	197	SIYNYFVWTHYYSVK	3.78
1205	P17643	TRP1	DPB1*33:01	186	200	NYFVWTHYYSVKKTF	4.16
1206	P17643	TRP1	DPB1*71:01	186	200	NYFVWTHYYSVKKTF	4.16
1207	P17643	TRP1	DPB1*04:01	184	198	IYNYFVWTHYYSVKK	4.56
1208	P17643	TRP1	DPB1*126:01	184	198	IYNYFVWTHYYSVKK	4.56
1209	P17643	TRP1	DPB1*23:01	184	198	IYNYFVWTHYYSVKK	4.56
1210	P17643	TRP1	DPB1*39:01	184	198	IYNYFVWTHYYSVKK	4.56
1211	P17643	TRP1	DPB1*02:01	184	198	IYNYFVWTHYYSVKK	4.57
1212	P17643	TRP1	DPB1*46:01	184	198	IYNYFVWTHYYSVKK	4.57
1213	P17643	TRP1	DPB1*81:01	184	198	IYNYFVWTHYYSVKK	4.57
1214	P17643	TRP1	DPB1*02:01	185	199	YNYFVWTHYYSVKKT	4.64
1215	P17643	TRP1	DPB1*46:01	185	199	YNYFVWTHYYSVKKT	4.64
1216	P17643	TRP1	DPB1*81:01	185	199	YNYFVWTHYYSVKKT	4.64
1217	P17643	TRP1	DPB1*04:01	185	199	YNYFVWTHYYSVKKT	4.79
1218	P17643	TRP1	DPB1*126:01	185	199	YNYFVWTHYYSVKKT	4.79
1219	P17643	TRP1	DPB1*23:01	185	199	YNYFVWTHYYSVKKT	4.79
1220	P17643	TRP1	DPB1*39:01	185	199	YNYFVWTHYYSVKKT	4.79
1221	P17643	TRP1	DPB1*33:01	182	196	ISIYNYFVWTHYYSV	4.95
1222	P17643	TRP1	DPB1*71:01	182	196	ISIYNYFVWTHYYSV	4.95
1223	P17643	TRP1	DPB1*33:01	187	201	YFVWTHYYSVKKTFL	5.02
1224	P17643	TRP1	DPB1*71:01	187	201	YFVWTHYYSVKKTFL	5.02
1225	P17643	TRP1	DPB1*04:01	183	197	SIYNYFVWTHYYSVK	5.03
1226	P17643	TRP1	DPB1*126:01	183	197	SIYNYFVWTHYYSVK	5.03
1227	P17643	TRP1	DPB1*23:01	183	197	SIYNYFVWTHYYSVK	5.03
1228	P17643	TRP1	DPB1*39:01	183	197	SIYNYFVWTHYYSVK	5.03
1229	P17643	TRP1	DPB1*72:01	184	198	IYNYFVWTHYYSVKK	5.16
1230	P17643	TRP1	DPB1*02:02	184	198	IYNYFVWTHYYSVKK	5.36
1231	P17643	TRP1	DPB1*47:01	184	198	IYNYFVWTHYYSVKK	5.36
1232	P17643	TRP1	DPB1*72:01	185	199	YNYFVWTHYYSVKKT	5.37
1233	P17643	TRP1	DPB1*02:02	185	199	YNYFVWTHYYSVKKT	5.48
1234	P17643	TRP1	DPB1*47:01	185	199	YNYFVWTHYYSVKKT	5.48
1235	P17643	TRP1	DPB1*02:01	186	200	NYFVWTHYYSVKKTF	5.50
1236	P17643	TRP1	DPB1*46:01	186	200	NYFVWTHYYSVKKTF	5.50
1237	P17643	TRP1	DPB1*81:01	186	200	NYFVWTHYYSVKKTF	5.50
1238	P17643	TRP1	DPB1*02:01	183	197	SIYNYFVWTHYYSVK	5.66
1239	P17643	TRP1	DPB1*46:01	183	197	SIYNYFVWTHYYSVK	5.66

1240	P17643	TRP1	DPB1*81:01	183	197	SIYNYFVWTHYYSVK	5.66
1241	P17643	TRP1	DPB1*72:01	183	197	SIYNYFVWTHYYSVK	6.08
1242	P17643	TRP1	DPB1*04:01	186	200	NYFVWTHYYSVKKTF	6.23
1243	P17643	TRP1	DPB1*126:01	186	200	NYFVWTHYYSVKKTF	6.23
1244	P17643	TRP1	DPB1*23:01	186	200	NYFVWTHYYSVKKTF	6.23
1245	P17643	TRP1	DPB1*39:01	186	200	NYFVWTHYYSVKKTF	6.23
1246	P17643	TRP1	DPB1*02:02	183	197	SIYNYFVWTHYYSVK	6.31
1247	P17643	TRP1	DPB1*47:01	183	197	SIYNYFVWTHYYSVK	6.31
1248	P17643	TRP1	DPB1*02:02	186	200	NYFVWTHYYSVKKTF	6.63
1249	P17643	TRP1	DPB1*47:01	186	200	NYFVWTHYYSVKKTF	6.63
1250	P17643	TRP1	DPB1*33:01	217	231	GPAFLTWHRYHLLRL	6.81
1251	P17643	TRP1	DPB1*71:01	217	231	GPAFLTWHRYHLLRL	6.81
1252	P17643	TRP1	DPB1*33:01	218	232	PAFLTWHRYHLLRLE	6.92
1253	P17643	TRP1	DPB1*71:01	218	232	PAFLTWHRYHLLRLE	6.92
1254	P17643	TRP1	DPB1*72:01	186	200	NYFVWTHYYSVKKTF	6.93
1255	P17643	TRP1	DPB1*04:01	182	196	ISIYNYFVWTHYYSV	6.95
1256	P17643	TRP1	DPB1*126:01	182	196	ISIYNYFVWTHYYSV	6.95
1257	P17643	TRP1	DPB1*23:01	182	196	ISIYNYFVWTHYYSV	6.95
1258	P17643	TRP1	DPB1*39:01	182	196	ISIYNYFVWTHYYSV	6.95
1259	P17643	TRP1	DPB1*02:01	187	201	YFVWTHYYSVKKTFL	7.20
1260	P17643	TRP1	DPB1*46:01	187	201	YFVWTHYYSVKKTFL	7.20
1261	P17643	TRP1	DPB1*81:01	187	201	YFVWTHYYSVKKTFL	7.20
1262	P17643	TRP1	DPB1*33:01	219	233	AFLTWHRYHLLRLEK	7.44
1263	P17643	TRP1	DPB1*71:01	219	233	AFLTWHRYHLLRLEK	7.44
1264	P17643	TRP1	DPB1*33:01	216	230	EGPAFLTWHRYHLLR	8.30
1265	P17643	TRP1	DPB1*71:01	216	230	EGPAFLTWHRYHLLR	8.30
1266	P17643	TRP1	DPB1*02:02	187	201	YFVWTHYYSVKKTFL	8.43
1267	P17643	TRP1	DPB1*47:01	187	201	YFVWTHYYSVKKTFL	8.43
1268	P17643	TRP1	DPB1*72:01	182	196	ISIYNYFVWTHYYSV	9.01
1269	P17643	TRP1	DPB1*04:01	187	201	YFVWTHYYSVKKTFL	9.08
1270	P17643	TRP1	DPB1*126:01	187	201	YFVWTHYYSVKKTFL	9.08
1271	P17643	TRP1	DPB1*23:01	187	201	YFVWTHYYSVKKTFL	9.08
1272	P17643	TRP1	DPB1*39:01	187	201	YFVWTHYYSVKKTFL	9.08
1273	P17643	TRP1	DPB1*02:01	182	196	ISIYNYFVWTHYYSV	9.41
1274	P17643	TRP1	DPB1*46:01	182	196	ISIYNYFVWTHYYSV	9.41
1275	P17643	TRP1	DPB1*81:01	182	196	ISIYNYFVWTHYYSV	9.41
1276	P17643	TRP1	DPB1*02:02	182	196	ISIYNYFVWTHYYSV	9.43
1277	P17643	TRP1	DPB1*47:01	182	196	ISIYNYFVWTHYYSV	9.43
1278	P17643	TRP1	DRB1*07:01	491	505	ALIFGTASYLIRARR	9.91
1279	P17643	TRP1	DPB1*33:01	215	229	HEGPAFLTWHRYHLL	10.01
1280	P17643	TRP1	DPB1*71:01	215	229	HEGPAFLTWHRYHLL	10.01
1281	P17643	TRP1	DRB1*07:01	490	504	VALIFGTASYLIRAR	10.07
1282	P17643	TRP1	DRB1*01:18	490	504	VALIFGTASYLIRAR	10.35

1283	P17643	TRP1	DPB1*33:01	220	234	FLTWHRYHLLRLEKD	10.71
1284	P17643	TRP1	DPB1*71:01	220	234	FLTWHRYHLLRLEKD	10.71
1285	P17643	TRP1	DPB1*72:01	187	201	YFVWTHYYSVKKTFL	10.89
1286	P17643	TRP1	DRB1*01:20	14	28	FPLLLFQQARAQFPR	11.09
1287	P17643	TRP1	DRB1*01:20	490	504	VALIFGTASYLIRAR	11.10
1288	P17643	TRP1	DPB1*41:01	185	199	YNYFVWTHYYSVKKT	11.48
1289	P17643	TRP1	DRB1*13:21	157	171	PLFVIATRRSEEILG	11.53
1290	P17643	TRP1	DPB1*41:01	184	198	IYNYFVWTHYYSVKK	11.61
1291	P17643	TRP1	DRB1*13:21	156	170	HPLFVIATRRSEEIL	12.00
1292	P17643	TRP1	DRB1*01:01	490	504	VALIFGTASYLIRAR	12.15
1293	P17643	TRP1	DRB1*01:01	15	29	PLLLFQQARAQFPRQ	12.28
1294	P17643	TRP1	DRB1*01:18	489	503	LVALIFGTASYLIRA	12.34
1295	P17643	TRP1	DRB1*11:04	156	170	HPLFVIATRRSEEIL	12.52
1296	P17643	TRP1	DRB1*11:46	156	170	HPLFVIATRRSEEIL	12.52
1297	P17643	TRP1	DRB1*11:58	156	170	HPLFVIATRRSEEIL	12.52
1298	P17643	TRP1	DRB1*13:11	156	170	HPLFVIATRRSEEIL	12.52
1299	P17643	TRP1	DRB1*11:04	155	169	THPLFVIATRRSEEI	12.59
1300	P17643	TRP1	DRB1*11:46	155	169	THPLFVIATRRSEEI	12.59
1301	P17643	TRP1	DRB1*11:58	155	169	THPLFVIATRRSEEI	12.59
1302	P17643	TRP1	DRB1*13:11	155	169	THPLFVIATRRSEEI	12.59
1303	P17643	TRP1	DRB1*01:18	15	29	PLLLFQQARAQFPRQ	12.64
1304	P17643	TRP1	DPB1*02:02	217	231	GPAFLTWHRYHLLRL	12.96
1305	P17643	TRP1	DPB1*47:01	217	231	GPAFLTWHRYHLLRL	12.96
1306	P17643	TRP1	DPB1*02:01	217	231	GPAFLTWHRYHLLRL	13.09
1307	P17643	TRP1	DPB1*46:01	217	231	GPAFLTWHRYHLLRL	13.09
1308	P17643	TRP1	DPB1*81:01	217	231	GPAFLTWHRYHLLRL	13.09
1309	P17643	TRP1	DPB1*16:01	184	198	IYNYFVWTHYYSVKK	13.16
1310	P17643	TRP1	DRB1*01:18	14	28	FPLLLFQQARAQFPR	13.19
1311	P17643	TRP1	DRB1*01:20	13	27	FFPLLLFQQARAQFP	13.29
1312	P17643	TRP1	DPB1*16:01	185	199	YNYFVWTHYYSVKKT	13.47
1313	P17643	TRP1	DPB1*02:02	218	232	PAFLTWHRYHLLRLE	13.50
1314	P17643	TRP1	DPB1*47:01	218	232	PAFLTWHRYHLLRLE	13.50
1315	P17643	TRP1	DRB1*13:21	158	172	LFVIATRRSEEILGP	13.50
1316	P17643	TRP1	DRB1*14:32	124	138	QRVLIVRRNLLDLSK	13.60
1317	P17643	TRP1	DRB1*07:01	492	506	LIFGTASYLIRARRS	13.71
1318	P17643	TRP1	DRB1*14:32	123	137	DQRVLIVRRNLLDLS	13.71
1319	P17643	TRP1	DRB1*11:01	156	170	HPLFVIATRRSEEIL	13.74
1320	P17643	TRP1	DRB1*11:10	156	170	HPLFVIATRRSEEIL	13.74
1321	P17643	TRP1	DRB1*11:12	156	170	HPLFVIATRRSEEIL	13.74
1322	P17643	TRP1	DRB1*11:28	156	170	HPLFVIATRRSEEIL	13.74
1323	P17643	TRP1	DRB1*11:29	156	170	HPLFVIATRRSEEIL	13.74
1324	P17643	TRP1	DRB1*11:49	156	170	HPLFVIATRRSEEIL	13.74
1325	P17643	TRP1	DRB1*11:62	156	170	HPLFVIATRRSEEIL	13.74

1326	P17643	TRP1	DRB1*11:74	156	170	HPLFVIATRRSEEIL	13.74
1327	P17643	TRP1	DRB1*13:05	156	170	HPLFVIATRRSEEIL	13.74
1328	P17643	TRP1	DRB1*13:14	156	170	HPLFVIATRRSEEIL	13.74
1329	P17643	TRP1	DRB1*13:50	156	170	HPLFVIATRRSEEIL	13.74
1330	P17643	TRP1	DRB1*01:18	491	505	ALIFGTASYLIRARR	13.82
1331	P17643	TRP1	DPB1*04:02	184	198	IYNYFVWTHYYSVKK	13.91
1332	P17643	TRP1	DPB1*105:01	184	198	IYNYFVWTHYYSVKK	13.91
1333	P17643	TRP1	DPB1*49:01	184	198	IYNYFVWTHYYSVKK	13.91
1334	P17643	TRP1	DRB1*13:21	155	169	THPLFVIATRRSEEI	13.94
1335	P17643	TRP1	DRB1*01:01	14	28	FPLLLFQQARAQFPR	14.01
1336	P17643	TRP1	DRB1*14:32	122	136	CDQRVLIVRRNLLDL	14.12
1337	P17643	TRP1	DPB1*15:01	184	198	IYNYFVWTHYYSVKK	14.20
1338	P17643	TRP1	DPB1*02:01	218	232	PAFLTWHRYHLLRLE	14.24
1339	P17643	TRP1	DPB1*46:01	218	232	PAFLTWHRYHLLRLE	14.24
1340	P17643	TRP1	DPB1*81:01	218	232	PAFLTWHRYHLLRLE	14.24
1341	P17643	TRP1	DRB1*11:42	155	169	THPLFVIATRRSEEI	14.39
1342	P17643	TRP1	DRB1*01:20	491	505	ALIFGTASYLIRARR	14.40
1343	P17643	TRP1	DPB1*41:01	186	200	NYFVWTHYYSVKKTF	14.41
1344	P17643	TRP1	DPB1*04:02	185	199	YNYFVWTHYYSVKKT	14.45
1345	P17643	TRP1	DPB1*105:01	185	199	YNYFVWTHYYSVKKT	14.45
1346	P17643	TRP1	DPB1*49:01	185	199	YNYFVWTHYYSVKKT	14.45
1347	P17643	TRP1	DRB1*11:42	123	137	DQRVLIVRRNLLDLS	14.53
1348	P17643	TRP1	DRB1*01:20	15	29	PLLLFQQARAQFPRQ	14.56
1349	P17643	TRP1	DPB1*02:02	219	233	AFLTWHRYHLLRLEK	14.60
1350	P17643	TRP1	DPB1*47:01	219	233	AFLTWHRYHLLRLEK	14.60
1351	P17643	TRP1	DRB1*11:42	124	138	QRVLIVRRNLLDLSK	14.60
1352	P17643	TRP1	DRB1*11:13	124	138	QRVLIVRRNLLDLSK	14.61
1353	P17643	TRP1	DRB1*10:01	396	410	NDPIFVLLHTFTDAV	14.63
1354	P17643	TRP1	DRB1*04:05	395	409	PNDPIFVLLHTFTDA	14.77
1355	P17643	TRP1	DPB1*15:01	185	199	YNYFVWTHYYSVKKT	14.80
1356	P17643	TRP1	DRB1*10:01	15	29	PLLLFQQARAQFPRQ	14.83
1357	P17643	TRP1	DRB1*13:21	153	167	RTTHPLFVIATRRSE	14.84
1358	P17643	TRP1	DRB1*10:01	397	411	DPIFVLLHTFTDAVF	14.96
1359	P17643	TRP1	DRB1*04:05	396	410	NDPIFVLLHTFTDAV	15.01
1360	P17643	TRP1	DRB1*07:01	489	503	LVALIFGTASYLIRA	15.15
1361	P17643	TRP1	DRB1*01:01	489	503	LVALIFGTASYLIRA	15.16
1362	P17643	TRP1	DRB1*11:01	155	169	THPLFVIATRRSEEI	15.19
1363	P17643	TRP1	DRB1*11:10	155	169	THPLFVIATRRSEEI	15.19
1364	P17643	TRP1	DRB1*11:12	155	169	THPLFVIATRRSEEI	15.19
1365	P17643	TRP1	DRB1*11:28	155	169	THPLFVIATRRSEEI	15.19
1366	P17643	TRP1	DRB1*11:29	155	169	THPLFVIATRRSEEI	15.19
1367	P17643	TRP1	DRB1*11:49	155	169	THPLFVIATRRSEEI	15.19
1368	P17643	TRP1	DRB1*11:62	155	169	THPLFVIATRRSEEI	15.19

1369	P17643	TRP1	DRB1*11:74	155	169	THPLFVIATRRSEEI	15.19
1370	P17643	TRP1	DRB1*13:05	155	169	THPLFVIATRRSEEI	15.19
1371	P17643	TRP1	DRB1*13:14	155	169	THPLFVIATRRSEEI	15.19
1372	P17643	TRP1	DRB1*13:50	155	169	THPLFVIATRRSEEI	15.19
1373	P17643	TRP1	DPB1*02:01	219	233	AFLTWHRYHLLRLEK	15.20
1374	P17643	TRP1	DPB1*46:01	219	233	AFLTWHRYHLLRLEK	15.20
1375	P17643	TRP1	DPB1*81:01	219	233	AFLTWHRYHLLRLEK	15.20
1376	P17643	TRP1	DRB1*11:13	123	137	DQRVLIVRRNLLDLS	15.21
1377	P17643	TRP1	DPB1*01:01	184	198	IYNYFVWTHYYSVKK	15.24
1378	P17643	TRP1	DRB1*11:01	157	171	PLFVIATRRSEEILG	15.42
1379	P17643	TRP1	DRB1*11:10	157	171	PLFVIATRRSEEILG	15.42
1380	P17643	TRP1	DRB1*11:12	157	171	PLFVIATRRSEEILG	15.42
1381	P17643	TRP1	DRB1*11:28	157	171	PLFVIATRRSEEILG	15.42
1382	P17643	TRP1	DRB1*11:29	157	171	PLFVIATRRSEEILG	15.42
1383	P17643	TRP1	DRB1*11:49	157	171	PLFVIATRRSEEILG	15.42
1384	P17643	TRP1	DRB1*11:62	157	171	PLFVIATRRSEEILG	15.42
1385	P17643	TRP1	DRB1*11:74	157	171	PLFVIATRRSEEILG	15.42
1386	P17643	TRP1	DRB1*13:05	157	171	PLFVIATRRSEEILG	15.42
1387	P17643	TRP1	DRB1*13:14	157	171	PLFVIATRRSEEILG	15.42
1388	P17643	TRP1	DRB1*13:50	157	171	PLFVIATRRSEEILG	15.42
1389	P17643	TRP1	DRB1*11:42	156	170	HPLFVIATRRSEEIL	15.43
1390	P17643	TRP1	DRB1*11:04	154	168	TTHPLFVIATRRSEE	15.45
1391	P17643	TRP1	DRB1*11:46	154	168	TTHPLFVIATRRSEE	15.45
1392	P17643	TRP1	DRB1*11:58	154	168	TTHPLFVIATRRSEE	15.45
1393	P17643	TRP1	DRB1*13:11	154	168	TTHPLFVIATRRSEE	15.45
1394	P17643	TRP1	DRB1*11:42	122	136	CDQRVLIVRRNLLDL	15.47
1395	P17643	TRP1	DRB1*01:20	489	503	LVALIFGTASYLIRA	15.60
1396	P17643	TRP1	DRB1*07:01	488	502	LLVALIFGTASYLIR	15.71
1397	P17643	TRP1	DRB1*11:13	122	136	CDQRVLIVRRNLLDL	15.74
1398	P17643	TRP1	DRB1*14:32	121	135	ACDQRVLIVRRNLLD	15.85
1399	P17643	TRP1	DPB1*02:02	216	230	EGPAFLTWHRYHLLR	16.04
1400	P17643	TRP1	DPB1*47:01	216	230	EGPAFLTWHRYHLLR	16.04
1401	P17643	TRP1	DRB1*04:05	397	411	DPIFVLLHTFTDAVF	16.23
1402	P17643	TRP1	DRB1*10:01	14	28	FPLLLFQQARAQFPR	16.30
1403	P17643	TRP1	DRB1*13:21	154	168	TTHPLFVIATRRSEE	16.40
1404	P17643	TRP1	DRB1*11:04	157	171	PLFVIATRRSEEILG	16.46
1405	P17643	TRP1	DRB1*11:46	157	171	PLFVIATRRSEEILG	16.46
1406	P17643	TRP1	DRB1*11:58	157	171	PLFVIATRRSEEILG	16.46
1407	P17643	TRP1	DRB1*13:11	157	171	PLFVIATRRSEEILG	16.46
1408	P17643	TRP1	DPB1*01:01	185	199	YNYFVWTHYYSVKKT	16.47
1409	P17643	TRP1	DRB1*01:01	16	30	LLLFQQARAQFPRQC	16.54
1410	P17643	TRP1	DPB1*33:01	188	202	FVWTHYYSVKKTFLG	16.66
1411	P17643	TRP1	DPB1*71:01	188	202	FVWTHYYSVKKTFLG	16.66

1412	P17643	TRP1	DRB1*10:01	395	409	PNDPIFVLLHTFTDA	16.67
1413	P17643	TRP1	DPB1*02:01	216	230	EGPAFLTWHRYHLLR	16.72
1414	P17643	TRP1	DPB1*46:01	216	230	EGPAFLTWHRYHLLR	16.72
1415	P17643	TRP1	DPB1*81:01	216	230	EGPAFLTWHRYHLLR	16.72
1416	P17643	TRP1	DRB1*14:32	120	134	AACDQRVLIVRRNLL	16.78
1417	P17643	TRP1	DRB1*01:18	488	502	LLVALIFGTASYLIR	16.91
1418	P17643	TRP1	DPB1*15:01	183	197	SIYNYFVWTHYYSVK	16.92
1419	P17643	TRP1	DRB1*11:42	154	168	TTHPLFVIATRRSEE	17.13
1420	P17643	TRP1	DRB1*11:04	153	167	RTTHPLFVIATRRSE	17.16
1421	P17643	TRP1	DRB1*11:46	153	167	RTTHPLFVIATRRSE	17.16
1422	P17643	TRP1	DRB1*11:58	153	167	RTTHPLFVIATRRSE	17.16
1423	P17643	TRP1	DRB1*13:11	153	167	RTTHPLFVIATRRSE	17.16
1424	P17643	TRP1	DRB1*01:18	16	30	LLLFQQARAQFPRQC	17.26
1425	P17643	TRP1	DPB1*01:01	183	197	SIYNYFVWTHYYSVK	17.30
1426	P17643	TRP1	DRB1*01:01	491	505	ALIFGTASYLIRARR	17.30
1427	P17643	TRP1	DRB1*01:20	12	26	IFFPLLLFQQARAQF	17.40
1428	P17643	TRP1	DRB1*11:04	124	138	QRVLIVRRNLLDLSK	17.56
1429	P17643	TRP1	DRB1*11:46	124	138	QRVLIVRRNLLDLSK	17.56
1430	P17643	TRP1	DRB1*11:58	124	138	QRVLIVRRNLLDLSK	17.56
1431	P17643	TRP1	DRB1*13:11	124	138	QRVLIVRRNLLDLSK	17.56
1432	P17643	TRP1	DPB1*40:01	184	198	IYNYFVWTHYYSVKK	17.63
1433	P17643	TRP1	DRB1*11:42	121	135	ACDQRVLIVRRNLLD	17.73
1434	P17643	TRP1	DPB1*04:02	183	197	SIYNYFVWTHYYSVK	18.01
1435	P17643	TRP1	DPB1*105:01	183	197	SIYNYFVWTHYYSVK	18.01
1436	P17643	TRP1	DPB1*49:01	183	197	SIYNYFVWTHYYSVK	18.01
1437	P17643	TRP1	DPB1*41:01	183	197	SIYNYFVWTHYYSVK	18.13
1438	P17643	TRP1	DRB1*11:13	121	135	ACDQRVLIVRRNLLD	18.40
1439	P17643	TRP1	DPB1*16:01	186	200	NYFVWTHYYSVKKTF	18.61
1440	P17643	TRP1	DPB1*40:01	185	199	YNYFVWTHYYSVKKT	18.64
1441	P17643	TRP1	DRB1*01:18	13	27	FFPLLLFQQARAQFP	18.79
1442	P17643	TRP1	DRB1*01:20	155	169	THPLFVIATRRSEEI	19.04
1443	P17643	TRP1	DRB1*14:32	125	139	RVLIVRRNLLDLSKE	19.06
1444	P17643	TRP1	DRB1*11:04	123	137	DQRVLIVRRNLLDLS	19.07
1445	P17643	TRP1	DRB1*11:46	123	137	DQRVLIVRRNLLDLS	19.07
1446	P17643	TRP1	DRB1*11:58	123	137	DQRVLIVRRNLLDLS	19.07
1447	P17643	TRP1	DRB1*13:11	123	137	DQRVLIVRRNLLDLS	19.07
1448	P17643	TRP1	DPB1*34:01	184	198	IYNYFVWTHYYSVKK	19.23
1449	P17643	TRP1	DPB1*16:01	183	197	SIYNYFVWTHYYSVK	19.26
1450	P17643	TRP1	DRB1*01:29	490	504	VALIFGTASYLIRAR	19.31
1451	P17643	TRP1	DRB1*11:42	153	167	RTTHPLFVIATRRSE	19.38
1452	P17643	TRP1	DRB1*11:01	154	168	TTHPLFVIATRRSEE	19.39
1453	P17643	TRP1	DRB1*11:10	154	168	TTHPLFVIATRRSEE	19.39
1454	P17643	TRP1	DRB1*11:12	154	168	TTHPLFVIATRRSEE	19.39

1455	P17643	TRP1	DRB1*11:28	154	168	TTHPLFVIATRRSEE	19.39
1456	P17643	TRP1	DRB1*11:29	154	168	TTHPLFVIATRRSEE	19.39
1457	P17643	TRP1	DRB1*11:49	154	168	TTHPLFVIATRRSEE	19.39
1458	P17643	TRP1	DRB1*11:62	154	168	TTHPLFVIATRRSEE	19.39
1459	P17643	TRP1	DRB1*11:74	154	168	TTHPLFVIATRRSEE	19.39
1460	P17643	TRP1	DRB1*13:05	154	168	TTHPLFVIATRRSEE	19.39
1461	P17643	TRP1	DRB1*13:14	154	168	TTHPLFVIATRRSEE	19.39
1462	P17643	TRP1	DRB1*13:50	154	168	TTHPLFVIATRRSEE	19.39
1463	P17643	TRP1	DRB1*01:24	490	504	VALIFGTASYLIRAR	19.40
1464	P17643	TRP1	DRB1*04:05	394	408	SPNDPIFVLLHTFTD	19.43
1465	P17643	TRP1	DRB1*11:13	125	139	RVLIVRRNLLDLSKE	19.51
1466	P17643	TRP1	DRB1*10:01	398	412	PIFVLLHTFTDAVFD	19.59
1467	P17643	TRP1	DPB1*34:01	185	199	YNYFVWTHYYSVKKT	19.84
1468	P17643	TRP1	DRB1*07:01	184	198	IYNYFVWTHYYSVKK	20.23
1469	P17643	TRP1	DRB1*11:02	124	138	QRVLIVRRNLLDLSK	20.24
1470	P17643	TRP1	DRB1*11:65	124	138	QRVLIVRRNLLDLSK	20.24
1471	P17643	TRP1	DRB1*13:01	124	138	QRVLIVRRNLLDLSK	20.24
1472	P17643	TRP1	DRB1*11:04	122	136	CDQRVLIVRRNLLDL	20.29
1473	P17643	TRP1	DRB1*11:46	122	136	CDQRVLIVRRNLLDL	20.29
1474	P17643	TRP1	DRB1*11:58	122	136	CDQRVLIVRRNLLDL	20.29
1475	P17643	TRP1	DRB1*13:11	122	136	CDQRVLIVRRNLLDL	20.29
1476	P17643	TRP1	DRB1*13:21	496	510	TASYLIRARRSMDEA	20.41
1477	P17643	TRP1	DRB1*04:05	398	412	PIFVLLHTFTDAVFD	20.52
1478	P17643	TRP1	DRB1*01:24	489	503	LVALIFGTASYLIRA	20.63
1479	P17643	TRP1	DRB1*10:01	16	30	LLLFQQARAQFPRQC	20.68
1480	P17643	TRP1	DRB1*01:20	156	170	HPLFVIATRRSEEIL	20.71
1481	P17643	TRP1	DPB1*15:01	186	200	NYFVWTHYYSVKKTF	20.85
1482	P17643	TRP1	DRB1*01:01	488	502	LLVALIFGTASYLIR	20.95
1483	P17643	TRP1	DPB1*04:02	186	200	NYFVWTHYYSVKKTF	21.07
1484	P17643	TRP1	DPB1*105:01	186	200	NYFVWTHYYSVKKTF	21.07
1485	P17643	TRP1	DPB1*49:01	186	200	NYFVWTHYYSVKKTF	21.07
1486	P17643	TRP1	DPB1*40:01	183	197	SIYNYFVWTHYYSVK	21.08
1487	P17643	TRP1	DRB1*07:01	185	199	YNYFVWTHYYSVKKT	21.34
1488	P17643	TRP1	DPB1*02:02	220	234	FLTWHRYHLLRLEKD	21.67
1489	P17643	TRP1	DPB1*47:01	220	234	FLTWHRYHLLRLEKD	21.67
1490	P17643	TRP1	DPB1*02:02	215	229	HEGPAFLTWHRYHLL	21.73
1491	P17643	TRP1	DPB1*47:01	215	229	HEGPAFLTWHRYHLL	21.73
1492	P17643	TRP1	DRB1*01:20	488	502	LLVALIFGTASYLIR	21.86
1493	P17643	TRP1	DRB1*11:42	14	28	FPLLLFQQARAQFPR	22.15
1494	P17643	TRP1	DRB1*01:20	370	384	DPAVRSLHNLAHLFL	22.20
1495	P17643	TRP1	DRB1*01:20	371	385	PAVRSLHNLAHLFLN	22.26
1496	P17643	TRP1	DPB1*41:01	187	201	YFVWTHYYSVKKTFL	22.39
1497	P17643	TRP1	DRB1*04:04	155	169	THPLFVIATRRSEEI	22.73

1498	P17643	TRP1	DRB1*11:42	157	171	PLFVIATRRSEEILG	22.73
1499	P17643	TRP1	DRB1*14:01	120	134	AACDQRVLIVRRNLL	22.80
1500	P17643	TRP1	DRB1*14:54	120	134	AACDQRVLIVRRNLL	22.80
1501	P17643	TRP1	DRB1*04:01	450	464	TEMFVTAPDNLGYTY	22.85
1502	P17643	TRP1	DRB1*01:18	155	169	THPLFVIATRRSEEI	22.89
1503	P17643	TRP1	DRB1*01:20	153	167	RTTHPLFVIATRRSE	22.89
1504	P17643	TRP1	DRB1*13:21	497	511	ASYLIRARRSMDEAN	22.90
1505	P17643	TRP1	DRB1*04:01	451	465	EMFVTAPDNLGYTYE	22.96
1506	P17643	TRP1	DRB1*15:01	490	504	VALIFGTASYLIRAR	22.98
1507	P17643	TRP1	DRB1*15:06	490	504	VALIFGTASYLIRAR	22.98
1508	P17643	TRP1	DPB1*02:01	220	234	FLTWHRYHLLRLEKD	23.00
1509	P17643	TRP1	DPB1*46:01	220	234	FLTWHRYHLLRLEKD	23.00
1510	P17643	TRP1	DPB1*81:01	220	234	FLTWHRYHLLRLEKD	23.00
1511	P17643	TRP1	DRB1*11:02	123	137	DQRVLIVRRNLLDLS	23.00
1512	P17643	TRP1	DRB1*11:65	123	137	DQRVLIVRRNLLDLS	23.00
1513	P17643	TRP1	DRB1*13:01	123	137	DQRVLIVRRNLLDLS	23.00
1514	P17643	TRP1	DRB1*01:18	397	411	DPIFVLLHTFTDAVF	23.02
1515	P17643	TRP1	DRB1*14:01	124	138	QRVLIVRRNLLDLSK	23.02
1516	P17643	TRP1	DRB1*14:54	124	138	QRVLIVRRNLLDLSK	23.02
1517	P17643	TRP1	DRB1*11:42	125	139	RVLIVRRNLLDLSKE	23.19
1518	P17643	TRP1	DPB1*01:01	182	196	ISIYNYFVWTHYYSV	23.26
1519	P17643	TRP1	DRB1*04:04	370	384	DPAVRSLHNLAHLFL	23.28
1520	P17643	TRP1	DRB1*01:20	16	30	LLLFQQARAQFPRQC	23.29
1521	P17643	TRP1	DRB1*01:20	154	168	TTHPLFVIATRRSEE	23.55
1522	P17643	TRP1	DPB1*01:01	186	200	NYFVWTHYYSVKKTF	23.72
1523	P17643	TRP1	DRB1*11:04	496	510	TASYLIRARRSMDEA	23.81
1524	P17643	TRP1	DRB1*11:46	496	510	TASYLIRARRSMDEA	23.81
1525	P17643	TRP1	DRB1*11:58	496	510	TASYLIRARRSMDEA	23.81
1526	P17643	TRP1	DRB1*13:11	496	510	TASYLIRARRSMDEA	23.81
1527	P17643	TRP1	DRB1*11:13	120	134	AACDQRVLIVRRNLL	23.87
1528	P17643	TRP1	DRB1*01:18	12	26	IFFPLLLFQQARAQF	23.88
1529	P17643	TRP1	DRB1*11:42	120	134	AACDQRVLIVRRNLL	24.06
1530	P17643	TRP1	DRB1*11:01	158	172	LFVIATRRSEEILGP	24.07
1531	P17643	TRP1	DRB1*11:10	158	172	LFVIATRRSEEILGP	24.07
1532	P17643	TRP1	DRB1*11:12	158	172	LFVIATRRSEEILGP	24.07
1533	P17643	TRP1	DRB1*11:28	158	172	LFVIATRRSEEILGP	24.07
1534	P17643	TRP1	DRB1*11:29	158	172	LFVIATRRSEEILGP	24.07
1535	P17643	TRP1	DRB1*11:49	158	172	LFVIATRRSEEILGP	24.07
1536	P17643	TRP1	DRB1*11:62	158	172	LFVIATRRSEEILGP	24.07
1537	P17643	TRP1	DRB1*11:74	158	172	LFVIATRRSEEILGP	24.07
1538	P17643	TRP1	DRB1*13:05	158	172	LFVIATRRSEEILGP	24.07
1539	P17643	TRP1	DRB1*13:14	158	172	LFVIATRRSEEILGP	24.07
1540	P17643	TRP1	DRB1*13:50	158	172	LFVIATRRSEEILGP	24.07

1541	P17643	TRP1	DRB1*11:14	344	358	TPPFYSNSTNSFRNT	24.09
1542	P17643	TRP1	DRB1*13:02	344	358	TPPFYSNSTNSFRNT	24.09
1543	P17643	TRP1	DRB1*13:23	344	358	TPPFYSNSTNSFRNT	24.09
1544	P17643	TRP1	DRB1*13:97	344	358	TPPFYSNSTNSFRNT	24.09
1545	P17643	TRP1	DRB1*13:21	495	509	GTASYLIRARRSMDE	24.11
1546	P17643	TRP1	DRB1*04:08	450	464	TEMFVTAPDNLGYTY	24.17
1547	P17643	TRP1	DRB1*11:04	121	135	ACDQRVLIVRRNLLD	24.43
1548	P17643	TRP1	DRB1*11:46	121	135	ACDQRVLIVRRNLLD	24.43
1549	P17643	TRP1	DRB1*11:58	121	135	ACDQRVLIVRRNLLD	24.43
1550	P17643	TRP1	DRB1*13:11	121	135	ACDQRVLIVRRNLLD	24.43
1551	P17643	TRP1	DRB1*11:01	153	167	RTTHPLFVIATRRSE	24.44
1552	P17643	TRP1	DRB1*11:10	153	167	RTTHPLFVIATRRSE	24.44
1553	P17643	TRP1	DRB1*11:12	153	167	RTTHPLFVIATRRSE	24.44
1554	P17643	TRP1	DRB1*11:28	153	167	RTTHPLFVIATRRSE	24.44
1555	P17643	TRP1	DRB1*11:29	153	167	RTTHPLFVIATRRSE	24.44
1556	P17643	TRP1	DRB1*11:49	153	167	RTTHPLFVIATRRSE	24.44
1557	P17643	TRP1	DRB1*11:62	153	167	RTTHPLFVIATRRSE	24.44
1558	P17643	TRP1	DRB1*11:74	153	167	RTTHPLFVIATRRSE	24.44
1559	P17643	TRP1	DRB1*13:05	153	167	RTTHPLFVIATRRSE	24.44
1560	P17643	TRP1	DRB1*13:14	153	167	RTTHPLFVIATRRSE	24.44
1561	P17643	TRP1	DRB1*13:50	153	167	RTTHPLFVIATRRSE	24.44
1562	P17643	TRP1	DRB1*01:01	246	260	LPYWNFATGKNVCDI	24.53
1563	P17643	TRP1	DRB1*01:29	489	503	LVALIFGTASYLIRA	24.53
1564	P17643	TRP1	DRB1*08:04	155	169	THPLFVIATRRSEEI	24.66
1565	P17643	TRP1	DPB1*15:01	182	196	ISIYNYFVWTHYYSV	24.75
1566	P17643	TRP1	DRB1*01:01	13	27	FFPLLLFQQARAQFP	24.79
1567	P17643	TRP1	DRB1*01:20	492	506	LIFGTASYLIRARRS	24.92
1568	P17643	TRP1	DPB1*19:01	184	198	IYNYFVWTHYYSVKK	25.06
1569	P17643	TRP1	DPB1*40:01	186	200	NYFVWTHYYSVKKTF	25.18
1570	P17643	TRP1	DRB1*04:08	451	465	EMFVTAPDNLGYTYE	25.27
1571	P17643	TRP1	DRB1*11:03	155	169	THPLFVIATRRSEEI	25.30
1572	P17643	TRP1	DRB1*11:02	122	136	CDQRVLIVRRNLLDL	25.37
1573	P17643	TRP1	DRB1*11:65	122	136	CDQRVLIVRRNLLDL	25.37
1574	P17643	TRP1	DRB1*13:01	122	136	CDQRVLIVRRNLLDL	25.37
1575	P17643	TRP1	DRB1*07:01	183	197	SIYNYFVWTHYYSVK	25.38
1576	P17643	TRP1	DRB1*11:42	496	510	TASYLIRARRSMDEA	25.57
1577	P17643	TRP1	DRB1*01:29	491	505	ALIFGTASYLIRARR	25.63
1578	P17643	TRP1	DRB1*01:11	490	504	VALIFGTASYLIRAR	25.64
1579	P17643	TRP1	DRB1*10:01	13	27	FFPLLLFQQARAQFP	25.64
1580	P17643	TRP1	DPB1*34:01	186	200	NYFVWTHYYSVKKTF	25.70
1581	P17643	TRP1	DRB1*04:04	371	385	PAVRSLHNLAHLFLN	25.72
1582	P17643	TRP1	DRB1*14:01	122	136	CDQRVLIVRRNLLDL	25.73
1583	P17643	TRP1	DRB1*14:54	122	136	CDQRVLIVRRNLLDL	25.73

1584	P17643	TRP1	DRB1*11:14	343	357	DTPPFYSNSTNSFRN	25.79
1585	P17643	TRP1	DRB1*13:02	343	357	DTPPFYSNSTNSFRN	25.79
1586	P17643	TRP1	DRB1*13:23	343	357	DTPPFYSNSTNSFRN	25.79
1587	P17643	TRP1	DRB1*13:97	343	357	DTPPFYSNSTNSFRN	25.79
1588	P17643	TRP1	DPB1*34:01	183	197	SIYNYFVWTHYYSVK	25.95
1589	P17643	TRP1	DPB1*16:01	187	201	YFVWTHYYSVKKTFL	25.97
1590	P17643	TRP1	DPB1*19:01	185	199	YNYFVWTHYYSVKKT	25.99
1591	P17643	TRP1	DRB1*01:29	15	29	PLLLFQQARAQFPRQ	25.99
1592	P17643	TRP1	DRB1*08:04	156	170	HPLFVIATRRSEEIL	25.99
1593	P17643	TRP1	DRB1*01:18	156	170	HPLFVIATRRSEEIL	26.05
1594	P17643	TRP1	DRB1*11:03	156	170	HPLFVIATRRSEEIL	26.12
1595	P17643	TRP1	DRB1*04:04	153	167	RTTHPLFVIATRRSE	26.14
1596	P17643	TRP1	DRB1*04:04	156	170	HPLFVIATRRSEEIL	26.21
1597	P17643	TRP1	DRB1*11:42	494	508	FGTASYLIRARRSMD	26.27
1598	P17643	TRP1	DRB1*14:01	123	137	DQRVLIVRRNLLDLS	26.29
1599	P17643	TRP1	DRB1*14:54	123	137	DQRVLIVRRNLLDLS	26.29
1600	P17643	TRP1	DPB1*02:01	215	229	HEGPAFLTWHRYHLL	26.31
1601	P17643	TRP1	DPB1*46:01	215	229	HEGPAFLTWHRYHLL	26.31
1602	P17643	TRP1	DPB1*81:01	215	229	HEGPAFLTWHRYHLL	26.31
1603	P17643	TRP1	DRB1*01:18	396	410	NDPIFVLLHTFTDAV	26.39
1604	P17643	TRP1	DRB1*13:21	498	512	SYLIRARRSMDEANQ	26.61
1605	P17643	TRP1	DRB1*11:03	124	138	QRVLIVRRNLLDLSK	26.66
1606	P17643	TRP1	DRB1*01:18	246	260	LPYWNFATGKNVCDI	26.73
1607	P17643	TRP1	DRB1*11:04	14	28	FPLLLFQQARAQFPR	26.77
1608	P17643	TRP1	DRB1*11:46	14	28	FPLLLFQQARAQFPR	26.77
1609	P17643	TRP1	DRB1*11:58	14	28	FPLLLFQQARAQFPR	26.77
1610	P17643	TRP1	DRB1*13:11	14	28	FPLLLFQQARAQFPR	26.77
1611	P17643	TRP1	DRB1*11:42	493	507	IFGTASYLIRARRSM	26.88
1612	P17643	TRP1	DRB1*11:08	156	170	HPLFVIATRRSEEIL	26.90
1613	P17643	TRP1	DRB1*11:42	495	509	GTASYLIRARRSMDE	26.94
1614	P17643	TRP1	DRB1*01:18	398	412	PIFVLLHTFTDAVFD	27.00
1615	P17643	TRP1	DRB1*01:29	14	28	FPLLLFQQARAQFPR	27.06
1616	P17643	TRP1	DRB1*11:04	125	139	RVLIVRRNLLDLSKE	27.27
1617	P17643	TRP1	DRB1*11:46	125	139	RVLIVRRNLLDLSKE	27.27
1618	P17643	TRP1	DRB1*11:58	125	139	RVLIVRRNLLDLSKE	27.27
1619	P17643	TRP1	DRB1*13:11	125	139	RVLIVRRNLLDLSKE	27.27
1620	P17643	TRP1	DRB1*04:04	154	168	TTHPLFVIATRRSEE	27.30
1621	P17643	TRP1	DRB1*01:18	492	506	LIFGTASYLIRARRS	27.38
1622	P17643	TRP1	DRB1*11:42	15	29	PLLLFQQARAQFPRQ	27.38
1623	P17643	TRP1	DPB1*33:01	221	235	LTWHRYHLLRLEKDM	27.66
1624	P17643	TRP1	DPB1*71:01	221	235	LTWHRYHLLRLEKDM	27.66
1625	P17643	TRP1	DRB1*01:01	17	31	LLFQQARAQFPRQCA	27.70
1626	P17643	TRP1	DRB1*11:08	155	169	THPLFVIATRRSEEI	27.70

1627	P17643	TRP1	DRB1*15:15	185	199	YNYFVWTHYYSVKKT	27.93
1628	P17643	TRP1	DRB1*11:03	493	507	IFGTASYLIRARRSM	28.00
1629	P17643	TRP1	DRB1*11:42	13	27	FFPLLLFQQARAQFP	28.09
1630	P17643	TRP1	DRB1*01:18	153	167	RTTHPLFVIATRRSE	28.14
1631	P17643	TRP1	DRB1*01:18	154	168	TTHPLFVIATRRSEE	28.19
1632	P17643	TRP1	DRB1*11:14	342	356	FDTPPFYSNSTNSFR	28.28
1633	P17643	TRP1	DRB1*13:02	342	356	FDTPPFYSNSTNSFR	28.28
1634	P17643	TRP1	DRB1*13:23	342	356	FDTPPFYSNSTNSFR	28.28
1635	P17643	TRP1	DRB1*13:97	342	356	FDTPPFYSNSTNSFR	28.28
1636	P17643	TRP1	DRB1*07:01	487	501	LLLVALIFGTASYLI	28.35
1637	P17643	TRP1	DRB1*04:01	449	463	NTEMFVTAPDNLGYT	28.47
1638	P17643	TRP1	DRB1*11:03	123	137	DQRVLIVRRNLLDLS	28.49
1639	P17643	TRP1	DRB1*01:20	11	25	CIFFPLLLFQQARAQ	28.61
1640	P17643	TRP1	DRB1*01:24	488	502	LLVALIFGTASYLIR	28.65
1641	P17643	TRP1	DRB1*11:04	497	511	ASYLIRARRSMDEAN	28.67
1642	P17643	TRP1	DRB1*11:46	497	511	ASYLIRARRSMDEAN	28.67
1643	P17643	TRP1	DRB1*11:58	497	511	ASYLIRARRSMDEAN	28.67
1644	P17643	TRP1	DRB1*13:11	497	511	ASYLIRARRSMDEAN	28.67
1645	P17643	TRP1	DRB1*07:01	186	200	NYFVWTHYYSVKKTF	28.79
1646	P17643	TRP1	DRB1*01:11	489	503	LVALIFGTASYLIRA	28.86
1647	P17643	TRP1	DRB1*01:24	15	29	PLLLFQQARAQFPRQ	28.91
1648	P17643	TRP1	DRB1*14:01	121	135	ACDQRVLIVRRNLLD	28.98
1649	P17643	TRP1	DRB1*14:54	121	135	ACDQRVLIVRRNLLD	28.98
1650	P17643	TRP1	DRB1*11:42	497	511	ASYLIRARRSMDEAN	29.06
1651	P17643	TRP1	DRB1*01:18	371	385	PAVRSLHNLAHLFLN	29.20
1652	P17643	TRP1	DRB1*11:37	156	170	HPLFVIATRRSEEIL	29.24
1653	P17643	TRP1	DRB1*13:07	156	170	HPLFVIATRRSEEIL	29.24
1654	P17643	TRP1	DRB1*11:04	495	509	GTASYLIRARRSMDE	29.26
1655	P17643	TRP1	DRB1*11:46	495	509	GTASYLIRARRSMDE	29.26
1656	P17643	TRP1	DRB1*11:58	495	509	GTASYLIRARRSMDE	29.26
1657	P17643	TRP1	DRB1*13:11	495	509	GTASYLIRARRSMDE	29.26
1658	P17643	TRP1	DRB1*01:24	491	505	ALIFGTASYLIRARR	29.39
1659	P17643	TRP1	DRB1*11:04	120	134	AACDQRVLIVRRNLL	29.49
1660	P17643	TRP1	DRB1*11:46	120	134	AACDQRVLIVRRNLL	29.49
1661	P17643	TRP1	DRB1*11:58	120	134	AACDQRVLIVRRNLL	29.49
1662	P17643	TRP1	DRB1*13:11	120	134	AACDQRVLIVRRNLL	29.49
1663	P17643	TRP1	DRB1*01:20	157	171	PLFVIATRRSEEILG	29.52
1664	P17643	TRP1	DRB1*11:03	494	508	FGTASYLIRARRSMD	29.56
1665	P17643	TRP1	DRB1*15:01	491	505	ALIFGTASYLIRARR	29.58
1666	P17643	TRP1	DRB1*15:06	491	505	ALIFGTASYLIRARR	29.58
1667	P17643	TRP1	DRB1*08:04	154	168	TTHPLFVIATRRSEE	29.70
1668	P17643	TRP1	DRB1*07:01	187	201	YFVWTHYYSVKKTFL	29.71
1669	P17643	TRP1	DRB1*10:01	269	283	RSNFDSTLISPNSVF	30.07

1670	P17643	TRP1	DRB1*08:01	156	170	HPLFVIATRRSEEIL	30.11
1671	P17643	TRP1	DRB1*11:13	14	28	FPLLLFQQARAQFPR	30.19
1672	P17643	TRP1	DRB1*11:04	494	508	FGTASYLIRARRSMD	30.25
1673	P17643	TRP1	DRB1*11:46	494	508	FGTASYLIRARRSMD	30.25
1674	P17643	TRP1	DRB1*11:58	494	508	FGTASYLIRARRSMD	30.25
1675	P17643	TRP1	DRB1*13:11	494	508	FGTASYLIRARRSMD	30.25
1676	P17643	TRP1	DRB1*04:08	449	463	NTEMFVTAPDNLGYT	30.41
1677	P17643	TRP1	DRB1*15:15	186	200	NYFVWTHYYSVKKTF	30.43
1678	P17643	TRP1	DRB1*01:20	372	386	AVRSLHNLAHLFLNG	30.45
1679	P17643	TRP1	DRB1*11:03	154	168	TTHPLFVIATRRSEE	30.51
1680	P17643	TRP1	DRB1*08:04	153	167	RTTHPLFVIATRRSE	30.55
1681	P17643	TRP1	DRB1*13:21	189	203	VWTHYYSVKKTFLGV	30.57
1682	P17643	TRP1	DPB1*33:01	398	412	PIFVLLHTFTDAVFD	30.61
1683	P17643	TRP1	DPB1*71:01	398	412	PIFVLLHTFTDAVFD	30.61
1684	P17643	TRP1	DRB1*01:18	185	199	YNYFVWTHYYSVKKT	30.68
1685	P17643	TRP1	DRB1*01:24	14	28	FPLLLFQQARAQFPR	30.70
1686	P17643	TRP1	DPB1*02:01	188	202	FVWTHYYSVKKTFLG	30.77
1687	P17643	TRP1	DPB1*46:01	188	202	FVWTHYYSVKKTFLG	30.77
1688	P17643	TRP1	DPB1*81:01	188	202	FVWTHYYSVKKTFLG	30.77
1689	P17643	TRP1	DRB1*04:04	369	383	YDPAVRSLHNLAHLF	30.79
1690	P17643	TRP1	DRB1*01:01	397	411	DPIFVLLHTFTDAVF	30.92
1691	P17643	TRP1	DRB1*11:04	493	507	IFGTASYLIRARRSM	30.93
1692	P17643	TRP1	DRB1*11:46	493	507	IFGTASYLIRARRSM	30.93
1693	P17643	TRP1	DRB1*11:58	493	507	IFGTASYLIRARRSM	30.93
1694	P17643	TRP1	DRB1*13:11	493	507	IFGTASYLIRARRSM	30.93
1695	P17643	TRP1	DRB1*11:01	189	203	VWTHYYSVKKTFLGV	31.01
1696	P17643	TRP1	DRB1*11:10	189	203	VWTHYYSVKKTFLGV	31.01
1697	P17643	TRP1	DRB1*11:12	189	203	VWTHYYSVKKTFLGV	31.01
1698	P17643	TRP1	DRB1*11:28	189	203	VWTHYYSVKKTFLGV	31.01
1699	P17643	TRP1	DRB1*11:29	189	203	VWTHYYSVKKTFLGV	31.01
1700	P17643	TRP1	DRB1*11:49	189	203	VWTHYYSVKKTFLGV	31.01
1701	P17643	TRP1	DRB1*11:62	189	203	VWTHYYSVKKTFLGV	31.01
1702	P17643	TRP1	DRB1*11:74	189	203	VWTHYYSVKKTFLGV	31.01
1703	P17643	TRP1	DRB1*13:05	189	203	VWTHYYSVKKTFLGV	31.01
1704	P17643	TRP1	DRB1*13:14	189	203	VWTHYYSVKKTFLGV	31.01
1705	P17643	TRP1	DRB1*13:50	189	203	VWTHYYSVKKTFLGV	31.01
1706	P17643	TRP1	DRB1*04:01	452	466	MFVTAPDNLGYTYEI	31.08
1707	P17643	TRP1	DRB1*10:01	12	26	IFFPLLLFQQARAQF	31.15
1708	P17643	TRP1	DRB1*11:42	492	506	LIFGTASYLIRARRS	31.15
1709	P17643	TRP1	DRB1*01:18	370	384	DPAVRSLHNLAHLFL	31.18
1710	P17643	TRP1	DRB1*13:21	494	508	FGTASYLIRARRSMD	31.19
1711	P17643	TRP1	DRB1*01:18	184	198	IYNYFVWTHYYSVKK	31.29
1712	P17643	TRP1	DRB1*11:14	345	359	PPFYSNSTNSFRNTV	31.34

1713	P17643	TRP1	DRB1*13:02	345	359	PPFYSNSTNSFRNTV	31.34
1714	P17643	TRP1	DRB1*13:23	345	359	PPFYSNSTNSFRNTV	31.34
1715	P17643	TRP1	DRB1*13:97	345	359	PPFYSNSTNSFRNTV	31.34
1716	P17643	TRP1	DRB1*11:02	125	139	RVLIVRRNLLDLSKE	31.53
1717	P17643	TRP1	DRB1*11:65	125	139	RVLIVRRNLLDLSKE	31.53
1718	P17643	TRP1	DRB1*13:01	125	139	RVLIVRRNLLDLSKE	31.53
1719	P17643	TRP1	DRB1*01:20	369	383	YDPAVRSLHNLAHLF	31.57
1720	P17643	TRP1	DRB1*11:04	145	159	VRALDMAKRTTHPLF	31.74
1721	P17643	TRP1	DRB1*11:46	145	159	VRALDMAKRTTHPLF	31.74
1722	P17643	TRP1	DRB1*11:58	145	159	VRALDMAKRTTHPLF	31.74
1723	P17643	TRP1	DRB1*13:11	145	159	VRALDMAKRTTHPLF	31.74
1724	P17643	TRP1	DPB1*33:01	397	411	DPIFVLLHTFTDAVF	31.99
1725	P17643	TRP1	DPB1*71:01	397	411	DPIFVLLHTFTDAVF	31.99
1726	P17643	TRP1	DPB1*15:01	187	201	YFVWTHYYSVKKTFL	32.01
1727	P17643	TRP1	DPB1*04:02	187	201	YFVWTHYYSVKKTFL	32.03
1728	P17643	TRP1	DPB1*105:01	187	201	YFVWTHYYSVKKTFL	32.03
1729	P17643	TRP1	DPB1*49:01	187	201	YFVWTHYYSVKKTFL	32.03
1730	P17643	TRP1	DRB1*11:14	307	321	EDGPIRRNPAGNVAR	32.26
1731	P17643	TRP1	DRB1*13:02	307	321	EDGPIRRNPAGNVAR	32.26
1732	P17643	TRP1	DRB1*13:23	307	321	EDGPIRRNPAGNVAR	32.26
1733	P17643	TRP1	DRB1*13:97	307	321	EDGPIRRNPAGNVAR	32.26
1734	P17643	TRP1	DPB1*19:01	183	197	SIYNYFVWTHYYSVK	32.27
1735	P17643	TRP1	DRB1*04:08	397	411	DPIFVLLHTFTDAVF	32.47
1736	P17643	TRP1	DRB1*11:02	121	135	ACDQRVLIVRRNLLD	32.47
1737	P17643	TRP1	DRB1*11:65	121	135	ACDQRVLIVRRNLLD	32.47
1738	P17643	TRP1	DRB1*13:01	121	135	ACDQRVLIVRRNLLD	32.47
1739	P17643	TRP1	DRB1*14:01	125	139	RVLIVRRNLLDLSKE	32.49
1740	P17643	TRP1	DRB1*14:54	125	139	RVLIVRRNLLDLSKE	32.49
1741	P17643	TRP1	DPB1*02:02	188	202	FVWTHYYSVKKTFLG	32.57
1742	P17643	TRP1	DPB1*47:01	188	202	FVWTHYYSVKKTFLG	32.57
1743	P17643	TRP1	DRB1*08:01	157	171	PLFVIATRRSEEILG	32.59
1744	P17643	TRP1	DRB1*11:03	496	510	TASYLIRARRSMDEA	32.61
1745	P17643	TRP1	DRB1*11:04	13	27	FFPLLLFQQARAQFP	32.64
1746	P17643	TRP1	DRB1*11:46	13	27	FFPLLLFQQARAQFP	32.64
1747	P17643	TRP1	DRB1*11:58	13	27	FFPLLLFQQARAQFP	32.64
1748	P17643	TRP1	DRB1*13:11	13	27	FFPLLLFQQARAQFP	32.64
1749	P17643	TRP1	DPB1*40:01	182	196	ISIYNYFVWTHYYSV	32.71
1750	P17643	TRP1	DRB1*11:84	124	138	QRVLIVRRNLLDLSK	32.71
1751	P17643	TRP1	DPB1*41:01	217	231	GPAFLTWHRYHLLRL	32.72
1752	P17643	TRP1	DRB1*11:14	390	404	QTHLSPNDPIFVLLH	32.73
1753	P17643	TRP1	DRB1*13:02	390	404	QTHLSPNDPIFVLLH	32.73
1754	P17643	TRP1	DRB1*13:23	390	404	QTHLSPNDPIFVLLH	32.73
1755	P17643	TRP1	DRB1*13:97	390	404	QTHLSPNDPIFVLLH	32.73

1756	P17643	TRP1	DRB1*11:01	190	204	WTHYYSVKKTFLGVG	32.76
1757	P17643	TRP1	DRB1*11:10	190	204	WTHYYSVKKTFLGVG	32.76
1758	P17643	TRP1	DRB1*11:12	190	204	WTHYYSVKKTFLGVG	32.76
1759	P17643	TRP1	DRB1*11:28	190	204	WTHYYSVKKTFLGVG	32.76
1760	P17643	TRP1	DRB1*11:29	190	204	WTHYYSVKKTFLGVG	32.76
1761	P17643	TRP1	DRB1*11:49	190	204	WTHYYSVKKTFLGVG	32.76
1762	P17643	TRP1	DRB1*11:62	190	204	WTHYYSVKKTFLGVG	32.76
1763	P17643	TRP1	DRB1*11:74	190	204	WTHYYSVKKTFLGVG	32.76
1764	P17643	TRP1	DRB1*13:05	190	204	WTHYYSVKKTFLGVG	32.76
1765	P17643	TRP1	DRB1*13:14	190	204	WTHYYSVKKTFLGVG	32.76
1766	P17643	TRP1	DRB1*13:50	190	204	WTHYYSVKKTFLGVG	32.76
1767	P17643	TRP1	DPB1*01:01	187	201	YFVWTHYYSVKKTFL	32.80
1768	P17643	TRP1	DRB1*01:18	17	31	LLFQQARAQFPRQCA	32.83
1769	P17643	TRP1	DPB1*41:01	218	232	PAFLTWHRYHLLRLE	32.84
1770	P17643	TRP1	DRB1*11:03	14	28	FPLLLFQQARAQFPR	32.89
1771	P17643	TRP1	DRB1*10:01	394	408	SPNDPIFVLLHTFTD	32.93
1772	P17643	TRP1	DRB1*13:21	124	138	QRVLIVRRNLLDLSK	33.15
1773	P17643	TRP1	DRB1*01:20	140	154	EKNHFVRALDMAKRT	33.21
1774	P17643	TRP1	DRB1*04:01	448	462	TNTEMFVTAPDNLGY	33.24
1775	P17643	TRP1	DRB1*11:37	155	169	THPLFVIATRRSEEI	33.39
1776	P17643	TRP1	DRB1*13:07	155	169	THPLFVIATRRSEEI	33.39
1777	P17643	TRP1	DRB1*01:18	141	155	KNHFVRALDMAKRTT	33.40
1778	P17643	TRP1	DRB1*01:01	396	410	NDPIFVLLHTFTDAV	33.42
1779	P17643	TRP1	DRB1*01:18	140	154	EKNHFVRALDMAKRT	33.42
1780	P17643	TRP1	DRB1*01:01	210	224	EVDFSHEGPAFLTWH	33.44
1781	P17643	TRP1	DRB1*13:21	188	202	FVWTHYYSVKKTFLG	33.53
1782	P17643	TRP1	DRB1*15:15	184	198	IYNYFVWTHYYSVKK	33.53
1783	P17643	TRP1	DRB1*11:03	122	136	CDQRVLIVRRNLLDL	33.61
1784	P17643	TRP1	DRB1*01:01	155	169	THPLFVIATRRSEEI	33.65
1785	P17643	TRP1	DPB1*04:02	182	196	ISIYNYFVWTHYYSV	33.93
1786	P17643	TRP1	DPB1*105:01	182	196	ISIYNYFVWTHYYSV	33.93
1787	P17643	TRP1	DPB1*49:01	182	196	ISIYNYFVWTHYYSV	33.93
1788	P17643	TRP1	DRB1*14:06	124	138	QRVLIVRRNLLDLSK	33.93
1789	P17643	TRP1	DRB1*01:20	152	166	KRTTHPLFVIATRRS	33.95
1790	P17643	TRP1	DRB1*11:03	121	135	ACDQRVLIVRRNLLD	34.00
1791	P17643	TRP1	DRB1*01:02	490	504	VALIFGTASYLIRAR	34.03
1792	P17643	TRP1	DRB1*10:01	155	169	THPLFVIATRRSEEI	34.07
1793	P17643	TRP1	DPB1*16:01	182	196	ISIYNYFVWTHYYSV	34.13
1794	P17643	TRP1	DRB1*15:15	187	201	YFVWTHYYSVKKTFL	34.18
1795	P17643	TRP1	DRB1*11:37	157	171	PLFVIATRRSEEILG	34.19
1796	P17643	TRP1	DRB1*13:07	157	171	PLFVIATRRSEEILG	34.19
1797	P17643	TRP1	DRB1*13:21	123	137	DQRVLIVRRNLLDLS	34.19
1798	P17643	TRP1	DRB1*11:14	389	403	GQTHLSPNDPIFVLL	34.38

1799	P17643	TRP1	DRB1*13:02	389	403	GQTHLSPNDPIFVLL	34.38
1800	P17643	TRP1	DRB1*13:23	389	403	GQTHLSPNDPIFVLL	34.38
1801	P17643	TRP1	DRB1*13:97	389	403	GQTHLSPNDPIFVLL	34.38
1802	P17643	TRP1	DRB1*01:01	245	259	SLPYWNFATGKNVCD	34.48
1803	P17643	TRP1	DRB1*11:02	14	28	FPLLLFQQARAQFPR	34.58
1804	P17643	TRP1	DRB1*11:65	14	28	FPLLLFQQARAQFPR	34.58
1805	P17643	TRP1	DRB1*13:01	14	28	FPLLLFQQARAQFPR	34.58
1806	P17643	TRP1	DRB1*10:01	222	236	TWHRYHLLRLEKDMQ	34.60
1807	P17643	TRP1	DRB1*11:03	157	171	PLFVIATRRSEEILG	34.81
1808	P17643	TRP1	DRB1*11:14	309	323	GPIRRNPAGNVARPM	35.10
1809	P17643	TRP1	DRB1*13:02	309	323	GPIRRNPAGNVARPM	35.10
1810	P17643	TRP1	DRB1*13:23	309	323	GPIRRNPAGNVARPM	35.10
1811	P17643	TRP1	DRB1*13:97	309	323	GPIRRNPAGNVARPM	35.10
1812	P17643	TRP1	DRB1*08:01	155	169	THPLFVIATRRSEEI	35.14
1813	P17643	TRP1	DRB1*11:08	157	171	PLFVIATRRSEEILG	35.15
1814	P17643	TRP1	DRB1*11:03	217	231	GPAFLTWHRYHLLRL	35.17
1815	P17643	TRP1	DRB1*11:03	153	167	RTTHPLFVIATRRSE	35.22
1816	P17643	TRP1	DRB1*11:02	217	231	GPAFLTWHRYHLLRL	35.26
1817	P17643	TRP1	DRB1*11:65	217	231	GPAFLTWHRYHLLRL	35.26
1818	P17643	TRP1	DRB1*13:01	217	231	GPAFLTWHRYHLLRL	35.26
1819	P17643	TRP1	DPB1*41:01	219	233	AFLTWHRYHLLRLEK	35.44
1820	P17643	TRP1	DRB1*01:20	141	155	KNHFVRALDMAKRTT	35.49
1821	P17643	TRP1	DRB1*15:01	13	27	FFPLLLFQQARAQFP	35.55
1822	P17643	TRP1	DRB1*15:06	13	27	FFPLLLFQQARAQFP	35.55
1823	P17643	TRP1	DRB1*01:29	488	502	LLVALIFGTASYLIR	35.64
1824	P17643	TRP1	DRB1*11:08	154	168	TTHPLFVIATRRSEE	35.68
1825	P17643	TRP1	DRB1*15:01	489	503	LVALIFGTASYLIRA	35.69
1826	P17643	TRP1	DRB1*15:06	489	503	LVALIFGTASYLIRA	35.69
1827	P17643	TRP1	DRB1*10:01	17	31	LLFQQARAQFPRQCA	35.73
1828	P17643	TRP1	DRB1*04:08	448	462	TNTEMFVTAPDNLGY	35.80
1829	P17643	TRP1	DRB1*13:21	122	136	CDQRVLIVRRNLLDL	35.86
1830	P17643	TRP1	DRB1*13:21	190	204	WTHYYSVKKTFLGVG	35.86
1831	P17643	TRP1	DRB1*01:01	492	506	LIFGTASYLIRARRS	35.92
1832	P17643	TRP1	DRB1*11:13	15	29	PLLLFQQARAQFPRQ	35.94
1833	P17643	TRP1	DRB1*11:03	495	509	GTASYLIRARRSMDE	36.11
1834	P17643	TRP1	DRB1*11:42	370	384	DPAVRSLHNLAHLFL	36.14
1835	P17643	TRP1	DRB1*01:29	16	30	LLLFQQARAQFPRQC	36.24
1836	P17643	TRP1	DRB1*13:21	187	201	YFVWTHYYSVKKTFL	36.31
1837	P17643	TRP1	DRB1*08:04	157	171	PLFVIATRRSEEILG	36.44
1838	P17643	TRP1	DRB1*01:18	372	386	AVRSLHNLAHLFLNG	36.47
1839	P17643	TRP1	DRB1*04:08	396	410	NDPIFVLLHTFTDAV	36.48
1840	P17643	TRP1	DRB1*11:13	156	170	HPLFVIATRRSEEIL	36.50
1841	P17643	TRP1	DRB1*04:08	452	466	MFVTAPDNLGYTYEI	36.69

1842	P17643	TRP1	DRB1*01:18	189	203	VWTHYYSVKKTFLGV	36.71
1843	P17643	TRP1	DRB1*01:24	16	30	LLLFQQARAQFPRQC	36.75
1844	P17643	TRP1	DRB1*11:14	308	322	DGPIRRNPAGNVARP	36.78
1845	P17643	TRP1	DRB1*13:02	308	322	DGPIRRNPAGNVARP	36.78
1846	P17643	TRP1	DRB1*13:23	308	322	DGPIRRNPAGNVARP	36.78
1847	P17643	TRP1	DRB1*13:97	308	322	DGPIRRNPAGNVARP	36.78
1848	P17643	TRP1	DRB1*11:13	13	27	FFPLLLFQQARAQFP	36.89
1849	P17643	TRP1	DRB1*11:42	371	385	PAVRSLHNLAHLFLN	36.94
1850	P17643	TRP1	DRB1*10:01	371	385	PAVRSLHNLAHLFLN	37.00
1851	P17643	TRP1	DRB1*01:01	12	26	IFFPLLLFQQARAQF	37.15
1852	P17643	TRP1	DRB1*11:03	216	230	EGPAFLTWHRYHLLR	37.27
1853	P17643	TRP1	DRB1*11:04	158	172	LFVIATRRSEEILGP	37.31
1854	P17643	TRP1	DRB1*11:46	158	172	LFVIATRRSEEILGP	37.31
1855	P17643	TRP1	DRB1*11:58	158	172	LFVIATRRSEEILGP	37.31
1856	P17643	TRP1	DRB1*13:11	158	172	LFVIATRRSEEILGP	37.31
1857	P17643	TRP1	DPB1*33:01	490	504	VALIFGTASYLIRAR	37.43
1858	P17643	TRP1	DPB1*71:01	490	504	VALIFGTASYLIRAR	37.43
1859	P17643	TRP1	DRB1*01:18	245	259	SLPYWNFATGKNVCD	37.59
1860	P17643	TRP1	DRB1*15:03	14	28	FPLLLFQQARAQFPR	37.62
1861	P17643	TRP1	DRB1*11:01	191	205	THYYSVKKTFLGVGQ	37.63
1862	P17643	TRP1	DRB1*11:10	191	205	THYYSVKKTFLGVGQ	37.63
1863	P17643	TRP1	DRB1*11:12	191	205	THYYSVKKTFLGVGQ	37.63
1864	P17643	TRP1	DRB1*11:28	191	205	THYYSVKKTFLGVGQ	37.63
1865	P17643	TRP1	DRB1*11:29	191	205	THYYSVKKTFLGVGQ	37.63
1866	P17643	TRP1	DRB1*11:49	191	205	THYYSVKKTFLGVGQ	37.63
1867	P17643	TRP1	DRB1*11:62	191	205	THYYSVKKTFLGVGQ	37.63
1868	P17643	TRP1	DRB1*11:74	191	205	THYYSVKKTFLGVGQ	37.63
1869	P17643	TRP1	DRB1*13:05	191	205	THYYSVKKTFLGVGQ	37.63
1870	P17643	TRP1	DRB1*13:14	191	205	THYYSVKKTFLGVGQ	37.63
1871	P17643	TRP1	DRB1*13:50	191	205	THYYSVKKTFLGVGQ	37.63
1872	P17643	TRP1	DPB1*19:01	186	200	NYFVWTHYYSVKKTF	37.68
1873	P17643	TRP1	DPB1*33:01	396	410	NDPIFVLLHTFTDAV	37.71
1874	P17643	TRP1	DPB1*71:01	396	410	NDPIFVLLHTFTDAV	37.71
1875	P17643	TRP1	DRB1*09:01	243	257	SFSLPYWNFATGKNV	37.71
1876	P17643	TRP1	DRB1*15:07	490	504	VALIFGTASYLIRAR	37.71
1877	P17643	TRP1	DRB1*10:01	153	167	RTTHPLFVIATRRSE	37.76
1878	P17643	TRP1	DRB1*11:13	155	169	THPLFVIATRRSEEI	37.81
1879	P17643	TRP1	DRB1*15:03	13	27	FFPLLLFQQARAQFP	37.81
1880	P17643	TRP1	DRB1*11:84	155	169	THPLFVIATRRSEEI	37.83
1881	P17643	TRP1	DRB1*11:84	123	137	DQRVLIVRRNLLDLS	37.89
1882	P17643	TRP1	DRB1*01:18	395	409	PNDPIFVLLHTFTDA	37.91
1883	P17643	TRP1	DRB1*11:02	155	169	THPLFVIATRRSEEI	37.93
1884	P17643	TRP1	DRB1*11:65	155	169	THPLFVIATRRSEEI	37.93

1885	P17643	TRP1	DRB1*13:01	155	169	THPLFVIATRRSEEI	37.93
1886	P17643	TRP1	DRB1*10:01	221	235	LTWHRYHLLRLEKDM	37.94
1887	P17643	TRP1	DRB1*01:20	139	153	EEKNHFVRALDMAKR	38.13
1888	P17643	TRP1	DRB1*11:42	144	158	FVRALDMAKRTTHPL	38.20
1889	P17643	TRP1	DRB1*11:42	145	159	VRALDMAKRTTHPLF	38.20
1890	P17643	TRP1	DRB1*04:08	398	412	PIFVLLHTFTDAVFD	38.22
1891	P17643	TRP1	DRB1*10:01	156	170	HPLFVIATRRSEEIL	38.31
1892	P17643	TRP1	DRB1*10:01	399	413	IFVLLHTFTDAVFDE	38.32
1893	P17643	TRP1	DRB1*01:18	190	204	WTHYYSVKKTFLGVG	38.39
1894	P17643	TRP1	DPB1*33:01	491	505	ALIFGTASYLIRARR	38.41
1895	P17643	TRP1	DPB1*71:01	491	505	ALIFGTASYLIRARR	38.41
1896	P17643	TRP1	DRB1*01:11	491	505	ALIFGTASYLIRARR	38.45
1897	P17643	TRP1	DRB1*01:18	187	201	YFVWTHYYSVKKTFL	38.50
1898	P17643	TRP1	DRB1*11:02	218	232	PAFLTWHRYHLLRLE	38.50
1899	P17643	TRP1	DRB1*11:65	218	232	PAFLTWHRYHLLRLE	38.50
1900	P17643	TRP1	DRB1*13:01	218	232	PAFLTWHRYHLLRLE	38.50
1901	P17643	TRP1	DRB1*14:06	123	137	DQRVLIVRRNLLDLS	38.56
1902	P17643	TRP1	DRB1*01:18	11	25	CIFFPLLLFQQARAQ	38.57
1903	P17643	TRP1	DRB1*09:01	490	504	VALIFGTASYLIRAR	38.61
1904	P17643	TRP1	DRB1*01:18	157	171	PLFVIATRRSEEILG	38.62
1905	P17643	TRP1	DRB1*01:01	244	258	FSLPYWNFATGKNVC	38.68
1906	P17643	TRP1	DRB1*01:20	235	249	MQEMLQEPSFSLPYW	38.72
1907	P17643	TRP1	DRB1*01:01	398	412	PIFVLLHTFTDAVFD	38.84
1908	P17643	TRP1	DRB1*11:04	15	29	PLLLFQQARAQFPRQ	38.91
1909	P17643	TRP1	DRB1*11:46	15	29	PLLLFQQARAQFPRQ	38.91
1910	P17643	TRP1	DRB1*11:58	15	29	PLLLFQQARAQFPRQ	38.91
1911	P17643	TRP1	DRB1*13:11	15	29	PLLLFQQARAQFPRQ	38.91
1912	P17643	TRP1	DRB1*11:04	144	158	FVRALDMAKRTTHPL	38.95
1913	P17643	TRP1	DRB1*11:46	144	158	FVRALDMAKRTTHPL	38.95
1914	P17643	TRP1	DRB1*11:58	144	158	FVRALDMAKRTTHPL	38.95
1915	P17643	TRP1	DRB1*13:11	144	158	FVRALDMAKRTTHPL	38.95
1916	P17643	TRP1	DRB1*10:01	370	384	DPAVRSLHNLAHLFL	38.99
1917	P17643	TRP1	DRB1*01:20	493	507	IFGTASYLIRARRSM	39.00
1918	P17643	TRP1	DRB1*11:42	12	26	IFFPLLLFQQARAQF	39.05
1919	P17643	TRP1	DRB1*08:04	124	138	QRVLIVRRNLLDLSK	39.07
1920	P17643	TRP1	DRB1*01:11	14	28	FPLLLFQQARAQFPR	39.18
1921	P17643	TRP1	DRB1*01:01	211	225	VDFSHEGPAFLTWHR	39.26
1922	P17643	TRP1	DRB1*11:03	218	232	PAFLTWHRYHLLRLE	39.35
1923	P17643	TRP1	DPB1*41:01	182	196	ISIYNYFVWTHYYSV	39.46
1924	P17643	TRP1	DRB1*07:01	182	196	ISIYNYFVWTHYYSV	39.49
1925	P17643	TRP1	DPB1*40:01	187	201	YFVWTHYYSVKKTFL	39.52
1926	P17643	TRP1	DRB1*01:02	14	28	FPLLLFQQARAQFPR	39.52
1927	P17643	TRP1	DRB1*11:04	498	512	SYLIRARRSMDEANQ	39.58

1928	P17643	TRP1	DRB1*11:46	498	512	SYLIRARRSMDEANQ	39.58
1929	P17643	TRP1	DRB1*11:58	498	512	SYLIRARRSMDEANQ	39.58
1930	P17643	TRP1	DRB1*13:11	498	512	SYLIRARRSMDEANQ	39.58
1931	P17643	TRP1	DRB1*11:14	346	360	PFYSNSTNSFRNTVE	39.62
1932	P17643	TRP1	DRB1*13:02	346	360	PFYSNSTNSFRNTVE	39.62
1933	P17643	TRP1	DRB1*13:23	346	360	PFYSNSTNSFRNTVE	39.62
1934	P17643	TRP1	DRB1*13:97	346	360	PFYSNSTNSFRNTVE	39.62
1935	P17643	TRP1	DRB1*11:03	497	511	ASYLIRARRSMDEAN	39.64
1936	P17643	TRP1	DRB1*11:01	496	510	TASYLIRARRSMDEA	39.65
1937	P17643	TRP1	DRB1*11:10	496	510	TASYLIRARRSMDEA	39.65
1938	P17643	TRP1	DRB1*11:12	496	510	TASYLIRARRSMDEA	39.65
1939	P17643	TRP1	DRB1*11:28	496	510	TASYLIRARRSMDEA	39.65
1940	P17643	TRP1	DRB1*11:29	496	510	TASYLIRARRSMDEA	39.65
1941	P17643	TRP1	DRB1*11:49	496	510	TASYLIRARRSMDEA	39.65
1942	P17643	TRP1	DRB1*11:62	496	510	TASYLIRARRSMDEA	39.65
1943	P17643	TRP1	DRB1*11:74	496	510	TASYLIRARRSMDEA	39.65
1944	P17643	TRP1	DRB1*13:05	496	510	TASYLIRARRSMDEA	39.65
1945	P17643	TRP1	DRB1*13:14	496	510	TASYLIRARRSMDEA	39.65
1946	P17643	TRP1	DRB1*13:50	496	510	TASYLIRARRSMDEA	39.65
1947	P17643	TRP1	DRB1*01:18	139	153	EEKNHFVRALDMAKR	39.69
1948	P17643	TRP1	DRB1*11:02	493	507	IFGTASYLIRARRSM	39.73
1949	P17643	TRP1	DRB1*11:65	493	507	IFGTASYLIRARRSM	39.73
1950	P17643	TRP1	DRB1*13:01	493	507	IFGTASYLIRARRSM	39.73
1951	P17643	TRP1	DRB1*10:01	154	168	TTHPLFVIATRRSEE	39.82
1952	P17643	TRP1	DRB1*14:32	14	28	FPLLLFQQARAQFPR	39.85
1953	P17643	TRP1	DRB1*08:24	156	170	HPLFVIATRRSEEIL	39.93
1954	P17643	TRP1	DRB1*11:42	143	157	HFVRALDMAKRTTHP	40.09
1955	P17643	TRP1	DRB1*08:24	155	169	THPLFVIATRRSEEI	40.10
1956	P17643	TRP1	DRB1*11:84	156	170	HPLFVIATRRSEEIL	40.18
1957	P17643	TRP1	DRB1*01:18	244	258	FSLPYWNFATGKNVC	40.22
1958	P17643	TRP1	DRB1*01:01	184	198	IYNYFVWTHYYSVKK	40.34
1959	P17643	TRP1	DRB1*01:01	185	199	YNYFVWTHYYSVKKT	40.38
1960	P17643	TRP1	DRB1*01:11	15	29	PLLLFQQARAQFPRQ	40.46
1961	P17643	TRP1	DRB1*11:03	15	29	PLLLFQQARAQFPRQ	40.46
1962	P17643	TRP1	DRB1*01:18	186	200	NYFVWTHYYSVKKTF	40.54
1963	P17643	TRP1	DRB1*11:42	491	505	ALIFGTASYLIRARR	40.56
1964	P17643	TRP1	DRB1*01:11	488	502	LLVALIFGTASYLIR	40.58
1965	P17643	TRP1	DRB1*13:96	390	404	QTHLSPNDPIFVLLH	40.61
1966	P17643	TRP1	DPB1*55:01	184	198	IYNYFVWTHYYSVKK	40.78
1967	P17643	TRP1	DRB1*11:02	156	170	HPLFVIATRRSEEIL	40.79
1968	P17643	TRP1	DRB1*11:65	156	170	HPLFVIATRRSEEIL	40.79
1969	P17643	TRP1	DRB1*13:01	156	170	HPLFVIATRRSEEIL	40.79
1970	P17643	TRP1	DRB1*01:01	156	170	HPLFVIATRRSEEIL	40.83

1971	P17643	TRP1	DRB1*15:01	14	28	FPLLLFQQARAQFPR	41.01
1972	P17643	TRP1	DRB1*15:06	14	28	FPLLLFQQARAQFPR	41.01
1973	P17643	TRP1	DPB1*34:01	187	201	YFVWTHYYSVKKTFL	41.02
1974	P17643	TRP1	DRB1*11:42	142	156	NHFVRALDMAKRTTH	41.14
1975	P17643	TRP1	DRB1*13:61	124	138	QRVLIVRRNLLDLSK	41.23
1976	P17643	TRP1	DPB1*41:01	216	230	EGPAFLTWHRYHLLR	41.33
1977	P17643	TRP1	DRB1*15:15	219	233	AFLTWHRYHLLRLEK	41.33
1978	P17643	TRP1	DRB1*11:84	122	136	CDQRVLIVRRNLLDL	41.52
1979	P17643	TRP1	DRB1*13:96	344	358	TPPFYSNSTNSFRNT	41.58
1980	P17643	TRP1	DRB1*15:02	185	199	YNYFVWTHYYSVKKT	41.60
1981	P17643	TRP1	DRB1*11:42	498	512	SYLIRARRSMDEANQ	41.67
1982	P17643	TRP1	DRB1*11:42	16	30	LLLFQQARAQFPRQC	41.87
1983	P17643	TRP1	DRB1*11:03	125	139	RVLIVRRNLLDLSKE	41.92
1984	P17643	TRP1	DRB1*04:10	370	384	DPAVRSLHNLAHLFL	41.97
1985	P17643	TRP1	DRB1*11:03	492	506	LIFGTASYLIRARRS	41.99
1986	P17643	TRP1	DRB1*01:01	209	223	GEVDFSHEGPAFLTW	42.17
1987	P17643	TRP1	DRB1*09:01	15	29	PLLLFQQARAQFPRQ	42.18
1988	P17643	TRP1	DRB1*13:21	191	205	THYYSVKKTFLGVGQ	42.33
1989	P17643	TRP1	DRB1*08:01	153	167	RTTHPLFVIATRRSE	42.40
1990	P17643	TRP1	DRB1*10:01	223	237	WHRYHLLRLEKDMQE	42.51
1991	P17643	TRP1	DRB1*14:04	122	136	CDQRVLIVRRNLLDL	42.51
1992	P17643	TRP1	DRB1*11:02	15	29	PLLLFQQARAQFPRQ	42.53
1993	P17643	TRP1	DRB1*11:65	15	29	PLLLFQQARAQFPRQ	42.53
1994	P17643	TRP1	DRB1*13:01	15	29	PLLLFQQARAQFPRQ	42.53
1995	P17643	TRP1	DRB1*11:13	491	505	ALIFGTASYLIRARR	42.55
1996	P17643	TRP1	DRB1*11:04	152	166	KRTTHPLFVIATRRS	42.57
1997	P17643	TRP1	DRB1*11:46	152	166	KRTTHPLFVIATRRS	42.57
1998	P17643	TRP1	DRB1*11:58	152	166	KRTTHPLFVIATRRS	42.57
1999	P17643	TRP1	DRB1*13:11	152	166	KRTTHPLFVIATRRS	42.57
2000	P17643	TRP1	DRB1*14:04	124	138	QRVLIVRRNLLDLSK	42.59
2001	P17643	TRP1	DRB1*14:32	13	27	FFPLLLFQQARAQFP	42.65
2002	P17643	TRP1	DRB1*01:18	487	501	LLLVALIFGTASYLI	42.66
2003	P17643	TRP1	DRB1*01:01	154	168	TTHPLFVIATRRSEE	42.82
2004	P17643	TRP1	DRB1*01:02	491	505	ALIFGTASYLIRARR	42.88
2005	P17643	TRP1	DRB1*01:01	247	261	PYWNFATGKNVCDIC	42.90
2006	P17643	TRP1	DRB1*09:01	491	505	ALIFGTASYLIRARR	43.03
2007	P17643	TRP1	DRB1*11:42	152	166	KRTTHPLFVIATRRS	43.05
2008	P17643	TRP1	DRB1*13:21	121	135	ACDQRVLIVRRNLLD	43.08
2009	P17643	TRP1	DRB1*11:14	310	324	PIRRNPAGNVARPMV	43.15
2010	P17643	TRP1	DRB1*13:02	310	324	PIRRNPAGNVARPMV	43.15
2011	P17643	TRP1	DRB1*13:23	310	324	PIRRNPAGNVARPMV	43.15
2012	P17643	TRP1	DRB1*13:97	310	324	PIRRNPAGNVARPMV	43.15
2013	P17643	TRP1	DRB1*16:09	185	199	YNYFVWTHYYSVKKT	43.18

2014	P17643	TRP1	DPB1*33:01	7	21	LSLGCIFFPLLLFQQ	43.26
2015	P17643	TRP1	DPB1*71:01	7	21	LSLGCIFFPLLLFQQ	43.26
2016	P17643	TRP1	DRB1*11:04	492	506	LIFGTASYLIRARRS	43.28
2017	P17643	TRP1	DRB1*11:46	492	506	LIFGTASYLIRARRS	43.28
2018	P17643	TRP1	DRB1*11:58	492	506	LIFGTASYLIRARRS	43.28
2019	P17643	TRP1	DRB1*13:11	492	506	LIFGTASYLIRARRS	43.28
2020	P17643	TRP1	DRB1*13:96	389	403	GQTHLSPNDPIFVLL	43.28
2021	P17643	TRP1	DRB1*01:29	13	27	FFPLLLFQQARAQFP	43.31
2022	P17643	TRP1	DRB1*04:72	450	464	TEMFVTAPDNLGYTY	43.32
2023	P17643	TRP1	DPB1*55:01	185	199	YNYFVWTHYYSVKKT	43.38
2024	P17643	TRP1	DRB1*11:02	120	134	AACDQRVLIVRRNLL	43.42
2025	P17643	TRP1	DRB1*11:65	120	134	AACDQRVLIVRRNLL	43.42
2026	P17643	TRP1	DRB1*13:01	120	134	AACDQRVLIVRRNLL	43.42
2027	P17643	TRP1	DRB1*09:01	246	260	LPYWNFATGKNVCDI	43.46
2028	P17643	TRP1	DRB1*11:14	391	405	THLSPNDPIFVLLHT	43.62
2029	P17643	TRP1	DRB1*13:02	391	405	THLSPNDPIFVLLHT	43.62
2030	P17643	TRP1	DRB1*13:23	391	405	THLSPNDPIFVLLHT	43.62
2031	P17643	TRP1	DRB1*13:97	391	405	THLSPNDPIFVLLHT	43.62
2032	P17643	TRP1	DRB1*08:01	154	168	TTHPLFVIATRRSEE	43.73
2033	P17643	TRP1	DRB1*14:06	122	136	CDQRVLIVRRNLLDL	43.83
2034	P17643	TRP1	DRB1*04:10	397	411	DPIFVLLHTFTDAVF	43.85
2035	P17643	TRP1	DRB1*15:02	184	198	IYNYFVWTHYYSVKK	43.85
2036	P17643	TRP1	DRB1*15:02	490	504	VALIFGTASYLIRAR	43.90
2037	P17643	TRP1	DRB1*01:18	152	166	KRTTHPLFVIATRRS	43.94
2038	P17643	TRP1	DRB1*15:15	218	232	PAFLTWHRYHLLRLE	43.95
2039	P17643	TRP1	DRB1*01:18	247	261	PYWNFATGKNVCDIC	44.04
2040	P17643	TRP1	DRB1*14:04	120	134	AACDQRVLIVRRNLL	44.06
2041	P17643	TRP1	DPB1*16:01	217	231	GPAFLTWHRYHLLRL	44.13
2042	P17643	TRP1	DRB1*11:42	141	155	KNHFVRALDMAKRTT	44.18
2043	P17643	TRP1	DRB1*09:01	14	28	FPLLLFQQARAQFPR	44.22
2044	P17643	TRP1	DRB1*01:18	191	205	THYYSVKKTFLGVGQ	44.24
2045	P17643	TRP1	DRB1*01:01	371	385	PAVRSLHNLAHLFLN	44.30
2046	P17643	TRP1	DRB1*01:01	380	394	AHLFLNGTGGQTHLS	44.30
2047	P17643	TRP1	DRB1*13:66	156	170	HPLFVIATRRSEEIL	44.42
2048	P17643	TRP1	DRB1*14:04	123	137	DQRVLIVRRNLLDLS	44.43
2049	P17643	TRP1	DRB1*11:02	216	230	EGPAFLTWHRYHLLR	44.44
2050	P17643	TRP1	DRB1*11:65	216	230	EGPAFLTWHRYHLLR	44.44
2051	P17643	TRP1	DRB1*13:01	216	230	EGPAFLTWHRYHLLR	44.44
2052	P17643	TRP1	DRB1*04:10	371	385	PAVRSLHNLAHLFLN	44.46
2053	P17643	TRP1	DRB1*14:32	156	170	HPLFVIATRRSEEIL	44.57
2054	P17643	TRP1	DRB1*04:05	399	413	IFVLLHTFTDAVFDE	44.63
2055	P17643	TRP1	DRB1*01:18	183	197	SIYNYFVWTHYYSVK	44.66
2056	P17643	TRP1	DRB1*10:01	268	282	SRSNFDSTLISPNSV	44.70

2057	P17643	TRP1	DRB1*08:04	123	137	DQRVLIVRRNLLDLS	44.74
2058	P17643	TRP1	DPB1*16:01	218	232	PAFLTWHRYHLLRLE	44.78
2059	P17643	TRP1	DRB1*01:01	370	384	DPAVRSLHNLAHLFL	44.80
2060	P17643	TRP1	DRB1*01:01	153	167	RTTHPLFVIATRRSE	44.82
2061	P17643	TRP1	DRB1*11:02	219	233	AFLTWHRYHLLRLEK	44.85
2062	P17643	TRP1	DRB1*11:65	219	233	AFLTWHRYHLLRLEK	44.85
2063	P17643	TRP1	DRB1*13:01	219	233	AFLTWHRYHLLRLEK	44.85
2064	P17643	TRP1	DRB1*11:37	154	168	TTHPLFVIATRRSEE	45.06
2065	P17643	TRP1	DRB1*13:07	154	168	TTHPLFVIATRRSEE	45.06
2066	P17643	TRP1	DRB1*01:18	210	224	EVDFSHEGPAFLTWH	45.08
2067	P17643	TRP1	DRB1*09:01	244	258	FSLPYWNFATGKNVC	45.10
2068	P17643	TRP1	DRB1*14:38	124	138	QRVLIVRRNLLDLSK	45.13
2069	P17643	TRP1	DRB1*04:04	152	166	KRTTHPLFVIATRRS	45.16
2070	P17643	TRP1	DRB1*11:08	153	167	RTTHPLFVIATRRSE	45.17
2071	P17643	TRP1	DRB1*01:20	234	248	DMQEMLQEPSFSLPY	45.21
2072	P17643	TRP1	DRB1*15:15	217	231	GPAFLTWHRYHLLRL	45.21
2073	P17643	TRP1	DRB1*15:01	12	26	IFFPLLLFQQARAQF	45.23
2074	P17643	TRP1	DRB1*15:06	12	26	IFFPLLLFQQARAQF	45.23
2075	P17643	TRP1	DRB1*11:14	306	320	TEDGPIRRNPAGNVA	45.36
2076	P17643	TRP1	DRB1*13:02	306	320	TEDGPIRRNPAGNVA	45.36
2077	P17643	TRP1	DRB1*13:23	306	320	TEDGPIRRNPAGNVA	45.36
2078	P17643	TRP1	DRB1*13:97	306	320	TEDGPIRRNPAGNVA	45.36
2079	P17643	TRP1	DRB1*08:01	158	172	LFVIATRRSEEILGP	45.40
2080	P17643	TRP1	DRB1*08:02	155	169	THPLFVIATRRSEEI	45.51
2081	P17643	TRP1	DRB1*11:42	158	172	LFVIATRRSEEILGP	45.56
2082	P17643	TRP1	DRB1*01:18	188	202	FVWTHYYSVKKTFLG	45.58
2083	P17643	TRP1	DRB1*15:15	188	202	FVWTHYYSVKKTFLG	45.64
2084	P17643	TRP1	DRB1*14:32	15	29	PLLLFQQARAQFPRQ	45.65
2085	P17643	TRP1	DRB1*15:37	490	504	VALIFGTASYLIRAR	45.68
2086	P17643	TRP1	DRB1*11:11	156	170	HPLFVIATRRSEEIL	45.73
2087	P17643	TRP1	DRB1*13:21	499	513	YLIRARRSMDEANQP	45.73
2088	P17643	TRP1	DRB1*01:01	395	409	PNDPIFVLLHTFTDA	45.75
2089	P17643	TRP1	DPB1*34:01	182	196	ISIYNYFVWTHYYSV	45.77
2090	P17643	TRP1	DRB1*04:04	14	28	FPLLLFQQARAQFPR	45.80
2091	P17643	TRP1	DRB1*01:01	140	154	EKNHFVRALDMAKRT	45.85
2092	P17643	TRP1	DRB1*13:96	343	357	DTPPFYSNSTNSFRN	45.85
2093	P17643	TRP1	DPB1*33:01	5	19	KLLSLGCIFFPLLLF	45.93
2094	P17643	TRP1	DPB1*71:01	5	19	KLLSLGCIFFPLLLF	45.93
2095	P17643	TRP1	DRB1*14:06	125	139	RVLIVRRNLLDLSKE	45.95
2096	P17643	TRP1	DRB1*11:03	89	103	VWPLRFFNRTCHCNG	45.96
2097	P17643	TRP1	DRB1*11:14	490	504	VALIFGTASYLIRAR	45.96
2098	P17643	TRP1	DRB1*13:02	490	504	VALIFGTASYLIRAR	45.96
2099	P17643	TRP1	DRB1*13:23	490	504	VALIFGTASYLIRAR	45.96

2100	P17643	TRP1	DRB1*13:97	490	504	VALIFGTASYLIRAR	45.96
2101	P17643	TRP1	DRB1*11:03	120	134	AACDQRVLIVRRNLL	46.05
2102	P17643	TRP1	DRB1*04:04	157	171	PLFVIATRRSEEILG	46.17
2103	P17643	TRP1	DRB1*11:01	497	511	ASYLIRARRSMDEAN	46.19
2104	P17643	TRP1	DRB1*11:10	497	511	ASYLIRARRSMDEAN	46.19
2105	P17643	TRP1	DRB1*11:12	497	511	ASYLIRARRSMDEAN	46.19
2106	P17643	TRP1	DRB1*11:28	497	511	ASYLIRARRSMDEAN	46.19
2107	P17643	TRP1	DRB1*11:29	497	511	ASYLIRARRSMDEAN	46.19
2108	P17643	TRP1	DRB1*11:49	497	511	ASYLIRARRSMDEAN	46.19
2109	P17643	TRP1	DRB1*11:62	497	511	ASYLIRARRSMDEAN	46.19
2110	P17643	TRP1	DRB1*11:74	497	511	ASYLIRARRSMDEAN	46.19
2111	P17643	TRP1	DRB1*13:05	497	511	ASYLIRARRSMDEAN	46.19
2112	P17643	TRP1	DRB1*13:14	497	511	ASYLIRARRSMDEAN	46.19
2113	P17643	TRP1	DRB1*13:50	497	511	ASYLIRARRSMDEAN	46.19
2114	P17643	TRP1	DRB1*14:32	490	504	VALIFGTASYLIRAR	46.20
2115	P17643	TRP1	DRB1*01:18	369	383	YDPAVRSLHNLAHLF	46.22
2116	P17643	TRP1	DRB1*11:02	13	27	FFPLLLFQQARAQFP	46.29
2117	P17643	TRP1	DRB1*11:65	13	27	FFPLLLFQQARAQFP	46.29
2118	P17643	TRP1	DRB1*13:01	13	27	FFPLLLFQQARAQFP	46.29
2119	P17643	TRP1	DRB1*08:02	156	170	HPLFVIATRRSEEIL	46.30
2120	P17643	TRP1	DRB1*01:01	189	203	VWTHYYSVKKTFLGV	46.38
2121	P17643	TRP1	DRB1*04:10	396	410	NDPIFVLLHTFTDAV	46.41
2122	P17643	TRP1	DRB1*13:66	157	171	PLFVIATRRSEEILG	46.44
2123	P17643	TRP1	DPB1*16:01	219	233	AFLTWHRYHLLRLEK	46.45
2124	P17643	TRP1	DRB1*11:13	157	171	PLFVIATRRSEEILG	46.62
2125	P17643	TRP1	DPB1*33:01	489	503	LVALIFGTASYLIRA	46.80
2126	P17643	TRP1	DPB1*71:01	489	503	LVALIFGTASYLIRA	46.80
2127	P17643	TRP1	DRB1*10:01	11	25	CIFFPLLLFQQARAQ	46.92
2128	P17643	TRP1	DRB1*11:42	369	383	YDPAVRSLHNLAHLF	46.98
2129	P17643	TRP1	DRB1*14:12	124	138	QRVLIVRRNLLDLSK	47.15
2130	P17643	TRP1	DRB1*15:03	219	233	AFLTWHRYHLLRLEK	47.24
2131	P17643	TRP1	DRB1*01:20	373	387	VRSLHNLAHLFLNGT	47.37
2132	P17643	TRP1	DRB1*10:01	184	198	IYNYFVWTHYYSVKK	47.37
2133	P17643	TRP1	DRB1*11:03	88	102	EVWPLRFFNRTCHCN	47.38
2134	P17643	TRP1	DRB1*04:08	395	409	PNDPIFVLLHTFTDA	47.45
2135	P17643	TRP1	DRB1*15:07	491	505	ALIFGTASYLIRARR	47.47
2136	P17643	TRP1	DPB1*33:01	8	22	SLGCIFFPLLLFQQA	47.48
2137	P17643	TRP1	DPB1*71:01	8	22	SLGCIFFPLLLFQQA	47.48
2138	P17643	TRP1	DRB1*10:01	140	154	EKNHFVRALDMAKRT	47.51
2139	P17643	TRP1	DPB1*33:01	399	413	IFVLLHTFTDAVFDE	47.53
2140	P17643	TRP1	DPB1*71:01	399	413	IFVLLHTFTDAVFDE	47.53
2141	P17643	TRP1	DRB1*11:42	140	154	EKNHFVRALDMAKRT	47.57
2142	P17643	TRP1	DRB1*13:21	493	507	IFGTASYLIRARRSM	47.58

2143	P17643	TRP1	DRB1*11:01	188	202	FVWTHYYSVKKTFLG	47.59
2144	P17643	TRP1	DRB1*11:10	188	202	FVWTHYYSVKKTFLG	47.59
2145	P17643	TRP1	DRB1*11:12	188	202	FVWTHYYSVKKTFLG	47.59
2146	P17643	TRP1	DRB1*11:28	188	202	FVWTHYYSVKKTFLG	47.59
2147	P17643	TRP1	DRB1*11:29	188	202	FVWTHYYSVKKTFLG	47.59
2148	P17643	TRP1	DRB1*11:49	188	202	FVWTHYYSVKKTFLG	47.59
2149	P17643	TRP1	DRB1*11:62	188	202	FVWTHYYSVKKTFLG	47.59
2150	P17643	TRP1	DRB1*11:74	188	202	FVWTHYYSVKKTFLG	47.59
2151	P17643	TRP1	DRB1*13:05	188	202	FVWTHYYSVKKTFLG	47.59
2152	P17643	TRP1	DRB1*13:14	188	202	FVWTHYYSVKKTFLG	47.59
2153	P17643	TRP1	DRB1*13:50	188	202	FVWTHYYSVKKTFLG	47.59
2154	P17643	TRP1	DRB1*11:03	145	159	VRALDMAKRTTHPLF	47.66
2155	P17643	TRP1	DRB1*16:02	490	504	VALIFGTASYLIRAR	47.68
2156	P17643	TRP1	DRB1*04:04	13	27	FFPLLLFQQARAQFP	47.72
2157	P17643	TRP1	DRB1*11:04	12	26	IFFPLLLFQQARAQF	47.75
2158	P17643	TRP1	DRB1*11:46	12	26	IFFPLLLFQQARAQF	47.75
2159	P17643	TRP1	DRB1*11:58	12	26	IFFPLLLFQQARAQF	47.75
2160	P17643	TRP1	DRB1*13:11	12	26	IFFPLLLFQQARAQF	47.75
2161	P17643	TRP1	DRB1*10:01	141	155	KNHFVRALDMAKRTT	47.87
2162	P17643	TRP1	DRB1*11:03	144	158	FVRALDMAKRTTHPL	48.10
2163	P17643	TRP1	DRB1*15:03	218	232	PAFLTWHRYHLLRLE	48.20
2164	P17643	TRP1	DRB1*13:21	217	231	GPAFLTWHRYHLLRL	48.21
2165	P17643	TRP1	DRB1*11:03	219	233	AFLTWHRYHLLRLEK	48.25
2166	P17643	TRP1	DRB1*15:03	217	231	GPAFLTWHRYHLLRL	48.26
2167	P17643	TRP1	DRB1*16:09	184	198	IYNYFVWTHYYSVKK	48.28
2168	P17643	TRP1	DRB1*01:01	141	155	KNHFVRALDMAKRTT	48.31
2169	P17643	TRP1	DRB1*08:01	496	510	TASYLIRARRSMDEA	48.32
2170	P17643	TRP1	DRB1*10:01	490	504	VALIFGTASYLIRAR	48.32
2171	P17643	TRP1	DRB1*10:01	460	474	LGYTYEIQWPSREFS	48.44
2172	P17643	TRP1	DRB1*01:18	373	387	VRSLHNLAHLFLNGT	48.47
2173	P17643	TRP1	DRB1*13:61	123	137	DQRVLIVRRNLLDLS	48.50
2174	P17643	TRP1	DRB1*01:01	187	201	YFVWTHYYSVKKTFL	48.52
2175	P17643	TRP1	DRB1*01:01	379	393	LAHLFLNGTGGQTHL	48.75
2176	P17643	TRP1	DRB1*08:24	154	168	TTHPLFVIATRRSEE	48.89
2177	P17643	TRP1	DRB1*14:06	14	28	FPLLLFQQARAQFPR	48.96
2178	P17643	TRP1	DRB1*11:11	155	169	THPLFVIATRRSEEI	49.02
2179	P17643	TRP1	DRB1*15:01	217	231	GPAFLTWHRYHLLRL	49.09
2180	P17643	TRP1	DRB1*15:06	217	231	GPAFLTWHRYHLLRL	49.09
2181	P17643	TRP1	DRB1*14:04	121	135	ACDQRVLIVRRNLLD	49.12
2182	P17643	TRP1	DRB1*15:02	186	200	NYFVWTHYYSVKKTF	49.12
2183	P17643	TRP1	DRB1*08:04	122	136	CDQRVLIVRRNLLDL	49.14
2184	P17643	TRP1	DRB1*08:04	14	28	FPLLLFQQARAQFPR	49.27
2185	P17643	TRP1	DRB1*11:02	492	506	LIFGTASYLIRARRS	49.31

2186	P17643	TRP1	DRB1*11:65	492	506	LIFGTASYLIRARRS	49.31
2187	P17643	TRP1	DRB1*13:01	492	506	LIFGTASYLIRARRS	49.31
2188	P17643	TRP1	DRB1*13:21	120	134	AACDQRVLIVRRNLL	49.48
2189	P17643	TRP1	DRB1*11:13	154	168	TTHPLFVIATRRSEE	49.64
2190	P17643	TRP1	DRB1*10:01	185	199	YNYFVWTHYYSVKKT	49.68
2191	P17643	TRP1	DRB1*13:21	222	236	TWHRYHLLRLEKDMQ	49.73
2192	P17643	TRP1	DPB1*41:01	220	234	FLTWHRYHLLRLEKD	49.77
2193	P17643	TRP1	DRB1*04:10	398	412	PIFVLLHTFTDAVFD	49.80
2194	P17643	TRP1	DRB1*10:01	152	166	KRTTHPLFVIATRRS	49.85
2195	P17643	TRP1	DRB1*11:84	154	168	TTHPLFVIATRRSEE	49.87
2196	P17643	TRP1	DRB1*16:09	186	200	NYFVWTHYYSVKKTF	49.93
2197	P40967	PMEL17	DRB1*01:20	286	300	VTAQVVLQAAIPLTS	5.84
2198	P40967	PMEL17	DRB1*01:20	14	28	VIGALLAVGATKVPR	6.21
2199	P40967	PMEL17	DRB1*01:01	286	300	VTAQVVLQAAIPLTS	6.37
2200	P40967	PMEL17	DRB1*01:18	286	300	VTAQVVLQAAIPLTS	6.37
2201	P40967	PMEL17	DRB1*01:20	287	301	TAQVVLQAAIPLTSC	6.38
2202	P40967	PMEL17	DRB1*01:01	14	28	VIGALLAVGATKVPR	6.60
2203	P40967	PMEL17	DRB1*01:20	13	27	AVIGALLAVGATKVP	6.67
2204	P40967	PMEL17	DRB1*01:18	14	28	VIGALLAVGATKVPR	6.76
2205	P40967	PMEL17	DRB1*01:01	287	301	TAQVVLQAAIPLTSC	6.93
2206	P40967	PMEL17	DRB1*01:20	285	299	PVTAQVVLQAAIPLT	7.04
2207	P40967	PMEL17	DRB1*01:18	287	301	TAQVVLQAAIPLTSC	7.07
2208	P40967	PMEL17	DRB1*01:01	15	29	IGALLAVGATKVPRN	7.11
2209	P40967	PMEL17	DRB1*01:18	15	29	IGALLAVGATKVPRN	7.12
2210	P40967	PMEL17	DRB1*01:20	288	302	AQVVLQAAIPLTSCG	7.41
2211	P40967	PMEL17	DRB1*01:01	156	170	WGQYWQVLGGPVSGL	7.45
2212	P40967	PMEL17	DRB1*01:20	15	29	IGALLAVGATKVPRN	7.50
2213	P40967	PMEL17	DRB1*01:01	157	171	GQYWQVLGGPVSGLS	7.51
2214	P40967	PMEL17	DRB1*01:01	285	299	PVTAQVVLQAAIPLT	7.59
2215	P40967	PMEL17	DRB1*01:01	13	27	AVIGALLAVGATKVP	7.61
2216	P40967	PMEL17	DRB1*01:18	13	27	AVIGALLAVGATKVP	7.63
2217	P40967	PMEL17	DRB1*01:18	156	170	WGQYWQVLGGPVSGL	7.87
2218	P40967	PMEL17	DRB1*01:18	285	299	PVTAQVVLQAAIPLT	7.91
2219	P40967	PMEL17	DRB1*01:18	157	171	GQYWQVLGGPVSGLS	7.92
2220	P40967	PMEL17	DRB1*01:01	288	302	AQVVLQAAIPLTSCG	8.19
2221	P40967	PMEL17	DRB1*01:18	288	302	AQVVLQAAIPLTSCG	8.22
2222	P40967	PMEL17	DRB1*11:14	228	242	GNKHFLRNQPLTFAL	8.61
2223	P40967	PMEL17	DRB1*13:02	228	242	GNKHFLRNQPLTFAL	8.61
2224	P40967	PMEL17	DRB1*13:23	228	242	GNKHFLRNQPLTFAL	8.61
2225	P40967	PMEL17	DRB1*13:97	228	242	GNKHFLRNQPLTFAL	8.61
2226	P40967	PMEL17	DRB1*01:20	12	26	LAVIGALLAVGATKV	8.62
2227	P40967	PMEL17	DRB1*11:14	229	243	NKHFLRNQPLTFALQ	8.69
2228	P40967	PMEL17	DRB1*13:02	229	243	NKHFLRNQPLTFALQ	8.69

2229	P40967	PMEL17	DRB1*13:23	229	243	NKHFLRNQPLTFALQ	8.69
2230	P40967	PMEL17	DRB1*13:97	229	243	NKHFLRNQPLTFALQ	8.69
2231	P40967	PMEL17	DRB1*01:01	16	30	GALLAVGATKVPRNQ	8.84
2232	P40967	PMEL17	DRB1*01:18	404	418	EVSIVVLSGTTAAQV	8.87
2233	P40967	PMEL17	DRB1*01:01	158	172	QYWQVLGGPVSGLSI	8.91
2234	P40967	PMEL17	DRB1*01:18	12	26	LAVIGALLAVGATKV	9.12
2235	P40967	PMEL17	DRB1*01:01	81	95	NASFSIALNFPGSQK	9.16
2236	P40967	PMEL17	DRB1*11:04	459	473	DGTATLRLVKRQVPL	9.31
2237	P40967	PMEL17	DRB1*11:46	459	473	DGTATLRLVKRQVPL	9.31
2238	P40967	PMEL17	DRB1*11:58	459	473	DGTATLRLVKRQVPL	9.31
2239	P40967	PMEL17	DRB1*13:11	459	473	DGTATLRLVKRQVPL	9.31
2240	P40967	PMEL17	DRB1*01:18	16	30	GALLAVGATKVPRNQ	9.32
2241	P40967	PMEL17	DRB1*01:18	403	417	AEVSIVVLSGTTAAQ	9.34
2242	P40967	PMEL17	DRB1*01:18	81	95	NASFSIALNFPGSQK	9.44
2243	P40967	PMEL17	DRB1*01:01	12	26	LAVIGALLAVGATKV	9.62
2244	P40967	PMEL17	DRB1*01:01	403	417	AEVSIVVLSGTTAAQ	9.68
2245	P40967	PMEL17	DRB1*01:18	158	172	QYWQVLGGPVSGLSI	9.71
2246	P40967	PMEL17	DRB1*01:01	404	418	EVSIVVLSGTTAAQV	9.77
2247	P40967	PMEL17	DRB1*11:14	230	244	KHFLRNQPLTFALQL	9.79
2248	P40967	PMEL17	DRB1*13:02	230	244	KHFLRNQPLTFALQL	9.79
2249	P40967	PMEL17	DRB1*13:23	230	244	KHFLRNQPLTFALQL	9.79
2250	P40967	PMEL17	DRB1*13:97	230	244	KHFLRNQPLTFALQL	9.79
2251	P40967	PMEL17	DRB1*01:20	289	303	QVVLQAAIPLTSCGS	9.87
2252	P40967	PMEL17	DRB1*01:20	16	30	GALLAVGATKVPRNQ	9.89
2253	P40967	PMEL17	DRB1*01:20	11	25	HLAVIGALLAVGATK	9.92
2254	P40967	PMEL17	DRB1*01:18	405	419	VSIVVLSGTTAAQVT	10.11
2255	P40967	PMEL17	DRB1*11:04	460	474	GTATLRLVKRQVPLD	10.14
2256	P40967	PMEL17	DRB1*11:46	460	474	GTATLRLVKRQVPLD	10.14
2257	P40967	PMEL17	DRB1*11:58	460	474	GTATLRLVKRQVPLD	10.14
2258	P40967	PMEL17	DRB1*13:11	460	474	GTATLRLVKRQVPLD	10.14
2259	P40967	PMEL17	DRB1*01:18	82	96	ASFSIALNFPGSQKV	10.24
2260	P40967	PMEL17	DRB1*01:01	82	96	ASFSIALNFPGSQKV	10.27
2261	P40967	PMEL17	DRB1*01:01	155	169	TWGQYWQVLGGPVSG	10.30
2262	P40967	PMEL17	DRB1*01:20	404	418	EVSIVVLSGTTAAQV	10.55
2263	P40967	PMEL17	DRB1*01:20	403	417	AEVSIVVLSGTTAAQ	10.60
2264	P40967	PMEL17	DRB1*01:18	11	25	HLAVIGALLAVGATK	10.72
2265	P40967	PMEL17	DRB1*11:42	459	473	DGTATLRLVKRQVPL	10.82
2266	P40967	PMEL17	DRB1*11:42	460	474	GTATLRLVKRQVPLD	10.87
2267	P40967	PMEL17	DRB1*01:18	155	169	TWGQYWQVLGGPVSG	11.07
2268	P40967	PMEL17	DRB1*01:01	80	94	ANASFSIALNFPGSQ	11.10
2269	P40967	PMEL17	DRB1*11:14	227	241	GGNKHFLRNQPLTFA	11.13
2270	P40967	PMEL17	DRB1*13:02	227	241	GGNKHFLRNQPLTFA	11.13
2271	P40967	PMEL17	DRB1*13:23	227	241	GGNKHFLRNQPLTFA	11.13

2272	P40967	PMEL17	DRB1*13:97	227	241	GGNKHFLRNQPLTFA	11.13
2273	P40967	PMEL17	DRB1*01:18	406	420	SIVVLSGTTAAQVTT	11.14
2274	P40967	PMEL17	DRB1*01:01	405	419	VSIVVLSGTTAAQVT	11.23
2275	P40967	PMEL17	DRB1*11:04	461	475	TATLRLVKRQVPLDC	11.26
2276	P40967	PMEL17	DRB1*11:46	461	475	TATLRLVKRQVPLDC	11.26
2277	P40967	PMEL17	DRB1*11:58	461	475	TATLRLVKRQVPLDC	11.26
2278	P40967	PMEL17	DRB1*13:11	461	475	TATLRLVKRQVPLDC	11.26
2279	P40967	PMEL17	DRB1*13:96	228	242	GNKHFLRNQPLTFAL	11.31
2280	P40967	PMEL17	DRB1*01:01	11	25	HLAVIGALLAVGATK	11.34
2281	P40967	PMEL17	DRB1*01:18	289	303	QVVLQAAIPLTSCGS	11.49
2282	P40967	PMEL17	DRB1*10:01	81	95	NASFSIALNFPGSQK	11.50
2283	P40967	PMEL17	DRB1*13:96	229	243	NKHFLRNQPLTFALQ	11.50
2284	P40967	PMEL17	DRB1*01:18	80	94	ANASFSIALNFPGSQ	11.53
2285	P40967	PMEL17	DRB1*01:20	406	420	SIVVLSGTTAAQVTT	11.66
2286	P40967	PMEL17	DRB1*01:20	405	419	VSIVVLSGTTAAQVT	11.75
2287	P40967	PMEL17	DRB1*01:01	406	420	SIVVLSGTTAAQVTT	11.80
2288	P40967	PMEL17	DRB1*01:01	289	303	QVVLQAAIPLTSCGS	11.98
2289	P40967	PMEL17	DRB1*01:29	14	28	VIGALLAVGATKVPR	12.09
2290	P40967	PMEL17	DRB1*01:18	402	416	PAEVSIVVLSGTTAA	12.12
2291	P40967	PMEL17	DRB1*11:42	461	475	TATLRLVKRQVPLDC	12.16
2292	P40967	PMEL17	DRB1*01:20	284	298	GPVTAQVVLQAAIPL	12.23
2293	P40967	PMEL17	DRB1*10:01	82	96	ASFSIALNFPGSQKV	12.27
2294	P40967	PMEL17	DRB1*11:03	609	623	VVLASLIYRRRLMKQ	12.36
2295	P40967	PMEL17	DRB1*01:01	402	416	PAEVSIVVLSGTTAA	12.54
2296	P40967	PMEL17	DRB1*01:24	14	28	VIGALLAVGATKVPR	12.55
2297	P40967	PMEL17	DRB1*01:24	286	300	VTAQVVLQAAIPLTS	12.60
2298	P40967	PMEL17	DRB1*01:01	17	31	ALLAVGATKVPRNQD	12.83
2299	P40967	PMEL17	DRB1*13:96	230	244	KHFLRNQPLTFALQL	12.84
2300	P40967	PMEL17	DRB1*10:01	80	94	ANASFSIALNFPGSQ	12.86
2301	P40967	PMEL17	DRB1*01:29	286	300	VTAQVVLQAAIPLTS	12.98
2302	P40967	PMEL17	DRB1*01:11	286	300	VTAQVVLQAAIPLTS	13.13
2303	P40967	PMEL17	DRB1*01:18	17	31	ALLAVGATKVPRNQD	13.20
2304	P40967	PMEL17	DRB1*11:03	610	624	VLASLIYRRRLMKQD	13.43
2305	P40967	PMEL17	DRB1*01:18	229	243	NKHFLRNQPLTFALQ	13.46
2306	P40967	PMEL17	DRB1*01:24	287	301	TAQVVLQAAIPLTSC	13.47
2307	P40967	PMEL17	DRB1*01:29	15	29	IGALLAVGATKVPRN	13.52
2308	P40967	PMEL17	DRB1*01:20	402	416	PAEVSIVVLSGTTAA	13.64
2309	P40967	PMEL17	DRB1*01:29	13	27	AVIGALLAVGATKVP	13.65
2310	P40967	PMEL17	DRB1*01:20	17	31	ALLAVGATKVPRNQD	13.76
2311	P40967	PMEL17	DRB1*01:18	228	242	GNKHFLRNQPLTFAL	13.77
2312	P40967	PMEL17	DRB1*11:03	611	625	LASLIYRRRLMKQDF	13.82
2313	P40967	PMEL17	DRB1*01:01	79	93	GANASFSIALNFPGS	14.29
2314	P40967	PMEL17	DRB1*01:18	284	298	GPVTAQVVLQAAIPL	14.56

2315	P40967	PMEL17	DRB1*01:24	15	29	IGALLAVGATKVPRN	14.57
2316	P40967	PMEL17	DRB1*01:24	13	27	AVIGALLAVGATKVP	14.65
2317	P40967	PMEL17	DRB1*01:18	79	93	GANASFSIALNFPGS	14.74
2318	P40967	PMEL17	DRB1*01:01	284	298	GPVTAQVVLQAAIPL	14.85
2319	P40967	PMEL17	DRB1*01:24	285	299	PVTAQVVLQAAIPLT	14.92
2320	P40967	PMEL17	DRB1*04:01	81	95	NASFSIALNFPGSQK	14.94
2321	P40967	PMEL17	DRB1*13:96	227	241	GGNKHFLRNQPLTFA	14.94
2322	P40967	PMEL17	DRB1*01:11	287	301	TAQVVLQAAIPLTSC	14.97
2323	P40967	PMEL17	DRB1*11:04	551	565	QLVLHQILKGGSGTY	15.12
2324	P40967	PMEL17	DRB1*11:46	551	565	QLVLHQILKGGSGTY	15.12
2325	P40967	PMEL17	DRB1*11:58	551	565	QLVLHQILKGGSGTY	15.12
2326	P40967	PMEL17	DRB1*13:11	551	565	QLVLHQILKGGSGTY	15.12
2327	P40967	PMEL17	DRB1*01:24	288	302	AQVVLQAAIPLTSCG	15.16
2328	P40967	PMEL17	DRB1*11:02	609	623	VVLASLIYRRRLMKQ	15.16
2329	P40967	PMEL17	DRB1*11:03	608	622	AVVLASLIYRRRLMK	15.16
2330	P40967	PMEL17	DRB1*11:65	609	623	VVLASLIYRRRLMKQ	15.16
2331	P40967	PMEL17	DRB1*13:01	609	623	VVLASLIYRRRLMKQ	15.16
2332	P40967	PMEL17	DRB1*01:29	287	301	TAQVVLQAAIPLTSC	15.17
2333	P40967	PMEL17	DRB1*01:18	83	97	SFSIALNFPGSQKVL	15.18
2334	P40967	PMEL17	DRB1*11:04	462	476	ATLRLVKRQVPLDCV	15.25
2335	P40967	PMEL17	DRB1*11:46	462	476	ATLRLVKRQVPLDCV	15.25
2336	P40967	PMEL17	DRB1*11:58	462	476	ATLRLVKRQVPLDCV	15.25
2337	P40967	PMEL17	DRB1*13:11	462	476	ATLRLVKRQVPLDCV	15.25
2338	P40967	PMEL17	DRB1*10:01	79	93	GANASFSIALNFPGS	15.26
2339	P40967	PMEL17	DRB1*04:01	82	96	ASFSIALNFPGSQKV	15.42
2340	P40967	PMEL17	DRB1*11:42	462	476	ATLRLVKRQVPLDCV	15.44
2341	P40967	PMEL17	DRB1*04:08	81	95	NASFSIALNFPGSQK	15.55
2342	P40967	PMEL17	DRB1*01:02	286	300	VTAQVVLQAAIPLTS	15.58
2343	P40967	PMEL17	DRB1*01:01	154	168	KTWGQYWQVLGGPVS	15.76
2344	P40967	PMEL17	DRB1*01:24	12	26	LAVIGALLAVGATKV	16.05
2345	P40967	PMEL17	DRB1*01:02	14	28	VIGALLAVGATKVPR	16.21
2346	P40967	PMEL17	DRB1*04:08	82	96	ASFSIALNFPGSQKV	16.27
2347	P40967	PMEL17	DRB1*01:18	10	24	LHLAVIGALLAVGAT	16.46
2348	P40967	PMEL17	DRB1*11:14	100	114	GQVIWVNNTIINGSQ	16.48
2349	P40967	PMEL17	DRB1*13:02	100	114	GQVIWVNNTIINGSQ	16.48
2350	P40967	PMEL17	DRB1*13:23	100	114	GQVIWVNNTIINGSQ	16.48
2351	P40967	PMEL17	DRB1*13:97	100	114	GQVIWVNNTIINGSQ	16.48
2352	P40967	PMEL17	DRB1*04:01	80	94	ANASFSIALNFPGSQ	16.57
2353	P40967	PMEL17	DRB1*01:29	12	26	LAVIGALLAVGATKV	16.62
2354	P40967	PMEL17	DRB1*01:02	287	301	TAQVVLQAAIPLTSC	16.69
2355	P40967	PMEL17	DRB1*07:01	14	28	VIGALLAVGATKVPR	16.71
2356	P40967	PMEL17	DRB1*01:01	83	97	SFSIALNFPGSQKVL	16.75
2357	P40967	PMEL17	DRB1*01:01	159	173	YWQVLGGPVSGLSIG	16.82

2358	P40967	PMEL17	DRB1*01:11	285	299	PVTAQVVLQAAIPLT	16.88
2359	P40967	PMEL17	DRB1*11:03	612	626	ASLIYRRRLMKQDFS	16.90
2360	P40967	PMEL17	DRB1*04:08	80	94	ANASFSIALNFPGSQ	16.92
2361	P40967	PMEL17	DRB1*11:02	608	622	AVVLASLIYRRRLMK	16.96
2362	P40967	PMEL17	DRB1*11:65	608	622	AVVLASLIYRRRLMK	16.96
2363	P40967	PMEL17	DRB1*13:01	608	622	AVVLASLIYRRRLMK	16.96
2364	P40967	PMEL17	DRB1*11:14	74	88	GPTLIGANASFSIAL	17.01
2365	P40967	PMEL17	DRB1*13:02	74	88	GPTLIGANASFSIAL	17.01
2366	P40967	PMEL17	DRB1*13:23	74	88	GPTLIGANASFSIAL	17.01
2367	P40967	PMEL17	DRB1*13:97	74	88	GPTLIGANASFSIAL	17.01
2368	P40967	PMEL17	DRB1*01:18	154	168	KTWGQYWQVLGGPVS	17.05
2369	P40967	PMEL17	DRB1*01:18	230	244	KHFLRNQPLTFALQL	17.05
2370	P40967	PMEL17	DRB1*01:11	14	28	VIGALLAVGATKVPR	17.12
2371	P40967	PMEL17	DRB1*01:01	229	243	NKHFLRNQPLTFALQ	17.34
2372	P40967	PMEL17	DRB1*01:24	404	418	EVSIVVLSGTTAAQV	17.34
2373	P40967	PMEL17	DRB1*11:03	459	473	DGTATLRLVKRQVPL	17.41
2374	P40967	PMEL17	DRB1*01:29	285	299	PVTAQVVLQAAIPLT	17.43
2375	P40967	PMEL17	DRB1*11:14	99	113	DGQVIWVNNTIINGS	17.49
2376	P40967	PMEL17	DRB1*13:02	99	113	DGQVIWVNNTIINGS	17.49
2377	P40967	PMEL17	DRB1*13:23	99	113	DGQVIWVNNTIINGS	17.49
2378	P40967	PMEL17	DRB1*13:97	99	113	DGQVIWVNNTIINGS	17.49
2379	P40967	PMEL17	DRB1*01:29	157	171	GQYWQVLGGPVSGLS	17.61
2380	P40967	PMEL17	DRB1*11:14	231	245	HFLRNQPLTFALQLH	17.65
2381	P40967	PMEL17	DRB1*13:02	231	245	HFLRNQPLTFALQLH	17.65
2382	P40967	PMEL17	DRB1*13:23	231	245	HFLRNQPLTFALQLH	17.65
2383	P40967	PMEL17	DRB1*13:97	231	245	HFLRNQPLTFALQLH	17.65
2384	P40967	PMEL17	DRB1*01:11	15	29	IGALLAVGATKVPRN	17.66
2385	P40967	PMEL17	DRB1*11:14	75	89	PTLIGANASFSIALN	17.72
2386	P40967	PMEL17	DRB1*13:02	75	89	PTLIGANASFSIALN	17.72
2387	P40967	PMEL17	DRB1*13:23	75	89	PTLIGANASFSIALN	17.72
2388	P40967	PMEL17	DRB1*13:97	75	89	PTLIGANASFSIALN	17.72
2389	P40967	PMEL17	DRB1*11:04	550	564	CQLVLHQILKGGSGT	17.75
2390	P40967	PMEL17	DRB1*11:46	550	564	CQLVLHQILKGGSGT	17.75
2391	P40967	PMEL17	DRB1*11:58	550	564	CQLVLHQILKGGSGT	17.75
2392	P40967	PMEL17	DRB1*13:11	550	564	CQLVLHQILKGGSGT	17.75
2393	P40967	PMEL17	DRB1*01:29	156	170	WGQYWQVLGGPVSGL	17.78
2394	P40967	PMEL17	DRB1*01:29	288	302	AQVVLQAAIPLTSCG	17.87
2395	P40967	PMEL17	DRB1*01:01	228	242	GNKHFLRNQPLTFAL	17.88
2396	P40967	PMEL17	DRB1*11:03	460	474	GTATLRLVKRQVPLD	17.89
2397	P40967	PMEL17	DRB1*11:01	146	160	KRSFVYVWKTWGQYW	17.94
2398	P40967	PMEL17	DRB1*11:10	146	160	KRSFVYVWKTWGQYW	17.94
2399	P40967	PMEL17	DRB1*11:12	146	160	KRSFVYVWKTWGQYW	17.94
2400	P40967	PMEL17	DRB1*11:28	146	160	KRSFVYVWKTWGQYW	17.94

2401	P40967	PMEL17	DRB1*11:29	146	160	KRSFVYVWKTWGQYW	17.94
2402	P40967	PMEL17	DRB1*11:49	146	160	KRSFVYVWKTWGQYW	17.94
2403	P40967	PMEL17	DRB1*11:62	146	160	KRSFVYVWKTWGQYW	17.94
2404	P40967	PMEL17	DRB1*11:74	146	160	KRSFVYVWKTWGQYW	17.94
2405	P40967	PMEL17	DRB1*13:05	146	160	KRSFVYVWKTWGQYW	17.94
2406	P40967	PMEL17	DRB1*13:14	146	160	KRSFVYVWKTWGQYW	17.94
2407	P40967	PMEL17	DRB1*13:50	146	160	KRSFVYVWKTWGQYW	17.94
2408	P40967	PMEL17	DRB1*01:02	285	299	PVTAQVVLQAAIPLT	18.24
2409	P40967	PMEL17	DRB1*11:14	226	240	DGGNKHFLRNQPLTF	18.26
2410	P40967	PMEL17	DRB1*13:02	226	240	DGGNKHFLRNQPLTF	18.26
2411	P40967	PMEL17	DRB1*13:23	226	240	DGGNKHFLRNQPLTF	18.26
2412	P40967	PMEL17	DRB1*13:97	226	240	DGGNKHFLRNQPLTF	18.26
2413	P40967	PMEL17	DRB1*11:01	459	473	DGTATLRLVKRQVPL	18.27
2414	P40967	PMEL17	DRB1*11:10	459	473	DGTATLRLVKRQVPL	18.27
2415	P40967	PMEL17	DRB1*11:12	459	473	DGTATLRLVKRQVPL	18.27
2416	P40967	PMEL17	DRB1*11:28	459	473	DGTATLRLVKRQVPL	18.27
2417	P40967	PMEL17	DRB1*11:29	459	473	DGTATLRLVKRQVPL	18.27
2418	P40967	PMEL17	DRB1*11:49	459	473	DGTATLRLVKRQVPL	18.27
2419	P40967	PMEL17	DRB1*11:62	459	473	DGTATLRLVKRQVPL	18.27
2420	P40967	PMEL17	DRB1*11:74	459	473	DGTATLRLVKRQVPL	18.27
2421	P40967	PMEL17	DRB1*13:05	459	473	DGTATLRLVKRQVPL	18.27
2422	P40967	PMEL17	DRB1*13:14	459	473	DGTATLRLVKRQVPL	18.27
2423	P40967	PMEL17	DRB1*13:50	459	473	DGTATLRLVKRQVPL	18.27
2424	P40967	PMEL17	DRB1*11:01	145	159	QKRSFVYVWKTWGQY	18.30
2425	P40967	PMEL17	DRB1*11:10	145	159	QKRSFVYVWKTWGQY	18.30
2426	P40967	PMEL17	DRB1*11:12	145	159	QKRSFVYVWKTWGQY	18.30
2427	P40967	PMEL17	DRB1*11:28	145	159	QKRSFVYVWKTWGQY	18.30
2428	P40967	PMEL17	DRB1*11:29	145	159	QKRSFVYVWKTWGQY	18.30
2429	P40967	PMEL17	DRB1*11:49	145	159	QKRSFVYVWKTWGQY	18.30
2430	P40967	PMEL17	DRB1*11:62	145	159	QKRSFVYVWKTWGQY	18.30
2431	P40967	PMEL17	DRB1*11:74	145	159	QKRSFVYVWKTWGQY	18.30
2432	P40967	PMEL17	DRB1*13:05	145	159	QKRSFVYVWKTWGQY	18.30
2433	P40967	PMEL17	DRB1*13:14	145	159	QKRSFVYVWKTWGQY	18.30
2434	P40967	PMEL17	DRB1*13:50	145	159	QKRSFVYVWKTWGQY	18.30
2435	P40967	PMEL17	DRB1*01:24	405	419	VSIVVLSGTTAAQVT	18.37
2436	P40967	PMEL17	DRB1*01:29	404	418	EVSIVVLSGTTAAQV	18.37
2437	P40967	PMEL17	DRB1*01:02	288	302	AQVVLQAAIPLTSCG	18.38
2438	P40967	PMEL17	DRB1*10:01	83	97	SFSIALNFPGSQKVL	18.44
2439	P40967	PMEL17	DRB1*11:14	101	115	QVIWVNNTIINGSQV	18.45
2440	P40967	PMEL17	DRB1*13:02	101	115	QVIWVNNTIINGSQV	18.45
2441	P40967	PMEL17	DRB1*13:23	101	115	QVIWVNNTIINGSQV	18.45
2442	P40967	PMEL17	DRB1*13:97	101	115	QVIWVNNTIINGSQV	18.45
2443	P40967	PMEL17	DRB1*01:18	227	241	GGNKHFLRNQPLTFA	18.46

2444	P40967	PMEL17	DRB1*11:42	609	623	VVLASLIYRRRLMKQ	18.47
2445	P40967	PMEL17	DRB1*07:01	15	29	IGALLAVGATKVPRN	18.72
2446	P40967	PMEL17	DRB1*11:42	608	622	AVVLASLIYRRRLMK	18.72
2447	P40967	PMEL17	DRB1*01:18	6	20	KRCLLHLAVIGALLA	18.73
2448	P40967	PMEL17	DRB1*01:02	13	27	AVIGALLAVGATKVP	18.78
2449	P40967	PMEL17	DRB1*01:11	288	302	AQVVLQAAIPLTSCG	18.79
2450	P40967	PMEL17	DRB1*11:02	610	624	VLASLIYRRRLMKQD	18.87
2451	P40967	PMEL17	DRB1*11:65	610	624	VLASLIYRRRLMKQD	18.87
2452	P40967	PMEL17	DRB1*13:01	610	624	VLASLIYRRRLMKQD	18.87
2453	P40967	PMEL17	DRB1*11:42	458	472	LDGTATLRLVKRQVP	19.07
2454	P40967	PMEL17	DRB1*04:01	79	93	GANASFSIALNFPGS	19.17
2455	P40967	PMEL17	DRB1*01:29	16	30	GALLAVGATKVPRNQ	19.18
2456	P40967	PMEL17	DRB1*01:24	156	170	WGQYWQVLGGPVSGL	19.21
2457	P40967	PMEL17	DRB1*11:04	609	623	VVLASLIYRRRLMKQ	19.21
2458	P40967	PMEL17	DRB1*11:46	609	623	VVLASLIYRRRLMKQ	19.21
2459	P40967	PMEL17	DRB1*11:58	609	623	VVLASLIYRRRLMKQ	19.21
2460	P40967	PMEL17	DRB1*13:11	609	623	VVLASLIYRRRLMKQ	19.21
2461	P40967	PMEL17	DRB1*01:11	404	418	EVSIVVLSGTTAAQV	19.29
2462	P40967	PMEL17	DRB1*07:01	13	27	AVIGALLAVGATKVP	19.40
2463	P40967	PMEL17	DRB1*01:24	157	171	GQYWQVLGGPVSGLS	19.53
2464	P40967	PMEL17	DRB1*11:14	98	112	PDGQVIWVNNTIING	19.68
2465	P40967	PMEL17	DRB1*13:02	98	112	PDGQVIWVNNTIING	19.68
2466	P40967	PMEL17	DRB1*13:23	98	112	PDGQVIWVNNTIING	19.68
2467	P40967	PMEL17	DRB1*13:97	98	112	PDGQVIWVNNTIING	19.68
2468	P40967	PMEL17	DRB1*01:20	10	24	LHLAVIGALLAVGAT	19.84
2469	P40967	PMEL17	DRB1*04:08	79	93	GANASFSIALNFPGS	19.98
2470	P40967	PMEL17	DRB1*11:04	549	563	ACQLVLHQILKGGSG	20.08
2471	P40967	PMEL17	DRB1*11:46	549	563	ACQLVLHQILKGGSG	20.08
2472	P40967	PMEL17	DRB1*11:58	549	563	ACQLVLHQILKGGSG	20.08
2473	P40967	PMEL17	DRB1*13:11	549	563	ACQLVLHQILKGGSG	20.08
2474	P40967	PMEL17	DRB1*11:13	459	473	DGTATLRLVKRQVPL	20.09
2475	P40967	PMEL17	DRB1*01:01	10	24	LHLAVIGALLAVGAT	20.12
2476	P40967	PMEL17	DRB1*11:13	460	474	GTATLRLVKRQVPLD	20.13
2477	P40967	PMEL17	DRB1*11:01	460	474	GTATLRLVKRQVPLD	20.16
2478	P40967	PMEL17	DRB1*11:10	460	474	GTATLRLVKRQVPLD	20.16
2479	P40967	PMEL17	DRB1*11:12	460	474	GTATLRLVKRQVPLD	20.16
2480	P40967	PMEL17	DRB1*11:28	460	474	GTATLRLVKRQVPLD	20.16
2481	P40967	PMEL17	DRB1*11:29	460	474	GTATLRLVKRQVPLD	20.16
2482	P40967	PMEL17	DRB1*11:49	460	474	GTATLRLVKRQVPLD	20.16
2483	P40967	PMEL17	DRB1*11:62	460	474	GTATLRLVKRQVPLD	20.16
2484	P40967	PMEL17	DRB1*11:74	460	474	GTATLRLVKRQVPLD	20.16
2485	P40967	PMEL17	DRB1*13:05	460	474	GTATLRLVKRQVPLD	20.16
2486	P40967	PMEL17	DRB1*13:14	460	474	GTATLRLVKRQVPLD	20.16

2487	P40967	PMEL17	DRB1*13:50	460	474	GTATLRLVKRQVPLD	20.16
2488	P40967	PMEL17	DRB1*11:03	461	475	TATLRLVKRQVPLDC	20.22
2489	P40967	PMEL17	DRB1*11:14	76	90	TLIGANASFSIALNF	20.23
2490	P40967	PMEL17	DRB1*13:02	76	90	TLIGANASFSIALNF	20.23
2491	P40967	PMEL17	DRB1*13:23	76	90	TLIGANASFSIALNF	20.23
2492	P40967	PMEL17	DRB1*13:97	76	90	TLIGANASFSIALNF	20.23
2493	P40967	PMEL17	DRB1*07:01	12	26	LAVIGALLAVGATKV	20.25
2494	P40967	PMEL17	DRB1*01:02	15	29	IGALLAVGATKVPRN	20.27
2495	P40967	PMEL17	DRB1*01:18	159	173	YWQVLGGPVSGLSIG	20.35
2496	P40967	PMEL17	DRB1*01:29	81	95	NASFSIALNFPGSQK	20.38
2497	P40967	PMEL17	DRB1*11:04	552	566	LVLHQILKGGSGTYC	20.38
2498	P40967	PMEL17	DRB1*11:46	552	566	LVLHQILKGGSGTYC	20.38
2499	P40967	PMEL17	DRB1*11:58	552	566	LVLHQILKGGSGTYC	20.38
2500	P40967	PMEL17	DRB1*13:11	552	566	LVLHQILKGGSGTYC	20.38
2501	P40967	PMEL17	DRB1*01:01	230	244	KHFLRNQPLTFALQL	20.40
2502	P40967	PMEL17	DRB1*01:24	11	25	HLAVIGALLAVGATK	20.49
2503	P40967	PMEL17	DRB1*01:24	16	30	GALLAVGATKVPRNQ	20.50
2504	P40967	PMEL17	DRB1*01:29	403	417	AEVSIVVLSGTTAAQ	20.55
2505	P40967	PMEL17	DRB1*11:14	286	300	VTAQVVLQAAIPLTS	20.55
2506	P40967	PMEL17	DRB1*13:02	286	300	VTAQVVLQAAIPLTS	20.55
2507	P40967	PMEL17	DRB1*13:23	286	300	VTAQVVLQAAIPLTS	20.55
2508	P40967	PMEL17	DRB1*13:97	286	300	VTAQVVLQAAIPLTS	20.55
2509	P40967	PMEL17	DRB1*10:01	289	303	QVVLQAAIPLTSCGS	20.57
2510	P40967	PMEL17	DRB1*10:01	156	170	WGQYWQVLGGPVSGL	20.64
2511	P40967	PMEL17	DRB1*01:29	11	25	HLAVIGALLAVGATK	20.77
2512	P40967	PMEL17	DRB1*01:24	403	417	AEVSIVVLSGTTAAQ	20.78
2513	P40967	PMEL17	DRB1*11:03	184	198	MEVTVYHRRGSRSYV	20.84
2514	P40967	PMEL17	DRB1*11:04	608	622	AVVLASLIYRRRLMK	20.89
2515	P40967	PMEL17	DRB1*11:46	608	622	AVVLASLIYRRRLMK	20.89
2516	P40967	PMEL17	DRB1*11:58	608	622	AVVLASLIYRRRLMK	20.89
2517	P40967	PMEL17	DRB1*13:11	608	622	AVVLASLIYRRRLMK	20.89
2518	P40967	PMEL17	DRB1*11:02	611	625	LASLIYRRRLMKQDF	21.10
2519	P40967	PMEL17	DRB1*11:65	611	625	LASLIYRRRLMKQDF	21.10
2520	P40967	PMEL17	DRB1*13:01	611	625	LASLIYRRRLMKQDF	21.10
2521	P40967	PMEL17	DRB1*13:21	459	473	DGTATLRLVKRQVPL	21.14
2522	P40967	PMEL17	DRB1*15:01	474	488	DCVLYRYGSFSVTLD	21.25
2523	P40967	PMEL17	DRB1*15:06	474	488	DCVLYRYGSFSVTLD	21.25
2524	P40967	PMEL17	DRB1*10:01	288	302	AQVVLQAAIPLTSCG	21.34
2525	P40967	PMEL17	DRB1*01:11	405	419	VSIVVLSGTTAAQVT	21.57
2526	P40967	PMEL17	DRB1*10:01	157	171	GQYWQVLGGPVSGLS	21.64
2527	P40967	PMEL17	DRB1*11:01	144	158	SQKRSFVYVWKTWGQ	21.74
2528	P40967	PMEL17	DRB1*11:10	144	158	SQKRSFVYVWKTWGQ	21.74
2529	P40967	PMEL17	DRB1*11:12	144	158	SQKRSFVYVWKTWGQ	21.74

2530	P40967	PMEL17	DRB1*11:28	144	158	SQKRSFVYVWKTWGQ	21.74
2531	P40967	PMEL17	DRB1*11:29	144	158	SQKRSFVYVWKTWGQ	21.74
2532	P40967	PMEL17	DRB1*11:49	144	158	SQKRSFVYVWKTWGQ	21.74
2533	P40967	PMEL17	DRB1*11:62	144	158	SQKRSFVYVWKTWGQ	21.74
2534	P40967	PMEL17	DRB1*11:74	144	158	SQKRSFVYVWKTWGQ	21.74
2535	P40967	PMEL17	DRB1*13:05	144	158	SQKRSFVYVWKTWGQ	21.74
2536	P40967	PMEL17	DRB1*13:14	144	158	SQKRSFVYVWKTWGQ	21.74
2537	P40967	PMEL17	DRB1*13:50	144	158	SQKRSFVYVWKTWGQ	21.74
2538	P40967	PMEL17	DRB1*11:02	607	621	MAVVLASLIYRRRLM	21.84
2539	P40967	PMEL17	DRB1*11:65	607	621	MAVVLASLIYRRRLM	21.84
2540	P40967	PMEL17	DRB1*13:01	607	621	MAVVLASLIYRRRLM	21.84
2541	P40967	PMEL17	DRB1*01:20	167	181	VSGLSIGTGRAMLGT	21.94
2542	P40967	PMEL17	DRB1*01:29	405	419	VSIVVLSGTTAAQVT	21.95
2543	P40967	PMEL17	DRB1*10:01	287	301	TAQVVLQAAIPLTSC	21.99
2544	P40967	PMEL17	DRB1*01:24	81	95	NASFSIALNFPGSQK	22.02
2545	P40967	PMEL17	DRB1*11:03	183	197	TMEVTVYHRRGSRSY	22.12
2546	P40967	PMEL17	DRB1*11:04	458	472	LDGTATLRLVKRQVP	22.43
2547	P40967	PMEL17	DRB1*11:46	458	472	LDGTATLRLVKRQVP	22.43
2548	P40967	PMEL17	DRB1*11:58	458	472	LDGTATLRLVKRQVP	22.43
2549	P40967	PMEL17	DRB1*13:11	458	472	LDGTATLRLVKRQVP	22.43
2550	P40967	PMEL17	DRB1*01:24	289	303	QVVLQAAIPLTSCGS	22.49
2551	P40967	PMEL17	DRB1*01:11	13	27	AVIGALLAVGATKVP	22.56
2552	P40967	PMEL17	DRB1*01:20	265	279	SSGTLISRALVVTHT	22.66
2553	P40967	PMEL17	DRB1*01:18	5	19	LKRCLLHLAVIGALL	22.72
2554	P40967	PMEL17	DRB1*15:01	472	486	PLDCVLYRYGSFSVT	22.75
2555	P40967	PMEL17	DRB1*15:06	472	486	PLDCVLYRYGSFSVT	22.75
2556	P40967	PMEL17	DRB1*11:42	610	624	VLASLIYRRRLMKQD	22.78
2557	P40967	PMEL17	DRB1*10:01	286	300	VTAQVVLQAAIPLTS	22.84
2558	P40967	PMEL17	DRB1*11:04	610	624	VLASLIYRRRLMKQD	22.87
2559	P40967	PMEL17	DRB1*11:46	610	624	VLASLIYRRRLMKQD	22.87
2560	P40967	PMEL17	DRB1*11:58	610	624	VLASLIYRRRLMKQD	22.87
2561	P40967	PMEL17	DRB1*13:11	610	624	VLASLIYRRRLMKQD	22.87
2562	P40967	PMEL17	DRB1*15:01	476	490	VLYRYGSFSVTLDIV	22.92
2563	P40967	PMEL17	DRB1*15:06	476	490	VLYRYGSFSVTLDIV	22.92
2564	P40967	PMEL17	DRB1*11:02	459	473	DGTATLRLVKRQVPL	22.93
2565	P40967	PMEL17	DRB1*11:65	459	473	DGTATLRLVKRQVPL	22.93
2566	P40967	PMEL17	DRB1*13:01	459	473	DGTATLRLVKRQVPL	22.93
2567	P40967	PMEL17	DRB1*01:18	9	23	LLHLAVIGALLAVGA	23.03
2568	P40967	PMEL17	DRB1*01:20	81	95	NASFSIALNFPGSQK	23.03
2569	P40967	PMEL17	DRB1*14:02	229	243	NKHFLRNQPLTFALQ	23.05
2570	P40967	PMEL17	DRB1*11:01	461	475	TATLRLVKRQVPLDC	23.06
2571	P40967	PMEL17	DRB1*11:10	461	475	TATLRLVKRQVPLDC	23.06
2572	P40967	PMEL17	DRB1*11:12	461	475	TATLRLVKRQVPLDC	23.06

2573	P40967	PMEL17	DRB1*11:28	461	475	TATLRLVKRQVPLDC	23.06
2574	P40967	PMEL17	DRB1*11:29	461	475	TATLRLVKRQVPLDC	23.06
2575	P40967	PMEL17	DRB1*11:49	461	475	TATLRLVKRQVPLDC	23.06
2576	P40967	PMEL17	DRB1*11:62	461	475	TATLRLVKRQVPLDC	23.06
2577	P40967	PMEL17	DRB1*11:74	461	475	TATLRLVKRQVPLDC	23.06
2578	P40967	PMEL17	DRB1*13:05	461	475	TATLRLVKRQVPLDC	23.06
2579	P40967	PMEL17	DRB1*13:14	461	475	TATLRLVKRQVPLDC	23.06
2580	P40967	PMEL17	DRB1*13:50	461	475	TATLRLVKRQVPLDC	23.06
2581	P40967	PMEL17	DRB1*01:01	227	241	GGNKHFLRNQPLTFA	23.30
2582	P40967	PMEL17	DRB1*13:96	231	245	HFLRNQPLTFALQLH	23.40
2583	P40967	PMEL17	DRB1*01:29	158	172	QYWQVLGGPVSGLSI	23.44
2584	P40967	PMEL17	DRB1*01:24	406	420	SIVVLSGTTAAQVTT	23.48
2585	P40967	PMEL17	DRB1*13:96	100	114	GQVIWVNNTIINGSQ	23.52
2586	P40967	PMEL17	DRB1*11:13	461	475	TATLRLVKRQVPLDC	23.54
2587	P40967	PMEL17	DRB1*01:24	82	96	ASFSIALNFPGSQKV	23.59
2588	P40967	PMEL17	DRB1*11:14	97	111	LPDGQVIWVNNTIIN	23.66
2589	P40967	PMEL17	DRB1*13:02	97	111	LPDGQVIWVNNTIIN	23.66
2590	P40967	PMEL17	DRB1*13:23	97	111	LPDGQVIWVNNTIIN	23.66
2591	P40967	PMEL17	DRB1*13:97	97	111	LPDGQVIWVNNTIIN	23.66
2592	P40967	PMEL17	DRB1*01:11	403	417	AEVSIVVLSGTTAAQ	23.79
2593	P40967	PMEL17	DRB1*11:03	613	627	SLIYRRRLMKQDFSV	23.80
2594	P40967	PMEL17	DRB1*01:20	407	421	IVVLSGTTAAQVTTT	23.88
2595	P40967	PMEL17	DRB1*01:20	229	243	NKHFLRNQPLTFALQ	23.92
2596	P40967	PMEL17	DRB1*01:20	82	96	ASFSIALNFPGSQKV	23.94
2597	P40967	PMEL17	DRB1*01:18	4	18	VLKRCLLHLAVIGAL	23.95
2598	P40967	PMEL17	DRB1*01:29	289	303	QVVLQAAIPLTSCGS	24.04
2599	P40967	PMEL17	DRB1*16:02	81	95	NASFSIALNFPGSQK	24.09
2600	P40967	PMEL17	DRB1*01:20	74	88	GPTLIGANASFSIAL	24.11
2601	P40967	PMEL17	DRB1*11:42	607	621	MAVVLASLIYRRRLM	24.17
2602	P40967	PMEL17	DRB1*07:01	264	278	DSSGTLISRALVVTH	24.30
2603	P40967	PMEL17	DRB1*15:01	473	487	LDCVLYRYGSFSVTL	24.33
2604	P40967	PMEL17	DRB1*15:06	473	487	LDCVLYRYGSFSVTL	24.33
2605	P40967	PMEL17	DRB1*11:14	73	87	DGPTLIGANASFSIA	24.48
2606	P40967	PMEL17	DRB1*13:02	73	87	DGPTLIGANASFSIA	24.48
2607	P40967	PMEL17	DRB1*13:23	73	87	DGPTLIGANASFSIA	24.48
2608	P40967	PMEL17	DRB1*13:97	73	87	DGPTLIGANASFSIA	24.48
2609	P40967	PMEL17	DRB1*08:04	459	473	DGTATLRLVKRQVPL	24.49
2610	P40967	PMEL17	DRB1*01:20	266	280	SGTLISRALVVTHTY	24.50
2611	P40967	PMEL17	DRB1*01:24	158	172	QYWQVLGGPVSGLSI	24.54
2612	P40967	PMEL17	DRB1*01:20	228	242	GNKHFLRNQPLTFAL	24.55
2613	P40967	PMEL17	DRB1*01:20	401	415	TPAEVSIVVLSGTTA	24.58
2614	P40967	PMEL17	DRB1*14:02	228	242	GNKHFLRNQPLTFAL	24.65
2615	P40967	PMEL17	DRB1*11:14	285	299	PVTAQVVLQAAIPLT	24.67

2616	P40967	PMEL17	DRB1*13:02	285	299	PVTAQVVLQAAIPLT	24.67
2617	P40967	PMEL17	DRB1*13:23	285	299	PVTAQVVLQAAIPLT	24.67
2618	P40967	PMEL17	DRB1*13:97	285	299	PVTAQVVLQAAIPLT	24.67
2619	P40967	PMEL17	DRB1*11:13	608	622	AVVLASLIYRRRLMK	24.83
2620	P40967	PMEL17	DRB1*01:02	289	303	QVVLQAAIPLTSCGS	24.97
2621	P40967	PMEL17	DRB1*13:96	101	115	QVIWVNNTIINGSQV	24.99
2622	P40967	PMEL17	DRB1*07:01	263	277	GDSSGTLISRALVVT	25.03
2623	P40967	PMEL17	DRB1*04:05	477	491	LYRYGSFSVTLDIVQ	25.04
2624	P40967	PMEL17	DRB1*11:42	551	565	QLVLHQILKGGSGTY	25.08
2625	P40967	PMEL17	DRB1*01:29	82	96	ASFSIALNFPGSQKV	25.21
2626	P40967	PMEL17	DRB1*11:03	462	476	ATLRLVKRQVPLDCV	25.27
2627	P40967	PMEL17	DRB1*01:20	166	180	PVSGLSIGTGRAMLG	25.47
2628	P40967	PMEL17	DRB1*01:18	195	209	RSYVPLAHSSSAFTI	25.50
2629	P40967	PMEL17	DRB1*13:96	99	113	DGQVIWVNNTIINGS	25.54
2630	P40967	PMEL17	DRB1*01:20	156	170	WGQYWQVLGGPVSGL	25.56
2631	P40967	PMEL17	DRB1*04:04	405	419	VSIVVLSGTTAAQVT	25.56
2632	P40967	PMEL17	DRB1*11:13	607	621	MAVVLASLIYRRRLM	25.56
2633	P40967	PMEL17	DRB1*01:02	12	26	LAVIGALLAVGATKV	25.58
2634	P40967	PMEL17	DRB1*01:20	157	171	GQYWQVLGGPVSGLS	25.60
2635	P40967	PMEL17	DRB1*01:20	290	304	VVLQAAIPLTSCGSS	25.60
2636	P40967	PMEL17	DRB1*01:11	12	26	LAVIGALLAVGATKV	25.64
2637	P40967	PMEL17	DRB1*13:21	460	474	GTATLRLVKRQVPLD	25.67
2638	P40967	PMEL17	DRB1*01:11	16	30	GALLAVGATKVPRNQ	25.72
2639	P40967	PMEL17	DRB1*01:24	402	416	PAEVSIVVLSGTTAA	25.74
2640	P40967	PMEL17	DRB1*01:29	155	169	TWGQYWQVLGGPVSG	25.74
2641	P40967	PMEL17	DRB1*13:96	226	240	DGGNKHFLRNQPLTF	25.84
2642	P40967	PMEL17	DRB1*10:01	158	172	QYWQVLGGPVSGLSI	26.21
2643	P40967	PMEL17	DRB1*01:29	406	420	SIVVLSGTTAAQVTT	26.34
2644	P40967	PMEL17	DRB1*11:42	463	477	TLRLVKRQVPLDCVL	26.46
2645	P40967	PMEL17	DRB1*01:20	230	244	KHFLRNQPLTFALQL	26.49
2646	P40967	PMEL17	DRB1*01:02	16	30	GALLAVGATKVPRNQ	26.56
2647	P40967	PMEL17	DRB1*11:02	460	474	GTATLRLVKRQVPLD	26.58
2648	P40967	PMEL17	DRB1*11:65	460	474	GTATLRLVKRQVPLD	26.58
2649	P40967	PMEL17	DRB1*13:01	460	474	GTATLRLVKRQVPLD	26.58
2650	P40967	PMEL17	DRB1*11:04	611	625	LASLIYRRRLMKQDF	26.62
2651	P40967	PMEL17	DRB1*11:46	611	625	LASLIYRRRLMKQDF	26.62
2652	P40967	PMEL17	DRB1*11:58	611	625	LASLIYRRRLMKQDF	26.62
2653	P40967	PMEL17	DRB1*13:11	611	625	LASLIYRRRLMKQDF	26.62
2654	P40967	PMEL17	DRB1*08:04	460	474	GTATLRLVKRQVPLD	26.63
2655	P40967	PMEL17	DRB1*04:05	476	490	VLYRYGSFSVTLDIV	26.64
2656	P40967	PMEL17	DRB1*07:01	265	279	SSGTLISRALVVTHT	26.64
2657	P40967	PMEL17	DRB1*11:02	606	620	LMAVVLASLIYRRRL	26.81
2658	P40967	PMEL17	DRB1*11:65	606	620	LMAVVLASLIYRRRL	26.81

2659	P40967	PMEL17	DRB1*13:01	606	620	LMAVVLASLIYRRRL	26.81
2660	P40967	PMEL17	DRB1*14:02	230	244	KHFLRNQPLTFALQL	26.81
2661	P40967	PMEL17	DPB1*33:01	476	490	VLYRYGSFSVTLDIV	26.84
2662	P40967	PMEL17	DPB1*71:01	476	490	VLYRYGSFSVTLDIV	26.84
2663	P40967	PMEL17	DRB1*04:01	83	97	SFSIALNFPGSQKVL	26.88
2664	P40967	PMEL17	DRB1*01:11	157	171	GQYWQVLGGPVSGLS	27.02
2665	P40967	PMEL17	DRB1*01:18	407	421	IVVLSGTTAAQVTTT	27.09
2666	P40967	PMEL17	DRB1*11:84	459	473	DGTATLRLVKRQVPL	27.15
2667	P40967	PMEL17	DRB1*01:11	156	170	WGQYWQVLGGPVSGL	27.26
2668	P40967	PMEL17	DRB1*01:29	80	94	ANASFSIALNFPGSQ	27.26
2669	P40967	PMEL17	DRB1*16:01	81	95	NASFSIALNFPGSQK	27.36
2670	P40967	PMEL17	DRB1*15:01	475	489	CVLYRYGSFSVTLDI	27.48
2671	P40967	PMEL17	DRB1*15:06	475	489	CVLYRYGSFSVTLDI	27.48
2672	P40967	PMEL17	DRB1*10:01	195	209	RSYVPLAHSSSAFTI	27.52
2673	P40967	PMEL17	DRB1*01:20	264	278	DSSGTLISRALVVTH	27.54
2674	P40967	PMEL17	DRB1*07:01	262	276	FGDSSGTLISRALVV	27.55
2675	P40967	PMEL17	DRB1*04:04	404	418	EVSIVVLSGTTAAQV	27.59
2676	P40967	PMEL17	DRB1*11:42	606	620	LMAVVLASLIYRRRL	27.63
2677	P40967	PMEL17	DRB1*13:66	228	242	GNKHFLRNQPLTFAL	27.75
2678	P40967	PMEL17	DRB1*01:24	80	94	ANASFSIALNFPGSQ	27.76
2679	P40967	PMEL17	DRB1*11:03	185	199	EVTVYHRRGSRSYVP	27.77
2680	P40967	PMEL17	DRB1*01:20	6	20	KRCLLHLAVIGALLA	27.86
2681	P40967	PMEL17	DRB1*13:96	74	88	GPTLIGANASFSIAL	27.88
2682	P40967	PMEL17	DRB1*01:01	195	209	RSYVPLAHSSSAFTI	27.89
2683	P40967	PMEL17	DRB1*11:14	83	97	SFSIALNFPGSQKVL	27.92
2684	P40967	PMEL17	DRB1*13:02	83	97	SFSIALNFPGSQKVL	27.92
2685	P40967	PMEL17	DRB1*13:23	83	97	SFSIALNFPGSQKVL	27.92
2686	P40967	PMEL17	DRB1*13:97	83	97	SFSIALNFPGSQKVL	27.92
2687	P40967	PMEL17	DRB1*01:11	229	243	NKHFLRNQPLTFALQ	27.95
2688	P40967	PMEL17	DRB1*01:18	401	415	TPAEVSIVVLSGTTA	28.06
2689	P40967	PMEL17	DRB1*01:01	6	20	KRCLLHLAVIGALLA	28.10
2690	P40967	PMEL17	DRB1*11:08	229	243	NKHFLRNQPLTFALQ	28.13
2691	P40967	PMEL17	DRB1*07:01	16	30	GALLAVGATKVPRNQ	28.16
2692	P40967	PMEL17	DRB1*11:13	606	620	LMAVVLASLIYRRRL	28.16
2693	P40967	PMEL17	DRB1*11:42	550	564	CQLVLHQILKGGSGT	28.37
2694	P40967	PMEL17	DRB1*16:02	80	94	ANASFSIALNFPGSQ	28.41
2695	P40967	PMEL17	DRB1*10:01	155	169	TWGQYWQVLGGPVSG	28.49
2696	P40967	PMEL17	DRB1*01:20	216	230	SVSVSQLRALDGGNK	28.54
2697	P40967	PMEL17	DRB1*01:18	265	279	SSGTLISRALVVTHT	28.55
2698	P40967	PMEL17	DRB1*04:08	83	97	SFSIALNFPGSQKVL	28.57
2699	P40967	PMEL17	DRB1*01:01	401	415	TPAEVSIVVLSGTTA	28.59
2700	P40967	PMEL17	DRB1*01:11	289	303	QVVLQAAIPLTSCGS	28.61
2701	P40967	PMEL17	DRB1*11:02	461	475	TATLRLVKRQVPLDC	28.64

2702	P40967	PMEL17	DRB1*11:65	461	475	TATLRLVKRQVPLDC	28.64
2703	P40967	PMEL17	DRB1*13:01	461	475	TATLRLVKRQVPLDC	28.64
2704	P40967	PMEL17	DRB1*01:20	75	89	PTLIGANASFSIALN	28.65
2705	P40967	PMEL17	DRB1*11:13	609	623	VVLASLIYRRRLMKQ	28.73
2706	P40967	PMEL17	DRB1*10:01	13	27	AVIGALLAVGATKVP	28.81
2707	P40967	PMEL17	DRB1*01:20	267	281	GTLISRALVVTHTYL	28.88
2708	P40967	PMEL17	DRB1*11:42	611	625	LASLIYRRRLMKQDF	28.95
2709	P40967	PMEL17	DRB1*01:18	266	280	SGTLISRALVVTHTY	29.00
2710	P40967	PMEL17	DRB1*11:84	460	474	GTATLRLVKRQVPLD	29.01
2711	P40967	PMEL17	DRB1*16:01	82	96	ASFSIALNFPGSQKV	29.02
2712	P40967	PMEL17	DRB1*11:14	287	301	TAQVVLQAAIPLTSC	29.07
2713	P40967	PMEL17	DRB1*13:02	287	301	TAQVVLQAAIPLTSC	29.07
2714	P40967	PMEL17	DRB1*13:23	287	301	TAQVVLQAAIPLTSC	29.07
2715	P40967	PMEL17	DRB1*13:97	287	301	TAQVVLQAAIPLTSC	29.07
2716	P40967	PMEL17	DRB1*01:11	228	242	GNKHFLRNQPLTFAL	29.17
2717	P40967	PMEL17	DRB1*13:96	75	89	PTLIGANASFSIALN	29.17
2718	P40967	PMEL17	DRB1*01:02	284	298	GPVTAQVVLQAAIPL	29.20
2719	P40967	PMEL17	DRB1*13:21	461	475	TATLRLVKRQVPLDC	29.21
2720	P40967	PMEL17	DRB1*01:29	17	31	ALLAVGATKVPRNQD	29.26
2721	P40967	PMEL17	DRB1*11:03	182	196	HTMEVTVYHRRGSRS	29.26
2722	P40967	PMEL17	DRB1*01:18	194	208	SRSYVPLAHSSSAFT	29.32
2723	P40967	PMEL17	DRB1*01:01	196	210	SYVPLAHSSSAFTIT	29.38
2724	P40967	PMEL17	DRB1*13:66	229	243	NKHFLRNQPLTFALQ	29.47
2725	P40967	PMEL17	DRB1*11:03	607	621	MAVVLASLIYRRRLM	29.58
2726	P40967	PMEL17	DRB1*07:01	405	419	VSIVVLSGTTAAQVT	29.66
2727	P40967	PMEL17	DRB1*16:02	82	96	ASFSIALNFPGSQKV	29.77
2728	P40967	PMEL17	DRB1*01:24	6	20	KRCLLHLAVIGALLA	29.79
2729	P40967	PMEL17	DRB1*01:29	402	416	PAEVSIVVLSGTTAA	29.81
2730	P40967	PMEL17	DRB1*10:01	78	92	IGANASFSIALNFPG	29.88
2731	P40967	PMEL17	DRB1*01:24	284	298	GPVTAQVVLQAAIPL	29.96
2732	P40967	PMEL17	DRB1*13:21	145	159	QKRSFVYVWKTWGQY	30.04
2733	P40967	PMEL17	DRB1*01:20	80	94	ANASFSIALNFPGSQ	30.12
2734	P40967	PMEL17	DRB1*01:24	155	169	TWGQYWQVLGGPVSG	30.15
2735	P40967	PMEL17	DRB1*01:11	406	420	SIVVLSGTTAAQVTT	30.24
2736	P40967	PMEL17	DRB1*11:01	147	161	RSFVYVWKTWGQYWQ	30.25
2737	P40967	PMEL17	DRB1*11:10	147	161	RSFVYVWKTWGQYWQ	30.25
2738	P40967	PMEL17	DRB1*11:12	147	161	RSFVYVWKTWGQYWQ	30.25
2739	P40967	PMEL17	DRB1*11:28	147	161	RSFVYVWKTWGQYWQ	30.25
2740	P40967	PMEL17	DRB1*11:29	147	161	RSFVYVWKTWGQYWQ	30.25
2741	P40967	PMEL17	DRB1*11:49	147	161	RSFVYVWKTWGQYWQ	30.25
2742	P40967	PMEL17	DRB1*11:62	147	161	RSFVYVWKTWGQYWQ	30.25
2743	P40967	PMEL17	DRB1*11:74	147	161	RSFVYVWKTWGQYWQ	30.25
2744	P40967	PMEL17	DRB1*13:05	147	161	RSFVYVWKTWGQYWQ	30.25

2745	P40967	PMEL17	DRB1*13:14	147	161	RSFVYVWKTWGQYWQ	30.25
2746	P40967	PMEL17	DRB1*13:50	147	161	RSFVYVWKTWGQYWQ	30.25
2747	P40967	PMEL17	DRB1*11:04	607	621	MAVVLASLIYRRRLM	30.33
2748	P40967	PMEL17	DRB1*11:46	607	621	MAVVLASLIYRRRLM	30.33
2749	P40967	PMEL17	DRB1*11:58	607	621	MAVVLASLIYRRRLM	30.33
2750	P40967	PMEL17	DRB1*13:11	607	621	MAVVLASLIYRRRLM	30.33
2751	P40967	PMEL17	DRB1*08:04	461	475	TATLRLVKRQVPLDC	30.43
2752	P40967	PMEL17	DRB1*10:01	194	208	SRSYVPLAHSSSAFT	30.43
2753	P40967	PMEL17	DRB1*01:24	17	31	ALLAVGATKVPRNQD	30.54
2754	P40967	PMEL17	DRB1*01:01	74	88	GPTLIGANASFSIAL	30.57
2755	P40967	PMEL17	DRB1*10:01	14	28	VIGALLAVGATKVPR	30.60
2756	P40967	PMEL17	DRB1*01:11	81	95	NASFSIALNFPGSQK	30.61
2757	P40967	PMEL17	DRB1*07:01	406	420	SIVVLSGTTAAQVTT	30.64
2758	P40967	PMEL17	DRB1*01:20	83	97	SFSIALNFPGSQKVL	30.68
2759	P40967	PMEL17	DRB1*01:11	230	244	KHFLRNQPLTFALQL	30.72
2760	P40967	PMEL17	DRB1*11:42	549	563	ACQLVLHQILKGGSG	30.74
2761	P40967	PMEL17	DRB1*01:18	196	210	SYVPLAHSSSAFTIT	30.77
2762	P40967	PMEL17	DRB1*13:96	98	112	PDGQVIWVNNTIING	30.82
2763	P40967	PMEL17	DRB1*01:20	158	172	QYWQVLGGPVSGLSI	30.88
2764	P40967	PMEL17	DRB1*11:02	612	626	ASLIYRRRLMKQDFS	30.90
2765	P40967	PMEL17	DRB1*11:65	612	626	ASLIYRRRLMKQDFS	30.90
2766	P40967	PMEL17	DRB1*13:01	612	626	ASLIYRRRLMKQDFS	30.90
2767	P40967	PMEL17	DRB1*01:18	7	21	RCLLHLAVIGALLAV	30.98
2768	P40967	PMEL17	DRB1*11:14	102	116	VIWVNNTIINGSQVW	31.07
2769	P40967	PMEL17	DRB1*13:02	102	116	VIWVNNTIINGSQVW	31.07
2770	P40967	PMEL17	DRB1*13:23	102	116	VIWVNNTIINGSQVW	31.07
2771	P40967	PMEL17	DRB1*13:97	102	116	VIWVNNTIINGSQVW	31.07
2772	P40967	PMEL17	DRB1*04:05	81	95	NASFSIALNFPGSQK	31.10
2773	P40967	PMEL17	DRB1*11:08	228	242	GNKHFLRNQPLTFAL	31.11
2774	P40967	PMEL17	DRB1*11:13	462	476	ATLRLVKRQVPLDCV	31.13
2775	P40967	PMEL17	DRB1*01:01	9	23	LLHLAVIGALLAVGA	31.21
2776	P40967	PMEL17	DRB1*01:20	4	18	VLKRCLLHLAVIGAL	31.22
2777	P40967	PMEL17	DRB1*01:01	167	181	VSGLSIGTGRAMLGT	31.26
2778	P40967	PMEL17	DRB1*13:96	76	90	TLIGANASFSIALNF	31.33
2779	P40967	PMEL17	DRB1*10:01	193	207	GSRSYVPLAHSSSAF	31.36
2780	P40967	PMEL17	DRB1*01:24	10	24	LHLAVIGALLAVGAT	31.38
2781	P40967	PMEL17	DRB1*04:04	81	95	NASFSIALNFPGSQK	31.52
2782	P40967	PMEL17	DRB1*04:04	82	96	ASFSIALNFPGSQKV	31.54
2783	P40967	PMEL17	DRB1*01:18	193	207	GSRSYVPLAHSSSAF	31.55
2784	P40967	PMEL17	DRB1*01:20	217	231	VSVSQLRALDGGNKH	31.55
2785	P40967	PMEL17	DRB1*01:20	168	182	SGLSIGTGRAMLGTH	31.60
2786	P40967	PMEL17	DRB1*10:01	11	25	HLAVIGALLAVGATK	31.61
2787	P40967	PMEL17	DRB1*04:05	80	94	ANASFSIALNFPGSQ	31.75

2788	P40967	PMEL17	DRB1*11:04	463	477	TLRLVKRQVPLDCVL	31.83
2789	P40967	PMEL17	DRB1*11:46	463	477	TLRLVKRQVPLDCVL	31.83
2790	P40967	PMEL17	DRB1*11:58	463	477	TLRLVKRQVPLDCVL	31.83
2791	P40967	PMEL17	DRB1*13:11	463	477	TLRLVKRQVPLDCVL	31.83
2792	P40967	PMEL17	DRB1*01:01	194	208	SRSYVPLAHSSSAFT	31.86
2793	P40967	PMEL17	DPB1*33:01	475	489	CVLYRYGSFSVTLDI	31.94
2794	P40967	PMEL17	DPB1*71:01	475	489	CVLYRYGSFSVTLDI	31.94
2795	P40967	PMEL17	DRB1*01:01	265	279	SSGTLISRALVVTHT	32.03
2796	P40967	PMEL17	DRB1*13:66	230	244	KHFLRNQPLTFALQL	32.11
2797	P40967	PMEL17	DRB1*14:06	608	622	AVVLASLIYRRRLMK	32.16
2798	P40967	PMEL17	DRB1*01:20	9	23	LLHLAVIGALLAVGA	32.18
2799	P40967	PMEL17	DRB1*13:21	144	158	SQKRSFVYVWKTWGQ	32.23
2800	P40967	PMEL17	DRB1*16:09	81	95	NASFSIALNFPGSQK	32.26
2801	P40967	PMEL17	DRB1*01:20	607	621	MAVVLASLIYRRRLM	32.58
2802	P40967	PMEL17	DRB1*11:01	462	476	ATLRLVKRQVPLDCV	32.58
2803	P40967	PMEL17	DRB1*11:10	462	476	ATLRLVKRQVPLDCV	32.58
2804	P40967	PMEL17	DRB1*11:12	462	476	ATLRLVKRQVPLDCV	32.58
2805	P40967	PMEL17	DRB1*11:28	462	476	ATLRLVKRQVPLDCV	32.58
2806	P40967	PMEL17	DRB1*11:29	462	476	ATLRLVKRQVPLDCV	32.58
2807	P40967	PMEL17	DRB1*11:49	462	476	ATLRLVKRQVPLDCV	32.58
2808	P40967	PMEL17	DRB1*11:62	462	476	ATLRLVKRQVPLDCV	32.58
2809	P40967	PMEL17	DRB1*11:74	462	476	ATLRLVKRQVPLDCV	32.58
2810	P40967	PMEL17	DRB1*13:05	462	476	ATLRLVKRQVPLDCV	32.58
2811	P40967	PMEL17	DRB1*13:14	462	476	ATLRLVKRQVPLDCV	32.58
2812	P40967	PMEL17	DRB1*13:50	462	476	ATLRLVKRQVPLDCV	32.58
2813	P40967	PMEL17	DRB1*01:20	165	179	GPVSGLSIGTGRAML	32.64
2814	P40967	PMEL17	DRB1*13:21	146	160	KRSFVYVWKTWGQYW	32.66
2815	P40967	PMEL17	DRB1*04:05	79	93	GANASFSIALNFPGS	32.67
2816	P40967	PMEL17	DRB1*01:20	606	620	LMAVVLASLIYRRRL	32.68
2817	P40967	PMEL17	DRB1*11:14	14	28	VIGALLAVGATKVPR	32.72
2818	P40967	PMEL17	DRB1*13:02	14	28	VIGALLAVGATKVPR	32.72
2819	P40967	PMEL17	DRB1*13:23	14	28	VIGALLAVGATKVPR	32.72
2820	P40967	PMEL17	DRB1*13:97	14	28	VIGALLAVGATKVPR	32.72
2821	P40967	PMEL17	DRB1*01:01	407	421	IVVLSGTTAAQVTTT	32.73
2822	P40967	PMEL17	DRB1*01:11	402	416	PAEVSIVVLSGTTAA	32.83
2823	P40967	PMEL17	DRB1*01:20	227	241	GGNKHFLRNQPLTFA	32.86
2824	P40967	PMEL17	DRB1*04:05	82	96	ASFSIALNFPGSQKV	32.99
2825	P40967	PMEL17	DRB1*07:01	266	280	SGTLISRALVVTHTY	33.04
2826	P40967	PMEL17	DRB1*01:20	5	19	LKRCLLHLAVIGALL	33.06
2827	P40967	PMEL17	DRB1*11:01	81	95	NASFSIALNFPGSQK	33.06
2828	P40967	PMEL17	DRB1*11:10	81	95	NASFSIALNFPGSQK	33.06
2829	P40967	PMEL17	DRB1*11:12	81	95	NASFSIALNFPGSQK	33.06
2830	P40967	PMEL17	DRB1*11:28	81	95	NASFSIALNFPGSQK	33.06

2831	P40967	PMEL17	DRB1*11:29	81	95	NASFSIALNFPGSQK	33.06
2832	P40967	PMEL17	DRB1*11:49	81	95	NASFSIALNFPGSQK	33.06
2833	P40967	PMEL17	DRB1*11:62	81	95	NASFSIALNFPGSQK	33.06
2834	P40967	PMEL17	DRB1*11:74	81	95	NASFSIALNFPGSQK	33.06
2835	P40967	PMEL17	DRB1*13:05	81	95	NASFSIALNFPGSQK	33.06
2836	P40967	PMEL17	DRB1*13:14	81	95	NASFSIALNFPGSQK	33.06
2837	P40967	PMEL17	DRB1*13:50	81	95	NASFSIALNFPGSQK	33.06
2838	P40967	PMEL17	DRB1*10:01	196	210	SYVPLAHSSSAFTIT	33.12
2839	P40967	PMEL17	DRB1*10:01	12	26	LAVIGALLAVGATKV	33.21
2840	P40967	PMEL17	DRB1*01:18	231	245	HFLRNQPLTFALQLH	33.26
2841	P40967	PMEL17	DRB1*01:18	267	281	GTLISRALVVTHTYL	33.36
2842	P40967	PMEL17	DRB1*16:09	82	96	ASFSIALNFPGSQKV	33.44
2843	P40967	PMEL17	DRB1*16:01	80	94	ANASFSIALNFPGSQ	33.47
2844	P40967	PMEL17	DRB1*01:01	266	280	SGTLISRALVVTHTY	33.49
2845	P40967	PMEL17	DRB1*01:02	17	31	ALLAVGATKVPRNQD	33.50
2846	P40967	PMEL17	DRB1*11:02	462	476	ATLRLVKRQVPLDCV	33.50
2847	P40967	PMEL17	DRB1*11:65	462	476	ATLRLVKRQVPLDCV	33.50
2848	P40967	PMEL17	DRB1*13:01	462	476	ATLRLVKRQVPLDCV	33.50
2849	P40967	PMEL17	DRB1*11:03	32	46	WLGVSRQLRTKAWNR	33.51
2850	P40967	PMEL17	DRB1*01:18	3	17	LVLKRCLLHLAVIGA	33.54
2851	P40967	PMEL17	DRB1*07:01	407	421	IVVLSGTTAAQVTTT	33.68
2852	P40967	PMEL17	DRB1*01:02	11	25	HLAVIGALLAVGATK	33.77
2853	P40967	PMEL17	DRB1*15:15	81	95	NASFSIALNFPGSQK	33.78
2854	P40967	PMEL17	DRB1*11:13	605	619	VLMAVVLASLIYRRR	33.79
2855	P40967	PMEL17	DRB1*01:18	74	88	GPTLIGANASFSIAL	33.82
2856	P40967	PMEL17	DRB1*01:18	264	278	DSSGTLISRALVVTH	33.84
2857	P40967	PMEL17	DRB1*01:29	284	298	GPVTAQVVLQAAIPL	33.99
2858	P40967	PMEL17	DPB1*33:01	474	488	DCVLYRYGSFSVTLD	34.01
2859	P40967	PMEL17	DPB1*71:01	474	488	DCVLYRYGSFSVTLD	34.01
2860	P40967	PMEL17	DRB1*11:02	184	198	MEVTVYHRRGSRSYV	34.06
2861	P40967	PMEL17	DRB1*11:65	184	198	MEVTVYHRRGSRSYV	34.06
2862	P40967	PMEL17	DRB1*13:01	184	198	MEVTVYHRRGSRSYV	34.06
2863	P40967	PMEL17	DRB1*11:42	605	619	VLMAVVLASLIYRRR	34.16
2864	P40967	PMEL17	DRB1*01:18	167	181	VSGLSIGTGRAMLGT	34.18
2865	P40967	PMEL17	DRB1*11:14	15	29	IGALLAVGATKVPRN	34.24
2866	P40967	PMEL17	DRB1*13:02	15	29	IGALLAVGATKVPRN	34.24
2867	P40967	PMEL17	DRB1*13:23	15	29	IGALLAVGATKVPRN	34.24
2868	P40967	PMEL17	DRB1*13:97	15	29	IGALLAVGATKVPRN	34.24
2869	P40967	PMEL17	DRB1*01:18	290	304	VVLQAAIPLTSCGSS	34.52
2870	P40967	PMEL17	DRB1*04:04	406	420	SIVVLSGTTAAQVTT	34.56
2871	P40967	PMEL17	DRB1*04:04	80	94	ANASFSIALNFPGSQ	34.62
2872	P40967	PMEL17	DRB1*13:21	462	476	ATLRLVKRQVPLDCV	34.66
2873	P40967	PMEL17	DRB1*14:06	607	621	MAVVLASLIYRRRLM	34.66

2874	P40967	PMEL17	DRB1*01:11	82	96	ASFSIALNFPGSQKV	34.74
2875	P40967	PMEL17	DRB1*11:14	82	96	ASFSIALNFPGSQKV	34.80
2876	P40967	PMEL17	DRB1*13:02	82	96	ASFSIALNFPGSQKV	34.80
2877	P40967	PMEL17	DRB1*13:23	82	96	ASFSIALNFPGSQKV	34.80
2878	P40967	PMEL17	DRB1*13:97	82	96	ASFSIALNFPGSQKV	34.80
2879	P40967	PMEL17	DRB1*11:42	30	44	QDWLGVSRQLRTKAW	34.81
2880	P40967	PMEL17	DRB1*11:03	606	620	LMAVVLASLIYRRRL	34.91
2881	P40967	PMEL17	DRB1*14:32	286	300	VTAQVVLQAAIPLTS	34.94
2882	P40967	PMEL17	DRB1*01:01	193	207	GSRSYVPLAHSSSAF	34.96
2883	P40967	PMEL17	DRB1*01:01	5	19	LKRCLLHLAVIGALL	35.17
2884	P40967	PMEL17	DRB1*03:11	482	496	SFSVTLDIVQGIESA	35.19
2885	P40967	PMEL17	DRB1*01:01	4	18	VLKRCLLHLAVIGAL	35.25
2886	P40967	PMEL17	DRB1*04:05	478	492	YRYGSFSVTLDIVQG	35.35
2887	P40967	PMEL17	DRB1*11:04	32	46	WLGVSRQLRTKAWNR	35.36
2888	P40967	PMEL17	DRB1*11:46	32	46	WLGVSRQLRTKAWNR	35.36
2889	P40967	PMEL17	DRB1*11:58	32	46	WLGVSRQLRTKAWNR	35.36
2890	P40967	PMEL17	DRB1*11:84	609	623	VVLASLIYRRRLMKQ	35.36
2891	P40967	PMEL17	DRB1*13:11	32	46	WLGVSRQLRTKAWNR	35.36
2892	P40967	PMEL17	DRB1*15:01	286	300	VTAQVVLQAAIPLTS	35.40
2893	P40967	PMEL17	DRB1*15:06	286	300	VTAQVVLQAAIPLTS	35.40
2894	P40967	PMEL17	DRB1*10:01	230	244	KHFLRNQPLTFALQL	35.42
2895	P40967	PMEL17	DRB1*11:13	458	472	LDGTATLRLVKRQVP	35.45
2896	P40967	PMEL17	DRB1*01:29	10	24	LHLAVIGALLAVGAT	35.46
2897	P40967	PMEL17	DRB1*13:66	227	241	GGNKHFLRNQPLTFA	35.47
2898	P40967	PMEL17	DRB1*14:32	460	474	GTATLRLVKRQVPLD	35.48
2899	P40967	PMEL17	DRB1*01:20	605	619	VLMAVVLASLIYRRR	35.51
2900	P40967	PMEL17	DRB1*16:02	79	93	GANASFSIALNFPGS	35.51
2901	P40967	PMEL17	DRB1*11:14	284	298	GPVTAQVVLQAAIPL	35.55
2902	P40967	PMEL17	DRB1*13:02	284	298	GPVTAQVVLQAAIPL	35.55
2903	P40967	PMEL17	DRB1*13:23	284	298	GPVTAQVVLQAAIPL	35.55
2904	P40967	PMEL17	DRB1*13:97	284	298	GPVTAQVVLQAAIPL	35.55
2905	P40967	PMEL17	DRB1*14:32	459	473	DGTATLRLVKRQVPL	35.57
2906	P40967	PMEL17	DRB1*01:18	226	240	DGGNKHFLRNQPLTF	35.59
2907	P40967	PMEL17	DRB1*09:01	196	210	SYVPLAHSSSAFTIT	35.62
2908	P40967	PMEL17	DRB1*01:24	79	93	GANASFSIALNFPGS	35.66
2909	P40967	PMEL17	DRB1*11:03	458	472	LDGTATLRLVKRQVP	35.67
2910	P40967	PMEL17	DRB1*10:01	229	243	NKHFLRNQPLTFALQ	35.72
2911	P40967	PMEL17	DRB1*11:84	461	475	TATLRLVKRQVPLDC	35.74
2912	P40967	PMEL17	DRB1*15:15	82	96	ASFSIALNFPGSQKV	35.75
2913	P40967	PMEL17	DRB1*04:05	475	489	CVLYRYGSFSVTLDI	35.84
2914	P40967	PMEL17	DRB1*07:01	404	418	EVSIVVLSGTTAAQV	35.86
2915	P40967	PMEL17	DRB1*11:04	612	626	ASLIYRRRLMKQDFS	36.01
2916	P40967	PMEL17	DRB1*11:46	612	626	ASLIYRRRLMKQDFS	36.01

2917	P40967	PMEL17	DRB1*11:58	612	626	ASLIYRRRLMKQDFS	36.01
2918	P40967	PMEL17	DRB1*13:11	612	626	ASLIYRRRLMKQDFS	36.01
2919	P40967	PMEL17	DRB1*10:01	290	304	VVLQAAIPLTSCGSS	36.16
2920	P40967	PMEL17	DPB1*33:01	473	487	LDCVLYRYGSFSVTL	36.17
2921	P40967	PMEL17	DPB1*71:01	473	487	LDCVLYRYGSFSVTL	36.17
2922	P40967	PMEL17	DRB1*01:01	264	278	DSSGTLISRALVVTH	36.19
2923	P40967	PMEL17	DRB1*01:18	78	92	IGANASFSIALNFPG	36.37
2924	P40967	PMEL17	DRB1*14:02	227	241	GGNKHFLRNQPLTFA	36.43
2925	P40967	PMEL17	DRB1*01:20	608	622	AVVLASLIYRRRLMK	36.60
2926	P40967	PMEL17	DRB1*11:04	548	562	PACQLVLHQILKGGS	36.62
2927	P40967	PMEL17	DRB1*11:46	548	562	PACQLVLHQILKGGS	36.62
2928	P40967	PMEL17	DRB1*11:58	548	562	PACQLVLHQILKGGS	36.62
2929	P40967	PMEL17	DRB1*13:11	548	562	PACQLVLHQILKGGS	36.62
2930	P40967	PMEL17	DRB1*11:03	30	44	QDWLGVSRQLRTKAW	36.72
2931	P40967	PMEL17	DRB1*01:24	83	97	SFSIALNFPGSQKVL	36.75
2932	P40967	PMEL17	DRB1*11:01	551	565	QLVLHQILKGGSGTY	36.77
2933	P40967	PMEL17	DRB1*11:10	551	565	QLVLHQILKGGSGTY	36.77
2934	P40967	PMEL17	DRB1*11:12	551	565	QLVLHQILKGGSGTY	36.77
2935	P40967	PMEL17	DRB1*11:28	551	565	QLVLHQILKGGSGTY	36.77
2936	P40967	PMEL17	DRB1*11:29	551	565	QLVLHQILKGGSGTY	36.77
2937	P40967	PMEL17	DRB1*11:49	551	565	QLVLHQILKGGSGTY	36.77
2938	P40967	PMEL17	DRB1*11:62	551	565	QLVLHQILKGGSGTY	36.77
2939	P40967	PMEL17	DRB1*11:74	551	565	QLVLHQILKGGSGTY	36.77
2940	P40967	PMEL17	DRB1*13:05	551	565	QLVLHQILKGGSGTY	36.77
2941	P40967	PMEL17	DRB1*13:14	551	565	QLVLHQILKGGSGTY	36.77
2942	P40967	PMEL17	DRB1*13:50	551	565	QLVLHQILKGGSGTY	36.77
2943	P40967	PMEL17	DRB1*11:14	77	91	LIGANASFSIALNFP	36.78
2944	P40967	PMEL17	DRB1*13:02	77	91	LIGANASFSIALNFP	36.78
2945	P40967	PMEL17	DRB1*13:23	77	91	LIGANASFSIALNFP	36.78
2946	P40967	PMEL17	DRB1*13:97	77	91	LIGANASFSIALNFP	36.78
2947	P40967	PMEL17	DRB1*01:01	290	304	VVLQAAIPLTSCGSS	36.79
2948	P40967	PMEL17	DRB1*01:01	166	180	PVSGLSIGTGRAMLG	36.90
2949	P40967	PMEL17	DRB1*11:08	230	244	KHFLRNQPLTFALQL	37.00
2950	P40967	PMEL17	DRB1*11:04	146	160	KRSFVYVWKTWGQYW	37.07
2951	P40967	PMEL17	DRB1*11:46	146	160	KRSFVYVWKTWGQYW	37.07
2952	P40967	PMEL17	DRB1*11:58	146	160	KRSFVYVWKTWGQYW	37.07
2953	P40967	PMEL17	DRB1*13:11	146	160	KRSFVYVWKTWGQYW	37.07
2954	P40967	PMEL17	DRB1*01:29	229	243	NKHFLRNQPLTFALQ	37.10
2955	P40967	PMEL17	DRB1*01:11	11	25	HLAVIGALLAVGATK	37.11
2956	P40967	PMEL17	DRB1*01:20	73	87	DGPTLIGANASFSIA	37.12
2957	P40967	PMEL17	DRB1*11:04	145	159	QKRSFVYVWKTWGQY	37.12
2958	P40967	PMEL17	DRB1*11:46	145	159	QKRSFVYVWKTWGQY	37.12
2959	P40967	PMEL17	DRB1*11:58	145	159	QKRSFVYVWKTWGQY	37.12

2960	P40967	PMEL17	DRB1*13:11	145	159	QKRSFVYVWKTWGQY	37.12
2961	P40967	PMEL17	DRB1*01:11	284	298	GPVTAQVVLQAAIPL	37.23
2962	P40967	PMEL17	DRB1*11:42	32	46	WLGVSRQLRTKAWNR	37.33
2963	P40967	PMEL17	DRB1*07:01	455	469	GPLLDGTATLRLVKR	37.34
2964	P40967	PMEL17	DRB1*01:20	196	210	SYVPLAHSSSAFTIT	37.37
2965	P40967	PMEL17	DRB1*01:01	75	89	PTLIGANASFSIALN	37.54
2966	P40967	PMEL17	DRB1*11:14	84	98	FSIALNFPGSQKVLP	37.57
2967	P40967	PMEL17	DRB1*13:02	84	98	FSIALNFPGSQKVLP	37.57
2968	P40967	PMEL17	DRB1*13:23	84	98	FSIALNFPGSQKVLP	37.57
2969	P40967	PMEL17	DRB1*13:97	84	98	FSIALNFPGSQKVLP	37.57
2970	P40967	PMEL17	DRB1*10:01	285	299	PVTAQVVLQAAIPLT	37.70
2971	P40967	PMEL17	DRB1*11:03	551	565	QLVLHQILKGGSGTY	37.72
2972	P40967	PMEL17	DRB1*11:02	605	619	VLMAVVLASLIYRRR	37.73
2973	P40967	PMEL17	DRB1*11:65	605	619	VLMAVVLASLIYRRR	37.73
2974	P40967	PMEL17	DRB1*13:01	605	619	VLMAVVLASLIYRRR	37.73
2975	P40967	PMEL17	DRB1*01:01	197	211	YVPLAHSSSAFTITD	37.85
2976	P40967	PMEL17	DRB1*01:29	6	20	KRCLLHLAVIGALLA	37.86
2977	P40967	PMEL17	DRB1*11:04	606	620	LMAVVLASLIYRRRL	37.88
2978	P40967	PMEL17	DRB1*11:46	606	620	LMAVVLASLIYRRRL	37.88
2979	P40967	PMEL17	DRB1*11:58	606	620	LMAVVLASLIYRRRL	37.88
2980	P40967	PMEL17	DRB1*13:11	606	620	LMAVVLASLIYRRRL	37.88
2981	P40967	PMEL17	DRB1*01:01	267	281	GTLISRALVVTHTYL	37.90
2982	P40967	PMEL17	DRB1*01:18	606	620	LMAVVLASLIYRRRL	37.92
2983	P40967	PMEL17	DRB1*08:04	551	565	QLVLHQILKGGSGTY	37.93
2984	P40967	PMEL17	DRB1*01:18	605	619	VLMAVVLASLIYRRR	38.07
2985	P40967	PMEL17	DRB1*01:20	215	229	FSVSVSQLRALDGGN	38.10
2986	P40967	PMEL17	DRB1*07:01	196	210	SYVPLAHSSSAFTIT	38.10
2987	P40967	PMEL17	DRB1*01:29	79	93	GANASFSIALNFPGS	38.11
2988	P40967	PMEL17	DRB1*14:32	285	299	PVTAQVVLQAAIPLT	38.11
2989	P40967	PMEL17	DRB1*01:11	158	172	QYWQVLGGPVSGLSI	38.16
2990	P40967	PMEL17	DRB1*07:01	175	189	GRAMLGTHTMEVTVY	38.21
2991	P40967	PMEL17	DRB1*11:13	286	300	VTAQVVLQAAIPLTS	38.25
2992	P40967	PMEL17	DRB1*14:06	609	623	VVLASLIYRRRLMKQ	38.33
2993	P40967	PMEL17	DRB1*11:03	181	195	THTMEVTVYHRRGSR	38.46
2994	P40967	PMEL17	DRB1*11:19	229	243	NKHFLRNQPLTFALQ	38.52
2995	P40967	PMEL17	DRB1*11:03	31	45	DWLGVSRQLRTKAWN	38.55
2996	P40967	PMEL17	DRB1*08:04	462	476	ATLRLVKRQVPLDCV	38.56
2997	P40967	PMEL17	DRB1*11:84	608	622	AVVLASLIYRRRLMK	38.65
2998	P40967	PMEL17	DRB1*01:20	155	169	TWGQYWQVLGGPVSG	38.70
2999	P40967	PMEL17	DRB1*07:01	228	242	GNKHFLRNQPLTFAL	38.73
3000	P40967	PMEL17	DRB1*11:06	459	473	DGTATLRLVKRQVPL	38.75
3001	P40967	PMEL17	DRB1*01:20	263	277	GDSSGTLISRALVVT	38.76
3002	P40967	PMEL17	DRB1*13:21	609	623	VVLASLIYRRRLMKQ	38.83

3003	P40967	PMEL17	DRB1*14:06	606	620	LMAVVLASLIYRRRL	38.83
3004	P40967	PMEL17	DRB1*01:24	5	19	LKRCLLHLAVIGALL	38.93
3005	P40967	PMEL17	DRB1*01:29	228	242	GNKHFLRNQPLTFAL	38.99
3006	P40967	PMEL17	DRB1*11:42	31	45	DWLGVSRQLRTKAWN	39.03
3007	P40967	PMEL17	DRB1*10:01	404	418	EVSIVVLSGTTAAQV	39.05
3008	P40967	PMEL17	DRB1*01:20	3	17	LVLKRCLLHLAVIGA	39.10
3009	P40967	PMEL17	DRB1*10:01	476	490	VLYRYGSFSVTLDIV	39.11
3010	P40967	PMEL17	DRB1*11:37	146	160	KRSFVYVWKTWGQYW	39.23
3011	P40967	PMEL17	DRB1*13:07	146	160	KRSFVYVWKTWGQYW	39.23
3012	P40967	PMEL17	DRB1*13:21	458	472	LDGTATLRLVKRQVP	39.45
3013	P40967	PMEL17	DRB1*11:01	194	208	SRSYVPLAHSSSAFT	39.52
3014	P40967	PMEL17	DRB1*11:10	194	208	SRSYVPLAHSSSAFT	39.52
3015	P40967	PMEL17	DRB1*11:12	194	208	SRSYVPLAHSSSAFT	39.52
3016	P40967	PMEL17	DRB1*11:28	194	208	SRSYVPLAHSSSAFT	39.52
3017	P40967	PMEL17	DRB1*11:29	194	208	SRSYVPLAHSSSAFT	39.52
3018	P40967	PMEL17	DRB1*11:49	194	208	SRSYVPLAHSSSAFT	39.52
3019	P40967	PMEL17	DRB1*11:62	194	208	SRSYVPLAHSSSAFT	39.52
3020	P40967	PMEL17	DRB1*11:74	194	208	SRSYVPLAHSSSAFT	39.52
3021	P40967	PMEL17	DRB1*13:05	194	208	SRSYVPLAHSSSAFT	39.52
3022	P40967	PMEL17	DRB1*13:14	194	208	SRSYVPLAHSSSAFT	39.52
3023	P40967	PMEL17	DRB1*13:50	194	208	SRSYVPLAHSSSAFT	39.52
3024	P40967	PMEL17	DRB1*10:01	477	491	LYRYGSFSVTLDIVQ	39.58
3025	P40967	PMEL17	DRB1*11:08	146	160	KRSFVYVWKTWGQYW	39.62
3026	P40967	PMEL17	DRB1*11:02	286	300	VTAQVVLQAAIPLTS	39.64
3027	P40967	PMEL17	DRB1*11:65	286	300	VTAQVVLQAAIPLTS	39.64
3028	P40967	PMEL17	DRB1*13:01	286	300	VTAQVVLQAAIPLTS	39.64
3029	P40967	PMEL17	DRB1*11:19	228	242	GNKHFLRNQPLTFAL	39.67
3030	P40967	PMEL17	DRB1*09:01	197	211	YVPLAHSSSAFTITD	39.68
3031	P40967	PMEL17	DRB1*11:01	193	207	GSRSYVPLAHSSSAF	39.70
3032	P40967	PMEL17	DRB1*11:10	193	207	GSRSYVPLAHSSSAF	39.70
3033	P40967	PMEL17	DRB1*11:12	193	207	GSRSYVPLAHSSSAF	39.70
3034	P40967	PMEL17	DRB1*11:28	193	207	GSRSYVPLAHSSSAF	39.70
3035	P40967	PMEL17	DRB1*11:29	193	207	GSRSYVPLAHSSSAF	39.70
3036	P40967	PMEL17	DRB1*11:49	193	207	GSRSYVPLAHSSSAF	39.70
3037	P40967	PMEL17	DRB1*11:62	193	207	GSRSYVPLAHSSSAF	39.70
3038	P40967	PMEL17	DRB1*11:74	193	207	GSRSYVPLAHSSSAF	39.70
3039	P40967	PMEL17	DRB1*13:05	193	207	GSRSYVPLAHSSSAF	39.70
3040	P40967	PMEL17	DRB1*13:14	193	207	GSRSYVPLAHSSSAF	39.70
3041	P40967	PMEL17	DRB1*13:50	193	207	GSRSYVPLAHSSSAF	39.70
3042	P40967	PMEL17	DRB1*01:29	154	168	KTWGQYWQVLGGPVS	39.78
3043	P40967	PMEL17	DRB1*01:01	78	92	IGANASFSIALNFPG	39.80
3044	P40967	PMEL17	DRB1*13:96	286	300	VTAQVVLQAAIPLTS	39.80
3045	P40967	PMEL17	DRB1*01:01	231	245	HFLRNQPLTFALQLH	39.84

3046	P40967	PMEL17	DRB1*11:37	145	159	QKRSFVYVWKTWGQY	39.89
3047	P40967	PMEL17	DRB1*13:07	145	159	QKRSFVYVWKTWGQY	39.89
3048	P40967	PMEL17	DRB1*01:18	75	89	PTLIGANASFSIALN	39.94
3049	P40967	PMEL17	DRB1*01:02	406	420	SIVVLSGTTAAQVTT	40.28
3050	P40967	PMEL17	DRB1*07:01	419	433	TTTEWVETTARELPI	40.29
3051	P40967	PMEL17	DRB1*10:01	154	168	KTWGQYWQVLGGPVS	40.32
3052	P40967	PMEL17	DRB1*16:09	80	94	ANASFSIALNFPGSQ	40.34
3053	P40967	PMEL17	DRB1*11:13	610	624	VLASLIYRRRLMKQD	40.36
3054	P40967	PMEL17	DRB1*04:04	79	93	GANASFSIALNFPGS	40.38
3055	P40967	PMEL17	DRB1*01:18	166	180	PVSGLSIGTGRAMLG	40.44
3056	P40967	PMEL17	DRB1*10:01	405	419	VSIVVLSGTTAAQVT	40.57
3057	P40967	PMEL17	DRB1*07:01	156	170	WGQYWQVLGGPVSGL	40.66
3058	P40967	PMEL17	DRB1*11:08	460	474	GTATLRLVKRQVPLD	40.71
3059	P40967	PMEL17	DRB1*11:08	459	473	DGTATLRLVKRQVPL	40.82
3060	P40967	PMEL17	DRB1*15:15	80	94	ANASFSIALNFPGSQ	40.83
3061	P40967	PMEL17	DRB1*11:03	33	47	LGVSRQLRTKAWNRQ	40.87
3062	P40967	PMEL17	DRB1*10:01	74	88	GPTLIGANASFSIAL	40.93
3063	P40967	PMEL17	DRB1*16:01	79	93	GANASFSIALNFPGS	40.98
3064	P40967	PMEL17	DRB1*13:21	143	157	WSQKRSFVYVWKTWG	41.02
3065	P40967	PMEL17	DRB1*09:01	195	209	RSYVPLAHSSSAFTI	41.07
3066	P40967	PMEL17	DRB1*07:01	229	243	NKHFLRNQPLTFALQ	41.14
3067	P40967	PMEL17	DRB1*10:01	228	242	GNKHFLRNQPLTFAL	41.14
3068	P40967	PMEL17	DRB1*11:01	143	157	WSQKRSFVYVWKTWG	41.25
3069	P40967	PMEL17	DRB1*11:10	143	157	WSQKRSFVYVWKTWG	41.25
3070	P40967	PMEL17	DRB1*11:12	143	157	WSQKRSFVYVWKTWG	41.25
3071	P40967	PMEL17	DRB1*11:28	143	157	WSQKRSFVYVWKTWG	41.25
3072	P40967	PMEL17	DRB1*11:29	143	157	WSQKRSFVYVWKTWG	41.25
3073	P40967	PMEL17	DRB1*11:49	143	157	WSQKRSFVYVWKTWG	41.25
3074	P40967	PMEL17	DRB1*11:62	143	157	WSQKRSFVYVWKTWG	41.25
3075	P40967	PMEL17	DRB1*11:74	143	157	WSQKRSFVYVWKTWG	41.25
3076	P40967	PMEL17	DRB1*13:05	143	157	WSQKRSFVYVWKTWG	41.25
3077	P40967	PMEL17	DRB1*13:14	143	157	WSQKRSFVYVWKTWG	41.25
3078	P40967	PMEL17	DRB1*13:50	143	157	WSQKRSFVYVWKTWG	41.25
3079	P40967	PMEL17	DRB1*01:24	9	23	LLHLAVIGALLAVGA	41.31
3080	P40967	PMEL17	DRB1*13:21	608	622	AVVLASLIYRRRLMK	41.50
3081	P40967	PMEL17	DRB1*13:96	73	87	DGPTLIGANASFSIA	41.50
3082	P40967	PMEL17	DRB1*10:01	267	281	GTLISRALVVTHTYL	41.51
3083	P40967	PMEL17	DRB1*11:08	145	159	QKRSFVYVWKTWGQY	41.63
3084	P40967	PMEL17	DRB1*01:11	17	31	ALLAVGATKVPRNQD	41.69
3085	P40967	PMEL17	DRB1*13:96	102	116	VIWVNNTIINGSQVW	41.76
3086	P40967	PMEL17	DRB1*11:03	463	477	TLRLVKRQVPLDCVL	41.82
3087	P40967	PMEL17	DRB1*11:02	229	243	NKHFLRNQPLTFALQ	41.83
3088	P40967	PMEL17	DRB1*11:65	229	243	NKHFLRNQPLTFALQ	41.83

3089	P40967	PMEL17	DRB1*13:01	229	243	NKHFLRNQPLTFALQ	41.83
3090	P40967	PMEL17	DRB1*14:06	459	473	DGTATLRLVKRQVPL	41.99
3091	P40967	PMEL17	DRB1*10:01	192	206	RGSRSYVPLAHSSSA	42.04
3092	P40967	PMEL17	DRB1*01:20	197	211	YVPLAHSSSAFTITD	42.06
3093	P40967	PMEL17	DRB1*14:32	461	475	TATLRLVKRQVPLDC	42.07
3094	P40967	PMEL17	DRB1*01:20	268	282	TLISRALVVTHTYLE	42.10
3095	P40967	PMEL17	DRB1*11:01	82	96	ASFSIALNFPGSQKV	42.13
3096	P40967	PMEL17	DRB1*11:10	82	96	ASFSIALNFPGSQKV	42.13
3097	P40967	PMEL17	DRB1*11:12	82	96	ASFSIALNFPGSQKV	42.13
3098	P40967	PMEL17	DRB1*11:28	82	96	ASFSIALNFPGSQKV	42.13
3099	P40967	PMEL17	DRB1*11:29	82	96	ASFSIALNFPGSQKV	42.13
3100	P40967	PMEL17	DRB1*11:49	82	96	ASFSIALNFPGSQKV	42.13
3101	P40967	PMEL17	DRB1*11:62	82	96	ASFSIALNFPGSQKV	42.13
3102	P40967	PMEL17	DRB1*11:74	82	96	ASFSIALNFPGSQKV	42.13
3103	P40967	PMEL17	DRB1*13:05	82	96	ASFSIALNFPGSQKV	42.13
3104	P40967	PMEL17	DRB1*13:14	82	96	ASFSIALNFPGSQKV	42.13
3105	P40967	PMEL17	DRB1*13:50	82	96	ASFSIALNFPGSQKV	42.13
3106	P40967	PMEL17	DRB1*01:11	155	169	TWGQYWQVLGGPVSG	42.16
3107	P40967	PMEL17	DRB1*03:11	481	495	GSFSVTLDIVQGIES	42.16
3108	P40967	PMEL17	DRB1*01:20	79	93	GANASFSIALNFPGS	42.19
3109	P40967	PMEL17	DRB1*15:01	471	485	VPLDCVLYRYGSFSV	42.20
3110	P40967	PMEL17	DRB1*15:06	471	485	VPLDCVLYRYGSFSV	42.20
3111	P40967	PMEL17	DRB1*11:02	183	197	TMEVTVYHRRGSRSY	42.25
3112	P40967	PMEL17	DRB1*11:65	183	197	TMEVTVYHRRGSRSY	42.25
3113	P40967	PMEL17	DRB1*13:01	183	197	TMEVTVYHRRGSRSY	42.25
3114	P40967	PMEL17	DRB1*07:01	195	209	RSYVPLAHSSSAFTI	42.27
3115	P40967	PMEL17	DRB1*01:11	80	94	ANASFSIALNFPGSQ	42.31
3116	P40967	PMEL17	DRB1*13:96	97	111	LPDGQVIWVNNTIIN	42.31
3117	P40967	PMEL17	DRB1*11:01	195	209	RSYVPLAHSSSAFTI	42.32
3118	P40967	PMEL17	DRB1*11:10	195	209	RSYVPLAHSSSAFTI	42.32
3119	P40967	PMEL17	DRB1*11:12	195	209	RSYVPLAHSSSAFTI	42.32
3120	P40967	PMEL17	DRB1*11:28	195	209	RSYVPLAHSSSAFTI	42.32
3121	P40967	PMEL17	DRB1*11:29	195	209	RSYVPLAHSSSAFTI	42.32
3122	P40967	PMEL17	DRB1*11:49	195	209	RSYVPLAHSSSAFTI	42.32
3123	P40967	PMEL17	DRB1*11:62	195	209	RSYVPLAHSSSAFTI	42.32
3124	P40967	PMEL17	DRB1*11:74	195	209	RSYVPLAHSSSAFTI	42.32
3125	P40967	PMEL17	DRB1*13:05	195	209	RSYVPLAHSSSAFTI	42.32
3126	P40967	PMEL17	DRB1*13:14	195	209	RSYVPLAHSSSAFTI	42.32
3127	P40967	PMEL17	DRB1*13:50	195	209	RSYVPLAHSSSAFTI	42.32
3128	P40967	PMEL17	DRB1*07:01	154	168	KTWGQYWQVLGGPVS	42.41
3129	P40967	PMEL17	DRB1*11:42	29	43	NQDWLGVSRQLRTKA	42.49
3130	P40967	PMEL17	DRB1*11:08	227	241	GGNKHFLRNQPLTFA	42.70
3131	P40967	PMEL17	DRB1*01:20	218	232	SVSQLRALDGGNKHF	42.72

3132	P40967	PMEL17	DRB1*13:96	83	97	SFSIALNFPGSQKVL	42.80
3133	P40967	PMEL17	DRB1*14:32	229	243	NKHFLRNQPLTFALQ	42.81
3134	P40967	PMEL17	DRB1*01:24	228	242	GNKHFLRNQPLTFAL	42.88
3135	P40967	PMEL17	DRB1*11:08	81	95	NASFSIALNFPGSQK	42.89
3136	P40967	PMEL17	DRB1*09:01	79	93	GANASFSIALNFPGS	42.92
3137	P40967	PMEL17	DRB1*11:42	552	566	LVLHQILKGGSGTYC	42.96
3138	P40967	PMEL17	DRB1*01:11	227	241	GGNKHFLRNQPLTFA	42.97
3139	P40967	PMEL17	DRB1*09:01	81	95	NASFSIALNFPGSQK	42.97
3140	P40967	PMEL17	DRB1*07:01	454	468	LGPLLDGTATLRLVK	43.03
3141	P40967	PMEL17	DRB1*01:24	229	243	NKHFLRNQPLTFALQ	43.04
3142	P40967	PMEL17	DRB1*08:04	550	564	CQLVLHQILKGGSGT	43.09
3143	P40967	PMEL17	DRB1*07:01	17	31	ALLAVGATKVPRNQD	43.16
3144	P40967	PMEL17	DRB1*11:14	405	419	VSIVVLSGTTAAQVT	43.21
3145	P40967	PMEL17	DRB1*13:02	405	419	VSIVVLSGTTAAQVT	43.21
3146	P40967	PMEL17	DRB1*13:23	405	419	VSIVVLSGTTAAQVT	43.21
3147	P40967	PMEL17	DRB1*13:97	405	419	VSIVVLSGTTAAQVT	43.21
3148	P40967	PMEL17	DRB1*10:01	15	29	IGALLAVGATKVPRN	43.24
3149	P40967	PMEL17	DRB1*04:04	403	417	AEVSIVVLSGTTAAQ	43.28
3150	P40967	PMEL17	DRB1*11:03	550	564	CQLVLHQILKGGSGT	43.28
3151	P40967	PMEL17	DRB1*11:14	288	302	AQVVLQAAIPLTSCG	43.39
3152	P40967	PMEL17	DRB1*13:02	288	302	AQVVLQAAIPLTSCG	43.39
3153	P40967	PMEL17	DRB1*13:23	288	302	AQVVLQAAIPLTSCG	43.39
3154	P40967	PMEL17	DRB1*13:97	288	302	AQVVLQAAIPLTSCG	43.39
3155	P40967	PMEL17	DRB1*01:29	83	97	SFSIALNFPGSQKVL	43.56
3156	P40967	PMEL17	DRB1*07:01	176	190	RAMLGTHTMEVTVYH	43.56
3157	P40967	PMEL17	DRB1*01:01	226	240	DGGNKHFLRNQPLTF	43.60
3158	P40967	PMEL17	DRB1*04:08	78	92	IGANASFSIALNFPG	43.75
3159	P40967	PMEL17	DRB1*04:01	78	92	IGANASFSIALNFPG	43.78
3160	P40967	PMEL17	DRB1*11:04	30	44	QDWLGVSRQLRTKAW	43.91
3161	P40967	PMEL17	DRB1*11:42	548	562	PACQLVLHQILKGGS	43.91
3162	P40967	PMEL17	DRB1*11:46	30	44	QDWLGVSRQLRTKAW	43.91
3163	P40967	PMEL17	DRB1*11:58	30	44	QDWLGVSRQLRTKAW	43.91
3164	P40967	PMEL17	DRB1*13:11	30	44	QDWLGVSRQLRTKAW	43.91
3165	P40967	PMEL17	DRB1*01:20	195	209	RSYVPLAHSSSAFTI	44.03
3166	P40967	PMEL17	DRB1*11:06	460	474	GTATLRLVKRQVPLD	44.04
3167	P40967	PMEL17	DRB1*10:01	406	420	SIVVLSGTTAAQVTT	44.05
3168	P40967	PMEL17	DRB1*01:01	168	182	SGLSIGTGRAMLGTH	44.21
3169	P40967	PMEL17	DRB1*01:20	214	228	PFSVSVSQLRALDGG	44.28
3170	P40967	PMEL17	DRB1*14:06	460	474	GTATLRLVKRQVPLD	44.30
3171	P40967	PMEL17	DRB1*09:01	80	94	ANASFSIALNFPGSQ	44.33
3172	P40967	PMEL17	DRB1*11:14	72	86	NDGPTLIGANASFSI	44.44
3173	P40967	PMEL17	DRB1*13:02	72	86	NDGPTLIGANASFSI	44.44
3174	P40967	PMEL17	DRB1*13:23	72	86	NDGPTLIGANASFSI	44.44

3175	P40967	PMEL17	DRB1*13:97	72	86	NDGPTLIGANASFSI	44.44
3176	P40967	PMEL17	DRB1*14:07	228	242	GNKHFLRNQPLTFAL	44.47
3177	P40967	PMEL17	DRB1*11:04	31	45	DWLGVSRQLRTKAWN	44.48
3178	P40967	PMEL17	DRB1*11:42	612	626	ASLIYRRRLMKQDFS	44.48
3179	P40967	PMEL17	DRB1*11:46	31	45	DWLGVSRQLRTKAWN	44.48
3180	P40967	PMEL17	DRB1*11:58	31	45	DWLGVSRQLRTKAWN	44.48
3181	P40967	PMEL17	DRB1*13:11	31	45	DWLGVSRQLRTKAWN	44.48
3182	P40967	PMEL17	DRB1*11:02	613	627	SLIYRRRLMKQDFSV	44.49
3183	P40967	PMEL17	DRB1*11:65	613	627	SLIYRRRLMKQDFSV	44.49
3184	P40967	PMEL17	DRB1*13:01	613	627	SLIYRRRLMKQDFSV	44.49
3185	P40967	PMEL17	DRB1*14:32	228	242	GNKHFLRNQPLTFAL	44.49
3186	P40967	PMEL17	DRB1*11:42	33	47	LGVSRQLRTKAWNRQ	44.51
3187	P40967	PMEL17	DRB1*12:03	607	621	MAVVLASLIYRRRLM	44.58
3188	P40967	PMEL17	DRB1*01:02	404	418	EVSIVVLSGTTAAQV	44.64
3189	P40967	PMEL17	DRB1*14:12	608	622	AVVLASLIYRRRLMK	44.80
3190	P40967	PMEL17	DRB1*01:18	8	22	CLLHLAVIGALLAVG	44.89
3191	P40967	PMEL17	DRB1*11:13	229	243	NKHFLRNQPLTFALQ	44.97
3192	P40967	PMEL17	DRB1*01:18	211	225	DQVPFSVSVSQLRAL	45.05
3193	P40967	PMEL17	DRB1*15:01	285	299	PVTAQVVLQAAIPLT	45.07
3194	P40967	PMEL17	DRB1*15:06	285	299	PVTAQVVLQAAIPLT	45.07
3195	P40967	PMEL17	DRB1*01:18	197	211	YVPLAHSSSAFTITD	45.10
3196	P40967	PMEL17	DRB1*04:72	81	95	NASFSIALNFPGSQK	45.10
3197	P40967	PMEL17	DRB1*14:06	605	619	VLMAVVLASLIYRRR	45.12
3198	P40967	PMEL17	DRB1*01:24	230	244	KHFLRNQPLTFALQL	45.15
3199	P40967	PMEL17	DRB1*13:61	608	622	AVVLASLIYRRRLMK	45.15
3200	P40967	PMEL17	DRB1*01:02	405	419	VSIVVLSGTTAAQVT	45.22
3201	P40967	PMEL17	DRB1*01:29	230	244	KHFLRNQPLTFALQL	45.23
3202	P40967	PMEL17	DRB1*01:29	5	19	LKRCLLHLAVIGALL	45.25
3203	P40967	PMEL17	DRB1*01:18	263	277	GDSSGTLISRALVVT	45.27
3204	P40967	PMEL17	DRB1*14:07	229	243	NKHFLRNQPLTFALQ	45.30
3205	P40967	PMEL17	DRB1*11:02	458	472	LDGTATLRLVKRQVP	45.35
3206	P40967	PMEL17	DRB1*11:65	458	472	LDGTATLRLVKRQVP	45.35
3207	P40967	PMEL17	DRB1*13:01	458	472	LDGTATLRLVKRQVP	45.35
3208	P40967	PMEL17	DRB1*01:20	169	183	GLSIGTGRAMLGTHT	45.38
3209	P40967	PMEL17	DRB1*13:61	609	623	VVLASLIYRRRLMKQ	45.40
3210	P40967	PMEL17	DRB1*07:01	174	188	TGRAMLGTHTMEVTV	45.41
3211	P40967	PMEL17	DRB1*11:01	550	564	CQLVLHQILKGGSGT	45.41
3212	P40967	PMEL17	DRB1*11:10	550	564	CQLVLHQILKGGSGT	45.41
3213	P40967	PMEL17	DRB1*11:12	550	564	CQLVLHQILKGGSGT	45.41
3214	P40967	PMEL17	DRB1*11:28	550	564	CQLVLHQILKGGSGT	45.41
3215	P40967	PMEL17	DRB1*11:29	550	564	CQLVLHQILKGGSGT	45.41
3216	P40967	PMEL17	DRB1*11:49	550	564	CQLVLHQILKGGSGT	45.41
3217	P40967	PMEL17	DRB1*11:62	550	564	CQLVLHQILKGGSGT	45.41

3218	P40967	PMEL17	DRB1*11:74	550	564	CQLVLHQILKGGSGT	45.41
3219	P40967	PMEL17	DRB1*13:05	550	564	CQLVLHQILKGGSGT	45.41
3220	P40967	PMEL17	DRB1*13:14	550	564	CQLVLHQILKGGSGT	45.41
3221	P40967	PMEL17	DRB1*13:50	550	564	CQLVLHQILKGGSGT	45.41
3222	P40967	PMEL17	DRB1*11:02	228	242	GNKHFLRNQPLTFAL	45.46
3223	P40967	PMEL17	DRB1*11:65	228	242	GNKHFLRNQPLTFAL	45.46
3224	P40967	PMEL17	DRB1*13:01	228	242	GNKHFLRNQPLTFAL	45.46
3225	P40967	PMEL17	DRB1*04:05	78	92	IGANASFSIALNFPG	45.54
3226	P40967	PMEL17	DRB1*11:42	146	160	KRSFVYVWKTWGQYW	45.58
3227	P40967	PMEL17	DRB1*04:08	405	419	VSIVVLSGTTAAQVT	45.64
3228	P40967	PMEL17	DPB1*33:01	477	491	LYRYGSFSVTLDIVQ	45.70
3229	P40967	PMEL17	DPB1*71:01	477	491	LYRYGSFSVTLDIVQ	45.70
3230	P40967	PMEL17	DRB1*11:84	610	624	VLASLIYRRRLMKQD	45.75
3231	P40967	PMEL17	DRB1*01:24	4	18	VLKRCLLHLAVIGAL	45.78
3232	P40967	PMEL17	DRB1*11:02	230	244	KHFLRNQPLTFALQL	45.78
3233	P40967	PMEL17	DRB1*11:65	230	244	KHFLRNQPLTFALQL	45.78
3234	P40967	PMEL17	DRB1*13:01	230	244	KHFLRNQPLTFALQL	45.78
3235	P40967	PMEL17	DRB1*01:20	231	245	HFLRNQPLTFALQLH	45.80
3236	P40967	PMEL17	DRB1*01:20	76	90	TLIGANASFSIALNF	45.92
3237	P40967	PMEL17	DRB1*11:13	287	301	TAQVVLQAAIPLTSC	45.92
3238	P40967	PMEL17	DRB1*01:02	403	417	AEVSIVVLSGTTAAQ	45.95
3239	P40967	PMEL17	DRB1*01:20	609	623	VVLASLIYRRRLMKQ	46.02
3240	P40967	PMEL17	DRB1*11:04	144	158	SQKRSFVYVWKTWGQ	46.19
3241	P40967	PMEL17	DRB1*11:46	144	158	SQKRSFVYVWKTWGQ	46.19
3242	P40967	PMEL17	DRB1*11:58	144	158	SQKRSFVYVWKTWGQ	46.19
3243	P40967	PMEL17	DRB1*13:11	144	158	SQKRSFVYVWKTWGQ	46.19
3244	P40967	PMEL17	DRB1*01:01	73	87	DGPTLIGANASFSIA	46.20
3245	P40967	PMEL17	DRB1*15:01	287	301	TAQVVLQAAIPLTSC	46.23
3246	P40967	PMEL17	DRB1*15:06	287	301	TAQVVLQAAIPLTSC	46.23
3247	P40967	PMEL17	DRB1*10:01	284	298	GPVTAQVVLQAAIPL	46.25
3248	P40967	PMEL17	DRB1*11:19	230	244	KHFLRNQPLTFALQL	46.32
3249	P40967	PMEL17	DRB1*11:03	549	563	ACQLVLHQILKGGSG	46.57
3250	P40967	PMEL17	DRB1*10:01	266	280	SGTLISRALVVTHTY	46.65
3251	P40967	PMEL17	DRB1*14:06	229	243	NKHFLRNQPLTFALQ	46.86
3252	P40967	PMEL17	DRB1*14:32	607	621	MAVVLASLIYRRRLM	46.89
3253	P40967	PMEL17	DRB1*11:14	13	27	AVIGALLAVGATKVP	46.91
3254	P40967	PMEL17	DRB1*13:02	13	27	AVIGALLAVGATKVP	46.91
3255	P40967	PMEL17	DRB1*13:23	13	27	AVIGALLAVGATKVP	46.91
3256	P40967	PMEL17	DRB1*13:97	13	27	AVIGALLAVGATKVP	46.91
3257	P40967	PMEL17	DRB1*13:21	610	624	VLASLIYRRRLMKQD	46.93
3258	P40967	PMEL17	DRB1*07:01	456	470	PLLDGTATLRLVKRQ	46.94
3259	P40967	PMEL17	DRB1*14:32	287	301	TAQVVLQAAIPLTSC	47.07
3260	P40967	PMEL17	DRB1*03:11	229	243	NKHFLRNQPLTFALQ	47.31

3261	P40967	PMEL17	DRB1*01:01	263	277	GDSSGTLISRALVVT	47.48
3262	P40967	PMEL17	DRB1*11:42	286	300	VTAQVVLQAAIPLTS	47.48
3263	P40967	PMEL17	DRB1*14:12	609	623	VVLASLIYRRRLMKQ	47.51
3264	P40967	PMEL17	DRB1*13:96	285	299	PVTAQVVLQAAIPLT	47.53
3265	P40967	PMEL17	DRB1*10:01	403	417	AEVSIVVLSGTTAAQ	47.54
3266	P40967	PMEL17	DRB1*07:01	417	431	QVTTTEWVETTAREL	47.62
3267	P40967	PMEL17	DRB1*04:44	405	419	VSIVVLSGTTAAQVT	47.75
3268	P40967	PMEL17	DRB1*01:18	607	621	MAVVLASLIYRRRLM	47.77
3269	P40967	PMEL17	DRB1*10:01	75	89	PTLIGANASFSIALN	47.77
3270	P40967	PMEL17	DRB1*11:13	285	299	PVTAQVVLQAAIPLT	47.85
3271	P40967	PMEL17	DRB1*11:54	459	473	DGTATLRLVKRQVPL	47.87
3272	P40967	PMEL17	DRB1*11:02	185	199	EVTVYHRRGSRSYVP	47.92
3273	P40967	PMEL17	DRB1*11:65	185	199	EVTVYHRRGSRSYVP	47.92
3274	P40967	PMEL17	DRB1*13:01	185	199	EVTVYHRRGSRSYVP	47.92
3275	P40967	PMEL17	DRB1*01:20	542	556	QPVLPSPACQLVLHQ	47.93
3276	P40967	PMEL17	DRB1*13:66	226	240	DGGNKHFLRNQPLTF	48.03
3277	P40967	PMEL17	DRB1*11:42	145	159	QKRSFVYVWKTWGQY	48.04
3278	P40967	PMEL17	DRB1*09:01	156	170	WGQYWQVLGGPVSGL	48.18
3279	P40967	PMEL17	DRB1*11:04	33	47	LGVSRQLRTKAWNRQ	48.19
3280	P40967	PMEL17	DRB1*11:46	33	47	LGVSRQLRTKAWNRQ	48.19
3281	P40967	PMEL17	DRB1*11:58	33	47	LGVSRQLRTKAWNRQ	48.19
3282	P40967	PMEL17	DRB1*13:11	33	47	LGVSRQLRTKAWNRQ	48.19
3283	P40967	PMEL17	DRB1*03:11	483	497	FSVTLDIVQGIESAE	48.27
3284	P40967	PMEL17	DRB1*01:01	3	17	LVLKRCLLHLAVIGA	48.29
3285	P40967	PMEL17	DRB1*01:24	7	21	RCLLHLAVIGALLAV	48.30
3286	P40967	PMEL17	DRB1*01:20	541	555	CQPVLPSPACQLVLH	48.38
3287	P40967	PMEL17	DRB1*11:03	29	43	NQDWLGVSRQLRTKA	48.39
3288	P40967	PMEL17	DRB1*11:03	186	200	VTVYHRRGSRSYVPL	48.46
3289	P40967	PMEL17	DRB1*04:72	82	96	ASFSIALNFPGSQKV	48.49
3290	P40967	PMEL17	DRB1*12:03	286	300	VTAQVVLQAAIPLTS	48.50
3291	P40967	PMEL17	DRB1*01:20	455	469	GPLLDGTATLRLVKR	48.61
3292	P40967	PMEL17	DRB1*11:02	463	477	TLRLVKRQVPLDCVL	48.63
3293	P40967	PMEL17	DRB1*11:65	463	477	TLRLVKRQVPLDCVL	48.63
3294	P40967	PMEL17	DRB1*13:01	463	477	TLRLVKRQVPLDCVL	48.63
3295	P40967	PMEL17	DRB1*13:61	229	243	NKHFLRNQPLTFALQ	48.63
3296	P40967	PMEL17	DRB1*01:29	4	18	VLKRCLLHLAVIGAL	48.64
3297	P40967	PMEL17	DRB1*09:01	78	92	IGANASFSIALNFPG	48.64
3298	P40967	PMEL17	DRB1*15:02	474	488	DCVLYRYGSFSVTLD	48.71
3299	P40967	PMEL17	DRB1*07:01	197	211	YVPLAHSSSAFTITD	48.73
3300	P40967	PMEL17	DRB1*14:32	608	622	AVVLASLIYRRRLMK	48.73
3301	P40967	PMEL17	DRB1*14:06	230	244	KHFLRNQPLTFALQL	48.80
3302	P40967	PMEL17	DRB1*11:42	229	243	NKHFLRNQPLTFALQ	48.81
3303	P40967	PMEL17	DRB1*14:32	284	298	GPVTAQVVLQAAIPL	48.86

3304	P40967	PMEL17	DPB1*33:01	472	486	PLDCVLYRYGSFSVT	48.96
3305	P40967	PMEL17	DPB1*71:01	472	486	PLDCVLYRYGSFSVT	48.96
3306	P40967	PMEL17	DRB1*15:02	476	490	VLYRYGSFSVTLDIV	48.99
3307	P40967	PMEL17	DRB1*15:07	474	488	DCVLYRYGSFSVTLD	49.01
3308	P40967	PMEL17	DRB1*11:14	81	95	NASFSIALNFPGSQK	49.03
3309	P40967	PMEL17	DRB1*13:02	81	95	NASFSIALNFPGSQK	49.03
3310	P40967	PMEL17	DRB1*13:23	81	95	NASFSIALNFPGSQK	49.03
3311	P40967	PMEL17	DRB1*13:97	81	95	NASFSIALNFPGSQK	49.03
3312	P40967	PMEL17	DRB1*11:14	404	418	EVSIVVLSGTTAAQV	49.09
3313	P40967	PMEL17	DRB1*13:02	404	418	EVSIVVLSGTTAAQV	49.09
3314	P40967	PMEL17	DRB1*13:23	404	418	EVSIVVLSGTTAAQV	49.09
3315	P40967	PMEL17	DRB1*13:97	404	418	EVSIVVLSGTTAAQV	49.09
3316	P40967	PMEL17	DRB1*07:01	420	434	TTEWVETTARELPIP	49.19
3317	P40967	PMEL17	DRB1*01:20	206	220	AFTITDQVPFSVSVS	49.22
3318	P40967	PMEL17	DRB1*08:04	549	563	ACQLVLHQILKGGSG	49.22
3319	P40967	PMEL17	DRB1*16:09	79	93	GANASFSIALNFPGS	49.43
3320	P40967	PMEL17	DRB1*11:01	80	94	ANASFSIALNFPGSQ	49.44
3321	P40967	PMEL17	DRB1*11:10	80	94	ANASFSIALNFPGSQ	49.44
3322	P40967	PMEL17	DRB1*11:12	80	94	ANASFSIALNFPGSQ	49.44
3323	P40967	PMEL17	DRB1*11:28	80	94	ANASFSIALNFPGSQ	49.44
3324	P40967	PMEL17	DRB1*11:29	80	94	ANASFSIALNFPGSQ	49.44
3325	P40967	PMEL17	DRB1*11:49	80	94	ANASFSIALNFPGSQ	49.44
3326	P40967	PMEL17	DRB1*11:62	80	94	ANASFSIALNFPGSQ	49.44
3327	P40967	PMEL17	DRB1*11:74	80	94	ANASFSIALNFPGSQ	49.44
3328	P40967	PMEL17	DRB1*13:05	80	94	ANASFSIALNFPGSQ	49.44
3329	P40967	PMEL17	DRB1*13:14	80	94	ANASFSIALNFPGSQ	49.44
3330	P40967	PMEL17	DRB1*13:50	80	94	ANASFSIALNFPGSQ	49.44
3331	P40967	PMEL17	DRB1*12:03	606	620	LMAVVLASLIYRRRL	49.45
3332	P40967	PMEL17	DRB1*11:08	461	475	TATLRLVKRQVPLDC	49.52
3333	P40967	PMEL17	DRB1*11:37	144	158	SQKRSFVYVWKTWGQ	49.54
3334	P40967	PMEL17	DRB1*13:07	144	158	SQKRSFVYVWKTWGQ	49.54
3335	P40967	PMEL17	DRB1*01:18	216	230	SVSVSQLRALDGGNK	49.57
3336	P40967	PMEL17	DRB1*01:29	195	209	RSYVPLAHSSSAFTI	49.65
3337	P40967	PMEL17	DRB1*09:01	82	96	ASFSIALNFPGSQKV	49.67
3338	P40967	PMEL17	DRB1*15:07	472	486	PLDCVLYRYGSFSVT	49.89
3339	P40967	PMEL17	DRB1*13:33	228	242	GNKHFLRNQPLTFAL	49.93
3340	P40967	PMEL17	DRB1*15:37	474	488	DCVLYRYGSFSVTLD	49.93
3341	P40967	PMEL17	DRB1*11:04	553	567	VLHQILKGGSGTYCL	49.95
3342	P40967	PMEL17	DRB1*11:46	553	567	VLHQILKGGSGTYCL	49.95
3343	P40967	PMEL17	DRB1*11:58	553	567	VLHQILKGGSGTYCL	49.95
3344	P40967	PMEL17	DRB1*13:11	553	567	VLHQILKGGSGTYCL	49.95
3345	P40967	PMEL17	DRB1*07:01	227	241	GGNKHFLRNQPLTFA	49.96
3346	P40967	PMEL17	DRB1*01:01	165	179	GPVSGLSIGTGRAML	49.97

3347	P40967	PMEL17	DRB1*01:24	154	168	KTWGQYWQVLGGPVS	49.99
3348	P43355	MAGE1	DRB1*01:18	95	109	ESLFRAVITKKVADL	5.72
3349	P43355	MAGE1	DRB1*01:01	95	109	ESLFRAVITKKVADL	6.03
3350	P43355	MAGE1	DRB1*01:18	96	110	SLFRAVITKKVADLV	6.08
3351	P43355	MAGE1	DRB1*01:18	93	107	ILESLFRAVITKKVA	6.54
3352	P43355	MAGE1	DRB1*01:01	96	110	SLFRAVITKKVADLV	6.68
3353	P43355	MAGE1	DRB1*01:18	94	108	LESLFRAVITKKVAD	6.74
3354	P43355	MAGE1	DRB1*01:01	93	107	ILESLFRAVITKKVA	7.20
3355	P43355	MAGE1	DRB1*01:01	94	108	LESLFRAVITKKVAD	7.20
3356	P43355	MAGE1	DRB1*07:01	92	106	CILESLFRAVITKKV	7.59
3357	P43355	MAGE1	DRB1*07:01	93	107	ILESLFRAVITKKVA	7.63
3358	P43355	MAGE1	DRB1*01:18	280	294	LEYVIKVSARVRFFF	7.83
3359	P43355	MAGE1	DRB1*01:18	258	272	DPARYEFLWGPRALA	7.84
3360	P43355	MAGE1	DRB1*01:01	258	272	DPARYEFLWGPRALA	7.86
3361	P43355	MAGE1	DRB1*01:18	279	293	VLEYVIKVSARVRFF	7.97
3362	P43355	MAGE1	DRB1*01:18	260	274	ARYEFLWGPRALAET	8.09
3363	P43355	MAGE1	DRB1*01:01	260	274	ARYEFLWGPRALAET	8.13
3364	P43355	MAGE1	DRB1*01:18	278	292	KVLEYVIKVSARVRF	8.36
3365	P43355	MAGE1	DRB1*07:01	95	109	ESLFRAVITKKVADL	8.52
3366	P43355	MAGE1	DRB1*01:18	259	273	PARYEFLWGPRALAE	8.62
3367	P43355	MAGE1	DRB1*01:18	97	111	LFRAVITKKVADLVG	8.78
3368	P43355	MAGE1	DRB1*01:01	259	273	PARYEFLWGPRALAE	8.83
3369	P43355	MAGE1	DRB1*07:01	96	110	SLFRAVITKKVADLV	8.83
3370	P43355	MAGE1	DPB1*33:01	90	104	TSCILESLFRAVITK	9.30
3371	P43355	MAGE1	DPB1*71:01	90	104	TSCILESLFRAVITK	9.30
3372	P43355	MAGE1	DRB1*11:02	280	294	LEYVIKVSARVRFFF	9.32
3373	P43355	MAGE1	DRB1*11:65	280	294	LEYVIKVSARVRFFF	9.32
3374	P43355	MAGE1	DRB1*13:01	280	294	LEYVIKVSARVRFFF	9.32
3375	P43355	MAGE1	DRB1*11:02	281	295	EYVIKVSARVRFFFP	9.36
3376	P43355	MAGE1	DRB1*11:65	281	295	EYVIKVSARVRFFFP	9.36
3377	P43355	MAGE1	DRB1*13:01	281	295	EYVIKVSARVRFFFP	9.36
3378	P43355	MAGE1	DRB1*01:29	95	109	ESLFRAVITKKVADL	9.45
3379	P43355	MAGE1	DRB1*01:01	257	271	SDPARYEFLWGPRAL	9.72
3380	P43355	MAGE1	DRB1*01:18	281	295	EYVIKVSARVRFFFP	9.76
3381	P43355	MAGE1	DRB1*01:18	277	291	VKVLEYVIKVSARVR	9.90
3382	P43355	MAGE1	DRB1*07:01	94	108	LESLFRAVITKKVAD	9.95
3383	P43355	MAGE1	DRB1*11:02	279	293	VLEYVIKVSARVRFF	9.97
3384	P43355	MAGE1	DRB1*11:65	279	293	VLEYVIKVSARVRFF	9.97
3385	P43355	MAGE1	DRB1*13:01	279	293	VLEYVIKVSARVRFF	9.97
3386	P43355	MAGE1	DRB1*01:18	257	271	SDPARYEFLWGPRAL	10.11
3387	P43355	MAGE1	DRB1*11:13	281	295	EYVIKVSARVRFFFP	10.34
3388	P43355	MAGE1	DRB1*11:13	280	294	LEYVIKVSARVRFFF	10.40
3389	P43355	MAGE1	DRB1*01:29	96	110	SLFRAVITKKVADLV	10.47

3390	P43355	MAGE1	DRB1*11:42	280	294	LEYVIKVSARVRFFF	10.49
3391	P43355	MAGE1	DRB1*11:42	279	293	VLEYVIKVSARVRFF	10.57
3392	P43355	MAGE1	DRB1*01:18	261	275	RYEFLWGPRALAETS	10.59
3393	P43355	MAGE1	DRB1*11:42	281	295	EYVIKVSARVRFFFP	10.60
3394	P43355	MAGE1	DRB1*01:01	97	111	LFRAVITKKVADLVG	10.68
3395	P43355	MAGE1	DRB1*11:42	278	292	KVLEYVIKVSARVRF	10.88
3396	P43355	MAGE1	DRB1*11:13	279	293	VLEYVIKVSARVRFF	10.92
3397	P43355	MAGE1	DRB1*01:01	261	275	RYEFLWGPRALAETS	11.06
3398	P43355	MAGE1	DRB1*01:18	92	106	CILESLFRAVITKKV	11.32
3399	P43355	MAGE1	DRB1*01:01	280	294	LEYVIKVSARVRFFF	11.33
3400	P43355	MAGE1	DRB1*01:29	93	107	ILESLFRAVITKKVA	11.49
3401	P43355	MAGE1	DPB1*33:01	89	103	STSCILESLFRAVIT	11.81
3402	P43355	MAGE1	DPB1*71:01	89	103	STSCILESLFRAVIT	11.81
3403	P43355	MAGE1	DRB1*01:29	94	108	LESLFRAVITKKVAD	11.81
3404	P43355	MAGE1	DPB1*33:01	88	102	PSTSCILESLFRAVI	11.82
3405	P43355	MAGE1	DPB1*71:01	88	102	PSTSCILESLFRAVI	11.82
3406	P43355	MAGE1	DRB1*01:01	279	293	VLEYVIKVSARVRFF	11.83
3407	P43355	MAGE1	DRB1*01:01	278	292	KVLEYVIKVSARVRF	11.89
3408	P43355	MAGE1	DRB1*01:20	197	211	IVLVMIAMEGGHAPE	12.17
3409	P43355	MAGE1	DRB1*01:24	95	109	ESLFRAVITKKVADL	12.26
3410	P43355	MAGE1	DPB1*33:01	87	101	GPSTSCILESLFRAV	12.40
3411	P43355	MAGE1	DPB1*71:01	87	101	GPSTSCILESLFRAV	12.40
3412	P43355	MAGE1	DRB1*11:02	282	296	YVIKVSARVRFFFPS	12.43
3413	P43355	MAGE1	DRB1*11:65	282	296	YVIKVSARVRFFFPS	12.43
3414	P43355	MAGE1	DRB1*13:01	282	296	YVIKVSARVRFFFPS	12.43
3415	P43355	MAGE1	DPB1*33:01	91	105	SCILESLFRAVITKK	12.55
3416	P43355	MAGE1	DPB1*71:01	91	105	SCILESLFRAVITKK	12.55
3417	P43355	MAGE1	DRB1*11:13	278	292	KVLEYVIKVSARVRF	12.69
3418	P43355	MAGE1	DRB1*01:20	196	210	IIVLVMIAMEGGHAP	12.70
3419	P43355	MAGE1	DRB1*01:24	96	110	SLFRAVITKKVADLV	12.74
3420	P43355	MAGE1	DRB1*11:13	282	296	YVIKVSARVRFFFPS	12.91
3421	P43355	MAGE1	DRB1*11:02	278	292	KVLEYVIKVSARVRF	12.95
3422	P43355	MAGE1	DRB1*11:65	278	292	KVLEYVIKVSARVRF	12.95
3423	P43355	MAGE1	DRB1*13:01	278	292	KVLEYVIKVSARVRF	12.95
3424	P43355	MAGE1	DRB1*14:32	281	295	EYVIKVSARVRFFFP	13.06
3425	P43355	MAGE1	DRB1*07:01	97	111	LFRAVITKKVADLVG	13.10
3426	P43355	MAGE1	DRB1*14:32	280	294	LEYVIKVSARVRFFF	13.51
3427	P43355	MAGE1	DRB1*01:01	281	295	EYVIKVSARVRFFFP	13.52
3428	P43355	MAGE1	DRB1*10:01	95	109	ESLFRAVITKKVADL	13.58
3429	P43355	MAGE1	DRB1*01:01	277	291	VKVLEYVIKVSARVR	13.87
3430	P43355	MAGE1	DRB1*01:01	92	106	CILESLFRAVITKKV	13.91
3431	P43355	MAGE1	DRB1*11:01	289	303	RVRFFFPSLREAALR	14.00
3432	P43355	MAGE1	DRB1*11:10	289	303	RVRFFFPSLREAALR	14.00

3433	P43355	MAGE1	DRB1*11:12	289	303	RVRFFFPSLREAALR	14.00
3434	P43355	MAGE1	DRB1*11:28	289	303	RVRFFFPSLREAALR	14.00
3435	P43355	MAGE1	DRB1*11:29	289	303	RVRFFFPSLREAALR	14.00
3436	P43355	MAGE1	DRB1*11:49	289	303	RVRFFFPSLREAALR	14.00
3437	P43355	MAGE1	DRB1*11:62	289	303	RVRFFFPSLREAALR	14.00
3438	P43355	MAGE1	DRB1*11:74	289	303	RVRFFFPSLREAALR	14.00
3439	P43355	MAGE1	DRB1*13:05	289	303	RVRFFFPSLREAALR	14.00
3440	P43355	MAGE1	DRB1*13:14	289	303	RVRFFFPSLREAALR	14.00
3441	P43355	MAGE1	DRB1*13:50	289	303	RVRFFFPSLREAALR	14.00
3442	P43355	MAGE1	DRB1*01:20	198	212	VLVMIAMEGGHAPEE	14.12
3443	P43355	MAGE1	DRB1*11:04	278	292	KVLEYVIKVSARVRF	14.12
3444	P43355	MAGE1	DRB1*11:46	278	292	KVLEYVIKVSARVRF	14.12
3445	P43355	MAGE1	DRB1*11:58	278	292	KVLEYVIKVSARVRF	14.12
3446	P43355	MAGE1	DRB1*13:11	278	292	KVLEYVIKVSARVRF	14.12
3447	P43355	MAGE1	DRB1*14:32	279	293	VLEYVIKVSARVRFF	14.14
3448	P43355	MAGE1	DRB1*01:11	95	109	ESLFRAVITKKVADL	14.30
3449	P43355	MAGE1	DRB1*11:42	282	296	YVIKVSARVRFFFPS	14.54
3450	P43355	MAGE1	DRB1*11:01	290	304	VRFFFPSLREAALRE	14.60
3451	P43355	MAGE1	DRB1*11:10	290	304	VRFFFPSLREAALRE	14.60
3452	P43355	MAGE1	DRB1*11:12	290	304	VRFFFPSLREAALRE	14.60
3453	P43355	MAGE1	DRB1*11:28	290	304	VRFFFPSLREAALRE	14.60
3454	P43355	MAGE1	DRB1*11:29	290	304	VRFFFPSLREAALRE	14.60
3455	P43355	MAGE1	DRB1*11:49	290	304	VRFFFPSLREAALRE	14.60
3456	P43355	MAGE1	DRB1*11:62	290	304	VRFFFPSLREAALRE	14.60
3457	P43355	MAGE1	DRB1*11:74	290	304	VRFFFPSLREAALRE	14.60
3458	P43355	MAGE1	DRB1*13:05	290	304	VRFFFPSLREAALRE	14.60
3459	P43355	MAGE1	DRB1*13:14	290	304	VRFFFPSLREAALRE	14.60
3460	P43355	MAGE1	DRB1*13:50	290	304	VRFFFPSLREAALRE	14.60
3461	P43355	MAGE1	DRB1*01:01	197	211	IVLVMIAMEGGHAPE	14.68
3462	P43355	MAGE1	DRB1*01:24	93	107	ILESLFRAVITKKVA	14.71
3463	P43355	MAGE1	DPB1*02:01	90	104	TSCILESLFRAVITK	14.80
3464	P43355	MAGE1	DPB1*46:01	90	104	TSCILESLFRAVITK	14.80
3465	P43355	MAGE1	DPB1*81:01	90	104	TSCILESLFRAVITK	14.80
3466	P43355	MAGE1	DPB1*02:02	90	104	TSCILESLFRAVITK	14.89
3467	P43355	MAGE1	DPB1*47:01	90	104	TSCILESLFRAVITK	14.89
3468	P43355	MAGE1	DRB1*01:29	280	294	LEYVIKVSARVRFFF	15.65
3469	P43355	MAGE1	DRB1*10:01	96	110	SLFRAVITKKVADLV	15.67
3470	P43355	MAGE1	DRB1*01:18	103	117	TKKVADLVGFLLLKY	15.79
3471	P43355	MAGE1	DRB1*10:01	93	107	ILESLFRAVITKKVA	15.80
3472	P43355	MAGE1	DRB1*01:01	196	210	IIVLVMIAMEGGHAP	15.82
3473	P43355	MAGE1	DRB1*11:42	277	291	VKVLEYVIKVSARVR	15.96
3474	P43355	MAGE1	DRB1*01:18	197	211	IVLVMIAMEGGHAPE	15.98
3475	P43355	MAGE1	DRB1*14:32	278	292	KVLEYVIKVSARVRF	16.02

3476	P43355	MAGE1	DRB1*01:24	94	108	LESLFRAVITKKVAD	16.11
3477	P43355	MAGE1	DRB1*10:01	94	108	LESLFRAVITKKVAD	16.11
3478	P43355	MAGE1	DRB1*14:32	282	296	YVIKVSARVRFFFPS	16.15
3479	P43355	MAGE1	DRB1*11:04	279	293	VLEYVIKVSARVRFF	16.18
3480	P43355	MAGE1	DRB1*11:46	279	293	VLEYVIKVSARVRFF	16.18
3481	P43355	MAGE1	DRB1*11:58	279	293	VLEYVIKVSARVRFF	16.18
3482	P43355	MAGE1	DRB1*13:11	279	293	VLEYVIKVSARVRFF	16.18
3483	P43355	MAGE1	DRB1*01:20	95	109	ESLFRAVITKKVADL	16.38
3484	P43355	MAGE1	DRB1*01:11	96	110	SLFRAVITKKVADLV	16.39
3485	P43355	MAGE1	DRB1*01:01	198	212	VLVMIAMEGGHAPEE	16.47
3486	P43355	MAGE1	DRB1*13:21	289	303	RVRFFFPSLREAALR	16.73
3487	P43355	MAGE1	DRB1*01:29	258	272	DPARYEFLWGPRALA	16.85
3488	P43355	MAGE1	DRB1*01:29	278	292	KVLEYVIKVSARVRF	17.06
3489	P43355	MAGE1	DRB1*01:29	279	293	VLEYVIKVSARVRFF	17.09
3490	P43355	MAGE1	DRB1*01:29	259	273	PARYEFLWGPRALAE	17.19
3491	P43355	MAGE1	DRB1*11:04	109	123	LVGFLLLKYRAREPV	17.19
3492	P43355	MAGE1	DRB1*11:46	109	123	LVGFLLLKYRAREPV	17.19
3493	P43355	MAGE1	DRB1*11:58	109	123	LVGFLLLKYRAREPV	17.19
3494	P43355	MAGE1	DRB1*13:11	109	123	LVGFLLLKYRAREPV	17.19
3495	P43355	MAGE1	DRB1*11:04	280	294	LEYVIKVSARVRFFF	17.21
3496	P43355	MAGE1	DRB1*11:46	280	294	LEYVIKVSARVRFFF	17.21
3497	P43355	MAGE1	DRB1*11:58	280	294	LEYVIKVSARVRFFF	17.21
3498	P43355	MAGE1	DRB1*13:11	280	294	LEYVIKVSARVRFFF	17.21
3499	P43355	MAGE1	DRB1*01:20	93	107	ILESLFRAVITKKVA	17.30
3500	P43355	MAGE1	DRB1*01:18	196	210	IIVLVMIAMEGGHAP	17.62
3501	P43355	MAGE1	DRB1*01:29	97	111	LFRAVITKKVADLVG	17.65
3502	P43355	MAGE1	DRB1*01:18	276	290	YVKVLEYVIKVSARV	17.68
3503	P43355	MAGE1	DRB1*11:04	281	295	EYVIKVSARVRFFFP	17.68
3504	P43355	MAGE1	DRB1*11:46	281	295	EYVIKVSARVRFFFP	17.68
3505	P43355	MAGE1	DRB1*11:58	281	295	EYVIKVSARVRFFFP	17.68
3506	P43355	MAGE1	DRB1*13:11	281	295	EYVIKVSARVRFFFP	17.68
3507	P43355	MAGE1	DPB1*33:01	104	118	KKVADLVGFLLLKYR	17.70
3508	P43355	MAGE1	DPB1*71:01	104	118	KKVADLVGFLLLKYR	17.70
3509	P43355	MAGE1	DRB1*16:09	96	110	SLFRAVITKKVADLV	17.72
3510	P43355	MAGE1	DRB1*11:03	281	295	EYVIKVSARVRFFFP	17.74
3511	P43355	MAGE1	DRB1*01:18	198	212	VLVMIAMEGGHAPEE	17.79
3512	P43355	MAGE1	DRB1*11:03	110	124	VGFLLLKYRAREPVT	17.79
3513	P43355	MAGE1	DRB1*13:61	280	294	LEYVIKVSARVRFFF	17.80
3514	P43355	MAGE1	DRB1*11:03	279	293	VLEYVIKVSARVRFF	17.85
3515	P43355	MAGE1	DRB1*01:18	102	116	ITKKVADLVGFLLLK	17.93
3516	P43355	MAGE1	DRB1*01:20	148	162	SESLQLVFGIDVKEA	17.94
3517	P43355	MAGE1	DRB1*01:11	93	107	ILESLFRAVITKKVA	17.96
3518	P43355	MAGE1	DRB1*11:04	108	122	DLVGFLLLKYRAREP	17.99

3519	P43355	MAGE1	DRB1*11:46	108	122	DLVGFLLLKYRAREP	17.99
3520	P43355	MAGE1	DRB1*11:58	108	122	DLVGFLLLKYRAREP	17.99
3521	P43355	MAGE1	DRB1*13:11	108	122	DLVGFLLLKYRAREP	17.99
3522	P43355	MAGE1	DRB1*01:29	260	274	ARYEFLWGPRALAET	18.01
3523	P43355	MAGE1	DRB1*16:02	95	109	ESLFRAVITKKVADL	18.02
3524	P43355	MAGE1	DRB1*01:20	103	117	TKKVADLVGFLLLKY	18.06
3525	P43355	MAGE1	DRB1*01:18	148	162	SESLQLVFGIDVKEA	18.19
3526	P43355	MAGE1	DRB1*03:11	281	295	EYVIKVSARVRFFFP	18.21
3527	P43355	MAGE1	DRB1*11:03	111	125	GFLLLKYRAREPVTK	18.27
3528	P43355	MAGE1	DRB1*10:01	258	272	DPARYEFLWGPRALA	18.33
3529	P43355	MAGE1	DRB1*01:20	96	110	SLFRAVITKKVADLV	18.38
3530	P43355	MAGE1	DRB1*01:20	199	213	LVMIAMEGGHAPEEE	18.41
3531	P43355	MAGE1	DRB1*01:24	280	294	LEYVIKVSARVRFFF	18.45
3532	P43355	MAGE1	DRB1*01:20	280	294	LEYVIKVSARVRFFF	18.46
3533	P43355	MAGE1	DRB1*03:11	280	294	LEYVIKVSARVRFFF	18.47
3534	P43355	MAGE1	DRB1*01:24	279	293	VLEYVIKVSARVRFF	18.49
3535	P43355	MAGE1	DRB1*01:29	281	295	EYVIKVSARVRFFFP	18.53
3536	P43355	MAGE1	DRB1*13:21	109	123	LVGFLLLKYRAREPV	18.53
3537	P43355	MAGE1	DRB1*13:61	281	295	EYVIKVSARVRFFFP	18.53
3538	P43355	MAGE1	DRB1*01:24	258	272	DPARYEFLWGPRALA	18.56
3539	P43355	MAGE1	DRB1*01:11	94	108	LESLFRAVITKKVAD	18.57
3540	P43355	MAGE1	DRB1*16:09	95	109	ESLFRAVITKKVADL	18.57
3541	P43355	MAGE1	DRB1*11:03	109	123	LVGFLLLKYRAREPV	18.65
3542	P43355	MAGE1	DRB1*11:03	280	294	LEYVIKVSARVRFFF	18.68
3543	P43355	MAGE1	DRB1*10:01	260	274	ARYEFLWGPRALAET	18.69
3544	P43355	MAGE1	DRB1*01:11	279	293	VLEYVIKVSARVRFF	18.70
3545	P43355	MAGE1	DRB1*01:24	97	111	LFRAVITKKVADLVG	18.77
3546	P43355	MAGE1	DPB1*33:01	268	282	PRALAETSYVKVLEY	18.80
3547	P43355	MAGE1	DPB1*71:01	268	282	PRALAETSYVKVLEY	18.80
3548	P43355	MAGE1	DRB1*11:01	291	305	RFFFPSLREAALREE	18.80
3549	P43355	MAGE1	DRB1*11:04	110	124	VGFLLLKYRAREPVT	18.80
3550	P43355	MAGE1	DRB1*11:10	291	305	RFFFPSLREAALREE	18.80
3551	P43355	MAGE1	DRB1*11:12	291	305	RFFFPSLREAALREE	18.80
3552	P43355	MAGE1	DRB1*11:28	291	305	RFFFPSLREAALREE	18.80
3553	P43355	MAGE1	DRB1*11:29	291	305	RFFFPSLREAALREE	18.80
3554	P43355	MAGE1	DRB1*11:46	110	124	VGFLLLKYRAREPVT	18.80
3555	P43355	MAGE1	DRB1*11:49	291	305	RFFFPSLREAALREE	18.80
3556	P43355	MAGE1	DRB1*11:58	110	124	VGFLLLKYRAREPVT	18.80
3557	P43355	MAGE1	DRB1*11:62	291	305	RFFFPSLREAALREE	18.80
3558	P43355	MAGE1	DRB1*11:74	291	305	RFFFPSLREAALREE	18.80
3559	P43355	MAGE1	DRB1*13:05	291	305	RFFFPSLREAALREE	18.80
3560	P43355	MAGE1	DRB1*13:11	110	124	VGFLLLKYRAREPVT	18.80
3561	P43355	MAGE1	DRB1*13:14	291	305	RFFFPSLREAALREE	18.80

3562	P43355	MAGE1	DRB1*13:50	291	305	RFFFPSLREAALREE	18.80
3563	P43355	MAGE1	DRB1*16:01	96	110	SLFRAVITKKVADLV	18.93
3564	P43355	MAGE1	DRB1*16:01	95	109	ESLFRAVITKKVADL	19.00
3565	P43355	MAGE1	DRB1*01:20	102	116	ITKKVADLVGFLLLK	19.09
3566	P43355	MAGE1	DPB1*33:01	92	106	CILESLFRAVITKKV	19.12
3567	P43355	MAGE1	DPB1*71:01	92	106	CILESLFRAVITKKV	19.12
3568	P43355	MAGE1	DRB1*01:20	94	108	LESLFRAVITKKVAD	19.18
3569	P43355	MAGE1	DRB1*13:21	290	304	VRFFFPSLREAALRE	19.25
3570	P43355	MAGE1	DRB1*01:11	280	294	LEYVIKVSARVRFFF	19.27
3571	P43355	MAGE1	DRB1*16:09	93	107	ILESLFRAVITKKVA	19.39
3572	P43355	MAGE1	DRB1*01:20	279	293	VLEYVIKVSARVRFF	19.43
3573	P43355	MAGE1	DRB1*10:01	259	273	PARYEFLWGPRALAE	19.45
3574	P43355	MAGE1	DRB1*01:29	277	291	VKVLEYVIKVSARVR	19.83
3575	P43355	MAGE1	DRB1*11:03	278	292	KVLEYVIKVSARVRF	19.93
3576	P43355	MAGE1	DRB1*01:24	259	273	PARYEFLWGPRALAE	20.02
3577	P43355	MAGE1	DRB1*09:01	93	107	ILESLFRAVITKKVA	20.12
3578	P43355	MAGE1	DRB1*13:21	288	302	ARVRFFFPSLREAAL	20.12
3579	P43355	MAGE1	DRB1*01:24	260	274	ARYEFLWGPRALAET	20.15
3580	P43355	MAGE1	DPB1*33:01	269	283	RALAETSYVKVLEYV	20.19
3581	P43355	MAGE1	DPB1*71:01	269	283	RALAETSYVKVLEYV	20.19
3582	P43355	MAGE1	DRB1*03:11	279	293	VLEYVIKVSARVRFF	20.27
3583	P43355	MAGE1	DRB1*11:13	277	291	VKVLEYVIKVSARVR	20.29
3584	P43355	MAGE1	DPB1*33:01	106	120	VADLVGFLLLKYRAR	20.34
3585	P43355	MAGE1	DPB1*71:01	106	120	VADLVGFLLLKYRAR	20.34
3586	P43355	MAGE1	DRB1*11:42	109	123	LVGFLLLKYRAREPV	20.39
3587	P43355	MAGE1	DRB1*01:11	278	292	KVLEYVIKVSARVRF	20.44
3588	P43355	MAGE1	DRB1*11:13	283	297	VIKVSARVRFFFPSL	20.51
3589	P43355	MAGE1	DRB1*16:02	93	107	ILESLFRAVITKKVA	20.52
3590	P43355	MAGE1	DPB1*02:02	89	103	STSCILESLFRAVIT	20.68
3591	P43355	MAGE1	DPB1*47:01	89	103	STSCILESLFRAVIT	20.68
3592	P43355	MAGE1	DRB1*01:01	256	270	DSDPARYEFLWGPRA	20.69
3593	P43355	MAGE1	DRB1*13:21	108	122	DLVGFLLLKYRAREP	20.76
3594	P43355	MAGE1	DRB1*16:01	93	107	ILESLFRAVITKKVA	20.87
3595	P43355	MAGE1	DRB1*13:21	110	124	VGFLLLKYRAREPVT	20.90
3596	P43355	MAGE1	DRB1*13:61	279	293	VLEYVIKVSARVRFF	20.95
3597	P43355	MAGE1	DRB1*11:42	108	122	DLVGFLLLKYRAREP	20.99
3598	P43355	MAGE1	DPB1*02:02	91	105	SCILESLFRAVITKK	21.07
3599	P43355	MAGE1	DPB1*47:01	91	105	SCILESLFRAVITKK	21.07
3600	P43355	MAGE1	DPB1*02:01	91	105	SCILESLFRAVITKK	21.10
3601	P43355	MAGE1	DPB1*46:01	91	105	SCILESLFRAVITKK	21.10
3602	P43355	MAGE1	DPB1*81:01	91	105	SCILESLFRAVITKK	21.10
3603	P43355	MAGE1	DRB1*01:18	104	118	KKVADLVGFLLLKYR	21.17
3604	P43355	MAGE1	DRB1*01:20	278	292	KVLEYVIKVSARVRF	21.27

3605	P43355	MAGE1	DRB1*14:01	281	295	EYVIKVSARVRFFFP	21.27
3606	P43355	MAGE1	DRB1*14:54	281	295	EYVIKVSARVRFFFP	21.27
3607	P43355	MAGE1	DRB1*16:02	96	110	SLFRAVITKKVADLV	21.29
3608	P43355	MAGE1	DRB1*01:20	281	295	EYVIKVSARVRFFFP	21.35
3609	P43355	MAGE1	DRB1*11:04	277	291	VKVLEYVIKVSARVR	21.36
3610	P43355	MAGE1	DRB1*11:46	277	291	VKVLEYVIKVSARVR	21.36
3611	P43355	MAGE1	DRB1*11:58	277	291	VKVLEYVIKVSARVR	21.36
3612	P43355	MAGE1	DRB1*13:11	277	291	VKVLEYVIKVSARVR	21.36
3613	P43355	MAGE1	DRB1*09:01	95	109	ESLFRAVITKKVADL	21.43
3614	P43355	MAGE1	DRB1*11:42	110	124	VGFLLLKYRAREPVT	21.61
3615	P43355	MAGE1	DRB1*01:24	278	292	KVLEYVIKVSARVRF	21.68
3616	P43355	MAGE1	DRB1*01:18	256	270	DSDPARYEFLWGPRA	21.70
3617	P43355	MAGE1	DRB1*11:03	112	126	FLLLKYRAREPVTKA	21.81
3618	P43355	MAGE1	DRB1*14:01	279	293	VLEYVIKVSARVRFF	21.81
3619	P43355	MAGE1	DRB1*14:54	279	293	VLEYVIKVSARVRFF	21.81
3620	P43355	MAGE1	DRB1*01:20	185	199	DNQIMPKTGFLIIVL	21.82
3621	P43355	MAGE1	DPB1*02:02	88	102	PSTSCILESLFRAVI	21.88
3622	P43355	MAGE1	DPB1*47:01	88	102	PSTSCILESLFRAVI	21.88
3623	P43355	MAGE1	DRB1*11:01	288	302	ARVRFFFPSLREAAL	21.89
3624	P43355	MAGE1	DRB1*11:10	288	302	ARVRFFFPSLREAAL	21.89
3625	P43355	MAGE1	DRB1*11:12	288	302	ARVRFFFPSLREAAL	21.89
3626	P43355	MAGE1	DRB1*11:28	288	302	ARVRFFFPSLREAAL	21.89
3627	P43355	MAGE1	DRB1*11:29	288	302	ARVRFFFPSLREAAL	21.89
3628	P43355	MAGE1	DRB1*11:49	288	302	ARVRFFFPSLREAAL	21.89
3629	P43355	MAGE1	DRB1*11:62	288	302	ARVRFFFPSLREAAL	21.89
3630	P43355	MAGE1	DRB1*11:74	288	302	ARVRFFFPSLREAAL	21.89
3631	P43355	MAGE1	DRB1*13:05	288	302	ARVRFFFPSLREAAL	21.89
3632	P43355	MAGE1	DRB1*13:14	288	302	ARVRFFFPSLREAAL	21.89
3633	P43355	MAGE1	DRB1*13:50	288	302	ARVRFFFPSLREAAL	21.89
3634	P43355	MAGE1	DRB1*16:02	94	108	LESLFRAVITKKVAD	21.91
3635	P43355	MAGE1	DRB1*11:02	283	297	VIKVSARVRFFFPSL	21.98
3636	P43355	MAGE1	DRB1*11:65	283	297	VIKVSARVRFFFPSL	21.98
3637	P43355	MAGE1	DRB1*13:01	283	297	VIKVSARVRFFFPSL	21.98
3638	P43355	MAGE1	DRB1*09:01	92	106	CILESLFRAVITKKV	22.14
3639	P43355	MAGE1	DRB1*01:01	199	213	LVMIAMEGGHAPEEE	22.25
3640	P43355	MAGE1	DRB1*11:37	289	303	RVRFFFPSLREAALR	22.26
3641	P43355	MAGE1	DRB1*13:07	289	303	RVRFFFPSLREAALR	22.26
3642	P43355	MAGE1	DRB1*11:04	107	121	ADLVGFLLLKYRARE	22.28
3643	P43355	MAGE1	DRB1*11:46	107	121	ADLVGFLLLKYRARE	22.28
3644	P43355	MAGE1	DRB1*11:58	107	121	ADLVGFLLLKYRARE	22.28
3645	P43355	MAGE1	DRB1*13:11	107	121	ADLVGFLLLKYRARE	22.28
3646	P43355	MAGE1	DRB1*01:01	148	162	SESLQLVFGIDVKEA	22.50
3647	P43355	MAGE1	DRB1*14:01	280	294	LEYVIKVSARVRFFF	22.69

3648	P43355	MAGE1	DRB1*14:54	280	294	LEYVIKVSARVRFFF	22.69
3649	P43355	MAGE1	DRB1*01:01	103	117	TKKVADLVGFLLLKY	22.77
3650	P43355	MAGE1	DRB1*01:20	104	118	KKVADLVGFLLLKYR	22.78
3651	P43355	MAGE1	DRB1*09:01	96	110	SLFRAVITKKVADLV	22.79
3652	P43355	MAGE1	DPB1*33:01	105	119	KVADLVGFLLLKYRA	22.96
3653	P43355	MAGE1	DPB1*71:01	105	119	KVADLVGFLLLKYRA	22.96
3654	P43355	MAGE1	DRB1*03:11	282	296	YVIKVSARVRFFFPS	23.05
3655	P43355	MAGE1	DRB1*14:01	278	292	KVLEYVIKVSARVRF	23.15
3656	P43355	MAGE1	DRB1*14:54	278	292	KVLEYVIKVSARVRF	23.15
3657	P43355	MAGE1	DPB1*02:01	89	103	STSCILESLFRAVIT	23.29
3658	P43355	MAGE1	DPB1*46:01	89	103	STSCILESLFRAVIT	23.29
3659	P43355	MAGE1	DPB1*81:01	89	103	STSCILESLFRAVIT	23.29
3660	P43355	MAGE1	DRB1*01:29	92	106	CILESLFRAVITKKV	23.29
3661	P43355	MAGE1	DRB1*01:18	282	296	YVIKVSARVRFFFPS	23.34
3662	P43355	MAGE1	DRB1*11:04	282	296	YVIKVSARVRFFFPS	23.47
3663	P43355	MAGE1	DRB1*11:46	282	296	YVIKVSARVRFFFPS	23.47
3664	P43355	MAGE1	DRB1*11:58	282	296	YVIKVSARVRFFFPS	23.47
3665	P43355	MAGE1	DRB1*13:11	282	296	YVIKVSARVRFFFPS	23.47
3666	P43355	MAGE1	DPB1*33:01	266	280	WGPRALAETSYVKVL	23.48
3667	P43355	MAGE1	DPB1*71:01	266	280	WGPRALAETSYVKVL	23.48
3668	P43355	MAGE1	DRB1*11:37	290	304	VRFFFPSLREAALRE	23.67
3669	P43355	MAGE1	DRB1*13:07	290	304	VRFFFPSLREAALRE	23.67
3670	P43355	MAGE1	DRB1*13:21	107	121	ADLVGFLLLKYRARE	23.80
3671	P43355	MAGE1	DRB1*01:20	147	161	ASESLQLVFGIDVKE	23.86
3672	P43355	MAGE1	DRB1*15:15	95	109	ESLFRAVITKKVADL	23.90
3673	P43355	MAGE1	DRB1*01:20	260	274	ARYEFLWGPRALAET	23.93
3674	P43355	MAGE1	DRB1*01:18	147	161	ASESLQLVFGIDVKE	23.94
3675	P43355	MAGE1	DRB1*14:06	280	294	LEYVIKVSARVRFFF	23.98
3676	P43355	MAGE1	DRB1*14:06	281	295	EYVIKVSARVRFFFP	24.03
3677	P43355	MAGE1	DRB1*10:01	257	271	SDPARYEFLWGPRAL	24.12
3678	P43355	MAGE1	DRB1*14:06	279	293	VLEYVIKVSARVRFF	24.13
3679	P43355	MAGE1	DRB1*01:20	186	200	NQIMPKTGFLIIVLV	24.19
3680	P43355	MAGE1	DRB1*16:09	94	108	LESLFRAVITKKVAD	24.24
3681	P43355	MAGE1	DPB1*33:01	107	121	ADLVGFLLLKYRARE	24.25
3682	P43355	MAGE1	DPB1*71:01	107	121	ADLVGFLLLKYRARE	24.25
3683	P43355	MAGE1	DRB1*11:14	280	294	LEYVIKVSARVRFFF	24.27
3684	P43355	MAGE1	DRB1*13:02	280	294	LEYVIKVSARVRFFF	24.27
3685	P43355	MAGE1	DRB1*13:23	280	294	LEYVIKVSARVRFFF	24.27
3686	P43355	MAGE1	DRB1*13:97	280	294	LEYVIKVSARVRFFF	24.27
3687	P43355	MAGE1	DPB1*33:01	267	281	GPRALAETSYVKVLE	24.39
3688	P43355	MAGE1	DPB1*71:01	267	281	GPRALAETSYVKVLE	24.39
3689	P43355	MAGE1	DRB1*01:18	199	213	LVMIAMEGGHAPEEE	24.40
3690	P43355	MAGE1	DRB1*01:18	149	163	ESLQLVFGIDVKEAD	24.50

3691	P43355	MAGE1	DRB1*01:20	195	209	LIIVLVMIAMEGGHA	24.56
3692	P43355	MAGE1	DRB1*09:01	94	108	LESLFRAVITKKVAD	24.59
3693	P43355	MAGE1	DRB1*15:15	96	110	SLFRAVITKKVADLV	24.66
3694	P43355	MAGE1	DRB1*11:02	277	291	VKVLEYVIKVSARVR	24.80
3695	P43355	MAGE1	DRB1*11:03	282	296	YVIKVSARVRFFFPS	24.80
3696	P43355	MAGE1	DRB1*11:65	277	291	VKVLEYVIKVSARVR	24.80
3697	P43355	MAGE1	DRB1*13:01	277	291	VKVLEYVIKVSARVR	24.80
3698	P43355	MAGE1	DRB1*16:01	94	108	LESLFRAVITKKVAD	24.81
3699	P43355	MAGE1	DRB1*11:42	107	121	ADLVGFLLLKYRARE	24.87
3700	P43355	MAGE1	DRB1*01:18	185	199	DNQIMPKTGFLIIVL	25.03
3701	P43355	MAGE1	DRB1*01:20	258	272	DPARYEFLWGPRALA	25.10
3702	P43355	MAGE1	DRB1*14:01	282	296	YVIKVSARVRFFFPS	25.10
3703	P43355	MAGE1	DRB1*14:54	282	296	YVIKVSARVRFFFPS	25.10
3704	P43355	MAGE1	DRB1*11:08	278	292	KVLEYVIKVSARVRF	25.12
3705	P43355	MAGE1	DRB1*11:03	108	122	DLVGFLLLKYRAREP	25.13
3706	P43355	MAGE1	DRB1*01:20	149	163	ESLQLVFGIDVKEAD	25.16
3707	P43355	MAGE1	DRB1*01:11	281	295	EYVIKVSARVRFFFP	25.20
3708	P43355	MAGE1	DRB1*01:24	281	295	EYVIKVSARVRFFFP	25.20
3709	P43355	MAGE1	DRB1*11:04	111	125	GFLLLKYRAREPVTK	25.28
3710	P43355	MAGE1	DRB1*11:46	111	125	GFLLLKYRAREPVTK	25.28
3711	P43355	MAGE1	DRB1*11:58	111	125	GFLLLKYRAREPVTK	25.28
3712	P43355	MAGE1	DRB1*13:11	111	125	GFLLLKYRAREPVTK	25.28
3713	P43355	MAGE1	DPB1*02:01	88	102	PSTSCILESLFRAVI	25.38
3714	P43355	MAGE1	DPB1*46:01	88	102	PSTSCILESLFRAVI	25.38
3715	P43355	MAGE1	DPB1*81:01	88	102	PSTSCILESLFRAVI	25.38
3716	P43355	MAGE1	DRB1*16:09	97	111	LFRAVITKKVADLVG	25.47
3717	P43355	MAGE1	DRB1*01:24	257	271	SDPARYEFLWGPRAL	25.56
3718	P43355	MAGE1	DRB1*11:01	278	292	KVLEYVIKVSARVRF	25.60
3719	P43355	MAGE1	DRB1*11:10	278	292	KVLEYVIKVSARVRF	25.60
3720	P43355	MAGE1	DRB1*11:12	278	292	KVLEYVIKVSARVRF	25.60
3721	P43355	MAGE1	DRB1*11:28	278	292	KVLEYVIKVSARVRF	25.60
3722	P43355	MAGE1	DRB1*11:29	278	292	KVLEYVIKVSARVRF	25.60
3723	P43355	MAGE1	DRB1*11:49	278	292	KVLEYVIKVSARVRF	25.60
3724	P43355	MAGE1	DRB1*11:62	278	292	KVLEYVIKVSARVRF	25.60
3725	P43355	MAGE1	DRB1*11:74	278	292	KVLEYVIKVSARVRF	25.60
3726	P43355	MAGE1	DRB1*13:05	278	292	KVLEYVIKVSARVRF	25.60
3727	P43355	MAGE1	DRB1*13:14	278	292	KVLEYVIKVSARVRF	25.60
3728	P43355	MAGE1	DRB1*13:50	278	292	KVLEYVIKVSARVRF	25.60
3729	P43355	MAGE1	DRB1*13:21	111	125	GFLLLKYRAREPVTK	25.61
3730	P43355	MAGE1	DPB1*33:01	103	117	TKKVADLVGFLLLKY	25.63
3731	P43355	MAGE1	DPB1*71:01	103	117	TKKVADLVGFLLLKY	25.63
3732	P43355	MAGE1	DRB1*01:01	102	116	ITKKVADLVGFLLLK	25.64
3733	P43355	MAGE1	DRB1*14:06	278	292	KVLEYVIKVSARVRF	25.72

3734	P43355	MAGE1	DRB1*01:11	258	272	DPARYEFLWGPRALA	25.76
3735	P43355	MAGE1	DRB1*13:61	282	296	YVIKVSARVRFFFPS	25.80
3736	P43355	MAGE1	DRB1*01:29	257	271	SDPARYEFLWGPRAL	25.85
3737	P43355	MAGE1	DRB1*01:01	276	290	YVKVLEYVIKVSARV	25.86
3738	P43355	MAGE1	DRB1*10:01	97	111	LFRAVITKKVADLVG	25.87
3739	P43355	MAGE1	DRB1*11:14	279	293	VLEYVIKVSARVRFF	25.89
3740	P43355	MAGE1	DRB1*13:02	279	293	VLEYVIKVSARVRFF	25.89
3741	P43355	MAGE1	DRB1*13:23	279	293	VLEYVIKVSARVRFF	25.89
3742	P43355	MAGE1	DRB1*13:97	279	293	VLEYVIKVSARVRFF	25.89
3743	P43355	MAGE1	DRB1*01:20	22	36	ALGLVCVQAATSSSS	26.14
3744	P43355	MAGE1	DPB1*15:01	104	118	KKVADLVGFLLLKYR	26.21
3745	P43355	MAGE1	DRB1*01:29	261	275	RYEFLWGPRALAETS	26.23
3746	P43355	MAGE1	DRB1*01:20	259	273	PARYEFLWGPRALAE	26.24
3747	P43355	MAGE1	DRB1*11:01	109	123	LVGFLLLKYRAREPV	26.38
3748	P43355	MAGE1	DRB1*11:10	109	123	LVGFLLLKYRAREPV	26.38
3749	P43355	MAGE1	DRB1*11:12	109	123	LVGFLLLKYRAREPV	26.38
3750	P43355	MAGE1	DRB1*11:28	109	123	LVGFLLLKYRAREPV	26.38
3751	P43355	MAGE1	DRB1*11:29	109	123	LVGFLLLKYRAREPV	26.38
3752	P43355	MAGE1	DRB1*11:49	109	123	LVGFLLLKYRAREPV	26.38
3753	P43355	MAGE1	DRB1*11:62	109	123	LVGFLLLKYRAREPV	26.38
3754	P43355	MAGE1	DRB1*11:74	109	123	LVGFLLLKYRAREPV	26.38
3755	P43355	MAGE1	DRB1*13:05	109	123	LVGFLLLKYRAREPV	26.38
3756	P43355	MAGE1	DRB1*13:14	109	123	LVGFLLLKYRAREPV	26.38
3757	P43355	MAGE1	DRB1*13:50	109	123	LVGFLLLKYRAREPV	26.38
3758	P43355	MAGE1	DRB1*11:14	281	295	EYVIKVSARVRFFFP	26.42
3759	P43355	MAGE1	DRB1*13:02	281	295	EYVIKVSARVRFFFP	26.42
3760	P43355	MAGE1	DRB1*13:23	281	295	EYVIKVSARVRFFFP	26.42
3761	P43355	MAGE1	DRB1*13:97	281	295	EYVIKVSARVRFFFP	26.42
3762	P43355	MAGE1	DRB1*13:21	291	305	RFFFPSLREAALREE	26.46
3763	P43355	MAGE1	DPB1*02:02	87	101	GPSTSCILESLFRAV	26.48
3764	P43355	MAGE1	DPB1*47:01	87	101	GPSTSCILESLFRAV	26.48
3765	P43355	MAGE1	DRB1*14:32	283	297	VIKVSARVRFFFPSL	26.51
3766	P43355	MAGE1	DRB1*15:15	93	107	ILESLFRAVITKKVA	26.52
3767	P43355	MAGE1	DRB1*01:11	277	291	VKVLEYVIKVSARVR	26.54
3768	P43355	MAGE1	DRB1*10:01	261	275	RYEFLWGPRALAETS	26.60
3769	P43355	MAGE1	DRB1*11:84	280	294	LEYVIKVSARVRFFF	26.66
3770	P43355	MAGE1	DRB1*01:18	98	112	FRAVITKKVADLVGF	26.71
3771	P43355	MAGE1	DRB1*01:18	186	200	NQIMPKTGFLIIVLV	26.87
3772	P43355	MAGE1	DRB1*11:08	279	293	VLEYVIKVSARVRFF	27.01
3773	P43355	MAGE1	DRB1*11:84	278	292	KVLEYVIKVSARVRF	27.07
3774	P43355	MAGE1	DRB1*01:20	184	198	GDNQIMPKTGFLIIV	27.24
3775	P43355	MAGE1	DRB1*01:18	273	287	ETSYVKVLEYVIKVS	27.29
3776	P43355	MAGE1	DRB1*11:42	283	297	VIKVSARVRFFFPSL	27.30

3777	P43355	MAGE1	DRB1*11:84	279	293	VLEYVIKVSARVRFF	27.42
3778	P43355	MAGE1	DRB1*01:11	259	273	PARYEFLWGPRALAE	27.46
3779	P43355	MAGE1	DRB1*13:21	96	110	SLFRAVITKKVADLV	27.52
3780	P43355	MAGE1	DRB1*11:08	280	294	LEYVIKVSARVRFFF	27.58
3781	P43355	MAGE1	DRB1*11:42	111	125	GFLLLKYRAREPVTK	27.65
3782	P43355	MAGE1	DRB1*11:02	110	124	VGFLLLKYRAREPVT	27.92
3783	P43355	MAGE1	DRB1*11:65	110	124	VGFLLLKYRAREPVT	27.92
3784	P43355	MAGE1	DRB1*13:01	110	124	VGFLLLKYRAREPVT	27.92
3785	P43355	MAGE1	DRB1*01:11	260	274	ARYEFLWGPRALAET	28.01
3786	P43355	MAGE1	DRB1*01:20	97	111	LFRAVITKKVADLVG	28.17
3787	P43355	MAGE1	DRB1*10:01	92	106	CILESLFRAVITKKV	28.20
3788	P43355	MAGE1	DRB1*11:84	281	295	EYVIKVSARVRFFFP	28.25
3789	P43355	MAGE1	DRB1*10:01	289	303	RVRFFFPSLREAALR	28.34
3790	P43355	MAGE1	DRB1*13:21	95	109	ESLFRAVITKKVADL	28.35
3791	P43355	MAGE1	DRB1*13:61	278	292	KVLEYVIKVSARVRF	28.47
3792	P43355	MAGE1	DRB1*01:11	97	111	LFRAVITKKVADLVG	28.66
3793	P43355	MAGE1	DRB1*08:04	278	292	KVLEYVIKVSARVRF	28.78
3794	P43355	MAGE1	DRB1*01:20	92	106	CILESLFRAVITKKV	28.79
3795	P43355	MAGE1	DRB1*11:01	108	122	DLVGFLLLKYRAREP	28.93
3796	P43355	MAGE1	DRB1*11:10	108	122	DLVGFLLLKYRAREP	28.93
3797	P43355	MAGE1	DRB1*11:12	108	122	DLVGFLLLKYRAREP	28.93
3798	P43355	MAGE1	DRB1*11:28	108	122	DLVGFLLLKYRAREP	28.93
3799	P43355	MAGE1	DRB1*11:29	108	122	DLVGFLLLKYRAREP	28.93
3800	P43355	MAGE1	DRB1*11:49	108	122	DLVGFLLLKYRAREP	28.93
3801	P43355	MAGE1	DRB1*11:62	108	122	DLVGFLLLKYRAREP	28.93
3802	P43355	MAGE1	DRB1*11:74	108	122	DLVGFLLLKYRAREP	28.93
3803	P43355	MAGE1	DRB1*13:05	108	122	DLVGFLLLKYRAREP	28.93
3804	P43355	MAGE1	DRB1*13:14	108	122	DLVGFLLLKYRAREP	28.93
3805	P43355	MAGE1	DRB1*13:50	108	122	DLVGFLLLKYRAREP	28.93
3806	P43355	MAGE1	DRB1*01:24	277	291	VKVLEYVIKVSARVR	28.97
3807	P43355	MAGE1	DRB1*11:02	111	125	GFLLLKYRAREPVTK	28.99
3808	P43355	MAGE1	DRB1*11:65	111	125	GFLLLKYRAREPVTK	28.99
3809	P43355	MAGE1	DRB1*13:01	111	125	GFLLLKYRAREPVTK	28.99
3810	P43355	MAGE1	DRB1*01:24	261	275	RYEFLWGPRALAETS	29.01
3811	P43355	MAGE1	DRB1*11:01	95	109	ESLFRAVITKKVADL	29.13
3812	P43355	MAGE1	DRB1*11:10	95	109	ESLFRAVITKKVADL	29.13
3813	P43355	MAGE1	DRB1*11:12	95	109	ESLFRAVITKKVADL	29.13
3814	P43355	MAGE1	DRB1*11:28	95	109	ESLFRAVITKKVADL	29.13
3815	P43355	MAGE1	DRB1*11:29	95	109	ESLFRAVITKKVADL	29.13
3816	P43355	MAGE1	DRB1*11:49	95	109	ESLFRAVITKKVADL	29.13
3817	P43355	MAGE1	DRB1*11:62	95	109	ESLFRAVITKKVADL	29.13
3818	P43355	MAGE1	DRB1*11:74	95	109	ESLFRAVITKKVADL	29.13
3819	P43355	MAGE1	DRB1*13:05	95	109	ESLFRAVITKKVADL	29.13

3820	P43355	MAGE1	DRB1*13:14	95	109	ESLFRAVITKKVADL	29.13
3821	P43355	MAGE1	DRB1*13:50	95	109	ESLFRAVITKKVADL	29.13
3822	P43355	MAGE1	DRB1*01:18	22	36	ALGLVCVQAATSSSS	29.18
3823	P43355	MAGE1	DRB1*11:14	278	292	KVLEYVIKVSARVRF	29.26
3824	P43355	MAGE1	DRB1*13:02	278	292	KVLEYVIKVSARVRF	29.26
3825	P43355	MAGE1	DRB1*13:23	278	292	KVLEYVIKVSARVRF	29.26
3826	P43355	MAGE1	DRB1*13:97	278	292	KVLEYVIKVSARVRF	29.26
3827	P43355	MAGE1	DPB1*02:01	87	101	GPSTSCILESLFRAV	29.28
3828	P43355	MAGE1	DPB1*46:01	87	101	GPSTSCILESLFRAV	29.28
3829	P43355	MAGE1	DPB1*81:01	87	101	GPSTSCILESLFRAV	29.28
3830	P43355	MAGE1	DRB1*13:21	93	107	ILESLFRAVITKKVA	29.28
3831	P43355	MAGE1	DPB1*15:01	106	120	VADLVGFLLLKYRAR	29.33
3832	P43355	MAGE1	DRB1*01:18	274	288	TSYVKVLEYVIKVSA	29.34
3833	P43355	MAGE1	DRB1*11:01	277	291	VKVLEYVIKVSARVR	29.38
3834	P43355	MAGE1	DRB1*11:10	277	291	VKVLEYVIKVSARVR	29.38
3835	P43355	MAGE1	DRB1*11:12	277	291	VKVLEYVIKVSARVR	29.38
3836	P43355	MAGE1	DRB1*11:28	277	291	VKVLEYVIKVSARVR	29.38
3837	P43355	MAGE1	DRB1*11:29	277	291	VKVLEYVIKVSARVR	29.38
3838	P43355	MAGE1	DRB1*11:49	277	291	VKVLEYVIKVSARVR	29.38
3839	P43355	MAGE1	DRB1*11:62	277	291	VKVLEYVIKVSARVR	29.38
3840	P43355	MAGE1	DRB1*11:74	277	291	VKVLEYVIKVSARVR	29.38
3841	P43355	MAGE1	DRB1*13:05	277	291	VKVLEYVIKVSARVR	29.38
3842	P43355	MAGE1	DRB1*13:14	277	291	VKVLEYVIKVSARVR	29.38
3843	P43355	MAGE1	DRB1*13:50	277	291	VKVLEYVIKVSARVR	29.38
3844	P43355	MAGE1	DRB1*01:20	277	291	VKVLEYVIKVSARVR	29.48
3845	P43355	MAGE1	DRB1*13:21	287	301	SARVRFFFPSLREAA	29.54
3846	P43355	MAGE1	DRB1*03:11	278	292	KVLEYVIKVSARVRF	29.59
3847	P43355	MAGE1	DRB1*11:02	109	123	LVGFLLLKYRAREPV	29.64
3848	P43355	MAGE1	DRB1*11:65	109	123	LVGFLLLKYRAREPV	29.64
3849	P43355	MAGE1	DRB1*13:01	109	123	LVGFLLLKYRAREPV	29.64
3850	P43355	MAGE1	DRB1*14:12	280	294	LEYVIKVSARVRFFF	29.78
3851	P43355	MAGE1	DRB1*16:04	96	110	SLFRAVITKKVADLV	29.92
3852	P43355	MAGE1	DRB1*15:15	94	108	LESLFRAVITKKVAD	29.96
3853	P43355	MAGE1	DRB1*16:01	97	111	LFRAVITKKVADLVG	30.22
3854	P43355	MAGE1	DRB1*10:01	288	302	ARVRFFFPSLREAAL	30.32
3855	P43355	MAGE1	DRB1*01:01	22	36	ALGLVCVQAATSSSS	30.65
3856	P43355	MAGE1	DRB1*01:18	289	303	RVRFFFPSLREAALR	30.75
3857	P43355	MAGE1	DRB1*14:12	281	295	EYVIKVSARVRFFFP	30.77
3858	P43355	MAGE1	DRB1*16:04	95	109	ESLFRAVITKKVADL	30.81
3859	P43355	MAGE1	DRB1*01:24	92	106	CILESLFRAVITKKV	30.97
3860	P43355	MAGE1	DRB1*11:08	277	291	VKVLEYVIKVSARVR	30.97
3861	P43355	MAGE1	DRB1*01:20	23	37	LGLVCVQAATSSSSP	30.99
3862	P43355	MAGE1	DRB1*01:01	147	161	ASESLQLVFGIDVKE	31.38

3863	P43355	MAGE1	DRB1*11:02	112	126	FLLLKYRAREPVTKA	31.43
3864	P43355	MAGE1	DRB1*11:65	112	126	FLLLKYRAREPVTKA	31.43
3865	P43355	MAGE1	DRB1*13:01	112	126	FLLLKYRAREPVTKA	31.43
3866	P43355	MAGE1	DRB1*11:08	289	303	RVRFFFPSLREAALR	31.48
3867	P43355	MAGE1	DRB1*11:37	291	305	RFFFPSLREAALREE	31.49
3868	P43355	MAGE1	DRB1*13:07	291	305	RFFFPSLREAALREE	31.49
3869	P43355	MAGE1	DRB1*11:03	107	121	ADLVGFLLLKYRARE	31.51
3870	P43355	MAGE1	DRB1*01:18	146	160	KASESLQLVFGIDVK	31.52
3871	P43355	MAGE1	DRB1*14:12	279	293	VLEYVIKVSARVRFF	31.56
3872	P43355	MAGE1	DRB1*11:42	93	107	ILESLFRAVITKKVA	31.61
3873	P43355	MAGE1	DRB1*01:18	272	286	AETSYVKVLEYVIKV	31.69
3874	P43355	MAGE1	DRB1*11:01	110	124	VGFLLLKYRAREPVT	31.71
3875	P43355	MAGE1	DRB1*11:10	110	124	VGFLLLKYRAREPVT	31.71
3876	P43355	MAGE1	DRB1*11:12	110	124	VGFLLLKYRAREPVT	31.71
3877	P43355	MAGE1	DRB1*11:28	110	124	VGFLLLKYRAREPVT	31.71
3878	P43355	MAGE1	DRB1*11:29	110	124	VGFLLLKYRAREPVT	31.71
3879	P43355	MAGE1	DRB1*11:49	110	124	VGFLLLKYRAREPVT	31.71
3880	P43355	MAGE1	DRB1*11:62	110	124	VGFLLLKYRAREPVT	31.71
3881	P43355	MAGE1	DRB1*11:74	110	124	VGFLLLKYRAREPVT	31.71
3882	P43355	MAGE1	DRB1*13:05	110	124	VGFLLLKYRAREPVT	31.71
3883	P43355	MAGE1	DRB1*13:14	110	124	VGFLLLKYRAREPVT	31.71
3884	P43355	MAGE1	DRB1*13:50	110	124	VGFLLLKYRAREPVT	31.71
3885	P43355	MAGE1	DRB1*11:01	279	293	VLEYVIKVSARVRFF	31.77
3886	P43355	MAGE1	DRB1*11:10	279	293	VLEYVIKVSARVRFF	31.77
3887	P43355	MAGE1	DRB1*11:12	279	293	VLEYVIKVSARVRFF	31.77
3888	P43355	MAGE1	DRB1*11:28	279	293	VLEYVIKVSARVRFF	31.77
3889	P43355	MAGE1	DRB1*11:29	279	293	VLEYVIKVSARVRFF	31.77
3890	P43355	MAGE1	DRB1*11:49	279	293	VLEYVIKVSARVRFF	31.77
3891	P43355	MAGE1	DRB1*11:62	279	293	VLEYVIKVSARVRFF	31.77
3892	P43355	MAGE1	DRB1*11:74	279	293	VLEYVIKVSARVRFF	31.77
3893	P43355	MAGE1	DRB1*13:05	279	293	VLEYVIKVSARVRFF	31.77
3894	P43355	MAGE1	DRB1*13:14	279	293	VLEYVIKVSARVRFF	31.77
3895	P43355	MAGE1	DRB1*13:50	279	293	VLEYVIKVSARVRFF	31.77
3896	P43355	MAGE1	DRB1*01:01	104	118	KKVADLVGFLLLKYR	31.94
3897	P43355	MAGE1	DPB1*15:01	105	119	KVADLVGFLLLKYRA	31.97
3898	P43355	MAGE1	DRB1*01:18	184	198	GDNQIMPKTGFLIIV	32.00
3899	P43355	MAGE1	DRB1*01:20	21	35	EALGLVCVQAATSSS	32.06
3900	P43355	MAGE1	DRB1*11:01	93	107	ILESLFRAVITKKVA	32.17
3901	P43355	MAGE1	DRB1*11:10	93	107	ILESLFRAVITKKVA	32.17
3902	P43355	MAGE1	DRB1*11:12	93	107	ILESLFRAVITKKVA	32.17
3903	P43355	MAGE1	DRB1*11:28	93	107	ILESLFRAVITKKVA	32.17
3904	P43355	MAGE1	DRB1*11:29	93	107	ILESLFRAVITKKVA	32.17
3905	P43355	MAGE1	DRB1*11:49	93	107	ILESLFRAVITKKVA	32.17

3906	P43355	MAGE1	DRB1*11:62	93	107	ILESLFRAVITKKVA	32.17
3907	P43355	MAGE1	DRB1*11:74	93	107	ILESLFRAVITKKVA	32.17
3908	P43355	MAGE1	DRB1*13:05	93	107	ILESLFRAVITKKVA	32.17
3909	P43355	MAGE1	DRB1*13:14	93	107	ILESLFRAVITKKVA	32.17
3910	P43355	MAGE1	DRB1*13:50	93	107	ILESLFRAVITKKVA	32.17
3911	P43355	MAGE1	DRB1*11:01	96	110	SLFRAVITKKVADLV	32.18
3912	P43355	MAGE1	DRB1*11:10	96	110	SLFRAVITKKVADLV	32.18
3913	P43355	MAGE1	DRB1*11:12	96	110	SLFRAVITKKVADLV	32.18
3914	P43355	MAGE1	DRB1*11:28	96	110	SLFRAVITKKVADLV	32.18
3915	P43355	MAGE1	DRB1*11:29	96	110	SLFRAVITKKVADLV	32.18
3916	P43355	MAGE1	DRB1*11:49	96	110	SLFRAVITKKVADLV	32.18
3917	P43355	MAGE1	DRB1*11:62	96	110	SLFRAVITKKVADLV	32.18
3918	P43355	MAGE1	DRB1*11:74	96	110	SLFRAVITKKVADLV	32.18
3919	P43355	MAGE1	DRB1*13:05	96	110	SLFRAVITKKVADLV	32.18
3920	P43355	MAGE1	DRB1*13:14	96	110	SLFRAVITKKVADLV	32.18
3921	P43355	MAGE1	DRB1*13:50	96	110	SLFRAVITKKVADLV	32.18
3922	P43355	MAGE1	DRB1*14:06	282	296	YVIKVSARVRFFFPS	32.54
3923	P43355	MAGE1	DRB1*01:20	288	302	ARVRFFFPSLREAAL	32.67
3924	P43355	MAGE1	DRB1*16:09	280	294	LEYVIKVSARVRFFF	32.69
3925	P43355	MAGE1	DRB1*03:15	281	295	EYVIKVSARVRFFFP	32.70
3926	P43355	MAGE1	DRB1*13:96	280	294	LEYVIKVSARVRFFF	32.92
3927	P43355	MAGE1	DRB1*11:04	289	303	RVRFFFPSLREAALR	32.94
3928	P43355	MAGE1	DRB1*11:46	289	303	RVRFFFPSLREAALR	32.94
3929	P43355	MAGE1	DRB1*11:58	289	303	RVRFFFPSLREAALR	32.94
3930	P43355	MAGE1	DRB1*13:11	289	303	RVRFFFPSLREAALR	32.94
3931	P43355	MAGE1	DRB1*13:96	279	293	VLEYVIKVSARVRFF	33.05
3932	P43355	MAGE1	DRB1*01:20	261	275	RYEFLWGPRALAETS	33.24
3933	P43355	MAGE1	DRB1*11:08	290	304	VRFFFPSLREAALRE	33.35
3934	P43355	MAGE1	DRB1*01:20	287	301	SARVRFFFPSLREAA	33.55
3935	P43355	MAGE1	DRB1*01:01	149	163	ESLQLVFGIDVKEAD	33.58
3936	P43355	MAGE1	DRB1*01:18	21	35	EALGLVCVQAATSSS	33.61
3937	P43355	MAGE1	DPB1*33:01	108	122	DLVGFLLLKYRAREP	33.68
3938	P43355	MAGE1	DPB1*71:01	108	122	DLVGFLLLKYRAREP	33.68
3939	P43355	MAGE1	DRB1*01:18	101	115	VITKKVADLVGFLLL	33.71
3940	P43355	MAGE1	DRB1*01:01	282	296	YVIKVSARVRFFFPS	33.79
3941	P43355	MAGE1	DRB1*01:20	282	296	YVIKVSARVRFFFPS	33.79
3942	P43355	MAGE1	DRB1*08:04	279	293	VLEYVIKVSARVRFF	33.80
3943	P43355	MAGE1	DRB1*11:01	260	274	ARYEFLWGPRALAET	33.84
3944	P43355	MAGE1	DRB1*11:10	260	274	ARYEFLWGPRALAET	33.84
3945	P43355	MAGE1	DRB1*11:12	260	274	ARYEFLWGPRALAET	33.84
3946	P43355	MAGE1	DRB1*11:28	260	274	ARYEFLWGPRALAET	33.84
3947	P43355	MAGE1	DRB1*11:29	260	274	ARYEFLWGPRALAET	33.84
3948	P43355	MAGE1	DRB1*11:49	260	274	ARYEFLWGPRALAET	33.84

3949	P43355	MAGE1	DRB1*11:62	260	274	ARYEFLWGPRALAET	33.84
3950	P43355	MAGE1	DRB1*11:74	260	274	ARYEFLWGPRALAET	33.84
3951	P43355	MAGE1	DRB1*13:05	260	274	ARYEFLWGPRALAET	33.84
3952	P43355	MAGE1	DRB1*13:14	260	274	ARYEFLWGPRALAET	33.84
3953	P43355	MAGE1	DRB1*13:50	260	274	ARYEFLWGPRALAET	33.84
3954	P43355	MAGE1	DRB1*11:04	112	126	FLLLKYRAREPVTKA	33.91
3955	P43355	MAGE1	DRB1*11:46	112	126	FLLLKYRAREPVTKA	33.91
3956	P43355	MAGE1	DRB1*11:58	112	126	FLLLKYRAREPVTKA	33.91
3957	P43355	MAGE1	DRB1*13:11	112	126	FLLLKYRAREPVTKA	33.91
3958	P43355	MAGE1	DRB1*11:42	106	120	VADLVGFLLLKYRAR	33.92
3959	P43355	MAGE1	DRB1*11:01	280	294	LEYVIKVSARVRFFF	34.02
3960	P43355	MAGE1	DRB1*11:10	280	294	LEYVIKVSARVRFFF	34.02
3961	P43355	MAGE1	DRB1*11:12	280	294	LEYVIKVSARVRFFF	34.02
3962	P43355	MAGE1	DRB1*11:28	280	294	LEYVIKVSARVRFFF	34.02
3963	P43355	MAGE1	DRB1*11:29	280	294	LEYVIKVSARVRFFF	34.02
3964	P43355	MAGE1	DRB1*11:49	280	294	LEYVIKVSARVRFFF	34.02
3965	P43355	MAGE1	DRB1*11:62	280	294	LEYVIKVSARVRFFF	34.02
3966	P43355	MAGE1	DRB1*11:74	280	294	LEYVIKVSARVRFFF	34.02
3967	P43355	MAGE1	DRB1*13:05	280	294	LEYVIKVSARVRFFF	34.02
3968	P43355	MAGE1	DRB1*13:14	280	294	LEYVIKVSARVRFFF	34.02
3969	P43355	MAGE1	DRB1*13:50	280	294	LEYVIKVSARVRFFF	34.02
3970	P43355	MAGE1	DRB1*11:01	107	121	ADLVGFLLLKYRARE	34.07
3971	P43355	MAGE1	DRB1*11:08	95	109	ESLFRAVITKKVADL	34.07
3972	P43355	MAGE1	DRB1*11:10	107	121	ADLVGFLLLKYRARE	34.07
3973	P43355	MAGE1	DRB1*11:12	107	121	ADLVGFLLLKYRARE	34.07
3974	P43355	MAGE1	DRB1*11:28	107	121	ADLVGFLLLKYRARE	34.07
3975	P43355	MAGE1	DRB1*11:29	107	121	ADLVGFLLLKYRARE	34.07
3976	P43355	MAGE1	DRB1*11:49	107	121	ADLVGFLLLKYRARE	34.07
3977	P43355	MAGE1	DRB1*11:62	107	121	ADLVGFLLLKYRARE	34.07
3978	P43355	MAGE1	DRB1*11:74	107	121	ADLVGFLLLKYRARE	34.07
3979	P43355	MAGE1	DRB1*13:05	107	121	ADLVGFLLLKYRARE	34.07
3980	P43355	MAGE1	DRB1*13:14	107	121	ADLVGFLLLKYRARE	34.07
3981	P43355	MAGE1	DRB1*13:50	107	121	ADLVGFLLLKYRARE	34.07
3982	P43355	MAGE1	DRB1*11:08	281	295	EYVIKVSARVRFFFP	34.14
3983	P43355	MAGE1	DRB1*16:04	93	107	ILESLFRAVITKKVA	34.19
3984	P43355	MAGE1	DPB1*33:01	240	254	TQDLVQEKYLEYRQV	34.55
3985	P43355	MAGE1	DPB1*71:01	240	254	TQDLVQEKYLEYRQV	34.55
3986	P43355	MAGE1	DRB1*01:18	23	37	LGLVCVQAATSSSSP	34.58
3987	P43355	MAGE1	DRB1*01:01	289	303	RVRFFFPSLREAALR	34.59
3988	P43355	MAGE1	DRB1*08:04	280	294	LEYVIKVSARVRFFF	34.64
3989	P43355	MAGE1	DRB1*11:13	276	290	YVKVLEYVIKVSARV	34.69
3990	P43355	MAGE1	DRB1*11:01	261	275	RYEFLWGPRALAETS	34.81
3991	P43355	MAGE1	DRB1*11:10	261	275	RYEFLWGPRALAETS	34.81

3992	P43355	MAGE1	DRB1*11:12	261	275	RYEFLWGPRALAETS	34.81
3993	P43355	MAGE1	DRB1*11:28	261	275	RYEFLWGPRALAETS	34.81
3994	P43355	MAGE1	DRB1*11:29	261	275	RYEFLWGPRALAETS	34.81
3995	P43355	MAGE1	DRB1*11:49	261	275	RYEFLWGPRALAETS	34.81
3996	P43355	MAGE1	DRB1*11:62	261	275	RYEFLWGPRALAETS	34.81
3997	P43355	MAGE1	DRB1*11:74	261	275	RYEFLWGPRALAETS	34.81
3998	P43355	MAGE1	DRB1*13:05	261	275	RYEFLWGPRALAETS	34.81
3999	P43355	MAGE1	DRB1*13:14	261	275	RYEFLWGPRALAETS	34.81
4000	P43355	MAGE1	DRB1*13:50	261	275	RYEFLWGPRALAETS	34.81
4001	P43355	MAGE1	DPB1*33:01	286	300	VSARVRFFFPSLREA	34.91
4002	P43355	MAGE1	DPB1*71:01	286	300	VSARVRFFFPSLREA	34.91
4003	P43355	MAGE1	DRB1*16:09	279	293	VLEYVIKVSARVRFF	34.95
4004	P43355	MAGE1	DRB1*09:01	97	111	LFRAVITKKVADLVG	35.07
4005	P43355	MAGE1	DRB1*11:42	96	110	SLFRAVITKKVADLV	35.07
4006	P43355	MAGE1	DRB1*14:12	278	292	KVLEYVIKVSARVRF	35.20
4007	P43355	MAGE1	DRB1*11:37	288	302	ARVRFFFPSLREAAL	35.21
4008	P43355	MAGE1	DRB1*13:07	288	302	ARVRFFFPSLREAAL	35.21
4009	P43355	MAGE1	DRB1*16:09	278	292	KVLEYVIKVSARVRF	35.31
4010	P43355	MAGE1	DRB1*01:01	185	199	DNQIMPKTGFLIIVL	35.32
4011	P43355	MAGE1	DRB1*01:20	200	214	VMIAMEGGHAPEEEI	35.40
4012	P43355	MAGE1	DPB1*04:01	90	104	TSCILESLFRAVITK	35.53
4013	P43355	MAGE1	DPB1*126:01	90	104	TSCILESLFRAVITK	35.53
4014	P43355	MAGE1	DPB1*23:01	90	104	TSCILESLFRAVITK	35.53
4015	P43355	MAGE1	DPB1*39:01	90	104	TSCILESLFRAVITK	35.53
4016	P43355	MAGE1	DRB1*11:42	95	109	ESLFRAVITKKVADL	35.53
4017	P43355	MAGE1	DRB1*01:01	21	35	EALGLVCVQAATSSS	35.59
4018	P43355	MAGE1	DRB1*01:02	197	211	IVLVMIAMEGGHAPE	35.73
4019	P43355	MAGE1	DRB1*03:15	279	293	VLEYVIKVSARVRFF	35.85
4020	P43355	MAGE1	DRB1*01:18	195	209	LIIVLVMIAMEGGHA	35.89
4021	P43355	MAGE1	DRB1*01:18	290	304	VRFFFPSLREAALRE	35.95
4022	P43355	MAGE1	DRB1*01:01	23	37	LGLVCVQAATSSSSP	35.99
4023	P43355	MAGE1	DPB1*15:01	103	117	TKKVADLVGFLLLKY	36.01
4024	P43355	MAGE1	DRB1*03:15	280	294	LEYVIKVSARVRFFF	36.01
4025	P43355	MAGE1	DRB1*11:03	277	291	VKVLEYVIKVSARVR	36.03
4026	P43355	MAGE1	DRB1*14:32	277	291	VKVLEYVIKVSARVR	36.10
4027	P43355	MAGE1	DRB1*11:42	276	290	YVKVLEYVIKVSARV	36.14
4028	P43355	MAGE1	DRB1*14:01	283	297	VIKVSARVRFFFPSL	36.15
4029	P43355	MAGE1	DRB1*14:54	283	297	VIKVSARVRFFFPSL	36.15
4030	P43355	MAGE1	DRB1*01:20	289	303	RVRFFFPSLREAALR	36.17
4031	P43355	MAGE1	DRB1*11:04	93	107	ILESLFRAVITKKVA	36.18
4032	P43355	MAGE1	DRB1*11:46	93	107	ILESLFRAVITKKVA	36.18
4033	P43355	MAGE1	DRB1*11:58	93	107	ILESLFRAVITKKVA	36.18
4034	P43355	MAGE1	DRB1*13:11	93	107	ILESLFRAVITKKVA	36.18

4035	P43355	MAGE1	DRB1*01:20	101	115	VITKKVADLVGFLLL	36.19
4036	P43355	MAGE1	DRB1*08:04	281	295	EYVIKVSARVRFFFP	36.25
4037	P43355	MAGE1	DRB1*03:01	281	295	EYVIKVSARVRFFFP	36.32
4038	P43355	MAGE1	DRB1*03:04	281	295	EYVIKVSARVRFFFP	36.32
4039	P43355	MAGE1	DRB1*03:13	281	295	EYVIKVSARVRFFFP	36.32
4040	P43355	MAGE1	DRB1*01:20	257	271	SDPARYEFLWGPRAL	36.34
4041	P43355	MAGE1	DRB1*07:01	280	294	LEYVIKVSARVRFFF	36.39
4042	P43355	MAGE1	DRB1*16:09	92	106	CILESLFRAVITKKV	36.39
4043	P43355	MAGE1	DRB1*11:13	109	123	LVGFLLLKYRAREPV	36.51
4044	P43355	MAGE1	DRB1*11:04	106	120	VADLVGFLLLKYRAR	36.58
4045	P43355	MAGE1	DRB1*11:46	106	120	VADLVGFLLLKYRAR	36.58
4046	P43355	MAGE1	DRB1*11:58	106	120	VADLVGFLLLKYRAR	36.58
4047	P43355	MAGE1	DRB1*13:11	106	120	VADLVGFLLLKYRAR	36.58
4048	P43355	MAGE1	DRB1*16:09	258	272	DPARYEFLWGPRALA	36.78
4049	P43355	MAGE1	DRB1*01:01	195	209	LIIVLVMIAMEGGHA	36.83
4050	P43355	MAGE1	DRB1*13:21	94	108	LESLFRAVITKKVAD	36.95
4051	P43355	MAGE1	DRB1*13:21	278	292	KVLEYVIKVSARVRF	37.00
4052	P43355	MAGE1	DRB1*01:20	146	160	KASESLQLVFGIDVK	37.02
4053	P43355	MAGE1	DRB1*13:21	112	126	FLLLKYRAREPVTKA	37.04
4054	P43355	MAGE1	DRB1*01:11	92	106	CILESLFRAVITKKV	37.22
4055	P43355	MAGE1	DPB1*33:01	241	255	QDLVQEKYLEYRQVP	37.23
4056	P43355	MAGE1	DPB1*71:01	241	255	QDLVQEKYLEYRQVP	37.23
4057	P43355	MAGE1	DRB1*14:04	281	295	EYVIKVSARVRFFFP	37.23
4058	P43355	MAGE1	DRB1*11:01	94	108	LESLFRAVITKKVAD	37.28
4059	P43355	MAGE1	DRB1*11:10	94	108	LESLFRAVITKKVAD	37.28
4060	P43355	MAGE1	DRB1*11:12	94	108	LESLFRAVITKKVAD	37.28
4061	P43355	MAGE1	DRB1*11:28	94	108	LESLFRAVITKKVAD	37.28
4062	P43355	MAGE1	DRB1*11:29	94	108	LESLFRAVITKKVAD	37.28
4063	P43355	MAGE1	DRB1*11:49	94	108	LESLFRAVITKKVAD	37.28
4064	P43355	MAGE1	DRB1*11:62	94	108	LESLFRAVITKKVAD	37.28
4065	P43355	MAGE1	DRB1*11:74	94	108	LESLFRAVITKKVAD	37.28
4066	P43355	MAGE1	DRB1*13:05	94	108	LESLFRAVITKKVAD	37.28
4067	P43355	MAGE1	DRB1*13:14	94	108	LESLFRAVITKKVAD	37.28
4068	P43355	MAGE1	DRB1*13:50	94	108	LESLFRAVITKKVAD	37.28
4069	P43355	MAGE1	DRB1*13:21	97	111	LFRAVITKKVADLVG	37.44
4070	P43355	MAGE1	DRB1*12:03	280	294	LEYVIKVSARVRFFF	37.49
4071	P43355	MAGE1	DRB1*11:08	93	107	ILESLFRAVITKKVA	37.50
4072	P43355	MAGE1	DPB1*15:01	107	121	ADLVGFLLLKYRARE	37.55
4073	P43355	MAGE1	DRB1*08:04	277	291	VKVLEYVIKVSARVR	37.55
4074	P43355	MAGE1	DRB1*16:02	278	292	KVLEYVIKVSARVRF	37.55
4075	P43355	MAGE1	DRB1*10:01	287	301	SARVRFFFPSLREAA	37.62
4076	P43355	MAGE1	DRB1*11:84	282	296	YVIKVSARVRFFFPS	37.73
4077	P43355	MAGE1	DRB1*01:02	198	212	VLVMIAMEGGHAPEE	37.74

4078	P43355	MAGE1	DRB1*11:01	259	273	PARYEFLWGPRALAE	37.79
4079	P43355	MAGE1	DRB1*11:10	259	273	PARYEFLWGPRALAE	37.79
4080	P43355	MAGE1	DRB1*11:12	259	273	PARYEFLWGPRALAE	37.79
4081	P43355	MAGE1	DRB1*11:28	259	273	PARYEFLWGPRALAE	37.79
4082	P43355	MAGE1	DRB1*11:29	259	273	PARYEFLWGPRALAE	37.79
4083	P43355	MAGE1	DRB1*11:49	259	273	PARYEFLWGPRALAE	37.79
4084	P43355	MAGE1	DRB1*11:62	259	273	PARYEFLWGPRALAE	37.79
4085	P43355	MAGE1	DRB1*11:74	259	273	PARYEFLWGPRALAE	37.79
4086	P43355	MAGE1	DRB1*13:05	259	273	PARYEFLWGPRALAE	37.79
4087	P43355	MAGE1	DRB1*13:14	259	273	PARYEFLWGPRALAE	37.79
4088	P43355	MAGE1	DRB1*13:50	259	273	PARYEFLWGPRALAE	37.79
4089	P43355	MAGE1	DRB1*10:01	290	304	VRFFFPSLREAALRE	37.99
4090	P43355	MAGE1	DRB1*14:38	280	294	LEYVIKVSARVRFFF	38.02
4091	P43355	MAGE1	DRB1*11:42	112	126	FLLLKYRAREPVTKA	38.03
4092	P43355	MAGE1	DRB1*15:15	97	111	LFRAVITKKVADLVG	38.05
4093	P43355	MAGE1	DPB1*02:02	92	106	CILESLFRAVITKKV	38.06
4094	P43355	MAGE1	DPB1*47:01	92	106	CILESLFRAVITKKV	38.06
4095	P43355	MAGE1	DRB1*16:09	260	274	ARYEFLWGPRALAET	38.11
4096	P43355	MAGE1	DRB1*11:42	94	108	LESLFRAVITKKVAD	38.13
4097	P43355	MAGE1	DRB1*11:08	94	108	LESLFRAVITKKVAD	38.14
4098	P43355	MAGE1	DRB1*01:01	186	200	NQIMPKTGFLIIVLV	38.15
4099	P43355	MAGE1	DRB1*16:02	92	106	CILESLFRAVITKKV	38.27
4100	P43355	MAGE1	DRB1*14:04	280	294	LEYVIKVSARVRFFF	38.32
4101	P43355	MAGE1	DRB1*11:04	290	304	VRFFFPSLREAALRE	38.38
4102	P43355	MAGE1	DRB1*11:46	290	304	VRFFFPSLREAALRE	38.38
4103	P43355	MAGE1	DRB1*11:58	290	304	VRFFFPSLREAALRE	38.38
4104	P43355	MAGE1	DRB1*13:11	290	304	VRFFFPSLREAALRE	38.38
4105	P43355	MAGE1	DRB1*13:96	281	295	EYVIKVSARVRFFFP	38.38
4106	P43355	MAGE1	DRB1*01:18	288	302	ARVRFFFPSLREAAL	38.57
4107	P43355	MAGE1	DRB1*01:20	183	197	LGDNQIMPKTGFLII	38.57
4108	P43355	MAGE1	DRB1*07:01	279	293	VLEYVIKVSARVRFF	38.63
4109	P43355	MAGE1	DRB1*12:03	281	295	EYVIKVSARVRFFFP	38.64
4110	P43355	MAGE1	DRB1*01:18	275	289	SYVKVLEYVIKVSAR	38.68
4111	P43355	MAGE1	DRB1*15:01	287	301	SARVRFFFPSLREAA	38.73
4112	P43355	MAGE1	DRB1*15:06	287	301	SARVRFFFPSLREAA	38.73
4113	P43355	MAGE1	DRB1*16:09	259	273	PARYEFLWGPRALAE	38.73
4114	P43355	MAGE1	DRB1*07:01	278	292	KVLEYVIKVSARVRF	38.80
4115	P43355	MAGE1	DRB1*01:11	257	271	SDPARYEFLWGPRAL	38.87
4116	P43355	MAGE1	DRB1*11:13	107	121	ADLVGFLLLKYRARE	38.99
4117	P43355	MAGE1	DPB1*33:01	288	302	ARVRFFFPSLREAAL	39.02
4118	P43355	MAGE1	DPB1*71:01	288	302	ARVRFFFPSLREAAL	39.02
4119	P43355	MAGE1	DRB1*01:29	197	211	IVLVMIAMEGGHAPE	39.03
4120	P43355	MAGE1	DRB1*11:84	277	291	VKVLEYVIKVSARVR	39.08

4121	P43355	MAGE1	DRB1*01:01	290	304	VRFFFPSLREAALRE	39.10
4122	P43355	MAGE1	DRB1*11:03	113	127	LLLKYRAREPVTKAE	39.10
4123	P43355	MAGE1	DRB1*01:18	262	276	YEFLWGPRALAETSY	39.13
4124	P43355	MAGE1	DRB1*11:84	109	123	LVGFLLLKYRAREPV	39.21
4125	P43355	MAGE1	DRB1*11:19	279	293	VLEYVIKVSARVRFF	39.25
4126	P43355	MAGE1	DRB1*11:42	275	289	SYVKVLEYVIKVSAR	39.26
4127	P43355	MAGE1	DRB1*16:01	258	272	DPARYEFLWGPRALA	39.27
4128	P43355	MAGE1	DPB1*33:01	287	301	SARVRFFFPSLREAA	39.28
4129	P43355	MAGE1	DPB1*71:01	287	301	SARVRFFFPSLREAA	39.28
4130	P43355	MAGE1	DRB1*16:02	97	111	LFRAVITKKVADLVG	39.28
4131	P43355	MAGE1	DRB1*14:04	279	293	VLEYVIKVSARVRFF	39.30
4132	P43355	MAGE1	DRB1*11:13	108	122	DLVGFLLLKYRAREP	39.33
4133	P43355	MAGE1	DRB1*01:20	109	123	LVGFLLLKYRAREPV	39.34
4134	P43355	MAGE1	DRB1*13:21	279	293	VLEYVIKVSARVRFF	39.34
4135	P43355	MAGE1	DRB1*12:03	279	293	VLEYVIKVSARVRFF	39.35
4136	P43355	MAGE1	DRB1*14:38	281	295	EYVIKVSARVRFFFP	39.37
4137	P43355	MAGE1	DRB1*15:15	258	272	DPARYEFLWGPRALA	39.43
4138	P43355	MAGE1	DRB1*03:01	280	294	LEYVIKVSARVRFFF	39.50
4139	P43355	MAGE1	DRB1*03:04	280	294	LEYVIKVSARVRFFF	39.50
4140	P43355	MAGE1	DRB1*03:13	280	294	LEYVIKVSARVRFFF	39.50
4141	P43355	MAGE1	DRB1*10:01	22	36	ALGLVCVQAATSSSS	39.53
4142	P43355	MAGE1	DRB1*11:01	281	295	EYVIKVSARVRFFFP	39.57
4143	P43355	MAGE1	DRB1*11:10	281	295	EYVIKVSARVRFFFP	39.57
4144	P43355	MAGE1	DRB1*11:12	281	295	EYVIKVSARVRFFFP	39.57
4145	P43355	MAGE1	DRB1*11:28	281	295	EYVIKVSARVRFFFP	39.57
4146	P43355	MAGE1	DRB1*11:29	281	295	EYVIKVSARVRFFFP	39.57
4147	P43355	MAGE1	DRB1*11:42	92	106	CILESLFRAVITKKV	39.57
4148	P43355	MAGE1	DRB1*11:49	281	295	EYVIKVSARVRFFFP	39.57
4149	P43355	MAGE1	DRB1*11:62	281	295	EYVIKVSARVRFFFP	39.57
4150	P43355	MAGE1	DRB1*11:74	281	295	EYVIKVSARVRFFFP	39.57
4151	P43355	MAGE1	DRB1*13:05	281	295	EYVIKVSARVRFFFP	39.57
4152	P43355	MAGE1	DRB1*13:14	281	295	EYVIKVSARVRFFFP	39.57
4153	P43355	MAGE1	DRB1*13:50	281	295	EYVIKVSARVRFFFP	39.57
4154	P43355	MAGE1	DRB1*16:02	279	293	VLEYVIKVSARVRFF	39.58
4155	P43355	MAGE1	DRB1*01:20	286	300	VSARVRFFFPSLREA	39.61
4156	P43355	MAGE1	DPB1*02:01	92	106	CILESLFRAVITKKV	39.93
4157	P43355	MAGE1	DPB1*46:01	92	106	CILESLFRAVITKKV	39.93
4158	P43355	MAGE1	DPB1*81:01	92	106	CILESLFRAVITKKV	39.93
4159	P43355	MAGE1	DRB1*01:01	98	112	FRAVITKKVADLVGF	39.93
4160	P43355	MAGE1	DRB1*11:42	289	303	RVRFFFPSLREAALR	39.93
4161	P43355	MAGE1	DRB1*01:02	196	210	IIVLVMIAMEGGHAP	40.00
4162	P43355	MAGE1	DRB1*11:19	278	292	KVLEYVIKVSARVRF	40.05
4163	P43355	MAGE1	DRB1*15:01	280	294	LEYVIKVSARVRFFF	40.10

4164	P43355	MAGE1	DRB1*15:06	280	294	LEYVIKVSARVRFFF	40.10
4165	P43355	MAGE1	DRB1*16:01	92	106	CILESLFRAVITKKV	40.12
4166	P43355	MAGE1	DRB1*13:21	281	295	EYVIKVSARVRFFFP	40.17
4167	P43355	MAGE1	DRB1*16:05	95	109	ESLFRAVITKKVADL	40.22
4168	P43355	MAGE1	DRB1*01:18	109	123	LVGFLLLKYRAREPV	40.27
4169	P43355	MAGE1	DRB1*16:02	280	294	LEYVIKVSARVRFFF	40.37
4170	P43355	MAGE1	DRB1*12:16	93	107	ILESLFRAVITKKVA	40.39
4171	P43355	MAGE1	DRB1*01:20	100	114	AVITKKVADLVGFLL	40.41
4172	P43355	MAGE1	DRB1*14:06	277	291	VKVLEYVIKVSARVR	40.43
4173	P43355	MAGE1	DPB1*33:01	285	299	KVSARVRFFFPSLRE	40.46
4174	P43355	MAGE1	DPB1*71:01	285	299	KVSARVRFFFPSLRE	40.46
4175	P43355	MAGE1	DRB1*11:13	275	289	SYVKVLEYVIKVSAR	40.48
4176	P43355	MAGE1	DRB1*14:38	279	293	VLEYVIKVSARVRFF	40.51
4177	P43355	MAGE1	DRB1*11:02	107	121	ADLVGFLLLKYRARE	40.53
4178	P43355	MAGE1	DRB1*11:65	107	121	ADLVGFLLLKYRARE	40.53
4179	P43355	MAGE1	DRB1*13:01	107	121	ADLVGFLLLKYRARE	40.53
4180	P43355	MAGE1	DRB1*01:20	187	201	QIMPKTGFLIIVLVM	40.75
4181	P43355	MAGE1	DRB1*10:01	278	292	KVLEYVIKVSARVRF	40.75
4182	P43355	MAGE1	DRB1*08:30	279	293	VLEYVIKVSARVRFF	40.81
4183	P43355	MAGE1	DRB1*16:01	259	273	PARYEFLWGPRALAE	40.85
4184	P43355	MAGE1	DRB1*11:02	284	298	IKVSARVRFFFPSLR	40.89
4185	P43355	MAGE1	DRB1*11:65	284	298	IKVSARVRFFFPSLR	40.89
4186	P43355	MAGE1	DRB1*13:01	284	298	IKVSARVRFFFPSLR	40.89
4187	P43355	MAGE1	DRB1*12:03	106	120	VADLVGFLLLKYRAR	40.91
4188	P43355	MAGE1	DRB1*03:11	283	297	VIKVSARVRFFFPSL	40.94
4189	P43355	MAGE1	DRB1*11:42	288	302	ARVRFFFPSLREAAL	41.02
4190	P43355	MAGE1	DRB1*16:02	258	272	DPARYEFLWGPRALA	41.06
4191	P43355	MAGE1	DRB1*11:03	289	303	RVRFFFPSLREAALR	41.13
4192	P43355	MAGE1	DRB1*16:04	94	108	LESLFRAVITKKVAD	41.16
4193	P43355	MAGE1	DRB1*01:29	198	212	VLVMIAMEGGHAPEE	41.18
4194	P43355	MAGE1	DRB1*13:21	280	294	LEYVIKVSARVRFFF	41.23
4195	P43355	MAGE1	DRB1*12:03	107	121	ADLVGFLLLKYRARE	41.32
4196	P43355	MAGE1	DRB1*11:08	96	110	SLFRAVITKKVADLV	41.43
4197	P43355	MAGE1	DRB1*01:20	20	34	QEALGLVCVQAATSS	41.47
4198	P43355	MAGE1	DRB1*11:13	93	107	ILESLFRAVITKKVA	41.60
4199	P43355	MAGE1	DPB1*02:02	268	282	PRALAETSYVKVLEY	41.63
4200	P43355	MAGE1	DPB1*47:01	268	282	PRALAETSYVKVLEY	41.63
4201	P43355	MAGE1	DRB1*13:96	278	292	KVLEYVIKVSARVRF	41.64
4202	P43355	MAGE1	DPB1*33:01	93	107	ILESLFRAVITKKVA	41.69
4203	P43355	MAGE1	DPB1*71:01	93	107	ILESLFRAVITKKVA	41.69
4204	P43355	MAGE1	DRB1*11:42	91	105	SCILESLFRAVITKK	41.73
4205	P43355	MAGE1	DRB1*10:01	21	35	EALGLVCVQAATSSS	41.78
4206	P43355	MAGE1	DPB1*33:01	270	284	ALAETSYVKVLEYVI	42.04

4207	P43355	MAGE1	DPB1*71:01	270	284	ALAETSYVKVLEYVI	42.04
4208	P43355	MAGE1	DRB1*15:03	287	301	SARVRFFFPSLREAA	42.04
4209	P43355	MAGE1	DRB1*11:13	110	124	VGFLLLKYRAREPVT	42.09
4210	P43355	MAGE1	DRB1*14:04	278	292	KVLEYVIKVSARVRF	42.10
4211	P43355	MAGE1	DRB1*01:24	148	162	SESLQLVFGIDVKEA	42.19
4212	P43355	MAGE1	DRB1*16:01	260	274	ARYEFLWGPRALAET	42.20
4213	P43355	MAGE1	DRB1*15:01	286	300	VSARVRFFFPSLREA	42.26
4214	P43355	MAGE1	DRB1*15:06	286	300	VSARVRFFFPSLREA	42.26
4215	P43355	MAGE1	DRB1*15:15	260	274	ARYEFLWGPRALAET	42.30
4216	P43355	MAGE1	DRB1*01:01	288	302	ARVRFFFPSLREAAL	42.38
4217	P43355	MAGE1	DRB1*11:03	288	302	ARVRFFFPSLREAAL	42.45
4218	P43355	MAGE1	DRB1*01:18	20	34	QEALGLVCVQAATSS	42.54
4219	P43355	MAGE1	DRB1*11:42	287	301	SARVRFFFPSLREAA	42.54
4220	P43355	MAGE1	DRB1*11:04	288	302	ARVRFFFPSLREAAL	42.61
4221	P43355	MAGE1	DRB1*11:46	288	302	ARVRFFFPSLREAAL	42.61
4222	P43355	MAGE1	DRB1*11:58	288	302	ARVRFFFPSLREAAL	42.61
4223	P43355	MAGE1	DRB1*13:11	288	302	ARVRFFFPSLREAAL	42.61
4224	P43355	MAGE1	DRB1*14:32	91	105	SCILESLFRAVITKK	42.62
4225	P43355	MAGE1	DRB1*01:24	103	117	TKKVADLVGFLLLKY	42.65
4226	P43355	MAGE1	DRB1*13:21	92	106	CILESLFRAVITKKV	42.81
4227	P43355	MAGE1	DRB1*11:02	108	122	DLVGFLLLKYRAREP	42.83
4228	P43355	MAGE1	DRB1*11:65	108	122	DLVGFLLLKYRAREP	42.83
4229	P43355	MAGE1	DRB1*13:01	108	122	DLVGFLLLKYRAREP	42.83
4230	P43355	MAGE1	DRB1*11:27	289	303	RVRFFFPSLREAALR	42.89
4231	P43355	MAGE1	DRB1*16:09	281	295	EYVIKVSARVRFFFP	42.89
4232	P43355	MAGE1	DRB1*15:01	111	125	GFLLLKYRAREPVTK	42.90
4233	P43355	MAGE1	DRB1*15:06	111	125	GFLLLKYRAREPVTK	42.90
4234	P43355	MAGE1	DRB1*01:11	261	275	RYEFLWGPRALAETS	42.97
4235	P43355	MAGE1	DRB1*16:09	277	291	VKVLEYVIKVSARVR	42.99
4236	P43355	MAGE1	DRB1*01:18	271	285	LAETSYVKVLEYVIK	43.02
4237	P43355	MAGE1	DRB1*01:29	196	210	IIVLVMIAMEGGHAP	43.02
4238	P43355	MAGE1	DRB1*11:19	280	294	LEYVIKVSARVRFFF	43.02
4239	P43355	MAGE1	DRB1*16:02	277	291	VKVLEYVIKVSARVR	43.03
4240	P43355	MAGE1	DRB1*01:29	276	290	YVKVLEYVIKVSARV	43.11
4241	P43355	MAGE1	DRB1*10:01	279	293	VLEYVIKVSARVRFF	43.12
4242	P43355	MAGE1	DRB1*01:20	24	38	GLVCVQAATSSSSPL	43.15
4243	P43355	MAGE1	DRB1*13:21	260	274	ARYEFLWGPRALAET	43.18
4244	P43355	MAGE1	DRB1*11:13	91	105	SCILESLFRAVITKK	43.25
4245	P43355	MAGE1	DRB1*15:15	280	294	LEYVIKVSARVRFFF	43.25
4246	P43355	MAGE1	DRB1*12:16	95	109	ESLFRAVITKKVADL	43.26
4247	P43355	MAGE1	DRB1*01:20	110	124	VGFLLLKYRAREPVT	43.35
4248	P43355	MAGE1	DRB1*15:01	279	293	VLEYVIKVSARVRFF	43.36
4249	P43355	MAGE1	DRB1*15:06	279	293	VLEYVIKVSARVRFF	43.36

4250	P43355	MAGE1	DRB1*14:05	279	293	VLEYVIKVSARVRFF	43.45
4251	P43355	MAGE1	DRB1*14:23	279	293	VLEYVIKVSARVRFF	43.45
4252	P43355	MAGE1	DRB1*11:42	97	111	LFRAVITKKVADLVG	43.51
4253	P43355	MAGE1	DRB1*01:29	148	162	SESLQLVFGIDVKEA	43.62
4254	P43355	MAGE1	DRB1*07:01	281	295	EYVIKVSARVRFFFP	43.67
4255	P43355	MAGE1	DRB1*14:05	281	295	EYVIKVSARVRFFFP	43.67
4256	P43355	MAGE1	DRB1*14:23	281	295	EYVIKVSARVRFFFP	43.67
4257	P43355	MAGE1	DRB1*15:15	259	273	PARYEFLWGPRALAE	43.71
4258	P43355	MAGE1	DRB1*03:01	279	293	VLEYVIKVSARVRFF	43.72
4259	P43355	MAGE1	DRB1*03:04	279	293	VLEYVIKVSARVRFF	43.72
4260	P43355	MAGE1	DRB1*03:13	279	293	VLEYVIKVSARVRFF	43.72
4261	P43355	MAGE1	DRB1*15:15	278	292	KVLEYVIKVSARVRF	43.72
4262	P43355	MAGE1	DRB1*12:16	107	121	ADLVGFLLLKYRARE	43.73
4263	P43355	MAGE1	DRB1*11:03	287	301	SARVRFFFPSLREAA	43.74
4264	P43355	MAGE1	DRB1*15:01	288	302	ARVRFFFPSLREAAL	43.77
4265	P43355	MAGE1	DRB1*15:06	288	302	ARVRFFFPSLREAAL	43.77
4266	P43355	MAGE1	DRB1*11:13	92	106	CILESLFRAVITKKV	43.87
4267	P43355	MAGE1	DRB1*12:16	108	122	DLVGFLLLKYRAREP	43.87
4268	P43355	MAGE1	DRB1*16:05	96	110	SLFRAVITKKVADLV	43.91
4269	P43355	MAGE1	DPB1*15:01	90	104	TSCILESLFRAVITK	43.93
4270	P43355	MAGE1	DRB1*01:18	291	305	RFFFPSLREAALREE	43.93
4271	P43355	MAGE1	DRB1*01:18	100	114	AVITKKVADLVGFLL	44.03
4272	P43355	MAGE1	DRB1*15:03	288	302	ARVRFFFPSLREAAL	44.07
4273	P43355	MAGE1	DRB1*14:38	282	296	YVIKVSARVRFFFPS	44.16
4274	P43355	MAGE1	DRB1*11:84	110	124	VGFLLLKYRAREPVT	44.21
4275	P43355	MAGE1	DRB1*11:04	96	110	SLFRAVITKKVADLV	44.25
4276	P43355	MAGE1	DRB1*11:46	96	110	SLFRAVITKKVADLV	44.25
4277	P43355	MAGE1	DRB1*11:58	96	110	SLFRAVITKKVADLV	44.25
4278	P43355	MAGE1	DRB1*13:11	96	110	SLFRAVITKKVADLV	44.25
4279	P43355	MAGE1	DRB1*08:30	280	294	LEYVIKVSARVRFFF	44.27
4280	P43355	MAGE1	DPB1*33:01	284	298	IKVSARVRFFFPSLR	44.30
4281	P43355	MAGE1	DPB1*71:01	284	298	IKVSARVRFFFPSLR	44.30
4282	P43355	MAGE1	DRB1*08:30	278	292	KVLEYVIKVSARVRF	44.36
4283	P43355	MAGE1	DRB1*11:08	288	302	ARVRFFFPSLREAAL	44.37
4284	P43355	MAGE1	DRB1*14:32	92	106	CILESLFRAVITKKV	44.43
4285	P43355	MAGE1	DRB1*11:04	95	109	ESLFRAVITKKVADL	44.48
4286	P43355	MAGE1	DRB1*11:46	95	109	ESLFRAVITKKVADL	44.48
4287	P43355	MAGE1	DRB1*11:58	95	109	ESLFRAVITKKVADL	44.48
4288	P43355	MAGE1	DRB1*13:11	95	109	ESLFRAVITKKVADL	44.48
4289	P43355	MAGE1	DRB1*03:15	278	292	KVLEYVIKVSARVRF	44.51
4290	P43355	MAGE1	DRB1*01:18	107	121	ADLVGFLLLKYRARE	44.56
4291	P43355	MAGE1	DRB1*15:03	286	300	VSARVRFFFPSLREA	44.58
4292	P43355	MAGE1	DRB1*08:02	278	292	KVLEYVIKVSARVRF	44.67

4293	P43355	MAGE1	DRB1*10:01	280	294	LEYVIKVSARVRFFF	44.71
4294	P43355	MAGE1	DRB1*16:04	97	111	LFRAVITKKVADLVG	44.71
4295	P43355	MAGE1	DRB1*11:07	281	295	EYVIKVSARVRFFFP	44.78
4296	P43355	MAGE1	DRB1*14:12	282	296	YVIKVSARVRFFFPS	44.84
4297	P43355	MAGE1	DRB1*11:02	287	301	SARVRFFFPSLREAA	44.90
4298	P43355	MAGE1	DRB1*11:65	287	301	SARVRFFFPSLREAA	44.90
4299	P43355	MAGE1	DRB1*13:01	287	301	SARVRFFFPSLREAA	44.90
4300	P43355	MAGE1	DRB1*07:01	277	291	VKVLEYVIKVSARVR	44.91
4301	P43355	MAGE1	DRB1*12:16	96	110	SLFRAVITKKVADLV	44.96
4302	P43355	MAGE1	DRB1*11:27	290	304	VRFFFPSLREAALRE	44.98
4303	P43355	MAGE1	DPB1*02:02	104	118	KKVADLVGFLLLKYR	45.02
4304	P43355	MAGE1	DPB1*47:01	104	118	KKVADLVGFLLLKYR	45.02
4305	P43355	MAGE1	DRB1*11:08	291	305	RFFFPSLREAALREE	45.07
4306	P43355	MAGE1	DRB1*16:02	259	273	PARYEFLWGPRALAE	45.18
4307	P43355	MAGE1	DRB1*11:01	292	306	FFFPSLREAALREEE	45.29
4308	P43355	MAGE1	DRB1*11:10	292	306	FFFPSLREAALREEE	45.29
4309	P43355	MAGE1	DRB1*11:12	292	306	FFFPSLREAALREEE	45.29
4310	P43355	MAGE1	DRB1*11:28	292	306	FFFPSLREAALREEE	45.29
4311	P43355	MAGE1	DRB1*11:29	292	306	FFFPSLREAALREEE	45.29
4312	P43355	MAGE1	DRB1*11:49	292	306	FFFPSLREAALREEE	45.29
4313	P43355	MAGE1	DRB1*11:62	292	306	FFFPSLREAALREEE	45.29
4314	P43355	MAGE1	DRB1*11:74	292	306	FFFPSLREAALREEE	45.29
4315	P43355	MAGE1	DRB1*13:05	292	306	FFFPSLREAALREEE	45.29
4316	P43355	MAGE1	DRB1*13:14	292	306	FFFPSLREAALREEE	45.29
4317	P43355	MAGE1	DRB1*13:50	292	306	FFFPSLREAALREEE	45.29
4318	P43355	MAGE1	DRB1*14:05	280	294	LEYVIKVSARVRFFF	45.32
4319	P43355	MAGE1	DRB1*14:23	280	294	LEYVIKVSARVRFFF	45.32
4320	P43355	MAGE1	DRB1*16:05	93	107	ILESLFRAVITKKVA	45.32
4321	P43355	MAGE1	DRB1*01:20	98	112	FRAVITKKVADLVGF	45.37
4322	P43355	MAGE1	DRB1*03:15	282	296	YVIKVSARVRFFFPS	45.46
4323	P43355	MAGE1	DPB1*33:01	265	279	LWGPRALAETSYVKV	45.55
4324	P43355	MAGE1	DPB1*71:01	265	279	LWGPRALAETSYVKV	45.55
4325	P43355	MAGE1	DRB1*11:42	126	140	AEMLESVIKNYKHCF	45.61
4326	P43355	MAGE1	DRB1*11:13	106	120	VADLVGFLLLKYRAR	45.62
4327	P43355	MAGE1	DRB1*13:21	259	273	PARYEFLWGPRALAE	45.62
4328	P43355	MAGE1	DPB1*02:02	106	120	VADLVGFLLLKYRAR	45.69
4329	P43355	MAGE1	DPB1*47:01	106	120	VADLVGFLLLKYRAR	45.69
4330	P43355	MAGE1	DRB1*16:02	260	274	ARYEFLWGPRALAET	45.70
4331	P43355	MAGE1	DRB1*15:15	279	293	VLEYVIKVSARVRFF	45.80
4332	P43355	MAGE1	DRB1*11:01	111	125	GFLLLKYRAREPVTK	45.81
4333	P43355	MAGE1	DRB1*11:10	111	125	GFLLLKYRAREPVTK	45.81
4334	P43355	MAGE1	DRB1*11:12	111	125	GFLLLKYRAREPVTK	45.81
4335	P43355	MAGE1	DRB1*11:28	111	125	GFLLLKYRAREPVTK	45.81

4336	P43355	MAGE1	DRB1*11:29	111	125	GFLLLKYRAREPVTK	45.81
4337	P43355	MAGE1	DRB1*11:49	111	125	GFLLLKYRAREPVTK	45.81
4338	P43355	MAGE1	DRB1*11:62	111	125	GFLLLKYRAREPVTK	45.81
4339	P43355	MAGE1	DRB1*11:74	111	125	GFLLLKYRAREPVTK	45.81
4340	P43355	MAGE1	DRB1*13:05	111	125	GFLLLKYRAREPVTK	45.81
4341	P43355	MAGE1	DRB1*13:14	111	125	GFLLLKYRAREPVTK	45.81
4342	P43355	MAGE1	DRB1*13:50	111	125	GFLLLKYRAREPVTK	45.81
4343	P43355	MAGE1	DRB1*11:84	108	122	DLVGFLLLKYRAREP	45.88
4344	P43355	MAGE1	DRB1*10:01	23	37	LGLVCVQAATSSSSP	45.92
4345	P43355	MAGE1	DRB1*14:32	93	107	ILESLFRAVITKKVA	45.94
4346	P43355	MAGE1	DRB1*11:13	287	301	SARVRFFFPSLREAA	45.95
4347	P43355	MAGE1	DPB1*72:01	90	104	TSCILESLFRAVITK	46.01
4348	P43355	MAGE1	DRB1*01:01	262	276	YEFLWGPRALAETSY	46.25
4349	P43355	MAGE1	DRB1*11:14	277	291	VKVLEYVIKVSARVR	46.25
4350	P43355	MAGE1	DRB1*13:02	277	291	VKVLEYVIKVSARVR	46.25
4351	P43355	MAGE1	DRB1*13:23	277	291	VKVLEYVIKVSARVR	46.25
4352	P43355	MAGE1	DRB1*13:97	277	291	VKVLEYVIKVSARVR	46.25
4353	P43355	MAGE1	DRB1*01:18	108	122	DLVGFLLLKYRAREP	46.26
4354	P43355	MAGE1	DRB1*14:38	278	292	KVLEYVIKVSARVRF	46.26
4355	P43355	MAGE1	DRB1*11:04	283	297	VIKVSARVRFFFPSL	46.43
4356	P43355	MAGE1	DRB1*11:46	283	297	VIKVSARVRFFFPSL	46.43
4357	P43355	MAGE1	DRB1*11:58	283	297	VIKVSARVRFFFPSL	46.43
4358	P43355	MAGE1	DRB1*13:11	283	297	VIKVSARVRFFFPSL	46.43
4359	P43355	MAGE1	DRB1*01:01	20	34	QEALGLVCVQAATSS	46.63
4360	P43355	MAGE1	DRB1*15:01	110	124	VGFLLLKYRAREPVT	46.64
4361	P43355	MAGE1	DRB1*15:06	110	124	VGFLLLKYRAREPVT	46.64
4362	P43355	MAGE1	DRB1*01:18	187	201	QIMPKTGFLIIVLVM	46.66
4363	P43355	MAGE1	DRB1*01:20	107	121	ADLVGFLLLKYRARE	46.66
4364	P43355	MAGE1	DRB1*12:16	109	123	LVGFLLLKYRAREPV	46.68
4365	P43355	MAGE1	DRB1*01:01	184	198	GDNQIMPKTGFLIIV	46.71
4366	P43355	MAGE1	DRB1*10:01	277	291	VKVLEYVIKVSARVR	46.73
4367	P43355	MAGE1	DRB1*01:20	105	119	KVADLVGFLLLKYRA	46.74
4368	P43355	MAGE1	DRB1*12:03	278	292	KVLEYVIKVSARVRF	46.87
4369	P43355	MAGE1	DRB1*11:07	280	294	LEYVIKVSARVRFFF	47.07
4370	P43355	MAGE1	DRB1*01:01	291	305	RFFFPSLREAALREE	47.18
4371	P43355	MAGE1	DRB1*10:01	20	34	QEALGLVCVQAATSS	47.19
4372	P43355	MAGE1	DRB1*11:13	111	125	GFLLLKYRAREPVTK	47.19
4373	P43355	MAGE1	DRB1*01:01	200	214	VMIAMEGGHAPEEEI	47.22
4374	P43355	MAGE1	DRB1*01:01	146	160	KASESLQLVFGIDVK	47.23
4375	P43355	MAGE1	DRB1*11:13	288	302	ARVRFFFPSLREAAL	47.26
4376	P43355	MAGE1	DRB1*15:02	279	293	VLEYVIKVSARVRFF	47.33
4377	P43355	MAGE1	DRB1*13:61	283	297	VIKVSARVRFFFPSL	47.46
4378	P43355	MAGE1	DRB1*01:18	150	164	SLQLVFGIDVKEADP	47.49

4379	P43355	MAGE1	DPB1*33:01	289	303	RVRFFFPSLREAALR	47.68
4380	P43355	MAGE1	DPB1*71:01	289	303	RVRFFFPSLREAALR	47.68
4381	P43355	MAGE1	DRB1*08:24	289	303	RVRFFFPSLREAALR	47.68
4382	P43355	MAGE1	DRB1*13:21	261	275	RYEFLWGPRALAETS	47.73
4383	P43355	MAGE1	DPB1*02:01	106	120	VADLVGFLLLKYRAR	48.01
4384	P43355	MAGE1	DPB1*46:01	106	120	VADLVGFLLLKYRAR	48.01
4385	P43355	MAGE1	DPB1*81:01	106	120	VADLVGFLLLKYRAR	48.01
4386	P43355	MAGE1	DRB1*01:20	108	122	DLVGFLLLKYRAREP	48.15
4387	P43355	MAGE1	DRB1*11:02	288	302	ARVRFFFPSLREAAL	48.15
4388	P43355	MAGE1	DRB1*11:65	288	302	ARVRFFFPSLREAAL	48.15
4389	P43355	MAGE1	DRB1*13:01	288	302	ARVRFFFPSLREAAL	48.15
4390	P43355	MAGE1	DRB1*13:21	106	120	VADLVGFLLLKYRAR	48.24
4391	P43355	MAGE1	DPB1*02:02	269	283	RALAETSYVKVLEYV	48.52
4392	P43355	MAGE1	DPB1*47:01	269	283	RALAETSYVKVLEYV	48.52
4393	P43355	MAGE1	DRB1*12:16	106	120	VADLVGFLLLKYRAR	48.62
4394	P43355	MAGE1	DRB1*01:29	103	117	TKKVADLVGFLLLKY	48.69
4395	P43355	MAGE1	DRB1*14:05	278	292	KVLEYVIKVSARVRF	48.78
4396	P43355	MAGE1	DRB1*14:23	278	292	KVLEYVIKVSARVRF	48.78
4397	P43355	MAGE1	DRB1*14:04	282	296	YVIKVSARVRFFFPS	48.90
4398	P43355	MAGE1	DRB1*08:24	290	304	VRFFFPSLREAALRE	48.96
4399	P43355	MAGE1	DRB1*11:42	290	304	VRFFFPSLREAALRE	48.96
4400	P43355	MAGE1	DRB1*11:01	287	301	SARVRFFFPSLREAA	49.09
4401	P43355	MAGE1	DRB1*11:10	287	301	SARVRFFFPSLREAA	49.09
4402	P43355	MAGE1	DRB1*11:12	287	301	SARVRFFFPSLREAA	49.09
4403	P43355	MAGE1	DRB1*11:28	287	301	SARVRFFFPSLREAA	49.09
4404	P43355	MAGE1	DRB1*11:29	287	301	SARVRFFFPSLREAA	49.09
4405	P43355	MAGE1	DRB1*11:49	287	301	SARVRFFFPSLREAA	49.09
4406	P43355	MAGE1	DRB1*11:62	287	301	SARVRFFFPSLREAA	49.09
4407	P43355	MAGE1	DRB1*11:74	287	301	SARVRFFFPSLREAA	49.09
4408	P43355	MAGE1	DRB1*13:05	287	301	SARVRFFFPSLREAA	49.09
4409	P43355	MAGE1	DRB1*13:14	287	301	SARVRFFFPSLREAA	49.09
4410	P43355	MAGE1	DRB1*13:50	287	301	SARVRFFFPSLREAA	49.09
4411	P43355	MAGE1	DRB1*01:18	110	124	VGFLLLKYRAREPVT	49.14
4412	P43355	MAGE1	DRB1*08:04	109	123	LVGFLLLKYRAREPV	49.14
4413	P43355	MAGE1	DRB1*15:15	92	106	CILESLFRAVITKKV	49.20
4414	P43355	MAGE1	DRB1*01:24	197	211	IVLVMIAMEGGHAPE	49.25
4415	P43355	MAGE1	DRB1*01:20	106	120	VADLVGFLLLKYRAR	49.37
4416	P43355	MAGE1	DRB1*11:04	92	106	CILESLFRAVITKKV	49.41
4417	P43355	MAGE1	DRB1*11:46	92	106	CILESLFRAVITKKV	49.41
4418	P43355	MAGE1	DRB1*11:58	92	106	CILESLFRAVITKKV	49.41
4419	P43355	MAGE1	DRB1*13:11	92	106	CILESLFRAVITKKV	49.41
4420	P43355	MAGE1	DRB1*15:01	186	200	NQIMPKTGFLIIVLV	49.46
4421	P43355	MAGE1	DRB1*15:06	186	200	NQIMPKTGFLIIVLV	49.46

4422	P43355	MAGE1	DRB1*01:18	105	119	KVADLVGFLLLKYRA	49.58
4423	P43355	MAGE1	DRB1*11:04	94	108	LESLFRAVITKKVAD	49.59
4424	P43355	MAGE1	DRB1*11:46	94	108	LESLFRAVITKKVAD	49.59
4425	P43355	MAGE1	DRB1*11:58	94	108	LESLFRAVITKKVAD	49.59
4426	P43355	MAGE1	DRB1*13:11	94	108	LESLFRAVITKKVAD	49.59
4427	P43355	MAGE1	DRB1*11:03	283	297	VIKVSARVRFFFPSL	49.68
4428	P43355	MAGE1	DRB1*15:02	280	294	LEYVIKVSARVRFFF	49.76
4429	P43355	MAGE1	DRB1*13:66	279	293	VLEYVIKVSARVRFF	49.80
4430	P43355	MAGE1	DRB1*12:03	108	122	DLVGFLLLKYRAREP	49.86
4431	P43355	MAGE1	DRB1*01:18	287	301	SARVRFFFPSLREAA	49.90
4432	P43355	MAGE1	DPB1*40:01	106	120	VADLVGFLLLKYRAR	49.91
4433	P43355	MAGE1	DRB1*12:03	282	296	YVIKVSARVRFFFPS	49.92
4434	P43355	MAGE1	DRB1*01:01	24	38	GLVCVQAATSSSSPL	49.94
4435	P43358	MAGE4	DRB1*01:01	103	117	ESLFREALSNKVDEL	4.49
4436	P43358	MAGE4	DRB1*01:01	104	118	SLFREALSNKVDELA	4.65
4437	P43358	MAGE4	DRB1*01:18	103	117	ESLFREALSNKVDEL	4.95
4438	P43358	MAGE4	DRB1*01:18	104	118	SLFREALSNKVDELA	5.10
4439	P43358	MAGE4	DRB1*01:01	102	116	AESLFREALSNKVDE	5.74
4440	P43358	MAGE4	DRB1*01:01	101	115	DAESLFREALSNKVD	5.80
4441	P43358	MAGE4	DRB1*07:01	100	114	PDAESLFREALSNKV	6.24
4442	P43358	MAGE4	DRB1*01:18	102	116	AESLFREALSNKVDE	6.51
4443	P43358	MAGE4	DRB1*01:18	101	115	DAESLFREALSNKVD	6.63
4444	P43358	MAGE4	DRB1*01:01	105	119	LFREALSNKVDELAH	7.18
4445	P43358	MAGE4	DRB1*07:01	104	118	SLFREALSNKVDELA	7.45
4446	P43358	MAGE4	DRB1*07:01	101	115	DAESLFREALSNKVD	7.47
4447	P43358	MAGE4	DRB1*07:01	103	117	ESLFREALSNKVDEL	7.59
4448	P43358	MAGE4	DRB1*01:18	266	280	NPARYEFLWGPRALA	7.84
4449	P43358	MAGE4	DRB1*01:01	266	280	NPARYEFLWGPRALA	7.86
4450	P43358	MAGE4	DRB1*01:18	105	119	LFREALSNKVDELAH	7.98
4451	P43358	MAGE4	DRB1*01:18	268	282	ARYEFLWGPRALAET	8.09
4452	P43358	MAGE4	DRB1*01:20	156	170	SESLKMIFGIDVKEV	8.10
4453	P43358	MAGE4	DRB1*01:01	268	282	ARYEFLWGPRALAET	8.13
4454	P43358	MAGE4	DRB1*01:18	267	281	PARYEFLWGPRALAE	8.62
4455	P43358	MAGE4	DRB1*01:01	267	281	PARYEFLWGPRALAE	8.83
4456	P43358	MAGE4	DRB1*01:29	103	117	ESLFREALSNKVDEL	9.05
4457	P43358	MAGE4	DRB1*01:20	155	169	ASESLKMIFGIDVKE	9.14
4458	P43358	MAGE4	DRB1*01:18	156	170	SESLKMIFGIDVKEV	9.27
4459	P43358	MAGE4	DRB1*01:01	156	170	SESLKMIFGIDVKEV	9.37
4460	P43358	MAGE4	DRB1*11:02	288	302	LEHVVRVNARVRIAY	9.70
4461	P43358	MAGE4	DRB1*11:65	288	302	LEHVVRVNARVRIAY	9.70
4462	P43358	MAGE4	DRB1*13:01	288	302	LEHVVRVNARVRIAY	9.70
4463	P43358	MAGE4	DRB1*01:01	265	279	SNPARYEFLWGPRAL	9.72
4464	P43358	MAGE4	DRB1*01:29	104	118	SLFREALSNKVDELA	9.83

4465	P43358	MAGE4	DRB1*07:01	102	116	AESLFREALSNKVDE	9.86
4466	P43358	MAGE4	DRB1*01:20	157	171	ESLKMIFGIDVKEVD	9.92
4467	P43358	MAGE4	DRB1*01:24	103	117	ESLFREALSNKVDEL	9.95
4468	P43358	MAGE4	DRB1*01:01	100	114	PDAESLFREALSNKV	9.96
4469	P43358	MAGE4	DRB1*01:18	265	279	SNPARYEFLWGPRAL	10.11
4470	P43358	MAGE4	DRB1*01:24	104	118	SLFREALSNKVDELA	10.49
4471	P43358	MAGE4	DRB1*01:18	269	283	RYEFLWGPRALAETS	10.59
4472	P43358	MAGE4	DRB1*01:01	155	169	ASESLKMIFGIDVKE	10.72
4473	P43358	MAGE4	DRB1*01:11	103	117	ESLFREALSNKVDEL	10.86
4474	P43358	MAGE4	DRB1*01:18	155	169	ASESLKMIFGIDVKE	10.97
4475	P43358	MAGE4	DRB1*07:01	105	119	LFREALSNKVDELAH	10.99
4476	P43358	MAGE4	DRB1*01:01	269	283	RYEFLWGPRALAETS	11.06
4477	P43358	MAGE4	DRB1*01:18	157	171	ESLKMIFGIDVKEVD	11.17
4478	P43358	MAGE4	DRB1*09:01	100	114	PDAESLFREALSNKV	11.19
4479	P43358	MAGE4	DRB1*01:18	100	114	PDAESLFREALSNKV	11.27
4480	P43358	MAGE4	DRB1*01:20	154	168	KASESLKMIFGIDVK	11.33
4481	P43358	MAGE4	DRB1*09:01	104	118	SLFREALSNKVDELA	11.50
4482	P43358	MAGE4	DRB1*11:02	289	303	EHVVRVNARVRIAYP	11.58
4483	P43358	MAGE4	DRB1*11:65	289	303	EHVVRVNARVRIAYP	11.58
4484	P43358	MAGE4	DRB1*13:01	289	303	EHVVRVNARVRIAYP	11.58
4485	P43358	MAGE4	DRB1*01:11	104	118	SLFREALSNKVDELA	11.61
4486	P43358	MAGE4	DRB1*01:01	157	171	ESLKMIFGIDVKEVD	12.00
4487	P43358	MAGE4	DRB1*09:01	103	117	ESLFREALSNKVDEL	12.12
4488	P43358	MAGE4	DRB1*01:29	102	116	AESLFREALSNKVDE	12.21
4489	P43358	MAGE4	DRB1*09:01	101	115	DAESLFREALSNKVD	12.29
4490	P43358	MAGE4	DRB1*11:02	287	301	VLEHVVRVNARVRIA	12.31
4491	P43358	MAGE4	DRB1*11:65	287	301	VLEHVVRVNARVRIA	12.31
4492	P43358	MAGE4	DRB1*13:01	287	301	VLEHVVRVNARVRIA	12.31
4493	P43358	MAGE4	DRB1*11:02	290	304	HVVRVNARVRIAYPS	12.34
4494	P43358	MAGE4	DRB1*11:65	290	304	HVVRVNARVRIAYPS	12.34
4495	P43358	MAGE4	DRB1*13:01	290	304	HVVRVNARVRIAYPS	12.34
4496	P43358	MAGE4	DRB1*11:42	288	302	LEHVVRVNARVRIAY	12.35
4497	P43358	MAGE4	DRB1*01:29	101	115	DAESLFREALSNKVD	13.37
4498	P43358	MAGE4	DRB1*10:01	103	117	ESLFREALSNKVDEL	13.38
4499	P43358	MAGE4	DRB1*11:42	286	300	KVLEHVVRVNARVRI	13.44
4500	P43358	MAGE4	DRB1*15:01	119	133	HFLLRKYRAKELVTK	13.46
4501	P43358	MAGE4	DRB1*15:06	119	133	HFLLRKYRAKELVTK	13.46
4502	P43358	MAGE4	DRB1*01:18	154	168	KASESLKMIFGIDVK	13.60
4503	P43358	MAGE4	DRB1*11:03	288	302	LEHVVRVNARVRIAY	13.70
4504	P43358	MAGE4	DRB1*01:24	102	116	AESLFREALSNKVDE	13.79
4505	P43358	MAGE4	DRB1*01:01	154	168	KASESLKMIFGIDVK	13.80
4506	P43358	MAGE4	DRB1*11:04	285	299	VKVLEHVVRVNARVR	13.92
4507	P43358	MAGE4	DRB1*11:46	285	299	VKVLEHVVRVNARVR	13.92

4508	P43358	MAGE4	DRB1*11:58	285	299	VKVLEHVVRVNARVR	13.92
4509	P43358	MAGE4	DRB1*13:11	285	299	VKVLEHVVRVNARVR	13.92
4510	P43358	MAGE4	DRB1*11:42	287	301	VLEHVVRVNARVRIA	14.07
4511	P43358	MAGE4	DRB1*11:13	288	302	LEHVVRVNARVRIAY	14.21
4512	P43358	MAGE4	DRB1*15:01	118	132	AHFLLRKYRAKELVT	14.28
4513	P43358	MAGE4	DRB1*15:06	118	132	AHFLLRKYRAKELVT	14.28
4514	P43358	MAGE4	DRB1*11:04	286	300	KVLEHVVRVNARVRI	14.30
4515	P43358	MAGE4	DRB1*11:46	286	300	KVLEHVVRVNARVRI	14.30
4516	P43358	MAGE4	DRB1*11:58	286	300	KVLEHVVRVNARVRI	14.30
4517	P43358	MAGE4	DRB1*13:11	286	300	KVLEHVVRVNARVRI	14.30
4518	P43358	MAGE4	DRB1*01:24	101	115	DAESLFREALSNKVD	14.67
4519	P43358	MAGE4	DRB1*10:01	104	118	SLFREALSNKVDELA	14.76
4520	P43358	MAGE4	DRB1*15:01	120	134	FLLRKYRAKELVTKA	14.76
4521	P43358	MAGE4	DRB1*15:06	120	134	FLLRKYRAKELVTKA	14.76
4522	P43358	MAGE4	DRB1*11:02	286	300	KVLEHVVRVNARVRI	14.80
4523	P43358	MAGE4	DRB1*11:65	286	300	KVLEHVVRVNARVRI	14.80
4524	P43358	MAGE4	DRB1*13:01	286	300	KVLEHVVRVNARVRI	14.80
4525	P43358	MAGE4	DRB1*11:42	289	303	EHVVRVNARVRIAYP	15.17
4526	P43358	MAGE4	DRB1*01:11	102	116	AESLFREALSNKVDE	15.33
4527	P43358	MAGE4	DRB1*09:01	102	116	AESLFREALSNKVDE	15.34
4528	P43358	MAGE4	DRB1*15:01	117	131	LAHFLLRKYRAKELV	15.40
4529	P43358	MAGE4	DRB1*15:06	117	131	LAHFLLRKYRAKELV	15.40
4530	P43358	MAGE4	DRB1*01:29	105	119	LFREALSNKVDELAH	15.46
4531	P43358	MAGE4	DRB1*01:20	103	117	ESLFREALSNKVDEL	15.57
4532	P43358	MAGE4	DRB1*13:61	288	302	LEHVVRVNARVRIAY	15.71
4533	P43358	MAGE4	DRB1*09:01	105	119	LFREALSNKVDELAH	15.87
4534	P43358	MAGE4	DRB1*11:42	285	299	VKVLEHVVRVNARVR	16.03
4535	P43358	MAGE4	DRB1*11:13	289	303	EHVVRVNARVRIAYP	16.16
4536	P43358	MAGE4	DRB1*01:11	101	115	DAESLFREALSNKVD	16.22
4537	P43358	MAGE4	DRB1*11:03	289	303	EHVVRVNARVRIAYP	16.52
4538	P43358	MAGE4	DRB1*11:13	287	301	VLEHVVRVNARVRIA	16.61
4539	P43358	MAGE4	DRB1*11:02	291	305	VVRVNARVRIAYPSL	16.81
4540	P43358	MAGE4	DRB1*11:65	291	305	VVRVNARVRIAYPSL	16.81
4541	P43358	MAGE4	DRB1*13:01	291	305	VVRVNARVRIAYPSL	16.81
4542	P43358	MAGE4	DRB1*01:29	266	280	NPARYEFLWGPRALA	16.85
4543	P43358	MAGE4	DRB1*01:24	105	119	LFREALSNKVDELAH	16.94
4544	P43358	MAGE4	DRB1*11:03	287	301	VLEHVVRVNARVRIA	17.08
4545	P43358	MAGE4	DRB1*01:29	267	281	PARYEFLWGPRALAE	17.19
4546	P43358	MAGE4	DRB1*11:14	288	302	LEHVVRVNARVRIAY	17.31
4547	P43358	MAGE4	DRB1*13:02	288	302	LEHVVRVNARVRIAY	17.31
4548	P43358	MAGE4	DRB1*13:23	288	302	LEHVVRVNARVRIAY	17.31
4549	P43358	MAGE4	DRB1*13:97	288	302	LEHVVRVNARVRIAY	17.31
4550	P43358	MAGE4	DRB1*11:03	290	304	HVVRVNARVRIAYPS	17.33

4551	P43358	MAGE4	DRB1*01:20	104	118	SLFREALSNKVDELA	17.43
4552	P43358	MAGE4	DRB1*10:01	102	116	AESLFREALSNKVDE	17.56
4553	P43358	MAGE4	DRB1*16:02	103	117	ESLFREALSNKVDEL	17.65
4554	P43358	MAGE4	DRB1*01:24	156	170	SESLKMIFGIDVKEV	17.73
4555	P43358	MAGE4	DRB1*10:01	101	115	DAESLFREALSNKVD	17.97
4556	P43358	MAGE4	DRB1*01:29	268	282	ARYEFLWGPRALAET	18.01
4557	P43358	MAGE4	DRB1*11:42	290	304	HVVRVNARVRIAYPS	18.19
4558	P43358	MAGE4	DRB1*11:13	286	300	KVLEHVVRVNARVRI	18.27
4559	P43358	MAGE4	DRB1*11:13	290	304	HVVRVNARVRIAYPS	18.31
4560	P43358	MAGE4	DRB1*01:29	156	170	SESLKMIFGIDVKEV	18.33
4561	P43358	MAGE4	DRB1*10:01	266	280	NPARYEFLWGPRALA	18.33
4562	P43358	MAGE4	DRB1*01:18	158	172	SLKMIFGIDVKEVDP	18.44
4563	P43358	MAGE4	DRB1*11:03	117	131	LAHFLLRKYRAKELV	18.47
4564	P43358	MAGE4	DRB1*01:24	266	280	NPARYEFLWGPRALA	18.56
4565	P43358	MAGE4	DRB1*10:01	268	282	ARYEFLWGPRALAET	18.69
4566	P43358	MAGE4	DRB1*01:20	158	172	SLKMIFGIDVKEVDP	18.88
4567	P43358	MAGE4	DRB1*11:03	116	130	ELAHFLLRKYRAKEL	19.32
4568	P43358	MAGE4	DRB1*01:11	105	119	LFREALSNKVDELAH	19.34
4569	P43358	MAGE4	DRB1*10:01	267	281	PARYEFLWGPRALAE	19.45
4570	P43358	MAGE4	DRB1*13:61	289	303	EHVVRVNARVRIAYP	19.49
4571	P43358	MAGE4	DRB1*11:03	286	300	KVLEHVVRVNARVRI	19.62
4572	P43358	MAGE4	DRB1*14:32	288	302	LEHVVRVNARVRIAY	19.69
4573	P43358	MAGE4	DRB1*11:04	284	298	YVKVLEHVVRVNARV	19.77
4574	P43358	MAGE4	DRB1*11:46	284	298	YVKVLEHVVRVNARV	19.77
4575	P43358	MAGE4	DRB1*11:58	284	298	YVKVLEHVVRVNARV	19.77
4576	P43358	MAGE4	DRB1*13:11	284	298	YVKVLEHVVRVNARV	19.77
4577	P43358	MAGE4	DRB1*11:04	116	130	ELAHFLLRKYRAKEL	19.95
4578	P43358	MAGE4	DRB1*11:46	116	130	ELAHFLLRKYRAKEL	19.95
4579	P43358	MAGE4	DRB1*11:58	116	130	ELAHFLLRKYRAKEL	19.95
4580	P43358	MAGE4	DRB1*13:11	116	130	ELAHFLLRKYRAKEL	19.95
4581	P43358	MAGE4	DRB1*01:24	267	281	PARYEFLWGPRALAE	20.02
4582	P43358	MAGE4	DRB1*01:24	268	282	ARYEFLWGPRALAET	20.15
4583	P43358	MAGE4	DRB1*16:02	104	118	SLFREALSNKVDELA	20.15
4584	P43358	MAGE4	DRB1*01:01	264	278	GSNPARYEFLWGPRA	20.69
4585	P43358	MAGE4	DRB1*11:14	287	301	VLEHVVRVNARVRIA	20.72
4586	P43358	MAGE4	DRB1*13:02	287	301	VLEHVVRVNARVRIA	20.72
4587	P43358	MAGE4	DRB1*13:23	287	301	VLEHVVRVNARVRIA	20.72
4588	P43358	MAGE4	DRB1*13:97	287	301	VLEHVVRVNARVRIA	20.72
4589	P43358	MAGE4	DRB1*11:14	289	303	EHVVRVNARVRIAYP	20.84
4590	P43358	MAGE4	DRB1*13:02	289	303	EHVVRVNARVRIAYP	20.84
4591	P43358	MAGE4	DRB1*13:23	289	303	EHVVRVNARVRIAYP	20.84
4592	P43358	MAGE4	DRB1*13:97	289	303	EHVVRVNARVRIAYP	20.84
4593	P43358	MAGE4	DRB1*13:61	287	301	VLEHVVRVNARVRIA	20.85

4594	P43358	MAGE4	DRB1*11:04	287	301	VLEHVVRVNARVRIA	20.88
4595	P43358	MAGE4	DRB1*11:46	287	301	VLEHVVRVNARVRIA	20.88
4596	P43358	MAGE4	DRB1*11:58	287	301	VLEHVVRVNARVRIA	20.88
4597	P43358	MAGE4	DRB1*13:11	287	301	VLEHVVRVNARVRIA	20.88
4598	P43358	MAGE4	DRB1*13:61	290	304	HVVRVNARVRIAYPS	21.02
4599	P43358	MAGE4	DRB1*11:04	117	131	LAHFLLRKYRAKELV	21.15
4600	P43358	MAGE4	DRB1*11:46	117	131	LAHFLLRKYRAKELV	21.15
4601	P43358	MAGE4	DRB1*11:58	117	131	LAHFLLRKYRAKELV	21.15
4602	P43358	MAGE4	DRB1*13:11	117	131	LAHFLLRKYRAKELV	21.15
4603	P43358	MAGE4	DRB1*11:03	118	132	AHFLLRKYRAKELVT	21.64
4604	P43358	MAGE4	DRB1*01:18	264	278	GSNPARYEFLWGPRA	21.70
4605	P43358	MAGE4	DRB1*01:24	157	171	ESLKMIFGIDVKEVD	21.72
4606	P43358	MAGE4	DRB1*13:21	117	131	LAHFLLRKYRAKELV	21.81
4607	P43358	MAGE4	DRB1*11:04	283	297	SYVKVLEHVVRVNAR	21.83
4608	P43358	MAGE4	DRB1*11:46	283	297	SYVKVLEHVVRVNAR	21.83
4609	P43358	MAGE4	DRB1*11:58	283	297	SYVKVLEHVVRVNAR	21.83
4610	P43358	MAGE4	DRB1*13:11	283	297	SYVKVLEHVVRVNAR	21.83
4611	P43358	MAGE4	DRB1*14:32	289	303	EHVVRVNARVRIAYP	21.91
4612	P43358	MAGE4	DRB1*01:20	102	116	AESLFREALSNKVDE	22.10
4613	P43358	MAGE4	DRB1*01:29	155	169	ASESLKMIFGIDVKE	22.10
4614	P43358	MAGE4	DRB1*11:42	284	298	YVKVLEHVVRVNARV	22.47
4615	P43358	MAGE4	DRB1*13:21	116	130	ELAHFLLRKYRAKEL	22.48
4616	P43358	MAGE4	DRB1*14:06	288	302	LEHVVRVNARVRIAY	22.55
4617	P43358	MAGE4	DRB1*01:29	100	114	PDAESLFREALSNKV	22.68
4618	P43358	MAGE4	DRB1*01:24	155	169	ASESLKMIFGIDVKE	22.73
4619	P43358	MAGE4	DRB1*11:03	291	305	VVRVNARVRIAYPSL	22.84
4620	P43358	MAGE4	DRB1*15:03	119	133	HFLLRKYRAKELVTK	22.90
4621	P43358	MAGE4	DRB1*01:20	101	115	DAESLFREALSNKVD	23.03
4622	P43358	MAGE4	DRB1*13:96	288	302	LEHVVRVNARVRIAY	23.21
4623	P43358	MAGE4	DPB1*33:01	274	288	WGPRALAETSYVKVL	23.48
4624	P43358	MAGE4	DPB1*71:01	274	288	WGPRALAETSYVKVL	23.48
4625	P43358	MAGE4	DRB1*11:14	290	304	HVVRVNARVRIAYPS	23.63
4626	P43358	MAGE4	DRB1*13:02	290	304	HVVRVNARVRIAYPS	23.63
4627	P43358	MAGE4	DRB1*13:23	290	304	HVVRVNARVRIAYPS	23.63
4628	P43358	MAGE4	DRB1*13:97	290	304	HVVRVNARVRIAYPS	23.63
4629	P43358	MAGE4	DRB1*15:03	117	131	LAHFLLRKYRAKELV	23.76
4630	P43358	MAGE4	DRB1*15:03	118	132	AHFLLRKYRAKELVT	23.85
4631	P43358	MAGE4	DRB1*15:03	120	134	FLLRKYRAKELVTKA	23.85
4632	P43358	MAGE4	DRB1*11:42	283	297	SYVKVLEHVVRVNAR	23.89
4633	P43358	MAGE4	DRB1*01:01	158	172	SLKMIFGIDVKEVDP	23.90
4634	P43358	MAGE4	DRB1*01:20	268	282	ARYEFLWGPRALAET	23.93
4635	P43358	MAGE4	DRB1*10:01	265	279	SNPARYEFLWGPRAL	24.12
4636	P43358	MAGE4	DRB1*11:02	292	306	VRVNARVRIAYPSLR	24.23

4637	P43358	MAGE4	DRB1*11:65	292	306	VRVNARVRIAYPSLR	24.23
4638	P43358	MAGE4	DRB1*13:01	292	306	VRVNARVRIAYPSLR	24.23
4639	P43358	MAGE4	DPB1*33:01	275	289	GPRALAETSYVKVLE	24.39
4640	P43358	MAGE4	DPB1*71:01	275	289	GPRALAETSYVKVLE	24.39
4641	P43358	MAGE4	DRB1*11:13	285	299	VKVLEHVVRVNARVR	24.40
4642	P43358	MAGE4	DRB1*14:32	287	301	VLEHVVRVNARVRIA	24.50
4643	P43358	MAGE4	DPB1*33:01	276	290	PRALAETSYVKVLEH	24.55
4644	P43358	MAGE4	DPB1*71:01	276	290	PRALAETSYVKVLEH	24.55
4645	P43358	MAGE4	DRB1*01:29	157	171	ESLKMIFGIDVKEVD	24.59
4646	P43358	MAGE4	DRB1*16:02	101	115	DAESLFREALSNKVD	24.60
4647	P43358	MAGE4	DRB1*01:18	193	207	NNQIFPKTGLLIIVL	24.71
4648	P43358	MAGE4	DRB1*01:02	156	170	SESLKMIFGIDVKEV	24.76
4649	P43358	MAGE4	DRB1*03:11	288	302	LEHVVRVNARVRIAY	24.86
4650	P43358	MAGE4	DRB1*10:01	105	119	LFREALSNKVDELAH	25.01
4651	P43358	MAGE4	DRB1*16:02	102	116	AESLFREALSNKVDE	25.03
4652	P43358	MAGE4	DRB1*11:13	291	305	VVRVNARVRIAYPSL	25.05
4653	P43358	MAGE4	DRB1*01:20	266	280	NPARYEFLWGPRALA	25.10
4654	P43358	MAGE4	DRB1*11:03	115	129	DELAHFLLRKYRAKE	25.19
4655	P43358	MAGE4	DRB1*01:24	100	114	PDAESLFREALSNKV	25.31
4656	P43358	MAGE4	DRB1*01:20	193	207	NNQIFPKTGLLIIVL	25.40
4657	P43358	MAGE4	DRB1*11:03	114	128	VDELAHFLLRKYRAK	25.44
4658	P43358	MAGE4	DRB1*11:42	291	305	VVRVNARVRIAYPSL	25.48
4659	P43358	MAGE4	DRB1*11:03	285	299	VKVLEHVVRVNARVR	25.51
4660	P43358	MAGE4	DRB1*01:18	282	296	TSYVKVLEHVVRVNA	25.53
4661	P43358	MAGE4	DRB1*01:24	265	279	SNPARYEFLWGPRAL	25.56
4662	P43358	MAGE4	DRB1*11:03	119	133	HFLLRKYRAKELVTK	25.58
4663	P43358	MAGE4	DRB1*15:01	121	135	LLRKYRAKELVTKAE	25.64
4664	P43358	MAGE4	DRB1*15:06	121	135	LLRKYRAKELVTKAE	25.64
4665	P43358	MAGE4	DRB1*11:42	116	130	ELAHFLLRKYRAKEL	25.66
4666	P43358	MAGE4	DRB1*11:04	115	129	DELAHFLLRKYRAKE	25.68
4667	P43358	MAGE4	DRB1*11:46	115	129	DELAHFLLRKYRAKE	25.68
4668	P43358	MAGE4	DRB1*11:58	115	129	DELAHFLLRKYRAKE	25.68
4669	P43358	MAGE4	DRB1*13:11	115	129	DELAHFLLRKYRAKE	25.68
4670	P43358	MAGE4	DRB1*01:11	266	280	NPARYEFLWGPRALA	25.76
4671	P43358	MAGE4	DRB1*01:11	100	114	PDAESLFREALSNKV	25.78
4672	P43358	MAGE4	DPB1*33:01	277	291	RALAETSYVKVLEHV	25.83
4673	P43358	MAGE4	DPB1*71:01	277	291	RALAETSYVKVLEHV	25.83
4674	P43358	MAGE4	DRB1*14:06	287	301	VLEHVVRVNARVRIA	25.83
4675	P43358	MAGE4	DRB1*01:29	265	279	SNPARYEFLWGPRAL	25.85
4676	P43358	MAGE4	DRB1*11:04	118	132	AHFLLRKYRAKELVT	26.10
4677	P43358	MAGE4	DRB1*11:46	118	132	AHFLLRKYRAKELVT	26.10
4678	P43358	MAGE4	DRB1*11:58	118	132	AHFLLRKYRAKELVT	26.10
4679	P43358	MAGE4	DRB1*13:11	118	132	AHFLLRKYRAKELVT	26.10

4680	P43358	MAGE4	DRB1*03:11	289	303	EHVVRVNARVRIAYP	26.15
4681	P43358	MAGE4	DRB1*01:29	269	283	RYEFLWGPRALAETS	26.23
4682	P43358	MAGE4	DRB1*01:20	267	281	PARYEFLWGPRALAE	26.24
4683	P43358	MAGE4	DRB1*01:11	156	170	SESLKMIFGIDVKEV	26.37
4684	P43358	MAGE4	DRB1*15:01	116	130	ELAHFLLRKYRAKEL	26.43
4685	P43358	MAGE4	DRB1*15:06	116	130	ELAHFLLRKYRAKEL	26.43
4686	P43358	MAGE4	DRB1*14:32	290	304	HVVRVNARVRIAYPS	26.51
4687	P43358	MAGE4	DRB1*11:14	286	300	KVLEHVVRVNARVRI	26.57
4688	P43358	MAGE4	DRB1*13:02	286	300	KVLEHVVRVNARVRI	26.57
4689	P43358	MAGE4	DRB1*13:23	286	300	KVLEHVVRVNARVRI	26.57
4690	P43358	MAGE4	DRB1*13:97	286	300	KVLEHVVRVNARVRI	26.57
4691	P43358	MAGE4	DRB1*10:01	269	283	RYEFLWGPRALAETS	26.60
4692	P43358	MAGE4	DRB1*11:04	288	302	LEHVVRVNARVRIAY	26.61
4693	P43358	MAGE4	DRB1*11:46	288	302	LEHVVRVNARVRIAY	26.61
4694	P43358	MAGE4	DRB1*11:58	288	302	LEHVVRVNARVRIAY	26.61
4695	P43358	MAGE4	DRB1*13:11	288	302	LEHVVRVNARVRIAY	26.61
4696	P43358	MAGE4	DRB1*11:03	137	151	LERVIKNYKRCFPVI	26.62
4697	P43358	MAGE4	DRB1*01:24	154	168	KASESLKMIFGIDVK	26.68
4698	P43358	MAGE4	DRB1*14:32	286	300	KVLEHVVRVNARVRI	26.76
4699	P43358	MAGE4	DRB1*01:20	148	162	FPVIFGKASESLKMI	26.81
4700	P43358	MAGE4	DRB1*11:42	117	131	LAHFLLRKYRAKELV	27.04
4701	P43358	MAGE4	DRB1*11:03	136	150	MLERVIKNYKRCFPV	27.24
4702	P43358	MAGE4	DRB1*11:02	285	299	VKVLEHVVRVNARVR	27.40
4703	P43358	MAGE4	DRB1*11:65	285	299	VKVLEHVVRVNARVR	27.40
4704	P43358	MAGE4	DRB1*13:01	285	299	VKVLEHVVRVNARVR	27.40
4705	P43358	MAGE4	DRB1*14:06	289	303	EHVVRVNARVRIAYP	27.45
4706	P43358	MAGE4	DRB1*01:11	267	281	PARYEFLWGPRALAE	27.46
4707	P43358	MAGE4	DRB1*15:15	119	133	HFLLRKYRAKELVTK	27.56
4708	P43358	MAGE4	DRB1*15:07	119	133	HFLLRKYRAKELVTK	27.60
4709	P43358	MAGE4	DRB1*14:06	286	300	KVLEHVVRVNARVRI	27.88
4710	P43358	MAGE4	DRB1*01:18	281	295	ETSYVKVLEHVVRVN	27.89
4711	P43358	MAGE4	DRB1*01:18	280	294	AETSYVKVLEHVVRV	27.90
4712	P43358	MAGE4	DRB1*01:11	268	282	ARYEFLWGPRALAET	28.01
4713	P43358	MAGE4	DRB1*10:01	100	114	PDAESLFREALSNKV	28.06
4714	P43358	MAGE4	DRB1*11:04	114	128	VDELAHFLLRKYRAK	28.08
4715	P43358	MAGE4	DRB1*11:46	114	128	VDELAHFLLRKYRAK	28.08
4716	P43358	MAGE4	DRB1*11:58	114	128	VDELAHFLLRKYRAK	28.08
4717	P43358	MAGE4	DRB1*13:11	114	128	VDELAHFLLRKYRAK	28.08
4718	P43358	MAGE4	DRB1*13:21	118	132	AHFLLRKYRAKELVT	28.14
4719	P43358	MAGE4	DRB1*13:96	287	301	VLEHVVRVNARVRIA	28.34
4720	P43358	MAGE4	DRB1*01:02	157	171	ESLKMIFGIDVKEVD	28.36
4721	P43358	MAGE4	DRB1*13:96	289	303	EHVVRVNARVRIAYP	28.40
4722	P43358	MAGE4	DRB1*01:20	192	206	GNNQIFPKTGLLIIV	28.64

4723	P43358	MAGE4	DRB1*01:02	155	169	ASESLKMIFGIDVKE	28.69
4724	P43358	MAGE4	DRB1*11:01	116	130	ELAHFLLRKYRAKEL	28.81
4725	P43358	MAGE4	DRB1*11:10	116	130	ELAHFLLRKYRAKEL	28.81
4726	P43358	MAGE4	DRB1*11:12	116	130	ELAHFLLRKYRAKEL	28.81
4727	P43358	MAGE4	DRB1*11:28	116	130	ELAHFLLRKYRAKEL	28.81
4728	P43358	MAGE4	DRB1*11:29	116	130	ELAHFLLRKYRAKEL	28.81
4729	P43358	MAGE4	DRB1*11:49	116	130	ELAHFLLRKYRAKEL	28.81
4730	P43358	MAGE4	DRB1*11:62	116	130	ELAHFLLRKYRAKEL	28.81
4731	P43358	MAGE4	DRB1*11:74	116	130	ELAHFLLRKYRAKEL	28.81
4732	P43358	MAGE4	DRB1*13:05	116	130	ELAHFLLRKYRAKEL	28.81
4733	P43358	MAGE4	DRB1*13:14	116	130	ELAHFLLRKYRAKEL	28.81
4734	P43358	MAGE4	DRB1*13:50	116	130	ELAHFLLRKYRAKEL	28.81
4735	P43358	MAGE4	DRB1*01:29	154	168	KASESLKMIFGIDVK	28.91
4736	P43358	MAGE4	DRB1*01:24	269	283	RYEFLWGPRALAETS	29.01
4737	P43358	MAGE4	DRB1*01:18	192	206	GNNQIFPKTGLLIIV	29.03
4738	P43358	MAGE4	DRB1*11:42	115	129	DELAHFLLRKYRAKE	29.07
4739	P43358	MAGE4	DRB1*03:11	287	301	VLEHVVRVNARVRIA	29.41
4740	P43358	MAGE4	DRB1*11:42	295	309	NARVRIAYPSLREAA	29.59
4741	P43358	MAGE4	DRB1*15:15	118	132	AHFLLRKYRAKELVT	29.66
4742	P43358	MAGE4	DRB1*11:01	117	131	LAHFLLRKYRAKELV	29.70
4743	P43358	MAGE4	DRB1*11:10	117	131	LAHFLLRKYRAKELV	29.70
4744	P43358	MAGE4	DRB1*11:12	117	131	LAHFLLRKYRAKELV	29.70
4745	P43358	MAGE4	DRB1*11:28	117	131	LAHFLLRKYRAKELV	29.70
4746	P43358	MAGE4	DRB1*11:29	117	131	LAHFLLRKYRAKELV	29.70
4747	P43358	MAGE4	DRB1*11:42	114	128	VDELAHFLLRKYRAK	29.70
4748	P43358	MAGE4	DRB1*11:49	117	131	LAHFLLRKYRAKELV	29.70
4749	P43358	MAGE4	DRB1*11:62	117	131	LAHFLLRKYRAKELV	29.70
4750	P43358	MAGE4	DRB1*11:74	117	131	LAHFLLRKYRAKELV	29.70
4751	P43358	MAGE4	DRB1*13:05	117	131	LAHFLLRKYRAKELV	29.70
4752	P43358	MAGE4	DRB1*13:14	117	131	LAHFLLRKYRAKELV	29.70
4753	P43358	MAGE4	DRB1*13:50	117	131	LAHFLLRKYRAKELV	29.70
4754	P43358	MAGE4	DRB1*11:13	284	298	YVKVLEHVVRVNARV	29.80
4755	P43358	MAGE4	DRB1*13:21	115	129	DELAHFLLRKYRAKE	29.82
4756	P43358	MAGE4	DRB1*11:02	293	307	RVNARVRIAYPSLRE	29.94
4757	P43358	MAGE4	DRB1*11:65	293	307	RVNARVRIAYPSLRE	29.94
4758	P43358	MAGE4	DRB1*13:01	293	307	RVNARVRIAYPSLRE	29.94
4759	P43358	MAGE4	DRB1*11:02	137	151	LERVIKNYKRCFPVI	30.05
4760	P43358	MAGE4	DRB1*11:65	137	151	LERVIKNYKRCFPVI	30.05
4761	P43358	MAGE4	DRB1*13:01	137	151	LERVIKNYKRCFPVI	30.05
4762	P43358	MAGE4	DRB1*13:61	291	305	VVRVNARVRIAYPSL	30.10
4763	P43358	MAGE4	DRB1*11:01	285	299	VKVLEHVVRVNARVR	30.17
4764	P43358	MAGE4	DRB1*11:10	285	299	VKVLEHVVRVNARVR	30.17
4765	P43358	MAGE4	DRB1*11:12	285	299	VKVLEHVVRVNARVR	30.17

4766	P43358	MAGE4	DRB1*11:28	285	299	VKVLEHVVRVNARVR	30.17
4767	P43358	MAGE4	DRB1*11:29	285	299	VKVLEHVVRVNARVR	30.17
4768	P43358	MAGE4	DRB1*11:49	285	299	VKVLEHVVRVNARVR	30.17
4769	P43358	MAGE4	DRB1*11:62	285	299	VKVLEHVVRVNARVR	30.17
4770	P43358	MAGE4	DRB1*11:74	285	299	VKVLEHVVRVNARVR	30.17
4771	P43358	MAGE4	DRB1*13:05	285	299	VKVLEHVVRVNARVR	30.17
4772	P43358	MAGE4	DRB1*13:14	285	299	VKVLEHVVRVNARVR	30.17
4773	P43358	MAGE4	DRB1*13:50	285	299	VKVLEHVVRVNARVR	30.17
4774	P43358	MAGE4	DRB1*01:20	295	309	NARVRIAYPSLREAA	30.32
4775	P43358	MAGE4	DRB1*13:61	286	300	KVLEHVVRVNARVRI	30.33
4776	P43358	MAGE4	DRB1*15:07	118	132	AHFLLRKYRAKELVT	30.41
4777	P43358	MAGE4	DRB1*11:13	283	297	SYVKVLEHVVRVNAR	30.46
4778	P43358	MAGE4	DRB1*15:15	117	131	LAHFLLRKYRAKELV	30.50
4779	P43358	MAGE4	DRB1*01:18	283	297	SYVKVLEHVVRVNAR	31.03
4780	P43358	MAGE4	DRB1*01:20	296	310	ARVRIAYPSLREAAL	31.08
4781	P43358	MAGE4	DRB1*15:07	120	134	FLLRKYRAKELVTKA	31.24
4782	P43358	MAGE4	DRB1*11:03	292	306	VRVNARVRIAYPSLR	31.25
4783	P43358	MAGE4	DRB1*14:06	290	304	HVVRVNARVRIAYPS	31.30
4784	P43358	MAGE4	DRB1*01:20	147	161	CFPVIFGKASESLKM	31.33
4785	P43358	MAGE4	DRB1*01:18	141	155	IKNYKRCFPVIFGKA	31.43
4786	P43358	MAGE4	DRB1*11:02	117	131	LAHFLLRKYRAKELV	31.45
4787	P43358	MAGE4	DRB1*11:65	117	131	LAHFLLRKYRAKELV	31.45
4788	P43358	MAGE4	DRB1*13:01	117	131	LAHFLLRKYRAKELV	31.45
4789	P43358	MAGE4	DRB1*11:01	286	300	KVLEHVVRVNARVRI	31.50
4790	P43358	MAGE4	DRB1*11:10	286	300	KVLEHVVRVNARVRI	31.50
4791	P43358	MAGE4	DRB1*11:12	286	300	KVLEHVVRVNARVRI	31.50
4792	P43358	MAGE4	DRB1*11:28	286	300	KVLEHVVRVNARVRI	31.50
4793	P43358	MAGE4	DRB1*11:29	286	300	KVLEHVVRVNARVRI	31.50
4794	P43358	MAGE4	DRB1*11:49	286	300	KVLEHVVRVNARVRI	31.50
4795	P43358	MAGE4	DRB1*11:62	286	300	KVLEHVVRVNARVRI	31.50
4796	P43358	MAGE4	DRB1*11:74	286	300	KVLEHVVRVNARVRI	31.50
4797	P43358	MAGE4	DRB1*13:05	286	300	KVLEHVVRVNARVRI	31.50
4798	P43358	MAGE4	DRB1*13:14	286	300	KVLEHVVRVNARVRI	31.50
4799	P43358	MAGE4	DRB1*13:50	286	300	KVLEHVVRVNARVRI	31.50
4800	P43358	MAGE4	DRB1*11:03	138	152	ERVIKNYKRCFPVIF	31.71
4801	P43358	MAGE4	DRB1*15:15	120	134	FLLRKYRAKELVTKA	31.71
4802	P43358	MAGE4	DRB1*01:20	194	208	NQIFPKTGLLIIVLG	31.90
4803	P43358	MAGE4	DRB1*11:42	282	296	TSYVKVLEHVVRVNA	32.24
4804	P43358	MAGE4	DRB1*01:18	194	208	NQIFPKTGLLIIVLG	32.27
4805	P43358	MAGE4	DRB1*01:20	288	302	LEHVVRVNARVRIAY	32.28
4806	P43358	MAGE4	DRB1*01:11	155	169	ASESLKMIFGIDVKE	32.49
4807	P43358	MAGE4	DRB1*01:18	148	162	FPVIFGKASESLKMI	32.50
4808	P43358	MAGE4	DRB1*13:96	290	304	HVVRVNARVRIAYPS	32.56

4809	P43358	MAGE4	DRB1*01:20	105	119	LFREALSNKVDELAH	32.68
4810	P43358	MAGE4	DRB1*11:02	136	150	MLERVIKNYKRCFPV	32.77
4811	P43358	MAGE4	DRB1*11:65	136	150	MLERVIKNYKRCFPV	32.77
4812	P43358	MAGE4	DRB1*13:01	136	150	MLERVIKNYKRCFPV	32.77
4813	P43358	MAGE4	DRB1*11:14	291	305	VVRVNARVRIAYPSL	32.87
4814	P43358	MAGE4	DRB1*13:02	291	305	VVRVNARVRIAYPSL	32.87
4815	P43358	MAGE4	DRB1*13:23	291	305	VVRVNARVRIAYPSL	32.87
4816	P43358	MAGE4	DRB1*13:97	291	305	VVRVNARVRIAYPSL	32.87
4817	P43358	MAGE4	DRB1*03:11	290	304	HVVRVNARVRIAYPS	33.12
4818	P43358	MAGE4	DRB1*11:13	282	296	TSYVKVLEHVVRVNA	33.19
4819	P43358	MAGE4	DRB1*01:20	269	283	RYEFLWGPRALAETS	33.24
4820	P43358	MAGE4	DRB1*15:07	117	131	LAHFLLRKYRAKELV	33.39
4821	P43358	MAGE4	DRB1*01:02	154	168	KASESLKMIFGIDVK	33.50
4822	P43358	MAGE4	DRB1*11:03	135	149	EMLERVIKNYKRCFP	33.57
4823	P43358	MAGE4	DRB1*11:01	268	282	ARYEFLWGPRALAET	33.84
4824	P43358	MAGE4	DRB1*11:10	268	282	ARYEFLWGPRALAET	33.84
4825	P43358	MAGE4	DRB1*11:12	268	282	ARYEFLWGPRALAET	33.84
4826	P43358	MAGE4	DRB1*11:28	268	282	ARYEFLWGPRALAET	33.84
4827	P43358	MAGE4	DRB1*11:29	268	282	ARYEFLWGPRALAET	33.84
4828	P43358	MAGE4	DRB1*11:42	294	308	VNARVRIAYPSLREA	33.84
4829	P43358	MAGE4	DRB1*11:49	268	282	ARYEFLWGPRALAET	33.84
4830	P43358	MAGE4	DRB1*11:62	268	282	ARYEFLWGPRALAET	33.84
4831	P43358	MAGE4	DRB1*11:74	268	282	ARYEFLWGPRALAET	33.84
4832	P43358	MAGE4	DRB1*13:05	268	282	ARYEFLWGPRALAET	33.84
4833	P43358	MAGE4	DRB1*13:14	268	282	ARYEFLWGPRALAET	33.84
4834	P43358	MAGE4	DRB1*13:50	268	282	ARYEFLWGPRALAET	33.84
4835	P43358	MAGE4	DRB1*16:01	103	117	ESLFREALSNKVDEL	33.94
4836	P43358	MAGE4	DRB1*01:20	119	133	HFLLRKYRAKELVTK	33.95
4837	P43358	MAGE4	DRB1*15:37	119	133	HFLLRKYRAKELVTK	33.99
4838	P43358	MAGE4	DRB1*11:84	285	299	VKVLEHVVRVNARVR	34.22
4839	P43358	MAGE4	DRB1*08:04	285	299	VKVLEHVVRVNARVR	34.31
4840	P43358	MAGE4	DRB1*11:02	116	130	ELAHFLLRKYRAKEL	34.56
4841	P43358	MAGE4	DRB1*11:65	116	130	ELAHFLLRKYRAKEL	34.56
4842	P43358	MAGE4	DRB1*13:01	116	130	ELAHFLLRKYRAKEL	34.56
4843	P43358	MAGE4	DRB1*08:04	286	300	KVLEHVVRVNARVRI	34.75
4844	P43358	MAGE4	DRB1*11:01	269	283	RYEFLWGPRALAETS	34.81
4845	P43358	MAGE4	DRB1*11:10	269	283	RYEFLWGPRALAETS	34.81
4846	P43358	MAGE4	DRB1*11:12	269	283	RYEFLWGPRALAETS	34.81
4847	P43358	MAGE4	DRB1*11:28	269	283	RYEFLWGPRALAETS	34.81
4848	P43358	MAGE4	DRB1*11:29	269	283	RYEFLWGPRALAETS	34.81
4849	P43358	MAGE4	DRB1*11:49	269	283	RYEFLWGPRALAETS	34.81
4850	P43358	MAGE4	DRB1*11:62	269	283	RYEFLWGPRALAETS	34.81
4851	P43358	MAGE4	DRB1*11:74	269	283	RYEFLWGPRALAETS	34.81

4852	P43358	MAGE4	DRB1*13:05	269	283	RYEFLWGPRALAETS	34.81
4853	P43358	MAGE4	DRB1*13:14	269	283	RYEFLWGPRALAETS	34.81
4854	P43358	MAGE4	DRB1*13:50	269	283	RYEFLWGPRALAETS	34.81
4855	P43358	MAGE4	DRB1*15:03	116	130	ELAHFLLRKYRAKEL	34.91
4856	P43358	MAGE4	DRB1*11:42	296	310	ARVRIAYPSLREAAL	35.02
4857	P43358	MAGE4	DRB1*11:42	118	132	AHFLLRKYRAKELVT	35.07
4858	P43358	MAGE4	DRB1*11:42	134	148	AEMLERVIKNYKRCF	35.25
4859	P43358	MAGE4	DRB1*01:20	294	308	VNARVRIAYPSLREA	35.36
4860	P43358	MAGE4	DRB1*01:24	158	172	SLKMIFGIDVKEVDP	35.57
4861	P43358	MAGE4	DRB1*11:04	289	303	EHVVRVNARVRIAYP	35.82
4862	P43358	MAGE4	DRB1*11:46	289	303	EHVVRVNARVRIAYP	35.82
4863	P43358	MAGE4	DRB1*11:58	289	303	EHVVRVNARVRIAYP	35.82
4864	P43358	MAGE4	DRB1*13:11	289	303	EHVVRVNARVRIAYP	35.82
4865	P43358	MAGE4	DRB1*11:02	118	132	AHFLLRKYRAKELVT	35.84
4866	P43358	MAGE4	DRB1*11:65	118	132	AHFLLRKYRAKELVT	35.84
4867	P43358	MAGE4	DRB1*13:01	118	132	AHFLLRKYRAKELVT	35.84
4868	P43358	MAGE4	DRB1*11:02	138	152	ERVIKNYKRCFPVIF	35.86
4869	P43358	MAGE4	DRB1*11:65	138	152	ERVIKNYKRCFPVIF	35.86
4870	P43358	MAGE4	DRB1*13:01	138	152	ERVIKNYKRCFPVIF	35.86
4871	P43358	MAGE4	DRB1*11:42	292	306	VRVNARVRIAYPSLR	36.00
4872	P43358	MAGE4	DRB1*11:84	286	300	KVLEHVVRVNARVRI	36.07
4873	P43358	MAGE4	DRB1*11:42	132	146	TKAEMLERVIKNYKR	36.32
4874	P43358	MAGE4	DRB1*01:20	265	279	SNPARYEFLWGPRAL	36.34
4875	P43358	MAGE4	DRB1*11:02	295	309	NARVRIAYPSLREAA	36.41
4876	P43358	MAGE4	DRB1*11:65	295	309	NARVRIAYPSLREAA	36.41
4877	P43358	MAGE4	DRB1*13:01	295	309	NARVRIAYPSLREAA	36.41
4878	P43358	MAGE4	DRB1*11:42	137	151	LERVIKNYKRCFPVI	36.49
4879	P43358	MAGE4	DRB1*14:12	288	302	LEHVVRVNARVRIAY	36.53
4880	P43358	MAGE4	DRB1*11:42	293	307	RVNARVRIAYPSLRE	36.60
4881	P43358	MAGE4	DRB1*16:09	266	280	NPARYEFLWGPRALA	36.73
4882	P43358	MAGE4	DRB1*01:20	287	301	VLEHVVRVNARVRIA	36.74
4883	P43358	MAGE4	DRB1*11:03	293	307	RVNARVRIAYPSLRE	36.79
4884	P43358	MAGE4	DRB1*11:13	292	306	VRVNARVRIAYPSLR	36.81
4885	P43358	MAGE4	DRB1*11:03	134	148	AEMLERVIKNYKRCF	36.93
4886	P43358	MAGE4	DRB1*01:01	193	207	NNQIFPKTGLLIIVL	37.00
4887	P43358	MAGE4	DRB1*11:02	294	308	VNARVRIAYPSLREA	37.07
4888	P43358	MAGE4	DRB1*11:65	294	308	VNARVRIAYPSLREA	37.07
4889	P43358	MAGE4	DRB1*13:01	294	308	VNARVRIAYPSLREA	37.07
4890	P43358	MAGE4	DRB1*01:11	157	171	ESLKMIFGIDVKEVD	37.13
4891	P43358	MAGE4	DRB1*01:01	148	162	FPVIFGKASESLKMI	37.17
4892	P43358	MAGE4	DRB1*16:01	104	118	SLFREALSNKVDELA	37.34
4893	P43358	MAGE4	DRB1*13:21	114	128	VDELAHFLLRKYRAK	37.58
4894	P43358	MAGE4	DRB1*11:42	136	150	MLERVIKNYKRCFPV	37.63

4895	P43358	MAGE4	DRB1*11:03	120	134	FLLRKYRAKELVTKA	37.77
4896	P43358	MAGE4	DRB1*11:01	267	281	PARYEFLWGPRALAE	37.79
4897	P43358	MAGE4	DRB1*11:10	267	281	PARYEFLWGPRALAE	37.79
4898	P43358	MAGE4	DRB1*11:12	267	281	PARYEFLWGPRALAE	37.79
4899	P43358	MAGE4	DRB1*11:28	267	281	PARYEFLWGPRALAE	37.79
4900	P43358	MAGE4	DRB1*11:29	267	281	PARYEFLWGPRALAE	37.79
4901	P43358	MAGE4	DRB1*11:49	267	281	PARYEFLWGPRALAE	37.79
4902	P43358	MAGE4	DRB1*11:62	267	281	PARYEFLWGPRALAE	37.79
4903	P43358	MAGE4	DRB1*11:74	267	281	PARYEFLWGPRALAE	37.79
4904	P43358	MAGE4	DRB1*13:05	267	281	PARYEFLWGPRALAE	37.79
4905	P43358	MAGE4	DRB1*13:14	267	281	PARYEFLWGPRALAE	37.79
4906	P43358	MAGE4	DRB1*13:50	267	281	PARYEFLWGPRALAE	37.79
4907	P43358	MAGE4	DRB1*13:21	119	133	HFLLRKYRAKELVTK	37.82
4908	P43358	MAGE4	DRB1*14:32	291	305	VVRVNARVRIAYPSL	37.95
4909	P43358	MAGE4	DRB1*11:02	119	133	HFLLRKYRAKELVTK	38.09
4910	P43358	MAGE4	DRB1*11:65	119	133	HFLLRKYRAKELVTK	38.09
4911	P43358	MAGE4	DRB1*13:01	119	133	HFLLRKYRAKELVTK	38.09
4912	P43358	MAGE4	DRB1*16:09	268	282	ARYEFLWGPRALAET	38.11
4913	P43358	MAGE4	DRB1*03:11	295	309	NARVRIAYPSLREAA	38.19
4914	P43358	MAGE4	DRB1*15:37	120	134	FLLRKYRAKELVTKA	38.24
4915	P43358	MAGE4	DRB1*01:01	141	155	IKNYKRCFPVIFGKA	38.57
4916	P43358	MAGE4	DRB1*01:18	153	167	GKASESLKMIFGIDV	38.59
4917	P43358	MAGE4	DRB1*16:09	267	281	PARYEFLWGPRALAE	38.73
4918	P43358	MAGE4	DRB1*11:04	119	133	HFLLRKYRAKELVTK	38.75
4919	P43358	MAGE4	DRB1*11:46	119	133	HFLLRKYRAKELVTK	38.75
4920	P43358	MAGE4	DRB1*11:58	119	133	HFLLRKYRAKELVTK	38.75
4921	P43358	MAGE4	DRB1*13:11	119	133	HFLLRKYRAKELVTK	38.75
4922	P43358	MAGE4	DRB1*01:20	118	132	AHFLLRKYRAKELVT	38.78
4923	P43358	MAGE4	DRB1*15:37	118	132	AHFLLRKYRAKELVT	38.82
4924	P43358	MAGE4	DRB1*01:11	265	279	SNPARYEFLWGPRAL	38.87
4925	P43358	MAGE4	DRB1*16:02	105	119	LFREALSNKVDELAH	38.98
4926	P43358	MAGE4	DRB1*11:01	115	129	DELAHFLLRKYRAKE	39.02
4927	P43358	MAGE4	DRB1*11:10	115	129	DELAHFLLRKYRAKE	39.02
4928	P43358	MAGE4	DRB1*11:12	115	129	DELAHFLLRKYRAKE	39.02
4929	P43358	MAGE4	DRB1*11:28	115	129	DELAHFLLRKYRAKE	39.02
4930	P43358	MAGE4	DRB1*11:29	115	129	DELAHFLLRKYRAKE	39.02
4931	P43358	MAGE4	DRB1*11:49	115	129	DELAHFLLRKYRAKE	39.02
4932	P43358	MAGE4	DRB1*11:62	115	129	DELAHFLLRKYRAKE	39.02
4933	P43358	MAGE4	DRB1*11:74	115	129	DELAHFLLRKYRAKE	39.02
4934	P43358	MAGE4	DRB1*13:05	115	129	DELAHFLLRKYRAKE	39.02
4935	P43358	MAGE4	DRB1*13:14	115	129	DELAHFLLRKYRAKE	39.02
4936	P43358	MAGE4	DRB1*13:50	115	129	DELAHFLLRKYRAKE	39.02
4937	P43358	MAGE4	DRB1*15:03	121	135	LLRKYRAKELVTKAE	39.05

4938	P43358	MAGE4	DRB1*15:15	103	117	ESLFREALSNKVDEL	39.09
4939	P43358	MAGE4	DRB1*01:18	270	284	YEFLWGPRALAETSY	39.13
4940	P43358	MAGE4	DRB1*01:18	119	133	HFLLRKYRAKELVTK	39.26
4941	P43358	MAGE4	DRB1*16:01	266	280	NPARYEFLWGPRALA	39.27
4942	P43358	MAGE4	DRB1*15:15	266	280	NPARYEFLWGPRALA	39.35
4943	P43358	MAGE4	DRB1*01:20	153	167	GKASESLKMIFGIDV	39.40
4944	P43358	MAGE4	DRB1*13:96	286	300	KVLEHVVRVNARVRI	39.49
4945	P43358	MAGE4	DRB1*01:20	191	205	LGNNQIFPKTGLLII	39.66
4946	P43358	MAGE4	DRB1*01:01	282	296	TSYVKVLEHVVRVNA	39.69
4947	P43358	MAGE4	DRB1*01:20	120	134	FLLRKYRAKELVTKA	39.88
4948	P43358	MAGE4	DRB1*15:01	156	170	SESLKMIFGIDVKEV	39.91
4949	P43358	MAGE4	DRB1*15:06	156	170	SESLKMIFGIDVKEV	39.91
4950	P43358	MAGE4	DRB1*11:04	290	304	HVVRVNARVRIAYPS	39.92
4951	P43358	MAGE4	DRB1*11:46	290	304	HVVRVNARVRIAYPS	39.92
4952	P43358	MAGE4	DRB1*11:58	290	304	HVVRVNARVRIAYPS	39.92
4953	P43358	MAGE4	DRB1*13:11	290	304	HVVRVNARVRIAYPS	39.92
4954	P43358	MAGE4	DRB1*03:11	286	300	KVLEHVVRVNARVRI	40.02
4955	P43358	MAGE4	DRB1*16:02	100	114	PDAESLFREALSNKV	40.04
4956	P43358	MAGE4	DRB1*01:18	147	161	CFPVIFGKASESLKM	40.20
4957	P43358	MAGE4	DRB1*01:18	142	156	KNYKRCFPVIFGKAS	40.27
4958	P43358	MAGE4	DRB1*01:18	140	154	VIKNYKRCFPVIFGK	40.29
4959	P43358	MAGE4	DRB1*11:01	284	298	YVKVLEHVVRVNARV	40.41
4960	P43358	MAGE4	DRB1*11:10	284	298	YVKVLEHVVRVNARV	40.41
4961	P43358	MAGE4	DRB1*11:12	284	298	YVKVLEHVVRVNARV	40.41
4962	P43358	MAGE4	DRB1*11:28	284	298	YVKVLEHVVRVNARV	40.41
4963	P43358	MAGE4	DRB1*11:29	284	298	YVKVLEHVVRVNARV	40.41
4964	P43358	MAGE4	DRB1*11:49	284	298	YVKVLEHVVRVNARV	40.41
4965	P43358	MAGE4	DRB1*11:62	284	298	YVKVLEHVVRVNARV	40.41
4966	P43358	MAGE4	DRB1*11:74	284	298	YVKVLEHVVRVNARV	40.41
4967	P43358	MAGE4	DRB1*13:05	284	298	YVKVLEHVVRVNARV	40.41
4968	P43358	MAGE4	DRB1*13:14	284	298	YVKVLEHVVRVNARV	40.41
4969	P43358	MAGE4	DRB1*13:50	284	298	YVKVLEHVVRVNARV	40.41
4970	P43358	MAGE4	DRB1*11:02	296	310	ARVRIAYPSLREAAL	40.70
4971	P43358	MAGE4	DRB1*11:65	296	310	ARVRIAYPSLREAAL	40.70
4972	P43358	MAGE4	DRB1*13:01	296	310	ARVRIAYPSLREAAL	40.70
4973	P43358	MAGE4	DRB1*11:02	135	149	EMLERVIKNYKRCFP	40.71
4974	P43358	MAGE4	DRB1*11:03	113	127	KVDELAHFLLRKYRA	40.71
4975	P43358	MAGE4	DRB1*11:65	135	149	EMLERVIKNYKRCFP	40.71
4976	P43358	MAGE4	DRB1*13:01	135	149	EMLERVIKNYKRCFP	40.71
4977	P43358	MAGE4	DRB1*11:42	135	149	EMLERVIKNYKRCFP	40.72
4978	P43358	MAGE4	DRB1*16:01	267	281	PARYEFLWGPRALAE	40.85
4979	P43358	MAGE4	DRB1*11:13	295	309	NARVRIAYPSLREAA	40.95
4980	P43358	MAGE4	DRB1*16:02	266	280	NPARYEFLWGPRALA	41.06

4981	P43358	MAGE4	DRB1*16:05	103	117	ESLFREALSNKVDEL	41.35
4982	P43358	MAGE4	DRB1*01:20	117	131	LAHFLLRKYRAKELV	41.54
4983	P43358	MAGE4	DRB1*01:01	281	295	ETSYVKVLEHVVRVN	41.60
4984	P43358	MAGE4	DRB1*01:20	297	311	RVRIAYPSLREAALL	41.60
4985	P43358	MAGE4	DRB1*15:15	104	118	SLFREALSNKVDELA	42.09
4986	P43358	MAGE4	DRB1*16:01	268	282	ARYEFLWGPRALAET	42.20
4987	P43358	MAGE4	DRB1*11:01	118	132	AHFLLRKYRAKELVT	42.25
4988	P43358	MAGE4	DRB1*11:10	118	132	AHFLLRKYRAKELVT	42.25
4989	P43358	MAGE4	DRB1*11:12	118	132	AHFLLRKYRAKELVT	42.25
4990	P43358	MAGE4	DRB1*11:28	118	132	AHFLLRKYRAKELVT	42.25
4991	P43358	MAGE4	DRB1*11:29	118	132	AHFLLRKYRAKELVT	42.25
4992	P43358	MAGE4	DRB1*11:49	118	132	AHFLLRKYRAKELVT	42.25
4993	P43358	MAGE4	DRB1*11:62	118	132	AHFLLRKYRAKELVT	42.25
4994	P43358	MAGE4	DRB1*11:74	118	132	AHFLLRKYRAKELVT	42.25
4995	P43358	MAGE4	DRB1*13:05	118	132	AHFLLRKYRAKELVT	42.25
4996	P43358	MAGE4	DRB1*13:14	118	132	AHFLLRKYRAKELVT	42.25
4997	P43358	MAGE4	DRB1*13:50	118	132	AHFLLRKYRAKELVT	42.25
4998	P43358	MAGE4	DRB1*15:15	268	282	ARYEFLWGPRALAET	42.30
4999	P43358	MAGE4	DRB1*14:12	287	301	VLEHVVRVNARVRIA	42.41
5000	P43358	MAGE4	DRB1*01:01	280	294	AETSYVKVLEHVVRV	42.67
5001	P43358	MAGE4	DRB1*01:18	288	302	LEHVVRVNARVRIAY	42.69
5002	P43358	MAGE4	DRB1*14:06	285	299	VKVLEHVVRVNARVR	42.90
5003	P43358	MAGE4	DRB1*11:11	288	302	LEHVVRVNARVRIAY	42.96
5004	P43358	MAGE4	DRB1*01:11	269	283	RYEFLWGPRALAETS	42.97
5005	P43358	MAGE4	DRB1*14:06	295	309	NARVRIAYPSLREAA	42.99
5006	P43358	MAGE4	DRB1*11:01	114	128	VDELAHFLLRKYRAK	43.08
5007	P43358	MAGE4	DRB1*11:10	114	128	VDELAHFLLRKYRAK	43.08
5008	P43358	MAGE4	DRB1*11:12	114	128	VDELAHFLLRKYRAK	43.08
5009	P43358	MAGE4	DRB1*11:28	114	128	VDELAHFLLRKYRAK	43.08
5010	P43358	MAGE4	DRB1*11:29	114	128	VDELAHFLLRKYRAK	43.08
5011	P43358	MAGE4	DRB1*11:49	114	128	VDELAHFLLRKYRAK	43.08
5012	P43358	MAGE4	DRB1*11:62	114	128	VDELAHFLLRKYRAK	43.08
5013	P43358	MAGE4	DRB1*11:74	114	128	VDELAHFLLRKYRAK	43.08
5014	P43358	MAGE4	DRB1*13:05	114	128	VDELAHFLLRKYRAK	43.08
5015	P43358	MAGE4	DRB1*13:14	114	128	VDELAHFLLRKYRAK	43.08
5016	P43358	MAGE4	DRB1*13:50	114	128	VDELAHFLLRKYRAK	43.08
5017	P43358	MAGE4	DRB1*13:21	268	282	ARYEFLWGPRALAET	43.18
5018	P43358	MAGE4	DRB1*11:42	113	127	KVDELAHFLLRKYRA	43.21
5019	P43358	MAGE4	DRB1*01:20	286	300	KVLEHVVRVNARVRI	43.27
5020	P43358	MAGE4	DRB1*11:84	288	302	LEHVVRVNARVRIAY	43.40
5021	P43358	MAGE4	DRB1*08:04	287	301	VLEHVVRVNARVRIA	43.42
5022	P43358	MAGE4	DRB1*11:02	134	148	AEMLERVIKNYKRCF	43.68
5023	P43358	MAGE4	DRB1*11:65	134	148	AEMLERVIKNYKRCF	43.68

5024	P43358	MAGE4	DRB1*13:01	134	148	AEMLERVIKNYKRCF	43.68
5025	P43358	MAGE4	DRB1*15:15	267	281	PARYEFLWGPRALAE	43.71
5026	P43358	MAGE4	DRB1*01:20	293	307	RVNARVRIAYPSLRE	43.79
5027	P43358	MAGE4	DRB1*01:11	154	168	KASESLKMIFGIDVK	43.85
5028	P43358	MAGE4	DRB1*11:13	293	307	RVNARVRIAYPSLRE	43.96
5029	P43358	MAGE4	DRB1*15:37	117	131	LAHFLLRKYRAKELV	44.07
5030	P43358	MAGE4	DRB1*01:01	192	206	GNNQIFPKTGLLIIV	44.30
5031	P43358	MAGE4	DRB1*11:42	133	147	KAEMLERVIKNYKRC	44.35
5032	P43358	MAGE4	DRB1*13:96	291	305	VVRVNARVRIAYPSL	44.43
5033	P43358	MAGE4	DRB1*07:01	156	170	SESLKMIFGIDVKEV	44.46
5034	P43358	MAGE4	DRB1*12:02	114	128	VDELAHFLLRKYRAK	44.46
5035	P43358	MAGE4	DRB1*01:01	147	161	CFPVIFGKASESLKM	44.50
5036	P43358	MAGE4	DRB1*03:11	294	308	VNARVRIAYPSLREA	44.53
5037	P43358	MAGE4	DRB1*01:18	279	293	LAETSYVKVLEHVVR	44.62
5038	P43358	MAGE4	DRB1*01:20	100	114	PDAESLFREALSNKV	44.71
5039	P43358	MAGE4	DRB1*08:04	284	298	YVKVLEHVVRVNARV	44.75
5040	P43358	MAGE4	DRB1*01:18	118	132	AHFLLRKYRAKELVT	44.84
5041	P43358	MAGE4	DRB1*11:03	284	298	YVKVLEHVVRVNARV	45.05
5042	P43358	MAGE4	DRB1*11:04	282	296	TSYVKVLEHVVRVNA	45.07
5043	P43358	MAGE4	DRB1*11:46	282	296	TSYVKVLEHVVRVNA	45.07
5044	P43358	MAGE4	DRB1*11:58	282	296	TSYVKVLEHVVRVNA	45.07
5045	P43358	MAGE4	DRB1*13:11	282	296	TSYVKVLEHVVRVNA	45.07
5046	P43358	MAGE4	DRB1*15:02	103	117	ESLFREALSNKVDEL	45.16
5047	P43358	MAGE4	DRB1*16:02	267	281	PARYEFLWGPRALAE	45.18
5048	P43358	MAGE4	DRB1*01:02	158	172	SLKMIFGIDVKEVDP	45.29
5049	P43358	MAGE4	DRB1*01:01	106	120	FREALSNKVDELAHF	45.35
5050	P43358	MAGE4	DRB1*11:04	113	127	KVDELAHFLLRKYRA	45.38
5051	P43358	MAGE4	DRB1*11:46	113	127	KVDELAHFLLRKYRA	45.38
5052	P43358	MAGE4	DRB1*11:58	113	127	KVDELAHFLLRKYRA	45.38
5053	P43358	MAGE4	DRB1*13:11	113	127	KVDELAHFLLRKYRA	45.38
5054	P43358	MAGE4	DRB1*11:08	288	302	LEHVVRVNARVRIAY	45.51
5055	P43358	MAGE4	DPB1*33:01	273	287	LWGPRALAETSYVKV	45.55
5056	P43358	MAGE4	DPB1*71:01	273	287	LWGPRALAETSYVKV	45.55
5057	P43358	MAGE4	DRB1*01:20	289	303	EHVVRVNARVRIAYP	45.61
5058	P43358	MAGE4	DRB1*01:20	146	160	RCFPVIFGKASESLK	45.62
5059	P43358	MAGE4	DRB1*13:21	267	281	PARYEFLWGPRALAE	45.62
5060	P43358	MAGE4	DRB1*16:02	268	282	ARYEFLWGPRALAET	45.70
5061	P43358	MAGE4	DRB1*01:18	191	205	LGNNQIFPKTGLLII	45.79
5062	P43358	MAGE4	DRB1*01:20	149	163	PVIFGKASESLKMIF	45.82
5063	P43358	MAGE4	DRB1*14:06	296	310	ARVRIAYPSLREAAL	45.97
5064	P43358	MAGE4	DRB1*11:08	286	300	KVLEHVVRVNARVRI	46.09
5065	P43358	MAGE4	DRB1*14:32	285	299	VKVLEHVVRVNARVR	46.12
5066	P43358	MAGE4	DRB1*01:01	270	284	YEFLWGPRALAETSY	46.25

5067	P43358	MAGE4	DRB1*11:14	103	117	ESLFREALSNKVDEL	46.34
5068	P43358	MAGE4	DRB1*13:02	103	117	ESLFREALSNKVDEL	46.34
5069	P43358	MAGE4	DRB1*13:23	103	117	ESLFREALSNKVDEL	46.34
5070	P43358	MAGE4	DRB1*13:97	103	117	ESLFREALSNKVDEL	46.34
5071	P43358	MAGE4	DRB1*14:12	289	303	EHVVRVNARVRIAYP	46.45
5072	P43358	MAGE4	DRB1*11:03	139	153	RVIKNYKRCFPVIFG	46.58
5073	P43358	MAGE4	DRB1*11:42	138	152	ERVIKNYKRCFPVIF	46.84
5074	P43358	MAGE4	DRB1*14:12	286	300	KVLEHVVRVNARVRI	46.98
5075	P43358	MAGE4	DRB1*11:42	119	133	HFLLRKYRAKELVTK	47.03
5076	P43358	MAGE4	DRB1*11:13	294	308	VNARVRIAYPSLREA	47.15
5077	P43358	MAGE4	DRB1*11:84	287	301	VLEHVVRVNARVRIA	47.19
5078	P43358	MAGE4	DRB1*11:11	117	131	LAHFLLRKYRAKELV	47.23
5079	P43358	MAGE4	DRB1*10:01	156	170	SESLKMIFGIDVKEV	47.27
5080	P43358	MAGE4	DRB1*01:18	287	301	VLEHVVRVNARVRIA	47.38
5081	P43358	MAGE4	DRB1*15:15	116	130	ELAHFLLRKYRAKEL	47.38
5082	P43358	MAGE4	DRB1*11:04	134	148	AEMLERVIKNYKRCF	47.43
5083	P43358	MAGE4	DRB1*11:46	134	148	AEMLERVIKNYKRCF	47.43
5084	P43358	MAGE4	DRB1*11:58	134	148	AEMLERVIKNYKRCF	47.43
5085	P43358	MAGE4	DRB1*13:11	134	148	AEMLERVIKNYKRCF	47.43
5086	P43358	MAGE4	DRB1*11:42	281	295	ETSYVKVLEHVVRVN	47.49
5087	P43358	MAGE4	DRB1*01:18	117	131	LAHFLLRKYRAKELV	47.55
5088	P43358	MAGE4	DRB1*03:11	296	310	ARVRIAYPSLREAAL	47.56
5089	P43358	MAGE4	DRB1*12:02	116	130	ELAHFLLRKYRAKEL	47.63
5090	P43358	MAGE4	DRB1*13:21	269	283	RYEFLWGPRALAETS	47.73
5091	P43358	MAGE4	DRB1*11:01	283	297	SYVKVLEHVVRVNAR	47.81
5092	P43358	MAGE4	DRB1*11:10	283	297	SYVKVLEHVVRVNAR	47.81
5093	P43358	MAGE4	DRB1*11:12	283	297	SYVKVLEHVVRVNAR	47.81
5094	P43358	MAGE4	DRB1*11:28	283	297	SYVKVLEHVVRVNAR	47.81
5095	P43358	MAGE4	DRB1*11:29	283	297	SYVKVLEHVVRVNAR	47.81
5096	P43358	MAGE4	DRB1*11:49	283	297	SYVKVLEHVVRVNAR	47.81
5097	P43358	MAGE4	DRB1*11:62	283	297	SYVKVLEHVVRVNAR	47.81
5098	P43358	MAGE4	DRB1*11:74	283	297	SYVKVLEHVVRVNAR	47.81
5099	P43358	MAGE4	DRB1*13:05	283	297	SYVKVLEHVVRVNAR	47.81
5100	P43358	MAGE4	DRB1*13:14	283	297	SYVKVLEHVVRVNAR	47.81
5101	P43358	MAGE4	DRB1*13:50	283	297	SYVKVLEHVVRVNAR	47.81
5102	P43358	MAGE4	DRB1*08:04	288	302	LEHVVRVNARVRIAY	47.93
5103	P43358	MAGE4	DRB1*11:13	296	310	ARVRIAYPSLREAAL	48.02
5104	P43358	MAGE4	DRB1*11:03	283	297	SYVKVLEHVVRVNAR	48.09
5105	P43358	MAGE4	DRB1*16:09	103	117	ESLFREALSNKVDEL	48.25
5106	P43358	MAGE4	DRB1*01:01	283	297	SYVKVLEHVVRVNAR	48.26
5107	P43358	MAGE4	DRB1*16:01	102	116	AESLFREALSNKVDE	48.38
5108	P43358	MAGE4	DRB1*01:29	158	172	SLKMIFGIDVKEVDP	48.41
5109	P43358	MAGE4	DPB1*33:01	248	262	TQDWVQENYLEYRQV	48.55

5110	P43358	MAGE4	DPB1*71:01	248	262	TQDWVQENYLEYRQV	48.55
5111	P43358	MAGE4	DRB1*11:13	281	295	ETSYVKVLEHVVRVN	48.55
5112	P43358	MAGE4	DRB1*01:18	106	120	FREALSNKVDELAHF	48.59
5113	P43358	MAGE4	DRB1*11:42	131	145	VTKAEMLERVIKNYK	48.80
5114	P43358	MAGE4	DRB1*14:06	291	305	VVRVNARVRIAYPSL	48.86
5115	P43358	MAGE4	DRB1*01:01	194	208	NQIFPKTGLLIIVLG	48.87
5116	P43358	MAGE4	DRB1*11:04	132	146	TKAEMLERVIKNYKR	48.98
5117	P43358	MAGE4	DRB1*11:46	132	146	TKAEMLERVIKNYKR	48.98
5118	P43358	MAGE4	DRB1*11:58	132	146	TKAEMLERVIKNYKR	48.98
5119	P43358	MAGE4	DRB1*13:11	132	146	TKAEMLERVIKNYKR	48.98
5120	P43358	MAGE4	DRB1*16:05	104	118	SLFREALSNKVDELA	49.13
5121	P43358	MAGE4	DRB1*01:01	142	156	KNYKRCFPVIFGKAS	49.20
5122	P43358	MAGE4	DRB1*01:18	120	134	FLLRKYRAKELVTKA	49.35
5123	P43358	MAGE4	DRB1*11:13	116	130	ELAHFLLRKYRAKEL	49.37
5124	P43358	MAGE4	DRB1*11:08	287	301	VLEHVVRVNARVRIA	49.56
5125	P43358	MAGE4	DRB1*01:01	140	154	VIKNYKRCFPVIFGK	49.60
5126	P43358	MAGE4	DRB1*04:01	103	117	ESLFREALSNKVDEL	49.61
5127	P43358	MAGE4	DRB1*01:18	295	309	NARVRIAYPSLREAA	49.78
5128	P43358	MAGE4	DRB1*15:01	155	169	ASESLKMIFGIDVKE	49.82
5129	P43358	MAGE4	DRB1*15:06	155	169	ASESLKMIFGIDVKE	49.82
5130	P78358	NY-ESO-1	DRB1*01:18	87	101	LLEFYLAMPFATPME	7.00
5131	P78358	NY-ESO-1	DRB1*01:01	87	101	LLEFYLAMPFATPME	7.20
5132	P78358	NY-ESO-1	DRB1*01:01	88	102	LEFYLAMPFATPMEA	7.29
5133	P78358	NY-ESO-1	DRB1*01:18	88	102	LEFYLAMPFATPMEA	7.42
5134	P78358	NY-ESO-1	DRB1*10:01	87	101	LLEFYLAMPFATPME	7.81
5135	P78358	NY-ESO-1	DRB1*10:01	88	102	LEFYLAMPFATPMEA	8.00
5136	P78358	NY-ESO-1	DRB1*01:18	86	100	RLLEFYLAMPFATPM	8.28
5137	P78358	NY-ESO-1	DRB1*01:01	86	100	RLLEFYLAMPFATPM	8.84
5138	P78358	NY-ESO-1	DRB1*10:01	86	100	RLLEFYLAMPFATPM	9.31
5139	P78358	NY-ESO-1	DRB1*01:18	85	99	SRLLEFYLAMPFATP	9.50
5140	P78358	NY-ESO-1	DRB1*01:24	87	101	LLEFYLAMPFATPME	9.74
5141	P78358	NY-ESO-1	DRB1*01:01	85	99	SRLLEFYLAMPFATP	9.86
5142	P78358	NY-ESO-1	DRB1*01:01	89	103	EFYLAMPFATPMEAE	10.19
5143	P78358	NY-ESO-1	DRB1*01:18	89	103	EFYLAMPFATPMEAE	10.40
5144	P78358	NY-ESO-1	DRB1*01:24	86	100	RLLEFYLAMPFATPM	10.64
5145	P78358	NY-ESO-1	DRB1*10:01	85	99	SRLLEFYLAMPFATP	10.66
5146	P78358	NY-ESO-1	DRB1*01:01	123	137	LKEFTVSGNILTIRL	10.80
5147	P78358	NY-ESO-1	DRB1*10:01	89	103	EFYLAMPFATPMEAE	10.91
5148	P78358	NY-ESO-1	DRB1*01:24	88	102	LEFYLAMPFATPMEA	11.19
5149	P78358	NY-ESO-1	DRB1*01:18	123	137	LKEFTVSGNILTIRL	11.60
5150	P78358	NY-ESO-1	DRB1*01:29	87	101	LLEFYLAMPFATPME	11.68
5151	P78358	NY-ESO-1	DRB1*01:01	122	136	LLKEFTVSGNILTIR	11.69
5152	P78358	NY-ESO-1	DRB1*01:29	88	102	LEFYLAMPFATPMEA	12.03

5153	P78358	NY-ESO-1	DRB1*01:18	122	136	LLKEFTVSGNILTIR	12.82
5154	P78358	NY-ESO-1	DPB1*33:01	155	169	QLSLLMWITQCFLPV	12.90
5155	P78358	NY-ESO-1	DPB1*71:01	155	169	QLSLLMWITQCFLPV	12.90
5156	P78358	NY-ESO-1	DRB1*01:01	124	138	KEFTVSGNILTIRLT	12.95
5157	P78358	NY-ESO-1	DRB1*01:24	85	99	SRLLEFYLAMPFATP	13.22
5158	P78358	NY-ESO-1	DPB1*33:01	160	174	MWITQCFLPVFLAQP	13.30
5159	P78358	NY-ESO-1	DPB1*71:01	160	174	MWITQCFLPVFLAQP	13.30
5160	P78358	NY-ESO-1	DRB1*07:01	142	156	HRQLQLSISSCLQQL	13.69
5161	P78358	NY-ESO-1	DRB1*01:18	124	138	KEFTVSGNILTIRLT	13.90
5162	P78358	NY-ESO-1	DRB1*07:01	141	155	DHRQLQLSISSCLQQ	14.17
5163	P78358	NY-ESO-1	DPB1*33:01	156	170	LSLLMWITQCFLPVF	14.20
5164	P78358	NY-ESO-1	DPB1*71:01	156	170	LSLLMWITQCFLPVF	14.20
5165	P78358	NY-ESO-1	DRB1*01:29	86	100	RLLEFYLAMPFATPM	14.23
5166	P78358	NY-ESO-1	DRB1*01:11	87	101	LLEFYLAMPFATPME	14.66
5167	P78358	NY-ESO-1	DPB1*33:01	159	173	LMWITQCFLPVFLAQ	14.71
5168	P78358	NY-ESO-1	DPB1*71:01	159	173	LMWITQCFLPVFLAQ	14.71
5169	P78358	NY-ESO-1	DPB1*04:01	155	169	QLSLLMWITQCFLPV	15.36
5170	P78358	NY-ESO-1	DPB1*126:01	155	169	QLSLLMWITQCFLPV	15.36
5171	P78358	NY-ESO-1	DPB1*23:01	155	169	QLSLLMWITQCFLPV	15.36
5172	P78358	NY-ESO-1	DPB1*39:01	155	169	QLSLLMWITQCFLPV	15.36
5173	P78358	NY-ESO-1	DRB1*07:01	140	154	ADHRQLQLSISSCLQ	15.82
5174	P78358	NY-ESO-1	DRB1*01:24	123	137	LKEFTVSGNILTIRL	15.84
5175	P78358	NY-ESO-1	DRB1*01:20	142	156	HRQLQLSISSCLQQL	16.16
5176	P78358	NY-ESO-1	DRB1*01:29	85	99	SRLLEFYLAMPFATP	16.31
5177	P78358	NY-ESO-1	DPB1*04:01	156	170	LSLLMWITQCFLPVF	16.80
5178	P78358	NY-ESO-1	DPB1*126:01	156	170	LSLLMWITQCFLPVF	16.80
5179	P78358	NY-ESO-1	DPB1*23:01	156	170	LSLLMWITQCFLPVF	16.80
5180	P78358	NY-ESO-1	DPB1*39:01	156	170	LSLLMWITQCFLPVF	16.80
5181	P78358	NY-ESO-1	DRB1*01:11	88	102	LEFYLAMPFATPMEA	16.85
5182	P78358	NY-ESO-1	DPB1*33:01	85	99	SRLLEFYLAMPFATP	16.88
5183	P78358	NY-ESO-1	DPB1*71:01	85	99	SRLLEFYLAMPFATP	16.88
5184	P78358	NY-ESO-1	DRB1*01:18	142	156	HRQLQLSISSCLQQL	17.06
5185	P78358	NY-ESO-1	DRB1*01:29	89	103	EFYLAMPFATPMEAE	17.32
5186	P78358	NY-ESO-1	DRB1*01:11	86	100	RLLEFYLAMPFATPM	17.69
5187	P78358	NY-ESO-1	DRB1*01:20	141	155	DHRQLQLSISSCLQQ	18.34
5188	P78358	NY-ESO-1	DRB1*01:01	142	156	HRQLQLSISSCLQQL	18.53
5189	P78358	NY-ESO-1	DRB1*01:24	89	103	EFYLAMPFATPMEAE	18.55
5190	P78358	NY-ESO-1	DPB1*33:01	86	100	RLLEFYLAMPFATPM	18.81
5191	P78358	NY-ESO-1	DPB1*71:01	86	100	RLLEFYLAMPFATPM	18.81
5192	P78358	NY-ESO-1	DPB1*04:01	160	174	MWITQCFLPVFLAQP	19.17
5193	P78358	NY-ESO-1	DPB1*126:01	160	174	MWITQCFLPVFLAQP	19.17
5194	P78358	NY-ESO-1	DPB1*23:01	160	174	MWITQCFLPVFLAQP	19.17
5195	P78358	NY-ESO-1	DPB1*39:01	160	174	MWITQCFLPVFLAQP	19.17

5196	P78358	NY-ESO-1	DPB1*04:01	159	173	LMWITQCFLPVFLAQ	19.43
5197	P78358	NY-ESO-1	DPB1*126:01	159	173	LMWITQCFLPVFLAQ	19.43
5198	P78358	NY-ESO-1	DPB1*23:01	159	173	LMWITQCFLPVFLAQ	19.43
5199	P78358	NY-ESO-1	DPB1*39:01	159	173	LMWITQCFLPVFLAQ	19.43
5200	P78358	NY-ESO-1	DRB1*01:24	122	136	LLKEFTVSGNILTIR	19.77
5201	P78358	NY-ESO-1	DPB1*33:01	158	172	LLMWITQCFLPVFLA	19.81
5202	P78358	NY-ESO-1	DPB1*71:01	158	172	LLMWITQCFLPVFLA	19.81
5203	P78358	NY-ESO-1	DRB1*01:18	141	155	DHRQLQLSISSCLQQ	19.82
5204	P78358	NY-ESO-1	DRB1*01:20	88	102	LEFYLAMPFATPMEA	19.88
5205	P78358	NY-ESO-1	DRB1*01:20	87	101	LLEFYLAMPFATPME	20.13
5206	P78358	NY-ESO-1	DRB1*01:18	84	98	ESRLLEFYLAMPFAT	20.78
5207	P78358	NY-ESO-1	DRB1*01:01	141	155	DHRQLQLSISSCLQQ	21.04
5208	P78358	NY-ESO-1	DRB1*07:01	143	157	RQLQLSISSCLQQLS	21.18
5209	P78358	NY-ESO-1	DRB1*01:11	123	137	LKEFTVSGNILTIRL	21.27
5210	P78358	NY-ESO-1	DRB1*01:24	124	138	KEFTVSGNILTIRLT	21.45
5211	P78358	NY-ESO-1	DPB1*33:01	87	101	LLEFYLAMPFATPME	21.79
5212	P78358	NY-ESO-1	DPB1*71:01	87	101	LLEFYLAMPFATPME	21.79
5213	P78358	NY-ESO-1	DPB1*33:01	157	171	SLLMWITQCFLPVFL	21.90
5214	P78358	NY-ESO-1	DPB1*71:01	157	171	SLLMWITQCFLPVFL	21.90
5215	P78358	NY-ESO-1	DRB1*07:01	139	153	AADHRQLQLSISSCL	21.95
5216	P78358	NY-ESO-1	DPB1*33:01	83	97	PESRLLEFYLAMPFA	22.17
5217	P78358	NY-ESO-1	DPB1*71:01	83	97	PESRLLEFYLAMPFA	22.17
5218	P78358	NY-ESO-1	DRB1*01:11	85	99	SRLLEFYLAMPFATP	22.39
5219	P78358	NY-ESO-1	DPB1*33:01	82	96	GPESRLLEFYLAMPF	22.48
5220	P78358	NY-ESO-1	DPB1*71:01	82	96	GPESRLLEFYLAMPF	22.48
5221	P78358	NY-ESO-1	DRB1*01:18	121	135	VLLKEFTVSGNILTI	22.94
5222	P78358	NY-ESO-1	DPB1*72:01	155	169	QLSLLMWITQCFLPV	23.16
5223	P78358	NY-ESO-1	DPB1*33:01	88	102	LEFYLAMPFATPMEA	23.29
5224	P78358	NY-ESO-1	DPB1*71:01	88	102	LEFYLAMPFATPMEA	23.29
5225	P78358	NY-ESO-1	DRB1*01:01	121	135	VLLKEFTVSGNILTI	23.95
5226	P78358	NY-ESO-1	DRB1*01:29	123	137	LKEFTVSGNILTIRL	24.03
5227	P78358	NY-ESO-1	DRB1*01:20	86	100	RLLEFYLAMPFATPM	24.34
5228	P78358	NY-ESO-1	DRB1*01:20	143	157	RQLQLSISSCLQQLS	24.36
5229	P78358	NY-ESO-1	DRB1*04:08	123	137	LKEFTVSGNILTIRL	24.55
5230	P78358	NY-ESO-1	DRB1*01:11	122	136	LLKEFTVSGNILTIR	24.56
5231	P78358	NY-ESO-1	DRB1*01:18	143	157	RQLQLSISSCLQQLS	24.72
5232	P78358	NY-ESO-1	DPB1*33:01	84	98	ESRLLEFYLAMPFAT	25.01
5233	P78358	NY-ESO-1	DPB1*71:01	84	98	ESRLLEFYLAMPFAT	25.01
5234	P78358	NY-ESO-1	DRB1*07:01	122	136	LLKEFTVSGNILTIR	25.13
5235	P78358	NY-ESO-1	DRB1*10:01	166	180	FLPVFLAQPPSGQRR	25.38
5236	P78358	NY-ESO-1	DPB1*04:01	158	172	LLMWITQCFLPVFLA	25.52
5237	P78358	NY-ESO-1	DPB1*126:01	158	172	LLMWITQCFLPVFLA	25.52
5238	P78358	NY-ESO-1	DPB1*23:01	158	172	LLMWITQCFLPVFLA	25.52

5239	P78358	NY-ESO-1	DPB1*39:01	158	172	LLMWITQCFLPVFLA	25.52
5240	P78358	NY-ESO-1	DRB1*01:29	122	136	LLKEFTVSGNILTIR	25.64
5241	P78358	NY-ESO-1	DRB1*04:01	122	136	LLKEFTVSGNILTIR	25.71
5242	P78358	NY-ESO-1	DRB1*09:01	88	102	LEFYLAMPFATPMEA	25.81
5243	P78358	NY-ESO-1	DRB1*01:01	84	98	ESRLLEFYLAMPFAT	25.98
5244	P78358	NY-ESO-1	DRB1*04:08	122	136	LLKEFTVSGNILTIR	26.00
5245	P78358	NY-ESO-1	DPB1*33:01	161	175	WITQCFLPVFLAQPP	26.21
5246	P78358	NY-ESO-1	DPB1*71:01	161	175	WITQCFLPVFLAQPP	26.21
5247	P78358	NY-ESO-1	DRB1*04:01	123	137	LKEFTVSGNILTIRL	26.23
5248	P78358	NY-ESO-1	DRB1*01:20	85	99	SRLLEFYLAMPFATP	26.35
5249	P78358	NY-ESO-1	DRB1*10:01	84	98	ESRLLEFYLAMPFAT	26.61
5250	P78358	NY-ESO-1	DRB1*10:01	123	137	LKEFTVSGNILTIRL	27.04
5251	P78358	NY-ESO-1	DRB1*07:01	123	137	LKEFTVSGNILTIRL	27.27
5252	P78358	NY-ESO-1	DPB1*02:02	160	174	MWITQCFLPVFLAQP	27.56
5253	P78358	NY-ESO-1	DPB1*47:01	160	174	MWITQCFLPVFLAQP	27.56
5254	P78358	NY-ESO-1	DPB1*04:01	157	171	SLLMWITQCFLPVFL	27.58
5255	P78358	NY-ESO-1	DPB1*126:01	157	171	SLLMWITQCFLPVFL	27.58
5256	P78358	NY-ESO-1	DPB1*23:01	157	171	SLLMWITQCFLPVFL	27.58
5257	P78358	NY-ESO-1	DPB1*39:01	157	171	SLLMWITQCFLPVFL	27.58
5258	P78358	NY-ESO-1	DRB1*01:11	89	103	EFYLAMPFATPMEAE	27.66
5259	P78358	NY-ESO-1	DRB1*01:20	140	154	ADHRQLQLSISSCLQ	27.91
5260	P78358	NY-ESO-1	DRB1*01:01	166	180	FLPVFLAQPPSGQRR	27.93
5261	P78358	NY-ESO-1	DRB1*11:14	123	137	LKEFTVSGNILTIRL	28.04
5262	P78358	NY-ESO-1	DRB1*13:02	123	137	LKEFTVSGNILTIRL	28.04
5263	P78358	NY-ESO-1	DRB1*13:23	123	137	LKEFTVSGNILTIRL	28.04
5264	P78358	NY-ESO-1	DRB1*13:97	123	137	LKEFTVSGNILTIRL	28.04
5265	P78358	NY-ESO-1	DRB1*01:20	89	103	EFYLAMPFATPMEAE	28.21
5266	P78358	NY-ESO-1	DRB1*09:01	87	101	LLEFYLAMPFATPME	28.28
5267	P78358	NY-ESO-1	DRB1*01:11	124	138	KEFTVSGNILTIRLT	28.32
5268	P78358	NY-ESO-1	DRB1*01:18	140	154	ADHRQLQLSISSCLQ	28.35
5269	P78358	NY-ESO-1	DPB1*72:01	160	174	MWITQCFLPVFLAQP	28.46
5270	P78358	NY-ESO-1	DRB1*01:18	125	139	EFTVSGNILTIRLTA	28.77
5271	P78358	NY-ESO-1	DRB1*10:01	122	136	LLKEFTVSGNILTIR	28.93
5272	P78358	NY-ESO-1	DRB1*15:01	85	99	SRLLEFYLAMPFATP	29.01
5273	P78358	NY-ESO-1	DRB1*15:06	85	99	SRLLEFYLAMPFATP	29.01
5274	P78358	NY-ESO-1	DPB1*02:01	160	174	MWITQCFLPVFLAQP	29.27
5275	P78358	NY-ESO-1	DPB1*46:01	160	174	MWITQCFLPVFLAQP	29.27
5276	P78358	NY-ESO-1	DPB1*81:01	160	174	MWITQCFLPVFLAQP	29.27
5277	P78358	NY-ESO-1	DRB1*01:01	143	157	RQLQLSISSCLQQLS	29.30
5278	P78358	NY-ESO-1	DRB1*09:01	85	99	SRLLEFYLAMPFATP	29.41
5279	P78358	NY-ESO-1	DRB1*01:18	90	104	FYLAMPFATPMEAEL	29.67
5280	P78358	NY-ESO-1	DRB1*11:14	122	136	LLKEFTVSGNILTIR	29.99
5281	P78358	NY-ESO-1	DRB1*13:02	122	136	LLKEFTVSGNILTIR	29.99

5282	P78358	NY-ESO-1	DRB1*13:23	122	136	LLKEFTVSGNILTIR	29.99
5283	P78358	NY-ESO-1	DRB1*13:97	122	136	LLKEFTVSGNILTIR	29.99
5284	P78358	NY-ESO-1	DRB1*04:72	123	137	LKEFTVSGNILTIRL	30.17
5285	P78358	NY-ESO-1	DRB1*01:29	124	138	KEFTVSGNILTIRLT	30.74
5286	P78358	NY-ESO-1	DRB1*01:24	84	98	ESRLLEFYLAMPFAT	31.04
5287	P78358	NY-ESO-1	DRB1*01:24	142	156	HRQLQLSISSCLQQL	31.20
5288	P78358	NY-ESO-1	DPB1*02:02	159	173	LMWITQCFLPVFLAQ	31.31
5289	P78358	NY-ESO-1	DPB1*47:01	159	173	LMWITQCFLPVFLAQ	31.31
5290	P78358	NY-ESO-1	DRB1*07:01	121	135	VLLKEFTVSGNILTI	31.61
5291	P78358	NY-ESO-1	DRB1*01:20	131	145	NILTIRLTAADHRQL	31.62
5292	P78358	NY-ESO-1	DRB1*01:01	140	154	ADHRQLQLSISSCLQ	31.72
5293	P78358	NY-ESO-1	DRB1*10:01	165	179	CFLPVFLAQPPSGQR	31.83
5294	P78358	NY-ESO-1	DPB1*72:01	156	170	LSLLMWITQCFLPVF	32.31
5295	P78358	NY-ESO-1	DRB1*01:01	90	104	FYLAMPFATPMEAEL	32.49
5296	P78358	NY-ESO-1	DRB1*11:42	130	144	GNILTIRLTAADHRQ	32.64
5297	P78358	NY-ESO-1	DRB1*04:72	122	136	LLKEFTVSGNILTIR	32.69
5298	P78358	NY-ESO-1	DRB1*10:01	124	138	KEFTVSGNILTIRLT	32.78
5299	P78358	NY-ESO-1	DPB1*02:01	159	173	LMWITQCFLPVFLAQ	33.13
5300	P78358	NY-ESO-1	DPB1*46:01	159	173	LMWITQCFLPVFLAQ	33.13
5301	P78358	NY-ESO-1	DPB1*81:01	159	173	LMWITQCFLPVFLAQ	33.13
5302	P78358	NY-ESO-1	DPB1*02:02	155	169	QLSLLMWITQCFLPV	33.15
5303	P78358	NY-ESO-1	DPB1*47:01	155	169	QLSLLMWITQCFLPV	33.15
5304	P78358	NY-ESO-1	DRB1*10:01	90	104	FYLAMPFATPMEAEL	33.15
5305	P78358	NY-ESO-1	DRB1*01:01	125	139	EFTVSGNILTIRLTA	33.16
5306	P78358	NY-ESO-1	DRB1*09:01	89	103	EFYLAMPFATPMEAE	33.24
5307	P78358	NY-ESO-1	DRB1*04:01	124	138	KEFTVSGNILTIRLT	33.31
5308	P78358	NY-ESO-1	DRB1*01:29	142	156	HRQLQLSISSCLQQL	33.39
5309	P78358	NY-ESO-1	DRB1*01:20	130	144	GNILTIRLTAADHRQ	33.57
5310	P78358	NY-ESO-1	DRB1*11:42	129	143	SGNILTIRLTAADHR	33.74
5311	P78358	NY-ESO-1	DRB1*01:20	166	180	FLPVFLAQPPSGQRR	33.75
5312	P78358	NY-ESO-1	DPB1*15:01	83	97	PESRLLEFYLAMPFA	33.96
5313	P78358	NY-ESO-1	DRB1*07:01	124	138	KEFTVSGNILTIRLT	33.96
5314	P78358	NY-ESO-1	DRB1*15:01	86	100	RLLEFYLAMPFATPM	34.19
5315	P78358	NY-ESO-1	DRB1*15:06	86	100	RLLEFYLAMPFATPM	34.19
5316	P78358	NY-ESO-1	DPB1*02:01	155	169	QLSLLMWITQCFLPV	34.36
5317	P78358	NY-ESO-1	DPB1*46:01	155	169	QLSLLMWITQCFLPV	34.36
5318	P78358	NY-ESO-1	DPB1*81:01	155	169	QLSLLMWITQCFLPV	34.36
5319	P78358	NY-ESO-1	DRB1*09:01	86	100	RLLEFYLAMPFATPM	34.37
5320	P78358	NY-ESO-1	DRB1*01:18	166	180	FLPVFLAQPPSGQRR	34.40
5321	P78358	NY-ESO-1	DPB1*02:02	156	170	LSLLMWITQCFLPVF	34.46
5322	P78358	NY-ESO-1	DPB1*47:01	156	170	LSLLMWITQCFLPVF	34.46
5323	P78358	NY-ESO-1	DRB1*04:08	124	138	KEFTVSGNILTIRLT	34.63
5324	P78358	NY-ESO-1	DRB1*01:20	129	143	SGNILTIRLTAADHR	34.64

5325	P78358	NY-ESO-1	DPB1*15:01	82	96	GPESRLLEFYLAMPF	34.85
5326	P78358	NY-ESO-1	DPB1*33:01	89	103	EFYLAMPFATPMEAE	34.98
5327	P78358	NY-ESO-1	DPB1*71:01	89	103	EFYLAMPFATPMEAE	34.98
5328	P78358	NY-ESO-1	DRB1*09:01	141	155	DHRQLQLSISSCLQQ	35.68
5329	P78358	NY-ESO-1	DRB1*01:20	132	146	ILTIRLTAADHRQLQ	36.07
5330	P78358	NY-ESO-1	DRB1*09:01	142	156	HRQLQLSISSCLQQL	36.07
5331	P78358	NY-ESO-1	DPB1*15:01	85	99	SRLLEFYLAMPFATP	36.24
5332	P78358	NY-ESO-1	DRB1*15:01	84	98	ESRLLEFYLAMPFAT	36.32
5333	P78358	NY-ESO-1	DRB1*15:06	84	98	ESRLLEFYLAMPFAT	36.32
5334	P78358	NY-ESO-1	DRB1*11:14	124	138	KEFTVSGNILTIRLT	36.33
5335	P78358	NY-ESO-1	DRB1*13:02	124	138	KEFTVSGNILTIRLT	36.33
5336	P78358	NY-ESO-1	DRB1*13:23	124	138	KEFTVSGNILTIRLT	36.33
5337	P78358	NY-ESO-1	DRB1*13:97	124	138	KEFTVSGNILTIRLT	36.33
5338	P78358	NY-ESO-1	DRB1*01:20	123	137	LKEFTVSGNILTIRL	36.40
5339	P78358	NY-ESO-1	DRB1*11:14	142	156	HRQLQLSISSCLQQL	36.44
5340	P78358	NY-ESO-1	DRB1*13:02	142	156	HRQLQLSISSCLQQL	36.44
5341	P78358	NY-ESO-1	DRB1*13:23	142	156	HRQLQLSISSCLQQL	36.44
5342	P78358	NY-ESO-1	DRB1*13:97	142	156	HRQLQLSISSCLQQL	36.44
5343	P78358	NY-ESO-1	DRB1*11:42	131	145	NILTIRLTAADHRQL	36.81
5344	P78358	NY-ESO-1	DRB1*11:42	132	146	ILTIRLTAADHRQLQ	36.90
5345	P78358	NY-ESO-1	DRB1*01:24	121	135	VLLKEFTVSGNILTI	36.91
5346	P78358	NY-ESO-1	DPB1*02:01	156	170	LSLLMWITQCFLPVF	36.97
5347	P78358	NY-ESO-1	DPB1*46:01	156	170	LSLLMWITQCFLPVF	36.97
5348	P78358	NY-ESO-1	DPB1*81:01	156	170	LSLLMWITQCFLPVF	36.97
5349	P78358	NY-ESO-1	DRB1*16:02	123	137	LKEFTVSGNILTIRL	37.07
5350	P78358	NY-ESO-1	DRB1*01:02	142	156	HRQLQLSISSCLQQL	37.14
5351	P78358	NY-ESO-1	DPB1*72:01	159	173	LMWITQCFLPVFLAQ	37.97
5352	P78358	NY-ESO-1	DRB1*16:02	122	136	LLKEFTVSGNILTIR	38.47
5353	P78358	NY-ESO-1	DRB1*07:01	120	134	GVLLKEFTVSGNILT	38.50
5354	P78358	NY-ESO-1	DPB1*33:01	81	95	RGPESRLLEFYLAMP	38.52
5355	P78358	NY-ESO-1	DPB1*71:01	81	95	RGPESRLLEFYLAMP	38.52
5356	P78358	NY-ESO-1	DRB1*01:29	141	155	DHRQLQLSISSCLQQ	38.66
5357	P78358	NY-ESO-1	DRB1*01:20	122	136	LLKEFTVSGNILTIR	39.87
5358	P78358	NY-ESO-1	DPB1*01:01	160	174	MWITQCFLPVFLAQP	40.19
5359	P78358	NY-ESO-1	DRB1*01:29	84	98	ESRLLEFYLAMPFAT	40.29
5360	P78358	NY-ESO-1	DRB1*03:11	133	147	LTIRLTAADHRQLQL	40.34
5361	P78358	NY-ESO-1	DRB1*01:24	141	155	DHRQLQLSISSCLQQ	40.38
5362	P78358	NY-ESO-1	DRB1*11:13	132	146	ILTIRLTAADHRQLQ	40.41
5363	P78358	NY-ESO-1	DRB1*13:96	123	137	LKEFTVSGNILTIRL	40.52
5364	P78358	NY-ESO-1	DRB1*01:11	142	156	HRQLQLSISSCLQQL	40.53
5365	P78358	NY-ESO-1	DRB1*01:20	124	138	KEFTVSGNILTIRLT	40.53
5366	P78358	NY-ESO-1	DRB1*11:13	131	145	NILTIRLTAADHRQL	40.62
5367	P78358	NY-ESO-1	DRB1*01:20	96	110	FATPMEAELARRSLA	40.84

5368	P78358	NY-ESO-1	DRB1*04:72	124	138	KEFTVSGNILTIRLT	41.00
5369	P78358	NY-ESO-1	DPB1*15:01	159	173	LMWITQCFLPVFLAQ	41.42
5370	P78358	NY-ESO-1	DRB1*01:18	120	134	GVLLKEFTVSGNILT	41.65
5371	P78358	NY-ESO-1	DPB1*72:01	85	99	SRLLEFYLAMPFATP	41.92
5372	P78358	NY-ESO-1	DRB1*01:01	165	179	CFLPVFLAQPPSGQR	41.92
5373	P78358	NY-ESO-1	DRB1*09:01	140	154	ADHRQLQLSISSCLQ	42.19
5374	P78358	NY-ESO-1	DPB1*01:01	85	99	SRLLEFYLAMPFATP	42.44
5375	P78358	NY-ESO-1	DRB1*11:14	141	155	DHRQLQLSISSCLQQ	42.47
5376	P78358	NY-ESO-1	DRB1*13:02	141	155	DHRQLQLSISSCLQQ	42.47
5377	P78358	NY-ESO-1	DRB1*13:23	141	155	DHRQLQLSISSCLQQ	42.47
5378	P78358	NY-ESO-1	DRB1*13:97	141	155	DHRQLQLSISSCLQQ	42.47
5379	P78358	NY-ESO-1	DRB1*11:02	132	146	ILTIRLTAADHRQLQ	42.73
5380	P78358	NY-ESO-1	DRB1*11:65	132	146	ILTIRLTAADHRQLQ	42.73
5381	P78358	NY-ESO-1	DRB1*13:01	132	146	ILTIRLTAADHRQLQ	42.73
5382	P78358	NY-ESO-1	DPB1*01:01	83	97	PESRLLEFYLAMPFA	43.22
5383	P78358	NY-ESO-1	DPB1*01:01	155	169	QLSLLMWITQCFLPV	43.49
5384	P78358	NY-ESO-1	DRB1*15:01	83	97	PESRLLEFYLAMPFA	43.74
5385	P78358	NY-ESO-1	DRB1*15:06	83	97	PESRLLEFYLAMPFA	43.74
5386	P78358	NY-ESO-1	DRB1*04:08	121	135	VLLKEFTVSGNILTI	43.81
5387	P78358	NY-ESO-1	DRB1*16:09	87	101	LLEFYLAMPFATPME	43.88
5388	P78358	NY-ESO-1	DPB1*15:01	84	98	ESRLLEFYLAMPFAT	43.89
5389	P78358	NY-ESO-1	DRB1*11:13	130	144	GNILTIRLTAADHRQ	44.05
5390	P78358	NY-ESO-1	DRB1*16:05	123	137	LKEFTVSGNILTIRL	44.06
5391	P78358	NY-ESO-1	DPB1*15:01	156	170	LSLLMWITQCFLPVF	44.12
5392	P78358	NY-ESO-1	DRB1*14:32	142	156	HRQLQLSISSCLQQL	44.18
5393	P78358	NY-ESO-1	DRB1*11:13	133	147	LTIRLTAADHRQLQL	44.52
5394	P78358	NY-ESO-1	DPB1*02:02	158	172	LLMWITQCFLPVFLA	44.65
5395	P78358	NY-ESO-1	DPB1*47:01	158	172	LLMWITQCFLPVFLA	44.65
5396	P78358	NY-ESO-1	DRB1*03:11	134	148	TIRLTAADHRQLQLS	44.73
5397	P78358	NY-ESO-1	DRB1*16:01	123	137	LKEFTVSGNILTIRL	44.77
5398	P78358	NY-ESO-1	DRB1*11:42	133	147	LTIRLTAADHRQLQL	44.84
5399	P78358	NY-ESO-1	DPB1*02:02	85	99	SRLLEFYLAMPFATP	45.05
5400	P78358	NY-ESO-1	DPB1*47:01	85	99	SRLLEFYLAMPFATP	45.05
5401	P78358	NY-ESO-1	DPB1*15:01	86	100	RLLEFYLAMPFATPM	45.06
5402	P78358	NY-ESO-1	DRB1*11:02	131	145	NILTIRLTAADHRQL	45.24
5403	P78358	NY-ESO-1	DRB1*11:65	131	145	NILTIRLTAADHRQL	45.24
5404	P78358	NY-ESO-1	DRB1*13:01	131	145	NILTIRLTAADHRQL	45.24
5405	P78358	NY-ESO-1	DRB1*01:20	97	111	ATPMEAELARRSLAQ	45.27
5406	P78358	NY-ESO-1	DPB1*01:01	84	98	ESRLLEFYLAMPFAT	45.32
5407	P78358	NY-ESO-1	DRB1*07:01	85	99	SRLLEFYLAMPFATP	45.61
5408	P78358	NY-ESO-1	DRB1*11:04	129	143	SGNILTIRLTAADHR	45.87
5409	P78358	NY-ESO-1	DRB1*11:46	129	143	SGNILTIRLTAADHR	45.87
5410	P78358	NY-ESO-1	DRB1*11:58	129	143	SGNILTIRLTAADHR	45.87

5411	P78358	NY-ESO-1	DRB1*13:11	129	143	SGNILTIRLTAADHR	45.87
5412	P78358	NY-ESO-1	DRB1*03:11	132	146	ILTIRLTAADHRQLQ	45.97
5413	P78358	NY-ESO-1	DPB1*01:01	159	173	LMWITQCFLPVFLAQ	45.99
5414	P78358	NY-ESO-1	DPB1*01:01	82	96	GPESRLLEFYLAMPF	46.07
5415	P78358	NY-ESO-1	DRB1*10:01	164	178	QCFLPVFLAQPPSGQ	46.29
5416	P78358	NY-ESO-1	DRB1*10:01	121	135	VLLKEFTVSGNILTI	46.32
5417	P78358	NY-ESO-1	DPB1*04:01	85	99	SRLLEFYLAMPFATP	46.39
5418	P78358	NY-ESO-1	DPB1*126:01	85	99	SRLLEFYLAMPFATP	46.39
5419	P78358	NY-ESO-1	DPB1*23:01	85	99	SRLLEFYLAMPFATP	46.39
5420	P78358	NY-ESO-1	DPB1*39:01	85	99	SRLLEFYLAMPFATP	46.39
5421	P78358	NY-ESO-1	DRB1*01:20	133	147	LTIRLTAADHRQLQL	46.46
5422	P78358	NY-ESO-1	DPB1*15:01	160	174	MWITQCFLPVFLAQP	46.54
5423	P78358	NY-ESO-1	DRB1*01:24	143	157	RQLQLSISSCLQQLS	46.80
5424	P78358	NY-ESO-1	DRB1*16:09	86	100	RLLEFYLAMPFATPM	46.86
5425	P78358	NY-ESO-1	DPB1*02:01	85	99	SRLLEFYLAMPFATP	47.03
5426	P78358	NY-ESO-1	DPB1*46:01	85	99	SRLLEFYLAMPFATP	47.03
5427	P78358	NY-ESO-1	DPB1*81:01	85	99	SRLLEFYLAMPFATP	47.03
5428	P78358	NY-ESO-1	DPB1*72:01	86	100	RLLEFYLAMPFATPM	47.30
5429	P78358	NY-ESO-1	DRB1*11:13	129	143	SGNILTIRLTAADHR	47.37
5430	P78358	NY-ESO-1	DRB1*01:02	141	155	DHRQLQLSISSCLQQ	47.40
5431	P78358	NY-ESO-1	DRB1*01:20	128	142	VSGNILTIRLTAADH	47.44
5432	P78358	NY-ESO-1	DPB1*02:01	86	100	RLLEFYLAMPFATPM	47.67
5433	P78358	NY-ESO-1	DPB1*46:01	86	100	RLLEFYLAMPFATPM	47.67
5434	P78358	NY-ESO-1	DPB1*81:01	86	100	RLLEFYLAMPFATPM	47.67
5435	P78358	NY-ESO-1	DRB1*01:20	165	179	CFLPVFLAQPPSGQR	47.69
5436	P78358	NY-ESO-1	DPB1*01:01	156	170	LSLLMWITQCFLPVF	47.73
5437	P78358	NY-ESO-1	DRB1*01:11	121	135	VLLKEFTVSGNILTI	48.00
5438	P78358	NY-ESO-1	DRB1*16:01	87	101	LLEFYLAMPFATPME	48.01
5439	P78358	NY-ESO-1	DPB1*15:01	155	169	QLSLLMWITQCFLPV	48.19
5440	P78358	NY-ESO-1	DPB1*02:02	83	97	PESRLLEFYLAMPFA	48.33
5441	P78358	NY-ESO-1	DPB1*47:01	83	97	PESRLLEFYLAMPFA	48.33
5442	P78358	NY-ESO-1	DRB1*15:03	85	99	SRLLEFYLAMPFATP	48.43
5443	P78358	NY-ESO-1	DRB1*04:01	121	135	VLLKEFTVSGNILTI	48.48
5444	P78358	NY-ESO-1	DPB1*02:01	158	172	LLMWITQCFLPVFLA	48.52
5445	P78358	NY-ESO-1	DPB1*46:01	158	172	LLMWITQCFLPVFLA	48.52
5446	P78358	NY-ESO-1	DPB1*81:01	158	172	LLMWITQCFLPVFLA	48.52
5447	P78358	NY-ESO-1	DRB1*14:32	141	155	DHRQLQLSISSCLQQ	48.59
5448	P78358	NY-ESO-1	DRB1*16:01	122	136	LLKEFTVSGNILTIR	48.67
5449	P78358	NY-ESO-1	DRB1*11:42	128	142	VSGNILTIRLTAADH	48.71
5450	P78358	NY-ESO-1	DRB1*14:06	131	145	NILTIRLTAADHRQL	48.71
5451	P78358	NY-ESO-1	DRB1*16:02	124	138	KEFTVSGNILTIRLT	48.72
5452	P78358	NY-ESO-1	DRB1*01:11	141	155	DHRQLQLSISSCLQQ	48.76
5453	P78358	NY-ESO-1	DRB1*07:01	87	101	LLEFYLAMPFATPME	49.15

5454	P78358	NY-ESO-1	DRB1*11:14	121	135	VLLKEFTVSGNILTI	49.15
5455	P78358	NY-ESO-1	DRB1*13:02	121	135	VLLKEFTVSGNILTI	49.15
5456	P78358	NY-ESO-1	DRB1*13:23	121	135	VLLKEFTVSGNILTI	49.15
5457	P78358	NY-ESO-1	DRB1*13:97	121	135	VLLKEFTVSGNILTI	49.15
5458	P78358	NY-ESO-1	DPB1*15:01	158	172	LLMWITQCFLPVFLA	49.16
5459	P78358	NY-ESO-1	DRB1*15:37	85	99	SRLLEFYLAMPFATP	49.34
5460	P78358	NY-ESO-1	DRB1*13:96	122	136	LLKEFTVSGNILTIR	49.38
5461	P78358	NY-ESO-1	DPB1*72:01	158	172	LLMWITQCFLPVFLA	49.67
5462	P78358	NY-ESO-1	DRB1*01:29	143	157	RQLQLSISSCLQQLS	49.86
5463	Q13072	BAGE	DRB1*01:18	4	18	RAVFLALSAQLLQAR	2.47
5464	Q13072	BAGE	DRB1*01:01	4	18	RAVFLALSAQLLQAR	2.61
5465	Q13072	BAGE	DRB1*01:18	3	17	ARAVFLALSAQLLQA	2.73
5466	Q13072	BAGE	DRB1*01:18	5	19	AVFLALSAQLLQARL	2.74
5467	Q13072	BAGE	DRB1*01:18	2	16	AARAVFLALSAQLLQ	2.88
5468	Q13072	BAGE	DRB1*01:01	3	17	ARAVFLALSAQLLQA	2.96
5469	Q13072	BAGE	DRB1*01:01	5	19	AVFLALSAQLLQARL	3.00
5470	Q13072	BAGE	DRB1*01:18	6	20	VFLALSAQLLQARLM	3.05
5471	Q13072	BAGE	DRB1*01:01	2	16	AARAVFLALSAQLLQ	3.09
5472	Q13072	BAGE	DRB1*01:24	4	18	RAVFLALSAQLLQAR	3.13
5473	Q13072	BAGE	DRB1*01:29	4	18	RAVFLALSAQLLQAR	3.20
5474	Q13072	BAGE	DRB1*01:01	6	20	VFLALSAQLLQARLM	3.41
5475	Q13072	BAGE	DRB1*01:24	3	17	ARAVFLALSAQLLQA	3.42
5476	Q13072	BAGE	DRB1*01:24	5	19	AVFLALSAQLLQARL	3.48
5477	Q13072	BAGE	DRB1*01:24	2	16	AARAVFLALSAQLLQ	3.64
5478	Q13072	BAGE	DRB1*01:29	3	17	ARAVFLALSAQLLQA	3.72
5479	Q13072	BAGE	DRB1*01:18	1	15	MAARAVFLALSAQLL	3.77
5480	Q13072	BAGE	DRB1*01:11	4	18	RAVFLALSAQLLQAR	3.85
5481	Q13072	BAGE	DRB1*01:29	5	19	AVFLALSAQLLQARL	3.89
5482	Q13072	BAGE	DRB1*01:24	6	20	VFLALSAQLLQARLM	3.92
5483	Q13072	BAGE	DRB1*01:01	1	15	MAARAVFLALSAQLL	3.96
5484	Q13072	BAGE	DRB1*01:29	2	16	AARAVFLALSAQLLQ	4.20
5485	Q13072	BAGE	DRB1*01:11	3	17	ARAVFLALSAQLLQA	4.46
5486	Q13072	BAGE	DRB1*01:11	5	19	AVFLALSAQLLQARL	4.64
5487	Q13072	BAGE	DRB1*01:24	1	15	MAARAVFLALSAQLL	4.77
5488	Q13072	BAGE	DRB1*01:29	6	20	VFLALSAQLLQARLM	4.82
5489	Q13072	BAGE	DRB1*01:11	2	16	AARAVFLALSAQLLQ	4.86
5490	Q13072	BAGE	DRB1*01:20	4	18	RAVFLALSAQLLQAR	4.91
5491	Q13072	BAGE	DRB1*01:18	7	21	FLALSAQLLQARLMK	5.62
5492	Q13072	BAGE	DRB1*01:11	6	20	VFLALSAQLLQARLM	5.78
5493	Q13072	BAGE	DRB1*01:29	1	15	MAARAVFLALSAQLL	6.19
5494	Q13072	BAGE	DRB1*10:01	4	18	RAVFLALSAQLLQAR	6.48
5495	Q13072	BAGE	DRB1*01:20	3	17	ARAVFLALSAQLLQA	6.56
5496	Q13072	BAGE	DRB1*01:20	5	19	AVFLALSAQLLQARL	6.68

5497	Q13072	BAGE	DRB1*01:11	1	15	MAARAVFLALSAQLL	6.80
5498	Q13072	BAGE	DRB1*01:01	7	21	FLALSAQLLQARLMK	6.96
5499	Q13072	BAGE	DRB1*01:20	2	16	AARAVFLALSAQLLQ	7.27
5500	Q13072	BAGE	DRB1*01:20	6	20	VFLALSAQLLQARLM	7.51
5501	Q13072	BAGE	DRB1*10:01	3	17	ARAVFLALSAQLLQA	8.02
5502	Q13072	BAGE	DRB1*16:02	4	18	RAVFLALSAQLLQAR	8.32
5503	Q13072	BAGE	DRB1*10:01	2	16	AARAVFLALSAQLLQ	8.68
5504	Q13072	BAGE	DRB1*10:01	5	19	AVFLALSAQLLQARL	8.74
5505	Q13072	BAGE	DRB1*01:24	7	21	FLALSAQLLQARLMK	9.15
5506	Q13072	BAGE	DRB1*16:09	4	18	RAVFLALSAQLLQAR	10.21
5507	Q13072	BAGE	DRB1*16:02	3	17	ARAVFLALSAQLLQA	10.51
5508	Q13072	BAGE	DRB1*01:20	1	15	MAARAVFLALSAQLL	10.66
5509	Q13072	BAGE	DRB1*01:20	7	21	FLALSAQLLQARLMK	10.67
5510	Q13072	BAGE	DRB1*01:29	7	21	FLALSAQLLQARLMK	10.95
5511	Q13072	BAGE	DRB1*10:01	6	20	VFLALSAQLLQARLM	11.26
5512	Q13072	BAGE	DRB1*10:01	1	15	MAARAVFLALSAQLL	11.60
5513	Q13072	BAGE	DRB1*16:05	4	18	RAVFLALSAQLLQAR	11.76
5514	Q13072	BAGE	DRB1*16:02	2	16	AARAVFLALSAQLLQ	12.16
5515	Q13072	BAGE	DRB1*16:01	4	18	RAVFLALSAQLLQAR	12.43
5516	Q13072	BAGE	DRB1*16:02	5	19	AVFLALSAQLLQARL	12.53
5517	Q13072	BAGE	DRB1*01:20	8	22	LALSAQLLQARLMKE	13.14
5518	Q13072	BAGE	DRB1*01:02	4	18	RAVFLALSAQLLQAR	13.44
5519	Q13072	BAGE	DRB1*16:05	3	17	ARAVFLALSAQLLQA	13.75
5520	Q13072	BAGE	DRB1*16:09	3	17	ARAVFLALSAQLLQA	13.76
5521	Q13072	BAGE	DRB1*01:20	9	23	ALSAQLLQARLMKEE	13.77
5522	Q13072	BAGE	DRB1*01:11	7	21	FLALSAQLLQARLMK	13.82
5523	Q13072	BAGE	DRB1*16:09	5	19	AVFLALSAQLLQARL	15.00
5524	Q13072	BAGE	DRB1*15:15	4	18	RAVFLALSAQLLQAR	15.20
5525	Q13072	BAGE	DRB1*01:18	8	22	LALSAQLLQARLMKE	15.91
5526	Q13072	BAGE	DRB1*16:09	2	16	AARAVFLALSAQLLQ	16.06
5527	Q13072	BAGE	DRB1*16:05	2	16	AARAVFLALSAQLLQ	16.10
5528	Q13072	BAGE	DRB1*15:02	4	18	RAVFLALSAQLLQAR	16.16
5529	Q13072	BAGE	DRB1*16:05	5	19	AVFLALSAQLLQARL	16.30
5530	Q13072	BAGE	DRB1*07:01	4	18	RAVFLALSAQLLQAR	16.48
5531	Q13072	BAGE	DRB1*01:03	4	18	RAVFLALSAQLLQAR	16.69
5532	Q13072	BAGE	DRB1*01:18	9	23	ALSAQLLQARLMKEE	16.88
5533	Q13072	BAGE	DRB1*16:04	4	18	RAVFLALSAQLLQAR	16.93
5534	Q13072	BAGE	DRB1*01:20	10	24	LSAQLLQARLMKEES	17.14
5535	Q13072	BAGE	DRB1*01:01	8	22	LALSAQLLQARLMKE	17.77
5536	Q13072	BAGE	DRB1*16:01	3	17	ARAVFLALSAQLLQA	17.84
5537	Q13072	BAGE	DRB1*01:01	9	23	ALSAQLLQARLMKEE	18.80
5538	Q13072	BAGE	DRB1*16:02	1	15	MAARAVFLALSAQLL	19.17
5539	Q13072	BAGE	DRB1*16:02	6	20	VFLALSAQLLQARLM	19.28

5540	Q13072	BAGE	DRB1*15:02	3	17	ARAVFLALSAQLLQA	19.33
5541	Q13072	BAGE	DRB1*07:01	3	17	ARAVFLALSAQLLQA	19.51
5542	Q13072	BAGE	DRB1*16:01	5	19	AVFLALSAQLLQARL	19.65
5543	Q13072	BAGE	DRB1*01:02	3	17	ARAVFLALSAQLLQA	19.72
5544	Q13072	BAGE	DRB1*07:01	2	16	AARAVFLALSAQLLQ	19.97
5545	Q13072	BAGE	DRB1*01:18	10	24	LSAQLLQARLMKEES	19.99
5546	Q13072	BAGE	DRB1*01:02	5	19	AVFLALSAQLLQARL	20.12
5547	Q13072	BAGE	DRB1*16:09	6	20	VFLALSAQLLQARLM	20.13
5548	Q13072	BAGE	DRB1*16:01	2	16	AARAVFLALSAQLLQ	20.56
5549	Q13072	BAGE	DRB1*01:02	6	20	VFLALSAQLLQARLM	20.81
5550	Q13072	BAGE	DRB1*07:01	1	15	MAARAVFLALSAQLL	21.55
5551	Q13072	BAGE	DRB1*01:01	10	24	LSAQLLQARLMKEES	22.28
5552	Q13072	BAGE	DRB1*01:02	2	16	AARAVFLALSAQLLQ	22.52
5553	Q13072	BAGE	DRB1*15:15	3	17	ARAVFLALSAQLLQA	22.79
5554	Q13072	BAGE	DRB1*15:02	2	16	AARAVFLALSAQLLQ	23.52
5555	Q13072	BAGE	DRB1*16:05	6	20	VFLALSAQLLQARLM	24.39
5556	Q13072	BAGE	DRB1*15:02	5	19	AVFLALSAQLLQARL	24.45
5557	Q13072	BAGE	DRB1*16:04	3	17	ARAVFLALSAQLLQA	24.59
5558	Q13072	BAGE	DRB1*16:05	1	15	MAARAVFLALSAQLL	24.73
5559	Q13072	BAGE	DRB1*10:01	7	21	FLALSAQLLQARLMK	24.93
5560	Q13072	BAGE	DRB1*15:15	5	19	AVFLALSAQLLQARL	25.32
5561	Q13072	BAGE	DPB1*33:01	4	18	RAVFLALSAQLLQAR	25.64
5562	Q13072	BAGE	DPB1*71:01	4	18	RAVFLALSAQLLQAR	25.64
5563	Q13072	BAGE	DRB1*16:09	1	15	MAARAVFLALSAQLL	26.79
5564	Q13072	BAGE	DRB1*15:15	2	16	AARAVFLALSAQLLQ	27.08
5565	Q13072	BAGE	DRB1*16:01	6	20	VFLALSAQLLQARLM	27.58
5566	Q13072	BAGE	DRB1*01:02	7	21	FLALSAQLLQARLMK	27.62
5567	Q13072	BAGE	DRB1*16:04	5	19	AVFLALSAQLLQARL	27.82
5568	Q13072	BAGE	DRB1*12:16	4	18	RAVFLALSAQLLQAR	28.77
5569	Q13072	BAGE	DRB1*01:03	3	17	ARAVFLALSAQLLQA	28.81
5570	Q13072	BAGE	DRB1*01:20	11	25	SAQLLQARLMKEESP	29.09
5571	Q13072	BAGE	DRB1*15:01	4	18	RAVFLALSAQLLQAR	29.22
5572	Q13072	BAGE	DRB1*15:06	4	18	RAVFLALSAQLLQAR	29.22
5573	Q13072	BAGE	DRB1*16:04	2	16	AARAVFLALSAQLLQ	29.82
5574	Q13072	BAGE	DRB1*01:03	5	19	AVFLALSAQLLQARL	30.44
5575	Q13072	BAGE	DRB1*09:01	4	18	RAVFLALSAQLLQAR	31.22
5576	Q13072	BAGE	DRB1*11:08	4	18	RAVFLALSAQLLQAR	31.24
5577	Q13072	BAGE	DRB1*07:01	5	19	AVFLALSAQLLQARL	31.60
5578	Q13072	BAGE	DRB1*01:02	1	15	MAARAVFLALSAQLL	31.67
5579	Q13072	BAGE	DRB1*16:01	1	15	MAARAVFLALSAQLL	33.90
5580	Q13072	BAGE	DPB1*33:01	3	17	ARAVFLALSAQLLQA	34.06
5581	Q13072	BAGE	DPB1*71:01	3	17	ARAVFLALSAQLLQA	34.06
5582	Q13072	BAGE	DRB1*01:18	11	25	SAQLLQARLMKEESP	34.26

5583	Q13072	BAGE	DPB1*33:01	1	15	MAARAVFLALSAQLL	34.77
5584	Q13072	BAGE	DPB1*71:01	1	15	MAARAVFLALSAQLL	34.77
5585	Q13072	BAGE	DRB1*15:15	6	20	VFLALSAQLLQARLM	35.27
5586	Q13072	BAGE	DRB1*15:01	3	17	ARAVFLALSAQLLQA	35.58
5587	Q13072	BAGE	DRB1*15:06	3	17	ARAVFLALSAQLLQA	35.58
5588	Q13072	BAGE	DRB1*01:03	2	16	AARAVFLALSAQLLQ	36.37
5589	Q13072	BAGE	DPB1*33:01	2	16	AARAVFLALSAQLLQ	36.86
5590	Q13072	BAGE	DPB1*71:01	2	16	AARAVFLALSAQLLQ	36.86
5591	Q13072	BAGE	DRB1*01:29	8	22	LALSAQLLQARLMKE	37.60
5592	Q13072	BAGE	DRB1*15:02	1	15	MAARAVFLALSAQLL	37.98
5593	Q13072	BAGE	DRB1*11:13	4	18	RAVFLALSAQLLQAR	38.10
5594	Q13072	BAGE	DRB1*01:01	11	25	SAQLLQARLMKEESP	38.35
5595	Q13072	BAGE	DRB1*01:03	6	20	VFLALSAQLLQARLM	38.58
5596	Q13072	BAGE	DRB1*15:02	6	20	VFLALSAQLLQARLM	39.35
5597	Q13072	BAGE	DRB1*14:32	4	18	RAVFLALSAQLLQAR	40.48
5598	Q13072	BAGE	DRB1*09:01	3	17	ARAVFLALSAQLLQA	41.19
5599	Q13072	BAGE	DPB1*33:01	5	19	AVFLALSAQLLQARL	41.32
5600	Q13072	BAGE	DPB1*71:01	5	19	AVFLALSAQLLQARL	41.32
5601	Q13072	BAGE	DRB1*15:07	4	18	RAVFLALSAQLLQAR	41.46
5602	Q13072	BAGE	DRB1*01:29	9	23	ALSAQLLQARLMKEE	42.28
5603	Q13072	BAGE	DRB1*11:19	4	18	RAVFLALSAQLLQAR	42.46
5604	Q13072	BAGE	DRB1*07:01	6	20	VFLALSAQLLQARLM	42.78
5605	Q13072	BAGE	DRB1*16:04	6	20	VFLALSAQLLQARLM	42.87
5606	Q13072	BAGE	DRB1*01:20	16	30	QARLMKEESPVVSWR	43.33
5607	Q13072	BAGE	DRB1*09:01	2	16	AARAVFLALSAQLLQ	43.55
5608	Q13072	BAGE	DRB1*01:20	17	31	ARLMKEESPVVSWRL	43.74
5609	Q13072	BAGE	DRB1*15:03	4	18	RAVFLALSAQLLQAR	43.91
5610	Q13072	BAGE	DRB1*08:30	4	18	RAVFLALSAQLLQAR	44.79
5611	Q13072	BAGE	DRB1*11:08	3	17	ARAVFLALSAQLLQA	44.86
5612	Q13072	BAGE	DRB1*15:01	2	16	AARAVFLALSAQLLQ	45.47
5613	Q13072	BAGE	DRB1*15:06	2	16	AARAVFLALSAQLLQ	45.47
5614	Q13072	BAGE	DRB1*12:16	3	17	ARAVFLALSAQLLQA	46.03
5615	Q13072	BAGE	DRB1*01:02	8	22	LALSAQLLQARLMKE	47.21
5616	Q13072	BAGE	DRB1*11:42	4	18	RAVFLALSAQLLQAR	47.37
5617	Q13072	BAGE	DRB1*12:16	2	16	AARAVFLALSAQLLQ	47.78
5618	Q13072	BAGE	DRB1*15:15	1	15	MAARAVFLALSAQLL	48.71
5619	Q13072	BAGE	DRB1*11:14	4	18	RAVFLALSAQLLQAR	48.97
5620	Q13072	BAGE	DRB1*13:02	4	18	RAVFLALSAQLLQAR	48.97
5621	Q13072	BAGE	DRB1*13:23	4	18	RAVFLALSAQLLQAR	48.97
5622	Q13072	BAGE	DRB1*13:97	4	18	RAVFLALSAQLLQAR	48.97
5623	Q13072	BAGE	DRB1*04:08	4	18	RAVFLALSAQLLQAR	49.46
5624	Q13072	BAGE	DRB1*11:01	4	18	RAVFLALSAQLLQAR	49.70
5625	Q13072	BAGE	DRB1*11:10	4	18	RAVFLALSAQLLQAR	49.70

5626	Q13072	BAGE	DRB1*11:12	4	18	RAVFLALSAQLLQAR	49.70
5627	Q13072	BAGE	DRB1*11:28	4	18	RAVFLALSAQLLQAR	49.70
5628	Q13072	BAGE	DRB1*11:29	4	18	RAVFLALSAQLLQAR	49.70
5629	Q13072	BAGE	DRB1*11:49	4	18	RAVFLALSAQLLQAR	49.70
5630	Q13072	BAGE	DRB1*11:62	4	18	RAVFLALSAQLLQAR	49.70
5631	Q13072	BAGE	DRB1*11:74	4	18	RAVFLALSAQLLQAR	49.70
5632	Q13072	BAGE	DRB1*13:05	4	18	RAVFLALSAQLLQAR	49.70
5633	Q13072	BAGE	DRB1*13:14	4	18	RAVFLALSAQLLQAR	49.70
5634	Q13072	BAGE	DRB1*13:50	4	18	RAVFLALSAQLLQAR	49.70
5635	Q13072	BAGE	DRB1*11:13	5	19	AVFLALSAQLLQARL	49.77
5636	Q13072	BAGE	DRB1*14:32	3	17	ARAVFLALSAQLLQA	49.80
5637	Q16385	SSX2	DRB1*13:21	44	58	EKIFYVYMKRKYEAM	8.54
5638	Q16385	SSX2	DRB1*13:21	45	59	KIFYVYMKRKYEAMT	8.90
5639	Q16385	SSX2	DRB1*10:01	60	74	KLGFKATLPPFMCNK	9.17
5640	Q16385	SSX2	DRB1*13:21	46	60	IFYVYMKRKYEAMTK	9.31
5641	Q16385	SSX2	DRB1*13:21	43	57	SEKIFYVYMKRKYEA	9.35
5642	Q16385	SSX2	DRB1*01:01	60	74	KLGFKATLPPFMCNK	9.87
5643	Q16385	SSX2	DRB1*13:21	42	56	ASEKIFYVYMKRKYE	10.40
5644	Q16385	SSX2	DRB1*01:18	60	74	KLGFKATLPPFMCNK	10.68
5645	Q16385	SSX2	DRB1*11:04	45	59	KIFYVYMKRKYEAMT	11.33
5646	Q16385	SSX2	DRB1*11:46	45	59	KIFYVYMKRKYEAMT	11.33
5647	Q16385	SSX2	DRB1*11:58	45	59	KIFYVYMKRKYEAMT	11.33
5648	Q16385	SSX2	DRB1*13:11	45	59	KIFYVYMKRKYEAMT	11.33
5649	Q16385	SSX2	DRB1*11:04	46	60	IFYVYMKRKYEAMTK	11.71
5650	Q16385	SSX2	DRB1*11:46	46	60	IFYVYMKRKYEAMTK	11.71
5651	Q16385	SSX2	DRB1*11:58	46	60	IFYVYMKRKYEAMTK	11.71
5652	Q16385	SSX2	DRB1*13:11	46	60	IFYVYMKRKYEAMTK	11.71
5653	Q16385	SSX2	DRB1*01:01	98	112	QMTFGRLQGISPKIM	11.75
5654	Q16385	SSX2	DRB1*10:01	59	73	TKLGFKATLPPFMCN	12.08
5655	Q16385	SSX2	DRB1*11:04	44	58	EKIFYVYMKRKYEAM	12.34
5656	Q16385	SSX2	DRB1*11:46	44	58	EKIFYVYMKRKYEAM	12.34
5657	Q16385	SSX2	DRB1*11:58	44	58	EKIFYVYMKRKYEAM	12.34
5658	Q16385	SSX2	DRB1*13:11	44	58	EKIFYVYMKRKYEAM	12.34
5659	Q16385	SSX2	DRB1*07:01	60	74	KLGFKATLPPFMCNK	12.37
5660	Q16385	SSX2	DRB1*13:21	47	61	FYVYMKRKYEAMTKL	12.40
5661	Q16385	SSX2	DRB1*01:18	98	112	QMTFGRLQGISPKIM	12.47
5662	Q16385	SSX2	DRB1*11:01	44	58	EKIFYVYMKRKYEAM	12.58
5663	Q16385	SSX2	DRB1*11:10	44	58	EKIFYVYMKRKYEAM	12.58
5664	Q16385	SSX2	DRB1*11:12	44	58	EKIFYVYMKRKYEAM	12.58
5665	Q16385	SSX2	DRB1*11:28	44	58	EKIFYVYMKRKYEAM	12.58
5666	Q16385	SSX2	DRB1*11:29	44	58	EKIFYVYMKRKYEAM	12.58
5667	Q16385	SSX2	DRB1*11:49	44	58	EKIFYVYMKRKYEAM	12.58
5668	Q16385	SSX2	DRB1*11:62	44	58	EKIFYVYMKRKYEAM	12.58

5669	Q16385	SSX2	DRB1*11:74	44	58	EKIFYVYMKRKYEAM	12.58
5670	Q16385	SSX2	DRB1*13:05	44	58	EKIFYVYMKRKYEAM	12.58
5671	Q16385	SSX2	DRB1*13:14	44	58	EKIFYVYMKRKYEAM	12.58
5672	Q16385	SSX2	DRB1*13:50	44	58	EKIFYVYMKRKYEAM	12.58
5673	Q16385	SSX2	DRB1*11:01	45	59	KIFYVYMKRKYEAMT	12.77
5674	Q16385	SSX2	DRB1*11:10	45	59	KIFYVYMKRKYEAMT	12.77
5675	Q16385	SSX2	DRB1*11:12	45	59	KIFYVYMKRKYEAMT	12.77
5676	Q16385	SSX2	DRB1*11:28	45	59	KIFYVYMKRKYEAMT	12.77
5677	Q16385	SSX2	DRB1*11:29	45	59	KIFYVYMKRKYEAMT	12.77
5678	Q16385	SSX2	DRB1*11:49	45	59	KIFYVYMKRKYEAMT	12.77
5679	Q16385	SSX2	DRB1*11:62	45	59	KIFYVYMKRKYEAMT	12.77
5680	Q16385	SSX2	DRB1*11:74	45	59	KIFYVYMKRKYEAMT	12.77
5681	Q16385	SSX2	DRB1*13:05	45	59	KIFYVYMKRKYEAMT	12.77
5682	Q16385	SSX2	DRB1*13:14	45	59	KIFYVYMKRKYEAMT	12.77
5683	Q16385	SSX2	DRB1*13:50	45	59	KIFYVYMKRKYEAMT	12.77
5684	Q16385	SSX2	DRB1*11:03	45	59	KIFYVYMKRKYEAMT	12.89
5685	Q16385	SSX2	DRB1*11:03	46	60	IFYVYMKRKYEAMTK	13.27
5686	Q16385	SSX2	DRB1*11:03	44	58	EKIFYVYMKRKYEAM	13.38
5687	Q16385	SSX2	DRB1*01:01	59	73	TKLGFKATLPPFMCN	13.86
5688	Q16385	SSX2	DRB1*07:01	59	73	TKLGFKATLPPFMCN	13.87
5689	Q16385	SSX2	DRB1*11:01	46	60	IFYVYMKRKYEAMTK	13.87
5690	Q16385	SSX2	DRB1*11:10	46	60	IFYVYMKRKYEAMTK	13.87
5691	Q16385	SSX2	DRB1*11:12	46	60	IFYVYMKRKYEAMTK	13.87
5692	Q16385	SSX2	DRB1*11:28	46	60	IFYVYMKRKYEAMTK	13.87
5693	Q16385	SSX2	DRB1*11:29	46	60	IFYVYMKRKYEAMTK	13.87
5694	Q16385	SSX2	DRB1*11:49	46	60	IFYVYMKRKYEAMTK	13.87
5695	Q16385	SSX2	DRB1*11:62	46	60	IFYVYMKRKYEAMTK	13.87
5696	Q16385	SSX2	DRB1*11:74	46	60	IFYVYMKRKYEAMTK	13.87
5697	Q16385	SSX2	DRB1*13:05	46	60	IFYVYMKRKYEAMTK	13.87
5698	Q16385	SSX2	DRB1*13:14	46	60	IFYVYMKRKYEAMTK	13.87
5699	Q16385	SSX2	DRB1*13:50	46	60	IFYVYMKRKYEAMTK	13.87
5700	Q16385	SSX2	DRB1*07:01	57	71	AMTKLGFKATLPPFM	14.22
5701	Q16385	SSX2	DRB1*01:01	97	111	PQMTFGRLQGISPKI	14.40
5702	Q16385	SSX2	DRB1*01:18	59	73	TKLGFKATLPPFMCN	14.66
5703	Q16385	SSX2	DRB1*10:01	61	75	LGFKATLPPFMCNKR	14.93
5704	Q16385	SSX2	DRB1*07:01	58	72	MTKLGFKATLPPFMC	15.13
5705	Q16385	SSX2	DRB1*11:01	43	57	SEKIFYVYMKRKYEA	15.39
5706	Q16385	SSX2	DRB1*11:10	43	57	SEKIFYVYMKRKYEA	15.39
5707	Q16385	SSX2	DRB1*11:12	43	57	SEKIFYVYMKRKYEA	15.39
5708	Q16385	SSX2	DRB1*11:28	43	57	SEKIFYVYMKRKYEA	15.39
5709	Q16385	SSX2	DRB1*11:29	43	57	SEKIFYVYMKRKYEA	15.39
5710	Q16385	SSX2	DRB1*11:49	43	57	SEKIFYVYMKRKYEA	15.39
5711	Q16385	SSX2	DRB1*11:62	43	57	SEKIFYVYMKRKYEA	15.39

5712	Q16385	SSX2	DRB1*11:74	43	57	SEKIFYVYMKRKYEA	15.39
5713	Q16385	SSX2	DRB1*13:05	43	57	SEKIFYVYMKRKYEA	15.39
5714	Q16385	SSX2	DRB1*13:14	43	57	SEKIFYVYMKRKYEA	15.39
5715	Q16385	SSX2	DRB1*13:50	43	57	SEKIFYVYMKRKYEA	15.39
5716	Q16385	SSX2	DRB1*11:04	47	61	FYVYMKRKYEAMTKL	16.24
5717	Q16385	SSX2	DRB1*11:46	47	61	FYVYMKRKYEAMTKL	16.24
5718	Q16385	SSX2	DRB1*11:58	47	61	FYVYMKRKYEAMTKL	16.24
5719	Q16385	SSX2	DRB1*13:11	47	61	FYVYMKRKYEAMTKL	16.24
5720	Q16385	SSX2	DRB1*11:03	47	61	FYVYMKRKYEAMTKL	16.28
5721	Q16385	SSX2	DRB1*01:18	97	111	PQMTFGRLQGISPKI	16.33
5722	Q16385	SSX2	DRB1*01:18	99	113	MTFGRLQGISPKIMP	16.45
5723	Q16385	SSX2	DRB1*10:01	58	72	MTKLGFKATLPPFMC	16.45
5724	Q16385	SSX2	DRB1*11:04	43	57	SEKIFYVYMKRKYEA	16.45
5725	Q16385	SSX2	DRB1*11:46	43	57	SEKIFYVYMKRKYEA	16.45
5726	Q16385	SSX2	DRB1*11:58	43	57	SEKIFYVYMKRKYEA	16.45
5727	Q16385	SSX2	DRB1*13:11	43	57	SEKIFYVYMKRKYEA	16.45
5728	Q16385	SSX2	DRB1*01:01	99	113	MTFGRLQGISPKIMP	17.02
5729	Q16385	SSX2	DRB1*01:18	61	75	LGFKATLPPFMCNKR	17.23
5730	Q16385	SSX2	DRB1*11:03	43	57	SEKIFYVYMKRKYEA	17.36
5731	Q16385	SSX2	DRB1*01:01	61	75	LGFKATLPPFMCNKR	17.49
5732	Q16385	SSX2	DRB1*11:42	44	58	EKIFYVYMKRKYEAM	18.11
5733	Q16385	SSX2	DRB1*11:42	45	59	KIFYVYMKRKYEAMT	18.28
5734	Q16385	SSX2	DRB1*09:01	60	74	KLGFKATLPPFMCNK	19.03
5735	Q16385	SSX2	DRB1*11:42	46	60	IFYVYMKRKYEAMTK	19.51
5736	Q16385	SSX2	DRB1*11:01	47	61	FYVYMKRKYEAMTKL	19.78
5737	Q16385	SSX2	DRB1*11:10	47	61	FYVYMKRKYEAMTKL	19.78
5738	Q16385	SSX2	DRB1*11:12	47	61	FYVYMKRKYEAMTKL	19.78
5739	Q16385	SSX2	DRB1*11:28	47	61	FYVYMKRKYEAMTKL	19.78
5740	Q16385	SSX2	DRB1*11:29	47	61	FYVYMKRKYEAMTKL	19.78
5741	Q16385	SSX2	DRB1*11:49	47	61	FYVYMKRKYEAMTKL	19.78
5742	Q16385	SSX2	DRB1*11:62	47	61	FYVYMKRKYEAMTKL	19.78
5743	Q16385	SSX2	DRB1*11:74	47	61	FYVYMKRKYEAMTKL	19.78
5744	Q16385	SSX2	DRB1*13:05	47	61	FYVYMKRKYEAMTKL	19.78
5745	Q16385	SSX2	DRB1*13:14	47	61	FYVYMKRKYEAMTKL	19.78
5746	Q16385	SSX2	DRB1*13:50	47	61	FYVYMKRKYEAMTKL	19.78
5747	Q16385	SSX2	DRB1*01:01	58	72	MTKLGFKATLPPFMC	20.03
5748	Q16385	SSX2	DRB1*01:18	58	72	MTKLGFKATLPPFMC	20.35
5749	Q16385	SSX2	DRB1*11:42	43	57	SEKIFYVYMKRKYEA	20.63
5750	Q16385	SSX2	DRB1*01:29	60	74	KLGFKATLPPFMCNK	20.78
5751	Q16385	SSX2	DRB1*07:01	61	75	LGFKATLPPFMCNKR	21.75
5752	Q16385	SSX2	DRB1*01:24	60	74	KLGFKATLPPFMCNK	21.80
5753	Q16385	SSX2	DRB1*09:01	59	73	TKLGFKATLPPFMCN	21.81
5754	Q16385	SSX2	DRB1*11:01	42	56	ASEKIFYVYMKRKYE	21.98

5755	Q16385	SSX2	DRB1*11:10	42	56	ASEKIFYVYMKRKYE	21.98
5756	Q16385	SSX2	DRB1*11:12	42	56	ASEKIFYVYMKRKYE	21.98
5757	Q16385	SSX2	DRB1*11:28	42	56	ASEKIFYVYMKRKYE	21.98
5758	Q16385	SSX2	DRB1*11:29	42	56	ASEKIFYVYMKRKYE	21.98
5759	Q16385	SSX2	DRB1*11:49	42	56	ASEKIFYVYMKRKYE	21.98
5760	Q16385	SSX2	DRB1*11:62	42	56	ASEKIFYVYMKRKYE	21.98
5761	Q16385	SSX2	DRB1*11:74	42	56	ASEKIFYVYMKRKYE	21.98
5762	Q16385	SSX2	DRB1*13:05	42	56	ASEKIFYVYMKRKYE	21.98
5763	Q16385	SSX2	DRB1*13:14	42	56	ASEKIFYVYMKRKYE	21.98
5764	Q16385	SSX2	DRB1*13:50	42	56	ASEKIFYVYMKRKYE	21.98
5765	Q16385	SSX2	DRB1*01:18	100	114	TFGRLQGISPKIMPK	21.99
5766	Q16385	SSX2	DRB1*01:20	101	115	FGRLQGISPKIMPKK	22.29
5767	Q16385	SSX2	DRB1*01:01	100	114	TFGRLQGISPKIMPK	23.64
5768	Q16385	SSX2	DRB1*01:01	96	110	RPQMTFGRLQGISPK	23.77
5769	Q16385	SSX2	DRB1*09:01	57	71	AMTKLGFKATLPPFM	24.36
5770	Q16385	SSX2	DRB1*10:01	57	71	AMTKLGFKATLPPFM	24.55
5771	Q16385	SSX2	DRB1*09:01	58	72	MTKLGFKATLPPFMC	24.82
5772	Q16385	SSX2	DRB1*01:11	60	74	KLGFKATLPPFMCNK	25.07
5773	Q16385	SSX2	DRB1*01:20	100	114	TFGRLQGISPKIMPK	25.76
5774	Q16385	SSX2	DRB1*11:02	45	59	KIFYVYMKRKYEAMT	26.59
5775	Q16385	SSX2	DRB1*11:65	45	59	KIFYVYMKRKYEAMT	26.59
5776	Q16385	SSX2	DRB1*13:01	45	59	KIFYVYMKRKYEAMT	26.59
5777	Q16385	SSX2	DRB1*10:01	98	112	QMTFGRLQGISPKIM	26.83
5778	Q16385	SSX2	DRB1*11:11	45	59	KIFYVYMKRKYEAMT	27.01
5779	Q16385	SSX2	DRB1*01:18	96	110	RPQMTFGRLQGISPK	27.06
5780	Q16385	SSX2	DRB1*11:11	44	58	EKIFYVYMKRKYEAM	27.25
5781	Q16385	SSX2	DRB1*11:37	45	59	KIFYVYMKRKYEAMT	27.34
5782	Q16385	SSX2	DRB1*13:07	45	59	KIFYVYMKRKYEAMT	27.34
5783	Q16385	SSX2	DRB1*01:20	98	112	QMTFGRLQGISPKIM	27.43
5784	Q16385	SSX2	DRB1*11:02	44	58	EKIFYVYMKRKYEAM	27.64
5785	Q16385	SSX2	DRB1*11:65	44	58	EKIFYVYMKRKYEAM	27.64
5786	Q16385	SSX2	DRB1*13:01	44	58	EKIFYVYMKRKYEAM	27.64
5787	Q16385	SSX2	DRB1*11:03	48	62	YVYMKRKYEAMTKLG	27.78
5788	Q16385	SSX2	DRB1*01:18	101	115	FGRLQGISPKIMPKK	27.98
5789	Q16385	SSX2	DRB1*11:42	47	61	FYVYMKRKYEAMTKL	28.04
5790	Q16385	SSX2	DRB1*11:37	44	58	EKIFYVYMKRKYEAM	28.09
5791	Q16385	SSX2	DRB1*13:07	44	58	EKIFYVYMKRKYEAM	28.09
5792	Q16385	SSX2	DRB1*11:04	42	56	ASEKIFYVYMKRKYE	28.14
5793	Q16385	SSX2	DRB1*11:46	42	56	ASEKIFYVYMKRKYE	28.14
5794	Q16385	SSX2	DRB1*11:58	42	56	ASEKIFYVYMKRKYE	28.14
5795	Q16385	SSX2	DRB1*13:11	42	56	ASEKIFYVYMKRKYE	28.14
5796	Q16385	SSX2	DRB1*01:29	59	73	TKLGFKATLPPFMCN	28.15
5797	Q16385	SSX2	DRB1*11:02	46	60	IFYVYMKRKYEAMTK	28.77

5798	Q16385	SSX2	DRB1*11:65	46	60	IFYVYMKRKYEAMTK	28.77
5799	Q16385	SSX2	DRB1*13:01	46	60	IFYVYMKRKYEAMTK	28.77
5800	Q16385	SSX2	DRB1*11:03	42	56	ASEKIFYVYMKRKYE	28.81
5801	Q16385	SSX2	DRB1*01:18	57	71	AMTKLGFKATLPPFM	29.04
5802	Q16385	SSX2	DRB1*10:01	62	76	GFKATLPPFMCNKRA	29.58
5803	Q16385	SSX2	DRB1*01:20	99	113	MTFGRLQGISPKIMP	29.64
5804	Q16385	SSX2	DRB1*01:01	57	71	AMTKLGFKATLPPFM	29.73
5805	Q16385	SSX2	DRB1*11:42	42	56	ASEKIFYVYMKRKYE	30.02
5806	Q16385	SSX2	DRB1*09:01	61	75	LGFKATLPPFMCNKR	30.82
5807	Q16385	SSX2	DRB1*01:24	98	112	QMTFGRLQGISPKIM	31.05
5808	Q16385	SSX2	DRB1*11:37	46	60	IFYVYMKRKYEAMTK	31.32
5809	Q16385	SSX2	DRB1*13:07	46	60	IFYVYMKRKYEAMTK	31.32
5810	Q16385	SSX2	DRB1*11:11	46	60	IFYVYMKRKYEAMTK	31.55
5811	Q16385	SSX2	DRB1*11:13	37	51	WEKMKASEKIFYVYM	31.70
5812	Q16385	SSX2	DRB1*11:08	44	58	EKIFYVYMKRKYEAM	31.94
5813	Q16385	SSX2	DRB1*01:24	59	73	TKLGFKATLPPFMCN	31.98
5814	Q16385	SSX2	DRB1*11:08	45	59	KIFYVYMKRKYEAMT	32.05
5815	Q16385	SSX2	DRB1*10:01	97	111	PQMTFGRLQGISPKI	33.03
5816	Q16385	SSX2	DRB1*01:01	101	115	FGRLQGISPKIMPKK	33.11
5817	Q16385	SSX2	DRB1*01:20	60	74	KLGFKATLPPFMCNK	33.19
5818	Q16385	SSX2	DRB1*01:18	62	76	GFKATLPPFMCNKRA	33.45
5819	Q16385	SSX2	DRB1*11:13	44	58	EKIFYVYMKRKYEAM	33.65
5820	Q16385	SSX2	DRB1*11:84	45	59	KIFYVYMKRKYEAMT	34.03
5821	Q16385	SSX2	DRB1*14:32	37	51	WEKMKASEKIFYVYM	34.22
5822	Q16385	SSX2	DRB1*11:11	43	57	SEKIFYVYMKRKYEA	34.60
5823	Q16385	SSX2	DRB1*11:42	37	51	WEKMKASEKIFYVYM	34.70
5824	Q16385	SSX2	DRB1*11:84	44	58	EKIFYVYMKRKYEAM	34.86
5825	Q16385	SSX2	DRB1*11:04	48	62	YVYMKRKYEAMTKLG	35.29
5826	Q16385	SSX2	DRB1*11:46	48	62	YVYMKRKYEAMTKLG	35.29
5827	Q16385	SSX2	DRB1*11:58	48	62	YVYMKRKYEAMTKLG	35.29
5828	Q16385	SSX2	DRB1*13:11	48	62	YVYMKRKYEAMTKLG	35.29
5829	Q16385	SSX2	DRB1*01:29	61	75	LGFKATLPPFMCNKR	35.33
5830	Q16385	SSX2	DRB1*10:01	99	113	MTFGRLQGISPKIMP	35.68
5831	Q16385	SSX2	DRB1*11:13	36	50	EWEKMKASEKIFYVY	35.83
5832	Q16385	SSX2	DRB1*11:84	46	60	IFYVYMKRKYEAMTK	35.97
5833	Q16385	SSX2	DRB1*14:32	36	50	EWEKMKASEKIFYVY	36.30
5834	Q16385	SSX2	DRB1*01:01	62	76	GFKATLPPFMCNKRA	36.57
5835	Q16385	SSX2	DRB1*11:13	45	59	KIFYVYMKRKYEAMT	36.57
5836	Q16385	SSX2	DRB1*11:02	43	57	SEKIFYVYMKRKYEA	37.10
5837	Q16385	SSX2	DRB1*11:65	43	57	SEKIFYVYMKRKYEA	37.10
5838	Q16385	SSX2	DRB1*13:01	43	57	SEKIFYVYMKRKYEA	37.10
5839	Q16385	SSX2	DRB1*11:37	43	57	SEKIFYVYMKRKYEA	37.23
5840	Q16385	SSX2	DRB1*13:07	43	57	SEKIFYVYMKRKYEA	37.23

5841	Q16385	SSX2	DRB1*08:01	44	58	EKIFYVYMKRKYEAM	37.24
5842	Q16385	SSX2	DRB1*11:13	43	57	SEKIFYVYMKRKYEA	37.35
5843	Q16385	SSX2	DRB1*11:13	38	52	EKMKASEKIFYVYMK	37.62
5844	Q16385	SSX2	DRB1*01:11	59	73	TKLGFKATLPPFMCN	37.73
5845	Q16385	SSX2	DRB1*08:01	45	59	KIFYVYMKRKYEAMT	37.96
5846	Q16385	SSX2	DRB1*01:29	58	72	MTKLGFKATLPPFMC	38.11
5847	Q16385	SSX2	DRB1*01:29	98	112	QMTFGRLQGISPKIM	38.38
5848	Q16385	SSX2	DRB1*13:21	51	65	MKRKYEAMTKLGFKA	38.53
5849	Q16385	SSX2	DRB1*11:08	43	57	SEKIFYVYMKRKYEA	38.70
5850	Q16385	SSX2	DRB1*11:02	47	61	FYVYMKRKYEAMTKL	39.09
5851	Q16385	SSX2	DRB1*11:65	47	61	FYVYMKRKYEAMTKL	39.09
5852	Q16385	SSX2	DRB1*13:01	47	61	FYVYMKRKYEAMTKL	39.09
5853	Q16385	SSX2	DRB1*11:27	45	59	KIFYVYMKRKYEAMT	39.72
5854	Q16385	SSX2	DRB1*01:24	61	75	LGFKATLPPFMCNKR	39.73
5855	Q16385	SSX2	DRB1*11:42	36	50	EWEKMKASEKIFYVY	39.75
5856	Q16385	SSX2	DRB1*11:27	44	58	EKIFYVYMKRKYEAM	39.90
5857	Q16385	SSX2	DRB1*11:03	41	55	KASEKIFYVYMKRKY	40.17
5858	Q16385	SSX2	DRB1*13:21	52	66	KRKYEAMTKLGFKAT	40.41
5859	Q16385	SSX2	DRB1*11:13	46	60	IFYVYMKRKYEAMTK	40.57
5860	Q16385	SSX2	DRB1*08:01	46	60	IFYVYMKRKYEAMTK	40.75
5861	Q16385	SSX2	DRB1*14:32	38	52	EKMKASEKIFYVYMK	40.87
5862	Q16385	SSX2	DRB1*01:20	97	111	PQMTFGRLQGISPKI	41.41
5863	Q16385	SSX2	DRB1*13:21	50	64	YMKRKYEAMTKLGFK	41.62
5864	Q16385	SSX2	DRB1*16:02	60	74	KLGFKATLPPFMCNK	41.89
5865	Q16385	SSX2	DRB1*03:11	37	51	WEKMKASEKIFYVYM	42.21
5866	Q16385	SSX2	DRB1*08:24	45	59	KIFYVYMKRKYEAMT	42.23
5867	Q16385	SSX2	DRB1*11:42	38	52	EKMKASEKIFYVYMK	42.26
5868	Q16385	SSX2	DRB1*01:20	102	116	GRLQGISPKIMPKKP	42.51
5869	Q16385	SSX2	DRB1*11:08	46	60	IFYVYMKRKYEAMTK	42.52
5870	Q16385	SSX2	DRB1*08:04	46	60	IFYVYMKRKYEAMTK	43.16
5871	Q16385	SSX2	DRB1*13:21	48	62	YVYMKRKYEAMTKLG	43.16
5872	Q16385	SSX2	DRB1*01:24	58	72	MTKLGFKATLPPFMC	43.21
5873	Q16385	SSX2	DRB1*01:24	99	113	MTFGRLQGISPKIMP	43.21
5874	Q16385	SSX2	DRB1*08:24	44	58	EKIFYVYMKRKYEAM	43.31
5875	Q16385	SSX2	DRB1*01:24	97	111	PQMTFGRLQGISPKI	43.72
5876	Q16385	SSX2	DRB1*14:32	35	49	EEWEKMKASEKIFYV	43.74
5877	Q16385	SSX2	DRB1*08:04	45	59	KIFYVYMKRKYEAMT	43.83
5878	Q16385	SSX2	DRB1*08:01	43	57	SEKIFYVYMKRKYEA	45.25
5879	Q16385	SSX2	DPB1*33:01	167	181	RLRERKQLVIYEEIS	45.34
5880	Q16385	SSX2	DPB1*71:01	167	181	RLRERKQLVIYEEIS	45.34
5881	Q16385	SSX2	DRB1*11:11	47	61	FYVYMKRKYEAMTKL	45.35
5882	Q16385	SSX2	DRB1*01:20	59	73	TKLGFKATLPPFMCN	45.37
5883	Q16385	SSX2	DRB1*08:04	44	58	EKIFYVYMKRKYEAM	45.57

5884	Q16385	SSX2	DRB1*11:02	37	51	WEKMKASEKIFYVYM	45.94
5885	Q16385	SSX2	DRB1*11:65	37	51	WEKMKASEKIFYVYM	45.94
5886	Q16385	SSX2	DRB1*13:01	37	51	WEKMKASEKIFYVYM	45.94
5887	Q16385	SSX2	DRB1*07:01	62	76	GFKATLPPFMCNKRA	46.32
5888	Q16385	SSX2	DRB1*03:11	36	50	EWEKMKASEKIFYVY	46.52
5889	Q16385	SSX2	DRB1*11:84	43	57	SEKIFYVYMKRKYEA	46.83
5890	Q16385	SSX2	DRB1*13:21	53	67	RKYEAMTKLGFKATL	47.08
5891	Q16385	SSX2	DRB1*11:01	52	66	KRKYEAMTKLGFKAT	47.42
5892	Q16385	SSX2	DRB1*11:10	52	66	KRKYEAMTKLGFKAT	47.42
5893	Q16385	SSX2	DRB1*11:12	52	66	KRKYEAMTKLGFKAT	47.42
5894	Q16385	SSX2	DRB1*11:28	52	66	KRKYEAMTKLGFKAT	47.42
5895	Q16385	SSX2	DRB1*11:29	52	66	KRKYEAMTKLGFKAT	47.42
5896	Q16385	SSX2	DRB1*11:49	52	66	KRKYEAMTKLGFKAT	47.42
5897	Q16385	SSX2	DRB1*11:62	52	66	KRKYEAMTKLGFKAT	47.42
5898	Q16385	SSX2	DRB1*11:74	52	66	KRKYEAMTKLGFKAT	47.42
5899	Q16385	SSX2	DRB1*13:05	52	66	KRKYEAMTKLGFKAT	47.42
5900	Q16385	SSX2	DRB1*13:14	52	66	KRKYEAMTKLGFKAT	47.42
5901	Q16385	SSX2	DRB1*13:50	52	66	KRKYEAMTKLGFKAT	47.42
5902	Q16385	SSX2	DRB1*11:01	51	65	MKRKYEAMTKLGFKA	47.51
5903	Q16385	SSX2	DRB1*11:10	51	65	MKRKYEAMTKLGFKA	47.51
5904	Q16385	SSX2	DRB1*11:12	51	65	MKRKYEAMTKLGFKA	47.51
5905	Q16385	SSX2	DRB1*11:28	51	65	MKRKYEAMTKLGFKA	47.51
5906	Q16385	SSX2	DRB1*11:29	51	65	MKRKYEAMTKLGFKA	47.51
5907	Q16385	SSX2	DRB1*11:49	51	65	MKRKYEAMTKLGFKA	47.51
5908	Q16385	SSX2	DRB1*11:62	51	65	MKRKYEAMTKLGFKA	47.51
5909	Q16385	SSX2	DRB1*11:74	51	65	MKRKYEAMTKLGFKA	47.51
5910	Q16385	SSX2	DRB1*13:05	51	65	MKRKYEAMTKLGFKA	47.51
5911	Q16385	SSX2	DRB1*13:14	51	65	MKRKYEAMTKLGFKA	47.51
5912	Q16385	SSX2	DRB1*13:50	51	65	MKRKYEAMTKLGFKA	47.51
5913	Q16385	SSX2	DRB1*10:01	100	114	TFGRLQGISPKIMPK	47.63
5914	Q16385	SSX2	DRB1*14:01	37	51	WEKMKASEKIFYVYM	47.73
5915	Q16385	SSX2	DRB1*14:54	37	51	WEKMKASEKIFYVYM	47.73
5916	Q16385	SSX2	DRB1*03:11	38	52	EKMKASEKIFYVYMK	48.68
5917	Q16385	SSX2	DRB1*11:42	41	55	KASEKIFYVYMKRKY	48.84
5918	Q16385	SSX2	DRB1*01:11	61	75	LGFKATLPPFMCNKR	49.21
5919	Q16385	SSX2	DRB1*11:13	35	49	EEWEKMKASEKIFYV	49.35
5920	Q16385	SSX2	DRB1*10:01	96	110	RPQMTFGRLQGISPK	49.80
5921	Q16385	SSX2	DRB1*01:29	99	113	MTFGRLQGISPKIMP	49.91
5922	Q16385	SSX2	DRB1*11:54	44	58	EKIFYVYMKRKYEAM	49.95
5923	Q16655	MELAN_A	DRB1*01:18	50	64	RRNGYRALMDKSLHV	16.46
5924	Q16655	MELAN_A	DRB1*01:18	51	65	RNGYRALMDKSLHVG	17.23
5925	Q16655	MELAN_A	DRB1*01:01	50	64	RRNGYRALMDKSLHV	18.26
5926	Q16655	MELAN_A	DRB1*01:01	51	65	RNGYRALMDKSLHVG	19.01

5927	Q16655	MELAN_A	DRB1*01:18	52	66	NGYRALMDKSLHVGT	23.03
5928	Q16655	MELAN_A	DRB1*01:18	49	63	RRRNGYRALMDKSLH	26.09
5929	Q16655	MELAN_A	DRB1*01:01	52	66	NGYRALMDKSLHVGT	26.36
5930	Q16655	MELAN_A	DRB1*01:01	49	63	RRRNGYRALMDKSLH	29.22
5931	Q16655	MELAN_A	DRB1*01:18	53	67	GYRALMDKSLHVGTQ	31.82
5932	Q16655	MELAN_A	DRB1*01:01	53	67	GYRALMDKSLHVGTQ	38.39
5933	Q16655	MELAN_A	DRB1*01:29	51	65	RNGYRALMDKSLHVG	41.89
5934	Q16655	MELAN_A	DRB1*01:29	50	64	RRNGYRALMDKSLHV	43.45
5935	Q16655	MELAN_A	DRB1*01:24	51	65	RNGYRALMDKSLHVG	44.52
5936	Q16655	MELAN_A	DRB1*03:11	53	67	GYRALMDKSLHVGTQ	45.36
5937	Q16655	MELAN_A	DRB1*10:01	99	113	NAPPAYEKLSAEQSP	46.48
5938	Q16655	MELAN_A	DRB1*01:24	50	64	RRNGYRALMDKSLHV	46.66
5939	Q16655	MELAN_A	DRB1*10:01	100	114	APPAYEKLSAEQSPP	48.28
5940	Q16655	MELAN_A	DRB1*01:01	100	114	APPAYEKLSAEQSPP	48.74
5941	Q16655	MELAN_A	DRB1*03:11	52	66	NGYRALMDKSLHVGT	49.33

Table S4. High binding affinity peptide-HLA-II pairs arranged by HLA-II allele.

Index		HLA protein	Peptide	Antigen	Protein ID	Start	End	IC_{50}
1	1	DPB1*01:01	VYDFFVWLHYYSVRD	TRP2	O75767	181	195	49.56
2	2	DPB1*01:01	ISIYNYFVWTHYYSV	TRP1	P17643	182	196	23.26
3	3	DPB1*01:01	IYNYFVWTHYYSVKK	TRP1	P17643	184	198	15.24
4	4	DPB1*01:01	NYFVWTHYYSVKKTF	TRP1	P17643	186	200	23.72
5	5	DPB1*01:01	SIYNYFVWTHYYSVK	TRP1	P17643	183	197	17.3
6	6	DPB1*01:01	YFVWTHYYSVKKTFL	TRP1	P17643	187	201	32.8
7	7	DPB1*01:01	YNYFVWTHYYSVKKT	TRP1	P17643	185	199	16.47
8	8	DPB1*01:01	ESRLLEFYLAMPFAT	NY-ESO-1	P78358	84	98	45.32
9	9	DPB1*01:01	GPESRLLEFYLAMPF	NY-ESO-1	P78358	82	96	46.07
10	10	DPB1*01:01	LMWITQCFLPVFLAQ	NY-ESO-1	P78358	159	173	45.99
11	11	DPB1*01:01	LSLLMWITQCFLPVF	NY-ESO-1	P78358	156	170	47.73
12	12	DPB1*01:01	MWITQCFLPVFLAQP	NY-ESO-1	P78358	160	174	40.19
13	13	DPB1*01:01	PESRLLEFYLAMPFA	NY-ESO-1	P78358	83	97	43.22
14	14	DPB1*01:01	QLSLLMWITQCFLPV	NY-ESO-1	P78358	155	169	43.49
15	15	DPB1*01:01	SRLLEFYLAMPFATP	NY-ESO-1	P78358	85	99	42.44
16	1	DPB1*02:01	CSVYDFFVWLHYYSV	TRP2	O75767	179	193	33
17	2	DPB1*02:01	DFFVWLHYYSVRDTL	TRP2	O75767	183	197	16.72
18	18	DPB1*02:01	FFVWLHYYSVRDTLL	TRP2	O75767	184	198	28.16
19	19	DPB1*02:01	SVYDFFVWLHYYSVR	TRP2	O75767	180	194	15.36
20	20	DPB1*02:01	VYDFFVWLHYYSVRD	TRP2	O75767	181	195	13.62
21	21	DPB1*02:01	YDFFVWLHYYSVRDT	TRP2	O75767	182	196	13.64
22	22	DPB1*02:01	ANDPIFLLHHAFVDS	Tyrosinase	P14679	381	395	43.78
23	23	DPB1*02:01	DPIFLLHHAFVDSIF	Tyrosinase	P14679	383	397	22.28
24	24	DPB1*02:01	EKDKFFAYLTLAKHT	Tyrosinase	P14679	130	144	49.43
25	25	DPB1*02:01	FLLHHAFVDSIFEQW	Tyrosinase	P14679	386	400	32.2
26	26	DPB1*02:01	HAFVDSIFEQWLRRH	Tyrosinase	P14679	390	404	48.15
27	27	DPB1*02:01	HHAFVDSIFEQWLRR	Tyrosinase	P14679	389	403	39.39
28	28	DPB1*02:01	HNRESYMVPFIPLYR	Tyrosinase	P14679	420	434	45.51
29	29	DPB1*02:01	IFLLHHAFVDSIFEQ	Tyrosinase	P14679	385	399	33.72
30	30	DPB1*02:01	KDKFFAYLTLAKHTI	Tyrosinase	P14679	131	145	46.71
31	31	DPB1*02:01	NDPIFLLHHAFVDSI	Tyrosinase	P14679	382	396	26.27
32	32	DPB1*02:01	NRESYMVPFIPLYRN	Tyrosinase	P14679	421	435	43.82
33	33	DPB1*02:01	PIFLLHHAFVDSIFE	Tyrosinase	P14679	384	398	20.9
34	34	DPB1*02:01	AFLTWHRYHLLRLEK	TRP1	P17643	219	233	15.2
35	35	DPB1*02:01	EGPAFLTWHRYHLLR	TRP1	P17643	216	230	16.72

36	36	DPB1*02:01	FLTWHRYHLLRLEKD	TRP1	P17643	220	234	23
37	37	DPB1*02:01	FVWTHYYSVKKTFLG	TRP1	P17643	188	202	30.77
38	38	DPB1*02:01	GPAFLTWHRYHLLRL	TRP1	P17643	217	231	13.09
39	39	DPB1*02:01	HEGPAFLTWHRYHLL	TRP1	P17643	215	229	26.31
40	40	DPB1*02:01	ISIYNYFVWTHYYSV	TRP1	P17643	182	196	9.41
41	41	DPB1*02:01	IYNYFVWTHYYSVKK	TRP1	P17643	184	198	4.57
42	42	DPB1*02:01	NYFVWTHYYSVKKTF	TRP1	P17643	186	200	5.5
43	43	DPB1*02:01	PAFLTWHRYHLLRLE	TRP1	P17643	218	232	14.24
44	44	DPB1*02:01	SIYNYFVWTHYYSVK	TRP1	P17643	183	197	5.66
45	45	DPB1*02:01	YFVWTHYYSVKKTFL	TRP1	P17643	187	201	7.2
46	46	DPB1*02:01	YNYFVWTHYYSVKKT	TRP1	P17643	185	199	4.64
47	47	DPB1*02:01	CILESLFRAVITKKV	MAGE1	P43355	92	106	39.93
48	48	DPB1*02:01	GPSTSCILESLFRAV	MAGE1	P43355	87	101	29.28
49	49	DPB1*02:01	PSTSCILESLFRAVI	MAGE1	P43355	88	102	25.38
50	50	DPB1*02:01	SCILESLFRAVITKK	MAGE1	P43355	91	105	21.1
51	51	DPB1*02:01	STSCILESLFRAVIT	MAGE1	P43355	89	103	23.29
52	52	DPB1*02:01	TSCILESLFRAVITK	MAGE1	P43355	90	104	14.8
53	53	DPB1*02:01	VADLVGFLLLKYRAR	MAGE1	P43355	106	120	48.01
54	54	DPB1*02:01	LLMWITQCFLPVFLA	NY-ESO-1	P78358	158	172	48.52
55	55	DPB1*02:01	LMWITQCFLPVFLAQ	NY-ESO-1	P78358	159	173	33.13
56	56	DPB1*02:01	LSLLMWITQCFLPVF	NY-ESO-1	P78358	156	170	36.97
57	57	DPB1*02:01	MWITQCFLPVFLAQP	NY-ESO-1	P78358	160	174	29.27
58	58	DPB1*02:01	QLSLLMWITQCFLPV	NY-ESO-1	P78358	155	169	34.36
59	59	DPB1*02:01	RLLEFYLAMPFATPM	NY-ESO-1	P78358	86	100	47.67
60	60	DPB1*02:01	SRLLEFYLAMPFATP	NY-ESO-1	P78358	85	99	47.03
61	1	DPB1*02:02	CSVYDFFVWLHYYSV	TRP2	O75767	179	193	28.85
62	2	DPB1*02:02	DFFVWLHYYSVRDTL	TRP2	O75767	183	197	17.81
63	3	DPB1*02:02	FFVWLHYYSVRDTLL	TRP2	O75767	184	198	28.6
64	4	DPB1*02:02	RDTLLGGFFPWLKVY	TRP2	O75767	194	208	49.46
65	5	DPB1*02:02	SVYDFFVWLHYYSVR	TRP2	O75767	180	194	15.68
66	6	DPB1*02:02	VYDFFVWLHYYSVRD	TRP2	O75767	181	195	14.25
67	7	DPB1*02:02	YDFFVWLHYYSVRDT	TRP2	O75767	182	196	15
68	8	DPB1*02:02	ANDPIFLLHHAFVDS	Tyrosinase	P14679	381	395	42.84
69	9	DPB1*02:02	DPIFLLHHAFVDSIF	Tyrosinase	P14679	383	397	22.77
70	10	DPB1*02:02	EKDKFFAYLTLAKHT	Tyrosinase	P14679	130	144	41.62
71	11	DPB1*02:02	FLLHHAFVDSIFEQW	Tyrosinase	P14679	386	400	33.94
72	12	DPB1*02:02	HAFVDSIFEQWLRRH	Tyrosinase	P14679	390	404	42.36
73	13	DPB1*02:02	HHAFVDSIFEQWLRR	Tyrosinase	P14679	389	403	32.91
74	14	DPB1*02:02	HNRESYMVPFIPLYR	Tyrosinase	P14679	420	434	43.13

75	15	DPB1*02:02	IFLLHHAFVDSIFEQ	Tyrosinase	P14679	385	399	34.65
76	16	DPB1*02:02	KDKFFAYLTLAKHTI	Tyrosinase	P14679	131	145	43.02
77	17	DPB1*02:02	NDPIFLLHHAFVDSI	Tyrosinase	P14679	382	396	27.56
78	18	DPB1*02:02	NRESYMVPFIPLYRN	Tyrosinase	P14679	421	435	41.71
79	19	DPB1*02:02	PEKDKFFAYLTLAKH	Tyrosinase	P14679	129	143	45.23
80	20	DPB1*02:02	PIFLLHHAFVDSIFE	Tyrosinase	P14679	384	398	21.63
81	21	DPB1*02:02	YDLFVWMHYYVSMDA	Tyrosinase	P14679	173	187	45.78
82	22	DPB1*02:02	AFLTWHRYHLLRLEK	TRP1	P17643	219	233	14.6
83	23	DPB1*02:02	EGPAFLTWHRYHLLR	TRP1	P17643	216	230	16.04
84	24	DPB1*02:02	FLTWHRYHLLRLEKD	TRP1	P17643	220	234	21.67
85	25	DPB1*02:02	FVWTHYYSVKKTFLG	TRP1	P17643	188	202	32.57
86	26	DPB1*02:02	GPAFLTWHRYHLLRL	TRP1	P17643	217	231	12.96
87	27	DPB1*02:02	HEGPAFLTWHRYHLL	TRP1	P17643	215	229	21.73
88	28	DPB1*02:02	ISIYNYFVWTHYYSV	TRP1	P17643	182	196	9.43
89	29	DPB1*02:02	IYNYFVWTHYYSVKK	TRP1	P17643	184	198	5.36
90	30	DPB1*02:02	NYFVWTHYYSVKKTF	TRP1	P17643	186	200	6.63
91	31	DPB1*02:02	PAFLTWHRYHLLRLE	TRP1	P17643	218	232	13.5
92	32	DPB1*02:02	SIYNYFVWTHYYSVK	TRP1	P17643	183	197	6.31
93	33	DPB1*02:02	YFVWTHYYSVKKTFL	TRP1	P17643	187	201	8.43
94	34	DPB1*02:02	YNYFVWTHYYSVKKT	TRP1	P17643	185	199	5.48
95	35	DPB1*02:02	CILESLFRAVITKKV	MAGE1	P43355	92	106	38.06
96	36	DPB1*02:02	GPSTSCILESLFRAV	MAGE1	P43355	87	101	26.48
97	37	DPB1*02:02	KKVADLVGFLLLKYR	MAGE1	P43355	104	118	45.02
98	38	DPB1*02:02	PRALAETSYVKVLEY	MAGE1	P43355	268	282	41.63
99	39	DPB1*02:02	PSTSCILESLFRAVI	MAGE1	P43355	88	102	21.88
100	40	DPB1*02:02	RALAETSYVKVLEYV	MAGE1	P43355	269	283	48.52
101	41	DPB1*02:02	SCILESLFRAVITKK	MAGE1	P43355	91	105	21.07
102	42	DPB1*02:02	STSCILESLFRAVIT	MAGE1	P43355	89	103	20.68
103	43	DPB1*02:02	TSCILESLFRAVITK	MAGE1	P43355	90	104	14.89
104	44	DPB1*02:02	VADLVGFLLLKYRAR	MAGE1	P43355	106	120	45.69
105	45	DPB1*02:02	LLMWITQCFLPVFLA	NY-ESO-1	P78358	158	172	44.65
106	46	DPB1*02:02	LMWITQCFLPVFLAQ	NY-ESO-1	P78358	159	173	31.31
107	47	DPB1*02:02	LSLLMWITQCFLPVF	NY-ESO-1	P78358	156	170	34.46
108	48	DPB1*02:02	MWITQCFLPVFLAQP	NY-ESO-1	P78358	160	174	27.56
109	49	DPB1*02:02	PESRLLEFYLAMPFA	NY-ESO-1	P78358	83	97	48.33
110	50	DPB1*02:02	QLSLLMWITQCFLPV	NY-ESO-1	P78358	155	169	33.15
111	51	DPB1*02:02	SRLLEFYLAMPFATP	NY-ESO-1	P78358	85	99	45.05
112	52	DPB1*04:01	CSVYDFFVWLHYYSV	TRP2	O75767	179	193	31.35
113	53	DPB1*04:01	DFFVWLHYYSVRDTL	TRP2	O75767	183	197	25.56

114	54	DPB1*04:01	SVYDFFVWLHYYSVR	TRP2	O75767	180	194	19.26
115	55	DPB1*04:01	VYDFFVWLHYYSVRD	TRP2	O75767	181	195	17.12
116	56	DPB1*04:01	YDFFVWLHYYSVRDT	TRP2	O75767	182	196	18.02
117	57	DPB1*04:01	AFVDSIFEQWLRRHR	Tyrosinase	P14679	391	405	44.17
118	58	DPB1*04:01	FLLHHAFVDSIFEQW	Tyrosinase	P14679	386	400	45.32
119	59	DPB1*04:01	HAFVDSIFEQWLRRH	Tyrosinase	P14679	390	404	23.3
120	60	DPB1*04:01	HHAFVDSIFEQWLRR	Tyrosinase	P14679	389	403	16.9
121	61	DPB1*04:01	HNRESYMVPFIPLYR	Tyrosinase	P14679	420	434	48.89
122	62	DPB1*04:01	LHHAFVDSIFEQWLR	Tyrosinase	P14679	388	402	26.61
123	63	DPB1*04:01	LLHHAFVDSIFEQWL	Tyrosinase	P14679	387	401	46.15
124	64	DPB1*04:01	NRESYMVPFIPLYRN	Tyrosinase	P14679	421	435	49.59
125	65	DPB1*04:01	ISIYNYFVWTHYYSV	TRP1	P17643	182	196	6.95
126	66	DPB1*04:01	IYNYFVWTHYYSVKK	TRP1	P17643	184	198	4.56
127	67	DPB1*04:01	NYFVWTHYYSVKKTF	TRP1	P17643	186	200	6.23
128	68	DPB1*04:01	SIYNYFVWTHYYSVK	TRP1	P17643	183	197	5.03
129	69	DPB1*04:01	YFVWTHYYSVKKTFL	TRP1	P17643	187	201	9.08
130	70	DPB1*04:01	YNYFVWTHYYSVKKT	TRP1	P17643	185	199	4.79
131	71	DPB1*04:01	TSCILESLFRAVITK	MAGE1	P43355	90	104	35.53
132	72	DPB1*04:01	LLMWITQCFLPVFLA	NY-ESO-1	P78358	158	172	25.52
133	73	DPB1*04:01	LMWITQCFLPVFLAQ	NY-ESO-1	P78358	159	173	19.43
134	74	DPB1*04:01	LSLLMWITQCFLPVF	NY-ESO-1	P78358	156	170	16.8
135	75	DPB1*04:01	MWITQCFLPVFLAQP	NY-ESO-1	P78358	160	174	19.17
136	76	DPB1*04:01	QLSLLMWITQCFLPV	NY-ESO-1	P78358	155	169	15.36
137	77	DPB1*04:01	SLLMWITQCFLPVFL	NY-ESO-1	P78358	157	171	27.58
138	78	DPB1*04:01	SRLLEFYLAMPFATP	NY-ESO-1	P78358	85	99	46.39
139	1	DPB1*04:02	ISIYNYFVWTHYYSV	TRP1	P17643	182	196	33.93
140	2	DPB1*04:02	IYNYFVWTHYYSVKK	TRP1	P17643	184	198	13.91
141	3	DPB1*04:02	NYFVWTHYYSVKKTF	TRP1	P17643	186	200	21.07
142	4	DPB1*04:02	SIYNYFVWTHYYSVK	TRP1	P17643	183	197	18.01
143	5	DPB1*04:02	YFVWTHYYSVKKTFL	TRP1	P17643	187	201	32.03
144	6	DPB1*04:02	YNYFVWTHYYSVKKT	TRP1	P17643	185	199	14.45
145	1	DPB1*105:01	ISIYNYFVWTHYYSV	TRP1	P17643	182	196	33.93
146	2	DPB1*105:01	IYNYFVWTHYYSVKK	TRP1	P17643	184	198	13.91
147	3	DPB1*105:01	NYFVWTHYYSVKKTF	TRP1	P17643	186	200	21.07
148	4	DPB1*105:01	SIYNYFVWTHYYSVK	TRP1	P17643	183	197	18.01
149	5	DPB1*105:01	YFVWTHYYSVKKTFL	TRP1	P17643	187	201	32.03
150	6	DPB1*105:01	YNYFVWTHYYSVKKT	TRP1	P17643	185	199	14.45

151	1	DPB1*126:01	CSVYDFFVWLHYYSV	TRP2	O75767	179	193	31.35
152	2	DPB1*126:01	DFFVWLHYYSVRDTL	TRP2	O75767	183	197	25.56
153	3	DPB1*126:01	SVYDFFVWLHYYSVR	TRP2	O75767	180	194	19.26
154	4	DPB1*126:01	VYDFFVWLHYYSVRD	TRP2	O75767	181	195	17.12
155	5	DPB1*126:01	YDFFVWLHYYSVRDT	TRP2	O75767	182	196	18.02
156	6	DPB1*126:01	AFVDSIFEQWLRRHR	Tyrosinase	P14679	391	405	44.17
157	7	DPB1*126:01	FLLHHAFVDSIFEQW	Tyrosinase	P14679	386	400	45.32
158	8	DPB1*126:01	HAFVDSIFEQWLRRH	Tyrosinase	P14679	390	404	23.3
159	9	DPB1*126:01	HHAFVDSIFEQWLRR	Tyrosinase	P14679	389	403	16.9
160	10	DPB1*126:01	HNRESYMVPFIPLYR	Tyrosinase	P14679	420	434	48.89
161	11	DPB1*126:01	LHHAFVDSIFEQWLR	Tyrosinase	P14679	388	402	26.61
162	12	DPB1*126:01	LLHHAFVDSIFEQWL	Tyrosinase	P14679	387	401	46.15
163	13	DPB1*126:01	NRESYMVPFIPLYRN	Tyrosinase	P14679	421	435	49.59
164	14	DPB1*126:01	ISIYNYFVWTHYYSV	TRP1	P17643	182	196	6.95
165	15	DPB1*126:01	IYNYFVWTHYYSVKK	TRP1	P17643	184	198	4.56
166	16	DPB1*126:01	NYFVWTHYYSVKKTF	TRP1	P17643	186	200	6.23
167	17	DPB1*126:01	SIYNYFVWTHYYSVK	TRP1	P17643	183	197	5.03
168	18	DPB1*126:01	YFVWTHYYSVKKTFL	TRP1	P17643	187	201	9.08
169	19	DPB1*126:01	YNYFVWTHYYSVKKT	TRP1	P17643	185	199	4.79
170	20	DPB1*126:01	TSCILESLFRAVITK	MAGE1	P43355	90	104	35.53
171	21	DPB1*126:01	LLMWITQCFLPVFLA	NY-ESO-1	P78358	158	172	25.52
172	22	DPB1*126:01	LMWITQCFLPVFLAQ	NY-ESO-1	P78358	159	173	19.43
173	23	DPB1*126:01	LSLLMWITQCFLPVF	NY-ESO-1	P78358	156	170	16.8
174	24	DPB1*126:01	MWITQCFLPVFLAQP	NY-ESO-1	P78358	160	174	19.17
175	25	DPB1*126:01	QLSLLMWITQCFLPV	NY-ESO-1	P78358	155	169	15.36
176	26	DPB1*126:01	SLLMWITQCFLPVFL	NY-ESO-1	P78358	157	171	27.58
177	27	DPB1*126:01	SRLLEFYLAMPFATP	NY-ESO-1	P78358	85	99	46.39
178	1	DPB1*15:01	DFFVWLHYYSVRDTL	TRP2	O75767	183	197	44.02
179	2	DPB1*15:01	SVYDFFVWLHYYSVR	TRP2	O75767	180	194	36.05
180	3	DPB1*15:01	VYDFFVWLHYYSVRD	TRP2	O75767	181	195	31.55
181	4	DPB1*15:01	YDFFVWLHYYSVRDT	TRP2	O75767	182	196	31.94
182	5	DPB1*15:01	HHAFVDSIFEQWLRR	Tyrosinase	P14679	389	403	41.88
183	6	DPB1*15:01	HNRESYMVPFIPLYR	Tyrosinase	P14679	420	434	37.46
184	7	DPB1*15:01	NRESYMVPFIPLYRN	Tyrosinase	P14679	421	435	35.48
185	8	DPB1*15:01	RESYMVPFIPLYRNG	Tyrosinase	P14679	422	436	48.09
186	9	DPB1*15:01	ISIYNYFVWTHYYSV	TRP1	P17643	182	196	24.75
187	10	DPB1*15:01	IYNYFVWTHYYSVKK	TRP1	P17643	184	198	14.2
188	11	DPB1*15:01	NYFVWTHYYSVKKTF	TRP1	P17643	186	200	20.85
189	12	DPB1*15:01	SIYNYFVWTHYYSVK	TRP1	P17643	183	197	16.92

190	13	DPB1*15:01	YFVWTHYYSVKKTFL	TRP1	P17643	187	201	32.01
191	14	DPB1*15:01	YNYFVWTHYYSVKKT	TRP1	P17643	185	199	14.8
192	15	DPB1*15:01	ADLVGFLLLKYRARE	MAGE1	P43355	107	121	37.55
193	16	DPB1*15:01	KKVADLVGFLLLKYR	MAGE1	P43355	104	118	26.21
194	17	DPB1*15:01	KVADLVGFLLLKYRA	MAGE1	P43355	105	119	31.97
195	18	DPB1*15:01	TKKVADLVGFLLLKY	MAGE1	P43355	103	117	36.01
196	19	DPB1*15:01	TSCILESLFRAVITK	MAGE1	P43355	90	104	43.93
197	20	DPB1*15:01	VADLVGFLLLKYRAR	MAGE1	P43355	106	120	29.33
198	21	DPB1*15:01	ESRLLEFYLAMPFAT	NY-ESO-1	P78358	84	98	43.89
199	22	DPB1*15:01	GPESRLLEFYLAMPF	NY-ESO-1	P78358	82	96	34.85
200	23	DPB1*15:01	LLMWITQCFLPVFLA	NY-ESO-1	P78358	158	172	49.16
201	24	DPB1*15:01	LMWITQCFLPVFLAQ	NY-ESO-1	P78358	159	173	41.42
202	25	DPB1*15:01	LSLLMWITQCFLPVF	NY-ESO-1	P78358	156	170	44.12
203	26	DPB1*15:01	MWITQCFLPVFLAQP	NY-ESO-1	P78358	160	174	46.54
204	27	DPB1*15:01	PESRLLEFYLAMPFA	NY-ESO-1	P78358	83	97	33.96
205	28	DPB1*15:01	QLSLLMWITQCFLPV	NY-ESO-1	P78358	155	169	48.19
206	29	DPB1*15:01	RLLEFYLAMPFATPM	NY-ESO-1	P78358	86	100	45.06
207	30	DPB1*15:01	SRLLEFYLAMPFATP	NY-ESO-1	P78358	85	99	36.24
208	1	DPB1*16:01	AFLTWHRYHLLRLEK	TRP1	P17643	219	233	46.45
209	2	DPB1*16:01	GPAFLTWHRYHLLRL	TRP1	P17643	217	231	44.13
210	3	DPB1*16:01	ISIYNYFVWTHYYSV	TRP1	P17643	182	196	34.13
211	4	DPB1*16:01	IYNYFVWTHYYSVKK	TRP1	P17643	184	198	13.16
212	5	DPB1*16:01	NYFVWTHYYSVKKTF	TRP1	P17643	186	200	18.61
213	6	DPB1*16:01	PAFLTWHRYHLLRLE	TRP1	P17643	218	232	44.78
214	7	DPB1*16:01	SIYNYFVWTHYYSVK	TRP1	P17643	183	197	19.26
215	8	DPB1*16:01	YFVWTHYYSVKKTFL	TRP1	P17643	187	201	25.97
216	9	DPB1*16:01	YNYFVWTHYYSVKKT	TRP1	P17643	185	199	13.47
217	1	DPB1*19:01	IYNYFVWTHYYSVKK	TRP1	P17643	184	198	25.06
218	2	DPB1*19:01	NYFVWTHYYSVKKTF	TRP1	P17643	186	200	37.68
219	3	DPB1*19:01	SIYNYFVWTHYYSVK	TRP1	P17643	183	197	32.27
220	4	DPB1*19:01	YNYFVWTHYYSVKKT	TRP1	P17643	185	199	25.99
221	1	DPB1*23:01	CSVYDFFVWLHYYSV	TRP2	O75767	179	193	31.35
222	2	DPB1*23:01	DFFVWLHYYSVRDTL	TRP2	O75767	183	197	25.56
223	3	DPB1*23:01	SVYDFFVWLHYYSVR	TRP2	O75767	180	194	19.26
224	4	DPB1*23:01	VYDFFVWLHYYSVRD	TRP2	O75767	181	195	17.12
225	5	DPB1*23:01	YDFFVWLHYYSVRDT	TRP2	O75767	182	196	18.02
226	6	DPB1*23:01	AFVDSIFEQWLRRHR	Tyrosinase	P14679	391	405	44.17

227	7	DPB1*23:01	FLLHHAFVDSIFEQW	Tyrosinase	P14679	386	400	45.32
228	8	DPB1*23:01	HAFVDSIFEQWLRRH	Tyrosinase	P14679	390	404	23.3
229	9	DPB1*23:01	HHAFVDSIFEQWLRR	Tyrosinase	P14679	389	403	16.9
230	10	DPB1*23:01	HNRESYMVPFIPLYR	Tyrosinase	P14679	420	434	48.89
231	11	DPB1*23:01	LHHAFVDSIFEQWLR	Tyrosinase	P14679	388	402	26.61
232	12	DPB1*23:01	LLHHAFVDSIFEQWL	Tyrosinase	P14679	387	401	46.15
233	13	DPB1*23:01	NRESYMVPFIPLYRN	Tyrosinase	P14679	421	435	49.59
234	14	DPB1*23:01	ISIYNYFVWTHYYSV	TRP1	P17643	182	196	6.95
235	15	DPB1*23:01	IYNYFVWTHYYSVKK	TRP1	P17643	184	198	4.56
236	16	DPB1*23:01	NYFVWTHYYSVKKTF	TRP1	P17643	186	200	6.23
237	17	DPB1*23:01	SIYNYFVWTHYYSVK	TRP1	P17643	183	197	5.03
238	18	DPB1*23:01	YFVWTHYYSVKKTFL	TRP1	P17643	187	201	9.08
239	19	DPB1*23:01	YNYFVWTHYYSVKKT	TRP1	P17643	185	199	4.79
240	20	DPB1*23:01	TSCILESLFRAVITK	MAGE1	P43355	90	104	35.53
241	21	DPB1*23:01	LLMWITQCFLPVFLA	NY-ESO-1	P78358	158	172	25.52
242	22	DPB1*23:01	LMWITQCFLPVFLAQ	NY-ESO-1	P78358	159	173	19.43
243	23	DPB1*23:01	LSLLMWITQCFLPVF	NY-ESO-1	P78358	156	170	16.8
244	24	DPB1*23:01	MWITQCFLPVFLAQP	NY-ESO-1	P78358	160	174	19.17
245	25	DPB1*23:01	QLSLLMWITQCFLPV	NY-ESO-1	P78358	155	169	15.36
246	26	DPB1*23:01	SLLMWITQCFLPVFL	NY-ESO-1	P78358	157	171	27.58
247	27	DPB1*23:01	SRLLEFYLAMPFATP	NY-ESO-1	P78358	85	99	46.39
248	1	DPB1*33:01	CSVYDFFVWLHYYSV	TRP2	O75767	179	193	12.36
249	2	DPB1*33:01	DFFVWLHYYSVRDTL	TRP2	O75767	183	197	8.37
250	3	DPB1*33:01	DTLLGGFFPWLKVYY	TRP2	O75767	195	209	29.79
251	4	DPB1*33:01	FFPWLKVYYYRFVIG	TRP2	O75767	201	215	43.88
252	5	DPB1*33:01	FFVWLHYYSVRDTLL	TRP2	O75767	184	198	12.95
253	6	DPB1*33:01	FVIGLRVWQWEVISC	TRP2	O75767	212	226	43.8
254	7	DPB1*33:01	FVWLHYYSVRDTLLG	TRP2	O75767	185	199	36.94
255	8	DPB1*33:01	GGFFPWLKVYYYRFV	TRP2	O75767	199	213	38.84
256	9	DPB1*33:01	GLRVWQWEVISCKLI	TRP2	O75767	215	229	45.96
257	10	DPB1*33:01	IGLRVWQWEVISCKL	TRP2	O75767	214	228	34.14
258	11	DPB1*33:01	KVYYYRFVIGLRVWQ	TRP2	O75767	206	220	45.12
259	12	DPB1*33:01	LGGFFPWLKVYYYRF	TRP2	O75767	198	212	43.46
260	13	DPB1*33:01	PWLKVYYYRFVIGLR	TRP2	O75767	203	217	44.82
261	14	DPB1*33:01	RDTLLGGFFPWLKVY	TRP2	O75767	194	208	19.47
262	15	DPB1*33:01	RFVIGLRVWQWEVIS	TRP2	O75767	211	225	27.72
263	16	DPB1*33:01	SVRDTLLGGFFPWLK	TRP2	O75767	192	206	35.17
264	17	DPB1*33:01	SVYDFFVWLHYYSVR	TRP2	O75767	180	194	7.64
265	18	DPB1*33:01	TLLGGFFPWLKVYYY	TRP2	O75767	196	210	39.8

266	19	DPB1*33:01	VIGLRVWQWEVISCK	TRP2	O75767	213	227	28.38
267	20	DPB1*33:01	VRDTLLGGFFPWLKV	TRP2	O75767	193	207	22.03
268	21	DPB1*33:01	VYDFFVWLHYYSVRD	TRP2	O75767	181	195	6.9
269	22	DPB1*33:01	WLKVYYYRFVIGLRV	TRP2	O75767	204	218	41.22
270	23	DPB1*33:01	YDFFVWLHYYSVRDT	TRP2	O75767	182	196	7.06
271	24	DPB1*33:01	YRFVIGLRVWQWEVI	TRP2	O75767	210	224	30.93
272	25	DPB1*33:01	AFVDSIFEQWLRRHR	Tyrosinase	P14679	391	405	24.56
273	26	DPB1*33:01	ANDPIFLLHHAFVDS	Tyrosinase	P14679	381	395	18.06
274	27	DPB1*33:01	APEKDKFFAYLTLAK	Tyrosinase	P14679	128	142	32.56
275	28	DPB1*33:01	DKFFAYLTLAKHTIS	Tyrosinase	P14679	132	146	33.5
276	29	DPB1*33:01	DLFVWMHYYVSMDAL	Tyrosinase	P14679	174	188	19.62
277	30	DPB1*33:01	DPIFLLHHAFVDSIF	Tyrosinase	P14679	383	397	12.43
278	31	DPB1*33:01	EAPAFLPWHRLFLLR	Tyrosinase	P14679	203	217	47.39
279	32	DPB1*33:01	EKDKFFAYLTLAKHT	Tyrosinase	P14679	130	144	22.68
280	33	DPB1*33:01	ESYMVPFIPLYRNGD	Tyrosinase	P14679	423	437	47.37
281	34	DPB1*33:01	FLLHHAFVDSIFEQW	Tyrosinase	P14679	386	400	13.93
282	35	DPB1*33:01	FVWMHYYVSMDALLG	Tyrosinase	P14679	176	190	41.15
283	36	DPB1*33:01	GHNRESYMVPFIPLY	Tyrosinase	P14679	419	433	23.48
284	37	DPB1*33:01	HAFVDSIFEQWLRRH	Tyrosinase	P14679	390	404	16.76
285	38	DPB1*33:01	HHAFVDSIFEQWLRR	Tyrosinase	P14679	389	403	13.53
286	39	DPB1*33:01	HNRESYMVPFIPLYR	Tyrosinase	P14679	420	434	17.46
287	40	DPB1*33:01	IFLLHHAFVDSIFEQ	Tyrosinase	P14679	385	399	17.14
288	41	DPB1*33:01	IGHNRESYMVPFIPL	Tyrosinase	P14679	418	432	35.97
289	42	DPB1*33:01	INIYDLFVWMHYYVS	Tyrosinase	P14679	170	184	40.06
290	43	DPB1*33:01	IYDLFVWMHYYVSMD	Tyrosinase	P14679	172	186	19.04
291	44	DPB1*33:01	KDKFFAYLTLAKHTI	Tyrosinase	P14679	131	145	23.41
292	45	DPB1*33:01	KFFAYLTLAKHTISS	Tyrosinase	P14679	133	147	48.9
293	46	DPB1*33:01	LFVWMHYYVSMDALL	Tyrosinase	P14679	175	189	29.2
294	47	DPB1*33:01	LHHAFVDSIFEQWLR	Tyrosinase	P14679	388	402	20.88
295	48	DPB1*33:01	LLHHAFVDSIFEQWL	Tyrosinase	P14679	387	401	27.29
296	49	DPB1*33:01	LSAPEKDKFFAYLTL	Tyrosinase	P14679	126	140	47.38
297	50	DPB1*33:01	NDPIFLLHHAFVDSI	Tyrosinase	P14679	382	396	13.88
298	51	DPB1*33:01	NIYDLFVWMHYYVSM	Tyrosinase	P14679	171	185	21.92
299	52	DPB1*33:01	NLLSPASFFSSWQIV	Tyrosinase	P14679	261	275	48.78
300	53	DPB1*33:01	NRESYMVPFIPLYRN	Tyrosinase	P14679	421	435	17.37
301	54	DPB1*33:01	PEKDKFFAYLTLAKH	Tyrosinase	P14679	129	143	22.14
302	55	DPB1*33:01	PIFLLHHAFVDSIFE	Tyrosinase	P14679	384	398	12
303	56	DPB1*33:01	RESYMVPFIPLYRNG	Tyrosinase	P14679	422	436	22.51
304	57	DPB1*33:01	SAPEKDKFFAYLTLA	Tyrosinase	P14679	127	141	39.98
305	58	DPB1*33:01	SPASFFSSWQIVCSR	Tyrosinase	P14679	264	278	49

306	59	DPB1*33:01	WHRLFLLRWEQEIQK	Tyrosinase	P14679	210	224	46.08
307	60	DPB1*33:01	YDLFVWMHYYVSMDA	Tyrosinase	P14679	173	187	17.24
308	61	DPB1*33:01	AFLTWHRYHLLRLEK	TRP1	P17643	219	233	7.44
309	62	DPB1*33:01	ALIFGTASYLIRARR	TRP1	P17643	491	505	38.41
310	63	DPB1*33:01	DPIFVLLHTFTDAVF	TRP1	P17643	397	411	31.99
311	64	DPB1*33:01	EGPAFLTWHRYHLLR	TRP1	P17643	216	230	8.3
312	65	DPB1*33:01	FLTWHRYHLLRLEKD	TRP1	P17643	220	234	10.71
313	66	DPB1*33:01	FVWTHYYSVKKTFLG	TRP1	P17643	188	202	16.66
314	67	DPB1*33:01	GPAFLTWHRYHLLRL	TRP1	P17643	217	231	6.81
315	68	DPB1*33:01	HEGPAFLTWHRYHLL	TRP1	P17643	215	229	10.01
316	69	DPB1*33:01	IFVLLHTFTDAVFDE	TRP1	P17643	399	413	47.53
317	70	DPB1*33:01	ISIYNYFVWTHYYSV	TRP1	P17643	182	196	4.95
318	71	DPB1*33:01	IYNYFVWTHYYSVKK	TRP1	P17643	184	198	3.5
319	72	DPB1*33:01	KLLSLGCIFFPLLLF	TRP1	P17643	5	19	45.93
320	73	DPB1*33:01	LSLGCIFFPLLLFQQ	TRP1	P17643	7	21	43.26
321	74	DPB1*33:01	LTWHRYHLLRLEKDM	TRP1	P17643	221	235	27.66
322	75	DPB1*33:01	LVALIFGTASYLIRA	TRP1	P17643	489	503	46.8
323	76	DPB1*33:01	NDPIFVLLHTFTDAV	TRP1	P17643	396	410	37.71
324	77	DPB1*33:01	NYFVWTHYYSVKKTF	TRP1	P17643	186	200	4.16
325	78	DPB1*33:01	PAFLTWHRYHLLRLE	TRP1	P17643	218	232	6.92
326	79	DPB1*33:01	PIFVLLHTFTDAVFD	TRP1	P17643	398	412	30.61
327	80	DPB1*33:01	SIYNYFVWTHYYSVK	TRP1	P17643	183	197	3.78
328	81	DPB1*33:01	SLGCIFFPLLLFQQA	TRP1	P17643	8	22	47.48
329	82	DPB1*33:01	VALIFGTASYLIRAR	TRP1	P17643	490	504	37.43
330	83	DPB1*33:01	YFVWTHYYSVKKTFL	TRP1	P17643	187	201	5.02
331	84	DPB1*33:01	YNYFVWTHYYSVKKT	TRP1	P17643	185	199	3.63
332	85	DPB1*33:01	CVLYRYGSFSVTLDI	PMEL17	P40967	475	489	31.94
333	86	DPB1*33:01	DCVLYRYGSFSVTLD	PMEL17	P40967	474	488	34.01
334	87	DPB1*33:01	LDCVLYRYGSFSVTL	PMEL17	P40967	473	487	36.17
335	88	DPB1*33:01	LYRYGSFSVTLDIVQ	PMEL17	P40967	477	491	45.7
336	89	DPB1*33:01	PLDCVLYRYGSFSVT	PMEL17	P40967	472	486	48.96
337	90	DPB1*33:01	VLYRYGSFSVTLDIV	PMEL17	P40967	476	490	26.84
338	91	DPB1*33:01	ADLVGFLLLKYRARE	MAGE1	P43355	107	121	24.25
339	92	DPB1*33:01	ALAETSYVKVLEYVI	MAGE1	P43355	270	284	42.04
340	93	DPB1*33:01	ARVRFFFPSLREAAL	MAGE1	P43355	288	302	39.02
341	94	DPB1*33:01	CILESLFRAVITKKV	MAGE1	P43355	92	106	19.12
342	95	DPB1*33:01	DLVGFLLLKYRAREP	MAGE1	P43355	108	122	33.68
343	96	DPB1*33:01	GPRALAETSYVKVLE	MAGE1	P43355	267	281	24.39
344	97	DPB1*33:01	GPSTSCILESLFRAV	MAGE1	P43355	87	101	12.4
345	98	DPB1*33:01	IKVSARVRFFFPSLR	MAGE1	P43355	284	298	44.3

346	99	DPB1*33:01	ILESLFRAVITKKVA	MAGE1	P43355	93	107	41.69
347	100	DPB1*33:01	KKVADLVGFLLLKYR	MAGE1	P43355	104	118	17.7
348	101	DPB1*33:01	KVADLVGFLLLKYRA	MAGE1	P43355	105	119	22.96
349	102	DPB1*33:01	KVSARVRFFFPSLRE	MAGE1	P43355	285	299	40.46
350	103	DPB1*33:01	LWGPRALAETSYVKV	MAGE1	P43355	265	279	45.55
351	104	DPB1*33:01	PRALAETSYVKVLEY	MAGE1	P43355	268	282	18.8
352	105	DPB1*33:01	PSTSCILESLFRAVI	MAGE1	P43355	88	102	11.82
353	106	DPB1*33:01	QDLVQEKYLEYRQVP	MAGE1	P43355	241	255	37.23
354	107	DPB1*33:01	RALAETSYVKVLEYV	MAGE1	P43355	269	283	20.19
355	108	DPB1*33:01	RVRFFFPSLREAALR	MAGE1	P43355	289	303	47.68
356	109	DPB1*33:01	SARVRFFFPSLREAA	MAGE1	P43355	287	301	39.28
357	110	DPB1*33:01	SCILESLFRAVITKK	MAGE1	P43355	91	105	12.55
358	111	DPB1*33:01	STSCILESLFRAVIT	MAGE1	P43355	89	103	11.81
359	112	DPB1*33:01	TKKVADLVGFLLLKY	MAGE1	P43355	103	117	25.63
360	113	DPB1*33:01	TQDLVQEKYLEYRQV	MAGE1	P43355	240	254	34.55
361	114	DPB1*33:01	TSCILESLFRAVITK	MAGE1	P43355	90	104	9.3
362	115	DPB1*33:01	VADLVGFLLLKYRAR	MAGE1	P43355	106	120	20.34
363	116	DPB1*33:01	VSARVRFFFPSLREA	MAGE1	P43355	286	300	34.91
364	117	DPB1*33:01	WGPRALAETSYVKVL	MAGE1	P43355	266	280	23.48
365	118	DPB1*33:01	GPRALAETSYVKVLE	MAGE4	P43358	275	289	24.39
366	119	DPB1*33:01	LWGPRALAETSYVKV	MAGE4	P43358	273	287	45.55
367	120	DPB1*33:01	PRALAETSYVKVLEH	MAGE4	P43358	276	290	24.55
368	121	DPB1*33:01	RALAETSYVKVLEHV	MAGE4	P43358	277	291	25.83
369	122	DPB1*33:01	TQDWVQENYLEYRQV	MAGE4	P43358	248	262	48.55
370	123	DPB1*33:01	WGPRALAETSYVKVL	MAGE4	P43358	274	288	23.48
371	124	DPB1*33:01	EFYLAMPFATPMEAE	NY-ESO-1	P78358	89	103	34.98
372	125	DPB1*33:01	ESRLLEFYLAMPFAT	NY-ESO-1	P78358	84	98	25.01
373	126	DPB1*33:01	GPESRLLEFYLAMPF	NY-ESO-1	P78358	82	96	22.48
374	127	DPB1*33:01	LEFYLAMPFATPMEA	NY-ESO-1	P78358	88	102	23.29
375	128	DPB1*33:01	LLEFYLAMPFATPME	NY-ESO-1	P78358	87	101	21.79
376	129	DPB1*33:01	LLMWITQCFLPVFLA	NY-ESO-1	P78358	158	172	19.81
377	130	DPB1*33:01	LMWITQCFLPVFLAQ	NY-ESO-1	P78358	159	173	14.71
378	131	DPB1*33:01	LSLLMWITQCFLPVF	NY-ESO-1	P78358	156	170	14.2
379	132	DPB1*33:01	MWITQCFLPVFLAQP	NY-ESO-1	P78358	160	174	13.3
380	133	DPB1*33:01	PESRLLEFYLAMPFA	NY-ESO-1	P78358	83	97	22.17
381	134	DPB1*33:01	QLSLLMWITQCFLPV	NY-ESO-1	P78358	155	169	12.9
382	135	DPB1*33:01	RGPESRLLEFYLAMP	NY-ESO-1	P78358	81	95	38.52
383	136	DPB1*33:01	RLLEFYLAMPFATPM	NY-ESO-1	P78358	86	100	18.81
384	137	DPB1*33:01	SLLMWITQCFLPVFL	NY-ESO-1	P78358	157	171	21.9
385	138	DPB1*33:01	SRLLEFYLAMPFATP	NY-ESO-1	P78358	85	99	16.88

386	139	DPB1*33:01	WITQCFLPVFLAQPP	NY-ESO-1	P78358	161	175	26.21
387	140	DPB1*33:01	AARAVFLALSAQLLQ	BAGE	Q13072	2	16	36.86
388	141	DPB1*33:01	ARAVFLALSAQLLQA	BAGE	Q13072	3	17	34.06
389	142	DPB1*33:01	AVFLALSAQLLQARL	BAGE	Q13072	5	19	41.32
390	143	DPB1*33:01	MAARAVFLALSAQLL	BAGE	Q13072	1	15	34.77
391	144	DPB1*33:01	RAVFLALSAQLLQAR	BAGE	Q13072	4	18	25.64
392	145	DPB1*33:01	RLRERKQLVIYEEIS	SSX2	Q16385	167	181	45.34
393	1	DPB1*34:01	ISIYNYFVWTHYYSV	TRP1	P17643	182	196	45.77
394	2	DPB1*34:01	IYNYFVWTHYYSVKK	TRP1	P17643	184	198	19.23
395	3	DPB1*34:01	NYFVWTHYYSVKKTF	TRP1	P17643	186	200	25.7
396	4	DPB1*34:01	SIYNYFVWTHYYSVK	TRP1	P17643	183	197	25.95
397	5	DPB1*34:01	YFVWTHYYSVKKTFL	TRP1	P17643	187	201	41.02
398	6	DPB1*34:01	YNYFVWTHYYSVKKT	TRP1	P17643	185	199	19.84
399	1	DPB1*39:01	CSVYDFFVWLHYYSV	TRP2	O75767	179	193	31.35
400	2	DPB1*39:01	DFFVWLHYYSVRDTL	TRP2	O75767	183	197	25.56
401	3	DPB1*39:01	SVYDFFVWLHYYSVR	TRP2	O75767	180	194	19.26
402	4	DPB1*39:01	VYDFFVWLHYYSVRD	TRP2	O75767	181	195	17.12
403	5	DPB1*39:01	YDFFVWLHYYSVRDT	TRP2	O75767	182	196	18.02
404	6	DPB1*39:01	AFVDSIFEQWLRRHR	Tyrosinase	P14679	391	405	44.17
405	7	DPB1*39:01	FLLHHAFVDSIFEQW	Tyrosinase	P14679	386	400	45.32
406	8	DPB1*39:01	HAFVDSIFEQWLRRH	Tyrosinase	P14679	390	404	23.3
407	9	DPB1*39:01	HHAFVDSIFEQWLRR	Tyrosinase	P14679	389	403	16.9
408	10	DPB1*39:01	HNRESYMVPFIPLYR	Tyrosinase	P14679	420	434	48.89
409	11	DPB1*39:01	LHHAFVDSIFEQWLR	Tyrosinase	P14679	388	402	26.61
410	12	DPB1*39:01	LLHHAFVDSIFEQWL	Tyrosinase	P14679	387	401	46.15
411	13	DPB1*39:01	NRESYMVPFIPLYRN	Tyrosinase	P14679	421	435	49.59
412	14	DPB1*39:01	ISIYNYFVWTHYYSV	TRP1	P17643	182	196	6.95
413	15	DPB1*39:01	IYNYFVWTHYYSVKK	TRP1	P17643	184	198	4.56
414	16	DPB1*39:01	NYFVWTHYYSVKKTF	TRP1	P17643	186	200	6.23
415	17	DPB1*39:01	SIYNYFVWTHYYSVK	TRP1	P17643	183	197	5.03
416	18	DPB1*39:01	YFVWTHYYSVKKTFL	TRP1	P17643	187	201	9.08
417	19	DPB1*39:01	YNYFVWTHYYSVKKT	TRP1	P17643	185	199	4.79
418	20	DPB1*39:01	TSCILESLFRAVITK	MAGE1	P43355	90	104	35.53
419	21	DPB1*39:01	LLMWITQCFLPVFLA	NY-ESO-1	P78358	158	172	25.52
420	22	DPB1*39:01	LMWITQCFLPVFLAQ	NY-ESO-1	P78358	159	173	19.43
421	23	DPB1*39:01	LSLLMWITQCFLPVF	NY-ESO-1	P78358	156	170	16.8
422	24	DPB1*39:01	MWITQCFLPVFLAQP	NY-ESO-1	P78358	160	174	19.17
423	25	DPB1*39:01	QLSLLMWITQCFLPV	NY-ESO-1	P78358	155	169	15.36

424	26	DPB1*39:01	SLLMWITQCFLPVFL	NY-ESO-1	P78358	157	171	27.58
425	27	DPB1*39:01	SRLLEFYLAMPFATP	NY-ESO-1	P78358	85	99	46.39
426	1	DPB1*40:01	ISIYNYFVWTHYYSV	TRP1	P17643	182	196	32.71
427	2	DPB1*40:01	IYNYFVWTHYYSVKK	TRP1	P17643	184	198	17.63
428	3	DPB1*40:01	NYFVWTHYYSVKKTF	TRP1	P17643	186	200	25.18
429	4	DPB1*40:01	SIYNYFVWTHYYSVK	TRP1	P17643	183	197	21.08
430	5	DPB1*40:01	YFVWTHYYSVKKTFL	TRP1	P17643	187	201	39.52
431	6	DPB1*40:01	YNYFVWTHYYSVKKT	TRP1	P17643	185	199	18.64
432	7	DPB1*40:01	VADLVGFLLLKYRAR	MAGE1	P43355	106	120	49.91
433	1	DPB1*41:01	AFLTWHRYHLLRLEK	TRP1	P17643	219	233	35.44
434	2	DPB1*41:01	EGPAFLTWHRYHLLR	TRP1	P17643	216	230	41.33
435	3	DPB1*41:01	FLTWHRYHLLRLEKD	TRP1	P17643	220	234	49.77
436	4	DPB1*41:01	GPAFLTWHRYHLLRL	TRP1	P17643	217	231	32.72
437	5	DPB1*41:01	ISIYNYFVWTHYYSV	TRP1	P17643	182	196	39.46
438	6	DPB1*41:01	IYNYFVWTHYYSVKK	TRP1	P17643	184	198	11.61
439	7	DPB1*41:01	NYFVWTHYYSVKKTF	TRP1	P17643	186	200	14.41
440	8	DPB1*41:01	PAFLTWHRYHLLRLE	TRP1	P17643	218	232	32.84
441	9	DPB1*41:01	SIYNYFVWTHYYSVK	TRP1	P17643	183	197	18.13
442	10	DPB1*41:01	YFVWTHYYSVKKTFL	TRP1	P17643	187	201	22.39
443	11	DPB1*41:01	YNYFVWTHYYSVKKT	TRP1	P17643	185	199	11.48
444	1	DPB1*46:01	CSVYDFFVWLHYYSV	TRP2	O75767	179	193	33
445	2	DPB1*46:01	DFFVWLHYYSVRDTL	TRP2	O75767	183	197	16.72
446	3	DPB1*46:01	FFVWLHYYSVRDTLL	TRP2	O75767	184	198	28.16
447	4	DPB1*46:01	SVYDFFVWLHYYSVR	TRP2	O75767	180	194	15.36
448	5	DPB1*46:01	VYDFFVWLHYYSVRD	TRP2	O75767	181	195	13.62
449	6	DPB1*46:01	YDFFVWLHYYSVRDT	TRP2	O75767	182	196	13.64
450	7	DPB1*46:01	ANDPIFLLHHAFVDS	Tyrosinase	P14679	381	395	43.78
451	8	DPB1*46:01	DPIFLLHHAFVDSIF	Tyrosinase	P14679	383	397	22.28
452	9	DPB1*46:01	EKDKFFAYLTLAKHT	Tyrosinase	P14679	130	144	49.43
453	10	DPB1*46:01	FLLHHAFVDSIFEQW	Tyrosinase	P14679	386	400	32.2
454	11	DPB1*46:01	HAFVDSIFEQWLRRH	Tyrosinase	P14679	390	404	48.15
455	12	DPB1*46:01	HHAFVDSIFEQWLRR	Tyrosinase	P14679	389	403	39.39
456	13	DPB1*46:01	HNRESYMVPFIPLYR	Tyrosinase	P14679	420	434	45.51
457	14	DPB1*46:01	IFLLHHAFVDSIFEQ	Tyrosinase	P14679	385	399	33.72
458	15	DPB1*46:01	KDKFFAYLTLAKHTI	Tyrosinase	P14679	131	145	46.71
459	16	DPB1*46:01	NDPIFLLHHAFVDSI	Tyrosinase	P14679	382	396	26.27
460	17	DPB1*46:01	NRESYMVPFIPLYRN	Tyrosinase	P14679	421	435	43.82

461	18	DPB1*46:01	PIFLLHHAFVDSIFE	Tyrosinase	P14679	384	398	20.9
462	19	DPB1*46:01	AFLTWHRYHLLRLEK	TRP1	P17643	219	233	15.2
463	20	DPB1*46:01	EGPAFLTWHRYHLLR	TRP1	P17643	216	230	16.72
464	21	DPB1*46:01	FLTWHRYHLLRLEKD	TRP1	P17643	220	234	23
465	22	DPB1*46:01	FVWTHYYSVKKTFLG	TRP1	P17643	188	202	30.77
466	23	DPB1*46:01	GPAFLTWHRYHLLRL	TRP1	P17643	217	231	13.09
467	24	DPB1*46:01	HEGPAFLTWHRYHLL	TRP1	P17643	215	229	26.31
468	25	DPB1*46:01	ISIYNYFVWTHYYSV	TRP1	P17643	182	196	9.41
469	26	DPB1*46:01	IYNYFVWTHYYSVKK	TRP1	P17643	184	198	4.57
470	27	DPB1*46:01	NYFVWTHYYSVKKTF	TRP1	P17643	186	200	5.5
471	28	DPB1*46:01	PAFLTWHRYHLLRLE	TRP1	P17643	218	232	14.24
472	29	DPB1*46:01	SIYNYFVWTHYYSVK	TRP1	P17643	183	197	5.66
473	30	DPB1*46:01	YFVWTHYYSVKKTFL	TRP1	P17643	187	201	7.2
474	31	DPB1*46:01	YNYFVWTHYYSVKKT	TRP1	P17643	185	199	4.64
475	32	DPB1*46:01	CILESLFRAVITKKV	MAGE1	P43355	92	106	39.93
476	33	DPB1*46:01	GPSTSCILESLFRAV	MAGE1	P43355	87	101	29.28
477	34	DPB1*46:01	PSTSCILESLFRAVI	MAGE1	P43355	88	102	25.38
478	35	DPB1*46:01	SCILESLFRAVITKK	MAGE1	P43355	91	105	21.1
479	36	DPB1*46:01	STSCILESLFRAVIT	MAGE1	P43355	89	103	23.29
480	37	DPB1*46:01	TSCILESLFRAVITK	MAGE1	P43355	90	104	14.8
481	38	DPB1*46:01	VADLVGFLLLKYRAR	MAGE1	P43355	106	120	48.01
482	39	DPB1*46:01	LLMWITQCFLPVFLA	NY-ESO-1	P78358	158	172	48.52
483	40	DPB1*46:01	LMWITQCFLPVFLAQ	NY-ESO-1	P78358	159	173	33.13
484	41	DPB1*46:01	LSLLMWITQCFLPVF	NY-ESO-1	P78358	156	170	36.97
485	42	DPB1*46:01	MWITQCFLPVFLAQP	NY-ESO-1	P78358	160	174	29.27
486	43	DPB1*46:01	QLSLLMWITQCFLPV	NY-ESO-1	P78358	155	169	34.36
487	44	DPB1*46:01	RLLEFYLAMPFATPM	NY-ESO-1	P78358	86	100	47.67
488	45	DPB1*46:01	SRLLEFYLAMPFATP	NY-ESO-1	P78358	85	99	47.03
489	1	DPB1*47:01	CSVYDFFVWLHYYSV	TRP2	O75767	179	193	28.85
490	2	DPB1*47:01	DFFVWLHYYSVRDTL	TRP2	O75767	183	197	17.81
491	3	DPB1*47:01	FFVWLHYYSVRDTLL	TRP2	O75767	184	198	28.6
492	4	DPB1*47:01	RDTLLGGFFPWLKVY	TRP2	O75767	194	208	49.46
493	5	DPB1*47:01	SVYDFFVWLHYYSVR	TRP2	O75767	180	194	15.68
494	6	DPB1*47:01	VYDFFVWLHYYSVRD	TRP2	O75767	181	195	14.25
495	7	DPB1*47:01	YDFFVWLHYYSVRDT	TRP2	O75767	182	196	15
496	8	DPB1*47:01	ANDPIFLLHHAFVDS	Tyrosinase	P14679	381	395	42.84
497	9	DPB1*47:01	DPIFLLHHAFVDSIF	Tyrosinase	P14679	383	397	22.77
498	10	DPB1*47:01	EKDKFFAYLTLAKHT	Tyrosinase	P14679	130	144	41.62
499	11	DPB1*47:01	FLLHHAFVDSIFEQW	Tyrosinase	P14679	386	400	33.94

500	12	DPB1*47:01	HAFVDSIFEQWLRRH	Tyrosinase	P14679	390	404	42.36
501	13	DPB1*47:01	HHAFVDSIFEQWLRR	Tyrosinase	P14679	389	403	32.91
502	14	DPB1*47:01	HNRESYMVPFIPLYR	Tyrosinase	P14679	420	434	43.13
503	15	DPB1*47:01	IFLLHHAFVDSIFEQ	Tyrosinase	P14679	385	399	34.65
504	16	DPB1*47:01	KDKFFAYLTLAKHTI	Tyrosinase	P14679	131	145	43.02
505	17	DPB1*47:01	NDPIFLLHHAFVDSI	Tyrosinase	P14679	382	396	27.56
506	18	DPB1*47:01	NRESYMVPFIPLYRN	Tyrosinase	P14679	421	435	41.71
507	19	DPB1*47:01	PEKDKFFAYLTLAKH	Tyrosinase	P14679	129	143	45.23
508	20	DPB1*47:01	PIFLLHHAFVDSIFE	Tyrosinase	P14679	384	398	21.63
509	21	DPB1*47:01	YDLFVWMHYYVSMDA	Tyrosinase	P14679	173	187	45.78
510	22	DPB1*47:01	AFLTWHRYHLLRLEK	TRP1	P17643	219	233	14.6
511	23	DPB1*47:01	EGPAFLTWHRYHLLR	TRP1	P17643	216	230	16.04
512	24	DPB1*47:01	FLTWHRYHLLRLEKD	TRP1	P17643	220	234	21.67
513	25	DPB1*47:01	FVWTHYYSVKKTFLG	TRP1	P17643	188	202	32.57
514	26	DPB1*47:01	GPAFLTWHRYHLLRL	TRP1	P17643	217	231	12.96
515	27	DPB1*47:01	HEGPAFLTWHRYHLL	TRP1	P17643	215	229	21.73
516	28	DPB1*47:01	ISIYNYFVWTHYYSV	TRP1	P17643	182	196	9.43
517	29	DPB1*47:01	IYNYFVWTHYYSVKK	TRP1	P17643	184	198	5.36
518	30	DPB1*47:01	NYFVWTHYYSVKKTF	TRP1	P17643	186	200	6.63
519	31	DPB1*47:01	PAFLTWHRYHLLRLE	TRP1	P17643	218	232	13.5
520	32	DPB1*47:01	SIYNYFVWTHYYSVK	TRP1	P17643	183	197	6.31
521	33	DPB1*47:01	YFVWTHYYSVKKTFL	TRP1	P17643	187	201	8.43
522	34	DPB1*47:01	YNYFVWTHYYSVKKT	TRP1	P17643	185	199	5.48
523	35	DPB1*47:01	CILESLFRAVITKKV	MAGE1	P43355	92	106	38.06
524	36	DPB1*47:01	GPSTSCILESLFRAV	MAGE1	P43355	87	101	26.48
525	37	DPB1*47:01	KKVADLVGFLLLKYR	MAGE1	P43355	104	118	45.02
526	38	DPB1*47:01	PRALAETSYVKVLEY	MAGE1	P43355	268	282	41.63
527	39	DPB1*47:01	PSTSCILESLFRAVI	MAGE1	P43355	88	102	21.88
528	40	DPB1*47:01	RALAETSYVKVLEYV	MAGE1	P43355	269	283	48.52
529	41	DPB1*47:01	SCILESLFRAVITKK	MAGE1	P43355	91	105	21.07
530	42	DPB1*47:01	STSCILESLFRAVIT	MAGE1	P43355	89	103	20.68
531	43	DPB1*47:01	TSCILESLFRAVITK	MAGE1	P43355	90	104	14.89
532	44	DPB1*47:01	VADLVGFLLLKYRAR	MAGE1	P43355	106	120	45.69
533	45	DPB1*47:01	LLMWITQCFLPVFLA	NY-ESO-1	P78358	158	172	44.65
534	46	DPB1*47:01	LMWITQCFLPVFLAQ	NY-ESO-1	P78358	159	173	31.31
535	47	DPB1*47:01	LSLLMWITQCFLPVF	NY-ESO-1	P78358	156	170	34.46
536	48	DPB1*47:01	MWITQCFLPVFLAQP	NY-ESO-1	P78358	160	174	27.56
537	49	DPB1*47:01	PESRLLEFYLAMPFA	NY-ESO-1	P78358	83	97	48.33
538	50	DPB1*47:01	QLSLLMWITQCFLPV	NY-ESO-1	P78358	155	169	33.15
539	51	DPB1*47:01	SRLLEFYLAMPFATP	NY-ESO-1	P78358	85	99	45.05

540	1	DPB1*49:01	ISIYNYFVWTHYYSV	TRP1	P17643	182	196	33.93
541	2	DPB1*49:01	IYNYFVWTHYYSVKK	TRP1	P17643	184	198	13.91
542	3	DPB1*49:01	NYFVWTHYYSVKKTF	TRP1	P17643	186	200	21.07
543	4	DPB1*49:01	SIYNYFVWTHYYSVK	TRP1	P17643	183	197	18.01
544	5	DPB1*49:01	YFVWTHYYSVKKTFL	TRP1	P17643	187	201	32.03
545	6	DPB1*49:01	YNYFVWTHYYSVKKT	TRP1	P17643	185	199	14.45
546	1	DPB1*55:01	IYNYFVWTHYYSVKK	TRP1	P17643	184	198	40.78
547	2	DPB1*55:01	YNYFVWTHYYSVKKT	TRP1	P17643	185	199	43.38
548	1	DPB1*71:01	CSVYDFFVWLHYYSV	TRP2	O75767	179	193	12.36
549	2	DPB1*71:01	DFFVWLHYYSVRDTL	TRP2	O75767	183	197	8.37
550	3	DPB1*71:01	DTLLGGFFPWLKVYY	TRP2	O75767	195	209	29.79
551	4	DPB1*71:01	FFPWLKVYYYRFVIG	TRP2	O75767	201	215	43.88
552	5	DPB1*71:01	FFVWLHYYSVRDTLL	TRP2	O75767	184	198	12.95
553	6	DPB1*71:01	FVIGLRVWQWEVISC	TRP2	O75767	212	226	43.8
554	7	DPB1*71:01	FVWLHYYSVRDTLLG	TRP2	O75767	185	199	36.94
555	8	DPB1*71:01	GGFFPWLKVYYYRFV	TRP2	O75767	199	213	38.84
556	9	DPB1*71:01	GLRVWQWEVISCKLI	TRP2	O75767	215	229	45.96
557	10	DPB1*71:01	IGLRVWQWEVISCKL	TRP2	O75767	214	228	34.14
558	11	DPB1*71:01	KVYYYRFVIGLRVWQ	TRP2	O75767	206	220	45.12
559	12	DPB1*71:01	LGGFFPWLKVYYYRF	TRP2	O75767	198	212	43.46
560	13	DPB1*71:01	PWLKVYYYRFVIGLR	TRP2	O75767	203	217	44.82
561	14	DPB1*71:01	RDTLLGGFFPWLKVY	TRP2	O75767	194	208	19.47
562	15	DPB1*71:01	RFVIGLRVWQWEVIS	TRP2	O75767	211	225	27.72
563	16	DPB1*71:01	SVRDTLLGGFFPWLK	TRP2	O75767	192	206	35.17
564	17	DPB1*71:01	SVYDFFVWLHYYSVR	TRP2	O75767	180	194	7.64
565	18	DPB1*71:01	TLLGGFFPWLKVYYY	TRP2	O75767	196	210	39.8
566	19	DPB1*71:01	VIGLRVWQWEVISCK	TRP2	O75767	213	227	28.38
567	20	DPB1*71:01	VRDTLLGGFFPWLKV	TRP2	O75767	193	207	22.03
568	21	DPB1*71:01	VYDFFVWLHYYSVRD	TRP2	O75767	181	195	6.9
569	22	DPB1*71:01	WLKVYYYRFVIGLRV	TRP2	O75767	204	218	41.22
570	23	DPB1*71:01	YDFFVWLHYYSVRDT	TRP2	O75767	182	196	7.06
571	24	DPB1*71:01	YRFVIGLRVWQWEVI	TRP2	O75767	210	224	30.93
572	25	DPB1*71:01	AFVDSIFEQWLRRHR	Tyrosinase	P14679	391	405	24.56
573	26	DPB1*71:01	ANDPIFLLHHAFVDS	Tyrosinase	P14679	381	395	18.06
574	27	DPB1*71:01	APEKDKFFAYLTLAK	Tyrosinase	P14679	128	142	32.56
575	28	DPB1*71:01	DKFFAYLTLAKHTIS	Tyrosinase	P14679	132	146	33.5
576	29	DPB1*71:01	DLFVWMHYYVSMDAL	Tyrosinase	P14679	174	188	19.62

577	30	DPB1*71:01	DPIFLLHHAFVDSIF	Tyrosinase	P14679	383	397	12.43
578	31	DPB1*71:01	EAPAFLPWHRLFLLR	Tyrosinase	P14679	203	217	47.39
579	32	DPB1*71:01	EKDKFFAYLTLAKHT	Tyrosinase	P14679	130	144	22.68
580	33	DPB1*71:01	ESYMVPFIPLYRNGD	Tyrosinase	P14679	423	437	47.37
581	34	DPB1*71:01	FLLHHAFVDSIFEQW	Tyrosinase	P14679	386	400	13.93
582	35	DPB1*71:01	FVWMHYYVSMDALLG	Tyrosinase	P14679	176	190	41.15
583	36	DPB1*71:01	GHNRESYMVPFIPLY	Tyrosinase	P14679	419	433	23.48
584	37	DPB1*71:01	HAFVDSIFEQWLRRH	Tyrosinase	P14679	390	404	16.76
585	38	DPB1*71:01	HHAFVDSIFEQWLRR	Tyrosinase	P14679	389	403	13.53
586	39	DPB1*71:01	HNRESYMVPFIPLYR	Tyrosinase	P14679	420	434	17.46
587	40	DPB1*71:01	IFLLHHAFVDSIFEQ	Tyrosinase	P14679	385	399	17.14
588	41	DPB1*71:01	IGHNRESYMVPFIPL	Tyrosinase	P14679	418	432	35.97
589	42	DPB1*71:01	INIYDLFVWMHYYVS	Tyrosinase	P14679	170	184	40.06
590	43	DPB1*71:01	IYDLFVWMHYYVSMD	Tyrosinase	P14679	172	186	19.04
591	44	DPB1*71:01	KDKFFAYLTLAKHTI	Tyrosinase	P14679	131	145	23.41
592	45	DPB1*71:01	KFFAYLTLAKHTISS	Tyrosinase	P14679	133	147	48.9
593	46	DPB1*71:01	LFVWMHYYVSMDALL	Tyrosinase	P14679	175	189	29.2
594	47	DPB1*71:01	LHHAFVDSIFEQWLR	Tyrosinase	P14679	388	402	20.88
595	48	DPB1*71:01	LLHHAFVDSIFEQWL	Tyrosinase	P14679	387	401	27.29
596	49	DPB1*71:01	LSAPEKDKFFAYLTL	Tyrosinase	P14679	126	140	47.38
597	50	DPB1*71:01	NDPIFLLHHAFVDSI	Tyrosinase	P14679	382	396	13.88
598	51	DPB1*71:01	NIYDLFVWMHYYVSM	Tyrosinase	P14679	171	185	21.92
599	52	DPB1*71:01	NLLSPASFFSSWQIV	Tyrosinase	P14679	261	275	48.78
600	53	DPB1*71:01	NRESYMVPFIPLYRN	Tyrosinase	P14679	421	435	17.37
601	54	DPB1*71:01	PEKDKFFAYLTLAKH	Tyrosinase	P14679	129	143	22.14
602	55	DPB1*71:01	PIFLLHHAFVDSIFE	Tyrosinase	P14679	384	398	12
603	56	DPB1*71:01	RESYMVPFIPLYRNG	Tyrosinase	P14679	422	436	22.51
604	57	DPB1*71:01	SAPEKDKFFAYLTLA	Tyrosinase	P14679	127	141	39.98
605	58	DPB1*71:01	SPASFFSSWQIVCSR	Tyrosinase	P14679	264	278	49
606	59	DPB1*71:01	WHRLFLLRWEQEIQK	Tyrosinase	P14679	210	224	46.08
607	60	DPB1*71:01	YDLFVWMHYYVSMDA	Tyrosinase	P14679	173	187	17.24
608	61	DPB1*71:01	AFLTWHRYHLLRLEK	TRP1	P17643	219	233	7.44
609	62	DPB1*71:01	ALIFGTASYLIRARR	TRP1	P17643	491	505	38.41
610	63	DPB1*71:01	DPIFVLLHTFTDAVF	TRP1	P17643	397	411	31.99
611	64	DPB1*71:01	EGPAFLTWHRYHLLR	TRP1	P17643	216	230	8.3
612	65	DPB1*71:01	FLTWHRYHLLRLEKD	TRP1	P17643	220	234	10.71
613	66	DPB1*71:01	FVWTHYYSVKKTFLG	TRP1	P17643	188	202	16.66
614	67	DPB1*71:01	GPAFLTWHRYHLLRL	TRP1	P17643	217	231	6.81
615	68	DPB1*71:01	HEGPAFLTWHRYHLL	TRP1	P17643	215	229	10.01
616	69	DPB1*71:01	IFVLLHTFTDAVFDE	TRP1	P17643	399	413	47.53

617	70	DPB1*71:01	ISIYNYFVWTHYYSV	TRP1	P17643	182	196	4.95
618	71	DPB1*71:01	IYNYFVWTHYYSVKK	TRP1	P17643	184	198	3.5
619	72	DPB1*71:01	KLLSLGCIFFPLLLF	TRP1	P17643	5	19	45.93
620	73	DPB1*71:01	LSLGCIFFPLLLFQQ	TRP1	P17643	7	21	43.26
621	74	DPB1*71:01	LTWHRYHLLRLEKDM	TRP1	P17643	221	235	27.66
622	75	DPB1*71:01	LVALIFGTASYLIRA	TRP1	P17643	489	503	46.8
623	76	DPB1*71:01	NDPIFVLLHTFTDAV	TRP1	P17643	396	410	37.71
624	77	DPB1*71:01	NYFVWTHYYSVKKTF	TRP1	P17643	186	200	4.16
625	78	DPB1*71:01	PAFLTWHRYHLLRLE	TRP1	P17643	218	232	6.92
626	79	DPB1*71:01	PIFVLLHTFTDAVFD	TRP1	P17643	398	412	30.61
627	80	DPB1*71:01	SIYNYFVWTHYYSVK	TRP1	P17643	183	197	3.78
628	81	DPB1*71:01	SLGCIFFPLLLFQQA	TRP1	P17643	8	22	47.48
629	82	DPB1*71:01	VALIFGTASYLIRAR	TRP1	P17643	490	504	37.43
630	83	DPB1*71:01	YFVWTHYYSVKKTFL	TRP1	P17643	187	201	5.02
631	84	DPB1*71:01	YNYFVWTHYYSVKKT	TRP1	P17643	185	199	3.63
632	85	DPB1*71:01	CVLYRYGSFSVTLDI	PMEL17	P40967	475	489	31.94
633	86	DPB1*71:01	DCVLYRYGSFSVTLD	PMEL17	P40967	474	488	34.01
634	87	DPB1*71:01	LDCVLYRYGSFSVTL	PMEL17	P40967	473	487	36.17
635	88	DPB1*71:01	LYRYGSFSVTLDIVQ	PMEL17	P40967	477	491	45.7
636	89	DPB1*71:01	PLDCVLYRYGSFSVT	PMEL17	P40967	472	486	48.96
637	90	DPB1*71:01	VLYRYGSFSVTLDIV	PMEL17	P40967	476	490	26.84
638	91	DPB1*71:01	ADLVGFLLLKYRARE	MAGE1	P43355	107	121	24.25
639	92	DPB1*71:01	ALAETSYVKVLEYVI	MAGE1	P43355	270	284	42.04
640	93	DPB1*71:01	ARVRFFFPSLREAAL	MAGE1	P43355	288	302	39.02
641	94	DPB1*71:01	CILESLFRAVITKKV	MAGE1	P43355	92	106	19.12
642	95	DPB1*71:01	DLVGFLLLKYRAREP	MAGE1	P43355	108	122	33.68
643	96	DPB1*71:01	GPRALAETSYVKVLE	MAGE1	P43355	267	281	24.39
644	97	DPB1*71:01	GPSTSCILESLFRAV	MAGE1	P43355	87	101	12.4
645	98	DPB1*71:01	IKVSARVRFFFPSLR	MAGE1	P43355	284	298	44.3
646	99	DPB1*71:01	ILESLFRAVITKKVA	MAGE1	P43355	93	107	41.69
647	100	DPB1*71:01	KKVADLVGFLLLKYR	MAGE1	P43355	104	118	17.7
648	101	DPB1*71:01	KVADLVGFLLLKYRA	MAGE1	P43355	105	119	22.96
649	102	DPB1*71:01	KVSARVRFFFPSLRE	MAGE1	P43355	285	299	40.46
650	103	DPB1*71:01	LWGPRALAETSYVKV	MAGE1	P43355	265	279	45.55
651	104	DPB1*71:01	PRALAETSYVKVLEY	MAGE1	P43355	268	282	18.8
652	105	DPB1*71:01	PSTSCILESLFRAVI	MAGE1	P43355	88	102	11.82
653	106	DPB1*71:01	QDLVQEKYLEYRQVP	MAGE1	P43355	241	255	37.23
654	107	DPB1*71:01	RALAETSYVKVLEYV	MAGE1	P43355	269	283	20.19
655	108	DPB1*71:01	RVRFFFPSLREAALR	MAGE1	P43355	289	303	47.68
656	109	DPB1*71:01	SARVRFFFPSLREAA	MAGE1	P43355	287	301	39.28

657	110	DPB1*71:01	SCILESLFRAVITKK	MAGE1	P43355	91	105	12.55
658	111	DPB1*71:01	STSCILESLFRAVIT	MAGE1	P43355	89	103	11.81
659	112	DPB1*71:01	TKKVADLVGFLLLKY	MAGE1	P43355	103	117	25.63
660	113	DPB1*71:01	TQDLVQEKYLEYRQV	MAGE1	P43355	240	254	34.55
661	114	DPB1*71:01	TSCILESLFRAVITK	MAGE1	P43355	90	104	9.3
662	115	DPB1*71:01	VADLVGFLLLKYRAR	MAGE1	P43355	106	120	20.34
663	116	DPB1*71:01	VSARVRFFFPSLREA	MAGE1	P43355	286	300	34.91
664	117	DPB1*71:01	WGPRALAETSYVKVL	MAGE1	P43355	266	280	23.48
665	118	DPB1*71:01	GPRALAETSYVKVLE	MAGE4	P43358	275	289	24.39
666	119	DPB1*71:01	LWGPRALAETSYVKV	MAGE4	P43358	273	287	45.55
667	120	DPB1*71:01	PRALAETSYVKVLEH	MAGE4	P43358	276	290	24.55
668	121	DPB1*71:01	RALAETSYVKVLEHV	MAGE4	P43358	277	291	25.83
669	122	DPB1*71:01	TQDWVQENYLEYRQV	MAGE4	P43358	248	262	48.55
670	123	DPB1*71:01	WGPRALAETSYVKVL	MAGE4	P43358	274	288	23.48
671	124	DPB1*71:01	EFYLAMPFATPMEAE	NY-ESO-1	P78358	89	103	34.98
672	125	DPB1*71:01	ESRLLEFYLAMPFAT	NY-ESO-1	P78358	84	98	25.01
673	126	DPB1*71:01	GPESRLLEFYLAMPF	NY-ESO-1	P78358	82	96	22.48
674	127	DPB1*71:01	LEFYLAMPFATPMEA	NY-ESO-1	P78358	88	102	23.29
675	128	DPB1*71:01	LLEFYLAMPFATPME	NY-ESO-1	P78358	87	101	21.79
676	129	DPB1*71:01	LLMWITQCFLPVFLA	NY-ESO-1	P78358	158	172	19.81
677	130	DPB1*71:01	LMWITQCFLPVFLAQ	NY-ESO-1	P78358	159	173	14.71
678	131	DPB1*71:01	LSLLMWITQCFLPVF	NY-ESO-1	P78358	156	170	14.2
679	132	DPB1*71:01	MWITQCFLPVFLAQP	NY-ESO-1	P78358	160	174	13.3
680	133	DPB1*71:01	PESRLLEFYLAMPFA	NY-ESO-1	P78358	83	97	22.17
681	134	DPB1*71:01	QLSLLMWITQCFLPV	NY-ESO-1	P78358	155	169	12.9
682	135	DPB1*71:01	RGPESRLLEFYLAMP	NY-ESO-1	P78358	81	95	38.52
683	136	DPB1*71:01	RLLEFYLAMPFATPM	NY-ESO-1	P78358	86	100	18.81
684	137	DPB1*71:01	SLLMWITQCFLPVFL	NY-ESO-1	P78358	157	171	21.9
685	138	DPB1*71:01	SRLLEFYLAMPFATP	NY-ESO-1	P78358	85	99	16.88
686	139	DPB1*71:01	WITQCFLPVFLAQPP	NY-ESO-1	P78358	161	175	26.21
687	140	DPB1*71:01	AARAVFLALSAQLLQ	BAGE	Q13072	2	16	36.86
688	141	DPB1*71:01	ARAVFLALSAQLLQA	BAGE	Q13072	3	17	34.06
689	142	DPB1*71:01	AVFLALSAQLLQARL	BAGE	Q13072	5	19	41.32
690	143	DPB1*71:01	MAARAVFLALSAQLL	BAGE	Q13072	1	15	34.77
691	144	DPB1*71:01	RAVFLALSAQLLQAR	BAGE	Q13072	4	18	25.64
692	145	DPB1*71:01	RLRERKQLVIYEEIS	SSX2	Q16385	167	181	45.34
693	1	DPB1*72:01	DFFVWLHYYSVRDTL	TRP2	O75767	183	197	31.85
694	2	DPB1*72:01	SVYDFFVWLHYYSVR	TRP2	O75767	180	194	28.48
695	3	DPB1*72:01	VYDFFVWLHYYSVRD	TRP2	O75767	181	195	22.94

696	4	DPB1*72:01	YDFFVWLHYYSVRDT	TRP2	O75767	182	196	23.67
697	5	DPB1*72:01	AFVDSIFEQWLRRHR	Tyrosinase	P14679	391	405	46.99
698	6	DPB1*72:01	FLLHHAFVDSIFEQW	Tyrosinase	P14679	386	400	47.66
699	7	DPB1*72:01	HAFVDSIFEQWLRRH	Tyrosinase	P14679	390	404	27.2
700	8	DPB1*72:01	HHAFVDSIFEQWLRR	Tyrosinase	P14679	389	403	18.99
701	9	DPB1*72:01	HNRESYMVPFIPLYR	Tyrosinase	P14679	420	434	43
702	10	DPB1*72:01	LHHAFVDSIFEQWLR	Tyrosinase	P14679	388	402	33.24
703	11	DPB1*72:01	NRESYMVPFIPLYRN	Tyrosinase	P14679	421	435	40.81
704	12	DPB1*72:01	ISIYNYFVWTHYYSV	TRP1	P17643	182	196	9.01
705	13	DPB1*72:01	IYNYFVWTHYYSVKK	TRP1	P17643	184	198	5.16
706	14	DPB1*72:01	NYFVWTHYYSVKKTF	TRP1	P17643	186	200	6.93
707	15	DPB1*72:01	SIYNYFVWTHYYSVK	TRP1	P17643	183	197	6.08
708	16	DPB1*72:01	YFVWTHYYSVKKTFL	TRP1	P17643	187	201	10.89
709	17	DPB1*72:01	YNYFVWTHYYSVKKT	TRP1	P17643	185	199	5.37
710	18	DPB1*72:01	TSCILESLFRAVITK	MAGE1	P43355	90	104	46.01
711	19	DPB1*72:01	LLMWITQCFLPVFLA	NY-ESO-1	P78358	158	172	49.67
712	20	DPB1*72:01	LMWITQCFLPVFLAQ	NY-ESO-1	P78358	159	173	37.97
713	21	DPB1*72:01	LSLLMWITQCFLPVF	NY-ESO-1	P78358	156	170	32.31
714	22	DPB1*72:01	MWITQCFLPVFLAQP	NY-ESO-1	P78358	160	174	28.46
715	23	DPB1*72:01	QLSLLMWITQCFLPV	NY-ESO-1	P78358	155	169	23.16
716	24	DPB1*72:01	RLLEFYLAMPFATPM	NY-ESO-1	P78358	86	100	47.3
717	25	DPB1*72:01	SRLLEFYLAMPFATP	NY-ESO-1	P78358	85	99	41.92
718	1	DPB1*81:01	CSVYDFFVWLHYYSV	TRP2	O75767	179	193	33
719	2	DPB1*81:01	DFFVWLHYYSVRDTL	TRP2	O75767	183	197	16.72
720	3	DPB1*81:01	FFVWLHYYSVRDTLL	TRP2	O75767	184	198	28.16
721	4	DPB1*81:01	SVYDFFVWLHYYSVR	TRP2	O75767	180	194	15.36
722	5	DPB1*81:01	VYDFFVWLHYYSVRD	TRP2	O75767	181	195	13.62
723	6	DPB1*81:01	YDFFVWLHYYSVRDT	TRP2	O75767	182	196	13.64
724	7	DPB1*81:01	ANDPIFLLHHAFVDS	Tyrosinase	P14679	381	395	43.78
725	8	DPB1*81:01	DPIFLLHHAFVDSIF	Tyrosinase	P14679	383	397	22.28
726	9	DPB1*81:01	EKDKFFAYLTLAKHT	Tyrosinase	P14679	130	144	49.43
727	10	DPB1*81:01	FLLHHAFVDSIFEQW	Tyrosinase	P14679	386	400	32.2
728	11	DPB1*81:01	HAFVDSIFEQWLRRH	Tyrosinase	P14679	390	404	48.15
729	12	DPB1*81:01	HHAFVDSIFEQWLRR	Tyrosinase	P14679	389	403	39.39
730	13	DPB1*81:01	HNRESYMVPFIPLYR	Tyrosinase	P14679	420	434	45.51
731	14	DPB1*81:01	IFLLHHAFVDSIFEQ	Tyrosinase	P14679	385	399	33.72
732	15	DPB1*81:01	KDKFFAYLTLAKHTI	Tyrosinase	P14679	131	145	46.71
733	16	DPB1*81:01	NDPIFLLHHAFVDSI	Tyrosinase	P14679	382	396	26.27
734	17	DPB1*81:01	NRESYMVPFIPLYRN	Tyrosinase	P14679	421	435	43.82

735	18	DPB1*81:01	PIFLLHHAFVDSIFE	Tyrosinase	P14679	384	398	20.9
736	19	DPB1*81:01	AFLTWHRYHLLRLEK	TRP1	P17643	219	233	15.2
737	20	DPB1*81:01	EGPAFLTWHRYHLLR	TRP1	P17643	216	230	16.72
738	21	DPB1*81:01	FLTWHRYHLLRLEKD	TRP1	P17643	220	234	23
739	22	DPB1*81:01	FVWTHYYSVKKTFLG	TRP1	P17643	188	202	30.77
740	23	DPB1*81:01	GPAFLTWHRYHLLRL	TRP1	P17643	217	231	13.09
741	24	DPB1*81:01	HEGPAFLTWHRYHLL	TRP1	P17643	215	229	26.31
742	25	DPB1*81:01	ISIYNYFVWTHYYSV	TRP1	P17643	182	196	9.41
743	26	DPB1*81:01	IYNYFVWTHYYSVKK	TRP1	P17643	184	198	4.57
744	27	DPB1*81:01	NYFVWTHYYSVKKTF	TRP1	P17643	186	200	5.5
745	28	DPB1*81:01	PAFLTWHRYHLLRLE	TRP1	P17643	218	232	14.24
746	29	DPB1*81:01	SIYNYFVWTHYYSVK	TRP1	P17643	183	197	5.66
747	30	DPB1*81:01	YFVWTHYYSVKKTFL	TRP1	P17643	187	201	7.2
748	31	DPB1*81:01	YNYFVWTHYYSVKKT	TRP1	P17643	185	199	4.64
749	32	DPB1*81:01	CILESLFRAVITKKV	MAGE1	P43355	92	106	39.93
750	33	DPB1*81:01	GPSTSCILESLFRAV	MAGE1	P43355	87	101	29.28
751	34	DPB1*81:01	PSTSCILESLFRAVI	MAGE1	P43355	88	102	25.38
752	35	DPB1*81:01	SCILESLFRAVITKK	MAGE1	P43355	91	105	21.1
753	36	DPB1*81:01	STSCILESLFRAVIT	MAGE1	P43355	89	103	23.29
754	37	DPB1*81:01	TSCILESLFRAVITK	MAGE1	P43355	90	104	14.8
755	38	DPB1*81:01	VADLVGFLLLKYRAR	MAGE1	P43355	106	120	48.01
756	39	DPB1*81:01	LLMWITQCFLPVFLA	NY-ESO-1	P78358	158	172	48.52
757	40	DPB1*81:01	LMWITQCFLPVFLAQ	NY-ESO-1	P78358	159	173	33.13
758	41	DPB1*81:01	LSLLMWITQCFLPVF	NY-ESO-1	P78358	156	170	36.97
759	42	DPB1*81:01	MWITQCFLPVFLAQP	NY-ESO-1	P78358	160	174	29.27
760	43	DPB1*81:01	QLSLLMWITQCFLPV	NY-ESO-1	P78358	155	169	34.36
761	44	DPB1*81:01	RLLEFYLAMPFATPM	NY-ESO-1	P78358	86	100	47.67
762	45	DPB1*81:01	SRLLEFYLAMPFATP	NY-ESO-1	P78358	85	99	47.03
763	1	DRB1*01:01	FVWLHYYSVRDTLLG	TRP2	O75767	185	199	46.81
764	2	DRB1*01:01	ITTQHWVGLLGPNGT	TRP2	O75767	158	172	28.99
765	3	DRB1*01:01	KVYYYRFVIGLRVWQ	TRP2	O75767	206	220	6.91
766	4	DRB1*01:01	LHYYSVRDTLLGGFF	TRP2	O75767	188	202	44.37
767	5	DRB1*01:01	LKVYYYRFVIGLRVW	TRP2	O75767	205	219	9.7
768	6	DRB1*01:01	QHWVGLLGPNGTQPQ	TRP2	O75767	161	175	31.19
769	7	DRB1*01:01	TQHWVGLLGPNGTQP	TRP2	O75767	160	174	23.93
770	8	DRB1*01:01	TTQHWVGLLGPNGTQ	TRP2	O75767	159	173	21.6
771	9	DRB1*01:01	VWLHYYSVRDTLLGG	TRP2	O75767	186	200	46.44
772	10	DRB1*01:01	VYYYRFVIGLRVWQW	TRP2	O75767	207	221	8.56
773	11	DRB1*01:01	WLHYYSVRDTLLGGF	TRP2	O75767	187	201	36.3

774	12	DRB1*01:01	WLKVYYYRFVIGLRV	TRP2	O75767	204	218	9.93
775	13	DRB1*01:01	YDFFVWLHYYSVRDT	TRP2	O75767	182	196	42.66
776	14	DRB1*01:01	YRFVIGLRVWQWEVI	TRP2	O75767	210	224	28.72
777	15	DRB1*01:01	YYRFVIGLRVWQWEV	TRP2	O75767	209	223	9.39
778	16	DRB1*01:01	YYYRFVIGLRVWQWE	TRP2	O75767	208	222	7.56
779	17	DRB1*01:01	AFVAMVTTACHEFFE	S100	P04271	74	88	31.14
780	18	DRB1*01:01	DFQEFMAFVAMVTTA	S100	P04271	68	82	39.72
781	19	DRB1*01:01	EFMAFVAMVTTACHE	S100	P04271	71	85	23.6
782	20	DRB1*01:01	FMAFVAMVTTACHEF	S100	P04271	72	86	26.13
783	21	DRB1*01:01	FQEFMAFVAMVTTAC	S100	P04271	69	83	45.9
784	22	DRB1*01:01	KKSELKELINNELSH	S100	P04271	27	41	48.94
785	23	DRB1*01:01	KSELKELINNELSHF	S100	P04271	28	42	33.6
786	24	DRB1*01:01	MAFVAMVTTACHEFF	S100	P04271	73	87	24.36
787	25	DRB1*01:01	QEFMAFVAMVTTACH	S100	P04271	70	84	27.21
788	26	DRB1*01:01	ALHIYMNGTMSQVQG	Tyrosinase	P14679	365	379	31.54
789	27	DRB1*01:01	ALLAGLVSLLCRHKR	Tyrosinase	P14679	490	504	38.99
790	28	DRB1*01:01	AMVGAVLTALLAGLV	Tyrosinase	P14679	482	496	24
791	29	DRB1*01:01	ASRIWSWLLGAAMVG	Tyrosinase	P14679	471	485	10.55
792	30	DRB1*01:01	AVLTALLAGLVSLLC	Tyrosinase	P14679	486	500	23.43
793	31	DRB1*01:01	AVLYCLLWSFQTSAG	Tyrosinase	P14679	4	18	34.95
794	32	DRB1*01:01	CQNILLSNAPLGPQF	Tyrosinase	P14679	55	69	24.12
795	33	DRB1*01:01	DIDFAHEAPAFLPWH	Tyrosinase	P14679	197	211	27.73
796	34	DRB1*01:01	DKFFAYLTLAKHTIS	Tyrosinase	P14679	132	146	18.08
797	35	DRB1*01:01	DPIFLLHHAFVDSIF	Tyrosinase	P14679	383	397	44.27
798	36	DRB1*01:01	DYIKSYLEQASRIWS	Tyrosinase	P14679	462	476	30.54
799	37	DRB1*01:01	EKDKFFAYLTLAKHT	Tyrosinase	P14679	130	144	25.93
800	38	DRB1*01:01	EQASRIWSWLLGAAM	Tyrosinase	P14679	469	483	25.47
801	39	DRB1*01:01	FAYLTLAKHTISSDY	Tyrosinase	P14679	135	149	36.68
802	40	DRB1*01:01	FFAYLTLAKHTISSD	Tyrosinase	P14679	134	148	27.66
803	41	DRB1*01:01	FVWMHYYVSMDALLG	Tyrosinase	P14679	176	190	11.07
804	42	DRB1*01:01	GAVLTALLAGLVSLL	Tyrosinase	P14679	485	499	14.36
805	43	DRB1*01:01	GSCQNILLSNAPLGP	Tyrosinase	P14679	53	67	40.72
806	44	DRB1*01:01	HNALHIYMNGTMSQV	Tyrosinase	P14679	363	377	35.6
807	45	DRB1*01:01	HYYVSMDALLGGSEI	Tyrosinase	P14679	180	194	7.66
808	46	DRB1*01:01	IDFAHEAPAFLPWHR	Tyrosinase	P14679	198	212	23.67
809	47	DRB1*01:01	IKSYLEQASRIWSWL	Tyrosinase	P14679	464	478	33.81
810	48	DRB1*01:01	IWSWLLGAAMVGAVL	Tyrosinase	P14679	474	488	26.95
811	49	DRB1*01:01	KDKFFAYLTLAKHTI	Tyrosinase	P14679	131	145	19.53
812	50	DRB1*01:01	KFFAYLTLAKHTISS	Tyrosinase	P14679	133	147	18
813	51	DRB1*01:01	KSYLEQASRIWSWLL	Tyrosinase	P14679	465	479	48.71

814	52	DRB1*01:01	LAVLYCLLWSFQTSA	Tyrosinase	P14679	3	17	46.23
815	53	DRB1*01:01	LHIYMNGTMSQVQGS	Tyrosinase	P14679	366	380	42.87
816	54	DRB1*01:01	LLWSFQTSAGHFPRA	Tyrosinase	P14679	9	23	45.88
817	55	DRB1*01:01	LTALLAGLVSLLCRH	Tyrosinase	P14679	488	502	30.25
818	56	DRB1*01:01	LYCLLWSFQTSAGHF	Tyrosinase	P14679	6	20	48.74
819	57	DRB1*01:01	MHYYVSMDALLGGSE	Tyrosinase	P14679	179	193	6.72
820	58	DRB1*01:01	MVGAVLTALLAGLVS	Tyrosinase	P14679	483	497	10.3
821	59	DRB1*01:01	NALHIYMNGTMSQVQ	Tyrosinase	P14679	364	378	29.38
822	60	DRB1*01:01	NILLSNAPLGPQFPF	Tyrosinase	P14679	57	71	49.45
823	61	DRB1*01:01	PEKDKFFAYLTLAKH	Tyrosinase	P14679	129	143	40.52
824	62	DRB1*01:01	PIFLLHHAFVDSIFE	Tyrosinase	P14679	384	398	47.49
825	63	DRB1*01:01	QASRIWSWLLGAAMV	Tyrosinase	P14679	470	484	13.94
826	64	DRB1*01:01	QDYIKSYLEQASRIW	Tyrosinase	P14679	461	475	42.27
827	65	DRB1*01:01	QNILLSNAPLGPQFP	Tyrosinase	P14679	56	70	24.12
828	66	DRB1*01:01	RDIDFAHEAPAFLPW	Tyrosinase	P14679	196	210	31.68
829	67	DRB1*01:01	RIWSWLLGAAMVGAV	Tyrosinase	P14679	473	487	11.66
830	68	DRB1*01:01	SCQNILLSNAPLGPQ	Tyrosinase	P14679	54	68	30.55
831	69	DRB1*01:01	SRIWSWLLGAAMVGA	Tyrosinase	P14679	472	486	8.69
832	70	DRB1*01:01	SWLLGAAMVGAVLTA	Tyrosinase	P14679	476	490	45.12
833	71	DRB1*01:01	TALLAGLVSLLCRHK	Tyrosinase	P14679	489	503	23.48
834	72	DRB1*01:01	VGAVLTALLAGLVSL	Tyrosinase	P14679	484	498	10.38
835	73	DRB1*01:01	VLTALLAGLVSLLCR	Tyrosinase	P14679	487	501	30.19
836	74	DRB1*01:01	VLYCLLWSFQTSAGH	Tyrosinase	P14679	5	19	40.64
837	75	DRB1*01:01	VWMHYYVSMDALLGG	Tyrosinase	P14679	177	191	6.98
838	76	DRB1*01:01	WMHYYVSMDALLGGS	Tyrosinase	P14679	178	192	6.14
839	77	DRB1*01:01	WRDIDFAHEAPAFLP	Tyrosinase	P14679	195	209	44.47
840	78	DRB1*01:01	WSWLLGAAMVGAVLT	Tyrosinase	P14679	475	489	44.08
841	79	DRB1*01:01	YIKSYLEQASRIWSW	Tyrosinase	P14679	463	477	27.34
842	80	DRB1*01:01	YVSMDALLGGSEIWR	Tyrosinase	P14679	182	196	28.17
843	81	DRB1*01:01	YYVSMDALLGGSEIW	Tyrosinase	P14679	181	195	9.22
844	82	DRB1*01:01	AHLFLNGTGGQTHLS	TRP1	P17643	380	394	44.3
845	83	DRB1*01:01	ALIFGTASYLIRARR	TRP1	P17643	491	505	17.3
846	84	DRB1*01:01	DPAVRSLHNLAHLFL	TRP1	P17643	370	384	44.8
847	85	DRB1*01:01	DPIFVLLHTFTDAVF	TRP1	P17643	397	411	30.92
848	86	DRB1*01:01	EKNHFVRALDMAKRT	TRP1	P17643	140	154	45.85
849	87	DRB1*01:01	EVDFSHEGPAFLTWH	TRP1	P17643	210	224	33.44
850	88	DRB1*01:01	FFPLLLFQQARAQFP	TRP1	P17643	13	27	24.79
851	89	DRB1*01:01	FPLLLFQQARAQFPR	TRP1	P17643	14	28	14.01
852	90	DRB1*01:01	FSLPYWNFATGKNVC	TRP1	P17643	244	258	38.68
853	91	DRB1*01:01	GEVDFSHEGPAFLTW	TRP1	P17643	209	223	42.17

854	92	DRB1*01:01	HPLFVIATRRSEEIL	TRP1	P17643	156	170	40.83
855	93	DRB1*01:01	IFFPLLLFQQARAQF	TRP1	P17643	12	26	37.15
856	94	DRB1*01:01	IYNYFVWTHYYSVKK	TRP1	P17643	184	198	40.34
857	95	DRB1*01:01	KNHFVRALDMAKRTT	TRP1	P17643	141	155	48.31
858	96	DRB1*01:01	LAHLFLNGTGGQTHL	TRP1	P17643	379	393	48.75
859	97	DRB1*01:01	LIFGTASYLIRARRS	TRP1	P17643	492	506	35.92
860	98	DRB1*01:01	LLFQQARAQFPRQCA	TRP1	P17643	17	31	27.7
861	99	DRB1*01:01	LLLFQQARAQFPRQC	TRP1	P17643	16	30	16.54
862	100	DRB1*01:01	LLVALIFGTASYLIR	TRP1	P17643	488	502	20.95
863	101	DRB1*01:01	LPYWNFATGKNVCDI	TRP1	P17643	246	260	24.53
864	102	DRB1*01:01	LVALIFGTASYLIRA	TRP1	P17643	489	503	15.16
865	103	DRB1*01:01	NDPIFVLLHTFTDAV	TRP1	P17643	396	410	33.42
866	104	DRB1*01:01	PAVRSLHNLAHLFLN	TRP1	P17643	371	385	44.3
867	105	DRB1*01:01	PIFVLLHTFTDAVFD	TRP1	P17643	398	412	38.84
868	106	DRB1*01:01	PLLLFQQARAQFPRQ	TRP1	P17643	15	29	12.28
869	107	DRB1*01:01	PNDPIFVLLHTFTDA	TRP1	P17643	395	409	45.75
870	108	DRB1*01:01	PYWNFATGKNVCDIC	TRP1	P17643	247	261	42.9
871	109	DRB1*01:01	RTTHPLFVIATRRSE	TRP1	P17643	153	167	44.82
872	110	DRB1*01:01	SLPYWNFATGKNVCD	TRP1	P17643	245	259	34.48
873	111	DRB1*01:01	THPLFVIATRRSEEI	TRP1	P17643	155	169	33.65
874	112	DRB1*01:01	TTHPLFVIATRRSEE	TRP1	P17643	154	168	42.82
875	113	DRB1*01:01	VALIFGTASYLIRAR	TRP1	P17643	490	504	12.15
876	114	DRB1*01:01	VDFSHEGPAFLTWHR	TRP1	P17643	211	225	39.26
877	115	DRB1*01:01	VWTHYYSVKKTFLGV	TRP1	P17643	189	203	46.38
878	116	DRB1*01:01	YFVWTHYYSVKKTFL	TRP1	P17643	187	201	48.52
879	117	DRB1*01:01	YNYFVWTHYYSVKKT	TRP1	P17643	185	199	40.38
880	118	DRB1*01:01	AEVSIVVLSGTTAAQ	PMEL17	P40967	403	417	9.68
881	119	DRB1*01:01	ALLAVGATKVPRNQD	PMEL17	P40967	17	31	12.83
882	120	DRB1*01:01	ANASFSIALNFPGSQ	PMEL17	P40967	80	94	11.1
883	121	DRB1*01:01	AQVVLQAAIPLTSCG	PMEL17	P40967	288	302	8.19
884	122	DRB1*01:01	ASFSIALNFPGSQKV	PMEL17	P40967	82	96	10.27
885	123	DRB1*01:01	AVIGALLAVGATKVP	PMEL17	P40967	13	27	7.61
886	124	DRB1*01:01	DGGNKHFLRNQPLTF	PMEL17	P40967	226	240	43.6
887	125	DRB1*01:01	DGPTLIGANASFSIA	PMEL17	P40967	73	87	46.2
888	126	DRB1*01:01	DSSGTLISRALVVTH	PMEL17	P40967	264	278	36.19
889	127	DRB1*01:01	EVSIVVLSGTTAAQV	PMEL17	P40967	404	418	9.77
890	128	DRB1*01:01	GALLAVGATKVPRNQ	PMEL17	P40967	16	30	8.84
891	129	DRB1*01:01	GANASFSIALNFPGS	PMEL17	P40967	79	93	14.29
892	130	DRB1*01:01	GDSSGTLISRALVVT	PMEL17	P40967	263	277	47.48
893	131	DRB1*01:01	GGNKHFLRNQPLTFA	PMEL17	P40967	227	241	23.3

894	132	DRB1*01:01	GNKHFLRNQPLTFAL	PMEL17	P40967	228	242	17.88
895	133	DRB1*01:01	GPTLIGANASFSIAL	PMEL17	P40967	74	88	30.57
896	134	DRB1*01:01	GPVSGLSIGTGRAML	PMEL17	P40967	165	179	49.97
897	135	DRB1*01:01	GPVTAQVVLQAAIPL	PMEL17	P40967	284	298	14.85
898	136	DRB1*01:01	GQYWQVLGGPVSGLS	PMEL17	P40967	157	171	7.51
899	137	DRB1*01:01	GSRSYVPLAHSSSAF	PMEL17	P40967	193	207	34.96
900	138	DRB1*01:01	GTLISRALVVTHTYL	PMEL17	P40967	267	281	37.9
901	139	DRB1*01:01	HFLRNQPLTFALQLH	PMEL17	P40967	231	245	39.84
902	140	DRB1*01:01	HLAVIGALLAVGATK	PMEL17	P40967	11	25	11.34
903	141	DRB1*01:01	IGALLAVGATKVPRN	PMEL17	P40967	15	29	7.11
904	142	DRB1*01:01	IGANASFSIALNFPG	PMEL17	P40967	78	92	39.8
905	143	DRB1*01:01	IVVLSGTTAAQVTTT	PMEL17	P40967	407	421	32.73
906	144	DRB1*01:01	KHFLRNQPLTFALQL	PMEL17	P40967	230	244	20.4
907	145	DRB1*01:01	KRCLLHLAVIGALLA	PMEL17	P40967	6	20	28.1
908	146	DRB1*01:01	KTWGQYWQVLGGPVS	PMEL17	P40967	154	168	15.76
909	147	DRB1*01:01	LAVIGALLAVGATKV	PMEL17	P40967	12	26	9.62
910	148	DRB1*01:01	LHLAVIGALLAVGAT	PMEL17	P40967	10	24	20.12
911	149	DRB1*01:01	LKRCLLHLAVIGALL	PMEL17	P40967	5	19	35.17
912	150	DRB1*01:01	LLHLAVIGALLAVGA	PMEL17	P40967	9	23	31.21
913	151	DRB1*01:01	LVLKRCLLHLAVIGA	PMEL17	P40967	3	17	48.29
914	152	DRB1*01:01	NASFSIALNFPGSQK	PMEL17	P40967	81	95	9.16
915	153	DRB1*01:01	NKHFLRNQPLTFALQ	PMEL17	P40967	229	243	17.34
916	154	DRB1*01:01	PAEVSIVVLSGTTAA	PMEL17	P40967	402	416	12.54
917	155	DRB1*01:01	PTLIGANASFSIALN	PMEL17	P40967	75	89	37.54
918	156	DRB1*01:01	PVSGLSIGTGRAMLG	PMEL17	P40967	166	180	36.9
919	157	DRB1*01:01	PVTAQVVLQAAIPLT	PMEL17	P40967	285	299	7.59
920	158	DRB1*01:01	QVVLQAAIPLTSCGS	PMEL17	P40967	289	303	11.98
921	159	DRB1*01:01	QYWQVLGGPVSGLSI	PMEL17	P40967	158	172	8.91
922	160	DRB1*01:01	RSYVPLAHSSSAFTI	PMEL17	P40967	195	209	27.89
923	161	DRB1*01:01	SFSIALNFPGSQKVL	PMEL17	P40967	83	97	16.75
924	162	DRB1*01:01	SGLSIGTGRAMLGTH	PMEL17	P40967	168	182	44.21
925	163	DRB1*01:01	SGTLISRALVVTHTY	PMEL17	P40967	266	280	33.49
926	164	DRB1*01:01	SIVVLSGTTAAQVTT	PMEL17	P40967	406	420	11.8
927	165	DRB1*01:01	SRSYVPLAHSSSAFT	PMEL17	P40967	194	208	31.86
928	166	DRB1*01:01	SSGTLISRALVVTHT	PMEL17	P40967	265	279	32.03
929	167	DRB1*01:01	SYVPLAHSSSAFTIT	PMEL17	P40967	196	210	29.38
930	168	DRB1*01:01	TAQVVLQAAIPLTSC	PMEL17	P40967	287	301	6.93
931	169	DRB1*01:01	TPAEVSIVVLSGTTA	PMEL17	P40967	401	415	28.59
932	170	DRB1*01:01	TWGQYWQVLGGPVSG	PMEL17	P40967	155	169	10.3
933	171	DRB1*01:01	VIGALLAVGATKVPR	PMEL17	P40967	14	28	6.6

934	172	DRB1*01:01	VLKRCLLHLAVIGAL	PMEL17	P40967	4	18	35.25
935	173	DRB1*01:01	VSGLSIGTGRAMLGT	PMEL17	P40967	167	181	31.26
936	174	DRB1*01:01	VSIVVLSGTTAAQVT	PMEL17	P40967	405	419	11.23
937	175	DRB1*01:01	VTAQVVLQAAIPLTS	PMEL17	P40967	286	300	6.37
938	176	DRB1*01:01	VVLQAAIPLTSCGSS	PMEL17	P40967	290	304	36.79
939	177	DRB1*01:01	WGQYWQVLGGPVSGL	PMEL17	P40967	156	170	7.45
940	178	DRB1*01:01	YVPLAHSSSAFTITD	PMEL17	P40967	197	211	37.85
941	179	DRB1*01:01	YWQVLGGPVSGLSIG	PMEL17	P40967	159	173	16.82
942	180	DRB1*01:01	ALGLVCVQAATSSSS	MAGE1	P43355	22	36	30.65
943	181	DRB1*01:01	ARVRFFFPSLREAAL	MAGE1	P43355	288	302	42.38
944	182	DRB1*01:01	ARYEFLWGPRALAET	MAGE1	P43355	260	274	8.13
945	183	DRB1*01:01	ASESLQLVFGIDVKE	MAGE1	P43355	147	161	31.38
946	184	DRB1*01:01	CILESLFRAVITKKV	MAGE1	P43355	92	106	13.91
947	185	DRB1*01:01	DNQIMPKTGFLIIVL	MAGE1	P43355	185	199	35.32
948	186	DRB1*01:01	DPARYEFLWGPRALA	MAGE1	P43355	258	272	7.86
949	187	DRB1*01:01	DSDPARYEFLWGPRA	MAGE1	P43355	256	270	20.69
950	188	DRB1*01:01	EALGLVCVQAATSSS	MAGE1	P43355	21	35	35.59
951	189	DRB1*01:01	ESLFRAVITKKVADL	MAGE1	P43355	95	109	6.03
952	190	DRB1*01:01	ESLQLVFGIDVKEAD	MAGE1	P43355	149	163	33.58
953	191	DRB1*01:01	EYVIKVSARVRFFFP	MAGE1	P43355	281	295	13.52
954	192	DRB1*01:01	FRAVITKKVADLVGF	MAGE1	P43355	98	112	39.93
955	193	DRB1*01:01	GDNQIMPKTGFLIIV	MAGE1	P43355	184	198	46.71
956	194	DRB1*01:01	GLVCVQAATSSSSPL	MAGE1	P43355	24	38	49.94
957	195	DRB1*01:01	IIVLVMIAMEGGHAP	MAGE1	P43355	196	210	15.82
958	196	DRB1*01:01	ILESLFRAVITKKVA	MAGE1	P43355	93	107	7.2
959	197	DRB1*01:01	ITKKVADLVGFLLLK	MAGE1	P43355	102	116	25.64
960	198	DRB1*01:01	IVLVMIAMEGGHAPE	MAGE1	P43355	197	211	14.68
961	199	DRB1*01:01	KASESLQLVFGIDVK	MAGE1	P43355	146	160	47.23
962	200	DRB1*01:01	KKVADLVGFLLLKYR	MAGE1	P43355	104	118	31.94
963	201	DRB1*01:01	KVLEYVIKVSARVRF	MAGE1	P43355	278	292	11.89
964	202	DRB1*01:01	LESLFRAVITKKVAD	MAGE1	P43355	94	108	7.2
965	203	DRB1*01:01	LEYVIKVSARVRFFF	MAGE1	P43355	280	294	11.33
966	204	DRB1*01:01	LFRAVITKKVADLVG	MAGE1	P43355	97	111	10.68
967	205	DRB1*01:01	LGLVCVQAATSSSSP	MAGE1	P43355	23	37	35.99
968	206	DRB1*01:01	LIIVLVMIAMEGGHA	MAGE1	P43355	195	209	36.83
969	207	DRB1*01:01	LVMIAMEGGHAPEEE	MAGE1	P43355	199	213	22.25
970	208	DRB1*01:01	NQIMPKTGFLIIVLV	MAGE1	P43355	186	200	38.15
971	209	DRB1*01:01	PARYEFLWGPRALAE	MAGE1	P43355	259	273	8.83
972	210	DRB1*01:01	QEALGLVCVQAATSS	MAGE1	P43355	20	34	46.63
973	211	DRB1*01:01	RFFFPSLREAALREE	MAGE1	P43355	291	305	47.18

974	212	DRB1*01:01	RVRFFFPSLREAALR	MAGE1	P43355	289	303	34.59
975	213	DRB1*01:01	RYEFLWGPRALAETS	MAGE1	P43355	261	275	11.06
976	214	DRB1*01:01	SDPARYEFLWGPRAL	MAGE1	P43355	257	271	9.72
977	215	DRB1*01:01	SESLQLVFGIDVKEA	MAGE1	P43355	148	162	22.5
978	216	DRB1*01:01	SLFRAVITKKVADLV	MAGE1	P43355	96	110	6.68
979	217	DRB1*01:01	TKKVADLVGFLLLKY	MAGE1	P43355	103	117	22.77
980	218	DRB1*01:01	VKVLEYVIKVSARVR	MAGE1	P43355	277	291	13.87
981	219	DRB1*01:01	VLEYVIKVSARVRFF	MAGE1	P43355	279	293	11.83
982	220	DRB1*01:01	VLVMIAMEGGHAPEE	MAGE1	P43355	198	212	16.47
983	221	DRB1*01:01	VMIAMEGGHAPEEEI	MAGE1	P43355	200	214	47.22
984	222	DRB1*01:01	VRFFFPSLREAALRE	MAGE1	P43355	290	304	39.1
985	223	DRB1*01:01	YEFLWGPRALAETSY	MAGE1	P43355	262	276	46.25
986	224	DRB1*01:01	YVIKVSARVRFFFPS	MAGE1	P43355	282	296	33.79
987	225	DRB1*01:01	YVKVLEYVIKVSARV	MAGE1	P43355	276	290	25.86
988	226	DRB1*01:01	AESLFREALSNKVDE	MAGE4	P43358	102	116	5.74
989	227	DRB1*01:01	AETSYVKVLEHVVRV	MAGE4	P43358	280	294	42.67
990	228	DRB1*01:01	ARYEFLWGPRALAET	MAGE4	P43358	268	282	8.13
991	229	DRB1*01:01	ASESLKMIFGIDVKE	MAGE4	P43358	155	169	10.72
992	230	DRB1*01:01	CFPVIFGKASESLKM	MAGE4	P43358	147	161	44.5
993	231	DRB1*01:01	DAESLFREALSNKVD	MAGE4	P43358	101	115	5.8
994	232	DRB1*01:01	ESLFREALSNKVDEL	MAGE4	P43358	103	117	4.49
995	233	DRB1*01:01	ESLKMIFGIDVKEVD	MAGE4	P43358	157	171	12
996	234	DRB1*01:01	ETSYVKVLEHVVRVN	MAGE4	P43358	281	295	41.6
997	235	DRB1*01:01	FPVIFGKASESLKMI	MAGE4	P43358	148	162	37.17
998	236	DRB1*01:01	FREALSNKVDELAHF	MAGE4	P43358	106	120	45.35
999	237	DRB1*01:01	GNNQIFPKTGLLIIV	MAGE4	P43358	192	206	44.3
1000	238	DRB1*01:01	GSNPARYEFLWGPRA	MAGE4	P43358	264	278	20.69
1001	239	DRB1*01:01	IKNYKRCFPVIFGKA	MAGE4	P43358	141	155	38.57
1002	240	DRB1*01:01	KASESLKMIFGIDVK	MAGE4	P43358	154	168	13.8
1003	241	DRB1*01:01	KNYKRCFPVIFGKAS	MAGE4	P43358	142	156	49.2
1004	242	DRB1*01:01	LFREALSNKVDELAH	MAGE4	P43358	105	119	7.18
1005	243	DRB1*01:01	NNQIFPKTGLLIIVL	MAGE4	P43358	193	207	37
1006	244	DRB1*01:01	NPARYEFLWGPRALA	MAGE4	P43358	266	280	7.86
1007	245	DRB1*01:01	NQIFPKTGLLIIVLG	MAGE4	P43358	194	208	48.87
1008	246	DRB1*01:01	PARYEFLWGPRALAE	MAGE4	P43358	267	281	8.83
1009	247	DRB1*01:01	PDAESLFREALSNKV	MAGE4	P43358	100	114	9.96
1010	248	DRB1*01:01	RYEFLWGPRALAETS	MAGE4	P43358	269	283	11.06
1011	249	DRB1*01:01	SESLKMIFGIDVKEV	MAGE4	P43358	156	170	9.37
1012	250	DRB1*01:01	SLFREALSNKVDELA	MAGE4	P43358	104	118	4.65
1013	251	DRB1*01:01	SLKMIFGIDVKEVDP	MAGE4	P43358	158	172	23.9

1014	252	DRB1*01:01	SNPARYEFLWGPRAL	MAGE4	P43358	265	279	9.72
1015	253	DRB1*01:01	SYVKVLEHVVRVNAR	MAGE4	P43358	283	297	48.26
1016	254	DRB1*01:01	TSYVKVLEHVVRVNA	MAGE4	P43358	282	296	39.69
1017	255	DRB1*01:01	VIKNYKRCFPVIFGK	MAGE4	P43358	140	154	49.6
1018	256	DRB1*01:01	YEFLWGPRALAETSY	MAGE4	P43358	270	284	46.25
1019	257	DRB1*01:01	ADHRQLQLSISSCLQ	NY-ESO-1	P78358	140	154	31.72
1020	258	DRB1*01:01	CFLPVFLAQPPSGQR	NY-ESO-1	P78358	165	179	41.92
1021	259	DRB1*01:01	DHRQLQLSISSCLQQ	NY-ESO-1	P78358	141	155	21.04
1022	260	DRB1*01:01	EFTVSGNILTIRLTA	NY-ESO-1	P78358	125	139	33.16
1023	261	DRB1*01:01	EFYLAMPFATPMEAE	NY-ESO-1	P78358	89	103	10.19
1024	262	DRB1*01:01	ESRLLEFYLAMPFAT	NY-ESO-1	P78358	84	98	25.98
1025	263	DRB1*01:01	FLPVFLAQPPSGQRR	NY-ESO-1	P78358	166	180	27.93
1026	264	DRB1*01:01	FYLAMPFATPMEAEL	NY-ESO-1	P78358	90	104	32.49
1027	265	DRB1*01:01	HRQLQLSISSCLQQL	NY-ESO-1	P78358	142	156	18.53
1028	266	DRB1*01:01	KEFTVSGNILTIRLT	NY-ESO-1	P78358	124	138	12.95
1029	267	DRB1*01:01	LEFYLAMPFATPMEA	NY-ESO-1	P78358	88	102	7.29
1030	268	DRB1*01:01	LKEFTVSGNILTIRL	NY-ESO-1	P78358	123	137	10.8
1031	269	DRB1*01:01	LLEFYLAMPFATPME	NY-ESO-1	P78358	87	101	7.2
1032	270	DRB1*01:01	LLKEFTVSGNILTIR	NY-ESO-1	P78358	122	136	11.69
1033	271	DRB1*01:01	RLLEFYLAMPFATPM	NY-ESO-1	P78358	86	100	8.84
1034	272	DRB1*01:01	RQLQLSISSCLQQLS	NY-ESO-1	P78358	143	157	29.3
1035	273	DRB1*01:01	SRLLEFYLAMPFATP	NY-ESO-1	P78358	85	99	9.86
1036	274	DRB1*01:01	VLLKEFTVSGNILTI	NY-ESO-1	P78358	121	135	23.95
1037	275	DRB1*01:01	AARAVFLALSAQLLQ	BAGE	Q13072	2	16	3.09
1038	276	DRB1*01:01	ALSAQLLQARLMKEE	BAGE	Q13072	9	23	18.8
1039	277	DRB1*01:01	ARAVFLALSAQLLQA	BAGE	Q13072	3	17	2.96
1040	278	DRB1*01:01	AVFLALSAQLLQARL	BAGE	Q13072	5	19	3
1041	279	DRB1*01:01	FLALSAQLLQARLMK	BAGE	Q13072	7	21	6.96
1042	280	DRB1*01:01	LALSAQLLQARLMKE	BAGE	Q13072	8	22	17.77
1043	281	DRB1*01:01	LSAQLLQARLMKEES	BAGE	Q13072	10	24	22.28
1044	282	DRB1*01:01	MAARAVFLALSAQLL	BAGE	Q13072	1	15	3.96
1045	283	DRB1*01:01	RAVFLALSAQLLQAR	BAGE	Q13072	4	18	2.61
1046	284	DRB1*01:01	SAQLLQARLMKEESP	BAGE	Q13072	11	25	38.35
1047	285	DRB1*01:01	VFLALSAQLLQARLM	BAGE	Q13072	6	20	3.41
1048	286	DRB1*01:01	AMTKLGFKATLPPFM	SSX2	Q16385	57	71	29.73
1049	287	DRB1*01:01	FGRLQGISPKIMPKK	SSX2	Q16385	101	115	33.11
1050	288	DRB1*01:01	GFKATLPPFMCNKRA	SSX2	Q16385	62	76	36.57
1051	289	DRB1*01:01	KLGFKATLPPFMCNK	SSX2	Q16385	60	74	9.87
1052	290	DRB1*01:01	LGFKATLPPFMCNKR	SSX2	Q16385	61	75	17.49
1053	291	DRB1*01:01	MTFGRLQGISPKIMP	SSX2	Q16385	99	113	17.02

1054	292	DRB1*01:01	MTKLGFKATLPPFMC	SSX2	Q16385	58	72	20.03
1055	293	DRB1*01:01	PQMTFGRLQGISPKI	SSX2	Q16385	97	111	14.4
1056	294	DRB1*01:01	QMTFGRLQGISPKIM	SSX2	Q16385	98	112	11.75
1057	295	DRB1*01:01	RPQMTFGRLQGISPK	SSX2	Q16385	96	110	23.77
1058	296	DRB1*01:01	TFGRLQGISPKIMPK	SSX2	Q16385	100	114	23.64
1059	297	DRB1*01:01	TKLGFKATLPPFMCN	SSX2	Q16385	59	73	13.86
1060	298	DRB1*01:01	APPAYEKLSAEQSPP	MELAN_A	Q16655	100	114	48.74
1061	299	DRB1*01:01	GYRALMDKSLHVGTQ	MELAN_A	Q16655	53	67	38.39
1062	300	DRB1*01:01	NGYRALMDKSLHVGT	MELAN_A	Q16655	52	66	26.36
1063	301	DRB1*01:01	RNGYRALMDKSLHVG	MELAN_A	Q16655	51	65	19.01
1064	302	DRB1*01:01	RRNGYRALMDKSLHV	MELAN_A	Q16655	50	64	18.26
1065	303	DRB1*01:01	RRRNGYRALMDKSLH	MELAN_A	Q16655	49	63	29.22
1066	1	DRB1*01:02	GAVLTALLAGLVSLL	Tyrosinase	P14679	485	499	48.43
1067	2	DRB1*01:02	MVGAVLTALLAGLVS	Tyrosinase	P14679	483	497	32.45
1068	3	DRB1*01:02	VGAVLTALLAGLVSL	Tyrosinase	P14679	484	498	35.82
1069	4	DRB1*01:02	ALIFGTASYLIRARR	TRP1	P17643	491	505	42.88
1070	5	DRB1*01:02	FPLLLFQQARAQFPR	TRP1	P17643	14	28	39.52
1071	6	DRB1*01:02	VALIFGTASYLIRAR	TRP1	P17643	490	504	34.03
1072	7	DRB1*01:02	AEVSIVVLSGTTAAQ	PMEL17	P40967	403	417	45.95
1073	8	DRB1*01:02	ALLAVGATKVPRNQD	PMEL17	P40967	17	31	33.5
1074	9	DRB1*01:02	AQVVLQAAIPLTSCG	PMEL17	P40967	288	302	18.38
1075	10	DRB1*01:02	AVIGALLAVGATKVP	PMEL17	P40967	13	27	18.78
1076	11	DRB1*01:02	EVSIVVLSGTTAAQV	PMEL17	P40967	404	418	44.64
1077	12	DRB1*01:02	GALLAVGATKVPRNQ	PMEL17	P40967	16	30	26.56
1078	13	DRB1*01:02	GPVTAQVVLQAAIPL	PMEL17	P40967	284	298	29.2
1079	14	DRB1*01:02	HLAVIGALLAVGATK	PMEL17	P40967	11	25	33.77
1080	15	DRB1*01:02	IGALLAVGATKVPRN	PMEL17	P40967	15	29	20.27
1081	16	DRB1*01:02	LAVIGALLAVGATKV	PMEL17	P40967	12	26	25.58
1082	17	DRB1*01:02	PVTAQVVLQAAIPLT	PMEL17	P40967	285	299	18.24
1083	18	DRB1*01:02	QVVLQAAIPLTSCGS	PMEL17	P40967	289	303	24.97
1084	19	DRB1*01:02	SIVVLSGTTAAQVTT	PMEL17	P40967	406	420	40.28
1085	20	DRB1*01:02	TAQVVLQAAIPLTSC	PMEL17	P40967	287	301	16.69
1086	21	DRB1*01:02	VIGALLAVGATKVPR	PMEL17	P40967	14	28	16.21
1087	22	DRB1*01:02	VSIVVLSGTTAAQVT	PMEL17	P40967	405	419	45.22
1088	23	DRB1*01:02	VTAQVVLQAAIPLTS	PMEL17	P40967	286	300	15.58
1089	24	DRB1*01:02	IIVLVMIAMEGGHAP	MAGE1	P43355	196	210	40
1090	25	DRB1*01:02	IVLVMIAMEGGHAPE	MAGE1	P43355	197	211	35.73
1091	26	DRB1*01:02	VLVMIAMEGGHAPEE	MAGE1	P43355	198	212	37.74
1092	27	DRB1*01:02	ASESLKMIFGIDVKE	MAGE4	P43358	155	169	28.69

1093	28	DRB1*01:02	ESLKMIFGIDVKEVD	MAGE4	P43358	157	171	28.36
1094	29	DRB1*01:02	KASESLKMIFGIDVK	MAGE4	P43358	154	168	33.5
1095	30	DRB1*01:02	SESLKMIFGIDVKEV	MAGE4	P43358	156	170	24.76
1096	31	DRB1*01:02	SLKMIFGIDVKEVDP	MAGE4	P43358	158	172	45.29
1097	32	DRB1*01:02	DHRQLQLSISSCLQQ	NY-ESO-1	P78358	141	155	47.4
1098	33	DRB1*01:02	HRQLQLSISSCLQQL	NY-ESO-1	P78358	142	156	37.14
1099	34	DRB1*01:02	AARAVFLALSAQLLQ	BAGE	Q13072	2	16	22.52
1100	35	DRB1*01:02	ARAVFLALSAQLLQA	BAGE	Q13072	3	17	19.72
1101	36	DRB1*01:02	AVFLALSAQLLQARL	BAGE	Q13072	5	19	20.12
1102	37	DRB1*01:02	FLALSAQLLQARLMK	BAGE	Q13072	7	21	27.62
1103	38	DRB1*01:02	LALSAQLLQARLMKE	BAGE	Q13072	8	22	47.21
1104	39	DRB1*01:02	MAARAVFLALSAQLL	BAGE	Q13072	1	15	31.67
1105	40	DRB1*01:02	RAVFLALSAQLLQAR	BAGE	Q13072	4	18	13.44
1106	41	DRB1*01:02	VFLALSAQLLQARLM	BAGE	Q13072	6	20	20.81
1107	1	DRB1*01:03	AARAVFLALSAQLLQ	BAGE	Q13072	2	16	36.37
1108	2	DRB1*01:03	ARAVFLALSAQLLQA	BAGE	Q13072	3	17	28.81
1109	3	DRB1*01:03	AVFLALSAQLLQARL	BAGE	Q13072	5	19	30.44
1110	4	DRB1*01:03	RAVFLALSAQLLQAR	BAGE	Q13072	4	18	16.69
1111	5	DRB1*01:03	VFLALSAQLLQARLM	BAGE	Q13072	6	20	38.58
1112	1	DRB1*01:11	KVYYYRFVIGLRVWQ	TRP2	O75767	206	220	9.65
1113	2	DRB1*01:11	LKVYYYRFVIGLRVW	TRP2	O75767	205	219	13.59
1114	3	DRB1*01:11	VYYYRFVIGLRVWQW	TRP2	O75767	207	221	11.19
1115	4	DRB1*01:11	WLKVYYYRFVIGLRV	TRP2	O75767	204	218	16.89
1116	5	DRB1*01:11	YYRFVIGLRVWQWEV	TRP2	O75767	209	223	16.56
1117	6	DRB1*01:11	YYYRFVIGLRVWQWE	TRP2	O75767	208	222	11.36
1118	7	DRB1*01:11	EFMAFVAMVTTACHE	S100	P04271	71	85	41.61
1119	8	DRB1*01:11	FMAFVAMVTTACHEF	S100	P04271	72	86	44.61
1120	9	DRB1*01:11	MAFVAMVTTACHEFF	S100	P04271	73	87	46.46
1121	10	DRB1*01:11	QEFMAFVAMVTTACH	S100	P04271	70	84	47.26
1122	11	DRB1*01:11	ASRIWSWLLGAAMVG	Tyrosinase	P14679	471	485	28.57
1123	12	DRB1*01:11	AVLTALLAGLVSLLC	Tyrosinase	P14679	486	500	47.46
1124	13	DRB1*01:11	FVWMHYYVSMDALLG	Tyrosinase	P14679	176	190	34.06
1125	14	DRB1*01:11	GAVLTALLAGLVSLL	Tyrosinase	P14679	485	499	31
1126	15	DRB1*01:11	HYYVSMDALLGGSEI	Tyrosinase	P14679	180	194	25.25
1127	16	DRB1*01:11	KFFAYLTLAKHTISS	Tyrosinase	P14679	133	147	49.54
1128	17	DRB1*01:11	MHYYVSMDALLGGSE	Tyrosinase	P14679	179	193	19.93
1129	18	DRB1*01:11	MVGAVLTALLAGLVS	Tyrosinase	P14679	483	497	33.51
1130	19	DRB1*01:11	QASRIWSWLLGAAMV	Tyrosinase	P14679	470	484	37.3

1131	20	DRB1*01:11	RIWSWLLGAAMVGAV	Tyrosinase	P14679	473	487	28.36
1132	21	DRB1*01:11	SRIWSWLLGAAMVGA	Tyrosinase	P14679	472	486	22.17
1133	22	DRB1*01:11	VGAVLTALLAGLVSL	Tyrosinase	P14679	484	498	28.25
1134	23	DRB1*01:11	VWMHYYVSMDALLGG	Tyrosinase	P14679	177	191	21.13
1135	24	DRB1*01:11	WMHYYVSMDALLGGS	Tyrosinase	P14679	178	192	17.88
1136	25	DRB1*01:11	YYVSMDALLGGSEIW	Tyrosinase	P14679	181	195	34.27
1137	26	DRB1*01:11	ALIFGTASYLIRARR	TRP1	P17643	491	505	38.45
1138	27	DRB1*01:11	FPLLLFQQARAQFPR	TRP1	P17643	14	28	39.18
1139	28	DRB1*01:11	LLVALIFGTASYLIR	TRP1	P17643	488	502	40.58
1140	29	DRB1*01:11	LVALIFGTASYLIRA	TRP1	P17643	489	503	28.86
1141	30	DRB1*01:11	PLLLFQQARAQFPRQ	TRP1	P17643	15	29	40.46
1142	31	DRB1*01:11	VALIFGTASYLIRAR	TRP1	P17643	490	504	25.64
1143	32	DRB1*01:11	AEVSIVVLSGTTAAQ	PMEL17	P40967	403	417	23.79
1144	33	DRB1*01:11	ALLAVGATKVPRNQD	PMEL17	P40967	17	31	41.69
1145	34	DRB1*01:11	ANASFSIALNFPGSQ	PMEL17	P40967	80	94	42.31
1146	35	DRB1*01:11	AQVVLQAAIPLTSCG	PMEL17	P40967	288	302	18.79
1147	36	DRB1*01:11	ASFSIALNFPGSQKV	PMEL17	P40967	82	96	34.74
1148	37	DRB1*01:11	AVIGALLAVGATKVP	PMEL17	P40967	13	27	22.56
1149	38	DRB1*01:11	EVSIVVLSGTTAAQV	PMEL17	P40967	404	418	19.29
1150	39	DRB1*01:11	GALLAVGATKVPRNQ	PMEL17	P40967	16	30	25.72
1151	40	DRB1*01:11	GGNKHFLRNQPLTFA	PMEL17	P40967	227	241	42.97
1152	41	DRB1*01:11	GNKHFLRNQPLTFAL	PMEL17	P40967	228	242	29.17
1153	42	DRB1*01:11	GPVTAQVVLQAAIPL	PMEL17	P40967	284	298	37.23
1154	43	DRB1*01:11	GQYWQVLGGPVSGLS	PMEL17	P40967	157	171	27.02
1155	44	DRB1*01:11	HLAVIGALLAVGATK	PMEL17	P40967	11	25	37.11
1156	45	DRB1*01:11	IGALLAVGATKVPRN	PMEL17	P40967	15	29	17.66
1157	46	DRB1*01:11	KHFLRNQPLTFALQL	PMEL17	P40967	230	244	30.72
1158	47	DRB1*01:11	LAVIGALLAVGATKV	PMEL17	P40967	12	26	25.64
1159	48	DRB1*01:11	NASFSIALNFPGSQK	PMEL17	P40967	81	95	30.61
1160	49	DRB1*01:11	NKHFLRNQPLTFALQ	PMEL17	P40967	229	243	27.95
1161	50	DRB1*01:11	PAEVSIVVLSGTTAA	PMEL17	P40967	402	416	32.83
1162	51	DRB1*01:11	PVTAQVVLQAAIPLT	PMEL17	P40967	285	299	16.88
1163	52	DRB1*01:11	QVVLQAAIPLTSCGS	PMEL17	P40967	289	303	28.61
1164	53	DRB1*01:11	QYWQVLGGPVSGLSI	PMEL17	P40967	158	172	38.16
1165	54	DRB1*01:11	SIVVLSGTTAAQVTT	PMEL17	P40967	406	420	30.24
1166	55	DRB1*01:11	TAQVVLQAAIPLTSC	PMEL17	P40967	287	301	14.97
1167	56	DRB1*01:11	TWGQYWQVLGGPVSG	PMEL17	P40967	155	169	42.16
1168	57	DRB1*01:11	VIGALLAVGATKVPR	PMEL17	P40967	14	28	17.12
1169	58	DRB1*01:11	VSIVVLSGTTAAQVT	PMEL17	P40967	405	419	21.57
1170	59	DRB1*01:11	VTAQVVLQAAIPLTS	PMEL17	P40967	286	300	13.13

1171	60	DRB1*01:11	WGQYWQVLGGPVSGL	PMEL17	P40967	156	170	27.26
1172	61	DRB1*01:11	ARYEFLWGPRALAET	MAGE1	P43355	260	274	28.01
1173	62	DRB1*01:11	CILESLFRAVITKKV	MAGE1	P43355	92	106	37.22
1174	63	DRB1*01:11	DPARYEFLWGPRALA	MAGE1	P43355	258	272	25.76
1175	64	DRB1*01:11	ESLFRAVITKKVADL	MAGE1	P43355	95	109	14.3
1176	65	DRB1*01:11	EYVIKVSARVRFFFP	MAGE1	P43355	281	295	25.2
1177	66	DRB1*01:11	ILESLFRAVITKKVA	MAGE1	P43355	93	107	17.96
1178	67	DRB1*01:11	KVLEYVIKVSARVRF	MAGE1	P43355	278	292	20.44
1179	68	DRB1*01:11	LESLFRAVITKKVAD	MAGE1	P43355	94	108	18.57
1180	69	DRB1*01:11	LEYVIKVSARVRFFF	MAGE1	P43355	280	294	19.27
1181	70	DRB1*01:11	LFRAVITKKVADLVG	MAGE1	P43355	97	111	28.66
1182	71	DRB1*01:11	PARYEFLWGPRALAE	MAGE1	P43355	259	273	27.46
1183	72	DRB1*01:11	RYEFLWGPRALAETS	MAGE1	P43355	261	275	42.97
1184	73	DRB1*01:11	SDPARYEFLWGPRAL	MAGE1	P43355	257	271	38.87
1185	74	DRB1*01:11	SLFRAVITKKVADLV	MAGE1	P43355	96	110	16.39
1186	75	DRB1*01:11	VKVLEYVIKVSARVR	MAGE1	P43355	277	291	26.54
1187	76	DRB1*01:11	VLEYVIKVSARVRFF	MAGE1	P43355	279	293	18.7
1188	77	DRB1*01:11	AESLFREALSNKVDE	MAGE4	P43358	102	116	15.33
1189	78	DRB1*01:11	ARYEFLWGPRALAET	MAGE4	P43358	268	282	28.01
1190	79	DRB1*01:11	ASESLKMIFGIDVKE	MAGE4	P43358	155	169	32.49
1191	80	DRB1*01:11	DAESLFREALSNKVD	MAGE4	P43358	101	115	16.22
1192	81	DRB1*01:11	ESLFREALSNKVDEL	MAGE4	P43358	103	117	10.86
1193	82	DRB1*01:11	ESLKMIFGIDVKEVD	MAGE4	P43358	157	171	37.13
1194	83	DRB1*01:11	KASESLKMIFGIDVK	MAGE4	P43358	154	168	43.85
1195	84	DRB1*01:11	LFREALSNKVDELAH	MAGE4	P43358	105	119	19.34
1196	85	DRB1*01:11	NPARYEFLWGPRALA	MAGE4	P43358	266	280	25.76
1197	86	DRB1*01:11	PARYEFLWGPRALAE	MAGE4	P43358	267	281	27.46
1198	87	DRB1*01:11	PDAESLFREALSNKV	MAGE4	P43358	100	114	25.78
1199	88	DRB1*01:11	RYEFLWGPRALAETS	MAGE4	P43358	269	283	42.97
1200	89	DRB1*01:11	SESLKMIFGIDVKEV	MAGE4	P43358	156	170	26.37
1201	90	DRB1*01:11	SLFREALSNKVDELA	MAGE4	P43358	104	118	11.61
1202	91	DRB1*01:11	SNPARYEFLWGPRAL	MAGE4	P43358	265	279	38.87
1203	92	DRB1*01:11	DHRQLQLSISSCLQQ	NY-ESO-1	P78358	141	155	48.76
1204	93	DRB1*01:11	EFYLAMPFATPMEAE	NY-ESO-1	P78358	89	103	27.66
1205	94	DRB1*01:11	HRQLQLSISSCLQQL	NY-ESO-1	P78358	142	156	40.53
1206	95	DRB1*01:11	KEFTVSGNILTIRLT	NY-ESO-1	P78358	124	138	28.32
1207	96	DRB1*01:11	LEFYLAMPFATPMEA	NY-ESO-1	P78358	88	102	16.85
1208	97	DRB1*01:11	LKEFTVSGNILTIRL	NY-ESO-1	P78358	123	137	21.27
1209	98	DRB1*01:11	LLEFYLAMPFATPME	NY-ESO-1	P78358	87	101	14.66
1210	99	DRB1*01:11	LLKEFTVSGNILTIR	NY-ESO-1	P78358	122	136	24.56

1211	100	DRB1*01:11	RLLEFYLAMPFATPM	NY-ESO-1	P78358	86	100	17.69
1212	101	DRB1*01:11	SRLLEFYLAMPFATP	NY-ESO-1	P78358	85	99	22.39
1213	102	DRB1*01:11	VLLKEFTVSGNILTI	NY-ESO-1	P78358	121	135	48
1214	103	DRB1*01:11	AARAVFLALSAQLLQ	BAGE	Q13072	2	16	4.86
1215	104	DRB1*01:11	ARAVFLALSAQLLQA	BAGE	Q13072	3	17	4.46
1216	105	DRB1*01:11	AVFLALSAQLLQARL	BAGE	Q13072	5	19	4.64
1217	106	DRB1*01:11	FLALSAQLLQARLMK	BAGE	Q13072	7	21	13.82
1218	107	DRB1*01:11	MAARAVFLALSAQLL	BAGE	Q13072	1	15	6.8
1219	108	DRB1*01:11	RAVFLALSAQLLQAR	BAGE	Q13072	4	18	3.85
1220	109	DRB1*01:11	VFLALSAQLLQARLM	BAGE	Q13072	6	20	5.78
1221	110	DRB1*01:11	KLGFKATLPPFMCNK	SSX2	Q16385	60	74	25.07
1222	111	DRB1*01:11	LGFKATLPPFMCNKR	SSX2	Q16385	61	75	49.21
1223	112	DRB1*01:11	TKLGFKATLPPFMCN	SSX2	Q16385	59	73	37.73
1224	1	DRB1*01:18	DFFVWLHYYSVRDTL	TRP2	O75767	183	197	33.13
1225	2	DRB1*01:18	EREQFLGALDLAKKR	TRP2	O75767	137	151	46.86
1226	3	DRB1*01:18	FFVWLHYYSVRDTLL	TRP2	O75767	184	198	35.64
1227	4	DRB1*01:18	FVWLHYYSVRDTLLG	TRP2	O75767	185	199	40.12
1228	5	DRB1*01:18	ITTQHWVGLLGPNGT	TRP2	O75767	158	172	38.08
1229	6	DRB1*01:18	KVYYYRFVIGLRVWQ	TRP2	O75767	206	220	5.31
1230	7	DRB1*01:18	LHYYSVRDTLLGGFF	TRP2	O75767	188	202	42.86
1231	8	DRB1*01:18	LKVYYYRFVIGLRVW	TRP2	O75767	205	219	6.9
1232	9	DRB1*01:18	QEREQFLGALDLAKK	TRP2	O75767	136	150	49
1233	10	DRB1*01:18	QHWVGLLGPNGTQPQ	TRP2	O75767	161	175	40.04
1234	11	DRB1*01:18	REQFLGALDLAKKRV	TRP2	O75767	138	152	44.68
1235	12	DRB1*01:18	SVYDFFVWLHYYSVR	TRP2	O75767	180	194	39.51
1236	13	DRB1*01:18	TQHWVGLLGPNGTQP	TRP2	O75767	160	174	31.38
1237	14	DRB1*01:18	TTQHWVGLLGPNGTQ	TRP2	O75767	159	173	27.9
1238	15	DRB1*01:18	VWLHYYSVRDTLLGG	TRP2	O75767	186	200	43.62
1239	16	DRB1*01:18	VYDFFVWLHYYSVRD	TRP2	O75767	181	195	32.05
1240	17	DRB1*01:18	VYYYRFVIGLRVWQW	TRP2	O75767	207	221	6.24
1241	18	DRB1*01:18	WLHYYSVRDTLLGGF	TRP2	O75767	187	201	35.91
1242	19	DRB1*01:18	WLKVYYYRFVIGLRV	TRP2	O75767	204	218	7.51
1243	20	DRB1*01:18	YDFFVWLHYYSVRDT	TRP2	O75767	182	196	27.59
1244	21	DRB1*01:18	YRFVIGLRVWQWEVI	TRP2	O75767	210	224	21.41
1245	22	DRB1*01:18	YYRFVIGLRVWQWEV	TRP2	O75767	209	223	7.14
1246	23	DRB1*01:18	YYYRFVIGLRVWQWE	TRP2	O75767	208	222	5.78
1247	24	DRB1*01:18	AFVAMVTTACHEFFE	S100	P04271	74	88	30.76
1248	25	DRB1*01:18	CDFQEFMAFVAMVTT	S100	P04271	67	81	30.35
1249	26	DRB1*01:18	DFQEFMAFVAMVTTA	S100	P04271	68	82	22.4

1250	27	DRB1*01:18	ECDFQEFMAFVAMVT	S100	P04271	66	80	32.52
1251	28	DRB1*01:18	EFMAFVAMVTTACHE	S100	P04271	71	85	20.92
1252	29	DRB1*01:18	ELKELINNELSHFLE	S100	P04271	30	44	49.73
1253	30	DRB1*01:18	FMAFVAMVTTACHEF	S100	P04271	72	86	22.4
1254	31	DRB1*01:18	FQEFMAFVAMVTTAC	S100	P04271	69	83	31.01
1255	32	DRB1*01:18	KKSELKELINNELSH	S100	P04271	27	41	40.51
1256	33	DRB1*01:18	KSELKELINNELSHF	S100	P04271	28	42	27.66
1257	34	DRB1*01:18	MAFVAMVTTACHEFF	S100	P04271	73	87	22.14
1258	35	DRB1*01:18	QEFMAFVAMVTTACH	S100	P04271	70	84	21.8
1259	36	DRB1*01:18	SELKELINNELSHFL	S100	P04271	29	43	40.69
1260	37	DRB1*01:18	ALHIYMNGTMSQVQG	Tyrosinase	P14679	365	379	31.88
1261	38	DRB1*01:18	ALLAGLVSLLCRHKR	Tyrosinase	P14679	490	504	28.62
1262	39	DRB1*01:18	AMVGAVLTALLAGLV	Tyrosinase	P14679	482	496	19.87
1263	40	DRB1*01:18	ANDPIFLLHHAFVDS	Tyrosinase	P14679	381	395	38.25
1264	41	DRB1*01:18	ASRIWSWLLGAAMVG	Tyrosinase	P14679	471	485	9.89
1265	42	DRB1*01:18	AVLTALLAGLVSLLC	Tyrosinase	P14679	486	500	18.24
1266	43	DRB1*01:18	AVLYCLLWSFQTSAG	Tyrosinase	P14679	4	18	30.66
1267	44	DRB1*01:18	CQNILLSNAPLGPQF	Tyrosinase	P14679	55	69	21.97
1268	45	DRB1*01:18	DIDFAHEAPAFLPWH	Tyrosinase	P14679	197	211	32.52
1269	46	DRB1*01:18	DKFFAYLTLAKHTIS	Tyrosinase	P14679	132	146	14.72
1270	47	DRB1*01:18	DPIFLLHHAFVDSIF	Tyrosinase	P14679	383	397	27.37
1271	48	DRB1*01:18	DYIKSYLEQASRIWS	Tyrosinase	P14679	462	476	26.19
1272	49	DRB1*01:18	EKDKFFAYLTLAKHT	Tyrosinase	P14679	130	144	20.08
1273	50	DRB1*01:18	EQASRIWSWLLGAAM	Tyrosinase	P14679	469	483	21.71
1274	51	DRB1*01:18	FAYLTLAKHTISSDY	Tyrosinase	P14679	135	149	32.03
1275	52	DRB1*01:18	FFAYLTLAKHTISSD	Tyrosinase	P14679	134	148	21
1276	53	DRB1*01:18	FVWMHYYVSMDALLG	Tyrosinase	P14679	176	190	11.92
1277	54	DRB1*01:18	GAVLTALLAGLVSLL	Tyrosinase	P14679	485	499	12.16
1278	55	DRB1*01:18	GSCQNILLSNAPLGP	Tyrosinase	P14679	53	67	34.26
1279	56	DRB1*01:18	HNALHIYMNGTMSQV	Tyrosinase	P14679	363	377	33.33
1280	57	DRB1*01:18	HYYVSMDALLGGSEI	Tyrosinase	P14679	180	194	8.3
1281	58	DRB1*01:18	IDFAHEAPAFLPWHR	Tyrosinase	P14679	198	212	29.19
1282	59	DRB1*01:18	IFLLHHAFVDSIFEQ	Tyrosinase	P14679	385	399	49.67
1283	60	DRB1*01:18	IKSYLEQASRIWSWL	Tyrosinase	P14679	464	478	28.18
1284	61	DRB1*01:18	IWSWLLGAAMVGAVL	Tyrosinase	P14679	474	488	20.43
1285	62	DRB1*01:18	KDKFFAYLTLAKHTI	Tyrosinase	P14679	131	145	15.02
1286	63	DRB1*01:18	KFFAYLTLAKHTISS	Tyrosinase	P14679	133	147	14.59
1287	64	DRB1*01:18	KSYLEQASRIWSWLL	Tyrosinase	P14679	465	479	39.46
1288	65	DRB1*01:18	LAVLYCLLWSFQTSA	Tyrosinase	P14679	3	17	37.78
1289	66	DRB1*01:18	LHIYMNGTMSQVQGS	Tyrosinase	P14679	366	380	43.9

1290	67	DRB1*01:18	LLAGLVSLLCRHKRK	Tyrosinase	P14679	491	505	45.21
1291	68	DRB1*01:18	LLWSFQTSAGHFPRA	Tyrosinase	P14679	9	23	43.93
1292	69	DRB1*01:18	LTALLAGLVSLLCRH	Tyrosinase	P14679	488	502	24.03
1293	70	DRB1*01:18	LYCLLWSFQTSAGHF	Tyrosinase	P14679	6	20	41.66
1294	71	DRB1*01:18	MHYYVSMDALLGGSE	Tyrosinase	P14679	179	193	7.11
1295	72	DRB1*01:18	MVGAVLTALLAGLVS	Tyrosinase	P14679	483	497	10.38
1296	73	DRB1*01:18	NALHIYMNGTMSQVQ	Tyrosinase	P14679	364	378	28.56
1297	74	DRB1*01:18	NDPIFLLHHAFVDSI	Tyrosinase	P14679	382	396	30.61
1298	75	DRB1*01:18	NILLSNAPLGPQFPF	Tyrosinase	P14679	57	71	45.9
1299	76	DRB1*01:18	PEKDKFFAYLTLAKH	Tyrosinase	P14679	129	143	29.2
1300	77	DRB1*01:18	PIFLLHHAFVDSIFE	Tyrosinase	P14679	384	398	32.2
1301	78	DRB1*01:18	QASRIWSWLLGAAMV	Tyrosinase	P14679	470	484	12.37
1302	79	DRB1*01:18	QDYIKSYLEQASRIW	Tyrosinase	P14679	461	475	34.94
1303	80	DRB1*01:18	QNILLSNAPLGPQFP	Tyrosinase	P14679	56	70	22.56
1304	81	DRB1*01:18	RDIDFAHEAPAFLPW	Tyrosinase	P14679	196	210	36.71
1305	82	DRB1*01:18	RIWSWLLGAAMVGAV	Tyrosinase	P14679	473	487	10.27
1306	83	DRB1*01:18	SCQNILLSNAPLGPQ	Tyrosinase	P14679	54	68	25.9
1307	84	DRB1*01:18	SRIWSWLLGAAMVGA	Tyrosinase	P14679	472	486	7.97
1308	85	DRB1*01:18	SWLLGAAMVGAVLTA	Tyrosinase	P14679	476	490	37.57
1309	86	DRB1*01:18	TALLAGLVSLLCRHK	Tyrosinase	P14679	489	503	20.32
1310	87	DRB1*01:18	VGAVLTALLAGLVSL	Tyrosinase	P14679	484	498	9.96
1311	88	DRB1*01:18	VLTALLAGLVSLLCR	Tyrosinase	P14679	487	501	22.16
1312	89	DRB1*01:18	VLYCLLWSFQTSAGH	Tyrosinase	P14679	5	19	36.26
1313	90	DRB1*01:18	VWMHYYVSMDALLGG	Tyrosinase	P14679	177	191	7.47
1314	91	DRB1*01:18	WMHYYVSMDALLGGS	Tyrosinase	P14679	178	192	6.47
1315	92	DRB1*01:18	WSWLLGAAMVGAVLT	Tyrosinase	P14679	475	489	33.19
1316	93	DRB1*01:18	YIKSYLEQASRIWSW	Tyrosinase	P14679	463	477	23.97
1317	94	DRB1*01:18	YVSMDALLGGSEIWR	Tyrosinase	P14679	182	196	32.68
1318	95	DRB1*01:18	YYVSMDALLGGSEIW	Tyrosinase	P14679	181	195	10.14
1319	96	DRB1*01:18	ALIFGTASYLIRARR	TRP1	P17643	491	505	13.82
1320	97	DRB1*01:18	AVRSLHNLAHLFLNG	TRP1	P17643	372	386	36.47
1321	98	DRB1*01:18	CIFFPLLLFQQARAQ	TRP1	P17643	11	25	38.57
1322	99	DRB1*01:18	DPAVRSLHNLAHLFL	TRP1	P17643	370	384	31.18
1323	100	DRB1*01:18	DPIFVLLHTFTDAVF	TRP1	P17643	397	411	23.02
1324	101	DRB1*01:18	EEKNHFVRALDMAKR	TRP1	P17643	139	153	39.69
1325	102	DRB1*01:18	EKNHFVRALDMAKRT	TRP1	P17643	140	154	33.42
1326	103	DRB1*01:18	EVDFSHEGPAFLTWH	TRP1	P17643	210	224	45.08
1327	104	DRB1*01:18	FFPLLLFQQARAQFP	TRP1	P17643	13	27	18.79
1328	105	DRB1*01:18	FPLLLFQQARAQFPR	TRP1	P17643	14	28	13.19
1329	106	DRB1*01:18	FSLPYWNFATGKNVC	TRP1	P17643	244	258	40.22

1330	107	DRB1*01:18	FVWTHYYSVKKTFLG	TRP1	P17643	188	202	45.58
1331	108	DRB1*01:18	HPLFVIATRRSEEIL	TRP1	P17643	156	170	26.05
1332	109	DRB1*01:18	IFFPLLLFQQARAQF	TRP1	P17643	12	26	23.88
1333	110	DRB1*01:18	IYNYFVWTHYYSVKK	TRP1	P17643	184	198	31.29
1334	111	DRB1*01:18	KNHFVRALDMAKRTT	TRP1	P17643	141	155	33.4
1335	112	DRB1*01:18	KRTTHPLFVIATRRS	TRP1	P17643	152	166	43.94
1336	113	DRB1*01:18	LIFGTASYLIRARRS	TRP1	P17643	492	506	27.38
1337	114	DRB1*01:18	LLFQQARAQFPRQCA	TRP1	P17643	17	31	32.83
1338	115	DRB1*01:18	LLLFQQARAQFPRQC	TRP1	P17643	16	30	17.26
1339	116	DRB1*01:18	LLLVALIFGTASYLI	TRP1	P17643	487	501	42.66
1340	117	DRB1*01:18	LLVALIFGTASYLIR	TRP1	P17643	488	502	16.91
1341	118	DRB1*01:18	LPYWNFATGKNVCDI	TRP1	P17643	246	260	26.73
1342	119	DRB1*01:18	LVALIFGTASYLIRA	TRP1	P17643	489	503	12.34
1343	120	DRB1*01:18	NDPIFVLLHTFTDAV	TRP1	P17643	396	410	26.39
1344	121	DRB1*01:18	NYFVWTHYYSVKKTF	TRP1	P17643	186	200	40.54
1345	122	DRB1*01:18	PAVRSLHNLAHLFLN	TRP1	P17643	371	385	29.2
1346	123	DRB1*01:18	PIFVLLHTFTDAVFD	TRP1	P17643	398	412	27
1347	124	DRB1*01:18	PLFVIATRRSEEILG	TRP1	P17643	157	171	38.62
1348	125	DRB1*01:18	PLLLFQQARAQFPRQ	TRP1	P17643	15	29	12.64
1349	126	DRB1*01:18	PNDPIFVLLHTFTDA	TRP1	P17643	395	409	37.91
1350	127	DRB1*01:18	PYWNFATGKNVCDIC	TRP1	P17643	247	261	44.04
1351	128	DRB1*01:18	RTTHPLFVIATRRSE	TRP1	P17643	153	167	28.14
1352	129	DRB1*01:18	SIYNYFVWTHYYSVK	TRP1	P17643	183	197	44.66
1353	130	DRB1*01:18	SLPYWNFATGKNVCD	TRP1	P17643	245	259	37.59
1354	131	DRB1*01:18	THPLFVIATRRSEEI	TRP1	P17643	155	169	22.89
1355	132	DRB1*01:18	THYYSVKKTFLGVGQ	TRP1	P17643	191	205	44.24
1356	133	DRB1*01:18	TTHPLFVIATRRSEE	TRP1	P17643	154	168	28.19
1357	134	DRB1*01:18	VALIFGTASYLIRAR	TRP1	P17643	490	504	10.35
1358	135	DRB1*01:18	VRSLHNLAHLFLNGT	TRP1	P17643	373	387	48.47
1359	136	DRB1*01:18	VWTHYYSVKKTFLGV	TRP1	P17643	189	203	36.71
1360	137	DRB1*01:18	WTHYYSVKKTFLGVG	TRP1	P17643	190	204	38.39
1361	138	DRB1*01:18	YDPAVRSLHNLAHLF	TRP1	P17643	369	383	46.22
1362	139	DRB1*01:18	YFVWTHYYSVKKTFL	TRP1	P17643	187	201	38.5
1363	140	DRB1*01:18	YNYFVWTHYYSVKKT	TRP1	P17643	185	199	30.68
1364	141	DRB1*01:18	AEVSIVVLSGTTAAQ	PMEL17	P40967	403	417	9.34
1365	142	DRB1*01:18	ALLAVGATKVPRNQD	PMEL17	P40967	17	31	13.2
1366	143	DRB1*01:18	ANASFSIALNFPGSQ	PMEL17	P40967	80	94	11.53
1367	144	DRB1*01:18	AQVVLQAAIPLTSCG	PMEL17	P40967	288	302	8.22
1368	145	DRB1*01:18	ASFSIALNFPGSQKV	PMEL17	P40967	82	96	10.24
1369	146	DRB1*01:18	AVIGALLAVGATKVP	PMEL17	P40967	13	27	7.63

1370	147	DRB1*01:18	CLLHLAVIGALLAVG	PMEL17	P40967	8	22	44.89
1371	148	DRB1*01:18	DGGNKHFLRNQPLTF	PMEL17	P40967	226	240	35.59
1372	149	DRB1*01:18	DQVPFSVSVSQLRAL	PMEL17	P40967	211	225	45.05
1373	150	DRB1*01:18	DSSGTLISRALVVTH	PMEL17	P40967	264	278	33.84
1374	151	DRB1*01:18	EVSIVVLSGTTAAQV	PMEL17	P40967	404	418	8.87
1375	152	DRB1*01:18	GALLAVGATKVPRNQ	PMEL17	P40967	16	30	9.32
1376	153	DRB1*01:18	GANASFSIALNFPGS	PMEL17	P40967	79	93	14.74
1377	154	DRB1*01:18	GDSSGTLISRALVVT	PMEL17	P40967	263	277	45.27
1378	155	DRB1*01:18	GGNKHFLRNQPLTFA	PMEL17	P40967	227	241	18.46
1379	156	DRB1*01:18	GNKHFLRNQPLTFAL	PMEL17	P40967	228	242	13.77
1380	157	DRB1*01:18	GPTLIGANASFSIAL	PMEL17	P40967	74	88	33.82
1381	158	DRB1*01:18	GPVTAQVVLQAAIPL	PMEL17	P40967	284	298	14.56
1382	159	DRB1*01:18	GQYWQVLGGPVSGLS	PMEL17	P40967	157	171	7.92
1383	160	DRB1*01:18	GSRSYVPLAHSSSAF	PMEL17	P40967	193	207	31.55
1384	161	DRB1*01:18	GTLISRALVVTHTYL	PMEL17	P40967	267	281	33.36
1385	162	DRB1*01:18	HFLRNQPLTFALQLH	PMEL17	P40967	231	245	33.26
1386	163	DRB1*01:18	HLAVIGALLAVGATK	PMEL17	P40967	11	25	10.72
1387	164	DRB1*01:18	IGALLAVGATKVPRN	PMEL17	P40967	15	29	7.12
1388	165	DRB1*01:18	IGANASFSIALNFPG	PMEL17	P40967	78	92	36.37
1389	166	DRB1*01:18	IVVLSGTTAAQVTTT	PMEL17	P40967	407	421	27.09
1390	167	DRB1*01:18	KHFLRNQPLTFALQL	PMEL17	P40967	230	244	17.05
1391	168	DRB1*01:18	KRCLLHLAVIGALLA	PMEL17	P40967	6	20	18.73
1392	169	DRB1*01:18	KTWGQYWQVLGGPVS	PMEL17	P40967	154	168	17.05
1393	170	DRB1*01:18	LAVIGALLAVGATKV	PMEL17	P40967	12	26	9.12
1394	171	DRB1*01:18	LHLAVIGALLAVGAT	PMEL17	P40967	10	24	16.46
1395	172	DRB1*01:18	LKRCLLHLAVIGALL	PMEL17	P40967	5	19	22.72
1396	173	DRB1*01:18	LLHLAVIGALLAVGA	PMEL17	P40967	9	23	23.03
1397	174	DRB1*01:18	LMAVVLASLIYRRRL	PMEL17	P40967	606	620	37.92
1398	175	DRB1*01:18	LVLKRCLLHLAVIGA	PMEL17	P40967	3	17	33.54
1399	176	DRB1*01:18	MAVVLASLIYRRRLM	PMEL17	P40967	607	621	47.77
1400	177	DRB1*01:18	NASFSIALNFPGSQK	PMEL17	P40967	81	95	9.44
1401	178	DRB1*01:18	NKHFLRNQPLTFALQ	PMEL17	P40967	229	243	13.46
1402	179	DRB1*01:18	PAEVSIVVLSGTTAA	PMEL17	P40967	402	416	12.12
1403	180	DRB1*01:18	PTLIGANASFSIALN	PMEL17	P40967	75	89	39.94
1404	181	DRB1*01:18	PVSGLSIGTGRAMLG	PMEL17	P40967	166	180	40.44
1405	182	DRB1*01:18	PVTAQVVLQAAIPLT	PMEL17	P40967	285	299	7.91
1406	183	DRB1*01:18	QVVLQAAIPLTSCGS	PMEL17	P40967	289	303	11.49
1407	184	DRB1*01:18	QYWQVLGGPVSGLSI	PMEL17	P40967	158	172	9.71
1408	185	DRB1*01:18	RCLLHLAVIGALLAV	PMEL17	P40967	7	21	30.98
1409	186	DRB1*01:18	RSYVPLAHSSSAFTI	PMEL17	P40967	195	209	25.5

1410	187	DRB1*01:18	SFSIALNFPGSQKVL	PMEL17	P40967	83	97	15.18
1411	188	DRB1*01:18	SGTLISRALVVTHTY	PMEL17	P40967	266	280	29
1412	189	DRB1*01:18	SIVVLSGTTAAQVTT	PMEL17	P40967	406	420	11.14
1413	190	DRB1*01:18	SRSYVPLAHSSSAFT	PMEL17	P40967	194	208	29.32
1414	191	DRB1*01:18	SSGTLISRALVVTHT	PMEL17	P40967	265	279	28.55
1415	192	DRB1*01:18	SVSVSQLRALDGGNK	PMEL17	P40967	216	230	49.57
1416	193	DRB1*01:18	SYVPLAHSSSAFTIT	PMEL17	P40967	196	210	30.77
1417	194	DRB1*01:18	TAQVVLQAAIPLTSC	PMEL17	P40967	287	301	7.07
1418	195	DRB1*01:18	TPAEVSIVVLSGTTA	PMEL17	P40967	401	415	28.06
1419	196	DRB1*01:18	TWGQYWQVLGGPVSG	PMEL17	P40967	155	169	11.07
1420	197	DRB1*01:18	VIGALLAVGATKVPR	PMEL17	P40967	14	28	6.76
1421	198	DRB1*01:18	VLKRCLLHLAVIGAL	PMEL17	P40967	4	18	23.95
1422	199	DRB1*01:18	VLMAVVLASLIYRRR	PMEL17	P40967	605	619	38.07
1423	200	DRB1*01:18	VSGLSIGTGRAMLGT	PMEL17	P40967	167	181	34.18
1424	201	DRB1*01:18	VSIVVLSGTTAAQVT	PMEL17	P40967	405	419	10.11
1425	202	DRB1*01:18	VTAQVVLQAAIPLTS	PMEL17	P40967	286	300	6.37
1426	203	DRB1*01:18	VVLQAAIPLTSCGSS	PMEL17	P40967	290	304	34.52
1427	204	DRB1*01:18	WGQYWQVLGGPVSGL	PMEL17	P40967	156	170	7.87
1428	205	DRB1*01:18	YVPLAHSSSAFTITD	PMEL17	P40967	197	211	45.1
1429	206	DRB1*01:18	YWQVLGGPVSGLSIG	PMEL17	P40967	159	173	20.35
1430	207	DRB1*01:18	ADLVGFLLLKYRARE	MAGE1	P43355	107	121	44.56
1431	208	DRB1*01:18	AETSYVKVLEYVIKV	MAGE1	P43355	272	286	31.69
1432	209	DRB1*01:18	ALGLVCVQAATSSSS	MAGE1	P43355	22	36	29.18
1433	210	DRB1*01:18	ARVRFFFPSLREAAL	MAGE1	P43355	288	302	38.57
1434	211	DRB1*01:18	ARYEFLWGPRALAET	MAGE1	P43355	260	274	8.09
1435	212	DRB1*01:18	ASESLQLVFGIDVKE	MAGE1	P43355	147	161	23.94
1436	213	DRB1*01:18	AVITKKVADLVGFLL	MAGE1	P43355	100	114	44.03
1437	214	DRB1*01:18	CILESLFRAVITKKV	MAGE1	P43355	92	106	11.32
1438	215	DRB1*01:18	DLVGFLLLKYRAREP	MAGE1	P43355	108	122	46.26
1439	216	DRB1*01:18	DNQIMPKTGFLIIVL	MAGE1	P43355	185	199	25.03
1440	217	DRB1*01:18	DPARYEFLWGPRALA	MAGE1	P43355	258	272	7.84
1441	218	DRB1*01:18	DSDPARYEFLWGPRA	MAGE1	P43355	256	270	21.7
1442	219	DRB1*01:18	EALGLVCVQAATSSS	MAGE1	P43355	21	35	33.61
1443	220	DRB1*01:18	ESLFRAVITKKVADL	MAGE1	P43355	95	109	5.72
1444	221	DRB1*01:18	ESLQLVFGIDVKEAD	MAGE1	P43355	149	163	24.5
1445	222	DRB1*01:18	ETSYVKVLEYVIKVS	MAGE1	P43355	273	287	27.29
1446	223	DRB1*01:18	EYVIKVSARVRFFFP	MAGE1	P43355	281	295	9.76
1447	224	DRB1*01:18	FRAVITKKVADLVGF	MAGE1	P43355	98	112	26.71
1448	225	DRB1*01:18	GDNQIMPKTGFLIIV	MAGE1	P43355	184	198	32
1449	226	DRB1*01:18	IIVLVMIAMEGGHAP	MAGE1	P43355	196	210	17.62

1450	227	DRB1*01:18	ILESLFRAVITKKVA	MAGE1	P43355	93	107	6.54
1451	228	DRB1*01:18	ITKKVADLVGFLLLK	MAGE1	P43355	102	116	17.93
1452	229	DRB1*01:18	IVLVMIAMEGGHAPE	MAGE1	P43355	197	211	15.98
1453	230	DRB1*01:18	KASESLQLVFGIDVK	MAGE1	P43355	146	160	31.52
1454	231	DRB1*01:18	KKVADLVGFLLLKYR	MAGE1	P43355	104	118	21.17
1455	232	DRB1*01:18	KVADLVGFLLLKYRA	MAGE1	P43355	105	119	49.58
1456	233	DRB1*01:18	KVLEYVIKVSARVRF	MAGE1	P43355	278	292	8.36
1457	234	DRB1*01:18	LAETSYVKVLEYVIK	MAGE1	P43355	271	285	43.02
1458	235	DRB1*01:18	LESLFRAVITKKVAD	MAGE1	P43355	94	108	6.74
1459	236	DRB1*01:18	LEYVIKVSARVRFFF	MAGE1	P43355	280	294	7.83
1460	237	DRB1*01:18	LFRAVITKKVADLVG	MAGE1	P43355	97	111	8.78
1461	238	DRB1*01:18	LGLVCVQAATSSSSP	MAGE1	P43355	23	37	34.58
1462	239	DRB1*01:18	LIIVLVMIAMEGGHA	MAGE1	P43355	195	209	35.89
1463	240	DRB1*01:18	LVGFLLLKYRAREPV	MAGE1	P43355	109	123	40.27
1464	241	DRB1*01:18	LVMIAMEGGHAPEEE	MAGE1	P43355	199	213	24.4
1465	242	DRB1*01:18	NQIMPKTGFLIIVLV	MAGE1	P43355	186	200	26.87
1466	243	DRB1*01:18	PARYEFLWGPRALAE	MAGE1	P43355	259	273	8.62
1467	244	DRB1*01:18	QEALGLVCVQAATSS	MAGE1	P43355	20	34	42.54
1468	245	DRB1*01:18	QIMPKTGFLIIVLVM	MAGE1	P43355	187	201	46.66
1469	246	DRB1*01:18	RFFFPSLREAALREE	MAGE1	P43355	291	305	43.93
1470	247	DRB1*01:18	RVRFFFPSLREAALR	MAGE1	P43355	289	303	30.75
1471	248	DRB1*01:18	RYEFLWGPRALAETS	MAGE1	P43355	261	275	10.59
1472	249	DRB1*01:18	SARVRFFFPSLREAA	MAGE1	P43355	287	301	49.9
1473	250	DRB1*01:18	SDPARYEFLWGPRAL	MAGE1	P43355	257	271	10.11
1474	251	DRB1*01:18	SESLQLVFGIDVKEA	MAGE1	P43355	148	162	18.19
1475	252	DRB1*01:18	SLFRAVITKKVADLV	MAGE1	P43355	96	110	6.08
1476	253	DRB1*01:18	SLQLVFGIDVKEADP	MAGE1	P43355	150	164	47.49
1477	254	DRB1*01:18	SYVKVLEYVIKVSAR	MAGE1	P43355	275	289	38.68
1478	255	DRB1*01:18	TKKVADLVGFLLLKY	MAGE1	P43355	103	117	15.79
1479	256	DRB1*01:18	TSYVKVLEYVIKVSA	MAGE1	P43355	274	288	29.34
1480	257	DRB1*01:18	VGFLLLKYRAREPVT	MAGE1	P43355	110	124	49.14
1481	258	DRB1*01:18	VITKKVADLVGFLLL	MAGE1	P43355	101	115	33.71
1482	259	DRB1*01:18	VKVLEYVIKVSARVR	MAGE1	P43355	277	291	9.9
1483	260	DRB1*01:18	VLEYVIKVSARVRFF	MAGE1	P43355	279	293	7.97
1484	261	DRB1*01:18	VLVMIAMEGGHAPEE	MAGE1	P43355	198	212	17.79
1485	262	DRB1*01:18	VRFFFPSLREAALRE	MAGE1	P43355	290	304	35.95
1486	263	DRB1*01:18	YEFLWGPRALAETSY	MAGE1	P43355	262	276	39.13
1487	264	DRB1*01:18	YVIKVSARVRFFFPS	MAGE1	P43355	282	296	23.34
1488	265	DRB1*01:18	YVKVLEYVIKVSARV	MAGE1	P43355	276	290	17.68
1489	266	DRB1*01:18	AESLFREALSNKVDE	MAGE4	P43358	102	116	6.51

1490	267	DRB1*01:18	AETSYVKVLEHVVRV	MAGE4	P43358	280	294	27.9
1491	268	DRB1*01:18	AHFLLRKYRAKELVT	MAGE4	P43358	118	132	44.84
1492	269	DRB1*01:18	ARYEFLWGPRALAET	MAGE4	P43358	268	282	8.09
1493	270	DRB1*01:18	ASESLKMIFGIDVKE	MAGE4	P43358	155	169	10.97
1494	271	DRB1*01:18	CFPVIFGKASESLKM	MAGE4	P43358	147	161	40.2
1495	272	DRB1*01:18	DAESLFREALSNKVD	MAGE4	P43358	101	115	6.63
1496	273	DRB1*01:18	ESLFREALSNKVDEL	MAGE4	P43358	103	117	4.95
1497	274	DRB1*01:18	ESLKMIFGIDVKEVD	MAGE4	P43358	157	171	11.17
1498	275	DRB1*01:18	ETSYVKVLEHVVRVN	MAGE4	P43358	281	295	27.89
1499	276	DRB1*01:18	FLLRKYRAKELVTKA	MAGE4	P43358	120	134	49.35
1500	277	DRB1*01:18	FPVIFGKASESLKMI	MAGE4	P43358	148	162	32.5
1501	278	DRB1*01:18	FREALSNKVDELAHF	MAGE4	P43358	106	120	48.59
1502	279	DRB1*01:18	GKASESLKMIFGIDV	MAGE4	P43358	153	167	38.59
1503	280	DRB1*01:18	GNNQIFPKTGLLIIV	MAGE4	P43358	192	206	29.03
1504	281	DRB1*01:18	GSNPARYEFLWGPRA	MAGE4	P43358	264	278	21.7
1505	282	DRB1*01:18	HFLLRKYRAKELVTK	MAGE4	P43358	119	133	39.26
1506	283	DRB1*01:18	IKNYKRCFPVIFGKA	MAGE4	P43358	141	155	31.43
1507	284	DRB1*01:18	KASESLKMIFGIDVK	MAGE4	P43358	154	168	13.6
1508	285	DRB1*01:18	KNYKRCFPVIFGKAS	MAGE4	P43358	142	156	40.27
1509	286	DRB1*01:18	LAETSYVKVLEHVVR	MAGE4	P43358	279	293	44.62
1510	287	DRB1*01:18	LAHFLLRKYRAKELV	MAGE4	P43358	117	131	47.55
1511	288	DRB1*01:18	LEHVVRVNARVRIAY	MAGE4	P43358	288	302	42.69
1512	289	DRB1*01:18	LFREALSNKVDELAH	MAGE4	P43358	105	119	7.98
1513	290	DRB1*01:18	LGNNQIFPKTGLLII	MAGE4	P43358	191	205	45.79
1514	291	DRB1*01:18	NARVRIAYPSLREAA	MAGE4	P43358	295	309	49.78
1515	292	DRB1*01:18	NNQIFPKTGLLIIVL	MAGE4	P43358	193	207	24.71
1516	293	DRB1*01:18	NPARYEFLWGPRALA	MAGE4	P43358	266	280	7.84
1517	294	DRB1*01:18	NQIFPKTGLLIIVLG	MAGE4	P43358	194	208	32.27
1518	295	DRB1*01:18	PARYEFLWGPRALAE	MAGE4	P43358	267	281	8.62
1519	296	DRB1*01:18	PDAESLFREALSNKV	MAGE4	P43358	100	114	11.27
1520	297	DRB1*01:18	RYEFLWGPRALAETS	MAGE4	P43358	269	283	10.59
1521	298	DRB1*01:18	SESLKMIFGIDVKEV	MAGE4	P43358	156	170	9.27
1522	299	DRB1*01:18	SLFREALSNKVDELA	MAGE4	P43358	104	118	5.1
1523	300	DRB1*01:18	SLKMIFGIDVKEVDP	MAGE4	P43358	158	172	18.44
1524	301	DRB1*01:18	SNPARYEFLWGPRAL	MAGE4	P43358	265	279	10.11
1525	302	DRB1*01:18	SYVKVLEHVVRVNAR	MAGE4	P43358	283	297	31.03
1526	303	DRB1*01:18	TSYVKVLEHVVRVNA	MAGE4	P43358	282	296	25.53
1527	304	DRB1*01:18	VIKNYKRCFPVIFGK	MAGE4	P43358	140	154	40.29
1528	305	DRB1*01:18	VLEHVVRVNARVRIA	MAGE4	P43358	287	301	47.38
1529	306	DRB1*01:18	YEFLWGPRALAETSY	MAGE4	P43358	270	284	39.13

1530	307	DRB1*01:18	ADHRQLQLSISSCLQ	NY-ESO-1	P78358	140	154	28.35
1531	308	DRB1*01:18	DHRQLQLSISSCLQQ	NY-ESO-1	P78358	141	155	19.82
1532	309	DRB1*01:18	EFTVSGNILTIRLTA	NY-ESO-1	P78358	125	139	28.77
1533	310	DRB1*01:18	EFYLAMPFATPMEAE	NY-ESO-1	P78358	89	103	10.4
1534	311	DRB1*01:18	ESRLLEFYLAMPFAT	NY-ESO-1	P78358	84	98	20.78
1535	312	DRB1*01:18	FLPVFLAQPPSGQRR	NY-ESO-1	P78358	166	180	34.4
1536	313	DRB1*01:18	FYLAMPFATPMEAEL	NY-ESO-1	P78358	90	104	29.67
1537	314	DRB1*01:18	GVLLKEFTVSGNILT	NY-ESO-1	P78358	120	134	41.65
1538	315	DRB1*01:18	HRQLQLSISSCLQQL	NY-ESO-1	P78358	142	156	17.06
1539	316	DRB1*01:18	KEFTVSGNILTIRLT	NY-ESO-1	P78358	124	138	13.9
1540	317	DRB1*01:18	LEFYLAMPFATPMEA	NY-ESO-1	P78358	88	102	7.42
1541	318	DRB1*01:18	LKEFTVSGNILTIRL	NY-ESO-1	P78358	123	137	11.6
1542	319	DRB1*01:18	LLEFYLAMPFATPME	NY-ESO-1	P78358	87	101	7
1543	320	DRB1*01:18	LLKEFTVSGNILTIR	NY-ESO-1	P78358	122	136	12.82
1544	321	DRB1*01:18	RLLEFYLAMPFATPM	NY-ESO-1	P78358	86	100	8.28
1545	322	DRB1*01:18	RQLQLSISSCLQQLS	NY-ESO-1	P78358	143	157	24.72
1546	323	DRB1*01:18	SRLLEFYLAMPFATP	NY-ESO-1	P78358	85	99	9.5
1547	324	DRB1*01:18	VLLKEFTVSGNILTI	NY-ESO-1	P78358	121	135	22.94
1548	325	DRB1*01:18	AARAVFLALSAQLLQ	BAGE	Q13072	2	16	2.88
1549	326	DRB1*01:18	ALSAQLLQARLMKEE	BAGE	Q13072	9	23	16.88
1550	327	DRB1*01:18	ARAVFLALSAQLLQA	BAGE	Q13072	3	17	2.73
1551	328	DRB1*01:18	AVFLALSAQLLQARL	BAGE	Q13072	5	19	2.74
1552	329	DRB1*01:18	FLALSAQLLQARLMK	BAGE	Q13072	7	21	5.62
1553	330	DRB1*01:18	LALSAQLLQARLMKE	BAGE	Q13072	8	22	15.91
1554	331	DRB1*01:18	LSAQLLQARLMKEES	BAGE	Q13072	10	24	19.99
1555	332	DRB1*01:18	MAARAVFLALSAQLL	BAGE	Q13072	1	15	3.77
1556	333	DRB1*01:18	RAVFLALSAQLLQAR	BAGE	Q13072	4	18	2.47
1557	334	DRB1*01:18	SAQLLQARLMKEESP	BAGE	Q13072	11	25	34.26
1558	335	DRB1*01:18	VFLALSAQLLQARLM	BAGE	Q13072	6	20	3.05
1559	336	DRB1*01:18	AMTKLGFKATLPPFM	SSX2	Q16385	57	71	29.04
1560	337	DRB1*01:18	FGRLQGISPKIMPKK	SSX2	Q16385	101	115	27.98
1561	338	DRB1*01:18	GFKATLPPFMCNKRA	SSX2	Q16385	62	76	33.45
1562	339	DRB1*01:18	KLGFKATLPPFMCNK	SSX2	Q16385	60	74	10.68
1563	340	DRB1*01:18	LGFKATLPPFMCNKR	SSX2	Q16385	61	75	17.23
1564	341	DRB1*01:18	MTFGRLQGISPKIMP	SSX2	Q16385	99	113	16.45
1565	342	DRB1*01:18	MTKLGFKATLPPFMC	SSX2	Q16385	58	72	20.35
1566	343	DRB1*01:18	PQMTFGRLQGISPKI	SSX2	Q16385	97	111	16.33
1567	344	DRB1*01:18	QMTFGRLQGISPKIM	SSX2	Q16385	98	112	12.47
1568	345	DRB1*01:18	RPQMTFGRLQGISPK	SSX2	Q16385	96	110	27.06
1569	346	DRB1*01:18	TFGRLQGISPKIMPK	SSX2	Q16385	100	114	21.99

1570	347	DRB1*01:18	TKLGFKATLPPFMCN	SSX2	Q16385	59	73	14.66
1571	348	DRB1*01:18	GYRALMDKSLHVGTQ	MELAN_A	Q16655	53	67	31.82
1572	349	DRB1*01:18	NGYRALMDKSLHVGT	MELAN_A	Q16655	52	66	23.03
1573	350	DRB1*01:18	RNGYRALMDKSLHVG	MELAN_A	Q16655	51	65	17.23
1574	351	DRB1*01:18	RRNGYRALMDKSLHV	MELAN_A	Q16655	50	64	16.46
1575	352	DRB1*01:18	RRRNGYRALMDKSLH	MELAN_A	Q16655	49	63	26.09
1576	1	DRB1*01:20	EREQFLGALDLAKKR	TRP2	O75767	137	151	49.71
1577	2	DRB1*01:20	KVYYYRFVIGLRVWQ	TRP2	O75767	206	220	25.87
1578	3	DRB1*01:20	LKVYYYRFVIGLRVW	TRP2	O75767	205	219	41.36
1579	4	DRB1*01:20	VYYYRFVIGLRVWQW	TRP2	O75767	207	221	29.94
1580	5	DRB1*01:20	WLKVYYYRFVIGLRV	TRP2	O75767	204	218	40.86
1581	6	DRB1*01:20	YYRFVIGLRVWQWEV	TRP2	O75767	209	223	31.31
1582	7	DRB1*01:20	YYYRFVIGLRVWQWE	TRP2	O75767	208	222	24.68
1583	8	DRB1*01:20	EKAMVALIDVFHQYS	S100	P04271	3	17	41.09
1584	9	DRB1*01:20	ELEKAMVALIDVFHQ	S100	P04271	1	15	48.56
1585	10	DRB1*01:20	ELKELINNELSHFLE	S100	P04271	30	44	40.73
1586	11	DRB1*01:20	FMAFVAMVTTACHEF	S100	P04271	72	86	49.78
1587	12	DRB1*01:20	KAMVALIDVFHQYSG	S100	P04271	4	18	47.4
1588	13	DRB1*01:20	KKSELKELINNELSH	S100	P04271	27	41	33.28
1589	14	DRB1*01:20	KSELKELINNELSHF	S100	P04271	28	42	23.27
1590	15	DRB1*01:20	LEKAMVALIDVFHQY	S100	P04271	2	16	48.67
1591	16	DRB1*01:20	LKKSELKELINNELS	S100	P04271	26	40	45.07
1592	17	DRB1*01:20	MAFVAMVTTACHEFF	S100	P04271	73	87	44.69
1593	18	DRB1*01:20	SELKELINNELSHFL	S100	P04271	29	43	32.83
1594	19	DRB1*01:20	ALHIYMNGTMSQVQG	Tyrosinase	P14679	365	379	40.68
1595	20	DRB1*01:20	ALLAGLVSLLCRHKR	Tyrosinase	P14679	490	504	23.97
1596	21	DRB1*01:20	AMVGAVLTALLAGLV	Tyrosinase	P14679	482	496	21.4
1597	22	DRB1*01:20	ASRIWSWLLGAAMVG	Tyrosinase	P14679	471	485	33.16
1598	23	DRB1*01:20	AVLTALLAGLVSLLC	Tyrosinase	P14679	486	500	25.23
1599	24	DRB1*01:20	CQNILLSNAPLGPQF	Tyrosinase	P14679	55	69	17.94
1600	25	DRB1*01:20	DKFFAYLTLAKHTIS	Tyrosinase	P14679	132	146	27.98
1601	26	DRB1*01:20	DYIKSYLEQASRIWS	Tyrosinase	P14679	462	476	44.8
1602	27	DRB1*01:20	EKDKFFAYLTLAKHT	Tyrosinase	P14679	130	144	37.89
1603	28	DRB1*01:20	FAYLTLAKHTISSDY	Tyrosinase	P14679	135	149	34.87
1604	29	DRB1*01:20	FFAYLTLAKHTISSD	Tyrosinase	P14679	134	148	33.11
1605	30	DRB1*01:20	FVWMHYYVSMDALLG	Tyrosinase	P14679	176	190	47.64
1606	31	DRB1*01:20	GAVLTALLAGLVSLL	Tyrosinase	P14679	485	499	16.49
1607	32	DRB1*01:20	GSCQNILLSNAPLGP	Tyrosinase	P14679	53	67	32.2
1608	33	DRB1*01:20	HNALHIYMNGTMSQV	Tyrosinase	P14679	363	377	39.75

1609	34	DRB1*01:20	HYYVSMDALLGGSEI	Tyrosinase	P14679	180	194	27.74
1610	35	DRB1*01:20	KDKFFAYLTLAKHTI	Tyrosinase	P14679	131	145	29.67
1611	36	DRB1*01:20	KFFAYLTLAKHTISS	Tyrosinase	P14679	133	147	26.01
1612	37	DRB1*01:20	LLAGLVSLLCRHKRK	Tyrosinase	P14679	491	505	35.8
1613	38	DRB1*01:20	LTALLAGLVSLLCRH	Tyrosinase	P14679	488	502	23.56
1614	39	DRB1*01:20	MHYYVSMDALLGGSE	Tyrosinase	P14679	179	193	24.19
1615	40	DRB1*01:20	MVGAVLTALLAGLVS	Tyrosinase	P14679	483	497	10.27
1616	41	DRB1*01:20	NALHIYMNGTMSQVQ	Tyrosinase	P14679	364	378	35.55
1617	42	DRB1*01:20	NILLSNAPLGPQFPF	Tyrosinase	P14679	57	71	37.47
1618	43	DRB1*01:20	NRESYMVPFIPLYRN	Tyrosinase	P14679	421	435	48.82
1619	44	DRB1*01:20	QASRIWSWLLGAAMV	Tyrosinase	P14679	470	484	40.45
1620	45	DRB1*01:20	QNILLSNAPLGPQFP	Tyrosinase	P14679	56	70	18.33
1621	46	DRB1*01:20	RESYMVPFIPLYRNG	Tyrosinase	P14679	422	436	48.43
1622	47	DRB1*01:20	RGSCQNILLSNAPLG	Tyrosinase	P14679	52	66	49.63
1623	48	DRB1*01:20	RIWSWLLGAAMVGAV	Tyrosinase	P14679	473	487	34.41
1624	49	DRB1*01:20	SCQNILLSNAPLGPQ	Tyrosinase	P14679	54	68	23.93
1625	50	DRB1*01:20	SRIWSWLLGAAMVGA	Tyrosinase	P14679	472	486	26.76
1626	51	DRB1*01:20	TALLAGLVSLLCRHK	Tyrosinase	P14679	489	503	17.15
1627	52	DRB1*01:20	VGAVLTALLAGLVSL	Tyrosinase	P14679	484	498	11.67
1628	53	DRB1*01:20	VLTALLAGLVSLLCR	Tyrosinase	P14679	487	501	27.38
1629	54	DRB1*01:20	VWMHYYVSMDALLGG	Tyrosinase	P14679	177	191	30.03
1630	55	DRB1*01:20	WMHYYVSMDALLGGS	Tyrosinase	P14679	178	192	23.91
1631	56	DRB1*01:20	YIKSYLEQASRIWSW	Tyrosinase	P14679	463	477	38.8
1632	57	DRB1*01:20	YYVSMDALLGGSEIW	Tyrosinase	P14679	181	195	34.9
1633	58	DRB1*01:20	ALIFGTASYLIRARR	TRP1	P17643	491	505	14.4
1634	59	DRB1*01:20	AVRSLHNLAHLFLNG	TRP1	P17643	372	386	30.45
1635	60	DRB1*01:20	CIFFPLLLFQQARAQ	TRP1	P17643	11	25	28.61
1636	61	DRB1*01:20	DMQEMLQEPSFSLPY	TRP1	P17643	234	248	45.21
1637	62	DRB1*01:20	DPAVRSLHNLAHLFL	TRP1	P17643	370	384	22.2
1638	63	DRB1*01:20	EEKNHFVRALDMAKR	TRP1	P17643	139	153	38.13
1639	64	DRB1*01:20	EKNHFVRALDMAKRT	TRP1	P17643	140	154	33.21
1640	65	DRB1*01:20	FFPLLLFQQARAQFP	TRP1	P17643	13	27	13.29
1641	66	DRB1*01:20	FPLLLFQQARAQFPR	TRP1	P17643	14	28	11.09
1642	67	DRB1*01:20	HPLFVIATRRSEEIL	TRP1	P17643	156	170	20.71
1643	68	DRB1*01:20	IFFPLLLFQQARAQF	TRP1	P17643	12	26	17.4
1644	69	DRB1*01:20	IFGTASYLIRARRSM	TRP1	P17643	493	507	39
1645	70	DRB1*01:20	KNHFVRALDMAKRTT	TRP1	P17643	141	155	35.49
1646	71	DRB1*01:20	KRTTHPLFVIATRRS	TRP1	P17643	152	166	33.95
1647	72	DRB1*01:20	LIFGTASYLIRARRS	TRP1	P17643	492	506	24.92
1648	73	DRB1*01:20	LLLFQQARAQFPRQC	TRP1	P17643	16	30	23.29

1649	74	DRB1*01:20	LLVALIFGTASYLIR	TRP1	P17643	488	502	21.86
1650	75	DRB1*01:20	LVALIFGTASYLIRA	TRP1	P17643	489	503	15.6
1651	76	DRB1*01:20	MQEMLQEPSFSLPYW	TRP1	P17643	235	249	38.72
1652	77	DRB1*01:20	PAVRSLHNLAHLFLN	TRP1	P17643	371	385	22.26
1653	78	DRB1*01:20	PLFVIATRRSEEILG	TRP1	P17643	157	171	29.52
1654	79	DRB1*01:20	PLLLFQQARAQFPRQ	TRP1	P17643	15	29	14.56
1655	80	DRB1*01:20	RTTHPLFVIATRRSE	TRP1	P17643	153	167	22.89
1656	81	DRB1*01:20	THPLFVIATRRSEEI	TRP1	P17643	155	169	19.04
1657	82	DRB1*01:20	TTHPLFVIATRRSEE	TRP1	P17643	154	168	23.55
1658	83	DRB1*01:20	VALIFGTASYLIRAR	TRP1	P17643	490	504	11.1
1659	84	DRB1*01:20	VRSLHNLAHLFLNGT	TRP1	P17643	373	387	47.37
1660	85	DRB1*01:20	YDPAVRSLHNLAHLF	TRP1	P17643	369	383	31.57
1661	86	DRB1*01:20	AEVSIVVLSGTTAAQ	PMEL17	P40967	403	417	10.6
1662	87	DRB1*01:20	AFTITDQVPFSVSVS	PMEL17	P40967	206	220	49.22
1663	88	DRB1*01:20	ALLAVGATKVPRNQD	PMEL17	P40967	17	31	13.76
1664	89	DRB1*01:20	ANASFSIALNFPGSQ	PMEL17	P40967	80	94	30.12
1665	90	DRB1*01:20	AQVVLQAAIPLTSCG	PMEL17	P40967	288	302	7.41
1666	91	DRB1*01:20	ASFSIALNFPGSQKV	PMEL17	P40967	82	96	23.94
1667	92	DRB1*01:20	AVIGALLAVGATKVP	PMEL17	P40967	13	27	6.67
1668	93	DRB1*01:20	AVVLASLIYRRRLMK	PMEL17	P40967	608	622	36.6
1669	94	DRB1*01:20	CQPVLPSPACQLVLH	PMEL17	P40967	541	555	48.38
1670	95	DRB1*01:20	DGPTLIGANASFSIA	PMEL17	P40967	73	87	37.12
1671	96	DRB1*01:20	DSSGTLISRALVVTH	PMEL17	P40967	264	278	27.54
1672	97	DRB1*01:20	EVSIVVLSGTTAAQV	PMEL17	P40967	404	418	10.55
1673	98	DRB1*01:20	FSVSVSQLRALDGGN	PMEL17	P40967	215	229	38.1
1674	99	DRB1*01:20	GALLAVGATKVPRNQ	PMEL17	P40967	16	30	9.89
1675	100	DRB1*01:20	GANASFSIALNFPGS	PMEL17	P40967	79	93	42.19
1676	101	DRB1*01:20	GDSSGTLISRALVVT	PMEL17	P40967	263	277	38.76
1677	102	DRB1*01:20	GGNKHFLRNQPLTFA	PMEL17	P40967	227	241	32.86
1678	103	DRB1*01:20	GLSIGTGRAMLGTHT	PMEL17	P40967	169	183	45.38
1679	104	DRB1*01:20	GNKHFLRNQPLTFAL	PMEL17	P40967	228	242	24.55
1680	105	DRB1*01:20	GPLLDGTATLRLVKR	PMEL17	P40967	455	469	48.61
1681	106	DRB1*01:20	GPTLIGANASFSIAL	PMEL17	P40967	74	88	24.11
1682	107	DRB1*01:20	GPVSGLSIGTGRAML	PMEL17	P40967	165	179	32.64
1683	108	DRB1*01:20	GPVTAQVVLQAAIPL	PMEL17	P40967	284	298	12.23
1684	109	DRB1*01:20	GQYWQVLGGPVSGLS	PMEL17	P40967	157	171	25.6
1685	110	DRB1*01:20	GTLISRALVVTHTYL	PMEL17	P40967	267	281	28.88
1686	111	DRB1*01:20	HFLRNQPLTFALQLH	PMEL17	P40967	231	245	45.8
1687	112	DRB1*01:20	HLAVIGALLAVGATK	PMEL17	P40967	11	25	9.92
1688	113	DRB1*01:20	IGALLAVGATKVPRN	PMEL17	P40967	15	29	7.5

1689	114	DRB1*01:20	IVVLSGTTAAQVTTT	PMEL17	P40967	407	421	23.88
1690	115	DRB1*01:20	KHFLRNQPLTFALQL	PMEL17	P40967	230	244	26.49
1691	116	DRB1*01:20	KRCLLHLAVIGALLA	PMEL17	P40967	6	20	27.86
1692	117	DRB1*01:20	LAVIGALLAVGATKV	PMEL17	P40967	12	26	8.62
1693	118	DRB1*01:20	LHLAVIGALLAVGAT	PMEL17	P40967	10	24	19.84
1694	119	DRB1*01:20	LKRCLLHLAVIGALL	PMEL17	P40967	5	19	33.06
1695	120	DRB1*01:20	LLHLAVIGALLAVGA	PMEL17	P40967	9	23	32.18
1696	121	DRB1*01:20	LMAVVLASLIYRRRL	PMEL17	P40967	606	620	32.68
1697	122	DRB1*01:20	LVLKRCLLHLAVIGA	PMEL17	P40967	3	17	39.1
1698	123	DRB1*01:20	MAVVLASLIYRRRLM	PMEL17	P40967	607	621	32.58
1699	124	DRB1*01:20	NASFSIALNFPGSQK	PMEL17	P40967	81	95	23.03
1700	125	DRB1*01:20	NKHFLRNQPLTFALQ	PMEL17	P40967	229	243	23.92
1701	126	DRB1*01:20	PAEVSIVVLSGTTAA	PMEL17	P40967	402	416	13.64
1702	127	DRB1*01:20	PFSVSVSQLRALDGG	PMEL17	P40967	214	228	44.28
1703	128	DRB1*01:20	PTLIGANASFSIALN	PMEL17	P40967	75	89	28.65
1704	129	DRB1*01:20	PVSGLSIGTGRAMLG	PMEL17	P40967	166	180	25.47
1705	130	DRB1*01:20	PVTAQVVLQAAIPLT	PMEL17	P40967	285	299	7.04
1706	131	DRB1*01:20	QPVLPSPACQLVLHQ	PMEL17	P40967	542	556	47.93
1707	132	DRB1*01:20	QVVLQAAIPLTSCGS	PMEL17	P40967	289	303	9.87
1708	133	DRB1*01:20	QYWQVLGGPVSGLSI	PMEL17	P40967	158	172	30.88
1709	134	DRB1*01:20	RSYVPLAHSSSAFTI	PMEL17	P40967	195	209	44.03
1710	135	DRB1*01:20	SFSIALNFPGSQKVL	PMEL17	P40967	83	97	30.68
1711	136	DRB1*01:20	SGLSIGTGRAMLGTH	PMEL17	P40967	168	182	31.6
1712	137	DRB1*01:20	SGTLISRALVVTHTY	PMEL17	P40967	266	280	24.5
1713	138	DRB1*01:20	SIVVLSGTTAAQVTT	PMEL17	P40967	406	420	11.66
1714	139	DRB1*01:20	SSGTLISRALVVTHT	PMEL17	P40967	265	279	22.66
1715	140	DRB1*01:20	SVSQLRALDGGNKHF	PMEL17	P40967	218	232	42.72
1716	141	DRB1*01:20	SVSVSQLRALDGGNK	PMEL17	P40967	216	230	28.54
1717	142	DRB1*01:20	SYVPLAHSSSAFTIT	PMEL17	P40967	196	210	37.37
1718	143	DRB1*01:20	TAQVVLQAAIPLTSC	PMEL17	P40967	287	301	6.38
1719	144	DRB1*01:20	TLIGANASFSIALNF	PMEL17	P40967	76	90	45.92
1720	145	DRB1*01:20	TLISRALVVTHTYLE	PMEL17	P40967	268	282	42.1
1721	146	DRB1*01:20	TPAEVSIVVLSGTTA	PMEL17	P40967	401	415	24.58
1722	147	DRB1*01:20	TWGQYWQVLGGPVSG	PMEL17	P40967	155	169	38.7
1723	148	DRB1*01:20	VIGALLAVGATKVPR	PMEL17	P40967	14	28	6.21
1724	149	DRB1*01:20	VLKRCLLHLAVIGAL	PMEL17	P40967	4	18	31.22
1725	150	DRB1*01:20	VLMAVVLASLIYRRR	PMEL17	P40967	605	619	35.51
1726	151	DRB1*01:20	VSGLSIGTGRAMLGT	PMEL17	P40967	167	181	21.94
1727	152	DRB1*01:20	VSIVVLSGTTAAQVT	PMEL17	P40967	405	419	11.75
1728	153	DRB1*01:20	VSVSQLRALDGGNKH	PMEL17	P40967	217	231	31.55

1729	154	DRB1*01:20	VTAQVVLQAAIPLTS	PMEL17	P40967	286	300	5.84
1730	155	DRB1*01:20	VVLASLIYRRRLMKQ	PMEL17	P40967	609	623	46.02
1731	156	DRB1*01:20	VVLQAAIPLTSCGSS	PMEL17	P40967	290	304	25.6
1732	157	DRB1*01:20	WGQYWQVLGGPVSGL	PMEL17	P40967	156	170	25.56
1733	158	DRB1*01:20	YVPLAHSSSAFTITD	PMEL17	P40967	197	211	42.06
1734	159	DRB1*01:20	ADLVGFLLLKYRARE	MAGE1	P43355	107	121	46.66
1735	160	DRB1*01:20	ALGLVCVQAATSSSS	MAGE1	P43355	22	36	26.14
1736	161	DRB1*01:20	ARVRFFFPSLREAAL	MAGE1	P43355	288	302	32.67
1737	162	DRB1*01:20	ARYEFLWGPRALAET	MAGE1	P43355	260	274	23.93
1738	163	DRB1*01:20	ASESLQLVFGIDVKE	MAGE1	P43355	147	161	23.86
1739	164	DRB1*01:20	AVITKKVADLVGFLL	MAGE1	P43355	100	114	40.41
1740	165	DRB1*01:20	CILESLFRAVITKKV	MAGE1	P43355	92	106	28.79
1741	166	DRB1*01:20	DLVGFLLLKYRAREP	MAGE1	P43355	108	122	48.15
1742	167	DRB1*01:20	DNQIMPKTGFLIIVL	MAGE1	P43355	185	199	21.82
1743	168	DRB1*01:20	DPARYEFLWGPRALA	MAGE1	P43355	258	272	25.1
1744	169	DRB1*01:20	EALGLVCVQAATSSS	MAGE1	P43355	21	35	32.06
1745	170	DRB1*01:20	ESLFRAVITKKVADL	MAGE1	P43355	95	109	16.38
1746	171	DRB1*01:20	ESLQLVFGIDVKEAD	MAGE1	P43355	149	163	25.16
1747	172	DRB1*01:20	EYVIKVSARVRFFFP	MAGE1	P43355	281	295	21.35
1748	173	DRB1*01:20	FRAVITKKVADLVGF	MAGE1	P43355	98	112	45.37
1749	174	DRB1*01:20	GDNQIMPKTGFLIIV	MAGE1	P43355	184	198	27.24
1750	175	DRB1*01:20	GLVCVQAATSSSSPL	MAGE1	P43355	24	38	43.15
1751	176	DRB1*01:20	IIVLVMIAMEGGHAP	MAGE1	P43355	196	210	12.7
1752	177	DRB1*01:20	ILESLFRAVITKKVA	MAGE1	P43355	93	107	17.3
1753	178	DRB1*01:20	ITKKVADLVGFLLLK	MAGE1	P43355	102	116	19.09
1754	179	DRB1*01:20	IVLVMIAMEGGHAPE	MAGE1	P43355	197	211	12.17
1755	180	DRB1*01:20	KASESLQLVFGIDVK	MAGE1	P43355	146	160	37.02
1756	181	DRB1*01:20	KKVADLVGFLLLKYR	MAGE1	P43355	104	118	22.78
1757	182	DRB1*01:20	KVADLVGFLLLKYRA	MAGE1	P43355	105	119	46.74
1758	183	DRB1*01:20	KVLEYVIKVSARVRF	MAGE1	P43355	278	292	21.27
1759	184	DRB1*01:20	LESLFRAVITKKVAD	MAGE1	P43355	94	108	19.18
1760	185	DRB1*01:20	LEYVIKVSARVRFFF	MAGE1	P43355	280	294	18.46
1761	186	DRB1*01:20	LFRAVITKKVADLVG	MAGE1	P43355	97	111	28.17
1762	187	DRB1*01:20	LGDNQIMPKTGFLII	MAGE1	P43355	183	197	38.57
1763	188	DRB1*01:20	LGLVCVQAATSSSSP	MAGE1	P43355	23	37	30.99
1764	189	DRB1*01:20	LIIVLVMIAMEGGHA	MAGE1	P43355	195	209	24.56
1765	190	DRB1*01:20	LVGFLLLKYRAREPV	MAGE1	P43355	109	123	39.34
1766	191	DRB1*01:20	LVMIAMEGGHAPEEE	MAGE1	P43355	199	213	18.41
1767	192	DRB1*01:20	NQIMPKTGFLIIVLV	MAGE1	P43355	186	200	24.19
1768	193	DRB1*01:20	PARYEFLWGPRALAE	MAGE1	P43355	259	273	26.24

1769	194	DRB1*01:20	QEALGLVCVQAATSS	MAGE1	P43355	20	34	41.47
1770	195	DRB1*01:20	QIMPKTGFLIIVLVM	MAGE1	P43355	187	201	40.75
1771	196	DRB1*01:20	RVRFFFPSLREAALR	MAGE1	P43355	289	303	36.17
1772	197	DRB1*01:20	RYEFLWGPRALAETS	MAGE1	P43355	261	275	33.24
1773	198	DRB1*01:20	SARVRFFFPSLREAA	MAGE1	P43355	287	301	33.55
1774	199	DRB1*01:20	SDPARYEFLWGPRAL	MAGE1	P43355	257	271	36.34
1775	200	DRB1*01:20	SESLQLVFGIDVKEA	MAGE1	P43355	148	162	17.94
1776	201	DRB1*01:20	SLFRAVITKKVADLV	MAGE1	P43355	96	110	18.38
1777	202	DRB1*01:20	TKKVADLVGFLLLKY	MAGE1	P43355	103	117	18.06
1778	203	DRB1*01:20	VADLVGFLLLKYRAR	MAGE1	P43355	106	120	49.37
1779	204	DRB1*01:20	VGFLLLKYRAREPVT	MAGE1	P43355	110	124	43.35
1780	205	DRB1*01:20	VITKKVADLVGFLLL	MAGE1	P43355	101	115	36.19
1781	206	DRB1*01:20	VKVLEYVIKVSARVR	MAGE1	P43355	277	291	29.48
1782	207	DRB1*01:20	VLEYVIKVSARVRFF	MAGE1	P43355	279	293	19.43
1783	208	DRB1*01:20	VLVMIAMEGGHAPEE	MAGE1	P43355	198	212	14.12
1784	209	DRB1*01:20	VMIAMEGGHAPEEEI	MAGE1	P43355	200	214	35.4
1785	210	DRB1*01:20	VSARVRFFFPSLREA	MAGE1	P43355	286	300	39.61
1786	211	DRB1*01:20	YVIKVSARVRFFFPS	MAGE1	P43355	282	296	33.79
1787	212	DRB1*01:20	AESLFREALSNKVDE	MAGE4	P43358	102	116	22.1
1788	213	DRB1*01:20	AHFLLRKYRAKELVT	MAGE4	P43358	118	132	38.78
1789	214	DRB1*01:20	ARVRIAYPSLREAAL	MAGE4	P43358	296	310	31.08
1790	215	DRB1*01:20	ARYEFLWGPRALAET	MAGE4	P43358	268	282	23.93
1791	216	DRB1*01:20	ASESLKMIFGIDVKE	MAGE4	P43358	155	169	9.14
1792	217	DRB1*01:20	CFPVIFGKASESLKM	MAGE4	P43358	147	161	31.33
1793	218	DRB1*01:20	DAESLFREALSNKVD	MAGE4	P43358	101	115	23.03
1794	219	DRB1*01:20	EHVVRVNARVRIAYP	MAGE4	P43358	289	303	45.61
1795	220	DRB1*01:20	ESLFREALSNKVDEL	MAGE4	P43358	103	117	15.57
1796	221	DRB1*01:20	ESLKMIFGIDVKEVD	MAGE4	P43358	157	171	9.92
1797	222	DRB1*01:20	FLLRKYRAKELVTKA	MAGE4	P43358	120	134	39.88
1798	223	DRB1*01:20	FPVIFGKASESLKMI	MAGE4	P43358	148	162	26.81
1799	224	DRB1*01:20	GKASESLKMIFGIDV	MAGE4	P43358	153	167	39.4
1800	225	DRB1*01:20	GNNQIFPKTGLLIIV	MAGE4	P43358	192	206	28.64
1801	226	DRB1*01:20	HFLLRKYRAKELVTK	MAGE4	P43358	119	133	33.95
1802	227	DRB1*01:20	KASESLKMIFGIDVK	MAGE4	P43358	154	168	11.33
1803	228	DRB1*01:20	KVLEHVVRVNARVRI	MAGE4	P43358	286	300	43.27
1804	229	DRB1*01:20	LAHFLLRKYRAKELV	MAGE4	P43358	117	131	41.54
1805	230	DRB1*01:20	LEHVVRVNARVRIAY	MAGE4	P43358	288	302	32.28
1806	231	DRB1*01:20	LFREALSNKVDELAH	MAGE4	P43358	105	119	32.68
1807	232	DRB1*01:20	LGNNQIFPKTGLLII	MAGE4	P43358	191	205	39.66
1808	233	DRB1*01:20	NARVRIAYPSLREAA	MAGE4	P43358	295	309	30.32

1809	234	DRB1*01:20	NNQIFPKTGLLIIVL	MAGE4	P43358	193	207	25.4
1810	235	DRB1*01:20	NPARYEFLWGPRALA	MAGE4	P43358	266	280	25.1
1811	236	DRB1*01:20	NQIFPKTGLLIIVLG	MAGE4	P43358	194	208	31.9
1812	237	DRB1*01:20	PARYEFLWGPRALAE	MAGE4	P43358	267	281	26.24
1813	238	DRB1*01:20	PDAESLFREALSNKV	MAGE4	P43358	100	114	44.71
1814	239	DRB1*01:20	PVIFGKASESLKMIF	MAGE4	P43358	149	163	45.82
1815	240	DRB1*01:20	RCFPVIFGKASESLK	MAGE4	P43358	146	160	45.62
1816	241	DRB1*01:20	RVNARVRIAYPSLRE	MAGE4	P43358	293	307	43.79
1817	242	DRB1*01:20	RVRIAYPSLREAALL	MAGE4	P43358	297	311	41.6
1818	243	DRB1*01:20	RYEFLWGPRALAETS	MAGE4	P43358	269	283	33.24
1819	244	DRB1*01:20	SESLKMIFGIDVKEV	MAGE4	P43358	156	170	8.1
1820	245	DRB1*01:20	SLFREALSNKVDELA	MAGE4	P43358	104	118	17.43
1821	246	DRB1*01:20	SLKMIFGIDVKEVDP	MAGE4	P43358	158	172	18.88
1822	247	DRB1*01:20	SNPARYEFLWGPRAL	MAGE4	P43358	265	279	36.34
1823	248	DRB1*01:20	VLEHVVRVNARVRIA	MAGE4	P43358	287	301	36.74
1824	249	DRB1*01:20	VNARVRIAYPSLREA	MAGE4	P43358	294	308	35.36
1825	250	DRB1*01:20	ADHRQLQLSISSCLQ	NY-ESO-1	P78358	140	154	27.91
1826	251	DRB1*01:20	ATPMEAELARRSLAQ	NY-ESO-1	P78358	97	111	45.27
1827	252	DRB1*01:20	CFLPVFLAQPPSGQR	NY-ESO-1	P78358	165	179	47.69
1828	253	DRB1*01:20	DHRQLQLSISSCLQQ	NY-ESO-1	P78358	141	155	18.34
1829	254	DRB1*01:20	EFYLAMPFATPMEAE	NY-ESO-1	P78358	89	103	28.21
1830	255	DRB1*01:20	FATPMEAELARRSLA	NY-ESO-1	P78358	96	110	40.84
1831	256	DRB1*01:20	FLPVFLAQPPSGQRR	NY-ESO-1	P78358	166	180	33.75
1832	257	DRB1*01:20	GNILTIRLTAADHRQ	NY-ESO-1	P78358	130	144	33.57
1833	258	DRB1*01:20	HRQLQLSISSCLQQL	NY-ESO-1	P78358	142	156	16.16
1834	259	DRB1*01:20	ILTIRLTAADHRQLQ	NY-ESO-1	P78358	132	146	36.07
1835	260	DRB1*01:20	KEFTVSGNILTIRLT	NY-ESO-1	P78358	124	138	40.53
1836	261	DRB1*01:20	LEFYLAMPFATPMEA	NY-ESO-1	P78358	88	102	19.88
1837	262	DRB1*01:20	LKEFTVSGNILTIRL	NY-ESO-1	P78358	123	137	36.4
1838	263	DRB1*01:20	LLEFYLAMPFATPME	NY-ESO-1	P78358	87	101	20.13
1839	264	DRB1*01:20	LLKEFTVSGNILTIR	NY-ESO-1	P78358	122	136	39.87
1840	265	DRB1*01:20	LTIRLTAADHRQLQL	NY-ESO-1	P78358	133	147	46.46
1841	266	DRB1*01:20	NILTIRLTAADHRQL	NY-ESO-1	P78358	131	145	31.62
1842	267	DRB1*01:20	RLLEFYLAMPFATPM	NY-ESO-1	P78358	86	100	24.34
1843	268	DRB1*01:20	RQLQLSISSCLQQLS	NY-ESO-1	P78358	143	157	24.36
1844	269	DRB1*01:20	SGNILTIRLTAADHR	NY-ESO-1	P78358	129	143	34.64
1845	270	DRB1*01:20	SRLLEFYLAMPFATP	NY-ESO-1	P78358	85	99	26.35
1846	271	DRB1*01:20	VSGNILTIRLTAADH	NY-ESO-1	P78358	128	142	47.44
1847	272	DRB1*01:20	AARAVFLALSAQLLQ	BAGE	Q13072	2	16	7.27
1848	273	DRB1*01:20	ALSAQLLQARLMKEE	BAGE	Q13072	9	23	13.77

1849	274	DRB1*01:20	ARAVFLALSAQLLQA	BAGE	Q13072	3	17	6.56
1850	275	DRB1*01:20	ARLMKEESPVVSWRL	BAGE	Q13072	17	31	43.74
1851	276	DRB1*01:20	AVFLALSAQLLQARL	BAGE	Q13072	5	19	6.68
1852	277	DRB1*01:20	FLALSAQLLQARLMK	BAGE	Q13072	7	21	10.67
1853	278	DRB1*01:20	LALSAQLLQARLMKE	BAGE	Q13072	8	22	13.14
1854	279	DRB1*01:20	LSAQLLQARLMKEES	BAGE	Q13072	10	24	17.14
1855	280	DRB1*01:20	MAARAVFLALSAQLL	BAGE	Q13072	1	15	10.66
1856	281	DRB1*01:20	QARLMKEESPVVSWR	BAGE	Q13072	16	30	43.33
1857	282	DRB1*01:20	RAVFLALSAQLLQAR	BAGE	Q13072	4	18	4.91
1858	283	DRB1*01:20	SAQLLQARLMKEESP	BAGE	Q13072	11	25	29.09
1859	284	DRB1*01:20	VFLALSAQLLQARLM	BAGE	Q13072	6	20	7.51
1860	285	DRB1*01:20	FGRLQGISPKIMPKK	SSX2	Q16385	101	115	22.29
1861	286	DRB1*01:20	GRLQGISPKIMPKKP	SSX2	Q16385	102	116	42.51
1862	287	DRB1*01:20	KLGFKATLPPFMCNK	SSX2	Q16385	60	74	33.19
1863	288	DRB1*01:20	MTFGRLQGISPKIMP	SSX2	Q16385	99	113	29.64
1864	289	DRB1*01:20	PQMTFGRLQGISPKI	SSX2	Q16385	97	111	41.41
1865	290	DRB1*01:20	QMTFGRLQGISPKIM	SSX2	Q16385	98	112	27.43
1866	291	DRB1*01:20	TFGRLQGISPKIMPK	SSX2	Q16385	100	114	25.76
1867	292	DRB1*01:20	TKLGFKATLPPFMCN	SSX2	Q16385	59	73	45.37
1868	1	DRB1*01:24	KVYYYRFVIGLRVWQ	TRP2	O75767	206	220	6.44
1869	2	DRB1*01:24	LKVYYYRFVIGLRVW	TRP2	O75767	205	219	8.17
1870	3	DRB1*01:24	VYYYRFVIGLRVWQW	TRP2	O75767	207	221	7.21
1871	4	DRB1*01:24	WLKVYYYRFVIGLRV	TRP2	O75767	204	218	10.06
1872	5	DRB1*01:24	YRFVIGLRVWQWEVI	TRP2	O75767	210	224	33.28
1873	6	DRB1*01:24	YYRFVIGLRVWQWEV	TRP2	O75767	209	223	9.55
1874	7	DRB1*01:24	YYYRFVIGLRVWQWE	TRP2	O75767	208	222	6.88
1875	8	DRB1*01:24	DFQEFMAFVAMVTTA	S100	P04271	68	82	43.3
1876	9	DRB1*01:24	EFMAFVAMVTTACHE	S100	P04271	71	85	34.66
1877	10	DRB1*01:24	FMAFVAMVTTACHEF	S100	P04271	72	86	36.98
1878	11	DRB1*01:24	MAFVAMVTTACHEFF	S100	P04271	73	87	39.17
1879	12	DRB1*01:24	QEFMAFVAMVTTACH	S100	P04271	70	84	33.55
1880	13	DRB1*01:24	AMVGAVLTALLAGLV	Tyrosinase	P14679	482	496	42.14
1881	14	DRB1*01:24	ASRIWSWLLGAAMVG	Tyrosinase	P14679	471	485	18.32
1882	15	DRB1*01:24	AVLTALLAGLVSLLC	Tyrosinase	P14679	486	500	22.21
1883	16	DRB1*01:24	AVLYCLLWSFQTSAG	Tyrosinase	P14679	4	18	49.86
1884	17	DRB1*01:24	DKFFAYLTLAKHTIS	Tyrosinase	P14679	132	146	45
1885	18	DRB1*01:24	EQASRIWSWLLGAAM	Tyrosinase	P14679	469	483	49.4
1886	19	DRB1*01:24	FVWMHYYVSMDALLG	Tyrosinase	P14679	176	190	24.65
1887	20	DRB1*01:24	GAVLTALLAGLVSLL	Tyrosinase	P14679	485	499	14.8

1888	21	DRB1*01:24	HYYVSMDALLGGSEI	Tyrosinase	P14679	180	194	20.06
1889	22	DRB1*01:24	IWSWLLGAAMVGAVL	Tyrosinase	P14679	474	488	33.81
1890	23	DRB1*01:24	KDKFFAYLTLAKHTI	Tyrosinase	P14679	131	145	47.12
1891	24	DRB1*01:24	KFFAYLTLAKHTISS	Tyrosinase	P14679	133	147	38.64
1892	25	DRB1*01:24	LTALLAGLVSLLCRH	Tyrosinase	P14679	488	502	42.01
1893	26	DRB1*01:24	MHYYVSMDALLGGSE	Tyrosinase	P14679	179	193	15.82
1894	27	DRB1*01:24	MVGAVLTALLAGLVS	Tyrosinase	P14679	483	497	17.95
1895	28	DRB1*01:24	QASRIWSWLLGAAMV	Tyrosinase	P14679	470	484	22.77
1896	29	DRB1*01:24	RIWSWLLGAAMVGAV	Tyrosinase	P14679	473	487	17.09
1897	30	DRB1*01:24	SRIWSWLLGAAMVGA	Tyrosinase	P14679	472	486	14.32
1898	31	DRB1*01:24	TALLAGLVSLLCRHK	Tyrosinase	P14679	489	503	38.53
1899	32	DRB1*01:24	VGAVLTALLAGLVSL	Tyrosinase	P14679	484	498	14.02
1900	33	DRB1*01:24	VLTALLAGLVSLLCR	Tyrosinase	P14679	487	501	31.75
1901	34	DRB1*01:24	VWMHYYVSMDALLGG	Tyrosinase	P14679	177	191	15.9
1902	35	DRB1*01:24	WMHYYVSMDALLGGS	Tyrosinase	P14679	178	192	13.67
1903	36	DRB1*01:24	YYVSMDALLGGSEIW	Tyrosinase	P14679	181	195	24.46
1904	37	DRB1*01:24	ALIFGTASYLIRARR	TRP1	P17643	491	505	29.39
1905	38	DRB1*01:24	FPLLLFQQARAQFPR	TRP1	P17643	14	28	30.7
1906	39	DRB1*01:24	LLLFQQARAQFPRQC	TRP1	P17643	16	30	36.75
1907	40	DRB1*01:24	LLVALIFGTASYLIR	TRP1	P17643	488	502	28.65
1908	41	DRB1*01:24	LVALIFGTASYLIRA	TRP1	P17643	489	503	20.63
1909	42	DRB1*01:24	PLLLFQQARAQFPRQ	TRP1	P17643	15	29	28.91
1910	43	DRB1*01:24	VALIFGTASYLIRAR	TRP1	P17643	490	504	19.4
1911	44	DRB1*01:24	AEVSIVVLSGTTAAQ	PMEL17	P40967	403	417	20.78
1912	45	DRB1*01:24	ALLAVGATKVPRNQD	PMEL17	P40967	17	31	30.54
1913	46	DRB1*01:24	ANASFSIALNFPGSQ	PMEL17	P40967	80	94	27.76
1914	47	DRB1*01:24	AQVVLQAAIPLTSCG	PMEL17	P40967	288	302	15.16
1915	48	DRB1*01:24	ASFSIALNFPGSQKV	PMEL17	P40967	82	96	23.59
1916	49	DRB1*01:24	AVIGALLAVGATKVP	PMEL17	P40967	13	27	14.65
1917	50	DRB1*01:24	EVSIVVLSGTTAAQV	PMEL17	P40967	404	418	17.34
1918	51	DRB1*01:24	GALLAVGATKVPRNQ	PMEL17	P40967	16	30	20.5
1919	52	DRB1*01:24	GANASFSIALNFPGS	PMEL17	P40967	79	93	35.66
1920	53	DRB1*01:24	GNKHFLRNQPLTFAL	PMEL17	P40967	228	242	42.88
1921	54	DRB1*01:24	GPVTAQVVLQAAIPL	PMEL17	P40967	284	298	29.96
1922	55	DRB1*01:24	GQYWQVLGGPVSGLS	PMEL17	P40967	157	171	19.53
1923	56	DRB1*01:24	HLAVIGALLAVGATK	PMEL17	P40967	11	25	20.49
1924	57	DRB1*01:24	IGALLAVGATKVPRN	PMEL17	P40967	15	29	14.57
1925	58	DRB1*01:24	KHFLRNQPLTFALQL	PMEL17	P40967	230	244	45.15
1926	59	DRB1*01:24	KRCLLHLAVIGALLA	PMEL17	P40967	6	20	29.79
1927	60	DRB1*01:24	KTWGQYWQVLGGPVS	PMEL17	P40967	154	168	49.99

1928	61	DRB1*01:24	LAVIGALLAVGATKV	PMEL17	P40967	12	26	16.05
1929	62	DRB1*01:24	LHLAVIGALLAVGAT	PMEL17	P40967	10	24	31.38
1930	63	DRB1*01:24	LKRCLLHLAVIGALL	PMEL17	P40967	5	19	38.93
1931	64	DRB1*01:24	LLHLAVIGALLAVGA	PMEL17	P40967	9	23	41.31
1932	65	DRB1*01:24	NASFSIALNFPGSQK	PMEL17	P40967	81	95	22.02
1933	66	DRB1*01:24	NKHFLRNQPLTFALQ	PMEL17	P40967	229	243	43.04
1934	67	DRB1*01:24	PAEVSIVVLSGTTAA	PMEL17	P40967	402	416	25.74
1935	68	DRB1*01:24	PVTAQVVLQAAIPLT	PMEL17	P40967	285	299	14.92
1936	69	DRB1*01:24	QVVLQAAIPLTSCGS	PMEL17	P40967	289	303	22.49
1937	70	DRB1*01:24	QYWQVLGGPVSGLSI	PMEL17	P40967	158	172	24.54
1938	71	DRB1*01:24	RCLLHLAVIGALLAV	PMEL17	P40967	7	21	48.3
1939	72	DRB1*01:24	SFSIALNFPGSQKVL	PMEL17	P40967	83	97	36.75
1940	73	DRB1*01:24	SIVVLSGTTAAQVTT	PMEL17	P40967	406	420	23.48
1941	74	DRB1*01:24	TAQVVLQAAIPLTSC	PMEL17	P40967	287	301	13.47
1942	75	DRB1*01:24	TWGQYWQVLGGPVSG	PMEL17	P40967	155	169	30.15
1943	76	DRB1*01:24	VIGALLAVGATKVPR	PMEL17	P40967	14	28	12.55
1944	77	DRB1*01:24	VLKRCLLHLAVIGAL	PMEL17	P40967	4	18	45.78
1945	78	DRB1*01:24	VSIVVLSGTTAAQVT	PMEL17	P40967	405	419	18.37
1946	79	DRB1*01:24	VTAQVVLQAAIPLTS	PMEL17	P40967	286	300	12.6
1947	80	DRB1*01:24	WGQYWQVLGGPVSGL	PMEL17	P40967	156	170	19.21
1948	81	DRB1*01:24	ARYEFLWGPRALAET	MAGE1	P43355	260	274	20.15
1949	82	DRB1*01:24	CILESLFRAVITKKV	MAGE1	P43355	92	106	30.97
1950	83	DRB1*01:24	DPARYEFLWGPRALA	MAGE1	P43355	258	272	18.56
1951	84	DRB1*01:24	ESLFRAVITKKVADL	MAGE1	P43355	95	109	12.26
1952	85	DRB1*01:24	EYVIKVSARVRFFFP	MAGE1	P43355	281	295	25.2
1953	86	DRB1*01:24	ILESLFRAVITKKVA	MAGE1	P43355	93	107	14.71
1954	87	DRB1*01:24	IVLVMIAMEGGHAPE	MAGE1	P43355	197	211	49.25
1955	88	DRB1*01:24	KVLEYVIKVSARVRF	MAGE1	P43355	278	292	21.68
1956	89	DRB1*01:24	LESLFRAVITKKVAD	MAGE1	P43355	94	108	16.11
1957	90	DRB1*01:24	LEYVIKVSARVRFFF	MAGE1	P43355	280	294	18.45
1958	91	DRB1*01:24	LFRAVITKKVADLVG	MAGE1	P43355	97	111	18.77
1959	92	DRB1*01:24	PARYEFLWGPRALAE	MAGE1	P43355	259	273	20.02
1960	93	DRB1*01:24	RYEFLWGPRALAETS	MAGE1	P43355	261	275	29.01
1961	94	DRB1*01:24	SDPARYEFLWGPRAL	MAGE1	P43355	257	271	25.56
1962	95	DRB1*01:24	SESLQLVFGIDVKEA	MAGE1	P43355	148	162	42.19
1963	96	DRB1*01:24	SLFRAVITKKVADLV	MAGE1	P43355	96	110	12.74
1964	97	DRB1*01:24	TKKVADLVGFLLLKY	MAGE1	P43355	103	117	42.65
1965	98	DRB1*01:24	VKVLEYVIKVSARVR	MAGE1	P43355	277	291	28.97
1966	99	DRB1*01:24	VLEYVIKVSARVRFF	MAGE1	P43355	279	293	18.49
1967	100	DRB1*01:24	AESLFREALSNKVDE	MAGE4	P43358	102	116	13.79

1968	101	DRB1*01:24	ARYEFLWGPRALAET	MAGE4	P43358	268	282	20.15
1969	102	DRB1*01:24	ASESLKMIFGIDVKE	MAGE4	P43358	155	169	22.73
1970	103	DRB1*01:24	DAESLFREALSNKVD	MAGE4	P43358	101	115	14.67
1971	104	DRB1*01:24	ESLFREALSNKVDEL	MAGE4	P43358	103	117	9.95
1972	105	DRB1*01:24	ESLKMIFGIDVKEVD	MAGE4	P43358	157	171	21.72
1973	106	DRB1*01:24	KASESLKMIFGIDVK	MAGE4	P43358	154	168	26.68
1974	107	DRB1*01:24	LFREALSNKVDELAH	MAGE4	P43358	105	119	16.94
1975	108	DRB1*01:24	NPARYEFLWGPRALA	MAGE4	P43358	266	280	18.56
1976	109	DRB1*01:24	PARYEFLWGPRALAE	MAGE4	P43358	267	281	20.02
1977	110	DRB1*01:24	PDAESLFREALSNKV	MAGE4	P43358	100	114	25.31
1978	111	DRB1*01:24	RYEFLWGPRALAETS	MAGE4	P43358	269	283	29.01
1979	112	DRB1*01:24	SESLKMIFGIDVKEV	MAGE4	P43358	156	170	17.73
1980	113	DRB1*01:24	SLFREALSNKVDELA	MAGE4	P43358	104	118	10.49
1981	114	DRB1*01:24	SLKMIFGIDVKEVDP	MAGE4	P43358	158	172	35.57
1982	115	DRB1*01:24	SNPARYEFLWGPRAL	MAGE4	P43358	265	279	25.56
1983	116	DRB1*01:24	DHRQLQLSISSCLQQ	NY-ESO-1	P78358	141	155	40.38
1984	117	DRB1*01:24	EFYLAMPFATPMEAE	NY-ESO-1	P78358	89	103	18.55
1985	118	DRB1*01:24	ESRLLEFYLAMPFAT	NY-ESO-1	P78358	84	98	31.04
1986	119	DRB1*01:24	HRQLQLSISSCLQQL	NY-ESO-1	P78358	142	156	31.2
1987	120	DRB1*01:24	KEFTVSGNILTIRLT	NY-ESO-1	P78358	124	138	21.45
1988	121	DRB1*01:24	LEFYLAMPFATPMEA	NY-ESO-1	P78358	88	102	11.19
1989	122	DRB1*01:24	LKEFTVSGNILTIRL	NY-ESO-1	P78358	123	137	15.84
1990	123	DRB1*01:24	LLEFYLAMPFATPME	NY-ESO-1	P78358	87	101	9.74
1991	124	DRB1*01:24	LLKEFTVSGNILTIR	NY-ESO-1	P78358	122	136	19.77
1992	125	DRB1*01:24	RLLEFYLAMPFATPM	NY-ESO-1	P78358	86	100	10.64
1993	126	DRB1*01:24	RQLQLSISSCLQQLS	NY-ESO-1	P78358	143	157	46.8
1994	127	DRB1*01:24	SRLLEFYLAMPFATP	NY-ESO-1	P78358	85	99	13.22
1995	128	DRB1*01:24	VLLKEFTVSGNILTI	NY-ESO-1	P78358	121	135	36.91
1996	129	DRB1*01:24	AARAVFLALSAQLLQ	BAGE	Q13072	2	16	3.64
1997	130	DRB1*01:24	ARAVFLALSAQLLQA	BAGE	Q13072	3	17	3.42
1998	131	DRB1*01:24	AVFLALSAQLLQARL	BAGE	Q13072	5	19	3.48
1999	132	DRB1*01:24	FLALSAQLLQARLMK	BAGE	Q13072	7	21	9.15
2000	133	DRB1*01:24	MAARAVFLALSAQLL	BAGE	Q13072	1	15	4.77
2001	134	DRB1*01:24	RAVFLALSAQLLQAR	BAGE	Q13072	4	18	3.13
2002	135	DRB1*01:24	VFLALSAQLLQARLM	BAGE	Q13072	6	20	3.92
2003	136	DRB1*01:24	KLGFKATLPPFMCNK	SSX2	Q16385	60	74	21.8
2004	137	DRB1*01:24	LGFKATLPPFMCNKR	SSX2	Q16385	61	75	39.73
2005	138	DRB1*01:24	MTFGRLQGISPKIMP	SSX2	Q16385	99	113	43.21
2006	139	DRB1*01:24	MTKLGFKATLPPFMC	SSX2	Q16385	58	72	43.21
2007	140	DRB1*01:24	PQMTFGRLQGISPKI	SSX2	Q16385	97	111	43.72

2008	141	DRB1*01:24	QMTFGRLQGISPKIM	SSX2	Q16385	98	112	31.05
2009	142	DRB1*01:24	TKLGFKATLPPFMCN	SSX2	Q16385	59	73	31.98
2010	143	DRB1*01:24	RNGYRALMDKSLHVG	MELAN_A	Q16655	51	65	44.52
2011	144	DRB1*01:24	RRNGYRALMDKSLHV	MELAN_A	Q16655	50	64	46.66
2012	1	DRB1*01:29	KVYYYRFVIGLRVWQ	TRP2	O75767	206	220	9.64
2013	2	DRB1*01:29	LKVYYYRFVIGLRVW	TRP2	O75767	205	219	14.7
2014	3	DRB1*01:29	VYYYRFVIGLRVWQW	TRP2	O75767	207	221	11.62
2015	4	DRB1*01:29	WLKVYYYRFVIGLRV	TRP2	O75767	204	218	16.28
2016	5	DRB1*01:29	YRFVIGLRVWQWEVI	TRP2	O75767	210	224	49.96
2017	6	DRB1*01:29	YYRFVIGLRVWQWEV	TRP2	O75767	209	223	14.91
2018	7	DRB1*01:29	YYYRFVIGLRVWQWE	TRP2	O75767	208	222	10.94
2019	8	DRB1*01:29	EFMAFVAMVTTACHE	S100	P04271	71	85	38.12
2020	9	DRB1*01:29	FMAFVAMVTTACHEF	S100	P04271	72	86	42.84
2021	10	DRB1*01:29	MAFVAMVTTACHEFF	S100	P04271	73	87	45.33
2022	11	DRB1*01:29	QEFMAFVAMVTTACH	S100	P04271	70	84	39.96
2023	12	DRB1*01:29	AMVGAVLTALLAGLV	Tyrosinase	P14679	482	496	36.8
2024	13	DRB1*01:29	ASRIWSWLLGAAMVG	Tyrosinase	P14679	471	485	19.7
2025	14	DRB1*01:29	AVLTALLAGLVSLLC	Tyrosinase	P14679	486	500	37.34
2026	15	DRB1*01:29	DKFFAYLTLAKHTIS	Tyrosinase	P14679	132	146	30.36
2027	16	DRB1*01:29	EKDKFFAYLTLAKHT	Tyrosinase	P14679	130	144	47.03
2028	17	DRB1*01:29	FFAYLTLAKHTISSD	Tyrosinase	P14679	134	148	45.71
2029	18	DRB1*01:29	FVWMHYYVSMDALLG	Tyrosinase	P14679	176	190	26.26
2030	19	DRB1*01:29	GAVLTALLAGLVSLL	Tyrosinase	P14679	485	499	22.75
2031	20	DRB1*01:29	HYYVSMDALLGGSEI	Tyrosinase	P14679	180	194	15.66
2032	21	DRB1*01:29	IWSWLLGAAMVGAVL	Tyrosinase	P14679	474	488	43.99
2033	22	DRB1*01:29	KDKFFAYLTLAKHTI	Tyrosinase	P14679	131	145	32.17
2034	23	DRB1*01:29	KFFAYLTLAKHTISS	Tyrosinase	P14679	133	147	29.56
2035	24	DRB1*01:29	MHYYVSMDALLGGSE	Tyrosinase	P14679	179	193	12.68
2036	25	DRB1*01:29	MVGAVLTALLAGLVS	Tyrosinase	P14679	483	497	18.42
2037	26	DRB1*01:29	QASRIWSWLLGAAMV	Tyrosinase	P14679	470	484	26.82
2038	27	DRB1*01:29	RIWSWLLGAAMVGAV	Tyrosinase	P14679	473	487	19.89
2039	28	DRB1*01:29	SRIWSWLLGAAMVGA	Tyrosinase	P14679	472	486	15.72
2040	29	DRB1*01:29	TALLAGLVSLLCRHK	Tyrosinase	P14679	489	503	42.73
2041	30	DRB1*01:29	VGAVLTALLAGLVSL	Tyrosinase	P14679	484	498	18.36
2042	31	DRB1*01:29	VLTALLAGLVSLLCR	Tyrosinase	P14679	487	501	46.6
2043	32	DRB1*01:29	VWMHYYVSMDALLGG	Tyrosinase	P14679	177	191	15.4
2044	33	DRB1*01:29	WMHYYVSMDALLGGS	Tyrosinase	P14679	178	192	12.32
2045	34	DRB1*01:29	YYVSMDALLGGSEIW	Tyrosinase	P14679	181	195	22.18
2046	35	DRB1*01:29	ALIFGTASYLIRARR	TRP1	P17643	491	505	25.63

2047	36	DRB1*01:29	FFPLLLFQQARAQFP	TRP1	P17643	13	27	43.31
2048	37	DRB1*01:29	FPLLLFQQARAQFPR	TRP1	P17643	14	28	27.06
2049	38	DRB1*01:29	LLLFQQARAQFPRQC	TRP1	P17643	16	30	36.24
2050	39	DRB1*01:29	LLVALIFGTASYLIR	TRP1	P17643	488	502	35.64
2051	40	DRB1*01:29	LVALIFGTASYLIRA	TRP1	P17643	489	503	24.53
2052	41	DRB1*01:29	PLLLFQQARAQFPRQ	TRP1	P17643	15	29	25.99
2053	42	DRB1*01:29	VALIFGTASYLIRAR	TRP1	P17643	490	504	19.31
2054	43	DRB1*01:29	AEVSIVVLSGTTAAQ	PMEL17	P40967	403	417	20.55
2055	44	DRB1*01:29	ALLAVGATKVPRNQD	PMEL17	P40967	17	31	29.26
2056	45	DRB1*01:29	ANASFSIALNFPGSQ	PMEL17	P40967	80	94	27.26
2057	46	DRB1*01:29	AQVVLQAAIPLTSCG	PMEL17	P40967	288	302	17.87
2058	47	DRB1*01:29	ASFSIALNFPGSQKV	PMEL17	P40967	82	96	25.21
2059	48	DRB1*01:29	AVIGALLAVGATKVP	PMEL17	P40967	13	27	13.65
2060	49	DRB1*01:29	EVSIVVLSGTTAAQV	PMEL17	P40967	404	418	18.37
2061	50	DRB1*01:29	GALLAVGATKVPRNQ	PMEL17	P40967	16	30	19.18
2062	51	DRB1*01:29	GANASFSIALNFPGS	PMEL17	P40967	79	93	38.11
2063	52	DRB1*01:29	GNKHFLRNQPLTFAL	PMEL17	P40967	228	242	38.99
2064	53	DRB1*01:29	GPVTAQVVLQAAIPL	PMEL17	P40967	284	298	33.99
2065	54	DRB1*01:29	GQYWQVLGGPVSGLS	PMEL17	P40967	157	171	17.61
2066	55	DRB1*01:29	HLAVIGALLAVGATK	PMEL17	P40967	11	25	20.77
2067	56	DRB1*01:29	IGALLAVGATKVPRN	PMEL17	P40967	15	29	13.52
2068	57	DRB1*01:29	KHFLRNQPLTFALQL	PMEL17	P40967	230	244	45.23
2069	58	DRB1*01:29	KRCLLHLAVIGALLA	PMEL17	P40967	6	20	37.86
2070	59	DRB1*01:29	KTWGQYWQVLGGPVS	PMEL17	P40967	154	168	39.78
2071	60	DRB1*01:29	LAVIGALLAVGATKV	PMEL17	P40967	12	26	16.62
2072	61	DRB1*01:29	LHLAVIGALLAVGAT	PMEL17	P40967	10	24	35.46
2073	62	DRB1*01:29	LKRCLLHLAVIGALL	PMEL17	P40967	5	19	45.25
2074	63	DRB1*01:29	NASFSIALNFPGSQK	PMEL17	P40967	81	95	20.38
2075	64	DRB1*01:29	NKHFLRNQPLTFALQ	PMEL17	P40967	229	243	37.1
2076	65	DRB1*01:29	PAEVSIVVLSGTTAA	PMEL17	P40967	402	416	29.81
2077	66	DRB1*01:29	PVTAQVVLQAAIPLT	PMEL17	P40967	285	299	17.43
2078	67	DRB1*01:29	QVVLQAAIPLTSCGS	PMEL17	P40967	289	303	24.04
2079	68	DRB1*01:29	QYWQVLGGPVSGLSI	PMEL17	P40967	158	172	23.44
2080	69	DRB1*01:29	RSYVPLAHSSSAFTI	PMEL17	P40967	195	209	49.65
2081	70	DRB1*01:29	SFSIALNFPGSQKVL	PMEL17	P40967	83	97	43.56
2082	71	DRB1*01:29	SIVVLSGTTAAQVTT	PMEL17	P40967	406	420	26.34
2083	72	DRB1*01:29	TAQVVLQAAIPLTSC	PMEL17	P40967	287	301	15.17
2084	73	DRB1*01:29	TWGQYWQVLGGPVSG	PMEL17	P40967	155	169	25.74
2085	74	DRB1*01:29	VIGALLAVGATKVPR	PMEL17	P40967	14	28	12.09
2086	75	DRB1*01:29	VLKRCLLHLAVIGAL	PMEL17	P40967	4	18	48.64

2087	76	DRB1*01:29	VSIVVLSGTTAAQVT	PMEL17	P40967	405	419	21.95
2088	77	DRB1*01:29	VTAQVVLQAAIPLTS	PMEL17	P40967	286	300	12.98
2089	78	DRB1*01:29	WGQYWQVLGGPVSGL	PMEL17	P40967	156	170	17.78
2090	79	DRB1*01:29	ARYEFLWGPRALAET	MAGE1	P43355	260	274	18.01
2091	80	DRB1*01:29	CILESLFRAVITKKV	MAGE1	P43355	92	106	23.29
2092	81	DRB1*01:29	DPARYEFLWGPRALA	MAGE1	P43355	258	272	16.85
2093	82	DRB1*01:29	ESLFRAVITKKVADL	MAGE1	P43355	95	109	9.45
2094	83	DRB1*01:29	EYVIKVSARVRFFFP	MAGE1	P43355	281	295	18.53
2095	84	DRB1*01:29	IIVLVMIAMEGGHAP	MAGE1	P43355	196	210	43.02
2096	85	DRB1*01:29	ILESLFRAVITKKVA	MAGE1	P43355	93	107	11.49
2097	86	DRB1*01:29	IVLVMIAMEGGHAPE	MAGE1	P43355	197	211	39.03
2098	87	DRB1*01:29	KVLEYVIKVSARVRF	MAGE1	P43355	278	292	17.06
2099	88	DRB1*01:29	LESLFRAVITKKVAD	MAGE1	P43355	94	108	11.81
2100	89	DRB1*01:29	LEYVIKVSARVRFFF	MAGE1	P43355	280	294	15.65
2101	90	DRB1*01:29	LFRAVITKKVADLVG	MAGE1	P43355	97	111	17.65
2102	91	DRB1*01:29	PARYEFLWGPRALAE	MAGE1	P43355	259	273	17.19
2103	92	DRB1*01:29	RYEFLWGPRALAETS	MAGE1	P43355	261	275	26.23
2104	93	DRB1*01:29	SDPARYEFLWGPRAL	MAGE1	P43355	257	271	25.85
2105	94	DRB1*01:29	SESLQLVFGIDVKEA	MAGE1	P43355	148	162	43.62
2106	95	DRB1*01:29	SLFRAVITKKVADLV	MAGE1	P43355	96	110	10.47
2107	96	DRB1*01:29	TKKVADLVGFLLLKY	MAGE1	P43355	103	117	48.69
2108	97	DRB1*01:29	VKVLEYVIKVSARVR	MAGE1	P43355	277	291	19.83
2109	98	DRB1*01:29	VLEYVIKVSARVRFF	MAGE1	P43355	279	293	17.09
2110	99	DRB1*01:29	VLVMIAMEGGHAPEE	MAGE1	P43355	198	212	41.18
2111	100	DRB1*01:29	YVKVLEYVIKVSARV	MAGE1	P43355	276	290	43.11
2112	101	DRB1*01:29	AESLFREALSNKVDE	MAGE4	P43358	102	116	12.21
2113	102	DRB1*01:29	ARYEFLWGPRALAET	MAGE4	P43358	268	282	18.01
2114	103	DRB1*01:29	ASESLKMIFGIDVKE	MAGE4	P43358	155	169	22.1
2115	104	DRB1*01:29	DAESLFREALSNKVD	MAGE4	P43358	101	115	13.37
2116	105	DRB1*01:29	ESLFREALSNKVDEL	MAGE4	P43358	103	117	9.05
2117	106	DRB1*01:29	ESLKMIFGIDVKEVD	MAGE4	P43358	157	171	24.59
2118	107	DRB1*01:29	KASESLKMIFGIDVK	MAGE4	P43358	154	168	28.91
2119	108	DRB1*01:29	LFREALSNKVDELAH	MAGE4	P43358	105	119	15.46
2120	109	DRB1*01:29	NPARYEFLWGPRALA	MAGE4	P43358	266	280	16.85
2121	110	DRB1*01:29	PARYEFLWGPRALAE	MAGE4	P43358	267	281	17.19
2122	111	DRB1*01:29	PDAESLFREALSNKV	MAGE4	P43358	100	114	22.68
2123	112	DRB1*01:29	RYEFLWGPRALAETS	MAGE4	P43358	269	283	26.23
2124	113	DRB1*01:29	SESLKMIFGIDVKEV	MAGE4	P43358	156	170	18.33
2125	114	DRB1*01:29	SLFREALSNKVDELA	MAGE4	P43358	104	118	9.83
2126	115	DRB1*01:29	SLKMIFGIDVKEVDP	MAGE4	P43358	158	172	48.41

2127	116	DRB1*01:29	SNPARYEFLWGPRAL	MAGE4	P43358	265	279	25.85
2128	117	DRB1*01:29	DHRQLQLSISSCLQQ	NY-ESO-1	P78358	141	155	38.66
2129	118	DRB1*01:29	EFYLAMPFATPMEAE	NY-ESO-1	P78358	89	103	17.32
2130	119	DRB1*01:29	ESRLLEFYLAMPFAT	NY-ESO-1	P78358	84	98	40.29
2131	120	DRB1*01:29	HRQLQLSISSCLQQL	NY-ESO-1	P78358	142	156	33.39
2132	121	DRB1*01:29	KEFTVSGNILTIRLT	NY-ESO-1	P78358	124	138	30.74
2133	122	DRB1*01:29	LEFYLAMPFATPMEA	NY-ESO-1	P78358	88	102	12.03
2134	123	DRB1*01:29	LKEFTVSGNILTIRL	NY-ESO-1	P78358	123	137	24.03
2135	124	DRB1*01:29	LLEFYLAMPFATPME	NY-ESO-1	P78358	87	101	11.68
2136	125	DRB1*01:29	LLKEFTVSGNILTIR	NY-ESO-1	P78358	122	136	25.64
2137	126	DRB1*01:29	RLLEFYLAMPFATPM	NY-ESO-1	P78358	86	100	14.23
2138	127	DRB1*01:29	RQLQLSISSCLQQLS	NY-ESO-1	P78358	143	157	49.86
2139	128	DRB1*01:29	SRLLEFYLAMPFATP	NY-ESO-1	P78358	85	99	16.31
2140	129	DRB1*01:29	AARAVFLALSAQLLQ	BAGE	Q13072	2	16	4.2
2141	130	DRB1*01:29	ALSAQLLQARLMKEE	BAGE	Q13072	9	23	42.28
2142	131	DRB1*01:29	ARAVFLALSAQLLQA	BAGE	Q13072	3	17	3.72
2143	132	DRB1*01:29	AVFLALSAQLLQARL	BAGE	Q13072	5	19	3.89
2144	133	DRB1*01:29	FLALSAQLLQARLMK	BAGE	Q13072	7	21	10.95
2145	134	DRB1*01:29	LALSAQLLQARLMKE	BAGE	Q13072	8	22	37.6
2146	135	DRB1*01:29	MAARAVFLALSAQLL	BAGE	Q13072	1	15	6.19
2147	136	DRB1*01:29	RAVFLALSAQLLQAR	BAGE	Q13072	4	18	3.2
2148	137	DRB1*01:29	VFLALSAQLLQARLM	BAGE	Q13072	6	20	4.82
2149	138	DRB1*01:29	KLGFKATLPPFMCNK	SSX2	Q16385	60	74	20.78
2150	139	DRB1*01:29	LGFKATLPPFMCNKR	SSX2	Q16385	61	75	35.33
2151	140	DRB1*01:29	MTFGRLQGISPKIMP	SSX2	Q16385	99	113	49.91
2152	141	DRB1*01:29	MTKLGFKATLPPFMC	SSX2	Q16385	58	72	38.11
2153	142	DRB1*01:29	QMTFGRLQGISPKIM	SSX2	Q16385	98	112	38.38
2154	143	DRB1*01:29	TKLGFKATLPPFMCN	SSX2	Q16385	59	73	28.15
2155	144	DRB1*01:29	RNGYRALMDKSLHVG	MELAN_A	Q16655	51	65	41.89
2156	145	DRB1*01:29	RRNGYRALMDKSLHV	MELAN_A	Q16655	50	64	43.45
2157	1	DRB1*03:01	AKHTISSDYVIPIGT	Tyrosinase	P14679	141	155	42.94
2158	2	DRB1*03:01	KHTISSDYVIPIGTY	Tyrosinase	P14679	142	156	35.22
2159	3	DRB1*03:01	EYVIKVSARVRFFFP	MAGE1	P43355	281	295	36.32
2160	4	DRB1*03:01	LEYVIKVSARVRFFF	MAGE1	P43355	280	294	39.5
2161	5	DRB1*03:01	VLEYVIKVSARVRFF	MAGE1	P43355	279	293	43.72
2162	1	DRB1*03:04	AKHTISSDYVIPIGT	Tyrosinase	P14679	141	155	42.94
2163	2	DRB1*03:04	KHTISSDYVIPIGTY	Tyrosinase	P14679	142	156	35.22
2164	3	DRB1*03:04	EYVIKVSARVRFFFP	MAGE1	P43355	281	295	36.32

2165	4	DRB1*03:04	LEYVIKVSARVRFFF	MAGE1	P43355	280	294	39.5
2166	5	DRB1*03:04	VLEYVIKVSARVRFF	MAGE1	P43355	279	293	43.72
2167	1	DRB1*03:11	CTEVRADTRPWSGPY	TRP2	O75767	61	75	28.01
2168	2	DRB1*03:11	EVRADTRPWSGPYIL	TRP2	O75767	63	77	40.17
2169	3	DRB1*03:11	GQCTEVRADTRPWSG	TRP2	O75767	59	73	32.62
2170	4	DRB1*03:11	QCTEVRADTRPWSGP	TRP2	O75767	60	74	33.23
2171	5	DRB1*03:11	RGQCTEVRADTRPWS	TRP2	O75767	58	72	49.15
2172	6	DRB1*03:11	TEVRADTRPWSGPYI	TRP2	O75767	62	76	28.24
2173	7	DRB1*03:11	AKHTISSDYVIPIGT	Tyrosinase	P14679	141	155	23.51
2174	8	DRB1*03:11	HTISSDYVIPIGTYG	Tyrosinase	P14679	143	157	33.4
2175	9	DRB1*03:11	KHTISSDYVIPIGTY	Tyrosinase	P14679	142	156	20.71
2176	10	DRB1*03:11	LAKHTISSDYVIPIG	Tyrosinase	P14679	140	154	28.63
2177	11	DRB1*03:11	FSVTLDIVQGIESAE	PMEL17	P40967	483	497	48.27
2178	12	DRB1*03:11	GSFSVTLDIVQGIES	PMEL17	P40967	481	495	42.16
2179	13	DRB1*03:11	NKHFLRNQPLTFALQ	PMEL17	P40967	229	243	47.31
2180	14	DRB1*03:11	SFSVTLDIVQGIESA	PMEL17	P40967	482	496	35.19
2181	15	DRB1*03:11	EYVIKVSARVRFFFP	MAGE1	P43355	281	295	18.21
2182	16	DRB1*03:11	KVLEYVIKVSARVRF	MAGE1	P43355	278	292	29.59
2183	17	DRB1*03:11	LEYVIKVSARVRFFF	MAGE1	P43355	280	294	18.47
2184	18	DRB1*03:11	VIKVSARVRFFFPSL	MAGE1	P43355	283	297	40.94
2185	19	DRB1*03:11	VLEYVIKVSARVRFF	MAGE1	P43355	279	293	20.27
2186	20	DRB1*03:11	YVIKVSARVRFFFPS	MAGE1	P43355	282	296	23.05
2187	21	DRB1*03:11	ARVRIAYPSLREAAL	MAGE4	P43358	296	310	47.56
2188	22	DRB1*03:11	EHVVRVNARVRIAYP	MAGE4	P43358	289	303	26.15
2189	23	DRB1*03:11	HVVRVNARVRIAYPS	MAGE4	P43358	290	304	33.12
2190	24	DRB1*03:11	KVLEHVVRVNARVRI	MAGE4	P43358	286	300	40.02
2191	25	DRB1*03:11	LEHVVRVNARVRIAY	MAGE4	P43358	288	302	24.86
2192	26	DRB1*03:11	NARVRIAYPSLREAA	MAGE4	P43358	295	309	38.19
2193	27	DRB1*03:11	VLEHVVRVNARVRIA	MAGE4	P43358	287	301	29.41
2194	28	DRB1*03:11	VNARVRIAYPSLREA	MAGE4	P43358	294	308	44.53
2195	29	DRB1*03:11	ILTIRLTAADHRQLQ	NY-ESO-1	P78358	132	146	45.97
2196	30	DRB1*03:11	LTIRLTAADHRQLQL	NY-ESO-1	P78358	133	147	40.34
2197	31	DRB1*03:11	TIRLTAADHRQLQLS	NY-ESO-1	P78358	134	148	44.73
2198	32	DRB1*03:11	EKMKASEKIFYVYMK	SSX2	Q16385	38	52	48.68
2199	33	DRB1*03:11	EWEKMKASEKIFYVY	SSX2	Q16385	36	50	46.52
2200	34	DRB1*03:11	WEKMKASEKIFYVYM	SSX2	Q16385	37	51	42.21
2201	35	DRB1*03:11	GYRALMDKSLHVGTQ	MELAN_A	Q16655	53	67	45.36
2202	36	DRB1*03:11	NGYRALMDKSLHVGT	MELAN_A	Q16655	52	66	49.33
2203	37	DRB1*03:13	AKHTISSDYVIPIGT	Tyrosinase	P14679	141	155	42.94

2204	38	DRB1*03:13	KHTISSDYVIPIGTY	Tyrosinase	P14679	142	156	35.22
2205	39	DRB1*03:13	EYVIKVSARVRFFFP	MAGE1	P43355	281	295	36.32
2206	40	DRB1*03:13	LEYVIKVSARVRFFF	MAGE1	P43355	280	294	39.5
2207	41	DRB1*03:13	VLEYVIKVSARVRFF	MAGE1	P43355	279	293	43.72
2208	1	DRB1*03:15	EYVIKVSARVRFFFP	MAGE1	P43355	281	295	32.7
2209	2	DRB1*03:15	KVLEYVIKVSARVRF	MAGE1	P43355	278	292	44.51
2210	3	DRB1*03:15	LEYVIKVSARVRFFF	MAGE1	P43355	280	294	36.01
2211	4	DRB1*03:15	VLEYVIKVSARVRFF	MAGE1	P43355	279	293	35.85
2212	5	DRB1*03:15	YVIKVSARVRFFFPS	MAGE1	P43355	282	296	45.46
2213	1	DRB1*04:01	HYYVSMDALLGGSEI	Tyrosinase	P14679	180	194	42.5
2214	2	DRB1*04:01	MHYYVSMDALLGGSE	Tyrosinase	P14679	179	193	34.35
2215	3	DRB1*04:01	VWMHYYVSMDALLGG	Tyrosinase	P14679	177	191	38.16
2216	4	DRB1*04:01	WMHYYVSMDALLGGS	Tyrosinase	P14679	178	192	32.52
2217	5	DRB1*04:01	EMFVTAPDNLGYTYE	TRP1	P17643	451	465	22.96
2218	6	DRB1*04:01	MFVTAPDNLGYTYEI	TRP1	P17643	452	466	31.08
2219	7	DRB1*04:01	NTEMFVTAPDNLGYT	TRP1	P17643	449	463	28.47
2220	8	DRB1*04:01	TEMFVTAPDNLGYTY	TRP1	P17643	450	464	22.85
2221	9	DRB1*04:01	TNTEMFVTAPDNLGY	TRP1	P17643	448	462	33.24
2222	10	DRB1*04:01	ANASFSIALNFPGSQ	PMEL17	P40967	80	94	16.57
2223	11	DRB1*04:01	ASFSIALNFPGSQKV	PMEL17	P40967	82	96	15.42
2224	12	DRB1*04:01	GANASFSIALNFPGS	PMEL17	P40967	79	93	19.17
2225	13	DRB1*04:01	IGANASFSIALNFPG	PMEL17	P40967	78	92	43.78
2226	14	DRB1*04:01	NASFSIALNFPGSQK	PMEL17	P40967	81	95	14.94
2227	15	DRB1*04:01	SFSIALNFPGSQKVL	PMEL17	P40967	83	97	26.88
2228	16	DRB1*04:01	ESLFREALSNKVDEL	MAGE4	P43358	103	117	49.61
2229	17	DRB1*04:01	KEFTVSGNILTIRLT	NY-ESO-1	P78358	124	138	33.31
2230	18	DRB1*04:01	LKEFTVSGNILTIRL	NY-ESO-1	P78358	123	137	26.23
2231	19	DRB1*04:01	LLKEFTVSGNILTIR	NY-ESO-1	P78358	122	136	25.71
2232	20	DRB1*04:01	VLLKEFTVSGNILTI	NY-ESO-1	P78358	121	135	48.48
2233	1	DRB1*04:04	AFVAMVTTACHEFFE	S100	P04271	74	88	43.34
2234	2	DRB1*04:04	EFMAFVAMVTTACHE	S100	P04271	71	85	46.77
2235	3	DRB1*04:04	EKAMVALIDVFHQYS	S100	P04271	3	17	27.71
2236	4	DRB1*04:04	FMAFVAMVTTACHEF	S100	P04271	72	86	33.27
2237	5	DRB1*04:04	KAMVALIDVFHQYSG	S100	P04271	4	18	29.93
2238	6	DRB1*04:04	LEKAMVALIDVFHQY	S100	P04271	2	16	35.24
2239	7	DRB1*04:04	MAFVAMVTTACHEFF	S100	P04271	73	87	28.91
2240	8	DRB1*04:04	DPAVRSLHNLAHLFL	TRP1	P17643	370	384	23.28
2241	9	DRB1*04:04	FFPLLLFQQARAQFP	TRP1	P17643	13	27	47.72

2242	10	DRB1*04:04	FPLLLFQQARAQFPR	TRP1	P17643	14	28	45.8
2243	11	DRB1*04:04	HPLFVIATRRSEEIL	TRP1	P17643	156	170	26.21
2244	12	DRB1*04:04	KRTTHPLFVIATRRS	TRP1	P17643	152	166	45.16
2245	13	DRB1*04:04	PAVRSLHNLAHLFLN	TRP1	P17643	371	385	25.72
2246	14	DRB1*04:04	PLFVIATRRSEEILG	TRP1	P17643	157	171	46.17
2247	15	DRB1*04:04	RTTHPLFVIATRRSE	TRP1	P17643	153	167	26.14
2248	16	DRB1*04:04	THPLFVIATRRSEEI	TRP1	P17643	155	169	22.73
2249	17	DRB1*04:04	TTHPLFVIATRRSEE	TRP1	P17643	154	168	27.3
2250	18	DRB1*04:04	YDPAVRSLHNLAHLF	TRP1	P17643	369	383	30.79
2251	19	DRB1*04:04	AEVSIVVLSGTTAAQ	PMEL17	P40967	403	417	43.28
2252	20	DRB1*04:04	ANASFSIALNFPGSQ	PMEL17	P40967	80	94	34.62
2253	21	DRB1*04:04	ASFSIALNFPGSQKV	PMEL17	P40967	82	96	31.54
2254	22	DRB1*04:04	EVSIVVLSGTTAAQV	PMEL17	P40967	404	418	27.59
2255	23	DRB1*04:04	GANASFSIALNFPGS	PMEL17	P40967	79	93	40.38
2256	24	DRB1*04:04	NASFSIALNFPGSQK	PMEL17	P40967	81	95	31.52
2257	25	DRB1*04:04	SIVVLSGTTAAQVTT	PMEL17	P40967	406	420	34.56
2258	26	DRB1*04:04	VSIVVLSGTTAAQVT	PMEL17	P40967	405	419	25.56
2259	1	DRB1*04:05	FVWMHYYVSMDALLG	Tyrosinase	P14679	176	190	38
2260	2	DRB1*04:05	MHYYVSMDALLGGSE	Tyrosinase	P14679	179	193	45.55
2261	3	DRB1*04:05	VWMHYYVSMDALLGG	Tyrosinase	P14679	177	191	38.24
2262	4	DRB1*04:05	WMHYYVSMDALLGGS	Tyrosinase	P14679	178	192	40.42
2263	5	DRB1*04:05	DPIFVLLHTFTDAVF	TRP1	P17643	397	411	16.23
2264	6	DRB1*04:05	IFVLLHTFTDAVFDE	TRP1	P17643	399	413	44.63
2265	7	DRB1*04:05	NDPIFVLLHTFTDAV	TRP1	P17643	396	410	15.01
2266	8	DRB1*04:05	PIFVLLHTFTDAVFD	TRP1	P17643	398	412	20.52
2267	9	DRB1*04:05	PNDPIFVLLHTFTDA	TRP1	P17643	395	409	14.77
2268	10	DRB1*04:05	SPNDPIFVLLHTFTD	TRP1	P17643	394	408	19.43
2269	11	DRB1*04:05	ANASFSIALNFPGSQ	PMEL17	P40967	80	94	31.75
2270	12	DRB1*04:05	ASFSIALNFPGSQKV	PMEL17	P40967	82	96	32.99
2271	13	DRB1*04:05	CVLYRYGSFSVTLDI	PMEL17	P40967	475	489	35.84
2272	14	DRB1*04:05	GANASFSIALNFPGS	PMEL17	P40967	79	93	32.67
2273	15	DRB1*04:05	IGANASFSIALNFPG	PMEL17	P40967	78	92	45.54
2274	16	DRB1*04:05	LYRYGSFSVTLDIVQ	PMEL17	P40967	477	491	25.04
2275	17	DRB1*04:05	NASFSIALNFPGSQK	PMEL17	P40967	81	95	31.1
2276	18	DRB1*04:05	VLYRYGSFSVTLDIV	PMEL17	P40967	476	490	26.64
2277	19	DRB1*04:05	YRYGSFSVTLDIVQG	PMEL17	P40967	478	492	35.35
2278	1	DRB1*04:08	EFMAFVAMVTTACHE	S100	P04271	71	85	46.13
2279	2	DRB1*04:08	FMAFVAMVTTACHEF	S100	P04271	72	86	40.92

2280	3	DRB1*04:08	MAFVAMVTTACHEFF	S100	P04271	73	87	38.29
2281	4	DRB1*04:08	FVWMHYYVSMDALLG	Tyrosinase	P14679	176	190	43.64
2282	5	DRB1*04:08	HYYVSMDALLGGSEI	Tyrosinase	P14679	180	194	36.79
2283	6	DRB1*04:08	MHYYVSMDALLGGSE	Tyrosinase	P14679	179	193	29.29
2284	7	DRB1*04:08	VWMHYYVSMDALLGG	Tyrosinase	P14679	177	191	29.69
2285	8	DRB1*04:08	WMHYYVSMDALLGGS	Tyrosinase	P14679	178	192	26.42
2286	9	DRB1*04:08	DPIFVLLHTFTDAVF	TRP1	P17643	397	411	32.47
2287	10	DRB1*04:08	EMFVTAPDNLGYTYE	TRP1	P17643	451	465	25.27
2288	11	DRB1*04:08	MFVTAPDNLGYTYEI	TRP1	P17643	452	466	36.69
2289	12	DRB1*04:08	NDPIFVLLHTFTDAV	TRP1	P17643	396	410	36.48
2290	13	DRB1*04:08	NTEMFVTAPDNLGYT	TRP1	P17643	449	463	30.41
2291	14	DRB1*04:08	PIFVLLHTFTDAVFD	TRP1	P17643	398	412	38.22
2292	15	DRB1*04:08	PNDPIFVLLHTFTDA	TRP1	P17643	395	409	47.45
2293	16	DRB1*04:08	TEMFVTAPDNLGYTY	TRP1	P17643	450	464	24.17
2294	17	DRB1*04:08	TNTEMFVTAPDNLGY	TRP1	P17643	448	462	35.8
2295	18	DRB1*04:08	ANASFSIALNFPGSQ	PMEL17	P40967	80	94	16.92
2296	19	DRB1*04:08	ASFSIALNFPGSQKV	PMEL17	P40967	82	96	16.27
2297	20	DRB1*04:08	GANASFSIALNFPGS	PMEL17	P40967	79	93	19.98
2298	21	DRB1*04:08	IGANASFSIALNFPG	PMEL17	P40967	78	92	43.75
2299	22	DRB1*04:08	NASFSIALNFPGSQK	PMEL17	P40967	81	95	15.55
2300	23	DRB1*04:08	SFSIALNFPGSQKVL	PMEL17	P40967	83	97	28.57
2301	24	DRB1*04:08	VSIVVLSGTTAAQVT	PMEL17	P40967	405	419	45.64
2302	25	DRB1*04:08	KEFTVSGNILTIRLT	NY-ESO-1	P78358	124	138	34.63
2303	26	DRB1*04:08	LKEFTVSGNILTIRL	NY-ESO-1	P78358	123	137	24.55
2304	27	DRB1*04:08	LLKEFTVSGNILTIR	NY-ESO-1	P78358	122	136	26
2305	28	DRB1*04:08	VLLKEFTVSGNILTI	NY-ESO-1	P78358	121	135	43.81
2306	29	DRB1*04:08	RAVFLALSAQLLQAR	BAGE	Q13072	4	18	49.46
2307	1	DRB1*04:10	EKAMVALIDVFHQYS	S100	P04271	3	17	39.03
2308	2	DRB1*04:10	KAMVALIDVFHQYSG	S100	P04271	4	18	48.8
2309	3	DRB1*04:10	LEKAMVALIDVFHQY	S100	P04271	2	16	42.14
2310	4	DRB1*04:10	DPAVRSLHNLAHLFL	TRP1	P17643	370	384	41.97
2311	5	DRB1*04:10	DPIFVLLHTFTDAVF	TRP1	P17643	397	411	43.85
2312	6	DRB1*04:10	NDPIFVLLHTFTDAV	TRP1	P17643	396	410	46.41
2313	7	DRB1*04:10	PAVRSLHNLAHLFLN	TRP1	P17643	371	385	44.46
2314	8	DRB1*04:10	PIFVLLHTFTDAVFD	TRP1	P17643	398	412	49.8
2315	1	DRB1*04:44	VSIVVLSGTTAAQVT	PMEL17	P40967	405	419	47.75
2316	1	DRB1*04:72	TEMFVTAPDNLGYTY	TRP1	P17643	450	464	43.32

2317	2	DRB1*04:72	ASFSIALNFPGSQKV	PMEL17	P40967	82	96	48.49
2318	3	DRB1*04:72	NASFSIALNFPGSQK	PMEL17	P40967	81	95	45.1
2319	4	DRB1*04:72	KEFTVSGNILTIRLT	NY-ESO-1	P78358	124	138	41
2320	5	DRB1*04:72	LKEFTVSGNILTIRL	NY-ESO-1	P78358	123	137	30.17
2321	6	DRB1*04:72	LLKEFTVSGNILTIR	NY-ESO-1	P78358	122	136	32.69
2322	1	DRB1*07:01	KVYYYRFVIGLRVWQ	TRP2	O75767	206	220	12.59
2323	2	DRB1*07:01	LKVYYYRFVIGLRVW	TRP2	O75767	205	219	15.43
2324	3	DRB1*07:01	VYYYRFVIGLRVWQW	TRP2	O75767	207	221	17.44
2325	4	DRB1*07:01	WLKVYYYRFVIGLRV	TRP2	O75767	204	218	9.46
2326	5	DRB1*07:01	YYRFVIGLRVWQWEV	TRP2	O75767	209	223	21.28
2327	6	DRB1*07:01	YYYRFVIGLRVWQWE	TRP2	O75767	208	222	15.65
2328	7	DRB1*07:01	DPIFLLHHAFVDSIF	Tyrosinase	P14679	383	397	39.65
2329	8	DRB1*07:01	NDPIFLLHHAFVDSI	Tyrosinase	P14679	382	396	46.64
2330	9	DRB1*07:01	PIFLLHHAFVDSIFE	Tyrosinase	P14679	384	398	41.9
2331	10	DRB1*07:01	ALIFGTASYLIRARR	TRP1	P17643	491	505	9.91
2332	11	DRB1*07:01	ISIYNYFVWTHYYSV	TRP1	P17643	182	196	39.49
2333	12	DRB1*07:01	IYNYFVWTHYYSVKK	TRP1	P17643	184	198	20.23
2334	13	DRB1*07:01	LIFGTASYLIRARRS	TRP1	P17643	492	506	13.71
2335	14	DRB1*07:01	LLLVALIFGTASYLI	TRP1	P17643	487	501	28.35
2336	15	DRB1*07:01	LLVALIFGTASYLIR	TRP1	P17643	488	502	15.71
2337	16	DRB1*07:01	LVALIFGTASYLIRA	TRP1	P17643	489	503	15.15
2338	17	DRB1*07:01	NYFVWTHYYSVKKTF	TRP1	P17643	186	200	28.79
2339	18	DRB1*07:01	SIYNYFVWTHYYSVK	TRP1	P17643	183	197	25.38
2340	19	DRB1*07:01	VALIFGTASYLIRAR	TRP1	P17643	490	504	10.07
2341	20	DRB1*07:01	YFVWTHYYSVKKTFL	TRP1	P17643	187	201	29.71
2342	21	DRB1*07:01	YNYFVWTHYYSVKKT	TRP1	P17643	185	199	21.34
2343	22	DRB1*07:01	ALLAVGATKVPRNQD	PMEL17	P40967	17	31	43.16
2344	23	DRB1*07:01	AVIGALLAVGATKVP	PMEL17	P40967	13	27	19.4
2345	24	DRB1*07:01	DSSGTLISRALVVTH	PMEL17	P40967	264	278	24.3
2346	25	DRB1*07:01	EVSIVVLSGTTAAQV	PMEL17	P40967	404	418	35.86
2347	26	DRB1*07:01	FGDSSGTLISRALVV	PMEL17	P40967	262	276	27.55
2348	27	DRB1*07:01	GALLAVGATKVPRNQ	PMEL17	P40967	16	30	28.16
2349	28	DRB1*07:01	GDSSGTLISRALVVT	PMEL17	P40967	263	277	25.03
2350	29	DRB1*07:01	GGNKHFLRNQPLTFA	PMEL17	P40967	227	241	49.96
2351	30	DRB1*07:01	GNKHFLRNQPLTFAL	PMEL17	P40967	228	242	38.73
2352	31	DRB1*07:01	GPLLDGTATLRLVKR	PMEL17	P40967	455	469	37.34
2353	32	DRB1*07:01	GRAMLGTHTMEVTVY	PMEL17	P40967	175	189	38.21
2354	33	DRB1*07:01	IGALLAVGATKVPRN	PMEL17	P40967	15	29	18.72
2355	34	DRB1*07:01	IVVLSGTTAAQVTTT	PMEL17	P40967	407	421	33.68

2356	35	DRB1*07:01	KTWGQYWQVLGGPVS	PMEL17	P40967	154	168	42.41
2357	36	DRB1*07:01	LAVIGALLAVGATKV	PMEL17	P40967	12	26	20.25
2358	37	DRB1*07:01	LGPLLDGTATLRLVK	PMEL17	P40967	454	468	43.03
2359	38	DRB1*07:01	NKHFLRNQPLTFALQ	PMEL17	P40967	229	243	41.14
2360	39	DRB1*07:01	PLLDGTATLRLVKRQ	PMEL17	P40967	456	470	46.94
2361	40	DRB1*07:01	QVTTTEWVETTAREL	PMEL17	P40967	417	431	47.62
2362	41	DRB1*07:01	RAMLGTHTMEVTVYH	PMEL17	P40967	176	190	43.56
2363	42	DRB1*07:01	RSYVPLAHSSSAFTI	PMEL17	P40967	195	209	42.27
2364	43	DRB1*07:01	SGTLISRALVVTHTY	PMEL17	P40967	266	280	33.04
2365	44	DRB1*07:01	SIVVLSGTTAAQVTT	PMEL17	P40967	406	420	30.64
2366	45	DRB1*07:01	SSGTLISRALVVTHT	PMEL17	P40967	265	279	26.64
2367	46	DRB1*07:01	SYVPLAHSSSAFTIT	PMEL17	P40967	196	210	38.1
2368	47	DRB1*07:01	TGRAMLGTHTMEVTV	PMEL17	P40967	174	188	45.41
2369	48	DRB1*07:01	TTEWVETTARELPIP	PMEL17	P40967	420	434	49.19
2370	49	DRB1*07:01	TTTEWVETTARELPI	PMEL17	P40967	419	433	40.29
2371	50	DRB1*07:01	VIGALLAVGATKVPR	PMEL17	P40967	14	28	16.71
2372	51	DRB1*07:01	VSIVVLSGTTAAQVT	PMEL17	P40967	405	419	29.66
2373	52	DRB1*07:01	WGQYWQVLGGPVSGL	PMEL17	P40967	156	170	40.66
2374	53	DRB1*07:01	YVPLAHSSSAFTITD	PMEL17	P40967	197	211	48.73
2375	54	DRB1*07:01	CILESLFRAVITKKV	MAGE1	P43355	92	106	7.59
2376	55	DRB1*07:01	ESLFRAVITKKVADL	MAGE1	P43355	95	109	8.52
2377	56	DRB1*07:01	EYVIKVSARVRFFFP	MAGE1	P43355	281	295	43.67
2378	57	DRB1*07:01	ILESLFRAVITKKVA	MAGE1	P43355	93	107	7.63
2379	58	DRB1*07:01	KVLEYVIKVSARVRF	MAGE1	P43355	278	292	38.8
2380	59	DRB1*07:01	LESLFRAVITKKVAD	MAGE1	P43355	94	108	9.95
2381	60	DRB1*07:01	LEYVIKVSARVRFFF	MAGE1	P43355	280	294	36.39
2382	61	DRB1*07:01	LFRAVITKKVADLVG	MAGE1	P43355	97	111	13.1
2383	62	DRB1*07:01	SLFRAVITKKVADLV	MAGE1	P43355	96	110	8.83
2384	63	DRB1*07:01	VKVLEYVIKVSARVR	MAGE1	P43355	277	291	44.91
2385	64	DRB1*07:01	VLEYVIKVSARVRFF	MAGE1	P43355	279	293	38.63
2386	65	DRB1*07:01	AESLFREALSNKVDE	MAGE4	P43358	102	116	9.86
2387	66	DRB1*07:01	DAESLFREALSNKVD	MAGE4	P43358	101	115	7.47
2388	67	DRB1*07:01	ESLFREALSNKVDEL	MAGE4	P43358	103	117	7.59
2389	68	DRB1*07:01	LFREALSNKVDELAH	MAGE4	P43358	105	119	10.99
2390	69	DRB1*07:01	PDAESLFREALSNKV	MAGE4	P43358	100	114	6.24
2391	70	DRB1*07:01	SESLKMIFGIDVKEV	MAGE4	P43358	156	170	44.46
2392	71	DRB1*07:01	SLFREALSNKVDELA	MAGE4	P43358	104	118	7.45
2393	72	DRB1*07:01	AADHRQLQLSISSCL	NY-ESO-1	P78358	139	153	21.95
2394	73	DRB1*07:01	ADHRQLQLSISSCLQ	NY-ESO-1	P78358	140	154	15.82
2395	74	DRB1*07:01	DHRQLQLSISSCLQQ	NY-ESO-1	P78358	141	155	14.17

2396	75	DRB1*07:01	GVLLKEFTVSGNILT	NY-ESO-1	P78358	120	134	38.5
2397	76	DRB1*07:01	HRQLQLSISSCLQQL	NY-ESO-1	P78358	142	156	13.69
2398	77	DRB1*07:01	KEFTVSGNILTIRLT	NY-ESO-1	P78358	124	138	33.96
2399	78	DRB1*07:01	LKEFTVSGNILTIRL	NY-ESO-1	P78358	123	137	27.27
2400	79	DRB1*07:01	LLEFYLAMPFATPME	NY-ESO-1	P78358	87	101	49.15
2401	80	DRB1*07:01	LLKEFTVSGNILTIR	NY-ESO-1	P78358	122	136	25.13
2402	81	DRB1*07:01	RQLQLSISSCLQQLS	NY-ESO-1	P78358	143	157	21.18
2403	82	DRB1*07:01	SRLLEFYLAMPFATP	NY-ESO-1	P78358	85	99	45.61
2404	83	DRB1*07:01	VLLKEFTVSGNILTI	NY-ESO-1	P78358	121	135	31.61
2405	84	DRB1*07:01	AARAVFLALSAQLLQ	BAGE	Q13072	2	16	19.97
2406	85	DRB1*07:01	ARAVFLALSAQLLQA	BAGE	Q13072	3	17	19.51
2407	86	DRB1*07:01	AVFLALSAQLLQARL	BAGE	Q13072	5	19	31.6
2408	87	DRB1*07:01	MAARAVFLALSAQLL	BAGE	Q13072	1	15	21.55
2409	88	DRB1*07:01	RAVFLALSAQLLQAR	BAGE	Q13072	4	18	16.48
2410	89	DRB1*07:01	VFLALSAQLLQARLM	BAGE	Q13072	6	20	42.78
2411	90	DRB1*07:01	AMTKLGFKATLPPFM	SSX2	Q16385	57	71	14.22
2412	91	DRB1*07:01	GFKATLPPFMCNKRA	SSX2	Q16385	62	76	46.32
2413	92	DRB1*07:01	KLGFKATLPPFMCNK	SSX2	Q16385	60	74	12.37
2414	93	DRB1*07:01	LGFKATLPPFMCNKR	SSX2	Q16385	61	75	21.75
2415	94	DRB1*07:01	MTKLGFKATLPPFMC	SSX2	Q16385	58	72	15.13
2416	95	DRB1*07:01	TKLGFKATLPPFMCN	SSX2	Q16385	59	73	13.87
2417	1	DRB1*08:01	MVPFIPLYRNGDFFI	Tyrosinase	P14679	426	440	42.22
2418	2	DRB1*08:01	VPFIPLYRNGDFFIS	Tyrosinase	P14679	427	441	48.28
2419	3	DRB1*08:01	HPLFVIATRRSEEIL	TRP1	P17643	156	170	30.11
2420	4	DRB1*08:01	LFVIATRRSEEILGP	TRP1	P17643	158	172	45.4
2421	5	DRB1*08:01	PLFVIATRRSEEILG	TRP1	P17643	157	171	32.59
2422	6	DRB1*08:01	RTTHPLFVIATRRSE	TRP1	P17643	153	167	42.4
2423	7	DRB1*08:01	TASYLIRARRSMDEA	TRP1	P17643	496	510	48.32
2424	8	DRB1*08:01	THPLFVIATRRSEEI	TRP1	P17643	155	169	35.14
2425	9	DRB1*08:01	TTHPLFVIATRRSEE	TRP1	P17643	154	168	43.73
2426	10	DRB1*08:01	EKIFYVYMKRKYEAM	SSX2	Q16385	44	58	37.24
2427	11	DRB1*08:01	IFYVYMKRKYEAMTK	SSX2	Q16385	46	60	40.75
2428	12	DRB1*08:01	KIFYVYMKRKYEAMT	SSX2	Q16385	45	59	37.96
2429	13	DRB1*08:01	SEKIFYVYMKRKYEA	SSX2	Q16385	43	57	45.25
2430	1	DRB1*08:02	HPLFVIATRRSEEIL	TRP1	P17643	156	170	46.3
2431	2	DRB1*08:02	THPLFVIATRRSEEI	TRP1	P17643	155	169	45.51
2432	3	DRB1*08:02	KVLEYVIKVSARVRF	MAGE1	P43355	278	292	44.67

2433	1	DRB1*08:04	EVISCKLIKRATTRQ	TRP2	O75767	222	236	36.35
2434	2	DRB1*08:04	VISCKLIKRATTRQP	TRP2	O75767	223	237	38.29
2435	3	DRB1*08:04	WEVISCKLIKRATTR	TRP2	O75767	221	235	38.83
2436	4	DRB1*08:04	FAYLTLAKHTISSDY	Tyrosinase	P14679	135	149	38.34
2437	5	DRB1*08:04	FFAYLTLAKHTISSD	Tyrosinase	P14679	134	148	46.68
2438	6	DRB1*08:04	KFFAYLTLAKHTISS	Tyrosinase	P14679	133	147	45.91
2439	7	DRB1*08:04	CDQRVLIVRRNLLDL	TRP1	P17643	122	136	49.14
2440	8	DRB1*08:04	DQRVLIVRRNLLDLS	TRP1	P17643	123	137	44.74
2441	9	DRB1*08:04	FPLLLFQQARAQFPR	TRP1	P17643	14	28	49.27
2442	10	DRB1*08:04	HPLFVIATRRSEEIL	TRP1	P17643	156	170	25.99
2443	11	DRB1*08:04	PLFVIATRRSEEILG	TRP1	P17643	157	171	36.44
2444	12	DRB1*08:04	QRVLIVRRNLLDLSK	TRP1	P17643	124	138	39.07
2445	13	DRB1*08:04	RTTHPLFVIATRRSE	TRP1	P17643	153	167	30.55
2446	14	DRB1*08:04	THPLFVIATRRSEEI	TRP1	P17643	155	169	24.66
2447	15	DRB1*08:04	TTHPLFVIATRRSEE	TRP1	P17643	154	168	29.7
2448	16	DRB1*08:04	ACQLVLHQILKGGSG	PMEL17	P40967	549	563	49.22
2449	17	DRB1*08:04	ATLRLVKRQVPLDCV	PMEL17	P40967	462	476	38.56
2450	18	DRB1*08:04	CQLVLHQILKGGSGT	PMEL17	P40967	550	564	43.09
2451	19	DRB1*08:04	DGTATLRLVKRQVPL	PMEL17	P40967	459	473	24.49
2452	20	DRB1*08:04	GTATLRLVKRQVPLD	PMEL17	P40967	460	474	26.63
2453	21	DRB1*08:04	QLVLHQILKGGSGTY	PMEL17	P40967	551	565	37.93
2454	22	DRB1*08:04	TATLRLVKRQVPLDC	PMEL17	P40967	461	475	30.43
2455	23	DRB1*08:04	EYVIKVSARVRFFFP	MAGE1	P43355	281	295	36.25
2456	24	DRB1*08:04	KVLEYVIKVSARVRF	MAGE1	P43355	278	292	28.78
2457	25	DRB1*08:04	LEYVIKVSARVRFFF	MAGE1	P43355	280	294	34.64
2458	26	DRB1*08:04	LVGFLLLKYRAREPV	MAGE1	P43355	109	123	49.14
2459	27	DRB1*08:04	VKVLEYVIKVSARVR	MAGE1	P43355	277	291	37.55
2460	28	DRB1*08:04	VLEYVIKVSARVRFF	MAGE1	P43355	279	293	33.8
2461	29	DRB1*08:04	KVLEHVVRVNARVRI	MAGE4	P43358	286	300	34.75
2462	30	DRB1*08:04	LEHVVRVNARVRIAY	MAGE4	P43358	288	302	47.93
2463	31	DRB1*08:04	VKVLEHVVRVNARVR	MAGE4	P43358	285	299	34.31
2464	32	DRB1*08:04	VLEHVVRVNARVRIA	MAGE4	P43358	287	301	43.42
2465	33	DRB1*08:04	YVKVLEHVVRVNARV	MAGE4	P43358	284	298	44.75
2466	34	DRB1*08:04	EKIFYVYMKRKYEAM	SSX2	Q16385	44	58	45.57
2467	35	DRB1*08:04	IFYVYMKRKYEAMTK	SSX2	Q16385	46	60	43.16
2468	36	DRB1*08:04	KIFYVYMKRKYEAMT	SSX2	Q16385	45	59	43.83
2469	1	DRB1*08:24	HPLFVIATRRSEEIL	TRP1	P17643	156	170	39.93
2470	2	DRB1*08:24	THPLFVIATRRSEEI	TRP1	P17643	155	169	40.1
2471	3	DRB1*08:24	TTHPLFVIATRRSEE	TRP1	P17643	154	168	48.89

2472	4	DRB1*08:24	RVRFFFPSLREAALR	MAGE1	P43355	289	303	47.68
2473	5	DRB1*08:24	VRFFFPSLREAALRE	MAGE1	P43355	290	304	48.96
2474	6	DRB1*08:24	EKIFYVYMKRKYEAM	SSX2	Q16385	44	58	43.31
2475	7	DRB1*08:24	KIFYVYMKRKYEAMT	SSX2	Q16385	45	59	42.23
2476	1	DRB1*08:30	KVLEYVIKVSARVRF	MAGE1	P43355	278	292	44.36
2477	2	DRB1*08:30	LEYVIKVSARVRFFF	MAGE1	P43355	280	294	44.27
2478	3	DRB1*08:30	VLEYVIKVSARVRFF	MAGE1	P43355	279	293	40.81
2479	4	DRB1*08:30	RAVFLALSAQLLQAR	BAGE	Q13072	4	18	44.79
2480	1	DRB1*09:01	DPIFLLHHAFVDSIF	Tyrosinase	P14679	383	397	45.8
2481	2	DRB1*09:01	PIFLLHHAFVDSIFE	Tyrosinase	P14679	384	398	47.97
2482	3	DRB1*09:01	ALIFGTASYLIRARR	TRP1	P17643	491	505	43.03
2483	4	DRB1*09:01	FPLLLFQQARAQFPR	TRP1	P17643	14	28	44.22
2484	5	DRB1*09:01	FSLPYWNFATGKNVC	TRP1	P17643	244	258	45.1
2485	6	DRB1*09:01	LPYWNFATGKNVCDI	TRP1	P17643	246	260	43.46
2486	7	DRB1*09:01	PLLLFQQARAQFPRQ	TRP1	P17643	15	29	42.18
2487	8	DRB1*09:01	SFSLPYWNFATGKNV	TRP1	P17643	243	257	37.71
2488	9	DRB1*09:01	VALIFGTASYLIRAR	TRP1	P17643	490	504	38.61
2489	10	DRB1*09:01	ANASFSIALNFPGSQ	PMEL17	P40967	80	94	44.33
2490	11	DRB1*09:01	ASFSIALNFPGSQKV	PMEL17	P40967	82	96	49.67
2491	12	DRB1*09:01	GANASFSIALNFPGS	PMEL17	P40967	79	93	42.92
2492	13	DRB1*09:01	IGANASFSIALNFPG	PMEL17	P40967	78	92	48.64
2493	14	DRB1*09:01	NASFSIALNFPGSQK	PMEL17	P40967	81	95	42.97
2494	15	DRB1*09:01	RSYVPLAHSSSAFTI	PMEL17	P40967	195	209	41.07
2495	16	DRB1*09:01	SYVPLAHSSSAFTIT	PMEL17	P40967	196	210	35.62
2496	17	DRB1*09:01	WGQYWQVLGGPVSGL	PMEL17	P40967	156	170	48.18
2497	18	DRB1*09:01	YVPLAHSSSAFTITD	PMEL17	P40967	197	211	39.68
2498	19	DRB1*09:01	CILESLFRAVITKKV	MAGE1	P43355	92	106	22.14
2499	20	DRB1*09:01	ESLFRAVITKKVADL	MAGE1	P43355	95	109	21.43
2500	21	DRB1*09:01	ILESLFRAVITKKVA	MAGE1	P43355	93	107	20.12
2501	22	DRB1*09:01	LESLFRAVITKKVAD	MAGE1	P43355	94	108	24.59
2502	23	DRB1*09:01	LFRAVITKKVADLVG	MAGE1	P43355	97	111	35.07
2503	24	DRB1*09:01	SLFRAVITKKVADLV	MAGE1	P43355	96	110	22.79
2504	25	DRB1*09:01	AESLFREALSNKVDE	MAGE4	P43358	102	116	15.34
2505	26	DRB1*09:01	DAESLFREALSNKVD	MAGE4	P43358	101	115	12.29
2506	27	DRB1*09:01	ESLFREALSNKVDEL	MAGE4	P43358	103	117	12.12
2507	28	DRB1*09:01	LFREALSNKVDELAH	MAGE4	P43358	105	119	15.87
2508	29	DRB1*09:01	PDAESLFREALSNKV	MAGE4	P43358	100	114	11.19
2509	30	DRB1*09:01	SLFREALSNKVDELA	MAGE4	P43358	104	118	11.5

2510	31	DRB1*09:01	ADHRQLQLSISSCLQ	NY-ESO-1	P78358	140	154	42.19
2511	32	DRB1*09:01	DHRQLQLSISSCLQQ	NY-ESO-1	P78358	141	155	35.68
2512	33	DRB1*09:01	EFYLAMPFATPMEAE	NY-ESO-1	P78358	89	103	33.24
2513	34	DRB1*09:01	HRQLQLSISSCLQQL	NY-ESO-1	P78358	142	156	36.07
2514	35	DRB1*09:01	LEFYLAMPFATPMEA	NY-ESO-1	P78358	88	102	25.81
2515	36	DRB1*09:01	LLEFYLAMPFATPME	NY-ESO-1	P78358	87	101	28.28
2516	37	DRB1*09:01	RLLEFYLAMPFATPM	NY-ESO-1	P78358	86	100	34.37
2517	38	DRB1*09:01	SRLLEFYLAMPFATP	NY-ESO-1	P78358	85	99	29.41
2518	39	DRB1*09:01	AARAVFLALSAQLLQ	BAGE	Q13072	2	16	43.55
2519	40	DRB1*09:01	ARAVFLALSAQLLQA	BAGE	Q13072	3	17	41.19
2520	41	DRB1*09:01	RAVFLALSAQLLQAR	BAGE	Q13072	4	18	31.22
2521	42	DRB1*09:01	AMTKLGFKATLPPFM	SSX2	Q16385	57	71	24.36
2522	43	DRB1*09:01	KLGFKATLPPFMCNK	SSX2	Q16385	60	74	19.03
2523	44	DRB1*09:01	LGFKATLPPFMCNKR	SSX2	Q16385	61	75	30.82
2524	45	DRB1*09:01	MTKLGFKATLPPFMC	SSX2	Q16385	58	72	24.82
2525	46	DRB1*09:01	TKLGFKATLPPFMCN	SSX2	Q16385	59	73	21.81
2526	47	DRB1*10:01	KVYYYRFVIGLRVWQ	TRP2	O75767	206	220	18.44
2527	1	DRB1*10:01	LKVYYYRFVIGLRVW	TRP2	O75767	205	219	26.89
2528	2	DRB1*10:01	TTQHWVGLLGPNGTQ	TRP2	O75767	159	173	45.93
2529	3	DRB1*10:01	VYYYRFVIGLRVWQW	TRP2	O75767	207	221	24.36
2530	4	DRB1*10:01	WLKVYYYRFVIGLRV	TRP2	O75767	204	218	25.68
2531	5	DRB1*10:01	YYRFVIGLRVWQWEV	TRP2	O75767	209	223	30.56
2532	6	DRB1*10:01	YYYRFVIGLRVWQWE	TRP2	O75767	208	222	21.78
2533	7	DRB1*10:01	AFVAMVTTACHEFFE	S100	P04271	74	88	28.91
2534	8	DRB1*10:01	DFQEFMAFVAMVTTA	S100	P04271	68	82	43.75
2535	9	DRB1*10:01	EFMAFVAMVTTACHE	S100	P04271	71	85	25.83
2536	10	DRB1*10:01	EKAMVALIDVFHQYS	S100	P04271	3	17	41.59
2537	11	DRB1*10:01	FMAFVAMVTTACHEF	S100	P04271	72	86	24.93
2538	12	DRB1*10:01	FQEFMAFVAMVTTAC	S100	P04271	69	83	48.37
2539	13	DRB1*10:01	KAMVALIDVFHQYSG	S100	P04271	4	18	46.68
2540	14	DRB1*10:01	LEKAMVALIDVFHQY	S100	P04271	2	16	46.06
2541	15	DRB1*10:01	MAFVAMVTTACHEFF	S100	P04271	73	87	22.8
2542	16	DRB1*10:01	QEFMAFVAMVTTACH	S100	P04271	70	84	32.16
2543	17	DRB1*10:01	ASFFSSWQIVCSRLE	Tyrosinase	P14679	266	280	49.92
2544	18	DRB1*10:01	ASRIWSWLLGAAMVG	Tyrosinase	P14679	471	485	28.64
2545	19	DRB1*10:01	CLLWSFQTSAGHFPR	Tyrosinase	P14679	8	22	39.46
2546	20	DRB1*10:01	DKFFAYLTLAKHTIS	Tyrosinase	P14679	132	146	8.37
2547	21	DRB1*10:01	DPIFLLHHAFVDSIF	Tyrosinase	P14679	383	397	37.62
2548	22	DRB1*10:01	EKDKFFAYLTLAKHT	Tyrosinase	P14679	130	144	10.21

2549	23	DRB1*10:01	EQASRIWSWLLGAAM	Tyrosinase	P14679	469	483	47.39
2550	24	DRB1*10:01	FAYLTLAKHTISSDY	Tyrosinase	P14679	135	149	44.28
2551	25	DRB1*10:01	FFAYLTLAKHTISSD	Tyrosinase	P14679	134	148	16.85
2552	26	DRB1*10:01	FQDYIKSYLEQASRI	Tyrosinase	P14679	460	474	40.9
2553	27	DRB1*10:01	FVWMHYYVSMDALLG	Tyrosinase	P14679	176	190	12.54
2554	28	DRB1*10:01	HYYVSMDALLGGSEI	Tyrosinase	P14679	180	194	10.58
2555	29	DRB1*10:01	IDFAHEAPAFLPWHR	Tyrosinase	P14679	198	212	43.41
2556	30	DRB1*10:01	KDKFFAYLTLAKHTI	Tyrosinase	P14679	131	145	8.52
2557	31	DRB1*10:01	KFFAYLTLAKHTISS	Tyrosinase	P14679	133	147	9.36
2558	32	DRB1*10:01	LLWSFQTSAGHFPRA	Tyrosinase	P14679	9	23	33.85
2559	33	DRB1*10:01	MHYYVSMDALLGGSE	Tyrosinase	P14679	179	193	9.68
2560	34	DRB1*10:01	MVGAVLTALLAGLVS	Tyrosinase	P14679	483	497	42.59
2561	35	DRB1*10:01	NALHIYMNGTMSQVQ	Tyrosinase	P14679	364	378	48.73
2562	36	DRB1*10:01	NDPIFLLHHAFVDSI	Tyrosinase	P14679	382	396	47.07
2563	37	DRB1*10:01	PEKDKFFAYLTLAKH	Tyrosinase	P14679	129	143	16.55
2564	38	DRB1*10:01	PIFLLHHAFVDSIFE	Tyrosinase	P14679	384	398	39.16
2565	39	DRB1*10:01	QASRIWSWLLGAAMV	Tyrosinase	P14679	470	484	32.83
2566	40	DRB1*10:01	RIWSWLLGAAMVGAV	Tyrosinase	P14679	473	487	36.16
2567	41	DRB1*10:01	SRIWSWLLGAAMVGA	Tyrosinase	P14679	472	486	25.36
2568	42	DRB1*10:01	TALLAGLVSLLCRHK	Tyrosinase	P14679	489	503	48.81
2569	43	DRB1*10:01	VGAVLTALLAGLVSL	Tyrosinase	P14679	484	498	47.38
2570	44	DRB1*10:01	VWMHYYVSMDALLGG	Tyrosinase	P14679	177	191	10.07
2571	45	DRB1*10:01	WMHYYVSMDALLGGS	Tyrosinase	P14679	178	192	9.19
2572	46	DRB1*10:01	YCLLWSFQTSAGHFP	Tyrosinase	P14679	7	21	49.84
2573	47	DRB1*10:01	YVSMDALLGGSEIWR	Tyrosinase	P14679	182	196	42.74
2574	48	DRB1*10:01	YYVSMDALLGGSEIW	Tyrosinase	P14679	181	195	12.51
2575	49	DRB1*10:01	CIFFPLLLFQQARAQ	TRP1	P17643	11	25	46.92
2576	50	DRB1*10:01	DPAVRSLHNLAHLFL	TRP1	P17643	370	384	38.99
2577	51	DRB1*10:01	DPIFVLLHTFTDAVF	TRP1	P17643	397	411	14.96
2578	52	DRB1*10:01	EKNHFVRALDMAKRT	TRP1	P17643	140	154	47.51
2579	53	DRB1*10:01	FFPLLLFQQARAQFP	TRP1	P17643	13	27	25.64
2580	54	DRB1*10:01	FPLLLFQQARAQFPR	TRP1	P17643	14	28	16.3
2581	55	DRB1*10:01	HPLFVIATRRSEEIL	TRP1	P17643	156	170	38.31
2582	56	DRB1*10:01	IFFPLLLFQQARAQF	TRP1	P17643	12	26	31.15
2583	57	DRB1*10:01	IFVLLHTFTDAVFDE	TRP1	P17643	399	413	38.32
2584	58	DRB1*10:01	IYNYFVWTHYYSVKK	TRP1	P17643	184	198	47.37
2585	59	DRB1*10:01	KNHFVRALDMAKRTT	TRP1	P17643	141	155	47.87
2586	60	DRB1*10:01	KRTTHPLFVIATRRS	TRP1	P17643	152	166	49.85
2587	61	DRB1*10:01	LGYTYEIQWPSREFS	TRP1	P17643	460	474	48.44
2588	62	DRB1*10:01	LLFQQARAQFPRQCA	TRP1	P17643	17	31	35.73

2589	63	DRB1*10:01	LLLFQQARAQFPRQC	TRP1	P17643	16	30	20.68
2590	64	DRB1*10:01	LTWHRYHLLRLEKDM	TRP1	P17643	221	235	37.94
2591	65	DRB1*10:01	NDPIFVLLHTFTDAV	TRP1	P17643	396	410	14.63
2592	66	DRB1*10:01	PAVRSLHNLAHLFLN	TRP1	P17643	371	385	37
2593	67	DRB1*10:01	PIFVLLHTFTDAVFD	TRP1	P17643	398	412	19.59
2594	68	DRB1*10:01	PLLLFQQARAQFPRQ	TRP1	P17643	15	29	14.83
2595	69	DRB1*10:01	PNDPIFVLLHTFTDA	TRP1	P17643	395	409	16.67
2596	70	DRB1*10:01	RSNFDSTLISPNSVF	TRP1	P17643	269	283	30.07
2597	71	DRB1*10:01	RTTHPLFVIATRRSE	TRP1	P17643	153	167	37.76
2598	72	DRB1*10:01	SPNDPIFVLLHTFTD	TRP1	P17643	394	408	32.93
2599	73	DRB1*10:01	SRSNFDSTLISPNSV	TRP1	P17643	268	282	44.7
2600	74	DRB1*10:01	THPLFVIATRRSEEI	TRP1	P17643	155	169	34.07
2601	75	DRB1*10:01	TTHPLFVIATRRSEE	TRP1	P17643	154	168	39.82
2602	76	DRB1*10:01	TWHRYHLLRLEKDMQ	TRP1	P17643	222	236	34.6
2603	77	DRB1*10:01	VALIFGTASYLIRAR	TRP1	P17643	490	504	48.32
2604	78	DRB1*10:01	WHRYHLLRLEKDMQE	TRP1	P17643	223	237	42.51
2605	79	DRB1*10:01	YNYFVWTHYYSVKKT	TRP1	P17643	185	199	49.68
2606	80	DRB1*10:01	AEVSIVVLSGTTAAQ	PMEL17	P40967	403	417	47.54
2607	81	DRB1*10:01	ANASFSIALNFPGSQ	PMEL17	P40967	80	94	12.86
2608	82	DRB1*10:01	AQVVLQAAIPLTSCG	PMEL17	P40967	288	302	21.34
2609	83	DRB1*10:01	ASFSIALNFPGSQKV	PMEL17	P40967	82	96	12.27
2610	84	DRB1*10:01	AVIGALLAVGATKVP	PMEL17	P40967	13	27	28.81
2611	85	DRB1*10:01	EVSIVVLSGTTAAQV	PMEL17	P40967	404	418	39.05
2612	86	DRB1*10:01	GANASFSIALNFPGS	PMEL17	P40967	79	93	15.26
2613	87	DRB1*10:01	GNKHFLRNQPLTFAL	PMEL17	P40967	228	242	41.14
2614	88	DRB1*10:01	GPTLIGANASFSIAL	PMEL17	P40967	74	88	40.93
2615	89	DRB1*10:01	GPVTAQVVLQAAIPL	PMEL17	P40967	284	298	46.25
2616	90	DRB1*10:01	GQYWQVLGGPVSGLS	PMEL17	P40967	157	171	21.64
2617	91	DRB1*10:01	GSRSYVPLAHSSSAF	PMEL17	P40967	193	207	31.36
2618	92	DRB1*10:01	GTLISRALVVTHTYL	PMEL17	P40967	267	281	41.51
2619	93	DRB1*10:01	HLAVIGALLAVGATK	PMEL17	P40967	11	25	31.61
2620	94	DRB1*10:01	IGALLAVGATKVPRN	PMEL17	P40967	15	29	43.24
2621	95	DRB1*10:01	IGANASFSIALNFPG	PMEL17	P40967	78	92	29.88
2622	96	DRB1*10:01	KHFLRNQPLTFALQL	PMEL17	P40967	230	244	35.42
2623	97	DRB1*10:01	KTWGQYWQVLGGPVS	PMEL17	P40967	154	168	40.32
2624	98	DRB1*10:01	LAVIGALLAVGATKV	PMEL17	P40967	12	26	33.21
2625	99	DRB1*10:01	LYRYGSFSVTLDIVQ	PMEL17	P40967	477	491	39.58
2626	100	DRB1*10:01	NASFSIALNFPGSQK	PMEL17	P40967	81	95	11.5
2627	101	DRB1*10:01	NKHFLRNQPLTFALQ	PMEL17	P40967	229	243	35.72
2628	102	DRB1*10:01	PTLIGANASFSIALN	PMEL17	P40967	75	89	47.77

2629	103	DRB1*10:01	PVTAQVVLQAAIPLT	PMEL17	P40967	285	299	37.7
2630	104	DRB1*10:01	QVVLQAAIPLTSCGS	PMEL17	P40967	289	303	20.57
2631	105	DRB1*10:01	QYWQVLGGPVSGLSI	PMEL17	P40967	158	172	26.21
2632	106	DRB1*10:01	RGSRSYVPLAHSSSA	PMEL17	P40967	192	206	42.04
2633	107	DRB1*10:01	RSYVPLAHSSSAFTI	PMEL17	P40967	195	209	27.52
2634	108	DRB1*10:01	SFSIALNFPGSQKVL	PMEL17	P40967	83	97	18.44
2635	109	DRB1*10:01	SGTLISRALVVTHTY	PMEL17	P40967	266	280	46.65
2636	110	DRB1*10:01	SIVVLSGTTAAQVTT	PMEL17	P40967	406	420	44.05
2637	111	DRB1*10:01	SRSYVPLAHSSSAFT	PMEL17	P40967	194	208	30.43
2638	112	DRB1*10:01	SYVPLAHSSSAFTIT	PMEL17	P40967	196	210	33.12
2639	113	DRB1*10:01	TAQVVLQAAIPLTSC	PMEL17	P40967	287	301	21.99
2640	114	DRB1*10:01	TWGQYWQVLGGPVSG	PMEL17	P40967	155	169	28.49
2641	115	DRB1*10:01	VIGALLAVGATKVPR	PMEL17	P40967	14	28	30.6
2642	116	DRB1*10:01	VLYRYGSFSVTLDIV	PMEL17	P40967	476	490	39.11
2643	117	DRB1*10:01	VSIVVLSGTTAAQVT	PMEL17	P40967	405	419	40.57
2644	118	DRB1*10:01	VTAQVVLQAAIPLTS	PMEL17	P40967	286	300	22.84
2645	119	DRB1*10:01	VVLQAAIPLTSCGSS	PMEL17	P40967	290	304	36.16
2646	120	DRB1*10:01	WGQYWQVLGGPVSGL	PMEL17	P40967	156	170	20.64
2647	121	DRB1*10:01	ALGLVCVQAATSSSS	MAGE1	P43355	22	36	39.53
2648	122	DRB1*10:01	ARVRFFFPSLREAAL	MAGE1	P43355	288	302	30.32
2649	123	DRB1*10:01	ARYEFLWGPRALAET	MAGE1	P43355	260	274	18.69
2650	124	DRB1*10:01	CILESLFRAVITKKV	MAGE1	P43355	92	106	28.2
2651	125	DRB1*10:01	DPARYEFLWGPRALA	MAGE1	P43355	258	272	18.33
2652	126	DRB1*10:01	EALGLVCVQAATSSS	MAGE1	P43355	21	35	41.78
2653	127	DRB1*10:01	ESLFRAVITKKVADL	MAGE1	P43355	95	109	13.58
2654	128	DRB1*10:01	ILESLFRAVITKKVA	MAGE1	P43355	93	107	15.8
2655	129	DRB1*10:01	KVLEYVIKVSARVRF	MAGE1	P43355	278	292	40.75
2656	130	DRB1*10:01	LESLFRAVITKKVAD	MAGE1	P43355	94	108	16.11
2657	131	DRB1*10:01	LEYVIKVSARVRFFF	MAGE1	P43355	280	294	44.71
2658	132	DRB1*10:01	LFRAVITKKVADLVG	MAGE1	P43355	97	111	25.87
2659	133	DRB1*10:01	LGLVCVQAATSSSSP	MAGE1	P43355	23	37	45.92
2660	134	DRB1*10:01	PARYEFLWGPRALAE	MAGE1	P43355	259	273	19.45
2661	135	DRB1*10:01	QEALGLVCVQAATSS	MAGE1	P43355	20	34	47.19
2662	136	DRB1*10:01	RVRFFFPSLREAALR	MAGE1	P43355	289	303	28.34
2663	137	DRB1*10:01	RYEFLWGPRALAETS	MAGE1	P43355	261	275	26.6
2664	138	DRB1*10:01	SARVRFFFPSLREAA	MAGE1	P43355	287	301	37.62
2665	139	DRB1*10:01	SDPARYEFLWGPRAL	MAGE1	P43355	257	271	24.12
2666	140	DRB1*10:01	SLFRAVITKKVADLV	MAGE1	P43355	96	110	15.67
2667	141	DRB1*10:01	VKVLEYVIKVSARVR	MAGE1	P43355	277	291	46.73
2668	142	DRB1*10:01	VLEYVIKVSARVRFF	MAGE1	P43355	279	293	43.12

2669	143	DRB1*10:01	VRFFFPSLREAALRE	MAGE1	P43355	290	304	37.99
2670	144	DRB1*10:01	AESLFREALSNKVDE	MAGE4	P43358	102	116	17.56
2671	145	DRB1*10:01	ARYEFLWGPRALAET	MAGE4	P43358	268	282	18.69
2672	146	DRB1*10:01	DAESLFREALSNKVD	MAGE4	P43358	101	115	17.97
2673	147	DRB1*10:01	ESLFREALSNKVDEL	MAGE4	P43358	103	117	13.38
2674	148	DRB1*10:01	LFREALSNKVDELAH	MAGE4	P43358	105	119	25.01
2675	149	DRB1*10:01	NPARYEFLWGPRALA	MAGE4	P43358	266	280	18.33
2676	150	DRB1*10:01	PARYEFLWGPRALAE	MAGE4	P43358	267	281	19.45
2677	151	DRB1*10:01	PDAESLFREALSNKV	MAGE4	P43358	100	114	28.06
2678	152	DRB1*10:01	RYEFLWGPRALAETS	MAGE4	P43358	269	283	26.6
2679	153	DRB1*10:01	SESLKMIFGIDVKEV	MAGE4	P43358	156	170	47.27
2680	154	DRB1*10:01	SLFREALSNKVDELA	MAGE4	P43358	104	118	14.76
2681	155	DRB1*10:01	SNPARYEFLWGPRAL	MAGE4	P43358	265	279	24.12
2682	156	DRB1*10:01	CFLPVFLAQPPSGQR	NY-ESO-1	P78358	165	179	31.83
2683	157	DRB1*10:01	EFYLAMPFATPMEAE	NY-ESO-1	P78358	89	103	10.91
2684	158	DRB1*10:01	ESRLLEFYLAMPFAT	NY-ESO-1	P78358	84	98	26.61
2685	159	DRB1*10:01	FLPVFLAQPPSGQRR	NY-ESO-1	P78358	166	180	25.38
2686	160	DRB1*10:01	FYLAMPFATPMEAEL	NY-ESO-1	P78358	90	104	33.15
2687	161	DRB1*10:01	KEFTVSGNILTIRLT	NY-ESO-1	P78358	124	138	32.78
2688	162	DRB1*10:01	LEFYLAMPFATPMEA	NY-ESO-1	P78358	88	102	8
2689	163	DRB1*10:01	LKEFTVSGNILTIRL	NY-ESO-1	P78358	123	137	27.04
2690	164	DRB1*10:01	LLEFYLAMPFATPME	NY-ESO-1	P78358	87	101	7.81
2691	165	DRB1*10:01	LLKEFTVSGNILTIR	NY-ESO-1	P78358	122	136	28.93
2692	166	DRB1*10:01	QCFLPVFLAQPPSGQ	NY-ESO-1	P78358	164	178	46.29
2693	167	DRB1*10:01	RLLEFYLAMPFATPM	NY-ESO-1	P78358	86	100	9.31
2694	168	DRB1*10:01	SRLLEFYLAMPFATP	NY-ESO-1	P78358	85	99	10.66
2695	169	DRB1*10:01	VLLKEFTVSGNILTI	NY-ESO-1	P78358	121	135	46.32
2696	170	DRB1*10:01	AARAVFLALSAQLLQ	BAGE	Q13072	2	16	8.68
2697	171	DRB1*10:01	ARAVFLALSAQLLQA	BAGE	Q13072	3	17	8.02
2698	172	DRB1*10:01	AVFLALSAQLLQARL	BAGE	Q13072	5	19	8.74
2699	173	DRB1*10:01	FLALSAQLLQARLMK	BAGE	Q13072	7	21	24.93
2700	174	DRB1*10:01	MAARAVFLALSAQLL	BAGE	Q13072	1	15	11.6
2701	175	DRB1*10:01	RAVFLALSAQLLQAR	BAGE	Q13072	4	18	6.48
2702	176	DRB1*10:01	VFLALSAQLLQARLM	BAGE	Q13072	6	20	11.26
2703	177	DRB1*10:01	AMTKLGFKATLPPFM	SSX2	Q16385	57	71	24.55
2704	178	DRB1*10:01	GFKATLPPFMCNKRA	SSX2	Q16385	62	76	29.58
2705	179	DRB1*10:01	KLGFKATLPPFMCNK	SSX2	Q16385	60	74	9.17
2706	180	DRB1*10:01	LGFKATLPPFMCNKR	SSX2	Q16385	61	75	14.93
2707	181	DRB1*10:01	MTFGRLQGISPKIMP	SSX2	Q16385	99	113	35.68
2708	182	DRB1*10:01	MTKLGFKATLPPFMC	SSX2	Q16385	58	72	16.45

2709	183	DRB1*10:01	PQMTFGRLQGISPKI	SSX2	Q16385	97	111	33.03
2710	184	DRB1*10:01	QMTFGRLQGISPKIM	SSX2	Q16385	98	112	26.83
2711	185	DRB1*10:01	RPQMTFGRLQGISPK	SSX2	Q16385	96	110	49.8
2712	186	DRB1*10:01	TFGRLQGISPKIMPK	SSX2	Q16385	100	114	47.63
2713	187	DRB1*10:01	TKLGFKATLPPFMCN	SSX2	Q16385	59	73	12.08
2714	188	DRB1*10:01	APPAYEKLSAEQSPP	MELAN_A	Q16655	100	114	48.28
2715	189	DRB1*10:01	NAPPAYEKLSAEQSP	MELAN_A	Q16655	99	113	46.48
2716	1	DRB1*11:01	EVISCKLIKRATTRQ	TRP2	O75767	222	236	30.92
2717	2	DRB1*11:01	RKFFHRTCKCTGNFA	TRP2	O75767	88	102	39.55
2718	3	DRB1*11:01	VISCKLIKRATTRQP	TRP2	O75767	223	237	33.86
2719	4	DRB1*11:01	WEVISCKLIKRATTR	TRP2	O75767	221	235	36.89
2720	5	DRB1*11:01	YYYRFVIGLRVWQWE	TRP2	O75767	208	222	49.95
2721	6	DRB1*11:01	DKFFAYLTLAKHTIS	Tyrosinase	P14679	132	146	26.01
2722	7	DRB1*11:01	FAYLTLAKHTISSDY	Tyrosinase	P14679	135	149	35.24
2723	8	DRB1*11:01	FFAYLTLAKHTISSD	Tyrosinase	P14679	134	148	28.57
2724	9	DRB1*11:01	KDKFFAYLTLAKHTI	Tyrosinase	P14679	131	145	33.33
2725	10	DRB1*11:01	KFFAYLTLAKHTISS	Tyrosinase	P14679	133	147	22.47
2726	11	DRB1*11:01	MVPFIPLYRNGDFFI	Tyrosinase	P14679	426	440	32.73
2727	12	DRB1*11:01	VPFIPLYRNGDFFIS	Tyrosinase	P14679	427	441	41.52
2728	13	DRB1*11:01	YMVPFIPLYRNGDFF	Tyrosinase	P14679	425	439	42.2
2729	14	DRB1*11:01	ASYLIRARRSMDEAN	TRP1	P17643	497	511	46.19
2730	15	DRB1*11:01	FVWTHYYSVKKTFLG	TRP1	P17643	188	202	47.59
2731	16	DRB1*11:01	HPLFVIATRRSEEIL	TRP1	P17643	156	170	13.74
2732	17	DRB1*11:01	LFVIATRRSEEILGP	TRP1	P17643	158	172	24.07
2733	18	DRB1*11:01	PLFVIATRRSEEILG	TRP1	P17643	157	171	15.42
2734	19	DRB1*11:01	RTTHPLFVIATRRSE	TRP1	P17643	153	167	24.44
2735	20	DRB1*11:01	TASYLIRARRSMDEA	TRP1	P17643	496	510	39.65
2736	21	DRB1*11:01	THPLFVIATRRSEEI	TRP1	P17643	155	169	15.19
2737	22	DRB1*11:01	THYYSVKKTFLGVGQ	TRP1	P17643	191	205	37.63
2738	23	DRB1*11:01	TTHPLFVIATRRSEE	TRP1	P17643	154	168	19.39
2739	24	DRB1*11:01	VWTHYYSVKKTFLGV	TRP1	P17643	189	203	31.01
2740	25	DRB1*11:01	WTHYYSVKKTFLGVG	TRP1	P17643	190	204	32.76
2741	26	DRB1*11:01	ANASFSIALNFPGSQ	PMEL17	P40967	80	94	49.44
2742	27	DRB1*11:01	ASFSIALNFPGSQKV	PMEL17	P40967	82	96	42.13
2743	28	DRB1*11:01	ATLRLVKRQVPLDCV	PMEL17	P40967	462	476	32.58
2744	29	DRB1*11:01	CQLVLHQILKGGSGT	PMEL17	P40967	550	564	45.41
2745	30	DRB1*11:01	DGTATLRLVKRQVPL	PMEL17	P40967	459	473	18.27
2746	31	DRB1*11:01	GSRSYVPLAHSSSAF	PMEL17	P40967	193	207	39.7
2747	32	DRB1*11:01	GTATLRLVKRQVPLD	PMEL17	P40967	460	474	20.16

2748	33	DRB1*11:01	KRSFVYVWKTWGQYW	PMEL17	P40967	146	160	17.94
2749	34	DRB1*11:01	NASFSIALNFPGSQK	PMEL17	P40967	81	95	33.06
2750	35	DRB1*11:01	QKRSFVYVWKTWGQY	PMEL17	P40967	145	159	18.3
2751	36	DRB1*11:01	QLVLHQILKGGSGTY	PMEL17	P40967	551	565	36.77
2752	37	DRB1*11:01	RSFVYVWKTWGQYWQ	PMEL17	P40967	147	161	30.25
2753	38	DRB1*11:01	RSYVPLAHSSSAFTI	PMEL17	P40967	195	209	42.32
2754	39	DRB1*11:01	SQKRSFVYVWKTWGQ	PMEL17	P40967	144	158	21.74
2755	40	DRB1*11:01	SRSYVPLAHSSSAFT	PMEL17	P40967	194	208	39.52
2756	41	DRB1*11:01	TATLRLVKRQVPLDC	PMEL17	P40967	461	475	23.06
2757	42	DRB1*11:01	WSQKRSFVYVWKTWG	PMEL17	P40967	143	157	41.25
2758	43	DRB1*11:01	ADLVGFLLLKYRARE	MAGE1	P43355	107	121	34.07
2759	44	DRB1*11:01	ARVRFFFPSLREAAL	MAGE1	P43355	288	302	21.89
2760	45	DRB1*11:01	ARYEFLWGPRALAET	MAGE1	P43355	260	274	33.84
2761	46	DRB1*11:01	DLVGFLLLKYRAREP	MAGE1	P43355	108	122	28.93
2762	47	DRB1*11:01	ESLFRAVITKKVADL	MAGE1	P43355	95	109	29.13
2763	48	DRB1*11:01	EYVIKVSARVRFFFP	MAGE1	P43355	281	295	39.57
2764	49	DRB1*11:01	FFFPSLREAALREEE	MAGE1	P43355	292	306	45.29
2765	50	DRB1*11:01	GFLLLKYRAREPVTK	MAGE1	P43355	111	125	45.81
2766	51	DRB1*11:01	ILESLFRAVITKKVA	MAGE1	P43355	93	107	32.17
2767	52	DRB1*11:01	KVLEYVIKVSARVRF	MAGE1	P43355	278	292	25.6
2768	53	DRB1*11:01	LESLFRAVITKKVAD	MAGE1	P43355	94	108	37.28
2769	54	DRB1*11:01	LEYVIKVSARVRFFF	MAGE1	P43355	280	294	34.02
2770	55	DRB1*11:01	LVGFLLLKYRAREPV	MAGE1	P43355	109	123	26.38
2771	56	DRB1*11:01	PARYEFLWGPRALAE	MAGE1	P43355	259	273	37.79
2772	57	DRB1*11:01	RFFFPSLREAALREE	MAGE1	P43355	291	305	18.8
2773	58	DRB1*11:01	RVRFFFPSLREAALR	MAGE1	P43355	289	303	14
2774	59	DRB1*11:01	RYEFLWGPRALAETS	MAGE1	P43355	261	275	34.81
2775	60	DRB1*11:01	SARVRFFFPSLREAA	MAGE1	P43355	287	301	49.09
2776	61	DRB1*11:01	SLFRAVITKKVADLV	MAGE1	P43355	96	110	32.18
2777	62	DRB1*11:01	VGFLLLKYRAREPVT	MAGE1	P43355	110	124	31.71
2778	63	DRB1*11:01	VKVLEYVIKVSARVR	MAGE1	P43355	277	291	29.38
2779	64	DRB1*11:01	VLEYVIKVSARVRFF	MAGE1	P43355	279	293	31.77
2780	65	DRB1*11:01	VRFFFPSLREAALRE	MAGE1	P43355	290	304	14.6
2781	66	DRB1*11:01	AHFLLRKYRAKELVT	MAGE4	P43358	118	132	42.25
2782	67	DRB1*11:01	ARYEFLWGPRALAET	MAGE4	P43358	268	282	33.84
2783	68	DRB1*11:01	DELAHFLLRKYRAKE	MAGE4	P43358	115	129	39.02
2784	69	DRB1*11:01	ELAHFLLRKYRAKEL	MAGE4	P43358	116	130	28.81
2785	70	DRB1*11:01	KVLEHVVRVNARVRI	MAGE4	P43358	286	300	31.5
2786	71	DRB1*11:01	LAHFLLRKYRAKELV	MAGE4	P43358	117	131	29.7
2787	72	DRB1*11:01	PARYEFLWGPRALAE	MAGE4	P43358	267	281	37.79

2788	73	DRB1*11:01	RYEFLWGPRALAETS	MAGE4	P43358	269	283	34.81
2789	74	DRB1*11:01	SYVKVLEHVVRVNAR	MAGE4	P43358	283	297	47.81
2790	75	DRB1*11:01	VDELAHFLLRKYRAK	MAGE4	P43358	114	128	43.08
2791	76	DRB1*11:01	VKVLEHVVRVNARVR	MAGE4	P43358	285	299	30.17
2792	77	DRB1*11:01	YVKVLEHVVRVNARV	MAGE4	P43358	284	298	40.41
2793	78	DRB1*11:01	RAVFLALSAQLLQAR	BAGE	Q13072	4	18	49.7
2794	79	DRB1*11:01	ASEKIFYVYMKRKYE	SSX2	Q16385	42	56	21.98
2795	80	DRB1*11:01	EKIFYVYMKRKYEAM	SSX2	Q16385	44	58	12.58
2796	81	DRB1*11:01	FYVYMKRKYEAMTKL	SSX2	Q16385	47	61	19.78
2797	82	DRB1*11:01	IFYVYMKRKYEAMTK	SSX2	Q16385	46	60	13.87
2798	83	DRB1*11:01	KIFYVYMKRKYEAMT	SSX2	Q16385	45	59	12.77
2799	84	DRB1*11:01	KRKYEAMTKLGFKAT	SSX2	Q16385	52	66	47.42
2800	85	DRB1*11:01	MKRKYEAMTKLGFKA	SSX2	Q16385	51	65	47.51
2801	86	DRB1*11:01	SEKIFYVYMKRKYEA	SSX2	Q16385	43	57	15.39
2802	1	DRB1*11:02	EVISCKLIKRATTRQ	TRP2	O75767	222	236	39.78
2803	2	DRB1*11:02	VISCKLIKRATTRQP	TRP2	O75767	223	237	48.12
2804	3	DRB1*11:02	WEVISCKLIKRATTR	TRP2	O75767	221	235	39.15
2805	4	DRB1*11:02	AGLVSLLCRHKRKQL	Tyrosinase	P14679	493	507	19.74
2806	5	DRB1*11:02	ALLAGLVSLLCRHKR	Tyrosinase	P14679	490	504	49.75
2807	6	DRB1*11:02	GLVSLLCRHKRKQLP	Tyrosinase	P14679	494	508	22.97
2808	7	DRB1*11:02	LAGLVSLLCRHKRKQ	Tyrosinase	P14679	492	506	26.28
2809	8	DRB1*11:02	LLAGLVSLLCRHKRK	Tyrosinase	P14679	491	505	29.59
2810	9	DRB1*11:02	LVSLLCRHKRKQLPE	Tyrosinase	P14679	495	509	32.55
2811	10	DRB1*11:02	NRESYMVPFIPLYRN	Tyrosinase	P14679	421	435	38.22
2812	11	DRB1*11:02	RESYMVPFIPLYRNG	Tyrosinase	P14679	422	436	37.93
2813	12	DRB1*11:02	AACDQRVLIVRRNLL	TRP1	P17643	120	134	43.42
2814	13	DRB1*11:02	ACDQRVLIVRRNLLD	TRP1	P17643	121	135	32.47
2815	14	DRB1*11:02	AFLTWHRYHLLRLEK	TRP1	P17643	219	233	44.85
2816	15	DRB1*11:02	CDQRVLIVRRNLLDL	TRP1	P17643	122	136	25.37
2817	16	DRB1*11:02	DQRVLIVRRNLLDLS	TRP1	P17643	123	137	23
2818	17	DRB1*11:02	EGPAFLTWHRYHLLR	TRP1	P17643	216	230	44.44
2819	18	DRB1*11:02	FFPLLLFQQARAQFP	TRP1	P17643	13	27	46.29
2820	19	DRB1*11:02	FPLLLFQQARAQFPR	TRP1	P17643	14	28	34.58
2821	20	DRB1*11:02	GPAFLTWHRYHLLRL	TRP1	P17643	217	231	35.26
2822	21	DRB1*11:02	HPLFVIATRRSEEIL	TRP1	P17643	156	170	40.79
2823	22	DRB1*11:02	IFGTASYLIRARRSM	TRP1	P17643	493	507	39.73
2824	23	DRB1*11:02	LIFGTASYLIRARRS	TRP1	P17643	492	506	49.31
2825	24	DRB1*11:02	PAFLTWHRYHLLRLE	TRP1	P17643	218	232	38.5
2826	25	DRB1*11:02	PLLLFQQARAQFPRQ	TRP1	P17643	15	29	42.53

2827	26	DRB1*11:02	QRVLIVRRNLLDLSK	TRP1	P17643	124	138	20.24
2828	27	DRB1*11:02	RVLIVRRNLLDLSKE	TRP1	P17643	125	139	31.53
2829	28	DRB1*11:02	THPLFVIATRRSEEI	TRP1	P17643	155	169	37.93
2830	29	DRB1*11:02	ASLIYRRRLMKQDFS	PMEL17	P40967	612	626	30.9
2831	30	DRB1*11:02	ATLRLVKRQVPLDCV	PMEL17	P40967	462	476	33.5
2832	31	DRB1*11:02	AVVLASLIYRRRLMK	PMEL17	P40967	608	622	16.96
2833	32	DRB1*11:02	DGTATLRLVKRQVPL	PMEL17	P40967	459	473	22.93
2834	33	DRB1*11:02	EVTVYHRRGSRSYVP	PMEL17	P40967	185	199	47.92
2835	34	DRB1*11:02	GNKHFLRNQPLTFAL	PMEL17	P40967	228	242	45.46
2836	35	DRB1*11:02	GTATLRLVKRQVPLD	PMEL17	P40967	460	474	26.58
2837	36	DRB1*11:02	KHFLRNQPLTFALQL	PMEL17	P40967	230	244	45.78
2838	37	DRB1*11:02	LASLIYRRRLMKQDF	PMEL17	P40967	611	625	21.1
2839	38	DRB1*11:02	LDGTATLRLVKRQVP	PMEL17	P40967	458	472	45.35
2840	39	DRB1*11:02	LMAVVLASLIYRRRL	PMEL17	P40967	606	620	26.81
2841	40	DRB1*11:02	MAVVLASLIYRRRLM	PMEL17	P40967	607	621	21.84
2842	41	DRB1*11:02	MEVTVYHRRGSRSYV	PMEL17	P40967	184	198	34.06
2843	42	DRB1*11:02	NKHFLRNQPLTFALQ	PMEL17	P40967	229	243	41.83
2844	43	DRB1*11:02	SLIYRRRLMKQDFSV	PMEL17	P40967	613	627	44.49
2845	44	DRB1*11:02	TATLRLVKRQVPLDC	PMEL17	P40967	461	475	28.64
2846	45	DRB1*11:02	TLRLVKRQVPLDCVL	PMEL17	P40967	463	477	48.63
2847	46	DRB1*11:02	TMEVTVYHRRGSRSY	PMEL17	P40967	183	197	42.25
2848	47	DRB1*11:02	VLASLIYRRRLMKQD	PMEL17	P40967	610	624	18.87
2849	48	DRB1*11:02	VLMAVVLASLIYRRR	PMEL17	P40967	605	619	37.73
2850	49	DRB1*11:02	VTAQVVLQAAIPLTS	PMEL17	P40967	286	300	39.64
2851	50	DRB1*11:02	VVLASLIYRRRLMKQ	PMEL17	P40967	609	623	15.16
2852	51	DRB1*11:02	ADLVGFLLLKYRARE	MAGE1	P43355	107	121	40.53
2853	52	DRB1*11:02	ARVRFFFPSLREAAL	MAGE1	P43355	288	302	48.15
2854	53	DRB1*11:02	DLVGFLLLKYRAREP	MAGE1	P43355	108	122	42.83
2855	54	DRB1*11:02	EYVIKVSARVRFFFP	MAGE1	P43355	281	295	9.36
2856	55	DRB1*11:02	FLLLKYRAREPVTKA	MAGE1	P43355	112	126	31.43
2857	56	DRB1*11:02	GFLLLKYRAREPVTK	MAGE1	P43355	111	125	28.99
2858	57	DRB1*11:02	IKVSARVRFFFPSLR	MAGE1	P43355	284	298	40.89
2859	58	DRB1*11:02	KVLEYVIKVSARVRF	MAGE1	P43355	278	292	12.95
2860	59	DRB1*11:02	LEYVIKVSARVRFFF	MAGE1	P43355	280	294	9.32
2861	60	DRB1*11:02	LVGFLLLKYRAREPV	MAGE1	P43355	109	123	29.64
2862	61	DRB1*11:02	SARVRFFFPSLREAA	MAGE1	P43355	287	301	44.9
2863	62	DRB1*11:02	VGFLLLKYRAREPVT	MAGE1	P43355	110	124	27.92
2864	63	DRB1*11:02	VIKVSARVRFFFPSL	MAGE1	P43355	283	297	21.98
2865	64	DRB1*11:02	VKVLEYVIKVSARVR	MAGE1	P43355	277	291	24.8
2866	65	DRB1*11:02	VLEYVIKVSARVRFF	MAGE1	P43355	279	293	9.97

2867	66	DRB1*11:02	YVIKVSARVRFFFPS	MAGE1	P43355	282	296	12.43
2868	67	DRB1*11:02	AEMLERVIKNYKRCF	MAGE4	P43358	134	148	43.68
2869	68	DRB1*11:02	AHFLLRKYRAKELVT	MAGE4	P43358	118	132	35.84
2870	69	DRB1*11:02	ARVRIAYPSLREAAL	MAGE4	P43358	296	310	40.7
2871	70	DRB1*11:02	EHVVRVNARVRIAYP	MAGE4	P43358	289	303	11.58
2872	71	DRB1*11:02	ELAHFLLRKYRAKEL	MAGE4	P43358	116	130	34.56
2873	72	DRB1*11:02	EMLERVIKNYKRCFP	MAGE4	P43358	135	149	40.71
2874	73	DRB1*11:02	ERVIKNYKRCFPVIF	MAGE4	P43358	138	152	35.86
2875	74	DRB1*11:02	HFLLRKYRAKELVTK	MAGE4	P43358	119	133	38.09
2876	75	DRB1*11:02	HVVRVNARVRIAYPS	MAGE4	P43358	290	304	12.34
2877	76	DRB1*11:02	KVLEHVVRVNARVRI	MAGE4	P43358	286	300	14.8
2878	77	DRB1*11:02	LAHFLLRKYRAKELV	MAGE4	P43358	117	131	31.45
2879	78	DRB1*11:02	LEHVVRVNARVRIAY	MAGE4	P43358	288	302	9.7
2880	79	DRB1*11:02	LERVIKNYKRCFPVI	MAGE4	P43358	137	151	30.05
2881	80	DRB1*11:02	MLERVIKNYKRCFPV	MAGE4	P43358	136	150	32.77
2882	81	DRB1*11:02	NARVRIAYPSLREAA	MAGE4	P43358	295	309	36.41
2883	82	DRB1*11:02	RVNARVRIAYPSLRE	MAGE4	P43358	293	307	29.94
2884	83	DRB1*11:02	VKVLEHVVRVNARVR	MAGE4	P43358	285	299	27.4
2885	84	DRB1*11:02	VLEHVVRVNARVRIA	MAGE4	P43358	287	301	12.31
2886	85	DRB1*11:02	VNARVRIAYPSLREA	MAGE4	P43358	294	308	37.07
2887	86	DRB1*11:02	VRVNARVRIAYPSLR	MAGE4	P43358	292	306	24.23
2888	87	DRB1*11:02	VVRVNARVRIAYPSL	MAGE4	P43358	291	305	16.81
2889	88	DRB1*11:02	ILTIRLTAADHRQLQ	NY-ESO-1	P78358	132	146	42.73
2890	89	DRB1*11:02	NILTIRLTAADHRQL	NY-ESO-1	P78358	131	145	45.24
2891	90	DRB1*11:02	EKIFYVYMKRKYEAM	SSX2	Q16385	44	58	27.64
2892	91	DRB1*11:02	FYVYMKRKYEAMTKL	SSX2	Q16385	47	61	39.09
2893	92	DRB1*11:02	IFYVYMKRKYEAMTK	SSX2	Q16385	46	60	28.77
2894	93	DRB1*11:02	KIFYVYMKRKYEAMT	SSX2	Q16385	45	59	26.59
2895	94	DRB1*11:02	SEKIFYVYMKRKYEA	SSX2	Q16385	43	57	37.1
2896	95	DRB1*11:02	WEKMKASEKIFYVYM	SSX2	Q16385	37	51	45.94
2897	1	DRB1*11:03	EVISCKLIKRATTRQ	TRP2	O75767	222	236	25.88
2898	2	DRB1*11:03	FLGALDLAKKRVHPD	TRP2	O75767	141	155	43.01
2899	3	DRB1*11:03	LGALDLAKKRVHPDY	TRP2	O75767	142	156	37.56
2900	4	DRB1*11:03	QFLGALDLAKKRVHP	TRP2	O75767	140	154	40.06
2901	5	DRB1*11:03	QWEVISCKLIKRATT	TRP2	O75767	220	234	39.71
2902	6	DRB1*11:03	VISCKLIKRATTRQP	TRP2	O75767	223	237	29.49
2903	7	DRB1*11:03	WEVISCKLIKRATTR	TRP2	O75767	221	235	27.69
2904	8	DRB1*11:03	AGLVSLLCRHKRKQL	Tyrosinase	P14679	493	507	12.77
2905	9	DRB1*11:03	ALLAGLVSLLCRHKR	Tyrosinase	P14679	490	504	38.43

2906	10	DRB1*11:03	FEQWLRRHRPLQEVY	Tyrosinase	P14679	397	411	47.11
2907	11	DRB1*11:03	GLVSLLCRHKRKQLP	Tyrosinase	P14679	494	508	14
2908	12	DRB1*11:03	IFEQWLRRHRPLQEV	Tyrosinase	P14679	396	410	44.32
2909	13	DRB1*11:03	LAGLVSLLCRHKRKQ	Tyrosinase	P14679	492	506	16.35
2910	14	DRB1*11:03	LLAGLVSLLCRHKRK	Tyrosinase	P14679	491	505	19.4
2911	15	DRB1*11:03	LVSLLCRHKRKQLPE	Tyrosinase	P14679	495	509	18.55
2912	16	DRB1*11:03	SIFEQWLRRHRPLQE	Tyrosinase	P14679	395	409	48.36
2913	17	DRB1*11:03	TERRLLVRRNIFDLS	Tyrosinase	P14679	113	127	49.6
2914	18	DRB1*11:03	VSLLCRHKRKQLPEE	Tyrosinase	P14679	496	510	34.76
2915	19	DRB1*11:03	AACDQRVLIVRRNLL	TRP1	P17643	120	134	46.05
2916	20	DRB1*11:03	ACDQRVLIVRRNLLD	TRP1	P17643	121	135	34
2917	21	DRB1*11:03	AFLTWHRYHLLRLEK	TRP1	P17643	219	233	48.25
2918	22	DRB1*11:03	ASYLIRARRSMDEAN	TRP1	P17643	497	511	39.64
2919	23	DRB1*11:03	CDQRVLIVRRNLLDL	TRP1	P17643	122	136	33.61
2920	24	DRB1*11:03	DQRVLIVRRNLLDLS	TRP1	P17643	123	137	28.49
2921	25	DRB1*11:03	EGPAFLTWHRYHLLR	TRP1	P17643	216	230	37.27
2922	26	DRB1*11:03	EVWPLRFFNRTCHCN	TRP1	P17643	88	102	47.38
2923	27	DRB1*11:03	FGTASYLIRARRSMD	TRP1	P17643	494	508	29.56
2924	28	DRB1*11:03	FPLLLFQQARAQFPR	TRP1	P17643	14	28	32.89
2925	29	DRB1*11:03	FVRALDMAKRTTHPL	TRP1	P17643	144	158	48.1
2926	30	DRB1*11:03	GPAFLTWHRYHLLRL	TRP1	P17643	217	231	35.17
2927	31	DRB1*11:03	GTASYLIRARRSMDE	TRP1	P17643	495	509	36.11
2928	32	DRB1*11:03	HPLFVIATRRSEEIL	TRP1	P17643	156	170	26.12
2929	33	DRB1*11:03	IFGTASYLIRARRSM	TRP1	P17643	493	507	28
2930	34	DRB1*11:03	LIFGTASYLIRARRS	TRP1	P17643	492	506	41.99
2931	35	DRB1*11:03	PAFLTWHRYHLLRLE	TRP1	P17643	218	232	39.35
2932	36	DRB1*11:03	PLFVIATRRSEEILG	TRP1	P17643	157	171	34.81
2933	37	DRB1*11:03	PLLLFQQARAQFPRQ	TRP1	P17643	15	29	40.46
2934	38	DRB1*11:03	QRVLIVRRNLLDLSK	TRP1	P17643	124	138	26.66
2935	39	DRB1*11:03	RTTHPLFVIATRRSE	TRP1	P17643	153	167	35.22
2936	40	DRB1*11:03	RVLIVRRNLLDLSKE	TRP1	P17643	125	139	41.92
2937	41	DRB1*11:03	TASYLIRARRSMDEA	TRP1	P17643	496	510	32.61
2938	42	DRB1*11:03	THPLFVIATRRSEEI	TRP1	P17643	155	169	25.3
2939	43	DRB1*11:03	TTHPLFVIATRRSEE	TRP1	P17643	154	168	30.51
2940	44	DRB1*11:03	VRALDMAKRTTHPLF	TRP1	P17643	145	159	47.66
2941	45	DRB1*11:03	VWPLRFFNRTCHCNG	TRP1	P17643	89	103	45.96
2942	46	DRB1*11:03	ACQLVLHQILKGGSG	PMEL17	P40967	549	563	46.57
2943	47	DRB1*11:03	ASLIYRRRLMKQDFS	PMEL17	P40967	612	626	16.9
2944	48	DRB1*11:03	ATLRLVKRQVPLDCV	PMEL17	P40967	462	476	25.27
2945	49	DRB1*11:03	AVVLASLIYRRRLMK	PMEL17	P40967	608	622	15.16

2946	50	DRB1*11:03	CQLVLHQILKGGSGT	PMEL17	P40967	550	564	43.28
2947	51	DRB1*11:03	DGTATLRLVKRQVPL	PMEL17	P40967	459	473	17.41
2948	52	DRB1*11:03	DWLGVSRQLRTKAWN	PMEL17	P40967	31	45	38.55
2949	53	DRB1*11:03	EVTVYHRRGSRSYVP	PMEL17	P40967	185	199	27.77
2950	54	DRB1*11:03	GTATLRLVKRQVPLD	PMEL17	P40967	460	474	17.89
2951	55	DRB1*11:03	HTMEVTVYHRRGSRS	PMEL17	P40967	182	196	29.26
2952	56	DRB1*11:03	LASLIYRRRLMKQDF	PMEL17	P40967	611	625	13.82
2953	57	DRB1*11:03	LDGTATLRLVKRQVP	PMEL17	P40967	458	472	35.67
2954	58	DRB1*11:03	LGVSRQLRTKAWNRQ	PMEL17	P40967	33	47	40.87
2955	59	DRB1*11:03	LMAVVLASLIYRRRL	PMEL17	P40967	606	620	34.91
2956	60	DRB1*11:03	MAVVLASLIYRRRLM	PMEL17	P40967	607	621	29.58
2957	61	DRB1*11:03	MEVTVYHRRGSRSYV	PMEL17	P40967	184	198	20.84
2958	62	DRB1*11:03	NQDWLGVSRQLRTKA	PMEL17	P40967	29	43	48.39
2959	63	DRB1*11:03	QDWLGVSRQLRTKAW	PMEL17	P40967	30	44	36.72
2960	64	DRB1*11:03	QLVLHQILKGGSGTY	PMEL17	P40967	551	565	37.72
2961	65	DRB1*11:03	SLIYRRRLMKQDFSV	PMEL17	P40967	613	627	23.8
2962	66	DRB1*11:03	TATLRLVKRQVPLDC	PMEL17	P40967	461	475	20.22
2963	67	DRB1*11:03	THTMEVTVYHRRGSR	PMEL17	P40967	181	195	38.46
2964	68	DRB1*11:03	TLRLVKRQVPLDCVL	PMEL17	P40967	463	477	41.82
2965	69	DRB1*11:03	TMEVTVYHRRGSRSY	PMEL17	P40967	183	197	22.12
2966	70	DRB1*11:03	VLASLIYRRRLMKQD	PMEL17	P40967	610	624	13.43
2967	71	DRB1*11:03	VTVYHRRGSRSYVPL	PMEL17	P40967	186	200	48.46
2968	72	DRB1*11:03	VVLASLIYRRRLMKQ	PMEL17	P40967	609	623	12.36
2969	73	DRB1*11:03	WLGVSRQLRTKAWNR	PMEL17	P40967	32	46	33.51
2970	74	DRB1*11:03	ADLVGFLLLKYRARE	MAGE1	P43355	107	121	31.51
2971	75	DRB1*11:03	ARVRFFFPSLREAAL	MAGE1	P43355	288	302	42.45
2972	76	DRB1*11:03	DLVGFLLLKYRAREP	MAGE1	P43355	108	122	25.13
2973	77	DRB1*11:03	EYVIKVSARVRFFFP	MAGE1	P43355	281	295	17.74
2974	78	DRB1*11:03	FLLLKYRAREPVTKA	MAGE1	P43355	112	126	21.81
2975	79	DRB1*11:03	GFLLLKYRAREPVTK	MAGE1	P43355	111	125	18.27
2976	80	DRB1*11:03	KVLEYVIKVSARVRF	MAGE1	P43355	278	292	19.93
2977	81	DRB1*11:03	LEYVIKVSARVRFFF	MAGE1	P43355	280	294	18.68
2978	82	DRB1*11:03	LLLKYRAREPVTKAE	MAGE1	P43355	113	127	39.1
2979	83	DRB1*11:03	LVGFLLLKYRAREPV	MAGE1	P43355	109	123	18.65
2980	84	DRB1*11:03	RVRFFFPSLREAALR	MAGE1	P43355	289	303	41.13
2981	85	DRB1*11:03	SARVRFFFPSLREAA	MAGE1	P43355	287	301	43.74
2982	86	DRB1*11:03	VGFLLLKYRAREPVT	MAGE1	P43355	110	124	17.79
2983	87	DRB1*11:03	VIKVSARVRFFFPSL	MAGE1	P43355	283	297	49.68
2984	88	DRB1*11:03	VKVLEYVIKVSARVR	MAGE1	P43355	277	291	36.03
2985	89	DRB1*11:03	VLEYVIKVSARVRFF	MAGE1	P43355	279	293	17.85

2986	90	DRB1*11:03	YVIKVSARVRFFFPS	MAGE1	P43355	282	296	24.8
2987	91	DRB1*11:03	AEMLERVIKNYKRCF	MAGE4	P43358	134	148	36.93
2988	92	DRB1*11:03	AHFLLRKYRAKELVT	MAGE4	P43358	118	132	21.64
2989	93	DRB1*11:03	DELAHFLLRKYRAKE	MAGE4	P43358	115	129	25.19
2990	94	DRB1*11:03	EHVVRVNARVRIAYP	MAGE4	P43358	289	303	16.52
2991	95	DRB1*11:03	ELAHFLLRKYRAKEL	MAGE4	P43358	116	130	19.32
2992	96	DRB1*11:03	EMLERVIKNYKRCFP	MAGE4	P43358	135	149	33.57
2993	97	DRB1*11:03	ERVIKNYKRCFPVIF	MAGE4	P43358	138	152	31.71
2994	98	DRB1*11:03	FLLRKYRAKELVTKA	MAGE4	P43358	120	134	37.77
2995	99	DRB1*11:03	HFLLRKYRAKELVTK	MAGE4	P43358	119	133	25.58
2996	100	DRB1*11:03	HVVRVNARVRIAYPS	MAGE4	P43358	290	304	17.33
2997	101	DRB1*11:03	KVDELAHFLLRKYRA	MAGE4	P43358	113	127	40.71
2998	102	DRB1*11:03	KVLEHVVRVNARVRI	MAGE4	P43358	286	300	19.62
2999	103	DRB1*11:03	LAHFLLRKYRAKELV	MAGE4	P43358	117	131	18.47
3000	104	DRB1*11:03	LEHVVRVNARVRIAY	MAGE4	P43358	288	302	13.7
3001	105	DRB1*11:03	LERVIKNYKRCFPVI	MAGE4	P43358	137	151	26.62
3002	106	DRB1*11:03	MLERVIKNYKRCFPV	MAGE4	P43358	136	150	27.24
3003	107	DRB1*11:03	RVIKNYKRCFPVIFG	MAGE4	P43358	139	153	46.58
3004	108	DRB1*11:03	RVNARVRIAYPSLRE	MAGE4	P43358	293	307	36.79
3005	109	DRB1*11:03	SYVKVLEHVVRVNAR	MAGE4	P43358	283	297	48.09
3006	110	DRB1*11:03	VDELAHFLLRKYRAK	MAGE4	P43358	114	128	25.44
3007	111	DRB1*11:03	VKVLEHVVRVNARVR	MAGE4	P43358	285	299	25.51
3008	112	DRB1*11:03	VLEHVVRVNARVRIA	MAGE4	P43358	287	301	17.08
3009	113	DRB1*11:03	VRVNARVRIAYPSLR	MAGE4	P43358	292	306	31.25
3010	114	DRB1*11:03	VVRVNARVRIAYPSL	MAGE4	P43358	291	305	22.84
3011	115	DRB1*11:03	YVKVLEHVVRVNARV	MAGE4	P43358	284	298	45.05
3012	116	DRB1*11:03	ASEKIFYVYMKRKYE	SSX2	Q16385	42	56	28.81
3013	117	DRB1*11:03	EKIFYVYMKRKYEAM	SSX2	Q16385	44	58	13.38
3014	118	DRB1*11:03	FYVYMKRKYEAMTKL	SSX2	Q16385	47	61	16.28
3015	119	DRB1*11:03	IFYVYMKRKYEAMTK	SSX2	Q16385	46	60	13.27
3016	120	DRB1*11:03	KASEKIFYVYMKRKY	SSX2	Q16385	41	55	40.17
3017	121	DRB1*11:03	KIFYVYMKRKYEAMT	SSX2	Q16385	45	59	12.89
3018	122	DRB1*11:03	SEKIFYVYMKRKYEA	SSX2	Q16385	43	57	17.36
3019	123	DRB1*11:03	YVYMKRKYEAMTKLG	SSX2	Q16385	48	62	27.78
3020	124	DRB1*11:04	EVISCKLIKRATTRQ	TRP2	O75767	222	236	19.29
3021	1	DRB1*11:04	FLGALDLAKKRVHPD	TRP2	O75767	141	155	39.69
3022	2	DRB1*11:04	LGALDLAKKRVHPDY	TRP2	O75767	142	156	30.57
3023	3	DRB1*11:04	QFLGALDLAKKRVHP	TRP2	O75767	140	154	35.1
3024	4	DRB1*11:04	QWEVISCKLIKRATT	TRP2	O75767	220	234	36.31

3025	5	DRB1*11:04	VISCKLIKRATTRQP	TRP2	O75767	223	237	20.64
3026	6	DRB1*11:04	WEVISCKLIKRATTR	TRP2	O75767	221	235	22.06
3027	7	DRB1*11:04	AGLVSLLCRHKRKQL	Tyrosinase	P14679	493	507	20.85
3028	8	DRB1*11:04	ALLAGLVSLLCRHKR	Tyrosinase	P14679	490	504	44.71
3029	9	DRB1*11:04	DKFFAYLTLAKHTIS	Tyrosinase	P14679	132	146	46.72
3030	10	DRB1*11:04	FAYLTLAKHTISSDY	Tyrosinase	P14679	135	149	27.46
3031	11	DRB1*11:04	FFAYLTLAKHTISSD	Tyrosinase	P14679	134	148	28.42
3032	12	DRB1*11:04	GLVSLLCRHKRKQLP	Tyrosinase	P14679	494	508	26.41
3033	13	DRB1*11:04	IKSYLEQASRIWSWL	Tyrosinase	P14679	464	478	46.23
3034	14	DRB1*11:04	KFFAYLTLAKHTISS	Tyrosinase	P14679	133	147	27.61
3035	15	DRB1*11:04	KSYLEQASRIWSWLL	Tyrosinase	P14679	465	479	48.45
3036	16	DRB1*11:04	LAGLVSLLCRHKRKQ	Tyrosinase	P14679	492	506	24.86
3037	17	DRB1*11:04	LLAGLVSLLCRHKRK	Tyrosinase	P14679	491	505	27.66
3038	18	DRB1*11:04	LVSLLCRHKRKQLPE	Tyrosinase	P14679	495	509	40.74
3039	19	DRB1*11:04	AACDQRVLIVRRNLL	TRP1	P17643	120	134	29.49
3040	20	DRB1*11:04	ACDQRVLIVRRNLLD	TRP1	P17643	121	135	24.43
3041	21	DRB1*11:04	ASYLIRARRSMDEAN	TRP1	P17643	497	511	28.67
3042	22	DRB1*11:04	CDQRVLIVRRNLLDL	TRP1	P17643	122	136	20.29
3043	23	DRB1*11:04	DQRVLIVRRNLLDLS	TRP1	P17643	123	137	19.07
3044	24	DRB1*11:04	FFPLLLFQQARAQFP	TRP1	P17643	13	27	32.64
3045	25	DRB1*11:04	FGTASYLIRARRSMD	TRP1	P17643	494	508	30.25
3046	26	DRB1*11:04	FPLLLFQQARAQFPR	TRP1	P17643	14	28	26.77
3047	27	DRB1*11:04	FVRALDMAKRTTHPL	TRP1	P17643	144	158	38.95
3048	28	DRB1*11:04	GTASYLIRARRSMDE	TRP1	P17643	495	509	29.26
3049	29	DRB1*11:04	HPLFVIATRRSEEIL	TRP1	P17643	156	170	12.52
3050	30	DRB1*11:04	IFFPLLLFQQARAQF	TRP1	P17643	12	26	47.75
3051	31	DRB1*11:04	IFGTASYLIRARRSM	TRP1	P17643	493	507	30.93
3052	32	DRB1*11:04	KRTTHPLFVIATRRS	TRP1	P17643	152	166	42.57
3053	33	DRB1*11:04	LFVIATRRSEEILGP	TRP1	P17643	158	172	37.31
3054	34	DRB1*11:04	LIFGTASYLIRARRS	TRP1	P17643	492	506	43.28
3055	35	DRB1*11:04	PLFVIATRRSEEILG	TRP1	P17643	157	171	16.46
3056	36	DRB1*11:04	PLLLFQQARAQFPRQ	TRP1	P17643	15	29	38.91
3057	37	DRB1*11:04	QRVLIVRRNLLDLSK	TRP1	P17643	124	138	17.56
3058	38	DRB1*11:04	RTTHPLFVIATRRSE	TRP1	P17643	153	167	17.16
3059	39	DRB1*11:04	RVLIVRRNLLDLSKE	TRP1	P17643	125	139	27.27
3060	40	DRB1*11:04	SYLIRARRSMDEANQ	TRP1	P17643	498	512	39.58
3061	41	DRB1*11:04	TASYLIRARRSMDEA	TRP1	P17643	496	510	23.81
3062	42	DRB1*11:04	THPLFVIATRRSEEI	TRP1	P17643	155	169	12.59
3063	43	DRB1*11:04	TTHPLFVIATRRSEE	TRP1	P17643	154	168	15.45
3064	44	DRB1*11:04	VRALDMAKRTTHPLF	TRP1	P17643	145	159	31.74

3065	45	DRB1*11:04	ACQLVLHQILKGGSG	PMEL17	P40967	549	563	20.08
3066	46	DRB1*11:04	ASLIYRRRLMKQDFS	PMEL17	P40967	612	626	36.01
3067	47	DRB1*11:04	ATLRLVKRQVPLDCV	PMEL17	P40967	462	476	15.25
3068	48	DRB1*11:04	AVVLASLIYRRRLMK	PMEL17	P40967	608	622	20.89
3069	49	DRB1*11:04	CQLVLHQILKGGSGT	PMEL17	P40967	550	564	17.75
3070	50	DRB1*11:04	DGTATLRLVKRQVPL	PMEL17	P40967	459	473	9.31
3071	51	DRB1*11:04	DWLGVSRQLRTKAWN	PMEL17	P40967	31	45	44.48
3072	52	DRB1*11:04	GTATLRLVKRQVPLD	PMEL17	P40967	460	474	10.14
3073	53	DRB1*11:04	KRSFVYVWKTWGQYW	PMEL17	P40967	146	160	37.07
3074	54	DRB1*11:04	LASLIYRRRLMKQDF	PMEL17	P40967	611	625	26.62
3075	55	DRB1*11:04	LDGTATLRLVKRQVP	PMEL17	P40967	458	472	22.43
3076	56	DRB1*11:04	LGVSRQLRTKAWNRQ	PMEL17	P40967	33	47	48.19
3077	57	DRB1*11:04	LMAVVLASLIYRRRL	PMEL17	P40967	606	620	37.88
3078	58	DRB1*11:04	LVLHQILKGGSGTYC	PMEL17	P40967	552	566	20.38
3079	59	DRB1*11:04	MAVVLASLIYRRRLM	PMEL17	P40967	607	621	30.33
3080	60	DRB1*11:04	PACQLVLHQILKGGS	PMEL17	P40967	548	562	36.62
3081	61	DRB1*11:04	QDWLGVSRQLRTKAW	PMEL17	P40967	30	44	43.91
3082	62	DRB1*11:04	QKRSFVYVWKTWGQY	PMEL17	P40967	145	159	37.12
3083	63	DRB1*11:04	QLVLHQILKGGSGTY	PMEL17	P40967	551	565	15.12
3084	64	DRB1*11:04	SQKRSFVYVWKTWGQ	PMEL17	P40967	144	158	46.19
3085	65	DRB1*11:04	TATLRLVKRQVPLDC	PMEL17	P40967	461	475	11.26
3086	66	DRB1*11:04	TLRLVKRQVPLDCVL	PMEL17	P40967	463	477	31.83
3087	67	DRB1*11:04	VLASLIYRRRLMKQD	PMEL17	P40967	610	624	22.87
3088	68	DRB1*11:04	VLHQILKGGSGTYCL	PMEL17	P40967	553	567	49.95
3089	69	DRB1*11:04	VVLASLIYRRRLMKQ	PMEL17	P40967	609	623	19.21
3090	70	DRB1*11:04	WLGVSRQLRTKAWNR	PMEL17	P40967	32	46	35.36
3091	71	DRB1*11:04	ADLVGFLLLKYRARE	MAGE1	P43355	107	121	22.28
3092	72	DRB1*11:04	ARVRFFFPSLREAAL	MAGE1	P43355	288	302	42.61
3093	73	DRB1*11:04	CILESLFRAVITKKV	MAGE1	P43355	92	106	49.41
3094	74	DRB1*11:04	DLVGFLLLKYRAREP	MAGE1	P43355	108	122	17.99
3095	75	DRB1*11:04	ESLFRAVITKKVADL	MAGE1	P43355	95	109	44.48
3096	76	DRB1*11:04	EYVIKVSARVRFFFP	MAGE1	P43355	281	295	17.68
3097	77	DRB1*11:04	FLLLKYRAREPVTKA	MAGE1	P43355	112	126	33.91
3098	78	DRB1*11:04	GFLLLKYRAREPVTK	MAGE1	P43355	111	125	25.28
3099	79	DRB1*11:04	ILESLFRAVITKKVA	MAGE1	P43355	93	107	36.18
3100	80	DRB1*11:04	KVLEYVIKVSARVRF	MAGE1	P43355	278	292	14.12
3101	81	DRB1*11:04	LESLFRAVITKKVAD	MAGE1	P43355	94	108	49.59
3102	82	DRB1*11:04	LEYVIKVSARVRFFF	MAGE1	P43355	280	294	17.21
3103	83	DRB1*11:04	LVGFLLLKYRAREPV	MAGE1	P43355	109	123	17.19
3104	84	DRB1*11:04	RVRFFFPSLREAALR	MAGE1	P43355	289	303	32.94

3105	85	DRB1*11:04	SLFRAVITKKVADLV	MAGE1	P43355	96	110	44.25
3106	86	DRB1*11:04	VADLVGFLLLKYRAR	MAGE1	P43355	106	120	36.58
3107	87	DRB1*11:04	VGFLLLKYRAREPVT	MAGE1	P43355	110	124	18.8
3108	88	DRB1*11:04	VIKVSARVRFFFPSL	MAGE1	P43355	283	297	46.43
3109	89	DRB1*11:04	VKVLEYVIKVSARVR	MAGE1	P43355	277	291	21.36
3110	90	DRB1*11:04	VLEYVIKVSARVRFF	MAGE1	P43355	279	293	16.18
3111	91	DRB1*11:04	VRFFFPSLREAALRE	MAGE1	P43355	290	304	38.38
3112	92	DRB1*11:04	YVIKVSARVRFFFPS	MAGE1	P43355	282	296	23.47
3113	93	DRB1*11:04	AEMLERVIKNYKRCF	MAGE4	P43358	134	148	47.43
3114	94	DRB1*11:04	AHFLLRKYRAKELVT	MAGE4	P43358	118	132	26.1
3115	95	DRB1*11:04	DELAHFLLRKYRAKE	MAGE4	P43358	115	129	25.68
3116	96	DRB1*11:04	EHVVRVNARVRIAYP	MAGE4	P43358	289	303	35.82
3117	97	DRB1*11:04	ELAHFLLRKYRAKEL	MAGE4	P43358	116	130	19.95
3118	98	DRB1*11:04	HFLLRKYRAKELVTK	MAGE4	P43358	119	133	38.75
3119	99	DRB1*11:04	HVVRVNARVRIAYPS	MAGE4	P43358	290	304	39.92
3120	100	DRB1*11:04	KVDELAHFLLRKYRA	MAGE4	P43358	113	127	45.38
3121	101	DRB1*11:04	KVLEHVVRVNARVRI	MAGE4	P43358	286	300	14.3
3122	102	DRB1*11:04	LAHFLLRKYRAKELV	MAGE4	P43358	117	131	21.15
3123	103	DRB1*11:04	LEHVVRVNARVRIAY	MAGE4	P43358	288	302	26.61
3124	104	DRB1*11:04	SYVKVLEHVVRVNAR	MAGE4	P43358	283	297	21.83
3125	105	DRB1*11:04	TKAEMLERVIKNYKR	MAGE4	P43358	132	146	48.98
3126	106	DRB1*11:04	TSYVKVLEHVVRVNA	MAGE4	P43358	282	296	45.07
3127	107	DRB1*11:04	VDELAHFLLRKYRAK	MAGE4	P43358	114	128	28.08
3128	108	DRB1*11:04	VKVLEHVVRVNARVR	MAGE4	P43358	285	299	13.92
3129	109	DRB1*11:04	VLEHVVRVNARVRIA	MAGE4	P43358	287	301	20.88
3130	110	DRB1*11:04	YVKVLEHVVRVNARV	MAGE4	P43358	284	298	19.77
3131	111	DRB1*11:04	SGNILTIRLTAADHR	NY-ESO-1	P78358	129	143	45.87
3132	112	DRB1*11:04	ASEKIFYVYMKRKYE	SSX2	Q16385	42	56	28.14
3133	113	DRB1*11:04	EKIFYVYMKRKYEAM	SSX2	Q16385	44	58	12.34
3134	114	DRB1*11:04	FYVYMKRKYEAMTKL	SSX2	Q16385	47	61	16.24
3135	115	DRB1*11:04	IFYVYMKRKYEAMTK	SSX2	Q16385	46	60	11.71
3136	116	DRB1*11:04	KIFYVYMKRKYEAMT	SSX2	Q16385	45	59	11.33
3137	117	DRB1*11:04	SEKIFYVYMKRKYEA	SSX2	Q16385	43	57	16.45
3138	118	DRB1*11:04	YVYMKRKYEAMTKLG	SSX2	Q16385	48	62	35.29
3139	1	DRB1*11:06	DGTATLRLVKRQVPL	PMEL17	P40967	459	473	38.75
3140	2	DRB1*11:06	GTATLRLVKRQVPLD	PMEL17	P40967	460	474	44.04
3141	1	DRB1*11:07	EYVIKVSARVRFFFP	MAGE1	P43355	281	295	44.78
3142	2	DRB1*11:07	LEYVIKVSARVRFFF	MAGE1	P43355	280	294	47.07

3143	1	DRB1*11:08	KVYYYRFVIGLRVWQ	TRP2	O75767	206	220	48.02
3144	2	DRB1*11:08	DKFFAYLTLAKHTIS	Tyrosinase	P14679	132	146	39.26
3145	3	DRB1*11:08	FFAYLTLAKHTISSD	Tyrosinase	P14679	134	148	42.23
3146	4	DRB1*11:08	KDKFFAYLTLAKHTI	Tyrosinase	P14679	131	145	49
3147	5	DRB1*11:08	KFFAYLTLAKHTISS	Tyrosinase	P14679	133	147	33.88
3148	6	DRB1*11:08	HPLFVIATRRSEEIL	TRP1	P17643	156	170	26.9
3149	7	DRB1*11:08	PLFVIATRRSEEILG	TRP1	P17643	157	171	35.15
3150	8	DRB1*11:08	RTTHPLFVIATRRSE	TRP1	P17643	153	167	45.17
3151	9	DRB1*11:08	THPLFVIATRRSEEI	TRP1	P17643	155	169	27.7
3152	10	DRB1*11:08	TTHPLFVIATRRSEE	TRP1	P17643	154	168	35.68
3153	11	DRB1*11:08	DGTATLRLVKRQVPL	PMEL17	P40967	459	473	40.82
3154	12	DRB1*11:08	GGNKHFLRNQPLTFA	PMEL17	P40967	227	241	42.7
3155	13	DRB1*11:08	GNKHFLRNQPLTFAL	PMEL17	P40967	228	242	31.11
3156	14	DRB1*11:08	GTATLRLVKRQVPLD	PMEL17	P40967	460	474	40.71
3157	15	DRB1*11:08	KHFLRNQPLTFALQL	PMEL17	P40967	230	244	37
3158	16	DRB1*11:08	KRSFVYVWKTWGQYW	PMEL17	P40967	146	160	39.62
3159	17	DRB1*11:08	NASFSIALNFPGSQK	PMEL17	P40967	81	95	42.89
3160	18	DRB1*11:08	NKHFLRNQPLTFALQ	PMEL17	P40967	229	243	28.13
3161	19	DRB1*11:08	QKRSFVYVWKTWGQY	PMEL17	P40967	145	159	41.63
3162	20	DRB1*11:08	TATLRLVKRQVPLDC	PMEL17	P40967	461	475	49.52
3163	21	DRB1*11:08	ARVRFFFPSLREAAL	MAGE1	P43355	288	302	44.37
3164	22	DRB1*11:08	ESLFRAVITKKVADL	MAGE1	P43355	95	109	34.07
3165	23	DRB1*11:08	EYVIKVSARVRFFFP	MAGE1	P43355	281	295	34.14
3166	24	DRB1*11:08	ILESLFRAVITKKVA	MAGE1	P43355	93	107	37.5
3167	25	DRB1*11:08	KVLEYVIKVSARVRF	MAGE1	P43355	278	292	25.12
3168	26	DRB1*11:08	LESLFRAVITKKVAD	MAGE1	P43355	94	108	38.14
3169	27	DRB1*11:08	LEYVIKVSARVRFFF	MAGE1	P43355	280	294	27.58
3170	28	DRB1*11:08	RFFFPSLREAALREE	MAGE1	P43355	291	305	45.07
3171	29	DRB1*11:08	RVRFFFPSLREAALR	MAGE1	P43355	289	303	31.48
3172	30	DRB1*11:08	SLFRAVITKKVADLV	MAGE1	P43355	96	110	41.43
3173	31	DRB1*11:08	VKVLEYVIKVSARVR	MAGE1	P43355	277	291	30.97
3174	32	DRB1*11:08	VLEYVIKVSARVRFF	MAGE1	P43355	279	293	27.01
3175	33	DRB1*11:08	VRFFFPSLREAALRE	MAGE1	P43355	290	304	33.35
3176	34	DRB1*11:08	KVLEHVVRVNARVRI	MAGE4	P43358	286	300	46.09
3177	35	DRB1*11:08	LEHVVRVNARVRIAY	MAGE4	P43358	288	302	45.51
3178	36	DRB1*11:08	VLEHVVRVNARVRIA	MAGE4	P43358	287	301	49.56
3179	37	DRB1*11:08	ARAVFLALSAQLLQA	BAGE	Q13072	3	17	44.86
3180	38	DRB1*11:08	RAVFLALSAQLLQAR	BAGE	Q13072	4	18	31.24
3181	39	DRB1*11:08	EKIFYVYMKRKYEAM	SSX2	Q16385	44	58	31.94

3182	40	DRB1*11:08	IFYVYMKRKYEAMTK	SSX2	Q16385	46	60	42.52
3183	41	DRB1*11:08	KIFYVYMKRKYEAMT	SSX2	Q16385	45	59	32.05
3184	42	DRB1*11:08	SEKIFYVYMKRKYEA	SSX2	Q16385	43	57	38.7
3185	1	DRB1*11:10	EVISCKLIKRATTRQ	TRP2	O75767	222	236	30.92
3186	2	DRB1*11:10	RKFFHRTCKCTGNFA	TRP2	O75767	88	102	39.55
3187	3	DRB1*11:10	VISCKLIKRATTRQP	TRP2	O75767	223	237	33.86
3188	4	DRB1*11:10	WEVISCKLIKRATTR	TRP2	O75767	221	235	36.89
3189	5	DRB1*11:10	YYYRFVIGLRVWQWE	TRP2	O75767	208	222	49.95
3190	6	DRB1*11:10	DKFFAYLTLAKHTIS	Tyrosinase	P14679	132	146	26.01
3191	7	DRB1*11:10	FAYLTLAKHTISSDY	Tyrosinase	P14679	135	149	35.24
3192	8	DRB1*11:10	FFAYLTLAKHTISSD	Tyrosinase	P14679	134	148	28.57
3193	9	DRB1*11:10	KDKFFAYLTLAKHTI	Tyrosinase	P14679	131	145	33.33
3194	10	DRB1*11:10	KFFAYLTLAKHTISS	Tyrosinase	P14679	133	147	22.47
3195	11	DRB1*11:10	MVPFIPLYRNGDFFI	Tyrosinase	P14679	426	440	32.73
3196	12	DRB1*11:10	VPFIPLYRNGDFFIS	Tyrosinase	P14679	427	441	41.52
3197	13	DRB1*11:10	YMVPFIPLYRNGDFF	Tyrosinase	P14679	425	439	42.2
3198	14	DRB1*11:10	ASYLIRARRSMDEAN	TRP1	P17643	497	511	46.19
3199	15	DRB1*11:10	FVWTHYYSVKKTFLG	TRP1	P17643	188	202	47.59
3200	16	DRB1*11:10	HPLFVIATRRSEEIL	TRP1	P17643	156	170	13.74
3201	17	DRB1*11:10	LFVIATRRSEEILGP	TRP1	P17643	158	172	24.07
3202	18	DRB1*11:10	PLFVIATRRSEEILG	TRP1	P17643	157	171	15.42
3203	19	DRB1*11:10	RTTHPLFVIATRRSE	TRP1	P17643	153	167	24.44
3204	20	DRB1*11:10	TASYLIRARRSMDEA	TRP1	P17643	496	510	39.65
3205	21	DRB1*11:10	THPLFVIATRRSEEI	TRP1	P17643	155	169	15.19
3206	22	DRB1*11:10	THYYSVKKTFLGVGQ	TRP1	P17643	191	205	37.63
3207	23	DRB1*11:10	TTHPLFVIATRRSEE	TRP1	P17643	154	168	19.39
3208	24	DRB1*11:10	VWTHYYSVKKTFLGV	TRP1	P17643	189	203	31.01
3209	25	DRB1*11:10	WTHYYSVKKTFLGVG	TRP1	P17643	190	204	32.76
3210	26	DRB1*11:10	ANASFSIALNFPGSQ	PMEL17	P40967	80	94	49.44
3211	27	DRB1*11:10	ASFSIALNFPGSQKV	PMEL17	P40967	82	96	42.13
3212	28	DRB1*11:10	ATLRLVKRQVPLDCV	PMEL17	P40967	462	476	32.58
3213	29	DRB1*11:10	CQLVLHQILKGGSGT	PMEL17	P40967	550	564	45.41
3214	30	DRB1*11:10	DGTATLRLVKRQVPL	PMEL17	P40967	459	473	18.27
3215	31	DRB1*11:10	GSRSYVPLAHSSSAF	PMEL17	P40967	193	207	39.7
3216	32	DRB1*11:10	GTATLRLVKRQVPLD	PMEL17	P40967	460	474	20.16
3217	33	DRB1*11:10	KRSFVYVWKTWGQYW	PMEL17	P40967	146	160	17.94
3218	34	DRB1*11:10	NASFSIALNFPGSQK	PMEL17	P40967	81	95	33.06
3219	35	DRB1*11:10	QKRSFVYVWKTWGQY	PMEL17	P40967	145	159	18.3
3220	36	DRB1*11:10	QLVLHQILKGGSGTY	PMEL17	P40967	551	565	36.77

3221	37	DRB1*11:10	RSFVYVWKTWGQYWQ	PMEL17	P40967	147	161	30.25
3222	38	DRB1*11:10	RSYVPLAHSSSAFTI	PMEL17	P40967	195	209	42.32
3223	39	DRB1*11:10	SQKRSFVYVWKTWGQ	PMEL17	P40967	144	158	21.74
3224	40	DRB1*11:10	SRSYVPLAHSSSAFT	PMEL17	P40967	194	208	39.52
3225	41	DRB1*11:10	TATLRLVKRQVPLDC	PMEL17	P40967	461	475	23.06
3226	42	DRB1*11:10	WSQKRSFVYVWKTWG	PMEL17	P40967	143	157	41.25
3227	43	DRB1*11:10	ADLVGFLLLKYRARE	MAGE1	P43355	107	121	34.07
3228	44	DRB1*11:10	ARVRFFFPSLREAAL	MAGE1	P43355	288	302	21.89
3229	45	DRB1*11:10	ARYEFLWGPRALAET	MAGE1	P43355	260	274	33.84
3230	46	DRB1*11:10	DLVGFLLLKYRAREP	MAGE1	P43355	108	122	28.93
3231	47	DRB1*11:10	ESLFRAVITKKVADL	MAGE1	P43355	95	109	29.13
3232	48	DRB1*11:10	EYVIKVSARVRFFFP	MAGE1	P43355	281	295	39.57
3233	49	DRB1*11:10	FFFPSLREAALREEE	MAGE1	P43355	292	306	45.29
3234	50	DRB1*11:10	GFLLLKYRAREPVTK	MAGE1	P43355	111	125	45.81
3235	51	DRB1*11:10	ILESLFRAVITKKVA	MAGE1	P43355	93	107	32.17
3236	52	DRB1*11:10	KVLEYVIKVSARVRF	MAGE1	P43355	278	292	25.6
3237	53	DRB1*11:10	LESLFRAVITKKVAD	MAGE1	P43355	94	108	37.28
3238	54	DRB1*11:10	LEYVIKVSARVRFFF	MAGE1	P43355	280	294	34.02
3239	55	DRB1*11:10	LVGFLLLKYRAREPV	MAGE1	P43355	109	123	26.38
3240	56	DRB1*11:10	PARYEFLWGPRALAE	MAGE1	P43355	259	273	37.79
3241	57	DRB1*11:10	RFFFPSLREAALREE	MAGE1	P43355	291	305	18.8
3242	58	DRB1*11:10	RVRFFFPSLREAALR	MAGE1	P43355	289	303	14
3243	59	DRB1*11:10	RYEFLWGPRALAETS	MAGE1	P43355	261	275	34.81
3244	60	DRB1*11:10	SARVRFFFPSLREAA	MAGE1	P43355	287	301	49.09
3245	61	DRB1*11:10	SLFRAVITKKVADLV	MAGE1	P43355	96	110	32.18
3246	62	DRB1*11:10	VGFLLLKYRAREPVT	MAGE1	P43355	110	124	31.71
3247	63	DRB1*11:10	VKVLEYVIKVSARVR	MAGE1	P43355	277	291	29.38
3248	64	DRB1*11:10	VLEYVIKVSARVRFF	MAGE1	P43355	279	293	31.77
3249	65	DRB1*11:10	VRFFFPSLREAALRE	MAGE1	P43355	290	304	14.6
3250	66	DRB1*11:10	AHFLLRKYRAKELVT	MAGE4	P43358	118	132	42.25
3251	67	DRB1*11:10	ARYEFLWGPRALAET	MAGE4	P43358	268	282	33.84
3252	68	DRB1*11:10	DELAHFLLRKYRAKE	MAGE4	P43358	115	129	39.02
3253	69	DRB1*11:10	ELAHFLLRKYRAKEL	MAGE4	P43358	116	130	28.81
3254	70	DRB1*11:10	KVLEHVVRVNARVRI	MAGE4	P43358	286	300	31.5
3255	71	DRB1*11:10	LAHFLLRKYRAKELV	MAGE4	P43358	117	131	29.7
3256	72	DRB1*11:10	PARYEFLWGPRALAE	MAGE4	P43358	267	281	37.79
3257	73	DRB1*11:10	RYEFLWGPRALAETS	MAGE4	P43358	269	283	34.81
3258	74	DRB1*11:10	SYVKVLEHVVRVNAR	MAGE4	P43358	283	297	47.81
3259	75	DRB1*11:10	VDELAHFLLRKYRAK	MAGE4	P43358	114	128	43.08
3260	76	DRB1*11:10	VKVLEHVVRVNARVR	MAGE4	P43358	285	299	30.17

3261	77	DRB1*11:10	YVKVLEHVVRVNARV	MAGE4	P43358	284	298	40.41
3262	78	DRB1*11:10	RAVFLALSAQLLQAR	BAGE	Q13072	4	18	49.7
3263	79	DRB1*11:10	ASEKIFYVYMKRKYE	SSX2	Q16385	42	56	21.98
3264	80	DRB1*11:10	EKIFYVYMKRKYEAM	SSX2	Q16385	44	58	12.58
3265	81	DRB1*11:10	FYVYMKRKYEAMTKL	SSX2	Q16385	47	61	19.78
3266	82	DRB1*11:10	IFYVYMKRKYEAMTK	SSX2	Q16385	46	60	13.87
3267	83	DRB1*11:10	KIFYVYMKRKYEAMT	SSX2	Q16385	45	59	12.77
3268	84	DRB1*11:10	KRKYEAMTKLGFKAT	SSX2	Q16385	52	66	47.42
3269	85	DRB1*11:10	MKRKYEAMTKLGFKA	SSX2	Q16385	51	65	47.51
3270	86	DRB1*11:10	SEKIFYVYMKRKYEA	SSX2	Q16385	43	57	15.39
3271	1	DRB1*11:11	HPLFVIATRRSEEIL	TRP1	P17643	156	170	45.73
3272	2	DRB1*11:11	THPLFVIATRRSEEI	TRP1	P17643	155	169	49.02
3273	3	DRB1*11:11	LAHFLLRKYRAKELV	MAGE4	P43358	117	131	47.23
3274	4	DRB1*11:11	LEHVVRVNARVRIAY	MAGE4	P43358	288	302	42.96
3275	5	DRB1*11:11	EKIFYVYMKRKYEAM	SSX2	Q16385	44	58	27.25
3276	6	DRB1*11:11	FYVYMKRKYEAMTKL	SSX2	Q16385	47	61	45.35
3277	7	DRB1*11:11	IFYVYMKRKYEAMTK	SSX2	Q16385	46	60	31.55
3278	8	DRB1*11:11	KIFYVYMKRKYEAMT	SSX2	Q16385	45	59	27.01
3279	9	DRB1*11:11	SEKIFYVYMKRKYEA	SSX2	Q16385	43	57	34.6
3280	1	DRB1*11:12	EVISCKLIKRATTRQ	TRP2	O75767	222	236	30.92
3281	2	DRB1*11:12	RKFFHRTCKCTGNFA	TRP2	O75767	88	102	39.55
3282	3	DRB1*11:12	VISCKLIKRATTRQP	TRP2	O75767	223	237	33.86
3283	4	DRB1*11:12	WEVISCKLIKRATTR	TRP2	O75767	221	235	36.89
3284	5	DRB1*11:12	YYYRFVIGLRVWQWE	TRP2	O75767	208	222	49.95
3285	6	DRB1*11:12	DKFFAYLTLAKHTIS	Tyrosinase	P14679	132	146	26.01
3286	7	DRB1*11:12	FAYLTLAKHTISSDY	Tyrosinase	P14679	135	149	35.24
3287	8	DRB1*11:12	FFAYLTLAKHTISSD	Tyrosinase	P14679	134	148	28.57
3288	9	DRB1*11:12	KDKFFAYLTLAKHTI	Tyrosinase	P14679	131	145	33.33
3289	10	DRB1*11:12	KFFAYLTLAKHTISS	Tyrosinase	P14679	133	147	22.47
3290	11	DRB1*11:12	MVPFIPLYRNGDFFI	Tyrosinase	P14679	426	440	32.73
3291	12	DRB1*11:12	VPFIPLYRNGDFFIS	Tyrosinase	P14679	427	441	41.52
3292	13	DRB1*11:12	YMVPFIPLYRNGDFF	Tyrosinase	P14679	425	439	42.2
3293	14	DRB1*11:12	ASYLIRARRSMDEAN	TRP1	P17643	497	511	46.19
3294	15	DRB1*11:12	FVWTHYYSVKKTFLG	TRP1	P17643	188	202	47.59
3295	16	DRB1*11:12	HPLFVIATRRSEEIL	TRP1	P17643	156	170	13.74
3296	17	DRB1*11:12	LFVIATRRSEEILGP	TRP1	P17643	158	172	24.07
3297	18	DRB1*11:12	PLFVIATRRSEEILG	TRP1	P17643	157	171	15.42
3298	19	DRB1*11:12	RTTHPLFVIATRRSE	TRP1	P17643	153	167	24.44

3299	20	DRB1*11:12	TASYLIRARRSMDEA	TRP1	P17643	496	510	39.65
3300	21	DRB1*11:12	THPLFVIATRRSEEI	TRP1	P17643	155	169	15.19
3301	22	DRB1*11:12	THYYSVKKTFLGVGQ	TRP1	P17643	191	205	37.63
3302	23	DRB1*11:12	TTHPLFVIATRRSEE	TRP1	P17643	154	168	19.39
3303	24	DRB1*11:12	VWTHYYSVKKTFLGV	TRP1	P17643	189	203	31.01
3304	25	DRB1*11:12	WTHYYSVKKTFLGVG	TRP1	P17643	190	204	32.76
3305	26	DRB1*11:12	ANASFSIALNFPGSQ	PMEL17	P40967	80	94	49.44
3306	27	DRB1*11:12	ASFSIALNFPGSQKV	PMEL17	P40967	82	96	42.13
3307	28	DRB1*11:12	ATLRLVKRQVPLDCV	PMEL17	P40967	462	476	32.58
3308	29	DRB1*11:12	CQLVLHQILKGGSGT	PMEL17	P40967	550	564	45.41
3309	30	DRB1*11:12	DGTATLRLVKRQVPL	PMEL17	P40967	459	473	18.27
3310	31	DRB1*11:12	GSRSYVPLAHSSSAF	PMEL17	P40967	193	207	39.7
3311	32	DRB1*11:12	GTATLRLVKRQVPLD	PMEL17	P40967	460	474	20.16
3312	33	DRB1*11:12	KRSFVYVWKTWGQYW	PMEL17	P40967	146	160	17.94
3313	34	DRB1*11:12	NASFSIALNFPGSQK	PMEL17	P40967	81	95	33.06
3314	35	DRB1*11:12	QKRSFVYVWKTWGQY	PMEL17	P40967	145	159	18.3
3315	36	DRB1*11:12	QLVLHQILKGGSGTY	PMEL17	P40967	551	565	36.77
3316	37	DRB1*11:12	RSFVYVWKTWGQYWQ	PMEL17	P40967	147	161	30.25
3317	38	DRB1*11:12	RSYVPLAHSSSAFTI	PMEL17	P40967	195	209	42.32
3318	39	DRB1*11:12	SQKRSFVYVWKTWGQ	PMEL17	P40967	144	158	21.74
3319	40	DRB1*11:12	SRSYVPLAHSSSAFT	PMEL17	P40967	194	208	39.52
3320	41	DRB1*11:12	TATLRLVKRQVPLDC	PMEL17	P40967	461	475	23.06
3321	42	DRB1*11:12	WSQKRSFVYVWKTWG	PMEL17	P40967	143	157	41.25
3322	43	DRB1*11:12	ADLVGFLLLKYRARE	MAGE1	P43355	107	121	34.07
3323	44	DRB1*11:12	ARVRFFFPSLREAAL	MAGE1	P43355	288	302	21.89
3324	45	DRB1*11:12	ARYEFLWGPRALAET	MAGE1	P43355	260	274	33.84
3325	46	DRB1*11:12	DLVGFLLLKYRAREP	MAGE1	P43355	108	122	28.93
3326	47	DRB1*11:12	ESLFRAVITKKVADL	MAGE1	P43355	95	109	29.13
3327	48	DRB1*11:12	EYVIKVSARVRFFFP	MAGE1	P43355	281	295	39.57
3328	49	DRB1*11:12	FFFPSLREAALREEE	MAGE1	P43355	292	306	45.29
3329	50	DRB1*11:12	GFLLLKYRAREPVTK	MAGE1	P43355	111	125	45.81
3330	51	DRB1*11:12	ILESLFRAVITKKVA	MAGE1	P43355	93	107	32.17
3331	52	DRB1*11:12	KVLEYVIKVSARVRF	MAGE1	P43355	278	292	25.6
3332	53	DRB1*11:12	LESLFRAVITKKVAD	MAGE1	P43355	94	108	37.28
3333	54	DRB1*11:12	LEYVIKVSARVRFFF	MAGE1	P43355	280	294	34.02
3334	55	DRB1*11:12	LVGFLLLKYRAREPV	MAGE1	P43355	109	123	26.38
3335	56	DRB1*11:12	PARYEFLWGPRALAE	MAGE1	P43355	259	273	37.79
3336	57	DRB1*11:12	RFFFPSLREAALREE	MAGE1	P43355	291	305	18.8
3337	58	DRB1*11:12	RVRFFFPSLREAALR	MAGE1	P43355	289	303	14
3338	59	DRB1*11:12	RYEFLWGPRALAETS	MAGE1	P43355	261	275	34.81

3339	60	DRB1*11:12	SARVRFFFPSLREAA	MAGE1	P43355	287	301	49.09
3340	61	DRB1*11:12	SLFRAVITKKVADLV	MAGE1	P43355	96	110	32.18
3341	62	DRB1*11:12	VGFLLLKYRAREPVT	MAGE1	P43355	110	124	31.71
3342	63	DRB1*11:12	VKVLEYVIKVSARVR	MAGE1	P43355	277	291	29.38
3343	64	DRB1*11:12	VLEYVIKVSARVRFF	MAGE1	P43355	279	293	31.77
3344	65	DRB1*11:12	VRFFFPSLREAALRE	MAGE1	P43355	290	304	14.6
3345	66	DRB1*11:12	AHFLLRKYRAKELVT	MAGE4	P43358	118	132	42.25
3346	67	DRB1*11:12	ARYEFLWGPRALAET	MAGE4	P43358	268	282	33.84
3347	68	DRB1*11:12	DELAHFLLRKYRAKE	MAGE4	P43358	115	129	39.02
3348	69	DRB1*11:12	ELAHFLLRKYRAKEL	MAGE4	P43358	116	130	28.81
3349	70	DRB1*11:12	KVLEHVVRVNARVRI	MAGE4	P43358	286	300	31.5
3350	71	DRB1*11:12	LAHFLLRKYRAKELV	MAGE4	P43358	117	131	29.7
3351	72	DRB1*11:12	PARYEFLWGPRALAE	MAGE4	P43358	267	281	37.79
3352	73	DRB1*11:12	RYEFLWGPRALAETS	MAGE4	P43358	269	283	34.81
3353	74	DRB1*11:12	SYVKVLEHVVRVNAR	MAGE4	P43358	283	297	47.81
3354	75	DRB1*11:12	VDELAHFLLRKYRAK	MAGE4	P43358	114	128	43.08
3355	76	DRB1*11:12	VKVLEHVVRVNARVR	MAGE4	P43358	285	299	30.17
3356	77	DRB1*11:12	YVKVLEHVVRVNARV	MAGE4	P43358	284	298	40.41
3357	78	DRB1*11:12	RAVFLALSAQLLQAR	BAGE	Q13072	4	18	49.7
3358	79	DRB1*11:12	ASEKIFYVYMKRKYE	SSX2	Q16385	42	56	21.98
3359	80	DRB1*11:12	EKIFYVYMKRKYEAM	SSX2	Q16385	44	58	12.58
3360	81	DRB1*11:12	FYVYMKRKYEAMTKL	SSX2	Q16385	47	61	19.78
3361	82	DRB1*11:12	IFYVYMKRKYEAMTK	SSX2	Q16385	46	60	13.87
3362	83	DRB1*11:12	KIFYVYMKRKYEAMT	SSX2	Q16385	45	59	12.77
3363	84	DRB1*11:12	KRKYEAMTKLGFKAT	SSX2	Q16385	52	66	47.42
3364	85	DRB1*11:12	MKRKYEAMTKLGFKA	SSX2	Q16385	51	65	47.51
3365	86	DRB1*11:12	SEKIFYVYMKRKYEA	SSX2	Q16385	43	57	15.39
3366	1	DRB1*11:13	EVISCKLIKRATTRQ	TRP2	O75767	222	236	46.1
3367	2	DRB1*11:13	WEVISCKLIKRATTR	TRP2	O75767	221	235	44.69
3368	3	DRB1*11:13	AGLVSLLCRHKRKQL	Tyrosinase	P14679	493	507	46.93
3369	4	DRB1*11:13	ESYMVPFIPLYRNGD	Tyrosinase	P14679	423	437	47.63
3370	5	DRB1*11:13	FAYLTLAKHTISSDY	Tyrosinase	P14679	135	149	45.35
3371	6	DRB1*11:13	FFAYLTLAKHTISSD	Tyrosinase	P14679	134	148	46.72
3372	7	DRB1*11:13	KFFAYLTLAKHTISS	Tyrosinase	P14679	133	147	44.78
3373	8	DRB1*11:13	RESYMVPFIPLYRNG	Tyrosinase	P14679	422	436	47.02
3374	9	DRB1*11:13	AACDQRVLIVRRNLL	TRP1	P17643	120	134	23.87
3375	10	DRB1*11:13	ACDQRVLIVRRNLLD	TRP1	P17643	121	135	18.4
3376	11	DRB1*11:13	ALIFGTASYLIRARR	TRP1	P17643	491	505	42.55
3377	12	DRB1*11:13	CDQRVLIVRRNLLDL	TRP1	P17643	122	136	15.74

3378	13	DRB1*11:13	DQRVLIVRRNLLDLS	TRP1	P17643	123	137	15.21
3379	14	DRB1*11:13	FFPLLLFQQARAQFP	TRP1	P17643	13	27	36.89
3380	15	DRB1*11:13	FPLLLFQQARAQFPR	TRP1	P17643	14	28	30.19
3381	16	DRB1*11:13	HPLFVIATRRSEEIL	TRP1	P17643	156	170	36.5
3382	17	DRB1*11:13	PLFVIATRRSEEILG	TRP1	P17643	157	171	46.62
3383	18	DRB1*11:13	PLLLFQQARAQFPRQ	TRP1	P17643	15	29	35.94
3384	19	DRB1*11:13	QRVLIVRRNLLDLSK	TRP1	P17643	124	138	14.61
3385	20	DRB1*11:13	RVLIVRRNLLDLSKE	TRP1	P17643	125	139	19.51
3386	21	DRB1*11:13	THPLFVIATRRSEEI	TRP1	P17643	155	169	37.81
3387	22	DRB1*11:13	TTHPLFVIATRRSEE	TRP1	P17643	154	168	49.64
3388	23	DRB1*11:13	ATLRLVKRQVPLDCV	PMEL17	P40967	462	476	31.13
3389	24	DRB1*11:13	AVVLASLIYRRRLMK	PMEL17	P40967	608	622	24.83
3390	25	DRB1*11:13	DGTATLRLVKRQVPL	PMEL17	P40967	459	473	20.09
3391	26	DRB1*11:13	GTATLRLVKRQVPLD	PMEL17	P40967	460	474	20.13
3392	27	DRB1*11:13	LDGTATLRLVKRQVP	PMEL17	P40967	458	472	35.45
3393	28	DRB1*11:13	LMAVVLASLIYRRRL	PMEL17	P40967	606	620	28.16
3394	29	DRB1*11:13	MAVVLASLIYRRRLM	PMEL17	P40967	607	621	25.56
3395	30	DRB1*11:13	NKHFLRNQPLTFALQ	PMEL17	P40967	229	243	44.97
3396	31	DRB1*11:13	PVTAQVVLQAAIPLT	PMEL17	P40967	285	299	47.85
3397	32	DRB1*11:13	TAQVVLQAAIPLTSC	PMEL17	P40967	287	301	45.92
3398	33	DRB1*11:13	TATLRLVKRQVPLDC	PMEL17	P40967	461	475	23.54
3399	34	DRB1*11:13	VLASLIYRRRLMKQD	PMEL17	P40967	610	624	40.36
3400	35	DRB1*11:13	VLMAVVLASLIYRRR	PMEL17	P40967	605	619	33.79
3401	36	DRB1*11:13	VTAQVVLQAAIPLTS	PMEL17	P40967	286	300	38.25
3402	37	DRB1*11:13	VVLASLIYRRRLMKQ	PMEL17	P40967	609	623	28.73
3403	38	DRB1*11:13	ADLVGFLLLKYRARE	MAGE1	P43355	107	121	38.99
3404	39	DRB1*11:13	ARVRFFFPSLREAAL	MAGE1	P43355	288	302	47.26
3405	40	DRB1*11:13	CILESLFRAVITKKV	MAGE1	P43355	92	106	43.87
3406	41	DRB1*11:13	DLVGFLLLKYRAREP	MAGE1	P43355	108	122	39.33
3407	42	DRB1*11:13	EYVIKVSARVRFFFP	MAGE1	P43355	281	295	10.34
3408	43	DRB1*11:13	GFLLLKYRAREPVTK	MAGE1	P43355	111	125	47.19
3409	44	DRB1*11:13	ILESLFRAVITKKVA	MAGE1	P43355	93	107	41.6
3410	45	DRB1*11:13	KVLEYVIKVSARVRF	MAGE1	P43355	278	292	12.69
3411	46	DRB1*11:13	LEYVIKVSARVRFFF	MAGE1	P43355	280	294	10.4
3412	47	DRB1*11:13	LVGFLLLKYRAREPV	MAGE1	P43355	109	123	36.51
3413	48	DRB1*11:13	SARVRFFFPSLREAA	MAGE1	P43355	287	301	45.95
3414	49	DRB1*11:13	SCILESLFRAVITKK	MAGE1	P43355	91	105	43.25
3415	50	DRB1*11:13	SYVKVLEYVIKVSAR	MAGE1	P43355	275	289	40.48
3416	51	DRB1*11:13	VADLVGFLLLKYRAR	MAGE1	P43355	106	120	45.62
3417	52	DRB1*11:13	VGFLLLKYRAREPVT	MAGE1	P43355	110	124	42.09

3418	53	DRB1*11:13	VIKVSARVRFFFPSL	MAGE1	P43355	283	297	20.51
3419	54	DRB1*11:13	VKVLEYVIKVSARVR	MAGE1	P43355	277	291	20.29
3420	55	DRB1*11:13	VLEYVIKVSARVRFF	MAGE1	P43355	279	293	10.92
3421	56	DRB1*11:13	YVIKVSARVRFFFPS	MAGE1	P43355	282	296	12.91
3422	57	DRB1*11:13	YVKVLEYVIKVSARV	MAGE1	P43355	276	290	34.69
3423	58	DRB1*11:13	ARVRIAYPSLREAAL	MAGE4	P43358	296	310	48.02
3424	59	DRB1*11:13	EHVVRVNARVRIAYP	MAGE4	P43358	289	303	16.16
3425	60	DRB1*11:13	ELAHFLLRKYRAKEL	MAGE4	P43358	116	130	49.37
3426	61	DRB1*11:13	ETSYVKVLEHVVRVN	MAGE4	P43358	281	295	48.55
3427	62	DRB1*11:13	HVVRVNARVRIAYPS	MAGE4	P43358	290	304	18.31
3428	63	DRB1*11:13	KVLEHVVRVNARVRI	MAGE4	P43358	286	300	18.27
3429	64	DRB1*11:13	LEHVVRVNARVRIAY	MAGE4	P43358	288	302	14.21
3430	65	DRB1*11:13	NARVRIAYPSLREAA	MAGE4	P43358	295	309	40.95
3431	66	DRB1*11:13	RVNARVRIAYPSLRE	MAGE4	P43358	293	307	43.96
3432	67	DRB1*11:13	SYVKVLEHVVRVNAR	MAGE4	P43358	283	297	30.46
3433	68	DRB1*11:13	TSYVKVLEHVVRVNA	MAGE4	P43358	282	296	33.19
3434	69	DRB1*11:13	VKVLEHVVRVNARVR	MAGE4	P43358	285	299	24.4
3435	70	DRB1*11:13	VLEHVVRVNARVRIA	MAGE4	P43358	287	301	16.61
3436	71	DRB1*11:13	VNARVRIAYPSLREA	MAGE4	P43358	294	308	47.15
3437	72	DRB1*11:13	VRVNARVRIAYPSLR	MAGE4	P43358	292	306	36.81
3438	73	DRB1*11:13	VVRVNARVRIAYPSL	MAGE4	P43358	291	305	25.05
3439	74	DRB1*11:13	YVKVLEHVVRVNARV	MAGE4	P43358	284	298	29.8
3440	75	DRB1*11:13	GNILTIRLTAADHRQ	NY-ESO-1	P78358	130	144	44.05
3441	76	DRB1*11:13	ILTIRLTAADHRQLQ	NY-ESO-1	P78358	132	146	40.41
3442	77	DRB1*11:13	LTIRLTAADHRQLQL	NY-ESO-1	P78358	133	147	44.52
3443	78	DRB1*11:13	NILTIRLTAADHRQL	NY-ESO-1	P78358	131	145	40.62
3444	79	DRB1*11:13	SGNILTIRLTAADHR	NY-ESO-1	P78358	129	143	47.37
3445	80	DRB1*11:13	AVFLALSAQLLQARL	BAGE	Q13072	5	19	49.77
3446	81	DRB1*11:13	RAVFLALSAQLLQAR	BAGE	Q13072	4	18	38.1
3447	82	DRB1*11:13	EEWEKMKASEKIFYV	SSX2	Q16385	35	49	49.35
3448	83	DRB1*11:13	EKIFYVYMKRKYEAM	SSX2	Q16385	44	58	33.65
3449	84	DRB1*11:13	EKMKASEKIFYVYMK	SSX2	Q16385	38	52	37.62
3450	85	DRB1*11:13	EWEKMKASEKIFYVY	SSX2	Q16385	36	50	35.83
3451	86	DRB1*11:13	IFYVYMKRKYEAMTK	SSX2	Q16385	46	60	40.57
3452	87	DRB1*11:13	KIFYVYMKRKYEAMT	SSX2	Q16385	45	59	36.57
3453	88	DRB1*11:13	SEKIFYVYMKRKYEA	SSX2	Q16385	43	57	37.35
3454	89	DRB1*11:13	WEKMKASEKIFYVYM	SSX2	Q16385	37	51	31.7
3455	1	DRB1*11:14	KKPPVIRQNIHSLSP	TRP2	O75767	121	135	47.85
3456	2	DRB1*11:14	KPPVIRQNIHSLSPQ	TRP2	O75767	122	136	47.66

3457	3	DRB1*11:14	RKKPPVIRQNIHSLS	TRP2	O75767	120	134	48.91
3458	4	DRB1*11:14	ELKELINNELSHFLE	S100	P04271	30	44	36.11
3459	5	DRB1*11:14	LKELINNELSHFLEE	S100	P04271	31	45	45.36
3460	6	DRB1*11:14	SELKELINNELSHFL	S100	P04271	29	43	34.41
3461	7	DRB1*11:14	ALHIYMNGTMSQVQG	Tyrosinase	P14679	365	379	24.22
3462	8	DRB1*11:14	CQNILLSNAPLGPQF	Tyrosinase	P14679	55	69	28.2
3463	9	DRB1*11:14	HNALHIYMNGTMSQV	Tyrosinase	P14679	363	377	23.28
3464	10	DRB1*11:14	KHTISSDYVIPIGTY	Tyrosinase	P14679	142	156	46.16
3465	11	DRB1*11:14	LHIYMNGTMSQVQGS	Tyrosinase	P14679	366	380	29.98
3466	12	DRB1*11:14	MHNALHIYMNGTMSQ	Tyrosinase	P14679	362	376	39.88
3467	13	DRB1*11:14	NALHIYMNGTMSQVQ	Tyrosinase	P14679	364	378	21.33
3468	14	DRB1*11:14	NILLSNAPLGPQFPF	Tyrosinase	P14679	57	71	47.26
3469	15	DRB1*11:14	QNILLSNAPLGPQFP	Tyrosinase	P14679	56	70	28.71
3470	16	DRB1*11:14	SCQNILLSNAPLGPQ	Tyrosinase	P14679	54	68	41.65
3471	17	DRB1*11:14	DGPIRRNPAGNVARP	TRP1	P17643	308	322	36.78
3472	18	DRB1*11:14	DTPPFYSNSTNSFRN	TRP1	P17643	343	357	25.79
3473	19	DRB1*11:14	EDGPIRRNPAGNVAR	TRP1	P17643	307	321	32.26
3474	20	DRB1*11:14	FDTPPFYSNSTNSFR	TRP1	P17643	342	356	28.28
3475	21	DRB1*11:14	GPIRRNPAGNVARPM	TRP1	P17643	309	323	35.1
3476	22	DRB1*11:14	GQTHLSPNDPIFVLL	TRP1	P17643	389	403	34.38
3477	23	DRB1*11:14	PFYSNSTNSFRNTVE	TRP1	P17643	346	360	39.62
3478	24	DRB1*11:14	PIRRNPAGNVARPMV	TRP1	P17643	310	324	43.15
3479	25	DRB1*11:14	PPFYSNSTNSFRNTV	TRP1	P17643	345	359	31.34
3480	26	DRB1*11:14	QTHLSPNDPIFVLLH	TRP1	P17643	390	404	32.73
3481	27	DRB1*11:14	TEDGPIRRNPAGNVA	TRP1	P17643	306	320	45.36
3482	28	DRB1*11:14	THLSPNDPIFVLLHT	TRP1	P17643	391	405	43.62
3483	29	DRB1*11:14	TPPFYSNSTNSFRNT	TRP1	P17643	344	358	24.09
3484	30	DRB1*11:14	VALIFGTASYLIRAR	TRP1	P17643	490	504	45.96
3485	31	DRB1*11:14	AQVVLQAAIPLTSCG	PMEL17	P40967	288	302	43.39
3486	32	DRB1*11:14	ASFSIALNFPGSQKV	PMEL17	P40967	82	96	34.8
3487	33	DRB1*11:14	AVIGALLAVGATKVP	PMEL17	P40967	13	27	46.91
3488	34	DRB1*11:14	DGGNKHFLRNQPLTF	PMEL17	P40967	226	240	18.26
3489	35	DRB1*11:14	DGPTLIGANASFSIA	PMEL17	P40967	73	87	24.48
3490	36	DRB1*11:14	DGQVIWVNNTIINGS	PMEL17	P40967	99	113	17.49
3491	37	DRB1*11:14	EVSIVVLSGTTAAQV	PMEL17	P40967	404	418	49.09
3492	38	DRB1*11:14	FSIALNFPGSQKVLP	PMEL17	P40967	84	98	37.57
3493	39	DRB1*11:14	GGNKHFLRNQPLTFA	PMEL17	P40967	227	241	11.13
3494	40	DRB1*11:14	GNKHFLRNQPLTFAL	PMEL17	P40967	228	242	8.61
3495	41	DRB1*11:14	GPTLIGANASFSIAL	PMEL17	P40967	74	88	17.01
3496	42	DRB1*11:14	GPVTAQVVLQAAIPL	PMEL17	P40967	284	298	35.55

3497	43	DRB1*11:14	GQVIWVNNTIINGSQ	PMEL17	P40967	100	114	16.48
3498	44	DRB1*11:14	HFLRNQPLTFALQLH	PMEL17	P40967	231	245	17.65
3499	45	DRB1*11:14	IGALLAVGATKVPRN	PMEL17	P40967	15	29	34.24
3500	46	DRB1*11:14	KHFLRNQPLTFALQL	PMEL17	P40967	230	244	9.79
3501	47	DRB1*11:14	LIGANASFSIALNFP	PMEL17	P40967	77	91	36.78
3502	48	DRB1*11:14	LPDGQVIWVNNTIIN	PMEL17	P40967	97	111	23.66
3503	49	DRB1*11:14	NASFSIALNFPGSQK	PMEL17	P40967	81	95	49.03
3504	50	DRB1*11:14	NDGPTLIGANASFSI	PMEL17	P40967	72	86	44.44
3505	51	DRB1*11:14	NKHFLRNQPLTFALQ	PMEL17	P40967	229	243	8.69
3506	52	DRB1*11:14	PDGQVIWVNNTIING	PMEL17	P40967	98	112	19.68
3507	53	DRB1*11:14	PTLIGANASFSIALN	PMEL17	P40967	75	89	17.72
3508	54	DRB1*11:14	PVTAQVVLQAAIPLT	PMEL17	P40967	285	299	24.67
3509	55	DRB1*11:14	QVIWVNNTIINGSQV	PMEL17	P40967	101	115	18.45
3510	56	DRB1*11:14	SFSIALNFPGSQKVL	PMEL17	P40967	83	97	27.92
3511	57	DRB1*11:14	TAQVVLQAAIPLTSC	PMEL17	P40967	287	301	29.07
3512	58	DRB1*11:14	TLIGANASFSIALNF	PMEL17	P40967	76	90	20.23
3513	59	DRB1*11:14	VIGALLAVGATKVPR	PMEL17	P40967	14	28	32.72
3514	60	DRB1*11:14	VIWVNNTIINGSQVW	PMEL17	P40967	102	116	31.07
3515	61	DRB1*11:14	VSIVVLSGTTAAQVT	PMEL17	P40967	405	419	43.21
3516	62	DRB1*11:14	VTAQVVLQAAIPLTS	PMEL17	P40967	286	300	20.55
3517	63	DRB1*11:14	EYVIKVSARVRFFFP	MAGE1	P43355	281	295	26.42
3518	64	DRB1*11:14	KVLEYVIKVSARVRF	MAGE1	P43355	278	292	29.26
3519	65	DRB1*11:14	LEYVIKVSARVRFFF	MAGE1	P43355	280	294	24.27
3520	66	DRB1*11:14	VKVLEYVIKVSARVR	MAGE1	P43355	277	291	46.25
3521	67	DRB1*11:14	VLEYVIKVSARVRFF	MAGE1	P43355	279	293	25.89
3522	68	DRB1*11:14	EHVVRVNARVRIAYP	MAGE4	P43358	289	303	20.84
3523	69	DRB1*11:14	ESLFREALSNKVDEL	MAGE4	P43358	103	117	46.34
3524	70	DRB1*11:14	HVVRVNARVRIAYPS	MAGE4	P43358	290	304	23.63
3525	71	DRB1*11:14	KVLEHVVRVNARVRI	MAGE4	P43358	286	300	26.57
3526	72	DRB1*11:14	LEHVVRVNARVRIAY	MAGE4	P43358	288	302	17.31
3527	73	DRB1*11:14	VLEHVVRVNARVRIA	MAGE4	P43358	287	301	20.72
3528	74	DRB1*11:14	VVRVNARVRIAYPSL	MAGE4	P43358	291	305	32.87
3529	75	DRB1*11:14	DHRQLQLSISSCLQQ	NY-ESO-1	P78358	141	155	42.47
3530	76	DRB1*11:14	HRQLQLSISSCLQQL	NY-ESO-1	P78358	142	156	36.44
3531	77	DRB1*11:14	KEFTVSGNILTIRLT	NY-ESO-1	P78358	124	138	36.33
3532	78	DRB1*11:14	LKEFTVSGNILTIRL	NY-ESO-1	P78358	123	137	28.04
3533	79	DRB1*11:14	LLKEFTVSGNILTIR	NY-ESO-1	P78358	122	136	29.99
3534	80	DRB1*11:14	VLLKEFTVSGNILTI	NY-ESO-1	P78358	121	135	49.15
3535	81	DRB1*11:14	RAVFLALSAQLLQAR	BAGE	Q13072	4	18	48.97

3536	1	DRB1*11:19	GNKHFLRNQPLTFAL	PMEL17	P40967	228	242	39.67
3537	2	DRB1*11:19	KHFLRNQPLTFALQL	PMEL17	P40967	230	244	46.32
3538	3	DRB1*11:19	NKHFLRNQPLTFALQ	PMEL17	P40967	229	243	38.52
3539	4	DRB1*11:19	KVLEYVIKVSARVRF	MAGE1	P43355	278	292	40.05
3540	5	DRB1*11:19	LEYVIKVSARVRFFF	MAGE1	P43355	280	294	43.02
3541	6	DRB1*11:19	VLEYVIKVSARVRFF	MAGE1	P43355	279	293	39.25
3542	7	DRB1*11:19	RAVFLALSAQLLQAR	BAGE	Q13072	4	18	42.46
3543	1	DRB1*11:27	RVRFFFPSLREAALR	MAGE1	P43355	289	303	42.89
3544	2	DRB1*11:27	VRFFFPSLREAALRE	MAGE1	P43355	290	304	44.98
3545	3	DRB1*11:27	EKIFYVYMKRKYEAM	SSX2	Q16385	44	58	39.9
3546	4	DRB1*11:27	KIFYVYMKRKYEAMT	SSX2	Q16385	45	59	39.72
3547	1	DRB1*11:28	EVISCKLIKRATTRQ	TRP2	O75767	222	236	30.92
3548	2	DRB1*11:28	RKFFHRTCKCTGNFA	TRP2	O75767	88	102	39.55
3549	3	DRB1*11:28	VISCKLIKRATTRQP	TRP2	O75767	223	237	33.86
3550	4	DRB1*11:28	WEVISCKLIKRATTR	TRP2	O75767	221	235	36.89
3551	5	DRB1*11:28	YYYRFVIGLRVWQWE	TRP2	O75767	208	222	49.95
3552	6	DRB1*11:28	DKFFAYLTLAKHTIS	Tyrosinase	P14679	132	146	26.01
3553	7	DRB1*11:28	FAYLTLAKHTISSDY	Tyrosinase	P14679	135	149	35.24
3554	8	DRB1*11:28	FFAYLTLAKHTISSD	Tyrosinase	P14679	134	148	28.57
3555	9	DRB1*11:28	KDKFFAYLTLAKHTI	Tyrosinase	P14679	131	145	33.33
3556	10	DRB1*11:28	KFFAYLTLAKHTISS	Tyrosinase	P14679	133	147	22.47
3557	11	DRB1*11:28	MVPFIPLYRNGDFFI	Tyrosinase	P14679	426	440	32.73
3558	12	DRB1*11:28	VPFIPLYRNGDFFIS	Tyrosinase	P14679	427	441	41.52
3559	13	DRB1*11:28	YMVPFIPLYRNGDFF	Tyrosinase	P14679	425	439	42.2
3560	14	DRB1*11:28	ASYLIRARRSMDEAN	TRP1	P17643	497	511	46.19
3561	15	DRB1*11:28	FVWTHYYSVKKTFLG	TRP1	P17643	188	202	47.59
3562	16	DRB1*11:28	HPLFVIATRRSEEIL	TRP1	P17643	156	170	13.74
3563	17	DRB1*11:28	LFVIATRRSEEILGP	TRP1	P17643	158	172	24.07
3564	18	DRB1*11:28	PLFVIATRRSEEILG	TRP1	P17643	157	171	15.42
3565	19	DRB1*11:28	RTTHPLFVIATRRSE	TRP1	P17643	153	167	24.44
3566	20	DRB1*11:28	TASYLIRARRSMDEA	TRP1	P17643	496	510	39.65
3567	21	DRB1*11:28	THPLFVIATRRSEEI	TRP1	P17643	155	169	15.19
3568	22	DRB1*11:28	THYYSVKKTFLGVGQ	TRP1	P17643	191	205	37.63
3569	23	DRB1*11:28	TTHPLFVIATRRSEE	TRP1	P17643	154	168	19.39
3570	24	DRB1*11:28	VWTHYYSVKKTFLGV	TRP1	P17643	189	203	31.01
3571	25	DRB1*11:28	WTHYYSVKKTFLGVG	TRP1	P17643	190	204	32.76
3572	26	DRB1*11:28	ANASFSIALNFPGSQ	PMEL17	P40967	80	94	49.44
3573	27	DRB1*11:28	ASFSIALNFPGSQKV	PMEL17	P40967	82	96	42.13

3574	28	DRB1*11:28	ATLRLVKRQVPLDCV	PMEL17	P40967	462	476	32.58
3575	29	DRB1*11:28	CQLVLHQILKGGSGT	PMEL17	P40967	550	564	45.41
3576	30	DRB1*11:28	DGTATLRLVKRQVPL	PMEL17	P40967	459	473	18.27
3577	31	DRB1*11:28	GSRSYVPLAHSSSAF	PMEL17	P40967	193	207	39.7
3578	32	DRB1*11:28	GTATLRLVKRQVPLD	PMEL17	P40967	460	474	20.16
3579	33	DRB1*11:28	KRSFVYVWKTWGQYW	PMEL17	P40967	146	160	17.94
3580	34	DRB1*11:28	NASFSIALNFPGSQK	PMEL17	P40967	81	95	33.06
3581	35	DRB1*11:28	QKRSFVYVWKTWGQY	PMEL17	P40967	145	159	18.3
3582	36	DRB1*11:28	QLVLHQILKGGSGTY	PMEL17	P40967	551	565	36.77
3583	37	DRB1*11:28	RSFVYVWKTWGQYWQ	PMEL17	P40967	147	161	30.25
3584	38	DRB1*11:28	RSYVPLAHSSSAFTI	PMEL17	P40967	195	209	42.32
3585	39	DRB1*11:28	SQKRSFVYVWKTWGQ	PMEL17	P40967	144	158	21.74
3586	40	DRB1*11:28	SRSYVPLAHSSSAFT	PMEL17	P40967	194	208	39.52
3587	41	DRB1*11:28	TATLRLVKRQVPLDC	PMEL17	P40967	461	475	23.06
3588	42	DRB1*11:28	WSQKRSFVYVWKTWG	PMEL17	P40967	143	157	41.25
3589	43	DRB1*11:28	ADLVGFLLLKYRARE	MAGE1	P43355	107	121	34.07
3590	44	DRB1*11:28	ARVRFFFPSLREAAL	MAGE1	P43355	288	302	21.89
3591	45	DRB1*11:28	ARYEFLWGPRALAET	MAGE1	P43355	260	274	33.84
3592	46	DRB1*11:28	DLVGFLLLKYRAREP	MAGE1	P43355	108	122	28.93
3593	47	DRB1*11:28	ESLFRAVITKKVADL	MAGE1	P43355	95	109	29.13
3594	48	DRB1*11:28	EYVIKVSARVRFFFP	MAGE1	P43355	281	295	39.57
3595	49	DRB1*11:28	FFFPSLREAALREEE	MAGE1	P43355	292	306	45.29
3596	50	DRB1*11:28	GFLLLKYRAREPVTK	MAGE1	P43355	111	125	45.81
3597	51	DRB1*11:28	ILESLFRAVITKKVA	MAGE1	P43355	93	107	32.17
3598	52	DRB1*11:28	KVLEYVIKVSARVRF	MAGE1	P43355	278	292	25.6
3599	53	DRB1*11:28	LESLFRAVITKKVAD	MAGE1	P43355	94	108	37.28
3600	54	DRB1*11:28	LEYVIKVSARVRFFF	MAGE1	P43355	280	294	34.02
3601	55	DRB1*11:28	LVGFLLLKYRAREPV	MAGE1	P43355	109	123	26.38
3602	56	DRB1*11:28	PARYEFLWGPRALAE	MAGE1	P43355	259	273	37.79
3603	57	DRB1*11:28	RFFFPSLREAALREE	MAGE1	P43355	291	305	18.8
3604	58	DRB1*11:28	RVRFFFPSLREAALR	MAGE1	P43355	289	303	14
3605	59	DRB1*11:28	RYEFLWGPRALAETS	MAGE1	P43355	261	275	34.81
3606	60	DRB1*11:28	SARVRFFFPSLREAA	MAGE1	P43355	287	301	49.09
3607	61	DRB1*11:28	SLFRAVITKKVADLV	MAGE1	P43355	96	110	32.18
3608	62	DRB1*11:28	VGFLLLKYRAREPVT	MAGE1	P43355	110	124	31.71
3609	63	DRB1*11:28	VKVLEYVIKVSARVR	MAGE1	P43355	277	291	29.38
3610	64	DRB1*11:28	VLEYVIKVSARVRFF	MAGE1	P43355	279	293	31.77
3611	65	DRB1*11:28	VRFFFPSLREAALRE	MAGE1	P43355	290	304	14.6
3612	66	DRB1*11:28	AHFLLRKYRAKELVT	MAGE4	P43358	118	132	42.25
3613	67	DRB1*11:28	ARYEFLWGPRALAET	MAGE4	P43358	268	282	33.84

3614	68	DRB1*11:28	DELAHFLLRKYRAKE	MAGE4	P43358	115	129	39.02
3615	69	DRB1*11:28	ELAHFLLRKYRAKEL	MAGE4	P43358	116	130	28.81
3616	70	DRB1*11:28	KVLEHVVRVNARVRI	MAGE4	P43358	286	300	31.5
3617	71	DRB1*11:28	LAHFLLRKYRAKELV	MAGE4	P43358	117	131	29.7
3618	72	DRB1*11:28	PARYEFLWGPRALAE	MAGE4	P43358	267	281	37.79
3619	73	DRB1*11:28	RYEFLWGPRALAETS	MAGE4	P43358	269	283	34.81
3620	74	DRB1*11:28	SYVKVLEHVVRVNAR	MAGE4	P43358	283	297	47.81
3621	75	DRB1*11:28	VDELAHFLLRKYRAK	MAGE4	P43358	114	128	43.08
3622	76	DRB1*11:28	VKVLEHVVRVNARVR	MAGE4	P43358	285	299	30.17
3623	77	DRB1*11:28	YVKVLEHVVRVNARV	MAGE4	P43358	284	298	40.41
3624	78	DRB1*11:28	RAVFLALSAQLLQAR	BAGE	Q13072	4	18	49.7
3625	79	DRB1*11:28	ASEKIFYVYMKRKYE	SSX2	Q16385	42	56	21.98
3626	80	DRB1*11:28	EKIFYVYMKRKYEAM	SSX2	Q16385	44	58	12.58
3627	81	DRB1*11:28	FYVYMKRKYEAMTKL	SSX2	Q16385	47	61	19.78
3628	82	DRB1*11:28	IFYVYMKRKYEAMTK	SSX2	Q16385	46	60	13.87
3629	83	DRB1*11:28	KIFYVYMKRKYEAMT	SSX2	Q16385	45	59	12.77
3630	84	DRB1*11:28	KRKYEAMTKLGFKAT	SSX2	Q16385	52	66	47.42
3631	85	DRB1*11:28	MKRKYEAMTKLGFKA	SSX2	Q16385	51	65	47.51
3632	86	DRB1*11:28	SEKIFYVYMKRKYEA	SSX2	Q16385	43	57	15.39
3633	1	DRB1*11:29	EVISCKLIKRATTRQ	TRP2	O75767	222	236	30.92
3634	2	DRB1*11:29	RKFFHRTCKCTGNFA	TRP2	O75767	88	102	39.55
3635	3	DRB1*11:29	VISCKLIKRATTRQP	TRP2	O75767	223	237	33.86
3636	4	DRB1*11:29	WEVISCKLIKRATTR	TRP2	O75767	221	235	36.89
3637	5	DRB1*11:29	YYYRFVIGLRVWQWE	TRP2	O75767	208	222	49.95
3638	6	DRB1*11:29	DKFFAYLTLAKHTIS	Tyrosinase	P14679	132	146	26.01
3639	7	DRB1*11:29	FAYLTLAKHTISSDY	Tyrosinase	P14679	135	149	35.24
3640	8	DRB1*11:29	FFAYLTLAKHTISSD	Tyrosinase	P14679	134	148	28.57
3641	9	DRB1*11:29	KDKFFAYLTLAKHTI	Tyrosinase	P14679	131	145	33.33
3642	10	DRB1*11:29	KFFAYLTLAKHTISS	Tyrosinase	P14679	133	147	22.47
3643	11	DRB1*11:29	MVPFIPLYRNGDFFI	Tyrosinase	P14679	426	440	32.73
3644	12	DRB1*11:29	VPFIPLYRNGDFFIS	Tyrosinase	P14679	427	441	41.52
3645	13	DRB1*11:29	YMVPFIPLYRNGDFF	Tyrosinase	P14679	425	439	42.2
3646	14	DRB1*11:29	ASYLIRARRSMDEAN	TRP1	P17643	497	511	46.19
3647	15	DRB1*11:29	FVWTHYYSVKKTFLG	TRP1	P17643	188	202	47.59
3648	16	DRB1*11:29	HPLFVIATRRSEEIL	TRP1	P17643	156	170	13.74
3649	17	DRB1*11:29	LFVIATRRSEEILGP	TRP1	P17643	158	172	24.07
3650	18	DRB1*11:29	PLFVIATRRSEEILG	TRP1	P17643	157	171	15.42
3651	19	DRB1*11:29	RTTHPLFVIATRRSE	TRP1	P17643	153	167	24.44
3652	20	DRB1*11:29	TASYLIRARRSMDEA	TRP1	P17643	496	510	39.65

3653	21	DRB1*11:29	THPLFVIATRRSEEI	TRP1	P17643	155	169	15.19
3654	22	DRB1*11:29	THYYSVKKTFLGVGQ	TRP1	P17643	191	205	37.63
3655	23	DRB1*11:29	TTHPLFVIATRRSEE	TRP1	P17643	154	168	19.39
3656	24	DRB1*11:29	VWTHYYSVKKTFLGV	TRP1	P17643	189	203	31.01
3657	25	DRB1*11:29	WTHYYSVKKTFLGVG	TRP1	P17643	190	204	32.76
3658	26	DRB1*11:29	ANASFSIALNFPGSQ	PMEL17	P40967	80	94	49.44
3659	27	DRB1*11:29	ASFSIALNFPGSQKV	PMEL17	P40967	82	96	42.13
3660	28	DRB1*11:29	ATLRLVKRQVPLDCV	PMEL17	P40967	462	476	32.58
3661	29	DRB1*11:29	CQLVLHQILKGGSGT	PMEL17	P40967	550	564	45.41
3662	30	DRB1*11:29	DGTATLRLVKRQVPL	PMEL17	P40967	459	473	18.27
3663	31	DRB1*11:29	GSRSYVPLAHSSSAF	PMEL17	P40967	193	207	39.7
3664	32	DRB1*11:29	GTATLRLVKRQVPLD	PMEL17	P40967	460	474	20.16
3665	33	DRB1*11:29	KRSFVYVWKTWGQYW	PMEL17	P40967	146	160	17.94
3666	34	DRB1*11:29	NASFSIALNFPGSQK	PMEL17	P40967	81	95	33.06
3667	35	DRB1*11:29	QKRSFVYVWKTWGQY	PMEL17	P40967	145	159	18.3
3668	36	DRB1*11:29	QLVLHQILKGGSGTY	PMEL17	P40967	551	565	36.77
3669	37	DRB1*11:29	RSFVYVWKTWGQYWQ	PMEL17	P40967	147	161	30.25
3670	38	DRB1*11:29	RSYVPLAHSSSAFTI	PMEL17	P40967	195	209	42.32
3671	39	DRB1*11:29	SQKRSFVYVWKTWGQ	PMEL17	P40967	144	158	21.74
3672	40	DRB1*11:29	SRSYVPLAHSSSAFT	PMEL17	P40967	194	208	39.52
3673	41	DRB1*11:29	TATLRLVKRQVPLDC	PMEL17	P40967	461	475	23.06
3674	42	DRB1*11:29	WSQKRSFVYVWKTWG	PMEL17	P40967	143	157	41.25
3675	43	DRB1*11:29	ADLVGFLLLKYRARE	MAGE1	P43355	107	121	34.07
3676	44	DRB1*11:29	ARVRFFFPSLREAAL	MAGE1	P43355	288	302	21.89
3677	45	DRB1*11:29	ARYEFLWGPRALAET	MAGE1	P43355	260	274	33.84
3678	46	DRB1*11:29	DLVGFLLLKYRAREP	MAGE1	P43355	108	122	28.93
3679	47	DRB1*11:29	ESLFRAVITKKVADL	MAGE1	P43355	95	109	29.13
3680	48	DRB1*11:29	EYVIKVSARVRFFFP	MAGE1	P43355	281	295	39.57
3681	49	DRB1*11:29	FFFPSLREAALREEE	MAGE1	P43355	292	306	45.29
3682	50	DRB1*11:29	GFLLLKYRAREPVTK	MAGE1	P43355	111	125	45.81
3683	51	DRB1*11:29	ILESLFRAVITKKVA	MAGE1	P43355	93	107	32.17
3684	52	DRB1*11:29	KVLEYVIKVSARVRF	MAGE1	P43355	278	292	25.6
3685	53	DRB1*11:29	LESLFRAVITKKVAD	MAGE1	P43355	94	108	37.28
3686	54	DRB1*11:29	LEYVIKVSARVRFFF	MAGE1	P43355	280	294	34.02
3687	55	DRB1*11:29	LVGFLLLKYRAREPV	MAGE1	P43355	109	123	26.38
3688	56	DRB1*11:29	PARYEFLWGPRALAE	MAGE1	P43355	259	273	37.79
3689	57	DRB1*11:29	RFFFPSLREAALREE	MAGE1	P43355	291	305	18.8
3690	58	DRB1*11:29	RVRFFFPSLREAALR	MAGE1	P43355	289	303	14
3691	59	DRB1*11:29	RYEFLWGPRALAETS	MAGE1	P43355	261	275	34.81
3692	60	DRB1*11:29	SARVRFFFPSLREAA	MAGE1	P43355	287	301	49.09

3693	61	DRB1*11:29	SLFRAVITKKVADLV	MAGE1	P43355	96	110	32.18
3694	62	DRB1*11:29	VGFLLLKYRAREPVT	MAGE1	P43355	110	124	31.71
3695	63	DRB1*11:29	VKVLEYVIKVSARVR	MAGE1	P43355	277	291	29.38
3696	64	DRB1*11:29	VLEYVIKVSARVRFF	MAGE1	P43355	279	293	31.77
3697	65	DRB1*11:29	VRFFFPSLREAALRE	MAGE1	P43355	290	304	14.6
3698	66	DRB1*11:29	AHFLLRKYRAKELVT	MAGE4	P43358	118	132	42.25
3699	67	DRB1*11:29	ARYEFLWGPRALAET	MAGE4	P43358	268	282	33.84
3700	68	DRB1*11:29	DELAHFLLRKYRAKE	MAGE4	P43358	115	129	39.02
3701	69	DRB1*11:29	ELAHFLLRKYRAKEL	MAGE4	P43358	116	130	28.81
3702	70	DRB1*11:29	KVLEHVVRVNARVRI	MAGE4	P43358	286	300	31.5
3703	71	DRB1*11:29	LAHFLLRKYRAKELV	MAGE4	P43358	117	131	29.7
3704	72	DRB1*11:29	PARYEFLWGPRALAE	MAGE4	P43358	267	281	37.79
3705	73	DRB1*11:29	RYEFLWGPRALAETS	MAGE4	P43358	269	283	34.81
3706	74	DRB1*11:29	SYVKVLEHVVRVNAR	MAGE4	P43358	283	297	47.81
3707	75	DRB1*11:29	VDELAHFLLRKYRAK	MAGE4	P43358	114	128	43.08
3708	76	DRB1*11:29	VKVLEHVVRVNARVR	MAGE4	P43358	285	299	30.17
3709	77	DRB1*11:29	YVKVLEHVVRVNARV	MAGE4	P43358	284	298	40.41
3710	78	DRB1*11:29	RAVFLALSAQLLQAR	BAGE	Q13072	4	18	49.7
3711	79	DRB1*11:29	ASEKIFYVYMKRKYE	SSX2	Q16385	42	56	21.98
3712	80	DRB1*11:29	EKIFYVYMKRKYEAM	SSX2	Q16385	44	58	12.58
3713	81	DRB1*11:29	FYVYMKRKYEAMTKL	SSX2	Q16385	47	61	19.78
3714	82	DRB1*11:29	IFYVYMKRKYEAMTK	SSX2	Q16385	46	60	13.87
3715	83	DRB1*11:29	KIFYVYMKRKYEAMT	SSX2	Q16385	45	59	12.77
3716	84	DRB1*11:29	KRKYEAMTKLGFKAT	SSX2	Q16385	52	66	47.42
3717	85	DRB1*11:29	MKRKYEAMTKLGFKA	SSX2	Q16385	51	65	47.51
3718	86	DRB1*11:29	SEKIFYVYMKRKYEA	SSX2	Q16385	43	57	15.39
3719	1	DRB1*11:37	KFFAYLTLAKHTISS	Tyrosinase	P14679	133	147	45.92
3720	2	DRB1*11:37	HPLFVIATRRSEEIL	TRP1	P17643	156	170	29.24
3721	3	DRB1*11:37	PLFVIATRRSEEILG	TRP1	P17643	157	171	34.19
3722	4	DRB1*11:37	THPLFVIATRRSEEI	TRP1	P17643	155	169	33.39
3723	5	DRB1*11:37	TTHPLFVIATRRSEE	TRP1	P17643	154	168	45.06
3724	6	DRB1*11:37	KRSFVYVWKTWGQYW	PMEL17	P40967	146	160	39.23
3725	7	DRB1*11:37	QKRSFVYVWKTWGQY	PMEL17	P40967	145	159	39.89
3726	8	DRB1*11:37	SQKRSFVYVWKTWGQ	PMEL17	P40967	144	158	49.54
3727	9	DRB1*11:37	ARVRFFFPSLREAAL	MAGE1	P43355	288	302	35.21
3728	10	DRB1*11:37	RFFFPSLREAALREE	MAGE1	P43355	291	305	31.49
3729	11	DRB1*11:37	RVRFFFPSLREAALR	MAGE1	P43355	289	303	22.26
3730	12	DRB1*11:37	VRFFFPSLREAALRE	MAGE1	P43355	290	304	23.67
3731	13	DRB1*11:37	EKIFYVYMKRKYEAM	SSX2	Q16385	44	58	28.09

3732	14	DRB1*11:37	IFYVYMKRKYEAMTK	SSX2	Q16385	46	60	31.32
3733	15	DRB1*11:37	KIFYVYMKRKYEAMT	SSX2	Q16385	45	59	27.34
3734	16	DRB1*11:37	SEKIFYVYMKRKYEA	SSX2	Q16385	43	57	37.23
3735	1	DRB1*11:42	EVISCKLIKRATTRQ	TRP2	O75767	222	236	22.36
3736	2	DRB1*11:42	FLGALDLAKKRVHPD	TRP2	O75767	141	155	45.97
3737	3	DRB1*11:42	LGALDLAKKRVHPDY	TRP2	O75767	142	156	38.96
3738	4	DRB1*11:42	QFLGALDLAKKRVHP	TRP2	O75767	140	154	41.77
3739	5	DRB1*11:42	QWEVISCKLIKRATT	TRP2	O75767	220	234	31.2
3740	6	DRB1*11:42	VISCKLIKRATTRQP	TRP2	O75767	223	237	25.2
3741	7	DRB1*11:42	WEVISCKLIKRATTR	TRP2	O75767	221	235	23.84
3742	8	DRB1*11:42	WQWEVISCKLIKRAT	TRP2	O75767	219	233	47.17
3743	9	DRB1*11:42	AGLVSLLCRHKRKQL	Tyrosinase	P14679	493	507	22.26
3744	10	DRB1*11:42	ALLAGLVSLLCRHKR	Tyrosinase	P14679	490	504	41.77
3745	11	DRB1*11:42	DKFFAYLTLAKHTIS	Tyrosinase	P14679	132	146	43.99
3746	12	DRB1*11:42	ESYMVPFIPLYRNGD	Tyrosinase	P14679	423	437	45.74
3747	13	DRB1*11:42	FAYLTLAKHTISSDY	Tyrosinase	P14679	135	149	31.03
3748	14	DRB1*11:42	FFAYLTLAKHTISSD	Tyrosinase	P14679	134	148	33.14
3749	15	DRB1*11:42	GLVSLLCRHKRKQLP	Tyrosinase	P14679	494	508	28.6
3750	16	DRB1*11:42	IKSYLEQASRIWSWL	Tyrosinase	P14679	464	478	49.53
3751	17	DRB1*11:42	KFFAYLTLAKHTISS	Tyrosinase	P14679	133	147	31.66
3752	18	DRB1*11:42	KSYLEQASRIWSWLL	Tyrosinase	P14679	465	479	49.02
3753	19	DRB1*11:42	LAGLVSLLCRHKRKQ	Tyrosinase	P14679	492	506	24.46
3754	20	DRB1*11:42	LLAGLVSLLCRHKRK	Tyrosinase	P14679	491	505	27.59
3755	21	DRB1*11:42	LVSLLCRHKRKQLPE	Tyrosinase	P14679	495	509	46.08
3756	22	DRB1*11:42	NRESYMVPFIPLYRN	Tyrosinase	P14679	421	435	49.18
3757	23	DRB1*11:42	RESYMVPFIPLYRNG	Tyrosinase	P14679	422	436	43.98
3758	24	DRB1*11:42	SYMVPFIPLYRNGDF	Tyrosinase	P14679	424	438	44.75
3759	25	DRB1*11:42	AACDQRVLIVRRNLL	TRP1	P17643	120	134	24.06
3760	26	DRB1*11:42	ACDQRVLIVRRNLLD	TRP1	P17643	121	135	17.73
3761	27	DRB1*11:42	ALIFGTASYLIRARR	TRP1	P17643	491	505	40.56
3762	28	DRB1*11:42	ASYLIRARRSMDEAN	TRP1	P17643	497	511	29.06
3763	29	DRB1*11:42	CDQRVLIVRRNLLDL	TRP1	P17643	122	136	15.47
3764	30	DRB1*11:42	DPAVRSLHNLAHLFL	TRP1	P17643	370	384	36.14
3765	31	DRB1*11:42	DQRVLIVRRNLLDLS	TRP1	P17643	123	137	14.53
3766	32	DRB1*11:42	EKNHFVRALDMAKRT	TRP1	P17643	140	154	47.57
3767	33	DRB1*11:42	FFPLLLFQQARAQFP	TRP1	P17643	13	27	28.09
3768	34	DRB1*11:42	FGTASYLIRARRSMD	TRP1	P17643	494	508	26.27
3769	35	DRB1*11:42	FPLLLFQQARAQFPR	TRP1	P17643	14	28	22.15
3770	36	DRB1*11:42	FVRALDMAKRTTHPL	TRP1	P17643	144	158	38.2

3771	37	DRB1*11:42	GTASYLIRARRSMDE	TRP1	P17643	495	509	26.94
3772	38	DRB1*11:42	HFVRALDMAKRTTHP	TRP1	P17643	143	157	40.09
3773	39	DRB1*11:42	HPLFVIATRRSEEIL	TRP1	P17643	156	170	15.43
3774	40	DRB1*11:42	IFFPLLLFQQARAQF	TRP1	P17643	12	26	39.05
3775	41	DRB1*11:42	IFGTASYLIRARRSM	TRP1	P17643	493	507	26.88
3776	42	DRB1*11:42	KNHFVRALDMAKRTT	TRP1	P17643	141	155	44.18
3777	43	DRB1*11:42	KRTTHPLFVIATRRS	TRP1	P17643	152	166	43.05
3778	44	DRB1*11:42	LFVIATRRSEEILGP	TRP1	P17643	158	172	45.56
3779	45	DRB1*11:42	LIFGTASYLIRARRS	TRP1	P17643	492	506	31.15
3780	46	DRB1*11:42	LLLFQQARAQFPRQC	TRP1	P17643	16	30	41.87
3781	47	DRB1*11:42	NHFVRALDMAKRTTH	TRP1	P17643	142	156	41.14
3782	48	DRB1*11:42	PAVRSLHNLAHLFLN	TRP1	P17643	371	385	36.94
3783	49	DRB1*11:42	PLFVIATRRSEEILG	TRP1	P17643	157	171	22.73
3784	50	DRB1*11:42	PLLLFQQARAQFPRQ	TRP1	P17643	15	29	27.38
3785	51	DRB1*11:42	QRVLIVRRNLLDLSK	TRP1	P17643	124	138	14.6
3786	52	DRB1*11:42	RTTHPLFVIATRRSE	TRP1	P17643	153	167	19.38
3787	53	DRB1*11:42	RVLIVRRNLLDLSKE	TRP1	P17643	125	139	23.19
3788	54	DRB1*11:42	SYLIRARRSMDEANQ	TRP1	P17643	498	512	41.67
3789	55	DRB1*11:42	TASYLIRARRSMDEA	TRP1	P17643	496	510	25.57
3790	56	DRB1*11:42	THPLFVIATRRSEEI	TRP1	P17643	155	169	14.39
3791	57	DRB1*11:42	TTHPLFVIATRRSEE	TRP1	P17643	154	168	17.13
3792	58	DRB1*11:42	VRALDMAKRTTHPLF	TRP1	P17643	145	159	38.2
3793	59	DRB1*11:42	YDPAVRSLHNLAHLF	TRP1	P17643	369	383	46.98
3794	60	DRB1*11:42	ACQLVLHQILKGGSG	PMEL17	P40967	549	563	30.74
3795	61	DRB1*11:42	ASLIYRRRLMKQDFS	PMEL17	P40967	612	626	44.48
3796	62	DRB1*11:42	ATLRLVKRQVPLDCV	PMEL17	P40967	462	476	15.44
3797	63	DRB1*11:42	AVVLASLIYRRRLMK	PMEL17	P40967	608	622	18.72
3798	64	DRB1*11:42	CQLVLHQILKGGSGT	PMEL17	P40967	550	564	28.37
3799	65	DRB1*11:42	DGTATLRLVKRQVPL	PMEL17	P40967	459	473	10.82
3800	66	DRB1*11:42	DWLGVSRQLRTKAWN	PMEL17	P40967	31	45	39.03
3801	67	DRB1*11:42	GTATLRLVKRQVPLD	PMEL17	P40967	460	474	10.87
3802	68	DRB1*11:42	KRSFVYVWKTWGQYW	PMEL17	P40967	146	160	45.58
3803	69	DRB1*11:42	LASLIYRRRLMKQDF	PMEL17	P40967	611	625	28.95
3804	70	DRB1*11:42	LDGTATLRLVKRQVP	PMEL17	P40967	458	472	19.07
3805	71	DRB1*11:42	LGVSRQLRTKAWNRQ	PMEL17	P40967	33	47	44.51
3806	72	DRB1*11:42	LMAVVLASLIYRRRL	PMEL17	P40967	606	620	27.63
3807	73	DRB1*11:42	LVLHQILKGGSGTYC	PMEL17	P40967	552	566	42.96
3808	74	DRB1*11:42	MAVVLASLIYRRRLM	PMEL17	P40967	607	621	24.17
3809	75	DRB1*11:42	NKHFLRNQPLTFALQ	PMEL17	P40967	229	243	48.81
3810	76	DRB1*11:42	NQDWLGVSRQLRTKA	PMEL17	P40967	29	43	42.49

3811	77	DRB1*11:42	PACQLVLHQILKGGS	PMEL17	P40967	548	562	43.91
3812	78	DRB1*11:42	QDWLGVSRQLRTKAW	PMEL17	P40967	30	44	34.81
3813	79	DRB1*11:42	QKRSFVYVWKTWGQY	PMEL17	P40967	145	159	48.04
3814	80	DRB1*11:42	QLVLHQILKGGSGTY	PMEL17	P40967	551	565	25.08
3815	81	DRB1*11:42	TATLRLVKRQVPLDC	PMEL17	P40967	461	475	12.16
3816	82	DRB1*11:42	TLRLVKRQVPLDCVL	PMEL17	P40967	463	477	26.46
3817	83	DRB1*11:42	VLASLIYRRRLMKQD	PMEL17	P40967	610	624	22.78
3818	84	DRB1*11:42	VLMAVVLASLIYRRR	PMEL17	P40967	605	619	34.16
3819	85	DRB1*11:42	VTAQVVLQAAIPLTS	PMEL17	P40967	286	300	47.48
3820	86	DRB1*11:42	VVLASLIYRRRLMKQ	PMEL17	P40967	609	623	18.47
3821	87	DRB1*11:42	WLGVSRQLRTKAWNR	PMEL17	P40967	32	46	37.33
3822	88	DRB1*11:42	ADLVGFLLLKYRARE	MAGE1	P43355	107	121	24.87
3823	89	DRB1*11:42	AEMLESVIKNYKHCF	MAGE1	P43355	126	140	45.61
3824	90	DRB1*11:42	ARVRFFFPSLREAAL	MAGE1	P43355	288	302	41.02
3825	91	DRB1*11:42	CILESLFRAVITKKV	MAGE1	P43355	92	106	39.57
3826	92	DRB1*11:42	DLVGFLLLKYRAREP	MAGE1	P43355	108	122	20.99
3827	93	DRB1*11:42	ESLFRAVITKKVADL	MAGE1	P43355	95	109	35.53
3828	94	DRB1*11:42	EYVIKVSARVRFFFP	MAGE1	P43355	281	295	10.6
3829	95	DRB1*11:42	FLLLKYRAREPVTKA	MAGE1	P43355	112	126	38.03
3830	96	DRB1*11:42	GFLLLKYRAREPVTK	MAGE1	P43355	111	125	27.65
3831	97	DRB1*11:42	ILESLFRAVITKKVA	MAGE1	P43355	93	107	31.61
3832	98	DRB1*11:42	KVLEYVIKVSARVRF	MAGE1	P43355	278	292	10.88
3833	99	DRB1*11:42	LESLFRAVITKKVAD	MAGE1	P43355	94	108	38.13
3834	100	DRB1*11:42	LEYVIKVSARVRFFF	MAGE1	P43355	280	294	10.49
3835	101	DRB1*11:42	LFRAVITKKVADLVG	MAGE1	P43355	97	111	43.51
3836	102	DRB1*11:42	LVGFLLLKYRAREPV	MAGE1	P43355	109	123	20.39
3837	103	DRB1*11:42	RVRFFFPSLREAALR	MAGE1	P43355	289	303	39.93
3838	104	DRB1*11:42	SARVRFFFPSLREAA	MAGE1	P43355	287	301	42.54
3839	105	DRB1*11:42	SCILESLFRAVITKK	MAGE1	P43355	91	105	41.73
3840	106	DRB1*11:42	SLFRAVITKKVADLV	MAGE1	P43355	96	110	35.07
3841	107	DRB1*11:42	SYVKVLEYVIKVSAR	MAGE1	P43355	275	289	39.26
3842	108	DRB1*11:42	VADLVGFLLLKYRAR	MAGE1	P43355	106	120	33.92
3843	109	DRB1*11:42	VGFLLLKYRAREPVT	MAGE1	P43355	110	124	21.61
3844	110	DRB1*11:42	VIKVSARVRFFFPSL	MAGE1	P43355	283	297	27.3
3845	111	DRB1*11:42	VKVLEYVIKVSARVR	MAGE1	P43355	277	291	15.96
3846	112	DRB1*11:42	VLEYVIKVSARVRFF	MAGE1	P43355	279	293	10.57
3847	113	DRB1*11:42	VRFFFPSLREAALRE	MAGE1	P43355	290	304	48.96
3848	114	DRB1*11:42	YVIKVSARVRFFFPS	MAGE1	P43355	282	296	14.54
3849	115	DRB1*11:42	YVKVLEYVIKVSARV	MAGE1	P43355	276	290	36.14
3850	116	DRB1*11:42	AEMLERVIKNYKRCF	MAGE4	P43358	134	148	35.25

3851	117	DRB1*11:42	AHFLLRKYRAKELVT	MAGE4	P43358	118	132	35.07
3852	118	DRB1*11:42	ARVRIAYPSLREAAL	MAGE4	P43358	296	310	35.02
3853	119	DRB1*11:42	DELAHFLLRKYRAKE	MAGE4	P43358	115	129	29.07
3854	120	DRB1*11:42	EHVVRVNARVRIAYP	MAGE4	P43358	289	303	15.17
3855	121	DRB1*11:42	ELAHFLLRKYRAKEL	MAGE4	P43358	116	130	25.66
3856	122	DRB1*11:42	EMLERVIKNYKRCFP	MAGE4	P43358	135	149	40.72
3857	123	DRB1*11:42	ERVIKNYKRCFPVIF	MAGE4	P43358	138	152	46.84
3858	124	DRB1*11:42	ETSYVKVLEHVVRVN	MAGE4	P43358	281	295	47.49
3859	125	DRB1*11:42	HFLLRKYRAKELVTK	MAGE4	P43358	119	133	47.03
3860	126	DRB1*11:42	HVVRVNARVRIAYPS	MAGE4	P43358	290	304	18.19
3861	127	DRB1*11:42	KAEMLERVIKNYKRC	MAGE4	P43358	133	147	44.35
3862	128	DRB1*11:42	KVDELAHFLLRKYRA	MAGE4	P43358	113	127	43.21
3863	129	DRB1*11:42	KVLEHVVRVNARVRI	MAGE4	P43358	286	300	13.44
3864	130	DRB1*11:42	LAHFLLRKYRAKELV	MAGE4	P43358	117	131	27.04
3865	131	DRB1*11:42	LEHVVRVNARVRIAY	MAGE4	P43358	288	302	12.35
3866	132	DRB1*11:42	LERVIKNYKRCFPVI	MAGE4	P43358	137	151	36.49
3867	133	DRB1*11:42	MLERVIKNYKRCFPV	MAGE4	P43358	136	150	37.63
3868	134	DRB1*11:42	NARVRIAYPSLREAA	MAGE4	P43358	295	309	29.59
3869	135	DRB1*11:42	RVNARVRIAYPSLRE	MAGE4	P43358	293	307	36.6
3870	136	DRB1*11:42	SYVKVLEHVVRVNAR	MAGE4	P43358	283	297	23.89
3871	137	DRB1*11:42	TKAEMLERVIKNYKR	MAGE4	P43358	132	146	36.32
3872	138	DRB1*11:42	TSYVKVLEHVVRVNA	MAGE4	P43358	282	296	32.24
3873	139	DRB1*11:42	VDELAHFLLRKYRAK	MAGE4	P43358	114	128	29.7
3874	140	DRB1*11:42	VKVLEHVVRVNARVR	MAGE4	P43358	285	299	16.03
3875	141	DRB1*11:42	VLEHVVRVNARVRIA	MAGE4	P43358	287	301	14.07
3876	142	DRB1*11:42	VNARVRIAYPSLREA	MAGE4	P43358	294	308	33.84
3877	143	DRB1*11:42	VRVNARVRIAYPSLR	MAGE4	P43358	292	306	36
3878	144	DRB1*11:42	VTKAEMLERVIKNYK	MAGE4	P43358	131	145	48.8
3879	145	DRB1*11:42	VVRVNARVRIAYPSL	MAGE4	P43358	291	305	25.48
3880	146	DRB1*11:42	YVKVLEHVVRVNARV	MAGE4	P43358	284	298	22.47
3881	147	DRB1*11:42	GNILTIRLTAADHRQ	NY-ESO-1	P78358	130	144	32.64
3882	148	DRB1*11:42	ILTIRLTAADHRQLQ	NY-ESO-1	P78358	132	146	36.9
3883	149	DRB1*11:42	LTIRLTAADHRQLQL	NY-ESO-1	P78358	133	147	44.84
3884	150	DRB1*11:42	NILTIRLTAADHRQL	NY-ESO-1	P78358	131	145	36.81
3885	151	DRB1*11:42	SGNILTIRLTAADHR	NY-ESO-1	P78358	129	143	33.74
3886	152	DRB1*11:42	VSGNILTIRLTAADH	NY-ESO-1	P78358	128	142	48.71
3887	153	DRB1*11:42	RAVFLALSAQLLQAR	BAGE	Q13072	4	18	47.37
3888	154	DRB1*11:42	ASEKIFYVYMKRKYE	SSX2	Q16385	42	56	30.02
3889	155	DRB1*11:42	EKIFYVYMKRKYEAM	SSX2	Q16385	44	58	18.11
3890	156	DRB1*11:42	EKMKASEKIFYVYMK	SSX2	Q16385	38	52	42.26

3891	157	DRB1*11:42	EWEKMKASEKIFYVY	SSX2	Q16385	36	50	39.75
3892	158	DRB1*11:42	FYVYMKRKYEAMTKL	SSX2	Q16385	47	61	28.04
3893	159	DRB1*11:42	IFYVYMKRKYEAMTK	SSX2	Q16385	46	60	19.51
3894	160	DRB1*11:42	KASEKIFYVYMKRKY	SSX2	Q16385	41	55	48.84
3895	161	DRB1*11:42	KIFYVYMKRKYEAMT	SSX2	Q16385	45	59	18.28
3896	162	DRB1*11:42	SEKIFYVYMKRKYEA	SSX2	Q16385	43	57	20.63
3897	163	DRB1*11:42	WEKMKASEKIFYVYM	SSX2	Q16385	37	51	34.7
3898	1	DRB1*11:46	EVISCKLIKRATTRQ	TRP2	O75767	222	236	19.29
3899	2	DRB1*11:46	FLGALDLAKKRVHPD	TRP2	O75767	141	155	39.69
3900	3	DRB1*11:46	LGALDLAKKRVHPDY	TRP2	O75767	142	156	30.57
3901	4	DRB1*11:46	QFLGALDLAKKRVHP	TRP2	O75767	140	154	35.1
3902	5	DRB1*11:46	QWEVISCKLIKRATT	TRP2	O75767	220	234	36.31
3903	6	DRB1*11:46	VISCKLIKRATTRQP	TRP2	O75767	223	237	20.64
3904	7	DRB1*11:46	WEVISCKLIKRATTR	TRP2	O75767	221	235	22.06
3905	8	DRB1*11:46	AGLVSLLCRHKRKQL	Tyrosinase	P14679	493	507	20.85
3906	9	DRB1*11:46	ALLAGLVSLLCRHKR	Tyrosinase	P14679	490	504	44.71
3907	10	DRB1*11:46	DKFFAYLTLAKHTIS	Tyrosinase	P14679	132	146	46.72
3908	11	DRB1*11:46	FAYLTLAKHTISSDY	Tyrosinase	P14679	135	149	27.46
3909	12	DRB1*11:46	FFAYLTLAKHTISSD	Tyrosinase	P14679	134	148	28.42
3910	13	DRB1*11:46	GLVSLLCRHKRKQLP	Tyrosinase	P14679	494	508	26.41
3911	14	DRB1*11:46	IKSYLEQASRIWSWL	Tyrosinase	P14679	464	478	46.23
3912	15	DRB1*11:46	KFFAYLTLAKHTISS	Tyrosinase	P14679	133	147	27.61
3913	16	DRB1*11:46	KSYLEQASRIWSWLL	Tyrosinase	P14679	465	479	48.45
3914	17	DRB1*11:46	LAGLVSLLCRHKRKQ	Tyrosinase	P14679	492	506	24.86
3915	18	DRB1*11:46	LLAGLVSLLCRHKRK	Tyrosinase	P14679	491	505	27.66
3916	19	DRB1*11:46	LVSLLCRHKRKQLPE	Tyrosinase	P14679	495	509	40.74
3917	20	DRB1*11:46	AACDQRVLIVRRNLL	TRP1	P17643	120	134	29.49
3918	21	DRB1*11:46	ACDQRVLIVRRNLLD	TRP1	P17643	121	135	24.43
3919	22	DRB1*11:46	ASYLIRARRSMDEAN	TRP1	P17643	497	511	28.67
3920	23	DRB1*11:46	CDQRVLIVRRNLLDL	TRP1	P17643	122	136	20.29
3921	24	DRB1*11:46	DQRVLIVRRNLLDLS	TRP1	P17643	123	137	19.07
3922	25	DRB1*11:46	FFPLLLFQQARAQFP	TRP1	P17643	13	27	32.64
3923	26	DRB1*11:46	FGTASYLIRARRSMD	TRP1	P17643	494	508	30.25
3924	27	DRB1*11:46	FPLLLFQQARAQFPR	TRP1	P17643	14	28	26.77
3925	28	DRB1*11:46	FVRALDMAKRTTHPL	TRP1	P17643	144	158	38.95
3926	29	DRB1*11:46	GTASYLIRARRSMDE	TRP1	P17643	495	509	29.26
3927	30	DRB1*11:46	HPLFVIATRRSEEIL	TRP1	P17643	156	170	12.52
3928	31	DRB1*11:46	IFFPLLLFQQARAQF	TRP1	P17643	12	26	47.75
3929	32	DRB1*11:46	IFGTASYLIRARRSM	TRP1	P17643	493	507	30.93

3930	33	DRB1*11:46	KRTTHPLFVIATRRS	TRP1	P17643	152	166	42.57
3931	34	DRB1*11:46	LFVIATRRSEEILGP	TRP1	P17643	158	172	37.31
3932	35	DRB1*11:46	LIFGTASYLIRARRS	TRP1	P17643	492	506	43.28
3933	36	DRB1*11:46	PLFVIATRRSEEILG	TRP1	P17643	157	171	16.46
3934	37	DRB1*11:46	PLLLFQQARAQFPRQ	TRP1	P17643	15	29	38.91
3935	38	DRB1*11:46	QRVLIVRRNLLDLSK	TRP1	P17643	124	138	17.56
3936	39	DRB1*11:46	RTTHPLFVIATRRSE	TRP1	P17643	153	167	17.16
3937	40	DRB1*11:46	RVLIVRRNLLDLSKE	TRP1	P17643	125	139	27.27
3938	41	DRB1*11:46	SYLIRARRSMDEANQ	TRP1	P17643	498	512	39.58
3939	42	DRB1*11:46	TASYLIRARRSMDEA	TRP1	P17643	496	510	23.81
3940	43	DRB1*11:46	THPLFVIATRRSEEI	TRP1	P17643	155	169	12.59
3941	44	DRB1*11:46	TTHPLFVIATRRSEE	TRP1	P17643	154	168	15.45
3942	45	DRB1*11:46	VRALDMAKRTTHPLF	TRP1	P17643	145	159	31.74
3943	46	DRB1*11:46	ACQLVLHQILKGGSG	PMEL17	P40967	549	563	20.08
3944	47	DRB1*11:46	ASLIYRRRLMKQDFS	PMEL17	P40967	612	626	36.01
3945	48	DRB1*11:46	ATLRLVKRQVPLDCV	PMEL17	P40967	462	476	15.25
3946	49	DRB1*11:46	AVVLASLIYRRRLMK	PMEL17	P40967	608	622	20.89
3947	50	DRB1*11:46	CQLVLHQILKGGSGT	PMEL17	P40967	550	564	17.75
3948	51	DRB1*11:46	DGTATLRLVKRQVPL	PMEL17	P40967	459	473	9.31
3949	52	DRB1*11:46	DWLGVSRQLRTKAWN	PMEL17	P40967	31	45	44.48
3950	53	DRB1*11:46	GTATLRLVKRQVPLD	PMEL17	P40967	460	474	10.14
3951	54	DRB1*11:46	KRSFVYVWKTWGQYW	PMEL17	P40967	146	160	37.07
3952	55	DRB1*11:46	LASLIYRRRLMKQDF	PMEL17	P40967	611	625	26.62
3953	56	DRB1*11:46	LDGTATLRLVKRQVP	PMEL17	P40967	458	472	22.43
3954	57	DRB1*11:46	LGVSRQLRTKAWNRQ	PMEL17	P40967	33	47	48.19
3955	58	DRB1*11:46	LMAVVLASLIYRRRL	PMEL17	P40967	606	620	37.88
3956	59	DRB1*11:46	LVLHQILKGGSGTYC	PMEL17	P40967	552	566	20.38
3957	60	DRB1*11:46	MAVVLASLIYRRRLM	PMEL17	P40967	607	621	30.33
3958	61	DRB1*11:46	PACQLVLHQILKGGS	PMEL17	P40967	548	562	36.62
3959	62	DRB1*11:46	QDWLGVSRQLRTKAW	PMEL17	P40967	30	44	43.91
3960	63	DRB1*11:46	QKRSFVYVWKTWGQY	PMEL17	P40967	145	159	37.12
3961	64	DRB1*11:46	QLVLHQILKGGSGTY	PMEL17	P40967	551	565	15.12
3962	65	DRB1*11:46	SQKRSFVYVWKTWGQ	PMEL17	P40967	144	158	46.19
3963	66	DRB1*11:46	TATLRLVKRQVPLDC	PMEL17	P40967	461	475	11.26
3964	67	DRB1*11:46	TLRLVKRQVPLDCVL	PMEL17	P40967	463	477	31.83
3965	68	DRB1*11:46	VLASLIYRRRLMKQD	PMEL17	P40967	610	624	22.87
3966	69	DRB1*11:46	VLHQILKGGSGTYCL	PMEL17	P40967	553	567	49.95
3967	70	DRB1*11:46	VVLASLIYRRRLMKQ	PMEL17	P40967	609	623	19.21
3968	71	DRB1*11:46	WLGVSRQLRTKAWNR	PMEL17	P40967	32	46	35.36
3969	72	DRB1*11:46	ADLVGFLLLKYRARE	MAGE1	P43355	107	121	22.28

3970	73	DRB1*11:46	ARVRFFFPSLREAAL	MAGE1	P43355	288	302	42.61
3971	74	DRB1*11:46	CILESLFRAVITKKV	MAGE1	P43355	92	106	49.41
3972	75	DRB1*11:46	DLVGFLLLKYRAREP	MAGE1	P43355	108	122	17.99
3973	76	DRB1*11:46	ESLFRAVITKKVADL	MAGE1	P43355	95	109	44.48
3974	77	DRB1*11:46	EYVIKVSARVRFFFP	MAGE1	P43355	281	295	17.68
3975	78	DRB1*11:46	FLLLKYRAREPVTKA	MAGE1	P43355	112	126	33.91
3976	79	DRB1*11:46	GFLLLKYRAREPVTK	MAGE1	P43355	111	125	25.28
3977	80	DRB1*11:46	ILESLFRAVITKKVA	MAGE1	P43355	93	107	36.18
3978	81	DRB1*11:46	KVLEYVIKVSARVRF	MAGE1	P43355	278	292	14.12
3979	82	DRB1*11:46	LESLFRAVITKKVAD	MAGE1	P43355	94	108	49.59
3980	83	DRB1*11:46	LEYVIKVSARVRFFF	MAGE1	P43355	280	294	17.21
3981	84	DRB1*11:46	LVGFLLLKYRAREPV	MAGE1	P43355	109	123	17.19
3982	85	DRB1*11:46	RVRFFFPSLREAALR	MAGE1	P43355	289	303	32.94
3983	86	DRB1*11:46	SLFRAVITKKVADLV	MAGE1	P43355	96	110	44.25
3984	87	DRB1*11:46	VADLVGFLLLKYRAR	MAGE1	P43355	106	120	36.58
3985	88	DRB1*11:46	VGFLLLKYRAREPVT	MAGE1	P43355	110	124	18.8
3986	89	DRB1*11:46	VIKVSARVRFFFPSL	MAGE1	P43355	283	297	46.43
3987	90	DRB1*11:46	VKVLEYVIKVSARVR	MAGE1	P43355	277	291	21.36
3988	91	DRB1*11:46	VLEYVIKVSARVRFF	MAGE1	P43355	279	293	16.18
3989	92	DRB1*11:46	VRFFFPSLREAALRE	MAGE1	P43355	290	304	38.38
3990	93	DRB1*11:46	YVIKVSARVRFFFPS	MAGE1	P43355	282	296	23.47
3991	94	DRB1*11:46	AEMLERVIKNYKRCF	MAGE4	P43358	134	148	47.43
3992	95	DRB1*11:46	AHFLLRKYRAKELVT	MAGE4	P43358	118	132	26.1
3993	96	DRB1*11:46	DELAHFLLRKYRAKE	MAGE4	P43358	115	129	25.68
3994	97	DRB1*11:46	EHVVRVNARVRIAYP	MAGE4	P43358	289	303	35.82
3995	98	DRB1*11:46	ELAHFLLRKYRAKEL	MAGE4	P43358	116	130	19.95
3996	99	DRB1*11:46	HFLLRKYRAKELVTK	MAGE4	P43358	119	133	38.75
3997	100	DRB1*11:46	HVVRVNARVRIAYPS	MAGE4	P43358	290	304	39.92
3998	101	DRB1*11:46	KVDELAHFLLRKYRA	MAGE4	P43358	113	127	45.38
3999	102	DRB1*11:46	KVLEHVVRVNARVRI	MAGE4	P43358	286	300	14.3
4000	103	DRB1*11:46	LAHFLLRKYRAKELV	MAGE4	P43358	117	131	21.15
4001	104	DRB1*11:46	LEHVVRVNARVRIAY	MAGE4	P43358	288	302	26.61
4002	105	DRB1*11:46	SYVKVLEHVVRVNAR	MAGE4	P43358	283	297	21.83
4003	106	DRB1*11:46	TKAEMLERVIKNYKR	MAGE4	P43358	132	146	48.98
4004	107	DRB1*11:46	TSYVKVLEHVVRVNA	MAGE4	P43358	282	296	45.07
4005	108	DRB1*11:46	VDELAHFLLRKYRAK	MAGE4	P43358	114	128	28.08
4006	109	DRB1*11:46	VKVLEHVVRVNARVR	MAGE4	P43358	285	299	13.92
4007	110	DRB1*11:46	VLEHVVRVNARVRIA	MAGE4	P43358	287	301	20.88
4008	111	DRB1*11:46	YVKVLEHVVRVNARV	MAGE4	P43358	284	298	19.77
4009	112	DRB1*11:46	SGNILTIRLTAADHR	NY-ESO-1	P78358	129	143	45.87

4010	113	DRB1*11:46	ASEKIFYVYMKRKYE	SSX2	Q16385	42	56	28.14
4011	114	DRB1*11:46	EKIFYVYMKRKYEAM	SSX2	Q16385	44	58	12.34
4012	115	DRB1*11:46	FYVYMKRKYEAMTKL	SSX2	Q16385	47	61	16.24
4013	116	DRB1*11:46	IFYVYMKRKYEAMTK	SSX2	Q16385	46	60	11.71
4014	117	DRB1*11:46	KIFYVYMKRKYEAMT	SSX2	Q16385	45	59	11.33
4015	118	DRB1*11:46	SEKIFYVYMKRKYEA	SSX2	Q16385	43	57	16.45
4016	119	DRB1*11:46	YVYMKRKYEAMTKLG	SSX2	Q16385	48	62	35.29
4017	1	DRB1*11:49	EVISCKLIKRATTRQ	TRP2	O75767	222	236	30.92
4018	2	DRB1*11:49	RKFFHRTCKCTGNFA	TRP2	O75767	88	102	39.55
4019	3	DRB1*11:49	VISCKLIKRATTRQP	TRP2	O75767	223	237	33.86
4020	4	DRB1*11:49	WEVISCKLIKRATTR	TRP2	O75767	221	235	36.89
4021	5	DRB1*11:49	YYYRFVIGLRVWQWE	TRP2	O75767	208	222	49.95
4022	6	DRB1*11:49	DKFFAYLTLAKHTIS	Tyrosinase	P14679	132	146	26.01
4023	7	DRB1*11:49	FAYLTLAKHTISSDY	Tyrosinase	P14679	135	149	35.24
4024	8	DRB1*11:49	FFAYLTLAKHTISSD	Tyrosinase	P14679	134	148	28.57
4025	9	DRB1*11:49	KDKFFAYLTLAKHTI	Tyrosinase	P14679	131	145	33.33
4026	10	DRB1*11:49	KFFAYLTLAKHTISS	Tyrosinase	P14679	133	147	22.47
4027	11	DRB1*11:49	MVPFIPLYRNGDFFI	Tyrosinase	P14679	426	440	32.73
4028	12	DRB1*11:49	VPFIPLYRNGDFFIS	Tyrosinase	P14679	427	441	41.52
4029	13	DRB1*11:49	YMVPFIPLYRNGDFF	Tyrosinase	P14679	425	439	42.2
4030	14	DRB1*11:49	ASYLIRARRSMDEAN	TRP1	P17643	497	511	46.19
4031	15	DRB1*11:49	FVWTHYYSVKKTFLG	TRP1	P17643	188	202	47.59
4032	16	DRB1*11:49	HPLFVIATRRSEEIL	TRP1	P17643	156	170	13.74
4033	17	DRB1*11:49	LFVIATRRSEEILGP	TRP1	P17643	158	172	24.07
4034	18	DRB1*11:49	PLFVIATRRSEEILG	TRP1	P17643	157	171	15.42
4035	19	DRB1*11:49	RTTHPLFVIATRRSE	TRP1	P17643	153	167	24.44
4036	20	DRB1*11:49	TASYLIRARRSMDEA	TRP1	P17643	496	510	39.65
4037	21	DRB1*11:49	THPLFVIATRRSEEI	TRP1	P17643	155	169	15.19
4038	22	DRB1*11:49	THYYSVKKTFLGVGQ	TRP1	P17643	191	205	37.63
4039	23	DRB1*11:49	TTHPLFVIATRRSEE	TRP1	P17643	154	168	19.39
4040	24	DRB1*11:49	VWTHYYSVKKTFLGV	TRP1	P17643	189	203	31.01
4041	25	DRB1*11:49	WTHYYSVKKTFLGVG	TRP1	P17643	190	204	32.76
4042	26	DRB1*11:49	ANASFSIALNFPGSQ	PMEL17	P40967	80	94	49.44
4043	27	DRB1*11:49	ASFSIALNFPGSQKV	PMEL17	P40967	82	96	42.13
4044	28	DRB1*11:49	ATLRLVKRQVPLDCV	PMEL17	P40967	462	476	32.58
4045	29	DRB1*11:49	CQLVLHQILKGGSGT	PMEL17	P40967	550	564	45.41
4046	30	DRB1*11:49	DGTATLRLVKRQVPL	PMEL17	P40967	459	473	18.27
4047	31	DRB1*11:49	GSRSYVPLAHSSSAF	PMEL17	P40967	193	207	39.7
4048	32	DRB1*11:49	GTATLRLVKRQVPLD	PMEL17	P40967	460	474	20.16

4049	33	DRB1*11:49	KRSFVYVWKTWGQYW	PMEL17	P40967	146	160	17.94
4050	34	DRB1*11:49	NASFSIALNFPGSQK	PMEL17	P40967	81	95	33.06
4051	35	DRB1*11:49	QKRSFVYVWKTWGQY	PMEL17	P40967	145	159	18.3
4052	36	DRB1*11:49	QLVLHQILKGGSGTY	PMEL17	P40967	551	565	36.77
4053	37	DRB1*11:49	RSFVYVWKTWGQYWQ	PMEL17	P40967	147	161	30.25
4054	38	DRB1*11:49	RSYVPLAHSSSAFTI	PMEL17	P40967	195	209	42.32
4055	39	DRB1*11:49	SQKRSFVYVWKTWGQ	PMEL17	P40967	144	158	21.74
4056	40	DRB1*11:49	SRSYVPLAHSSSAFT	PMEL17	P40967	194	208	39.52
4057	41	DRB1*11:49	TATLRLVKRQVPLDC	PMEL17	P40967	461	475	23.06
4058	42	DRB1*11:49	WSQKRSFVYVWKTWG	PMEL17	P40967	143	157	41.25
4059	43	DRB1*11:49	ADLVGFLLLKYRARE	MAGE1	P43355	107	121	34.07
4060	44	DRB1*11:49	ARVRFFFPSLREAAL	MAGE1	P43355	288	302	21.89
4061	45	DRB1*11:49	ARYEFLWGPRALAET	MAGE1	P43355	260	274	33.84
4062	46	DRB1*11:49	DLVGFLLLKYRAREP	MAGE1	P43355	108	122	28.93
4063	47	DRB1*11:49	ESLFRAVITKKVADL	MAGE1	P43355	95	109	29.13
4064	48	DRB1*11:49	EYVIKVSARVRFFFP	MAGE1	P43355	281	295	39.57
4065	49	DRB1*11:49	FFFPSLREAALREEE	MAGE1	P43355	292	306	45.29
4066	50	DRB1*11:49	GFLLLKYRAREPVTK	MAGE1	P43355	111	125	45.81
4067	51	DRB1*11:49	ILESLFRAVITKKVA	MAGE1	P43355	93	107	32.17
4068	52	DRB1*11:49	KVLEYVIKVSARVRF	MAGE1	P43355	278	292	25.6
4069	53	DRB1*11:49	LESLFRAVITKKVAD	MAGE1	P43355	94	108	37.28
4070	54	DRB1*11:49	LEYVIKVSARVRFFF	MAGE1	P43355	280	294	34.02
4071	55	DRB1*11:49	LVGFLLLKYRAREPV	MAGE1	P43355	109	123	26.38
4072	56	DRB1*11:49	PARYEFLWGPRALAE	MAGE1	P43355	259	273	37.79
4073	57	DRB1*11:49	RFFFPSLREAALREE	MAGE1	P43355	291	305	18.8
4074	58	DRB1*11:49	RVRFFFPSLREAALR	MAGE1	P43355	289	303	14
4075	59	DRB1*11:49	RYEFLWGPRALAETS	MAGE1	P43355	261	275	34.81
4076	60	DRB1*11:49	SARVRFFFPSLREAA	MAGE1	P43355	287	301	49.09
4077	61	DRB1*11:49	SLFRAVITKKVADLV	MAGE1	P43355	96	110	32.18
4078	62	DRB1*11:49	VGFLLLKYRAREPVT	MAGE1	P43355	110	124	31.71
4079	63	DRB1*11:49	VKVLEYVIKVSARVR	MAGE1	P43355	277	291	29.38
4080	64	DRB1*11:49	VLEYVIKVSARVRFF	MAGE1	P43355	279	293	31.77
4081	65	DRB1*11:49	VRFFFPSLREAALRE	MAGE1	P43355	290	304	14.6
4082	66	DRB1*11:49	AHFLLRKYRAKELVT	MAGE4	P43358	118	132	42.25
4083	67	DRB1*11:49	ARYEFLWGPRALAET	MAGE4	P43358	268	282	33.84
4084	68	DRB1*11:49	DELAHFLLRKYRAKE	MAGE4	P43358	115	129	39.02
4085	69	DRB1*11:49	ELAHFLLRKYRAKEL	MAGE4	P43358	116	130	28.81
4086	70	DRB1*11:49	KVLEHVVRVNARVRI	MAGE4	P43358	286	300	31.5
4087	71	DRB1*11:49	LAHFLLRKYRAKELV	MAGE4	P43358	117	131	29.7
4088	72	DRB1*11:49	PARYEFLWGPRALAE	MAGE4	P43358	267	281	37.79

4089	73	DRB1*11:49	RYEFLWGPRALAETS	MAGE4	P43358	269	283	34.81
4090	74	DRB1*11:49	SYVKVLEHVVRVNAR	MAGE4	P43358	283	297	47.81
4091	75	DRB1*11:49	VDELAHFLLRKYRAK	MAGE4	P43358	114	128	43.08
4092	76	DRB1*11:49	VKVLEHVVRVNARVR	MAGE4	P43358	285	299	30.17
4093	77	DRB1*11:49	YVKVLEHVVRVNARV	MAGE4	P43358	284	298	40.41
4094	78	DRB1*11:49	RAVFLALSAQLLQAR	BAGE	Q13072	4	18	49.7
4095	79	DRB1*11:49	ASEKIFYVYMKRKYE	SSX2	Q16385	42	56	21.98
4096	80	DRB1*11:49	EKIFYVYMKRKYEAM	SSX2	Q16385	44	58	12.58
4097	81	DRB1*11:49	FYVYMKRKYEAMTKL	SSX2	Q16385	47	61	19.78
4098	82	DRB1*11:49	IFYVYMKRKYEAMTK	SSX2	Q16385	46	60	13.87
4099	83	DRB1*11:49	KIFYVYMKRKYEAMT	SSX2	Q16385	45	59	12.77
4100	84	DRB1*11:49	KRKYEAMTKLGFKAT	SSX2	Q16385	52	66	47.42
4101	85	DRB1*11:49	MKRKYEAMTKLGFKA	SSX2	Q16385	51	65	47.51
4102	86	DRB1*11:49	SEKIFYVYMKRKYEA	SSX2	Q16385	43	57	15.39
4103	1	DRB1*11:54	DGTATLRLVKRQVPL	PMEL17	P40967	459	473	47.87
4104	2	DRB1*11:54	EKIFYVYMKRKYEAM	SSX2	Q16385	44	58	49.95
4105	1	DRB1*11:58	EVISCKLIKRATTRQ	TRP2	O75767	222	236	19.29
4106	2	DRB1*11:58	FLGALDLAKKRVHPD	TRP2	O75767	141	155	39.69
4107	3	DRB1*11:58	LGALDLAKKRVHPDY	TRP2	O75767	142	156	30.57
4108	4	DRB1*11:58	QFLGALDLAKKRVHP	TRP2	O75767	140	154	35.1
4109	5	DRB1*11:58	QWEVISCKLIKRATT	TRP2	O75767	220	234	36.31
4110	6	DRB1*11:58	VISCKLIKRATTRQP	TRP2	O75767	223	237	20.64
4111	7	DRB1*11:58	WEVISCKLIKRATTR	TRP2	O75767	221	235	22.06
4112	8	DRB1*11:58	AGLVSLLCRHKRKQL	Tyrosinase	P14679	493	507	20.85
4113	9	DRB1*11:58	ALLAGLVSLLCRHKR	Tyrosinase	P14679	490	504	44.71
4114	10	DRB1*11:58	DKFFAYLTLAKHTIS	Tyrosinase	P14679	132	146	46.72
4115	11	DRB1*11:58	FAYLTLAKHTISSDY	Tyrosinase	P14679	135	149	27.46
4116	12	DRB1*11:58	FFAYLTLAKHTISSD	Tyrosinase	P14679	134	148	28.42
4117	13	DRB1*11:58	GLVSLLCRHKRKQLP	Tyrosinase	P14679	494	508	26.41
4118	14	DRB1*11:58	IKSYLEQASRIWSWL	Tyrosinase	P14679	464	478	46.23
4119	15	DRB1*11:58	KFFAYLTLAKHTISS	Tyrosinase	P14679	133	147	27.61
4120	16	DRB1*11:58	KSYLEQASRIWSWLL	Tyrosinase	P14679	465	479	48.45
4121	17	DRB1*11:58	LAGLVSLLCRHKRKQ	Tyrosinase	P14679	492	506	24.86
4122	18	DRB1*11:58	LLAGLVSLLCRHKRK	Tyrosinase	P14679	491	505	27.66
4123	19	DRB1*11:58	LVSLLCRHKRKQLPE	Tyrosinase	P14679	495	509	40.74
4124	20	DRB1*11:58	AACDQRVLIVRRNLL	TRP1	P17643	120	134	29.49
4125	21	DRB1*11:58	ACDQRVLIVRRNLLD	TRP1	P17643	121	135	24.43
4126	22	DRB1*11:58	ASYLIRARRSMDEAN	TRP1	P17643	497	511	28.67

4127	23	DRB1*11:58	CDQRVLIVRRNLLDL	TRP1	P17643	122	136	20.29
4128	24	DRB1*11:58	DQRVLIVRRNLLDLS	TRP1	P17643	123	137	19.07
4129	25	DRB1*11:58	FFPLLLFQQARAQFP	TRP1	P17643	13	27	32.64
4130	26	DRB1*11:58	FGTASYLIRARRSMD	TRP1	P17643	494	508	30.25
4131	27	DRB1*11:58	FPLLLFQQARAQFPR	TRP1	P17643	14	28	26.77
4132	28	DRB1*11:58	FVRALDMAKRTTHPL	TRP1	P17643	144	158	38.95
4133	29	DRB1*11:58	GTASYLIRARRSMDE	TRP1	P17643	495	509	29.26
4134	30	DRB1*11:58	HPLFVIATRRSEEIL	TRP1	P17643	156	170	12.52
4135	31	DRB1*11:58	IFFPLLLFQQARAQF	TRP1	P17643	12	26	47.75
4136	32	DRB1*11:58	IFGTASYLIRARRSM	TRP1	P17643	493	507	30.93
4137	33	DRB1*11:58	KRTTHPLFVIATRRS	TRP1	P17643	152	166	42.57
4138	34	DRB1*11:58	LFVIATRRSEEILGP	TRP1	P17643	158	172	37.31
4139	35	DRB1*11:58	LIFGTASYLIRARRS	TRP1	P17643	492	506	43.28
4140	36	DRB1*11:58	PLFVIATRRSEEILG	TRP1	P17643	157	171	16.46
4141	37	DRB1*11:58	PLLLFQQARAQFPRQ	TRP1	P17643	15	29	38.91
4142	38	DRB1*11:58	QRVLIVRRNLLDLSK	TRP1	P17643	124	138	17.56
4143	39	DRB1*11:58	RTTHPLFVIATRRSE	TRP1	P17643	153	167	17.16
4144	40	DRB1*11:58	RVLIVRRNLLDLSKE	TRP1	P17643	125	139	27.27
4145	41	DRB1*11:58	SYLIRARRSMDEANQ	TRP1	P17643	498	512	39.58
4146	42	DRB1*11:58	TASYLIRARRSMDEA	TRP1	P17643	496	510	23.81
4147	43	DRB1*11:58	THPLFVIATRRSEEI	TRP1	P17643	155	169	12.59
4148	44	DRB1*11:58	TTHPLFVIATRRSEE	TRP1	P17643	154	168	15.45
4149	45	DRB1*11:58	VRALDMAKRTTHPLF	TRP1	P17643	145	159	31.74
4150	46	DRB1*11:58	ACQLVLHQILKGGSG	PMEL17	P40967	549	563	20.08
4151	47	DRB1*11:58	ASLIYRRRLMKQDFS	PMEL17	P40967	612	626	36.01
4152	48	DRB1*11:58	ATLRLVKRQVPLDCV	PMEL17	P40967	462	476	15.25
4153	49	DRB1*11:58	AVVLASLIYRRRLMK	PMEL17	P40967	608	622	20.89
4154	50	DRB1*11:58	CQLVLHQILKGGSGT	PMEL17	P40967	550	564	17.75
4155	51	DRB1*11:58	DGTATLRLVKRQVPL	PMEL17	P40967	459	473	9.31
4156	52	DRB1*11:58	DWLGVSRQLRTKAWN	PMEL17	P40967	31	45	44.48
4157	53	DRB1*11:58	GTATLRLVKRQVPLD	PMEL17	P40967	460	474	10.14
4158	54	DRB1*11:58	KRSFVYVWKTWGQYW	PMEL17	P40967	146	160	37.07
4159	55	DRB1*11:58	LASLIYRRRLMKQDF	PMEL17	P40967	611	625	26.62
4160	56	DRB1*11:58	LDGTATLRLVKRQVP	PMEL17	P40967	458	472	22.43
4161	57	DRB1*11:58	LGVSRQLRTKAWNRQ	PMEL17	P40967	33	47	48.19
4162	58	DRB1*11:58	LMAVVLASLIYRRRL	PMEL17	P40967	606	620	37.88
4163	59	DRB1*11:58	LVLHQILKGGSGTYC	PMEL17	P40967	552	566	20.38
4164	60	DRB1*11:58	MAVVLASLIYRRRLM	PMEL17	P40967	607	621	30.33
4165	61	DRB1*11:58	PACQLVLHQILKGGS	PMEL17	P40967	548	562	36.62
4166	62	DRB1*11:58	QDWLGVSRQLRTKAW	PMEL17	P40967	30	44	43.91

4167	63	DRB1*11:58	QKRSFVYVWKTWGQY	PMEL17	P40967	145	159	37.12
4168	64	DRB1*11:58	QLVLHQILKGGSGTY	PMEL17	P40967	551	565	15.12
4169	65	DRB1*11:58	SQKRSFVYVWKTWGQ	PMEL17	P40967	144	158	46.19
4170	66	DRB1*11:58	TATLRLVKRQVPLDC	PMEL17	P40967	461	475	11.26
4171	67	DRB1*11:58	TLRLVKRQVPLDCVL	PMEL17	P40967	463	477	31.83
4172	68	DRB1*11:58	VLASLIYRRRLMKQD	PMEL17	P40967	610	624	22.87
4173	69	DRB1*11:58	VLHQILKGGSGTYCL	PMEL17	P40967	553	567	49.95
4174	70	DRB1*11:58	VVLASLIYRRRLMKQ	PMEL17	P40967	609	623	19.21
4175	71	DRB1*11:58	WLGVSRQLRTKAWNR	PMEL17	P40967	32	46	35.36
4176	72	DRB1*11:58	ADLVGFLLLKYRARE	MAGE1	P43355	107	121	22.28
4177	73	DRB1*11:58	ARVRFFFPSLREAAL	MAGE1	P43355	288	302	42.61
4178	74	DRB1*11:58	CILESLFRAVITKKV	MAGE1	P43355	92	106	49.41
4179	75	DRB1*11:58	DLVGFLLLKYRAREP	MAGE1	P43355	108	122	17.99
4180	76	DRB1*11:58	ESLFRAVITKKVADL	MAGE1	P43355	95	109	44.48
4181	77	DRB1*11:58	EYVIKVSARVRFFFP	MAGE1	P43355	281	295	17.68
4182	78	DRB1*11:58	FLLLKYRAREPVTKA	MAGE1	P43355	112	126	33.91
4183	79	DRB1*11:58	GFLLLKYRAREPVTK	MAGE1	P43355	111	125	25.28
4184	80	DRB1*11:58	ILESLFRAVITKKVA	MAGE1	P43355	93	107	36.18
4185	81	DRB1*11:58	KVLEYVIKVSARVRF	MAGE1	P43355	278	292	14.12
4186	82	DRB1*11:58	LESLFRAVITKKVAD	MAGE1	P43355	94	108	49.59
4187	83	DRB1*11:58	LEYVIKVSARVRFFF	MAGE1	P43355	280	294	17.21
4188	84	DRB1*11:58	LVGFLLLKYRAREPV	MAGE1	P43355	109	123	17.19
4189	85	DRB1*11:58	RVRFFFPSLREAALR	MAGE1	P43355	289	303	32.94
4190	86	DRB1*11:58	SLFRAVITKKVADLV	MAGE1	P43355	96	110	44.25
4191	87	DRB1*11:58	VADLVGFLLLKYRAR	MAGE1	P43355	106	120	36.58
4192	88	DRB1*11:58	VGFLLLKYRAREPVT	MAGE1	P43355	110	124	18.8
4193	89	DRB1*11:58	VIKVSARVRFFFPSL	MAGE1	P43355	283	297	46.43
4194	90	DRB1*11:58	VKVLEYVIKVSARVR	MAGE1	P43355	277	291	21.36
4195	91	DRB1*11:58	VLEYVIKVSARVRFF	MAGE1	P43355	279	293	16.18
4196	92	DRB1*11:58	VRFFFPSLREAALRE	MAGE1	P43355	290	304	38.38
4197	93	DRB1*11:58	YVIKVSARVRFFFPS	MAGE1	P43355	282	296	23.47
4198	94	DRB1*11:58	AEMLERVIKNYKRCF	MAGE4	P43358	134	148	47.43
4199	95	DRB1*11:58	AHFLLRKYRAKELVT	MAGE4	P43358	118	132	26.1
4200	96	DRB1*11:58	DELAHFLLRKYRAKE	MAGE4	P43358	115	129	25.68
4201	97	DRB1*11:58	EHVVRVNARVRIAYP	MAGE4	P43358	289	303	35.82
4202	98	DRB1*11:58	ELAHFLLRKYRAKEL	MAGE4	P43358	116	130	19.95
4203	99	DRB1*11:58	HFLLRKYRAKELVTK	MAGE4	P43358	119	133	38.75
4204	100	DRB1*11:58	HVVRVNARVRIAYPS	MAGE4	P43358	290	304	39.92
4205	101	DRB1*11:58	KVDELAHFLLRKYRA	MAGE4	P43358	113	127	45.38
4206	102	DRB1*11:58	KVLEHVVRVNARVRI	MAGE4	P43358	286	300	14.3

4207	103	DRB1*11:58	LAHFLLRKYRAKELV	MAGE4	P43358	117	131	21.15
4208	104	DRB1*11:58	LEHVVRVNARVRIAY	MAGE4	P43358	288	302	26.61
4209	105	DRB1*11:58	SYVKVLEHVVRVNAR	MAGE4	P43358	283	297	21.83
4210	106	DRB1*11:58	TKAEMLERVIKNYKR	MAGE4	P43358	132	146	48.98
4211	107	DRB1*11:58	TSYVKVLEHVVRVNA	MAGE4	P43358	282	296	45.07
4212	108	DRB1*11:58	VDELAHFLLRKYRAK	MAGE4	P43358	114	128	28.08
4213	109	DRB1*11:58	VKVLEHVVRVNARVR	MAGE4	P43358	285	299	13.92
4214	110	DRB1*11:58	VLEHVVRVNARVRIA	MAGE4	P43358	287	301	20.88
4215	111	DRB1*11:58	YVKVLEHVVRVNARV	MAGE4	P43358	284	298	19.77
4216	112	DRB1*11:58	SGNILTIRLTAADHR	NY-ESO-1	P78358	129	143	45.87
4217	113	DRB1*11:58	ASEKIFYVYMKRKYE	SSX2	Q16385	42	56	28.14
4218	114	DRB1*11:58	EKIFYVYMKRKYEAM	SSX2	Q16385	44	58	12.34
4219	115	DRB1*11:58	FYVYMKRKYEAMTKL	SSX2	Q16385	47	61	16.24
4220	116	DRB1*11:58	IFYVYMKRKYEAMTK	SSX2	Q16385	46	60	11.71
4221	117	DRB1*11:58	KIFYVYMKRKYEAMT	SSX2	Q16385	45	59	11.33
4222	118	DRB1*11:58	SEKIFYVYMKRKYEA	SSX2	Q16385	43	57	16.45
4223	119	DRB1*11:58	YVYMKRKYEAMTKLG	SSX2	Q16385	48	62	35.29
4224	1	DRB1*11:62	EVISCKLIKRATTRQ	TRP2	O75767	222	236	30.92
4225	2	DRB1*11:62	RKFFHRTCKCTGNFA	TRP2	O75767	88	102	39.55
4226	3	DRB1*11:62	VISCKLIKRATTRQP	TRP2	O75767	223	237	33.86
4227	4	DRB1*11:62	WEVISCKLIKRATTR	TRP2	O75767	221	235	36.89
4228	5	DRB1*11:62	YYYRFVIGLRVWQWE	TRP2	O75767	208	222	49.95
4229	6	DRB1*11:62	DKFFAYLTLAKHTIS	Tyrosinase	P14679	132	146	26.01
4230	7	DRB1*11:62	FAYLTLAKHTISSDY	Tyrosinase	P14679	135	149	35.24
4231	8	DRB1*11:62	FFAYLTLAKHTISSD	Tyrosinase	P14679	134	148	28.57
4232	9	DRB1*11:62	KDKFFAYLTLAKHTI	Tyrosinase	P14679	131	145	33.33
4233	10	DRB1*11:62	KFFAYLTLAKHTISS	Tyrosinase	P14679	133	147	22.47
4234	11	DRB1*11:62	MVPFIPLYRNGDFFI	Tyrosinase	P14679	426	440	32.73
4235	12	DRB1*11:62	VPFIPLYRNGDFFIS	Tyrosinase	P14679	427	441	41.52
4236	13	DRB1*11:62	YMVPFIPLYRNGDFF	Tyrosinase	P14679	425	439	42.2
4237	14	DRB1*11:62	ASYLIRARRSMDEAN	TRP1	P17643	497	511	46.19
4238	15	DRB1*11:62	FVWTHYYSVKKTFLG	TRP1	P17643	188	202	47.59
4239	16	DRB1*11:62	HPLFVIATRRSEEIL	TRP1	P17643	156	170	13.74
4240	17	DRB1*11:62	LFVIATRRSEEILGP	TRP1	P17643	158	172	24.07
4241	18	DRB1*11:62	PLFVIATRRSEEILG	TRP1	P17643	157	171	15.42
4242	19	DRB1*11:62	RTTHPLFVIATRRSE	TRP1	P17643	153	167	24.44
4243	20	DRB1*11:62	TASYLIRARRSMDEA	TRP1	P17643	496	510	39.65
4244	21	DRB1*11:62	THPLFVIATRRSEEI	TRP1	P17643	155	169	15.19
4245	22	DRB1*11:62	THYYSVKKTFLGVGQ	TRP1	P17643	191	205	37.63

4246	23	DRB1*11:62	TTHPLFVIATRRSEE	TRP1	P17643	154	168	19.39
4247	24	DRB1*11:62	VWTHYYSVKKTFLGV	TRP1	P17643	189	203	31.01
4248	25	DRB1*11:62	WTHYYSVKKTFLGVG	TRP1	P17643	190	204	32.76
4249	26	DRB1*11:62	ANASFSIALNFPGSQ	PMEL17	P40967	80	94	49.44
4250	27	DRB1*11:62	ASFSIALNFPGSQKV	PMEL17	P40967	82	96	42.13
4251	28	DRB1*11:62	ATLRLVKRQVPLDCV	PMEL17	P40967	462	476	32.58
4252	29	DRB1*11:62	CQLVLHQILKGGSGT	PMEL17	P40967	550	564	45.41
4253	30	DRB1*11:62	DGTATLRLVKRQVPL	PMEL17	P40967	459	473	18.27
4254	31	DRB1*11:62	GSRSYVPLAHSSSAF	PMEL17	P40967	193	207	39.7
4255	32	DRB1*11:62	GTATLRLVKRQVPLD	PMEL17	P40967	460	474	20.16
4256	33	DRB1*11:62	KRSFVYVWKTWGQYW	PMEL17	P40967	146	160	17.94
4257	34	DRB1*11:62	NASFSIALNFPGSQK	PMEL17	P40967	81	95	33.06
4258	35	DRB1*11:62	QKRSFVYVWKTWGQY	PMEL17	P40967	145	159	18.3
4259	36	DRB1*11:62	QLVLHQILKGGSGTY	PMEL17	P40967	551	565	36.77
4260	37	DRB1*11:62	RSFVYVWKTWGQYWQ	PMEL17	P40967	147	161	30.25
4261	38	DRB1*11:62	RSYVPLAHSSSAFTI	PMEL17	P40967	195	209	42.32
4262	39	DRB1*11:62	SQKRSFVYVWKTWGQ	PMEL17	P40967	144	158	21.74
4263	40	DRB1*11:62	SRSYVPLAHSSSAFT	PMEL17	P40967	194	208	39.52
4264	41	DRB1*11:62	TATLRLVKRQVPLDC	PMEL17	P40967	461	475	23.06
4265	42	DRB1*11:62	WSQKRSFVYVWKTWG	PMEL17	P40967	143	157	41.25
4266	43	DRB1*11:62	ADLVGFLLLKYRARE	MAGE1	P43355	107	121	34.07
4267	44	DRB1*11:62	ARVRFFFPSLREAAL	MAGE1	P43355	288	302	21.89
4268	45	DRB1*11:62	ARYEFLWGPRALAET	MAGE1	P43355	260	274	33.84
4269	46	DRB1*11:62	DLVGFLLLKYRAREP	MAGE1	P43355	108	122	28.93
4270	47	DRB1*11:62	ESLFRAVITKKVADL	MAGE1	P43355	95	109	29.13
4271	48	DRB1*11:62	EYVIKVSARVRFFFP	MAGE1	P43355	281	295	39.57
4272	49	DRB1*11:62	FFFPSLREAALREEE	MAGE1	P43355	292	306	45.29
4273	50	DRB1*11:62	GFLLLKYRAREPVTK	MAGE1	P43355	111	125	45.81
4274	51	DRB1*11:62	ILESLFRAVITKKVA	MAGE1	P43355	93	107	32.17
4275	52	DRB1*11:62	KVLEYVIKVSARVRF	MAGE1	P43355	278	292	25.6
4276	53	DRB1*11:62	LESLFRAVITKKVAD	MAGE1	P43355	94	108	37.28
4277	54	DRB1*11:62	LEYVIKVSARVRFFF	MAGE1	P43355	280	294	34.02
4278	55	DRB1*11:62	LVGFLLLKYRAREPV	MAGE1	P43355	109	123	26.38
4279	56	DRB1*11:62	PARYEFLWGPRALAE	MAGE1	P43355	259	273	37.79
4280	57	DRB1*11:62	RFFFPSLREAALREE	MAGE1	P43355	291	305	18.8
4281	58	DRB1*11:62	RVRFFFPSLREAALR	MAGE1	P43355	289	303	14
4282	59	DRB1*11:62	RYEFLWGPRALAETS	MAGE1	P43355	261	275	34.81
4283	60	DRB1*11:62	SARVRFFFPSLREAA	MAGE1	P43355	287	301	49.09
4284	61	DRB1*11:62	SLFRAVITKKVADLV	MAGE1	P43355	96	110	32.18
4285	62	DRB1*11:62	VGFLLLKYRAREPVT	MAGE1	P43355	110	124	31.71

4286	63	DRB1*11:62	VKVLEYVIKVSARVR	MAGE1	P43355	277	291	29.38
4287	64	DRB1*11:62	VLEYVIKVSARVRFF	MAGE1	P43355	279	293	31.77
4288	65	DRB1*11:62	VRFFFPSLREAALRE	MAGE1	P43355	290	304	14.6
4289	66	DRB1*11:62	AHFLLRKYRAKELVT	MAGE4	P43358	118	132	42.25
4290	67	DRB1*11:62	ARYEFLWGPRALAET	MAGE4	P43358	268	282	33.84
4291	68	DRB1*11:62	DELAHFLLRKYRAKE	MAGE4	P43358	115	129	39.02
4292	69	DRB1*11:62	ELAHFLLRKYRAKEL	MAGE4	P43358	116	130	28.81
4293	70	DRB1*11:62	KVLEHVVRVNARVRI	MAGE4	P43358	286	300	31.5
4294	71	DRB1*11:62	LAHFLLRKYRAKELV	MAGE4	P43358	117	131	29.7
4295	72	DRB1*11:62	PARYEFLWGPRALAE	MAGE4	P43358	267	281	37.79
4296	73	DRB1*11:62	RYEFLWGPRALAETS	MAGE4	P43358	269	283	34.81
4297	74	DRB1*11:62	SYVKVLEHVVRVNAR	MAGE4	P43358	283	297	47.81
4298	75	DRB1*11:62	VDELAHFLLRKYRAK	MAGE4	P43358	114	128	43.08
4299	76	DRB1*11:62	VKVLEHVVRVNARVR	MAGE4	P43358	285	299	30.17
4300	77	DRB1*11:62	YVKVLEHVVRVNARV	MAGE4	P43358	284	298	40.41
4301	78	DRB1*11:62	RAVFLALSAQLLQAR	BAGE	Q13072	4	18	49.7
4302	79	DRB1*11:62	ASEKIFYVYMKRKYE	SSX2	Q16385	42	56	21.98
4303	80	DRB1*11:62	EKIFYVYMKRKYEAM	SSX2	Q16385	44	58	12.58
4304	81	DRB1*11:62	FYVYMKRKYEAMTKL	SSX2	Q16385	47	61	19.78
4305	82	DRB1*11:62	IFYVYMKRKYEAMTK	SSX2	Q16385	46	60	13.87
4306	83	DRB1*11:62	KIFYVYMKRKYEAMT	SSX2	Q16385	45	59	12.77
4307	84	DRB1*11:62	KRKYEAMTKLGFKAT	SSX2	Q16385	52	66	47.42
4308	85	DRB1*11:62	MKRKYEAMTKLGFKA	SSX2	Q16385	51	65	47.51
4309	86	DRB1*11:62	SEKIFYVYMKRKYEA	SSX2	Q16385	43	57	15.39
4310	1	DRB1*11:65	EVISCKLIKRATTRQ	TRP2	O75767	222	236	39.78
4311	2	DRB1*11:65	VISCKLIKRATTRQP	TRP2	O75767	223	237	48.12
4312	3	DRB1*11:65	WEVISCKLIKRATTR	TRP2	O75767	221	235	39.15
4313	4	DRB1*11:65	AGLVSLLCRHKRKQL	Tyrosinase	P14679	493	507	19.74
4314	5	DRB1*11:65	ALLAGLVSLLCRHKR	Tyrosinase	P14679	490	504	49.75
4315	6	DRB1*11:65	GLVSLLCRHKRKQLP	Tyrosinase	P14679	494	508	22.97
4316	7	DRB1*11:65	LAGLVSLLCRHKRKQ	Tyrosinase	P14679	492	506	26.28
4317	8	DRB1*11:65	LLAGLVSLLCRHKRK	Tyrosinase	P14679	491	505	29.59
4318	9	DRB1*11:65	LVSLLCRHKRKQLPE	Tyrosinase	P14679	495	509	32.55
4319	10	DRB1*11:65	NRESYMVPFIPLYRN	Tyrosinase	P14679	421	435	38.22
4320	11	DRB1*11:65	RESYMVPFIPLYRNG	Tyrosinase	P14679	422	436	37.93
4321	12	DRB1*11:65	AACDQRVLIVRRNLL	TRP1	P17643	120	134	43.42
4322	13	DRB1*11:65	ACDQRVLIVRRNLLD	TRP1	P17643	121	135	32.47
4323	14	DRB1*11:65	AFLTWHRYHLLRLEK	TRP1	P17643	219	233	44.85
4324	15	DRB1*11:65	CDQRVLIVRRNLLDL	TRP1	P17643	122	136	25.37

4325	16	DRB1*11:65	DQRVLIVRRNLLDLS	TRP1	P17643	123	137	23
4326	17	DRB1*11:65	EGPAFLTWHRYHLLR	TRP1	P17643	216	230	44.44
4327	18	DRB1*11:65	FFPLLLFQQARAQFP	TRP1	P17643	13	27	46.29
4328	19	DRB1*11:65	FPLLLFQQARAQFPR	TRP1	P17643	14	28	34.58
4329	20	DRB1*11:65	GPAFLTWHRYHLLRL	TRP1	P17643	217	231	35.26
4330	21	DRB1*11:65	HPLFVIATRRSEEIL	TRP1	P17643	156	170	40.79
4331	22	DRB1*11:65	IFGTASYLIRARRSM	TRP1	P17643	493	507	39.73
4332	23	DRB1*11:65	LIFGTASYLIRARRS	TRP1	P17643	492	506	49.31
4333	24	DRB1*11:65	PAFLTWHRYHLLRLE	TRP1	P17643	218	232	38.5
4334	25	DRB1*11:65	PLLLFQQARAQFPRQ	TRP1	P17643	15	29	42.53
4335	26	DRB1*11:65	QRVLIVRRNLLDLSK	TRP1	P17643	124	138	20.24
4336	27	DRB1*11:65	RVLIVRRNLLDLSKE	TRP1	P17643	125	139	31.53
4337	28	DRB1*11:65	THPLFVIATRRSEEI	TRP1	P17643	155	169	37.93
4338	29	DRB1*11:65	ASLIYRRRLMKQDFS	PMEL17	P40967	612	626	30.9
4339	30	DRB1*11:65	ATLRLVKRQVPLDCV	PMEL17	P40967	462	476	33.5
4340	31	DRB1*11:65	AVVLASLIYRRRLMK	PMEL17	P40967	608	622	16.96
4341	32	DRB1*11:65	DGTATLRLVKRQVPL	PMEL17	P40967	459	473	22.93
4342	33	DRB1*11:65	EVTVYHRRGSRSYVP	PMEL17	P40967	185	199	47.92
4343	34	DRB1*11:65	GNKHFLRNQPLTFAL	PMEL17	P40967	228	242	45.46
4344	35	DRB1*11:65	GTATLRLVKRQVPLD	PMEL17	P40967	460	474	26.58
4345	36	DRB1*11:65	KHFLRNQPLTFALQL	PMEL17	P40967	230	244	45.78
4346	37	DRB1*11:65	LASLIYRRRLMKQDF	PMEL17	P40967	611	625	21.1
4347	38	DRB1*11:65	LDGTATLRLVKRQVP	PMEL17	P40967	458	472	45.35
4348	39	DRB1*11:65	LMAVVLASLIYRRRL	PMEL17	P40967	606	620	26.81
4349	40	DRB1*11:65	MAVVLASLIYRRRLM	PMEL17	P40967	607	621	21.84
4350	41	DRB1*11:65	MEVTVYHRRGSRSYV	PMEL17	P40967	184	198	34.06
4351	42	DRB1*11:65	NKHFLRNQPLTFALQ	PMEL17	P40967	229	243	41.83
4352	43	DRB1*11:65	SLIYRRRLMKQDFSV	PMEL17	P40967	613	627	44.49
4353	44	DRB1*11:65	TATLRLVKRQVPLDC	PMEL17	P40967	461	475	28.64
4354	45	DRB1*11:65	TLRLVKRQVPLDCVL	PMEL17	P40967	463	477	48.63
4355	46	DRB1*11:65	TMEVTVYHRRGSRSY	PMEL17	P40967	183	197	42.25
4356	47	DRB1*11:65	VLASLIYRRRLMKQD	PMEL17	P40967	610	624	18.87
4357	48	DRB1*11:65	VLMAVVLASLIYRRR	PMEL17	P40967	605	619	37.73
4358	49	DRB1*11:65	VTAQVVLQAAIPLTS	PMEL17	P40967	286	300	39.64
4359	50	DRB1*11:65	VVLASLIYRRRLMKQ	PMEL17	P40967	609	623	15.16
4360	51	DRB1*11:65	ADLVGFLLLKYRARE	MAGE1	P43355	107	121	40.53
4361	52	DRB1*11:65	ARVRFFFPSLREAAL	MAGE1	P43355	288	302	48.15
4362	53	DRB1*11:65	DLVGFLLLKYRAREP	MAGE1	P43355	108	122	42.83
4363	54	DRB1*11:65	EYVIKVSARVRFFFP	MAGE1	P43355	281	295	9.36
4364	55	DRB1*11:65	FLLLKYRAREPVTKA	MAGE1	P43355	112	126	31.43

4365	56	DRB1*11:65	GFLLLKYRAREPVTK	MAGE1	P43355	111	125	28.99
4366	57	DRB1*11:65	IKVSARVRFFFPSLR	MAGE1	P43355	284	298	40.89
4367	58	DRB1*11:65	KVLEYVIKVSARVRF	MAGE1	P43355	278	292	12.95
4368	59	DRB1*11:65	LEYVIKVSARVRFFF	MAGE1	P43355	280	294	9.32
4369	60	DRB1*11:65	LVGFLLLKYRAREPV	MAGE1	P43355	109	123	29.64
4370	61	DRB1*11:65	SARVRFFFPSLREAA	MAGE1	P43355	287	301	44.9
4371	62	DRB1*11:65	VGFLLLKYRAREPVT	MAGE1	P43355	110	124	27.92
4372	63	DRB1*11:65	VIKVSARVRFFFPSL	MAGE1	P43355	283	297	21.98
4373	64	DRB1*11:65	VKVLEYVIKVSARVR	MAGE1	P43355	277	291	24.8
4374	65	DRB1*11:65	VLEYVIKVSARVRFF	MAGE1	P43355	279	293	9.97
4375	66	DRB1*11:65	YVIKVSARVRFFFPS	MAGE1	P43355	282	296	12.43
4376	67	DRB1*11:65	AEMLERVIKNYKRCF	MAGE4	P43358	134	148	43.68
4377	68	DRB1*11:65	AHFLLRKYRAKELVT	MAGE4	P43358	118	132	35.84
4378	69	DRB1*11:65	ARVRIAYPSLREAAL	MAGE4	P43358	296	310	40.7
4379	70	DRB1*11:65	EHVVRVNARVRIAYP	MAGE4	P43358	289	303	11.58
4380	71	DRB1*11:65	ELAHFLLRKYRAKEL	MAGE4	P43358	116	130	34.56
4381	72	DRB1*11:65	EMLERVIKNYKRCFP	MAGE4	P43358	135	149	40.71
4382	73	DRB1*11:65	ERVIKNYKRCFPVIF	MAGE4	P43358	138	152	35.86
4383	74	DRB1*11:65	HFLLRKYRAKELVTK	MAGE4	P43358	119	133	38.09
4384	75	DRB1*11:65	HVVRVNARVRIAYPS	MAGE4	P43358	290	304	12.34
4385	76	DRB1*11:65	KVLEHVVRVNARVRI	MAGE4	P43358	286	300	14.8
4386	77	DRB1*11:65	LAHFLLRKYRAKELV	MAGE4	P43358	117	131	31.45
4387	78	DRB1*11:65	LEHVVRVNARVRIAY	MAGE4	P43358	288	302	9.7
4388	79	DRB1*11:65	LERVIKNYKRCFPVI	MAGE4	P43358	137	151	30.05
4389	80	DRB1*11:65	MLERVIKNYKRCFPV	MAGE4	P43358	136	150	32.77
4390	81	DRB1*11:65	NARVRIAYPSLREAA	MAGE4	P43358	295	309	36.41
4391	82	DRB1*11:65	RVNARVRIAYPSLRE	MAGE4	P43358	293	307	29.94
4392	83	DRB1*11:65	VKVLEHVVRVNARVR	MAGE4	P43358	285	299	27.4
4393	84	DRB1*11:65	VLEHVVRVNARVRIA	MAGE4	P43358	287	301	12.31
4394	85	DRB1*11:65	VNARVRIAYPSLREA	MAGE4	P43358	294	308	37.07
4395	86	DRB1*11:65	VRVNARVRIAYPSLR	MAGE4	P43358	292	306	24.23
4396	87	DRB1*11:65	VVRVNARVRIAYPSL	MAGE4	P43358	291	305	16.81
4397	88	DRB1*11:65	ILTIRLTAADHRQLQ	NY-ESO-1	P78358	132	146	42.73
4398	89	DRB1*11:65	NILTIRLTAADHRQL	NY-ESO-1	P78358	131	145	45.24
4399	90	DRB1*11:65	EKIFYVYMKRKYEAM	SSX2	Q16385	44	58	27.64
4400	91	DRB1*11:65	FYVYMKRKYEAMTKL	SSX2	Q16385	47	61	39.09
4401	92	DRB1*11:65	IFYVYMKRKYEAMTK	SSX2	Q16385	46	60	28.77
4402	93	DRB1*11:65	KIFYVYMKRKYEAMT	SSX2	Q16385	45	59	26.59
4403	94	DRB1*11:65	SEKIFYVYMKRKYEA	SSX2	Q16385	43	57	37.1
4404	95	DRB1*11:65	WEKMKASEKIFYVYM	SSX2	Q16385	37	51	45.94

4405	1	DRB1*11:74	EVISCKLIKRATTRQ	TRP2	O75767	222	236	30.92
4406	2	DRB1*11:74	RKFFHRTCKCTGNFA	TRP2	O75767	88	102	39.55
4407	3	DRB1*11:74	VISCKLIKRATTRQP	TRP2	O75767	223	237	33.86
4408	4	DRB1*11:74	WEVISCKLIKRATTR	TRP2	O75767	221	235	36.89
4409	5	DRB1*11:74	YYYRFVIGLRVWQWE	TRP2	O75767	208	222	49.95
4410	6	DRB1*11:74	DKFFAYLTLAKHTIS	Tyrosinase	P14679	132	146	26.01
4411	7	DRB1*11:74	FAYLTLAKHTISSDY	Tyrosinase	P14679	135	149	35.24
4412	8	DRB1*11:74	FFAYLTLAKHTISSD	Tyrosinase	P14679	134	148	28.57
4413	9	DRB1*11:74	KDKFFAYLTLAKHTI	Tyrosinase	P14679	131	145	33.33
4414	10	DRB1*11:74	KFFAYLTLAKHTISS	Tyrosinase	P14679	133	147	22.47
4415	11	DRB1*11:74	MVPFIPLYRNGDFFI	Tyrosinase	P14679	426	440	32.73
4416	12	DRB1*11:74	VPFIPLYRNGDFFIS	Tyrosinase	P14679	427	441	41.52
4417	13	DRB1*11:74	YMVPFIPLYRNGDFF	Tyrosinase	P14679	425	439	42.2
4418	14	DRB1*11:74	ASYLIRARRSMDEAN	TRP1	P17643	497	511	46.19
4419	15	DRB1*11:74	FVWTHYYSVKKTFLG	TRP1	P17643	188	202	47.59
4420	16	DRB1*11:74	HPLFVIATRRSEEIL	TRP1	P17643	156	170	13.74
4421	17	DRB1*11:74	LFVIATRRSEEILGP	TRP1	P17643	158	172	24.07
4422	18	DRB1*11:74	PLFVIATRRSEEILG	TRP1	P17643	157	171	15.42
4423	19	DRB1*11:74	RTTHPLFVIATRRSE	TRP1	P17643	153	167	24.44
4424	20	DRB1*11:74	TASYLIRARRSMDEA	TRP1	P17643	496	510	39.65
4425	21	DRB1*11:74	THPLFVIATRRSEEI	TRP1	P17643	155	169	15.19
4426	22	DRB1*11:74	THYYSVKKTFLGVGQ	TRP1	P17643	191	205	37.63
4427	23	DRB1*11:74	TTHPLFVIATRRSEE	TRP1	P17643	154	168	19.39
4428	24	DRB1*11:74	VWTHYYSVKKTFLGV	TRP1	P17643	189	203	31.01
4429	25	DRB1*11:74	WTHYYSVKKTFLGVG	TRP1	P17643	190	204	32.76
4430	26	DRB1*11:74	ANASFSIALNFPGSQ	PMEL17	P40967	80	94	49.44
4431	27	DRB1*11:74	ASFSIALNFPGSQKV	PMEL17	P40967	82	96	42.13
4432	28	DRB1*11:74	ATLRLVKRQVPLDCV	PMEL17	P40967	462	476	32.58
4433	29	DRB1*11:74	CQLVLHQILKGGSGT	PMEL17	P40967	550	564	45.41
4434	30	DRB1*11:74	DGTATLRLVKRQVPL	PMEL17	P40967	459	473	18.27
4435	31	DRB1*11:74	GSRSYVPLAHSSSAF	PMEL17	P40967	193	207	39.7
4436	32	DRB1*11:74	GTATLRLVKRQVPLD	PMEL17	P40967	460	474	20.16
4437	33	DRB1*11:74	KRSFVYVWKTWGQYW	PMEL17	P40967	146	160	17.94
4438	34	DRB1*11:74	NASFSIALNFPGSQK	PMEL17	P40967	81	95	33.06
4439	35	DRB1*11:74	QKRSFVYVWKTWGQY	PMEL17	P40967	145	159	18.3
4440	36	DRB1*11:74	QLVLHQILKGGSGTY	PMEL17	P40967	551	565	36.77
4441	37	DRB1*11:74	RSFVYVWKTWGQYWQ	PMEL17	P40967	147	161	30.25
4442	38	DRB1*11:74	RSYVPLAHSSSAFTI	PMEL17	P40967	195	209	42.32
4443	39	DRB1*11:74	SQKRSFVYVWKTWGQ	PMEL17	P40967	144	158	21.74

4444	40	DRB1*11:74	SRSYVPLAHSSSAFT	PMEL17	P40967	194	208	39.52
4445	41	DRB1*11:74	TATLRLVKRQVPLDC	PMEL17	P40967	461	475	23.06
4446	42	DRB1*11:74	WSQKRSFVYVWKTWG	PMEL17	P40967	143	157	41.25
4447	43	DRB1*11:74	ADLVGFLLLKYRARE	MAGE1	P43355	107	121	34.07
4448	44	DRB1*11:74	ARVRFFFPSLREAAL	MAGE1	P43355	288	302	21.89
4449	45	DRB1*11:74	ARYEFLWGPRALAET	MAGE1	P43355	260	274	33.84
4450	46	DRB1*11:74	DLVGFLLLKYRAREP	MAGE1	P43355	108	122	28.93
4451	47	DRB1*11:74	ESLFRAVITKKVADL	MAGE1	P43355	95	109	29.13
4452	48	DRB1*11:74	EYVIKVSARVRFFFP	MAGE1	P43355	281	295	39.57
4453	49	DRB1*11:74	FFFPSLREAALREEE	MAGE1	P43355	292	306	45.29
4454	50	DRB1*11:74	GFLLLKYRAREPVTK	MAGE1	P43355	111	125	45.81
4455	51	DRB1*11:74	ILESLFRAVITKKVA	MAGE1	P43355	93	107	32.17
4456	52	DRB1*11:74	KVLEYVIKVSARVRF	MAGE1	P43355	278	292	25.6
4457	53	DRB1*11:74	LESLFRAVITKKVAD	MAGE1	P43355	94	108	37.28
4458	54	DRB1*11:74	LEYVIKVSARVRFFF	MAGE1	P43355	280	294	34.02
4459	55	DRB1*11:74	LVGFLLLKYRAREPV	MAGE1	P43355	109	123	26.38
4460	56	DRB1*11:74	PARYEFLWGPRALAE	MAGE1	P43355	259	273	37.79
4461	57	DRB1*11:74	RFFFPSLREAALREE	MAGE1	P43355	291	305	18.8
4462	58	DRB1*11:74	RVRFFFPSLREAALR	MAGE1	P43355	289	303	14
4463	59	DRB1*11:74	RYEFLWGPRALAETS	MAGE1	P43355	261	275	34.81
4464	60	DRB1*11:74	SARVRFFFPSLREAA	MAGE1	P43355	287	301	49.09
4465	61	DRB1*11:74	SLFRAVITKKVADLV	MAGE1	P43355	96	110	32.18
4466	62	DRB1*11:74	VGFLLLKYRAREPVT	MAGE1	P43355	110	124	31.71
4467	63	DRB1*11:74	VKVLEYVIKVSARVR	MAGE1	P43355	277	291	29.38
4468	64	DRB1*11:74	VLEYVIKVSARVRFF	MAGE1	P43355	279	293	31.77
4469	65	DRB1*11:74	VRFFFPSLREAALRE	MAGE1	P43355	290	304	14.6
4470	66	DRB1*11:74	AHFLLRKYRAKELVT	MAGE4	P43358	118	132	42.25
4471	67	DRB1*11:74	ARYEFLWGPRALAET	MAGE4	P43358	268	282	33.84
4472	68	DRB1*11:74	DELAHFLLRKYRAKE	MAGE4	P43358	115	129	39.02
4473	69	DRB1*11:74	ELAHFLLRKYRAKEL	MAGE4	P43358	116	130	28.81
4474	70	DRB1*11:74	KVLEHVVRVNARVRI	MAGE4	P43358	286	300	31.5
4475	71	DRB1*11:74	LAHFLLRKYRAKELV	MAGE4	P43358	117	131	29.7
4476	72	DRB1*11:74	PARYEFLWGPRALAE	MAGE4	P43358	267	281	37.79
4477	73	DRB1*11:74	RYEFLWGPRALAETS	MAGE4	P43358	269	283	34.81
4478	74	DRB1*11:74	SYVKVLEHVVRVNAR	MAGE4	P43358	283	297	47.81
4479	75	DRB1*11:74	VDELAHFLLRKYRAK	MAGE4	P43358	114	128	43.08
4480	76	DRB1*11:74	VKVLEHVVRVNARVR	MAGE4	P43358	285	299	30.17
4481	77	DRB1*11:74	YVKVLEHVVRVNARV	MAGE4	P43358	284	298	40.41
4482	78	DRB1*11:74	RAVFLALSAQLLQAR	BAGE	Q13072	4	18	49.7
4483	79	DRB1*11:74	ASEKIFYVYMKRKYE	SSX2	Q16385	42	56	21.98

4484	80	DRB1*11:74	EKIFYVYMKRKYEAM	SSX2	Q16385	44	58	12.58
4485	81	DRB1*11:74	FYVYMKRKYEAMTKL	SSX2	Q16385	47	61	19.78
4486	82	DRB1*11:74	IFYVYMKRKYEAMTK	SSX2	Q16385	46	60	13.87
4487	83	DRB1*11:74	KIFYVYMKRKYEAMT	SSX2	Q16385	45	59	12.77
4488	84	DRB1*11:74	KRKYEAMTKLGFKAT	SSX2	Q16385	52	66	47.42
4489	85	DRB1*11:74	MKRKYEAMTKLGFKA	SSX2	Q16385	51	65	47.51
4490	86	DRB1*11:74	SEKIFYVYMKRKYEA	SSX2	Q16385	43	57	15.39
4491	1	DRB1*11:84	EVISCKLIKRATTRQ	TRP2	O75767	222	236	46.41
4492	2	DRB1*11:84	AGLVSLLCRHKRKQL	Tyrosinase	P14679	493	507	44.98
4493	3	DRB1*11:84	CDQRVLIVRRNLLDL	TRP1	P17643	122	136	41.52
4494	4	DRB1*11:84	DQRVLIVRRNLLDLS	TRP1	P17643	123	137	37.89
4495	5	DRB1*11:84	HPLFVIATRRSEEIL	TRP1	P17643	156	170	40.18
4496	6	DRB1*11:84	QRVLIVRRNLLDLSK	TRP1	P17643	124	138	32.71
4497	7	DRB1*11:84	THPLFVIATRRSEEI	TRP1	P17643	155	169	37.83
4498	8	DRB1*11:84	TTHPLFVIATRRSEE	TRP1	P17643	154	168	49.87
4499	9	DRB1*11:84	AVVLASLIYRRRLMK	PMEL17	P40967	608	622	38.65
4500	10	DRB1*11:84	DGTATLRLVKRQVPL	PMEL17	P40967	459	473	27.15
4501	11	DRB1*11:84	GTATLRLVKRQVPLD	PMEL17	P40967	460	474	29.01
4502	12	DRB1*11:84	TATLRLVKRQVPLDC	PMEL17	P40967	461	475	35.74
4503	13	DRB1*11:84	VLASLIYRRRLMKQD	PMEL17	P40967	610	624	45.75
4504	14	DRB1*11:84	VVLASLIYRRRLMKQ	PMEL17	P40967	609	623	35.36
4505	15	DRB1*11:84	DLVGFLLLKYRAREP	MAGE1	P43355	108	122	45.88
4506	16	DRB1*11:84	EYVIKVSARVRFFFP	MAGE1	P43355	281	295	28.25
4507	17	DRB1*11:84	KVLEYVIKVSARVRF	MAGE1	P43355	278	292	27.07
4508	18	DRB1*11:84	LEYVIKVSARVRFFF	MAGE1	P43355	280	294	26.66
4509	19	DRB1*11:84	LVGFLLLKYRAREPV	MAGE1	P43355	109	123	39.21
4510	20	DRB1*11:84	VGFLLLKYRAREPVT	MAGE1	P43355	110	124	44.21
4511	21	DRB1*11:84	VKVLEYVIKVSARVR	MAGE1	P43355	277	291	39.08
4512	22	DRB1*11:84	VLEYVIKVSARVRFF	MAGE1	P43355	279	293	27.42
4513	23	DRB1*11:84	YVIKVSARVRFFFPS	MAGE1	P43355	282	296	37.73
4514	24	DRB1*11:84	KVLEHVVRVNARVRI	MAGE4	P43358	286	300	36.07
4515	25	DRB1*11:84	LEHVVRVNARVRIAY	MAGE4	P43358	288	302	43.4
4516	26	DRB1*11:84	VKVLEHVVRVNARVR	MAGE4	P43358	285	299	34.22
4517	27	DRB1*11:84	VLEHVVRVNARVRIA	MAGE4	P43358	287	301	47.19
4518	28	DRB1*11:84	EKIFYVYMKRKYEAM	SSX2	Q16385	44	58	34.86
4519	29	DRB1*11:84	IFYVYMKRKYEAMTK	SSX2	Q16385	46	60	35.97
4520	30	DRB1*11:84	KIFYVYMKRKYEAMT	SSX2	Q16385	45	59	34.03
4521	31	DRB1*11:84	SEKIFYVYMKRKYEA	SSX2	Q16385	43	57	46.83

4522	1	DRB1*12:02	ELAHFLLRKYRAKEL	MAGE4	P43358	116	130	47.63
4523	2	DRB1*12:02	VDELAHFLLRKYRAK	MAGE4	P43358	114	128	44.46
4524	1	DRB1*12:03	LMAVVLASLIYRRRL	PMEL17	P40967	606	620	49.45
4525	2	DRB1*12:03	MAVVLASLIYRRRLM	PMEL17	P40967	607	621	44.58
4526	3	DRB1*12:03	VTAQVVLQAAIPLTS	PMEL17	P40967	286	300	48.5
4527	4	DRB1*12:03	ADLVGFLLLKYRARE	MAGE1	P43355	107	121	41.32
4528	5	DRB1*12:03	DLVGFLLLKYRAREP	MAGE1	P43355	108	122	49.86
4529	6	DRB1*12:03	EYVIKVSARVRFFFP	MAGE1	P43355	281	295	38.64
4530	7	DRB1*12:03	KVLEYVIKVSARVRF	MAGE1	P43355	278	292	46.87
4531	8	DRB1*12:03	LEYVIKVSARVRFFF	MAGE1	P43355	280	294	37.49
4532	9	DRB1*12:03	VADLVGFLLLKYRAR	MAGE1	P43355	106	120	40.91
4533	10	DRB1*12:03	VLEYVIKVSARVRFF	MAGE1	P43355	279	293	39.35
4534	11	DRB1*12:03	YVIKVSARVRFFFPS	MAGE1	P43355	282	296	49.92
4535	1	DRB1*12:16	DKFFAYLTLAKHTIS	Tyrosinase	P14679	132	146	38.91
4536	2	DRB1*12:16	KDKFFAYLTLAKHTI	Tyrosinase	P14679	131	145	37.91
4537	3	DRB1*12:16	KFFAYLTLAKHTISS	Tyrosinase	P14679	133	147	38.94
4538	4	DRB1*12:16	ADLVGFLLLKYRARE	MAGE1	P43355	107	121	43.73
4539	5	DRB1*12:16	DLVGFLLLKYRAREP	MAGE1	P43355	108	122	43.87
4540	6	DRB1*12:16	ESLFRAVITKKVADL	MAGE1	P43355	95	109	43.26
4541	7	DRB1*12:16	ILESLFRAVITKKVA	MAGE1	P43355	93	107	40.39
4542	8	DRB1*12:16	LVGFLLLKYRAREPV	MAGE1	P43355	109	123	46.68
4543	9	DRB1*12:16	SLFRAVITKKVADLV	MAGE1	P43355	96	110	44.96
4544	10	DRB1*12:16	VADLVGFLLLKYRAR	MAGE1	P43355	106	120	48.62
4545	11	DRB1*12:16	AARAVFLALSAQLLQ	BAGE	Q13072	2	16	47.78
4546	12	DRB1*12:16	ARAVFLALSAQLLQA	BAGE	Q13072	3	17	46.03
4547	13	DRB1*12:16	RAVFLALSAQLLQAR	BAGE	Q13072	4	18	28.77
4548	14	DRB1*13:01	EVISCKLIKRATTRQ	TRP2	O75767	222	236	39.78
4549	15	DRB1*13:01	VISCKLIKRATTRQP	TRP2	O75767	223	237	48.12
4550	16	DRB1*13:01	WEVISCKLIKRATTR	TRP2	O75767	221	235	39.15
4551	17	DRB1*13:01	AGLVSLLCRHKRKQL	Tyrosinase	P14679	493	507	19.74
4552	18	DRB1*13:01	ALLAGLVSLLCRHKR	Tyrosinase	P14679	490	504	49.75
4553	19	DRB1*13:01	GLVSLLCRHKRKQLP	Tyrosinase	P14679	494	508	22.97
4554	20	DRB1*13:01	LAGLVSLLCRHKRKQ	Tyrosinase	P14679	492	506	26.28
4555	21	DRB1*13:01	LLAGLVSLLCRHKRK	Tyrosinase	P14679	491	505	29.59
4556	22	DRB1*13:01	LVSLLCRHKRKQLPE	Tyrosinase	P14679	495	509	32.55
4557	23	DRB1*13:01	NRESYMVPFIPLYRN	Tyrosinase	P14679	421	435	38.22
4558	24	DRB1*13:01	RESYMVPFIPLYRNG	Tyrosinase	P14679	422	436	37.93

4559	25	DRB1*13:01	AACDQRVLIVRRNLL	TRP1	P17643	120	134	43.42
4560	26	DRB1*13:01	ACDQRVLIVRRNLLD	TRP1	P17643	121	135	32.47
4561	27	DRB1*13:01	AFLTWHRYHLLRLEK	TRP1	P17643	219	233	44.85
4562	28	DRB1*13:01	CDQRVLIVRRNLLDL	TRP1	P17643	122	136	25.37
4563	29	DRB1*13:01	DQRVLIVRRNLLDLS	TRP1	P17643	123	137	23
4564	30	DRB1*13:01	EGPAFLTWHRYHLLR	TRP1	P17643	216	230	44.44
4565	31	DRB1*13:01	FFPLLLFQQARAQFP	TRP1	P17643	13	27	46.29
4566	32	DRB1*13:01	FPLLLFQQARAQFPR	TRP1	P17643	14	28	34.58
4567	33	DRB1*13:01	GPAFLTWHRYHLLRL	TRP1	P17643	217	231	35.26
4568	34	DRB1*13:01	HPLFVIATRRSEEIL	TRP1	P17643	156	170	40.79
4569	35	DRB1*13:01	IFGTASYLIRARRSM	TRP1	P17643	493	507	39.73
4570	36	DRB1*13:01	LIFGTASYLIRARRS	TRP1	P17643	492	506	49.31
4571	37	DRB1*13:01	PAFLTWHRYHLLRLE	TRP1	P17643	218	232	38.5
4572	38	DRB1*13:01	PLLLFQQARAQFPRQ	TRP1	P17643	15	29	42.53
4573	39	DRB1*13:01	QRVLIVRRNLLDLSK	TRP1	P17643	124	138	20.24
4574	40	DRB1*13:01	RVLIVRRNLLDLSKE	TRP1	P17643	125	139	31.53
4575	41	DRB1*13:01	THPLFVIATRRSEEI	TRP1	P17643	155	169	37.93
4576	42	DRB1*13:01	ASLIYRRRLMKQDFS	PMEL17	P40967	612	626	30.9
4577	43	DRB1*13:01	ATLRLVKRQVPLDCV	PMEL17	P40967	462	476	33.5
4578	44	DRB1*13:01	AVVLASLIYRRRLMK	PMEL17	P40967	608	622	16.96
4579	45	DRB1*13:01	DGTATLRLVKRQVPL	PMEL17	P40967	459	473	22.93
4580	46	DRB1*13:01	EVTVYHRRGSRSYVP	PMEL17	P40967	185	199	47.92
4581	47	DRB1*13:01	GNKHFLRNQPLTFAL	PMEL17	P40967	228	242	45.46
4582	48	DRB1*13:01	GTATLRLVKRQVPLD	PMEL17	P40967	460	474	26.58
4583	49	DRB1*13:01	KHFLRNQPLTFALQL	PMEL17	P40967	230	244	45.78
4584	50	DRB1*13:01	LASLIYRRRLMKQDF	PMEL17	P40967	611	625	21.1
4585	51	DRB1*13:01	LDGTATLRLVKRQVP	PMEL17	P40967	458	472	45.35
4586	52	DRB1*13:01	LMAVVLASLIYRRRL	PMEL17	P40967	606	620	26.81
4587	53	DRB1*13:01	MAVVLASLIYRRRLM	PMEL17	P40967	607	621	21.84
4588	54	DRB1*13:01	MEVTVYHRRGSRSYV	PMEL17	P40967	184	198	34.06
4589	55	DRB1*13:01	NKHFLRNQPLTFALQ	PMEL17	P40967	229	243	41.83
4590	56	DRB1*13:01	SLIYRRRLMKQDFSV	PMEL17	P40967	613	627	44.49
4591	57	DRB1*13:01	TATLRLVKRQVPLDC	PMEL17	P40967	461	475	28.64
4592	58	DRB1*13:01	TLRLVKRQVPLDCVL	PMEL17	P40967	463	477	48.63
4593	59	DRB1*13:01	TMEVTVYHRRGSRSY	PMEL17	P40967	183	197	42.25
4594	60	DRB1*13:01	VLASLIYRRRLMKQD	PMEL17	P40967	610	624	18.87
4595	61	DRB1*13:01	VLMAVVLASLIYRRR	PMEL17	P40967	605	619	37.73
4596	62	DRB1*13:01	VTAQVVLQAAIPLTS	PMEL17	P40967	286	300	39.64
4597	63	DRB1*13:01	VVLASLIYRRRLMKQ	PMEL17	P40967	609	623	15.16
4598	64	DRB1*13:01	ADLVGFLLLKYRARE	MAGE1	P43355	107	121	40.53

4599	65	DRB1*13:01	ARVRFFFPSLREAAL	MAGE1	P43355	288	302	48.15
4600	66	DRB1*13:01	DLVGFLLLKYRAREP	MAGE1	P43355	108	122	42.83
4601	67	DRB1*13:01	EYVIKVSARVRFFFP	MAGE1	P43355	281	295	9.36
4602	68	DRB1*13:01	FLLLKYRAREPVTKA	MAGE1	P43355	112	126	31.43
4603	69	DRB1*13:01	GFLLLKYRAREPVTK	MAGE1	P43355	111	125	28.99
4604	70	DRB1*13:01	IKVSARVRFFFPSLR	MAGE1	P43355	284	298	40.89
4605	71	DRB1*13:01	KVLEYVIKVSARVRF	MAGE1	P43355	278	292	12.95
4606	72	DRB1*13:01	LEYVIKVSARVRFFF	MAGE1	P43355	280	294	9.32
4607	73	DRB1*13:01	LVGFLLLKYRAREPV	MAGE1	P43355	109	123	29.64
4608	74	DRB1*13:01	SARVRFFFPSLREAA	MAGE1	P43355	287	301	44.9
4609	75	DRB1*13:01	VGFLLLKYRAREPVT	MAGE1	P43355	110	124	27.92
4610	76	DRB1*13:01	VIKVSARVRFFFPSL	MAGE1	P43355	283	297	21.98
4611	77	DRB1*13:01	VKVLEYVIKVSARVR	MAGE1	P43355	277	291	24.8
4612	78	DRB1*13:01	VLEYVIKVSARVRFF	MAGE1	P43355	279	293	9.97
4613	79	DRB1*13:01	YVIKVSARVRFFFPS	MAGE1	P43355	282	296	12.43
4614	80	DRB1*13:01	AEMLERVIKNYKRCF	MAGE4	P43358	134	148	43.68
4615	81	DRB1*13:01	AHFLLRKYRAKELVT	MAGE4	P43358	118	132	35.84
4616	82	DRB1*13:01	ARVRIAYPSLREAAL	MAGE4	P43358	296	310	40.7
4617	83	DRB1*13:01	EHVVRVNARVRIAYP	MAGE4	P43358	289	303	11.58
4618	84	DRB1*13:01	ELAHFLLRKYRAKEL	MAGE4	P43358	116	130	34.56
4619	85	DRB1*13:01	EMLERVIKNYKRCFP	MAGE4	P43358	135	149	40.71
4620	86	DRB1*13:01	ERVIKNYKRCFPVIF	MAGE4	P43358	138	152	35.86
4621	87	DRB1*13:01	HFLLRKYRAKELVTK	MAGE4	P43358	119	133	38.09
4622	88	DRB1*13:01	HVVRVNARVRIAYPS	MAGE4	P43358	290	304	12.34
4623	89	DRB1*13:01	KVLEHVVRVNARVRI	MAGE4	P43358	286	300	14.8
4624	90	DRB1*13:01	LAHFLLRKYRAKELV	MAGE4	P43358	117	131	31.45
4625	91	DRB1*13:01	LEHVVRVNARVRIAY	MAGE4	P43358	288	302	9.7
4626	92	DRB1*13:01	LERVIKNYKRCFPVI	MAGE4	P43358	137	151	30.05
4627	93	DRB1*13:01	MLERVIKNYKRCFPV	MAGE4	P43358	136	150	32.77
4628	94	DRB1*13:01	NARVRIAYPSLREAA	MAGE4	P43358	295	309	36.41
4629	95	DRB1*13:01	RVNARVRIAYPSLRE	MAGE4	P43358	293	307	29.94
4630	96	DRB1*13:01	VKVLEHVVRVNARVR	MAGE4	P43358	285	299	27.4
4631	97	DRB1*13:01	VLEHVVRVNARVRIA	MAGE4	P43358	287	301	12.31
4632	98	DRB1*13:01	VNARVRIAYPSLREA	MAGE4	P43358	294	308	37.07
4633	99	DRB1*13:01	VRVNARVRIAYPSLR	MAGE4	P43358	292	306	24.23
4634	100	DRB1*13:01	VVRVNARVRIAYPSL	MAGE4	P43358	291	305	16.81
4635	101	DRB1*13:01	ILTIRLTAADHRQLQ	NY-ESO-1	P78358	132	146	42.73
4636	102	DRB1*13:01	NILTIRLTAADHRQL	NY-ESO-1	P78358	131	145	45.24
4637	103	DRB1*13:01	EKIFYVYMKRKYEAM	SSX2	Q16385	44	58	27.64
4638	104	DRB1*13:01	FYVYMKRKYEAMTKL	SSX2	Q16385	47	61	39.09

4639	105	DRB1*13:01	IFYVYMKRKYEAMTK	SSX2	Q16385	46	60	28.77
4640	106	DRB1*13:01	KIFYVYMKRKYEAMT	SSX2	Q16385	45	59	26.59
4641	107	DRB1*13:01	SEKIFYVYMKRKYEA	SSX2	Q16385	43	57	37.1
4642	108	DRB1*13:01	WEKMKASEKIFYVYM	SSX2	Q16385	37	51	45.94
4643	1	DRB1*13:02	KKPPVIRQNIHSLSP	TRP2	O75767	121	135	47.85
4644	2	DRB1*13:02	KPPVIRQNIHSLSPQ	TRP2	O75767	122	136	47.66
4645	3	DRB1*13:02	RKKPPVIRQNIHSLS	TRP2	O75767	120	134	48.91
4646	4	DRB1*13:02	ELKELINNELSHFLE	S100	P04271	30	44	36.11
4647	5	DRB1*13:02	LKELINNELSHFLEE	S100	P04271	31	45	45.36
4648	6	DRB1*13:02	SELKELINNELSHFL	S100	P04271	29	43	34.41
4649	7	DRB1*13:02	ALHIYMNGTMSQVQG	Tyrosinase	P14679	365	379	24.22
4650	8	DRB1*13:02	CQNILLSNAPLGPQF	Tyrosinase	P14679	55	69	28.2
4651	9	DRB1*13:02	HNALHIYMNGTMSQV	Tyrosinase	P14679	363	377	23.28
4652	10	DRB1*13:02	KHTISSDYVIPIGTY	Tyrosinase	P14679	142	156	46.16
4653	11	DRB1*13:02	LHIYMNGTMSQVQGS	Tyrosinase	P14679	366	380	29.98
4654	12	DRB1*13:02	MHNALHIYMNGTMSQ	Tyrosinase	P14679	362	376	39.88
4655	13	DRB1*13:02	NALHIYMNGTMSQVQ	Tyrosinase	P14679	364	378	21.33
4656	14	DRB1*13:02	NILLSNAPLGPQFPF	Tyrosinase	P14679	57	71	47.26
4657	15	DRB1*13:02	QNILLSNAPLGPQFP	Tyrosinase	P14679	56	70	28.71
4658	16	DRB1*13:02	SCQNILLSNAPLGPQ	Tyrosinase	P14679	54	68	41.65
4659	17	DRB1*13:02	DGPIRRNPAGNVARP	TRP1	P17643	308	322	36.78
4660	18	DRB1*13:02	DTPPFYSNSTNSFRN	TRP1	P17643	343	357	25.79
4661	19	DRB1*13:02	EDGPIRRNPAGNVAR	TRP1	P17643	307	321	32.26
4662	20	DRB1*13:02	FDTPPFYSNSTNSFR	TRP1	P17643	342	356	28.28
4663	21	DRB1*13:02	GPIRRNPAGNVARPM	TRP1	P17643	309	323	35.1
4664	22	DRB1*13:02	GQTHLSPNDPIFVLL	TRP1	P17643	389	403	34.38
4665	23	DRB1*13:02	PFYSNSTNSFRNTVE	TRP1	P17643	346	360	39.62
4666	24	DRB1*13:02	PIRRNPAGNVARPMV	TRP1	P17643	310	324	43.15
4667	25	DRB1*13:02	PPFYSNSTNSFRNTV	TRP1	P17643	345	359	31.34
4668	26	DRB1*13:02	QTHLSPNDPIFVLLH	TRP1	P17643	390	404	32.73
4669	27	DRB1*13:02	TEDGPIRRNPAGNVA	TRP1	P17643	306	320	45.36
4670	28	DRB1*13:02	THLSPNDPIFVLLHT	TRP1	P17643	391	405	43.62
4671	29	DRB1*13:02	TPPFYSNSTNSFRNT	TRP1	P17643	344	358	24.09
4672	30	DRB1*13:02	VALIFGTASYLIRAR	TRP1	P17643	490	504	45.96
4673	31	DRB1*13:02	AQVVLQAAIPLTSCG	PMEL17	P40967	288	302	43.39
4674	32	DRB1*13:02	ASFSIALNFPGSQKV	PMEL17	P40967	82	96	34.8
4675	33	DRB1*13:02	AVIGALLAVGATKVP	PMEL17	P40967	13	27	46.91
4676	34	DRB1*13:02	DGGNKHFLRNQPLTF	PMEL17	P40967	226	240	18.26
4677	35	DRB1*13:02	DGPTLIGANASFSIA	PMEL17	P40967	73	87	24.48

4678	36	DRB1*13:02	DGQVIWVNNTIINGS	PMEL17	P40967	99	113	17.49
4679	37	DRB1*13:02	EVSIVVLSGTTAAQV	PMEL17	P40967	404	418	49.09
4680	38	DRB1*13:02	FSIALNFPGSQKVLP	PMEL17	P40967	84	98	37.57
4681	39	DRB1*13:02	GGNKHFLRNQPLTFA	PMEL17	P40967	227	241	11.13
4682	40	DRB1*13:02	GNKHFLRNQPLTFAL	PMEL17	P40967	228	242	8.61
4683	41	DRB1*13:02	GPTLIGANASFSIAL	PMEL17	P40967	74	88	17.01
4684	42	DRB1*13:02	GPVTAQVVLQAAIPL	PMEL17	P40967	284	298	35.55
4685	43	DRB1*13:02	GQVIWVNNTIINGSQ	PMEL17	P40967	100	114	16.48
4686	44	DRB1*13:02	HFLRNQPLTFALQLH	PMEL17	P40967	231	245	17.65
4687	45	DRB1*13:02	IGALLAVGATKVPRN	PMEL17	P40967	15	29	34.24
4688	46	DRB1*13:02	KHFLRNQPLTFALQL	PMEL17	P40967	230	244	9.79
4689	47	DRB1*13:02	LIGANASFSIALNFP	PMEL17	P40967	77	91	36.78
4690	48	DRB1*13:02	LPDGQVIWVNNTIIN	PMEL17	P40967	97	111	23.66
4691	49	DRB1*13:02	NASFSIALNFPGSQK	PMEL17	P40967	81	95	49.03
4692	50	DRB1*13:02	NDGPTLIGANASFSI	PMEL17	P40967	72	86	44.44
4693	51	DRB1*13:02	NKHFLRNQPLTFALQ	PMEL17	P40967	229	243	8.69
4694	52	DRB1*13:02	PDGQVIWVNNTIING	PMEL17	P40967	98	112	19.68
4695	53	DRB1*13:02	PTLIGANASFSIALN	PMEL17	P40967	75	89	17.72
4696	54	DRB1*13:02	PVTAQVVLQAAIPLT	PMEL17	P40967	285	299	24.67
4697	55	DRB1*13:02	QVIWVNNTIINGSQV	PMEL17	P40967	101	115	18.45
4698	56	DRB1*13:02	SFSIALNFPGSQKVL	PMEL17	P40967	83	97	27.92
4699	57	DRB1*13:02	TAQVVLQAAIPLTSC	PMEL17	P40967	287	301	29.07
4700	58	DRB1*13:02	TLIGANASFSIALNF	PMEL17	P40967	76	90	20.23
4701	59	DRB1*13:02	VIGALLAVGATKVPR	PMEL17	P40967	14	28	32.72
4702	60	DRB1*13:02	VIWVNNTIINGSQVW	PMEL17	P40967	102	116	31.07
4703	61	DRB1*13:02	VSIVVLSGTTAAQVT	PMEL17	P40967	405	419	43.21
4704	62	DRB1*13:02	VTAQVVLQAAIPLTS	PMEL17	P40967	286	300	20.55
4705	63	DRB1*13:02	EYVIKVSARVRFFFP	MAGE1	P43355	281	295	26.42
4706	64	DRB1*13:02	KVLEYVIKVSARVRF	MAGE1	P43355	278	292	29.26
4707	65	DRB1*13:02	LEYVIKVSARVRFFF	MAGE1	P43355	280	294	24.27
4708	66	DRB1*13:02	VKVLEYVIKVSARVR	MAGE1	P43355	277	291	46.25
4709	67	DRB1*13:02	VLEYVIKVSARVRFF	MAGE1	P43355	279	293	25.89
4710	68	DRB1*13:02	EHVVRVNARVRIAYP	MAGE4	P43358	289	303	20.84
4711	69	DRB1*13:02	ESLFREALSNKVDEL	MAGE4	P43358	103	117	46.34
4712	70	DRB1*13:02	HVVRVNARVRIAYPS	MAGE4	P43358	290	304	23.63
4713	71	DRB1*13:02	KVLEHVVRVNARVRI	MAGE4	P43358	286	300	26.57
4714	72	DRB1*13:02	LEHVVRVNARVRIAY	MAGE4	P43358	288	302	17.31
4715	73	DRB1*13:02	VLEHVVRVNARVRIA	MAGE4	P43358	287	301	20.72
4716	74	DRB1*13:02	VVRVNARVRIAYPSL	MAGE4	P43358	291	305	32.87
4717	75	DRB1*13:02	DHRQLQLSISSCLQQ	NY-ESO-1	P78358	141	155	42.47

4718	76	DRB1*13:02	HRQLQLSISSCLQQL	NY-ESO-1	P78358	142	156	36.44
4719	77	DRB1*13:02	KEFTVSGNILTIRLT	NY-ESO-1	P78358	124	138	36.33
4720	78	DRB1*13:02	LKEFTVSGNILTIRL	NY-ESO-1	P78358	123	137	28.04
4721	79	DRB1*13:02	LLKEFTVSGNILTIR	NY-ESO-1	P78358	122	136	29.99
4722	80	DRB1*13:02	VLLKEFTVSGNILTI	NY-ESO-1	P78358	121	135	49.15
4723	81	DRB1*13:02	RAVFLALSAQLLQAR	BAGE	Q13072	4	18	48.97
4724	1	DRB1*13:05	EVISCKLIKRATTRQ	TRP2	O75767	222	236	30.92
4725	2	DRB1*13:05	RKFFHRTCKCTGNFA	TRP2	O75767	88	102	39.55
4726	3	DRB1*13:05	VISCKLIKRATTRQP	TRP2	O75767	223	237	33.86
4727	4	DRB1*13:05	WEVISCKLIKRATTR	TRP2	O75767	221	235	36.89
4728	5	DRB1*13:05	YYYRFVIGLRVWQWE	TRP2	O75767	208	222	49.95
4729	6	DRB1*13:05	DKFFAYLTLAKHTIS	Tyrosinase	P14679	132	146	26.01
4730	7	DRB1*13:05	FAYLTLAKHTISSDY	Tyrosinase	P14679	135	149	35.24
4731	8	DRB1*13:05	FFAYLTLAKHTISSD	Tyrosinase	P14679	134	148	28.57
4732	9	DRB1*13:05	KDKFFAYLTLAKHTI	Tyrosinase	P14679	131	145	33.33
4733	10	DRB1*13:05	KFFAYLTLAKHTISS	Tyrosinase	P14679	133	147	22.47
4734	11	DRB1*13:05	MVPFIPLYRNGDFFI	Tyrosinase	P14679	426	440	32.73
4735	12	DRB1*13:05	VPFIPLYRNGDFFIS	Tyrosinase	P14679	427	441	41.52
4736	13	DRB1*13:05	YMVPFIPLYRNGDFF	Tyrosinase	P14679	425	439	42.2
4737	14	DRB1*13:05	ASYLIRARRSMDEAN	TRP1	P17643	497	511	46.19
4738	15	DRB1*13:05	FVWTHYYSVKKTFLG	TRP1	P17643	188	202	47.59
4739	16	DRB1*13:05	HPLFVIATRRSEEIL	TRP1	P17643	156	170	13.74
4740	17	DRB1*13:05	LFVIATRRSEEILGP	TRP1	P17643	158	172	24.07
4741	18	DRB1*13:05	PLFVIATRRSEEILG	TRP1	P17643	157	171	15.42
4742	19	DRB1*13:05	RTTHPLFVIATRRSE	TRP1	P17643	153	167	24.44
4743	20	DRB1*13:05	TASYLIRARRSMDEA	TRP1	P17643	496	510	39.65
4744	21	DRB1*13:05	THPLFVIATRRSEEI	TRP1	P17643	155	169	15.19
4745	22	DRB1*13:05	THYYSVKKTFLGVGQ	TRP1	P17643	191	205	37.63
4746	23	DRB1*13:05	TTHPLFVIATRRSEE	TRP1	P17643	154	168	19.39
4747	24	DRB1*13:05	VWTHYYSVKKTFLGV	TRP1	P17643	189	203	31.01
4748	25	DRB1*13:05	WTHYYSVKKTFLGVG	TRP1	P17643	190	204	32.76
4749	26	DRB1*13:05	ANASFSIALNFPGSQ	PMEL17	P40967	80	94	49.44
4750	27	DRB1*13:05	ASFSIALNFPGSQKV	PMEL17	P40967	82	96	42.13
4751	28	DRB1*13:05	ATLRLVKRQVPLDCV	PMEL17	P40967	462	476	32.58
4752	29	DRB1*13:05	CQLVLHQILKGGSGT	PMEL17	P40967	550	564	45.41
4753	30	DRB1*13:05	DGTATLRLVKRQVPL	PMEL17	P40967	459	473	18.27
4754	31	DRB1*13:05	GSRSYVPLAHSSSAF	PMEL17	P40967	193	207	39.7
4755	32	DRB1*13:05	GTATLRLVKRQVPLD	PMEL17	P40967	460	474	20.16
4756	33	DRB1*13:05	KRSFVYVWKTWGQYW	PMEL17	P40967	146	160	17.94

4757	34	DRB1*13:05	NASFSIALNFPGSQK	PMEL17	P40967	81	95	33.06
4758	35	DRB1*13:05	QKRSFVYVWKTWGQY	PMEL17	P40967	145	159	18.3
4759	36	DRB1*13:05	QLVLHQILKGGSGTY	PMEL17	P40967	551	565	36.77
4760	37	DRB1*13:05	RSFVYVWKTWGQYWQ	PMEL17	P40967	147	161	30.25
4761	38	DRB1*13:05	RSYVPLAHSSSAFTI	PMEL17	P40967	195	209	42.32
4762	39	DRB1*13:05	SQKRSFVYVWKTWGQ	PMEL17	P40967	144	158	21.74
4763	40	DRB1*13:05	SRSYVPLAHSSSAFT	PMEL17	P40967	194	208	39.52
4764	41	DRB1*13:05	TATLRLVKRQVPLDC	PMEL17	P40967	461	475	23.06
4765	42	DRB1*13:05	WSQKRSFVYVWKTWG	PMEL17	P40967	143	157	41.25
4766	43	DRB1*13:05	ADLVGFLLLKYRARE	MAGE1	P43355	107	121	34.07
4767	44	DRB1*13:05	ARVRFFFPSLREAAL	MAGE1	P43355	288	302	21.89
4768	45	DRB1*13:05	ARYEFLWGPRALAET	MAGE1	P43355	260	274	33.84
4769	46	DRB1*13:05	DLVGFLLLKYRAREP	MAGE1	P43355	108	122	28.93
4770	47	DRB1*13:05	ESLFRAVITKKVADL	MAGE1	P43355	95	109	29.13
4771	48	DRB1*13:05	EYVIKVSARVRFFFP	MAGE1	P43355	281	295	39.57
4772	49	DRB1*13:05	FFFPSLREAALREEE	MAGE1	P43355	292	306	45.29
4773	50	DRB1*13:05	GFLLLKYRAREPVTK	MAGE1	P43355	111	125	45.81
4774	51	DRB1*13:05	ILESLFRAVITKKVA	MAGE1	P43355	93	107	32.17
4775	52	DRB1*13:05	KVLEYVIKVSARVRF	MAGE1	P43355	278	292	25.6
4776	53	DRB1*13:05	LESLFRAVITKKVAD	MAGE1	P43355	94	108	37.28
4777	54	DRB1*13:05	LEYVIKVSARVRFFF	MAGE1	P43355	280	294	34.02
4778	55	DRB1*13:05	LVGFLLLKYRAREPV	MAGE1	P43355	109	123	26.38
4779	56	DRB1*13:05	PARYEFLWGPRALAE	MAGE1	P43355	259	273	37.79
4780	57	DRB1*13:05	RFFFPSLREAALREE	MAGE1	P43355	291	305	18.8
4781	58	DRB1*13:05	RVRFFFPSLREAALR	MAGE1	P43355	289	303	14
4782	59	DRB1*13:05	RYEFLWGPRALAETS	MAGE1	P43355	261	275	34.81
4783	60	DRB1*13:05	SARVRFFFPSLREAA	MAGE1	P43355	287	301	49.09
4784	61	DRB1*13:05	SLFRAVITKKVADLV	MAGE1	P43355	96	110	32.18
4785	62	DRB1*13:05	VGFLLLKYRAREPVT	MAGE1	P43355	110	124	31.71
4786	63	DRB1*13:05	VKVLEYVIKVSARVR	MAGE1	P43355	277	291	29.38
4787	64	DRB1*13:05	VLEYVIKVSARVRFF	MAGE1	P43355	279	293	31.77
4788	65	DRB1*13:05	VRFFFPSLREAALRE	MAGE1	P43355	290	304	14.6
4789	66	DRB1*13:05	AHFLLRKYRAKELVT	MAGE4	P43358	118	132	42.25
4790	67	DRB1*13:05	ARYEFLWGPRALAET	MAGE4	P43358	268	282	33.84
4791	68	DRB1*13:05	DELAHFLLRKYRAKE	MAGE4	P43358	115	129	39.02
4792	69	DRB1*13:05	ELAHFLLRKYRAKEL	MAGE4	P43358	116	130	28.81
4793	70	DRB1*13:05	KVLEHVVRVNARVRI	MAGE4	P43358	286	300	31.5
4794	71	DRB1*13:05	LAHFLLRKYRAKELV	MAGE4	P43358	117	131	29.7
4795	72	DRB1*13:05	PARYEFLWGPRALAE	MAGE4	P43358	267	281	37.79
4796	73	DRB1*13:05	RYEFLWGPRALAETS	MAGE4	P43358	269	283	34.81

4797	74	DRB1*13:05	SYVKVLEHVVRVNAR	MAGE4	P43358	283	297	47.81
4798	75	DRB1*13:05	VDELAHFLLRKYRAK	MAGE4	P43358	114	128	43.08
4799	76	DRB1*13:05	VKVLEHVVRVNARVR	MAGE4	P43358	285	299	30.17
4800	77	DRB1*13:05	YVKVLEHVVRVNARV	MAGE4	P43358	284	298	40.41
4801	78	DRB1*13:05	RAVFLALSAQLLQAR	BAGE	Q13072	4	18	49.7
4802	79	DRB1*13:05	ASEKIFYVYMKRKYE	SSX2	Q16385	42	56	21.98
4803	80	DRB1*13:05	EKIFYVYMKRKYEAM	SSX2	Q16385	44	58	12.58
4804	81	DRB1*13:05	FYVYMKRKYEAMTKL	SSX2	Q16385	47	61	19.78
4805	82	DRB1*13:05	IFYVYMKRKYEAMTK	SSX2	Q16385	46	60	13.87
4806	83	DRB1*13:05	KIFYVYMKRKYEAMT	SSX2	Q16385	45	59	12.77
4807	84	DRB1*13:05	KRKYEAMTKLGFKAT	SSX2	Q16385	52	66	47.42
4808	85	DRB1*13:05	MKRKYEAMTKLGFKA	SSX2	Q16385	51	65	47.51
4809	86	DRB1*13:05	SEKIFYVYMKRKYEA	SSX2	Q16385	43	57	15.39
4810	1	DRB1*13:07	KFFAYLTLAKHTISS	Tyrosinase	P14679	133	147	45.92
4811	2	DRB1*13:07	HPLFVIATRRSEEIL	TRP1	P17643	156	170	29.24
4812	3	DRB1*13:07	PLFVIATRRSEEILG	TRP1	P17643	157	171	34.19
4813	4	DRB1*13:07	THPLFVIATRRSEEI	TRP1	P17643	155	169	33.39
4814	5	DRB1*13:07	TTHPLFVIATRRSEE	TRP1	P17643	154	168	45.06
4815	6	DRB1*13:07	KRSFVYVWKTWGQYW	PMEL17	P40967	146	160	39.23
4816	7	DRB1*13:07	QKRSFVYVWKTWGQY	PMEL17	P40967	145	159	39.89
4817	8	DRB1*13:07	SQKRSFVYVWKTWGQ	PMEL17	P40967	144	158	49.54
4818	9	DRB1*13:07	ARVRFFFPSLREAAL	MAGE1	P43355	288	302	35.21
4819	10	DRB1*13:07	RFFFPSLREAALREE	MAGE1	P43355	291	305	31.49
4820	11	DRB1*13:07	RVRFFFPSLREAALR	MAGE1	P43355	289	303	22.26
4821	12	DRB1*13:07	VRFFFPSLREAALRE	MAGE1	P43355	290	304	23.67
4822	13	DRB1*13:07	EKIFYVYMKRKYEAM	SSX2	Q16385	44	58	28.09
4823	14	DRB1*13:07	IFYVYMKRKYEAMTK	SSX2	Q16385	46	60	31.32
4824	15	DRB1*13:07	KIFYVYMKRKYEAMT	SSX2	Q16385	45	59	27.34
4825	16	DRB1*13:07	SEKIFYVYMKRKYEA	SSX2	Q16385	43	57	37.23
4826	1	DRB1*13:11	EVISCKLIKRATTRQ	TRP2	O75767	222	236	19.29
4827	2	DRB1*13:11	FLGALDLAKKRVHPD	TRP2	O75767	141	155	39.69
4828	3	DRB1*13:11	LGALDLAKKRVHPDY	TRP2	O75767	142	156	30.57
4829	4	DRB1*13:11	QFLGALDLAKKRVHP	TRP2	O75767	140	154	35.1
4830	5	DRB1*13:11	QWEVISCKLIKRATT	TRP2	O75767	220	234	36.31
4831	6	DRB1*13:11	VISCKLIKRATTRQP	TRP2	O75767	223	237	20.64
4832	7	DRB1*13:11	WEVISCKLIKRATTR	TRP2	O75767	221	235	22.06
4833	8	DRB1*13:11	AGLVSLLCRHKRKQL	Tyrosinase	P14679	493	507	20.85
4834	9	DRB1*13:11	ALLAGLVSLLCRHKR	Tyrosinase	P14679	490	504	44.71

4835	10	DRB1*13:11	DKFFAYLTLAKHTIS	Tyrosinase	P14679	132	146	46.72
4836	11	DRB1*13:11	FAYLTLAKHTISSDY	Tyrosinase	P14679	135	149	27.46
4837	12	DRB1*13:11	FFAYLTLAKHTISSD	Tyrosinase	P14679	134	148	28.42
4838	13	DRB1*13:11	GLVSLLCRHKRKQLP	Tyrosinase	P14679	494	508	26.41
4839	14	DRB1*13:11	IKSYLEQASRIWSWL	Tyrosinase	P14679	464	478	46.23
4840	15	DRB1*13:11	KFFAYLTLAKHTISS	Tyrosinase	P14679	133	147	27.61
4841	16	DRB1*13:11	KSYLEQASRIWSWLL	Tyrosinase	P14679	465	479	48.45
4842	17	DRB1*13:11	LAGLVSLLCRHKRKQ	Tyrosinase	P14679	492	506	24.86
4843	18	DRB1*13:11	LLAGLVSLLCRHKRK	Tyrosinase	P14679	491	505	27.66
4844	19	DRB1*13:11	LVSLLCRHKRKQLPE	Tyrosinase	P14679	495	509	40.74
4845	20	DRB1*13:11	AACDQRVLIVRRNLL	TRP1	P17643	120	134	29.49
4846	21	DRB1*13:11	ACDQRVLIVRRNLLD	TRP1	P17643	121	135	24.43
4847	22	DRB1*13:11	ASYLIRARRSMDEAN	TRP1	P17643	497	511	28.67
4848	23	DRB1*13:11	CDQRVLIVRRNLLDL	TRP1	P17643	122	136	20.29
4849	24	DRB1*13:11	DQRVLIVRRNLLDLS	TRP1	P17643	123	137	19.07
4850	25	DRB1*13:11	FFPLLLFQQARAQFP	TRP1	P17643	13	27	32.64
4851	26	DRB1*13:11	FGTASYLIRARRSMD	TRP1	P17643	494	508	30.25
4852	27	DRB1*13:11	FPLLLFQQARAQFPR	TRP1	P17643	14	28	26.77
4853	28	DRB1*13:11	FVRALDMAKRTTHPL	TRP1	P17643	144	158	38.95
4854	29	DRB1*13:11	GTASYLIRARRSMDE	TRP1	P17643	495	509	29.26
4855	30	DRB1*13:11	HPLFVIATRRSEEIL	TRP1	P17643	156	170	12.52
4856	31	DRB1*13:11	IFFPLLLFQQARAQF	TRP1	P17643	12	26	47.75
4857	32	DRB1*13:11	IFGTASYLIRARRSM	TRP1	P17643	493	507	30.93
4858	33	DRB1*13:11	KRTTHPLFVIATRRS	TRP1	P17643	152	166	42.57
4859	34	DRB1*13:11	LFVIATRRSEEILGP	TRP1	P17643	158	172	37.31
4860	35	DRB1*13:11	LIFGTASYLIRARRS	TRP1	P17643	492	506	43.28
4861	36	DRB1*13:11	PLFVIATRRSEEILG	TRP1	P17643	157	171	16.46
4862	37	DRB1*13:11	PLLLFQQARAQFPRQ	TRP1	P17643	15	29	38.91
4863	38	DRB1*13:11	QRVLIVRRNLLDLSK	TRP1	P17643	124	138	17.56
4864	39	DRB1*13:11	RTTHPLFVIATRRSE	TRP1	P17643	153	167	17.16
4865	40	DRB1*13:11	RVLIVRRNLLDLSKE	TRP1	P17643	125	139	27.27
4866	41	DRB1*13:11	SYLIRARRSMDEANQ	TRP1	P17643	498	512	39.58
4867	42	DRB1*13:11	TASYLIRARRSMDEA	TRP1	P17643	496	510	23.81
4868	43	DRB1*13:11	THPLFVIATRRSEEI	TRP1	P17643	155	169	12.59
4869	44	DRB1*13:11	TTHPLFVIATRRSEE	TRP1	P17643	154	168	15.45
4870	45	DRB1*13:11	VRALDMAKRTTHPLF	TRP1	P17643	145	159	31.74
4871	46	DRB1*13:11	ACQLVLHQILKGGSG	PMEL17	P40967	549	563	20.08
4872	47	DRB1*13:11	ASLIYRRRLMKQDFS	PMEL17	P40967	612	626	36.01
4873	48	DRB1*13:11	ATLRLVKRQVPLDCV	PMEL17	P40967	462	476	15.25
4874	49	DRB1*13:11	AVVLASLIYRRRLMK	PMEL17	P40967	608	622	20.89

4875	50	DRB1*13:11	CQLVLHQILKGGSGT	PMEL17	P40967	550	564	17.75
4876	51	DRB1*13:11	DGTATLRLVKRQVPL	PMEL17	P40967	459	473	9.31
4877	52	DRB1*13:11	DWLGVSRQLRTKAWN	PMEL17	P40967	31	45	44.48
4878	53	DRB1*13:11	GTATLRLVKRQVPLD	PMEL17	P40967	460	474	10.14
4879	54	DRB1*13:11	KRSFVYVWKTWGQYW	PMEL17	P40967	146	160	37.07
4880	55	DRB1*13:11	LASLIYRRRLMKQDF	PMEL17	P40967	611	625	26.62
4881	56	DRB1*13:11	LDGTATLRLVKRQVP	PMEL17	P40967	458	472	22.43
4882	57	DRB1*13:11	LGVSRQLRTKAWNRQ	PMEL17	P40967	33	47	48.19
4883	58	DRB1*13:11	LMAVVLASLIYRRRL	PMEL17	P40967	606	620	37.88
4884	59	DRB1*13:11	LVLHQILKGGSGTYC	PMEL17	P40967	552	566	20.38
4885	60	DRB1*13:11	MAVVLASLIYRRRLM	PMEL17	P40967	607	621	30.33
4886	61	DRB1*13:11	PACQLVLHQILKGGS	PMEL17	P40967	548	562	36.62
4887	62	DRB1*13:11	QDWLGVSRQLRTKAW	PMEL17	P40967	30	44	43.91
4888	63	DRB1*13:11	QKRSFVYVWKTWGQY	PMEL17	P40967	145	159	37.12
4889	64	DRB1*13:11	QLVLHQILKGGSGTY	PMEL17	P40967	551	565	15.12
4890	65	DRB1*13:11	SQKRSFVYVWKTWGQ	PMEL17	P40967	144	158	46.19
4891	66	DRB1*13:11	TATLRLVKRQVPLDC	PMEL17	P40967	461	475	11.26
4892	67	DRB1*13:11	TLRLVKRQVPLDCVL	PMEL17	P40967	463	477	31.83
4893	68	DRB1*13:11	VLASLIYRRRLMKQD	PMEL17	P40967	610	624	22.87
4894	69	DRB1*13:11	VLHQILKGGSGTYCL	PMEL17	P40967	553	567	49.95
4895	70	DRB1*13:11	VVLASLIYRRRLMKQ	PMEL17	P40967	609	623	19.21
4896	71	DRB1*13:11	WLGVSRQLRTKAWNR	PMEL17	P40967	32	46	35.36
4897	72	DRB1*13:11	ADLVGFLLLKYRARE	MAGE1	P43355	107	121	22.28
4898	73	DRB1*13:11	ARVRFFFPSLREAAL	MAGE1	P43355	288	302	42.61
4899	74	DRB1*13:11	CILESLFRAVITKKV	MAGE1	P43355	92	106	49.41
4900	75	DRB1*13:11	DLVGFLLLKYRAREP	MAGE1	P43355	108	122	17.99
4901	76	DRB1*13:11	ESLFRAVITKKVADL	MAGE1	P43355	95	109	44.48
4902	77	DRB1*13:11	EYVIKVSARVRFFFP	MAGE1	P43355	281	295	17.68
4903	78	DRB1*13:11	FLLLKYRAREPVTKA	MAGE1	P43355	112	126	33.91
4904	79	DRB1*13:11	GFLLLKYRAREPVTK	MAGE1	P43355	111	125	25.28
4905	80	DRB1*13:11	ILESLFRAVITKKVA	MAGE1	P43355	93	107	36.18
4906	81	DRB1*13:11	KVLEYVIKVSARVRF	MAGE1	P43355	278	292	14.12
4907	82	DRB1*13:11	LESLFRAVITKKVAD	MAGE1	P43355	94	108	49.59
4908	83	DRB1*13:11	LEYVIKVSARVRFFF	MAGE1	P43355	280	294	17.21
4909	84	DRB1*13:11	LVGFLLLKYRAREPV	MAGE1	P43355	109	123	17.19
4910	85	DRB1*13:11	RVRFFFPSLREAALR	MAGE1	P43355	289	303	32.94
4911	86	DRB1*13:11	SLFRAVITKKVADLV	MAGE1	P43355	96	110	44.25
4912	87	DRB1*13:11	VADLVGFLLLKYRAR	MAGE1	P43355	106	120	36.58
4913	88	DRB1*13:11	VGFLLLKYRAREPVT	MAGE1	P43355	110	124	18.8
4914	89	DRB1*13:11	VIKVSARVRFFFPSL	MAGE1	P43355	283	297	46.43

4915	90	DRB1*13:11	VKVLEYVIKVSARVR	MAGE1	P43355	277	291	21.36
4916	91	DRB1*13:11	VLEYVIKVSARVRFF	MAGE1	P43355	279	293	16.18
4917	92	DRB1*13:11	VRFFFPSLREAALRE	MAGE1	P43355	290	304	38.38
4918	93	DRB1*13:11	YVIKVSARVRFFFPS	MAGE1	P43355	282	296	23.47
4919	94	DRB1*13:11	AEMLERVIKNYKRCF	MAGE4	P43358	134	148	47.43
4920	95	DRB1*13:11	AHFLLRKYRAKELVT	MAGE4	P43358	118	132	26.1
4921	96	DRB1*13:11	DELAHFLLRKYRAKE	MAGE4	P43358	115	129	25.68
4922	97	DRB1*13:11	EHVVRVNARVRIAYP	MAGE4	P43358	289	303	35.82
4923	98	DRB1*13:11	ELAHFLLRKYRAKEL	MAGE4	P43358	116	130	19.95
4924	99	DRB1*13:11	HFLLRKYRAKELVTK	MAGE4	P43358	119	133	38.75
4925	100	DRB1*13:11	HVVRVNARVRIAYPS	MAGE4	P43358	290	304	39.92
4926	101	DRB1*13:11	KVDELAHFLLRKYRA	MAGE4	P43358	113	127	45.38
4927	102	DRB1*13:11	KVLEHVVRVNARVRI	MAGE4	P43358	286	300	14.3
4928	103	DRB1*13:11	LAHFLLRKYRAKELV	MAGE4	P43358	117	131	21.15
4929	104	DRB1*13:11	LEHVVRVNARVRIAY	MAGE4	P43358	288	302	26.61
4930	105	DRB1*13:11	SYVKVLEHVVRVNAR	MAGE4	P43358	283	297	21.83
4931	106	DRB1*13:11	TKAEMLERVIKNYKR	MAGE4	P43358	132	146	48.98
4932	107	DRB1*13:11	TSYVKVLEHVVRVNA	MAGE4	P43358	282	296	45.07
4933	108	DRB1*13:11	VDELAHFLLRKYRAK	MAGE4	P43358	114	128	28.08
4934	109	DRB1*13:11	VKVLEHVVRVNARVR	MAGE4	P43358	285	299	13.92
4935	110	DRB1*13:11	VLEHVVRVNARVRIA	MAGE4	P43358	287	301	20.88
4936	111	DRB1*13:11	YVKVLEHVVRVNARV	MAGE4	P43358	284	298	19.77
4937	112	DRB1*13:11	SGNILTIRLTAADHR	NY-ESO-1	P78358	129	143	45.87
4938	113	DRB1*13:11	ASEKIFYVYMKRKYE	SSX2	Q16385	42	56	28.14
4939	114	DRB1*13:11	EKIFYVYMKRKYEAM	SSX2	Q16385	44	58	12.34
4940	115	DRB1*13:11	FYVYMKRKYEAMTKL	SSX2	Q16385	47	61	16.24
4941	116	DRB1*13:11	IFYVYMKRKYEAMTK	SSX2	Q16385	46	60	11.71
4942	117	DRB1*13:11	KIFYVYMKRKYEAMT	SSX2	Q16385	45	59	11.33
4943	118	DRB1*13:11	SEKIFYVYMKRKYEA	SSX2	Q16385	43	57	16.45
4944	119	DRB1*13:11	YVYMKRKYEAMTKLG	SSX2	Q16385	48	62	35.29
4945	1	DRB1*13:14	EVISCKLIKRATTRQ	TRP2	O75767	222	236	30.92
4946	2	DRB1*13:14	RKFFHRTCKCTGNFA	TRP2	O75767	88	102	39.55
4947	3	DRB1*13:14	VISCKLIKRATTRQP	TRP2	O75767	223	237	33.86
4948	4	DRB1*13:14	WEVISCKLIKRATTR	TRP2	O75767	221	235	36.89
4949	5	DRB1*13:14	YYYRFVIGLRVWQWE	TRP2	O75767	208	222	49.95
4950	6	DRB1*13:14	DKFFAYLTLAKHTIS	Tyrosinase	P14679	132	146	26.01
4951	7	DRB1*13:14	FAYLTLAKHTISSDY	Tyrosinase	P14679	135	149	35.24
4952	8	DRB1*13:14	FFAYLTLAKHTISSD	Tyrosinase	P14679	134	148	28.57
4953	9	DRB1*13:14	KDKFFAYLTLAKHTI	Tyrosinase	P14679	131	145	33.33

4954	10	DRB1*13:14	KFFAYLTLAKHTISS	Tyrosinase	P14679	133	147	22.47
4955	11	DRB1*13:14	MVPFIPLYRNGDFFI	Tyrosinase	P14679	426	440	32.73
4956	12	DRB1*13:14	VPFIPLYRNGDFFIS	Tyrosinase	P14679	427	441	41.52
4957	13	DRB1*13:14	YMVPFIPLYRNGDFF	Tyrosinase	P14679	425	439	42.2
4958	14	DRB1*13:14	ASYLIRARRSMDEAN	TRP1	P17643	497	511	46.19
4959	15	DRB1*13:14	FVWTHYYSVKKTFLG	TRP1	P17643	188	202	47.59
4960	16	DRB1*13:14	HPLFVIATRRSEEIL	TRP1	P17643	156	170	13.74
4961	17	DRB1*13:14	LFVIATRRSEEILGP	TRP1	P17643	158	172	24.07
4962	18	DRB1*13:14	PLFVIATRRSEEILG	TRP1	P17643	157	171	15.42
4963	19	DRB1*13:14	RTTHPLFVIATRRSE	TRP1	P17643	153	167	24.44
4964	20	DRB1*13:14	TASYLIRARRSMDEA	TRP1	P17643	496	510	39.65
4965	21	DRB1*13:14	THPLFVIATRRSEEI	TRP1	P17643	155	169	15.19
4966	22	DRB1*13:14	THYYSVKKTFLGVGQ	TRP1	P17643	191	205	37.63
4967	23	DRB1*13:14	TTHPLFVIATRRSEE	TRP1	P17643	154	168	19.39
4968	24	DRB1*13:14	VWTHYYSVKKTFLGV	TRP1	P17643	189	203	31.01
4969	25	DRB1*13:14	WTHYYSVKKTFLGVG	TRP1	P17643	190	204	32.76
4970	26	DRB1*13:14	ANASFSIALNFPGSQ	PMEL17	P40967	80	94	49.44
4971	27	DRB1*13:14	ASFSIALNFPGSQKV	PMEL17	P40967	82	96	42.13
4972	28	DRB1*13:14	ATLRLVKRQVPLDCV	PMEL17	P40967	462	476	32.58
4973	29	DRB1*13:14	CQLVLHQILKGGSGT	PMEL17	P40967	550	564	45.41
4974	30	DRB1*13:14	DGTATLRLVKRQVPL	PMEL17	P40967	459	473	18.27
4975	31	DRB1*13:14	GSRSYVPLAHSSSAF	PMEL17	P40967	193	207	39.7
4976	32	DRB1*13:14	GTATLRLVKRQVPLD	PMEL17	P40967	460	474	20.16
4977	33	DRB1*13:14	KRSFVYVWKTWGQYW	PMEL17	P40967	146	160	17.94
4978	34	DRB1*13:14	NASFSIALNFPGSQK	PMEL17	P40967	81	95	33.06
4979	35	DRB1*13:14	QKRSFVYVWKTWGQY	PMEL17	P40967	145	159	18.3
4980	36	DRB1*13:14	QLVLHQILKGGSGTY	PMEL17	P40967	551	565	36.77
4981	37	DRB1*13:14	RSFVYVWKTWGQYWQ	PMEL17	P40967	147	161	30.25
4982	38	DRB1*13:14	RSYVPLAHSSSAFTI	PMEL17	P40967	195	209	42.32
4983	39	DRB1*13:14	SQKRSFVYVWKTWGQ	PMEL17	P40967	144	158	21.74
4984	40	DRB1*13:14	SRSYVPLAHSSSAFT	PMEL17	P40967	194	208	39.52
4985	41	DRB1*13:14	TATLRLVKRQVPLDC	PMEL17	P40967	461	475	23.06
4986	42	DRB1*13:14	WSQKRSFVYVWKTWG	PMEL17	P40967	143	157	41.25
4987	43	DRB1*13:14	ADLVGFLLLKYRARE	MAGE1	P43355	107	121	34.07
4988	44	DRB1*13:14	ARVRFFFPSLREAAL	MAGE1	P43355	288	302	21.89
4989	45	DRB1*13:14	ARYEFLWGPRALAET	MAGE1	P43355	260	274	33.84
4990	46	DRB1*13:14	DLVGFLLLKYRAREP	MAGE1	P43355	108	122	28.93
4991	47	DRB1*13:14	ESLFRAVITKKVADL	MAGE1	P43355	95	109	29.13
4992	48	DRB1*13:14	EYVIKVSARVRFFFP	MAGE1	P43355	281	295	39.57
4993	49	DRB1*13:14	FFFPSLREAALREEE	MAGE1	P43355	292	306	45.29

4994	50	DRB1*13:14	GFLLLKYRAREPVTK	MAGE1	P43355	111	125	45.81
4995	51	DRB1*13:14	ILESLFRAVITKKVA	MAGE1	P43355	93	107	32.17
4996	52	DRB1*13:14	KVLEYVIKVSARVRF	MAGE1	P43355	278	292	25.6
4997	53	DRB1*13:14	LESLFRAVITKKVAD	MAGE1	P43355	94	108	37.28
4998	54	DRB1*13:14	LEYVIKVSARVRFFF	MAGE1	P43355	280	294	34.02
4999	55	DRB1*13:14	LVGFLLLKYRAREPV	MAGE1	P43355	109	123	26.38
5000	56	DRB1*13:14	PARYEFLWGPRALAE	MAGE1	P43355	259	273	37.79
5001	57	DRB1*13:14	RFFFPSLREAALREE	MAGE1	P43355	291	305	18.8
5002	58	DRB1*13:14	RVRFFFPSLREAALR	MAGE1	P43355	289	303	14
5003	59	DRB1*13:14	RYEFLWGPRALAETS	MAGE1	P43355	261	275	34.81
5004	60	DRB1*13:14	SARVRFFFPSLREAA	MAGE1	P43355	287	301	49.09
5005	61	DRB1*13:14	SLFRAVITKKVADLV	MAGE1	P43355	96	110	32.18
5006	62	DRB1*13:14	VGFLLLKYRAREPVT	MAGE1	P43355	110	124	31.71
5007	63	DRB1*13:14	VKVLEYVIKVSARVR	MAGE1	P43355	277	291	29.38
5008	64	DRB1*13:14	VLEYVIKVSARVRFF	MAGE1	P43355	279	293	31.77
5009	65	DRB1*13:14	VRFFFPSLREAALRE	MAGE1	P43355	290	304	14.6
5010	66	DRB1*13:14	AHFLLRKYRAKELVT	MAGE4	P43358	118	132	42.25
5011	67	DRB1*13:14	ARYEFLWGPRALAET	MAGE4	P43358	268	282	33.84
5012	68	DRB1*13:14	DELAHFLLRKYRAKE	MAGE4	P43358	115	129	39.02
5013	69	DRB1*13:14	ELAHFLLRKYRAKEL	MAGE4	P43358	116	130	28.81
5014	70	DRB1*13:14	KVLEHVVRVNARVRI	MAGE4	P43358	286	300	31.5
5015	71	DRB1*13:14	LAHFLLRKYRAKELV	MAGE4	P43358	117	131	29.7
5016	72	DRB1*13:14	PARYEFLWGPRALAE	MAGE4	P43358	267	281	37.79
5017	73	DRB1*13:14	RYEFLWGPRALAETS	MAGE4	P43358	269	283	34.81
5018	74	DRB1*13:14	SYVKVLEHVVRVNAR	MAGE4	P43358	283	297	47.81
5019	75	DRB1*13:14	VDELAHFLLRKYRAK	MAGE4	P43358	114	128	43.08
5020	76	DRB1*13:14	VKVLEHVVRVNARVR	MAGE4	P43358	285	299	30.17
5021	77	DRB1*13:14	YVKVLEHVVRVNARV	MAGE4	P43358	284	298	40.41
5022	78	DRB1*13:14	RAVFLALSAQLLQAR	BAGE	Q13072	4	18	49.7
5023	79	DRB1*13:14	ASEKIFYVYMKRKYE	SSX2	Q16385	42	56	21.98
5024	80	DRB1*13:14	EKIFYVYMKRKYEAM	SSX2	Q16385	44	58	12.58
5025	81	DRB1*13:14	FYVYMKRKYEAMTKL	SSX2	Q16385	47	61	19.78
5026	82	DRB1*13:14	IFYVYMKRKYEAMTK	SSX2	Q16385	46	60	13.87
5027	83	DRB1*13:14	KIFYVYMKRKYEAMT	SSX2	Q16385	45	59	12.77
5028	84	DRB1*13:14	KRKYEAMTKLGFKAT	SSX2	Q16385	52	66	47.42
5029	85	DRB1*13:14	MKRKYEAMTKLGFKA	SSX2	Q16385	51	65	47.51
5030	86	DRB1*13:14	SEKIFYVYMKRKYEA	SSX2	Q16385	43	57	15.39
5031	1	DRB1*13:21	DKFFAYLTLAKHTIS	Tyrosinase	P14679	132	146	24.12
5032	2	DRB1*13:21	EKDKFFAYLTLAKHT	Tyrosinase	P14679	130	144	39.49

5033	3	DRB1*13:21	ESYMVPFIPLYRNGD	Tyrosinase	P14679	423	437	32.19
5034	4	DRB1*13:21	FAYLTLAKHTISSDY	Tyrosinase	P14679	135	149	38.61
5035	5	DRB1*13:21	FFAYLTLAKHTISSD	Tyrosinase	P14679	134	148	28.46
5036	6	DRB1*13:21	KDKFFAYLTLAKHTI	Tyrosinase	P14679	131	145	25.02
5037	7	DRB1*13:21	KFFAYLTLAKHTISS	Tyrosinase	P14679	133	147	23.4
5038	8	DRB1*13:21	MVPFIPLYRNGDFFI	Tyrosinase	P14679	426	440	14.15
5039	9	DRB1*13:21	PFIPLYRNGDFFISS	Tyrosinase	P14679	428	442	19.53
5040	10	DRB1*13:21	SYMVPFIPLYRNGDF	Tyrosinase	P14679	424	438	20.22
5041	11	DRB1*13:21	VPFIPLYRNGDFFIS	Tyrosinase	P14679	427	441	15.28
5042	12	DRB1*13:21	YMVPFIPLYRNGDFF	Tyrosinase	P14679	425	439	16.43
5043	13	DRB1*13:21	AACDQRVLIVRRNLL	TRP1	P17643	120	134	49.48
5044	14	DRB1*13:21	ACDQRVLIVRRNLLD	TRP1	P17643	121	135	43.08
5045	15	DRB1*13:21	ASYLIRARRSMDEAN	TRP1	P17643	497	511	22.9
5046	16	DRB1*13:21	CDQRVLIVRRNLLDL	TRP1	P17643	122	136	35.86
5047	17	DRB1*13:21	DQRVLIVRRNLLDLS	TRP1	P17643	123	137	34.19
5048	18	DRB1*13:21	FGTASYLIRARRSMD	TRP1	P17643	494	508	31.19
5049	19	DRB1*13:21	FVWTHYYSVKKTFLG	TRP1	P17643	188	202	33.53
5050	20	DRB1*13:21	GPAFLTWHRYHLLRL	TRP1	P17643	217	231	48.21
5051	21	DRB1*13:21	GTASYLIRARRSMDE	TRP1	P17643	495	509	24.11
5052	22	DRB1*13:21	HPLFVIATRRSEEIL	TRP1	P17643	156	170	12
5053	23	DRB1*13:21	IFGTASYLIRARRSM	TRP1	P17643	493	507	47.58
5054	24	DRB1*13:21	LFVIATRRSEEILGP	TRP1	P17643	158	172	13.5
5055	25	DRB1*13:21	PLFVIATRRSEEILG	TRP1	P17643	157	171	11.53
5056	26	DRB1*13:21	QRVLIVRRNLLDLSK	TRP1	P17643	124	138	33.15
5057	27	DRB1*13:21	RTTHPLFVIATRRSE	TRP1	P17643	153	167	14.84
5058	28	DRB1*13:21	SYLIRARRSMDEANQ	TRP1	P17643	498	512	26.61
5059	29	DRB1*13:21	TASYLIRARRSMDEA	TRP1	P17643	496	510	20.41
5060	30	DRB1*13:21	THPLFVIATRRSEEI	TRP1	P17643	155	169	13.94
5061	31	DRB1*13:21	THYYSVKKTFLGVGQ	TRP1	P17643	191	205	42.33
5062	32	DRB1*13:21	TTHPLFVIATRRSEE	TRP1	P17643	154	168	16.4
5063	33	DRB1*13:21	TWHRYHLLRLEKDMQ	TRP1	P17643	222	236	49.73
5064	34	DRB1*13:21	VWTHYYSVKKTFLGV	TRP1	P17643	189	203	30.57
5065	35	DRB1*13:21	WTHYYSVKKTFLGVG	TRP1	P17643	190	204	35.86
5066	36	DRB1*13:21	YFVWTHYYSVKKTFL	TRP1	P17643	187	201	36.31
5067	37	DRB1*13:21	YLIRARRSMDEANQP	TRP1	P17643	499	513	45.73
5068	38	DRB1*13:21	ATLRLVKRQVPLDCV	PMEL17	P40967	462	476	34.66
5069	39	DRB1*13:21	AVVLASLIYRRRLMK	PMEL17	P40967	608	622	41.5
5070	40	DRB1*13:21	DGTATLRLVKRQVPL	PMEL17	P40967	459	473	21.14
5071	41	DRB1*13:21	GTATLRLVKRQVPLD	PMEL17	P40967	460	474	25.67
5072	42	DRB1*13:21	KRSFVYVWKTWGQYW	PMEL17	P40967	146	160	32.66

5073	43	DRB1*13:21	LDGTATLRLVKRQVP	PMEL17	P40967	458	472	39.45
5074	44	DRB1*13:21	QKRSFVYVWKTWGQY	PMEL17	P40967	145	159	30.04
5075	45	DRB1*13:21	SQKRSFVYVWKTWGQ	PMEL17	P40967	144	158	32.23
5076	46	DRB1*13:21	TATLRLVKRQVPLDC	PMEL17	P40967	461	475	29.21
5077	47	DRB1*13:21	VLASLIYRRRLMKQD	PMEL17	P40967	610	624	46.93
5078	48	DRB1*13:21	VVLASLIYRRRLMKQ	PMEL17	P40967	609	623	38.83
5079	49	DRB1*13:21	WSQKRSFVYVWKTWG	PMEL17	P40967	143	157	41.02
5080	50	DRB1*13:21	ADLVGFLLLKYRARE	MAGE1	P43355	107	121	23.8
5081	51	DRB1*13:21	ARVRFFFPSLREAAL	MAGE1	P43355	288	302	20.12
5082	52	DRB1*13:21	ARYEFLWGPRALAET	MAGE1	P43355	260	274	43.18
5083	53	DRB1*13:21	CILESLFRAVITKKV	MAGE1	P43355	92	106	42.81
5084	54	DRB1*13:21	DLVGFLLLKYRAREP	MAGE1	P43355	108	122	20.76
5085	55	DRB1*13:21	ESLFRAVITKKVADL	MAGE1	P43355	95	109	28.35
5086	56	DRB1*13:21	EYVIKVSARVRFFFP	MAGE1	P43355	281	295	40.17
5087	57	DRB1*13:21	FLLLKYRAREPVTKA	MAGE1	P43355	112	126	37.04
5088	58	DRB1*13:21	GFLLLKYRAREPVTK	MAGE1	P43355	111	125	25.61
5089	59	DRB1*13:21	ILESLFRAVITKKVA	MAGE1	P43355	93	107	29.28
5090	60	DRB1*13:21	KVLEYVIKVSARVRF	MAGE1	P43355	278	292	37
5091	61	DRB1*13:21	LESLFRAVITKKVAD	MAGE1	P43355	94	108	36.95
5092	62	DRB1*13:21	LEYVIKVSARVRFFF	MAGE1	P43355	280	294	41.23
5093	63	DRB1*13:21	LFRAVITKKVADLVG	MAGE1	P43355	97	111	37.44
5094	64	DRB1*13:21	LVGFLLLKYRAREPV	MAGE1	P43355	109	123	18.53
5095	65	DRB1*13:21	PARYEFLWGPRALAE	MAGE1	P43355	259	273	45.62
5096	66	DRB1*13:21	RFFFPSLREAALREE	MAGE1	P43355	291	305	26.46
5097	67	DRB1*13:21	RVRFFFPSLREAALR	MAGE1	P43355	289	303	16.73
5098	68	DRB1*13:21	RYEFLWGPRALAETS	MAGE1	P43355	261	275	47.73
5099	69	DRB1*13:21	SARVRFFFPSLREAA	MAGE1	P43355	287	301	29.54
5100	70	DRB1*13:21	SLFRAVITKKVADLV	MAGE1	P43355	96	110	27.52
5101	71	DRB1*13:21	VADLVGFLLLKYRAR	MAGE1	P43355	106	120	48.24
5102	72	DRB1*13:21	VGFLLLKYRAREPVT	MAGE1	P43355	110	124	20.9
5103	73	DRB1*13:21	VLEYVIKVSARVRFF	MAGE1	P43355	279	293	39.34
5104	74	DRB1*13:21	VRFFFPSLREAALRE	MAGE1	P43355	290	304	19.25
5105	75	DRB1*13:21	AHFLLRKYRAKELVT	MAGE4	P43358	118	132	28.14
5106	76	DRB1*13:21	ARYEFLWGPRALAET	MAGE4	P43358	268	282	43.18
5107	77	DRB1*13:21	DELAHFLLRKYRAKE	MAGE4	P43358	115	129	29.82
5108	78	DRB1*13:21	ELAHFLLRKYRAKEL	MAGE4	P43358	116	130	22.48
5109	79	DRB1*13:21	HFLLRKYRAKELVTK	MAGE4	P43358	119	133	37.82
5110	80	DRB1*13:21	LAHFLLRKYRAKELV	MAGE4	P43358	117	131	21.81
5111	81	DRB1*13:21	PARYEFLWGPRALAE	MAGE4	P43358	267	281	45.62
5112	82	DRB1*13:21	RYEFLWGPRALAETS	MAGE4	P43358	269	283	47.73

5113	83	DRB1*13:21	VDELAHFLLRKYRAK	MAGE4	P43358	114	128	37.58
5114	84	DRB1*13:21	ASEKIFYVYMKRKYE	SSX2	Q16385	42	56	10.4
5115	85	DRB1*13:21	EKIFYVYMKRKYEAM	SSX2	Q16385	44	58	8.54
5116	86	DRB1*13:21	FYVYMKRKYEAMTKL	SSX2	Q16385	47	61	12.4
5117	87	DRB1*13:21	IFYVYMKRKYEAMTK	SSX2	Q16385	46	60	9.31
5118	88	DRB1*13:21	KIFYVYMKRKYEAMT	SSX2	Q16385	45	59	8.9
5119	89	DRB1*13:21	KRKYEAMTKLGFKAT	SSX2	Q16385	52	66	40.41
5120	90	DRB1*13:21	MKRKYEAMTKLGFKA	SSX2	Q16385	51	65	38.53
5121	91	DRB1*13:21	RKYEAMTKLGFKATL	SSX2	Q16385	53	67	47.08
5122	92	DRB1*13:21	SEKIFYVYMKRKYEA	SSX2	Q16385	43	57	9.35
5123	93	DRB1*13:21	YMKRKYEAMTKLGFK	SSX2	Q16385	50	64	41.62
5124	94	DRB1*13:21	YVYMKRKYEAMTKLG	SSX2	Q16385	48	62	43.16
5125	1	DRB1*13:23	KKPPVIRQNIHSLSP	TRP2	O75767	121	135	47.85
5126	2	DRB1*13:23	KPPVIRQNIHSLSPQ	TRP2	O75767	122	136	47.66
5127	3	DRB1*13:23	RKKPPVIRQNIHSLS	TRP2	O75767	120	134	48.91
5128	4	DRB1*13:23	ELKELINNELSHFLE	S100	P04271	30	44	36.11
5129	5	DRB1*13:23	LKELINNELSHFLEE	S100	P04271	31	45	45.36
5130	6	DRB1*13:23	SELKELINNELSHFL	S100	P04271	29	43	34.41
5131	7	DRB1*13:23	ALHIYMNGTMSQVQG	Tyrosinase	P14679	365	379	24.22
5132	8	DRB1*13:23	CQNILLSNAPLGPQF	Tyrosinase	P14679	55	69	28.2
5133	9	DRB1*13:23	HNALHIYMNGTMSQV	Tyrosinase	P14679	363	377	23.28
5134	10	DRB1*13:23	KHTISSDYVIPIGTY	Tyrosinase	P14679	142	156	46.16
5135	11	DRB1*13:23	LHIYMNGTMSQVQGS	Tyrosinase	P14679	366	380	29.98
5136	12	DRB1*13:23	MHNALHIYMNGTMSQ	Tyrosinase	P14679	362	376	39.88
5137	13	DRB1*13:23	NALHIYMNGTMSQVQ	Tyrosinase	P14679	364	378	21.33
5138	14	DRB1*13:23	NILLSNAPLGPQFPF	Tyrosinase	P14679	57	71	47.26
5139	15	DRB1*13:23	QNILLSNAPLGPQFP	Tyrosinase	P14679	56	70	28.71
5140	16	DRB1*13:23	SCQNILLSNAPLGPQ	Tyrosinase	P14679	54	68	41.65
5141	17	DRB1*13:23	DGPIRRNPAGNVARP	TRP1	P17643	308	322	36.78
5142	18	DRB1*13:23	DTPPFYSNSTNSFRN	TRP1	P17643	343	357	25.79
5143	19	DRB1*13:23	EDGPIRRNPAGNVAR	TRP1	P17643	307	321	32.26
5144	20	DRB1*13:23	FDTPPFYSNSTNSFR	TRP1	P17643	342	356	28.28
5145	21	DRB1*13:23	GPIRRNPAGNVARPM	TRP1	P17643	309	323	35.1
5146	22	DRB1*13:23	GQTHLSPNDPIFVLL	TRP1	P17643	389	403	34.38
5147	23	DRB1*13:23	PFYSNSTNSFRNTVE	TRP1	P17643	346	360	39.62
5148	24	DRB1*13:23	PIRRNPAGNVARPMV	TRP1	P17643	310	324	43.15
5149	25	DRB1*13:23	PPFYSNSTNSFRNTV	TRP1	P17643	345	359	31.34
5150	26	DRB1*13:23	QTHLSPNDPIFVLLH	TRP1	P17643	390	404	32.73
5151	27	DRB1*13:23	TEDGPIRRNPAGNVA	TRP1	P17643	306	320	45.36

5152	28	DRB1*13:23	THLSPNDPIFVLLHT	TRP1	P17643	391	405	43.62
5153	29	DRB1*13:23	TPPFYSNSTNSFRNT	TRP1	P17643	344	358	24.09
5154	30	DRB1*13:23	VALIFGTASYLIRAR	TRP1	P17643	490	504	45.96
5155	31	DRB1*13:23	AQVVLQAAIPLTSCG	PMEL17	P40967	288	302	43.39
5156	32	DRB1*13:23	ASFSIALNFPGSQKV	PMEL17	P40967	82	96	34.8
5157	33	DRB1*13:23	AVIGALLAVGATKVP	PMEL17	P40967	13	27	46.91
5158	34	DRB1*13:23	DGGNKHFLRNQPLTF	PMEL17	P40967	226	240	18.26
5159	35	DRB1*13:23	DGPTLIGANASFSIA	PMEL17	P40967	73	87	24.48
5160	36	DRB1*13:23	DGQVIWVNNTIINGS	PMEL17	P40967	99	113	17.49
5161	37	DRB1*13:23	EVSIVVLSGTTAAQV	PMEL17	P40967	404	418	49.09
5162	38	DRB1*13:23	FSIALNFPGSQKVLP	PMEL17	P40967	84	98	37.57
5163	39	DRB1*13:23	GGNKHFLRNQPLTFA	PMEL17	P40967	227	241	11.13
5164	40	DRB1*13:23	GNKHFLRNQPLTFAL	PMEL17	P40967	228	242	8.61
5165	41	DRB1*13:23	GPTLIGANASFSIAL	PMEL17	P40967	74	88	17.01
5166	42	DRB1*13:23	GPVTAQVVLQAAIPL	PMEL17	P40967	284	298	35.55
5167	43	DRB1*13:23	GQVIWVNNTIINGSQ	PMEL17	P40967	100	114	16.48
5168	44	DRB1*13:23	HFLRNQPLTFALQLH	PMEL17	P40967	231	245	17.65
5169	45	DRB1*13:23	IGALLAVGATKVPRN	PMEL17	P40967	15	29	34.24
5170	46	DRB1*13:23	KHFLRNQPLTFALQL	PMEL17	P40967	230	244	9.79
5171	47	DRB1*13:23	LIGANASFSIALNFP	PMEL17	P40967	77	91	36.78
5172	48	DRB1*13:23	LPDGQVIWVNNTIIN	PMEL17	P40967	97	111	23.66
5173	49	DRB1*13:23	NASFSIALNFPGSQK	PMEL17	P40967	81	95	49.03
5174	50	DRB1*13:23	NDGPTLIGANASFSI	PMEL17	P40967	72	86	44.44
5175	51	DRB1*13:23	NKHFLRNQPLTFALQ	PMEL17	P40967	229	243	8.69
5176	52	DRB1*13:23	PDGQVIWVNNTIING	PMEL17	P40967	98	112	19.68
5177	53	DRB1*13:23	PTLIGANASFSIALN	PMEL17	P40967	75	89	17.72
5178	54	DRB1*13:23	PVTAQVVLQAAIPLT	PMEL17	P40967	285	299	24.67
5179	55	DRB1*13:23	QVIWVNNTIINGSQV	PMEL17	P40967	101	115	18.45
5180	56	DRB1*13:23	SFSIALNFPGSQKVL	PMEL17	P40967	83	97	27.92
5181	57	DRB1*13:23	TAQVVLQAAIPLTSC	PMEL17	P40967	287	301	29.07
5182	58	DRB1*13:23	TLIGANASFSIALNF	PMEL17	P40967	76	90	20.23
5183	59	DRB1*13:23	VIGALLAVGATKVPR	PMEL17	P40967	14	28	32.72
5184	60	DRB1*13:23	VIWVNNTIINGSQVW	PMEL17	P40967	102	116	31.07
5185	61	DRB1*13:23	VSIVVLSGTTAAQVT	PMEL17	P40967	405	419	43.21
5186	62	DRB1*13:23	VTAQVVLQAAIPLTS	PMEL17	P40967	286	300	20.55
5187	63	DRB1*13:23	EYVIKVSARVRFFFP	MAGE1	P43355	281	295	26.42
5188	64	DRB1*13:23	KVLEYVIKVSARVRF	MAGE1	P43355	278	292	29.26
5189	65	DRB1*13:23	LEYVIKVSARVRFFF	MAGE1	P43355	280	294	24.27
5190	66	DRB1*13:23	VKVLEYVIKVSARVR	MAGE1	P43355	277	291	46.25
5191	67	DRB1*13:23	VLEYVIKVSARVRFF	MAGE1	P43355	279	293	25.89

5192	68	DRB1*13:23	EHVVRVNARVRIAYP	MAGE4	P43358	289	303	20.84
5193	69	DRB1*13:23	ESLFREALSNKVDEL	MAGE4	P43358	103	117	46.34
5194	70	DRB1*13:23	HVVRVNARVRIAYPS	MAGE4	P43358	290	304	23.63
5195	71	DRB1*13:23	KVLEHVVRVNARVRI	MAGE4	P43358	286	300	26.57
5196	72	DRB1*13:23	LEHVVRVNARVRIAY	MAGE4	P43358	288	302	17.31
5197	73	DRB1*13:23	VLEHVVRVNARVRIA	MAGE4	P43358	287	301	20.72
5198	74	DRB1*13:23	VVRVNARVRIAYPSL	MAGE4	P43358	291	305	32.87
5199	75	DRB1*13:23	DHRQLQLSISSCLQQ	NY-ESO-1	P78358	141	155	42.47
5200	76	DRB1*13:23	HRQLQLSISSCLQQL	NY-ESO-1	P78358	142	156	36.44
5201	77	DRB1*13:23	KEFTVSGNILTIRLT	NY-ESO-1	P78358	124	138	36.33
5202	78	DRB1*13:23	LKEFTVSGNILTIRL	NY-ESO-1	P78358	123	137	28.04
5203	79	DRB1*13:23	LLKEFTVSGNILTIR	NY-ESO-1	P78358	122	136	29.99
5204	80	DRB1*13:23	VLLKEFTVSGNILTI	NY-ESO-1	P78358	121	135	49.15
5205	81	DRB1*13:23	RAVFLALSAQLLQAR	BAGE	Q13072	4	18	48.97
5206	1	DRB1*13:33	GNKHFLRNQPLTFAL	PMEL17	P40967	228	242	49.93
5207	1	DRB1*13:50	EVISCKLIKRATTRQ	TRP2	O75767	222	236	30.92
5208	2	DRB1*13:50	RKFFHRTCKCTGNFA	TRP2	O75767	88	102	39.55
5209	3	DRB1*13:50	VISCKLIKRATTRQP	TRP2	O75767	223	237	33.86
5210	4	DRB1*13:50	WEVISCKLIKRATTR	TRP2	O75767	221	235	36.89
5211	5	DRB1*13:50	YYYRFVIGLRVWQWE	TRP2	O75767	208	222	49.95
5212	6	DRB1*13:50	DKFFAYLTLAKHTIS	Tyrosinase	P14679	132	146	26.01
5213	7	DRB1*13:50	FAYLTLAKHTISSDY	Tyrosinase	P14679	135	149	35.24
5214	8	DRB1*13:50	FFAYLTLAKHTISSD	Tyrosinase	P14679	134	148	28.57
5215	9	DRB1*13:50	KDKFFAYLTLAKHTI	Tyrosinase	P14679	131	145	33.33
5216	10	DRB1*13:50	KFFAYLTLAKHTISS	Tyrosinase	P14679	133	147	22.47
5217	11	DRB1*13:50	MVPFIPLYRNGDFFI	Tyrosinase	P14679	426	440	32.73
5218	12	DRB1*13:50	VPFIPLYRNGDFFIS	Tyrosinase	P14679	427	441	41.52
5219	13	DRB1*13:50	YMVPFIPLYRNGDFF	Tyrosinase	P14679	425	439	42.2
5220	14	DRB1*13:50	ASYLIRARRSMDEAN	TRP1	P17643	497	511	46.19
5221	15	DRB1*13:50	FVWTHYYSVKKTFLG	TRP1	P17643	188	202	47.59
5222	16	DRB1*13:50	HPLFVIATRRSEEIL	TRP1	P17643	156	170	13.74
5223	17	DRB1*13:50	LFVIATRRSEEILGP	TRP1	P17643	158	172	24.07
5224	18	DRB1*13:50	PLFVIATRRSEEILG	TRP1	P17643	157	171	15.42
5225	19	DRB1*13:50	RTTHPLFVIATRRSE	TRP1	P17643	153	167	24.44
5226	20	DRB1*13:50	TASYLIRARRSMDEA	TRP1	P17643	496	510	39.65
5227	21	DRB1*13:50	THPLFVIATRRSEEI	TRP1	P17643	155	169	15.19
5228	22	DRB1*13:50	THYYSVKKTFLGVGQ	TRP1	P17643	191	205	37.63
5229	23	DRB1*13:50	TTHPLFVIATRRSEE	TRP1	P17643	154	168	19.39

5230	24	DRB1*13:50	VWTHYYSVKKTFLGV	TRP1	P17643	189	203	31.01
5231	25	DRB1*13:50	WTHYYSVKKTFLGVG	TRP1	P17643	190	204	32.76
5232	26	DRB1*13:50	ANASFSIALNFPGSQ	PMEL17	P40967	80	94	49.44
5233	27	DRB1*13:50	ASFSIALNFPGSQKV	PMEL17	P40967	82	96	42.13
5234	28	DRB1*13:50	ATLRLVKRQVPLDCV	PMEL17	P40967	462	476	32.58
5235	29	DRB1*13:50	CQLVLHQILKGGSGT	PMEL17	P40967	550	564	45.41
5236	30	DRB1*13:50	DGTATLRLVKRQVPL	PMEL17	P40967	459	473	18.27
5237	31	DRB1*13:50	GSRSYVPLAHSSSAF	PMEL17	P40967	193	207	39.7
5238	32	DRB1*13:50	GTATLRLVKRQVPLD	PMEL17	P40967	460	474	20.16
5239	33	DRB1*13:50	KRSFVYVWKTWGQYW	PMEL17	P40967	146	160	17.94
5240	34	DRB1*13:50	NASFSIALNFPGSQK	PMEL17	P40967	81	95	33.06
5241	35	DRB1*13:50	QKRSFVYVWKTWGQY	PMEL17	P40967	145	159	18.3
5242	36	DRB1*13:50	QLVLHQILKGGSGTY	PMEL17	P40967	551	565	36.77
5243	37	DRB1*13:50	RSFVYVWKTWGQYWQ	PMEL17	P40967	147	161	30.25
5244	38	DRB1*13:50	RSYVPLAHSSSAFTI	PMEL17	P40967	195	209	42.32
5245	39	DRB1*13:50	SQKRSFVYVWKTWGQ	PMEL17	P40967	144	158	21.74
5246	40	DRB1*13:50	SRSYVPLAHSSSAFT	PMEL17	P40967	194	208	39.52
5247	41	DRB1*13:50	TATLRLVKRQVPLDC	PMEL17	P40967	461	475	23.06
5248	42	DRB1*13:50	WSQKRSFVYVWKTWG	PMEL17	P40967	143	157	41.25
5249	43	DRB1*13:50	ADLVGFLLLKYRARE	MAGE1	P43355	107	121	34.07
5250	44	DRB1*13:50	ARVRFFFPSLREAAL	MAGE1	P43355	288	302	21.89
5251	45	DRB1*13:50	ARYEFLWGPRALAET	MAGE1	P43355	260	274	33.84
5252	46	DRB1*13:50	DLVGFLLLKYRAREP	MAGE1	P43355	108	122	28.93
5253	47	DRB1*13:50	ESLFRAVITKKVADL	MAGE1	P43355	95	109	29.13
5254	48	DRB1*13:50	EYVIKVSARVRFFFP	MAGE1	P43355	281	295	39.57
5255	49	DRB1*13:50	FFFPSLREAALREEE	MAGE1	P43355	292	306	45.29
5256	50	DRB1*13:50	GFLLLKYRAREPVTK	MAGE1	P43355	111	125	45.81
5257	51	DRB1*13:50	ILESLFRAVITKKVA	MAGE1	P43355	93	107	32.17
5258	52	DRB1*13:50	KVLEYVIKVSARVRF	MAGE1	P43355	278	292	25.6
5259	53	DRB1*13:50	LESLFRAVITKKVAD	MAGE1	P43355	94	108	37.28
5260	54	DRB1*13:50	LEYVIKVSARVRFFF	MAGE1	P43355	280	294	34.02
5261	55	DRB1*13:50	LVGFLLLKYRAREPV	MAGE1	P43355	109	123	26.38
5262	56	DRB1*13:50	PARYEFLWGPRALAE	MAGE1	P43355	259	273	37.79
5263	57	DRB1*13:50	RFFFPSLREAALREE	MAGE1	P43355	291	305	18.8
5264	58	DRB1*13:50	RVRFFFPSLREAALR	MAGE1	P43355	289	303	14
5265	59	DRB1*13:50	RYEFLWGPRALAETS	MAGE1	P43355	261	275	34.81
5266	60	DRB1*13:50	SARVRFFFPSLREAA	MAGE1	P43355	287	301	49.09
5267	61	DRB1*13:50	SLFRAVITKKVADLV	MAGE1	P43355	96	110	32.18
5268	62	DRB1*13:50	VGFLLLKYRAREPVT	MAGE1	P43355	110	124	31.71
5269	63	DRB1*13:50	VKVLEYVIKVSARVR	MAGE1	P43355	277	291	29.38

5270	64	DRB1*13:50	VLEYVIKVSARVRFF	MAGE1	P43355	279	293	31.77
5271	65	DRB1*13:50	VRFFFPSLREAALRE	MAGE1	P43355	290	304	14.6
5272	66	DRB1*13:50	AHFLLRKYRAKELVT	MAGE4	P43358	118	132	42.25
5273	67	DRB1*13:50	ARYEFLWGPRALAET	MAGE4	P43358	268	282	33.84
5274	68	DRB1*13:50	DELAHFLLRKYRAKE	MAGE4	P43358	115	129	39.02
5275	69	DRB1*13:50	ELAHFLLRKYRAKEL	MAGE4	P43358	116	130	28.81
5276	70	DRB1*13:50	KVLEHVVRVNARVRI	MAGE4	P43358	286	300	31.5
5277	71	DRB1*13:50	LAHFLLRKYRAKELV	MAGE4	P43358	117	131	29.7
5278	72	DRB1*13:50	PARYEFLWGPRALAE	MAGE4	P43358	267	281	37.79
5279	73	DRB1*13:50	RYEFLWGPRALAETS	MAGE4	P43358	269	283	34.81
5280	74	DRB1*13:50	SYVKVLEHVVRVNAR	MAGE4	P43358	283	297	47.81
5281	75	DRB1*13:50	VDELAHFLLRKYRAK	MAGE4	P43358	114	128	43.08
5282	76	DRB1*13:50	VKVLEHVVRVNARVR	MAGE4	P43358	285	299	30.17
5283	77	DRB1*13:50	YVKVLEHVVRVNARV	MAGE4	P43358	284	298	40.41
5284	78	DRB1*13:50	RAVFLALSAQLLQAR	BAGE	Q13072	4	18	49.7
5285	79	DRB1*13:50	ASEKIFYVYMKRKYE	SSX2	Q16385	42	56	21.98
5286	80	DRB1*13:50	EKIFYVYMKRKYEAM	SSX2	Q16385	44	58	12.58
5287	81	DRB1*13:50	FYVYMKRKYEAMTKL	SSX2	Q16385	47	61	19.78
5288	82	DRB1*13:50	IFYVYMKRKYEAMTK	SSX2	Q16385	46	60	13.87
5289	83	DRB1*13:50	KIFYVYMKRKYEAMT	SSX2	Q16385	45	59	12.77
5290	84	DRB1*13:50	KRKYEAMTKLGFKAT	SSX2	Q16385	52	66	47.42
5291	85	DRB1*13:50	MKRKYEAMTKLGFKA	SSX2	Q16385	51	65	47.51
5292	86	DRB1*13:50	SEKIFYVYMKRKYEA	SSX2	Q16385	43	57	15.39
5293	1	DRB1*13:61	DQRVLIVRRNLLDLS	TRP1	P17643	123	137	48.5
5294	2	DRB1*13:61	QRVLIVRRNLLDLSK	TRP1	P17643	124	138	41.23
5295	3	DRB1*13:61	AVVLASLIYRRRLMK	PMEL17	P40967	608	622	45.15
5296	4	DRB1*13:61	NKHFLRNQPLTFALQ	PMEL17	P40967	229	243	48.63
5297	5	DRB1*13:61	VVLASLIYRRRLMKQ	PMEL17	P40967	609	623	45.4
5298	6	DRB1*13:61	EYVIKVSARVRFFFP	MAGE1	P43355	281	295	18.53
5299	7	DRB1*13:61	KVLEYVIKVSARVRF	MAGE1	P43355	278	292	28.47
5300	8	DRB1*13:61	LEYVIKVSARVRFFF	MAGE1	P43355	280	294	17.8
5301	9	DRB1*13:61	VIKVSARVRFFFPSL	MAGE1	P43355	283	297	47.46
5302	10	DRB1*13:61	VLEYVIKVSARVRFF	MAGE1	P43355	279	293	20.95
5303	11	DRB1*13:61	YVIKVSARVRFFFPS	MAGE1	P43355	282	296	25.8
5304	12	DRB1*13:61	EHVVRVNARVRIAYP	MAGE4	P43358	289	303	19.49
5305	13	DRB1*13:61	HVVRVNARVRIAYPS	MAGE4	P43358	290	304	21.02
5306	14	DRB1*13:61	KVLEHVVRVNARVRI	MAGE4	P43358	286	300	30.33
5307	15	DRB1*13:61	LEHVVRVNARVRIAY	MAGE4	P43358	288	302	15.71
5308	16	DRB1*13:61	VLEHVVRVNARVRIA	MAGE4	P43358	287	301	20.85

5309	17	DRB1*13:61	VVRVNARVRIAYPSL	MAGE4	P43358	291	305	30.1
5310	1	DRB1*13:66	HPLFVIATRRSEEIL	TRP1	P17643	156	170	44.42
5311	2	DRB1*13:66	PLFVIATRRSEEILG	TRP1	P17643	157	171	46.44
5312	3	DRB1*13:66	DGGNKHFLRNQPLTF	PMEL17	P40967	226	240	48.03
5313	4	DRB1*13:66	GGNKHFLRNQPLTFA	PMEL17	P40967	227	241	35.47
5314	5	DRB1*13:66	GNKHFLRNQPLTFAL	PMEL17	P40967	228	242	27.75
5315	6	DRB1*13:66	KHFLRNQPLTFALQL	PMEL17	P40967	230	244	32.11
5316	7	DRB1*13:66	NKHFLRNQPLTFALQ	PMEL17	P40967	229	243	29.47
5317	8	DRB1*13:66	VLEYVIKVSARVRFF	MAGE1	P43355	279	293	49.8
5318	1	DRB1*13:96	ELKELINNELSHFLE	S100	P04271	30	44	46.72
5319	2	DRB1*13:96	SELKELINNELSHFL	S100	P04271	29	43	44.29
5320	3	DRB1*13:96	ALHIYMNGTMSQVQG	Tyrosinase	P14679	365	379	47.79
5321	4	DRB1*13:96	HNALHIYMNGTMSQV	Tyrosinase	P14679	363	377	47.7
5322	5	DRB1*13:96	NALHIYMNGTMSQVQ	Tyrosinase	P14679	364	378	42.15
5323	6	DRB1*13:96	DTPPFYSNSTNSFRN	TRP1	P17643	343	357	45.85
5324	7	DRB1*13:96	GQTHLSPNDPIFVLL	TRP1	P17643	389	403	43.28
5325	8	DRB1*13:96	QTHLSPNDPIFVLLH	TRP1	P17643	390	404	40.61
5326	9	DRB1*13:96	TPPFYSNSTNSFRNT	TRP1	P17643	344	358	41.58
5327	10	DRB1*13:96	DGGNKHFLRNQPLTF	PMEL17	P40967	226	240	25.84
5328	11	DRB1*13:96	DGPTLIGANASFSIA	PMEL17	P40967	73	87	41.5
5329	12	DRB1*13:96	DGQVIWVNNTIINGS	PMEL17	P40967	99	113	25.54
5330	13	DRB1*13:96	GGNKHFLRNQPLTFA	PMEL17	P40967	227	241	14.94
5331	14	DRB1*13:96	GNKHFLRNQPLTFAL	PMEL17	P40967	228	242	11.31
5332	15	DRB1*13:96	GPTLIGANASFSIAL	PMEL17	P40967	74	88	27.88
5333	16	DRB1*13:96	GQVIWVNNTIINGSQ	PMEL17	P40967	100	114	23.52
5334	17	DRB1*13:96	HFLRNQPLTFALQLH	PMEL17	P40967	231	245	23.4
5335	18	DRB1*13:96	KHFLRNQPLTFALQL	PMEL17	P40967	230	244	12.84
5336	19	DRB1*13:96	LPDGQVIWVNNTIIN	PMEL17	P40967	97	111	42.31
5337	20	DRB1*13:96	NKHFLRNQPLTFALQ	PMEL17	P40967	229	243	11.5
5338	21	DRB1*13:96	PDGQVIWVNNTIING	PMEL17	P40967	98	112	30.82
5339	22	DRB1*13:96	PTLIGANASFSIALN	PMEL17	P40967	75	89	29.17
5340	23	DRB1*13:96	PVTAQVVLQAAIPLT	PMEL17	P40967	285	299	47.53
5341	24	DRB1*13:96	QVIWVNNTIINGSQV	PMEL17	P40967	101	115	24.99
5342	25	DRB1*13:96	SFSIALNFPGSQKVL	PMEL17	P40967	83	97	42.8
5343	26	DRB1*13:96	TLIGANASFSIALNF	PMEL17	P40967	76	90	31.33
5344	27	DRB1*13:96	VIWVNNTIINGSQVW	PMEL17	P40967	102	116	41.76
5345	28	DRB1*13:96	VTAQVVLQAAIPLTS	PMEL17	P40967	286	300	39.8
5346	29	DRB1*13:96	EYVIKVSARVRFFFP	MAGE1	P43355	281	295	38.38

5347	30	DRB1*13:96	KVLEYVIKVSARVRF	MAGE1	P43355	278	292	41.64
5348	31	DRB1*13:96	LEYVIKVSARVRFFF	MAGE1	P43355	280	294	32.92
5349	32	DRB1*13:96	VLEYVIKVSARVRFF	MAGE1	P43355	279	293	33.05
5350	33	DRB1*13:96	EHVVRVNARVRIAYP	MAGE4	P43358	289	303	28.4
5351	34	DRB1*13:96	HVVRVNARVRIAYPS	MAGE4	P43358	290	304	32.56
5352	35	DRB1*13:96	KVLEHVVRVNARVRI	MAGE4	P43358	286	300	39.49
5353	36	DRB1*13:96	LEHVVRVNARVRIAY	MAGE4	P43358	288	302	23.21
5354	37	DRB1*13:96	VLEHVVRVNARVRIA	MAGE4	P43358	287	301	28.34
5355	38	DRB1*13:96	VVRVNARVRIAYPSL	MAGE4	P43358	291	305	44.43
5356	39	DRB1*13:96	LKEFTVSGNILTIRL	NY-ESO-1	P78358	123	137	40.52
5357	40	DRB1*13:96	LLKEFTVSGNILTIR	NY-ESO-1	P78358	122	136	49.38
5358	1	DRB1*13:97	KKPPVIRQNIHSLSP	TRP2	O75767	121	135	47.85
5359	2	DRB1*13:97	KPPVIRQNIHSLSPQ	TRP2	O75767	122	136	47.66
5360	3	DRB1*13:97	RKKPPVIRQNIHSLS	TRP2	O75767	120	134	48.91
5361	4	DRB1*13:97	ELKELINNELSHFLE	S100	P04271	30	44	36.11
5362	5	DRB1*13:97	LKELINNELSHFLEE	S100	P04271	31	45	45.36
5363	6	DRB1*13:97	SELKELINNELSHFL	S100	P04271	29	43	34.41
5364	7	DRB1*13:97	ALHIYMNGTMSQVQG	Tyrosinase	P14679	365	379	24.22
5365	8	DRB1*13:97	CQNILLSNAPLGPQF	Tyrosinase	P14679	55	69	28.2
5366	9	DRB1*13:97	HNALHIYMNGTMSQV	Tyrosinase	P14679	363	377	23.28
5367	10	DRB1*13:97	KHTISSDYVIPIGTY	Tyrosinase	P14679	142	156	46.16
5368	11	DRB1*13:97	LHIYMNGTMSQVQGS	Tyrosinase	P14679	366	380	29.98
5369	12	DRB1*13:97	MHNALHIYMNGTMSQ	Tyrosinase	P14679	362	376	39.88
5370	13	DRB1*13:97	NALHIYMNGTMSQVQ	Tyrosinase	P14679	364	378	21.33
5371	14	DRB1*13:97	NILLSNAPLGPQFPF	Tyrosinase	P14679	57	71	47.26
5372	15	DRB1*13:97	QNILLSNAPLGPQFP	Tyrosinase	P14679	56	70	28.71
5373	16	DRB1*13:97	SCQNILLSNAPLGPQ	Tyrosinase	P14679	54	68	41.65
5374	17	DRB1*13:97	DGPIRRNPAGNVARP	TRP1	P17643	308	322	36.78
5375	18	DRB1*13:97	DTPPFYSNSTNSFRN	TRP1	P17643	343	357	25.79
5376	19	DRB1*13:97	EDGPIRRNPAGNVAR	TRP1	P17643	307	321	32.26
5377	20	DRB1*13:97	FDTPPFYSNSTNSFR	TRP1	P17643	342	356	28.28
5378	21	DRB1*13:97	GPIRRNPAGNVARPM	TRP1	P17643	309	323	35.1
5379	22	DRB1*13:97	GQTHLSPNDPIFVLL	TRP1	P17643	389	403	34.38
5380	23	DRB1*13:97	PFYSNSTNSFRNTVE	TRP1	P17643	346	360	39.62
5381	24	DRB1*13:97	PIRRNPAGNVARPMV	TRP1	P17643	310	324	43.15
5382	25	DRB1*13:97	PPFYSNSTNSFRNTV	TRP1	P17643	345	359	31.34
5383	26	DRB1*13:97	QTHLSPNDPIFVLLH	TRP1	P17643	390	404	32.73
5384	27	DRB1*13:97	TEDGPIRRNPAGNVA	TRP1	P17643	306	320	45.36
5385	28	DRB1*13:97	THLSPNDPIFVLLHT	TRP1	P17643	391	405	43.62

5386	29	DRB1*13:97	TPPFYSNSTNSFRNT	TRP1	P17643	344	358	24.09
5387	30	DRB1*13:97	VALIFGTASYLIRAR	TRP1	P17643	490	504	45.96
5388	31	DRB1*13:97	AQVVLQAAIPLTSCG	PMEL17	P40967	288	302	43.39
5389	32	DRB1*13:97	ASFSIALNFPGSQKV	PMEL17	P40967	82	96	34.8
5390	33	DRB1*13:97	AVIGALLAVGATKVP	PMEL17	P40967	13	27	46.91
5391	34	DRB1*13:97	DGGNKHFLRNQPLTF	PMEL17	P40967	226	240	18.26
5392	35	DRB1*13:97	DGPTLIGANASFSIA	PMEL17	P40967	73	87	24.48
5393	36	DRB1*13:97	DGQVIWVNNTIINGS	PMEL17	P40967	99	113	17.49
5394	37	DRB1*13:97	EVSIVVLSGTTAAQV	PMEL17	P40967	404	418	49.09
5395	38	DRB1*13:97	FSIALNFPGSQKVLP	PMEL17	P40967	84	98	37.57
5396	39	DRB1*13:97	GGNKHFLRNQPLTFA	PMEL17	P40967	227	241	11.13
5397	40	DRB1*13:97	GNKHFLRNQPLTFAL	PMEL17	P40967	228	242	8.61
5398	41	DRB1*13:97	GPTLIGANASFSIAL	PMEL17	P40967	74	88	17.01
5399	42	DRB1*13:97	GPVTAQVVLQAAIPL	PMEL17	P40967	284	298	35.55
5400	43	DRB1*13:97	GQVIWVNNTIINGSQ	PMEL17	P40967	100	114	16.48
5401	44	DRB1*13:97	HFLRNQPLTFALQLH	PMEL17	P40967	231	245	17.65
5402	45	DRB1*13:97	IGALLAVGATKVPRN	PMEL17	P40967	15	29	34.24
5403	46	DRB1*13:97	KHFLRNQPLTFALQL	PMEL17	P40967	230	244	9.79
5404	47	DRB1*13:97	LIGANASFSIALNFP	PMEL17	P40967	77	91	36.78
5405	48	DRB1*13:97	LPDGQVIWVNNTIIN	PMEL17	P40967	97	111	23.66
5406	49	DRB1*13:97	NASFSIALNFPGSQK	PMEL17	P40967	81	95	49.03
5407	50	DRB1*13:97	NDGPTLIGANASFSI	PMEL17	P40967	72	86	44.44
5408	51	DRB1*13:97	NKHFLRNQPLTFALQ	PMEL17	P40967	229	243	8.69
5409	52	DRB1*13:97	PDGQVIWVNNTIING	PMEL17	P40967	98	112	19.68
5410	53	DRB1*13:97	PTLIGANASFSIALN	PMEL17	P40967	75	89	17.72
5411	54	DRB1*13:97	PVTAQVVLQAAIPLT	PMEL17	P40967	285	299	24.67
5412	55	DRB1*13:97	QVIWVNNTIINGSQV	PMEL17	P40967	101	115	18.45
5413	56	DRB1*13:97	SFSIALNFPGSQKVL	PMEL17	P40967	83	97	27.92
5414	57	DRB1*13:97	TAQVVLQAAIPLTSC	PMEL17	P40967	287	301	29.07
5415	58	DRB1*13:97	TLIGANASFSIALNF	PMEL17	P40967	76	90	20.23
5416	59	DRB1*13:97	VIGALLAVGATKVPR	PMEL17	P40967	14	28	32.72
5417	60	DRB1*13:97	VIWVNNTIINGSQVW	PMEL17	P40967	102	116	31.07
5418	61	DRB1*13:97	VSIVVLSGTTAAQVT	PMEL17	P40967	405	419	43.21
5419	62	DRB1*13:97	VTAQVVLQAAIPLTS	PMEL17	P40967	286	300	20.55
5420	63	DRB1*13:97	EYVIKVSARVRFFFP	MAGE1	P43355	281	295	26.42
5421	64	DRB1*13:97	KVLEYVIKVSARVRF	MAGE1	P43355	278	292	29.26
5422	65	DRB1*13:97	LEYVIKVSARVRFFF	MAGE1	P43355	280	294	24.27
5423	66	DRB1*13:97	VKVLEYVIKVSARVR	MAGE1	P43355	277	291	46.25
5424	67	DRB1*13:97	VLEYVIKVSARVRFF	MAGE1	P43355	279	293	25.89
5425	68	DRB1*13:97	EHVVRVNARVRIAYP	MAGE4	P43358	289	303	20.84

5426	69	DRB1*13:97	ESLFREALSNKVDEL	MAGE4	P43358	103	117	46.34
5427	70	DRB1*13:97	HVVRVNARVRIAYPS	MAGE4	P43358	290	304	23.63
5428	71	DRB1*13:97	KVLEHVVRVNARVRI	MAGE4	P43358	286	300	26.57
5429	72	DRB1*13:97	LEHVVRVNARVRIAY	MAGE4	P43358	288	302	17.31
5430	73	DRB1*13:97	VLEHVVRVNARVRIA	MAGE4	P43358	287	301	20.72
5431	74	DRB1*13:97	VVRVNARVRIAYPSL	MAGE4	P43358	291	305	32.87
5432	75	DRB1*13:97	DHRQLQLSISSCLQQ	NY-ESO-1	P78358	141	155	42.47
5433	76	DRB1*13:97	HRQLQLSISSCLQQL	NY-ESO-1	P78358	142	156	36.44
5434	77	DRB1*13:97	KEFTVSGNILTIRLT	NY-ESO-1	P78358	124	138	36.33
5435	78	DRB1*13:97	LKEFTVSGNILTIRL	NY-ESO-1	P78358	123	137	28.04
5436	79	DRB1*13:97	LLKEFTVSGNILTIR	NY-ESO-1	P78358	122	136	29.99
5437	80	DRB1*13:97	VLLKEFTVSGNILTI	NY-ESO-1	P78358	121	135	49.15
5438	81	DRB1*13:97	RAVFLALSAQLLQAR	BAGE	Q13072	4	18	48.97
5439	1	DRB1*14:01	AACDQRVLIVRRNLL	TRP1	P17643	120	134	22.8
5440	2	DRB1*14:01	ACDQRVLIVRRNLLD	TRP1	P17643	121	135	28.98
5441	3	DRB1*14:01	CDQRVLIVRRNLLDL	TRP1	P17643	122	136	25.73
5442	4	DRB1*14:01	DQRVLIVRRNLLDLS	TRP1	P17643	123	137	26.29
5443	5	DRB1*14:01	QRVLIVRRNLLDLSK	TRP1	P17643	124	138	23.02
5444	6	DRB1*14:01	RVLIVRRNLLDLSKE	TRP1	P17643	125	139	32.49
5445	7	DRB1*14:01	EYVIKVSARVRFFFP	MAGE1	P43355	281	295	21.27
5446	8	DRB1*14:01	KVLEYVIKVSARVRF	MAGE1	P43355	278	292	23.15
5447	9	DRB1*14:01	LEYVIKVSARVRFFF	MAGE1	P43355	280	294	22.69
5448	10	DRB1*14:01	VIKVSARVRFFFPSL	MAGE1	P43355	283	297	36.15
5449	11	DRB1*14:01	VLEYVIKVSARVRFF	MAGE1	P43355	279	293	21.81
5450	12	DRB1*14:01	YVIKVSARVRFFFPS	MAGE1	P43355	282	296	25.1
5451	13	DRB1*14:01	WEKMKASEKIFYVYM	SSX2	Q16385	37	51	47.73
5452	1	DRB1*14:02	GGNKHFLRNQPLTFA	PMEL17	P40967	227	241	36.43
5453	2	DRB1*14:02	GNKHFLRNQPLTFAL	PMEL17	P40967	228	242	24.65
5454	3	DRB1*14:02	KHFLRNQPLTFALQL	PMEL17	P40967	230	244	26.81
5455	4	DRB1*14:02	NKHFLRNQPLTFALQ	PMEL17	P40967	229	243	23.05
5456	1	DRB1*14:04	AACDQRVLIVRRNLL	TRP1	P17643	120	134	44.06
5457	2	DRB1*14:04	ACDQRVLIVRRNLLD	TRP1	P17643	121	135	49.12
5458	3	DRB1*14:04	CDQRVLIVRRNLLDL	TRP1	P17643	122	136	42.51
5459	4	DRB1*14:04	DQRVLIVRRNLLDLS	TRP1	P17643	123	137	44.43
5460	5	DRB1*14:04	QRVLIVRRNLLDLSK	TRP1	P17643	124	138	42.59
5461	6	DRB1*14:04	EYVIKVSARVRFFFP	MAGE1	P43355	281	295	37.23
5462	7	DRB1*14:04	KVLEYVIKVSARVRF	MAGE1	P43355	278	292	42.1

5463	8	DRB1*14:04	LEYVIKVSARVRFFF	MAGE1	P43355	280	294	38.32
5464	9	DRB1*14:04	VLEYVIKVSARVRFF	MAGE1	P43355	279	293	39.3
5465	10	DRB1*14:04	YVIKVSARVRFFFPS	MAGE1	P43355	282	296	48.9
5466	1	DRB1*14:05	EYVIKVSARVRFFFP	MAGE1	P43355	281	295	43.67
5467	2	DRB1*14:05	KVLEYVIKVSARVRF	MAGE1	P43355	278	292	48.78
5468	3	DRB1*14:05	LEYVIKVSARVRFFF	MAGE1	P43355	280	294	45.32
5469	4	DRB1*14:05	VLEYVIKVSARVRFF	MAGE1	P43355	279	293	43.45
5470	1	DRB1*14:06	WEVISCKLIKRATTR	TRP2	O75767	221	235	48.94
5471	2	DRB1*14:06	CDQRVLIVRRNLLDL	TRP1	P17643	122	136	43.83
5472	3	DRB1*14:06	DQRVLIVRRNLLDLS	TRP1	P17643	123	137	38.56
5473	4	DRB1*14:06	FPLLLFQQARAQFPR	TRP1	P17643	14	28	48.96
5474	5	DRB1*14:06	QRVLIVRRNLLDLSK	TRP1	P17643	124	138	33.93
5475	6	DRB1*14:06	RVLIVRRNLLDLSKE	TRP1	P17643	125	139	45.95
5476	7	DRB1*14:06	AVVLASLIYRRRLMK	PMEL17	P40967	608	622	32.16
5477	8	DRB1*14:06	DGTATLRLVKRQVPL	PMEL17	P40967	459	473	41.99
5478	9	DRB1*14:06	GTATLRLVKRQVPLD	PMEL17	P40967	460	474	44.3
5479	10	DRB1*14:06	KHFLRNQPLTFALQL	PMEL17	P40967	230	244	48.8
5480	11	DRB1*14:06	LMAVVLASLIYRRRL	PMEL17	P40967	606	620	38.83
5481	12	DRB1*14:06	MAVVLASLIYRRRLM	PMEL17	P40967	607	621	34.66
5482	13	DRB1*14:06	NKHFLRNQPLTFALQ	PMEL17	P40967	229	243	46.86
5483	14	DRB1*14:06	VLMAVVLASLIYRRR	PMEL17	P40967	605	619	45.12
5484	15	DRB1*14:06	VVLASLIYRRRLMKQ	PMEL17	P40967	609	623	38.33
5485	16	DRB1*14:06	EYVIKVSARVRFFFP	MAGE1	P43355	281	295	24.03
5486	17	DRB1*14:06	KVLEYVIKVSARVRF	MAGE1	P43355	278	292	25.72
5487	18	DRB1*14:06	LEYVIKVSARVRFFF	MAGE1	P43355	280	294	23.98
5488	19	DRB1*14:06	VKVLEYVIKVSARVR	MAGE1	P43355	277	291	40.43
5489	20	DRB1*14:06	VLEYVIKVSARVRFF	MAGE1	P43355	279	293	24.13
5490	21	DRB1*14:06	YVIKVSARVRFFFPS	MAGE1	P43355	282	296	32.54
5491	22	DRB1*14:06	ARVRIAYPSLREAAL	MAGE4	P43358	296	310	45.97
5492	23	DRB1*14:06	EHVVRVNARVRIAYP	MAGE4	P43358	289	303	27.45
5493	24	DRB1*14:06	HVVRVNARVRIAYPS	MAGE4	P43358	290	304	31.3
5494	25	DRB1*14:06	KVLEHVVRVNARVRI	MAGE4	P43358	286	300	27.88
5495	26	DRB1*14:06	LEHVVRVNARVRIAY	MAGE4	P43358	288	302	22.55
5496	27	DRB1*14:06	NARVRIAYPSLREAA	MAGE4	P43358	295	309	42.99
5497	28	DRB1*14:06	VKVLEHVVRVNARVR	MAGE4	P43358	285	299	42.9
5498	29	DRB1*14:06	VLEHVVRVNARVRIA	MAGE4	P43358	287	301	25.83
5499	30	DRB1*14:06	VVRVNARVRIAYPSL	MAGE4	P43358	291	305	48.86
5500	31	DRB1*14:06	NILTIRLTAADHRQL	NY-ESO-1	P78358	131	145	48.71

5501	1	DRB1*14:07	GNKHFLRNQPLTFAL	PMEL17	P40967	228	242	44.47
5502	2	DRB1*14:07	NKHFLRNQPLTFALQ	PMEL17	P40967	229	243	45.3
5503	1	DRB1*14:12	QRVLIVRRNLLDLSK	TRP1	P17643	124	138	47.15
5504	2	DRB1*14:12	AVVLASLIYRRRLMK	PMEL17	P40967	608	622	44.8
5505	3	DRB1*14:12	VVLASLIYRRRLMKQ	PMEL17	P40967	609	623	47.51
5506	4	DRB1*14:12	EYVIKVSARVRFFFP	MAGE1	P43355	281	295	30.77
5507	5	DRB1*14:12	KVLEYVIKVSARVRF	MAGE1	P43355	278	292	35.2
5508	6	DRB1*14:12	LEYVIKVSARVRFFF	MAGE1	P43355	280	294	29.78
5509	7	DRB1*14:12	VLEYVIKVSARVRFF	MAGE1	P43355	279	293	31.56
5510	8	DRB1*14:12	YVIKVSARVRFFFPS	MAGE1	P43355	282	296	44.84
5511	9	DRB1*14:12	EHVVRVNARVRIAYP	MAGE4	P43358	289	303	46.45
5512	10	DRB1*14:12	KVLEHVVRVNARVRI	MAGE4	P43358	286	300	46.98
5513	11	DRB1*14:12	LEHVVRVNARVRIAY	MAGE4	P43358	288	302	36.53
5514	12	DRB1*14:12	VLEHVVRVNARVRIA	MAGE4	P43358	287	301	42.41
5515	1	DRB1*14:23	EYVIKVSARVRFFFP	MAGE1	P43355	281	295	43.67
5516	2	DRB1*14:23	KVLEYVIKVSARVRF	MAGE1	P43355	278	292	48.78
5517	3	DRB1*14:23	LEYVIKVSARVRFFF	MAGE1	P43355	280	294	45.32
5518	4	DRB1*14:23	VLEYVIKVSARVRFF	MAGE1	P43355	279	293	43.45
5519	1	DRB1*14:32	NDPIFLLHHAFVDSI	Tyrosinase	P14679	382	396	49.29
5520	2	DRB1*14:32	AACDQRVLIVRRNLL	TRP1	P17643	120	134	16.78
5521	3	DRB1*14:32	ACDQRVLIVRRNLLD	TRP1	P17643	121	135	15.85
5522	4	DRB1*14:32	CDQRVLIVRRNLLDL	TRP1	P17643	122	136	14.12
5523	5	DRB1*14:32	DQRVLIVRRNLLDLS	TRP1	P17643	123	137	13.71
5524	6	DRB1*14:32	FFPLLLFQQARAQFP	TRP1	P17643	13	27	42.65
5525	7	DRB1*14:32	FPLLLFQQARAQFPR	TRP1	P17643	14	28	39.85
5526	8	DRB1*14:32	HPLFVIATRRSEEIL	TRP1	P17643	156	170	44.57
5527	9	DRB1*14:32	PLLLFQQARAQFPRQ	TRP1	P17643	15	29	45.65
5528	10	DRB1*14:32	QRVLIVRRNLLDLSK	TRP1	P17643	124	138	13.6
5529	11	DRB1*14:32	RVLIVRRNLLDLSKE	TRP1	P17643	125	139	19.06
5530	12	DRB1*14:32	VALIFGTASYLIRAR	TRP1	P17643	490	504	46.2
5531	13	DRB1*14:32	AVVLASLIYRRRLMK	PMEL17	P40967	608	622	48.73
5532	14	DRB1*14:32	DGTATLRLVKRQVPL	PMEL17	P40967	459	473	35.57
5533	15	DRB1*14:32	GNKHFLRNQPLTFAL	PMEL17	P40967	228	242	44.49
5534	16	DRB1*14:32	GPVTAQVVLQAAIPL	PMEL17	P40967	284	298	48.86
5535	17	DRB1*14:32	GTATLRLVKRQVPLD	PMEL17	P40967	460	474	35.48
5536	18	DRB1*14:32	MAVVLASLIYRRRLM	PMEL17	P40967	607	621	46.89

5537	19	DRB1*14:32	NKHFLRNQPLTFALQ	PMEL17	P40967	229	243	42.81
5538	20	DRB1*14:32	PVTAQVVLQAAIPLT	PMEL17	P40967	285	299	38.11
5539	21	DRB1*14:32	TAQVVLQAAIPLTSC	PMEL17	P40967	287	301	47.07
5540	22	DRB1*14:32	TATLRLVKRQVPLDC	PMEL17	P40967	461	475	42.07
5541	23	DRB1*14:32	VTAQVVLQAAIPLTS	PMEL17	P40967	286	300	34.94
5542	24	DRB1*14:32	CILESLFRAVITKKV	MAGE1	P43355	92	106	44.43
5543	25	DRB1*14:32	EYVIKVSARVRFFFP	MAGE1	P43355	281	295	13.06
5544	26	DRB1*14:32	ILESLFRAVITKKVA	MAGE1	P43355	93	107	45.94
5545	27	DRB1*14:32	KVLEYVIKVSARVRF	MAGE1	P43355	278	292	16.02
5546	28	DRB1*14:32	LEYVIKVSARVRFFF	MAGE1	P43355	280	294	13.51
5547	29	DRB1*14:32	SCILESLFRAVITKK	MAGE1	P43355	91	105	42.62
5548	30	DRB1*14:32	VIKVSARVRFFFPSL	MAGE1	P43355	283	297	26.51
5549	31	DRB1*14:32	VKVLEYVIKVSARVR	MAGE1	P43355	277	291	36.1
5550	32	DRB1*14:32	VLEYVIKVSARVRFF	MAGE1	P43355	279	293	14.14
5551	33	DRB1*14:32	YVIKVSARVRFFFPS	MAGE1	P43355	282	296	16.15
5552	34	DRB1*14:32	EHVVRVNARVRIAYP	MAGE4	P43358	289	303	21.91
5553	35	DRB1*14:32	HVVRVNARVRIAYPS	MAGE4	P43358	290	304	26.51
5554	36	DRB1*14:32	KVLEHVVRVNARVRI	MAGE4	P43358	286	300	26.76
5555	37	DRB1*14:32	LEHVVRVNARVRIAY	MAGE4	P43358	288	302	19.69
5556	38	DRB1*14:32	VKVLEHVVRVNARVR	MAGE4	P43358	285	299	46.12
5557	39	DRB1*14:32	VLEHVVRVNARVRIA	MAGE4	P43358	287	301	24.5
5558	40	DRB1*14:32	VVRVNARVRIAYPSL	MAGE4	P43358	291	305	37.95
5559	41	DRB1*14:32	DHRQLQLSISSCLQQ	NY-ESO-1	P78358	141	155	48.59
5560	42	DRB1*14:32	HRQLQLSISSCLQQL	NY-ESO-1	P78358	142	156	44.18
5561	43	DRB1*14:32	ARAVFLALSAQLLQA	BAGE	Q13072	3	17	49.8
5562	44	DRB1*14:32	RAVFLALSAQLLQAR	BAGE	Q13072	4	18	40.48
5563	45	DRB1*14:32	EEWEKMKASEKIFYV	SSX2	Q16385	35	49	43.74
5564	46	DRB1*14:32	EKMKASEKIFYVYMK	SSX2	Q16385	38	52	40.87
5565	47	DRB1*14:32	EWEKMKASEKIFYVY	SSX2	Q16385	36	50	36.3
5566	48	DRB1*14:32	WEKMKASEKIFYVYM	SSX2	Q16385	37	51	34.22
5567	1	DRB1*14:38	QRVLIVRRNLLDLSK	TRP1	P17643	124	138	45.13
5568	2	DRB1*14:38	EYVIKVSARVRFFFP	MAGE1	P43355	281	295	39.37
5569	3	DRB1*14:38	KVLEYVIKVSARVRF	MAGE1	P43355	278	292	46.26
5570	4	DRB1*14:38	LEYVIKVSARVRFFF	MAGE1	P43355	280	294	38.02
5571	5	DRB1*14:38	VLEYVIKVSARVRFF	MAGE1	P43355	279	293	40.51
5572	6	DRB1*14:38	YVIKVSARVRFFFPS	MAGE1	P43355	282	296	44.16
5573	1	DRB1*14:54	AACDQRVLIVRRNLL	TRP1	P17643	120	134	22.8
5574	2	DRB1*14:54	ACDQRVLIVRRNLLD	TRP1	P17643	121	135	28.98

5575	3	DRB1*14:54	CDQRVLIVRRNLLDL	TRP1	P17643	122	136	25.73
5576	4	DRB1*14:54	DQRVLIVRRNLLDLS	TRP1	P17643	123	137	26.29
5577	5	DRB1*14:54	QRVLIVRRNLLDLSK	TRP1	P17643	124	138	23.02
5578	6	DRB1*14:54	RVLIVRRNLLDLSKE	TRP1	P17643	125	139	32.49
5579	7	DRB1*14:54	EYVIKVSARVRFFFP	MAGE1	P43355	281	295	21.27
5580	8	DRB1*14:54	KVLEYVIKVSARVRF	MAGE1	P43355	278	292	23.15
5581	9	DRB1*14:54	LEYVIKVSARVRFFF	MAGE1	P43355	280	294	22.69
5582	10	DRB1*14:54	VIKVSARVRFFFPSL	MAGE1	P43355	283	297	36.15
5583	11	DRB1*14:54	VLEYVIKVSARVRFF	MAGE1	P43355	279	293	21.81
5584	12	DRB1*14:54	YVIKVSARVRFFFPS	MAGE1	P43355	282	296	25.1
5585	13	DRB1*14:54	WEKMKASEKIFYVYM	SSX2	Q16385	37	51	47.73
5586	1	DRB1*15:01	DFFVWLHYYSVRDTL	TRP2	O75767	183	197	37.9
5587	2	DRB1*15:01	FFVWLHYYSVRDTLL	TRP2	O75767	184	198	35.04
5588	3	DRB1*15:01	FVWLHYYSVRDTLLG	TRP2	O75767	185	199	37.62
5589	4	DRB1*15:01	ALHIYMNGTMSQVQG	Tyrosinase	P14679	365	379	37.02
5590	5	DRB1*15:01	HNALHIYMNGTMSQV	Tyrosinase	P14679	363	377	21.63
5591	6	DRB1*15:01	MHNALHIYMNGTMSQ	Tyrosinase	P14679	362	376	26.91
5592	7	DRB1*15:01	NALHIYMNGTMSQVQ	Tyrosinase	P14679	364	378	23.69
5593	8	DRB1*15:01	SMHNALHIYMNGTMS	Tyrosinase	P14679	361	375	30.89
5594	9	DRB1*15:01	ALIFGTASYLIRARR	TRP1	P17643	491	505	29.58
5595	10	DRB1*15:01	FFPLLLFQQARAQFP	TRP1	P17643	13	27	35.55
5596	11	DRB1*15:01	FPLLLFQQARAQFPR	TRP1	P17643	14	28	41.01
5597	12	DRB1*15:01	GPAFLTWHRYHLLRL	TRP1	P17643	217	231	49.09
5598	13	DRB1*15:01	IFFPLLLFQQARAQF	TRP1	P17643	12	26	45.23
5599	14	DRB1*15:01	LVALIFGTASYLIRA	TRP1	P17643	489	503	35.69
5600	15	DRB1*15:01	VALIFGTASYLIRAR	TRP1	P17643	490	504	22.98
5601	16	DRB1*15:01	CVLYRYGSFSVTLDI	PMEL17	P40967	475	489	27.48
5602	17	DRB1*15:01	DCVLYRYGSFSVTLD	PMEL17	P40967	474	488	21.25
5603	18	DRB1*15:01	LDCVLYRYGSFSVTL	PMEL17	P40967	473	487	24.33
5604	19	DRB1*15:01	PLDCVLYRYGSFSVT	PMEL17	P40967	472	486	22.75
5605	20	DRB1*15:01	PVTAQVVLQAAIPLT	PMEL17	P40967	285	299	45.07
5606	21	DRB1*15:01	TAQVVLQAAIPLTSC	PMEL17	P40967	287	301	46.23
5607	22	DRB1*15:01	VLYRYGSFSVTLDIV	PMEL17	P40967	476	490	22.92
5608	23	DRB1*15:01	VPLDCVLYRYGSFSV	PMEL17	P40967	471	485	42.2
5609	24	DRB1*15:01	VTAQVVLQAAIPLTS	PMEL17	P40967	286	300	35.4
5610	25	DRB1*15:01	ARVRFFFPSLREAAL	MAGE1	P43355	288	302	43.77
5611	26	DRB1*15:01	GFLLLKYRAREPVTK	MAGE1	P43355	111	125	42.9
5612	27	DRB1*15:01	LEYVIKVSARVRFFF	MAGE1	P43355	280	294	40.1
5613	28	DRB1*15:01	NQIMPKTGFLIIVLV	MAGE1	P43355	186	200	49.46

5614	29	DRB1*15:01	SARVRFFFPSLREAA	MAGE1	P43355	287	301	38.73
5615	30	DRB1*15:01	VGFLLLKYRAREPVT	MAGE1	P43355	110	124	46.64
5616	31	DRB1*15:01	VLEYVIKVSARVRFF	MAGE1	P43355	279	293	43.36
5617	32	DRB1*15:01	VSARVRFFFPSLREA	MAGE1	P43355	286	300	42.26
5618	33	DRB1*15:01	AHFLLRKYRAKELVT	MAGE4	P43358	118	132	14.28
5619	34	DRB1*15:01	ASESLKMIFGIDVKE	MAGE4	P43358	155	169	49.82
5620	35	DRB1*15:01	ELAHFLLRKYRAKEL	MAGE4	P43358	116	130	26.43
5621	36	DRB1*15:01	FLLRKYRAKELVTKA	MAGE4	P43358	120	134	14.76
5622	37	DRB1*15:01	HFLLRKYRAKELVTK	MAGE4	P43358	119	133	13.46
5623	38	DRB1*15:01	LAHFLLRKYRAKELV	MAGE4	P43358	117	131	15.4
5624	39	DRB1*15:01	LLRKYRAKELVTKAE	MAGE4	P43358	121	135	25.64
5625	40	DRB1*15:01	SESLKMIFGIDVKEV	MAGE4	P43358	156	170	39.91
5626	41	DRB1*15:01	ESRLLEFYLAMPFAT	NY-ESO-1	P78358	84	98	36.32
5627	42	DRB1*15:01	PESRLLEFYLAMPFA	NY-ESO-1	P78358	83	97	43.74
5628	43	DRB1*15:01	RLLEFYLAMPFATPM	NY-ESO-1	P78358	86	100	34.19
5629	44	DRB1*15:01	SRLLEFYLAMPFATP	NY-ESO-1	P78358	85	99	29.01
5630	45	DRB1*15:01	AARAVFLALSAQLLQ	BAGE	Q13072	2	16	45.47
5631	46	DRB1*15:01	ARAVFLALSAQLLQA	BAGE	Q13072	3	17	35.58
5632	47	DRB1*15:01	RAVFLALSAQLLQAR	BAGE	Q13072	4	18	29.22
5633	1	DRB1*15:02	KVYYYRFVIGLRVWQ	TRP2	O75767	206	220	24.09
5634	2	DRB1*15:02	LKVYYYRFVIGLRVW	TRP2	O75767	205	219	30.65
5635	3	DRB1*15:02	VYYYRFVIGLRVWQW	TRP2	O75767	207	221	29.64
5636	4	DRB1*15:02	WLKVYYYRFVIGLRV	TRP2	O75767	204	218	33.96
5637	5	DRB1*15:02	YYYRFVIGLRVWQWE	TRP2	O75767	208	222	30.94
5638	6	DRB1*15:02	KDKFFAYLTLAKHTI	Tyrosinase	P14679	131	145	47.17
5639	7	DRB1*15:02	IYNYFVWTHYYSVKK	TRP1	P17643	184	198	43.85
5640	8	DRB1*15:02	NYFVWTHYYSVKKTF	TRP1	P17643	186	200	49.12
5641	9	DRB1*15:02	VALIFGTASYLIRAR	TRP1	P17643	490	504	43.9
5642	10	DRB1*15:02	YNYFVWTHYYSVKKT	TRP1	P17643	185	199	41.6
5643	11	DRB1*15:02	DCVLYRYGSFSVTLD	PMEL17	P40967	474	488	48.71
5644	12	DRB1*15:02	VLYRYGSFSVTLDIV	PMEL17	P40967	476	490	48.99
5645	13	DRB1*15:02	LEYVIKVSARVRFFF	MAGE1	P43355	280	294	49.76
5646	14	DRB1*15:02	VLEYVIKVSARVRFF	MAGE1	P43355	279	293	47.33
5647	15	DRB1*15:02	ESLFREALSNKVDEL	MAGE4	P43358	103	117	45.16
5648	16	DRB1*15:02	AARAVFLALSAQLLQ	BAGE	Q13072	2	16	23.52
5649	17	DRB1*15:02	ARAVFLALSAQLLQA	BAGE	Q13072	3	17	19.33
5650	18	DRB1*15:02	AVFLALSAQLLQARL	BAGE	Q13072	5	19	24.45
5651	19	DRB1*15:02	MAARAVFLALSAQLL	BAGE	Q13072	1	15	37.98
5652	20	DRB1*15:02	RAVFLALSAQLLQAR	BAGE	Q13072	4	18	16.16

5653	21	DRB1*15:02	VFLALSAQLLQARLM	BAGE	Q13072	6	20	39.35
5654	1	DRB1*15:03	AFLTWHRYHLLRLEK	TRP1	P17643	219	233	47.24
5655	2	DRB1*15:03	FFPLLLFQQARAQFP	TRP1	P17643	13	27	37.81
5656	3	DRB1*15:03	FPLLLFQQARAQFPR	TRP1	P17643	14	28	37.62
5657	4	DRB1*15:03	GPAFLTWHRYHLLRL	TRP1	P17643	217	231	48.26
5658	5	DRB1*15:03	PAFLTWHRYHLLRLE	TRP1	P17643	218	232	48.2
5659	6	DRB1*15:03	ARVRFFFPSLREAAL	MAGE1	P43355	288	302	44.07
5660	7	DRB1*15:03	SARVRFFFPSLREAA	MAGE1	P43355	287	301	42.04
5661	8	DRB1*15:03	VSARVRFFFPSLREA	MAGE1	P43355	286	300	44.58
5662	9	DRB1*15:03	AHFLLRKYRAKELVT	MAGE4	P43358	118	132	23.85
5663	10	DRB1*15:03	ELAHFLLRKYRAKEL	MAGE4	P43358	116	130	34.91
5664	11	DRB1*15:03	FLLRKYRAKELVTKA	MAGE4	P43358	120	134	23.85
5665	12	DRB1*15:03	HFLLRKYRAKELVTK	MAGE4	P43358	119	133	22.9
5666	13	DRB1*15:03	LAHFLLRKYRAKELV	MAGE4	P43358	117	131	23.76
5667	14	DRB1*15:03	LLRKYRAKELVTKAE	MAGE4	P43358	121	135	39.05
5668	15	DRB1*15:03	SRLLEFYLAMPFATP	NY-ESO-1	P78358	85	99	48.43
5669	16	DRB1*15:03	RAVFLALSAQLLQAR	BAGE	Q13072	4	18	43.91
5670	1	DRB1*15:06	DFFVWLHYYSVRDTL	TRP2	O75767	183	197	37.9
5671	2	DRB1*15:06	FFVWLHYYSVRDTLL	TRP2	O75767	184	198	35.04
5672	3	DRB1*15:06	FVWLHYYSVRDTLLG	TRP2	O75767	185	199	37.62
5673	4	DRB1*15:06	ALHIYMNGTMSQVQG	Tyrosinase	P14679	365	379	37.02
5674	5	DRB1*15:06	HNALHIYMNGTMSQV	Tyrosinase	P14679	363	377	21.63
5675	6	DRB1*15:06	MHNALHIYMNGTMSQ	Tyrosinase	P14679	362	376	26.91
5676	7	DRB1*15:06	NALHIYMNGTMSQVQ	Tyrosinase	P14679	364	378	23.69
5677	8	DRB1*15:06	SMHNALHIYMNGTMS	Tyrosinase	P14679	361	375	30.89
5678	9	DRB1*15:06	ALIFGTASYLIRARR	TRP1	P17643	491	505	29.58
5679	10	DRB1*15:06	FFPLLLFQQARAQFP	TRP1	P17643	13	27	35.55
5680	11	DRB1*15:06	FPLLLFQQARAQFPR	TRP1	P17643	14	28	41.01
5681	12	DRB1*15:06	GPAFLTWHRYHLLRL	TRP1	P17643	217	231	49.09
5682	13	DRB1*15:06	IFFPLLLFQQARAQF	TRP1	P17643	12	26	45.23
5683	14	DRB1*15:06	LVALIFGTASYLIRA	TRP1	P17643	489	503	35.69
5684	15	DRB1*15:06	VALIFGTASYLIRAR	TRP1	P17643	490	504	22.98
5685	16	DRB1*15:06	CVLYRYGSFSVTLDI	PMEL17	P40967	475	489	27.48
5686	17	DRB1*15:06	DCVLYRYGSFSVTLD	PMEL17	P40967	474	488	21.25
5687	18	DRB1*15:06	LDCVLYRYGSFSVTL	PMEL17	P40967	473	487	24.33
5688	19	DRB1*15:06	PLDCVLYRYGSFSVT	PMEL17	P40967	472	486	22.75
5689	20	DRB1*15:06	PVTAQVVLQAAIPLT	PMEL17	P40967	285	299	45.07
5690	21	DRB1*15:06	TAQVVLQAAIPLTSC	PMEL17	P40967	287	301	46.23

5691	22	DRB1*15:06	VLYRYGSFSVTLDIV	PMEL17	P40967	476	490	22.92
5692	23	DRB1*15:06	VPLDCVLYRYGSFSV	PMEL17	P40967	471	485	42.2
5693	24	DRB1*15:06	VTAQVVLQAAIPLTS	PMEL17	P40967	286	300	35.4
5694	25	DRB1*15:06	ARVRFFFPSLREAAL	MAGE1	P43355	288	302	43.77
5695	26	DRB1*15:06	GFLLLKYRAREPVTK	MAGE1	P43355	111	125	42.9
5696	27	DRB1*15:06	LEYVIKVSARVRFFF	MAGE1	P43355	280	294	40.1
5697	28	DRB1*15:06	NQIMPKTGFLIIVLV	MAGE1	P43355	186	200	49.46
5698	29	DRB1*15:06	SARVRFFFPSLREAA	MAGE1	P43355	287	301	38.73
5699	30	DRB1*15:06	VGFLLLKYRAREPVT	MAGE1	P43355	110	124	46.64
5700	31	DRB1*15:06	VLEYVIKVSARVRFF	MAGE1	P43355	279	293	43.36
5701	32	DRB1*15:06	VSARVRFFFPSLREA	MAGE1	P43355	286	300	42.26
5702	33	DRB1*15:06	AHFLLRKYRAKELVT	MAGE4	P43358	118	132	14.28
5703	34	DRB1*15:06	ASESLKMIFGIDVKE	MAGE4	P43358	155	169	49.82
5704	35	DRB1*15:06	ELAHFLLRKYRAKEL	MAGE4	P43358	116	130	26.43
5705	36	DRB1*15:06	FLLRKYRAKELVTKA	MAGE4	P43358	120	134	14.76
5706	37	DRB1*15:06	HFLLRKYRAKELVTK	MAGE4	P43358	119	133	13.46
5707	38	DRB1*15:06	LAHFLLRKYRAKELV	MAGE4	P43358	117	131	15.4
5708	39	DRB1*15:06	LLRKYRAKELVTKAE	MAGE4	P43358	121	135	25.64
5709	40	DRB1*15:06	SESLKMIFGIDVKEV	MAGE4	P43358	156	170	39.91
5710	41	DRB1*15:06	ESRLLEFYLAMPFAT	NY-ESO-1	P78358	84	98	36.32
5711	42	DRB1*15:06	PESRLLEFYLAMPFA	NY-ESO-1	P78358	83	97	43.74
5712	43	DRB1*15:06	RLLEFYLAMPFATPM	NY-ESO-1	P78358	86	100	34.19
5713	44	DRB1*15:06	SRLLEFYLAMPFATP	NY-ESO-1	P78358	85	99	29.01
5714	45	DRB1*15:06	AARAVFLALSAQLLQ	BAGE	Q13072	2	16	45.47
5715	46	DRB1*15:06	ARAVFLALSAQLLQA	BAGE	Q13072	3	17	35.58
5716	47	DRB1*15:06	RAVFLALSAQLLQAR	BAGE	Q13072	4	18	29.22
5717	1	DRB1*15:07	HNALHIYMNGTMSQV	Tyrosinase	P14679	363	377	45.9
5718	2	DRB1*15:07	ALIFGTASYLIRARR	TRP1	P17643	491	505	47.47
5719	3	DRB1*15:07	VALIFGTASYLIRAR	TRP1	P17643	490	504	37.71
5720	4	DRB1*15:07	DCVLYRYGSFSVTLD	PMEL17	P40967	474	488	49.01
5721	5	DRB1*15:07	PLDCVLYRYGSFSVT	PMEL17	P40967	472	486	49.89
5722	6	DRB1*15:07	AHFLLRKYRAKELVT	MAGE4	P43358	118	132	30.41
5723	7	DRB1*15:07	FLLRKYRAKELVTKA	MAGE4	P43358	120	134	31.24
5724	8	DRB1*15:07	HFLLRKYRAKELVTK	MAGE4	P43358	119	133	27.6
5725	9	DRB1*15:07	LAHFLLRKYRAKELV	MAGE4	P43358	117	131	33.39
5726	10	DRB1*15:07	RAVFLALSAQLLQAR	BAGE	Q13072	4	18	41.46
5727	1	DRB1*15:15	DFFVWLHYYSVRDTL	TRP2	O75767	183	197	48.79
5728	2	DRB1*15:15	KVYYYRFVIGLRVWQ	TRP2	O75767	206	220	30.51

5729	3	DRB1*15:15	LKVYYYRFVIGLRVW	TRP2	O75767	205	219	43.07
5730	4	DRB1*15:15	VYYYRFVIGLRVWQW	TRP2	O75767	207	221	43.09
5731	5	DRB1*15:15	WLKVYYYRFVIGLRV	TRP2	O75767	204	218	40.86
5732	6	DRB1*15:15	YYYRFVIGLRVWQWE	TRP2	O75767	208	222	41.32
5733	7	DRB1*15:15	DKFFAYLTLAKHTIS	Tyrosinase	P14679	132	146	36.67
5734	8	DRB1*15:15	EKDKFFAYLTLAKHT	Tyrosinase	P14679	130	144	31.94
5735	9	DRB1*15:15	KDKFFAYLTLAKHTI	Tyrosinase	P14679	131	145	27.49
5736	10	DRB1*15:15	KFFAYLTLAKHTISS	Tyrosinase	P14679	133	147	42.63
5737	11	DRB1*15:15	PEKDKFFAYLTLAKH	Tyrosinase	P14679	129	143	34.63
5738	12	DRB1*15:15	AFLTWHRYHLLRLEK	TRP1	P17643	219	233	41.33
5739	13	DRB1*15:15	FVWTHYYSVKKTFLG	TRP1	P17643	188	202	45.64
5740	14	DRB1*15:15	GPAFLTWHRYHLLRL	TRP1	P17643	217	231	45.21
5741	15	DRB1*15:15	IYNYFVWTHYYSVKK	TRP1	P17643	184	198	33.53
5742	16	DRB1*15:15	NYFVWTHYYSVKKTF	TRP1	P17643	186	200	30.43
5743	17	DRB1*15:15	PAFLTWHRYHLLRLE	TRP1	P17643	218	232	43.95
5744	18	DRB1*15:15	YFVWTHYYSVKKTFL	TRP1	P17643	187	201	34.18
5745	19	DRB1*15:15	YNYFVWTHYYSVKKT	TRP1	P17643	185	199	27.93
5746	20	DRB1*15:15	ANASFSIALNFPGSQ	PMEL17	P40967	80	94	40.83
5747	21	DRB1*15:15	ASFSIALNFPGSQKV	PMEL17	P40967	82	96	35.75
5748	22	DRB1*15:15	NASFSIALNFPGSQK	PMEL17	P40967	81	95	33.78
5749	23	DRB1*15:15	ARYEFLWGPRALAET	MAGE1	P43355	260	274	42.3
5750	24	DRB1*15:15	CILESLFRAVITKKV	MAGE1	P43355	92	106	49.2
5751	25	DRB1*15:15	DPARYEFLWGPRALA	MAGE1	P43355	258	272	39.43
5752	26	DRB1*15:15	ESLFRAVITKKVADL	MAGE1	P43355	95	109	23.9
5753	27	DRB1*15:15	ILESLFRAVITKKVA	MAGE1	P43355	93	107	26.52
5754	28	DRB1*15:15	KVLEYVIKVSARVRF	MAGE1	P43355	278	292	43.72
5755	29	DRB1*15:15	LESLFRAVITKKVAD	MAGE1	P43355	94	108	29.96
5756	30	DRB1*15:15	LEYVIKVSARVRFFF	MAGE1	P43355	280	294	43.25
5757	31	DRB1*15:15	LFRAVITKKVADLVG	MAGE1	P43355	97	111	38.05
5758	32	DRB1*15:15	PARYEFLWGPRALAE	MAGE1	P43355	259	273	43.71
5759	33	DRB1*15:15	SLFRAVITKKVADLV	MAGE1	P43355	96	110	24.66
5760	34	DRB1*15:15	VLEYVIKVSARVRFF	MAGE1	P43355	279	293	45.8
5761	35	DRB1*15:15	AHFLLRKYRAKELVT	MAGE4	P43358	118	132	29.66
5762	36	DRB1*15:15	ARYEFLWGPRALAET	MAGE4	P43358	268	282	42.3
5763	37	DRB1*15:15	ELAHFLLRKYRAKEL	MAGE4	P43358	116	130	47.38
5764	38	DRB1*15:15	ESLFREALSNKVDEL	MAGE4	P43358	103	117	39.09
5765	39	DRB1*15:15	FLLRKYRAKELVTKA	MAGE4	P43358	120	134	31.71
5766	40	DRB1*15:15	HFLLRKYRAKELVTK	MAGE4	P43358	119	133	27.56
5767	41	DRB1*15:15	LAHFLLRKYRAKELV	MAGE4	P43358	117	131	30.5
5768	42	DRB1*15:15	NPARYEFLWGPRALA	MAGE4	P43358	266	280	39.35

5769	43	DRB1*15:15	PARYEFLWGPRALAE	MAGE4	P43358	267	281	43.71
5770	44	DRB1*15:15	SLFREALSNKVDELA	MAGE4	P43358	104	118	42.09
5771	45	DRB1*15:15	AARAVFLALSAQLLQ	BAGE	Q13072	2	16	27.08
5772	46	DRB1*15:15	ARAVFLALSAQLLQA	BAGE	Q13072	3	17	22.79
5773	47	DRB1*15:15	AVFLALSAQLLQARL	BAGE	Q13072	5	19	25.32
5774	48	DRB1*15:15	MAARAVFLALSAQLL	BAGE	Q13072	1	15	48.71
5775	49	DRB1*15:15	RAVFLALSAQLLQAR	BAGE	Q13072	4	18	15.2
5776	50	DRB1*15:15	VFLALSAQLLQARLM	BAGE	Q13072	6	20	35.27
5777	1	DRB1*15:37	VALIFGTASYLIRAR	TRP1	P17643	490	504	45.68
5778	2	DRB1*15:37	DCVLYRYGSFSVTLD	PMEL17	P40967	474	488	49.93
5779	3	DRB1*15:37	AHFLLRKYRAKELVT	MAGE4	P43358	118	132	38.82
5780	4	DRB1*15:37	FLLRKYRAKELVTKA	MAGE4	P43358	120	134	38.24
5781	5	DRB1*15:37	HFLLRKYRAKELVTK	MAGE4	P43358	119	133	33.99
5782	6	DRB1*15:37	LAHFLLRKYRAKELV	MAGE4	P43358	117	131	44.07
5783	7	DRB1*15:37	SRLLEFYLAMPFATP	NY-ESO-1	P78358	85	99	49.34
5784	1	DRB1*16:01	KVYYYRFVIGLRVWQ	TRP2	O75767	206	220	29.62
5785	2	DRB1*16:01	LKVYYYRFVIGLRVW	TRP2	O75767	205	219	45.2
5786	3	DRB1*16:01	VYYYRFVIGLRVWQW	TRP2	O75767	207	221	38.89
5787	4	DRB1*16:01	WLKVYYYRFVIGLRV	TRP2	O75767	204	218	45.54
5788	5	DRB1*16:01	YYYRFVIGLRVWQWE	TRP2	O75767	208	222	36.45
5789	6	DRB1*16:01	KDKFFAYLTLAKHTI	Tyrosinase	P14679	131	145	46.43
5790	7	DRB1*16:01	WMHYYVSMDALLGGS	Tyrosinase	P14679	178	192	49.25
5791	8	DRB1*16:01	ANASFSIALNFPGSQ	PMEL17	P40967	80	94	33.47
5792	9	DRB1*16:01	ASFSIALNFPGSQKV	PMEL17	P40967	82	96	29.02
5793	10	DRB1*16:01	GANASFSIALNFPGS	PMEL17	P40967	79	93	40.98
5794	11	DRB1*16:01	NASFSIALNFPGSQK	PMEL17	P40967	81	95	27.36
5795	12	DRB1*16:01	ARYEFLWGPRALAET	MAGE1	P43355	260	274	42.2
5796	13	DRB1*16:01	CILESLFRAVITKKV	MAGE1	P43355	92	106	40.12
5797	14	DRB1*16:01	DPARYEFLWGPRALA	MAGE1	P43355	258	272	39.27
5798	15	DRB1*16:01	ESLFRAVITKKVADL	MAGE1	P43355	95	109	19
5799	16	DRB1*16:01	ILESLFRAVITKKVA	MAGE1	P43355	93	107	20.87
5800	17	DRB1*16:01	LESLFRAVITKKVAD	MAGE1	P43355	94	108	24.81
5801	18	DRB1*16:01	LFRAVITKKVADLVG	MAGE1	P43355	97	111	30.22
5802	19	DRB1*16:01	PARYEFLWGPRALAE	MAGE1	P43355	259	273	40.85
5803	20	DRB1*16:01	SLFRAVITKKVADLV	MAGE1	P43355	96	110	18.93
5804	21	DRB1*16:01	AESLFREALSNKVDE	MAGE4	P43358	102	116	48.38
5805	22	DRB1*16:01	ARYEFLWGPRALAET	MAGE4	P43358	268	282	42.2
5806	23	DRB1*16:01	ESLFREALSNKVDEL	MAGE4	P43358	103	117	33.94

5807	24	DRB1*16:01	NPARYEFLWGPRALA	MAGE4	P43358	266	280	39.27
5808	25	DRB1*16:01	PARYEFLWGPRALAE	MAGE4	P43358	267	281	40.85
5809	26	DRB1*16:01	SLFREALSNKVDELA	MAGE4	P43358	104	118	37.34
5810	27	DRB1*16:01	LKEFTVSGNILTIRL	NY-ESO-1	P78358	123	137	44.77
5811	28	DRB1*16:01	LLEFYLAMPFATPME	NY-ESO-1	P78358	87	101	48.01
5812	29	DRB1*16:01	LLKEFTVSGNILTIR	NY-ESO-1	P78358	122	136	48.67
5813	30	DRB1*16:01	AARAVFLALSAQLLQ	BAGE	Q13072	2	16	20.56
5814	31	DRB1*16:01	ARAVFLALSAQLLQA	BAGE	Q13072	3	17	17.84
5815	32	DRB1*16:01	AVFLALSAQLLQARL	BAGE	Q13072	5	19	19.65
5816	33	DRB1*16:01	MAARAVFLALSAQLL	BAGE	Q13072	1	15	33.9
5817	34	DRB1*16:01	RAVFLALSAQLLQAR	BAGE	Q13072	4	18	12.43
5818	35	DRB1*16:01	VFLALSAQLLQARLM	BAGE	Q13072	6	20	27.58
5819	1	DRB1*16:02	KVYYYRFVIGLRVWQ	TRP2	O75767	206	220	22.86
5820	2	DRB1*16:02	LKVYYYRFVIGLRVW	TRP2	O75767	205	219	32.79
5821	3	DRB1*16:02	VYYYRFVIGLRVWQW	TRP2	O75767	207	221	29.7
5822	4	DRB1*16:02	WLKVYYYRFVIGLRV	TRP2	O75767	204	218	31.11
5823	5	DRB1*16:02	YYRFVIGLRVWQWEV	TRP2	O75767	209	223	45.76
5824	6	DRB1*16:02	YYYRFVIGLRVWQWE	TRP2	O75767	208	222	28.76
5825	7	DRB1*16:02	KDKFFAYLTLAKHTI	Tyrosinase	P14679	131	145	40.61
5826	8	DRB1*16:02	MHYYVSMDALLGGSE	Tyrosinase	P14679	179	193	38.58
5827	9	DRB1*16:02	VWMHYYVSMDALLGG	Tyrosinase	P14679	177	191	41.31
5828	10	DRB1*16:02	WMHYYVSMDALLGGS	Tyrosinase	P14679	178	192	34.8
5829	11	DRB1*16:02	VALIFGTASYLIRAR	TRP1	P17643	490	504	47.68
5830	12	DRB1*16:02	ANASFSIALNFPGSQ	PMEL17	P40967	80	94	28.41
5831	13	DRB1*16:02	ASFSIALNFPGSQKV	PMEL17	P40967	82	96	29.77
5832	14	DRB1*16:02	GANASFSIALNFPGS	PMEL17	P40967	79	93	35.51
5833	15	DRB1*16:02	NASFSIALNFPGSQK	PMEL17	P40967	81	95	24.09
5834	16	DRB1*16:02	ARYEFLWGPRALAET	MAGE1	P43355	260	274	45.7
5835	17	DRB1*16:02	CILESLFRAVITKKV	MAGE1	P43355	92	106	38.27
5836	18	DRB1*16:02	DPARYEFLWGPRALA	MAGE1	P43355	258	272	41.06
5837	19	DRB1*16:02	ESLFRAVITKKVADL	MAGE1	P43355	95	109	18.02
5838	20	DRB1*16:02	ILESLFRAVITKKVA	MAGE1	P43355	93	107	20.52
5839	21	DRB1*16:02	KVLEYVIKVSARVRF	MAGE1	P43355	278	292	37.55
5840	22	DRB1*16:02	LESLFRAVITKKVAD	MAGE1	P43355	94	108	21.91
5841	23	DRB1*16:02	LEYVIKVSARVRFFF	MAGE1	P43355	280	294	40.37
5842	24	DRB1*16:02	LFRAVITKKVADLVG	MAGE1	P43355	97	111	39.28
5843	25	DRB1*16:02	PARYEFLWGPRALAE	MAGE1	P43355	259	273	45.18
5844	26	DRB1*16:02	SLFRAVITKKVADLV	MAGE1	P43355	96	110	21.29
5845	27	DRB1*16:02	VKVLEYVIKVSARVR	MAGE1	P43355	277	291	43.03

5846	28	DRB1*16:02	VLEYVIKVSARVRFF	MAGE1	P43355	279	293	39.58
5847	29	DRB1*16:02	AESLFREALSNKVDE	MAGE4	P43358	102	116	25.03
5848	30	DRB1*16:02	ARYEFLWGPRALAET	MAGE4	P43358	268	282	45.7
5849	31	DRB1*16:02	DAESLFREALSNKVD	MAGE4	P43358	101	115	24.6
5850	32	DRB1*16:02	ESLFREALSNKVDEL	MAGE4	P43358	103	117	17.65
5851	33	DRB1*16:02	LFREALSNKVDELAH	MAGE4	P43358	105	119	38.98
5852	34	DRB1*16:02	NPARYEFLWGPRALA	MAGE4	P43358	266	280	41.06
5853	35	DRB1*16:02	PARYEFLWGPRALAE	MAGE4	P43358	267	281	45.18
5854	36	DRB1*16:02	PDAESLFREALSNKV	MAGE4	P43358	100	114	40.04
5855	37	DRB1*16:02	SLFREALSNKVDELA	MAGE4	P43358	104	118	20.15
5856	38	DRB1*16:02	KEFTVSGNILTIRLT	NY-ESO-1	P78358	124	138	48.72
5857	39	DRB1*16:02	LKEFTVSGNILTIRL	NY-ESO-1	P78358	123	137	37.07
5858	40	DRB1*16:02	LLKEFTVSGNILTIR	NY-ESO-1	P78358	122	136	38.47
5859	41	DRB1*16:02	AARAVFLALSAQLLQ	BAGE	Q13072	2	16	12.16
5860	42	DRB1*16:02	ARAVFLALSAQLLQA	BAGE	Q13072	3	17	10.51
5861	43	DRB1*16:02	AVFLALSAQLLQARL	BAGE	Q13072	5	19	12.53
5862	44	DRB1*16:02	MAARAVFLALSAQLL	BAGE	Q13072	1	15	19.17
5863	45	DRB1*16:02	RAVFLALSAQLLQAR	BAGE	Q13072	4	18	8.32
5864	46	DRB1*16:02	VFLALSAQLLQARLM	BAGE	Q13072	6	20	19.28
5865	47	DRB1*16:02	KLGFKATLPPFMCNK	SSX2	Q16385	60	74	41.89
5866	1	DRB1*16:04	KVYYYRFVIGLRVWQ	TRP2	O75767	206	220	37.18
5867	2	DRB1*16:04	VYYYRFVIGLRVWQW	TRP2	O75767	207	221	47.58
5868	3	DRB1*16:04	YYYRFVIGLRVWQWE	TRP2	O75767	208	222	43.5
5869	4	DRB1*16:04	ESLFRAVITKKVADL	MAGE1	P43355	95	109	30.81
5870	5	DRB1*16:04	ILESLFRAVITKKVA	MAGE1	P43355	93	107	34.19
5871	6	DRB1*16:04	LESLFRAVITKKVAD	MAGE1	P43355	94	108	41.16
5872	7	DRB1*16:04	LFRAVITKKVADLVG	MAGE1	P43355	97	111	44.71
5873	8	DRB1*16:04	SLFRAVITKKVADLV	MAGE1	P43355	96	110	29.92
5874	9	DRB1*16:04	AARAVFLALSAQLLQ	BAGE	Q13072	2	16	29.82
5875	10	DRB1*16:04	ARAVFLALSAQLLQA	BAGE	Q13072	3	17	24.59
5876	11	DRB1*16:04	AVFLALSAQLLQARL	BAGE	Q13072	5	19	27.82
5877	12	DRB1*16:04	RAVFLALSAQLLQAR	BAGE	Q13072	4	18	16.93
5878	13	DRB1*16:04	VFLALSAQLLQARLM	BAGE	Q13072	6	20	42.87
5879	1	DRB1*16:05	KVYYYRFVIGLRVWQ	TRP2	O75767	206	220	22.24
5880	2	DRB1*16:05	LKVYYYRFVIGLRVW	TRP2	O75767	205	219	28.85
5881	3	DRB1*16:05	VYYYRFVIGLRVWQW	TRP2	O75767	207	221	26.4
5882	4	DRB1*16:05	WLKVYYYRFVIGLRV	TRP2	O75767	204	218	32.26
5883	5	DRB1*16:05	YYRFVIGLRVWQWEV	TRP2	O75767	209	223	46.5

5884	6	DRB1*16:05	YYYRFVIGLRVWQWE	TRP2	O75767	208	222	28.04
5885	7	DRB1*16:05	ESLFRAVITKKVADL	MAGE1	P43355	95	109	40.22
5886	8	DRB1*16:05	ILESLFRAVITKKVA	MAGE1	P43355	93	107	45.32
5887	9	DRB1*16:05	SLFRAVITKKVADLV	MAGE1	P43355	96	110	43.91
5888	10	DRB1*16:05	ESLFREALSNKVDEL	MAGE4	P43358	103	117	41.35
5889	11	DRB1*16:05	SLFREALSNKVDELA	MAGE4	P43358	104	118	49.13
5890	12	DRB1*16:05	LKEFTVSGNILTIRL	NY-ESO-1	P78358	123	137	44.06
5891	13	DRB1*16:05	AARAVFLALSAQLLQ	BAGE	Q13072	2	16	16.1
5892	14	DRB1*16:05	ARAVFLALSAQLLQA	BAGE	Q13072	3	17	13.75
5893	15	DRB1*16:05	AVFLALSAQLLQARL	BAGE	Q13072	5	19	16.3
5894	16	DRB1*16:05	MAARAVFLALSAQLL	BAGE	Q13072	1	15	24.73
5895	17	DRB1*16:05	RAVFLALSAQLLQAR	BAGE	Q13072	4	18	11.76
5896	18	DRB1*16:05	VFLALSAQLLQARLM	BAGE	Q13072	6	20	24.39
5897	1	DRB1*16:09	KVYYYRFVIGLRVWQ	TRP2	O75767	206	220	19.39
5898	2	DRB1*16:09	LKVYYYRFVIGLRVW	TRP2	O75767	205	219	27.73
5899	3	DRB1*16:09	VYYYRFVIGLRVWQW	TRP2	O75767	207	221	25.57
5900	4	DRB1*16:09	WLKVYYYRFVIGLRV	TRP2	O75767	204	218	26.84
5901	5	DRB1*16:09	YYRFVIGLRVWQWEV	TRP2	O75767	209	223	31.95
5902	6	DRB1*16:09	YYYRFVIGLRVWQWE	TRP2	O75767	208	222	22.89
5903	7	DRB1*16:09	DKFFAYLTLAKHTIS	Tyrosinase	P14679	132	146	34.14
5904	8	DRB1*16:09	EKDKFFAYLTLAKHT	Tyrosinase	P14679	130	144	38.06
5905	9	DRB1*16:09	KDKFFAYLTLAKHTI	Tyrosinase	P14679	131	145	28.43
5906	10	DRB1*16:09	KFFAYLTLAKHTISS	Tyrosinase	P14679	133	147	36.33
5907	11	DRB1*16:09	PEKDKFFAYLTLAKH	Tyrosinase	P14679	129	143	44.53
5908	12	DRB1*16:09	WMHYYVSMDALLGGS	Tyrosinase	P14679	178	192	46.83
5909	13	DRB1*16:09	IYNYFVWTHYYSVKK	TRP1	P17643	184	198	48.28
5910	14	DRB1*16:09	NYFVWTHYYSVKKTF	TRP1	P17643	186	200	49.93
5911	15	DRB1*16:09	YNYFVWTHYYSVKKT	TRP1	P17643	185	199	43.18
5912	16	DRB1*16:09	ANASFSIALNFPGSQ	PMEL17	P40967	80	94	40.34
5913	17	DRB1*16:09	ASFSIALNFPGSQKV	PMEL17	P40967	82	96	33.44
5914	18	DRB1*16:09	GANASFSIALNFPGS	PMEL17	P40967	79	93	49.43
5915	19	DRB1*16:09	NASFSIALNFPGSQK	PMEL17	P40967	81	95	32.26
5916	20	DRB1*16:09	ARYEFLWGPRALAET	MAGE1	P43355	260	274	38.11
5917	21	DRB1*16:09	CILESLFRAVITKKV	MAGE1	P43355	92	106	36.39
5918	22	DRB1*16:09	DPARYEFLWGPRALA	MAGE1	P43355	258	272	36.78
5919	23	DRB1*16:09	ESLFRAVITKKVADL	MAGE1	P43355	95	109	18.57
5920	24	DRB1*16:09	EYVIKVSARVRFFFP	MAGE1	P43355	281	295	42.89
5921	25	DRB1*16:09	ILESLFRAVITKKVA	MAGE1	P43355	93	107	19.39
5922	26	DRB1*16:09	KVLEYVIKVSARVRF	MAGE1	P43355	278	292	35.31

5923	27	DRB1*16:09	LESLFRAVITKKVAD	MAGE1	P43355	94	108	24.24
5924	28	DRB1*16:09	LEYVIKVSARVRFFF	MAGE1	P43355	280	294	32.69
5925	29	DRB1*16:09	LFRAVITKKVADLVG	MAGE1	P43355	97	111	25.47
5926	30	DRB1*16:09	PARYEFLWGPRALAE	MAGE1	P43355	259	273	38.73
5927	31	DRB1*16:09	SLFRAVITKKVADLV	MAGE1	P43355	96	110	17.72
5928	32	DRB1*16:09	VKVLEYVIKVSARVR	MAGE1	P43355	277	291	42.99
5929	33	DRB1*16:09	VLEYVIKVSARVRFF	MAGE1	P43355	279	293	34.95
5930	34	DRB1*16:09	ARYEFLWGPRALAET	MAGE4	P43358	268	282	38.11
5931	35	DRB1*16:09	ESLFREALSNKVDEL	MAGE4	P43358	103	117	48.25
5932	36	DRB1*16:09	NPARYEFLWGPRALA	MAGE4	P43358	266	280	36.73
5933	37	DRB1*16:09	PARYEFLWGPRALAE	MAGE4	P43358	267	281	38.73
5934	38	DRB1*16:09	LLEFYLAMPFATPME	NY-ESO-1	P78358	87	101	43.88
5935	39	DRB1*16:09	RLLEFYLAMPFATPM	NY-ESO-1	P78358	86	100	46.86
5936	40	DRB1*16:09	AARAVFLALSAQLLQ	BAGE	Q13072	2	16	16.06
5937	41	DRB1*16:09	ARAVFLALSAQLLQA	BAGE	Q13072	3	17	13.76
5938	42	DRB1*16:09	AVFLALSAQLLQARL	BAGE	Q13072	5	19	15
5939	43	DRB1*16:09	MAARAVFLALSAQLL	BAGE	Q13072	1	15	26.79
5940	44	DRB1*16:09	RAVFLALSAQLLQAR	BAGE	Q13072	4	18	10.21
5941	45	DRB1*16:09	VFLALSAQLLQARLM	BAGE	Q13072	6	20	20.13

Table S5. High binding affinity peptide-HLA-II pairs arranged by peptide.

Index	Peptide	Allele	Protein	Antigen	Start	End	IC_{50}
1	AACDQRVLIVRRNLL	DRB1*11:02	P17643	TRP1	120	134	43.42
2	AACDQRVLIVRRNLL	DRB1*11:03	P17643	TRP1	120	134	46.05
3	AACDQRVLIVRRNLL	DRB1*11:04	P17643	TRP1	120	134	29.49
4	AACDQRVLIVRRNLL	DRB1*11:13	P17643	TRP1	120	134	23.87
5	AACDQRVLIVRRNLL	DRB1*11:42	P17643	TRP1	120	134	24.06
6	AACDQRVLIVRRNLL	DRB1*11:46	P17643	TRP1	120	134	29.49
7	AACDQRVLIVRRNLL	DRB1*11:58	P17643	TRP1	120	134	29.49
8	AACDQRVLIVRRNLL	DRB1*11:65	P17643	TRP1	120	134	43.42
9	AACDQRVLIVRRNLL	DRB1*13:01	P17643	TRP1	120	134	43.42
10	AACDQRVLIVRRNLL	DRB1*13:11	P17643	TRP1	120	134	29.49
11	AACDQRVLIVRRNLL	DRB1*13:21	P17643	TRP1	120	134	49.48
12	AACDQRVLIVRRNLL	DRB1*14:01	P17643	TRP1	120	134	22.8
13	AACDQRVLIVRRNLL	DRB1*14:04	P17643	TRP1	120	134	44.06
14	AACDQRVLIVRRNLL	DRB1*14:32	P17643	TRP1	120	134	16.78
15	AACDQRVLIVRRNLL	DRB1*14:54	P17643	TRP1	120	134	22.8
16	AADHRQLQLSISSCL	DRB1*07:01	P78358	NY-ESO-1	139	153	21.95
17	AARAVFLALSAQLLQ	DPB1*33:01	Q13072	BAGE	2	16	36.86
18	AARAVFLALSAQLLQ	DPB1*71:01	Q13072	BAGE	2	16	36.86
19	AARAVFLALSAQLLQ	DRB1*01:01	Q13072	BAGE	2	16	3.09
20	AARAVFLALSAQLLQ	DRB1*01:02	Q13072	BAGE	2	16	22.52
21	AARAVFLALSAQLLQ	DRB1*01:03	Q13072	BAGE	2	16	36.37
22	AARAVFLALSAQLLQ	DRB1*01:11	Q13072	BAGE	2	16	4.86
23	AARAVFLALSAQLLQ	DRB1*01:18	Q13072	BAGE	2	16	2.88
24	AARAVFLALSAQLLQ	DRB1*01:20	Q13072	BAGE	2	16	7.27
25	AARAVFLALSAQLLQ	DRB1*01:24	Q13072	BAGE	2	16	3.64
26	AARAVFLALSAQLLQ	DRB1*01:29	Q13072	BAGE	2	16	4.2
27	AARAVFLALSAQLLQ	DRB1*07:01	Q13072	BAGE	2	16	19.97
28	AARAVFLALSAQLLQ	DRB1*09:01	Q13072	BAGE	2	16	43.55
29	AARAVFLALSAQLLQ	DRB1*10:01	Q13072	BAGE	2	16	8.68
30	AARAVFLALSAQLLQ	DRB1*12:16	Q13072	BAGE	2	16	47.78
31	AARAVFLALSAQLLQ	DRB1*15:01	Q13072	BAGE	2	16	45.47
32	AARAVFLALSAQLLQ	DRB1*15:02	Q13072	BAGE	2	16	23.52
33	AARAVFLALSAQLLQ	DRB1*15:06	Q13072	BAGE	2	16	45.47
34	AARAVFLALSAQLLQ	DRB1*15:15	Q13072	BAGE	2	16	27.08
35	AARAVFLALSAQLLQ	DRB1*16:01	Q13072	BAGE	2	16	20.56
36	AARAVFLALSAQLLQ	DRB1*16:02	Q13072	BAGE	2	16	12.16

37	AARAVFLALSAQLLQ	DRB1*16:04	Q13072	BAGE	2	16	29.82
38	AARAVFLALSAQLLQ	DRB1*16:05	Q13072	BAGE	2	16	16.1
39	AARAVFLALSAQLLQ	DRB1*16:09	Q13072	BAGE	2	16	16.06
40	ACDQRVLIVRRNLLD	DRB1*11:02	P17643	TRP1	121	135	32.47
41	ACDQRVLIVRRNLLD	DRB1*11:03	P17643	TRP1	121	135	34
42	ACDQRVLIVRRNLLD	DRB1*11:04	P17643	TRP1	121	135	24.43
43	ACDQRVLIVRRNLLD	DRB1*11:13	P17643	TRP1	121	135	18.4
44	ACDQRVLIVRRNLLD	DRB1*11:42	P17643	TRP1	121	135	17.73
45	ACDQRVLIVRRNLLD	DRB1*11:46	P17643	TRP1	121	135	24.43
46	ACDQRVLIVRRNLLD	DRB1*11:58	P17643	TRP1	121	135	24.43
47	ACDQRVLIVRRNLLD	DRB1*11:65	P17643	TRP1	121	135	32.47
48	ACDQRVLIVRRNLLD	DRB1*13:01	P17643	TRP1	121	135	32.47
49	ACDQRVLIVRRNLLD	DRB1*13:11	P17643	TRP1	121	135	24.43
50	ACDQRVLIVRRNLLD	DRB1*13:21	P17643	TRP1	121	135	43.08
51	ACDQRVLIVRRNLLD	DRB1*14:01	P17643	TRP1	121	135	28.98
52	ACDQRVLIVRRNLLD	DRB1*14:04	P17643	TRP1	121	135	49.12
53	ACDQRVLIVRRNLLD	DRB1*14:32	P17643	TRP1	121	135	15.85
54	ACDQRVLIVRRNLLD	DRB1*14:54	P17643	TRP1	121	135	28.98
55	ACQLVLHQILKGGSG	DRB1*08:04	P40967	PMEL17	549	563	49.22
56	ACQLVLHQILKGGSG	DRB1*11:03	P40967	PMEL17	549	563	46.57
57	ACQLVLHQILKGGSG	DRB1*11:04	P40967	PMEL17	549	563	20.08
58	ACQLVLHQILKGGSG	DRB1*11:42	P40967	PMEL17	549	563	30.74
59	ACQLVLHQILKGGSG	DRB1*11:46	P40967	PMEL17	549	563	20.08
60	ACQLVLHQILKGGSG	DRB1*11:58	P40967	PMEL17	549	563	20.08
61	ACQLVLHQILKGGSG	DRB1*13:11	P40967	PMEL17	549	563	20.08
62	ADHRQLQLSISSCLQ	DRB1*01:01	P78358	NY-ESO-1	140	154	31.72
63	ADHRQLQLSISSCLQ	DRB1*01:18	P78358	NY-ESO-1	140	154	28.35
64	ADHRQLQLSISSCLQ	DRB1*01:20	P78358	NY-ESO-1	140	154	27.91
65	ADHRQLQLSISSCLQ	DRB1*07:01	P78358	NY-ESO-1	140	154	15.82
66	ADHRQLQLSISSCLQ	DRB1*09:01	P78358	NY-ESO-1	140	154	42.19
67	ADLVGFLLLKYRARE	DPB1*15:01	P43355	MAGE1	107	121	37.55
68	ADLVGFLLLKYRARE	DPB1*33:01	P43355	MAGE1	107	121	24.25
69	ADLVGFLLLKYRARE	DPB1*71:01	P43355	MAGE1	107	121	24.25
70	ADLVGFLLLKYRARE	DRB1*01:18	P43355	MAGE1	107	121	44.56
71	ADLVGFLLLKYRARE	DRB1*01:20	P43355	MAGE1	107	121	46.66
72	ADLVGFLLLKYRARE	DRB1*11:01	P43355	MAGE1	107	121	34.07
73	ADLVGFLLLKYRARE	DRB1*11:02	P43355	MAGE1	107	121	40.53
74	ADLVGFLLLKYRARE	DRB1*11:03	P43355	MAGE1	107	121	31.51
75	ADLVGFLLLKYRARE	DRB1*11:04	P43355	MAGE1	107	121	22.28
76	ADLVGFLLLKYRARE	DRB1*11:10	P43355	MAGE1	107	121	34.07

77	ADLVGFLLLKYRARE	DRB1*11:12	P43355	MAGE1	107	121	34.07
78	ADLVGFLLLKYRARE	DRB1*11:13	P43355	MAGE1	107	121	38.99
79	ADLVGFLLLKYRARE	DRB1*11:28	P43355	MAGE1	107	121	34.07
80	ADLVGFLLLKYRARE	DRB1*11:29	P43355	MAGE1	107	121	34.07
81	ADLVGFLLLKYRARE	DRB1*11:42	P43355	MAGE1	107	121	24.87
82	ADLVGFLLLKYRARE	DRB1*11:46	P43355	MAGE1	107	121	22.28
83	ADLVGFLLLKYRARE	DRB1*11:49	P43355	MAGE1	107	121	34.07
84	ADLVGFLLLKYRARE	DRB1*11:58	P43355	MAGE1	107	121	22.28
85	ADLVGFLLLKYRARE	DRB1*11:62	P43355	MAGE1	107	121	34.07
86	ADLVGFLLLKYRARE	DRB1*11:65	P43355	MAGE1	107	121	40.53
87	ADLVGFLLLKYRARE	DRB1*11:74	P43355	MAGE1	107	121	34.07
88	ADLVGFLLLKYRARE	DRB1*12:03	P43355	MAGE1	107	121	41.32
89	ADLVGFLLLKYRARE	DRB1*12:16	P43355	MAGE1	107	121	43.73
90	ADLVGFLLLKYRARE	DRB1*13:01	P43355	MAGE1	107	121	40.53
91	ADLVGFLLLKYRARE	DRB1*13:05	P43355	MAGE1	107	121	34.07
92	ADLVGFLLLKYRARE	DRB1*13:11	P43355	MAGE1	107	121	22.28
93	ADLVGFLLLKYRARE	DRB1*13:14	P43355	MAGE1	107	121	34.07
94	ADLVGFLLLKYRARE	DRB1*13:21	P43355	MAGE1	107	121	23.8
95	ADLVGFLLLKYRARE	DRB1*13:50	P43355	MAGE1	107	121	34.07
96	AEMLERVIKNYKRCF	DRB1*11:02	P43358	MAGE4	134	148	43.68
97	AEMLERVIKNYKRCF	DRB1*11:03	P43358	MAGE4	134	148	36.93
98	AEMLERVIKNYKRCF	DRB1*11:04	P43358	MAGE4	134	148	47.43
99	AEMLERVIKNYKRCF	DRB1*11:42	P43358	MAGE4	134	148	35.25
100	AEMLERVIKNYKRCF	DRB1*11:46	P43358	MAGE4	134	148	47.43
101	AEMLERVIKNYKRCF	DRB1*11:58	P43358	MAGE4	134	148	47.43
102	AEMLERVIKNYKRCF	DRB1*11:65	P43358	MAGE4	134	148	43.68
103	AEMLERVIKNYKRCF	DRB1*13:01	P43358	MAGE4	134	148	43.68
104	AEMLERVIKNYKRCF	DRB1*13:11	P43358	MAGE4	134	148	47.43
105	AEMLESVIKNYKHCF	DRB1*11:42	P43355	MAGE1	126	140	45.61
106	AESLFREALSNKVDE	DRB1*01:01	P43358	MAGE4	102	116	5.74
107	AESLFREALSNKVDE	DRB1*01:11	P43358	MAGE4	102	116	15.33
108	AESLFREALSNKVDE	DRB1*01:18	P43358	MAGE4	102	116	6.51
109	AESLFREALSNKVDE	DRB1*01:20	P43358	MAGE4	102	116	22.1
110	AESLFREALSNKVDE	DRB1*01:24	P43358	MAGE4	102	116	13.79
111	AESLFREALSNKVDE	DRB1*01:29	P43358	MAGE4	102	116	12.21
112	AESLFREALSNKVDE	DRB1*07:01	P43358	MAGE4	102	116	9.86
113	AESLFREALSNKVDE	DRB1*09:01	P43358	MAGE4	102	116	15.34
114	AESLFREALSNKVDE	DRB1*10:01	P43358	MAGE4	102	116	17.56
115	AESLFREALSNKVDE	DRB1*16:01	P43358	MAGE4	102	116	48.38
116	AESLFREALSNKVDE	DRB1*16:02	P43358	MAGE4	102	116	25.03

117	AETSYVKVLEHVVRV	DRB1*01:01	P43358	MAGE4	280	294	42.67
118	AETSYVKVLEHVVRV	DRB1*01:18	P43358	MAGE4	280	294	27.9
119	AETSYVKVLEYVIKV	DRB1*01:18	P43355	MAGE1	272	286	31.69
120	AEVSIVVLSGTTAAQ	DRB1*01:01	P40967	PMEL17	403	417	9.68
121	AEVSIVVLSGTTAAQ	DRB1*01:02	P40967	PMEL17	403	417	45.95
122	AEVSIVVLSGTTAAQ	DRB1*01:11	P40967	PMEL17	403	417	23.79
123	AEVSIVVLSGTTAAQ	DRB1*01:18	P40967	PMEL17	403	417	9.34
124	AEVSIVVLSGTTAAQ	DRB1*01:20	P40967	PMEL17	403	417	10.6
125	AEVSIVVLSGTTAAQ	DRB1*01:24	P40967	PMEL17	403	417	20.78
126	AEVSIVVLSGTTAAQ	DRB1*01:29	P40967	PMEL17	403	417	20.55
127	AEVSIVVLSGTTAAQ	DRB1*04:04	P40967	PMEL17	403	417	43.28
128	AEVSIVVLSGTTAAQ	DRB1*10:01	P40967	PMEL17	403	417	47.54
129	AFLTWHRYHLLRLEK	DPB1*02:01	P17643	TRP1	219	233	15.2
130	AFLTWHRYHLLRLEK	DPB1*02:02	P17643	TRP1	219	233	14.6
131	AFLTWHRYHLLRLEK	DPB1*16:01	P17643	TRP1	219	233	46.45
132	AFLTWHRYHLLRLEK	DPB1*33:01	P17643	TRP1	219	233	7.44
133	AFLTWHRYHLLRLEK	DPB1*41:01	P17643	TRP1	219	233	35.44
134	AFLTWHRYHLLRLEK	DPB1*46:01	P17643	TRP1	219	233	15.2
135	AFLTWHRYHLLRLEK	DPB1*47:01	P17643	TRP1	219	233	14.6
136	AFLTWHRYHLLRLEK	DPB1*71:01	P17643	TRP1	219	233	7.44
137	AFLTWHRYHLLRLEK	DPB1*81:01	P17643	TRP1	219	233	15.2
138	AFLTWHRYHLLRLEK	DRB1*11:02	P17643	TRP1	219	233	44.85
139	AFLTWHRYHLLRLEK	DRB1*11:03	P17643	TRP1	219	233	48.25
140	AFLTWHRYHLLRLEK	DRB1*11:65	P17643	TRP1	219	233	44.85
141	AFLTWHRYHLLRLEK	DRB1*13:01	P17643	TRP1	219	233	44.85
142	AFLTWHRYHLLRLEK	DRB1*15:03	P17643	TRP1	219	233	47.24
143	AFLTWHRYHLLRLEK	DRB1*15:15	P17643	TRP1	219	233	41.33
144	AFTITDQVPFSVSVS	DRB1*01:20	P40967	PMEL17	206	220	49.22
145	AFVAMVTTACHEFFE	DRB1*01:01	P04271	S100	74	88	31.14
146	AFVAMVTTACHEFFE	DRB1*01:18	P04271	S100	74	88	30.76
147	AFVAMVTTACHEFFE	DRB1*04:04	P04271	S100	74	88	43.34
148	AFVAMVTTACHEFFE	DRB1*10:01	P04271	S100	74	88	28.91
149	AFVDSIFEQWLRRHR	DPB1*04:01	P14679	Tyrosinase	391	405	44.17
150	AFVDSIFEQWLRRHR	DPB1*126:01	P14679	Tyrosinase	391	405	44.17
151	AFVDSIFEQWLRRHR	DPB1*23:01	P14679	Tyrosinase	391	405	44.17
152	AFVDSIFEQWLRRHR	DPB1*33:01	P14679	Tyrosinase	391	405	24.56
153	AFVDSIFEQWLRRHR	DPB1*39:01	P14679	Tyrosinase	391	405	44.17
154	AFVDSIFEQWLRRHR	DPB1*71:01	P14679	Tyrosinase	391	405	24.56
155	AFVDSIFEQWLRRHR	DPB1*72:01	P14679	Tyrosinase	391	405	46.99
156	AGLVSLLCRHKRKQL	DRB1*11:02	P14679	Tyrosinase	493	507	19.74

157	AGLVSLLCRHKRKQL	DRB1*11:03	P14679	Tyrosinase	493	507	12.77
158	AGLVSLLCRHKRKQL	DRB1*11:04	P14679	Tyrosinase	493	507	20.85
159	AGLVSLLCRHKRKQL	DRB1*11:13	P14679	Tyrosinase	493	507	46.93
160	AGLVSLLCRHKRKQL	DRB1*11:42	P14679	Tyrosinase	493	507	22.26
161	AGLVSLLCRHKRKQL	DRB1*11:46	P14679	Tyrosinase	493	507	20.85
162	AGLVSLLCRHKRKQL	DRB1*11:58	P14679	Tyrosinase	493	507	20.85
163	AGLVSLLCRHKRKQL	DRB1*11:65	P14679	Tyrosinase	493	507	19.74
164	AGLVSLLCRHKRKQL	DRB1*11:84	P14679	Tyrosinase	493	507	44.98
165	AGLVSLLCRHKRKQL	DRB1*13:01	P14679	Tyrosinase	493	507	19.74
166	AGLVSLLCRHKRKQL	DRB1*13:11	P14679	Tyrosinase	493	507	20.85
167	AHFLLRKYRAKELVT	DRB1*01:18	P43358	MAGE4	118	132	44.84
168	AHFLLRKYRAKELVT	DRB1*01:20	P43358	MAGE4	118	132	38.78
169	AHFLLRKYRAKELVT	DRB1*11:01	P43358	MAGE4	118	132	42.25
170	AHFLLRKYRAKELVT	DRB1*11:02	P43358	MAGE4	118	132	35.84
171	AHFLLRKYRAKELVT	DRB1*11:03	P43358	MAGE4	118	132	21.64
172	AHFLLRKYRAKELVT	DRB1*11:04	P43358	MAGE4	118	132	26.1
173	AHFLLRKYRAKELVT	DRB1*11:10	P43358	MAGE4	118	132	42.25
174	AHFLLRKYRAKELVT	DRB1*11:12	P43358	MAGE4	118	132	42.25
175	AHFLLRKYRAKELVT	DRB1*11:28	P43358	MAGE4	118	132	42.25
176	AHFLLRKYRAKELVT	DRB1*11:29	P43358	MAGE4	118	132	42.25
177	AHFLLRKYRAKELVT	DRB1*11:42	P43358	MAGE4	118	132	35.07
178	AHFLLRKYRAKELVT	DRB1*11:46	P43358	MAGE4	118	132	26.1
179	AHFLLRKYRAKELVT	DRB1*11:49	P43358	MAGE4	118	132	42.25
180	AHFLLRKYRAKELVT	DRB1*11:58	P43358	MAGE4	118	132	26.1
181	AHFLLRKYRAKELVT	DRB1*11:62	P43358	MAGE4	118	132	42.25
182	AHFLLRKYRAKELVT	DRB1*11:65	P43358	MAGE4	118	132	35.84
183	AHFLLRKYRAKELVT	DRB1*11:74	P43358	MAGE4	118	132	42.25
184	AHFLLRKYRAKELVT	DRB1*13:01	P43358	MAGE4	118	132	35.84
185	AHFLLRKYRAKELVT	DRB1*13:05	P43358	MAGE4	118	132	42.25
186	AHFLLRKYRAKELVT	DRB1*13:11	P43358	MAGE4	118	132	26.1
187	AHFLLRKYRAKELVT	DRB1*13:14	P43358	MAGE4	118	132	42.25
188	AHFLLRKYRAKELVT	DRB1*13:21	P43358	MAGE4	118	132	28.14
189	AHFLLRKYRAKELVT	DRB1*13:50	P43358	MAGE4	118	132	42.25
190	AHFLLRKYRAKELVT	DRB1*15:01	P43358	MAGE4	118	132	14.28
191	AHFLLRKYRAKELVT	DRB1*15:03	P43358	MAGE4	118	132	23.85
192	AHFLLRKYRAKELVT	DRB1*15:06	P43358	MAGE4	118	132	14.28
193	AHFLLRKYRAKELVT	DRB1*15:07	P43358	MAGE4	118	132	30.41
194	AHFLLRKYRAKELVT	DRB1*15:15	P43358	MAGE4	118	132	29.66
195	AHFLLRKYRAKELVT	DRB1*15:37	P43358	MAGE4	118	132	38.82
196	AHLFLNGTGGQTHLS	DRB1*01:01	P17643	TRP1	380	394	44.3

197	AKHTISSDYVIPIGT	DRB1*03:01	P14679	Tyrosinase	141	155	42.94
198	AKHTISSDYVIPIGT	DRB1*03:04	P14679	Tyrosinase	141	155	42.94
199	AKHTISSDYVIPIGT	DRB1*03:11	P14679	Tyrosinase	141	155	23.51
200	AKHTISSDYVIPIGT	DRB1*03:13	P14679	Tyrosinase	141	155	42.94
201	ALAETSYVKVLEYVI	DPB1*33:01	P43355	MAGE1	270	284	42.04
202	ALAETSYVKVLEYVI	DPB1*71:01	P43355	MAGE1	270	284	42.04
203	ALGLVCVQAATSSSS	DRB1*01:01	P43355	MAGE1	22	36	30.65
204	ALGLVCVQAATSSSS	DRB1*01:18	P43355	MAGE1	22	36	29.18
205	ALGLVCVQAATSSSS	DRB1*01:20	P43355	MAGE1	22	36	26.14
206	ALGLVCVQAATSSSS	DRB1*10:01	P43355	MAGE1	22	36	39.53
207	ALHIYMNGTMSQVQG	DRB1*01:01	P14679	Tyrosinase	365	379	31.54
208	ALHIYMNGTMSQVQG	DRB1*01:18	P14679	Tyrosinase	365	379	31.88
209	ALHIYMNGTMSQVQG	DRB1*01:20	P14679	Tyrosinase	365	379	40.68
210	ALHIYMNGTMSQVQG	DRB1*11:14	P14679	Tyrosinase	365	379	24.22
211	ALHIYMNGTMSQVQG	DRB1*13:02	P14679	Tyrosinase	365	379	24.22
212	ALHIYMNGTMSQVQG	DRB1*13:23	P14679	Tyrosinase	365	379	24.22
213	ALHIYMNGTMSQVQG	DRB1*13:96	P14679	Tyrosinase	365	379	47.79
214	ALHIYMNGTMSQVQG	DRB1*13:97	P14679	Tyrosinase	365	379	24.22
215	ALHIYMNGTMSQVQG	DRB1*15:01	P14679	Tyrosinase	365	379	37.02
216	ALHIYMNGTMSQVQG	DRB1*15:06	P14679	Tyrosinase	365	379	37.02
217	ALIFGTASYLIRARR	DPB1*33:01	P17643	TRP1	491	505	38.41
218	ALIFGTASYLIRARR	DPB1*71:01	P17643	TRP1	491	505	38.41
219	ALIFGTASYLIRARR	DRB1*01:01	P17643	TRP1	491	505	17.3
220	ALIFGTASYLIRARR	DRB1*01:02	P17643	TRP1	491	505	42.88
221	ALIFGTASYLIRARR	DRB1*01:11	P17643	TRP1	491	505	38.45
222	ALIFGTASYLIRARR	DRB1*01:18	P17643	TRP1	491	505	13.82
223	ALIFGTASYLIRARR	DRB1*01:20	P17643	TRP1	491	505	14.4
224	ALIFGTASYLIRARR	DRB1*01:24	P17643	TRP1	491	505	29.39
225	ALIFGTASYLIRARR	DRB1*01:29	P17643	TRP1	491	505	25.63
226	ALIFGTASYLIRARR	DRB1*07:01	P17643	TRP1	491	505	9.91
227	ALIFGTASYLIRARR	DRB1*09:01	P17643	TRP1	491	505	43.03
228	ALIFGTASYLIRARR	DRB1*11:13	P17643	TRP1	491	505	42.55
229	ALIFGTASYLIRARR	DRB1*11:42	P17643	TRP1	491	505	40.56
230	ALIFGTASYLIRARR	DRB1*15:01	P17643	TRP1	491	505	29.58
231	ALIFGTASYLIRARR	DRB1*15:06	P17643	TRP1	491	505	29.58
232	ALIFGTASYLIRARR	DRB1*15:07	P17643	TRP1	491	505	47.47
233	ALLAGLVSLLCRHKR	DRB1*01:01	P14679	Tyrosinase	490	504	38.99
234	ALLAGLVSLLCRHKR	DRB1*01:18	P14679	Tyrosinase	490	504	28.62
235	ALLAGLVSLLCRHKR	DRB1*01:20	P14679	Tyrosinase	490	504	23.97
236	ALLAGLVSLLCRHKR	DRB1*11:02	P14679	Tyrosinase	490	504	49.75

237	ALLAGLVSLLCRHKR	DRB1*11:03	P14679	Tyrosinase	490	504	38.43
238	ALLAGLVSLLCRHKR	DRB1*11:04	P14679	Tyrosinase	490	504	44.71
239	ALLAGLVSLLCRHKR	DRB1*11:42	P14679	Tyrosinase	490	504	41.77
240	ALLAGLVSLLCRHKR	DRB1*11:46	P14679	Tyrosinase	490	504	44.71
241	ALLAGLVSLLCRHKR	DRB1*11:58	P14679	Tyrosinase	490	504	44.71
242	ALLAGLVSLLCRHKR	DRB1*11:65	P14679	Tyrosinase	490	504	49.75
243	ALLAGLVSLLCRHKR	DRB1*13:01	P14679	Tyrosinase	490	504	49.75
244	ALLAGLVSLLCRHKR	DRB1*13:11	P14679	Tyrosinase	490	504	44.71
245	ALLAVGATKVPRNQD	DRB1*01:01	P40967	PMEL17	17	31	12.83
246	ALLAVGATKVPRNQD	DRB1*01:02	P40967	PMEL17	17	31	33.5
247	ALLAVGATKVPRNQD	DRB1*01:11	P40967	PMEL17	17	31	41.69
248	ALLAVGATKVPRNQD	DRB1*01:18	P40967	PMEL17	17	31	13.2
249	ALLAVGATKVPRNQD	DRB1*01:20	P40967	PMEL17	17	31	13.76
250	ALLAVGATKVPRNQD	DRB1*01:24	P40967	PMEL17	17	31	30.54
251	ALLAVGATKVPRNQD	DRB1*01:29	P40967	PMEL17	17	31	29.26
252	ALLAVGATKVPRNQD	DRB1*07:01	P40967	PMEL17	17	31	43.16
253	ALSAQLLQARLMKEE	DRB1*01:01	Q13072	BAGE	9	23	18.8
254	ALSAQLLQARLMKEE	DRB1*01:18	Q13072	BAGE	9	23	16.88
255	ALSAQLLQARLMKEE	DRB1*01:20	Q13072	BAGE	9	23	13.77
256	ALSAQLLQARLMKEE	DRB1*01:29	Q13072	BAGE	9	23	42.28
257	AMTKLGFKATLPPFM	DRB1*01:01	Q16385	SSX2	57	71	29.73
258	AMTKLGFKATLPPFM	DRB1*01:18	Q16385	SSX2	57	71	29.04
259	AMTKLGFKATLPPFM	DRB1*07:01	Q16385	SSX2	57	71	14.22
260	AMTKLGFKATLPPFM	DRB1*09:01	Q16385	SSX2	57	71	24.36
261	AMTKLGFKATLPPFM	DRB1*10:01	Q16385	SSX2	57	71	24.55
262	AMVGAVLTALLAGLV	DRB1*01:01	P14679	Tyrosinase	482	496	24
263	AMVGAVLTALLAGLV	DRB1*01:18	P14679	Tyrosinase	482	496	19.87
264	AMVGAVLTALLAGLV	DRB1*01:20	P14679	Tyrosinase	482	496	21.4
265	AMVGAVLTALLAGLV	DRB1*01:24	P14679	Tyrosinase	482	496	42.14
266	AMVGAVLTALLAGLV	DRB1*01:29	P14679	Tyrosinase	482	496	36.8
267	ANASFSIALNFPGSQ	DRB1*01:01	P40967	PMEL17	80	94	11.1
268	ANASFSIALNFPGSQ	DRB1*01:11	P40967	PMEL17	80	94	42.31
269	ANASFSIALNFPGSQ	DRB1*01:18	P40967	PMEL17	80	94	11.53
270	ANASFSIALNFPGSQ	DRB1*01:20	P40967	PMEL17	80	94	30.12
271	ANASFSIALNFPGSQ	DRB1*01:24	P40967	PMEL17	80	94	27.76
272	ANASFSIALNFPGSQ	DRB1*01:29	P40967	PMEL17	80	94	27.26
273	ANASFSIALNFPGSQ	DRB1*04:01	P40967	PMEL17	80	94	16.57
274	ANASFSIALNFPGSQ	DRB1*04:04	P40967	PMEL17	80	94	34.62
275	ANASFSIALNFPGSQ	DRB1*04:05	P40967	PMEL17	80	94	31.75
276	ANASFSIALNFPGSQ	DRB1*04:08	P40967	PMEL17	80	94	16.92

277	ANASFSIALNFPGSQ	DRB1*09:01	P40967	PMEL17	80	94	44.33
278	ANASFSIALNFPGSQ	DRB1*10:01	P40967	PMEL17	80	94	12.86
279	ANASFSIALNFPGSQ	DRB1*11:01	P40967	PMEL17	80	94	49.44
280	ANASFSIALNFPGSQ	DRB1*11:10	P40967	PMEL17	80	94	49.44
281	ANASFSIALNFPGSQ	DRB1*11:12	P40967	PMEL17	80	94	49.44
282	ANASFSIALNFPGSQ	DRB1*11:28	P40967	PMEL17	80	94	49.44
283	ANASFSIALNFPGSQ	DRB1*11:29	P40967	PMEL17	80	94	49.44
284	ANASFSIALNFPGSQ	DRB1*11:49	P40967	PMEL17	80	94	49.44
285	ANASFSIALNFPGSQ	DRB1*11:62	P40967	PMEL17	80	94	49.44
286	ANASFSIALNFPGSQ	DRB1*11:74	P40967	PMEL17	80	94	49.44
287	ANASFSIALNFPGSQ	DRB1*13:05	P40967	PMEL17	80	94	49.44
288	ANASFSIALNFPGSQ	DRB1*13:14	P40967	PMEL17	80	94	49.44
289	ANASFSIALNFPGSQ	DRB1*13:50	P40967	PMEL17	80	94	49.44
290	ANASFSIALNFPGSQ	DRB1*15:15	P40967	PMEL17	80	94	40.83
291	ANASFSIALNFPGSQ	DRB1*16:01	P40967	PMEL17	80	94	33.47
292	ANASFSIALNFPGSQ	DRB1*16:02	P40967	PMEL17	80	94	28.41
293	ANASFSIALNFPGSQ	DRB1*16:09	P40967	PMEL17	80	94	40.34
294	ANDPIFLLHHAFVDS	DPB1*02:01	P14679	Tyrosinase	381	395	43.78
295	ANDPIFLLHHAFVDS	DPB1*02:02	P14679	Tyrosinase	381	395	42.84
296	ANDPIFLLHHAFVDS	DPB1*33:01	P14679	Tyrosinase	381	395	18.06
297	ANDPIFLLHHAFVDS	DPB1*46:01	P14679	Tyrosinase	381	395	43.78
298	ANDPIFLLHHAFVDS	DPB1*47:01	P14679	Tyrosinase	381	395	42.84
299	ANDPIFLLHHAFVDS	DPB1*71:01	P14679	Tyrosinase	381	395	18.06
300	ANDPIFLLHHAFVDS	DPB1*81:01	P14679	Tyrosinase	381	395	43.78
301	ANDPIFLLHHAFVDS	DRB1*01:18	P14679	Tyrosinase	381	395	38.25
302	APEKDKFFAYLTLAK	DPB1*33:01	P14679	Tyrosinase	128	142	32.56
303	APEKDKFFAYLTLAK	DPB1*71:01	P14679	Tyrosinase	128	142	32.56
304	APPAYEKLSAEQSPP	DRB1*01:01	Q16655	MELAN_A	100	114	48.74
305	APPAYEKLSAEQSPP	DRB1*10:01	Q16655	MELAN_A	100	114	48.28
306	AQVVLQAAIPLTSCG	DRB1*01:01	P40967	PMEL17	288	302	8.19
307	AQVVLQAAIPLTSCG	DRB1*01:02	P40967	PMEL17	288	302	18.38
308	AQVVLQAAIPLTSCG	DRB1*01:11	P40967	PMEL17	288	302	18.79
309	AQVVLQAAIPLTSCG	DRB1*01:18	P40967	PMEL17	288	302	8.22
310	AQVVLQAAIPLTSCG	DRB1*01:20	P40967	PMEL17	288	302	7.41
311	AQVVLQAAIPLTSCG	DRB1*01:24	P40967	PMEL17	288	302	15.16
312	AQVVLQAAIPLTSCG	DRB1*01:29	P40967	PMEL17	288	302	17.87
313	AQVVLQAAIPLTSCG	DRB1*10:01	P40967	PMEL17	288	302	21.34
314	AQVVLQAAIPLTSCG	DRB1*11:14	P40967	PMEL17	288	302	43.39
315	AQVVLQAAIPLTSCG	DRB1*13:02	P40967	PMEL17	288	302	43.39
316	AQVVLQAAIPLTSCG	DRB1*13:23	P40967	PMEL17	288	302	43.39

317	AQVVLQAAIPLTSCG	DRB1*13:97	P40967	PMEL17	288	302	43.39
318	ARAVFLALSAQLLQA	DPB1*33:01	Q13072	BAGE	3	17	34.06
319	ARAVFLALSAQLLQA	DPB1*71:01	Q13072	BAGE	3	17	34.06
320	ARAVFLALSAQLLQA	DRB1*01:01	Q13072	BAGE	3	17	2.96
321	ARAVFLALSAQLLQA	DRB1*01:02	Q13072	BAGE	3	17	19.72
322	ARAVFLALSAQLLQA	DRB1*01:03	Q13072	BAGE	3	17	28.81
323	ARAVFLALSAQLLQA	DRB1*01:11	Q13072	BAGE	3	17	4.46
324	ARAVFLALSAQLLQA	DRB1*01:18	Q13072	BAGE	3	17	2.73
325	ARAVFLALSAQLLQA	DRB1*01:20	Q13072	BAGE	3	17	6.56
326	ARAVFLALSAQLLQA	DRB1*01:24	Q13072	BAGE	3	17	3.42
327	ARAVFLALSAQLLQA	DRB1*01:29	Q13072	BAGE	3	17	3.72
328	ARAVFLALSAQLLQA	DRB1*07:01	Q13072	BAGE	3	17	19.51
329	ARAVFLALSAQLLQA	DRB1*09:01	Q13072	BAGE	3	17	41.19
330	ARAVFLALSAQLLQA	DRB1*10:01	Q13072	BAGE	3	17	8.02
331	ARAVFLALSAQLLQA	DRB1*11:08	Q13072	BAGE	3	17	44.86
332	ARAVFLALSAQLLQA	DRB1*12:16	Q13072	BAGE	3	17	46.03
333	ARAVFLALSAQLLQA	DRB1*14:32	Q13072	BAGE	3	17	49.8
334	ARAVFLALSAQLLQA	DRB1*15:01	Q13072	BAGE	3	17	35.58
335	ARAVFLALSAQLLQA	DRB1*15:02	Q13072	BAGE	3	17	19.33
336	ARAVFLALSAQLLQA	DRB1*15:06	Q13072	BAGE	3	17	35.58
337	ARAVFLALSAQLLQA	DRB1*15:15	Q13072	BAGE	3	17	22.79
338	ARAVFLALSAQLLQA	DRB1*16:01	Q13072	BAGE	3	17	17.84
339	ARAVFLALSAQLLQA	DRB1*16:02	Q13072	BAGE	3	17	10.51
340	ARAVFLALSAQLLQA	DRB1*16:04	Q13072	BAGE	3	17	24.59
341	ARAVFLALSAQLLQA	DRB1*16:05	Q13072	BAGE	3	17	13.75
342	ARAVFLALSAQLLQA	DRB1*16:09	Q13072	BAGE	3	17	13.76
343	ARLMKEESPVVSWRL	DRB1*01:20	Q13072	BAGE	17	31	43.74
344	ARVRFFFPSLREAAL	DPB1*33:01	P43355	MAGE1	288	302	39.02
345	ARVRFFFPSLREAAL	DPB1*71:01	P43355	MAGE1	288	302	39.02
346	ARVRFFFPSLREAAL	DRB1*01:01	P43355	MAGE1	288	302	42.38
347	ARVRFFFPSLREAAL	DRB1*01:18	P43355	MAGE1	288	302	38.57
348	ARVRFFFPSLREAAL	DRB1*01:20	P43355	MAGE1	288	302	32.67
349	ARVRFFFPSLREAAL	DRB1*10:01	P43355	MAGE1	288	302	30.32
350	ARVRFFFPSLREAAL	DRB1*11:01	P43355	MAGE1	288	302	21.89
351	ARVRFFFPSLREAAL	DRB1*11:02	P43355	MAGE1	288	302	48.15
352	ARVRFFFPSLREAAL	DRB1*11:03	P43355	MAGE1	288	302	42.45
353	ARVRFFFPSLREAAL	DRB1*11:04	P43355	MAGE1	288	302	42.61
354	ARVRFFFPSLREAAL	DRB1*11:08	P43355	MAGE1	288	302	44.37
355	ARVRFFFPSLREAAL	DRB1*11:10	P43355	MAGE1	288	302	21.89
356	ARVRFFFPSLREAAL	DRB1*11:12	P43355	MAGE1	288	302	21.89

357	ARVRFFFPSLREAAL	DRB1*11:13	P43355	MAGE1	288	302	47.26
358	ARVRFFFPSLREAAL	DRB1*11:28	P43355	MAGE1	288	302	21.89
359	ARVRFFFPSLREAAL	DRB1*11:29	P43355	MAGE1	288	302	21.89
360	ARVRFFFPSLREAAL	DRB1*11:37	P43355	MAGE1	288	302	35.21
361	ARVRFFFPSLREAAL	DRB1*11:42	P43355	MAGE1	288	302	41.02
362	ARVRFFFPSLREAAL	DRB1*11:46	P43355	MAGE1	288	302	42.61
363	ARVRFFFPSLREAAL	DRB1*11:49	P43355	MAGE1	288	302	21.89
364	ARVRFFFPSLREAAL	DRB1*11:58	P43355	MAGE1	288	302	42.61
365	ARVRFFFPSLREAAL	DRB1*11:62	P43355	MAGE1	288	302	21.89
366	ARVRFFFPSLREAAL	DRB1*11:65	P43355	MAGE1	288	302	48.15
367	ARVRFFFPSLREAAL	DRB1*11:74	P43355	MAGE1	288	302	21.89
368	ARVRFFFPSLREAAL	DRB1*13:01	P43355	MAGE1	288	302	48.15
369	ARVRFFFPSLREAAL	DRB1*13:05	P43355	MAGE1	288	302	21.89
370	ARVRFFFPSLREAAL	DRB1*13:07	P43355	MAGE1	288	302	35.21
371	ARVRFFFPSLREAAL	DRB1*13:11	P43355	MAGE1	288	302	42.61
372	ARVRFFFPSLREAAL	DRB1*13:14	P43355	MAGE1	288	302	21.89
373	ARVRFFFPSLREAAL	DRB1*13:21	P43355	MAGE1	288	302	20.12
374	ARVRFFFPSLREAAL	DRB1*13:50	P43355	MAGE1	288	302	21.89
375	ARVRFFFPSLREAAL	DRB1*15:01	P43355	MAGE1	288	302	43.77
376	ARVRFFFPSLREAAL	DRB1*15:03	P43355	MAGE1	288	302	44.07
377	ARVRFFFPSLREAAL	DRB1*15:06	P43355	MAGE1	288	302	43.77
378	ARVRIAYPSLREAAL	DRB1*01:20	P43358	MAGE4	296	310	31.08
379	ARVRIAYPSLREAAL	DRB1*03:11	P43358	MAGE4	296	310	47.56
380	ARVRIAYPSLREAAL	DRB1*11:02	P43358	MAGE4	296	310	40.7
381	ARVRIAYPSLREAAL	DRB1*11:13	P43358	MAGE4	296	310	48.02
382	ARVRIAYPSLREAAL	DRB1*11:42	P43358	MAGE4	296	310	35.02
383	ARVRIAYPSLREAAL	DRB1*11:65	P43358	MAGE4	296	310	40.7
384	ARVRIAYPSLREAAL	DRB1*13:01	P43358	MAGE4	296	310	40.7
385	ARVRIAYPSLREAAL	DRB1*14:06	P43358	MAGE4	296	310	45.97
386	ARYEFLWGPRALAET	DRB1*01:01	P43355	MAGE1	260	274	8.13
387	ARYEFLWGPRALAET	DRB1*01:01	P43358	MAGE4	268	282	8.13
388	ARYEFLWGPRALAET	DRB1*01:11	P43355	MAGE1	260	274	28.01
389	ARYEFLWGPRALAET	DRB1*01:11	P43358	MAGE4	268	282	28.01
390	ARYEFLWGPRALAET	DRB1*01:18	P43355	MAGE1	260	274	8.09
391	ARYEFLWGPRALAET	DRB1*01:18	P43358	MAGE4	268	282	8.09
392	ARYEFLWGPRALAET	DRB1*01:20	P43355	MAGE1	260	274	23.93
393	ARYEFLWGPRALAET	DRB1*01:20	P43358	MAGE4	268	282	23.93
394	ARYEFLWGPRALAET	DRB1*01:24	P43355	MAGE1	260	274	20.15
395	ARYEFLWGPRALAET	DRB1*01:24	P43358	MAGE4	268	282	20.15
396	ARYEFLWGPRALAET	DRB1*01:29	P43355	MAGE1	260	274	18.01

397	ARYEFLWGPRALAET	DRB1*01:29	P43358	MAGE4	268	282	18.01
398	ARYEFLWGPRALAET	DRB1*10:01	P43355	MAGE1	260	274	18.69
399	ARYEFLWGPRALAET	DRB1*10:01	P43358	MAGE4	268	282	18.69
400	ARYEFLWGPRALAET	DRB1*11:01	P43355	MAGE1	260	274	33.84
401	ARYEFLWGPRALAET	DRB1*11:01	P43358	MAGE4	268	282	33.84
402	ARYEFLWGPRALAET	DRB1*11:10	P43355	MAGE1	260	274	33.84
403	ARYEFLWGPRALAET	DRB1*11:10	P43358	MAGE4	268	282	33.84
404	ARYEFLWGPRALAET	DRB1*11:12	P43355	MAGE1	260	274	33.84
405	ARYEFLWGPRALAET	DRB1*11:12	P43358	MAGE4	268	282	33.84
406	ARYEFLWGPRALAET	DRB1*11:28	P43355	MAGE1	260	274	33.84
407	ARYEFLWGPRALAET	DRB1*11:28	P43358	MAGE4	268	282	33.84
408	ARYEFLWGPRALAET	DRB1*11:29	P43355	MAGE1	260	274	33.84
409	ARYEFLWGPRALAET	DRB1*11:29	P43358	MAGE4	268	282	33.84
410	ARYEFLWGPRALAET	DRB1*11:49	P43355	MAGE1	260	274	33.84
411	ARYEFLWGPRALAET	DRB1*11:49	P43358	MAGE4	268	282	33.84
412	ARYEFLWGPRALAET	DRB1*11:62	P43355	MAGE1	260	274	33.84
413	ARYEFLWGPRALAET	DRB1*11:62	P43358	MAGE4	268	282	33.84
414	ARYEFLWGPRALAET	DRB1*11:74	P43355	MAGE1	260	274	33.84
415	ARYEFLWGPRALAET	DRB1*11:74	P43358	MAGE4	268	282	33.84
416	ARYEFLWGPRALAET	DRB1*13:05	P43355	MAGE1	260	274	33.84
417	ARYEFLWGPRALAET	DRB1*13:05	P43358	MAGE4	268	282	33.84
418	ARYEFLWGPRALAET	DRB1*13:14	P43355	MAGE1	260	274	33.84
419	ARYEFLWGPRALAET	DRB1*13:14	P43358	MAGE4	268	282	33.84
420	ARYEFLWGPRALAET	DRB1*13:21	P43355	MAGE1	260	274	43.18
421	ARYEFLWGPRALAET	DRB1*13:21	P43358	MAGE4	268	282	43.18
422	ARYEFLWGPRALAET	DRB1*13:50	P43355	MAGE1	260	274	33.84
423	ARYEFLWGPRALAET	DRB1*13:50	P43358	MAGE4	268	282	33.84
424	ARYEFLWGPRALAET	DRB1*15:15	P43355	MAGE1	260	274	42.3
425	ARYEFLWGPRALAET	DRB1*15:15	P43358	MAGE4	268	282	42.3
426	ARYEFLWGPRALAET	DRB1*16:01	P43355	MAGE1	260	274	42.2
427	ARYEFLWGPRALAET	DRB1*16:01	P43358	MAGE4	268	282	42.2
428	ARYEFLWGPRALAET	DRB1*16:02	P43355	MAGE1	260	274	45.7
429	ARYEFLWGPRALAET	DRB1*16:02	P43358	MAGE4	268	282	45.7
430	ARYEFLWGPRALAET	DRB1*16:09	P43355	MAGE1	260	274	38.11
431	ARYEFLWGPRALAET	DRB1*16:09	P43358	MAGE4	268	282	38.11
432	ASEKIFYVYMKRKYE	DRB1*11:01	Q16385	SSX2	42	56	21.98
433	ASEKIFYVYMKRKYE	DRB1*11:03	Q16385	SSX2	42	56	28.81
434	ASEKIFYVYMKRKYE	DRB1*11:04	Q16385	SSX2	42	56	28.14
435	ASEKIFYVYMKRKYE	DRB1*11:10	Q16385	SSX2	42	56	21.98
436	ASEKIFYVYMKRKYE	DRB1*11:12	Q16385	SSX2	42	56	21.98

437	ASEKIFYVYMKRKYE	DRB1*11:28	Q16385	SSX2	42	56	21.98
438	ASEKIFYVYMKRKYE	DRB1*11:29	Q16385	SSX2	42	56	21.98
439	ASEKIFYVYMKRKYE	DRB1*11:42	Q16385	SSX2	42	56	30.02
440	ASEKIFYVYMKRKYE	DRB1*11:46	Q16385	SSX2	42	56	28.14
441	ASEKIFYVYMKRKYE	DRB1*11:49	Q16385	SSX2	42	56	21.98
442	ASEKIFYVYMKRKYE	DRB1*11:58	Q16385	SSX2	42	56	28.14
443	ASEKIFYVYMKRKYE	DRB1*11:62	Q16385	SSX2	42	56	21.98
444	ASEKIFYVYMKRKYE	DRB1*11:74	Q16385	SSX2	42	56	21.98
445	ASEKIFYVYMKRKYE	DRB1*13:05	Q16385	SSX2	42	56	21.98
446	ASEKIFYVYMKRKYE	DRB1*13:11	Q16385	SSX2	42	56	28.14
447	ASEKIFYVYMKRKYE	DRB1*13:14	Q16385	SSX2	42	56	21.98
448	ASEKIFYVYMKRKYE	DRB1*13:21	Q16385	SSX2	42	56	10.4
449	ASEKIFYVYMKRKYE	DRB1*13:50	Q16385	SSX2	42	56	21.98
450	ASESLKMIFGIDVKE	DRB1*01:01	P43358	MAGE4	155	169	10.72
451	ASESLKMIFGIDVKE	DRB1*01:02	P43358	MAGE4	155	169	28.69
452	ASESLKMIFGIDVKE	DRB1*01:11	P43358	MAGE4	155	169	32.49
453	ASESLKMIFGIDVKE	DRB1*01:18	P43358	MAGE4	155	169	10.97
454	ASESLKMIFGIDVKE	DRB1*01:20	P43358	MAGE4	155	169	9.14
455	ASESLKMIFGIDVKE	DRB1*01:24	P43358	MAGE4	155	169	22.73
456	ASESLKMIFGIDVKE	DRB1*01:29	P43358	MAGE4	155	169	22.1
457	ASESLKMIFGIDVKE	DRB1*15:01	P43358	MAGE4	155	169	49.82
458	ASESLKMIFGIDVKE	DRB1*15:06	P43358	MAGE4	155	169	49.82
459	ASESLQLVFGIDVKE	DRB1*01:01	P43355	MAGE1	147	161	31.38
460	ASESLQLVFGIDVKE	DRB1*01:18	P43355	MAGE1	147	161	23.94
461	ASESLQLVFGIDVKE	DRB1*01:20	P43355	MAGE1	147	161	23.86
462	ASFFSSWQIVCSRLE	DRB1*10:01	P14679	Tyrosinase	266	280	49.92
463	ASFSIALNFPGSQKV	DRB1*01:01	P40967	PMEL17	82	96	10.27
464	ASFSIALNFPGSQKV	DRB1*01:11	P40967	PMEL17	82	96	34.74
465	ASFSIALNFPGSQKV	DRB1*01:18	P40967	PMEL17	82	96	10.24
466	ASFSIALNFPGSQKV	DRB1*01:20	P40967	PMEL17	82	96	23.94
467	ASFSIALNFPGSQKV	DRB1*01:24	P40967	PMEL17	82	96	23.59
468	ASFSIALNFPGSQKV	DRB1*01:29	P40967	PMEL17	82	96	25.21
469	ASFSIALNFPGSQKV	DRB1*04:01	P40967	PMEL17	82	96	15.42
470	ASFSIALNFPGSQKV	DRB1*04:04	P40967	PMEL17	82	96	31.54
471	ASFSIALNFPGSQKV	DRB1*04:05	P40967	PMEL17	82	96	32.99
472	ASFSIALNFPGSQKV	DRB1*04:08	P40967	PMEL17	82	96	16.27
473	ASFSIALNFPGSQKV	DRB1*04:72	P40967	PMEL17	82	96	48.49
474	ASFSIALNFPGSQKV	DRB1*09:01	P40967	PMEL17	82	96	49.67
475	ASFSIALNFPGSQKV	DRB1*10:01	P40967	PMEL17	82	96	12.27
476	ASFSIALNFPGSQKV	DRB1*11:01	P40967	PMEL17	82	96	42.13

477	ASFSIALNFPGSQKV	DRB1*11:10	P40967	PMEL17	82	96	42.13
478	ASFSIALNFPGSQKV	DRB1*11:12	P40967	PMEL17	82	96	42.13
479	ASFSIALNFPGSQKV	DRB1*11:14	P40967	PMEL17	82	96	34.8
480	ASFSIALNFPGSQKV	DRB1*11:28	P40967	PMEL17	82	96	42.13
481	ASFSIALNFPGSQKV	DRB1*11:29	P40967	PMEL17	82	96	42.13
482	ASFSIALNFPGSQKV	DRB1*11:49	P40967	PMEL17	82	96	42.13
483	ASFSIALNFPGSQKV	DRB1*11:62	P40967	PMEL17	82	96	42.13
484	ASFSIALNFPGSQKV	DRB1*11:74	P40967	PMEL17	82	96	42.13
485	ASFSIALNFPGSQKV	DRB1*13:02	P40967	PMEL17	82	96	34.8
486	ASFSIALNFPGSQKV	DRB1*13:05	P40967	PMEL17	82	96	42.13
487	ASFSIALNFPGSQKV	DRB1*13:14	P40967	PMEL17	82	96	42.13
488	ASFSIALNFPGSQKV	DRB1*13:23	P40967	PMEL17	82	96	34.8
489	ASFSIALNFPGSQKV	DRB1*13:50	P40967	PMEL17	82	96	42.13
490	ASFSIALNFPGSQKV	DRB1*13:97	P40967	PMEL17	82	96	34.8
491	ASFSIALNFPGSQKV	DRB1*15:15	P40967	PMEL17	82	96	35.75
492	ASFSIALNFPGSQKV	DRB1*16:01	P40967	PMEL17	82	96	29.02
493	ASFSIALNFPGSQKV	DRB1*16:02	P40967	PMEL17	82	96	29.77
494	ASFSIALNFPGSQKV	DRB1*16:09	P40967	PMEL17	82	96	33.44
495	ASLIYRRRLMKQDFS	DRB1*11:02	P40967	PMEL17	612	626	30.9
496	ASLIYRRRLMKQDFS	DRB1*11:03	P40967	PMEL17	612	626	16.9
497	ASLIYRRRLMKQDFS	DRB1*11:04	P40967	PMEL17	612	626	36.01
498	ASLIYRRRLMKQDFS	DRB1*11:42	P40967	PMEL17	612	626	44.48
499	ASLIYRRRLMKQDFS	DRB1*11:46	P40967	PMEL17	612	626	36.01
500	ASLIYRRRLMKQDFS	DRB1*11:58	P40967	PMEL17	612	626	36.01
501	ASLIYRRRLMKQDFS	DRB1*11:65	P40967	PMEL17	612	626	30.9
502	ASLIYRRRLMKQDFS	DRB1*13:01	P40967	PMEL17	612	626	30.9
503	ASLIYRRRLMKQDFS	DRB1*13:11	P40967	PMEL17	612	626	36.01
504	ASRIWSWLLGAAMVG	DRB1*01:01	P14679	Tyrosinase	471	485	10.55
505	ASRIWSWLLGAAMVG	DRB1*01:11	P14679	Tyrosinase	471	485	28.57
506	ASRIWSWLLGAAMVG	DRB1*01:18	P14679	Tyrosinase	471	485	9.89
507	ASRIWSWLLGAAMVG	DRB1*01:20	P14679	Tyrosinase	471	485	33.16
508	ASRIWSWLLGAAMVG	DRB1*01:24	P14679	Tyrosinase	471	485	18.32
509	ASRIWSWLLGAAMVG	DRB1*01:29	P14679	Tyrosinase	471	485	19.7
510	ASRIWSWLLGAAMVG	DRB1*10:01	P14679	Tyrosinase	471	485	28.64
511	ASYLIRARRSMDEAN	DRB1*11:01	P17643	TRP1	497	511	46.19
512	ASYLIRARRSMDEAN	DRB1*11:03	P17643	TRP1	497	511	39.64
513	ASYLIRARRSMDEAN	DRB1*11:04	P17643	TRP1	497	511	28.67
514	ASYLIRARRSMDEAN	DRB1*11:10	P17643	TRP1	497	511	46.19
515	ASYLIRARRSMDEAN	DRB1*11:12	P17643	TRP1	497	511	46.19
516	ASYLIRARRSMDEAN	DRB1*11:28	P17643	TRP1	497	511	46.19

517	ASYLIRARRSMDEAN	DRB1*11:29	P17643	TRP1	497	511	46.19
518	ASYLIRARRSMDEAN	DRB1*11:42	P17643	TRP1	497	511	29.06
519	ASYLIRARRSMDEAN	DRB1*11:46	P17643	TRP1	497	511	28.67
520	ASYLIRARRSMDEAN	DRB1*11:49	P17643	TRP1	497	511	46.19
521	ASYLIRARRSMDEAN	DRB1*11:58	P17643	TRP1	497	511	28.67
522	ASYLIRARRSMDEAN	DRB1*11:62	P17643	TRP1	497	511	46.19
523	ASYLIRARRSMDEAN	DRB1*11:74	P17643	TRP1	497	511	46.19
524	ASYLIRARRSMDEAN	DRB1*13:05	P17643	TRP1	497	511	46.19
525	ASYLIRARRSMDEAN	DRB1*13:11	P17643	TRP1	497	511	28.67
526	ASYLIRARRSMDEAN	DRB1*13:14	P17643	TRP1	497	511	46.19
527	ASYLIRARRSMDEAN	DRB1*13:21	P17643	TRP1	497	511	22.9
528	ASYLIRARRSMDEAN	DRB1*13:50	P17643	TRP1	497	511	46.19
529	ATLRLVKRQVPLDCV	DRB1*08:04	P40967	PMEL17	462	476	38.56
530	ATLRLVKRQVPLDCV	DRB1*11:01	P40967	PMEL17	462	476	32.58
531	ATLRLVKRQVPLDCV	DRB1*11:02	P40967	PMEL17	462	476	33.5
532	ATLRLVKRQVPLDCV	DRB1*11:03	P40967	PMEL17	462	476	25.27
533	ATLRLVKRQVPLDCV	DRB1*11:04	P40967	PMEL17	462	476	15.25
534	ATLRLVKRQVPLDCV	DRB1*11:10	P40967	PMEL17	462	476	32.58
535	ATLRLVKRQVPLDCV	DRB1*11:12	P40967	PMEL17	462	476	32.58
536	ATLRLVKRQVPLDCV	DRB1*11:13	P40967	PMEL17	462	476	31.13
537	ATLRLVKRQVPLDCV	DRB1*11:28	P40967	PMEL17	462	476	32.58
538	ATLRLVKRQVPLDCV	DRB1*11:29	P40967	PMEL17	462	476	32.58
539	ATLRLVKRQVPLDCV	DRB1*11:42	P40967	PMEL17	462	476	15.44
540	ATLRLVKRQVPLDCV	DRB1*11:46	P40967	PMEL17	462	476	15.25
541	ATLRLVKRQVPLDCV	DRB1*11:49	P40967	PMEL17	462	476	32.58
542	ATLRLVKRQVPLDCV	DRB1*11:58	P40967	PMEL17	462	476	15.25
543	ATLRLVKRQVPLDCV	DRB1*11:62	P40967	PMEL17	462	476	32.58
544	ATLRLVKRQVPLDCV	DRB1*11:65	P40967	PMEL17	462	476	33.5
545	ATLRLVKRQVPLDCV	DRB1*11:74	P40967	PMEL17	462	476	32.58
546	ATLRLVKRQVPLDCV	DRB1*13:01	P40967	PMEL17	462	476	33.5
547	ATLRLVKRQVPLDCV	DRB1*13:05	P40967	PMEL17	462	476	32.58
548	ATLRLVKRQVPLDCV	DRB1*13:11	P40967	PMEL17	462	476	15.25
549	ATLRLVKRQVPLDCV	DRB1*13:14	P40967	PMEL17	462	476	32.58
550	ATLRLVKRQVPLDCV	DRB1*13:21	P40967	PMEL17	462	476	34.66
551	ATLRLVKRQVPLDCV	DRB1*13:50	P40967	PMEL17	462	476	32.58
552	ATPMEAELARRSLAQ	DRB1*01:20	P78358	NY-ESO-1	97	111	45.27
553	AVFLALSAQLLQARL	DPB1*33:01	Q13072	BAGE	5	19	41.32
554	AVFLALSAQLLQARL	DPB1*71:01	Q13072	BAGE	5	19	41.32
555	AVFLALSAQLLQARL	DRB1*01:01	Q13072	BAGE	5	19	3
556	AVFLALSAQLLQARL	DRB1*01:02	Q13072	BAGE	5	19	20.12

557	AVFLALSAQLLQARL	DRB1*01:03	Q13072	BAGE	5	19	30.44
558	AVFLALSAQLLQARL	DRB1*01:11	Q13072	BAGE	5	19	4.64
559	AVFLALSAQLLQARL	DRB1*01:18	Q13072	BAGE	5	19	2.74
560	AVFLALSAQLLQARL	DRB1*01:20	Q13072	BAGE	5	19	6.68
561	AVFLALSAQLLQARL	DRB1*01:24	Q13072	BAGE	5	19	3.48
562	AVFLALSAQLLQARL	DRB1*01:29	Q13072	BAGE	5	19	3.89
563	AVFLALSAQLLQARL	DRB1*07:01	Q13072	BAGE	5	19	31.6
564	AVFLALSAQLLQARL	DRB1*10:01	Q13072	BAGE	5	19	8.74
565	AVFLALSAQLLQARL	DRB1*11:13	Q13072	BAGE	5	19	49.77
566	AVFLALSAQLLQARL	DRB1*15:02	Q13072	BAGE	5	19	24.45
567	AVFLALSAQLLQARL	DRB1*15:15	Q13072	BAGE	5	19	25.32
568	AVFLALSAQLLQARL	DRB1*16:01	Q13072	BAGE	5	19	19.65
569	AVFLALSAQLLQARL	DRB1*16:02	Q13072	BAGE	5	19	12.53
570	AVFLALSAQLLQARL	DRB1*16:04	Q13072	BAGE	5	19	27.82
571	AVFLALSAQLLQARL	DRB1*16:05	Q13072	BAGE	5	19	16.3
572	AVFLALSAQLLQARL	DRB1*16:09	Q13072	BAGE	5	19	15
573	AVIGALLAVGATKVP	DRB1*01:01	P40967	PMEL17	13	27	7.61
574	AVIGALLAVGATKVP	DRB1*01:02	P40967	PMEL17	13	27	18.78
575	AVIGALLAVGATKVP	DRB1*01:11	P40967	PMEL17	13	27	22.56
576	AVIGALLAVGATKVP	DRB1*01:18	P40967	PMEL17	13	27	7.63
577	AVIGALLAVGATKVP	DRB1*01:20	P40967	PMEL17	13	27	6.67
578	AVIGALLAVGATKVP	DRB1*01:24	P40967	PMEL17	13	27	14.65
579	AVIGALLAVGATKVP	DRB1*01:29	P40967	PMEL17	13	27	13.65
580	AVIGALLAVGATKVP	DRB1*07:01	P40967	PMEL17	13	27	19.4
581	AVIGALLAVGATKVP	DRB1*10:01	P40967	PMEL17	13	27	28.81
582	AVIGALLAVGATKVP	DRB1*11:14	P40967	PMEL17	13	27	46.91
583	AVIGALLAVGATKVP	DRB1*13:02	P40967	PMEL17	13	27	46.91
584	AVIGALLAVGATKVP	DRB1*13:23	P40967	PMEL17	13	27	46.91
585	AVIGALLAVGATKVP	DRB1*13:97	P40967	PMEL17	13	27	46.91
586	AVITKKVADLVGFLL	DRB1*01:18	P43355	MAGE1	100	114	44.03
587	AVITKKVADLVGFLL	DRB1*01:20	P43355	MAGE1	100	114	40.41
588	AVLTALLAGLVSLLC	DRB1*01:01	P14679	Tyrosinase	486	500	23.43
589	AVLTALLAGLVSLLC	DRB1*01:11	P14679	Tyrosinase	486	500	47.46
590	AVLTALLAGLVSLLC	DRB1*01:18	P14679	Tyrosinase	486	500	18.24
591	AVLTALLAGLVSLLC	DRB1*01:20	P14679	Tyrosinase	486	500	25.23
592	AVLTALLAGLVSLLC	DRB1*01:24	P14679	Tyrosinase	486	500	22.21
593	AVLTALLAGLVSLLC	DRB1*01:29	P14679	Tyrosinase	486	500	37.34
594	AVLYCLLWSFQTSAG	DRB1*01:01	P14679	Tyrosinase	4	18	34.95
595	AVLYCLLWSFQTSAG	DRB1*01:18	P14679	Tyrosinase	4	18	30.66
596	AVLYCLLWSFQTSAG	DRB1*01:24	P14679	Tyrosinase	4	18	49.86

597	AVRSLHNLAHLFLNG	DRB1*01:18	P17643	TRP1	372	386	36.47
598	AVRSLHNLAHLFLNG	DRB1*01:20	P17643	TRP1	372	386	30.45
599	AVVLASLIYRRRLMK	DRB1*01:20	P40967	PMEL17	608	622	36.6
600	AVVLASLIYRRRLMK	DRB1*11:02	P40967	PMEL17	608	622	16.96
601	AVVLASLIYRRRLMK	DRB1*11:03	P40967	PMEL17	608	622	15.16
602	AVVLASLIYRRRLMK	DRB1*11:04	P40967	PMEL17	608	622	20.89
603	AVVLASLIYRRRLMK	DRB1*11:13	P40967	PMEL17	608	622	24.83
604	AVVLASLIYRRRLMK	DRB1*11:42	P40967	PMEL17	608	622	18.72
605	AVVLASLIYRRRLMK	DRB1*11:46	P40967	PMEL17	608	622	20.89
606	AVVLASLIYRRRLMK	DRB1*11:58	P40967	PMEL17	608	622	20.89
607	AVVLASLIYRRRLMK	DRB1*11:65	P40967	PMEL17	608	622	16.96
608	AVVLASLIYRRRLMK	DRB1*11:84	P40967	PMEL17	608	622	38.65
609	AVVLASLIYRRRLMK	DRB1*13:01	P40967	PMEL17	608	622	16.96
610	AVVLASLIYRRRLMK	DRB1*13:11	P40967	PMEL17	608	622	20.89
611	AVVLASLIYRRRLMK	DRB1*13:21	P40967	PMEL17	608	622	41.5
612	AVVLASLIYRRRLMK	DRB1*13:61	P40967	PMEL17	608	622	45.15
613	AVVLASLIYRRRLMK	DRB1*14:06	P40967	PMEL17	608	622	32.16
614	AVVLASLIYRRRLMK	DRB1*14:12	P40967	PMEL17	608	622	44.8
615	AVVLASLIYRRRLMK	DRB1*14:32	P40967	PMEL17	608	622	48.73
616	CDFQEFMAFVAMVTT	DRB1*01:18	P04271	S100	67	81	30.35
617	CDQRVLIVRRNLLDL	DRB1*08:04	P17643	TRP1	122	136	49.14
618	CDQRVLIVRRNLLDL	DRB1*11:02	P17643	TRP1	122	136	25.37
619	CDQRVLIVRRNLLDL	DRB1*11:03	P17643	TRP1	122	136	33.61
620	CDQRVLIVRRNLLDL	DRB1*11:04	P17643	TRP1	122	136	20.29
621	CDQRVLIVRRNLLDL	DRB1*11:13	P17643	TRP1	122	136	15.74
622	CDQRVLIVRRNLLDL	DRB1*11:42	P17643	TRP1	122	136	15.47
623	CDQRVLIVRRNLLDL	DRB1*11:46	P17643	TRP1	122	136	20.29
624	CDQRVLIVRRNLLDL	DRB1*11:58	P17643	TRP1	122	136	20.29
625	CDQRVLIVRRNLLDL	DRB1*11:65	P17643	TRP1	122	136	25.37
626	CDQRVLIVRRNLLDL	DRB1*11:84	P17643	TRP1	122	136	41.52
627	CDQRVLIVRRNLLDL	DRB1*13:01	P17643	TRP1	122	136	25.37
628	CDQRVLIVRRNLLDL	DRB1*13:11	P17643	TRP1	122	136	20.29
629	CDQRVLIVRRNLLDL	DRB1*13:21	P17643	TRP1	122	136	35.86
630	CDQRVLIVRRNLLDL	DRB1*14:01	P17643	TRP1	122	136	25.73
631	CDQRVLIVRRNLLDL	DRB1*14:04	P17643	TRP1	122	136	42.51
632	CDQRVLIVRRNLLDL	DRB1*14:06	P17643	TRP1	122	136	43.83
633	CDQRVLIVRRNLLDL	DRB1*14:32	P17643	TRP1	122	136	14.12
634	CDQRVLIVRRNLLDL	DRB1*14:54	P17643	TRP1	122	136	25.73
635	CFLPVFLAQPPSGQR	DRB1*01:01	P78358	NY-ESO-1	165	179	41.92
636	CFLPVFLAQPPSGQR	DRB1*01:20	P78358	NY-ESO-1	165	179	47.69

637	CFLPVFLAQPPSGQR	DRB1*10:01	P78358	NY-ESO-1	165	179	31.83
638	CFPVIFGKASESLKM	DRB1*01:01	P43358	MAGE4	147	161	44.5
639	CFPVIFGKASESLKM	DRB1*01:18	P43358	MAGE4	147	161	40.2
640	CFPVIFGKASESLKM	DRB1*01:20	P43358	MAGE4	147	161	31.33
641	CIFFPLLLFQQARAQ	DRB1*01:18	P17643	TRP1	11	25	38.57
642	CIFFPLLLFQQARAQ	DRB1*01:20	P17643	TRP1	11	25	28.61
643	CIFFPLLLFQQARAQ	DRB1*10:01	P17643	TRP1	11	25	46.92
644	CILESLFRAVITKKV	DPB1*02:01	P43355	MAGE1	92	106	39.93
645	CILESLFRAVITKKV	DPB1*02:02	P43355	MAGE1	92	106	38.06
646	CILESLFRAVITKKV	DPB1*33:01	P43355	MAGE1	92	106	19.12
647	CILESLFRAVITKKV	DPB1*46:01	P43355	MAGE1	92	106	39.93
648	CILESLFRAVITKKV	DPB1*47:01	P43355	MAGE1	92	106	38.06
649	CILESLFRAVITKKV	DPB1*71:01	P43355	MAGE1	92	106	19.12
650	CILESLFRAVITKKV	DPB1*81:01	P43355	MAGE1	92	106	39.93
651	CILESLFRAVITKKV	DRB1*01:01	P43355	MAGE1	92	106	13.91
652	CILESLFRAVITKKV	DRB1*01:11	P43355	MAGE1	92	106	37.22
653	CILESLFRAVITKKV	DRB1*01:18	P43355	MAGE1	92	106	11.32
654	CILESLFRAVITKKV	DRB1*01:20	P43355	MAGE1	92	106	28.79
655	CILESLFRAVITKKV	DRB1*01:24	P43355	MAGE1	92	106	30.97
656	CILESLFRAVITKKV	DRB1*01:29	P43355	MAGE1	92	106	23.29
657	CILESLFRAVITKKV	DRB1*07:01	P43355	MAGE1	92	106	7.59
658	CILESLFRAVITKKV	DRB1*09:01	P43355	MAGE1	92	106	22.14
659	CILESLFRAVITKKV	DRB1*10:01	P43355	MAGE1	92	106	28.2
660	CILESLFRAVITKKV	DRB1*11:04	P43355	MAGE1	92	106	49.41
661	CILESLFRAVITKKV	DRB1*11:13	P43355	MAGE1	92	106	43.87
662	CILESLFRAVITKKV	DRB1*11:42	P43355	MAGE1	92	106	39.57
663	CILESLFRAVITKKV	DRB1*11:46	P43355	MAGE1	92	106	49.41
664	CILESLFRAVITKKV	DRB1*11:58	P43355	MAGE1	92	106	49.41
665	CILESLFRAVITKKV	DRB1*13:11	P43355	MAGE1	92	106	49.41
666	CILESLFRAVITKKV	DRB1*13:21	P43355	MAGE1	92	106	42.81
667	CILESLFRAVITKKV	DRB1*14:32	P43355	MAGE1	92	106	44.43
668	CILESLFRAVITKKV	DRB1*15:15	P43355	MAGE1	92	106	49.2
669	CILESLFRAVITKKV	DRB1*16:01	P43355	MAGE1	92	106	40.12
670	CILESLFRAVITKKV	DRB1*16:02	P43355	MAGE1	92	106	38.27
671	CILESLFRAVITKKV	DRB1*16:09	P43355	MAGE1	92	106	36.39
672	CLLHLAVIGALLAVG	DRB1*01:18	P40967	PMEL17	8	22	44.89
673	CLLWSFQTSAGHFPR	DRB1*10:01	P14679	Tyrosinase	8	22	39.46
674	CQLVLHQILKGGSGT	DRB1*08:04	P40967	PMEL17	550	564	43.09
675	CQLVLHQILKGGSGT	DRB1*11:01	P40967	PMEL17	550	564	45.41
676	CQLVLHQILKGGSGT	DRB1*11:03	P40967	PMEL17	550	564	43.28

677	CQLVLHQILKGGSGT	DRB1*11:04	P40967	PMEL17	550	564	17.75
678	CQLVLHQILKGGSGT	DRB1*11:10	P40967	PMEL17	550	564	45.41
679	CQLVLHQILKGGSGT	DRB1*11:12	P40967	PMEL17	550	564	45.41
680	CQLVLHQILKGGSGT	DRB1*11:28	P40967	PMEL17	550	564	45.41
681	CQLVLHQILKGGSGT	DRB1*11:29	P40967	PMEL17	550	564	45.41
682	CQLVLHQILKGGSGT	DRB1*11:42	P40967	PMEL17	550	564	28.37
683	CQLVLHQILKGGSGT	DRB1*11:46	P40967	PMEL17	550	564	17.75
684	CQLVLHQILKGGSGT	DRB1*11:49	P40967	PMEL17	550	564	45.41
685	CQLVLHQILKGGSGT	DRB1*11:58	P40967	PMEL17	550	564	17.75
686	CQLVLHQILKGGSGT	DRB1*11:62	P40967	PMEL17	550	564	45.41
687	CQLVLHQILKGGSGT	DRB1*11:74	P40967	PMEL17	550	564	45.41
688	CQLVLHQILKGGSGT	DRB1*13:05	P40967	PMEL17	550	564	45.41
689	CQLVLHQILKGGSGT	DRB1*13:11	P40967	PMEL17	550	564	17.75
690	CQLVLHQILKGGSGT	DRB1*13:14	P40967	PMEL17	550	564	45.41
691	CQLVLHQILKGGSGT	DRB1*13:50	P40967	PMEL17	550	564	45.41
692	CQNILLSNAPLGPQF	DRB1*01:01	P14679	Tyrosinase	55	69	24.12
693	CQNILLSNAPLGPQF	DRB1*01:18	P14679	Tyrosinase	55	69	21.97
694	CQNILLSNAPLGPQF	DRB1*01:20	P14679	Tyrosinase	55	69	17.94
695	CQNILLSNAPLGPQF	DRB1*11:14	P14679	Tyrosinase	55	69	28.2
696	CQNILLSNAPLGPQF	DRB1*13:02	P14679	Tyrosinase	55	69	28.2
697	CQNILLSNAPLGPQF	DRB1*13:23	P14679	Tyrosinase	55	69	28.2
698	CQNILLSNAPLGPQF	DRB1*13:97	P14679	Tyrosinase	55	69	28.2
699	CQPVLPSPACQLVLH	DRB1*01:20	P40967	PMEL17	541	555	48.38
700	CSVYDFFVWLHYYSV	DPB1*02:01	O75767	TRP2	179	193	33
701	CSVYDFFVWLHYYSV	DPB1*02:02	O75767	TRP2	179	193	28.85
702	CSVYDFFVWLHYYSV	DPB1*04:01	O75767	TRP2	179	193	31.35
703	CSVYDFFVWLHYYSV	DPB1*126:01	O75767	TRP2	179	193	31.35
704	CSVYDFFVWLHYYSV	DPB1*23:01	O75767	TRP2	179	193	31.35
705	CSVYDFFVWLHYYSV	DPB1*33:01	O75767	TRP2	179	193	12.36
706	CSVYDFFVWLHYYSV	DPB1*39:01	O75767	TRP2	179	193	31.35
707	CSVYDFFVWLHYYSV	DPB1*46:01	O75767	TRP2	179	193	33
708	CSVYDFFVWLHYYSV	DPB1*47:01	O75767	TRP2	179	193	28.85
709	CSVYDFFVWLHYYSV	DPB1*71:01	O75767	TRP2	179	193	12.36
710	CSVYDFFVWLHYYSV	DPB1*81:01	O75767	TRP2	179	193	33
711	CTEVRADTRPWSGPY	DRB1*03:11	O75767	TRP2	61	75	28.01
712	CVLYRYGSFSVTLDI	DPB1*33:01	P40967	PMEL17	475	489	31.94
713	CVLYRYGSFSVTLDI	DPB1*71:01	P40967	PMEL17	475	489	31.94
714	CVLYRYGSFSVTLDI	DRB1*04:05	P40967	PMEL17	475	489	35.84
715	CVLYRYGSFSVTLDI	DRB1*15:01	P40967	PMEL17	475	489	27.48
716	CVLYRYGSFSVTLDI	DRB1*15:06	P40967	PMEL17	475	489	27.48

717	DAESLFREALSNKVD	DRB1*01:01	P43358	MAGE4	101	115	5.8
718	DAESLFREALSNKVD	DRB1*01:11	P43358	MAGE4	101	115	16.22
719	DAESLFREALSNKVD	DRB1*01:18	P43358	MAGE4	101	115	6.63
720	DAESLFREALSNKVD	DRB1*01:20	P43358	MAGE4	101	115	23.03
721	DAESLFREALSNKVD	DRB1*01:24	P43358	MAGE4	101	115	14.67
722	DAESLFREALSNKVD	DRB1*01:29	P43358	MAGE4	101	115	13.37
723	DAESLFREALSNKVD	DRB1*07:01	P43358	MAGE4	101	115	7.47
724	DAESLFREALSNKVD	DRB1*09:01	P43358	MAGE4	101	115	12.29
725	DAESLFREALSNKVD	DRB1*10:01	P43358	MAGE4	101	115	17.97
726	DAESLFREALSNKVD	DRB1*16:02	P43358	MAGE4	101	115	24.6
727	DCVLYRYGSFSVTLD	DPB1*33:01	P40967	PMEL17	474	488	34.01
728	DCVLYRYGSFSVTLD	DPB1*71:01	P40967	PMEL17	474	488	34.01
729	DCVLYRYGSFSVTLD	DRB1*15:01	P40967	PMEL17	474	488	21.25
730	DCVLYRYGSFSVTLD	DRB1*15:02	P40967	PMEL17	474	488	48.71
731	DCVLYRYGSFSVTLD	DRB1*15:06	P40967	PMEL17	474	488	21.25
732	DCVLYRYGSFSVTLD	DRB1*15:07	P40967	PMEL17	474	488	49.01
733	DCVLYRYGSFSVTLD	DRB1*15:37	P40967	PMEL17	474	488	49.93
734	DELAHFLLRKYRAKE	DRB1*11:01	P43358	MAGE4	115	129	39.02
735	DELAHFLLRKYRAKE	DRB1*11:03	P43358	MAGE4	115	129	25.19
736	DELAHFLLRKYRAKE	DRB1*11:04	P43358	MAGE4	115	129	25.68
737	DELAHFLLRKYRAKE	DRB1*11:10	P43358	MAGE4	115	129	39.02
738	DELAHFLLRKYRAKE	DRB1*11:12	P43358	MAGE4	115	129	39.02
739	DELAHFLLRKYRAKE	DRB1*11:28	P43358	MAGE4	115	129	39.02
740	DELAHFLLRKYRAKE	DRB1*11:29	P43358	MAGE4	115	129	39.02
741	DELAHFLLRKYRAKE	DRB1*11:42	P43358	MAGE4	115	129	29.07
742	DELAHFLLRKYRAKE	DRB1*11:46	P43358	MAGE4	115	129	25.68
743	DELAHFLLRKYRAKE	DRB1*11:49	P43358	MAGE4	115	129	39.02
744	DELAHFLLRKYRAKE	DRB1*11:58	P43358	MAGE4	115	129	25.68
745	DELAHFLLRKYRAKE	DRB1*11:62	P43358	MAGE4	115	129	39.02
746	DELAHFLLRKYRAKE	DRB1*11:74	P43358	MAGE4	115	129	39.02
747	DELAHFLLRKYRAKE	DRB1*13:05	P43358	MAGE4	115	129	39.02
748	DELAHFLLRKYRAKE	DRB1*13:11	P43358	MAGE4	115	129	25.68
749	DELAHFLLRKYRAKE	DRB1*13:14	P43358	MAGE4	115	129	39.02
750	DELAHFLLRKYRAKE	DRB1*13:21	P43358	MAGE4	115	129	29.82
751	DELAHFLLRKYRAKE	DRB1*13:50	P43358	MAGE4	115	129	39.02
752	DFFVWLHYYSVRDTL	DPB1*02:01	O75767	TRP2	183	197	16.72
753	DFFVWLHYYSVRDTL	DPB1*02:02	O75767	TRP2	183	197	17.81
754	DFFVWLHYYSVRDTL	DPB1*04:01	O75767	TRP2	183	197	25.56
755	DFFVWLHYYSVRDTL	DPB1*126:01	O75767	TRP2	183	197	25.56
756	DFFVWLHYYSVRDTL	DPB1*15:01	O75767	TRP2	183	197	44.02

757	DFFVWLHYYSVRDTL	DPB1*23:01	O75767	TRP2	183	197	25.56
758	DFFVWLHYYSVRDTL	DPB1*33:01	O75767	TRP2	183	197	8.37
759	DFFVWLHYYSVRDTL	DPB1*39:01	O75767	TRP2	183	197	25.56
760	DFFVWLHYYSVRDTL	DPB1*46:01	O75767	TRP2	183	197	16.72
761	DFFVWLHYYSVRDTL	DPB1*47:01	O75767	TRP2	183	197	17.81
762	DFFVWLHYYSVRDTL	DPB1*71:01	O75767	TRP2	183	197	8.37
763	DFFVWLHYYSVRDTL	DPB1*72:01	O75767	TRP2	183	197	31.85
764	DFFVWLHYYSVRDTL	DPB1*81:01	O75767	TRP2	183	197	16.72
765	DFFVWLHYYSVRDTL	DRB1*01:18	O75767	TRP2	183	197	33.13
766	DFFVWLHYYSVRDTL	DRB1*15:01	O75767	TRP2	183	197	37.9
767	DFFVWLHYYSVRDTL	DRB1*15:06	O75767	TRP2	183	197	37.9
768	DFFVWLHYYSVRDTL	DRB1*15:15	O75767	TRP2	183	197	48.79
769	DFQEFMAFVAMVTTA	DRB1*01:01	P04271	S100	68	82	39.72
770	DFQEFMAFVAMVTTA	DRB1*01:18	P04271	S100	68	82	22.4
771	DFQEFMAFVAMVTTA	DRB1*01:24	P04271	S100	68	82	43.3
772	DFQEFMAFVAMVTTA	DRB1*10:01	P04271	S100	68	82	43.75
773	DGGNKHFLRNQPLTF	DRB1*01:01	P40967	PMEL17	226	240	43.6
774	DGGNKHFLRNQPLTF	DRB1*01:18	P40967	PMEL17	226	240	35.59
775	DGGNKHFLRNQPLTF	DRB1*11:14	P40967	PMEL17	226	240	18.26
776	DGGNKHFLRNQPLTF	DRB1*13:02	P40967	PMEL17	226	240	18.26
777	DGGNKHFLRNQPLTF	DRB1*13:23	P40967	PMEL17	226	240	18.26
778	DGGNKHFLRNQPLTF	DRB1*13:66	P40967	PMEL17	226	240	48.03
779	DGGNKHFLRNQPLTF	DRB1*13:96	P40967	PMEL17	226	240	25.84
780	DGGNKHFLRNQPLTF	DRB1*13:97	P40967	PMEL17	226	240	18.26
781	DGPIRRNPAGNVARP	DRB1*11:14	P17643	TRP1	308	322	36.78
782	DGPIRRNPAGNVARP	DRB1*13:02	P17643	TRP1	308	322	36.78
783	DGPIRRNPAGNVARP	DRB1*13:23	P17643	TRP1	308	322	36.78
784	DGPIRRNPAGNVARP	DRB1*13:97	P17643	TRP1	308	322	36.78
785	DGPTLIGANASFSIA	DRB1*01:01	P40967	PMEL17	73	87	46.2
786	DGPTLIGANASFSIA	DRB1*01:20	P40967	PMEL17	73	87	37.12
787	DGPTLIGANASFSIA	DRB1*11:14	P40967	PMEL17	73	87	24.48
788	DGPTLIGANASFSIA	DRB1*13:02	P40967	PMEL17	73	87	24.48
789	DGPTLIGANASFSIA	DRB1*13:23	P40967	PMEL17	73	87	24.48
790	DGPTLIGANASFSIA	DRB1*13:96	P40967	PMEL17	73	87	41.5
791	DGPTLIGANASFSIA	DRB1*13:97	P40967	PMEL17	73	87	24.48
792	DGQVIWVNNTIINGS	DRB1*11:14	P40967	PMEL17	99	113	17.49
793	DGQVIWVNNTIINGS	DRB1*13:02	P40967	PMEL17	99	113	17.49
794	DGQVIWVNNTIINGS	DRB1*13:23	P40967	PMEL17	99	113	17.49
795	DGQVIWVNNTIINGS	DRB1*13:96	P40967	PMEL17	99	113	25.54
796	DGQVIWVNNTIINGS	DRB1*13:97	P40967	PMEL17	99	113	17.49

797	DGTATLRLVKRQVPL	DRB1*08:04	P40967	PMEL17	459	473	24.49
798	DGTATLRLVKRQVPL	DRB1*11:01	P40967	PMEL17	459	473	18.27
799	DGTATLRLVKRQVPL	DRB1*11:02	P40967	PMEL17	459	473	22.93
800	DGTATLRLVKRQVPL	DRB1*11:03	P40967	PMEL17	459	473	17.41
801	DGTATLRLVKRQVPL	DRB1*11:04	P40967	PMEL17	459	473	9.31
802	DGTATLRLVKRQVPL	DRB1*11:06	P40967	PMEL17	459	473	38.75
803	DGTATLRLVKRQVPL	DRB1*11:08	P40967	PMEL17	459	473	40.82
804	DGTATLRLVKRQVPL	DRB1*11:10	P40967	PMEL17	459	473	18.27
805	DGTATLRLVKRQVPL	DRB1*11:12	P40967	PMEL17	459	473	18.27
806	DGTATLRLVKRQVPL	DRB1*11:13	P40967	PMEL17	459	473	20.09
807	DGTATLRLVKRQVPL	DRB1*11:28	P40967	PMEL17	459	473	18.27
808	DGTATLRLVKRQVPL	DRB1*11:29	P40967	PMEL17	459	473	18.27
809	DGTATLRLVKRQVPL	DRB1*11:42	P40967	PMEL17	459	473	10.82
810	DGTATLRLVKRQVPL	DRB1*11:46	P40967	PMEL17	459	473	9.31
811	DGTATLRLVKRQVPL	DRB1*11:49	P40967	PMEL17	459	473	18.27
812	DGTATLRLVKRQVPL	DRB1*11:54	P40967	PMEL17	459	473	47.87
813	DGTATLRLVKRQVPL	DRB1*11:58	P40967	PMEL17	459	473	9.31
814	DGTATLRLVKRQVPL	DRB1*11:62	P40967	PMEL17	459	473	18.27
815	DGTATLRLVKRQVPL	DRB1*11:65	P40967	PMEL17	459	473	22.93
816	DGTATLRLVKRQVPL	DRB1*11:74	P40967	PMEL17	459	473	18.27
817	DGTATLRLVKRQVPL	DRB1*11:84	P40967	PMEL17	459	473	27.15
818	DGTATLRLVKRQVPL	DRB1*13:01	P40967	PMEL17	459	473	22.93
819	DGTATLRLVKRQVPL	DRB1*13:05	P40967	PMEL17	459	473	18.27
820	DGTATLRLVKRQVPL	DRB1*13:11	P40967	PMEL17	459	473	9.31
821	DGTATLRLVKRQVPL	DRB1*13:14	P40967	PMEL17	459	473	18.27
822	DGTATLRLVKRQVPL	DRB1*13:21	P40967	PMEL17	459	473	21.14
823	DGTATLRLVKRQVPL	DRB1*13:50	P40967	PMEL17	459	473	18.27
824	DGTATLRLVKRQVPL	DRB1*14:06	P40967	PMEL17	459	473	41.99
825	DGTATLRLVKRQVPL	DRB1*14:32	P40967	PMEL17	459	473	35.57
826	DHRQLQLSISSCLQQ	DRB1*01:01	P78358	NY-ESO-1	141	155	21.04
827	DHRQLQLSISSCLQQ	DRB1*01:02	P78358	NY-ESO-1	141	155	47.4
828	DHRQLQLSISSCLQQ	DRB1*01:11	P78358	NY-ESO-1	141	155	48.76
829	DHRQLQLSISSCLQQ	DRB1*01:18	P78358	NY-ESO-1	141	155	19.82
830	DHRQLQLSISSCLQQ	DRB1*01:20	P78358	NY-ESO-1	141	155	18.34
831	DHRQLQLSISSCLQQ	DRB1*01:24	P78358	NY-ESO-1	141	155	40.38
832	DHRQLQLSISSCLQQ	DRB1*01:29	P78358	NY-ESO-1	141	155	38.66
833	DHRQLQLSISSCLQQ	DRB1*07:01	P78358	NY-ESO-1	141	155	14.17
834	DHRQLQLSISSCLQQ	DRB1*09:01	P78358	NY-ESO-1	141	155	35.68
835	DHRQLQLSISSCLQQ	DRB1*11:14	P78358	NY-ESO-1	141	155	42.47
836	DHRQLQLSISSCLQQ	DRB1*13:02	P78358	NY-ESO-1	141	155	42.47

837	DHRQLQLSISSCLQQ	DRB1*13:23	P78358	NY-ESO-1	141	155	42.47
838	DHRQLQLSISSCLQQ	DRB1*13:97	P78358	NY-ESO-1	141	155	42.47
839	DHRQLQLSISSCLQQ	DRB1*14:32	P78358	NY-ESO-1	141	155	48.59
840	DIDFAHEAPAFLPWH	DRB1*01:01	P14679	Tyrosinase	197	211	27.73
841	DIDFAHEAPAFLPWH	DRB1*01:18	P14679	Tyrosinase	197	211	32.52
842	DKFFAYLTLAKHTIS	DPB1*33:01	P14679	Tyrosinase	132	146	33.5
843	DKFFAYLTLAKHTIS	DPB1*71:01	P14679	Tyrosinase	132	146	33.5
844	DKFFAYLTLAKHTIS	DRB1*01:01	P14679	Tyrosinase	132	146	18.08
845	DKFFAYLTLAKHTIS	DRB1*01:18	P14679	Tyrosinase	132	146	14.72
846	DKFFAYLTLAKHTIS	DRB1*01:20	P14679	Tyrosinase	132	146	27.98
847	DKFFAYLTLAKHTIS	DRB1*01:24	P14679	Tyrosinase	132	146	45
848	DKFFAYLTLAKHTIS	DRB1*01:29	P14679	Tyrosinase	132	146	30.36
849	DKFFAYLTLAKHTIS	DRB1*10:01	P14679	Tyrosinase	132	146	8.37
850	DKFFAYLTLAKHTIS	DRB1*11:01	P14679	Tyrosinase	132	146	26.01
851	DKFFAYLTLAKHTIS	DRB1*11:04	P14679	Tyrosinase	132	146	46.72
852	DKFFAYLTLAKHTIS	DRB1*11:08	P14679	Tyrosinase	132	146	39.26
853	DKFFAYLTLAKHTIS	DRB1*11:10	P14679	Tyrosinase	132	146	26.01
854	DKFFAYLTLAKHTIS	DRB1*11:12	P14679	Tyrosinase	132	146	26.01
855	DKFFAYLTLAKHTIS	DRB1*11:28	P14679	Tyrosinase	132	146	26.01
856	DKFFAYLTLAKHTIS	DRB1*11:29	P14679	Tyrosinase	132	146	26.01
857	DKFFAYLTLAKHTIS	DRB1*11:42	P14679	Tyrosinase	132	146	43.99
858	DKFFAYLTLAKHTIS	DRB1*11:46	P14679	Tyrosinase	132	146	46.72
859	DKFFAYLTLAKHTIS	DRB1*11:49	P14679	Tyrosinase	132	146	26.01
860	DKFFAYLTLAKHTIS	DRB1*11:58	P14679	Tyrosinase	132	146	46.72
861	DKFFAYLTLAKHTIS	DRB1*11:62	P14679	Tyrosinase	132	146	26.01
862	DKFFAYLTLAKHTIS	DRB1*11:74	P14679	Tyrosinase	132	146	26.01
863	DKFFAYLTLAKHTIS	DRB1*12:16	P14679	Tyrosinase	132	146	38.91
864	DKFFAYLTLAKHTIS	DRB1*13:05	P14679	Tyrosinase	132	146	26.01
865	DKFFAYLTLAKHTIS	DRB1*13:11	P14679	Tyrosinase	132	146	46.72
866	DKFFAYLTLAKHTIS	DRB1*13:14	P14679	Tyrosinase	132	146	26.01
867	DKFFAYLTLAKHTIS	DRB1*13:21	P14679	Tyrosinase	132	146	24.12
868	DKFFAYLTLAKHTIS	DRB1*13:50	P14679	Tyrosinase	132	146	26.01
869	DKFFAYLTLAKHTIS	DRB1*15:15	P14679	Tyrosinase	132	146	36.67
870	DKFFAYLTLAKHTIS	DRB1*16:09	P14679	Tyrosinase	132	146	34.14
871	DLFVWMHYYVSMDAL	DPB1*33:01	P14679	Tyrosinase	174	188	19.62
872	DLFVWMHYYVSMDAL	DPB1*71:01	P14679	Tyrosinase	174	188	19.62
873	DLVGFLLLKYRAREP	DPB1*33:01	P43355	MAGE1	108	122	33.68
874	DLVGFLLLKYRAREP	DPB1*71:01	P43355	MAGE1	108	122	33.68
875	DLVGFLLLKYRAREP	DRB1*01:18	P43355	MAGE1	108	122	46.26
876	DLVGFLLLKYRAREP	DRB1*01:20	P43355	MAGE1	108	122	48.15

877	DLVGFLLLKYRAREP	DRB1*11:01	P43355	MAGE1	108	122	28.93
878	DLVGFLLLKYRAREP	DRB1*11:02	P43355	MAGE1	108	122	42.83
879	DLVGFLLLKYRAREP	DRB1*11:03	P43355	MAGE1	108	122	25.13
880	DLVGFLLLKYRAREP	DRB1*11:04	P43355	MAGE1	108	122	17.99
881	DLVGFLLLKYRAREP	DRB1*11:10	P43355	MAGE1	108	122	28.93
882	DLVGFLLLKYRAREP	DRB1*11:12	P43355	MAGE1	108	122	28.93
883	DLVGFLLLKYRAREP	DRB1*11:13	P43355	MAGE1	108	122	39.33
884	DLVGFLLLKYRAREP	DRB1*11:28	P43355	MAGE1	108	122	28.93
885	DLVGFLLLKYRAREP	DRB1*11:29	P43355	MAGE1	108	122	28.93
886	DLVGFLLLKYRAREP	DRB1*11:42	P43355	MAGE1	108	122	20.99
887	DLVGFLLLKYRAREP	DRB1*11:46	P43355	MAGE1	108	122	17.99
888	DLVGFLLLKYRAREP	DRB1*11:49	P43355	MAGE1	108	122	28.93
889	DLVGFLLLKYRAREP	DRB1*11:58	P43355	MAGE1	108	122	17.99
890	DLVGFLLLKYRAREP	DRB1*11:62	P43355	MAGE1	108	122	28.93
891	DLVGFLLLKYRAREP	DRB1*11:65	P43355	MAGE1	108	122	42.83
892	DLVGFLLLKYRAREP	DRB1*11:74	P43355	MAGE1	108	122	28.93
893	DLVGFLLLKYRAREP	DRB1*11:84	P43355	MAGE1	108	122	45.88
894	DLVGFLLLKYRAREP	DRB1*12:03	P43355	MAGE1	108	122	49.86
895	DLVGFLLLKYRAREP	DRB1*12:16	P43355	MAGE1	108	122	43.87
896	DLVGFLLLKYRAREP	DRB1*13:01	P43355	MAGE1	108	122	42.83
897	DLVGFLLLKYRAREP	DRB1*13:05	P43355	MAGE1	108	122	28.93
898	DLVGFLLLKYRAREP	DRB1*13:11	P43355	MAGE1	108	122	17.99
899	DLVGFLLLKYRAREP	DRB1*13:14	P43355	MAGE1	108	122	28.93
900	DLVGFLLLKYRAREP	DRB1*13:21	P43355	MAGE1	108	122	20.76
901	DLVGFLLLKYRAREP	DRB1*13:50	P43355	MAGE1	108	122	28.93
902	DMQEMLQEPSFSLPY	DRB1*01:20	P17643	TRP1	234	248	45.21
903	DNQIMPKTGFLIIVL	DRB1*01:01	P43355	MAGE1	185	199	35.32
904	DNQIMPKTGFLIIVL	DRB1*01:18	P43355	MAGE1	185	199	25.03
905	DNQIMPKTGFLIIVL	DRB1*01:20	P43355	MAGE1	185	199	21.82
906	DPARYEFLWGPRALA	DRB1*01:01	P43355	MAGE1	258	272	7.86
907	DPARYEFLWGPRALA	DRB1*01:11	P43355	MAGE1	258	272	25.76
908	DPARYEFLWGPRALA	DRB1*01:18	P43355	MAGE1	258	272	7.84
909	DPARYEFLWGPRALA	DRB1*01:20	P43355	MAGE1	258	272	25.1
910	DPARYEFLWGPRALA	DRB1*01:24	P43355	MAGE1	258	272	18.56
911	DPARYEFLWGPRALA	DRB1*01:29	P43355	MAGE1	258	272	16.85
912	DPARYEFLWGPRALA	DRB1*10:01	P43355	MAGE1	258	272	18.33
913	DPARYEFLWGPRALA	DRB1*15:15	P43355	MAGE1	258	272	39.43
914	DPARYEFLWGPRALA	DRB1*16:01	P43355	MAGE1	258	272	39.27
915	DPARYEFLWGPRALA	DRB1*16:02	P43355	MAGE1	258	272	41.06
916	DPARYEFLWGPRALA	DRB1*16:09	P43355	MAGE1	258	272	36.78

917	DPAVRSLHNLAHLFL	DRB1*01:01	P17643	TRP1	370	384	44.8
918	DPAVRSLHNLAHLFL	DRB1*01:18	P17643	TRP1	370	384	31.18
919	DPAVRSLHNLAHLFL	DRB1*01:20	P17643	TRP1	370	384	22.2
920	DPAVRSLHNLAHLFL	DRB1*04:04	P17643	TRP1	370	384	23.28
921	DPAVRSLHNLAHLFL	DRB1*04:10	P17643	TRP1	370	384	41.97
922	DPAVRSLHNLAHLFL	DRB1*10:01	P17643	TRP1	370	384	38.99
923	DPAVRSLHNLAHLFL	DRB1*11:42	P17643	TRP1	370	384	36.14
924	DPIFLLHHAFVDSIF	DPB1*02:01	P14679	Tyrosinase	383	397	22.28
925	DPIFLLHHAFVDSIF	DPB1*02:02	P14679	Tyrosinase	383	397	22.77
926	DPIFLLHHAFVDSIF	DPB1*33:01	P14679	Tyrosinase	383	397	12.43
927	DPIFLLHHAFVDSIF	DPB1*46:01	P14679	Tyrosinase	383	397	22.28
928	DPIFLLHHAFVDSIF	DPB1*47:01	P14679	Tyrosinase	383	397	22.77
929	DPIFLLHHAFVDSIF	DPB1*71:01	P14679	Tyrosinase	383	397	12.43
930	DPIFLLHHAFVDSIF	DPB1*81:01	P14679	Tyrosinase	383	397	22.28
931	DPIFLLHHAFVDSIF	DRB1*01:01	P14679	Tyrosinase	383	397	44.27
932	DPIFLLHHAFVDSIF	DRB1*01:18	P14679	Tyrosinase	383	397	27.37
933	DPIFLLHHAFVDSIF	DRB1*07:01	P14679	Tyrosinase	383	397	39.65
934	DPIFLLHHAFVDSIF	DRB1*09:01	P14679	Tyrosinase	383	397	45.8
935	DPIFLLHHAFVDSIF	DRB1*10:01	P14679	Tyrosinase	383	397	37.62
936	DPIFVLLHTFTDAVF	DPB1*33:01	P17643	TRP1	397	411	31.99
937	DPIFVLLHTFTDAVF	DPB1*71:01	P17643	TRP1	397	411	31.99
938	DPIFVLLHTFTDAVF	DRB1*01:01	P17643	TRP1	397	411	30.92
939	DPIFVLLHTFTDAVF	DRB1*01:18	P17643	TRP1	397	411	23.02
940	DPIFVLLHTFTDAVF	DRB1*04:05	P17643	TRP1	397	411	16.23
941	DPIFVLLHTFTDAVF	DRB1*04:08	P17643	TRP1	397	411	32.47
942	DPIFVLLHTFTDAVF	DRB1*04:10	P17643	TRP1	397	411	43.85
943	DPIFVLLHTFTDAVF	DRB1*10:01	P17643	TRP1	397	411	14.96
944	DQRVLIVRRNLLDLS	DRB1*08:04	P17643	TRP1	123	137	44.74
945	DQRVLIVRRNLLDLS	DRB1*11:02	P17643	TRP1	123	137	23
946	DQRVLIVRRNLLDLS	DRB1*11:03	P17643	TRP1	123	137	28.49
947	DQRVLIVRRNLLDLS	DRB1*11:04	P17643	TRP1	123	137	19.07
948	DQRVLIVRRNLLDLS	DRB1*11:13	P17643	TRP1	123	137	15.21
949	DQRVLIVRRNLLDLS	DRB1*11:42	P17643	TRP1	123	137	14.53
950	DQRVLIVRRNLLDLS	DRB1*11:46	P17643	TRP1	123	137	19.07
951	DQRVLIVRRNLLDLS	DRB1*11:58	P17643	TRP1	123	137	19.07
952	DQRVLIVRRNLLDLS	DRB1*11:65	P17643	TRP1	123	137	23
953	DQRVLIVRRNLLDLS	DRB1*11:84	P17643	TRP1	123	137	37.89
954	DQRVLIVRRNLLDLS	DRB1*13:01	P17643	TRP1	123	137	23
955	DQRVLIVRRNLLDLS	DRB1*13:11	P17643	TRP1	123	137	19.07
956	DQRVLIVRRNLLDLS	DRB1*13:21	P17643	TRP1	123	137	34.19

957	DQRVLIVRRNLLDLS	DRB1*13:61	P17643	TRP1	123	137	48.5
958	DQRVLIVRRNLLDLS	DRB1*14:01	P17643	TRP1	123	137	26.29
959	DQRVLIVRRNLLDLS	DRB1*14:04	P17643	TRP1	123	137	44.43
960	DQRVLIVRRNLLDLS	DRB1*14:06	P17643	TRP1	123	137	38.56
961	DQRVLIVRRNLLDLS	DRB1*14:32	P17643	TRP1	123	137	13.71
962	DQRVLIVRRNLLDLS	DRB1*14:54	P17643	TRP1	123	137	26.29
963	DQVPFSVSVSQLRAL	DRB1*01:18	P40967	PMEL17	211	225	45.05
964	DSDPARYEFLWGPRA	DRB1*01:01	P43355	MAGE1	256	270	20.69
965	DSDPARYEFLWGPRA	DRB1*01:18	P43355	MAGE1	256	270	21.7
966	DSSGTLISRALVVTH	DRB1*01:01	P40967	PMEL17	264	278	36.19
967	DSSGTLISRALVVTH	DRB1*01:18	P40967	PMEL17	264	278	33.84
968	DSSGTLISRALVVTH	DRB1*01:20	P40967	PMEL17	264	278	27.54
969	DSSGTLISRALVVTH	DRB1*07:01	P40967	PMEL17	264	278	24.3
970	DTLLGGFFPWLKVYY	DPB1*33:01	O75767	TRP2	195	209	29.79
971	DTLLGGFFPWLKVYY	DPB1*71:01	O75767	TRP2	195	209	29.79
972	DTPPFYSNSTNSFRN	DRB1*11:14	P17643	TRP1	343	357	25.79
973	DTPPFYSNSTNSFRN	DRB1*13:02	P17643	TRP1	343	357	25.79
974	DTPPFYSNSTNSFRN	DRB1*13:23	P17643	TRP1	343	357	25.79
975	DTPPFYSNSTNSFRN	DRB1*13:96	P17643	TRP1	343	357	45.85
976	DTPPFYSNSTNSFRN	DRB1*13:97	P17643	TRP1	343	357	25.79
977	DWLGVSRQLRTKAWN	DRB1*11:03	P40967	PMEL17	31	45	38.55
978	DWLGVSRQLRTKAWN	DRB1*11:04	P40967	PMEL17	31	45	44.48
979	DWLGVSRQLRTKAWN	DRB1*11:42	P40967	PMEL17	31	45	39.03
980	DWLGVSRQLRTKAWN	DRB1*11:46	P40967	PMEL17	31	45	44.48
981	DWLGVSRQLRTKAWN	DRB1*11:58	P40967	PMEL17	31	45	44.48
982	DWLGVSRQLRTKAWN	DRB1*13:11	P40967	PMEL17	31	45	44.48
983	DYIKSYLEQASRIWS	DRB1*01:01	P14679	Tyrosinase	462	476	30.54
984	DYIKSYLEQASRIWS	DRB1*01:18	P14679	Tyrosinase	462	476	26.19
985	DYIKSYLEQASRIWS	DRB1*01:20	P14679	Tyrosinase	462	476	44.8
986	EALGLVCVQAATSSS	DRB1*01:01	P43355	MAGE1	21	35	35.59
987	EALGLVCVQAATSSS	DRB1*01:18	P43355	MAGE1	21	35	33.61
988	EALGLVCVQAATSSS	DRB1*01:20	P43355	MAGE1	21	35	32.06
989	EALGLVCVQAATSSS	DRB1*10:01	P43355	MAGE1	21	35	41.78
990	EAPAFLPWHRLFLLR	DPB1*33:01	P14679	Tyrosinase	203	217	47.39
991	EAPAFLPWHRLFLLR	DPB1*71:01	P14679	Tyrosinase	203	217	47.39
992	ECDFQEFMAFVAMVT	DRB1*01:18	P04271	S100	66	80	32.52
993	EDGPIRRNPAGNVAR	DRB1*11:14	P17643	TRP1	307	321	32.26
994	EDGPIRRNPAGNVAR	DRB1*13:02	P17643	TRP1	307	321	32.26
995	EDGPIRRNPAGNVAR	DRB1*13:23	P17643	TRP1	307	321	32.26
996	EDGPIRRNPAGNVAR	DRB1*13:97	P17643	TRP1	307	321	32.26

997	EEKNHFVRALDMAKR	DRB1*01:18	P17643	TRP1	139	153	39.69
998	EEKNHFVRALDMAKR	DRB1*01:20	P17643	TRP1	139	153	38.13
999	EEWEKMKASEKIFYV	DRB1*11:13	Q16385	SSX2	35	49	49.35
1000	EEWEKMKASEKIFYV	DRB1*14:32	Q16385	SSX2	35	49	43.74
1001	EFMAFVAMVTTACHE	DRB1*01:01	P04271	S100	71	85	23.6
1002	EFMAFVAMVTTACHE	DRB1*01:11	P04271	S100	71	85	41.61
1003	EFMAFVAMVTTACHE	DRB1*01:18	P04271	S100	71	85	20.92
1004	EFMAFVAMVTTACHE	DRB1*01:24	P04271	S100	71	85	34.66
1005	EFMAFVAMVTTACHE	DRB1*01:29	P04271	S100	71	85	38.12
1006	EFMAFVAMVTTACHE	DRB1*04:04	P04271	S100	71	85	46.77
1007	EFMAFVAMVTTACHE	DRB1*04:08	P04271	S100	71	85	46.13
1008	EFMAFVAMVTTACHE	DRB1*10:01	P04271	S100	71	85	25.83
1009	EFTVSGNILTIRLTA	DRB1*01:01	P78358	NY-ESO-1	125	139	33.16
1010	EFTVSGNILTIRLTA	DRB1*01:18	P78358	NY-ESO-1	125	139	28.77
1011	EFYLAMPFATPMEAE	DPB1*33:01	P78358	NY-ESO-1	89	103	34.98
1012	EFYLAMPFATPMEAE	DPB1*71:01	P78358	NY-ESO-1	89	103	34.98
1013	EFYLAMPFATPMEAE	DRB1*01:01	P78358	NY-ESO-1	89	103	10.19
1014	EFYLAMPFATPMEAE	DRB1*01:11	P78358	NY-ESO-1	89	103	27.66
1015	EFYLAMPFATPMEAE	DRB1*01:18	P78358	NY-ESO-1	89	103	10.4
1016	EFYLAMPFATPMEAE	DRB1*01:20	P78358	NY-ESO-1	89	103	28.21
1017	EFYLAMPFATPMEAE	DRB1*01:24	P78358	NY-ESO-1	89	103	18.55
1018	EFYLAMPFATPMEAE	DRB1*01:29	P78358	NY-ESO-1	89	103	17.32
1019	EFYLAMPFATPMEAE	DRB1*09:01	P78358	NY-ESO-1	89	103	33.24
1020	EFYLAMPFATPMEAE	DRB1*10:01	P78358	NY-ESO-1	89	103	10.91
1021	EGPAFLTWHRYHLLR	DPB1*02:01	P17643	TRP1	216	230	16.72
1022	EGPAFLTWHRYHLLR	DPB1*02:02	P17643	TRP1	216	230	16.04
1023	EGPAFLTWHRYHLLR	DPB1*33:01	P17643	TRP1	216	230	8.3
1024	EGPAFLTWHRYHLLR	DPB1*41:01	P17643	TRP1	216	230	41.33
1025	EGPAFLTWHRYHLLR	DPB1*46:01	P17643	TRP1	216	230	16.72
1026	EGPAFLTWHRYHLLR	DPB1*47:01	P17643	TRP1	216	230	16.04
1027	EGPAFLTWHRYHLLR	DPB1*71:01	P17643	TRP1	216	230	8.3
1028	EGPAFLTWHRYHLLR	DPB1*81:01	P17643	TRP1	216	230	16.72
1029	EGPAFLTWHRYHLLR	DRB1*11:02	P17643	TRP1	216	230	44.44
1030	EGPAFLTWHRYHLLR	DRB1*11:03	P17643	TRP1	216	230	37.27
1031	EGPAFLTWHRYHLLR	DRB1*11:65	P17643	TRP1	216	230	44.44
1032	EGPAFLTWHRYHLLR	DRB1*13:01	P17643	TRP1	216	230	44.44
1033	EHVVRVNARVRIAYP	DRB1*01:20	P43358	MAGE4	289	303	45.61
1034	EHVVRVNARVRIAYP	DRB1*03:11	P43358	MAGE4	289	303	26.15
1035	EHVVRVNARVRIAYP	DRB1*11:02	P43358	MAGE4	289	303	11.58
1036	EHVVRVNARVRIAYP	DRB1*11:03	P43358	MAGE4	289	303	16.52

1037	EHVVRVNARVRIAYP	DRB1*11:04	P43358	MAGE4	289	303	35.82
1038	EHVVRVNARVRIAYP	DRB1*11:13	P43358	MAGE4	289	303	16.16
1039	EHVVRVNARVRIAYP	DRB1*11:14	P43358	MAGE4	289	303	20.84
1040	EHVVRVNARVRIAYP	DRB1*11:42	P43358	MAGE4	289	303	15.17
1041	EHVVRVNARVRIAYP	DRB1*11:46	P43358	MAGE4	289	303	35.82
1042	EHVVRVNARVRIAYP	DRB1*11:58	P43358	MAGE4	289	303	35.82
1043	EHVVRVNARVRIAYP	DRB1*11:65	P43358	MAGE4	289	303	11.58
1044	EHVVRVNARVRIAYP	DRB1*13:01	P43358	MAGE4	289	303	11.58
1045	EHVVRVNARVRIAYP	DRB1*13:02	P43358	MAGE4	289	303	20.84
1046	EHVVRVNARVRIAYP	DRB1*13:11	P43358	MAGE4	289	303	35.82
1047	EHVVRVNARVRIAYP	DRB1*13:23	P43358	MAGE4	289	303	20.84
1048	EHVVRVNARVRIAYP	DRB1*13:61	P43358	MAGE4	289	303	19.49
1049	EHVVRVNARVRIAYP	DRB1*13:96	P43358	MAGE4	289	303	28.4
1050	EHVVRVNARVRIAYP	DRB1*13:97	P43358	MAGE4	289	303	20.84
1051	EHVVRVNARVRIAYP	DRB1*14:06	P43358	MAGE4	289	303	27.45
1052	EHVVRVNARVRIAYP	DRB1*14:12	P43358	MAGE4	289	303	46.45
1053	EHVVRVNARVRIAYP	DRB1*14:32	P43358	MAGE4	289	303	21.91
1054	EKAMVALIDVFHQYS	DRB1*01:20	P04271	S100	3	17	41.09
1055	EKAMVALIDVFHQYS	DRB1*04:04	P04271	S100	3	17	27.71
1056	EKAMVALIDVFHQYS	DRB1*04:10	P04271	S100	3	17	39.03
1057	EKAMVALIDVFHQYS	DRB1*10:01	P04271	S100	3	17	41.59
1058	EKDKFFAYLTLAKHT	DPB1*02:01	P14679	Tyrosinase	130	144	49.43
1059	EKDKFFAYLTLAKHT	DPB1*02:02	P14679	Tyrosinase	130	144	41.62
1060	EKDKFFAYLTLAKHT	DPB1*33:01	P14679	Tyrosinase	130	144	22.68
1061	EKDKFFAYLTLAKHT	DPB1*46:01	P14679	Tyrosinase	130	144	49.43
1062	EKDKFFAYLTLAKHT	DPB1*47:01	P14679	Tyrosinase	130	144	41.62
1063	EKDKFFAYLTLAKHT	DPB1*71:01	P14679	Tyrosinase	130	144	22.68
1064	EKDKFFAYLTLAKHT	DPB1*81:01	P14679	Tyrosinase	130	144	49.43
1065	EKDKFFAYLTLAKHT	DRB1*01:01	P14679	Tyrosinase	130	144	25.93
1066	EKDKFFAYLTLAKHT	DRB1*01:18	P14679	Tyrosinase	130	144	20.08
1067	EKDKFFAYLTLAKHT	DRB1*01:20	P14679	Tyrosinase	130	144	37.89
1068	EKDKFFAYLTLAKHT	DRB1*01:29	P14679	Tyrosinase	130	144	47.03
1069	EKDKFFAYLTLAKHT	DRB1*10:01	P14679	Tyrosinase	130	144	10.21
1070	EKDKFFAYLTLAKHT	DRB1*13:21	P14679	Tyrosinase	130	144	39.49
1071	EKDKFFAYLTLAKHT	DRB1*15:15	P14679	Tyrosinase	130	144	31.94
1072	EKDKFFAYLTLAKHT	DRB1*16:09	P14679	Tyrosinase	130	144	38.06
1073	EKIFYVYMKRKYEAM	DRB1*08:01	Q16385	SSX2	44	58	37.24
1074	EKIFYVYMKRKYEAM	DRB1*08:04	Q16385	SSX2	44	58	45.57
1075	EKIFYVYMKRKYEAM	DRB1*08:24	Q16385	SSX2	44	58	43.31
1076	EKIFYVYMKRKYEAM	DRB1*11:01	Q16385	SSX2	44	58	12.58

1077	EKIFYVYMKRKYEAM	DRB1*11:02	Q16385	SSX2	44	58	27.64
1078	EKIFYVYMKRKYEAM	DRB1*11:03	Q16385	SSX2	44	58	13.38
1079	EKIFYVYMKRKYEAM	DRB1*11:04	Q16385	SSX2	44	58	12.34
1080	EKIFYVYMKRKYEAM	DRB1*11:08	Q16385	SSX2	44	58	31.94
1081	EKIFYVYMKRKYEAM	DRB1*11:10	Q16385	SSX2	44	58	12.58
1082	EKIFYVYMKRKYEAM	DRB1*11:11	Q16385	SSX2	44	58	27.25
1083	EKIFYVYMKRKYEAM	DRB1*11:12	Q16385	SSX2	44	58	12.58
1084	EKIFYVYMKRKYEAM	DRB1*11:13	Q16385	SSX2	44	58	33.65
1085	EKIFYVYMKRKYEAM	DRB1*11:27	Q16385	SSX2	44	58	39.9
1086	EKIFYVYMKRKYEAM	DRB1*11:28	Q16385	SSX2	44	58	12.58
1087	EKIFYVYMKRKYEAM	DRB1*11:29	Q16385	SSX2	44	58	12.58
1088	EKIFYVYMKRKYEAM	DRB1*11:37	Q16385	SSX2	44	58	28.09
1089	EKIFYVYMKRKYEAM	DRB1*11:42	Q16385	SSX2	44	58	18.11
1090	EKIFYVYMKRKYEAM	DRB1*11:46	Q16385	SSX2	44	58	12.34
1091	EKIFYVYMKRKYEAM	DRB1*11:49	Q16385	SSX2	44	58	12.58
1092	EKIFYVYMKRKYEAM	DRB1*11:54	Q16385	SSX2	44	58	49.95
1093	EKIFYVYMKRKYEAM	DRB1*11:58	Q16385	SSX2	44	58	12.34
1094	EKIFYVYMKRKYEAM	DRB1*11:62	Q16385	SSX2	44	58	12.58
1095	EKIFYVYMKRKYEAM	DRB1*11:65	Q16385	SSX2	44	58	27.64
1096	EKIFYVYMKRKYEAM	DRB1*11:74	Q16385	SSX2	44	58	12.58
1097	EKIFYVYMKRKYEAM	DRB1*11:84	Q16385	SSX2	44	58	34.86
1098	EKIFYVYMKRKYEAM	DRB1*13:01	Q16385	SSX2	44	58	27.64
1099	EKIFYVYMKRKYEAM	DRB1*13:05	Q16385	SSX2	44	58	12.58
1100	EKIFYVYMKRKYEAM	DRB1*13:07	Q16385	SSX2	44	58	28.09
1101	EKIFYVYMKRKYEAM	DRB1*13:11	Q16385	SSX2	44	58	12.34
1102	EKIFYVYMKRKYEAM	DRB1*13:14	Q16385	SSX2	44	58	12.58
1103	EKIFYVYMKRKYEAM	DRB1*13:21	Q16385	SSX2	44	58	8.54
1104	EKIFYVYMKRKYEAM	DRB1*13:50	Q16385	SSX2	44	58	12.58
1105	EKMKASEKIFYVYMK	DRB1*03:11	Q16385	SSX2	38	52	48.68
1106	EKMKASEKIFYVYMK	DRB1*11:13	Q16385	SSX2	38	52	37.62
1107	EKMKASEKIFYVYMK	DRB1*11:42	Q16385	SSX2	38	52	42.26
1108	EKMKASEKIFYVYMK	DRB1*14:32	Q16385	SSX2	38	52	40.87
1109	EKNHFVRALDMAKRT	DRB1*01:01	P17643	TRP1	140	154	45.85
1110	EKNHFVRALDMAKRT	DRB1*01:18	P17643	TRP1	140	154	33.42
1111	EKNHFVRALDMAKRT	DRB1*01:20	P17643	TRP1	140	154	33.21
1112	EKNHFVRALDMAKRT	DRB1*10:01	P17643	TRP1	140	154	47.51
1113	EKNHFVRALDMAKRT	DRB1*11:42	P17643	TRP1	140	154	47.57
1114	ELAHFLLRKYRAKEL	DRB1*11:01	P43358	MAGE4	116	130	28.81
1115	ELAHFLLRKYRAKEL	DRB1*11:02	P43358	MAGE4	116	130	34.56
1116	ELAHFLLRKYRAKEL	DRB1*11:03	P43358	MAGE4	116	130	19.32

1117	ELAHFLLRKYRAKEL	DRB1*11:04	P43358	MAGE4	116	130	19.95
1118	ELAHFLLRKYRAKEL	DRB1*11:10	P43358	MAGE4	116	130	28.81
1119	ELAHFLLRKYRAKEL	DRB1*11:12	P43358	MAGE4	116	130	28.81
1120	ELAHFLLRKYRAKEL	DRB1*11:13	P43358	MAGE4	116	130	49.37
1121	ELAHFLLRKYRAKEL	DRB1*11:28	P43358	MAGE4	116	130	28.81
1122	ELAHFLLRKYRAKEL	DRB1*11:29	P43358	MAGE4	116	130	28.81
1123	ELAHFLLRKYRAKEL	DRB1*11:42	P43358	MAGE4	116	130	25.66
1124	ELAHFLLRKYRAKEL	DRB1*11:46	P43358	MAGE4	116	130	19.95
1125	ELAHFLLRKYRAKEL	DRB1*11:49	P43358	MAGE4	116	130	28.81
1126	ELAHFLLRKYRAKEL	DRB1*11:58	P43358	MAGE4	116	130	19.95
1127	ELAHFLLRKYRAKEL	DRB1*11:62	P43358	MAGE4	116	130	28.81
1128	ELAHFLLRKYRAKEL	DRB1*11:65	P43358	MAGE4	116	130	34.56
1129	ELAHFLLRKYRAKEL	DRB1*11:74	P43358	MAGE4	116	130	28.81
1130	ELAHFLLRKYRAKEL	DRB1*12:02	P43358	MAGE4	116	130	47.63
1131	ELAHFLLRKYRAKEL	DRB1*13:01	P43358	MAGE4	116	130	34.56
1132	ELAHFLLRKYRAKEL	DRB1*13:05	P43358	MAGE4	116	130	28.81
1133	ELAHFLLRKYRAKEL	DRB1*13:11	P43358	MAGE4	116	130	19.95
1134	ELAHFLLRKYRAKEL	DRB1*13:14	P43358	MAGE4	116	130	28.81
1135	ELAHFLLRKYRAKEL	DRB1*13:21	P43358	MAGE4	116	130	22.48
1136	ELAHFLLRKYRAKEL	DRB1*13:50	P43358	MAGE4	116	130	28.81
1137	ELAHFLLRKYRAKEL	DRB1*15:01	P43358	MAGE4	116	130	26.43
1138	ELAHFLLRKYRAKEL	DRB1*15:03	P43358	MAGE4	116	130	34.91
1139	ELAHFLLRKYRAKEL	DRB1*15:06	P43358	MAGE4	116	130	26.43
1140	ELAHFLLRKYRAKEL	DRB1*15:15	P43358	MAGE4	116	130	47.38
1141	ELEKAMVALIDVFHQ	DRB1*01:20	P04271	S100	1	15	48.56
1142	ELKELINNELSHFLE	DRB1*01:18	P04271	S100	30	44	49.73
1143	ELKELINNELSHFLE	DRB1*01:20	P04271	S100	30	44	40.73
1144	ELKELINNELSHFLE	DRB1*11:14	P04271	S100	30	44	36.11
1145	ELKELINNELSHFLE	DRB1*13:02	P04271	S100	30	44	36.11
1146	ELKELINNELSHFLE	DRB1*13:23	P04271	S100	30	44	36.11
1147	ELKELINNELSHFLE	DRB1*13:96	P04271	S100	30	44	46.72
1148	ELKELINNELSHFLE	DRB1*13:97	P04271	S100	30	44	36.11
1149	EMFVTAPDNLGYTYE	DRB1*04:01	P17643	TRP1	451	465	22.96
1150	EMFVTAPDNLGYTYE	DRB1*04:08	P17643	TRP1	451	465	25.27
1151	EMLERVIKNYKRCFP	DRB1*11:02	P43358	MAGE4	135	149	40.71
1152	EMLERVIKNYKRCFP	DRB1*11:03	P43358	MAGE4	135	149	33.57
1153	EMLERVIKNYKRCFP	DRB1*11:42	P43358	MAGE4	135	149	40.72
1154	EMLERVIKNYKRCFP	DRB1*11:65	P43358	MAGE4	135	149	40.71
1155	EMLERVIKNYKRCFP	DRB1*13:01	P43358	MAGE4	135	149	40.71
1156	EQASRIWSWLLGAAM	DRB1*01:01	P14679	Tyrosinase	469	483	25.47

1157	EQASRIWSWLLGAAM	DRB1*01:18	P14679	Tyrosinase	469	483	21.71
1158	EQASRIWSWLLGAAM	DRB1*01:24	P14679	Tyrosinase	469	483	49.4
1159	EQASRIWSWLLGAAM	DRB1*10:01	P14679	Tyrosinase	469	483	47.39
1160	EREQFLGALDLAKKR	DRB1*01:18	O75767	TRP2	137	151	46.86
1161	EREQFLGALDLAKKR	DRB1*01:20	O75767	TRP2	137	151	49.71
1162	ERVIKNYKRCFPVIF	DRB1*11:02	P43358	MAGE4	138	152	35.86
1163	ERVIKNYKRCFPVIF	DRB1*11:03	P43358	MAGE4	138	152	31.71
1164	ERVIKNYKRCFPVIF	DRB1*11:42	P43358	MAGE4	138	152	46.84
1165	ERVIKNYKRCFPVIF	DRB1*11:65	P43358	MAGE4	138	152	35.86
1166	ERVIKNYKRCFPVIF	DRB1*13:01	P43358	MAGE4	138	152	35.86
1167	ESLFRAVITKKVADL	DRB1*01:01	P43355	MAGE1	95	109	6.03
1168	ESLFRAVITKKVADL	DRB1*01:11	P43355	MAGE1	95	109	14.3
1169	ESLFRAVITKKVADL	DRB1*01:18	P43355	MAGE1	95	109	5.72
1170	ESLFRAVITKKVADL	DRB1*01:20	P43355	MAGE1	95	109	16.38
1171	ESLFRAVITKKVADL	DRB1*01:24	P43355	MAGE1	95	109	12.26
1172	ESLFRAVITKKVADL	DRB1*01:29	P43355	MAGE1	95	109	9.45
1173	ESLFRAVITKKVADL	DRB1*07:01	P43355	MAGE1	95	109	8.52
1174	ESLFRAVITKKVADL	DRB1*09:01	P43355	MAGE1	95	109	21.43
1175	ESLFRAVITKKVADL	DRB1*10:01	P43355	MAGE1	95	109	13.58
1176	ESLFRAVITKKVADL	DRB1*11:01	P43355	MAGE1	95	109	29.13
1177	ESLFRAVITKKVADL	DRB1*11:04	P43355	MAGE1	95	109	44.48
1178	ESLFRAVITKKVADL	DRB1*11:08	P43355	MAGE1	95	109	34.07
1179	ESLFRAVITKKVADL	DRB1*11:10	P43355	MAGE1	95	109	29.13
1180	ESLFRAVITKKVADL	DRB1*11:12	P43355	MAGE1	95	109	29.13
1181	ESLFRAVITKKVADL	DRB1*11:28	P43355	MAGE1	95	109	29.13
1182	ESLFRAVITKKVADL	DRB1*11:29	P43355	MAGE1	95	109	29.13
1183	ESLFRAVITKKVADL	DRB1*11:42	P43355	MAGE1	95	109	35.53
1184	ESLFRAVITKKVADL	DRB1*11:46	P43355	MAGE1	95	109	44.48
1185	ESLFRAVITKKVADL	DRB1*11:49	P43355	MAGE1	95	109	29.13
1186	ESLFRAVITKKVADL	DRB1*11:58	P43355	MAGE1	95	109	44.48
1187	ESLFRAVITKKVADL	DRB1*11:62	P43355	MAGE1	95	109	29.13
1188	ESLFRAVITKKVADL	DRB1*11:74	P43355	MAGE1	95	109	29.13
1189	ESLFRAVITKKVADL	DRB1*12:16	P43355	MAGE1	95	109	43.26
1190	ESLFRAVITKKVADL	DRB1*13:05	P43355	MAGE1	95	109	29.13
1191	ESLFRAVITKKVADL	DRB1*13:11	P43355	MAGE1	95	109	44.48
1192	ESLFRAVITKKVADL	DRB1*13:14	P43355	MAGE1	95	109	29.13
1193	ESLFRAVITKKVADL	DRB1*13:21	P43355	MAGE1	95	109	28.35
1194	ESLFRAVITKKVADL	DRB1*13:50	P43355	MAGE1	95	109	29.13
1195	ESLFRAVITKKVADL	DRB1*15:15	P43355	MAGE1	95	109	23.9
1196	ESLFRAVITKKVADL	DRB1*16:01	P43355	MAGE1	95	109	19

1197	ESLFRAVITKKVADL	DRB1*16:02	P43355	MAGE1	95	109	18.02
1198	ESLFRAVITKKVADL	DRB1*16:04	P43355	MAGE1	95	109	30.81
1199	ESLFRAVITKKVADL	DRB1*16:05	P43355	MAGE1	95	109	40.22
1200	ESLFRAVITKKVADL	DRB1*16:09	P43355	MAGE1	95	109	18.57
1201	ESLFREALSNKVDEL	DRB1*01:01	P43358	MAGE4	103	117	4.49
1202	ESLFREALSNKVDEL	DRB1*01:11	P43358	MAGE4	103	117	10.86
1203	ESLFREALSNKVDEL	DRB1*01:18	P43358	MAGE4	103	117	4.95
1204	ESLFREALSNKVDEL	DRB1*01:20	P43358	MAGE4	103	117	15.57
1205	ESLFREALSNKVDEL	DRB1*01:24	P43358	MAGE4	103	117	9.95
1206	ESLFREALSNKVDEL	DRB1*01:29	P43358	MAGE4	103	117	9.05
1207	ESLFREALSNKVDEL	DRB1*04:01	P43358	MAGE4	103	117	49.61
1208	ESLFREALSNKVDEL	DRB1*07:01	P43358	MAGE4	103	117	7.59
1209	ESLFREALSNKVDEL	DRB1*09:01	P43358	MAGE4	103	117	12.12
1210	ESLFREALSNKVDEL	DRB1*10:01	P43358	MAGE4	103	117	13.38
1211	ESLFREALSNKVDEL	DRB1*11:14	P43358	MAGE4	103	117	46.34
1212	ESLFREALSNKVDEL	DRB1*13:02	P43358	MAGE4	103	117	46.34
1213	ESLFREALSNKVDEL	DRB1*13:23	P43358	MAGE4	103	117	46.34
1214	ESLFREALSNKVDEL	DRB1*13:97	P43358	MAGE4	103	117	46.34
1215	ESLFREALSNKVDEL	DRB1*15:02	P43358	MAGE4	103	117	45.16
1216	ESLFREALSNKVDEL	DRB1*15:15	P43358	MAGE4	103	117	39.09
1217	ESLFREALSNKVDEL	DRB1*16:01	P43358	MAGE4	103	117	33.94
1218	ESLFREALSNKVDEL	DRB1*16:02	P43358	MAGE4	103	117	17.65
1219	ESLFREALSNKVDEL	DRB1*16:05	P43358	MAGE4	103	117	41.35
1220	ESLFREALSNKVDEL	DRB1*16:09	P43358	MAGE4	103	117	48.25
1221	ESLKMIFGIDVKEVD	DRB1*01:01	P43358	MAGE4	157	171	12
1222	ESLKMIFGIDVKEVD	DRB1*01:02	P43358	MAGE4	157	171	28.36
1223	ESLKMIFGIDVKEVD	DRB1*01:11	P43358	MAGE4	157	171	37.13
1224	ESLKMIFGIDVKEVD	DRB1*01:18	P43358	MAGE4	157	171	11.17
1225	ESLKMIFGIDVKEVD	DRB1*01:20	P43358	MAGE4	157	171	9.92
1226	ESLKMIFGIDVKEVD	DRB1*01:24	P43358	MAGE4	157	171	21.72
1227	ESLKMIFGIDVKEVD	DRB1*01:29	P43358	MAGE4	157	171	24.59
1228	ESLQLVFGIDVKEAD	DRB1*01:01	P43355	MAGE1	149	163	33.58
1229	ESLQLVFGIDVKEAD	DRB1*01:18	P43355	MAGE1	149	163	24.5
1230	ESLQLVFGIDVKEAD	DRB1*01:20	P43355	MAGE1	149	163	25.16
1231	ESRLLEFYLAMPFAT	DPB1*01:01	P78358	NY-ESO-1	84	98	45.32
1232	ESRLLEFYLAMPFAT	DPB1*15:01	P78358	NY-ESO-1	84	98	43.89
1233	ESRLLEFYLAMPFAT	DPB1*33:01	P78358	NY-ESO-1	84	98	25.01
1234	ESRLLEFYLAMPFAT	DPB1*71:01	P78358	NY-ESO-1	84	98	25.01
1235	ESRLLEFYLAMPFAT	DRB1*01:01	P78358	NY-ESO-1	84	98	25.98
1236	ESRLLEFYLAMPFAT	DRB1*01:18	P78358	NY-ESO-1	84	98	20.78

1237	ESRLLEFYLAMPFAT	DRB1*01:24	P78358	NY-ESO-1	84	98	31.04
1238	ESRLLEFYLAMPFAT	DRB1*01:29	P78358	NY-ESO-1	84	98	40.29
1239	ESRLLEFYLAMPFAT	DRB1*10:01	P78358	NY-ESO-1	84	98	26.61
1240	ESRLLEFYLAMPFAT	DRB1*15:01	P78358	NY-ESO-1	84	98	36.32
1241	ESRLLEFYLAMPFAT	DRB1*15:06	P78358	NY-ESO-1	84	98	36.32
1242	ESYMVPFIPLYRNGD	DPB1*33:01	P14679	Tyrosinase	423	437	47.37
1243	ESYMVPFIPLYRNGD	DPB1*71:01	P14679	Tyrosinase	423	437	47.37
1244	ESYMVPFIPLYRNGD	DRB1*11:13	P14679	Tyrosinase	423	437	47.63
1245	ESYMVPFIPLYRNGD	DRB1*11:42	P14679	Tyrosinase	423	437	45.74
1246	ESYMVPFIPLYRNGD	DRB1*13:21	P14679	Tyrosinase	423	437	32.19
1247	ETSYVKVLEHVVRVN	DRB1*01:01	P43358	MAGE4	281	295	41.6
1248	ETSYVKVLEHVVRVN	DRB1*01:18	P43358	MAGE4	281	295	27.89
1249	ETSYVKVLEHVVRVN	DRB1*11:13	P43358	MAGE4	281	295	48.55
1250	ETSYVKVLEHVVRVN	DRB1*11:42	P43358	MAGE4	281	295	47.49
1251	ETSYVKVLEYVIKVS	DRB1*01:18	P43355	MAGE1	273	287	27.29
1252	EVDFSHEGPAFLTWH	DRB1*01:01	P17643	TRP1	210	224	33.44
1253	EVDFSHEGPAFLTWH	DRB1*01:18	P17643	TRP1	210	224	45.08
1254	EVISCKLIKRATTRQ	DRB1*08:04	O75767	TRP2	222	236	36.35
1255	EVISCKLIKRATTRQ	DRB1*11:01	O75767	TRP2	222	236	30.92
1256	EVISCKLIKRATTRQ	DRB1*11:02	O75767	TRP2	222	236	39.78
1257	EVISCKLIKRATTRQ	DRB1*11:03	O75767	TRP2	222	236	25.88
1258	EVISCKLIKRATTRQ	DRB1*11:04	O75767	TRP2	222	236	19.29
1259	EVISCKLIKRATTRQ	DRB1*11:10	O75767	TRP2	222	236	30.92
1260	EVISCKLIKRATTRQ	DRB1*11:12	O75767	TRP2	222	236	30.92
1261	EVISCKLIKRATTRQ	DRB1*11:13	O75767	TRP2	222	236	46.1
1262	EVISCKLIKRATTRQ	DRB1*11:28	O75767	TRP2	222	236	30.92
1263	EVISCKLIKRATTRQ	DRB1*11:29	O75767	TRP2	222	236	30.92
1264	EVISCKLIKRATTRQ	DRB1*11:42	O75767	TRP2	222	236	22.36
1265	EVISCKLIKRATTRQ	DRB1*11:46	O75767	TRP2	222	236	19.29
1266	EVISCKLIKRATTRQ	DRB1*11:49	O75767	TRP2	222	236	30.92
1267	EVISCKLIKRATTRQ	DRB1*11:58	O75767	TRP2	222	236	19.29
1268	EVISCKLIKRATTRQ	DRB1*11:62	O75767	TRP2	222	236	30.92
1269	EVISCKLIKRATTRQ	DRB1*11:65	O75767	TRP2	222	236	39.78
1270	EVISCKLIKRATTRQ	DRB1*11:74	O75767	TRP2	222	236	30.92
1271	EVISCKLIKRATTRQ	DRB1*11:84	O75767	TRP2	222	236	46.41
1272	EVISCKLIKRATTRQ	DRB1*13:01	O75767	TRP2	222	236	39.78
1273	EVISCKLIKRATTRQ	DRB1*13:05	O75767	TRP2	222	236	30.92
1274	EVISCKLIKRATTRQ	DRB1*13:11	O75767	TRP2	222	236	19.29
1275	EVISCKLIKRATTRQ	DRB1*13:14	O75767	TRP2	222	236	30.92
1276	EVISCKLIKRATTRQ	DRB1*13:50	O75767	TRP2	222	236	30.92

1277	EVRADTRPWSGPYIL	DRB1*03:11	O75767	TRP2	63	77	40.17
1278	EVSIVVLSGTTAAQV	DRB1*01:01	P40967	PMEL17	404	418	9.77
1279	EVSIVVLSGTTAAQV	DRB1*01:02	P40967	PMEL17	404	418	44.64
1280	EVSIVVLSGTTAAQV	DRB1*01:11	P40967	PMEL17	404	418	19.29
1281	EVSIVVLSGTTAAQV	DRB1*01:18	P40967	PMEL17	404	418	8.87
1282	EVSIVVLSGTTAAQV	DRB1*01:20	P40967	PMEL17	404	418	10.55
1283	EVSIVVLSGTTAAQV	DRB1*01:24	P40967	PMEL17	404	418	17.34
1284	EVSIVVLSGTTAAQV	DRB1*01:29	P40967	PMEL17	404	418	18.37
1285	EVSIVVLSGTTAAQV	DRB1*04:04	P40967	PMEL17	404	418	27.59
1286	EVSIVVLSGTTAAQV	DRB1*07:01	P40967	PMEL17	404	418	35.86
1287	EVSIVVLSGTTAAQV	DRB1*10:01	P40967	PMEL17	404	418	39.05
1288	EVSIVVLSGTTAAQV	DRB1*11:14	P40967	PMEL17	404	418	49.09
1289	EVSIVVLSGTTAAQV	DRB1*13:02	P40967	PMEL17	404	418	49.09
1290	EVSIVVLSGTTAAQV	DRB1*13:23	P40967	PMEL17	404	418	49.09
1291	EVSIVVLSGTTAAQV	DRB1*13:97	P40967	PMEL17	404	418	49.09
1292	EVTVYHRRGSRSYVP	DRB1*11:02	P40967	PMEL17	185	199	47.92
1293	EVTVYHRRGSRSYVP	DRB1*11:03	P40967	PMEL17	185	199	27.77
1294	EVTVYHRRGSRSYVP	DRB1*11:65	P40967	PMEL17	185	199	47.92
1295	EVTVYHRRGSRSYVP	DRB1*13:01	P40967	PMEL17	185	199	47.92
1296	EVWPLRFFNRTCHCN	DRB1*11:03	P17643	TRP1	88	102	47.38
1297	EWEKMKASEKIFYVY	DRB1*03:11	Q16385	SSX2	36	50	46.52
1298	EWEKMKASEKIFYVY	DRB1*11:13	Q16385	SSX2	36	50	35.83
1299	EWEKMKASEKIFYVY	DRB1*11:42	Q16385	SSX2	36	50	39.75
1300	EWEKMKASEKIFYVY	DRB1*14:32	Q16385	SSX2	36	50	36.3
1301	EYVIKVSARVRFFFP	DRB1*01:01	P43355	MAGE1	281	295	13.52
1302	EYVIKVSARVRFFFP	DRB1*01:11	P43355	MAGE1	281	295	25.2
1303	EYVIKVSARVRFFFP	DRB1*01:18	P43355	MAGE1	281	295	9.76
1304	EYVIKVSARVRFFFP	DRB1*01:20	P43355	MAGE1	281	295	21.35
1305	EYVIKVSARVRFFFP	DRB1*01:24	P43355	MAGE1	281	295	25.2
1306	EYVIKVSARVRFFFP	DRB1*01:29	P43355	MAGE1	281	295	18.53
1307	EYVIKVSARVRFFFP	DRB1*03:01	P43355	MAGE1	281	295	36.32
1308	EYVIKVSARVRFFFP	DRB1*03:04	P43355	MAGE1	281	295	36.32
1309	EYVIKVSARVRFFFP	DRB1*03:11	P43355	MAGE1	281	295	18.21
1310	EYVIKVSARVRFFFP	DRB1*03:13	P43355	MAGE1	281	295	36.32
1311	EYVIKVSARVRFFFP	DRB1*03:15	P43355	MAGE1	281	295	32.7
1312	EYVIKVSARVRFFFP	DRB1*07:01	P43355	MAGE1	281	295	43.67
1313	EYVIKVSARVRFFFP	DRB1*08:04	P43355	MAGE1	281	295	36.25
1314	EYVIKVSARVRFFFP	DRB1*11:01	P43355	MAGE1	281	295	39.57
1315	EYVIKVSARVRFFFP	DRB1*11:02	P43355	MAGE1	281	295	9.36
1316	EYVIKVSARVRFFFP	DRB1*11:03	P43355	MAGE1	281	295	17.74

1317	EYVIKVSARVRFFFP	DRB1*11:04	P43355	MAGE1	281	295	17.68
1318	EYVIKVSARVRFFFP	DRB1*11:07	P43355	MAGE1	281	295	44.78
1319	EYVIKVSARVRFFFP	DRB1*11:08	P43355	MAGE1	281	295	34.14
1320	EYVIKVSARVRFFFP	DRB1*11:10	P43355	MAGE1	281	295	39.57
1321	EYVIKVSARVRFFFP	DRB1*11:12	P43355	MAGE1	281	295	39.57
1322	EYVIKVSARVRFFFP	DRB1*11:13	P43355	MAGE1	281	295	10.34
1323	EYVIKVSARVRFFFP	DRB1*11:14	P43355	MAGE1	281	295	26.42
1324	EYVIKVSARVRFFFP	DRB1*11:28	P43355	MAGE1	281	295	39.57
1325	EYVIKVSARVRFFFP	DRB1*11:29	P43355	MAGE1	281	295	39.57
1326	EYVIKVSARVRFFFP	DRB1*11:42	P43355	MAGE1	281	295	10.6
1327	EYVIKVSARVRFFFP	DRB1*11:46	P43355	MAGE1	281	295	17.68
1328	EYVIKVSARVRFFFP	DRB1*11:49	P43355	MAGE1	281	295	39.57
1329	EYVIKVSARVRFFFP	DRB1*11:58	P43355	MAGE1	281	295	17.68
1330	EYVIKVSARVRFFFP	DRB1*11:62	P43355	MAGE1	281	295	39.57
1331	EYVIKVSARVRFFFP	DRB1*11:65	P43355	MAGE1	281	295	9.36
1332	EYVIKVSARVRFFFP	DRB1*11:74	P43355	MAGE1	281	295	39.57
1333	EYVIKVSARVRFFFP	DRB1*11:84	P43355	MAGE1	281	295	28.25
1334	EYVIKVSARVRFFFP	DRB1*12:03	P43355	MAGE1	281	295	38.64
1335	EYVIKVSARVRFFFP	DRB1*13:01	P43355	MAGE1	281	295	9.36
1336	EYVIKVSARVRFFFP	DRB1*13:02	P43355	MAGE1	281	295	26.42
1337	EYVIKVSARVRFFFP	DRB1*13:05	P43355	MAGE1	281	295	39.57
1338	EYVIKVSARVRFFFP	DRB1*13:11	P43355	MAGE1	281	295	17.68
1339	EYVIKVSARVRFFFP	DRB1*13:14	P43355	MAGE1	281	295	39.57
1340	EYVIKVSARVRFFFP	DRB1*13:21	P43355	MAGE1	281	295	40.17
1341	EYVIKVSARVRFFFP	DRB1*13:23	P43355	MAGE1	281	295	26.42
1342	EYVIKVSARVRFFFP	DRB1*13:50	P43355	MAGE1	281	295	39.57
1343	EYVIKVSARVRFFFP	DRB1*13:61	P43355	MAGE1	281	295	18.53
1344	EYVIKVSARVRFFFP	DRB1*13:96	P43355	MAGE1	281	295	38.38
1345	EYVIKVSARVRFFFP	DRB1*13:97	P43355	MAGE1	281	295	26.42
1346	EYVIKVSARVRFFFP	DRB1*14:01	P43355	MAGE1	281	295	21.27
1347	EYVIKVSARVRFFFP	DRB1*14:04	P43355	MAGE1	281	295	37.23
1348	EYVIKVSARVRFFFP	DRB1*14:05	P43355	MAGE1	281	295	43.67
1349	EYVIKVSARVRFFFP	DRB1*14:06	P43355	MAGE1	281	295	24.03
1350	EYVIKVSARVRFFFP	DRB1*14:12	P43355	MAGE1	281	295	30.77
1351	EYVIKVSARVRFFFP	DRB1*14:23	P43355	MAGE1	281	295	43.67
1352	EYVIKVSARVRFFFP	DRB1*14:32	P43355	MAGE1	281	295	13.06
1353	EYVIKVSARVRFFFP	DRB1*14:38	P43355	MAGE1	281	295	39.37
1354	EYVIKVSARVRFFFP	DRB1*14:54	P43355	MAGE1	281	295	21.27
1355	EYVIKVSARVRFFFP	DRB1*16:09	P43355	MAGE1	281	295	42.89
1356	FATPMEAELARRSLA	DRB1*01:20	P78358	NY-ESO-1	96	110	40.84

1357	FAYLTLAKHTISSDY	DRB1*01:01	P14679	Tyrosinase	135	149	36.68
1358	FAYLTLAKHTISSDY	DRB1*01:18	P14679	Tyrosinase	135	149	32.03
1359	FAYLTLAKHTISSDY	DRB1*01:20	P14679	Tyrosinase	135	149	34.87
1360	FAYLTLAKHTISSDY	DRB1*08:04	P14679	Tyrosinase	135	149	38.34
1361	FAYLTLAKHTISSDY	DRB1*10:01	P14679	Tyrosinase	135	149	44.28
1362	FAYLTLAKHTISSDY	DRB1*11:01	P14679	Tyrosinase	135	149	35.24
1363	FAYLTLAKHTISSDY	DRB1*11:04	P14679	Tyrosinase	135	149	27.46
1364	FAYLTLAKHTISSDY	DRB1*11:10	P14679	Tyrosinase	135	149	35.24
1365	FAYLTLAKHTISSDY	DRB1*11:12	P14679	Tyrosinase	135	149	35.24
1366	FAYLTLAKHTISSDY	DRB1*11:13	P14679	Tyrosinase	135	149	45.35
1367	FAYLTLAKHTISSDY	DRB1*11:28	P14679	Tyrosinase	135	149	35.24
1368	FAYLTLAKHTISSDY	DRB1*11:29	P14679	Tyrosinase	135	149	35.24
1369	FAYLTLAKHTISSDY	DRB1*11:42	P14679	Tyrosinase	135	149	31.03
1370	FAYLTLAKHTISSDY	DRB1*11:46	P14679	Tyrosinase	135	149	27.46
1371	FAYLTLAKHTISSDY	DRB1*11:49	P14679	Tyrosinase	135	149	35.24
1372	FAYLTLAKHTISSDY	DRB1*11:58	P14679	Tyrosinase	135	149	27.46
1373	FAYLTLAKHTISSDY	DRB1*11:62	P14679	Tyrosinase	135	149	35.24
1374	FAYLTLAKHTISSDY	DRB1*11:74	P14679	Tyrosinase	135	149	35.24
1375	FAYLTLAKHTISSDY	DRB1*13:05	P14679	Tyrosinase	135	149	35.24
1376	FAYLTLAKHTISSDY	DRB1*13:11	P14679	Tyrosinase	135	149	27.46
1377	FAYLTLAKHTISSDY	DRB1*13:14	P14679	Tyrosinase	135	149	35.24
1378	FAYLTLAKHTISSDY	DRB1*13:21	P14679	Tyrosinase	135	149	38.61
1379	FAYLTLAKHTISSDY	DRB1*13:50	P14679	Tyrosinase	135	149	35.24
1380	FDTPPFYSNSTNSFR	DRB1*11:14	P17643	TRP1	342	356	28.28
1381	FDTPPFYSNSTNSFR	DRB1*13:02	P17643	TRP1	342	356	28.28
1382	FDTPPFYSNSTNSFR	DRB1*13:23	P17643	TRP1	342	356	28.28
1383	FDTPPFYSNSTNSFR	DRB1*13:97	P17643	TRP1	342	356	28.28
1384	FEQWLRRHRPLQEVY	DRB1*11:03	P14679	Tyrosinase	397	411	47.11
1385	FFAYLTLAKHTISSD	DRB1*01:01	P14679	Tyrosinase	134	148	27.66
1386	FFAYLTLAKHTISSD	DRB1*01:18	P14679	Tyrosinase	134	148	21
1387	FFAYLTLAKHTISSD	DRB1*01:20	P14679	Tyrosinase	134	148	33.11
1388	FFAYLTLAKHTISSD	DRB1*01:29	P14679	Tyrosinase	134	148	45.71
1389	FFAYLTLAKHTISSD	DRB1*08:04	P14679	Tyrosinase	134	148	46.68
1390	FFAYLTLAKHTISSD	DRB1*10:01	P14679	Tyrosinase	134	148	16.85
1391	FFAYLTLAKHTISSD	DRB1*11:01	P14679	Tyrosinase	134	148	28.57
1392	FFAYLTLAKHTISSD	DRB1*11:04	P14679	Tyrosinase	134	148	28.42
1393	FFAYLTLAKHTISSD	DRB1*11:08	P14679	Tyrosinase	134	148	42.23
1394	FFAYLTLAKHTISSD	DRB1*11:10	P14679	Tyrosinase	134	148	28.57
1395	FFAYLTLAKHTISSD	DRB1*11:12	P14679	Tyrosinase	134	148	28.57
1396	FFAYLTLAKHTISSD	DRB1*11:13	P14679	Tyrosinase	134	148	46.72

1397	FFAYLTLAKHTISSD	DRB1*11:28	P14679	Tyrosinase	134	148	28.57
1398	FFAYLTLAKHTISSD	DRB1*11:29	P14679	Tyrosinase	134	148	28.57
1399	FFAYLTLAKHTISSD	DRB1*11:42	P14679	Tyrosinase	134	148	33.14
1400	FFAYLTLAKHTISSD	DRB1*11:46	P14679	Tyrosinase	134	148	28.42
1401	FFAYLTLAKHTISSD	DRB1*11:49	P14679	Tyrosinase	134	148	28.57
1402	FFAYLTLAKHTISSD	DRB1*11:58	P14679	Tyrosinase	134	148	28.42
1403	FFAYLTLAKHTISSD	DRB1*11:62	P14679	Tyrosinase	134	148	28.57
1404	FFAYLTLAKHTISSD	DRB1*11:74	P14679	Tyrosinase	134	148	28.57
1405	FFAYLTLAKHTISSD	DRB1*13:05	P14679	Tyrosinase	134	148	28.57
1406	FFAYLTLAKHTISSD	DRB1*13:11	P14679	Tyrosinase	134	148	28.42
1407	FFAYLTLAKHTISSD	DRB1*13:14	P14679	Tyrosinase	134	148	28.57
1408	FFAYLTLAKHTISSD	DRB1*13:21	P14679	Tyrosinase	134	148	28.46
1409	FFAYLTLAKHTISSD	DRB1*13:50	P14679	Tyrosinase	134	148	28.57
1410	FFFPSLREAALREEE	DRB1*11:01	P43355	MAGE1	292	306	45.29
1411	FFFPSLREAALREEE	DRB1*11:10	P43355	MAGE1	292	306	45.29
1412	FFFPSLREAALREEE	DRB1*11:12	P43355	MAGE1	292	306	45.29
1413	FFFPSLREAALREEE	DRB1*11:28	P43355	MAGE1	292	306	45.29
1414	FFFPSLREAALREEE	DRB1*11:29	P43355	MAGE1	292	306	45.29
1415	FFFPSLREAALREEE	DRB1*11:49	P43355	MAGE1	292	306	45.29
1416	FFFPSLREAALREEE	DRB1*11:62	P43355	MAGE1	292	306	45.29
1417	FFFPSLREAALREEE	DRB1*11:74	P43355	MAGE1	292	306	45.29
1418	FFFPSLREAALREEE	DRB1*13:05	P43355	MAGE1	292	306	45.29
1419	FFFPSLREAALREEE	DRB1*13:14	P43355	MAGE1	292	306	45.29
1420	FFFPSLREAALREEE	DRB1*13:50	P43355	MAGE1	292	306	45.29
1421	FFPLLLFQQARAQFP	DRB1*01:01	P17643	TRP1	13	27	24.79
1422	FFPLLLFQQARAQFP	DRB1*01:18	P17643	TRP1	13	27	18.79
1423	FFPLLLFQQARAQFP	DRB1*01:20	P17643	TRP1	13	27	13.29
1424	FFPLLLFQQARAQFP	DRB1*01:29	P17643	TRP1	13	27	43.31
1425	FFPLLLFQQARAQFP	DRB1*04:04	P17643	TRP1	13	27	47.72
1426	FFPLLLFQQARAQFP	DRB1*10:01	P17643	TRP1	13	27	25.64
1427	FFPLLLFQQARAQFP	DRB1*11:02	P17643	TRP1	13	27	46.29
1428	FFPLLLFQQARAQFP	DRB1*11:04	P17643	TRP1	13	27	32.64
1429	FFPLLLFQQARAQFP	DRB1*11:13	P17643	TRP1	13	27	36.89
1430	FFPLLLFQQARAQFP	DRB1*11:42	P17643	TRP1	13	27	28.09
1431	FFPLLLFQQARAQFP	DRB1*11:46	P17643	TRP1	13	27	32.64
1432	FFPLLLFQQARAQFP	DRB1*11:58	P17643	TRP1	13	27	32.64
1433	FFPLLLFQQARAQFP	DRB1*11:65	P17643	TRP1	13	27	46.29
1434	FFPLLLFQQARAQFP	DRB1*13:01	P17643	TRP1	13	27	46.29
1435	FFPLLLFQQARAQFP	DRB1*13:11	P17643	TRP1	13	27	32.64
1436	FFPLLLFQQARAQFP	DRB1*14:32	P17643	TRP1	13	27	42.65

1437	FFPLLLFQQARAQFP	DRB1*15:01	P17643	TRP1	13	27	35.55
1438	FFPLLLFQQARAQFP	DRB1*15:03	P17643	TRP1	13	27	37.81
1439	FFPLLLFQQARAQFP	DRB1*15:06	P17643	TRP1	13	27	35.55
1440	FFPWLKVYYYRFVIG	DPB1*33:01	O75767	TRP2	201	215	43.88
1441	FFPWLKVYYYRFVIG	DPB1*71:01	O75767	TRP2	201	215	43.88
1442	FFVWLHYYSVRDTLL	DPB1*02:01	O75767	TRP2	184	198	28.16
1443	FFVWLHYYSVRDTLL	DPB1*02:02	O75767	TRP2	184	198	28.6
1444	FFVWLHYYSVRDTLL	DPB1*33:01	O75767	TRP2	184	198	12.95
1445	FFVWLHYYSVRDTLL	DPB1*46:01	O75767	TRP2	184	198	28.16
1446	FFVWLHYYSVRDTLL	DPB1*47:01	O75767	TRP2	184	198	28.6
1447	FFVWLHYYSVRDTLL	DPB1*71:01	O75767	TRP2	184	198	12.95
1448	FFVWLHYYSVRDTLL	DPB1*81:01	O75767	TRP2	184	198	28.16
1449	FFVWLHYYSVRDTLL	DRB1*01:18	O75767	TRP2	184	198	35.64
1450	FFVWLHYYSVRDTLL	DRB1*15:01	O75767	TRP2	184	198	35.04
1451	FFVWLHYYSVRDTLL	DRB1*15:06	O75767	TRP2	184	198	35.04
1452	FGDSSGTLISRALVV	DRB1*07:01	P40967	PMEL17	262	276	27.55
1453	FGRLQGISPKIMPKK	DRB1*01:01	Q16385	SSX2	101	115	33.11
1454	FGRLQGISPKIMPKK	DRB1*01:18	Q16385	SSX2	101	115	27.98
1455	FGRLQGISPKIMPKK	DRB1*01:20	Q16385	SSX2	101	115	22.29
1456	FGTASYLIRARRSMD	DRB1*11:03	P17643	TRP1	494	508	29.56
1457	FGTASYLIRARRSMD	DRB1*11:04	P17643	TRP1	494	508	30.25
1458	FGTASYLIRARRSMD	DRB1*11:42	P17643	TRP1	494	508	26.27
1459	FGTASYLIRARRSMD	DRB1*11:46	P17643	TRP1	494	508	30.25
1460	FGTASYLIRARRSMD	DRB1*11:58	P17643	TRP1	494	508	30.25
1461	FGTASYLIRARRSMD	DRB1*13:11	P17643	TRP1	494	508	30.25
1462	FGTASYLIRARRSMD	DRB1*13:21	P17643	TRP1	494	508	31.19
1463	FLALSAQLLQARLMK	DRB1*01:01	Q13072	BAGE	7	21	6.96
1464	FLALSAQLLQARLMK	DRB1*01:02	Q13072	BAGE	7	21	27.62
1465	FLALSAQLLQARLMK	DRB1*01:11	Q13072	BAGE	7	21	13.82
1466	FLALSAQLLQARLMK	DRB1*01:18	Q13072	BAGE	7	21	5.62
1467	FLALSAQLLQARLMK	DRB1*01:20	Q13072	BAGE	7	21	10.67
1468	FLALSAQLLQARLMK	DRB1*01:24	Q13072	BAGE	7	21	9.15
1469	FLALSAQLLQARLMK	DRB1*01:29	Q13072	BAGE	7	21	10.95
1470	FLALSAQLLQARLMK	DRB1*10:01	Q13072	BAGE	7	21	24.93
1471	FLGALDLAKKRVHPD	DRB1*11:03	O75767	TRP2	141	155	43.01
1472	FLGALDLAKKRVHPD	DRB1*11:04	O75767	TRP2	141	155	39.69
1473	FLGALDLAKKRVHPD	DRB1*11:42	O75767	TRP2	141	155	45.97
1474	FLGALDLAKKRVHPD	DRB1*11:46	O75767	TRP2	141	155	39.69
1475	FLGALDLAKKRVHPD	DRB1*11:58	O75767	TRP2	141	155	39.69
1476	FLGALDLAKKRVHPD	DRB1*13:11	O75767	TRP2	141	155	39.69

1477	FLLHHAFVDSIFEQW	DPB1*02:01	P14679	Tyrosinase	386	400	32.2
1478	FLLHHAFVDSIFEQW	DPB1*02:02	P14679	Tyrosinase	386	400	33.94
1479	FLLHHAFVDSIFEQW	DPB1*04:01	P14679	Tyrosinase	386	400	45.32
1480	FLLHHAFVDSIFEQW	DPB1*126:01	P14679	Tyrosinase	386	400	45.32
1481	FLLHHAFVDSIFEQW	DPB1*23:01	P14679	Tyrosinase	386	400	45.32
1482	FLLHHAFVDSIFEQW	DPB1*33:01	P14679	Tyrosinase	386	400	13.93
1483	FLLHHAFVDSIFEQW	DPB1*39:01	P14679	Tyrosinase	386	400	45.32
1484	FLLHHAFVDSIFEQW	DPB1*46:01	P14679	Tyrosinase	386	400	32.2
1485	FLLHHAFVDSIFEQW	DPB1*47:01	P14679	Tyrosinase	386	400	33.94
1486	FLLHHAFVDSIFEQW	DPB1*71:01	P14679	Tyrosinase	386	400	13.93
1487	FLLHHAFVDSIFEQW	DPB1*72:01	P14679	Tyrosinase	386	400	47.66
1488	FLLHHAFVDSIFEQW	DPB1*81:01	P14679	Tyrosinase	386	400	32.2
1489	FLLLKYRAREPVTKA	DRB1*11:02	P43355	MAGE1	112	126	31.43
1490	FLLLKYRAREPVTKA	DRB1*11:03	P43355	MAGE1	112	126	21.81
1491	FLLLKYRAREPVTKA	DRB1*11:04	P43355	MAGE1	112	126	33.91
1492	FLLLKYRAREPVTKA	DRB1*11:42	P43355	MAGE1	112	126	38.03
1493	FLLLKYRAREPVTKA	DRB1*11:46	P43355	MAGE1	112	126	33.91
1494	FLLLKYRAREPVTKA	DRB1*11:58	P43355	MAGE1	112	126	33.91
1495	FLLLKYRAREPVTKA	DRB1*11:65	P43355	MAGE1	112	126	31.43
1496	FLLLKYRAREPVTKA	DRB1*13:01	P43355	MAGE1	112	126	31.43
1497	FLLLKYRAREPVTKA	DRB1*13:11	P43355	MAGE1	112	126	33.91
1498	FLLLKYRAREPVTKA	DRB1*13:21	P43355	MAGE1	112	126	37.04
1499	FLLRKYRAKELVTKA	DRB1*01:18	P43358	MAGE4	120	134	49.35
1500	FLLRKYRAKELVTKA	DRB1*01:20	P43358	MAGE4	120	134	39.88
1501	FLLRKYRAKELVTKA	DRB1*11:03	P43358	MAGE4	120	134	37.77
1502	FLLRKYRAKELVTKA	DRB1*15:01	P43358	MAGE4	120	134	14.76
1503	FLLRKYRAKELVTKA	DRB1*15:03	P43358	MAGE4	120	134	23.85
1504	FLLRKYRAKELVTKA	DRB1*15:06	P43358	MAGE4	120	134	14.76
1505	FLLRKYRAKELVTKA	DRB1*15:07	P43358	MAGE4	120	134	31.24
1506	FLLRKYRAKELVTKA	DRB1*15:15	P43358	MAGE4	120	134	31.71
1507	FLLRKYRAKELVTKA	DRB1*15:37	P43358	MAGE4	120	134	38.24
1508	FLPVFLAQPPSGQRR	DRB1*01:01	P78358	NY-ESO-1	166	180	27.93
1509	FLPVFLAQPPSGQRR	DRB1*01:18	P78358	NY-ESO-1	166	180	34.4
1510	FLPVFLAQPPSGQRR	DRB1*01:20	P78358	NY-ESO-1	166	180	33.75
1511	FLPVFLAQPPSGQRR	DRB1*10:01	P78358	NY-ESO-1	166	180	25.38
1512	FLTWHRYHLLRLEKD	DPB1*02:01	P17643	TRP1	220	234	23
1513	FLTWHRYHLLRLEKD	DPB1*02:02	P17643	TRP1	220	234	21.67
1514	FLTWHRYHLLRLEKD	DPB1*33:01	P17643	TRP1	220	234	10.71
1515	FLTWHRYHLLRLEKD	DPB1*41:01	P17643	TRP1	220	234	49.77
1516	FLTWHRYHLLRLEKD	DPB1*46:01	P17643	TRP1	220	234	23

1517	FLTWHRYHLLRLEKD	DPB1*47:01	P17643	TRP1	220	234	21.67
1518	FLTWHRYHLLRLEKD	DPB1*71:01	P17643	TRP1	220	234	10.71
1519	FLTWHRYHLLRLEKD	DPB1*81:01	P17643	TRP1	220	234	23
1520	FMAFVAMVTTACHEF	DRB1*01:01	P04271	S100	72	86	26.13
1521	FMAFVAMVTTACHEF	DRB1*01:11	P04271	S100	72	86	44.61
1522	FMAFVAMVTTACHEF	DRB1*01:18	P04271	S100	72	86	22.4
1523	FMAFVAMVTTACHEF	DRB1*01:20	P04271	S100	72	86	49.78
1524	FMAFVAMVTTACHEF	DRB1*01:24	P04271	S100	72	86	36.98
1525	FMAFVAMVTTACHEF	DRB1*01:29	P04271	S100	72	86	42.84
1526	FMAFVAMVTTACHEF	DRB1*04:04	P04271	S100	72	86	33.27
1527	FMAFVAMVTTACHEF	DRB1*04:08	P04271	S100	72	86	40.92
1528	FMAFVAMVTTACHEF	DRB1*10:01	P04271	S100	72	86	24.93
1529	FPLLLFQQARAQFPR	DRB1*01:01	P17643	TRP1	14	28	14.01
1530	FPLLLFQQARAQFPR	DRB1*01:02	P17643	TRP1	14	28	39.52
1531	FPLLLFQQARAQFPR	DRB1*01:11	P17643	TRP1	14	28	39.18
1532	FPLLLFQQARAQFPR	DRB1*01:18	P17643	TRP1	14	28	13.19
1533	FPLLLFQQARAQFPR	DRB1*01:20	P17643	TRP1	14	28	11.09
1534	FPLLLFQQARAQFPR	DRB1*01:24	P17643	TRP1	14	28	30.7
1535	FPLLLFQQARAQFPR	DRB1*01:29	P17643	TRP1	14	28	27.06
1536	FPLLLFQQARAQFPR	DRB1*04:04	P17643	TRP1	14	28	45.8
1537	FPLLLFQQARAQFPR	DRB1*08:04	P17643	TRP1	14	28	49.27
1538	FPLLLFQQARAQFPR	DRB1*09:01	P17643	TRP1	14	28	44.22
1539	FPLLLFQQARAQFPR	DRB1*10:01	P17643	TRP1	14	28	16.3
1540	FPLLLFQQARAQFPR	DRB1*11:02	P17643	TRP1	14	28	34.58
1541	FPLLLFQQARAQFPR	DRB1*11:03	P17643	TRP1	14	28	32.89
1542	FPLLLFQQARAQFPR	DRB1*11:04	P17643	TRP1	14	28	26.77
1543	FPLLLFQQARAQFPR	DRB1*11:13	P17643	TRP1	14	28	30.19
1544	FPLLLFQQARAQFPR	DRB1*11:42	P17643	TRP1	14	28	22.15
1545	FPLLLFQQARAQFPR	DRB1*11:46	P17643	TRP1	14	28	26.77
1546	FPLLLFQQARAQFPR	DRB1*11:58	P17643	TRP1	14	28	26.77
1547	FPLLLFQQARAQFPR	DRB1*11:65	P17643	TRP1	14	28	34.58
1548	FPLLLFQQARAQFPR	DRB1*13:01	P17643	TRP1	14	28	34.58
1549	FPLLLFQQARAQFPR	DRB1*13:11	P17643	TRP1	14	28	26.77
1550	FPLLLFQQARAQFPR	DRB1*14:06	P17643	TRP1	14	28	48.96
1551	FPLLLFQQARAQFPR	DRB1*14:32	P17643	TRP1	14	28	39.85
1552	FPLLLFQQARAQFPR	DRB1*15:01	P17643	TRP1	14	28	41.01
1553	FPLLLFQQARAQFPR	DRB1*15:03	P17643	TRP1	14	28	37.62
1554	FPLLLFQQARAQFPR	DRB1*15:06	P17643	TRP1	14	28	41.01
1555	FPVIFGKASESLKMI	DRB1*01:01	P43358	MAGE4	148	162	37.17
1556	FPVIFGKASESLKMI	DRB1*01:18	P43358	MAGE4	148	162	32.5

1557	FPVIFGKASESLKMI	DRB1*01:20	P43358	MAGE4	148	162	26.81
1558	FQDYIKSYLEQASRI	DRB1*10:01	P14679	Tyrosinase	460	474	40.9
1559	FQEFMAFVAMVTTAC	DRB1*01:01	P04271	S100	69	83	45.9
1560	FQEFMAFVAMVTTAC	DRB1*01:18	P04271	S100	69	83	31.01
1561	FQEFMAFVAMVTTAC	DRB1*10:01	P04271	S100	69	83	48.37
1562	FRAVITKKVADLVGF	DRB1*01:01	P43355	MAGE1	98	112	39.93
1563	FRAVITKKVADLVGF	DRB1*01:18	P43355	MAGE1	98	112	26.71
1564	FRAVITKKVADLVGF	DRB1*01:20	P43355	MAGE1	98	112	45.37
1565	FREALSNKVDELAHF	DRB1*01:01	P43358	MAGE4	106	120	45.35
1566	FREALSNKVDELAHF	DRB1*01:18	P43358	MAGE4	106	120	48.59
1567	FSIALNFPGSQKVLP	DRB1*11:14	P40967	PMEL17	84	98	37.57
1568	FSIALNFPGSQKVLP	DRB1*13:02	P40967	PMEL17	84	98	37.57
1569	FSIALNFPGSQKVLP	DRB1*13:23	P40967	PMEL17	84	98	37.57
1570	FSIALNFPGSQKVLP	DRB1*13:97	P40967	PMEL17	84	98	37.57
1571	FSLPYWNFATGKNVC	DRB1*01:01	P17643	TRP1	244	258	38.68
1572	FSLPYWNFATGKNVC	DRB1*01:18	P17643	TRP1	244	258	40.22
1573	FSLPYWNFATGKNVC	DRB1*09:01	P17643	TRP1	244	258	45.1
1574	FSVSVSQLRALDGGN	DRB1*01:20	P40967	PMEL17	215	229	38.1
1575	FSVTLDIVQGIESAE	DRB1*03:11	P40967	PMEL17	483	497	48.27
1576	FVIGLRVWQWEVISC	DPB1*33:01	O75767	TRP2	212	226	43.8
1577	FVIGLRVWQWEVISC	DPB1*71:01	O75767	TRP2	212	226	43.8
1578	FVRALDMAKRTTHPL	DRB1*11:03	P17643	TRP1	144	158	48.1
1579	FVRALDMAKRTTHPL	DRB1*11:04	P17643	TRP1	144	158	38.95
1580	FVRALDMAKRTTHPL	DRB1*11:42	P17643	TRP1	144	158	38.2
1581	FVRALDMAKRTTHPL	DRB1*11:46	P17643	TRP1	144	158	38.95
1582	FVRALDMAKRTTHPL	DRB1*11:58	P17643	TRP1	144	158	38.95
1583	FVRALDMAKRTTHPL	DRB1*13:11	P17643	TRP1	144	158	38.95
1584	FVWLHYYSVRDTLLG	DPB1*33:01	O75767	TRP2	185	199	36.94
1585	FVWLHYYSVRDTLLG	DPB1*71:01	O75767	TRP2	185	199	36.94
1586	FVWLHYYSVRDTLLG	DRB1*01:01	O75767	TRP2	185	199	46.81
1587	FVWLHYYSVRDTLLG	DRB1*01:18	O75767	TRP2	185	199	40.12
1588	FVWLHYYSVRDTLLG	DRB1*15:01	O75767	TRP2	185	199	37.62
1589	FVWLHYYSVRDTLLG	DRB1*15:06	O75767	TRP2	185	199	37.62
1590	FVWMHYYVSMDALLG	DPB1*33:01	P14679	Tyrosinase	176	190	41.15
1591	FVWMHYYVSMDALLG	DPB1*71:01	P14679	Tyrosinase	176	190	41.15
1592	FVWMHYYVSMDALLG	DRB1*01:01	P14679	Tyrosinase	176	190	11.07
1593	FVWMHYYVSMDALLG	DRB1*01:11	P14679	Tyrosinase	176	190	34.06
1594	FVWMHYYVSMDALLG	DRB1*01:18	P14679	Tyrosinase	176	190	11.92
1595	FVWMHYYVSMDALLG	DRB1*01:20	P14679	Tyrosinase	176	190	47.64
1596	FVWMHYYVSMDALLG	DRB1*01:24	P14679	Tyrosinase	176	190	24.65

1597	FVWMHYYVSMDALLG	DRB1*01:29	P14679	Tyrosinase	176	190	26.26
1598	FVWMHYYVSMDALLG	DRB1*04:05	P14679	Tyrosinase	176	190	38
1599	FVWMHYYVSMDALLG	DRB1*04:08	P14679	Tyrosinase	176	190	43.64
1600	FVWMHYYVSMDALLG	DRB1*10:01	P14679	Tyrosinase	176	190	12.54
1601	FVWTHYYSVKKTFLG	DPB1*02:01	P17643	TRP1	188	202	30.77
1602	FVWTHYYSVKKTFLG	DPB1*02:02	P17643	TRP1	188	202	32.57
1603	FVWTHYYSVKKTFLG	DPB1*33:01	P17643	TRP1	188	202	16.66
1604	FVWTHYYSVKKTFLG	DPB1*46:01	P17643	TRP1	188	202	30.77
1605	FVWTHYYSVKKTFLG	DPB1*47:01	P17643	TRP1	188	202	32.57
1606	FVWTHYYSVKKTFLG	DPB1*71:01	P17643	TRP1	188	202	16.66
1607	FVWTHYYSVKKTFLG	DPB1*81:01	P17643	TRP1	188	202	30.77
1608	FVWTHYYSVKKTFLG	DRB1*01:18	P17643	TRP1	188	202	45.58
1609	FVWTHYYSVKKTFLG	DRB1*11:01	P17643	TRP1	188	202	47.59
1610	FVWTHYYSVKKTFLG	DRB1*11:10	P17643	TRP1	188	202	47.59
1611	FVWTHYYSVKKTFLG	DRB1*11:12	P17643	TRP1	188	202	47.59
1612	FVWTHYYSVKKTFLG	DRB1*11:28	P17643	TRP1	188	202	47.59
1613	FVWTHYYSVKKTFLG	DRB1*11:29	P17643	TRP1	188	202	47.59
1614	FVWTHYYSVKKTFLG	DRB1*11:49	P17643	TRP1	188	202	47.59
1615	FVWTHYYSVKKTFLG	DRB1*11:62	P17643	TRP1	188	202	47.59
1616	FVWTHYYSVKKTFLG	DRB1*11:74	P17643	TRP1	188	202	47.59
1617	FVWTHYYSVKKTFLG	DRB1*13:05	P17643	TRP1	188	202	47.59
1618	FVWTHYYSVKKTFLG	DRB1*13:14	P17643	TRP1	188	202	47.59
1619	FVWTHYYSVKKTFLG	DRB1*13:21	P17643	TRP1	188	202	33.53
1620	FVWTHYYSVKKTFLG	DRB1*13:50	P17643	TRP1	188	202	47.59
1621	FVWTHYYSVKKTFLG	DRB1*15:15	P17643	TRP1	188	202	45.64
1622	FYLAMPFATPMEAEL	DRB1*01:01	P78358	NY-ESO-1	90	104	32.49
1623	FYLAMPFATPMEAEL	DRB1*01:18	P78358	NY-ESO-1	90	104	29.67
1624	FYLAMPFATPMEAEL	DRB1*10:01	P78358	NY-ESO-1	90	104	33.15
1625	FYVYMKRKYEAMTKL	DRB1*11:01	Q16385	SSX2	47	61	19.78
1626	FYVYMKRKYEAMTKL	DRB1*11:02	Q16385	SSX2	47	61	39.09
1627	FYVYMKRKYEAMTKL	DRB1*11:03	Q16385	SSX2	47	61	16.28
1628	FYVYMKRKYEAMTKL	DRB1*11:04	Q16385	SSX2	47	61	16.24
1629	FYVYMKRKYEAMTKL	DRB1*11:10	Q16385	SSX2	47	61	19.78
1630	FYVYMKRKYEAMTKL	DRB1*11:11	Q16385	SSX2	47	61	45.35
1631	FYVYMKRKYEAMTKL	DRB1*11:12	Q16385	SSX2	47	61	19.78
1632	FYVYMKRKYEAMTKL	DRB1*11:28	Q16385	SSX2	47	61	19.78
1633	FYVYMKRKYEAMTKL	DRB1*11:29	Q16385	SSX2	47	61	19.78
1634	FYVYMKRKYEAMTKL	DRB1*11:42	Q16385	SSX2	47	61	28.04
1635	FYVYMKRKYEAMTKL	DRB1*11:46	Q16385	SSX2	47	61	16.24
1636	FYVYMKRKYEAMTKL	DRB1*11:49	Q16385	SSX2	47	61	19.78

1637	FYVYMKRKYEAMTKL	DRB1*11:58	Q16385	SSX2	47	61	16.24
1638	FYVYMKRKYEAMTKL	DRB1*11:62	Q16385	SSX2	47	61	19.78
1639	FYVYMKRKYEAMTKL	DRB1*11:65	Q16385	SSX2	47	61	39.09
1640	FYVYMKRKYEAMTKL	DRB1*11:74	Q16385	SSX2	47	61	19.78
1641	FYVYMKRKYEAMTKL	DRB1*13:01	Q16385	SSX2	47	61	39.09
1642	FYVYMKRKYEAMTKL	DRB1*13:05	Q16385	SSX2	47	61	19.78
1643	FYVYMKRKYEAMTKL	DRB1*13:11	Q16385	SSX2	47	61	16.24
1644	FYVYMKRKYEAMTKL	DRB1*13:14	Q16385	SSX2	47	61	19.78
1645	FYVYMKRKYEAMTKL	DRB1*13:21	Q16385	SSX2	47	61	12.4
1646	FYVYMKRKYEAMTKL	DRB1*13:50	Q16385	SSX2	47	61	19.78
1647	GALLAVGATKVPRNQ	DRB1*01:01	P40967	PMEL17	16	30	8.84
1648	GALLAVGATKVPRNQ	DRB1*01:02	P40967	PMEL17	16	30	26.56
1649	GALLAVGATKVPRNQ	DRB1*01:11	P40967	PMEL17	16	30	25.72
1650	GALLAVGATKVPRNQ	DRB1*01:18	P40967	PMEL17	16	30	9.32
1651	GALLAVGATKVPRNQ	DRB1*01:20	P40967	PMEL17	16	30	9.89
1652	GALLAVGATKVPRNQ	DRB1*01:24	P40967	PMEL17	16	30	20.5
1653	GALLAVGATKVPRNQ	DRB1*01:29	P40967	PMEL17	16	30	19.18
1654	GALLAVGATKVPRNQ	DRB1*07:01	P40967	PMEL17	16	30	28.16
1655	GANASFSIALNFPGS	DRB1*01:01	P40967	PMEL17	79	93	14.29
1656	GANASFSIALNFPGS	DRB1*01:18	P40967	PMEL17	79	93	14.74
1657	GANASFSIALNFPGS	DRB1*01:20	P40967	PMEL17	79	93	42.19
1658	GANASFSIALNFPGS	DRB1*01:24	P40967	PMEL17	79	93	35.66
1659	GANASFSIALNFPGS	DRB1*01:29	P40967	PMEL17	79	93	38.11
1660	GANASFSIALNFPGS	DRB1*04:01	P40967	PMEL17	79	93	19.17
1661	GANASFSIALNFPGS	DRB1*04:04	P40967	PMEL17	79	93	40.38
1662	GANASFSIALNFPGS	DRB1*04:05	P40967	PMEL17	79	93	32.67
1663	GANASFSIALNFPGS	DRB1*04:08	P40967	PMEL17	79	93	19.98
1664	GANASFSIALNFPGS	DRB1*09:01	P40967	PMEL17	79	93	42.92
1665	GANASFSIALNFPGS	DRB1*10:01	P40967	PMEL17	79	93	15.26
1666	GANASFSIALNFPGS	DRB1*16:01	P40967	PMEL17	79	93	40.98
1667	GANASFSIALNFPGS	DRB1*16:02	P40967	PMEL17	79	93	35.51
1668	GANASFSIALNFPGS	DRB1*16:09	P40967	PMEL17	79	93	49.43
1669	GAVLTALLAGLVSLL	DRB1*01:01	P14679	Tyrosinase	485	499	14.36
1670	GAVLTALLAGLVSLL	DRB1*01:02	P14679	Tyrosinase	485	499	48.43
1671	GAVLTALLAGLVSLL	DRB1*01:11	P14679	Tyrosinase	485	499	31
1672	GAVLTALLAGLVSLL	DRB1*01:18	P14679	Tyrosinase	485	499	12.16
1673	GAVLTALLAGLVSLL	DRB1*01:20	P14679	Tyrosinase	485	499	16.49
1674	GAVLTALLAGLVSLL	DRB1*01:24	P14679	Tyrosinase	485	499	14.8
1675	GAVLTALLAGLVSLL	DRB1*01:29	P14679	Tyrosinase	485	499	22.75
1676	GDNQIMPKTGFLIIV	DRB1*01:01	P43355	MAGE1	184	198	46.71

1677	GDNQIMPKTGFLIIV	DRB1*01:18	P43355	MAGE1	184	198	32
1678	GDNQIMPKTGFLIIV	DRB1*01:20	P43355	MAGE1	184	198	27.24
1679	GDSSGTLISRALVVT	DRB1*01:01	P40967	PMEL17	263	277	47.48
1680	GDSSGTLISRALVVT	DRB1*01:18	P40967	PMEL17	263	277	45.27
1681	GDSSGTLISRALVVT	DRB1*01:20	P40967	PMEL17	263	277	38.76
1682	GDSSGTLISRALVVT	DRB1*07:01	P40967	PMEL17	263	277	25.03
1683	GEVDFSHEGPAFLTW	DRB1*01:01	P17643	TRP1	209	223	42.17
1684	GFKATLPPFMCNKRA	DRB1*01:01	Q16385	SSX2	62	76	36.57
1685	GFKATLPPFMCNKRA	DRB1*01:18	Q16385	SSX2	62	76	33.45
1686	GFKATLPPFMCNKRA	DRB1*07:01	Q16385	SSX2	62	76	46.32
1687	GFKATLPPFMCNKRA	DRB1*10:01	Q16385	SSX2	62	76	29.58
1688	GFLLLKYRAREPVTK	DRB1*11:01	P43355	MAGE1	111	125	45.81
1689	GFLLLKYRAREPVTK	DRB1*11:02	P43355	MAGE1	111	125	28.99
1690	GFLLLKYRAREPVTK	DRB1*11:03	P43355	MAGE1	111	125	18.27
1691	GFLLLKYRAREPVTK	DRB1*11:04	P43355	MAGE1	111	125	25.28
1692	GFLLLKYRAREPVTK	DRB1*11:10	P43355	MAGE1	111	125	45.81
1693	GFLLLKYRAREPVTK	DRB1*11:12	P43355	MAGE1	111	125	45.81
1694	GFLLLKYRAREPVTK	DRB1*11:13	P43355	MAGE1	111	125	47.19
1695	GFLLLKYRAREPVTK	DRB1*11:28	P43355	MAGE1	111	125	45.81
1696	GFLLLKYRAREPVTK	DRB1*11:29	P43355	MAGE1	111	125	45.81
1697	GFLLLKYRAREPVTK	DRB1*11:42	P43355	MAGE1	111	125	27.65
1698	GFLLLKYRAREPVTK	DRB1*11:46	P43355	MAGE1	111	125	25.28
1699	GFLLLKYRAREPVTK	DRB1*11:49	P43355	MAGE1	111	125	45.81
1700	GFLLLKYRAREPVTK	DRB1*11:58	P43355	MAGE1	111	125	25.28
1701	GFLLLKYRAREPVTK	DRB1*11:62	P43355	MAGE1	111	125	45.81
1702	GFLLLKYRAREPVTK	DRB1*11:65	P43355	MAGE1	111	125	28.99
1703	GFLLLKYRAREPVTK	DRB1*11:74	P43355	MAGE1	111	125	45.81
1704	GFLLLKYRAREPVTK	DRB1*13:01	P43355	MAGE1	111	125	28.99
1705	GFLLLKYRAREPVTK	DRB1*13:05	P43355	MAGE1	111	125	45.81
1706	GFLLLKYRAREPVTK	DRB1*13:11	P43355	MAGE1	111	125	25.28
1707	GFLLLKYRAREPVTK	DRB1*13:14	P43355	MAGE1	111	125	45.81
1708	GFLLLKYRAREPVTK	DRB1*13:21	P43355	MAGE1	111	125	25.61
1709	GFLLLKYRAREPVTK	DRB1*13:50	P43355	MAGE1	111	125	45.81
1710	GFLLLKYRAREPVTK	DRB1*15:01	P43355	MAGE1	111	125	42.9
1711	GFLLLKYRAREPVTK	DRB1*15:06	P43355	MAGE1	111	125	42.9
1712	GGFFPWLKVYYYRFV	DPB1*33:01	O75767	TRP2	199	213	38.84
1713	GGFFPWLKVYYYRFV	DPB1*71:01	O75767	TRP2	199	213	38.84
1714	GGNKHFLRNQPLTFA	DRB1*01:01	P40967	PMEL17	227	241	23.3
1715	GGNKHFLRNQPLTFA	DRB1*01:11	P40967	PMEL17	227	241	42.97
1716	GGNKHFLRNQPLTFA	DRB1*01:18	P40967	PMEL17	227	241	18.46

1717	GGNKHFLRNQPLTFA	DRB1*01:20	P40967	PMEL17	227	241	32.86
1718	GGNKHFLRNQPLTFA	DRB1*07:01	P40967	PMEL17	227	241	49.96
1719	GGNKHFLRNQPLTFA	DRB1*11:08	P40967	PMEL17	227	241	42.7
1720	GGNKHFLRNQPLTFA	DRB1*11:14	P40967	PMEL17	227	241	11.13
1721	GGNKHFLRNQPLTFA	DRB1*13:02	P40967	PMEL17	227	241	11.13
1722	GGNKHFLRNQPLTFA	DRB1*13:23	P40967	PMEL17	227	241	11.13
1723	GGNKHFLRNQPLTFA	DRB1*13:66	P40967	PMEL17	227	241	35.47
1724	GGNKHFLRNQPLTFA	DRB1*13:96	P40967	PMEL17	227	241	14.94
1725	GGNKHFLRNQPLTFA	DRB1*13:97	P40967	PMEL17	227	241	11.13
1726	GGNKHFLRNQPLTFA	DRB1*14:02	P40967	PMEL17	227	241	36.43
1727	GHNRESYMVPFIPLY	DPB1*33:01	P14679	Tyrosinase	419	433	23.48
1728	GHNRESYMVPFIPLY	DPB1*71:01	P14679	Tyrosinase	419	433	23.48
1729	GKASESLKMIFGIDV	DRB1*01:18	P43358	MAGE4	153	167	38.59
1730	GKASESLKMIFGIDV	DRB1*01:20	P43358	MAGE4	153	167	39.4
1731	GLRVWQWEVISCKLI	DPB1*33:01	O75767	TRP2	215	229	45.96
1732	GLRVWQWEVISCKLI	DPB1*71:01	O75767	TRP2	215	229	45.96
1733	GLSIGTGRAMLGTHT	DRB1*01:20	P40967	PMEL17	169	183	45.38
1734	GLVCVQAATSSSSPL	DRB1*01:01	P43355	MAGE1	24	38	49.94
1735	GLVCVQAATSSSSPL	DRB1*01:20	P43355	MAGE1	24	38	43.15
1736	GLVSLLCRHKRKQLP	DRB1*11:02	P14679	Tyrosinase	494	508	22.97
1737	GLVSLLCRHKRKQLP	DRB1*11:03	P14679	Tyrosinase	494	508	14
1738	GLVSLLCRHKRKQLP	DRB1*11:04	P14679	Tyrosinase	494	508	26.41
1739	GLVSLLCRHKRKQLP	DRB1*11:42	P14679	Tyrosinase	494	508	28.6
1740	GLVSLLCRHKRKQLP	DRB1*11:46	P14679	Tyrosinase	494	508	26.41
1741	GLVSLLCRHKRKQLP	DRB1*11:58	P14679	Tyrosinase	494	508	26.41
1742	GLVSLLCRHKRKQLP	DRB1*11:65	P14679	Tyrosinase	494	508	22.97
1743	GLVSLLCRHKRKQLP	DRB1*13:01	P14679	Tyrosinase	494	508	22.97
1744	GLVSLLCRHKRKQLP	DRB1*13:11	P14679	Tyrosinase	494	508	26.41
1745	GNILTIRLTAADHRQ	DRB1*01:20	P78358	NY-ESO-1	130	144	33.57
1746	GNILTIRLTAADHRQ	DRB1*11:13	P78358	NY-ESO-1	130	144	44.05
1747	GNILTIRLTAADHRQ	DRB1*11:42	P78358	NY-ESO-1	130	144	32.64
1748	GNKHFLRNQPLTFAL	DRB1*01:01	P40967	PMEL17	228	242	17.88
1749	GNKHFLRNQPLTFAL	DRB1*01:11	P40967	PMEL17	228	242	29.17
1750	GNKHFLRNQPLTFAL	DRB1*01:18	P40967	PMEL17	228	242	13.77
1751	GNKHFLRNQPLTFAL	DRB1*01:20	P40967	PMEL17	228	242	24.55
1752	GNKHFLRNQPLTFAL	DRB1*01:24	P40967	PMEL17	228	242	42.88
1753	GNKHFLRNQPLTFAL	DRB1*01:29	P40967	PMEL17	228	242	38.99
1754	GNKHFLRNQPLTFAL	DRB1*07:01	P40967	PMEL17	228	242	38.73
1755	GNKHFLRNQPLTFAL	DRB1*10:01	P40967	PMEL17	228	242	41.14
1756	GNKHFLRNQPLTFAL	DRB1*11:02	P40967	PMEL17	228	242	45.46

1757	GNKHFLRNQPLTFAL	DRB1*11:08	P40967	PMEL17	228	242	31.11
1758	GNKHFLRNQPLTFAL	DRB1*11:14	P40967	PMEL17	228	242	8.61
1759	GNKHFLRNQPLTFAL	DRB1*11:19	P40967	PMEL17	228	242	39.67
1760	GNKHFLRNQPLTFAL	DRB1*11:65	P40967	PMEL17	228	242	45.46
1761	GNKHFLRNQPLTFAL	DRB1*13:01	P40967	PMEL17	228	242	45.46
1762	GNKHFLRNQPLTFAL	DRB1*13:02	P40967	PMEL17	228	242	8.61
1763	GNKHFLRNQPLTFAL	DRB1*13:23	P40967	PMEL17	228	242	8.61
1764	GNKHFLRNQPLTFAL	DRB1*13:33	P40967	PMEL17	228	242	49.93
1765	GNKHFLRNQPLTFAL	DRB1*13:66	P40967	PMEL17	228	242	27.75
1766	GNKHFLRNQPLTFAL	DRB1*13:96	P40967	PMEL17	228	242	11.31
1767	GNKHFLRNQPLTFAL	DRB1*13:97	P40967	PMEL17	228	242	8.61
1768	GNKHFLRNQPLTFAL	DRB1*14:02	P40967	PMEL17	228	242	24.65
1769	GNKHFLRNQPLTFAL	DRB1*14:07	P40967	PMEL17	228	242	44.47
1770	GNKHFLRNQPLTFAL	DRB1*14:32	P40967	PMEL17	228	242	44.49
1771	GNNQIFPKTGLLIIV	DRB1*01:01	P43358	MAGE4	192	206	44.3
1772	GNNQIFPKTGLLIIV	DRB1*01:18	P43358	MAGE4	192	206	29.03
1773	GNNQIFPKTGLLIIV	DRB1*01:20	P43358	MAGE4	192	206	28.64
1774	GPAFLTWHRYHLLRL	DPB1*02:01	P17643	TRP1	217	231	13.09
1775	GPAFLTWHRYHLLRL	DPB1*02:02	P17643	TRP1	217	231	12.96
1776	GPAFLTWHRYHLLRL	DPB1*16:01	P17643	TRP1	217	231	44.13
1777	GPAFLTWHRYHLLRL	DPB1*33:01	P17643	TRP1	217	231	6.81
1778	GPAFLTWHRYHLLRL	DPB1*41:01	P17643	TRP1	217	231	32.72
1779	GPAFLTWHRYHLLRL	DPB1*46:01	P17643	TRP1	217	231	13.09
1780	GPAFLTWHRYHLLRL	DPB1*47:01	P17643	TRP1	217	231	12.96
1781	GPAFLTWHRYHLLRL	DPB1*71:01	P17643	TRP1	217	231	6.81
1782	GPAFLTWHRYHLLRL	DPB1*81:01	P17643	TRP1	217	231	13.09
1783	GPAFLTWHRYHLLRL	DRB1*11:02	P17643	TRP1	217	231	35.26
1784	GPAFLTWHRYHLLRL	DRB1*11:03	P17643	TRP1	217	231	35.17
1785	GPAFLTWHRYHLLRL	DRB1*11:65	P17643	TRP1	217	231	35.26
1786	GPAFLTWHRYHLLRL	DRB1*13:01	P17643	TRP1	217	231	35.26
1787	GPAFLTWHRYHLLRL	DRB1*13:21	P17643	TRP1	217	231	48.21
1788	GPAFLTWHRYHLLRL	DRB1*15:01	P17643	TRP1	217	231	49.09
1789	GPAFLTWHRYHLLRL	DRB1*15:03	P17643	TRP1	217	231	48.26
1790	GPAFLTWHRYHLLRL	DRB1*15:06	P17643	TRP1	217	231	49.09
1791	GPAFLTWHRYHLLRL	DRB1*15:15	P17643	TRP1	217	231	45.21
1792	GPESRLLEFYLAMPF	DPB1*01:01	P78358	NY-ESO-1	82	96	46.07
1793	GPESRLLEFYLAMPF	DPB1*15:01	P78358	NY-ESO-1	82	96	34.85
1794	GPESRLLEFYLAMPF	DPB1*33:01	P78358	NY-ESO-1	82	96	22.48
1795	GPESRLLEFYLAMPF	DPB1*71:01	P78358	NY-ESO-1	82	96	22.48
1796	GPIRRNPAGNVARPM	DRB1*11:14	P17643	TRP1	309	323	35.1

1797	GPIRRNPAGNVARPM	DRB1*13:02	P17643	TRP1	309	323	35.1
1798	GPIRRNPAGNVARPM	DRB1*13:23	P17643	TRP1	309	323	35.1
1799	GPIRRNPAGNVARPM	DRB1*13:97	P17643	TRP1	309	323	35.1
1800	GPLLDGTATLRLVKR	DRB1*01:20	P40967	PMEL17	455	469	48.61
1801	GPLLDGTATLRLVKR	DRB1*07:01	P40967	PMEL17	455	469	37.34
1802	GPRALAETSYVKVLE	DPB1*33:01	P43355	MAGE1	267	281	24.39
1803	GPRALAETSYVKVLE	DPB1*33:01	P43358	MAGE4	275	289	24.39
1804	GPRALAETSYVKVLE	DPB1*71:01	P43355	MAGE1	267	281	24.39
1805	GPRALAETSYVKVLE	DPB1*71:01	P43358	MAGE4	275	289	24.39
1806	GPSTSCILESLFRAV	DPB1*02:01	P43355	MAGE1	87	101	29.28
1807	GPSTSCILESLFRAV	DPB1*02:02	P43355	MAGE1	87	101	26.48
1808	GPSTSCILESLFRAV	DPB1*33:01	P43355	MAGE1	87	101	12.4
1809	GPSTSCILESLFRAV	DPB1*46:01	P43355	MAGE1	87	101	29.28
1810	GPSTSCILESLFRAV	DPB1*47:01	P43355	MAGE1	87	101	26.48
1811	GPSTSCILESLFRAV	DPB1*71:01	P43355	MAGE1	87	101	12.4
1812	GPSTSCILESLFRAV	DPB1*81:01	P43355	MAGE1	87	101	29.28
1813	GPTLIGANASFSIAL	DRB1*01:01	P40967	PMEL17	74	88	30.57
1814	GPTLIGANASFSIAL	DRB1*01:18	P40967	PMEL17	74	88	33.82
1815	GPTLIGANASFSIAL	DRB1*01:20	P40967	PMEL17	74	88	24.11
1816	GPTLIGANASFSIAL	DRB1*10:01	P40967	PMEL17	74	88	40.93
1817	GPTLIGANASFSIAL	DRB1*11:14	P40967	PMEL17	74	88	17.01
1818	GPTLIGANASFSIAL	DRB1*13:02	P40967	PMEL17	74	88	17.01
1819	GPTLIGANASFSIAL	DRB1*13:23	P40967	PMEL17	74	88	17.01
1820	GPTLIGANASFSIAL	DRB1*13:96	P40967	PMEL17	74	88	27.88
1821	GPTLIGANASFSIAL	DRB1*13:97	P40967	PMEL17	74	88	17.01
1822	GPVSGLSIGTGRAML	DRB1*01:01	P40967	PMEL17	165	179	49.97
1823	GPVSGLSIGTGRAML	DRB1*01:20	P40967	PMEL17	165	179	32.64
1824	GPVTAQVVLQAAIPL	DRB1*01:01	P40967	PMEL17	284	298	14.85
1825	GPVTAQVVLQAAIPL	DRB1*01:02	P40967	PMEL17	284	298	29.2
1826	GPVTAQVVLQAAIPL	DRB1*01:11	P40967	PMEL17	284	298	37.23
1827	GPVTAQVVLQAAIPL	DRB1*01:18	P40967	PMEL17	284	298	14.56
1828	GPVTAQVVLQAAIPL	DRB1*01:20	P40967	PMEL17	284	298	12.23
1829	GPVTAQVVLQAAIPL	DRB1*01:24	P40967	PMEL17	284	298	29.96
1830	GPVTAQVVLQAAIPL	DRB1*01:29	P40967	PMEL17	284	298	33.99
1831	GPVTAQVVLQAAIPL	DRB1*10:01	P40967	PMEL17	284	298	46.25
1832	GPVTAQVVLQAAIPL	DRB1*11:14	P40967	PMEL17	284	298	35.55
1833	GPVTAQVVLQAAIPL	DRB1*13:02	P40967	PMEL17	284	298	35.55
1834	GPVTAQVVLQAAIPL	DRB1*13:23	P40967	PMEL17	284	298	35.55
1835	GPVTAQVVLQAAIPL	DRB1*13:97	P40967	PMEL17	284	298	35.55
1836	GPVTAQVVLQAAIPL	DRB1*14:32	P40967	PMEL17	284	298	48.86

1837	GQCTEVRADTRPWSG	DRB1*03:11	O75767	TRP2	59	73	32.62
1838	GQTHLSPNDPIFVLL	DRB1*11:14	P17643	TRP1	389	403	34.38
1839	GQTHLSPNDPIFVLL	DRB1*13:02	P17643	TRP1	389	403	34.38
1840	GQTHLSPNDPIFVLL	DRB1*13:23	P17643	TRP1	389	403	34.38
1841	GQTHLSPNDPIFVLL	DRB1*13:96	P17643	TRP1	389	403	43.28
1842	GQTHLSPNDPIFVLL	DRB1*13:97	P17643	TRP1	389	403	34.38
1843	GQVIWVNNTIINGSQ	DRB1*11:14	P40967	PMEL17	100	114	16.48
1844	GQVIWVNNTIINGSQ	DRB1*13:02	P40967	PMEL17	100	114	16.48
1845	GQVIWVNNTIINGSQ	DRB1*13:23	P40967	PMEL17	100	114	16.48
1846	GQVIWVNNTIINGSQ	DRB1*13:96	P40967	PMEL17	100	114	23.52
1847	GQVIWVNNTIINGSQ	DRB1*13:97	P40967	PMEL17	100	114	16.48
1848	GQYWQVLGGPVSGLS	DRB1*01:01	P40967	PMEL17	157	171	7.51
1849	GQYWQVLGGPVSGLS	DRB1*01:11	P40967	PMEL17	157	171	27.02
1850	GQYWQVLGGPVSGLS	DRB1*01:18	P40967	PMEL17	157	171	7.92
1851	GQYWQVLGGPVSGLS	DRB1*01:20	P40967	PMEL17	157	171	25.6
1852	GQYWQVLGGPVSGLS	DRB1*01:24	P40967	PMEL17	157	171	19.53
1853	GQYWQVLGGPVSGLS	DRB1*01:29	P40967	PMEL17	157	171	17.61
1854	GQYWQVLGGPVSGLS	DRB1*10:01	P40967	PMEL17	157	171	21.64
1855	GRAMLGTHTMEVTVY	DRB1*07:01	P40967	PMEL17	175	189	38.21
1856	GRLQGISPKIMPKKP	DRB1*01:20	Q16385	SSX2	102	116	42.51
1857	GSCQNILLSNAPLGP	DRB1*01:01	P14679	Tyrosinase	53	67	40.72
1858	GSCQNILLSNAPLGP	DRB1*01:18	P14679	Tyrosinase	53	67	34.26
1859	GSCQNILLSNAPLGP	DRB1*01:20	P14679	Tyrosinase	53	67	32.2
1860	GSFSVTLDIVQGIES	DRB1*03:11	P40967	PMEL17	481	495	42.16
1861	GSNPARYEFLWGPRA	DRB1*01:01	P43358	MAGE4	264	278	20.69
1862	GSNPARYEFLWGPRA	DRB1*01:18	P43358	MAGE4	264	278	21.7
1863	GSRSYVPLAHSSSAF	DRB1*01:01	P40967	PMEL17	193	207	34.96
1864	GSRSYVPLAHSSSAF	DRB1*01:18	P40967	PMEL17	193	207	31.55
1865	GSRSYVPLAHSSSAF	DRB1*10:01	P40967	PMEL17	193	207	31.36
1866	GSRSYVPLAHSSSAF	DRB1*11:01	P40967	PMEL17	193	207	39.7
1867	GSRSYVPLAHSSSAF	DRB1*11:10	P40967	PMEL17	193	207	39.7
1868	GSRSYVPLAHSSSAF	DRB1*11:12	P40967	PMEL17	193	207	39.7
1869	GSRSYVPLAHSSSAF	DRB1*11:28	P40967	PMEL17	193	207	39.7
1870	GSRSYVPLAHSSSAF	DRB1*11:29	P40967	PMEL17	193	207	39.7
1871	GSRSYVPLAHSSSAF	DRB1*11:49	P40967	PMEL17	193	207	39.7
1872	GSRSYVPLAHSSSAF	DRB1*11:62	P40967	PMEL17	193	207	39.7
1873	GSRSYVPLAHSSSAF	DRB1*11:74	P40967	PMEL17	193	207	39.7
1874	GSRSYVPLAHSSSAF	DRB1*13:05	P40967	PMEL17	193	207	39.7
1875	GSRSYVPLAHSSSAF	DRB1*13:14	P40967	PMEL17	193	207	39.7
1876	GSRSYVPLAHSSSAF	DRB1*13:50	P40967	PMEL17	193	207	39.7

1877	GTASYLIRARRSMDE	DRB1*11:03	P17643	TRP1	495	509	36.11
1878	GTASYLIRARRSMDE	DRB1*11:04	P17643	TRP1	495	509	29.26
1879	GTASYLIRARRSMDE	DRB1*11:42	P17643	TRP1	495	509	26.94
1880	GTASYLIRARRSMDE	DRB1*11:46	P17643	TRP1	495	509	29.26
1881	GTASYLIRARRSMDE	DRB1*11:58	P17643	TRP1	495	509	29.26
1882	GTASYLIRARRSMDE	DRB1*13:11	P17643	TRP1	495	509	29.26
1883	GTASYLIRARRSMDE	DRB1*13:21	P17643	TRP1	495	509	24.11
1884	GTATLRLVKRQVPLD	DRB1*08:04	P40967	PMEL17	460	474	26.63
1885	GTATLRLVKRQVPLD	DRB1*11:01	P40967	PMEL17	460	474	20.16
1886	GTATLRLVKRQVPLD	DRB1*11:02	P40967	PMEL17	460	474	26.58
1887	GTATLRLVKRQVPLD	DRB1*11:03	P40967	PMEL17	460	474	17.89
1888	GTATLRLVKRQVPLD	DRB1*11:04	P40967	PMEL17	460	474	10.14
1889	GTATLRLVKRQVPLD	DRB1*11:06	P40967	PMEL17	460	474	44.04
1890	GTATLRLVKRQVPLD	DRB1*11:08	P40967	PMEL17	460	474	40.71
1891	GTATLRLVKRQVPLD	DRB1*11:10	P40967	PMEL17	460	474	20.16
1892	GTATLRLVKRQVPLD	DRB1*11:12	P40967	PMEL17	460	474	20.16
1893	GTATLRLVKRQVPLD	DRB1*11:13	P40967	PMEL17	460	474	20.13
1894	GTATLRLVKRQVPLD	DRB1*11:28	P40967	PMEL17	460	474	20.16
1895	GTATLRLVKRQVPLD	DRB1*11:29	P40967	PMEL17	460	474	20.16
1896	GTATLRLVKRQVPLD	DRB1*11:42	P40967	PMEL17	460	474	10.87
1897	GTATLRLVKRQVPLD	DRB1*11:46	P40967	PMEL17	460	474	10.14
1898	GTATLRLVKRQVPLD	DRB1*11:49	P40967	PMEL17	460	474	20.16
1899	GTATLRLVKRQVPLD	DRB1*11:58	P40967	PMEL17	460	474	10.14
1900	GTATLRLVKRQVPLD	DRB1*11:62	P40967	PMEL17	460	474	20.16
1901	GTATLRLVKRQVPLD	DRB1*11:65	P40967	PMEL17	460	474	26.58
1902	GTATLRLVKRQVPLD	DRB1*11:74	P40967	PMEL17	460	474	20.16
1903	GTATLRLVKRQVPLD	DRB1*11:84	P40967	PMEL17	460	474	29.01
1904	GTATLRLVKRQVPLD	DRB1*13:01	P40967	PMEL17	460	474	26.58
1905	GTATLRLVKRQVPLD	DRB1*13:05	P40967	PMEL17	460	474	20.16
1906	GTATLRLVKRQVPLD	DRB1*13:11	P40967	PMEL17	460	474	10.14
1907	GTATLRLVKRQVPLD	DRB1*13:14	P40967	PMEL17	460	474	20.16
1908	GTATLRLVKRQVPLD	DRB1*13:21	P40967	PMEL17	460	474	25.67
1909	GTATLRLVKRQVPLD	DRB1*13:50	P40967	PMEL17	460	474	20.16
1910	GTATLRLVKRQVPLD	DRB1*14:06	P40967	PMEL17	460	474	44.3
1911	GTATLRLVKRQVPLD	DRB1*14:32	P40967	PMEL17	460	474	35.48
1912	GTLISRALVVTHTYL	DRB1*01:01	P40967	PMEL17	267	281	37.9
1913	GTLISRALVVTHTYL	DRB1*01:18	P40967	PMEL17	267	281	33.36
1914	GTLISRALVVTHTYL	DRB1*01:20	P40967	PMEL17	267	281	28.88
1915	GTLISRALVVTHTYL	DRB1*10:01	P40967	PMEL17	267	281	41.51
1916	GVLLKEFTVSGNILT	DRB1*01:18	P78358	NY-ESO-1	120	134	41.65

1917	GVLLKEFTVSGNILT	DRB1*07:01	P78358	NY-ESO-1	120	134	38.5
1918	GYRALMDKSLHVGTQ	DRB1*01:01	Q16655	MELAN_A	53	67	38.39
1919	GYRALMDKSLHVGTQ	DRB1*01:18	Q16655	MELAN_A	53	67	31.82
1920	GYRALMDKSLHVGTQ	DRB1*03:11	Q16655	MELAN_A	53	67	45.36
1921	HAFVDSIFEQWLRRH	DPB1*02:01	P14679	Tyrosinase	390	404	48.15
1922	HAFVDSIFEQWLRRH	DPB1*02:02	P14679	Tyrosinase	390	404	42.36
1923	HAFVDSIFEQWLRRH	DPB1*04:01	P14679	Tyrosinase	390	404	23.3
1924	HAFVDSIFEQWLRRH	DPB1*126:01	P14679	Tyrosinase	390	404	23.3
1925	HAFVDSIFEQWLRRH	DPB1*23:01	P14679	Tyrosinase	390	404	23.3
1926	HAFVDSIFEQWLRRH	DPB1*33:01	P14679	Tyrosinase	390	404	16.76
1927	HAFVDSIFEQWLRRH	DPB1*39:01	P14679	Tyrosinase	390	404	23.3
1928	HAFVDSIFEQWLRRH	DPB1*46:01	P14679	Tyrosinase	390	404	48.15
1929	HAFVDSIFEQWLRRH	DPB1*47:01	P14679	Tyrosinase	390	404	42.36
1930	HAFVDSIFEQWLRRH	DPB1*71:01	P14679	Tyrosinase	390	404	16.76
1931	HAFVDSIFEQWLRRH	DPB1*72:01	P14679	Tyrosinase	390	404	27.2
1932	HAFVDSIFEQWLRRH	DPB1*81:01	P14679	Tyrosinase	390	404	48.15
1933	HEGPAFLTWHRYHLL	DPB1*02:01	P17643	TRP1	215	229	26.31
1934	HEGPAFLTWHRYHLL	DPB1*02:02	P17643	TRP1	215	229	21.73
1935	HEGPAFLTWHRYHLL	DPB1*33:01	P17643	TRP1	215	229	10.01
1936	HEGPAFLTWHRYHLL	DPB1*46:01	P17643	TRP1	215	229	26.31
1937	HEGPAFLTWHRYHLL	DPB1*47:01	P17643	TRP1	215	229	21.73
1938	HEGPAFLTWHRYHLL	DPB1*71:01	P17643	TRP1	215	229	10.01
1939	HEGPAFLTWHRYHLL	DPB1*81:01	P17643	TRP1	215	229	26.31
1940	HFLLRKYRAKELVTK	DRB1*01:18	P43358	MAGE4	119	133	39.26
1941	HFLLRKYRAKELVTK	DRB1*01:20	P43358	MAGE4	119	133	33.95
1942	HFLLRKYRAKELVTK	DRB1*11:02	P43358	MAGE4	119	133	38.09
1943	HFLLRKYRAKELVTK	DRB1*11:03	P43358	MAGE4	119	133	25.58
1944	HFLLRKYRAKELVTK	DRB1*11:04	P43358	MAGE4	119	133	38.75
1945	HFLLRKYRAKELVTK	DRB1*11:42	P43358	MAGE4	119	133	47.03
1946	HFLLRKYRAKELVTK	DRB1*11:46	P43358	MAGE4	119	133	38.75
1947	HFLLRKYRAKELVTK	DRB1*11:58	P43358	MAGE4	119	133	38.75
1948	HFLLRKYRAKELVTK	DRB1*11:65	P43358	MAGE4	119	133	38.09
1949	HFLLRKYRAKELVTK	DRB1*13:01	P43358	MAGE4	119	133	38.09
1950	HFLLRKYRAKELVTK	DRB1*13:11	P43358	MAGE4	119	133	38.75
1951	HFLLRKYRAKELVTK	DRB1*13:21	P43358	MAGE4	119	133	37.82
1952	HFLLRKYRAKELVTK	DRB1*15:01	P43358	MAGE4	119	133	13.46
1953	HFLLRKYRAKELVTK	DRB1*15:03	P43358	MAGE4	119	133	22.9
1954	HFLLRKYRAKELVTK	DRB1*15:06	P43358	MAGE4	119	133	13.46
1955	HFLLRKYRAKELVTK	DRB1*15:07	P43358	MAGE4	119	133	27.6
1956	HFLLRKYRAKELVTK	DRB1*15:15	P43358	MAGE4	119	133	27.56

1957	HFLLRKYRAKELVTK	DRB1*15:37	P43358	MAGE4	119	133	33.99
1958	HFLRNQPLTFALQLH	DRB1*01:01	P40967	PMEL17	231	245	39.84
1959	HFLRNQPLTFALQLH	DRB1*01:18	P40967	PMEL17	231	245	33.26
1960	HFLRNQPLTFALQLH	DRB1*01:20	P40967	PMEL17	231	245	45.8
1961	HFLRNQPLTFALQLH	DRB1*11:14	P40967	PMEL17	231	245	17.65
1962	HFLRNQPLTFALQLH	DRB1*13:02	P40967	PMEL17	231	245	17.65
1963	HFLRNQPLTFALQLH	DRB1*13:23	P40967	PMEL17	231	245	17.65
1964	HFLRNQPLTFALQLH	DRB1*13:96	P40967	PMEL17	231	245	23.4
1965	HFLRNQPLTFALQLH	DRB1*13:97	P40967	PMEL17	231	245	17.65
1966	HFVRALDMAKRTTHP	DRB1*11:42	P17643	TRP1	143	157	40.09
1967	HHAFVDSIFEQWLRR	DPB1*02:01	P14679	Tyrosinase	389	403	39.39
1968	HHAFVDSIFEQWLRR	DPB1*02:02	P14679	Tyrosinase	389	403	32.91
1969	HHAFVDSIFEQWLRR	DPB1*04:01	P14679	Tyrosinase	389	403	16.9
1970	HHAFVDSIFEQWLRR	DPB1*126:01	P14679	Tyrosinase	389	403	16.9
1971	HHAFVDSIFEQWLRR	DPB1*15:01	P14679	Tyrosinase	389	403	41.88
1972	HHAFVDSIFEQWLRR	DPB1*23:01	P14679	Tyrosinase	389	403	16.9
1973	HHAFVDSIFEQWLRR	DPB1*33:01	P14679	Tyrosinase	389	403	13.53
1974	HHAFVDSIFEQWLRR	DPB1*39:01	P14679	Tyrosinase	389	403	16.9
1975	HHAFVDSIFEQWLRR	DPB1*46:01	P14679	Tyrosinase	389	403	39.39
1976	HHAFVDSIFEQWLRR	DPB1*47:01	P14679	Tyrosinase	389	403	32.91
1977	HHAFVDSIFEQWLRR	DPB1*71:01	P14679	Tyrosinase	389	403	13.53
1978	HHAFVDSIFEQWLRR	DPB1*72:01	P14679	Tyrosinase	389	403	18.99
1979	HHAFVDSIFEQWLRR	DPB1*81:01	P14679	Tyrosinase	389	403	39.39
1980	HLAVIGALLAVGATK	DRB1*01:01	P40967	PMEL17	11	25	11.34
1981	HLAVIGALLAVGATK	DRB1*01:02	P40967	PMEL17	11	25	33.77
1982	HLAVIGALLAVGATK	DRB1*01:11	P40967	PMEL17	11	25	37.11
1983	HLAVIGALLAVGATK	DRB1*01:18	P40967	PMEL17	11	25	10.72
1984	HLAVIGALLAVGATK	DRB1*01:20	P40967	PMEL17	11	25	9.92
1985	HLAVIGALLAVGATK	DRB1*01:24	P40967	PMEL17	11	25	20.49
1986	HLAVIGALLAVGATK	DRB1*01:29	P40967	PMEL17	11	25	20.77
1987	HLAVIGALLAVGATK	DRB1*10:01	P40967	PMEL17	11	25	31.61
1988	HNALHIYMNGTMSQV	DRB1*01:01	P14679	Tyrosinase	363	377	35.6
1989	HNALHIYMNGTMSQV	DRB1*01:18	P14679	Tyrosinase	363	377	33.33
1990	HNALHIYMNGTMSQV	DRB1*01:20	P14679	Tyrosinase	363	377	39.75
1991	HNALHIYMNGTMSQV	DRB1*11:14	P14679	Tyrosinase	363	377	23.28
1992	HNALHIYMNGTMSQV	DRB1*13:02	P14679	Tyrosinase	363	377	23.28
1993	HNALHIYMNGTMSQV	DRB1*13:23	P14679	Tyrosinase	363	377	23.28
1994	HNALHIYMNGTMSQV	DRB1*13:96	P14679	Tyrosinase	363	377	47.7
1995	HNALHIYMNGTMSQV	DRB1*13:97	P14679	Tyrosinase	363	377	23.28
1996	HNALHIYMNGTMSQV	DRB1*15:01	P14679	Tyrosinase	363	377	21.63

1997	HNALHIYMNGTMSQV	DRB1*15:06	P14679	Tyrosinase	363	377	21.63
1998	HNALHIYMNGTMSQV	DRB1*15:07	P14679	Tyrosinase	363	377	45.9
1999	HNRESYMVPFIPLYR	DPB1*02:01	P14679	Tyrosinase	420	434	45.51
2000	HNRESYMVPFIPLYR	DPB1*02:02	P14679	Tyrosinase	420	434	43.13
2001	HNRESYMVPFIPLYR	DPB1*04:01	P14679	Tyrosinase	420	434	48.89
2002	HNRESYMVPFIPLYR	DPB1*126:01	P14679	Tyrosinase	420	434	48.89
2003	HNRESYMVPFIPLYR	DPB1*15:01	P14679	Tyrosinase	420	434	37.46
2004	HNRESYMVPFIPLYR	DPB1*23:01	P14679	Tyrosinase	420	434	48.89
2005	HNRESYMVPFIPLYR	DPB1*33:01	P14679	Tyrosinase	420	434	17.46
2006	HNRESYMVPFIPLYR	DPB1*39:01	P14679	Tyrosinase	420	434	48.89
2007	HNRESYMVPFIPLYR	DPB1*46:01	P14679	Tyrosinase	420	434	45.51
2008	HNRESYMVPFIPLYR	DPB1*47:01	P14679	Tyrosinase	420	434	43.13
2009	HNRESYMVPFIPLYR	DPB1*71:01	P14679	Tyrosinase	420	434	17.46
2010	HNRESYMVPFIPLYR	DPB1*72:01	P14679	Tyrosinase	420	434	43
2011	HNRESYMVPFIPLYR	DPB1*81:01	P14679	Tyrosinase	420	434	45.51
2012	HPLFVIATRRSEEIL	DRB1*01:01	P17643	TRP1	156	170	40.83
2013	HPLFVIATRRSEEIL	DRB1*01:18	P17643	TRP1	156	170	26.05
2014	HPLFVIATRRSEEIL	DRB1*01:20	P17643	TRP1	156	170	20.71
2015	HPLFVIATRRSEEIL	DRB1*04:04	P17643	TRP1	156	170	26.21
2016	HPLFVIATRRSEEIL	DRB1*08:01	P17643	TRP1	156	170	30.11
2017	HPLFVIATRRSEEIL	DRB1*08:02	P17643	TRP1	156	170	46.3
2018	HPLFVIATRRSEEIL	DRB1*08:04	P17643	TRP1	156	170	25.99
2019	HPLFVIATRRSEEIL	DRB1*08:24	P17643	TRP1	156	170	39.93
2020	HPLFVIATRRSEEIL	DRB1*10:01	P17643	TRP1	156	170	38.31
2021	HPLFVIATRRSEEIL	DRB1*11:01	P17643	TRP1	156	170	13.74
2022	HPLFVIATRRSEEIL	DRB1*11:02	P17643	TRP1	156	170	40.79
2023	HPLFVIATRRSEEIL	DRB1*11:03	P17643	TRP1	156	170	26.12
2024	HPLFVIATRRSEEIL	DRB1*11:04	P17643	TRP1	156	170	12.52
2025	HPLFVIATRRSEEIL	DRB1*11:08	P17643	TRP1	156	170	26.9
2026	HPLFVIATRRSEEIL	DRB1*11:10	P17643	TRP1	156	170	13.74
2027	HPLFVIATRRSEEIL	DRB1*11:11	P17643	TRP1	156	170	45.73
2028	HPLFVIATRRSEEIL	DRB1*11:12	P17643	TRP1	156	170	13.74
2029	HPLFVIATRRSEEIL	DRB1*11:13	P17643	TRP1	156	170	36.5
2030	HPLFVIATRRSEEIL	DRB1*11:28	P17643	TRP1	156	170	13.74
2031	HPLFVIATRRSEEIL	DRB1*11:29	P17643	TRP1	156	170	13.74
2032	HPLFVIATRRSEEIL	DRB1*11:37	P17643	TRP1	156	170	29.24
2033	HPLFVIATRRSEEIL	DRB1*11:42	P17643	TRP1	156	170	15.43
2034	HPLFVIATRRSEEIL	DRB1*11:46	P17643	TRP1	156	170	12.52
2035	HPLFVIATRRSEEIL	DRB1*11:49	P17643	TRP1	156	170	13.74
2036	HPLFVIATRRSEEIL	DRB1*11:58	P17643	TRP1	156	170	12.52

2037	HPLFVIATRRSEEIL	DRB1*11:62	P17643	TRP1	156	170	13.74
2038	HPLFVIATRRSEEIL	DRB1*11:65	P17643	TRP1	156	170	40.79
2039	HPLFVIATRRSEEIL	DRB1*11:74	P17643	TRP1	156	170	13.74
2040	HPLFVIATRRSEEIL	DRB1*11:84	P17643	TRP1	156	170	40.18
2041	HPLFVIATRRSEEIL	DRB1*13:01	P17643	TRP1	156	170	40.79
2042	HPLFVIATRRSEEIL	DRB1*13:05	P17643	TRP1	156	170	13.74
2043	HPLFVIATRRSEEIL	DRB1*13:07	P17643	TRP1	156	170	29.24
2044	HPLFVIATRRSEEIL	DRB1*13:11	P17643	TRP1	156	170	12.52
2045	HPLFVIATRRSEEIL	DRB1*13:14	P17643	TRP1	156	170	13.74
2046	HPLFVIATRRSEEIL	DRB1*13:21	P17643	TRP1	156	170	12
2047	HPLFVIATRRSEEIL	DRB1*13:50	P17643	TRP1	156	170	13.74
2048	HPLFVIATRRSEEIL	DRB1*13:66	P17643	TRP1	156	170	44.42
2049	HPLFVIATRRSEEIL	DRB1*14:32	P17643	TRP1	156	170	44.57
2050	HRQLQLSISSCLQQL	DRB1*01:01	P78358	NY-ESO-1	142	156	18.53
2051	HRQLQLSISSCLQQL	DRB1*01:02	P78358	NY-ESO-1	142	156	37.14
2052	HRQLQLSISSCLQQL	DRB1*01:11	P78358	NY-ESO-1	142	156	40.53
2053	HRQLQLSISSCLQQL	DRB1*01:18	P78358	NY-ESO-1	142	156	17.06
2054	HRQLQLSISSCLQQL	DRB1*01:20	P78358	NY-ESO-1	142	156	16.16
2055	HRQLQLSISSCLQQL	DRB1*01:24	P78358	NY-ESO-1	142	156	31.2
2056	HRQLQLSISSCLQQL	DRB1*01:29	P78358	NY-ESO-1	142	156	33.39
2057	HRQLQLSISSCLQQL	DRB1*07:01	P78358	NY-ESO-1	142	156	13.69
2058	HRQLQLSISSCLQQL	DRB1*09:01	P78358	NY-ESO-1	142	156	36.07
2059	HRQLQLSISSCLQQL	DRB1*11:14	P78358	NY-ESO-1	142	156	36.44
2060	HRQLQLSISSCLQQL	DRB1*13:02	P78358	NY-ESO-1	142	156	36.44
2061	HRQLQLSISSCLQQL	DRB1*13:23	P78358	NY-ESO-1	142	156	36.44
2062	HRQLQLSISSCLQQL	DRB1*13:97	P78358	NY-ESO-1	142	156	36.44
2063	HRQLQLSISSCLQQL	DRB1*14:32	P78358	NY-ESO-1	142	156	44.18
2064	HTISSDYVIPIGTYG	DRB1*03:11	P14679	Tyrosinase	143	157	33.4
2065	HTMEVTVYHRRGSRS	DRB1*11:03	P40967	PMEL17	182	196	29.26
2066	HVVRVNARVRIAYPS	DRB1*03:11	P43358	MAGE4	290	304	33.12
2067	HVVRVNARVRIAYPS	DRB1*11:02	P43358	MAGE4	290	304	12.34
2068	HVVRVNARVRIAYPS	DRB1*11:03	P43358	MAGE4	290	304	17.33
2069	HVVRVNARVRIAYPS	DRB1*11:04	P43358	MAGE4	290	304	39.92
2070	HVVRVNARVRIAYPS	DRB1*11:13	P43358	MAGE4	290	304	18.31
2071	HVVRVNARVRIAYPS	DRB1*11:14	P43358	MAGE4	290	304	23.63
2072	HVVRVNARVRIAYPS	DRB1*11:42	P43358	MAGE4	290	304	18.19
2073	HVVRVNARVRIAYPS	DRB1*11:46	P43358	MAGE4	290	304	39.92
2074	HVVRVNARVRIAYPS	DRB1*11:58	P43358	MAGE4	290	304	39.92
2075	HVVRVNARVRIAYPS	DRB1*11:65	P43358	MAGE4	290	304	12.34
2076	HVVRVNARVRIAYPS	DRB1*13:01	P43358	MAGE4	290	304	12.34

2077	HVVRVNARVRIAYPS	DRB1*13:02	P43358	MAGE4	290	304	23.63
2078	HVVRVNARVRIAYPS	DRB1*13:11	P43358	MAGE4	290	304	39.92
2079	HVVRVNARVRIAYPS	DRB1*13:23	P43358	MAGE4	290	304	23.63
2080	HVVRVNARVRIAYPS	DRB1*13:61	P43358	MAGE4	290	304	21.02
2081	HVVRVNARVRIAYPS	DRB1*13:96	P43358	MAGE4	290	304	32.56
2082	HVVRVNARVRIAYPS	DRB1*13:97	P43358	MAGE4	290	304	23.63
2083	HVVRVNARVRIAYPS	DRB1*14:06	P43358	MAGE4	290	304	31.3
2084	HVVRVNARVRIAYPS	DRB1*14:32	P43358	MAGE4	290	304	26.51
2085	HYYVSMDALLGGSEI	DRB1*01:01	P14679	Tyrosinase	180	194	7.66
2086	HYYVSMDALLGGSEI	DRB1*01:11	P14679	Tyrosinase	180	194	25.25
2087	HYYVSMDALLGGSEI	DRB1*01:18	P14679	Tyrosinase	180	194	8.3
2088	HYYVSMDALLGGSEI	DRB1*01:20	P14679	Tyrosinase	180	194	27.74
2089	HYYVSMDALLGGSEI	DRB1*01:24	P14679	Tyrosinase	180	194	20.06
2090	HYYVSMDALLGGSEI	DRB1*01:29	P14679	Tyrosinase	180	194	15.66
2091	HYYVSMDALLGGSEI	DRB1*04:01	P14679	Tyrosinase	180	194	42.5
2092	HYYVSMDALLGGSEI	DRB1*04:08	P14679	Tyrosinase	180	194	36.79
2093	HYYVSMDALLGGSEI	DRB1*10:01	P14679	Tyrosinase	180	194	10.58
2094	IDFAHEAPAFLPWHR	DRB1*01:01	P14679	Tyrosinase	198	212	23.67
2095	IDFAHEAPAFLPWHR	DRB1*01:18	P14679	Tyrosinase	198	212	29.19
2096	IDFAHEAPAFLPWHR	DRB1*10:01	P14679	Tyrosinase	198	212	43.41
2097	IFEQWLRRHRPLQEV	DRB1*11:03	P14679	Tyrosinase	396	410	44.32
2098	IFFPLLLFQQARAQF	DRB1*01:01	P17643	TRP1	12	26	37.15
2099	IFFPLLLFQQARAQF	DRB1*01:18	P17643	TRP1	12	26	23.88
2100	IFFPLLLFQQARAQF	DRB1*01:20	P17643	TRP1	12	26	17.4
2101	IFFPLLLFQQARAQF	DRB1*10:01	P17643	TRP1	12	26	31.15
2102	IFFPLLLFQQARAQF	DRB1*11:04	P17643	TRP1	12	26	47.75
2103	IFFPLLLFQQARAQF	DRB1*11:42	P17643	TRP1	12	26	39.05
2104	IFFPLLLFQQARAQF	DRB1*11:46	P17643	TRP1	12	26	47.75
2105	IFFPLLLFQQARAQF	DRB1*11:58	P17643	TRP1	12	26	47.75
2106	IFFPLLLFQQARAQF	DRB1*13:11	P17643	TRP1	12	26	47.75
2107	IFFPLLLFQQARAQF	DRB1*15:01	P17643	TRP1	12	26	45.23
2108	IFFPLLLFQQARAQF	DRB1*15:06	P17643	TRP1	12	26	45.23
2109	IFGTASYLIRARRSM	DRB1*01:20	P17643	TRP1	493	507	39
2110	IFGTASYLIRARRSM	DRB1*11:02	P17643	TRP1	493	507	39.73
2111	IFGTASYLIRARRSM	DRB1*11:03	P17643	TRP1	493	507	28
2112	IFGTASYLIRARRSM	DRB1*11:04	P17643	TRP1	493	507	30.93
2113	IFGTASYLIRARRSM	DRB1*11:42	P17643	TRP1	493	507	26.88
2114	IFGTASYLIRARRSM	DRB1*11:46	P17643	TRP1	493	507	30.93
2115	IFGTASYLIRARRSM	DRB1*11:58	P17643	TRP1	493	507	30.93
2116	IFGTASYLIRARRSM	DRB1*11:65	P17643	TRP1	493	507	39.73

2117	IFGTASYLIRARRSM	DRB1*13:01	P17643	TRP1	493	507	39.73
2118	IFGTASYLIRARRSM	DRB1*13:11	P17643	TRP1	493	507	30.93
2119	IFGTASYLIRARRSM	DRB1*13:21	P17643	TRP1	493	507	47.58
2120	IFLLHHAFVDSIFEQ	DPB1*02:01	P14679	Tyrosinase	385	399	33.72
2121	IFLLHHAFVDSIFEQ	DPB1*02:02	P14679	Tyrosinase	385	399	34.65
2122	IFLLHHAFVDSIFEQ	DPB1*33:01	P14679	Tyrosinase	385	399	17.14
2123	IFLLHHAFVDSIFEQ	DPB1*46:01	P14679	Tyrosinase	385	399	33.72
2124	IFLLHHAFVDSIFEQ	DPB1*47:01	P14679	Tyrosinase	385	399	34.65
2125	IFLLHHAFVDSIFEQ	DPB1*71:01	P14679	Tyrosinase	385	399	17.14
2126	IFLLHHAFVDSIFEQ	DPB1*81:01	P14679	Tyrosinase	385	399	33.72
2127	IFLLHHAFVDSIFEQ	DRB1*01:18	P14679	Tyrosinase	385	399	49.67
2128	IFVLLHTFTDAVFDE	DPB1*33:01	P17643	TRP1	399	413	47.53
2129	IFVLLHTFTDAVFDE	DPB1*71:01	P17643	TRP1	399	413	47.53
2130	IFVLLHTFTDAVFDE	DRB1*04:05	P17643	TRP1	399	413	44.63
2131	IFVLLHTFTDAVFDE	DRB1*10:01	P17643	TRP1	399	413	38.32
2132	IFYVYMKRKYEAMTK	DRB1*08:01	Q16385	SSX2	46	60	40.75
2133	IFYVYMKRKYEAMTK	DRB1*08:04	Q16385	SSX2	46	60	43.16
2134	IFYVYMKRKYEAMTK	DRB1*11:01	Q16385	SSX2	46	60	13.87
2135	IFYVYMKRKYEAMTK	DRB1*11:02	Q16385	SSX2	46	60	28.77
2136	IFYVYMKRKYEAMTK	DRB1*11:03	Q16385	SSX2	46	60	13.27
2137	IFYVYMKRKYEAMTK	DRB1*11:04	Q16385	SSX2	46	60	11.71
2138	IFYVYMKRKYEAMTK	DRB1*11:08	Q16385	SSX2	46	60	42.52
2139	IFYVYMKRKYEAMTK	DRB1*11:10	Q16385	SSX2	46	60	13.87
2140	IFYVYMKRKYEAMTK	DRB1*11:11	Q16385	SSX2	46	60	31.55
2141	IFYVYMKRKYEAMTK	DRB1*11:12	Q16385	SSX2	46	60	13.87
2142	IFYVYMKRKYEAMTK	DRB1*11:13	Q16385	SSX2	46	60	40.57
2143	IFYVYMKRKYEAMTK	DRB1*11:28	Q16385	SSX2	46	60	13.87
2144	IFYVYMKRKYEAMTK	DRB1*11:29	Q16385	SSX2	46	60	13.87
2145	IFYVYMKRKYEAMTK	DRB1*11:37	Q16385	SSX2	46	60	31.32
2146	IFYVYMKRKYEAMTK	DRB1*11:42	Q16385	SSX2	46	60	19.51
2147	IFYVYMKRKYEAMTK	DRB1*11:46	Q16385	SSX2	46	60	11.71
2148	IFYVYMKRKYEAMTK	DRB1*11:49	Q16385	SSX2	46	60	13.87
2149	IFYVYMKRKYEAMTK	DRB1*11:58	Q16385	SSX2	46	60	11.71
2150	IFYVYMKRKYEAMTK	DRB1*11:62	Q16385	SSX2	46	60	13.87
2151	IFYVYMKRKYEAMTK	DRB1*11:65	Q16385	SSX2	46	60	28.77
2152	IFYVYMKRKYEAMTK	DRB1*11:74	Q16385	SSX2	46	60	13.87
2153	IFYVYMKRKYEAMTK	DRB1*11:84	Q16385	SSX2	46	60	35.97
2154	IFYVYMKRKYEAMTK	DRB1*13:01	Q16385	SSX2	46	60	28.77
2155	IFYVYMKRKYEAMTK	DRB1*13:05	Q16385	SSX2	46	60	13.87
2156	IFYVYMKRKYEAMTK	DRB1*13:07	Q16385	SSX2	46	60	31.32

2157	IFYVYMKRKYEAMTK	DRB1*13:11	Q16385	SSX2	46	60	11.71
2158	IFYVYMKRKYEAMTK	DRB1*13:14	Q16385	SSX2	46	60	13.87
2159	IFYVYMKRKYEAMTK	DRB1*13:21	Q16385	SSX2	46	60	9.31
2160	IFYVYMKRKYEAMTK	DRB1*13:50	Q16385	SSX2	46	60	13.87
2161	IGALLAVGATKVPRN	DRB1*01:01	P40967	PMEL17	15	29	7.11
2162	IGALLAVGATKVPRN	DRB1*01:02	P40967	PMEL17	15	29	20.27
2163	IGALLAVGATKVPRN	DRB1*01:11	P40967	PMEL17	15	29	17.66
2164	IGALLAVGATKVPRN	DRB1*01:18	P40967	PMEL17	15	29	7.12
2165	IGALLAVGATKVPRN	DRB1*01:20	P40967	PMEL17	15	29	7.5
2166	IGALLAVGATKVPRN	DRB1*01:24	P40967	PMEL17	15	29	14.57
2167	IGALLAVGATKVPRN	DRB1*01:29	P40967	PMEL17	15	29	13.52
2168	IGALLAVGATKVPRN	DRB1*07:01	P40967	PMEL17	15	29	18.72
2169	IGALLAVGATKVPRN	DRB1*10:01	P40967	PMEL17	15	29	43.24
2170	IGALLAVGATKVPRN	DRB1*11:14	P40967	PMEL17	15	29	34.24
2171	IGALLAVGATKVPRN	DRB1*13:02	P40967	PMEL17	15	29	34.24
2172	IGALLAVGATKVPRN	DRB1*13:23	P40967	PMEL17	15	29	34.24
2173	IGALLAVGATKVPRN	DRB1*13:97	P40967	PMEL17	15	29	34.24
2174	IGANASFSIALNFPG	DRB1*01:01	P40967	PMEL17	78	92	39.8
2175	IGANASFSIALNFPG	DRB1*01:18	P40967	PMEL17	78	92	36.37
2176	IGANASFSIALNFPG	DRB1*04:01	P40967	PMEL17	78	92	43.78
2177	IGANASFSIALNFPG	DRB1*04:05	P40967	PMEL17	78	92	45.54
2178	IGANASFSIALNFPG	DRB1*04:08	P40967	PMEL17	78	92	43.75
2179	IGANASFSIALNFPG	DRB1*09:01	P40967	PMEL17	78	92	48.64
2180	IGANASFSIALNFPG	DRB1*10:01	P40967	PMEL17	78	92	29.88
2181	IGHNRESYMVPFIPL	DPB1*33:01	P14679	Tyrosinase	418	432	35.97
2182	IGHNRESYMVPFIPL	DPB1*71:01	P14679	Tyrosinase	418	432	35.97
2183	IGLRVWQWEVISCKL	DPB1*33:01	O75767	TRP2	214	228	34.14
2184	IGLRVWQWEVISCKL	DPB1*71:01	O75767	TRP2	214	228	34.14
2185	IIVLVMIAMEGGHAP	DRB1*01:01	P43355	MAGE1	196	210	15.82
2186	IIVLVMIAMEGGHAP	DRB1*01:02	P43355	MAGE1	196	210	40
2187	IIVLVMIAMEGGHAP	DRB1*01:18	P43355	MAGE1	196	210	17.62
2188	IIVLVMIAMEGGHAP	DRB1*01:20	P43355	MAGE1	196	210	12.7
2189	IIVLVMIAMEGGHAP	DRB1*01:29	P43355	MAGE1	196	210	43.02
2190	IKNYKRCFPVIFGKA	DRB1*01:01	P43358	MAGE4	141	155	38.57
2191	IKNYKRCFPVIFGKA	DRB1*01:18	P43358	MAGE4	141	155	31.43
2192	IKSYLEQASRIWSWL	DRB1*01:01	P14679	Tyrosinase	464	478	33.81
2193	IKSYLEQASRIWSWL	DRB1*01:18	P14679	Tyrosinase	464	478	28.18
2194	IKSYLEQASRIWSWL	DRB1*11:04	P14679	Tyrosinase	464	478	46.23
2195	IKSYLEQASRIWSWL	DRB1*11:42	P14679	Tyrosinase	464	478	49.53
2196	IKSYLEQASRIWSWL	DRB1*11:46	P14679	Tyrosinase	464	478	46.23

2197	IKSYLEQASRIWSWL	DRB1*11:58	P14679	Tyrosinase	464	478	46.23
2198	IKSYLEQASRIWSWL	DRB1*13:11	P14679	Tyrosinase	464	478	46.23
2199	IKVSARVRFFFPSLR	DPB1*33:01	P43355	MAGE1	284	298	44.3
2200	IKVSARVRFFFPSLR	DPB1*71:01	P43355	MAGE1	284	298	44.3
2201	IKVSARVRFFFPSLR	DRB1*11:02	P43355	MAGE1	284	298	40.89
2202	IKVSARVRFFFPSLR	DRB1*11:65	P43355	MAGE1	284	298	40.89
2203	IKVSARVRFFFPSLR	DRB1*13:01	P43355	MAGE1	284	298	40.89
2204	ILESLFRAVITKKVA	DPB1*33:01	P43355	MAGE1	93	107	41.69
2205	ILESLFRAVITKKVA	DPB1*71:01	P43355	MAGE1	93	107	41.69
2206	ILESLFRAVITKKVA	DRB1*01:01	P43355	MAGE1	93	107	7.2
2207	ILESLFRAVITKKVA	DRB1*01:11	P43355	MAGE1	93	107	17.96
2208	ILESLFRAVITKKVA	DRB1*01:18	P43355	MAGE1	93	107	6.54
2209	ILESLFRAVITKKVA	DRB1*01:20	P43355	MAGE1	93	107	17.3
2210	ILESLFRAVITKKVA	DRB1*01:24	P43355	MAGE1	93	107	14.71
2211	ILESLFRAVITKKVA	DRB1*01:29	P43355	MAGE1	93	107	11.49
2212	ILESLFRAVITKKVA	DRB1*07:01	P43355	MAGE1	93	107	7.63
2213	ILESLFRAVITKKVA	DRB1*09:01	P43355	MAGE1	93	107	20.12
2214	ILESLFRAVITKKVA	DRB1*10:01	P43355	MAGE1	93	107	15.8
2215	ILESLFRAVITKKVA	DRB1*11:01	P43355	MAGE1	93	107	32.17
2216	ILESLFRAVITKKVA	DRB1*11:04	P43355	MAGE1	93	107	36.18
2217	ILESLFRAVITKKVA	DRB1*11:08	P43355	MAGE1	93	107	37.5
2218	ILESLFRAVITKKVA	DRB1*11:10	P43355	MAGE1	93	107	32.17
2219	ILESLFRAVITKKVA	DRB1*11:12	P43355	MAGE1	93	107	32.17
2220	ILESLFRAVITKKVA	DRB1*11:13	P43355	MAGE1	93	107	41.6
2221	ILESLFRAVITKKVA	DRB1*11:28	P43355	MAGE1	93	107	32.17
2222	ILESLFRAVITKKVA	DRB1*11:29	P43355	MAGE1	93	107	32.17
2223	ILESLFRAVITKKVA	DRB1*11:42	P43355	MAGE1	93	107	31.61
2224	ILESLFRAVITKKVA	DRB1*11:46	P43355	MAGE1	93	107	36.18
2225	ILESLFRAVITKKVA	DRB1*11:49	P43355	MAGE1	93	107	32.17
2226	ILESLFRAVITKKVA	DRB1*11:58	P43355	MAGE1	93	107	36.18
2227	ILESLFRAVITKKVA	DRB1*11:62	P43355	MAGE1	93	107	32.17
2228	ILESLFRAVITKKVA	DRB1*11:74	P43355	MAGE1	93	107	32.17
2229	ILESLFRAVITKKVA	DRB1*12:16	P43355	MAGE1	93	107	40.39
2230	ILESLFRAVITKKVA	DRB1*13:05	P43355	MAGE1	93	107	32.17
2231	ILESLFRAVITKKVA	DRB1*13:11	P43355	MAGE1	93	107	36.18
2232	ILESLFRAVITKKVA	DRB1*13:14	P43355	MAGE1	93	107	32.17
2233	ILESLFRAVITKKVA	DRB1*13:21	P43355	MAGE1	93	107	29.28
2234	ILESLFRAVITKKVA	DRB1*13:50	P43355	MAGE1	93	107	32.17
2235	ILESLFRAVITKKVA	DRB1*14:32	P43355	MAGE1	93	107	45.94
2236	ILESLFRAVITKKVA	DRB1*15:15	P43355	MAGE1	93	107	26.52

2237	ILESLFRAVITKKVA	DRB1*16:01	P43355	MAGE1	93	107	20.87
2238	ILESLFRAVITKKVA	DRB1*16:02	P43355	MAGE1	93	107	20.52
2239	ILESLFRAVITKKVA	DRB1*16:04	P43355	MAGE1	93	107	34.19
2240	ILESLFRAVITKKVA	DRB1*16:05	P43355	MAGE1	93	107	45.32
2241	ILESLFRAVITKKVA	DRB1*16:09	P43355	MAGE1	93	107	19.39
2242	ILTIRLTAADHRQLQ	DRB1*01:20	P78358	NY-ESO-1	132	146	36.07
2243	ILTIRLTAADHRQLQ	DRB1*03:11	P78358	NY-ESO-1	132	146	45.97
2244	ILTIRLTAADHRQLQ	DRB1*11:02	P78358	NY-ESO-1	132	146	42.73
2245	ILTIRLTAADHRQLQ	DRB1*11:13	P78358	NY-ESO-1	132	146	40.41
2246	ILTIRLTAADHRQLQ	DRB1*11:42	P78358	NY-ESO-1	132	146	36.9
2247	ILTIRLTAADHRQLQ	DRB1*11:65	P78358	NY-ESO-1	132	146	42.73
2248	ILTIRLTAADHRQLQ	DRB1*13:01	P78358	NY-ESO-1	132	146	42.73
2249	INIYDLFVWMHYYVS	DPB1*33:01	P14679	Tyrosinase	170	184	40.06
2250	INIYDLFVWMHYYVS	DPB1*71:01	P14679	Tyrosinase	170	184	40.06
2251	ISIYNYFVWTHYYSV	DPB1*01:01	P17643	TRP1	182	196	23.26
2252	ISIYNYFVWTHYYSV	DPB1*02:01	P17643	TRP1	182	196	9.41
2253	ISIYNYFVWTHYYSV	DPB1*02:02	P17643	TRP1	182	196	9.43
2254	ISIYNYFVWTHYYSV	DPB1*04:01	P17643	TRP1	182	196	6.95
2255	ISIYNYFVWTHYYSV	DPB1*04:02	P17643	TRP1	182	196	33.93
2256	ISIYNYFVWTHYYSV	DPB1*105:01	P17643	TRP1	182	196	33.93
2257	ISIYNYFVWTHYYSV	DPB1*126:01	P17643	TRP1	182	196	6.95
2258	ISIYNYFVWTHYYSV	DPB1*15:01	P17643	TRP1	182	196	24.75
2259	ISIYNYFVWTHYYSV	DPB1*16:01	P17643	TRP1	182	196	34.13
2260	ISIYNYFVWTHYYSV	DPB1*23:01	P17643	TRP1	182	196	6.95
2261	ISIYNYFVWTHYYSV	DPB1*33:01	P17643	TRP1	182	196	4.95
2262	ISIYNYFVWTHYYSV	DPB1*34:01	P17643	TRP1	182	196	45.77
2263	ISIYNYFVWTHYYSV	DPB1*39:01	P17643	TRP1	182	196	6.95
2264	ISIYNYFVWTHYYSV	DPB1*40:01	P17643	TRP1	182	196	32.71
2265	ISIYNYFVWTHYYSV	DPB1*41:01	P17643	TRP1	182	196	39.46
2266	ISIYNYFVWTHYYSV	DPB1*46:01	P17643	TRP1	182	196	9.41
2267	ISIYNYFVWTHYYSV	DPB1*47:01	P17643	TRP1	182	196	9.43
2268	ISIYNYFVWTHYYSV	DPB1*49:01	P17643	TRP1	182	196	33.93
2269	ISIYNYFVWTHYYSV	DPB1*71:01	P17643	TRP1	182	196	4.95
2270	ISIYNYFVWTHYYSV	DPB1*72:01	P17643	TRP1	182	196	9.01
2271	ISIYNYFVWTHYYSV	DPB1*81:01	P17643	TRP1	182	196	9.41
2272	ISIYNYFVWTHYYSV	DRB1*07:01	P17643	TRP1	182	196	39.49
2273	ITKKVADLVGFLLLK	DRB1*01:01	P43355	MAGE1	102	116	25.64
2274	ITKKVADLVGFLLLK	DRB1*01:18	P43355	MAGE1	102	116	17.93
2275	ITKKVADLVGFLLLK	DRB1*01:20	P43355	MAGE1	102	116	19.09
2276	ITTQHWVGLLGPNGT	DRB1*01:01	O75767	TRP2	158	172	28.99

2277	ITTQHWVGLLGPNGT	DRB1*01:18	O75767	TRP2	158	172	38.08
2278	IVLVMIAMEGGHAPE	DRB1*01:01	P43355	MAGE1	197	211	14.68
2279	IVLVMIAMEGGHAPE	DRB1*01:02	P43355	MAGE1	197	211	35.73
2280	IVLVMIAMEGGHAPE	DRB1*01:18	P43355	MAGE1	197	211	15.98
2281	IVLVMIAMEGGHAPE	DRB1*01:20	P43355	MAGE1	197	211	12.17
2282	IVLVMIAMEGGHAPE	DRB1*01:24	P43355	MAGE1	197	211	49.25
2283	IVLVMIAMEGGHAPE	DRB1*01:29	P43355	MAGE1	197	211	39.03
2284	IVVLSGTTAAQVTTT	DRB1*01:01	P40967	PMEL17	407	421	32.73
2285	IVVLSGTTAAQVTTT	DRB1*01:18	P40967	PMEL17	407	421	27.09
2286	IVVLSGTTAAQVTTT	DRB1*01:20	P40967	PMEL17	407	421	23.88
2287	IVVLSGTTAAQVTTT	DRB1*07:01	P40967	PMEL17	407	421	33.68
2288	IWSWLLGAAMVGAVL	DRB1*01:01	P14679	Tyrosinase	474	488	26.95
2289	IWSWLLGAAMVGAVL	DRB1*01:18	P14679	Tyrosinase	474	488	20.43
2290	IWSWLLGAAMVGAVL	DRB1*01:24	P14679	Tyrosinase	474	488	33.81
2291	IWSWLLGAAMVGAVL	DRB1*01:29	P14679	Tyrosinase	474	488	43.99
2292	IYDLFVWMHYYVSMD	DPB1*33:01	P14679	Tyrosinase	172	186	19.04
2293	IYDLFVWMHYYVSMD	DPB1*71:01	P14679	Tyrosinase	172	186	19.04
2294	IYNYFVWTHYYSVKK	DPB1*01:01	P17643	TRP1	184	198	15.24
2295	IYNYFVWTHYYSVKK	DPB1*02:01	P17643	TRP1	184	198	4.57
2296	IYNYFVWTHYYSVKK	DPB1*02:02	P17643	TRP1	184	198	5.36
2297	IYNYFVWTHYYSVKK	DPB1*04:01	P17643	TRP1	184	198	4.56
2298	IYNYFVWTHYYSVKK	DPB1*04:02	P17643	TRP1	184	198	13.91
2299	IYNYFVWTHYYSVKK	DPB1*105:01	P17643	TRP1	184	198	13.91
2300	IYNYFVWTHYYSVKK	DPB1*126:01	P17643	TRP1	184	198	4.56
2301	IYNYFVWTHYYSVKK	DPB1*15:01	P17643	TRP1	184	198	14.2
2302	IYNYFVWTHYYSVKK	DPB1*16:01	P17643	TRP1	184	198	13.16
2303	IYNYFVWTHYYSVKK	DPB1*19:01	P17643	TRP1	184	198	25.06
2304	IYNYFVWTHYYSVKK	DPB1*23:01	P17643	TRP1	184	198	4.56
2305	IYNYFVWTHYYSVKK	DPB1*33:01	P17643	TRP1	184	198	3.5
2306	IYNYFVWTHYYSVKK	DPB1*34:01	P17643	TRP1	184	198	19.23
2307	IYNYFVWTHYYSVKK	DPB1*39:01	P17643	TRP1	184	198	4.56
2308	IYNYFVWTHYYSVKK	DPB1*40:01	P17643	TRP1	184	198	17.63
2309	IYNYFVWTHYYSVKK	DPB1*41:01	P17643	TRP1	184	198	11.61
2310	IYNYFVWTHYYSVKK	DPB1*46:01	P17643	TRP1	184	198	4.57
2311	IYNYFVWTHYYSVKK	DPB1*47:01	P17643	TRP1	184	198	5.36
2312	IYNYFVWTHYYSVKK	DPB1*49:01	P17643	TRP1	184	198	13.91
2313	IYNYFVWTHYYSVKK	DPB1*55:01	P17643	TRP1	184	198	40.78
2314	IYNYFVWTHYYSVKK	DPB1*71:01	P17643	TRP1	184	198	3.5
2315	IYNYFVWTHYYSVKK	DPB1*72:01	P17643	TRP1	184	198	5.16
2316	IYNYFVWTHYYSVKK	DPB1*81:01	P17643	TRP1	184	198	4.57

2317	IYNYFVWTHYYSVKK	DRB1*01:01	P17643	TRP1	184	198	40.34
2318	IYNYFVWTHYYSVKK	DRB1*01:18	P17643	TRP1	184	198	31.29
2319	IYNYFVWTHYYSVKK	DRB1*07:01	P17643	TRP1	184	198	20.23
2320	IYNYFVWTHYYSVKK	DRB1*10:01	P17643	TRP1	184	198	47.37
2321	IYNYFVWTHYYSVKK	DRB1*15:02	P17643	TRP1	184	198	43.85
2322	IYNYFVWTHYYSVKK	DRB1*15:15	P17643	TRP1	184	198	33.53
2323	IYNYFVWTHYYSVKK	DRB1*16:09	P17643	TRP1	184	198	48.28
2324	KAEMLERVIKNYKRC	DRB1*11:42	P43358	MAGE4	133	147	44.35
2325	KAMVALIDVFHQYSG	DRB1*01:20	P04271	S100	4	18	47.4
2326	KAMVALIDVFHQYSG	DRB1*04:04	P04271	S100	4	18	29.93
2327	KAMVALIDVFHQYSG	DRB1*04:10	P04271	S100	4	18	48.8
2328	KAMVALIDVFHQYSG	DRB1*10:01	P04271	S100	4	18	46.68
2329	KASEKIFYVYMKRKY	DRB1*11:03	Q16385	SSX2	41	55	40.17
2330	KASEKIFYVYMKRKY	DRB1*11:42	Q16385	SSX2	41	55	48.84
2331	KASESLKMIFGIDVK	DRB1*01:01	P43358	MAGE4	154	168	13.8
2332	KASESLKMIFGIDVK	DRB1*01:02	P43358	MAGE4	154	168	33.5
2333	KASESLKMIFGIDVK	DRB1*01:11	P43358	MAGE4	154	168	43.85
2334	KASESLKMIFGIDVK	DRB1*01:18	P43358	MAGE4	154	168	13.6
2335	KASESLKMIFGIDVK	DRB1*01:20	P43358	MAGE4	154	168	11.33
2336	KASESLKMIFGIDVK	DRB1*01:24	P43358	MAGE4	154	168	26.68
2337	KASESLKMIFGIDVK	DRB1*01:29	P43358	MAGE4	154	168	28.91
2338	KASESLQLVFGIDVK	DRB1*01:01	P43355	MAGE1	146	160	47.23
2339	KASESLQLVFGIDVK	DRB1*01:18	P43355	MAGE1	146	160	31.52
2340	KASESLQLVFGIDVK	DRB1*01:20	P43355	MAGE1	146	160	37.02
2341	KDKFFAYLTLAKHTI	DPB1*02:01	P14679	Tyrosinase	131	145	46.71
2342	KDKFFAYLTLAKHTI	DPB1*02:02	P14679	Tyrosinase	131	145	43.02
2343	KDKFFAYLTLAKHTI	DPB1*33:01	P14679	Tyrosinase	131	145	23.41
2344	KDKFFAYLTLAKHTI	DPB1*46:01	P14679	Tyrosinase	131	145	46.71
2345	KDKFFAYLTLAKHTI	DPB1*47:01	P14679	Tyrosinase	131	145	43.02
2346	KDKFFAYLTLAKHTI	DPB1*71:01	P14679	Tyrosinase	131	145	23.41
2347	KDKFFAYLTLAKHTI	DPB1*81:01	P14679	Tyrosinase	131	145	46.71
2348	KDKFFAYLTLAKHTI	DRB1*01:01	P14679	Tyrosinase	131	145	19.53
2349	KDKFFAYLTLAKHTI	DRB1*01:18	P14679	Tyrosinase	131	145	15.02
2350	KDKFFAYLTLAKHTI	DRB1*01:20	P14679	Tyrosinase	131	145	29.67
2351	KDKFFAYLTLAKHTI	DRB1*01:24	P14679	Tyrosinase	131	145	47.12
2352	KDKFFAYLTLAKHTI	DRB1*01:29	P14679	Tyrosinase	131	145	32.17
2353	KDKFFAYLTLAKHTI	DRB1*10:01	P14679	Tyrosinase	131	145	8.52
2354	KDKFFAYLTLAKHTI	DRB1*11:01	P14679	Tyrosinase	131	145	33.33
2355	KDKFFAYLTLAKHTI	DRB1*11:08	P14679	Tyrosinase	131	145	49
2356	KDKFFAYLTLAKHTI	DRB1*11:10	P14679	Tyrosinase	131	145	33.33

2357	KDKFFAYLTLAKHTI	DRB1*11:12	P14679	Tyrosinase	131	145	33.33
2358	KDKFFAYLTLAKHTI	DRB1*11:28	P14679	Tyrosinase	131	145	33.33
2359	KDKFFAYLTLAKHTI	DRB1*11:29	P14679	Tyrosinase	131	145	33.33
2360	KDKFFAYLTLAKHTI	DRB1*11:49	P14679	Tyrosinase	131	145	33.33
2361	KDKFFAYLTLAKHTI	DRB1*11:62	P14679	Tyrosinase	131	145	33.33
2362	KDKFFAYLTLAKHTI	DRB1*11:74	P14679	Tyrosinase	131	145	33.33
2363	KDKFFAYLTLAKHTI	DRB1*12:16	P14679	Tyrosinase	131	145	37.91
2364	KDKFFAYLTLAKHTI	DRB1*13:05	P14679	Tyrosinase	131	145	33.33
2365	KDKFFAYLTLAKHTI	DRB1*13:14	P14679	Tyrosinase	131	145	33.33
2366	KDKFFAYLTLAKHTI	DRB1*13:21	P14679	Tyrosinase	131	145	25.02
2367	KDKFFAYLTLAKHTI	DRB1*13:50	P14679	Tyrosinase	131	145	33.33
2368	KDKFFAYLTLAKHTI	DRB1*15:02	P14679	Tyrosinase	131	145	47.17
2369	KDKFFAYLTLAKHTI	DRB1*15:15	P14679	Tyrosinase	131	145	27.49
2370	KDKFFAYLTLAKHTI	DRB1*16:01	P14679	Tyrosinase	131	145	46.43
2371	KDKFFAYLTLAKHTI	DRB1*16:02	P14679	Tyrosinase	131	145	40.61
2372	KDKFFAYLTLAKHTI	DRB1*16:09	P14679	Tyrosinase	131	145	28.43
2373	KEFTVSGNILTIRLT	DRB1*01:01	P78358	NY-ESO-1	124	138	12.95
2374	KEFTVSGNILTIRLT	DRB1*01:11	P78358	NY-ESO-1	124	138	28.32
2375	KEFTVSGNILTIRLT	DRB1*01:18	P78358	NY-ESO-1	124	138	13.9
2376	KEFTVSGNILTIRLT	DRB1*01:20	P78358	NY-ESO-1	124	138	40.53
2377	KEFTVSGNILTIRLT	DRB1*01:24	P78358	NY-ESO-1	124	138	21.45
2378	KEFTVSGNILTIRLT	DRB1*01:29	P78358	NY-ESO-1	124	138	30.74
2379	KEFTVSGNILTIRLT	DRB1*04:01	P78358	NY-ESO-1	124	138	33.31
2380	KEFTVSGNILTIRLT	DRB1*04:08	P78358	NY-ESO-1	124	138	34.63
2381	KEFTVSGNILTIRLT	DRB1*04:72	P78358	NY-ESO-1	124	138	41
2382	KEFTVSGNILTIRLT	DRB1*07:01	P78358	NY-ESO-1	124	138	33.96
2383	KEFTVSGNILTIRLT	DRB1*10:01	P78358	NY-ESO-1	124	138	32.78
2384	KEFTVSGNILTIRLT	DRB1*11:14	P78358	NY-ESO-1	124	138	36.33
2385	KEFTVSGNILTIRLT	DRB1*13:02	P78358	NY-ESO-1	124	138	36.33
2386	KEFTVSGNILTIRLT	DRB1*13:23	P78358	NY-ESO-1	124	138	36.33
2387	KEFTVSGNILTIRLT	DRB1*13:97	P78358	NY-ESO-1	124	138	36.33
2388	KEFTVSGNILTIRLT	DRB1*16:02	P78358	NY-ESO-1	124	138	48.72
2389	KFFAYLTLAKHTISS	DPB1*33:01	P14679	Tyrosinase	133	147	48.9
2390	KFFAYLTLAKHTISS	DPB1*71:01	P14679	Tyrosinase	133	147	48.9
2391	KFFAYLTLAKHTISS	DRB1*01:01	P14679	Tyrosinase	133	147	18
2392	KFFAYLTLAKHTISS	DRB1*01:11	P14679	Tyrosinase	133	147	49.54
2393	KFFAYLTLAKHTISS	DRB1*01:18	P14679	Tyrosinase	133	147	14.59
2394	KFFAYLTLAKHTISS	DRB1*01:20	P14679	Tyrosinase	133	147	26.01
2395	KFFAYLTLAKHTISS	DRB1*01:24	P14679	Tyrosinase	133	147	38.64
2396	KFFAYLTLAKHTISS	DRB1*01:29	P14679	Tyrosinase	133	147	29.56

2397	KFFAYLTLAKHTISS	DRB1*08:04	P14679	Tyrosinase	133	147	45.91
2398	KFFAYLTLAKHTISS	DRB1*10:01	P14679	Tyrosinase	133	147	9.36
2399	KFFAYLTLAKHTISS	DRB1*11:01	P14679	Tyrosinase	133	147	22.47
2400	KFFAYLTLAKHTISS	DRB1*11:04	P14679	Tyrosinase	133	147	27.61
2401	KFFAYLTLAKHTISS	DRB1*11:08	P14679	Tyrosinase	133	147	33.88
2402	KFFAYLTLAKHTISS	DRB1*11:10	P14679	Tyrosinase	133	147	22.47
2403	KFFAYLTLAKHTISS	DRB1*11:12	P14679	Tyrosinase	133	147	22.47
2404	KFFAYLTLAKHTISS	DRB1*11:13	P14679	Tyrosinase	133	147	44.78
2405	KFFAYLTLAKHTISS	DRB1*11:28	P14679	Tyrosinase	133	147	22.47
2406	KFFAYLTLAKHTISS	DRB1*11:29	P14679	Tyrosinase	133	147	22.47
2407	KFFAYLTLAKHTISS	DRB1*11:37	P14679	Tyrosinase	133	147	45.92
2408	KFFAYLTLAKHTISS	DRB1*11:42	P14679	Tyrosinase	133	147	31.66
2409	KFFAYLTLAKHTISS	DRB1*11:46	P14679	Tyrosinase	133	147	27.61
2410	KFFAYLTLAKHTISS	DRB1*11:49	P14679	Tyrosinase	133	147	22.47
2411	KFFAYLTLAKHTISS	DRB1*11:58	P14679	Tyrosinase	133	147	27.61
2412	KFFAYLTLAKHTISS	DRB1*11:62	P14679	Tyrosinase	133	147	22.47
2413	KFFAYLTLAKHTISS	DRB1*11:74	P14679	Tyrosinase	133	147	22.47
2414	KFFAYLTLAKHTISS	DRB1*12:16	P14679	Tyrosinase	133	147	38.94
2415	KFFAYLTLAKHTISS	DRB1*13:05	P14679	Tyrosinase	133	147	22.47
2416	KFFAYLTLAKHTISS	DRB1*13:07	P14679	Tyrosinase	133	147	45.92
2417	KFFAYLTLAKHTISS	DRB1*13:11	P14679	Tyrosinase	133	147	27.61
2418	KFFAYLTLAKHTISS	DRB1*13:14	P14679	Tyrosinase	133	147	22.47
2419	KFFAYLTLAKHTISS	DRB1*13:21	P14679	Tyrosinase	133	147	23.4
2420	KFFAYLTLAKHTISS	DRB1*13:50	P14679	Tyrosinase	133	147	22.47
2421	KFFAYLTLAKHTISS	DRB1*15:15	P14679	Tyrosinase	133	147	42.63
2422	KFFAYLTLAKHTISS	DRB1*16:09	P14679	Tyrosinase	133	147	36.33
2423	KHFLRNQPLTFALQL	DRB1*01:01	P40967	PMEL17	230	244	20.4
2424	KHFLRNQPLTFALQL	DRB1*01:11	P40967	PMEL17	230	244	30.72
2425	KHFLRNQPLTFALQL	DRB1*01:18	P40967	PMEL17	230	244	17.05
2426	KHFLRNQPLTFALQL	DRB1*01:20	P40967	PMEL17	230	244	26.49
2427	KHFLRNQPLTFALQL	DRB1*01:24	P40967	PMEL17	230	244	45.15
2428	KHFLRNQPLTFALQL	DRB1*01:29	P40967	PMEL17	230	244	45.23
2429	KHFLRNQPLTFALQL	DRB1*10:01	P40967	PMEL17	230	244	35.42
2430	KHFLRNQPLTFALQL	DRB1*11:02	P40967	PMEL17	230	244	45.78
2431	KHFLRNQPLTFALQL	DRB1*11:08	P40967	PMEL17	230	244	37
2432	KHFLRNQPLTFALQL	DRB1*11:14	P40967	PMEL17	230	244	9.79
2433	KHFLRNQPLTFALQL	DRB1*11:19	P40967	PMEL17	230	244	46.32
2434	KHFLRNQPLTFALQL	DRB1*11:65	P40967	PMEL17	230	244	45.78
2435	KHFLRNQPLTFALQL	DRB1*13:01	P40967	PMEL17	230	244	45.78
2436	KHFLRNQPLTFALQL	DRB1*13:02	P40967	PMEL17	230	244	9.79

2437	KHFLRNQPLTFALQL	DRB1*13:23	P40967	PMEL17	230	244	9.79
2438	KHFLRNQPLTFALQL	DRB1*13:66	P40967	PMEL17	230	244	32.11
2439	KHFLRNQPLTFALQL	DRB1*13:96	P40967	PMEL17	230	244	12.84
2440	KHFLRNQPLTFALQL	DRB1*13:97	P40967	PMEL17	230	244	9.79
2441	KHFLRNQPLTFALQL	DRB1*14:02	P40967	PMEL17	230	244	26.81
2442	KHFLRNQPLTFALQL	DRB1*14:06	P40967	PMEL17	230	244	48.8
2443	KHTISSDYVIPIGTY	DRB1*03:01	P14679	Tyrosinase	142	156	35.22
2444	KHTISSDYVIPIGTY	DRB1*03:04	P14679	Tyrosinase	142	156	35.22
2445	KHTISSDYVIPIGTY	DRB1*03:11	P14679	Tyrosinase	142	156	20.71
2446	KHTISSDYVIPIGTY	DRB1*03:13	P14679	Tyrosinase	142	156	35.22
2447	KHTISSDYVIPIGTY	DRB1*11:14	P14679	Tyrosinase	142	156	46.16
2448	KHTISSDYVIPIGTY	DRB1*13:02	P14679	Tyrosinase	142	156	46.16
2449	KHTISSDYVIPIGTY	DRB1*13:23	P14679	Tyrosinase	142	156	46.16
2450	KHTISSDYVIPIGTY	DRB1*13:97	P14679	Tyrosinase	142	156	46.16
2451	KIFYVYMKRKYEAMT	DRB1*08:01	Q16385	SSX2	45	59	37.96
2452	KIFYVYMKRKYEAMT	DRB1*08:04	Q16385	SSX2	45	59	43.83
2453	KIFYVYMKRKYEAMT	DRB1*08:24	Q16385	SSX2	45	59	42.23
2454	KIFYVYMKRKYEAMT	DRB1*11:01	Q16385	SSX2	45	59	12.77
2455	KIFYVYMKRKYEAMT	DRB1*11:02	Q16385	SSX2	45	59	26.59
2456	KIFYVYMKRKYEAMT	DRB1*11:03	Q16385	SSX2	45	59	12.89
2457	KIFYVYMKRKYEAMT	DRB1*11:04	Q16385	SSX2	45	59	11.33
2458	KIFYVYMKRKYEAMT	DRB1*11:08	Q16385	SSX2	45	59	32.05
2459	KIFYVYMKRKYEAMT	DRB1*11:10	Q16385	SSX2	45	59	12.77
2460	KIFYVYMKRKYEAMT	DRB1*11:11	Q16385	SSX2	45	59	27.01
2461	KIFYVYMKRKYEAMT	DRB1*11:12	Q16385	SSX2	45	59	12.77
2462	KIFYVYMKRKYEAMT	DRB1*11:13	Q16385	SSX2	45	59	36.57
2463	KIFYVYMKRKYEAMT	DRB1*11:27	Q16385	SSX2	45	59	39.72
2464	KIFYVYMKRKYEAMT	DRB1*11:28	Q16385	SSX2	45	59	12.77
2465	KIFYVYMKRKYEAMT	DRB1*11:29	Q16385	SSX2	45	59	12.77
2466	KIFYVYMKRKYEAMT	DRB1*11:37	Q16385	SSX2	45	59	27.34
2467	KIFYVYMKRKYEAMT	DRB1*11:42	Q16385	SSX2	45	59	18.28
2468	KIFYVYMKRKYEAMT	DRB1*11:46	Q16385	SSX2	45	59	11.33
2469	KIFYVYMKRKYEAMT	DRB1*11:49	Q16385	SSX2	45	59	12.77
2470	KIFYVYMKRKYEAMT	DRB1*11:58	Q16385	SSX2	45	59	11.33
2471	KIFYVYMKRKYEAMT	DRB1*11:62	Q16385	SSX2	45	59	12.77
2472	KIFYVYMKRKYEAMT	DRB1*11:65	Q16385	SSX2	45	59	26.59
2473	KIFYVYMKRKYEAMT	DRB1*11:74	Q16385	SSX2	45	59	12.77
2474	KIFYVYMKRKYEAMT	DRB1*11:84	Q16385	SSX2	45	59	34.03
2475	KIFYVYMKRKYEAMT	DRB1*13:01	Q16385	SSX2	45	59	26.59
2476	KIFYVYMKRKYEAMT	DRB1*13:05	Q16385	SSX2	45	59	12.77

2477	KIFYVYMKRKYEAMT	DRB1*13:07	Q16385	SSX2	45	59	27.34
2478	KIFYVYMKRKYEAMT	DRB1*13:11	Q16385	SSX2	45	59	11.33
2479	KIFYVYMKRKYEAMT	DRB1*13:14	Q16385	SSX2	45	59	12.77
2480	KIFYVYMKRKYEAMT	DRB1*13:21	Q16385	SSX2	45	59	8.9
2481	KIFYVYMKRKYEAMT	DRB1*13:50	Q16385	SSX2	45	59	12.77
2482	KKPPVIRQNIHSLSP	DRB1*11:14	O75767	TRP2	121	135	47.85
2483	KKPPVIRQNIHSLSP	DRB1*13:02	O75767	TRP2	121	135	47.85
2484	KKPPVIRQNIHSLSP	DRB1*13:23	O75767	TRP2	121	135	47.85
2485	KKPPVIRQNIHSLSP	DRB1*13:97	O75767	TRP2	121	135	47.85
2486	KKSELKELINNELSH	DRB1*01:01	P04271	S100	27	41	48.94
2487	KKSELKELINNELSH	DRB1*01:18	P04271	S100	27	41	40.51
2488	KKSELKELINNELSH	DRB1*01:20	P04271	S100	27	41	33.28
2489	KKVADLVGFLLLKYR	DPB1*02:02	P43355	MAGE1	104	118	45.02
2490	KKVADLVGFLLLKYR	DPB1*15:01	P43355	MAGE1	104	118	26.21
2491	KKVADLVGFLLLKYR	DPB1*33:01	P43355	MAGE1	104	118	17.7
2492	KKVADLVGFLLLKYR	DPB1*47:01	P43355	MAGE1	104	118	45.02
2493	KKVADLVGFLLLKYR	DPB1*71:01	P43355	MAGE1	104	118	17.7
2494	KKVADLVGFLLLKYR	DRB1*01:01	P43355	MAGE1	104	118	31.94
2495	KKVADLVGFLLLKYR	DRB1*01:18	P43355	MAGE1	104	118	21.17
2496	KKVADLVGFLLLKYR	DRB1*01:20	P43355	MAGE1	104	118	22.78
2497	KLGFKATLPPFMCNK	DRB1*01:01	Q16385	SSX2	60	74	9.87
2498	KLGFKATLPPFMCNK	DRB1*01:11	Q16385	SSX2	60	74	25.07
2499	KLGFKATLPPFMCNK	DRB1*01:18	Q16385	SSX2	60	74	10.68
2500	KLGFKATLPPFMCNK	DRB1*01:20	Q16385	SSX2	60	74	33.19
2501	KLGFKATLPPFMCNK	DRB1*01:24	Q16385	SSX2	60	74	21.8
2502	KLGFKATLPPFMCNK	DRB1*01:29	Q16385	SSX2	60	74	20.78
2503	KLGFKATLPPFMCNK	DRB1*07:01	Q16385	SSX2	60	74	12.37
2504	KLGFKATLPPFMCNK	DRB1*09:01	Q16385	SSX2	60	74	19.03
2505	KLGFKATLPPFMCNK	DRB1*10:01	Q16385	SSX2	60	74	9.17
2506	KLGFKATLPPFMCNK	DRB1*16:02	Q16385	SSX2	60	74	41.89
2507	KLLSLGCIFFPLLLF	DPB1*33:01	P17643	TRP1	5	19	45.93
2508	KLLSLGCIFFPLLLF	DPB1*71:01	P17643	TRP1	5	19	45.93
2509	KNHFVRALDMAKRTT	DRB1*01:01	P17643	TRP1	141	155	48.31
2510	KNHFVRALDMAKRTT	DRB1*01:18	P17643	TRP1	141	155	33.4
2511	KNHFVRALDMAKRTT	DRB1*01:20	P17643	TRP1	141	155	35.49
2512	KNHFVRALDMAKRTT	DRB1*10:01	P17643	TRP1	141	155	47.87
2513	KNHFVRALDMAKRTT	DRB1*11:42	P17643	TRP1	141	155	44.18
2514	KNYKRCFPVIFGKAS	DRB1*01:01	P43358	MAGE4	142	156	49.2
2515	KNYKRCFPVIFGKAS	DRB1*01:18	P43358	MAGE4	142	156	40.27
2516	KPPVIRQNIHSLSPQ	DRB1*11:14	O75767	TRP2	122	136	47.66

2517	KPPVIRQNIHSLSPQ	DRB1*13:02	O75767	TRP2	122	136	47.66
2518	KPPVIRQNIHSLSPQ	DRB1*13:23	O75767	TRP2	122	136	47.66
2519	KPPVIRQNIHSLSPQ	DRB1*13:97	O75767	TRP2	122	136	47.66
2520	KRCLLHLAVIGALLA	DRB1*01:01	P40967	PMEL17	6	20	28.1
2521	KRCLLHLAVIGALLA	DRB1*01:18	P40967	PMEL17	6	20	18.73
2522	KRCLLHLAVIGALLA	DRB1*01:20	P40967	PMEL17	6	20	27.86
2523	KRCLLHLAVIGALLA	DRB1*01:24	P40967	PMEL17	6	20	29.79
2524	KRCLLHLAVIGALLA	DRB1*01:29	P40967	PMEL17	6	20	37.86
2525	KRKYEAMTKLGFKAT	DRB1*11:01	Q16385	SSX2	52	66	47.42
2526	KRKYEAMTKLGFKAT	DRB1*11:10	Q16385	SSX2	52	66	47.42
2527	KRKYEAMTKLGFKAT	DRB1*11:12	Q16385	SSX2	52	66	47.42
2528	KRKYEAMTKLGFKAT	DRB1*11:28	Q16385	SSX2	52	66	47.42
2529	KRKYEAMTKLGFKAT	DRB1*11:29	Q16385	SSX2	52	66	47.42
2530	KRKYEAMTKLGFKAT	DRB1*11:49	Q16385	SSX2	52	66	47.42
2531	KRKYEAMTKLGFKAT	DRB1*11:62	Q16385	SSX2	52	66	47.42
2532	KRKYEAMTKLGFKAT	DRB1*11:74	Q16385	SSX2	52	66	47.42
2533	KRKYEAMTKLGFKAT	DRB1*13:05	Q16385	SSX2	52	66	47.42
2534	KRKYEAMTKLGFKAT	DRB1*13:14	Q16385	SSX2	52	66	47.42
2535	KRKYEAMTKLGFKAT	DRB1*13:21	Q16385	SSX2	52	66	40.41
2536	KRKYEAMTKLGFKAT	DRB1*13:50	Q16385	SSX2	52	66	47.42
2537	KRSFVYVWKTWGQYW	DRB1*11:01	P40967	PMEL17	146	160	17.94
2538	KRSFVYVWKTWGQYW	DRB1*11:04	P40967	PMEL17	146	160	37.07
2539	KRSFVYVWKTWGQYW	DRB1*11:08	P40967	PMEL17	146	160	39.62
2540	KRSFVYVWKTWGQYW	DRB1*11:10	P40967	PMEL17	146	160	17.94
2541	KRSFVYVWKTWGQYW	DRB1*11:12	P40967	PMEL17	146	160	17.94
2542	KRSFVYVWKTWGQYW	DRB1*11:28	P40967	PMEL17	146	160	17.94
2543	KRSFVYVWKTWGQYW	DRB1*11:29	P40967	PMEL17	146	160	17.94
2544	KRSFVYVWKTWGQYW	DRB1*11:37	P40967	PMEL17	146	160	39.23
2545	KRSFVYVWKTWGQYW	DRB1*11:42	P40967	PMEL17	146	160	45.58
2546	KRSFVYVWKTWGQYW	DRB1*11:46	P40967	PMEL17	146	160	37.07
2547	KRSFVYVWKTWGQYW	DRB1*11:49	P40967	PMEL17	146	160	17.94
2548	KRSFVYVWKTWGQYW	DRB1*11:58	P40967	PMEL17	146	160	37.07
2549	KRSFVYVWKTWGQYW	DRB1*11:62	P40967	PMEL17	146	160	17.94
2550	KRSFVYVWKTWGQYW	DRB1*11:74	P40967	PMEL17	146	160	17.94
2551	KRSFVYVWKTWGQYW	DRB1*13:05	P40967	PMEL17	146	160	17.94
2552	KRSFVYVWKTWGQYW	DRB1*13:07	P40967	PMEL17	146	160	39.23
2553	KRSFVYVWKTWGQYW	DRB1*13:11	P40967	PMEL17	146	160	37.07
2554	KRSFVYVWKTWGQYW	DRB1*13:14	P40967	PMEL17	146	160	17.94
2555	KRSFVYVWKTWGQYW	DRB1*13:21	P40967	PMEL17	146	160	32.66
2556	KRSFVYVWKTWGQYW	DRB1*13:50	P40967	PMEL17	146	160	17.94

2557	KRTTHPLFVIATRRS	DRB1*01:18	P17643	TRP1	152	166	43.94
2558	KRTTHPLFVIATRRS	DRB1*01:20	P17643	TRP1	152	166	33.95
2559	KRTTHPLFVIATRRS	DRB1*04:04	P17643	TRP1	152	166	45.16
2560	KRTTHPLFVIATRRS	DRB1*10:01	P17643	TRP1	152	166	49.85
2561	KRTTHPLFVIATRRS	DRB1*11:04	P17643	TRP1	152	166	42.57
2562	KRTTHPLFVIATRRS	DRB1*11:42	P17643	TRP1	152	166	43.05
2563	KRTTHPLFVIATRRS	DRB1*11:46	P17643	TRP1	152	166	42.57
2564	KRTTHPLFVIATRRS	DRB1*11:58	P17643	TRP1	152	166	42.57
2565	KRTTHPLFVIATRRS	DRB1*13:11	P17643	TRP1	152	166	42.57
2566	KSELKELINNELSHF	DRB1*01:01	P04271	S100	28	42	33.6
2567	KSELKELINNELSHF	DRB1*01:18	P04271	S100	28	42	27.66
2568	KSELKELINNELSHF	DRB1*01:20	P04271	S100	28	42	23.27
2569	KSYLEQASRIWSWLL	DRB1*01:01	P14679	Tyrosinase	465	479	48.71
2570	KSYLEQASRIWSWLL	DRB1*01:18	P14679	Tyrosinase	465	479	39.46
2571	KSYLEQASRIWSWLL	DRB1*11:04	P14679	Tyrosinase	465	479	48.45
2572	KSYLEQASRIWSWLL	DRB1*11:42	P14679	Tyrosinase	465	479	49.02
2573	KSYLEQASRIWSWLL	DRB1*11:46	P14679	Tyrosinase	465	479	48.45
2574	KSYLEQASRIWSWLL	DRB1*11:58	P14679	Tyrosinase	465	479	48.45
2575	KSYLEQASRIWSWLL	DRB1*13:11	P14679	Tyrosinase	465	479	48.45
2576	KTWGQYWQVLGGPVS	DRB1*01:01	P40967	PMEL17	154	168	15.76
2577	KTWGQYWQVLGGPVS	DRB1*01:18	P40967	PMEL17	154	168	17.05
2578	KTWGQYWQVLGGPVS	DRB1*01:24	P40967	PMEL17	154	168	49.99
2579	KTWGQYWQVLGGPVS	DRB1*01:29	P40967	PMEL17	154	168	39.78
2580	KTWGQYWQVLGGPVS	DRB1*07:01	P40967	PMEL17	154	168	42.41
2581	KTWGQYWQVLGGPVS	DRB1*10:01	P40967	PMEL17	154	168	40.32
2582	KVADLVGFLLLKYRA	DPB1*15:01	P43355	MAGE1	105	119	31.97
2583	KVADLVGFLLLKYRA	DPB1*33:01	P43355	MAGE1	105	119	22.96
2584	KVADLVGFLLLKYRA	DPB1*71:01	P43355	MAGE1	105	119	22.96
2585	KVADLVGFLLLKYRA	DRB1*01:18	P43355	MAGE1	105	119	49.58
2586	KVADLVGFLLLKYRA	DRB1*01:20	P43355	MAGE1	105	119	46.74
2587	KVDELAHFLLRKYRA	DRB1*11:03	P43358	MAGE4	113	127	40.71
2588	KVDELAHFLLRKYRA	DRB1*11:04	P43358	MAGE4	113	127	45.38
2589	KVDELAHFLLRKYRA	DRB1*11:42	P43358	MAGE4	113	127	43.21
2590	KVDELAHFLLRKYRA	DRB1*11:46	P43358	MAGE4	113	127	45.38
2591	KVDELAHFLLRKYRA	DRB1*11:58	P43358	MAGE4	113	127	45.38
2592	KVDELAHFLLRKYRA	DRB1*13:11	P43358	MAGE4	113	127	45.38
2593	KVLEHVVRVNARVRI	DRB1*01:20	P43358	MAGE4	286	300	43.27
2594	KVLEHVVRVNARVRI	DRB1*03:11	P43358	MAGE4	286	300	40.02
2595	KVLEHVVRVNARVRI	DRB1*08:04	P43358	MAGE4	286	300	34.75
2596	KVLEHVVRVNARVRI	DRB1*11:01	P43358	MAGE4	286	300	31.5

2597	KVLEHVVRVNARVRI	DRB1*11:02	P43358	MAGE4	286	300	14.8
2598	KVLEHVVRVNARVRI	DRB1*11:03	P43358	MAGE4	286	300	19.62
2599	KVLEHVVRVNARVRI	DRB1*11:04	P43358	MAGE4	286	300	14.3
2600	KVLEHVVRVNARVRI	DRB1*11:08	P43358	MAGE4	286	300	46.09
2601	KVLEHVVRVNARVRI	DRB1*11:10	P43358	MAGE4	286	300	31.5
2602	KVLEHVVRVNARVRI	DRB1*11:12	P43358	MAGE4	286	300	31.5
2603	KVLEHVVRVNARVRI	DRB1*11:13	P43358	MAGE4	286	300	18.27
2604	KVLEHVVRVNARVRI	DRB1*11:14	P43358	MAGE4	286	300	26.57
2605	KVLEHVVRVNARVRI	DRB1*11:28	P43358	MAGE4	286	300	31.5
2606	KVLEHVVRVNARVRI	DRB1*11:29	P43358	MAGE4	286	300	31.5
2607	KVLEHVVRVNARVRI	DRB1*11:42	P43358	MAGE4	286	300	13.44
2608	KVLEHVVRVNARVRI	DRB1*11:46	P43358	MAGE4	286	300	14.3
2609	KVLEHVVRVNARVRI	DRB1*11:49	P43358	MAGE4	286	300	31.5
2610	KVLEHVVRVNARVRI	DRB1*11:58	P43358	MAGE4	286	300	14.3
2611	KVLEHVVRVNARVRI	DRB1*11:62	P43358	MAGE4	286	300	31.5
2612	KVLEHVVRVNARVRI	DRB1*11:65	P43358	MAGE4	286	300	14.8
2613	KVLEHVVRVNARVRI	DRB1*11:74	P43358	MAGE4	286	300	31.5
2614	KVLEHVVRVNARVRI	DRB1*11:84	P43358	MAGE4	286	300	36.07
2615	KVLEHVVRVNARVRI	DRB1*13:01	P43358	MAGE4	286	300	14.8
2616	KVLEHVVRVNARVRI	DRB1*13:02	P43358	MAGE4	286	300	26.57
2617	KVLEHVVRVNARVRI	DRB1*13:05	P43358	MAGE4	286	300	31.5
2618	KVLEHVVRVNARVRI	DRB1*13:11	P43358	MAGE4	286	300	14.3
2619	KVLEHVVRVNARVRI	DRB1*13:14	P43358	MAGE4	286	300	31.5
2620	KVLEHVVRVNARVRI	DRB1*13:23	P43358	MAGE4	286	300	26.57
2621	KVLEHVVRVNARVRI	DRB1*13:50	P43358	MAGE4	286	300	31.5
2622	KVLEHVVRVNARVRI	DRB1*13:61	P43358	MAGE4	286	300	30.33
2623	KVLEHVVRVNARVRI	DRB1*13:96	P43358	MAGE4	286	300	39.49
2624	KVLEHVVRVNARVRI	DRB1*13:97	P43358	MAGE4	286	300	26.57
2625	KVLEHVVRVNARVRI	DRB1*14:06	P43358	MAGE4	286	300	27.88
2626	KVLEHVVRVNARVRI	DRB1*14:12	P43358	MAGE4	286	300	46.98
2627	KVLEHVVRVNARVRI	DRB1*14:32	P43358	MAGE4	286	300	26.76
2628	KVLEYVIKVSARVRF	DRB1*01:01	P43355	MAGE1	278	292	11.89
2629	KVLEYVIKVSARVRF	DRB1*01:11	P43355	MAGE1	278	292	20.44
2630	KVLEYVIKVSARVRF	DRB1*01:18	P43355	MAGE1	278	292	8.36
2631	KVLEYVIKVSARVRF	DRB1*01:20	P43355	MAGE1	278	292	21.27
2632	KVLEYVIKVSARVRF	DRB1*01:24	P43355	MAGE1	278	292	21.68
2633	KVLEYVIKVSARVRF	DRB1*01:29	P43355	MAGE1	278	292	17.06
2634	KVLEYVIKVSARVRF	DRB1*03:11	P43355	MAGE1	278	292	29.59
2635	KVLEYVIKVSARVRF	DRB1*03:15	P43355	MAGE1	278	292	44.51
2636	KVLEYVIKVSARVRF	DRB1*07:01	P43355	MAGE1	278	292	38.8

2637	KVLEYVIKVSARVRF	DRB1*08:02	P43355	MAGE1	278	292	44.67
2638	KVLEYVIKVSARVRF	DRB1*08:04	P43355	MAGE1	278	292	28.78
2639	KVLEYVIKVSARVRF	DRB1*08:30	P43355	MAGE1	278	292	44.36
2640	KVLEYVIKVSARVRF	DRB1*10:01	P43355	MAGE1	278	292	40.75
2641	KVLEYVIKVSARVRF	DRB1*11:01	P43355	MAGE1	278	292	25.6
2642	KVLEYVIKVSARVRF	DRB1*11:02	P43355	MAGE1	278	292	12.95
2643	KVLEYVIKVSARVRF	DRB1*11:03	P43355	MAGE1	278	292	19.93
2644	KVLEYVIKVSARVRF	DRB1*11:04	P43355	MAGE1	278	292	14.12
2645	KVLEYVIKVSARVRF	DRB1*11:08	P43355	MAGE1	278	292	25.12
2646	KVLEYVIKVSARVRF	DRB1*11:10	P43355	MAGE1	278	292	25.6
2647	KVLEYVIKVSARVRF	DRB1*11:12	P43355	MAGE1	278	292	25.6
2648	KVLEYVIKVSARVRF	DRB1*11:13	P43355	MAGE1	278	292	12.69
2649	KVLEYVIKVSARVRF	DRB1*11:14	P43355	MAGE1	278	292	29.26
2650	KVLEYVIKVSARVRF	DRB1*11:19	P43355	MAGE1	278	292	40.05
2651	KVLEYVIKVSARVRF	DRB1*11:28	P43355	MAGE1	278	292	25.6
2652	KVLEYVIKVSARVRF	DRB1*11:29	P43355	MAGE1	278	292	25.6
2653	KVLEYVIKVSARVRF	DRB1*11:42	P43355	MAGE1	278	292	10.88
2654	KVLEYVIKVSARVRF	DRB1*11:46	P43355	MAGE1	278	292	14.12
2655	KVLEYVIKVSARVRF	DRB1*11:49	P43355	MAGE1	278	292	25.6
2656	KVLEYVIKVSARVRF	DRB1*11:58	P43355	MAGE1	278	292	14.12
2657	KVLEYVIKVSARVRF	DRB1*11:62	P43355	MAGE1	278	292	25.6
2658	KVLEYVIKVSARVRF	DRB1*11:65	P43355	MAGE1	278	292	12.95
2659	KVLEYVIKVSARVRF	DRB1*11:74	P43355	MAGE1	278	292	25.6
2660	KVLEYVIKVSARVRF	DRB1*11:84	P43355	MAGE1	278	292	27.07
2661	KVLEYVIKVSARVRF	DRB1*12:03	P43355	MAGE1	278	292	46.87
2662	KVLEYVIKVSARVRF	DRB1*13:01	P43355	MAGE1	278	292	12.95
2663	KVLEYVIKVSARVRF	DRB1*13:02	P43355	MAGE1	278	292	29.26
2664	KVLEYVIKVSARVRF	DRB1*13:05	P43355	MAGE1	278	292	25.6
2665	KVLEYVIKVSARVRF	DRB1*13:11	P43355	MAGE1	278	292	14.12
2666	KVLEYVIKVSARVRF	DRB1*13:14	P43355	MAGE1	278	292	25.6
2667	KVLEYVIKVSARVRF	DRB1*13:21	P43355	MAGE1	278	292	37
2668	KVLEYVIKVSARVRF	DRB1*13:23	P43355	MAGE1	278	292	29.26
2669	KVLEYVIKVSARVRF	DRB1*13:50	P43355	MAGE1	278	292	25.6
2670	KVLEYVIKVSARVRF	DRB1*13:61	P43355	MAGE1	278	292	28.47
2671	KVLEYVIKVSARVRF	DRB1*13:96	P43355	MAGE1	278	292	41.64
2672	KVLEYVIKVSARVRF	DRB1*13:97	P43355	MAGE1	278	292	29.26
2673	KVLEYVIKVSARVRF	DRB1*14:01	P43355	MAGE1	278	292	23.15
2674	KVLEYVIKVSARVRF	DRB1*14:04	P43355	MAGE1	278	292	42.1
2675	KVLEYVIKVSARVRF	DRB1*14:05	P43355	MAGE1	278	292	48.78
2676	KVLEYVIKVSARVRF	DRB1*14:06	P43355	MAGE1	278	292	25.72

2677	KVLEYVIKVSARVRF	DRB1*14:12	P43355	MAGE1	278	292	35.2
2678	KVLEYVIKVSARVRF	DRB1*14:23	P43355	MAGE1	278	292	48.78
2679	KVLEYVIKVSARVRF	DRB1*14:32	P43355	MAGE1	278	292	16.02
2680	KVLEYVIKVSARVRF	DRB1*14:38	P43355	MAGE1	278	292	46.26
2681	KVLEYVIKVSARVRF	DRB1*14:54	P43355	MAGE1	278	292	23.15
2682	KVLEYVIKVSARVRF	DRB1*15:15	P43355	MAGE1	278	292	43.72
2683	KVLEYVIKVSARVRF	DRB1*16:02	P43355	MAGE1	278	292	37.55
2684	KVLEYVIKVSARVRF	DRB1*16:09	P43355	MAGE1	278	292	35.31
2685	KVSARVRFFFPSLRE	DPB1*33:01	P43355	MAGE1	285	299	40.46
2686	KVSARVRFFFPSLRE	DPB1*71:01	P43355	MAGE1	285	299	40.46
2687	KVYYYRFVIGLRVWQ	DPB1*33:01	O75767	TRP2	206	220	45.12
2688	KVYYYRFVIGLRVWQ	DPB1*71:01	O75767	TRP2	206	220	45.12
2689	KVYYYRFVIGLRVWQ	DRB1*01:01	O75767	TRP2	206	220	6.91
2690	KVYYYRFVIGLRVWQ	DRB1*01:11	O75767	TRP2	206	220	9.65
2691	KVYYYRFVIGLRVWQ	DRB1*01:18	O75767	TRP2	206	220	5.31
2692	KVYYYRFVIGLRVWQ	DRB1*01:20	O75767	TRP2	206	220	25.87
2693	KVYYYRFVIGLRVWQ	DRB1*01:24	O75767	TRP2	206	220	6.44
2694	KVYYYRFVIGLRVWQ	DRB1*01:29	O75767	TRP2	206	220	9.64
2695	KVYYYRFVIGLRVWQ	DRB1*07:01	O75767	TRP2	206	220	12.59
2696	KVYYYRFVIGLRVWQ	DRB1*10:01	O75767	TRP2	206	220	18.44
2697	KVYYYRFVIGLRVWQ	DRB1*11:08	O75767	TRP2	206	220	48.02
2698	KVYYYRFVIGLRVWQ	DRB1*15:02	O75767	TRP2	206	220	24.09
2699	KVYYYRFVIGLRVWQ	DRB1*15:15	O75767	TRP2	206	220	30.51
2700	KVYYYRFVIGLRVWQ	DRB1*16:01	O75767	TRP2	206	220	29.62
2701	KVYYYRFVIGLRVWQ	DRB1*16:02	O75767	TRP2	206	220	22.86
2702	KVYYYRFVIGLRVWQ	DRB1*16:04	O75767	TRP2	206	220	37.18
2703	KVYYYRFVIGLRVWQ	DRB1*16:05	O75767	TRP2	206	220	22.24
2704	KVYYYRFVIGLRVWQ	DRB1*16:09	O75767	TRP2	206	220	19.39
2705	LAETSYVKVLEHVVR	DRB1*01:18	P43358	MAGE4	279	293	44.62
2706	LAETSYVKVLEYVIK	DRB1*01:18	P43355	MAGE1	271	285	43.02
2707	LAGLVSLLCRHKRKQ	DRB1*11:02	P14679	Tyrosinase	492	506	26.28
2708	LAGLVSLLCRHKRKQ	DRB1*11:03	P14679	Tyrosinase	492	506	16.35
2709	LAGLVSLLCRHKRKQ	DRB1*11:04	P14679	Tyrosinase	492	506	24.86
2710	LAGLVSLLCRHKRKQ	DRB1*11:42	P14679	Tyrosinase	492	506	24.46
2711	LAGLVSLLCRHKRKQ	DRB1*11:46	P14679	Tyrosinase	492	506	24.86
2712	LAGLVSLLCRHKRKQ	DRB1*11:58	P14679	Tyrosinase	492	506	24.86
2713	LAGLVSLLCRHKRKQ	DRB1*11:65	P14679	Tyrosinase	492	506	26.28
2714	LAGLVSLLCRHKRKQ	DRB1*13:01	P14679	Tyrosinase	492	506	26.28
2715	LAGLVSLLCRHKRKQ	DRB1*13:11	P14679	Tyrosinase	492	506	24.86
2716	LAHFLLRKYRAKELV	DRB1*01:18	P43358	MAGE4	117	131	47.55

2717	LAHFLLRKYRAKELV	DRB1*01:20	P43358	MAGE4	117	131	41.54
2718	LAHFLLRKYRAKELV	DRB1*11:01	P43358	MAGE4	117	131	29.7
2719	LAHFLLRKYRAKELV	DRB1*11:02	P43358	MAGE4	117	131	31.45
2720	LAHFLLRKYRAKELV	DRB1*11:03	P43358	MAGE4	117	131	18.47
2721	LAHFLLRKYRAKELV	DRB1*11:04	P43358	MAGE4	117	131	21.15
2722	LAHFLLRKYRAKELV	DRB1*11:10	P43358	MAGE4	117	131	29.7
2723	LAHFLLRKYRAKELV	DRB1*11:11	P43358	MAGE4	117	131	47.23
2724	LAHFLLRKYRAKELV	DRB1*11:12	P43358	MAGE4	117	131	29.7
2725	LAHFLLRKYRAKELV	DRB1*11:28	P43358	MAGE4	117	131	29.7
2726	LAHFLLRKYRAKELV	DRB1*11:29	P43358	MAGE4	117	131	29.7
2727	LAHFLLRKYRAKELV	DRB1*11:42	P43358	MAGE4	117	131	27.04
2728	LAHFLLRKYRAKELV	DRB1*11:46	P43358	MAGE4	117	131	21.15
2729	LAHFLLRKYRAKELV	DRB1*11:49	P43358	MAGE4	117	131	29.7
2730	LAHFLLRKYRAKELV	DRB1*11:58	P43358	MAGE4	117	131	21.15
2731	LAHFLLRKYRAKELV	DRB1*11:62	P43358	MAGE4	117	131	29.7
2732	LAHFLLRKYRAKELV	DRB1*11:65	P43358	MAGE4	117	131	31.45
2733	LAHFLLRKYRAKELV	DRB1*11:74	P43358	MAGE4	117	131	29.7
2734	LAHFLLRKYRAKELV	DRB1*13:01	P43358	MAGE4	117	131	31.45
2735	LAHFLLRKYRAKELV	DRB1*13:05	P43358	MAGE4	117	131	29.7
2736	LAHFLLRKYRAKELV	DRB1*13:11	P43358	MAGE4	117	131	21.15
2737	LAHFLLRKYRAKELV	DRB1*13:14	P43358	MAGE4	117	131	29.7
2738	LAHFLLRKYRAKELV	DRB1*13:21	P43358	MAGE4	117	131	21.81
2739	LAHFLLRKYRAKELV	DRB1*13:50	P43358	MAGE4	117	131	29.7
2740	LAHFLLRKYRAKELV	DRB1*15:01	P43358	MAGE4	117	131	15.4
2741	LAHFLLRKYRAKELV	DRB1*15:03	P43358	MAGE4	117	131	23.76
2742	LAHFLLRKYRAKELV	DRB1*15:06	P43358	MAGE4	117	131	15.4
2743	LAHFLLRKYRAKELV	DRB1*15:07	P43358	MAGE4	117	131	33.39
2744	LAHFLLRKYRAKELV	DRB1*15:15	P43358	MAGE4	117	131	30.5
2745	LAHFLLRKYRAKELV	DRB1*15:37	P43358	MAGE4	117	131	44.07
2746	LAHLFLNGTGGQTHL	DRB1*01:01	P17643	TRP1	379	393	48.75
2747	LAKHTISSDYVIPIG	DRB1*03:11	P14679	Tyrosinase	140	154	28.63
2748	LALSAQLLQARLMKE	DRB1*01:01	Q13072	BAGE	8	22	17.77
2749	LALSAQLLQARLMKE	DRB1*01:02	Q13072	BAGE	8	22	47.21
2750	LALSAQLLQARLMKE	DRB1*01:18	Q13072	BAGE	8	22	15.91
2751	LALSAQLLQARLMKE	DRB1*01:20	Q13072	BAGE	8	22	13.14
2752	LALSAQLLQARLMKE	DRB1*01:29	Q13072	BAGE	8	22	37.6
2753	LASLIYRRRLMKQDF	DRB1*11:02	P40967	PMEL17	611	625	21.1
2754	LASLIYRRRLMKQDF	DRB1*11:03	P40967	PMEL17	611	625	13.82
2755	LASLIYRRRLMKQDF	DRB1*11:04	P40967	PMEL17	611	625	26.62
2756	LASLIYRRRLMKQDF	DRB1*11:42	P40967	PMEL17	611	625	28.95

2757	LASLIYRRRLMKQDF	DRB1*11:46	P40967	PMEL17	611	625	26.62
2758	LASLIYRRRLMKQDF	DRB1*11:58	P40967	PMEL17	611	625	26.62
2759	LASLIYRRRLMKQDF	DRB1*11:65	P40967	PMEL17	611	625	21.1
2760	LASLIYRRRLMKQDF	DRB1*13:01	P40967	PMEL17	611	625	21.1
2761	LASLIYRRRLMKQDF	DRB1*13:11	P40967	PMEL17	611	625	26.62
2762	LAVIGALLAVGATKV	DRB1*01:01	P40967	PMEL17	12	26	9.62
2763	LAVIGALLAVGATKV	DRB1*01:02	P40967	PMEL17	12	26	25.58
2764	LAVIGALLAVGATKV	DRB1*01:11	P40967	PMEL17	12	26	25.64
2765	LAVIGALLAVGATKV	DRB1*01:18	P40967	PMEL17	12	26	9.12
2766	LAVIGALLAVGATKV	DRB1*01:20	P40967	PMEL17	12	26	8.62
2767	LAVIGALLAVGATKV	DRB1*01:24	P40967	PMEL17	12	26	16.05
2768	LAVIGALLAVGATKV	DRB1*01:29	P40967	PMEL17	12	26	16.62
2769	LAVIGALLAVGATKV	DRB1*07:01	P40967	PMEL17	12	26	20.25
2770	LAVIGALLAVGATKV	DRB1*10:01	P40967	PMEL17	12	26	33.21
2771	LAVLYCLLWSFQTSA	DRB1*01:01	P14679	Tyrosinase	3	17	46.23
2772	LAVLYCLLWSFQTSA	DRB1*01:18	P14679	Tyrosinase	3	17	37.78
2773	LDCVLYRYGSFSVTL	DPB1*33:01	P40967	PMEL17	473	487	36.17
2774	LDCVLYRYGSFSVTL	DPB1*71:01	P40967	PMEL17	473	487	36.17
2775	LDCVLYRYGSFSVTL	DRB1*15:01	P40967	PMEL17	473	487	24.33
2776	LDCVLYRYGSFSVTL	DRB1*15:06	P40967	PMEL17	473	487	24.33
2777	LDGTATLRLVKRQVP	DRB1*11:02	P40967	PMEL17	458	472	45.35
2778	LDGTATLRLVKRQVP	DRB1*11:03	P40967	PMEL17	458	472	35.67
2779	LDGTATLRLVKRQVP	DRB1*11:04	P40967	PMEL17	458	472	22.43
2780	LDGTATLRLVKRQVP	DRB1*11:13	P40967	PMEL17	458	472	35.45
2781	LDGTATLRLVKRQVP	DRB1*11:42	P40967	PMEL17	458	472	19.07
2782	LDGTATLRLVKRQVP	DRB1*11:46	P40967	PMEL17	458	472	22.43
2783	LDGTATLRLVKRQVP	DRB1*11:58	P40967	PMEL17	458	472	22.43
2784	LDGTATLRLVKRQVP	DRB1*11:65	P40967	PMEL17	458	472	45.35
2785	LDGTATLRLVKRQVP	DRB1*13:01	P40967	PMEL17	458	472	45.35
2786	LDGTATLRLVKRQVP	DRB1*13:11	P40967	PMEL17	458	472	22.43
2787	LDGTATLRLVKRQVP	DRB1*13:21	P40967	PMEL17	458	472	39.45
2788	LEFYLAMPFATPMEA	DPB1*33:01	P78358	NY-ESO-1	88	102	23.29
2789	LEFYLAMPFATPMEA	DPB1*71:01	P78358	NY-ESO-1	88	102	23.29
2790	LEFYLAMPFATPMEA	DRB1*01:01	P78358	NY-ESO-1	88	102	7.29
2791	LEFYLAMPFATPMEA	DRB1*01:11	P78358	NY-ESO-1	88	102	16.85
2792	LEFYLAMPFATPMEA	DRB1*01:18	P78358	NY-ESO-1	88	102	7.42
2793	LEFYLAMPFATPMEA	DRB1*01:20	P78358	NY-ESO-1	88	102	19.88
2794	LEFYLAMPFATPMEA	DRB1*01:24	P78358	NY-ESO-1	88	102	11.19
2795	LEFYLAMPFATPMEA	DRB1*01:29	P78358	NY-ESO-1	88	102	12.03
2796	LEFYLAMPFATPMEA	DRB1*09:01	P78358	NY-ESO-1	88	102	25.81

2797	LEFYLAMPFATPMEA	DRB1*10:01	P78358	NY-ESO-1	88	102	8
2798	LEHVVRVNARVRIAY	DRB1*01:18	P43358	MAGE4	288	302	42.69
2799	LEHVVRVNARVRIAY	DRB1*01:20	P43358	MAGE4	288	302	32.28
2800	LEHVVRVNARVRIAY	DRB1*03:11	P43358	MAGE4	288	302	24.86
2801	LEHVVRVNARVRIAY	DRB1*08:04	P43358	MAGE4	288	302	47.93
2802	LEHVVRVNARVRIAY	DRB1*11:02	P43358	MAGE4	288	302	9.7
2803	LEHVVRVNARVRIAY	DRB1*11:03	P43358	MAGE4	288	302	13.7
2804	LEHVVRVNARVRIAY	DRB1*11:04	P43358	MAGE4	288	302	26.61
2805	LEHVVRVNARVRIAY	DRB1*11:08	P43358	MAGE4	288	302	45.51
2806	LEHVVRVNARVRIAY	DRB1*11:11	P43358	MAGE4	288	302	42.96
2807	LEHVVRVNARVRIAY	DRB1*11:13	P43358	MAGE4	288	302	14.21
2808	LEHVVRVNARVRIAY	DRB1*11:14	P43358	MAGE4	288	302	17.31
2809	LEHVVRVNARVRIAY	DRB1*11:42	P43358	MAGE4	288	302	12.35
2810	LEHVVRVNARVRIAY	DRB1*11:46	P43358	MAGE4	288	302	26.61
2811	LEHVVRVNARVRIAY	DRB1*11:58	P43358	MAGE4	288	302	26.61
2812	LEHVVRVNARVRIAY	DRB1*11:65	P43358	MAGE4	288	302	9.7
2813	LEHVVRVNARVRIAY	DRB1*11:84	P43358	MAGE4	288	302	43.4
2814	LEHVVRVNARVRIAY	DRB1*13:01	P43358	MAGE4	288	302	9.7
2815	LEHVVRVNARVRIAY	DRB1*13:02	P43358	MAGE4	288	302	17.31
2816	LEHVVRVNARVRIAY	DRB1*13:11	P43358	MAGE4	288	302	26.61
2817	LEHVVRVNARVRIAY	DRB1*13:23	P43358	MAGE4	288	302	17.31
2818	LEHVVRVNARVRIAY	DRB1*13:61	P43358	MAGE4	288	302	15.71
2819	LEHVVRVNARVRIAY	DRB1*13:96	P43358	MAGE4	288	302	23.21
2820	LEHVVRVNARVRIAY	DRB1*13:97	P43358	MAGE4	288	302	17.31
2821	LEHVVRVNARVRIAY	DRB1*14:06	P43358	MAGE4	288	302	22.55
2822	LEHVVRVNARVRIAY	DRB1*14:12	P43358	MAGE4	288	302	36.53
2823	LEHVVRVNARVRIAY	DRB1*14:32	P43358	MAGE4	288	302	19.69
2824	LEKAMVALIDVFHQY	DRB1*01:20	P04271	S100	2	16	48.67
2825	LEKAMVALIDVFHQY	DRB1*04:04	P04271	S100	2	16	35.24
2826	LEKAMVALIDVFHQY	DRB1*04:10	P04271	S100	2	16	42.14
2827	LEKAMVALIDVFHQY	DRB1*10:01	P04271	S100	2	16	46.06
2828	LERVIKNYKRCFPVI	DRB1*11:02	P43358	MAGE4	137	151	30.05
2829	LERVIKNYKRCFPVI	DRB1*11:03	P43358	MAGE4	137	151	26.62
2830	LERVIKNYKRCFPVI	DRB1*11:42	P43358	MAGE4	137	151	36.49
2831	LERVIKNYKRCFPVI	DRB1*11:65	P43358	MAGE4	137	151	30.05
2832	LERVIKNYKRCFPVI	DRB1*13:01	P43358	MAGE4	137	151	30.05
2833	LESLFRAVITKKVAD	DRB1*01:01	P43355	MAGE1	94	108	7.2
2834	LESLFRAVITKKVAD	DRB1*01:11	P43355	MAGE1	94	108	18.57
2835	LESLFRAVITKKVAD	DRB1*01:18	P43355	MAGE1	94	108	6.74
2836	LESLFRAVITKKVAD	DRB1*01:20	P43355	MAGE1	94	108	19.18

2837	LESLFRAVITKKVAD	DRB1*01:24	P43355	MAGE1	94	108	16.11
2838	LESLFRAVITKKVAD	DRB1*01:29	P43355	MAGE1	94	108	11.81
2839	LESLFRAVITKKVAD	DRB1*07:01	P43355	MAGE1	94	108	9.95
2840	LESLFRAVITKKVAD	DRB1*09:01	P43355	MAGE1	94	108	24.59
2841	LESLFRAVITKKVAD	DRB1*10:01	P43355	MAGE1	94	108	16.11
2842	LESLFRAVITKKVAD	DRB1*11:01	P43355	MAGE1	94	108	37.28
2843	LESLFRAVITKKVAD	DRB1*11:04	P43355	MAGE1	94	108	49.59
2844	LESLFRAVITKKVAD	DRB1*11:08	P43355	MAGE1	94	108	38.14
2845	LESLFRAVITKKVAD	DRB1*11:10	P43355	MAGE1	94	108	37.28
2846	LESLFRAVITKKVAD	DRB1*11:12	P43355	MAGE1	94	108	37.28
2847	LESLFRAVITKKVAD	DRB1*11:28	P43355	MAGE1	94	108	37.28
2848	LESLFRAVITKKVAD	DRB1*11:29	P43355	MAGE1	94	108	37.28
2849	LESLFRAVITKKVAD	DRB1*11:42	P43355	MAGE1	94	108	38.13
2850	LESLFRAVITKKVAD	DRB1*11:46	P43355	MAGE1	94	108	49.59
2851	LESLFRAVITKKVAD	DRB1*11:49	P43355	MAGE1	94	108	37.28
2852	LESLFRAVITKKVAD	DRB1*11:58	P43355	MAGE1	94	108	49.59
2853	LESLFRAVITKKVAD	DRB1*11:62	P43355	MAGE1	94	108	37.28
2854	LESLFRAVITKKVAD	DRB1*11:74	P43355	MAGE1	94	108	37.28
2855	LESLFRAVITKKVAD	DRB1*13:05	P43355	MAGE1	94	108	37.28
2856	LESLFRAVITKKVAD	DRB1*13:11	P43355	MAGE1	94	108	49.59
2857	LESLFRAVITKKVAD	DRB1*13:14	P43355	MAGE1	94	108	37.28
2858	LESLFRAVITKKVAD	DRB1*13:21	P43355	MAGE1	94	108	36.95
2859	LESLFRAVITKKVAD	DRB1*13:50	P43355	MAGE1	94	108	37.28
2860	LESLFRAVITKKVAD	DRB1*15:15	P43355	MAGE1	94	108	29.96
2861	LESLFRAVITKKVAD	DRB1*16:01	P43355	MAGE1	94	108	24.81
2862	LESLFRAVITKKVAD	DRB1*16:02	P43355	MAGE1	94	108	21.91
2863	LESLFRAVITKKVAD	DRB1*16:04	P43355	MAGE1	94	108	41.16
2864	LESLFRAVITKKVAD	DRB1*16:09	P43355	MAGE1	94	108	24.24
2865	LEYVIKVSARVRFFF	DRB1*01:01	P43355	MAGE1	280	294	11.33
2866	LEYVIKVSARVRFFF	DRB1*01:11	P43355	MAGE1	280	294	19.27
2867	LEYVIKVSARVRFFF	DRB1*01:18	P43355	MAGE1	280	294	7.83
2868	LEYVIKVSARVRFFF	DRB1*01:20	P43355	MAGE1	280	294	18.46
2869	LEYVIKVSARVRFFF	DRB1*01:24	P43355	MAGE1	280	294	18.45
2870	LEYVIKVSARVRFFF	DRB1*01:29	P43355	MAGE1	280	294	15.65
2871	LEYVIKVSARVRFFF	DRB1*03:01	P43355	MAGE1	280	294	39.5
2872	LEYVIKVSARVRFFF	DRB1*03:04	P43355	MAGE1	280	294	39.5
2873	LEYVIKVSARVRFFF	DRB1*03:11	P43355	MAGE1	280	294	18.47
2874	LEYVIKVSARVRFFF	DRB1*03:13	P43355	MAGE1	280	294	39.5
2875	LEYVIKVSARVRFFF	DRB1*03:15	P43355	MAGE1	280	294	36.01
2876	LEYVIKVSARVRFFF	DRB1*07:01	P43355	MAGE1	280	294	36.39

2877	LEYVIKVSARVRFFF	DRB1*08:04	P43355	MAGE1	280	294	34.64
2878	LEYVIKVSARVRFFF	DRB1*08:30	P43355	MAGE1	280	294	44.27
2879	LEYVIKVSARVRFFF	DRB1*10:01	P43355	MAGE1	280	294	44.71
2880	LEYVIKVSARVRFFF	DRB1*11:01	P43355	MAGE1	280	294	34.02
2881	LEYVIKVSARVRFFF	DRB1*11:02	P43355	MAGE1	280	294	9.32
2882	LEYVIKVSARVRFFF	DRB1*11:03	P43355	MAGE1	280	294	18.68
2883	LEYVIKVSARVRFFF	DRB1*11:04	P43355	MAGE1	280	294	17.21
2884	LEYVIKVSARVRFFF	DRB1*11:07	P43355	MAGE1	280	294	47.07
2885	LEYVIKVSARVRFFF	DRB1*11:08	P43355	MAGE1	280	294	27.58
2886	LEYVIKVSARVRFFF	DRB1*11:10	P43355	MAGE1	280	294	34.02
2887	LEYVIKVSARVRFFF	DRB1*11:12	P43355	MAGE1	280	294	34.02
2888	LEYVIKVSARVRFFF	DRB1*11:13	P43355	MAGE1	280	294	10.4
2889	LEYVIKVSARVRFFF	DRB1*11:14	P43355	MAGE1	280	294	24.27
2890	LEYVIKVSARVRFFF	DRB1*11:19	P43355	MAGE1	280	294	43.02
2891	LEYVIKVSARVRFFF	DRB1*11:28	P43355	MAGE1	280	294	34.02
2892	LEYVIKVSARVRFFF	DRB1*11:29	P43355	MAGE1	280	294	34.02
2893	LEYVIKVSARVRFFF	DRB1*11:42	P43355	MAGE1	280	294	10.49
2894	LEYVIKVSARVRFFF	DRB1*11:46	P43355	MAGE1	280	294	17.21
2895	LEYVIKVSARVRFFF	DRB1*11:49	P43355	MAGE1	280	294	34.02
2896	LEYVIKVSARVRFFF	DRB1*11:58	P43355	MAGE1	280	294	17.21
2897	LEYVIKVSARVRFFF	DRB1*11:62	P43355	MAGE1	280	294	34.02
2898	LEYVIKVSARVRFFF	DRB1*11:65	P43355	MAGE1	280	294	9.32
2899	LEYVIKVSARVRFFF	DRB1*11:74	P43355	MAGE1	280	294	34.02
2900	LEYVIKVSARVRFFF	DRB1*11:84	P43355	MAGE1	280	294	26.66
2901	LEYVIKVSARVRFFF	DRB1*12:03	P43355	MAGE1	280	294	37.49
2902	LEYVIKVSARVRFFF	DRB1*13:01	P43355	MAGE1	280	294	9.32
2903	LEYVIKVSARVRFFF	DRB1*13:02	P43355	MAGE1	280	294	24.27
2904	LEYVIKVSARVRFFF	DRB1*13:05	P43355	MAGE1	280	294	34.02
2905	LEYVIKVSARVRFFF	DRB1*13:11	P43355	MAGE1	280	294	17.21
2906	LEYVIKVSARVRFFF	DRB1*13:14	P43355	MAGE1	280	294	34.02
2907	LEYVIKVSARVRFFF	DRB1*13:21	P43355	MAGE1	280	294	41.23
2908	LEYVIKVSARVRFFF	DRB1*13:23	P43355	MAGE1	280	294	24.27
2909	LEYVIKVSARVRFFF	DRB1*13:50	P43355	MAGE1	280	294	34.02
2910	LEYVIKVSARVRFFF	DRB1*13:61	P43355	MAGE1	280	294	17.8
2911	LEYVIKVSARVRFFF	DRB1*13:96	P43355	MAGE1	280	294	32.92
2912	LEYVIKVSARVRFFF	DRB1*13:97	P43355	MAGE1	280	294	24.27
2913	LEYVIKVSARVRFFF	DRB1*14:01	P43355	MAGE1	280	294	22.69
2914	LEYVIKVSARVRFFF	DRB1*14:04	P43355	MAGE1	280	294	38.32
2915	LEYVIKVSARVRFFF	DRB1*14:05	P43355	MAGE1	280	294	45.32
2916	LEYVIKVSARVRFFF	DRB1*14:06	P43355	MAGE1	280	294	23.98

2917	LEYVIKVSARVRFFF	DRB1*14:12	P43355	MAGE1	280	294	29.78
2918	LEYVIKVSARVRFFF	DRB1*14:23	P43355	MAGE1	280	294	45.32
2919	LEYVIKVSARVRFFF	DRB1*14:32	P43355	MAGE1	280	294	13.51
2920	LEYVIKVSARVRFFF	DRB1*14:38	P43355	MAGE1	280	294	38.02
2921	LEYVIKVSARVRFFF	DRB1*14:54	P43355	MAGE1	280	294	22.69
2922	LEYVIKVSARVRFFF	DRB1*15:01	P43355	MAGE1	280	294	40.1
2923	LEYVIKVSARVRFFF	DRB1*15:02	P43355	MAGE1	280	294	49.76
2924	LEYVIKVSARVRFFF	DRB1*15:06	P43355	MAGE1	280	294	40.1
2925	LEYVIKVSARVRFFF	DRB1*15:15	P43355	MAGE1	280	294	43.25
2926	LEYVIKVSARVRFFF	DRB1*16:02	P43355	MAGE1	280	294	40.37
2927	LEYVIKVSARVRFFF	DRB1*16:09	P43355	MAGE1	280	294	32.69
2928	LFRAVITKKVADLVG	DRB1*01:01	P43355	MAGE1	97	111	10.68
2929	LFRAVITKKVADLVG	DRB1*01:11	P43355	MAGE1	97	111	28.66
2930	LFRAVITKKVADLVG	DRB1*01:18	P43355	MAGE1	97	111	8.78
2931	LFRAVITKKVADLVG	DRB1*01:20	P43355	MAGE1	97	111	28.17
2932	LFRAVITKKVADLVG	DRB1*01:24	P43355	MAGE1	97	111	18.77
2933	LFRAVITKKVADLVG	DRB1*01:29	P43355	MAGE1	97	111	17.65
2934	LFRAVITKKVADLVG	DRB1*07:01	P43355	MAGE1	97	111	13.1
2935	LFRAVITKKVADLVG	DRB1*09:01	P43355	MAGE1	97	111	35.07
2936	LFRAVITKKVADLVG	DRB1*10:01	P43355	MAGE1	97	111	25.87
2937	LFRAVITKKVADLVG	DRB1*11:42	P43355	MAGE1	97	111	43.51
2938	LFRAVITKKVADLVG	DRB1*13:21	P43355	MAGE1	97	111	37.44
2939	LFRAVITKKVADLVG	DRB1*15:15	P43355	MAGE1	97	111	38.05
2940	LFRAVITKKVADLVG	DRB1*16:01	P43355	MAGE1	97	111	30.22
2941	LFRAVITKKVADLVG	DRB1*16:02	P43355	MAGE1	97	111	39.28
2942	LFRAVITKKVADLVG	DRB1*16:04	P43355	MAGE1	97	111	44.71
2943	LFRAVITKKVADLVG	DRB1*16:09	P43355	MAGE1	97	111	25.47
2944	LFREALSNKVDELAH	DRB1*01:01	P43358	MAGE4	105	119	7.18
2945	LFREALSNKVDELAH	DRB1*01:11	P43358	MAGE4	105	119	19.34
2946	LFREALSNKVDELAH	DRB1*01:18	P43358	MAGE4	105	119	7.98
2947	LFREALSNKVDELAH	DRB1*01:20	P43358	MAGE4	105	119	32.68
2948	LFREALSNKVDELAH	DRB1*01:24	P43358	MAGE4	105	119	16.94
2949	LFREALSNKVDELAH	DRB1*01:29	P43358	MAGE4	105	119	15.46
2950	LFREALSNKVDELAH	DRB1*07:01	P43358	MAGE4	105	119	10.99
2951	LFREALSNKVDELAH	DRB1*09:01	P43358	MAGE4	105	119	15.87
2952	LFREALSNKVDELAH	DRB1*10:01	P43358	MAGE4	105	119	25.01
2953	LFREALSNKVDELAH	DRB1*16:02	P43358	MAGE4	105	119	38.98
2954	LFVIATRRSEEILGP	DRB1*08:01	P17643	TRP1	158	172	45.4
2955	LFVIATRRSEEILGP	DRB1*11:01	P17643	TRP1	158	172	24.07
2956	LFVIATRRSEEILGP	DRB1*11:04	P17643	TRP1	158	172	37.31

2957	LFVIATRRSEEILGP	DRB1*11:10	P17643	TRP1	158	172	24.07
2958	LFVIATRRSEEILGP	DRB1*11:12	P17643	TRP1	158	172	24.07
2959	LFVIATRRSEEILGP	DRB1*11:28	P17643	TRP1	158	172	24.07
2960	LFVIATRRSEEILGP	DRB1*11:29	P17643	TRP1	158	172	24.07
2961	LFVIATRRSEEILGP	DRB1*11:42	P17643	TRP1	158	172	45.56
2962	LFVIATRRSEEILGP	DRB1*11:46	P17643	TRP1	158	172	37.31
2963	LFVIATRRSEEILGP	DRB1*11:49	P17643	TRP1	158	172	24.07
2964	LFVIATRRSEEILGP	DRB1*11:58	P17643	TRP1	158	172	37.31
2965	LFVIATRRSEEILGP	DRB1*11:62	P17643	TRP1	158	172	24.07
2966	LFVIATRRSEEILGP	DRB1*11:74	P17643	TRP1	158	172	24.07
2967	LFVIATRRSEEILGP	DRB1*13:05	P17643	TRP1	158	172	24.07
2968	LFVIATRRSEEILGP	DRB1*13:11	P17643	TRP1	158	172	37.31
2969	LFVIATRRSEEILGP	DRB1*13:14	P17643	TRP1	158	172	24.07
2970	LFVIATRRSEEILGP	DRB1*13:21	P17643	TRP1	158	172	13.5
2971	LFVIATRRSEEILGP	DRB1*13:50	P17643	TRP1	158	172	24.07
2972	LFVWMHYYVSMDALL	DPB1*33:01	P14679	Tyrosinase	175	189	29.2
2973	LFVWMHYYVSMDALL	DPB1*71:01	P14679	Tyrosinase	175	189	29.2
2974	LGALDLAKKRVHPDY	DRB1*11:03	O75767	TRP2	142	156	37.56
2975	LGALDLAKKRVHPDY	DRB1*11:04	O75767	TRP2	142	156	30.57
2976	LGALDLAKKRVHPDY	DRB1*11:42	O75767	TRP2	142	156	38.96
2977	LGALDLAKKRVHPDY	DRB1*11:46	O75767	TRP2	142	156	30.57
2978	LGALDLAKKRVHPDY	DRB1*11:58	O75767	TRP2	142	156	30.57
2979	LGALDLAKKRVHPDY	DRB1*13:11	O75767	TRP2	142	156	30.57
2980	LGDNQIMPKTGFLII	DRB1*01:20	P43355	MAGE1	183	197	38.57
2981	LGFKATLPPFMCNKR	DRB1*01:01	Q16385	SSX2	61	75	17.49
2982	LGFKATLPPFMCNKR	DRB1*01:11	Q16385	SSX2	61	75	49.21
2983	LGFKATLPPFMCNKR	DRB1*01:18	Q16385	SSX2	61	75	17.23
2984	LGFKATLPPFMCNKR	DRB1*01:24	Q16385	SSX2	61	75	39.73
2985	LGFKATLPPFMCNKR	DRB1*01:29	Q16385	SSX2	61	75	35.33
2986	LGFKATLPPFMCNKR	DRB1*07:01	Q16385	SSX2	61	75	21.75
2987	LGFKATLPPFMCNKR	DRB1*09:01	Q16385	SSX2	61	75	30.82
2988	LGFKATLPPFMCNKR	DRB1*10:01	Q16385	SSX2	61	75	14.93
2989	LGGFFPWLKVYYYRF	DPB1*33:01	O75767	TRP2	198	212	43.46
2990	LGGFFPWLKVYYYRF	DPB1*71:01	O75767	TRP2	198	212	43.46
2991	LGLVCVQAATSSSSP	DRB1*01:01	P43355	MAGE1	23	37	35.99
2992	LGLVCVQAATSSSSP	DRB1*01:18	P43355	MAGE1	23	37	34.58
2993	LGLVCVQAATSSSSP	DRB1*01:20	P43355	MAGE1	23	37	30.99
2994	LGLVCVQAATSSSSP	DRB1*10:01	P43355	MAGE1	23	37	45.92
2995	LGNNQIFPKTGLLII	DRB1*01:18	P43358	MAGE4	191	205	45.79
2996	LGNNQIFPKTGLLII	DRB1*01:20	P43358	MAGE4	191	205	39.66

2997	LGPLLDGTATLRLVK	DRB1*07:01	P40967	PMEL17	454	468	43.03
2998	LGVSRQLRTKAWNRQ	DRB1*11:03	P40967	PMEL17	33	47	40.87
2999	LGVSRQLRTKAWNRQ	DRB1*11:04	P40967	PMEL17	33	47	48.19
3000	LGVSRQLRTKAWNRQ	DRB1*11:42	P40967	PMEL17	33	47	44.51
3001	LGVSRQLRTKAWNRQ	DRB1*11:46	P40967	PMEL17	33	47	48.19
3002	LGVSRQLRTKAWNRQ	DRB1*11:58	P40967	PMEL17	33	47	48.19
3003	LGVSRQLRTKAWNRQ	DRB1*13:11	P40967	PMEL17	33	47	48.19
3004	LGYTYEIQWPSREFS	DRB1*10:01	P17643	TRP1	460	474	48.44
3005	LHHAFVDSIFEQWLR	DPB1*04:01	P14679	Tyrosinase	388	402	26.61
3006	LHHAFVDSIFEQWLR	DPB1*126:01	P14679	Tyrosinase	388	402	26.61
3007	LHHAFVDSIFEQWLR	DPB1*23:01	P14679	Tyrosinase	388	402	26.61
3008	LHHAFVDSIFEQWLR	DPB1*33:01	P14679	Tyrosinase	388	402	20.88
3009	LHHAFVDSIFEQWLR	DPB1*39:01	P14679	Tyrosinase	388	402	26.61
3010	LHHAFVDSIFEQWLR	DPB1*71:01	P14679	Tyrosinase	388	402	20.88
3011	LHHAFVDSIFEQWLR	DPB1*72:01	P14679	Tyrosinase	388	402	33.24
3012	LHIYMNGTMSQVQGS	DRB1*01:01	P14679	Tyrosinase	366	380	42.87
3013	LHIYMNGTMSQVQGS	DRB1*01:18	P14679	Tyrosinase	366	380	43.9
3014	LHIYMNGTMSQVQGS	DRB1*11:14	P14679	Tyrosinase	366	380	29.98
3015	LHIYMNGTMSQVQGS	DRB1*13:02	P14679	Tyrosinase	366	380	29.98
3016	LHIYMNGTMSQVQGS	DRB1*13:23	P14679	Tyrosinase	366	380	29.98
3017	LHIYMNGTMSQVQGS	DRB1*13:97	P14679	Tyrosinase	366	380	29.98
3018	LHLAVIGALLAVGAT	DRB1*01:01	P40967	PMEL17	10	24	20.12
3019	LHLAVIGALLAVGAT	DRB1*01:18	P40967	PMEL17	10	24	16.46
3020	LHLAVIGALLAVGAT	DRB1*01:20	P40967	PMEL17	10	24	19.84
3021	LHLAVIGALLAVGAT	DRB1*01:24	P40967	PMEL17	10	24	31.38
3022	LHLAVIGALLAVGAT	DRB1*01:29	P40967	PMEL17	10	24	35.46
3023	LHYYSVRDTLLGGFF	DRB1*01:01	O75767	TRP2	188	202	44.37
3024	LHYYSVRDTLLGGFF	DRB1*01:18	O75767	TRP2	188	202	42.86
3025	LIFGTASYLIRARRS	DRB1*01:01	P17643	TRP1	492	506	35.92
3026	LIFGTASYLIRARRS	DRB1*01:18	P17643	TRP1	492	506	27.38
3027	LIFGTASYLIRARRS	DRB1*01:20	P17643	TRP1	492	506	24.92
3028	LIFGTASYLIRARRS	DRB1*07:01	P17643	TRP1	492	506	13.71
3029	LIFGTASYLIRARRS	DRB1*11:02	P17643	TRP1	492	506	49.31
3030	LIFGTASYLIRARRS	DRB1*11:03	P17643	TRP1	492	506	41.99
3031	LIFGTASYLIRARRS	DRB1*11:04	P17643	TRP1	492	506	43.28
3032	LIFGTASYLIRARRS	DRB1*11:42	P17643	TRP1	492	506	31.15
3033	LIFGTASYLIRARRS	DRB1*11:46	P17643	TRP1	492	506	43.28
3034	LIFGTASYLIRARRS	DRB1*11:58	P17643	TRP1	492	506	43.28
3035	LIFGTASYLIRARRS	DRB1*11:65	P17643	TRP1	492	506	49.31
3036	LIFGTASYLIRARRS	DRB1*13:01	P17643	TRP1	492	506	49.31

3037	LIFGTASYLIRARRS	DRB1*13:11	P17643	TRP1	492	506	43.28
3038	LIGANASFSIALNFP	DRB1*11:14	P40967	PMEL17	77	91	36.78
3039	LIGANASFSIALNFP	DRB1*13:02	P40967	PMEL17	77	91	36.78
3040	LIGANASFSIALNFP	DRB1*13:23	P40967	PMEL17	77	91	36.78
3041	LIGANASFSIALNFP	DRB1*13:97	P40967	PMEL17	77	91	36.78
3042	LIIVLVMIAMEGGHA	DRB1*01:01	P43355	MAGE1	195	209	36.83
3043	LIIVLVMIAMEGGHA	DRB1*01:18	P43355	MAGE1	195	209	35.89
3044	LIIVLVMIAMEGGHA	DRB1*01:20	P43355	MAGE1	195	209	24.56
3045	LKEFTVSGNILTIRL	DRB1*01:01	P78358	NY-ESO-1	123	137	10.8
3046	LKEFTVSGNILTIRL	DRB1*01:11	P78358	NY-ESO-1	123	137	21.27
3047	LKEFTVSGNILTIRL	DRB1*01:18	P78358	NY-ESO-1	123	137	11.6
3048	LKEFTVSGNILTIRL	DRB1*01:20	P78358	NY-ESO-1	123	137	36.4
3049	LKEFTVSGNILTIRL	DRB1*01:24	P78358	NY-ESO-1	123	137	15.84
3050	LKEFTVSGNILTIRL	DRB1*01:29	P78358	NY-ESO-1	123	137	24.03
3051	LKEFTVSGNILTIRL	DRB1*04:01	P78358	NY-ESO-1	123	137	26.23
3052	LKEFTVSGNILTIRL	DRB1*04:08	P78358	NY-ESO-1	123	137	24.55
3053	LKEFTVSGNILTIRL	DRB1*04:72	P78358	NY-ESO-1	123	137	30.17
3054	LKEFTVSGNILTIRL	DRB1*07:01	P78358	NY-ESO-1	123	137	27.27
3055	LKEFTVSGNILTIRL	DRB1*10:01	P78358	NY-ESO-1	123	137	27.04
3056	LKEFTVSGNILTIRL	DRB1*11:14	P78358	NY-ESO-1	123	137	28.04
3057	LKEFTVSGNILTIRL	DRB1*13:02	P78358	NY-ESO-1	123	137	28.04
3058	LKEFTVSGNILTIRL	DRB1*13:23	P78358	NY-ESO-1	123	137	28.04
3059	LKEFTVSGNILTIRL	DRB1*13:96	P78358	NY-ESO-1	123	137	40.52
3060	LKEFTVSGNILTIRL	DRB1*13:97	P78358	NY-ESO-1	123	137	28.04
3061	LKEFTVSGNILTIRL	DRB1*16:01	P78358	NY-ESO-1	123	137	44.77
3062	LKEFTVSGNILTIRL	DRB1*16:02	P78358	NY-ESO-1	123	137	37.07
3063	LKEFTVSGNILTIRL	DRB1*16:05	P78358	NY-ESO-1	123	137	44.06
3064	LKELINNELSHFLEE	DRB1*11:14	P04271	S100	31	45	45.36
3065	LKELINNELSHFLEE	DRB1*13:02	P04271	S100	31	45	45.36
3066	LKELINNELSHFLEE	DRB1*13:23	P04271	S100	31	45	45.36
3067	LKELINNELSHFLEE	DRB1*13:97	P04271	S100	31	45	45.36
3068	LKKSELKELINNELS	DRB1*01:20	P04271	S100	26	40	45.07
3069	LKRCLLHLAVIGALL	DRB1*01:01	P40967	PMEL17	5	19	35.17
3070	LKRCLLHLAVIGALL	DRB1*01:18	P40967	PMEL17	5	19	22.72
3071	LKRCLLHLAVIGALL	DRB1*01:20	P40967	PMEL17	5	19	33.06
3072	LKRCLLHLAVIGALL	DRB1*01:24	P40967	PMEL17	5	19	38.93
3073	LKRCLLHLAVIGALL	DRB1*01:29	P40967	PMEL17	5	19	45.25
3074	LKVYYYRFVIGLRVW	DRB1*01:01	O75767	TRP2	205	219	9.7
3075	LKVYYYRFVIGLRVW	DRB1*01:11	O75767	TRP2	205	219	13.59
3076	LKVYYYRFVIGLRVW	DRB1*01:18	O75767	TRP2	205	219	6.9

3077	LKVYYYRFVIGLRVW	DRB1*01:20	O75767	TRP2	205	219	41.36
3078	LKVYYYRFVIGLRVW	DRB1*01:24	O75767	TRP2	205	219	8.17
3079	LKVYYYRFVIGLRVW	DRB1*01:29	O75767	TRP2	205	219	14.7
3080	LKVYYYRFVIGLRVW	DRB1*07:01	O75767	TRP2	205	219	15.43
3081	LKVYYYRFVIGLRVW	DRB1*10:01	O75767	TRP2	205	219	26.89
3082	LKVYYYRFVIGLRVW	DRB1*15:02	O75767	TRP2	205	219	30.65
3083	LKVYYYRFVIGLRVW	DRB1*15:15	O75767	TRP2	205	219	43.07
3084	LKVYYYRFVIGLRVW	DRB1*16:01	O75767	TRP2	205	219	45.2
3085	LKVYYYRFVIGLRVW	DRB1*16:02	O75767	TRP2	205	219	32.79
3086	LKVYYYRFVIGLRVW	DRB1*16:05	O75767	TRP2	205	219	28.85
3087	LKVYYYRFVIGLRVW	DRB1*16:09	O75767	TRP2	205	219	27.73
3088	LLAGLVSLLCRHKRK	DRB1*01:18	P14679	Tyrosinase	491	505	45.21
3089	LLAGLVSLLCRHKRK	DRB1*01:20	P14679	Tyrosinase	491	505	35.8
3090	LLAGLVSLLCRHKRK	DRB1*11:02	P14679	Tyrosinase	491	505	29.59
3091	LLAGLVSLLCRHKRK	DRB1*11:03	P14679	Tyrosinase	491	505	19.4
3092	LLAGLVSLLCRHKRK	DRB1*11:04	P14679	Tyrosinase	491	505	27.66
3093	LLAGLVSLLCRHKRK	DRB1*11:42	P14679	Tyrosinase	491	505	27.59
3094	LLAGLVSLLCRHKRK	DRB1*11:46	P14679	Tyrosinase	491	505	27.66
3095	LLAGLVSLLCRHKRK	DRB1*11:58	P14679	Tyrosinase	491	505	27.66
3096	LLAGLVSLLCRHKRK	DRB1*11:65	P14679	Tyrosinase	491	505	29.59
3097	LLAGLVSLLCRHKRK	DRB1*13:01	P14679	Tyrosinase	491	505	29.59
3098	LLAGLVSLLCRHKRK	DRB1*13:11	P14679	Tyrosinase	491	505	27.66
3099	LLEFYLAMPFATPME	DPB1*33:01	P78358	NY-ESO-1	87	101	21.79
3100	LLEFYLAMPFATPME	DPB1*71:01	P78358	NY-ESO-1	87	101	21.79
3101	LLEFYLAMPFATPME	DRB1*01:01	P78358	NY-ESO-1	87	101	7.2
3102	LLEFYLAMPFATPME	DRB1*01:11	P78358	NY-ESO-1	87	101	14.66
3103	LLEFYLAMPFATPME	DRB1*01:18	P78358	NY-ESO-1	87	101	7
3104	LLEFYLAMPFATPME	DRB1*01:20	P78358	NY-ESO-1	87	101	20.13
3105	LLEFYLAMPFATPME	DRB1*01:24	P78358	NY-ESO-1	87	101	9.74
3106	LLEFYLAMPFATPME	DRB1*01:29	P78358	NY-ESO-1	87	101	11.68
3107	LLEFYLAMPFATPME	DRB1*07:01	P78358	NY-ESO-1	87	101	49.15
3108	LLEFYLAMPFATPME	DRB1*09:01	P78358	NY-ESO-1	87	101	28.28
3109	LLEFYLAMPFATPME	DRB1*10:01	P78358	NY-ESO-1	87	101	7.81
3110	LLEFYLAMPFATPME	DRB1*16:01	P78358	NY-ESO-1	87	101	48.01
3111	LLEFYLAMPFATPME	DRB1*16:09	P78358	NY-ESO-1	87	101	43.88
3112	LLFQQARAQFPRQCA	DRB1*01:01	P17643	TRP1	17	31	27.7
3113	LLFQQARAQFPRQCA	DRB1*01:18	P17643	TRP1	17	31	32.83
3114	LLFQQARAQFPRQCA	DRB1*10:01	P17643	TRP1	17	31	35.73
3115	LLHHAFVDSIFEQWL	DPB1*04:01	P14679	Tyrosinase	387	401	46.15
3116	LLHHAFVDSIFEQWL	DPB1*126:01	P14679	Tyrosinase	387	401	46.15

3117	LLHHAFVDSIFEQWL	DPB1*23:01	P14679	Tyrosinase	387	401	46.15
3118	LLHHAFVDSIFEQWL	DPB1*33:01	P14679	Tyrosinase	387	401	27.29
3119	LLHHAFVDSIFEQWL	DPB1*39:01	P14679	Tyrosinase	387	401	46.15
3120	LLHHAFVDSIFEQWL	DPB1*71:01	P14679	Tyrosinase	387	401	27.29
3121	LLHLAVIGALLAVGA	DRB1*01:01	P40967	PMEL17	9	23	31.21
3122	LLHLAVIGALLAVGA	DRB1*01:18	P40967	PMEL17	9	23	23.03
3123	LLHLAVIGALLAVGA	DRB1*01:20	P40967	PMEL17	9	23	32.18
3124	LLHLAVIGALLAVGA	DRB1*01:24	P40967	PMEL17	9	23	41.31
3125	LLKEFTVSGNILTIR	DRB1*01:01	P78358	NY-ESO-1	122	136	11.69
3126	LLKEFTVSGNILTIR	DRB1*01:11	P78358	NY-ESO-1	122	136	24.56
3127	LLKEFTVSGNILTIR	DRB1*01:18	P78358	NY-ESO-1	122	136	12.82
3128	LLKEFTVSGNILTIR	DRB1*01:20	P78358	NY-ESO-1	122	136	39.87
3129	LLKEFTVSGNILTIR	DRB1*01:24	P78358	NY-ESO-1	122	136	19.77
3130	LLKEFTVSGNILTIR	DRB1*01:29	P78358	NY-ESO-1	122	136	25.64
3131	LLKEFTVSGNILTIR	DRB1*04:01	P78358	NY-ESO-1	122	136	25.71
3132	LLKEFTVSGNILTIR	DRB1*04:08	P78358	NY-ESO-1	122	136	26
3133	LLKEFTVSGNILTIR	DRB1*04:72	P78358	NY-ESO-1	122	136	32.69
3134	LLKEFTVSGNILTIR	DRB1*07:01	P78358	NY-ESO-1	122	136	25.13
3135	LLKEFTVSGNILTIR	DRB1*10:01	P78358	NY-ESO-1	122	136	28.93
3136	LLKEFTVSGNILTIR	DRB1*11:14	P78358	NY-ESO-1	122	136	29.99
3137	LLKEFTVSGNILTIR	DRB1*13:02	P78358	NY-ESO-1	122	136	29.99
3138	LLKEFTVSGNILTIR	DRB1*13:23	P78358	NY-ESO-1	122	136	29.99
3139	LLKEFTVSGNILTIR	DRB1*13:96	P78358	NY-ESO-1	122	136	49.38
3140	LLKEFTVSGNILTIR	DRB1*13:97	P78358	NY-ESO-1	122	136	29.99
3141	LLKEFTVSGNILTIR	DRB1*16:01	P78358	NY-ESO-1	122	136	48.67
3142	LLKEFTVSGNILTIR	DRB1*16:02	P78358	NY-ESO-1	122	136	38.47
3143	LLLFQQARAQFPRQC	DRB1*01:01	P17643	TRP1	16	30	16.54
3144	LLLFQQARAQFPRQC	DRB1*01:18	P17643	TRP1	16	30	17.26
3145	LLLFQQARAQFPRQC	DRB1*01:20	P17643	TRP1	16	30	23.29
3146	LLLFQQARAQFPRQC	DRB1*01:24	P17643	TRP1	16	30	36.75
3147	LLLFQQARAQFPRQC	DRB1*01:29	P17643	TRP1	16	30	36.24
3148	LLLFQQARAQFPRQC	DRB1*10:01	P17643	TRP1	16	30	20.68
3149	LLLFQQARAQFPRQC	DRB1*11:42	P17643	TRP1	16	30	41.87
3150	LLLKYRAREPVTKAE	DRB1*11:03	P43355	MAGE1	113	127	39.1
3151	LLLVALIFGTASYLI	DRB1*01:18	P17643	TRP1	487	501	42.66
3152	LLLVALIFGTASYLI	DRB1*07:01	P17643	TRP1	487	501	28.35
3153	LLMWITQCFLPVFLA	DPB1*02:01	P78358	NY-ESO-1	158	172	48.52
3154	LLMWITQCFLPVFLA	DPB1*02:02	P78358	NY-ESO-1	158	172	44.65
3155	LLMWITQCFLPVFLA	DPB1*04:01	P78358	NY-ESO-1	158	172	25.52
3156	LLMWITQCFLPVFLA	DPB1*126:01	P78358	NY-ESO-1	158	172	25.52

3157	LLMWITQCFLPVFLA	DPB1*15:01	P78358	NY-ESO-1	158	172	49.16
3158	LLMWITQCFLPVFLA	DPB1*23:01	P78358	NY-ESO-1	158	172	25.52
3159	LLMWITQCFLPVFLA	DPB1*33:01	P78358	NY-ESO-1	158	172	19.81
3160	LLMWITQCFLPVFLA	DPB1*39:01	P78358	NY-ESO-1	158	172	25.52
3161	LLMWITQCFLPVFLA	DPB1*46:01	P78358	NY-ESO-1	158	172	48.52
3162	LLMWITQCFLPVFLA	DPB1*47:01	P78358	NY-ESO-1	158	172	44.65
3163	LLMWITQCFLPVFLA	DPB1*71:01	P78358	NY-ESO-1	158	172	19.81
3164	LLMWITQCFLPVFLA	DPB1*72:01	P78358	NY-ESO-1	158	172	49.67
3165	LLMWITQCFLPVFLA	DPB1*81:01	P78358	NY-ESO-1	158	172	48.52
3166	LLRKYRAKELVTKAE	DRB1*15:01	P43358	MAGE4	121	135	25.64
3167	LLRKYRAKELVTKAE	DRB1*15:03	P43358	MAGE4	121	135	39.05
3168	LLRKYRAKELVTKAE	DRB1*15:06	P43358	MAGE4	121	135	25.64
3169	LLVALIFGTASYLIR	DRB1*01:01	P17643	TRP1	488	502	20.95
3170	LLVALIFGTASYLIR	DRB1*01:11	P17643	TRP1	488	502	40.58
3171	LLVALIFGTASYLIR	DRB1*01:18	P17643	TRP1	488	502	16.91
3172	LLVALIFGTASYLIR	DRB1*01:20	P17643	TRP1	488	502	21.86
3173	LLVALIFGTASYLIR	DRB1*01:24	P17643	TRP1	488	502	28.65
3174	LLVALIFGTASYLIR	DRB1*01:29	P17643	TRP1	488	502	35.64
3175	LLVALIFGTASYLIR	DRB1*07:01	P17643	TRP1	488	502	15.71
3176	LLWSFQTSAGHFPRA	DRB1*01:01	P14679	Tyrosinase	9	23	45.88
3177	LLWSFQTSAGHFPRA	DRB1*01:18	P14679	Tyrosinase	9	23	43.93
3178	LLWSFQTSAGHFPRA	DRB1*10:01	P14679	Tyrosinase	9	23	33.85
3179	LMAVVLASLIYRRRL	DRB1*01:18	P40967	PMEL17	606	620	37.92
3180	LMAVVLASLIYRRRL	DRB1*01:20	P40967	PMEL17	606	620	32.68
3181	LMAVVLASLIYRRRL	DRB1*11:02	P40967	PMEL17	606	620	26.81
3182	LMAVVLASLIYRRRL	DRB1*11:03	P40967	PMEL17	606	620	34.91
3183	LMAVVLASLIYRRRL	DRB1*11:04	P40967	PMEL17	606	620	37.88
3184	LMAVVLASLIYRRRL	DRB1*11:13	P40967	PMEL17	606	620	28.16
3185	LMAVVLASLIYRRRL	DRB1*11:42	P40967	PMEL17	606	620	27.63
3186	LMAVVLASLIYRRRL	DRB1*11:46	P40967	PMEL17	606	620	37.88
3187	LMAVVLASLIYRRRL	DRB1*11:58	P40967	PMEL17	606	620	37.88
3188	LMAVVLASLIYRRRL	DRB1*11:65	P40967	PMEL17	606	620	26.81
3189	LMAVVLASLIYRRRL	DRB1*12:03	P40967	PMEL17	606	620	49.45
3190	LMAVVLASLIYRRRL	DRB1*13:01	P40967	PMEL17	606	620	26.81
3191	LMAVVLASLIYRRRL	DRB1*13:11	P40967	PMEL17	606	620	37.88
3192	LMAVVLASLIYRRRL	DRB1*14:06	P40967	PMEL17	606	620	38.83
3193	LMWITQCFLPVFLAQ	DPB1*01:01	P78358	NY-ESO-1	159	173	45.99
3194	LMWITQCFLPVFLAQ	DPB1*02:01	P78358	NY-ESO-1	159	173	33.13
3195	LMWITQCFLPVFLAQ	DPB1*02:02	P78358	NY-ESO-1	159	173	31.31
3196	LMWITQCFLPVFLAQ	DPB1*04:01	P78358	NY-ESO-1	159	173	19.43

3197	LMWITQCFLPVFLAQ	DPB1*126:01	P78358	NY-ESO-1	159	173	19.43
3198	LMWITQCFLPVFLAQ	DPB1*15:01	P78358	NY-ESO-1	159	173	41.42
3199	LMWITQCFLPVFLAQ	DPB1*23:01	P78358	NY-ESO-1	159	173	19.43
3200	LMWITQCFLPVFLAQ	DPB1*33:01	P78358	NY-ESO-1	159	173	14.71
3201	LMWITQCFLPVFLAQ	DPB1*39:01	P78358	NY-ESO-1	159	173	19.43
3202	LMWITQCFLPVFLAQ	DPB1*46:01	P78358	NY-ESO-1	159	173	33.13
3203	LMWITQCFLPVFLAQ	DPB1*47:01	P78358	NY-ESO-1	159	173	31.31
3204	LMWITQCFLPVFLAQ	DPB1*71:01	P78358	NY-ESO-1	159	173	14.71
3205	LMWITQCFLPVFLAQ	DPB1*72:01	P78358	NY-ESO-1	159	173	37.97
3206	LMWITQCFLPVFLAQ	DPB1*81:01	P78358	NY-ESO-1	159	173	33.13
3207	LPDGQVIWVNNTIIN	DRB1*11:14	P40967	PMEL17	97	111	23.66
3208	LPDGQVIWVNNTIIN	DRB1*13:02	P40967	PMEL17	97	111	23.66
3209	LPDGQVIWVNNTIIN	DRB1*13:23	P40967	PMEL17	97	111	23.66
3210	LPDGQVIWVNNTIIN	DRB1*13:96	P40967	PMEL17	97	111	42.31
3211	LPDGQVIWVNNTIIN	DRB1*13:97	P40967	PMEL17	97	111	23.66
3212	LPYWNFATGKNVCDI	DRB1*01:01	P17643	TRP1	246	260	24.53
3213	LPYWNFATGKNVCDI	DRB1*01:18	P17643	TRP1	246	260	26.73
3214	LPYWNFATGKNVCDI	DRB1*09:01	P17643	TRP1	246	260	43.46
3215	LSAPEKDKFFAYLTL	DPB1*33:01	P14679	Tyrosinase	126	140	47.38
3216	LSAPEKDKFFAYLTL	DPB1*71:01	P14679	Tyrosinase	126	140	47.38
3217	LSAQLLQARLMKEES	DRB1*01:01	Q13072	BAGE	10	24	22.28
3218	LSAQLLQARLMKEES	DRB1*01:18	Q13072	BAGE	10	24	19.99
3219	LSAQLLQARLMKEES	DRB1*01:20	Q13072	BAGE	10	24	17.14
3220	LSLGCIFFPLLLFQQ	DPB1*33:01	P17643	TRP1	7	21	43.26
3221	LSLGCIFFPLLLFQQ	DPB1*71:01	P17643	TRP1	7	21	43.26
3222	LSLLMWITQCFLPVF	DPB1*01:01	P78358	NY-ESO-1	156	170	47.73
3223	LSLLMWITQCFLPVF	DPB1*02:01	P78358	NY-ESO-1	156	170	36.97
3224	LSLLMWITQCFLPVF	DPB1*02:02	P78358	NY-ESO-1	156	170	34.46
3225	LSLLMWITQCFLPVF	DPB1*04:01	P78358	NY-ESO-1	156	170	16.8
3226	LSLLMWITQCFLPVF	DPB1*126:01	P78358	NY-ESO-1	156	170	16.8
3227	LSLLMWITQCFLPVF	DPB1*15:01	P78358	NY-ESO-1	156	170	44.12
3228	LSLLMWITQCFLPVF	DPB1*23:01	P78358	NY-ESO-1	156	170	16.8
3229	LSLLMWITQCFLPVF	DPB1*33:01	P78358	NY-ESO-1	156	170	14.2
3230	LSLLMWITQCFLPVF	DPB1*39:01	P78358	NY-ESO-1	156	170	16.8
3231	LSLLMWITQCFLPVF	DPB1*46:01	P78358	NY-ESO-1	156	170	36.97
3232	LSLLMWITQCFLPVF	DPB1*47:01	P78358	NY-ESO-1	156	170	34.46
3233	LSLLMWITQCFLPVF	DPB1*71:01	P78358	NY-ESO-1	156	170	14.2
3234	LSLLMWITQCFLPVF	DPB1*72:01	P78358	NY-ESO-1	156	170	32.31
3235	LSLLMWITQCFLPVF	DPB1*81:01	P78358	NY-ESO-1	156	170	36.97
3236	LTALLAGLVSLLCRH	DRB1*01:01	P14679	Tyrosinase	488	502	30.25

3237	LTALLAGLVSLLCRH	DRB1*01:18	P14679	Tyrosinase	488	502	24.03
3238	LTALLAGLVSLLCRH	DRB1*01:20	P14679	Tyrosinase	488	502	23.56
3239	LTALLAGLVSLLCRH	DRB1*01:24	P14679	Tyrosinase	488	502	42.01
3240	LTIRLTAADHRQLQL	DRB1*01:20	P78358	NY-ESO-1	133	147	46.46
3241	LTIRLTAADHRQLQL	DRB1*03:11	P78358	NY-ESO-1	133	147	40.34
3242	LTIRLTAADHRQLQL	DRB1*11:13	P78358	NY-ESO-1	133	147	44.52
3243	LTIRLTAADHRQLQL	DRB1*11:42	P78358	NY-ESO-1	133	147	44.84
3244	LTWHRYHLLRLEKDM	DPB1*33:01	P17643	TRP1	221	235	27.66
3245	LTWHRYHLLRLEKDM	DPB1*71:01	P17643	TRP1	221	235	27.66
3246	LTWHRYHLLRLEKDM	DRB1*10:01	P17643	TRP1	221	235	37.94
3247	LVALIFGTASYLIRA	DPB1*33:01	P17643	TRP1	489	503	46.8
3248	LVALIFGTASYLIRA	DPB1*71:01	P17643	TRP1	489	503	46.8
3249	LVALIFGTASYLIRA	DRB1*01:01	P17643	TRP1	489	503	15.16
3250	LVALIFGTASYLIRA	DRB1*01:11	P17643	TRP1	489	503	28.86
3251	LVALIFGTASYLIRA	DRB1*01:18	P17643	TRP1	489	503	12.34
3252	LVALIFGTASYLIRA	DRB1*01:20	P17643	TRP1	489	503	15.6
3253	LVALIFGTASYLIRA	DRB1*01:24	P17643	TRP1	489	503	20.63
3254	LVALIFGTASYLIRA	DRB1*01:29	P17643	TRP1	489	503	24.53
3255	LVALIFGTASYLIRA	DRB1*07:01	P17643	TRP1	489	503	15.15
3256	LVALIFGTASYLIRA	DRB1*15:01	P17643	TRP1	489	503	35.69
3257	LVALIFGTASYLIRA	DRB1*15:06	P17643	TRP1	489	503	35.69
3258	LVGFLLLKYRAREPV	DRB1*01:18	P43355	MAGE1	109	123	40.27
3259	LVGFLLLKYRAREPV	DRB1*01:20	P43355	MAGE1	109	123	39.34
3260	LVGFLLLKYRAREPV	DRB1*08:04	P43355	MAGE1	109	123	49.14
3261	LVGFLLLKYRAREPV	DRB1*11:01	P43355	MAGE1	109	123	26.38
3262	LVGFLLLKYRAREPV	DRB1*11:02	P43355	MAGE1	109	123	29.64
3263	LVGFLLLKYRAREPV	DRB1*11:03	P43355	MAGE1	109	123	18.65
3264	LVGFLLLKYRAREPV	DRB1*11:04	P43355	MAGE1	109	123	17.19
3265	LVGFLLLKYRAREPV	DRB1*11:10	P43355	MAGE1	109	123	26.38
3266	LVGFLLLKYRAREPV	DRB1*11:12	P43355	MAGE1	109	123	26.38
3267	LVGFLLLKYRAREPV	DRB1*11:13	P43355	MAGE1	109	123	36.51
3268	LVGFLLLKYRAREPV	DRB1*11:28	P43355	MAGE1	109	123	26.38
3269	LVGFLLLKYRAREPV	DRB1*11:29	P43355	MAGE1	109	123	26.38
3270	LVGFLLLKYRAREPV	DRB1*11:42	P43355	MAGE1	109	123	20.39
3271	LVGFLLLKYRAREPV	DRB1*11:46	P43355	MAGE1	109	123	17.19
3272	LVGFLLLKYRAREPV	DRB1*11:49	P43355	MAGE1	109	123	26.38
3273	LVGFLLLKYRAREPV	DRB1*11:58	P43355	MAGE1	109	123	17.19
3274	LVGFLLLKYRAREPV	DRB1*11:62	P43355	MAGE1	109	123	26.38
3275	LVGFLLLKYRAREPV	DRB1*11:65	P43355	MAGE1	109	123	29.64
3276	LVGFLLLKYRAREPV	DRB1*11:74	P43355	MAGE1	109	123	26.38

3277	LVGFLLLKYRAREPV	DRB1*11:84	P43355	MAGE1	109	123	39.21
3278	LVGFLLLKYRAREPV	DRB1*12:16	P43355	MAGE1	109	123	46.68
3279	LVGFLLLKYRAREPV	DRB1*13:01	P43355	MAGE1	109	123	29.64
3280	LVGFLLLKYRAREPV	DRB1*13:05	P43355	MAGE1	109	123	26.38
3281	LVGFLLLKYRAREPV	DRB1*13:11	P43355	MAGE1	109	123	17.19
3282	LVGFLLLKYRAREPV	DRB1*13:14	P43355	MAGE1	109	123	26.38
3283	LVGFLLLKYRAREPV	DRB1*13:21	P43355	MAGE1	109	123	18.53
3284	LVGFLLLKYRAREPV	DRB1*13:50	P43355	MAGE1	109	123	26.38
3285	LVLHQILKGGSGTYC	DRB1*11:04	P40967	PMEL17	552	566	20.38
3286	LVLHQILKGGSGTYC	DRB1*11:42	P40967	PMEL17	552	566	42.96
3287	LVLHQILKGGSGTYC	DRB1*11:46	P40967	PMEL17	552	566	20.38
3288	LVLHQILKGGSGTYC	DRB1*11:58	P40967	PMEL17	552	566	20.38
3289	LVLHQILKGGSGTYC	DRB1*13:11	P40967	PMEL17	552	566	20.38
3290	LVLKRCLLHLAVIGA	DRB1*01:01	P40967	PMEL17	3	17	48.29
3291	LVLKRCLLHLAVIGA	DRB1*01:18	P40967	PMEL17	3	17	33.54
3292	LVLKRCLLHLAVIGA	DRB1*01:20	P40967	PMEL17	3	17	39.1
3293	LVMIAMEGGHAPEEE	DRB1*01:01	P43355	MAGE1	199	213	22.25
3294	LVMIAMEGGHAPEEE	DRB1*01:18	P43355	MAGE1	199	213	24.4
3295	LVMIAMEGGHAPEEE	DRB1*01:20	P43355	MAGE1	199	213	18.41
3296	LVSLLCRHKRKQLPE	DRB1*11:02	P14679	Tyrosinase	495	509	32.55
3297	LVSLLCRHKRKQLPE	DRB1*11:03	P14679	Tyrosinase	495	509	18.55
3298	LVSLLCRHKRKQLPE	DRB1*11:04	P14679	Tyrosinase	495	509	40.74
3299	LVSLLCRHKRKQLPE	DRB1*11:42	P14679	Tyrosinase	495	509	46.08
3300	LVSLLCRHKRKQLPE	DRB1*11:46	P14679	Tyrosinase	495	509	40.74
3301	LVSLLCRHKRKQLPE	DRB1*11:58	P14679	Tyrosinase	495	509	40.74
3302	LVSLLCRHKRKQLPE	DRB1*11:65	P14679	Tyrosinase	495	509	32.55
3303	LVSLLCRHKRKQLPE	DRB1*13:01	P14679	Tyrosinase	495	509	32.55
3304	LVSLLCRHKRKQLPE	DRB1*13:11	P14679	Tyrosinase	495	509	40.74
3305	LWGPRALAETSYVKV	DPB1*33:01	P43355	MAGE1	265	279	45.55
3306	LWGPRALAETSYVKV	DPB1*33:01	P43358	MAGE4	273	287	45.55
3307	LWGPRALAETSYVKV	DPB1*71:01	P43355	MAGE1	265	279	45.55
3308	LWGPRALAETSYVKV	DPB1*71:01	P43358	MAGE4	273	287	45.55
3309	LYCLLWSFQTSAGHF	DRB1*01:01	P14679	Tyrosinase	6	20	48.74
3310	LYCLLWSFQTSAGHF	DRB1*01:18	P14679	Tyrosinase	6	20	41.66
3311	LYRYGSFSVTLDIVQ	DPB1*33:01	P40967	PMEL17	477	491	45.7
3312	LYRYGSFSVTLDIVQ	DPB1*71:01	P40967	PMEL17	477	491	45.7
3313	LYRYGSFSVTLDIVQ	DRB1*04:05	P40967	PMEL17	477	491	25.04
3314	LYRYGSFSVTLDIVQ	DRB1*10:01	P40967	PMEL17	477	491	39.58
3315	MAARAVFLALSAQLL	DPB1*33:01	Q13072	BAGE	1	15	34.77
3316	MAARAVFLALSAQLL	DPB1*71:01	Q13072	BAGE	1	15	34.77

3317	MAARAVFLALSAQLL	DRB1*01:01	Q13072	BAGE	1	15	3.96
3318	MAARAVFLALSAQLL	DRB1*01:02	Q13072	BAGE	1	15	31.67
3319	MAARAVFLALSAQLL	DRB1*01:11	Q13072	BAGE	1	15	6.8
3320	MAARAVFLALSAQLL	DRB1*01:18	Q13072	BAGE	1	15	3.77
3321	MAARAVFLALSAQLL	DRB1*01:20	Q13072	BAGE	1	15	10.66
3322	MAARAVFLALSAQLL	DRB1*01:24	Q13072	BAGE	1	15	4.77
3323	MAARAVFLALSAQLL	DRB1*01:29	Q13072	BAGE	1	15	6.19
3324	MAARAVFLALSAQLL	DRB1*07:01	Q13072	BAGE	1	15	21.55
3325	MAARAVFLALSAQLL	DRB1*10:01	Q13072	BAGE	1	15	11.6
3326	MAARAVFLALSAQLL	DRB1*15:02	Q13072	BAGE	1	15	37.98
3327	MAARAVFLALSAQLL	DRB1*15:15	Q13072	BAGE	1	15	48.71
3328	MAARAVFLALSAQLL	DRB1*16:01	Q13072	BAGE	1	15	33.9
3329	MAARAVFLALSAQLL	DRB1*16:02	Q13072	BAGE	1	15	19.17
3330	MAARAVFLALSAQLL	DRB1*16:05	Q13072	BAGE	1	15	24.73
3331	MAARAVFLALSAQLL	DRB1*16:09	Q13072	BAGE	1	15	26.79
3332	MAFVAMVTTACHEFF	DRB1*01:01	P04271	S100	73	87	24.36
3333	MAFVAMVTTACHEFF	DRB1*01:11	P04271	S100	73	87	46.46
3334	MAFVAMVTTACHEFF	DRB1*01:18	P04271	S100	73	87	22.14
3335	MAFVAMVTTACHEFF	DRB1*01:20	P04271	S100	73	87	44.69
3336	MAFVAMVTTACHEFF	DRB1*01:24	P04271	S100	73	87	39.17
3337	MAFVAMVTTACHEFF	DRB1*01:29	P04271	S100	73	87	45.33
3338	MAFVAMVTTACHEFF	DRB1*04:04	P04271	S100	73	87	28.91
3339	MAFVAMVTTACHEFF	DRB1*04:08	P04271	S100	73	87	38.29
3340	MAFVAMVTTACHEFF	DRB1*10:01	P04271	S100	73	87	22.8
3341	MAVVLASLIYRRRLM	DRB1*01:18	P40967	PMEL17	607	621	47.77
3342	MAVVLASLIYRRRLM	DRB1*01:20	P40967	PMEL17	607	621	32.58
3343	MAVVLASLIYRRRLM	DRB1*11:02	P40967	PMEL17	607	621	21.84
3344	MAVVLASLIYRRRLM	DRB1*11:03	P40967	PMEL17	607	621	29.58
3345	MAVVLASLIYRRRLM	DRB1*11:04	P40967	PMEL17	607	621	30.33
3346	MAVVLASLIYRRRLM	DRB1*11:13	P40967	PMEL17	607	621	25.56
3347	MAVVLASLIYRRRLM	DRB1*11:42	P40967	PMEL17	607	621	24.17
3348	MAVVLASLIYRRRLM	DRB1*11:46	P40967	PMEL17	607	621	30.33
3349	MAVVLASLIYRRRLM	DRB1*11:58	P40967	PMEL17	607	621	30.33
3350	MAVVLASLIYRRRLM	DRB1*11:65	P40967	PMEL17	607	621	21.84
3351	MAVVLASLIYRRRLM	DRB1*12:03	P40967	PMEL17	607	621	44.58
3352	MAVVLASLIYRRRLM	DRB1*13:01	P40967	PMEL17	607	621	21.84
3353	MAVVLASLIYRRRLM	DRB1*13:11	P40967	PMEL17	607	621	30.33
3354	MAVVLASLIYRRRLM	DRB1*14:06	P40967	PMEL17	607	621	34.66
3355	MAVVLASLIYRRRLM	DRB1*14:32	P40967	PMEL17	607	621	46.89
3356	MEVTVYHRRGSRSYV	DRB1*11:02	P40967	PMEL17	184	198	34.06

3357	MEVTVYHRRGSRSYV	DRB1*11:03	P40967	PMEL17	184	198	20.84
3358	MEVTVYHRRGSRSYV	DRB1*11:65	P40967	PMEL17	184	198	34.06
3359	MEVTVYHRRGSRSYV	DRB1*13:01	P40967	PMEL17	184	198	34.06
3360	MFVTAPDNLGYTYEI	DRB1*04:01	P17643	TRP1	452	466	31.08
3361	MFVTAPDNLGYTYEI	DRB1*04:08	P17643	TRP1	452	466	36.69
3362	MHNALHIYMNGTMSQ	DRB1*11:14	P14679	Tyrosinase	362	376	39.88
3363	MHNALHIYMNGTMSQ	DRB1*13:02	P14679	Tyrosinase	362	376	39.88
3364	MHNALHIYMNGTMSQ	DRB1*13:23	P14679	Tyrosinase	362	376	39.88
3365	MHNALHIYMNGTMSQ	DRB1*13:97	P14679	Tyrosinase	362	376	39.88
3366	MHNALHIYMNGTMSQ	DRB1*15:01	P14679	Tyrosinase	362	376	26.91
3367	MHNALHIYMNGTMSQ	DRB1*15:06	P14679	Tyrosinase	362	376	26.91
3368	MHYYVSMDALLGGSE	DRB1*01:01	P14679	Tyrosinase	179	193	6.72
3369	MHYYVSMDALLGGSE	DRB1*01:11	P14679	Tyrosinase	179	193	19.93
3370	MHYYVSMDALLGGSE	DRB1*01:18	P14679	Tyrosinase	179	193	7.11
3371	MHYYVSMDALLGGSE	DRB1*01:20	P14679	Tyrosinase	179	193	24.19
3372	MHYYVSMDALLGGSE	DRB1*01:24	P14679	Tyrosinase	179	193	15.82
3373	MHYYVSMDALLGGSE	DRB1*01:29	P14679	Tyrosinase	179	193	12.68
3374	MHYYVSMDALLGGSE	DRB1*04:01	P14679	Tyrosinase	179	193	34.35
3375	MHYYVSMDALLGGSE	DRB1*04:05	P14679	Tyrosinase	179	193	45.55
3376	MHYYVSMDALLGGSE	DRB1*04:08	P14679	Tyrosinase	179	193	29.29
3377	MHYYVSMDALLGGSE	DRB1*10:01	P14679	Tyrosinase	179	193	9.68
3378	MHYYVSMDALLGGSE	DRB1*16:02	P14679	Tyrosinase	179	193	38.58
3379	MKRKYEAMTKLGFKA	DRB1*11:01	Q16385	SSX2	51	65	47.51
3380	MKRKYEAMTKLGFKA	DRB1*11:10	Q16385	SSX2	51	65	47.51
3381	MKRKYEAMTKLGFKA	DRB1*11:12	Q16385	SSX2	51	65	47.51
3382	MKRKYEAMTKLGFKA	DRB1*11:28	Q16385	SSX2	51	65	47.51
3383	MKRKYEAMTKLGFKA	DRB1*11:29	Q16385	SSX2	51	65	47.51
3384	MKRKYEAMTKLGFKA	DRB1*11:49	Q16385	SSX2	51	65	47.51
3385	MKRKYEAMTKLGFKA	DRB1*11:62	Q16385	SSX2	51	65	47.51
3386	MKRKYEAMTKLGFKA	DRB1*11:74	Q16385	SSX2	51	65	47.51
3387	MKRKYEAMTKLGFKA	DRB1*13:05	Q16385	SSX2	51	65	47.51
3388	MKRKYEAMTKLGFKA	DRB1*13:14	Q16385	SSX2	51	65	47.51
3389	MKRKYEAMTKLGFKA	DRB1*13:21	Q16385	SSX2	51	65	38.53
3390	MKRKYEAMTKLGFKA	DRB1*13:50	Q16385	SSX2	51	65	47.51
3391	MLERVIKNYKRCFPV	DRB1*11:02	P43358	MAGE4	136	150	32.77
3392	MLERVIKNYKRCFPV	DRB1*11:03	P43358	MAGE4	136	150	27.24
3393	MLERVIKNYKRCFPV	DRB1*11:42	P43358	MAGE4	136	150	37.63
3394	MLERVIKNYKRCFPV	DRB1*11:65	P43358	MAGE4	136	150	32.77
3395	MLERVIKNYKRCFPV	DRB1*13:01	P43358	MAGE4	136	150	32.77
3396	MQEMLQEPSFSLPYW	DRB1*01:20	P17643	TRP1	235	249	38.72

3397	MTFGRLQGISPKIMP	DRB1*01:01	Q16385	SSX2	99	113	17.02
3398	MTFGRLQGISPKIMP	DRB1*01:18	Q16385	SSX2	99	113	16.45
3399	MTFGRLQGISPKIMP	DRB1*01:20	Q16385	SSX2	99	113	29.64
3400	MTFGRLQGISPKIMP	DRB1*01:24	Q16385	SSX2	99	113	43.21
3401	MTFGRLQGISPKIMP	DRB1*01:29	Q16385	SSX2	99	113	49.91
3402	MTFGRLQGISPKIMP	DRB1*10:01	Q16385	SSX2	99	113	35.68
3403	MTKLGFKATLPPFMC	DRB1*01:01	Q16385	SSX2	58	72	20.03
3404	MTKLGFKATLPPFMC	DRB1*01:18	Q16385	SSX2	58	72	20.35
3405	MTKLGFKATLPPFMC	DRB1*01:24	Q16385	SSX2	58	72	43.21
3406	MTKLGFKATLPPFMC	DRB1*01:29	Q16385	SSX2	58	72	38.11
3407	MTKLGFKATLPPFMC	DRB1*07:01	Q16385	SSX2	58	72	15.13
3408	MTKLGFKATLPPFMC	DRB1*09:01	Q16385	SSX2	58	72	24.82
3409	MTKLGFKATLPPFMC	DRB1*10:01	Q16385	SSX2	58	72	16.45
3410	MVGAVLTALLAGLVS	DRB1*01:01	P14679	Tyrosinase	483	497	10.3
3411	MVGAVLTALLAGLVS	DRB1*01:02	P14679	Tyrosinase	483	497	32.45
3412	MVGAVLTALLAGLVS	DRB1*01:11	P14679	Tyrosinase	483	497	33.51
3413	MVGAVLTALLAGLVS	DRB1*01:18	P14679	Tyrosinase	483	497	10.38
3414	MVGAVLTALLAGLVS	DRB1*01:20	P14679	Tyrosinase	483	497	10.27
3415	MVGAVLTALLAGLVS	DRB1*01:24	P14679	Tyrosinase	483	497	17.95
3416	MVGAVLTALLAGLVS	DRB1*01:29	P14679	Tyrosinase	483	497	18.42
3417	MVGAVLTALLAGLVS	DRB1*10:01	P14679	Tyrosinase	483	497	42.59
3418	MVPFIPLYRNGDFFI	DRB1*08:01	P14679	Tyrosinase	426	440	42.22
3419	MVPFIPLYRNGDFFI	DRB1*11:01	P14679	Tyrosinase	426	440	32.73
3420	MVPFIPLYRNGDFFI	DRB1*11:10	P14679	Tyrosinase	426	440	32.73
3421	MVPFIPLYRNGDFFI	DRB1*11:12	P14679	Tyrosinase	426	440	32.73
3422	MVPFIPLYRNGDFFI	DRB1*11:28	P14679	Tyrosinase	426	440	32.73
3423	MVPFIPLYRNGDFFI	DRB1*11:29	P14679	Tyrosinase	426	440	32.73
3424	MVPFIPLYRNGDFFI	DRB1*11:49	P14679	Tyrosinase	426	440	32.73
3425	MVPFIPLYRNGDFFI	DRB1*11:62	P14679	Tyrosinase	426	440	32.73
3426	MVPFIPLYRNGDFFI	DRB1*11:74	P14679	Tyrosinase	426	440	32.73
3427	MVPFIPLYRNGDFFI	DRB1*13:05	P14679	Tyrosinase	426	440	32.73
3428	MVPFIPLYRNGDFFI	DRB1*13:14	P14679	Tyrosinase	426	440	32.73
3429	MVPFIPLYRNGDFFI	DRB1*13:21	P14679	Tyrosinase	426	440	14.15
3430	MVPFIPLYRNGDFFI	DRB1*13:50	P14679	Tyrosinase	426	440	32.73
3431	MWITQCFLPVFLAQP	DPB1*01:01	P78358	NY-ESO-1	160	174	40.19
3432	MWITQCFLPVFLAQP	DPB1*02:01	P78358	NY-ESO-1	160	174	29.27
3433	MWITQCFLPVFLAQP	DPB1*02:02	P78358	NY-ESO-1	160	174	27.56
3434	MWITQCFLPVFLAQP	DPB1*04:01	P78358	NY-ESO-1	160	174	19.17
3435	MWITQCFLPVFLAQP	DPB1*126:01	P78358	NY-ESO-1	160	174	19.17
3436	MWITQCFLPVFLAQP	DPB1*15:01	P78358	NY-ESO-1	160	174	46.54

3437	MWITQCFLPVFLAQP	DPB1*23:01	P78358	NY-ESO-1	160	174	19.17
3438	MWITQCFLPVFLAQP	DPB1*33:01	P78358	NY-ESO-1	160	174	13.3
3439	MWITQCFLPVFLAQP	DPB1*39:01	P78358	NY-ESO-1	160	174	19.17
3440	MWITQCFLPVFLAQP	DPB1*46:01	P78358	NY-ESO-1	160	174	29.27
3441	MWITQCFLPVFLAQP	DPB1*47:01	P78358	NY-ESO-1	160	174	27.56
3442	MWITQCFLPVFLAQP	DPB1*71:01	P78358	NY-ESO-1	160	174	13.3
3443	MWITQCFLPVFLAQP	DPB1*72:01	P78358	NY-ESO-1	160	174	28.46
3444	MWITQCFLPVFLAQP	DPB1*81:01	P78358	NY-ESO-1	160	174	29.27
3445	NALHIYMNGTMSQVQ	DRB1*01:01	P14679	Tyrosinase	364	378	29.38
3446	NALHIYMNGTMSQVQ	DRB1*01:18	P14679	Tyrosinase	364	378	28.56
3447	NALHIYMNGTMSQVQ	DRB1*01:20	P14679	Tyrosinase	364	378	35.55
3448	NALHIYMNGTMSQVQ	DRB1*10:01	P14679	Tyrosinase	364	378	48.73
3449	NALHIYMNGTMSQVQ	DRB1*11:14	P14679	Tyrosinase	364	378	21.33
3450	NALHIYMNGTMSQVQ	DRB1*13:02	P14679	Tyrosinase	364	378	21.33
3451	NALHIYMNGTMSQVQ	DRB1*13:23	P14679	Tyrosinase	364	378	21.33
3452	NALHIYMNGTMSQVQ	DRB1*13:96	P14679	Tyrosinase	364	378	42.15
3453	NALHIYMNGTMSQVQ	DRB1*13:97	P14679	Tyrosinase	364	378	21.33
3454	NALHIYMNGTMSQVQ	DRB1*15:01	P14679	Tyrosinase	364	378	23.69
3455	NALHIYMNGTMSQVQ	DRB1*15:06	P14679	Tyrosinase	364	378	23.69
3456	NAPPAYEKLSAEQSP	DRB1*10:01	Q16655	MELAN_A	99	113	46.48
3457	NARVRIAYPSLREAA	DRB1*01:18	P43358	MAGE4	295	309	49.78
3458	NARVRIAYPSLREAA	DRB1*01:20	P43358	MAGE4	295	309	30.32
3459	NARVRIAYPSLREAA	DRB1*03:11	P43358	MAGE4	295	309	38.19
3460	NARVRIAYPSLREAA	DRB1*11:02	P43358	MAGE4	295	309	36.41
3461	NARVRIAYPSLREAA	DRB1*11:13	P43358	MAGE4	295	309	40.95
3462	NARVRIAYPSLREAA	DRB1*11:42	P43358	MAGE4	295	309	29.59
3463	NARVRIAYPSLREAA	DRB1*11:65	P43358	MAGE4	295	309	36.41
3464	NARVRIAYPSLREAA	DRB1*13:01	P43358	MAGE4	295	309	36.41
3465	NARVRIAYPSLREAA	DRB1*14:06	P43358	MAGE4	295	309	42.99
3466	NASFSIALNFPGSQK	DRB1*01:01	P40967	PMEL17	81	95	9.16
3467	NASFSIALNFPGSQK	DRB1*01:11	P40967	PMEL17	81	95	30.61
3468	NASFSIALNFPGSQK	DRB1*01:18	P40967	PMEL17	81	95	9.44
3469	NASFSIALNFPGSQK	DRB1*01:20	P40967	PMEL17	81	95	23.03
3470	NASFSIALNFPGSQK	DRB1*01:24	P40967	PMEL17	81	95	22.02
3471	NASFSIALNFPGSQK	DRB1*01:29	P40967	PMEL17	81	95	20.38
3472	NASFSIALNFPGSQK	DRB1*04:01	P40967	PMEL17	81	95	14.94
3473	NASFSIALNFPGSQK	DRB1*04:04	P40967	PMEL17	81	95	31.52
3474	NASFSIALNFPGSQK	DRB1*04:05	P40967	PMEL17	81	95	31.1
3475	NASFSIALNFPGSQK	DRB1*04:08	P40967	PMEL17	81	95	15.55
3476	NASFSIALNFPGSQK	DRB1*04:72	P40967	PMEL17	81	95	45.1

3477	NASFSIALNFPGSQK	DRB1*09:01	P40967	PMEL17	81	95	42.97
3478	NASFSIALNFPGSQK	DRB1*10:01	P40967	PMEL17	81	95	11.5
3479	NASFSIALNFPGSQK	DRB1*11:01	P40967	PMEL17	81	95	33.06
3480	NASFSIALNFPGSQK	DRB1*11:08	P40967	PMEL17	81	95	42.89
3481	NASFSIALNFPGSQK	DRB1*11:10	P40967	PMEL17	81	95	33.06
3482	NASFSIALNFPGSQK	DRB1*11:12	P40967	PMEL17	81	95	33.06
3483	NASFSIALNFPGSQK	DRB1*11:14	P40967	PMEL17	81	95	49.03
3484	NASFSIALNFPGSQK	DRB1*11:28	P40967	PMEL17	81	95	33.06
3485	NASFSIALNFPGSQK	DRB1*11:29	P40967	PMEL17	81	95	33.06
3486	NASFSIALNFPGSQK	DRB1*11:49	P40967	PMEL17	81	95	33.06
3487	NASFSIALNFPGSQK	DRB1*11:62	P40967	PMEL17	81	95	33.06
3488	NASFSIALNFPGSQK	DRB1*11:74	P40967	PMEL17	81	95	33.06
3489	NASFSIALNFPGSQK	DRB1*13:02	P40967	PMEL17	81	95	49.03
3490	NASFSIALNFPGSQK	DRB1*13:05	P40967	PMEL17	81	95	33.06
3491	NASFSIALNFPGSQK	DRB1*13:14	P40967	PMEL17	81	95	33.06
3492	NASFSIALNFPGSQK	DRB1*13:23	P40967	PMEL17	81	95	49.03
3493	NASFSIALNFPGSQK	DRB1*13:50	P40967	PMEL17	81	95	33.06
3494	NASFSIALNFPGSQK	DRB1*13:97	P40967	PMEL17	81	95	49.03
3495	NASFSIALNFPGSQK	DRB1*15:15	P40967	PMEL17	81	95	33.78
3496	NASFSIALNFPGSQK	DRB1*16:01	P40967	PMEL17	81	95	27.36
3497	NASFSIALNFPGSQK	DRB1*16:02	P40967	PMEL17	81	95	24.09
3498	NASFSIALNFPGSQK	DRB1*16:09	P40967	PMEL17	81	95	32.26
3499	NDGPTLIGANASFSI	DRB1*11:14	P40967	PMEL17	72	86	44.44
3500	NDGPTLIGANASFSI	DRB1*13:02	P40967	PMEL17	72	86	44.44
3501	NDGPTLIGANASFSI	DRB1*13:23	P40967	PMEL17	72	86	44.44
3502	NDGPTLIGANASFSI	DRB1*13:97	P40967	PMEL17	72	86	44.44
3503	NDPIFLLHHAFVDSI	DPB1*02:01	P14679	Tyrosinase	382	396	26.27
3504	NDPIFLLHHAFVDSI	DPB1*02:02	P14679	Tyrosinase	382	396	27.56
3505	NDPIFLLHHAFVDSI	DPB1*33:01	P14679	Tyrosinase	382	396	13.88
3506	NDPIFLLHHAFVDSI	DPB1*46:01	P14679	Tyrosinase	382	396	26.27
3507	NDPIFLLHHAFVDSI	DPB1*47:01	P14679	Tyrosinase	382	396	27.56
3508	NDPIFLLHHAFVDSI	DPB1*71:01	P14679	Tyrosinase	382	396	13.88
3509	NDPIFLLHHAFVDSI	DPB1*81:01	P14679	Tyrosinase	382	396	26.27
3510	NDPIFLLHHAFVDSI	DRB1*01:18	P14679	Tyrosinase	382	396	30.61
3511	NDPIFLLHHAFVDSI	DRB1*07:01	P14679	Tyrosinase	382	396	46.64
3512	NDPIFLLHHAFVDSI	DRB1*10:01	P14679	Tyrosinase	382	396	47.07
3513	NDPIFLLHHAFVDSI	DRB1*14:32	P14679	Tyrosinase	382	396	49.29
3514	NDPIFVLLHTFTDAV	DPB1*33:01	P17643	TRP1	396	410	37.71
3515	NDPIFVLLHTFTDAV	DPB1*71:01	P17643	TRP1	396	410	37.71
3516	NDPIFVLLHTFTDAV	DRB1*01:01	P17643	TRP1	396	410	33.42

3517	NDPIFVLLHTFTDAV	DRB1*01:18	P17643	TRP1	396	410	26.39
3518	NDPIFVLLHTFTDAV	DRB1*04:05	P17643	TRP1	396	410	15.01
3519	NDPIFVLLHTFTDAV	DRB1*04:08	P17643	TRP1	396	410	36.48
3520	NDPIFVLLHTFTDAV	DRB1*04:10	P17643	TRP1	396	410	46.41
3521	NDPIFVLLHTFTDAV	DRB1*10:01	P17643	TRP1	396	410	14.63
3522	NGYRALMDKSLHVGT	DRB1*01:01	Q16655	MELAN_A	52	66	26.36
3523	NGYRALMDKSLHVGT	DRB1*01:18	Q16655	MELAN_A	52	66	23.03
3524	NGYRALMDKSLHVGT	DRB1*03:11	Q16655	MELAN_A	52	66	49.33
3525	NHFVRALDMAKRTTH	DRB1*11:42	P17643	TRP1	142	156	41.14
3526	NILLSNAPLGPQFPF	DRB1*01:01	P14679	Tyrosinase	57	71	49.45
3527	NILLSNAPLGPQFPF	DRB1*01:18	P14679	Tyrosinase	57	71	45.9
3528	NILLSNAPLGPQFPF	DRB1*01:20	P14679	Tyrosinase	57	71	37.47
3529	NILLSNAPLGPQFPF	DRB1*11:14	P14679	Tyrosinase	57	71	47.26
3530	NILLSNAPLGPQFPF	DRB1*13:02	P14679	Tyrosinase	57	71	47.26
3531	NILLSNAPLGPQFPF	DRB1*13:23	P14679	Tyrosinase	57	71	47.26
3532	NILLSNAPLGPQFPF	DRB1*13:97	P14679	Tyrosinase	57	71	47.26
3533	NILTIRLTAADHRQL	DRB1*01:20	P78358	NY-ESO-1	131	145	31.62
3534	NILTIRLTAADHRQL	DRB1*11:02	P78358	NY-ESO-1	131	145	45.24
3535	NILTIRLTAADHRQL	DRB1*11:13	P78358	NY-ESO-1	131	145	40.62
3536	NILTIRLTAADHRQL	DRB1*11:42	P78358	NY-ESO-1	131	145	36.81
3537	NILTIRLTAADHRQL	DRB1*11:65	P78358	NY-ESO-1	131	145	45.24
3538	NILTIRLTAADHRQL	DRB1*13:01	P78358	NY-ESO-1	131	145	45.24
3539	NILTIRLTAADHRQL	DRB1*14:06	P78358	NY-ESO-1	131	145	48.71
3540	NIYDLFVWMHYYVSM	DPB1*33:01	P14679	Tyrosinase	171	185	21.92
3541	NIYDLFVWMHYYVSM	DPB1*71:01	P14679	Tyrosinase	171	185	21.92
3542	NKHFLRNQPLTFALQ	DRB1*01:01	P40967	PMEL17	229	243	17.34
3543	NKHFLRNQPLTFALQ	DRB1*01:11	P40967	PMEL17	229	243	27.95
3544	NKHFLRNQPLTFALQ	DRB1*01:18	P40967	PMEL17	229	243	13.46
3545	NKHFLRNQPLTFALQ	DRB1*01:20	P40967	PMEL17	229	243	23.92
3546	NKHFLRNQPLTFALQ	DRB1*01:24	P40967	PMEL17	229	243	43.04
3547	NKHFLRNQPLTFALQ	DRB1*01:29	P40967	PMEL17	229	243	37.1
3548	NKHFLRNQPLTFALQ	DRB1*03:11	P40967	PMEL17	229	243	47.31
3549	NKHFLRNQPLTFALQ	DRB1*07:01	P40967	PMEL17	229	243	41.14
3550	NKHFLRNQPLTFALQ	DRB1*10:01	P40967	PMEL17	229	243	35.72
3551	NKHFLRNQPLTFALQ	DRB1*11:02	P40967	PMEL17	229	243	41.83
3552	NKHFLRNQPLTFALQ	DRB1*11:08	P40967	PMEL17	229	243	28.13
3553	NKHFLRNQPLTFALQ	DRB1*11:13	P40967	PMEL17	229	243	44.97
3554	NKHFLRNQPLTFALQ	DRB1*11:14	P40967	PMEL17	229	243	8.69
3555	NKHFLRNQPLTFALQ	DRB1*11:19	P40967	PMEL17	229	243	38.52
3556	NKHFLRNQPLTFALQ	DRB1*11:42	P40967	PMEL17	229	243	48.81

3557	NKHFLRNQPLTFALQ	DRB1*11:65	P40967	PMEL17	229	243	41.83
3558	NKHFLRNQPLTFALQ	DRB1*13:01	P40967	PMEL17	229	243	41.83
3559	NKHFLRNQPLTFALQ	DRB1*13:02	P40967	PMEL17	229	243	8.69
3560	NKHFLRNQPLTFALQ	DRB1*13:23	P40967	PMEL17	229	243	8.69
3561	NKHFLRNQPLTFALQ	DRB1*13:61	P40967	PMEL17	229	243	48.63
3562	NKHFLRNQPLTFALQ	DRB1*13:66	P40967	PMEL17	229	243	29.47
3563	NKHFLRNQPLTFALQ	DRB1*13:96	P40967	PMEL17	229	243	11.5
3564	NKHFLRNQPLTFALQ	DRB1*13:97	P40967	PMEL17	229	243	8.69
3565	NKHFLRNQPLTFALQ	DRB1*14:02	P40967	PMEL17	229	243	23.05
3566	NKHFLRNQPLTFALQ	DRB1*14:06	P40967	PMEL17	229	243	46.86
3567	NKHFLRNQPLTFALQ	DRB1*14:07	P40967	PMEL17	229	243	45.3
3568	NKHFLRNQPLTFALQ	DRB1*14:32	P40967	PMEL17	229	243	42.81
3569	NLLSPASFFSSWQIV	DPB1*33:01	P14679	Tyrosinase	261	275	48.78
3570	NLLSPASFFSSWQIV	DPB1*71:01	P14679	Tyrosinase	261	275	48.78
3571	NNQIFPKTGLLIIVL	DRB1*01:01	P43358	MAGE4	193	207	37
3572	NNQIFPKTGLLIIVL	DRB1*01:18	P43358	MAGE4	193	207	24.71
3573	NNQIFPKTGLLIIVL	DRB1*01:20	P43358	MAGE4	193	207	25.4
3574	NPARYEFLWGPRALA	DRB1*01:01	P43358	MAGE4	266	280	7.86
3575	NPARYEFLWGPRALA	DRB1*01:11	P43358	MAGE4	266	280	25.76
3576	NPARYEFLWGPRALA	DRB1*01:18	P43358	MAGE4	266	280	7.84
3577	NPARYEFLWGPRALA	DRB1*01:20	P43358	MAGE4	266	280	25.1
3578	NPARYEFLWGPRALA	DRB1*01:24	P43358	MAGE4	266	280	18.56
3579	NPARYEFLWGPRALA	DRB1*01:29	P43358	MAGE4	266	280	16.85
3580	NPARYEFLWGPRALA	DRB1*10:01	P43358	MAGE4	266	280	18.33
3581	NPARYEFLWGPRALA	DRB1*15:15	P43358	MAGE4	266	280	39.35
3582	NPARYEFLWGPRALA	DRB1*16:01	P43358	MAGE4	266	280	39.27
3583	NPARYEFLWGPRALA	DRB1*16:02	P43358	MAGE4	266	280	41.06
3584	NPARYEFLWGPRALA	DRB1*16:09	P43358	MAGE4	266	280	36.73
3585	NQDWLGVSRQLRTKA	DRB1*11:03	P40967	PMEL17	29	43	48.39
3586	NQDWLGVSRQLRTKA	DRB1*11:42	P40967	PMEL17	29	43	42.49
3587	NQIFPKTGLLIIVLG	DRB1*01:01	P43358	MAGE4	194	208	48.87
3588	NQIFPKTGLLIIVLG	DRB1*01:18	P43358	MAGE4	194	208	32.27
3589	NQIFPKTGLLIIVLG	DRB1*01:20	P43358	MAGE4	194	208	31.9
3590	NQIMPKTGFLIIVLV	DRB1*01:01	P43355	MAGE1	186	200	38.15
3591	NQIMPKTGFLIIVLV	DRB1*01:18	P43355	MAGE1	186	200	26.87
3592	NQIMPKTGFLIIVLV	DRB1*01:20	P43355	MAGE1	186	200	24.19
3593	NQIMPKTGFLIIVLV	DRB1*15:01	P43355	MAGE1	186	200	49.46
3594	NQIMPKTGFLIIVLV	DRB1*15:06	P43355	MAGE1	186	200	49.46
3595	NRESYMVPFIPLYRN	DPB1*02:01	P14679	Tyrosinase	421	435	43.82
3596	NRESYMVPFIPLYRN	DPB1*02:02	P14679	Tyrosinase	421	435	41.71

3597	NRESYMVPFIPLYRN	DPB1*04:01	P14679	Tyrosinase	421	435	49.59
3598	NRESYMVPFIPLYRN	DPB1*126:01	P14679	Tyrosinase	421	435	49.59
3599	NRESYMVPFIPLYRN	DPB1*15:01	P14679	Tyrosinase	421	435	35.48
3600	NRESYMVPFIPLYRN	DPB1*23:01	P14679	Tyrosinase	421	435	49.59
3601	NRESYMVPFIPLYRN	DPB1*33:01	P14679	Tyrosinase	421	435	17.37
3602	NRESYMVPFIPLYRN	DPB1*39:01	P14679	Tyrosinase	421	435	49.59
3603	NRESYMVPFIPLYRN	DPB1*46:01	P14679	Tyrosinase	421	435	43.82
3604	NRESYMVPFIPLYRN	DPB1*47:01	P14679	Tyrosinase	421	435	41.71
3605	NRESYMVPFIPLYRN	DPB1*71:01	P14679	Tyrosinase	421	435	17.37
3606	NRESYMVPFIPLYRN	DPB1*72:01	P14679	Tyrosinase	421	435	40.81
3607	NRESYMVPFIPLYRN	DPB1*81:01	P14679	Tyrosinase	421	435	43.82
3608	NRESYMVPFIPLYRN	DRB1*01:20	P14679	Tyrosinase	421	435	48.82
3609	NRESYMVPFIPLYRN	DRB1*11:02	P14679	Tyrosinase	421	435	38.22
3610	NRESYMVPFIPLYRN	DRB1*11:42	P14679	Tyrosinase	421	435	49.18
3611	NRESYMVPFIPLYRN	DRB1*11:65	P14679	Tyrosinase	421	435	38.22
3612	NRESYMVPFIPLYRN	DRB1*13:01	P14679	Tyrosinase	421	435	38.22
3613	NTEMFVTAPDNLGYT	DRB1*04:01	P17643	TRP1	449	463	28.47
3614	NTEMFVTAPDNLGYT	DRB1*04:08	P17643	TRP1	449	463	30.41
3615	NYFVWTHYYSVKKTF	DPB1*01:01	P17643	TRP1	186	200	23.72
3616	NYFVWTHYYSVKKTF	DPB1*02:01	P17643	TRP1	186	200	5.5
3617	NYFVWTHYYSVKKTF	DPB1*02:02	P17643	TRP1	186	200	6.63
3618	NYFVWTHYYSVKKTF	DPB1*04:01	P17643	TRP1	186	200	6.23
3619	NYFVWTHYYSVKKTF	DPB1*04:02	P17643	TRP1	186	200	21.07
3620	NYFVWTHYYSVKKTF	DPB1*105:01	P17643	TRP1	186	200	21.07
3621	NYFVWTHYYSVKKTF	DPB1*126:01	P17643	TRP1	186	200	6.23
3622	NYFVWTHYYSVKKTF	DPB1*15:01	P17643	TRP1	186	200	20.85
3623	NYFVWTHYYSVKKTF	DPB1*16:01	P17643	TRP1	186	200	18.61
3624	NYFVWTHYYSVKKTF	DPB1*19:01	P17643	TRP1	186	200	37.68
3625	NYFVWTHYYSVKKTF	DPB1*23:01	P17643	TRP1	186	200	6.23
3626	NYFVWTHYYSVKKTF	DPB1*33:01	P17643	TRP1	186	200	4.16
3627	NYFVWTHYYSVKKTF	DPB1*34:01	P17643	TRP1	186	200	25.7
3628	NYFVWTHYYSVKKTF	DPB1*39:01	P17643	TRP1	186	200	6.23
3629	NYFVWTHYYSVKKTF	DPB1*40:01	P17643	TRP1	186	200	25.18
3630	NYFVWTHYYSVKKTF	DPB1*41:01	P17643	TRP1	186	200	14.41
3631	NYFVWTHYYSVKKTF	DPB1*46:01	P17643	TRP1	186	200	5.5
3632	NYFVWTHYYSVKKTF	DPB1*47:01	P17643	TRP1	186	200	6.63
3633	NYFVWTHYYSVKKTF	DPB1*49:01	P17643	TRP1	186	200	21.07
3634	NYFVWTHYYSVKKTF	DPB1*71:01	P17643	TRP1	186	200	4.16
3635	NYFVWTHYYSVKKTF	DPB1*72:01	P17643	TRP1	186	200	6.93
3636	NYFVWTHYYSVKKTF	DPB1*81:01	P17643	TRP1	186	200	5.5

3637	NYFVWTHYYSVKKTF	DRB1*01:18	P17643	TRP1	186	200	40.54
3638	NYFVWTHYYSVKKTF	DRB1*07:01	P17643	TRP1	186	200	28.79
3639	NYFVWTHYYSVKKTF	DRB1*15:02	P17643	TRP1	186	200	49.12
3640	NYFVWTHYYSVKKTF	DRB1*15:15	P17643	TRP1	186	200	30.43
3641	NYFVWTHYYSVKKTF	DRB1*16:09	P17643	TRP1	186	200	49.93
3642	PACQLVLHQILKGGS	DRB1*11:04	P40967	PMEL17	548	562	36.62
3643	PACQLVLHQILKGGS	DRB1*11:42	P40967	PMEL17	548	562	43.91
3644	PACQLVLHQILKGGS	DRB1*11:46	P40967	PMEL17	548	562	36.62
3645	PACQLVLHQILKGGS	DRB1*11:58	P40967	PMEL17	548	562	36.62
3646	PACQLVLHQILKGGS	DRB1*13:11	P40967	PMEL17	548	562	36.62
3647	PAEVSIVVLSGTTAA	DRB1*01:01	P40967	PMEL17	402	416	12.54
3648	PAEVSIVVLSGTTAA	DRB1*01:11	P40967	PMEL17	402	416	32.83
3649	PAEVSIVVLSGTTAA	DRB1*01:18	P40967	PMEL17	402	416	12.12
3650	PAEVSIVVLSGTTAA	DRB1*01:20	P40967	PMEL17	402	416	13.64
3651	PAEVSIVVLSGTTAA	DRB1*01:24	P40967	PMEL17	402	416	25.74
3652	PAEVSIVVLSGTTAA	DRB1*01:29	P40967	PMEL17	402	416	29.81
3653	PAFLTWHRYHLLRLE	DPB1*02:01	P17643	TRP1	218	232	14.24
3654	PAFLTWHRYHLLRLE	DPB1*02:02	P17643	TRP1	218	232	13.5
3655	PAFLTWHRYHLLRLE	DPB1*16:01	P17643	TRP1	218	232	44.78
3656	PAFLTWHRYHLLRLE	DPB1*33:01	P17643	TRP1	218	232	6.92
3657	PAFLTWHRYHLLRLE	DPB1*41:01	P17643	TRP1	218	232	32.84
3658	PAFLTWHRYHLLRLE	DPB1*46:01	P17643	TRP1	218	232	14.24
3659	PAFLTWHRYHLLRLE	DPB1*47:01	P17643	TRP1	218	232	13.5
3660	PAFLTWHRYHLLRLE	DPB1*71:01	P17643	TRP1	218	232	6.92
3661	PAFLTWHRYHLLRLE	DPB1*81:01	P17643	TRP1	218	232	14.24
3662	PAFLTWHRYHLLRLE	DRB1*11:02	P17643	TRP1	218	232	38.5
3663	PAFLTWHRYHLLRLE	DRB1*11:03	P17643	TRP1	218	232	39.35
3664	PAFLTWHRYHLLRLE	DRB1*11:65	P17643	TRP1	218	232	38.5
3665	PAFLTWHRYHLLRLE	DRB1*13:01	P17643	TRP1	218	232	38.5
3666	PAFLTWHRYHLLRLE	DRB1*15:03	P17643	TRP1	218	232	48.2
3667	PAFLTWHRYHLLRLE	DRB1*15:15	P17643	TRP1	218	232	43.95
3668	PARYEFLWGPRALAE	DRB1*01:01	P43355	MAGE1	259	273	8.83
3669	PARYEFLWGPRALAE	DRB1*01:01	P43358	MAGE4	267	281	8.83
3670	PARYEFLWGPRALAE	DRB1*01:11	P43355	MAGE1	259	273	27.46
3671	PARYEFLWGPRALAE	DRB1*01:11	P43358	MAGE4	267	281	27.46
3672	PARYEFLWGPRALAE	DRB1*01:18	P43355	MAGE1	259	273	8.62
3673	PARYEFLWGPRALAE	DRB1*01:18	P43358	MAGE4	267	281	8.62
3674	PARYEFLWGPRALAE	DRB1*01:20	P43355	MAGE1	259	273	26.24
3675	PARYEFLWGPRALAE	DRB1*01:20	P43358	MAGE4	267	281	26.24
3676	PARYEFLWGPRALAE	DRB1*01:24	P43355	MAGE1	259	273	20.02

3677	PARYEFLWGPRALAE	DRB1*01:24	P43358	MAGE4	267	281	20.02
3678	PARYEFLWGPRALAE	DRB1*01:29	P43355	MAGE1	259	273	17.19
3679	PARYEFLWGPRALAE	DRB1*01:29	P43358	MAGE4	267	281	17.19
3680	PARYEFLWGPRALAE	DRB1*10:01	P43355	MAGE1	259	273	19.45
3681	PARYEFLWGPRALAE	DRB1*10:01	P43358	MAGE4	267	281	19.45
3682	PARYEFLWGPRALAE	DRB1*11:01	P43355	MAGE1	259	273	37.79
3683	PARYEFLWGPRALAE	DRB1*11:01	P43358	MAGE4	267	281	37.79
3684	PARYEFLWGPRALAE	DRB1*11:10	P43355	MAGE1	259	273	37.79
3685	PARYEFLWGPRALAE	DRB1*11:10	P43358	MAGE4	267	281	37.79
3686	PARYEFLWGPRALAE	DRB1*11:12	P43355	MAGE1	259	273	37.79
3687	PARYEFLWGPRALAE	DRB1*11:12	P43358	MAGE4	267	281	37.79
3688	PARYEFLWGPRALAE	DRB1*11:28	P43355	MAGE1	259	273	37.79
3689	PARYEFLWGPRALAE	DRB1*11:28	P43358	MAGE4	267	281	37.79
3690	PARYEFLWGPRALAE	DRB1*11:29	P43355	MAGE1	259	273	37.79
3691	PARYEFLWGPRALAE	DRB1*11:29	P43358	MAGE4	267	281	37.79
3692	PARYEFLWGPRALAE	DRB1*11:49	P43355	MAGE1	259	273	37.79
3693	PARYEFLWGPRALAE	DRB1*11:49	P43358	MAGE4	267	281	37.79
3694	PARYEFLWGPRALAE	DRB1*11:62	P43355	MAGE1	259	273	37.79
3695	PARYEFLWGPRALAE	DRB1*11:62	P43358	MAGE4	267	281	37.79
3696	PARYEFLWGPRALAE	DRB1*11:74	P43355	MAGE1	259	273	37.79
3697	PARYEFLWGPRALAE	DRB1*11:74	P43358	MAGE4	267	281	37.79
3698	PARYEFLWGPRALAE	DRB1*13:05	P43355	MAGE1	259	273	37.79
3699	PARYEFLWGPRALAE	DRB1*13:05	P43358	MAGE4	267	281	37.79
3700	PARYEFLWGPRALAE	DRB1*13:14	P43355	MAGE1	259	273	37.79
3701	PARYEFLWGPRALAE	DRB1*13:14	P43358	MAGE4	267	281	37.79
3702	PARYEFLWGPRALAE	DRB1*13:21	P43355	MAGE1	259	273	45.62
3703	PARYEFLWGPRALAE	DRB1*13:21	P43358	MAGE4	267	281	45.62
3704	PARYEFLWGPRALAE	DRB1*13:50	P43355	MAGE1	259	273	37.79
3705	PARYEFLWGPRALAE	DRB1*13:50	P43358	MAGE4	267	281	37.79
3706	PARYEFLWGPRALAE	DRB1*15:15	P43355	MAGE1	259	273	43.71
3707	PARYEFLWGPRALAE	DRB1*15:15	P43358	MAGE4	267	281	43.71
3708	PARYEFLWGPRALAE	DRB1*16:01	P43355	MAGE1	259	273	40.85
3709	PARYEFLWGPRALAE	DRB1*16:01	P43358	MAGE4	267	281	40.85
3710	PARYEFLWGPRALAE	DRB1*16:02	P43355	MAGE1	259	273	45.18
3711	PARYEFLWGPRALAE	DRB1*16:02	P43358	MAGE4	267	281	45.18
3712	PARYEFLWGPRALAE	DRB1*16:09	P43355	MAGE1	259	273	38.73
3713	PARYEFLWGPRALAE	DRB1*16:09	P43358	MAGE4	267	281	38.73
3714	PAVRSLHNLAHLFLN	DRB1*01:01	P17643	TRP1	371	385	44.3
3715	PAVRSLHNLAHLFLN	DRB1*01:18	P17643	TRP1	371	385	29.2
3716	PAVRSLHNLAHLFLN	DRB1*01:20	P17643	TRP1	371	385	22.26

3717	PAVRSLHNLAHLFLN	DRB1*04:04	P17643	TRP1	371	385	25.72
3718	PAVRSLHNLAHLFLN	DRB1*04:10	P17643	TRP1	371	385	44.46
3719	PAVRSLHNLAHLFLN	DRB1*10:01	P17643	TRP1	371	385	37
3720	PAVRSLHNLAHLFLN	DRB1*11:42	P17643	TRP1	371	385	36.94
3721	PDAESLFREALSNKV	DRB1*01:01	P43358	MAGE4	100	114	9.96
3722	PDAESLFREALSNKV	DRB1*01:11	P43358	MAGE4	100	114	25.78
3723	PDAESLFREALSNKV	DRB1*01:18	P43358	MAGE4	100	114	11.27
3724	PDAESLFREALSNKV	DRB1*01:20	P43358	MAGE4	100	114	44.71
3725	PDAESLFREALSNKV	DRB1*01:24	P43358	MAGE4	100	114	25.31
3726	PDAESLFREALSNKV	DRB1*01:29	P43358	MAGE4	100	114	22.68
3727	PDAESLFREALSNKV	DRB1*07:01	P43358	MAGE4	100	114	6.24
3728	PDAESLFREALSNKV	DRB1*09:01	P43358	MAGE4	100	114	11.19
3729	PDAESLFREALSNKV	DRB1*10:01	P43358	MAGE4	100	114	28.06
3730	PDAESLFREALSNKV	DRB1*16:02	P43358	MAGE4	100	114	40.04
3731	PDGQVIWVNNTIING	DRB1*11:14	P40967	PMEL17	98	112	19.68
3732	PDGQVIWVNNTIING	DRB1*13:02	P40967	PMEL17	98	112	19.68
3733	PDGQVIWVNNTIING	DRB1*13:23	P40967	PMEL17	98	112	19.68
3734	PDGQVIWVNNTIING	DRB1*13:96	P40967	PMEL17	98	112	30.82
3735	PDGQVIWVNNTIING	DRB1*13:97	P40967	PMEL17	98	112	19.68
3736	PEKDKFFAYLTLAKH	DPB1*02:02	P14679	Tyrosinase	129	143	45.23
3737	PEKDKFFAYLTLAKH	DPB1*33:01	P14679	Tyrosinase	129	143	22.14
3738	PEKDKFFAYLTLAKH	DPB1*47:01	P14679	Tyrosinase	129	143	45.23
3739	PEKDKFFAYLTLAKH	DPB1*71:01	P14679	Tyrosinase	129	143	22.14
3740	PEKDKFFAYLTLAKH	DRB1*01:01	P14679	Tyrosinase	129	143	40.52
3741	PEKDKFFAYLTLAKH	DRB1*01:18	P14679	Tyrosinase	129	143	29.2
3742	PEKDKFFAYLTLAKH	DRB1*10:01	P14679	Tyrosinase	129	143	16.55
3743	PEKDKFFAYLTLAKH	DRB1*15:15	P14679	Tyrosinase	129	143	34.63
3744	PEKDKFFAYLTLAKH	DRB1*16:09	P14679	Tyrosinase	129	143	44.53
3745	PESRLLEFYLAMPFA	DPB1*01:01	P78358	NY-ESO-1	83	97	43.22
3746	PESRLLEFYLAMPFA	DPB1*02:02	P78358	NY-ESO-1	83	97	48.33
3747	PESRLLEFYLAMPFA	DPB1*15:01	P78358	NY-ESO-1	83	97	33.96
3748	PESRLLEFYLAMPFA	DPB1*33:01	P78358	NY-ESO-1	83	97	22.17
3749	PESRLLEFYLAMPFA	DPB1*47:01	P78358	NY-ESO-1	83	97	48.33
3750	PESRLLEFYLAMPFA	DPB1*71:01	P78358	NY-ESO-1	83	97	22.17
3751	PESRLLEFYLAMPFA	DRB1*15:01	P78358	NY-ESO-1	83	97	43.74
3752	PESRLLEFYLAMPFA	DRB1*15:06	P78358	NY-ESO-1	83	97	43.74
3753	PFIPLYRNGDFFISS	DRB1*13:21	P14679	Tyrosinase	428	442	19.53
3754	PFSVSVSQLRALDGG	DRB1*01:20	P40967	PMEL17	214	228	44.28
3755	PFYSNSTNSFRNTVE	DRB1*11:14	P17643	TRP1	346	360	39.62
3756	PFYSNSTNSFRNTVE	DRB1*13:02	P17643	TRP1	346	360	39.62

3757	PFYSNSTNSFRNTVE	DRB1*13:23	P17643	TRP1	346	360	39.62
3758	PFYSNSTNSFRNTVE	DRB1*13:97	P17643	TRP1	346	360	39.62
3759	PIFLLHHAFVDSIFE	DPB1*02:01	P14679	Tyrosinase	384	398	20.9
3760	PIFLLHHAFVDSIFE	DPB1*02:02	P14679	Tyrosinase	384	398	21.63
3761	PIFLLHHAFVDSIFE	DPB1*33:01	P14679	Tyrosinase	384	398	12
3762	PIFLLHHAFVDSIFE	DPB1*46:01	P14679	Tyrosinase	384	398	20.9
3763	PIFLLHHAFVDSIFE	DPB1*47:01	P14679	Tyrosinase	384	398	21.63
3764	PIFLLHHAFVDSIFE	DPB1*71:01	P14679	Tyrosinase	384	398	12
3765	PIFLLHHAFVDSIFE	DPB1*81:01	P14679	Tyrosinase	384	398	20.9
3766	PIFLLHHAFVDSIFE	DRB1*01:01	P14679	Tyrosinase	384	398	47.49
3767	PIFLLHHAFVDSIFE	DRB1*01:18	P14679	Tyrosinase	384	398	32.2
3768	PIFLLHHAFVDSIFE	DRB1*07:01	P14679	Tyrosinase	384	398	41.9
3769	PIFLLHHAFVDSIFE	DRB1*09:01	P14679	Tyrosinase	384	398	47.97
3770	PIFLLHHAFVDSIFE	DRB1*10:01	P14679	Tyrosinase	384	398	39.16
3771	PIFVLLHTFTDAVFD	DPB1*33:01	P17643	TRP1	398	412	30.61
3772	PIFVLLHTFTDAVFD	DPB1*71:01	P17643	TRP1	398	412	30.61
3773	PIFVLLHTFTDAVFD	DRB1*01:01	P17643	TRP1	398	412	38.84
3774	PIFVLLHTFTDAVFD	DRB1*01:18	P17643	TRP1	398	412	27
3775	PIFVLLHTFTDAVFD	DRB1*04:05	P17643	TRP1	398	412	20.52
3776	PIFVLLHTFTDAVFD	DRB1*04:08	P17643	TRP1	398	412	38.22
3777	PIFVLLHTFTDAVFD	DRB1*04:10	P17643	TRP1	398	412	49.8
3778	PIFVLLHTFTDAVFD	DRB1*10:01	P17643	TRP1	398	412	19.59
3779	PIRRNPAGNVARPMV	DRB1*11:14	P17643	TRP1	310	324	43.15
3780	PIRRNPAGNVARPMV	DRB1*13:02	P17643	TRP1	310	324	43.15
3781	PIRRNPAGNVARPMV	DRB1*13:23	P17643	TRP1	310	324	43.15
3782	PIRRNPAGNVARPMV	DRB1*13:97	P17643	TRP1	310	324	43.15
3783	PLDCVLYRYGSFSVT	DPB1*33:01	P40967	PMEL17	472	486	48.96
3784	PLDCVLYRYGSFSVT	DPB1*71:01	P40967	PMEL17	472	486	48.96
3785	PLDCVLYRYGSFSVT	DRB1*15:01	P40967	PMEL17	472	486	22.75
3786	PLDCVLYRYGSFSVT	DRB1*15:06	P40967	PMEL17	472	486	22.75
3787	PLDCVLYRYGSFSVT	DRB1*15:07	P40967	PMEL17	472	486	49.89
3788	PLFVIATRRSEEILG	DRB1*01:18	P17643	TRP1	157	171	38.62
3789	PLFVIATRRSEEILG	DRB1*01:20	P17643	TRP1	157	171	29.52
3790	PLFVIATRRSEEILG	DRB1*04:04	P17643	TRP1	157	171	46.17
3791	PLFVIATRRSEEILG	DRB1*08:01	P17643	TRP1	157	171	32.59
3792	PLFVIATRRSEEILG	DRB1*08:04	P17643	TRP1	157	171	36.44
3793	PLFVIATRRSEEILG	DRB1*11:01	P17643	TRP1	157	171	15.42
3794	PLFVIATRRSEEILG	DRB1*11:03	P17643	TRP1	157	171	34.81
3795	PLFVIATRRSEEILG	DRB1*11:04	P17643	TRP1	157	171	16.46
3796	PLFVIATRRSEEILG	DRB1*11:08	P17643	TRP1	157	171	35.15

3797	PLFVIATRRSEEILG	DRB1*11:10	P17643	TRP1	157	171	15.42
3798	PLFVIATRRSEEILG	DRB1*11:12	P17643	TRP1	157	171	15.42
3799	PLFVIATRRSEEILG	DRB1*11:13	P17643	TRP1	157	171	46.62
3800	PLFVIATRRSEEILG	DRB1*11:28	P17643	TRP1	157	171	15.42
3801	PLFVIATRRSEEILG	DRB1*11:29	P17643	TRP1	157	171	15.42
3802	PLFVIATRRSEEILG	DRB1*11:37	P17643	TRP1	157	171	34.19
3803	PLFVIATRRSEEILG	DRB1*11:42	P17643	TRP1	157	171	22.73
3804	PLFVIATRRSEEILG	DRB1*11:46	P17643	TRP1	157	171	16.46
3805	PLFVIATRRSEEILG	DRB1*11:49	P17643	TRP1	157	171	15.42
3806	PLFVIATRRSEEILG	DRB1*11:58	P17643	TRP1	157	171	16.46
3807	PLFVIATRRSEEILG	DRB1*11:62	P17643	TRP1	157	171	15.42
3808	PLFVIATRRSEEILG	DRB1*11:74	P17643	TRP1	157	171	15.42
3809	PLFVIATRRSEEILG	DRB1*13:05	P17643	TRP1	157	171	15.42
3810	PLFVIATRRSEEILG	DRB1*13:07	P17643	TRP1	157	171	34.19
3811	PLFVIATRRSEEILG	DRB1*13:11	P17643	TRP1	157	171	16.46
3812	PLFVIATRRSEEILG	DRB1*13:14	P17643	TRP1	157	171	15.42
3813	PLFVIATRRSEEILG	DRB1*13:21	P17643	TRP1	157	171	11.53
3814	PLFVIATRRSEEILG	DRB1*13:50	P17643	TRP1	157	171	15.42
3815	PLFVIATRRSEEILG	DRB1*13:66	P17643	TRP1	157	171	46.44
3816	PLLDGTATLRLVKRQ	DRB1*07:01	P40967	PMEL17	456	470	46.94
3817	PLLLFQQARAQFPRQ	DRB1*01:01	P17643	TRP1	15	29	12.28
3818	PLLLFQQARAQFPRQ	DRB1*01:11	P17643	TRP1	15	29	40.46
3819	PLLLFQQARAQFPRQ	DRB1*01:18	P17643	TRP1	15	29	12.64
3820	PLLLFQQARAQFPRQ	DRB1*01:20	P17643	TRP1	15	29	14.56
3821	PLLLFQQARAQFPRQ	DRB1*01:24	P17643	TRP1	15	29	28.91
3822	PLLLFQQARAQFPRQ	DRB1*01:29	P17643	TRP1	15	29	25.99
3823	PLLLFQQARAQFPRQ	DRB1*09:01	P17643	TRP1	15	29	42.18
3824	PLLLFQQARAQFPRQ	DRB1*10:01	P17643	TRP1	15	29	14.83
3825	PLLLFQQARAQFPRQ	DRB1*11:02	P17643	TRP1	15	29	42.53
3826	PLLLFQQARAQFPRQ	DRB1*11:03	P17643	TRP1	15	29	40.46
3827	PLLLFQQARAQFPRQ	DRB1*11:04	P17643	TRP1	15	29	38.91
3828	PLLLFQQARAQFPRQ	DRB1*11:13	P17643	TRP1	15	29	35.94
3829	PLLLFQQARAQFPRQ	DRB1*11:42	P17643	TRP1	15	29	27.38
3830	PLLLFQQARAQFPRQ	DRB1*11:46	P17643	TRP1	15	29	38.91
3831	PLLLFQQARAQFPRQ	DRB1*11:58	P17643	TRP1	15	29	38.91
3832	PLLLFQQARAQFPRQ	DRB1*11:65	P17643	TRP1	15	29	42.53
3833	PLLLFQQARAQFPRQ	DRB1*13:01	P17643	TRP1	15	29	42.53
3834	PLLLFQQARAQFPRQ	DRB1*13:11	P17643	TRP1	15	29	38.91
3835	PLLLFQQARAQFPRQ	DRB1*14:32	P17643	TRP1	15	29	45.65
3836	PNDPIFVLLHTFTDA	DRB1*01:01	P17643	TRP1	395	409	45.75

3837	PNDPIFVLLHTFTDA	DRB1*01:18	P17643	TRP1	395	409	37.91
3838	PNDPIFVLLHTFTDA	DRB1*04:05	P17643	TRP1	395	409	14.77
3839	PNDPIFVLLHTFTDA	DRB1*04:08	P17643	TRP1	395	409	47.45
3840	PNDPIFVLLHTFTDA	DRB1*10:01	P17643	TRP1	395	409	16.67
3841	PPFYSNSTNSFRNTV	DRB1*11:14	P17643	TRP1	345	359	31.34
3842	PPFYSNSTNSFRNTV	DRB1*13:02	P17643	TRP1	345	359	31.34
3843	PPFYSNSTNSFRNTV	DRB1*13:23	P17643	TRP1	345	359	31.34
3844	PPFYSNSTNSFRNTV	DRB1*13:97	P17643	TRP1	345	359	31.34
3845	PQMTFGRLQGISPKI	DRB1*01:01	Q16385	SSX2	97	111	14.4
3846	PQMTFGRLQGISPKI	DRB1*01:18	Q16385	SSX2	97	111	16.33
3847	PQMTFGRLQGISPKI	DRB1*01:20	Q16385	SSX2	97	111	41.41
3848	PQMTFGRLQGISPKI	DRB1*01:24	Q16385	SSX2	97	111	43.72
3849	PQMTFGRLQGISPKI	DRB1*10:01	Q16385	SSX2	97	111	33.03
3850	PRALAETSYVKVLEH	DPB1*33:01	P43358	MAGE4	276	290	24.55
3851	PRALAETSYVKVLEH	DPB1*71:01	P43358	MAGE4	276	290	24.55
3852	PRALAETSYVKVLEY	DPB1*02:02	P43355	MAGE1	268	282	41.63
3853	PRALAETSYVKVLEY	DPB1*33:01	P43355	MAGE1	268	282	18.8
3854	PRALAETSYVKVLEY	DPB1*47:01	P43355	MAGE1	268	282	41.63
3855	PRALAETSYVKVLEY	DPB1*71:01	P43355	MAGE1	268	282	18.8
3856	PSTSCILESLFRAVI	DPB1*02:01	P43355	MAGE1	88	102	25.38
3857	PSTSCILESLFRAVI	DPB1*02:02	P43355	MAGE1	88	102	21.88
3858	PSTSCILESLFRAVI	DPB1*33:01	P43355	MAGE1	88	102	11.82
3859	PSTSCILESLFRAVI	DPB1*46:01	P43355	MAGE1	88	102	25.38
3860	PSTSCILESLFRAVI	DPB1*47:01	P43355	MAGE1	88	102	21.88
3861	PSTSCILESLFRAVI	DPB1*71:01	P43355	MAGE1	88	102	11.82
3862	PSTSCILESLFRAVI	DPB1*81:01	P43355	MAGE1	88	102	25.38
3863	PTLIGANASFSIALN	DRB1*01:01	P40967	PMEL17	75	89	37.54
3864	PTLIGANASFSIALN	DRB1*01:18	P40967	PMEL17	75	89	39.94
3865	PTLIGANASFSIALN	DRB1*01:20	P40967	PMEL17	75	89	28.65
3866	PTLIGANASFSIALN	DRB1*10:01	P40967	PMEL17	75	89	47.77
3867	PTLIGANASFSIALN	DRB1*11:14	P40967	PMEL17	75	89	17.72
3868	PTLIGANASFSIALN	DRB1*13:02	P40967	PMEL17	75	89	17.72
3869	PTLIGANASFSIALN	DRB1*13:23	P40967	PMEL17	75	89	17.72
3870	PTLIGANASFSIALN	DRB1*13:96	P40967	PMEL17	75	89	29.17
3871	PTLIGANASFSIALN	DRB1*13:97	P40967	PMEL17	75	89	17.72
3872	PVIFGKASESLKMIF	DRB1*01:20	P43358	MAGE4	149	163	45.82
3873	PVSGLSIGTGRAMLG	DRB1*01:01	P40967	PMEL17	166	180	36.9
3874	PVSGLSIGTGRAMLG	DRB1*01:18	P40967	PMEL17	166	180	40.44
3875	PVSGLSIGTGRAMLG	DRB1*01:20	P40967	PMEL17	166	180	25.47
3876	PVTAQVVLQAAIPLT	DRB1*01:01	P40967	PMEL17	285	299	7.59

3877	PVTAQVVLQAAIPLT	DRB1*01:02	P40967	PMEL17	285	299	18.24
3878	PVTAQVVLQAAIPLT	DRB1*01:11	P40967	PMEL17	285	299	16.88
3879	PVTAQVVLQAAIPLT	DRB1*01:18	P40967	PMEL17	285	299	7.91
3880	PVTAQVVLQAAIPLT	DRB1*01:20	P40967	PMEL17	285	299	7.04
3881	PVTAQVVLQAAIPLT	DRB1*01:24	P40967	PMEL17	285	299	14.92
3882	PVTAQVVLQAAIPLT	DRB1*01:29	P40967	PMEL17	285	299	17.43
3883	PVTAQVVLQAAIPLT	DRB1*10:01	P40967	PMEL17	285	299	37.7
3884	PVTAQVVLQAAIPLT	DRB1*11:13	P40967	PMEL17	285	299	47.85
3885	PVTAQVVLQAAIPLT	DRB1*11:14	P40967	PMEL17	285	299	24.67
3886	PVTAQVVLQAAIPLT	DRB1*13:02	P40967	PMEL17	285	299	24.67
3887	PVTAQVVLQAAIPLT	DRB1*13:23	P40967	PMEL17	285	299	24.67
3888	PVTAQVVLQAAIPLT	DRB1*13:96	P40967	PMEL17	285	299	47.53
3889	PVTAQVVLQAAIPLT	DRB1*13:97	P40967	PMEL17	285	299	24.67
3890	PVTAQVVLQAAIPLT	DRB1*14:32	P40967	PMEL17	285	299	38.11
3891	PVTAQVVLQAAIPLT	DRB1*15:01	P40967	PMEL17	285	299	45.07
3892	PVTAQVVLQAAIPLT	DRB1*15:06	P40967	PMEL17	285	299	45.07
3893	PWLKVYYYRFVIGLR	DPB1*33:01	O75767	TRP2	203	217	44.82
3894	PWLKVYYYRFVIGLR	DPB1*71:01	O75767	TRP2	203	217	44.82
3895	PYWNFATGKNVCDIC	DRB1*01:01	P17643	TRP1	247	261	42.9
3896	PYWNFATGKNVCDIC	DRB1*01:18	P17643	TRP1	247	261	44.04
3897	QARLMKEESPVVSWR	DRB1*01:20	Q13072	BAGE	16	30	43.33
3898	QASRIWSWLLGAAMV	DRB1*01:01	P14679	Tyrosinase	470	484	13.94
3899	QASRIWSWLLGAAMV	DRB1*01:11	P14679	Tyrosinase	470	484	37.3
3900	QASRIWSWLLGAAMV	DRB1*01:18	P14679	Tyrosinase	470	484	12.37
3901	QASRIWSWLLGAAMV	DRB1*01:20	P14679	Tyrosinase	470	484	40.45
3902	QASRIWSWLLGAAMV	DRB1*01:24	P14679	Tyrosinase	470	484	22.77
3903	QASRIWSWLLGAAMV	DRB1*01:29	P14679	Tyrosinase	470	484	26.82
3904	QASRIWSWLLGAAMV	DRB1*10:01	P14679	Tyrosinase	470	484	32.83
3905	QCFLPVFLAQPPSGQ	DRB1*10:01	P78358	NY-ESO-1	164	178	46.29
3906	QCTEVRADTRPWSGP	DRB1*03:11	O75767	TRP2	60	74	33.23
3907	QDLVQEKYLEYRQVP	DPB1*33:01	P43355	MAGE1	241	255	37.23
3908	QDLVQEKYLEYRQVP	DPB1*71:01	P43355	MAGE1	241	255	37.23
3909	QDWLGVSRQLRTKAW	DRB1*11:03	P40967	PMEL17	30	44	36.72
3910	QDWLGVSRQLRTKAW	DRB1*11:04	P40967	PMEL17	30	44	43.91
3911	QDWLGVSRQLRTKAW	DRB1*11:42	P40967	PMEL17	30	44	34.81
3912	QDWLGVSRQLRTKAW	DRB1*11:46	P40967	PMEL17	30	44	43.91
3913	QDWLGVSRQLRTKAW	DRB1*11:58	P40967	PMEL17	30	44	43.91
3914	QDWLGVSRQLRTKAW	DRB1*13:11	P40967	PMEL17	30	44	43.91
3915	QDYIKSYLEQASRIW	DRB1*01:01	P14679	Tyrosinase	461	475	42.27
3916	QDYIKSYLEQASRIW	DRB1*01:18	P14679	Tyrosinase	461	475	34.94

3917	QEALGLVCVQAATSS	DRB1*01:01	P43355	MAGE1	20	34	46.63
3918	QEALGLVCVQAATSS	DRB1*01:18	P43355	MAGE1	20	34	42.54
3919	QEALGLVCVQAATSS	DRB1*01:20	P43355	MAGE1	20	34	41.47
3920	QEALGLVCVQAATSS	DRB1*10:01	P43355	MAGE1	20	34	47.19
3921	QEFMAFVAMVTTACH	DRB1*01:01	P04271	S100	70	84	27.21
3922	QEFMAFVAMVTTACH	DRB1*01:11	P04271	S100	70	84	47.26
3923	QEFMAFVAMVTTACH	DRB1*01:18	P04271	S100	70	84	21.8
3924	QEFMAFVAMVTTACH	DRB1*01:24	P04271	S100	70	84	33.55
3925	QEFMAFVAMVTTACH	DRB1*01:29	P04271	S100	70	84	39.96
3926	QEFMAFVAMVTTACH	DRB1*10:01	P04271	S100	70	84	32.16
3927	QEREQFLGALDLAKK	DRB1*01:18	O75767	TRP2	136	150	49
3928	QFLGALDLAKKRVHP	DRB1*11:03	O75767	TRP2	140	154	40.06
3929	QFLGALDLAKKRVHP	DRB1*11:04	O75767	TRP2	140	154	35.1
3930	QFLGALDLAKKRVHP	DRB1*11:42	O75767	TRP2	140	154	41.77
3931	QFLGALDLAKKRVHP	DRB1*11:46	O75767	TRP2	140	154	35.1
3932	QFLGALDLAKKRVHP	DRB1*11:58	O75767	TRP2	140	154	35.1
3933	QFLGALDLAKKRVHP	DRB1*13:11	O75767	TRP2	140	154	35.1
3934	QHWVGLLGPNGTQPQ	DRB1*01:01	O75767	TRP2	161	175	31.19
3935	QHWVGLLGPNGTQPQ	DRB1*01:18	O75767	TRP2	161	175	40.04
3936	QIMPKTGFLIIVLVM	DRB1*01:18	P43355	MAGE1	187	201	46.66
3937	QIMPKTGFLIIVLVM	DRB1*01:20	P43355	MAGE1	187	201	40.75
3938	QKRSFVYVWKTWGQY	DRB1*11:01	P40967	PMEL17	145	159	18.3
3939	QKRSFVYVWKTWGQY	DRB1*11:04	P40967	PMEL17	145	159	37.12
3940	QKRSFVYVWKTWGQY	DRB1*11:08	P40967	PMEL17	145	159	41.63
3941	QKRSFVYVWKTWGQY	DRB1*11:10	P40967	PMEL17	145	159	18.3
3942	QKRSFVYVWKTWGQY	DRB1*11:12	P40967	PMEL17	145	159	18.3
3943	QKRSFVYVWKTWGQY	DRB1*11:28	P40967	PMEL17	145	159	18.3
3944	QKRSFVYVWKTWGQY	DRB1*11:29	P40967	PMEL17	145	159	18.3
3945	QKRSFVYVWKTWGQY	DRB1*11:37	P40967	PMEL17	145	159	39.89
3946	QKRSFVYVWKTWGQY	DRB1*11:42	P40967	PMEL17	145	159	48.04
3947	QKRSFVYVWKTWGQY	DRB1*11:46	P40967	PMEL17	145	159	37.12
3948	QKRSFVYVWKTWGQY	DRB1*11:49	P40967	PMEL17	145	159	18.3
3949	QKRSFVYVWKTWGQY	DRB1*11:58	P40967	PMEL17	145	159	37.12
3950	QKRSFVYVWKTWGQY	DRB1*11:62	P40967	PMEL17	145	159	18.3
3951	QKRSFVYVWKTWGQY	DRB1*11:74	P40967	PMEL17	145	159	18.3
3952	QKRSFVYVWKTWGQY	DRB1*13:05	P40967	PMEL17	145	159	18.3
3953	QKRSFVYVWKTWGQY	DRB1*13:07	P40967	PMEL17	145	159	39.89
3954	QKRSFVYVWKTWGQY	DRB1*13:11	P40967	PMEL17	145	159	37.12
3955	QKRSFVYVWKTWGQY	DRB1*13:14	P40967	PMEL17	145	159	18.3
3956	QKRSFVYVWKTWGQY	DRB1*13:21	P40967	PMEL17	145	159	30.04

3957	QKRSFVYVWKTWGQY	DRB1*13:50	P40967	PMEL17	145	159	18.3
3958	QLSLLMWITQCFLPV	DPB1*01:01	P78358	NY-ESO-1	155	169	43.49
3959	QLSLLMWITQCFLPV	DPB1*02:01	P78358	NY-ESO-1	155	169	34.36
3960	QLSLLMWITQCFLPV	DPB1*02:02	P78358	NY-ESO-1	155	169	33.15
3961	QLSLLMWITQCFLPV	DPB1*04:01	P78358	NY-ESO-1	155	169	15.36
3962	QLSLLMWITQCFLPV	DPB1*126:01	P78358	NY-ESO-1	155	169	15.36
3963	QLSLLMWITQCFLPV	DPB1*15:01	P78358	NY-ESO-1	155	169	48.19
3964	QLSLLMWITQCFLPV	DPB1*23:01	P78358	NY-ESO-1	155	169	15.36
3965	QLSLLMWITQCFLPV	DPB1*33:01	P78358	NY-ESO-1	155	169	12.9
3966	QLSLLMWITQCFLPV	DPB1*39:01	P78358	NY-ESO-1	155	169	15.36
3967	QLSLLMWITQCFLPV	DPB1*46:01	P78358	NY-ESO-1	155	169	34.36
3968	QLSLLMWITQCFLPV	DPB1*47:01	P78358	NY-ESO-1	155	169	33.15
3969	QLSLLMWITQCFLPV	DPB1*71:01	P78358	NY-ESO-1	155	169	12.9
3970	QLSLLMWITQCFLPV	DPB1*72:01	P78358	NY-ESO-1	155	169	23.16
3971	QLSLLMWITQCFLPV	DPB1*81:01	P78358	NY-ESO-1	155	169	34.36
3972	QLVLHQILKGGSGTY	DRB1*08:04	P40967	PMEL17	551	565	37.93
3973	QLVLHQILKGGSGTY	DRB1*11:01	P40967	PMEL17	551	565	36.77
3974	QLVLHQILKGGSGTY	DRB1*11:03	P40967	PMEL17	551	565	37.72
3975	QLVLHQILKGGSGTY	DRB1*11:04	P40967	PMEL17	551	565	15.12
3976	QLVLHQILKGGSGTY	DRB1*11:10	P40967	PMEL17	551	565	36.77
3977	QLVLHQILKGGSGTY	DRB1*11:12	P40967	PMEL17	551	565	36.77
3978	QLVLHQILKGGSGTY	DRB1*11:28	P40967	PMEL17	551	565	36.77
3979	QLVLHQILKGGSGTY	DRB1*11:29	P40967	PMEL17	551	565	36.77
3980	QLVLHQILKGGSGTY	DRB1*11:42	P40967	PMEL17	551	565	25.08
3981	QLVLHQILKGGSGTY	DRB1*11:46	P40967	PMEL17	551	565	15.12
3982	QLVLHQILKGGSGTY	DRB1*11:49	P40967	PMEL17	551	565	36.77
3983	QLVLHQILKGGSGTY	DRB1*11:58	P40967	PMEL17	551	565	15.12
3984	QLVLHQILKGGSGTY	DRB1*11:62	P40967	PMEL17	551	565	36.77
3985	QLVLHQILKGGSGTY	DRB1*11:74	P40967	PMEL17	551	565	36.77
3986	QLVLHQILKGGSGTY	DRB1*13:05	P40967	PMEL17	551	565	36.77
3987	QLVLHQILKGGSGTY	DRB1*13:11	P40967	PMEL17	551	565	15.12
3988	QLVLHQILKGGSGTY	DRB1*13:14	P40967	PMEL17	551	565	36.77
3989	QLVLHQILKGGSGTY	DRB1*13:50	P40967	PMEL17	551	565	36.77
3990	QMTFGRLQGISPKIM	DRB1*01:01	Q16385	SSX2	98	112	11.75
3991	QMTFGRLQGISPKIM	DRB1*01:18	Q16385	SSX2	98	112	12.47
3992	QMTFGRLQGISPKIM	DRB1*01:20	Q16385	SSX2	98	112	27.43
3993	QMTFGRLQGISPKIM	DRB1*01:24	Q16385	SSX2	98	112	31.05
3994	QMTFGRLQGISPKIM	DRB1*01:29	Q16385	SSX2	98	112	38.38
3995	QMTFGRLQGISPKIM	DRB1*10:01	Q16385	SSX2	98	112	26.83
3996	QNILLSNAPLGPQFP	DRB1*01:01	P14679	Tyrosinase	56	70	24.12

3997	QNILLSNAPLGPQFP	DRB1*01:18	P14679	Tyrosinase	56	70	22.56
3998	QNILLSNAPLGPQFP	DRB1*01:20	P14679	Tyrosinase	56	70	18.33
3999	QNILLSNAPLGPQFP	DRB1*11:14	P14679	Tyrosinase	56	70	28.71
4000	QNILLSNAPLGPQFP	DRB1*13:02	P14679	Tyrosinase	56	70	28.71
4001	QNILLSNAPLGPQFP	DRB1*13:23	P14679	Tyrosinase	56	70	28.71
4002	QNILLSNAPLGPQFP	DRB1*13:97	P14679	Tyrosinase	56	70	28.71
4003	QPVLPSPACQLVLHQ	DRB1*01:20	P40967	PMEL17	542	556	47.93
4004	QRVLIVRRNLLDLSK	DRB1*08:04	P17643	TRP1	124	138	39.07
4005	QRVLIVRRNLLDLSK	DRB1*11:02	P17643	TRP1	124	138	20.24
4006	QRVLIVRRNLLDLSK	DRB1*11:03	P17643	TRP1	124	138	26.66
4007	QRVLIVRRNLLDLSK	DRB1*11:04	P17643	TRP1	124	138	17.56
4008	QRVLIVRRNLLDLSK	DRB1*11:13	P17643	TRP1	124	138	14.61
4009	QRVLIVRRNLLDLSK	DRB1*11:42	P17643	TRP1	124	138	14.6
4010	QRVLIVRRNLLDLSK	DRB1*11:46	P17643	TRP1	124	138	17.56
4011	QRVLIVRRNLLDLSK	DRB1*11:58	P17643	TRP1	124	138	17.56
4012	QRVLIVRRNLLDLSK	DRB1*11:65	P17643	TRP1	124	138	20.24
4013	QRVLIVRRNLLDLSK	DRB1*11:84	P17643	TRP1	124	138	32.71
4014	QRVLIVRRNLLDLSK	DRB1*13:01	P17643	TRP1	124	138	20.24
4015	QRVLIVRRNLLDLSK	DRB1*13:11	P17643	TRP1	124	138	17.56
4016	QRVLIVRRNLLDLSK	DRB1*13:21	P17643	TRP1	124	138	33.15
4017	QRVLIVRRNLLDLSK	DRB1*13:61	P17643	TRP1	124	138	41.23
4018	QRVLIVRRNLLDLSK	DRB1*14:01	P17643	TRP1	124	138	23.02
4019	QRVLIVRRNLLDLSK	DRB1*14:04	P17643	TRP1	124	138	42.59
4020	QRVLIVRRNLLDLSK	DRB1*14:06	P17643	TRP1	124	138	33.93
4021	QRVLIVRRNLLDLSK	DRB1*14:12	P17643	TRP1	124	138	47.15
4022	QRVLIVRRNLLDLSK	DRB1*14:32	P17643	TRP1	124	138	13.6
4023	QRVLIVRRNLLDLSK	DRB1*14:38	P17643	TRP1	124	138	45.13
4024	QRVLIVRRNLLDLSK	DRB1*14:54	P17643	TRP1	124	138	23.02
4025	QTHLSPNDPIFVLLH	DRB1*11:14	P17643	TRP1	390	404	32.73
4026	QTHLSPNDPIFVLLH	DRB1*13:02	P17643	TRP1	390	404	32.73
4027	QTHLSPNDPIFVLLH	DRB1*13:23	P17643	TRP1	390	404	32.73
4028	QTHLSPNDPIFVLLH	DRB1*13:96	P17643	TRP1	390	404	40.61
4029	QTHLSPNDPIFVLLH	DRB1*13:97	P17643	TRP1	390	404	32.73
4030	QVIWVNNTIINGSQV	DRB1*11:14	P40967	PMEL17	101	115	18.45
4031	QVIWVNNTIINGSQV	DRB1*13:02	P40967	PMEL17	101	115	18.45
4032	QVIWVNNTIINGSQV	DRB1*13:23	P40967	PMEL17	101	115	18.45
4033	QVIWVNNTIINGSQV	DRB1*13:96	P40967	PMEL17	101	115	24.99
4034	QVIWVNNTIINGSQV	DRB1*13:97	P40967	PMEL17	101	115	18.45
4035	QVTTTEWVETTAREL	DRB1*07:01	P40967	PMEL17	417	431	47.62
4036	QVVLQAAIPLTSCGS	DRB1*01:01	P40967	PMEL17	289	303	11.98

4037	QVVLQAAIPLTSCGS	DRB1*01:02	P40967	PMEL17	289	303	24.97
4038	QVVLQAAIPLTSCGS	DRB1*01:11	P40967	PMEL17	289	303	28.61
4039	QVVLQAAIPLTSCGS	DRB1*01:18	P40967	PMEL17	289	303	11.49
4040	QVVLQAAIPLTSCGS	DRB1*01:20	P40967	PMEL17	289	303	9.87
4041	QVVLQAAIPLTSCGS	DRB1*01:24	P40967	PMEL17	289	303	22.49
4042	QVVLQAAIPLTSCGS	DRB1*01:29	P40967	PMEL17	289	303	24.04
4043	QVVLQAAIPLTSCGS	DRB1*10:01	P40967	PMEL17	289	303	20.57
4044	QWEVISCKLIKRATT	DRB1*11:03	O75767	TRP2	220	234	39.71
4045	QWEVISCKLIKRATT	DRB1*11:04	O75767	TRP2	220	234	36.31
4046	QWEVISCKLIKRATT	DRB1*11:42	O75767	TRP2	220	234	31.2
4047	QWEVISCKLIKRATT	DRB1*11:46	O75767	TRP2	220	234	36.31
4048	QWEVISCKLIKRATT	DRB1*11:58	O75767	TRP2	220	234	36.31
4049	QWEVISCKLIKRATT	DRB1*13:11	O75767	TRP2	220	234	36.31
4050	QYWQVLGGPVSGLSI	DRB1*01:01	P40967	PMEL17	158	172	8.91
4051	QYWQVLGGPVSGLSI	DRB1*01:11	P40967	PMEL17	158	172	38.16
4052	QYWQVLGGPVSGLSI	DRB1*01:18	P40967	PMEL17	158	172	9.71
4053	QYWQVLGGPVSGLSI	DRB1*01:20	P40967	PMEL17	158	172	30.88
4054	QYWQVLGGPVSGLSI	DRB1*01:24	P40967	PMEL17	158	172	24.54
4055	QYWQVLGGPVSGLSI	DRB1*01:29	P40967	PMEL17	158	172	23.44
4056	QYWQVLGGPVSGLSI	DRB1*10:01	P40967	PMEL17	158	172	26.21
4057	RALAETSYVKVLEHV	DPB1*33:01	P43358	MAGE4	277	291	25.83
4058	RALAETSYVKVLEHV	DPB1*71:01	P43358	MAGE4	277	291	25.83
4059	RALAETSYVKVLEYV	DPB1*02:02	P43355	MAGE1	269	283	48.52
4060	RALAETSYVKVLEYV	DPB1*33:01	P43355	MAGE1	269	283	20.19
4061	RALAETSYVKVLEYV	DPB1*47:01	P43355	MAGE1	269	283	48.52
4062	RALAETSYVKVLEYV	DPB1*71:01	P43355	MAGE1	269	283	20.19
4063	RAMLGTHTMEVTVYH	DRB1*07:01	P40967	PMEL17	176	190	43.56
4064	RAVFLALSAQLLQAR	DPB1*33:01	Q13072	BAGE	4	18	25.64
4065	RAVFLALSAQLLQAR	DPB1*71:01	Q13072	BAGE	4	18	25.64
4066	RAVFLALSAQLLQAR	DRB1*01:01	Q13072	BAGE	4	18	2.61
4067	RAVFLALSAQLLQAR	DRB1*01:02	Q13072	BAGE	4	18	13.44
4068	RAVFLALSAQLLQAR	DRB1*01:03	Q13072	BAGE	4	18	16.69
4069	RAVFLALSAQLLQAR	DRB1*01:11	Q13072	BAGE	4	18	3.85
4070	RAVFLALSAQLLQAR	DRB1*01:18	Q13072	BAGE	4	18	2.47
4071	RAVFLALSAQLLQAR	DRB1*01:20	Q13072	BAGE	4	18	4.91
4072	RAVFLALSAQLLQAR	DRB1*01:24	Q13072	BAGE	4	18	3.13
4073	RAVFLALSAQLLQAR	DRB1*01:29	Q13072	BAGE	4	18	3.2
4074	RAVFLALSAQLLQAR	DRB1*04:08	Q13072	BAGE	4	18	49.46
4075	RAVFLALSAQLLQAR	DRB1*07:01	Q13072	BAGE	4	18	16.48
4076	RAVFLALSAQLLQAR	DRB1*08:30	Q13072	BAGE	4	18	44.79

4077	RAVFLALSAQLLQAR	DRB1*09:01	Q13072	BAGE	4	18	31.22
4078	RAVFLALSAQLLQAR	DRB1*10:01	Q13072	BAGE	4	18	6.48
4079	RAVFLALSAQLLQAR	DRB1*11:01	Q13072	BAGE	4	18	49.7
4080	RAVFLALSAQLLQAR	DRB1*11:08	Q13072	BAGE	4	18	31.24
4081	RAVFLALSAQLLQAR	DRB1*11:10	Q13072	BAGE	4	18	49.7
4082	RAVFLALSAQLLQAR	DRB1*11:12	Q13072	BAGE	4	18	49.7
4083	RAVFLALSAQLLQAR	DRB1*11:13	Q13072	BAGE	4	18	38.1
4084	RAVFLALSAQLLQAR	DRB1*11:14	Q13072	BAGE	4	18	48.97
4085	RAVFLALSAQLLQAR	DRB1*11:19	Q13072	BAGE	4	18	42.46
4086	RAVFLALSAQLLQAR	DRB1*11:28	Q13072	BAGE	4	18	49.7
4087	RAVFLALSAQLLQAR	DRB1*11:29	Q13072	BAGE	4	18	49.7
4088	RAVFLALSAQLLQAR	DRB1*11:42	Q13072	BAGE	4	18	47.37
4089	RAVFLALSAQLLQAR	DRB1*11:49	Q13072	BAGE	4	18	49.7
4090	RAVFLALSAQLLQAR	DRB1*11:62	Q13072	BAGE	4	18	49.7
4091	RAVFLALSAQLLQAR	DRB1*11:74	Q13072	BAGE	4	18	49.7
4092	RAVFLALSAQLLQAR	DRB1*12:16	Q13072	BAGE	4	18	28.77
4093	RAVFLALSAQLLQAR	DRB1*13:02	Q13072	BAGE	4	18	48.97
4094	RAVFLALSAQLLQAR	DRB1*13:05	Q13072	BAGE	4	18	49.7
4095	RAVFLALSAQLLQAR	DRB1*13:14	Q13072	BAGE	4	18	49.7
4096	RAVFLALSAQLLQAR	DRB1*13:23	Q13072	BAGE	4	18	48.97
4097	RAVFLALSAQLLQAR	DRB1*13:50	Q13072	BAGE	4	18	49.7
4098	RAVFLALSAQLLQAR	DRB1*13:97	Q13072	BAGE	4	18	48.97
4099	RAVFLALSAQLLQAR	DRB1*14:32	Q13072	BAGE	4	18	40.48
4100	RAVFLALSAQLLQAR	DRB1*15:01	Q13072	BAGE	4	18	29.22
4101	RAVFLALSAQLLQAR	DRB1*15:02	Q13072	BAGE	4	18	16.16
4102	RAVFLALSAQLLQAR	DRB1*15:03	Q13072	BAGE	4	18	43.91
4103	RAVFLALSAQLLQAR	DRB1*15:06	Q13072	BAGE	4	18	29.22
4104	RAVFLALSAQLLQAR	DRB1*15:07	Q13072	BAGE	4	18	41.46
4105	RAVFLALSAQLLQAR	DRB1*15:15	Q13072	BAGE	4	18	15.2
4106	RAVFLALSAQLLQAR	DRB1*16:01	Q13072	BAGE	4	18	12.43
4107	RAVFLALSAQLLQAR	DRB1*16:02	Q13072	BAGE	4	18	8.32
4108	RAVFLALSAQLLQAR	DRB1*16:04	Q13072	BAGE	4	18	16.93
4109	RAVFLALSAQLLQAR	DRB1*16:05	Q13072	BAGE	4	18	11.76
4110	RAVFLALSAQLLQAR	DRB1*16:09	Q13072	BAGE	4	18	10.21
4111	RCFPVIFGKASESLK	DRB1*01:20	P43358	MAGE4	146	160	45.62
4112	RCLLHLAVIGALLAV	DRB1*01:18	P40967	PMEL17	7	21	30.98
4113	RCLLHLAVIGALLAV	DRB1*01:24	P40967	PMEL17	7	21	48.3
4114	RDIDFAHEAPAFLPW	DRB1*01:01	P14679	Tyrosinase	196	210	31.68
4115	RDIDFAHEAPAFLPW	DRB1*01:18	P14679	Tyrosinase	196	210	36.71
4116	RDTLLGGFFPWLKVY	DPB1*02:02	O75767	TRP2	194	208	49.46

4117	RDTLLGGFFPWLKVY	DPB1*33:01	O75767	TRP2	194	208	19.47
4118	RDTLLGGFFPWLKVY	DPB1*47:01	O75767	TRP2	194	208	49.46
4119	RDTLLGGFFPWLKVY	DPB1*71:01	O75767	TRP2	194	208	19.47
4120	REQFLGALDLAKKRV	DRB1*01:18	O75767	TRP2	138	152	44.68
4121	RESYMVPFIPLYRNG	DPB1*15:01	P14679	Tyrosinase	422	436	48.09
4122	RESYMVPFIPLYRNG	DPB1*33:01	P14679	Tyrosinase	422	436	22.51
4123	RESYMVPFIPLYRNG	DPB1*71:01	P14679	Tyrosinase	422	436	22.51
4124	RESYMVPFIPLYRNG	DRB1*01:20	P14679	Tyrosinase	422	436	48.43
4125	RESYMVPFIPLYRNG	DRB1*11:02	P14679	Tyrosinase	422	436	37.93
4126	RESYMVPFIPLYRNG	DRB1*11:13	P14679	Tyrosinase	422	436	47.02
4127	RESYMVPFIPLYRNG	DRB1*11:42	P14679	Tyrosinase	422	436	43.98
4128	RESYMVPFIPLYRNG	DRB1*11:65	P14679	Tyrosinase	422	436	37.93
4129	RESYMVPFIPLYRNG	DRB1*13:01	P14679	Tyrosinase	422	436	37.93
4130	RFFFPSLREAALREE	DRB1*01:01	P43355	MAGE1	291	305	47.18
4131	RFFFPSLREAALREE	DRB1*01:18	P43355	MAGE1	291	305	43.93
4132	RFFFPSLREAALREE	DRB1*11:01	P43355	MAGE1	291	305	18.8
4133	RFFFPSLREAALREE	DRB1*11:08	P43355	MAGE1	291	305	45.07
4134	RFFFPSLREAALREE	DRB1*11:10	P43355	MAGE1	291	305	18.8
4135	RFFFPSLREAALREE	DRB1*11:12	P43355	MAGE1	291	305	18.8
4136	RFFFPSLREAALREE	DRB1*11:28	P43355	MAGE1	291	305	18.8
4137	RFFFPSLREAALREE	DRB1*11:29	P43355	MAGE1	291	305	18.8
4138	RFFFPSLREAALREE	DRB1*11:37	P43355	MAGE1	291	305	31.49
4139	RFFFPSLREAALREE	DRB1*11:49	P43355	MAGE1	291	305	18.8
4140	RFFFPSLREAALREE	DRB1*11:62	P43355	MAGE1	291	305	18.8
4141	RFFFPSLREAALREE	DRB1*11:74	P43355	MAGE1	291	305	18.8
4142	RFFFPSLREAALREE	DRB1*13:05	P43355	MAGE1	291	305	18.8
4143	RFFFPSLREAALREE	DRB1*13:07	P43355	MAGE1	291	305	31.49
4144	RFFFPSLREAALREE	DRB1*13:14	P43355	MAGE1	291	305	18.8
4145	RFFFPSLREAALREE	DRB1*13:21	P43355	MAGE1	291	305	26.46
4146	RFFFPSLREAALREE	DRB1*13:50	P43355	MAGE1	291	305	18.8
4147	RFVIGLRVWQWEVIS	DPB1*33:01	O75767	TRP2	211	225	27.72
4148	RFVIGLRVWQWEVIS	DPB1*71:01	O75767	TRP2	211	225	27.72
4149	RGPESRLLEFYLAMP	DPB1*33:01	P78358	NY-ESO-1	81	95	38.52
4150	RGPESRLLEFYLAMP	DPB1*71:01	P78358	NY-ESO-1	81	95	38.52
4151	RGQCTEVRADTRPWS	DRB1*03:11	O75767	TRP2	58	72	49.15
4152	RGSCQNILLSNAPLG	DRB1*01:20	P14679	Tyrosinase	52	66	49.63
4153	RGSRSYVPLAHSSSA	DRB1*10:01	P40967	PMEL17	192	206	42.04
4154	RIWSWLLGAAMVGAV	DRB1*01:01	P14679	Tyrosinase	473	487	11.66
4155	RIWSWLLGAAMVGAV	DRB1*01:11	P14679	Tyrosinase	473	487	28.36
4156	RIWSWLLGAAMVGAV	DRB1*01:18	P14679	Tyrosinase	473	487	10.27

4157	RIWSWLLGAAMVGAV	DRB1*01:20	P14679	Tyrosinase	473	487	34.41
4158	RIWSWLLGAAMVGAV	DRB1*01:24	P14679	Tyrosinase	473	487	17.09
4159	RIWSWLLGAAMVGAV	DRB1*01:29	P14679	Tyrosinase	473	487	19.89
4160	RIWSWLLGAAMVGAV	DRB1*10:01	P14679	Tyrosinase	473	487	36.16
4161	RKFFHRTCKCTGNFA	DRB1*11:01	O75767	TRP2	88	102	39.55
4162	RKFFHRTCKCTGNFA	DRB1*11:10	O75767	TRP2	88	102	39.55
4163	RKFFHRTCKCTGNFA	DRB1*11:12	O75767	TRP2	88	102	39.55
4164	RKFFHRTCKCTGNFA	DRB1*11:28	O75767	TRP2	88	102	39.55
4165	RKFFHRTCKCTGNFA	DRB1*11:29	O75767	TRP2	88	102	39.55
4166	RKFFHRTCKCTGNFA	DRB1*11:49	O75767	TRP2	88	102	39.55
4167	RKFFHRTCKCTGNFA	DRB1*11:62	O75767	TRP2	88	102	39.55
4168	RKFFHRTCKCTGNFA	DRB1*11:74	O75767	TRP2	88	102	39.55
4169	RKFFHRTCKCTGNFA	DRB1*13:05	O75767	TRP2	88	102	39.55
4170	RKFFHRTCKCTGNFA	DRB1*13:14	O75767	TRP2	88	102	39.55
4171	RKFFHRTCKCTGNFA	DRB1*13:50	O75767	TRP2	88	102	39.55
4172	RKKPPVIRQNIHSLS	DRB1*11:14	O75767	TRP2	120	134	48.91
4173	RKKPPVIRQNIHSLS	DRB1*13:02	O75767	TRP2	120	134	48.91
4174	RKKPPVIRQNIHSLS	DRB1*13:23	O75767	TRP2	120	134	48.91
4175	RKKPPVIRQNIHSLS	DRB1*13:97	O75767	TRP2	120	134	48.91
4176	RKYEAMTKLGFKATL	DRB1*13:21	Q16385	SSX2	53	67	47.08
4177	RLLEFYLAMPFATPM	DPB1*02:01	P78358	NY-ESO-1	86	100	47.67
4178	RLLEFYLAMPFATPM	DPB1*15:01	P78358	NY-ESO-1	86	100	45.06
4179	RLLEFYLAMPFATPM	DPB1*33:01	P78358	NY-ESO-1	86	100	18.81
4180	RLLEFYLAMPFATPM	DPB1*46:01	P78358	NY-ESO-1	86	100	47.67
4181	RLLEFYLAMPFATPM	DPB1*71:01	P78358	NY-ESO-1	86	100	18.81
4182	RLLEFYLAMPFATPM	DPB1*72:01	P78358	NY-ESO-1	86	100	47.3
4183	RLLEFYLAMPFATPM	DPB1*81:01	P78358	NY-ESO-1	86	100	47.67
4184	RLLEFYLAMPFATPM	DRB1*01:01	P78358	NY-ESO-1	86	100	8.84
4185	RLLEFYLAMPFATPM	DRB1*01:11	P78358	NY-ESO-1	86	100	17.69
4186	RLLEFYLAMPFATPM	DRB1*01:18	P78358	NY-ESO-1	86	100	8.28
4187	RLLEFYLAMPFATPM	DRB1*01:20	P78358	NY-ESO-1	86	100	24.34
4188	RLLEFYLAMPFATPM	DRB1*01:24	P78358	NY-ESO-1	86	100	10.64
4189	RLLEFYLAMPFATPM	DRB1*01:29	P78358	NY-ESO-1	86	100	14.23
4190	RLLEFYLAMPFATPM	DRB1*09:01	P78358	NY-ESO-1	86	100	34.37
4191	RLLEFYLAMPFATPM	DRB1*10:01	P78358	NY-ESO-1	86	100	9.31
4192	RLLEFYLAMPFATPM	DRB1*15:01	P78358	NY-ESO-1	86	100	34.19
4193	RLLEFYLAMPFATPM	DRB1*15:06	P78358	NY-ESO-1	86	100	34.19
4194	RLLEFYLAMPFATPM	DRB1*16:09	P78358	NY-ESO-1	86	100	46.86
4195	RLRERKQLVIYEEIS	DPB1*33:01	Q16385	SSX2	167	181	45.34
4196	RLRERKQLVIYEEIS	DPB1*71:01	Q16385	SSX2	167	181	45.34

4197	RNGYRALMDKSLHVG	DRB1*01:01	Q16655	MELAN_A	51	65	19.01
4198	RNGYRALMDKSLHVG	DRB1*01:18	Q16655	MELAN_A	51	65	17.23
4199	RNGYRALMDKSLHVG	DRB1*01:24	Q16655	MELAN_A	51	65	44.52
4200	RNGYRALMDKSLHVG	DRB1*01:29	Q16655	MELAN_A	51	65	41.89
4201	RPQMTFGRLQGISPK	DRB1*01:01	Q16385	SSX2	96	110	23.77
4202	RPQMTFGRLQGISPK	DRB1*01:18	Q16385	SSX2	96	110	27.06
4203	RPQMTFGRLQGISPK	DRB1*10:01	Q16385	SSX2	96	110	49.8
4204	RQLQLSISSCLQQLS	DRB1*01:01	P78358	NY-ESO-1	143	157	29.3
4205	RQLQLSISSCLQQLS	DRB1*01:18	P78358	NY-ESO-1	143	157	24.72
4206	RQLQLSISSCLQQLS	DRB1*01:20	P78358	NY-ESO-1	143	157	24.36
4207	RQLQLSISSCLQQLS	DRB1*01:24	P78358	NY-ESO-1	143	157	46.8
4208	RQLQLSISSCLQQLS	DRB1*01:29	P78358	NY-ESO-1	143	157	49.86
4209	RQLQLSISSCLQQLS	DRB1*07:01	P78358	NY-ESO-1	143	157	21.18
4210	RRNGYRALMDKSLHV	DRB1*01:01	Q16655	MELAN_A	50	64	18.26
4211	RRNGYRALMDKSLHV	DRB1*01:18	Q16655	MELAN_A	50	64	16.46
4212	RRNGYRALMDKSLHV	DRB1*01:24	Q16655	MELAN_A	50	64	46.66
4213	RRNGYRALMDKSLHV	DRB1*01:29	Q16655	MELAN_A	50	64	43.45
4214	RRRNGYRALMDKSLH	DRB1*01:01	Q16655	MELAN_A	49	63	29.22
4215	RRRNGYRALMDKSLH	DRB1*01:18	Q16655	MELAN_A	49	63	26.09
4216	RSFVYVWKTWGQYWQ	DRB1*11:01	P40967	PMEL17	147	161	30.25
4217	RSFVYVWKTWGQYWQ	DRB1*11:10	P40967	PMEL17	147	161	30.25
4218	RSFVYVWKTWGQYWQ	DRB1*11:12	P40967	PMEL17	147	161	30.25
4219	RSFVYVWKTWGQYWQ	DRB1*11:28	P40967	PMEL17	147	161	30.25
4220	RSFVYVWKTWGQYWQ	DRB1*11:29	P40967	PMEL17	147	161	30.25
4221	RSFVYVWKTWGQYWQ	DRB1*11:49	P40967	PMEL17	147	161	30.25
4222	RSFVYVWKTWGQYWQ	DRB1*11:62	P40967	PMEL17	147	161	30.25
4223	RSFVYVWKTWGQYWQ	DRB1*11:74	P40967	PMEL17	147	161	30.25
4224	RSFVYVWKTWGQYWQ	DRB1*13:05	P40967	PMEL17	147	161	30.25
4225	RSFVYVWKTWGQYWQ	DRB1*13:14	P40967	PMEL17	147	161	30.25
4226	RSFVYVWKTWGQYWQ	DRB1*13:50	P40967	PMEL17	147	161	30.25
4227	RSNFDSTLISPNSVF	DRB1*10:01	P17643	TRP1	269	283	30.07
4228	RSYVPLAHSSSAFTI	DRB1*01:01	P40967	PMEL17	195	209	27.89
4229	RSYVPLAHSSSAFTI	DRB1*01:18	P40967	PMEL17	195	209	25.5
4230	RSYVPLAHSSSAFTI	DRB1*01:20	P40967	PMEL17	195	209	44.03
4231	RSYVPLAHSSSAFTI	DRB1*01:29	P40967	PMEL17	195	209	49.65
4232	RSYVPLAHSSSAFTI	DRB1*07:01	P40967	PMEL17	195	209	42.27
4233	RSYVPLAHSSSAFTI	DRB1*09:01	P40967	PMEL17	195	209	41.07
4234	RSYVPLAHSSSAFTI	DRB1*10:01	P40967	PMEL17	195	209	27.52
4235	RSYVPLAHSSSAFTI	DRB1*11:01	P40967	PMEL17	195	209	42.32
4236	RSYVPLAHSSSAFTI	DRB1*11:10	P40967	PMEL17	195	209	42.32

4237	RSYVPLAHSSSAFTI	DRB1*11:12	P40967	PMEL17	195	209	42.32
4238	RSYVPLAHSSSAFTI	DRB1*11:28	P40967	PMEL17	195	209	42.32
4239	RSYVPLAHSSSAFTI	DRB1*11:29	P40967	PMEL17	195	209	42.32
4240	RSYVPLAHSSSAFTI	DRB1*11:49	P40967	PMEL17	195	209	42.32
4241	RSYVPLAHSSSAFTI	DRB1*11:62	P40967	PMEL17	195	209	42.32
4242	RSYVPLAHSSSAFTI	DRB1*11:74	P40967	PMEL17	195	209	42.32
4243	RSYVPLAHSSSAFTI	DRB1*13:05	P40967	PMEL17	195	209	42.32
4244	RSYVPLAHSSSAFTI	DRB1*13:14	P40967	PMEL17	195	209	42.32
4245	RSYVPLAHSSSAFTI	DRB1*13:50	P40967	PMEL17	195	209	42.32
4246	RTTHPLFVIATRRSE	DRB1*01:01	P17643	TRP1	153	167	44.82
4247	RTTHPLFVIATRRSE	DRB1*01:18	P17643	TRP1	153	167	28.14
4248	RTTHPLFVIATRRSE	DRB1*01:20	P17643	TRP1	153	167	22.89
4249	RTTHPLFVIATRRSE	DRB1*04:04	P17643	TRP1	153	167	26.14
4250	RTTHPLFVIATRRSE	DRB1*08:01	P17643	TRP1	153	167	42.4
4251	RTTHPLFVIATRRSE	DRB1*08:04	P17643	TRP1	153	167	30.55
4252	RTTHPLFVIATRRSE	DRB1*10:01	P17643	TRP1	153	167	37.76
4253	RTTHPLFVIATRRSE	DRB1*11:01	P17643	TRP1	153	167	24.44
4254	RTTHPLFVIATRRSE	DRB1*11:03	P17643	TRP1	153	167	35.22
4255	RTTHPLFVIATRRSE	DRB1*11:04	P17643	TRP1	153	167	17.16
4256	RTTHPLFVIATRRSE	DRB1*11:08	P17643	TRP1	153	167	45.17
4257	RTTHPLFVIATRRSE	DRB1*11:10	P17643	TRP1	153	167	24.44
4258	RTTHPLFVIATRRSE	DRB1*11:12	P17643	TRP1	153	167	24.44
4259	RTTHPLFVIATRRSE	DRB1*11:28	P17643	TRP1	153	167	24.44
4260	RTTHPLFVIATRRSE	DRB1*11:29	P17643	TRP1	153	167	24.44
4261	RTTHPLFVIATRRSE	DRB1*11:42	P17643	TRP1	153	167	19.38
4262	RTTHPLFVIATRRSE	DRB1*11:46	P17643	TRP1	153	167	17.16
4263	RTTHPLFVIATRRSE	DRB1*11:49	P17643	TRP1	153	167	24.44
4264	RTTHPLFVIATRRSE	DRB1*11:58	P17643	TRP1	153	167	17.16
4265	RTTHPLFVIATRRSE	DRB1*11:62	P17643	TRP1	153	167	24.44
4266	RTTHPLFVIATRRSE	DRB1*11:74	P17643	TRP1	153	167	24.44
4267	RTTHPLFVIATRRSE	DRB1*13:05	P17643	TRP1	153	167	24.44
4268	RTTHPLFVIATRRSE	DRB1*13:11	P17643	TRP1	153	167	17.16
4269	RTTHPLFVIATRRSE	DRB1*13:14	P17643	TRP1	153	167	24.44
4270	RTTHPLFVIATRRSE	DRB1*13:21	P17643	TRP1	153	167	14.84
4271	RTTHPLFVIATRRSE	DRB1*13:50	P17643	TRP1	153	167	24.44
4272	RVIKNYKRCFPVIFG	DRB1*11:03	P43358	MAGE4	139	153	46.58
4273	RVLIVRRNLLDLSKE	DRB1*11:02	P17643	TRP1	125	139	31.53
4274	RVLIVRRNLLDLSKE	DRB1*11:03	P17643	TRP1	125	139	41.92
4275	RVLIVRRNLLDLSKE	DRB1*11:04	P17643	TRP1	125	139	27.27
4276	RVLIVRRNLLDLSKE	DRB1*11:13	P17643	TRP1	125	139	19.51

4277	RVLIVRRNLLDLSKE	DRB1*11:42	P17643	TRP1	125	139	23.19
4278	RVLIVRRNLLDLSKE	DRB1*11:46	P17643	TRP1	125	139	27.27
4279	RVLIVRRNLLDLSKE	DRB1*11:58	P17643	TRP1	125	139	27.27
4280	RVLIVRRNLLDLSKE	DRB1*11:65	P17643	TRP1	125	139	31.53
4281	RVLIVRRNLLDLSKE	DRB1*13:01	P17643	TRP1	125	139	31.53
4282	RVLIVRRNLLDLSKE	DRB1*13:11	P17643	TRP1	125	139	27.27
4283	RVLIVRRNLLDLSKE	DRB1*14:01	P17643	TRP1	125	139	32.49
4284	RVLIVRRNLLDLSKE	DRB1*14:06	P17643	TRP1	125	139	45.95
4285	RVLIVRRNLLDLSKE	DRB1*14:32	P17643	TRP1	125	139	19.06
4286	RVLIVRRNLLDLSKE	DRB1*14:54	P17643	TRP1	125	139	32.49
4287	RVNARVRIAYPSLRE	DRB1*01:20	P43358	MAGE4	293	307	43.79
4288	RVNARVRIAYPSLRE	DRB1*11:02	P43358	MAGE4	293	307	29.94
4289	RVNARVRIAYPSLRE	DRB1*11:03	P43358	MAGE4	293	307	36.79
4290	RVNARVRIAYPSLRE	DRB1*11:13	P43358	MAGE4	293	307	43.96
4291	RVNARVRIAYPSLRE	DRB1*11:42	P43358	MAGE4	293	307	36.6
4292	RVNARVRIAYPSLRE	DRB1*11:65	P43358	MAGE4	293	307	29.94
4293	RVNARVRIAYPSLRE	DRB1*13:01	P43358	MAGE4	293	307	29.94
4294	RVRFFFPSLREAALR	DPB1*33:01	P43355	MAGE1	289	303	47.68
4295	RVRFFFPSLREAALR	DPB1*71:01	P43355	MAGE1	289	303	47.68
4296	RVRFFFPSLREAALR	DRB1*01:01	P43355	MAGE1	289	303	34.59
4297	RVRFFFPSLREAALR	DRB1*01:18	P43355	MAGE1	289	303	30.75
4298	RVRFFFPSLREAALR	DRB1*01:20	P43355	MAGE1	289	303	36.17
4299	RVRFFFPSLREAALR	DRB1*08:24	P43355	MAGE1	289	303	47.68
4300	RVRFFFPSLREAALR	DRB1*10:01	P43355	MAGE1	289	303	28.34
4301	RVRFFFPSLREAALR	DRB1*11:01	P43355	MAGE1	289	303	14
4302	RVRFFFPSLREAALR	DRB1*11:03	P43355	MAGE1	289	303	41.13
4303	RVRFFFPSLREAALR	DRB1*11:04	P43355	MAGE1	289	303	32.94
4304	RVRFFFPSLREAALR	DRB1*11:08	P43355	MAGE1	289	303	31.48
4305	RVRFFFPSLREAALR	DRB1*11:10	P43355	MAGE1	289	303	14
4306	RVRFFFPSLREAALR	DRB1*11:12	P43355	MAGE1	289	303	14
4307	RVRFFFPSLREAALR	DRB1*11:27	P43355	MAGE1	289	303	42.89
4308	RVRFFFPSLREAALR	DRB1*11:28	P43355	MAGE1	289	303	14
4309	RVRFFFPSLREAALR	DRB1*11:29	P43355	MAGE1	289	303	14
4310	RVRFFFPSLREAALR	DRB1*11:37	P43355	MAGE1	289	303	22.26
4311	RVRFFFPSLREAALR	DRB1*11:42	P43355	MAGE1	289	303	39.93
4312	RVRFFFPSLREAALR	DRB1*11:46	P43355	MAGE1	289	303	32.94
4313	RVRFFFPSLREAALR	DRB1*11:49	P43355	MAGE1	289	303	14
4314	RVRFFFPSLREAALR	DRB1*11:58	P43355	MAGE1	289	303	32.94
4315	RVRFFFPSLREAALR	DRB1*11:62	P43355	MAGE1	289	303	14
4316	RVRFFFPSLREAALR	DRB1*11:74	P43355	MAGE1	289	303	14

4317	RVRFFFPSLREAALR	DRB1*13:05	P43355	MAGE1	289	303	14
4318	RVRFFFPSLREAALR	DRB1*13:07	P43355	MAGE1	289	303	22.26
4319	RVRFFFPSLREAALR	DRB1*13:11	P43355	MAGE1	289	303	32.94
4320	RVRFFFPSLREAALR	DRB1*13:14	P43355	MAGE1	289	303	14
4321	RVRFFFPSLREAALR	DRB1*13:21	P43355	MAGE1	289	303	16.73
4322	RVRFFFPSLREAALR	DRB1*13:50	P43355	MAGE1	289	303	14
4323	RVRIAYPSLREAALL	DRB1*01:20	P43358	MAGE4	297	311	41.6
4324	RYEFLWGPRALAETS	DRB1*01:01	P43355	MAGE1	261	275	11.06
4325	RYEFLWGPRALAETS	DRB1*01:01	P43358	MAGE4	269	283	11.06
4326	RYEFLWGPRALAETS	DRB1*01:11	P43355	MAGE1	261	275	42.97
4327	RYEFLWGPRALAETS	DRB1*01:11	P43358	MAGE4	269	283	42.97
4328	RYEFLWGPRALAETS	DRB1*01:18	P43355	MAGE1	261	275	10.59
4329	RYEFLWGPRALAETS	DRB1*01:18	P43358	MAGE4	269	283	10.59
4330	RYEFLWGPRALAETS	DRB1*01:20	P43355	MAGE1	261	275	33.24
4331	RYEFLWGPRALAETS	DRB1*01:20	P43358	MAGE4	269	283	33.24
4332	RYEFLWGPRALAETS	DRB1*01:24	P43355	MAGE1	261	275	29.01
4333	RYEFLWGPRALAETS	DRB1*01:24	P43358	MAGE4	269	283	29.01
4334	RYEFLWGPRALAETS	DRB1*01:29	P43355	MAGE1	261	275	26.23
4335	RYEFLWGPRALAETS	DRB1*01:29	P43358	MAGE4	269	283	26.23
4336	RYEFLWGPRALAETS	DRB1*10:01	P43355	MAGE1	261	275	26.6
4337	RYEFLWGPRALAETS	DRB1*10:01	P43358	MAGE4	269	283	26.6
4338	RYEFLWGPRALAETS	DRB1*11:01	P43355	MAGE1	261	275	34.81
4339	RYEFLWGPRALAETS	DRB1*11:01	P43358	MAGE4	269	283	34.81
4340	RYEFLWGPRALAETS	DRB1*11:10	P43355	MAGE1	261	275	34.81
4341	RYEFLWGPRALAETS	DRB1*11:10	P43358	MAGE4	269	283	34.81
4342	RYEFLWGPRALAETS	DRB1*11:12	P43355	MAGE1	261	275	34.81
4343	RYEFLWGPRALAETS	DRB1*11:12	P43358	MAGE4	269	283	34.81
4344	RYEFLWGPRALAETS	DRB1*11:28	P43355	MAGE1	261	275	34.81
4345	RYEFLWGPRALAETS	DRB1*11:28	P43358	MAGE4	269	283	34.81
4346	RYEFLWGPRALAETS	DRB1*11:29	P43355	MAGE1	261	275	34.81
4347	RYEFLWGPRALAETS	DRB1*11:29	P43358	MAGE4	269	283	34.81
4348	RYEFLWGPRALAETS	DRB1*11:49	P43355	MAGE1	261	275	34.81
4349	RYEFLWGPRALAETS	DRB1*11:49	P43358	MAGE4	269	283	34.81
4350	RYEFLWGPRALAETS	DRB1*11:62	P43355	MAGE1	261	275	34.81
4351	RYEFLWGPRALAETS	DRB1*11:62	P43358	MAGE4	269	283	34.81
4352	RYEFLWGPRALAETS	DRB1*11:74	P43355	MAGE1	261	275	34.81
4353	RYEFLWGPRALAETS	DRB1*11:74	P43358	MAGE4	269	283	34.81
4354	RYEFLWGPRALAETS	DRB1*13:05	P43355	MAGE1	261	275	34.81
4355	RYEFLWGPRALAETS	DRB1*13:05	P43358	MAGE4	269	283	34.81
4356	RYEFLWGPRALAETS	DRB1*13:14	P43355	MAGE1	261	275	34.81

4357	RYEFLWGPRALAETS	DRB1*13:14	P43358	MAGE4	269	283	34.81
4358	RYEFLWGPRALAETS	DRB1*13:21	P43355	MAGE1	261	275	47.73
4359	RYEFLWGPRALAETS	DRB1*13:21	P43358	MAGE4	269	283	47.73
4360	RYEFLWGPRALAETS	DRB1*13:50	P43355	MAGE1	261	275	34.81
4361	RYEFLWGPRALAETS	DRB1*13:50	P43358	MAGE4	269	283	34.81
4362	SAPEKDKFFAYLTLA	DPB1*33:01	P14679	Tyrosinase	127	141	39.98
4363	SAPEKDKFFAYLTLA	DPB1*71:01	P14679	Tyrosinase	127	141	39.98
4364	SAQLLQARLMKEESP	DRB1*01:01	Q13072	BAGE	11	25	38.35
4365	SAQLLQARLMKEESP	DRB1*01:18	Q13072	BAGE	11	25	34.26
4366	SAQLLQARLMKEESP	DRB1*01:20	Q13072	BAGE	11	25	29.09
4367	SARVRFFFPSLREAA	DPB1*33:01	P43355	MAGE1	287	301	39.28
4368	SARVRFFFPSLREAA	DPB1*71:01	P43355	MAGE1	287	301	39.28
4369	SARVRFFFPSLREAA	DRB1*01:18	P43355	MAGE1	287	301	49.9
4370	SARVRFFFPSLREAA	DRB1*01:20	P43355	MAGE1	287	301	33.55
4371	SARVRFFFPSLREAA	DRB1*10:01	P43355	MAGE1	287	301	37.62
4372	SARVRFFFPSLREAA	DRB1*11:01	P43355	MAGE1	287	301	49.09
4373	SARVRFFFPSLREAA	DRB1*11:02	P43355	MAGE1	287	301	44.9
4374	SARVRFFFPSLREAA	DRB1*11:03	P43355	MAGE1	287	301	43.74
4375	SARVRFFFPSLREAA	DRB1*11:10	P43355	MAGE1	287	301	49.09
4376	SARVRFFFPSLREAA	DRB1*11:12	P43355	MAGE1	287	301	49.09
4377	SARVRFFFPSLREAA	DRB1*11:13	P43355	MAGE1	287	301	45.95
4378	SARVRFFFPSLREAA	DRB1*11:28	P43355	MAGE1	287	301	49.09
4379	SARVRFFFPSLREAA	DRB1*11:29	P43355	MAGE1	287	301	49.09
4380	SARVRFFFPSLREAA	DRB1*11:42	P43355	MAGE1	287	301	42.54
4381	SARVRFFFPSLREAA	DRB1*11:49	P43355	MAGE1	287	301	49.09
4382	SARVRFFFPSLREAA	DRB1*11:62	P43355	MAGE1	287	301	49.09
4383	SARVRFFFPSLREAA	DRB1*11:65	P43355	MAGE1	287	301	44.9
4384	SARVRFFFPSLREAA	DRB1*11:74	P43355	MAGE1	287	301	49.09
4385	SARVRFFFPSLREAA	DRB1*13:01	P43355	MAGE1	287	301	44.9
4386	SARVRFFFPSLREAA	DRB1*13:05	P43355	MAGE1	287	301	49.09
4387	SARVRFFFPSLREAA	DRB1*13:14	P43355	MAGE1	287	301	49.09
4388	SARVRFFFPSLREAA	DRB1*13:21	P43355	MAGE1	287	301	29.54
4389	SARVRFFFPSLREAA	DRB1*13:50	P43355	MAGE1	287	301	49.09
4390	SARVRFFFPSLREAA	DRB1*15:01	P43355	MAGE1	287	301	38.73
4391	SARVRFFFPSLREAA	DRB1*15:03	P43355	MAGE1	287	301	42.04
4392	SARVRFFFPSLREAA	DRB1*15:06	P43355	MAGE1	287	301	38.73
4393	SCILESLFRAVITKK	DPB1*02:01	P43355	MAGE1	91	105	21.1
4394	SCILESLFRAVITKK	DPB1*02:02	P43355	MAGE1	91	105	21.07
4395	SCILESLFRAVITKK	DPB1*33:01	P43355	MAGE1	91	105	12.55
4396	SCILESLFRAVITKK	DPB1*46:01	P43355	MAGE1	91	105	21.1

4397	SCILESLFRAVITKK	DPB1*47:01	P43355	MAGE1	91	105	21.07
4398	SCILESLFRAVITKK	DPB1*71:01	P43355	MAGE1	91	105	12.55
4399	SCILESLFRAVITKK	DPB1*81:01	P43355	MAGE1	91	105	21.1
4400	SCILESLFRAVITKK	DRB1*11:13	P43355	MAGE1	91	105	43.25
4401	SCILESLFRAVITKK	DRB1*11:42	P43355	MAGE1	91	105	41.73
4402	SCILESLFRAVITKK	DRB1*14:32	P43355	MAGE1	91	105	42.62
4403	SCQNILLSNAPLGPQ	DRB1*01:01	P14679	Tyrosinase	54	68	30.55
4404	SCQNILLSNAPLGPQ	DRB1*01:18	P14679	Tyrosinase	54	68	25.9
4405	SCQNILLSNAPLGPQ	DRB1*01:20	P14679	Tyrosinase	54	68	23.93
4406	SCQNILLSNAPLGPQ	DRB1*11:14	P14679	Tyrosinase	54	68	41.65
4407	SCQNILLSNAPLGPQ	DRB1*13:02	P14679	Tyrosinase	54	68	41.65
4408	SCQNILLSNAPLGPQ	DRB1*13:23	P14679	Tyrosinase	54	68	41.65
4409	SCQNILLSNAPLGPQ	DRB1*13:97	P14679	Tyrosinase	54	68	41.65
4410	SDPARYEFLWGPRAL	DRB1*01:01	P43355	MAGE1	257	271	9.72
4411	SDPARYEFLWGPRAL	DRB1*01:11	P43355	MAGE1	257	271	38.87
4412	SDPARYEFLWGPRAL	DRB1*01:18	P43355	MAGE1	257	271	10.11
4413	SDPARYEFLWGPRAL	DRB1*01:20	P43355	MAGE1	257	271	36.34
4414	SDPARYEFLWGPRAL	DRB1*01:24	P43355	MAGE1	257	271	25.56
4415	SDPARYEFLWGPRAL	DRB1*01:29	P43355	MAGE1	257	271	25.85
4416	SDPARYEFLWGPRAL	DRB1*10:01	P43355	MAGE1	257	271	24.12
4417	SEKIFYVYMKRKYEA	DRB1*08:01	Q16385	SSX2	43	57	45.25
4418	SEKIFYVYMKRKYEA	DRB1*11:01	Q16385	SSX2	43	57	15.39
4419	SEKIFYVYMKRKYEA	DRB1*11:02	Q16385	SSX2	43	57	37.1
4420	SEKIFYVYMKRKYEA	DRB1*11:03	Q16385	SSX2	43	57	17.36
4421	SEKIFYVYMKRKYEA	DRB1*11:04	Q16385	SSX2	43	57	16.45
4422	SEKIFYVYMKRKYEA	DRB1*11:08	Q16385	SSX2	43	57	38.7
4423	SEKIFYVYMKRKYEA	DRB1*11:10	Q16385	SSX2	43	57	15.39
4424	SEKIFYVYMKRKYEA	DRB1*11:11	Q16385	SSX2	43	57	34.6
4425	SEKIFYVYMKRKYEA	DRB1*11:12	Q16385	SSX2	43	57	15.39
4426	SEKIFYVYMKRKYEA	DRB1*11:13	Q16385	SSX2	43	57	37.35
4427	SEKIFYVYMKRKYEA	DRB1*11:28	Q16385	SSX2	43	57	15.39
4428	SEKIFYVYMKRKYEA	DRB1*11:29	Q16385	SSX2	43	57	15.39
4429	SEKIFYVYMKRKYEA	DRB1*11:37	Q16385	SSX2	43	57	37.23
4430	SEKIFYVYMKRKYEA	DRB1*11:42	Q16385	SSX2	43	57	20.63
4431	SEKIFYVYMKRKYEA	DRB1*11:46	Q16385	SSX2	43	57	16.45
4432	SEKIFYVYMKRKYEA	DRB1*11:49	Q16385	SSX2	43	57	15.39
4433	SEKIFYVYMKRKYEA	DRB1*11:58	Q16385	SSX2	43	57	16.45
4434	SEKIFYVYMKRKYEA	DRB1*11:62	Q16385	SSX2	43	57	15.39
4435	SEKIFYVYMKRKYEA	DRB1*11:65	Q16385	SSX2	43	57	37.1
4436	SEKIFYVYMKRKYEA	DRB1*11:74	Q16385	SSX2	43	57	15.39

4437	SEKIFYVYMKRKYEA	DRB1*11:84	Q16385	SSX2	43	57	46.83
4438	SEKIFYVYMKRKYEA	DRB1*13:01	Q16385	SSX2	43	57	37.1
4439	SEKIFYVYMKRKYEA	DRB1*13:05	Q16385	SSX2	43	57	15.39
4440	SEKIFYVYMKRKYEA	DRB1*13:07	Q16385	SSX2	43	57	37.23
4441	SEKIFYVYMKRKYEA	DRB1*13:11	Q16385	SSX2	43	57	16.45
4442	SEKIFYVYMKRKYEA	DRB1*13:14	Q16385	SSX2	43	57	15.39
4443	SEKIFYVYMKRKYEA	DRB1*13:21	Q16385	SSX2	43	57	9.35
4444	SEKIFYVYMKRKYEA	DRB1*13:50	Q16385	SSX2	43	57	15.39
4445	SELKELINNELSHFL	DRB1*01:18	P04271	S100	29	43	40.69
4446	SELKELINNELSHFL	DRB1*01:20	P04271	S100	29	43	32.83
4447	SELKELINNELSHFL	DRB1*11:14	P04271	S100	29	43	34.41
4448	SELKELINNELSHFL	DRB1*13:02	P04271	S100	29	43	34.41
4449	SELKELINNELSHFL	DRB1*13:23	P04271	S100	29	43	34.41
4450	SELKELINNELSHFL	DRB1*13:96	P04271	S100	29	43	44.29
4451	SELKELINNELSHFL	DRB1*13:97	P04271	S100	29	43	34.41
4452	SESLKMIFGIDVKEV	DRB1*01:01	P43358	MAGE4	156	170	9.37
4453	SESLKMIFGIDVKEV	DRB1*01:02	P43358	MAGE4	156	170	24.76
4454	SESLKMIFGIDVKEV	DRB1*01:11	P43358	MAGE4	156	170	26.37
4455	SESLKMIFGIDVKEV	DRB1*01:18	P43358	MAGE4	156	170	9.27
4456	SESLKMIFGIDVKEV	DRB1*01:20	P43358	MAGE4	156	170	8.1
4457	SESLKMIFGIDVKEV	DRB1*01:24	P43358	MAGE4	156	170	17.73
4458	SESLKMIFGIDVKEV	DRB1*01:29	P43358	MAGE4	156	170	18.33
4459	SESLKMIFGIDVKEV	DRB1*07:01	P43358	MAGE4	156	170	44.46
4460	SESLKMIFGIDVKEV	DRB1*10:01	P43358	MAGE4	156	170	47.27
4461	SESLKMIFGIDVKEV	DRB1*15:01	P43358	MAGE4	156	170	39.91
4462	SESLKMIFGIDVKEV	DRB1*15:06	P43358	MAGE4	156	170	39.91
4463	SESLQLVFGIDVKEA	DRB1*01:01	P43355	MAGE1	148	162	22.5
4464	SESLQLVFGIDVKEA	DRB1*01:18	P43355	MAGE1	148	162	18.19
4465	SESLQLVFGIDVKEA	DRB1*01:20	P43355	MAGE1	148	162	17.94
4466	SESLQLVFGIDVKEA	DRB1*01:24	P43355	MAGE1	148	162	42.19
4467	SESLQLVFGIDVKEA	DRB1*01:29	P43355	MAGE1	148	162	43.62
4468	SFSIALNFPGSQKVL	DRB1*01:01	P40967	PMEL17	83	97	16.75
4469	SFSIALNFPGSQKVL	DRB1*01:18	P40967	PMEL17	83	97	15.18
4470	SFSIALNFPGSQKVL	DRB1*01:20	P40967	PMEL17	83	97	30.68
4471	SFSIALNFPGSQKVL	DRB1*01:24	P40967	PMEL17	83	97	36.75
4472	SFSIALNFPGSQKVL	DRB1*01:29	P40967	PMEL17	83	97	43.56
4473	SFSIALNFPGSQKVL	DRB1*04:01	P40967	PMEL17	83	97	26.88
4474	SFSIALNFPGSQKVL	DRB1*04:08	P40967	PMEL17	83	97	28.57
4475	SFSIALNFPGSQKVL	DRB1*10:01	P40967	PMEL17	83	97	18.44
4476	SFSIALNFPGSQKVL	DRB1*11:14	P40967	PMEL17	83	97	27.92

4477	SFSIALNFPGSQKVL	DRB1*13:02	P40967	PMEL17	83	97	27.92
4478	SFSIALNFPGSQKVL	DRB1*13:23	P40967	PMEL17	83	97	27.92
4479	SFSIALNFPGSQKVL	DRB1*13:96	P40967	PMEL17	83	97	42.8
4480	SFSIALNFPGSQKVL	DRB1*13:97	P40967	PMEL17	83	97	27.92
4481	SFSLPYWNFATGKNV	DRB1*09:01	P17643	TRP1	243	257	37.71
4482	SFSVTLDIVQGIESA	DRB1*03:11	P40967	PMEL17	482	496	35.19
4483	SGLSIGTGRAMLGTH	DRB1*01:01	P40967	PMEL17	168	182	44.21
4484	SGLSIGTGRAMLGTH	DRB1*01:20	P40967	PMEL17	168	182	31.6
4485	SGNILTIRLTAADHR	DRB1*01:20	P78358	NY-ESO-1	129	143	34.64
4486	SGNILTIRLTAADHR	DRB1*11:04	P78358	NY-ESO-1	129	143	45.87
4487	SGNILTIRLTAADHR	DRB1*11:13	P78358	NY-ESO-1	129	143	47.37
4488	SGNILTIRLTAADHR	DRB1*11:42	P78358	NY-ESO-1	129	143	33.74
4489	SGNILTIRLTAADHR	DRB1*11:46	P78358	NY-ESO-1	129	143	45.87
4490	SGNILTIRLTAADHR	DRB1*11:58	P78358	NY-ESO-1	129	143	45.87
4491	SGNILTIRLTAADHR	DRB1*13:11	P78358	NY-ESO-1	129	143	45.87
4492	SGTLISRALVVTHTY	DRB1*01:01	P40967	PMEL17	266	280	33.49
4493	SGTLISRALVVTHTY	DRB1*01:18	P40967	PMEL17	266	280	29
4494	SGTLISRALVVTHTY	DRB1*01:20	P40967	PMEL17	266	280	24.5
4495	SGTLISRALVVTHTY	DRB1*07:01	P40967	PMEL17	266	280	33.04
4496	SGTLISRALVVTHTY	DRB1*10:01	P40967	PMEL17	266	280	46.65
4497	SIFEQWLRRHRPLQE	DRB1*11:03	P14679	Tyrosinase	395	409	48.36
4498	SIVVLSGTTAAQVTT	DRB1*01:01	P40967	PMEL17	406	420	11.8
4499	SIVVLSGTTAAQVTT	DRB1*01:02	P40967	PMEL17	406	420	40.28
4500	SIVVLSGTTAAQVTT	DRB1*01:11	P40967	PMEL17	406	420	30.24
4501	SIVVLSGTTAAQVTT	DRB1*01:18	P40967	PMEL17	406	420	11.14
4502	SIVVLSGTTAAQVTT	DRB1*01:20	P40967	PMEL17	406	420	11.66
4503	SIVVLSGTTAAQVTT	DRB1*01:24	P40967	PMEL17	406	420	23.48
4504	SIVVLSGTTAAQVTT	DRB1*01:29	P40967	PMEL17	406	420	26.34
4505	SIVVLSGTTAAQVTT	DRB1*04:04	P40967	PMEL17	406	420	34.56
4506	SIVVLSGTTAAQVTT	DRB1*07:01	P40967	PMEL17	406	420	30.64
4507	SIVVLSGTTAAQVTT	DRB1*10:01	P40967	PMEL17	406	420	44.05
4508	SIYNYFVWTHYYSVK	DPB1*01:01	P17643	TRP1	183	197	17.3
4509	SIYNYFVWTHYYSVK	DPB1*02:01	P17643	TRP1	183	197	5.66
4510	SIYNYFVWTHYYSVK	DPB1*02:02	P17643	TRP1	183	197	6.31
4511	SIYNYFVWTHYYSVK	DPB1*04:01	P17643	TRP1	183	197	5.03
4512	SIYNYFVWTHYYSVK	DPB1*04:02	P17643	TRP1	183	197	18.01
4513	SIYNYFVWTHYYSVK	DPB1*105:01	P17643	TRP1	183	197	18.01
4514	SIYNYFVWTHYYSVK	DPB1*126:01	P17643	TRP1	183	197	5.03
4515	SIYNYFVWTHYYSVK	DPB1*15:01	P17643	TRP1	183	197	16.92
4516	SIYNYFVWTHYYSVK	DPB1*16:01	P17643	TRP1	183	197	19.26

4517	SIYNYFVWTHYYSVK	DPB1*19:01	P17643	TRP1	183	197	32.27
4518	SIYNYFVWTHYYSVK	DPB1*23:01	P17643	TRP1	183	197	5.03
4519	SIYNYFVWTHYYSVK	DPB1*33:01	P17643	TRP1	183	197	3.78
4520	SIYNYFVWTHYYSVK	DPB1*34:01	P17643	TRP1	183	197	25.95
4521	SIYNYFVWTHYYSVK	DPB1*39:01	P17643	TRP1	183	197	5.03
4522	SIYNYFVWTHYYSVK	DPB1*40:01	P17643	TRP1	183	197	21.08
4523	SIYNYFVWTHYYSVK	DPB1*41:01	P17643	TRP1	183	197	18.13
4524	SIYNYFVWTHYYSVK	DPB1*46:01	P17643	TRP1	183	197	5.66
4525	SIYNYFVWTHYYSVK	DPB1*47:01	P17643	TRP1	183	197	6.31
4526	SIYNYFVWTHYYSVK	DPB1*49:01	P17643	TRP1	183	197	18.01
4527	SIYNYFVWTHYYSVK	DPB1*71:01	P17643	TRP1	183	197	3.78
4528	SIYNYFVWTHYYSVK	DPB1*72:01	P17643	TRP1	183	197	6.08
4529	SIYNYFVWTHYYSVK	DPB1*81:01	P17643	TRP1	183	197	5.66
4530	SIYNYFVWTHYYSVK	DRB1*01:18	P17643	TRP1	183	197	44.66
4531	SIYNYFVWTHYYSVK	DRB1*07:01	P17643	TRP1	183	197	25.38
4532	SLFRAVITKKVADLV	DRB1*01:01	P43355	MAGE1	96	110	6.68
4533	SLFRAVITKKVADLV	DRB1*01:11	P43355	MAGE1	96	110	16.39
4534	SLFRAVITKKVADLV	DRB1*01:18	P43355	MAGE1	96	110	6.08
4535	SLFRAVITKKVADLV	DRB1*01:20	P43355	MAGE1	96	110	18.38
4536	SLFRAVITKKVADLV	DRB1*01:24	P43355	MAGE1	96	110	12.74
4537	SLFRAVITKKVADLV	DRB1*01:29	P43355	MAGE1	96	110	10.47
4538	SLFRAVITKKVADLV	DRB1*07:01	P43355	MAGE1	96	110	8.83
4539	SLFRAVITKKVADLV	DRB1*09:01	P43355	MAGE1	96	110	22.79
4540	SLFRAVITKKVADLV	DRB1*10:01	P43355	MAGE1	96	110	15.67
4541	SLFRAVITKKVADLV	DRB1*11:01	P43355	MAGE1	96	110	32.18
4542	SLFRAVITKKVADLV	DRB1*11:04	P43355	MAGE1	96	110	44.25
4543	SLFRAVITKKVADLV	DRB1*11:08	P43355	MAGE1	96	110	41.43
4544	SLFRAVITKKVADLV	DRB1*11:10	P43355	MAGE1	96	110	32.18
4545	SLFRAVITKKVADLV	DRB1*11:12	P43355	MAGE1	96	110	32.18
4546	SLFRAVITKKVADLV	DRB1*11:28	P43355	MAGE1	96	110	32.18
4547	SLFRAVITKKVADLV	DRB1*11:29	P43355	MAGE1	96	110	32.18
4548	SLFRAVITKKVADLV	DRB1*11:42	P43355	MAGE1	96	110	35.07
4549	SLFRAVITKKVADLV	DRB1*11:46	P43355	MAGE1	96	110	44.25
4550	SLFRAVITKKVADLV	DRB1*11:49	P43355	MAGE1	96	110	32.18
4551	SLFRAVITKKVADLV	DRB1*11:58	P43355	MAGE1	96	110	44.25
4552	SLFRAVITKKVADLV	DRB1*11:62	P43355	MAGE1	96	110	32.18
4553	SLFRAVITKKVADLV	DRB1*11:74	P43355	MAGE1	96	110	32.18
4554	SLFRAVITKKVADLV	DRB1*12:16	P43355	MAGE1	96	110	44.96
4555	SLFRAVITKKVADLV	DRB1*13:05	P43355	MAGE1	96	110	32.18
4556	SLFRAVITKKVADLV	DRB1*13:11	P43355	MAGE1	96	110	44.25

4557	SLFRAVITKKVADLV	DRB1*13:14	P43355	MAGE1	96	110	32.18
4558	SLFRAVITKKVADLV	DRB1*13:21	P43355	MAGE1	96	110	27.52
4559	SLFRAVITKKVADLV	DRB1*13:50	P43355	MAGE1	96	110	32.18
4560	SLFRAVITKKVADLV	DRB1*15:15	P43355	MAGE1	96	110	24.66
4561	SLFRAVITKKVADLV	DRB1*16:01	P43355	MAGE1	96	110	18.93
4562	SLFRAVITKKVADLV	DRB1*16:02	P43355	MAGE1	96	110	21.29
4563	SLFRAVITKKVADLV	DRB1*16:04	P43355	MAGE1	96	110	29.92
4564	SLFRAVITKKVADLV	DRB1*16:05	P43355	MAGE1	96	110	43.91
4565	SLFRAVITKKVADLV	DRB1*16:09	P43355	MAGE1	96	110	17.72
4566	SLFREALSNKVDELA	DRB1*01:01	P43358	MAGE4	104	118	4.65
4567	SLFREALSNKVDELA	DRB1*01:11	P43358	MAGE4	104	118	11.61
4568	SLFREALSNKVDELA	DRB1*01:18	P43358	MAGE4	104	118	5.1
4569	SLFREALSNKVDELA	DRB1*01:20	P43358	MAGE4	104	118	17.43
4570	SLFREALSNKVDELA	DRB1*01:24	P43358	MAGE4	104	118	10.49
4571	SLFREALSNKVDELA	DRB1*01:29	P43358	MAGE4	104	118	9.83
4572	SLFREALSNKVDELA	DRB1*07:01	P43358	MAGE4	104	118	7.45
4573	SLFREALSNKVDELA	DRB1*09:01	P43358	MAGE4	104	118	11.5
4574	SLFREALSNKVDELA	DRB1*10:01	P43358	MAGE4	104	118	14.76
4575	SLFREALSNKVDELA	DRB1*15:15	P43358	MAGE4	104	118	42.09
4576	SLFREALSNKVDELA	DRB1*16:01	P43358	MAGE4	104	118	37.34
4577	SLFREALSNKVDELA	DRB1*16:02	P43358	MAGE4	104	118	20.15
4578	SLFREALSNKVDELA	DRB1*16:05	P43358	MAGE4	104	118	49.13
4579	SLGCIFFPLLLFQQA	DPB1*33:01	P17643	TRP1	8	22	47.48
4580	SLGCIFFPLLLFQQA	DPB1*71:01	P17643	TRP1	8	22	47.48
4581	SLIYRRRLMKQDFSV	DRB1*11:02	P40967	PMEL17	613	627	44.49
4582	SLIYRRRLMKQDFSV	DRB1*11:03	P40967	PMEL17	613	627	23.8
4583	SLIYRRRLMKQDFSV	DRB1*11:65	P40967	PMEL17	613	627	44.49
4584	SLIYRRRLMKQDFSV	DRB1*13:01	P40967	PMEL17	613	627	44.49
4585	SLKMIFGIDVKEVDP	DRB1*01:01	P43358	MAGE4	158	172	23.9
4586	SLKMIFGIDVKEVDP	DRB1*01:02	P43358	MAGE4	158	172	45.29
4587	SLKMIFGIDVKEVDP	DRB1*01:18	P43358	MAGE4	158	172	18.44
4588	SLKMIFGIDVKEVDP	DRB1*01:20	P43358	MAGE4	158	172	18.88
4589	SLKMIFGIDVKEVDP	DRB1*01:24	P43358	MAGE4	158	172	35.57
4590	SLKMIFGIDVKEVDP	DRB1*01:29	P43358	MAGE4	158	172	48.41
4591	SLLMWITQCFLPVFL	DPB1*04:01	P78358	NY-ESO-1	157	171	27.58
4592	SLLMWITQCFLPVFL	DPB1*126:01	P78358	NY-ESO-1	157	171	27.58
4593	SLLMWITQCFLPVFL	DPB1*23:01	P78358	NY-ESO-1	157	171	27.58
4594	SLLMWITQCFLPVFL	DPB1*33:01	P78358	NY-ESO-1	157	171	21.9
4595	SLLMWITQCFLPVFL	DPB1*39:01	P78358	NY-ESO-1	157	171	27.58
4596	SLLMWITQCFLPVFL	DPB1*71:01	P78358	NY-ESO-1	157	171	21.9

4597	SLPYWNFATGKNVCD	DRB1*01:01	P17643	TRP1	245	259	34.48
4598	SLPYWNFATGKNVCD	DRB1*01:18	P17643	TRP1	245	259	37.59
4599	SLQLVFGIDVKEADP	DRB1*01:18	P43355	MAGE1	150	164	47.49
4600	SMHNALHIYMNGTMS	DRB1*15:01	P14679	Tyrosinase	361	375	30.89
4601	SMHNALHIYMNGTMS	DRB1*15:06	P14679	Tyrosinase	361	375	30.89
4602	SNPARYEFLWGPRAL	DRB1*01:01	P43358	MAGE4	265	279	9.72
4603	SNPARYEFLWGPRAL	DRB1*01:11	P43358	MAGE4	265	279	38.87
4604	SNPARYEFLWGPRAL	DRB1*01:18	P43358	MAGE4	265	279	10.11
4605	SNPARYEFLWGPRAL	DRB1*01:20	P43358	MAGE4	265	279	36.34
4606	SNPARYEFLWGPRAL	DRB1*01:24	P43358	MAGE4	265	279	25.56
4607	SNPARYEFLWGPRAL	DRB1*01:29	P43358	MAGE4	265	279	25.85
4608	SNPARYEFLWGPRAL	DRB1*10:01	P43358	MAGE4	265	279	24.12
4609	SPASFFSSWQIVCSR	DPB1*33:01	P14679	Tyrosinase	264	278	49
4610	SPASFFSSWQIVCSR	DPB1*71:01	P14679	Tyrosinase	264	278	49
4611	SPNDPIFVLLHTFTD	DRB1*04:05	P17643	TRP1	394	408	19.43
4612	SPNDPIFVLLHTFTD	DRB1*10:01	P17643	TRP1	394	408	32.93
4613	SQKRSFVYVWKTWGQ	DRB1*11:01	P40967	PMEL17	144	158	21.74
4614	SQKRSFVYVWKTWGQ	DRB1*11:04	P40967	PMEL17	144	158	46.19
4615	SQKRSFVYVWKTWGQ	DRB1*11:10	P40967	PMEL17	144	158	21.74
4616	SQKRSFVYVWKTWGQ	DRB1*11:12	P40967	PMEL17	144	158	21.74
4617	SQKRSFVYVWKTWGQ	DRB1*11:28	P40967	PMEL17	144	158	21.74
4618	SQKRSFVYVWKTWGQ	DRB1*11:29	P40967	PMEL17	144	158	21.74
4619	SQKRSFVYVWKTWGQ	DRB1*11:37	P40967	PMEL17	144	158	49.54
4620	SQKRSFVYVWKTWGQ	DRB1*11:46	P40967	PMEL17	144	158	46.19
4621	SQKRSFVYVWKTWGQ	DRB1*11:49	P40967	PMEL17	144	158	21.74
4622	SQKRSFVYVWKTWGQ	DRB1*11:58	P40967	PMEL17	144	158	46.19
4623	SQKRSFVYVWKTWGQ	DRB1*11:62	P40967	PMEL17	144	158	21.74
4624	SQKRSFVYVWKTWGQ	DRB1*11:74	P40967	PMEL17	144	158	21.74
4625	SQKRSFVYVWKTWGQ	DRB1*13:05	P40967	PMEL17	144	158	21.74
4626	SQKRSFVYVWKTWGQ	DRB1*13:07	P40967	PMEL17	144	158	49.54
4627	SQKRSFVYVWKTWGQ	DRB1*13:11	P40967	PMEL17	144	158	46.19
4628	SQKRSFVYVWKTWGQ	DRB1*13:14	P40967	PMEL17	144	158	21.74
4629	SQKRSFVYVWKTWGQ	DRB1*13:21	P40967	PMEL17	144	158	32.23
4630	SQKRSFVYVWKTWGQ	DRB1*13:50	P40967	PMEL17	144	158	21.74
4631	SRIWSWLLGAAMVGA	DRB1*01:01	P14679	Tyrosinase	472	486	8.69
4632	SRIWSWLLGAAMVGA	DRB1*01:11	P14679	Tyrosinase	472	486	22.17
4633	SRIWSWLLGAAMVGA	DRB1*01:18	P14679	Tyrosinase	472	486	7.97
4634	SRIWSWLLGAAMVGA	DRB1*01:20	P14679	Tyrosinase	472	486	26.76
4635	SRIWSWLLGAAMVGA	DRB1*01:24	P14679	Tyrosinase	472	486	14.32
4636	SRIWSWLLGAAMVGA	DRB1*01:29	P14679	Tyrosinase	472	486	15.72

4637	SRIWSWLLGAAMVGA	DRB1*10:01	P14679	Tyrosinase	472	486	25.36
4638	SRLLEFYLAMPFATP	DPB1*01:01	P78358	NY-ESO-1	85	99	42.44
4639	SRLLEFYLAMPFATP	DPB1*02:01	P78358	NY-ESO-1	85	99	47.03
4640	SRLLEFYLAMPFATP	DPB1*02:02	P78358	NY-ESO-1	85	99	45.05
4641	SRLLEFYLAMPFATP	DPB1*04:01	P78358	NY-ESO-1	85	99	46.39
4642	SRLLEFYLAMPFATP	DPB1*126:01	P78358	NY-ESO-1	85	99	46.39
4643	SRLLEFYLAMPFATP	DPB1*15:01	P78358	NY-ESO-1	85	99	36.24
4644	SRLLEFYLAMPFATP	DPB1*23:01	P78358	NY-ESO-1	85	99	46.39
4645	SRLLEFYLAMPFATP	DPB1*33:01	P78358	NY-ESO-1	85	99	16.88
4646	SRLLEFYLAMPFATP	DPB1*39:01	P78358	NY-ESO-1	85	99	46.39
4647	SRLLEFYLAMPFATP	DPB1*46:01	P78358	NY-ESO-1	85	99	47.03
4648	SRLLEFYLAMPFATP	DPB1*47:01	P78358	NY-ESO-1	85	99	45.05
4649	SRLLEFYLAMPFATP	DPB1*71:01	P78358	NY-ESO-1	85	99	16.88
4650	SRLLEFYLAMPFATP	DPB1*72:01	P78358	NY-ESO-1	85	99	41.92
4651	SRLLEFYLAMPFATP	DPB1*81:01	P78358	NY-ESO-1	85	99	47.03
4652	SRLLEFYLAMPFATP	DRB1*01:01	P78358	NY-ESO-1	85	99	9.86
4653	SRLLEFYLAMPFATP	DRB1*01:11	P78358	NY-ESO-1	85	99	22.39
4654	SRLLEFYLAMPFATP	DRB1*01:18	P78358	NY-ESO-1	85	99	9.5
4655	SRLLEFYLAMPFATP	DRB1*01:20	P78358	NY-ESO-1	85	99	26.35
4656	SRLLEFYLAMPFATP	DRB1*01:24	P78358	NY-ESO-1	85	99	13.22
4657	SRLLEFYLAMPFATP	DRB1*01:29	P78358	NY-ESO-1	85	99	16.31
4658	SRLLEFYLAMPFATP	DRB1*07:01	P78358	NY-ESO-1	85	99	45.61
4659	SRLLEFYLAMPFATP	DRB1*09:01	P78358	NY-ESO-1	85	99	29.41
4660	SRLLEFYLAMPFATP	DRB1*10:01	P78358	NY-ESO-1	85	99	10.66
4661	SRLLEFYLAMPFATP	DRB1*15:01	P78358	NY-ESO-1	85	99	29.01
4662	SRLLEFYLAMPFATP	DRB1*15:03	P78358	NY-ESO-1	85	99	48.43
4663	SRLLEFYLAMPFATP	DRB1*15:06	P78358	NY-ESO-1	85	99	29.01
4664	SRLLEFYLAMPFATP	DRB1*15:37	P78358	NY-ESO-1	85	99	49.34
4665	SRSNFDSTLISPNSV	DRB1*10:01	P17643	TRP1	268	282	44.7
4666	SRSYVPLAHSSSAFT	DRB1*01:01	P40967	PMEL17	194	208	31.86
4667	SRSYVPLAHSSSAFT	DRB1*01:18	P40967	PMEL17	194	208	29.32
4668	SRSYVPLAHSSSAFT	DRB1*10:01	P40967	PMEL17	194	208	30.43
4669	SRSYVPLAHSSSAFT	DRB1*11:01	P40967	PMEL17	194	208	39.52
4670	SRSYVPLAHSSSAFT	DRB1*11:10	P40967	PMEL17	194	208	39.52
4671	SRSYVPLAHSSSAFT	DRB1*11:12	P40967	PMEL17	194	208	39.52
4672	SRSYVPLAHSSSAFT	DRB1*11:28	P40967	PMEL17	194	208	39.52
4673	SRSYVPLAHSSSAFT	DRB1*11:29	P40967	PMEL17	194	208	39.52
4674	SRSYVPLAHSSSAFT	DRB1*11:49	P40967	PMEL17	194	208	39.52
4675	SRSYVPLAHSSSAFT	DRB1*11:62	P40967	PMEL17	194	208	39.52
4676	SRSYVPLAHSSSAFT	DRB1*11:74	P40967	PMEL17	194	208	39.52

4677	SRSYVPLAHSSSAFT	DRB1*13:05	P40967	PMEL17	194	208	39.52
4678	SRSYVPLAHSSSAFT	DRB1*13:14	P40967	PMEL17	194	208	39.52
4679	SRSYVPLAHSSSAFT	DRB1*13:50	P40967	PMEL17	194	208	39.52
4680	SSGTLISRALVVTHT	DRB1*01:01	P40967	PMEL17	265	279	32.03
4681	SSGTLISRALVVTHT	DRB1*01:18	P40967	PMEL17	265	279	28.55
4682	SSGTLISRALVVTHT	DRB1*01:20	P40967	PMEL17	265	279	22.66
4683	SSGTLISRALVVTHT	DRB1*07:01	P40967	PMEL17	265	279	26.64
4684	STSCILESLFRAVIT	DPB1*02:01	P43355	MAGE1	89	103	23.29
4685	STSCILESLFRAVIT	DPB1*02:02	P43355	MAGE1	89	103	20.68
4686	STSCILESLFRAVIT	DPB1*33:01	P43355	MAGE1	89	103	11.81
4687	STSCILESLFRAVIT	DPB1*46:01	P43355	MAGE1	89	103	23.29
4688	STSCILESLFRAVIT	DPB1*47:01	P43355	MAGE1	89	103	20.68
4689	STSCILESLFRAVIT	DPB1*71:01	P43355	MAGE1	89	103	11.81
4690	STSCILESLFRAVIT	DPB1*81:01	P43355	MAGE1	89	103	23.29
4691	SVRDTLLGGFFPWLK	DPB1*33:01	O75767	TRP2	192	206	35.17
4692	SVRDTLLGGFFPWLK	DPB1*71:01	O75767	TRP2	192	206	35.17
4693	SVSQLRALDGGNKHF	DRB1*01:20	P40967	PMEL17	218	232	42.72
4694	SVSVSQLRALDGGNK	DRB1*01:18	P40967	PMEL17	216	230	49.57
4695	SVSVSQLRALDGGNK	DRB1*01:20	P40967	PMEL17	216	230	28.54
4696	SVYDFFVWLHYYSVR	DPB1*02:01	O75767	TRP2	180	194	15.36
4697	SVYDFFVWLHYYSVR	DPB1*02:02	O75767	TRP2	180	194	15.68
4698	SVYDFFVWLHYYSVR	DPB1*04:01	O75767	TRP2	180	194	19.26
4699	SVYDFFVWLHYYSVR	DPB1*126:01	O75767	TRP2	180	194	19.26
4700	SVYDFFVWLHYYSVR	DPB1*15:01	O75767	TRP2	180	194	36.05
4701	SVYDFFVWLHYYSVR	DPB1*23:01	O75767	TRP2	180	194	19.26
4702	SVYDFFVWLHYYSVR	DPB1*33:01	O75767	TRP2	180	194	7.64
4703	SVYDFFVWLHYYSVR	DPB1*39:01	O75767	TRP2	180	194	19.26
4704	SVYDFFVWLHYYSVR	DPB1*46:01	O75767	TRP2	180	194	15.36
4705	SVYDFFVWLHYYSVR	DPB1*47:01	O75767	TRP2	180	194	15.68
4706	SVYDFFVWLHYYSVR	DPB1*71:01	O75767	TRP2	180	194	7.64
4707	SVYDFFVWLHYYSVR	DPB1*72:01	O75767	TRP2	180	194	28.48
4708	SVYDFFVWLHYYSVR	DPB1*81:01	O75767	TRP2	180	194	15.36
4709	SVYDFFVWLHYYSVR	DRB1*01:18	O75767	TRP2	180	194	39.51
4710	SWLLGAAMVGAVLTA	DRB1*01:01	P14679	Tyrosinase	476	490	45.12
4711	SWLLGAAMVGAVLTA	DRB1*01:18	P14679	Tyrosinase	476	490	37.57
4712	SYLIRARRSMDEANQ	DRB1*11:04	P17643	TRP1	498	512	39.58
4713	SYLIRARRSMDEANQ	DRB1*11:42	P17643	TRP1	498	512	41.67
4714	SYLIRARRSMDEANQ	DRB1*11:46	P17643	TRP1	498	512	39.58
4715	SYLIRARRSMDEANQ	DRB1*11:58	P17643	TRP1	498	512	39.58
4716	SYLIRARRSMDEANQ	DRB1*13:11	P17643	TRP1	498	512	39.58

4717	SYLIRARRSMDEANQ	DRB1*13:21	P17643	TRP1	498	512	26.61
4718	SYMVPFIPLYRNGDF	DRB1*11:42	P14679	Tyrosinase	424	438	44.75
4719	SYMVPFIPLYRNGDF	DRB1*13:21	P14679	Tyrosinase	424	438	20.22
4720	SYVKVLEHVVRVNAR	DRB1*01:01	P43358	MAGE4	283	297	48.26
4721	SYVKVLEHVVRVNAR	DRB1*01:18	P43358	MAGE4	283	297	31.03
4722	SYVKVLEHVVRVNAR	DRB1*11:01	P43358	MAGE4	283	297	47.81
4723	SYVKVLEHVVRVNAR	DRB1*11:03	P43358	MAGE4	283	297	48.09
4724	SYVKVLEHVVRVNAR	DRB1*11:04	P43358	MAGE4	283	297	21.83
4725	SYVKVLEHVVRVNAR	DRB1*11:10	P43358	MAGE4	283	297	47.81
4726	SYVKVLEHVVRVNAR	DRB1*11:12	P43358	MAGE4	283	297	47.81
4727	SYVKVLEHVVRVNAR	DRB1*11:13	P43358	MAGE4	283	297	30.46
4728	SYVKVLEHVVRVNAR	DRB1*11:28	P43358	MAGE4	283	297	47.81
4729	SYVKVLEHVVRVNAR	DRB1*11:29	P43358	MAGE4	283	297	47.81
4730	SYVKVLEHVVRVNAR	DRB1*11:42	P43358	MAGE4	283	297	23.89
4731	SYVKVLEHVVRVNAR	DRB1*11:46	P43358	MAGE4	283	297	21.83
4732	SYVKVLEHVVRVNAR	DRB1*11:49	P43358	MAGE4	283	297	47.81
4733	SYVKVLEHVVRVNAR	DRB1*11:58	P43358	MAGE4	283	297	21.83
4734	SYVKVLEHVVRVNAR	DRB1*11:62	P43358	MAGE4	283	297	47.81
4735	SYVKVLEHVVRVNAR	DRB1*11:74	P43358	MAGE4	283	297	47.81
4736	SYVKVLEHVVRVNAR	DRB1*13:05	P43358	MAGE4	283	297	47.81
4737	SYVKVLEHVVRVNAR	DRB1*13:11	P43358	MAGE4	283	297	21.83
4738	SYVKVLEHVVRVNAR	DRB1*13:14	P43358	MAGE4	283	297	47.81
4739	SYVKVLEHVVRVNAR	DRB1*13:50	P43358	MAGE4	283	297	47.81
4740	SYVKVLEYVIKVSAR	DRB1*01:18	P43355	MAGE1	275	289	38.68
4741	SYVKVLEYVIKVSAR	DRB1*11:13	P43355	MAGE1	275	289	40.48
4742	SYVKVLEYVIKVSAR	DRB1*11:42	P43355	MAGE1	275	289	39.26
4743	SYVPLAHSSSAFTIT	DRB1*01:01	P40967	PMEL17	196	210	29.38
4744	SYVPLAHSSSAFTIT	DRB1*01:18	P40967	PMEL17	196	210	30.77
4745	SYVPLAHSSSAFTIT	DRB1*01:20	P40967	PMEL17	196	210	37.37
4746	SYVPLAHSSSAFTIT	DRB1*07:01	P40967	PMEL17	196	210	38.1
4747	SYVPLAHSSSAFTIT	DRB1*09:01	P40967	PMEL17	196	210	35.62
4748	SYVPLAHSSSAFTIT	DRB1*10:01	P40967	PMEL17	196	210	33.12
4749	TALLAGLVSLLCRHK	DRB1*01:01	P14679	Tyrosinase	489	503	23.48
4750	TALLAGLVSLLCRHK	DRB1*01:18	P14679	Tyrosinase	489	503	20.32
4751	TALLAGLVSLLCRHK	DRB1*01:20	P14679	Tyrosinase	489	503	17.15
4752	TALLAGLVSLLCRHK	DRB1*01:24	P14679	Tyrosinase	489	503	38.53
4753	TALLAGLVSLLCRHK	DRB1*01:29	P14679	Tyrosinase	489	503	42.73
4754	TALLAGLVSLLCRHK	DRB1*10:01	P14679	Tyrosinase	489	503	48.81
4755	TAQVVLQAAIPLTSC	DRB1*01:01	P40967	PMEL17	287	301	6.93
4756	TAQVVLQAAIPLTSC	DRB1*01:02	P40967	PMEL17	287	301	16.69

4757	TAQVVLQAAIPLTSC	DRB1*01:11	P40967	PMEL17	287	301	14.97
4758	TAQVVLQAAIPLTSC	DRB1*01:18	P40967	PMEL17	287	301	7.07
4759	TAQVVLQAAIPLTSC	DRB1*01:20	P40967	PMEL17	287	301	6.38
4760	TAQVVLQAAIPLTSC	DRB1*01:24	P40967	PMEL17	287	301	13.47
4761	TAQVVLQAAIPLTSC	DRB1*01:29	P40967	PMEL17	287	301	15.17
4762	TAQVVLQAAIPLTSC	DRB1*10:01	P40967	PMEL17	287	301	21.99
4763	TAQVVLQAAIPLTSC	DRB1*11:13	P40967	PMEL17	287	301	45.92
4764	TAQVVLQAAIPLTSC	DRB1*11:14	P40967	PMEL17	287	301	29.07
4765	TAQVVLQAAIPLTSC	DRB1*13:02	P40967	PMEL17	287	301	29.07
4766	TAQVVLQAAIPLTSC	DRB1*13:23	P40967	PMEL17	287	301	29.07
4767	TAQVVLQAAIPLTSC	DRB1*13:97	P40967	PMEL17	287	301	29.07
4768	TAQVVLQAAIPLTSC	DRB1*14:32	P40967	PMEL17	287	301	47.07
4769	TAQVVLQAAIPLTSC	DRB1*15:01	P40967	PMEL17	287	301	46.23
4770	TAQVVLQAAIPLTSC	DRB1*15:06	P40967	PMEL17	287	301	46.23
4771	TASYLIRARRSMDEA	DRB1*08:01	P17643	TRP1	496	510	48.32
4772	TASYLIRARRSMDEA	DRB1*11:01	P17643	TRP1	496	510	39.65
4773	TASYLIRARRSMDEA	DRB1*11:03	P17643	TRP1	496	510	32.61
4774	TASYLIRARRSMDEA	DRB1*11:04	P17643	TRP1	496	510	23.81
4775	TASYLIRARRSMDEA	DRB1*11:10	P17643	TRP1	496	510	39.65
4776	TASYLIRARRSMDEA	DRB1*11:12	P17643	TRP1	496	510	39.65
4777	TASYLIRARRSMDEA	DRB1*11:28	P17643	TRP1	496	510	39.65
4778	TASYLIRARRSMDEA	DRB1*11:29	P17643	TRP1	496	510	39.65
4779	TASYLIRARRSMDEA	DRB1*11:42	P17643	TRP1	496	510	25.57
4780	TASYLIRARRSMDEA	DRB1*11:46	P17643	TRP1	496	510	23.81
4781	TASYLIRARRSMDEA	DRB1*11:49	P17643	TRP1	496	510	39.65
4782	TASYLIRARRSMDEA	DRB1*11:58	P17643	TRP1	496	510	23.81
4783	TASYLIRARRSMDEA	DRB1*11:62	P17643	TRP1	496	510	39.65
4784	TASYLIRARRSMDEA	DRB1*11:74	P17643	TRP1	496	510	39.65
4785	TASYLIRARRSMDEA	DRB1*13:05	P17643	TRP1	496	510	39.65
4786	TASYLIRARRSMDEA	DRB1*13:11	P17643	TRP1	496	510	23.81
4787	TASYLIRARRSMDEA	DRB1*13:14	P17643	TRP1	496	510	39.65
4788	TASYLIRARRSMDEA	DRB1*13:21	P17643	TRP1	496	510	20.41
4789	TASYLIRARRSMDEA	DRB1*13:50	P17643	TRP1	496	510	39.65
4790	TATLRLVKRQVPLDC	DRB1*08:04	P40967	PMEL17	461	475	30.43
4791	TATLRLVKRQVPLDC	DRB1*11:01	P40967	PMEL17	461	475	23.06
4792	TATLRLVKRQVPLDC	DRB1*11:02	P40967	PMEL17	461	475	28.64
4793	TATLRLVKRQVPLDC	DRB1*11:03	P40967	PMEL17	461	475	20.22
4794	TATLRLVKRQVPLDC	DRB1*11:04	P40967	PMEL17	461	475	11.26
4795	TATLRLVKRQVPLDC	DRB1*11:08	P40967	PMEL17	461	475	49.52
4796	TATLRLVKRQVPLDC	DRB1*11:10	P40967	PMEL17	461	475	23.06

4797	TATLRLVKRQVPLDC	DRB1*11:12	P40967	PMEL17	461	475	23.06
4798	TATLRLVKRQVPLDC	DRB1*11:13	P40967	PMEL17	461	475	23.54
4799	TATLRLVKRQVPLDC	DRB1*11:28	P40967	PMEL17	461	475	23.06
4800	TATLRLVKRQVPLDC	DRB1*11:29	P40967	PMEL17	461	475	23.06
4801	TATLRLVKRQVPLDC	DRB1*11:42	P40967	PMEL17	461	475	12.16
4802	TATLRLVKRQVPLDC	DRB1*11:46	P40967	PMEL17	461	475	11.26
4803	TATLRLVKRQVPLDC	DRB1*11:49	P40967	PMEL17	461	475	23.06
4804	TATLRLVKRQVPLDC	DRB1*11:58	P40967	PMEL17	461	475	11.26
4805	TATLRLVKRQVPLDC	DRB1*11:62	P40967	PMEL17	461	475	23.06
4806	TATLRLVKRQVPLDC	DRB1*11:65	P40967	PMEL17	461	475	28.64
4807	TATLRLVKRQVPLDC	DRB1*11:74	P40967	PMEL17	461	475	23.06
4808	TATLRLVKRQVPLDC	DRB1*11:84	P40967	PMEL17	461	475	35.74
4809	TATLRLVKRQVPLDC	DRB1*13:01	P40967	PMEL17	461	475	28.64
4810	TATLRLVKRQVPLDC	DRB1*13:05	P40967	PMEL17	461	475	23.06
4811	TATLRLVKRQVPLDC	DRB1*13:11	P40967	PMEL17	461	475	11.26
4812	TATLRLVKRQVPLDC	DRB1*13:14	P40967	PMEL17	461	475	23.06
4813	TATLRLVKRQVPLDC	DRB1*13:21	P40967	PMEL17	461	475	29.21
4814	TATLRLVKRQVPLDC	DRB1*13:50	P40967	PMEL17	461	475	23.06
4815	TATLRLVKRQVPLDC	DRB1*14:32	P40967	PMEL17	461	475	42.07
4816	TEDGPIRRNPAGNVA	DRB1*11:14	P17643	TRP1	306	320	45.36
4817	TEDGPIRRNPAGNVA	DRB1*13:02	P17643	TRP1	306	320	45.36
4818	TEDGPIRRNPAGNVA	DRB1*13:23	P17643	TRP1	306	320	45.36
4819	TEDGPIRRNPAGNVA	DRB1*13:97	P17643	TRP1	306	320	45.36
4820	TEMFVTAPDNLGYTY	DRB1*04:01	P17643	TRP1	450	464	22.85
4821	TEMFVTAPDNLGYTY	DRB1*04:08	P17643	TRP1	450	464	24.17
4822	TEMFVTAPDNLGYTY	DRB1*04:72	P17643	TRP1	450	464	43.32
4823	TERRLLVRRNIFDLS	DRB1*11:03	P14679	Tyrosinase	113	127	49.6
4824	TEVRADTRPWSGPYI	DRB1*03:11	O75767	TRP2	62	76	28.24
4825	TFGRLQGISPKIMPK	DRB1*01:01	Q16385	SSX2	100	114	23.64
4826	TFGRLQGISPKIMPK	DRB1*01:18	Q16385	SSX2	100	114	21.99
4827	TFGRLQGISPKIMPK	DRB1*01:20	Q16385	SSX2	100	114	25.76
4828	TFGRLQGISPKIMPK	DRB1*10:01	Q16385	SSX2	100	114	47.63
4829	TGRAMLGTHTMEVTV	DRB1*07:01	P40967	PMEL17	174	188	45.41
4830	THLSPNDPIFVLLHT	DRB1*11:14	P17643	TRP1	391	405	43.62
4831	THLSPNDPIFVLLHT	DRB1*13:02	P17643	TRP1	391	405	43.62
4832	THLSPNDPIFVLLHT	DRB1*13:23	P17643	TRP1	391	405	43.62
4833	THLSPNDPIFVLLHT	DRB1*13:97	P17643	TRP1	391	405	43.62
4834	THPLFVIATRRSEEI	DRB1*01:01	P17643	TRP1	155	169	33.65
4835	THPLFVIATRRSEEI	DRB1*01:18	P17643	TRP1	155	169	22.89
4836	THPLFVIATRRSEEI	DRB1*01:20	P17643	TRP1	155	169	19.04

4837	THPLFVIATRRSEEI	DRB1*04:04	P17643	TRP1	155	169	22.73
4838	THPLFVIATRRSEEI	DRB1*08:01	P17643	TRP1	155	169	35.14
4839	THPLFVIATRRSEEI	DRB1*08:02	P17643	TRP1	155	169	45.51
4840	THPLFVIATRRSEEI	DRB1*08:04	P17643	TRP1	155	169	24.66
4841	THPLFVIATRRSEEI	DRB1*08:24	P17643	TRP1	155	169	40.1
4842	THPLFVIATRRSEEI	DRB1*10:01	P17643	TRP1	155	169	34.07
4843	THPLFVIATRRSEEI	DRB1*11:01	P17643	TRP1	155	169	15.19
4844	THPLFVIATRRSEEI	DRB1*11:02	P17643	TRP1	155	169	37.93
4845	THPLFVIATRRSEEI	DRB1*11:03	P17643	TRP1	155	169	25.3
4846	THPLFVIATRRSEEI	DRB1*11:04	P17643	TRP1	155	169	12.59
4847	THPLFVIATRRSEEI	DRB1*11:08	P17643	TRP1	155	169	27.7
4848	THPLFVIATRRSEEI	DRB1*11:10	P17643	TRP1	155	169	15.19
4849	THPLFVIATRRSEEI	DRB1*11:11	P17643	TRP1	155	169	49.02
4850	THPLFVIATRRSEEI	DRB1*11:12	P17643	TRP1	155	169	15.19
4851	THPLFVIATRRSEEI	DRB1*11:13	P17643	TRP1	155	169	37.81
4852	THPLFVIATRRSEEI	DRB1*11:28	P17643	TRP1	155	169	15.19
4853	THPLFVIATRRSEEI	DRB1*11:29	P17643	TRP1	155	169	15.19
4854	THPLFVIATRRSEEI	DRB1*11:37	P17643	TRP1	155	169	33.39
4855	THPLFVIATRRSEEI	DRB1*11:42	P17643	TRP1	155	169	14.39
4856	THPLFVIATRRSEEI	DRB1*11:46	P17643	TRP1	155	169	12.59
4857	THPLFVIATRRSEEI	DRB1*11:49	P17643	TRP1	155	169	15.19
4858	THPLFVIATRRSEEI	DRB1*11:58	P17643	TRP1	155	169	12.59
4859	THPLFVIATRRSEEI	DRB1*11:62	P17643	TRP1	155	169	15.19
4860	THPLFVIATRRSEEI	DRB1*11:65	P17643	TRP1	155	169	37.93
4861	THPLFVIATRRSEEI	DRB1*11:74	P17643	TRP1	155	169	15.19
4862	THPLFVIATRRSEEI	DRB1*11:84	P17643	TRP1	155	169	37.83
4863	THPLFVIATRRSEEI	DRB1*13:01	P17643	TRP1	155	169	37.93
4864	THPLFVIATRRSEEI	DRB1*13:05	P17643	TRP1	155	169	15.19
4865	THPLFVIATRRSEEI	DRB1*13:07	P17643	TRP1	155	169	33.39
4866	THPLFVIATRRSEEI	DRB1*13:11	P17643	TRP1	155	169	12.59
4867	THPLFVIATRRSEEI	DRB1*13:14	P17643	TRP1	155	169	15.19
4868	THPLFVIATRRSEEI	DRB1*13:21	P17643	TRP1	155	169	13.94
4869	THPLFVIATRRSEEI	DRB1*13:50	P17643	TRP1	155	169	15.19
4870	THTMEVTVYHRRGSR	DRB1*11:03	P40967	PMEL17	181	195	38.46
4871	THYYSVKKTFLGVGQ	DRB1*01:18	P17643	TRP1	191	205	44.24
4872	THYYSVKKTFLGVGQ	DRB1*11:01	P17643	TRP1	191	205	37.63
4873	THYYSVKKTFLGVGQ	DRB1*11:10	P17643	TRP1	191	205	37.63
4874	THYYSVKKTFLGVGQ	DRB1*11:12	P17643	TRP1	191	205	37.63
4875	THYYSVKKTFLGVGQ	DRB1*11:28	P17643	TRP1	191	205	37.63
4876	THYYSVKKTFLGVGQ	DRB1*11:29	P17643	TRP1	191	205	37.63

4877	THYYSVKKTFLGVGQ	DRB1*11:49	P17643	TRP1	191	205	37.63
4878	THYYSVKKTFLGVGQ	DRB1*11:62	P17643	TRP1	191	205	37.63
4879	THYYSVKKTFLGVGQ	DRB1*11:74	P17643	TRP1	191	205	37.63
4880	THYYSVKKTFLGVGQ	DRB1*13:05	P17643	TRP1	191	205	37.63
4881	THYYSVKKTFLGVGQ	DRB1*13:14	P17643	TRP1	191	205	37.63
4882	THYYSVKKTFLGVGQ	DRB1*13:21	P17643	TRP1	191	205	42.33
4883	THYYSVKKTFLGVGQ	DRB1*13:50	P17643	TRP1	191	205	37.63
4884	TIRLTAADHRQLQLS	DRB1*03:11	P78358	NY-ESO-1	134	148	44.73
4885	TKAEMLERVIKNYKR	DRB1*11:04	P43358	MAGE4	132	146	48.98
4886	TKAEMLERVIKNYKR	DRB1*11:42	P43358	MAGE4	132	146	36.32
4887	TKAEMLERVIKNYKR	DRB1*11:46	P43358	MAGE4	132	146	48.98
4888	TKAEMLERVIKNYKR	DRB1*11:58	P43358	MAGE4	132	146	48.98
4889	TKAEMLERVIKNYKR	DRB1*13:11	P43358	MAGE4	132	146	48.98
4890	TKKVADLVGFLLLKY	DPB1*15:01	P43355	MAGE1	103	117	36.01
4891	TKKVADLVGFLLLKY	DPB1*33:01	P43355	MAGE1	103	117	25.63
4892	TKKVADLVGFLLLKY	DPB1*71:01	P43355	MAGE1	103	117	25.63
4893	TKKVADLVGFLLLKY	DRB1*01:01	P43355	MAGE1	103	117	22.77
4894	TKKVADLVGFLLLKY	DRB1*01:18	P43355	MAGE1	103	117	15.79
4895	TKKVADLVGFLLLKY	DRB1*01:20	P43355	MAGE1	103	117	18.06
4896	TKKVADLVGFLLLKY	DRB1*01:24	P43355	MAGE1	103	117	42.65
4897	TKKVADLVGFLLLKY	DRB1*01:29	P43355	MAGE1	103	117	48.69
4898	TKLGFKATLPPFMCN	DRB1*01:01	Q16385	SSX2	59	73	13.86
4899	TKLGFKATLPPFMCN	DRB1*01:11	Q16385	SSX2	59	73	37.73
4900	TKLGFKATLPPFMCN	DRB1*01:18	Q16385	SSX2	59	73	14.66
4901	TKLGFKATLPPFMCN	DRB1*01:20	Q16385	SSX2	59	73	45.37
4902	TKLGFKATLPPFMCN	DRB1*01:24	Q16385	SSX2	59	73	31.98
4903	TKLGFKATLPPFMCN	DRB1*01:29	Q16385	SSX2	59	73	28.15
4904	TKLGFKATLPPFMCN	DRB1*07:01	Q16385	SSX2	59	73	13.87
4905	TKLGFKATLPPFMCN	DRB1*09:01	Q16385	SSX2	59	73	21.81
4906	TKLGFKATLPPFMCN	DRB1*10:01	Q16385	SSX2	59	73	12.08
4907	TLIGANASFSIALNF	DRB1*01:20	P40967	PMEL17	76	90	45.92
4908	TLIGANASFSIALNF	DRB1*11:14	P40967	PMEL17	76	90	20.23
4909	TLIGANASFSIALNF	DRB1*13:02	P40967	PMEL17	76	90	20.23
4910	TLIGANASFSIALNF	DRB1*13:23	P40967	PMEL17	76	90	20.23
4911	TLIGANASFSIALNF	DRB1*13:96	P40967	PMEL17	76	90	31.33
4912	TLIGANASFSIALNF	DRB1*13:97	P40967	PMEL17	76	90	20.23
4913	TLISRALVVTHTYLE	DRB1*01:20	P40967	PMEL17	268	282	42.1
4914	TLLGGFFPWLKVYYY	DPB1*33:01	O75767	TRP2	196	210	39.8
4915	TLLGGFFPWLKVYYY	DPB1*71:01	O75767	TRP2	196	210	39.8
4916	TLRLVKRQVPLDCVL	DRB1*11:02	P40967	PMEL17	463	477	48.63

4917	TLRLVKRQVPLDCVL	DRB1*11:03	P40967	PMEL17	463	477	41.82
4918	TLRLVKRQVPLDCVL	DRB1*11:04	P40967	PMEL17	463	477	31.83
4919	TLRLVKRQVPLDCVL	DRB1*11:42	P40967	PMEL17	463	477	26.46
4920	TLRLVKRQVPLDCVL	DRB1*11:46	P40967	PMEL17	463	477	31.83
4921	TLRLVKRQVPLDCVL	DRB1*11:58	P40967	PMEL17	463	477	31.83
4922	TLRLVKRQVPLDCVL	DRB1*11:65	P40967	PMEL17	463	477	48.63
4923	TLRLVKRQVPLDCVL	DRB1*13:01	P40967	PMEL17	463	477	48.63
4924	TLRLVKRQVPLDCVL	DRB1*13:11	P40967	PMEL17	463	477	31.83
4925	TMEVTVYHRRGSRSY	DRB1*11:02	P40967	PMEL17	183	197	42.25
4926	TMEVTVYHRRGSRSY	DRB1*11:03	P40967	PMEL17	183	197	22.12
4927	TMEVTVYHRRGSRSY	DRB1*11:65	P40967	PMEL17	183	197	42.25
4928	TMEVTVYHRRGSRSY	DRB1*13:01	P40967	PMEL17	183	197	42.25
4929	TNTEMFVTAPDNLGY	DRB1*04:01	P17643	TRP1	448	462	33.24
4930	TNTEMFVTAPDNLGY	DRB1*04:08	P17643	TRP1	448	462	35.8
4931	TPAEVSIVVLSGTTA	DRB1*01:01	P40967	PMEL17	401	415	28.59
4932	TPAEVSIVVLSGTTA	DRB1*01:18	P40967	PMEL17	401	415	28.06
4933	TPAEVSIVVLSGTTA	DRB1*01:20	P40967	PMEL17	401	415	24.58
4934	TPPFYSNSTNSFRNT	DRB1*11:14	P17643	TRP1	344	358	24.09
4935	TPPFYSNSTNSFRNT	DRB1*13:02	P17643	TRP1	344	358	24.09
4936	TPPFYSNSTNSFRNT	DRB1*13:23	P17643	TRP1	344	358	24.09
4937	TPPFYSNSTNSFRNT	DRB1*13:96	P17643	TRP1	344	358	41.58
4938	TPPFYSNSTNSFRNT	DRB1*13:97	P17643	TRP1	344	358	24.09
4939	TQDLVQEKYLEYRQV	DPB1*33:01	P43355	MAGE1	240	254	34.55
4940	TQDLVQEKYLEYRQV	DPB1*71:01	P43355	MAGE1	240	254	34.55
4941	TQDWVQENYLEYRQV	DPB1*33:01	P43358	MAGE4	248	262	48.55
4942	TQDWVQENYLEYRQV	DPB1*71:01	P43358	MAGE4	248	262	48.55
4943	TQHWVGLLGPNGTQP	DRB1*01:01	O75767	TRP2	160	174	23.93
4944	TQHWVGLLGPNGTQP	DRB1*01:18	O75767	TRP2	160	174	31.38
4945	TSCILESLFRAVITK	DPB1*02:01	P43355	MAGE1	90	104	14.8
4946	TSCILESLFRAVITK	DPB1*02:02	P43355	MAGE1	90	104	14.89
4947	TSCILESLFRAVITK	DPB1*04:01	P43355	MAGE1	90	104	35.53
4948	TSCILESLFRAVITK	DPB1*126:01	P43355	MAGE1	90	104	35.53
4949	TSCILESLFRAVITK	DPB1*15:01	P43355	MAGE1	90	104	43.93
4950	TSCILESLFRAVITK	DPB1*23:01	P43355	MAGE1	90	104	35.53
4951	TSCILESLFRAVITK	DPB1*33:01	P43355	MAGE1	90	104	9.3
4952	TSCILESLFRAVITK	DPB1*39:01	P43355	MAGE1	90	104	35.53
4953	TSCILESLFRAVITK	DPB1*46:01	P43355	MAGE1	90	104	14.8
4954	TSCILESLFRAVITK	DPB1*47:01	P43355	MAGE1	90	104	14.89
4955	TSCILESLFRAVITK	DPB1*71:01	P43355	MAGE1	90	104	9.3
4956	TSCILESLFRAVITK	DPB1*72:01	P43355	MAGE1	90	104	46.01

4957	TSCILESLFRAVITK	DPB1*81:01	P43355	MAGE1	90	104	14.8
4958	TSYVKVLEHVVRVNA	DRB1*01:01	P43358	MAGE4	282	296	39.69
4959	TSYVKVLEHVVRVNA	DRB1*01:18	P43358	MAGE4	282	296	25.53
4960	TSYVKVLEHVVRVNA	DRB1*11:04	P43358	MAGE4	282	296	45.07
4961	TSYVKVLEHVVRVNA	DRB1*11:13	P43358	MAGE4	282	296	33.19
4962	TSYVKVLEHVVRVNA	DRB1*11:42	P43358	MAGE4	282	296	32.24
4963	TSYVKVLEHVVRVNA	DRB1*11:46	P43358	MAGE4	282	296	45.07
4964	TSYVKVLEHVVRVNA	DRB1*11:58	P43358	MAGE4	282	296	45.07
4965	TSYVKVLEHVVRVNA	DRB1*13:11	P43358	MAGE4	282	296	45.07
4966	TSYVKVLEYVIKVSA	DRB1*01:18	P43355	MAGE1	274	288	29.34
4967	TTEWVETTARELPIP	DRB1*07:01	P40967	PMEL17	420	434	49.19
4968	TTHPLFVIATRRSEE	DRB1*01:01	P17643	TRP1	154	168	42.82
4969	TTHPLFVIATRRSEE	DRB1*01:18	P17643	TRP1	154	168	28.19
4970	TTHPLFVIATRRSEE	DRB1*01:20	P17643	TRP1	154	168	23.55
4971	TTHPLFVIATRRSEE	DRB1*04:04	P17643	TRP1	154	168	27.3
4972	TTHPLFVIATRRSEE	DRB1*08:01	P17643	TRP1	154	168	43.73
4973	TTHPLFVIATRRSEE	DRB1*08:04	P17643	TRP1	154	168	29.7
4974	TTHPLFVIATRRSEE	DRB1*08:24	P17643	TRP1	154	168	48.89
4975	TTHPLFVIATRRSEE	DRB1*10:01	P17643	TRP1	154	168	39.82
4976	TTHPLFVIATRRSEE	DRB1*11:01	P17643	TRP1	154	168	19.39
4977	TTHPLFVIATRRSEE	DRB1*11:03	P17643	TRP1	154	168	30.51
4978	TTHPLFVIATRRSEE	DRB1*11:04	P17643	TRP1	154	168	15.45
4979	TTHPLFVIATRRSEE	DRB1*11:08	P17643	TRP1	154	168	35.68
4980	TTHPLFVIATRRSEE	DRB1*11:10	P17643	TRP1	154	168	19.39
4981	TTHPLFVIATRRSEE	DRB1*11:12	P17643	TRP1	154	168	19.39
4982	TTHPLFVIATRRSEE	DRB1*11:13	P17643	TRP1	154	168	49.64
4983	TTHPLFVIATRRSEE	DRB1*11:28	P17643	TRP1	154	168	19.39
4984	TTHPLFVIATRRSEE	DRB1*11:29	P17643	TRP1	154	168	19.39
4985	TTHPLFVIATRRSEE	DRB1*11:37	P17643	TRP1	154	168	45.06
4986	TTHPLFVIATRRSEE	DRB1*11:42	P17643	TRP1	154	168	17.13
4987	TTHPLFVIATRRSEE	DRB1*11:46	P17643	TRP1	154	168	15.45
4988	TTHPLFVIATRRSEE	DRB1*11:49	P17643	TRP1	154	168	19.39
4989	TTHPLFVIATRRSEE	DRB1*11:58	P17643	TRP1	154	168	15.45
4990	TTHPLFVIATRRSEE	DRB1*11:62	P17643	TRP1	154	168	19.39
4991	TTHPLFVIATRRSEE	DRB1*11:74	P17643	TRP1	154	168	19.39
4992	TTHPLFVIATRRSEE	DRB1*11:84	P17643	TRP1	154	168	49.87
4993	TTHPLFVIATRRSEE	DRB1*13:05	P17643	TRP1	154	168	19.39
4994	TTHPLFVIATRRSEE	DRB1*13:07	P17643	TRP1	154	168	45.06
4995	TTHPLFVIATRRSEE	DRB1*13:11	P17643	TRP1	154	168	15.45
4996	TTHPLFVIATRRSEE	DRB1*13:14	P17643	TRP1	154	168	19.39

4997	TTHPLFVIATRRSEE	DRB1*13:21	P17643	TRP1	154	168	16.4
4998	TTHPLFVIATRRSEE	DRB1*13:50	P17643	TRP1	154	168	19.39
4999	TTQHWVGLLGPNGTQ	DRB1*01:01	O75767	TRP2	159	173	21.6
5000	TTQHWVGLLGPNGTQ	DRB1*01:18	O75767	TRP2	159	173	27.9
5001	TTQHWVGLLGPNGTQ	DRB1*10:01	O75767	TRP2	159	173	45.93
5002	TTTEWVETTARELPI	DRB1*07:01	P40967	PMEL17	419	433	40.29
5003	TWGQYWQVLGGPVSG	DRB1*01:01	P40967	PMEL17	155	169	10.3
5004	TWGQYWQVLGGPVSG	DRB1*01:11	P40967	PMEL17	155	169	42.16
5005	TWGQYWQVLGGPVSG	DRB1*01:18	P40967	PMEL17	155	169	11.07
5006	TWGQYWQVLGGPVSG	DRB1*01:20	P40967	PMEL17	155	169	38.7
5007	TWGQYWQVLGGPVSG	DRB1*01:24	P40967	PMEL17	155	169	30.15
5008	TWGQYWQVLGGPVSG	DRB1*01:29	P40967	PMEL17	155	169	25.74
5009	TWGQYWQVLGGPVSG	DRB1*10:01	P40967	PMEL17	155	169	28.49
5010	TWHRYHLLRLEKDMQ	DRB1*10:01	P17643	TRP1	222	236	34.6
5011	TWHRYHLLRLEKDMQ	DRB1*13:21	P17643	TRP1	222	236	49.73
5012	VADLVGFLLLKYRAR	DPB1*02:01	P43355	MAGE1	106	120	48.01
5013	VADLVGFLLLKYRAR	DPB1*02:02	P43355	MAGE1	106	120	45.69
5014	VADLVGFLLLKYRAR	DPB1*15:01	P43355	MAGE1	106	120	29.33
5015	VADLVGFLLLKYRAR	DPB1*33:01	P43355	MAGE1	106	120	20.34
5016	VADLVGFLLLKYRAR	DPB1*40:01	P43355	MAGE1	106	120	49.91
5017	VADLVGFLLLKYRAR	DPB1*46:01	P43355	MAGE1	106	120	48.01
5018	VADLVGFLLLKYRAR	DPB1*47:01	P43355	MAGE1	106	120	45.69
5019	VADLVGFLLLKYRAR	DPB1*71:01	P43355	MAGE1	106	120	20.34
5020	VADLVGFLLLKYRAR	DPB1*81:01	P43355	MAGE1	106	120	48.01
5021	VADLVGFLLLKYRAR	DRB1*01:20	P43355	MAGE1	106	120	49.37
5022	VADLVGFLLLKYRAR	DRB1*11:04	P43355	MAGE1	106	120	36.58
5023	VADLVGFLLLKYRAR	DRB1*11:13	P43355	MAGE1	106	120	45.62
5024	VADLVGFLLLKYRAR	DRB1*11:42	P43355	MAGE1	106	120	33.92
5025	VADLVGFLLLKYRAR	DRB1*11:46	P43355	MAGE1	106	120	36.58
5026	VADLVGFLLLKYRAR	DRB1*11:58	P43355	MAGE1	106	120	36.58
5027	VADLVGFLLLKYRAR	DRB1*12:03	P43355	MAGE1	106	120	40.91
5028	VADLVGFLLLKYRAR	DRB1*12:16	P43355	MAGE1	106	120	48.62
5029	VADLVGFLLLKYRAR	DRB1*13:11	P43355	MAGE1	106	120	36.58
5030	VADLVGFLLLKYRAR	DRB1*13:21	P43355	MAGE1	106	120	48.24
5031	VALIFGTASYLIRAR	DPB1*33:01	P17643	TRP1	490	504	37.43
5032	VALIFGTASYLIRAR	DPB1*71:01	P17643	TRP1	490	504	37.43
5033	VALIFGTASYLIRAR	DRB1*01:01	P17643	TRP1	490	504	12.15
5034	VALIFGTASYLIRAR	DRB1*01:02	P17643	TRP1	490	504	34.03
5035	VALIFGTASYLIRAR	DRB1*01:11	P17643	TRP1	490	504	25.64
5036	VALIFGTASYLIRAR	DRB1*01:18	P17643	TRP1	490	504	10.35

5037	VALIFGTASYLIRAR	DRB1*01:20	P17643	TRP1	490	504	11.1
5038	VALIFGTASYLIRAR	DRB1*01:24	P17643	TRP1	490	504	19.4
5039	VALIFGTASYLIRAR	DRB1*01:29	P17643	TRP1	490	504	19.31
5040	VALIFGTASYLIRAR	DRB1*07:01	P17643	TRP1	490	504	10.07
5041	VALIFGTASYLIRAR	DRB1*09:01	P17643	TRP1	490	504	38.61
5042	VALIFGTASYLIRAR	DRB1*10:01	P17643	TRP1	490	504	48.32
5043	VALIFGTASYLIRAR	DRB1*11:14	P17643	TRP1	490	504	45.96
5044	VALIFGTASYLIRAR	DRB1*13:02	P17643	TRP1	490	504	45.96
5045	VALIFGTASYLIRAR	DRB1*13:23	P17643	TRP1	490	504	45.96
5046	VALIFGTASYLIRAR	DRB1*13:97	P17643	TRP1	490	504	45.96
5047	VALIFGTASYLIRAR	DRB1*14:32	P17643	TRP1	490	504	46.2
5048	VALIFGTASYLIRAR	DRB1*15:01	P17643	TRP1	490	504	22.98
5049	VALIFGTASYLIRAR	DRB1*15:02	P17643	TRP1	490	504	43.9
5050	VALIFGTASYLIRAR	DRB1*15:06	P17643	TRP1	490	504	22.98
5051	VALIFGTASYLIRAR	DRB1*15:07	P17643	TRP1	490	504	37.71
5052	VALIFGTASYLIRAR	DRB1*15:37	P17643	TRP1	490	504	45.68
5053	VALIFGTASYLIRAR	DRB1*16:02	P17643	TRP1	490	504	47.68
5054	VDELAHFLLRKYRAK	DRB1*11:01	P43358	MAGE4	114	128	43.08
5055	VDELAHFLLRKYRAK	DRB1*11:03	P43358	MAGE4	114	128	25.44
5056	VDELAHFLLRKYRAK	DRB1*11:04	P43358	MAGE4	114	128	28.08
5057	VDELAHFLLRKYRAK	DRB1*11:10	P43358	MAGE4	114	128	43.08
5058	VDELAHFLLRKYRAK	DRB1*11:12	P43358	MAGE4	114	128	43.08
5059	VDELAHFLLRKYRAK	DRB1*11:28	P43358	MAGE4	114	128	43.08
5060	VDELAHFLLRKYRAK	DRB1*11:29	P43358	MAGE4	114	128	43.08
5061	VDELAHFLLRKYRAK	DRB1*11:42	P43358	MAGE4	114	128	29.7
5062	VDELAHFLLRKYRAK	DRB1*11:46	P43358	MAGE4	114	128	28.08
5063	VDELAHFLLRKYRAK	DRB1*11:49	P43358	MAGE4	114	128	43.08
5064	VDELAHFLLRKYRAK	DRB1*11:58	P43358	MAGE4	114	128	28.08
5065	VDELAHFLLRKYRAK	DRB1*11:62	P43358	MAGE4	114	128	43.08
5066	VDELAHFLLRKYRAK	DRB1*11:74	P43358	MAGE4	114	128	43.08
5067	VDELAHFLLRKYRAK	DRB1*12:02	P43358	MAGE4	114	128	44.46
5068	VDELAHFLLRKYRAK	DRB1*13:05	P43358	MAGE4	114	128	43.08
5069	VDELAHFLLRKYRAK	DRB1*13:11	P43358	MAGE4	114	128	28.08
5070	VDELAHFLLRKYRAK	DRB1*13:14	P43358	MAGE4	114	128	43.08
5071	VDELAHFLLRKYRAK	DRB1*13:21	P43358	MAGE4	114	128	37.58
5072	VDELAHFLLRKYRAK	DRB1*13:50	P43358	MAGE4	114	128	43.08
5073	VDFSHEGPAFLTWHR	DRB1*01:01	P17643	TRP1	211	225	39.26
5074	VFLALSAQLLQARLM	DRB1*01:01	Q13072	BAGE	6	20	3.41
5075	VFLALSAQLLQARLM	DRB1*01:02	Q13072	BAGE	6	20	20.81
5076	VFLALSAQLLQARLM	DRB1*01:03	Q13072	BAGE	6	20	38.58

5077	VFLALSAQLLQARLM	DRB1*01:11	Q13072	BAGE	6	20	5.78
5078	VFLALSAQLLQARLM	DRB1*01:18	Q13072	BAGE	6	20	3.05
5079	VFLALSAQLLQARLM	DRB1*01:20	Q13072	BAGE	6	20	7.51
5080	VFLALSAQLLQARLM	DRB1*01:24	Q13072	BAGE	6	20	3.92
5081	VFLALSAQLLQARLM	DRB1*01:29	Q13072	BAGE	6	20	4.82
5082	VFLALSAQLLQARLM	DRB1*07:01	Q13072	BAGE	6	20	42.78
5083	VFLALSAQLLQARLM	DRB1*10:01	Q13072	BAGE	6	20	11.26
5084	VFLALSAQLLQARLM	DRB1*15:02	Q13072	BAGE	6	20	39.35
5085	VFLALSAQLLQARLM	DRB1*15:15	Q13072	BAGE	6	20	35.27
5086	VFLALSAQLLQARLM	DRB1*16:01	Q13072	BAGE	6	20	27.58
5087	VFLALSAQLLQARLM	DRB1*16:02	Q13072	BAGE	6	20	19.28
5088	VFLALSAQLLQARLM	DRB1*16:04	Q13072	BAGE	6	20	42.87
5089	VFLALSAQLLQARLM	DRB1*16:05	Q13072	BAGE	6	20	24.39
5090	VFLALSAQLLQARLM	DRB1*16:09	Q13072	BAGE	6	20	20.13
5091	VGAVLTALLAGLVSL	DRB1*01:01	P14679	Tyrosinase	484	498	10.38
5092	VGAVLTALLAGLVSL	DRB1*01:02	P14679	Tyrosinase	484	498	35.82
5093	VGAVLTALLAGLVSL	DRB1*01:11	P14679	Tyrosinase	484	498	28.25
5094	VGAVLTALLAGLVSL	DRB1*01:18	P14679	Tyrosinase	484	498	9.96
5095	VGAVLTALLAGLVSL	DRB1*01:20	P14679	Tyrosinase	484	498	11.67
5096	VGAVLTALLAGLVSL	DRB1*01:24	P14679	Tyrosinase	484	498	14.02
5097	VGAVLTALLAGLVSL	DRB1*01:29	P14679	Tyrosinase	484	498	18.36
5098	VGAVLTALLAGLVSL	DRB1*10:01	P14679	Tyrosinase	484	498	47.38
5099	VGFLLLKYRAREPVT	DRB1*01:18	P43355	MAGE1	110	124	49.14
5100	VGFLLLKYRAREPVT	DRB1*01:20	P43355	MAGE1	110	124	43.35
5101	VGFLLLKYRAREPVT	DRB1*11:01	P43355	MAGE1	110	124	31.71
5102	VGFLLLKYRAREPVT	DRB1*11:02	P43355	MAGE1	110	124	27.92
5103	VGFLLLKYRAREPVT	DRB1*11:03	P43355	MAGE1	110	124	17.79
5104	VGFLLLKYRAREPVT	DRB1*11:04	P43355	MAGE1	110	124	18.8
5105	VGFLLLKYRAREPVT	DRB1*11:10	P43355	MAGE1	110	124	31.71
5106	VGFLLLKYRAREPVT	DRB1*11:12	P43355	MAGE1	110	124	31.71
5107	VGFLLLKYRAREPVT	DRB1*11:13	P43355	MAGE1	110	124	42.09
5108	VGFLLLKYRAREPVT	DRB1*11:28	P43355	MAGE1	110	124	31.71
5109	VGFLLLKYRAREPVT	DRB1*11:29	P43355	MAGE1	110	124	31.71
5110	VGFLLLKYRAREPVT	DRB1*11:42	P43355	MAGE1	110	124	21.61
5111	VGFLLLKYRAREPVT	DRB1*11:46	P43355	MAGE1	110	124	18.8
5112	VGFLLLKYRAREPVT	DRB1*11:49	P43355	MAGE1	110	124	31.71
5113	VGFLLLKYRAREPVT	DRB1*11:58	P43355	MAGE1	110	124	18.8
5114	VGFLLLKYRAREPVT	DRB1*11:62	P43355	MAGE1	110	124	31.71
5115	VGFLLLKYRAREPVT	DRB1*11:65	P43355	MAGE1	110	124	27.92
5116	VGFLLLKYRAREPVT	DRB1*11:74	P43355	MAGE1	110	124	31.71

5117	VGFLLLKYRAREPVT	DRB1*11:84	P43355	MAGE1	110	124	44.21
5118	VGFLLLKYRAREPVT	DRB1*13:01	P43355	MAGE1	110	124	27.92
5119	VGFLLLKYRAREPVT	DRB1*13:05	P43355	MAGE1	110	124	31.71
5120	VGFLLLKYRAREPVT	DRB1*13:11	P43355	MAGE1	110	124	18.8
5121	VGFLLLKYRAREPVT	DRB1*13:14	P43355	MAGE1	110	124	31.71
5122	VGFLLLKYRAREPVT	DRB1*13:21	P43355	MAGE1	110	124	20.9
5123	VGFLLLKYRAREPVT	DRB1*13:50	P43355	MAGE1	110	124	31.71
5124	VGFLLLKYRAREPVT	DRB1*15:01	P43355	MAGE1	110	124	46.64
5125	VGFLLLKYRAREPVT	DRB1*15:06	P43355	MAGE1	110	124	46.64
5126	VIGALLAVGATKVPR	DRB1*01:01	P40967	PMEL17	14	28	6.6
5127	VIGALLAVGATKVPR	DRB1*01:02	P40967	PMEL17	14	28	16.21
5128	VIGALLAVGATKVPR	DRB1*01:11	P40967	PMEL17	14	28	17.12
5129	VIGALLAVGATKVPR	DRB1*01:18	P40967	PMEL17	14	28	6.76
5130	VIGALLAVGATKVPR	DRB1*01:20	P40967	PMEL17	14	28	6.21
5131	VIGALLAVGATKVPR	DRB1*01:24	P40967	PMEL17	14	28	12.55
5132	VIGALLAVGATKVPR	DRB1*01:29	P40967	PMEL17	14	28	12.09
5133	VIGALLAVGATKVPR	DRB1*07:01	P40967	PMEL17	14	28	16.71
5134	VIGALLAVGATKVPR	DRB1*10:01	P40967	PMEL17	14	28	30.6
5135	VIGALLAVGATKVPR	DRB1*11:14	P40967	PMEL17	14	28	32.72
5136	VIGALLAVGATKVPR	DRB1*13:02	P40967	PMEL17	14	28	32.72
5137	VIGALLAVGATKVPR	DRB1*13:23	P40967	PMEL17	14	28	32.72
5138	VIGALLAVGATKVPR	DRB1*13:97	P40967	PMEL17	14	28	32.72
5139	VIGLRVWQWEVISCK	DPB1*33:01	O75767	TRP2	213	227	28.38
5140	VIGLRVWQWEVISCK	DPB1*71:01	O75767	TRP2	213	227	28.38
5141	VIKNYKRCFPVIFGK	DRB1*01:01	P43358	MAGE4	140	154	49.6
5142	VIKNYKRCFPVIFGK	DRB1*01:18	P43358	MAGE4	140	154	40.29
5143	VIKVSARVRFFFPSL	DRB1*03:11	P43355	MAGE1	283	297	40.94
5144	VIKVSARVRFFFPSL	DRB1*11:02	P43355	MAGE1	283	297	21.98
5145	VIKVSARVRFFFPSL	DRB1*11:03	P43355	MAGE1	283	297	49.68
5146	VIKVSARVRFFFPSL	DRB1*11:04	P43355	MAGE1	283	297	46.43
5147	VIKVSARVRFFFPSL	DRB1*11:13	P43355	MAGE1	283	297	20.51
5148	VIKVSARVRFFFPSL	DRB1*11:42	P43355	MAGE1	283	297	27.3
5149	VIKVSARVRFFFPSL	DRB1*11:46	P43355	MAGE1	283	297	46.43
5150	VIKVSARVRFFFPSL	DRB1*11:58	P43355	MAGE1	283	297	46.43
5151	VIKVSARVRFFFPSL	DRB1*11:65	P43355	MAGE1	283	297	21.98
5152	VIKVSARVRFFFPSL	DRB1*13:01	P43355	MAGE1	283	297	21.98
5153	VIKVSARVRFFFPSL	DRB1*13:11	P43355	MAGE1	283	297	46.43
5154	VIKVSARVRFFFPSL	DRB1*13:61	P43355	MAGE1	283	297	47.46
5155	VIKVSARVRFFFPSL	DRB1*14:01	P43355	MAGE1	283	297	36.15
5156	VIKVSARVRFFFPSL	DRB1*14:32	P43355	MAGE1	283	297	26.51

5157	VIKVSARVRFFFPSL	DRB1*14:54	P43355	MAGE1	283	297	36.15
5158	VISCKLIKRATTRQP	DRB1*08:04	O75767	TRP2	223	237	38.29
5159	VISCKLIKRATTRQP	DRB1*11:01	O75767	TRP2	223	237	33.86
5160	VISCKLIKRATTRQP	DRB1*11:02	O75767	TRP2	223	237	48.12
5161	VISCKLIKRATTRQP	DRB1*11:03	O75767	TRP2	223	237	29.49
5162	VISCKLIKRATTRQP	DRB1*11:04	O75767	TRP2	223	237	20.64
5163	VISCKLIKRATTRQP	DRB1*11:10	O75767	TRP2	223	237	33.86
5164	VISCKLIKRATTRQP	DRB1*11:12	O75767	TRP2	223	237	33.86
5165	VISCKLIKRATTRQP	DRB1*11:28	O75767	TRP2	223	237	33.86
5166	VISCKLIKRATTRQP	DRB1*11:29	O75767	TRP2	223	237	33.86
5167	VISCKLIKRATTRQP	DRB1*11:42	O75767	TRP2	223	237	25.2
5168	VISCKLIKRATTRQP	DRB1*11:46	O75767	TRP2	223	237	20.64
5169	VISCKLIKRATTRQP	DRB1*11:49	O75767	TRP2	223	237	33.86
5170	VISCKLIKRATTRQP	DRB1*11:58	O75767	TRP2	223	237	20.64
5171	VISCKLIKRATTRQP	DRB1*11:62	O75767	TRP2	223	237	33.86
5172	VISCKLIKRATTRQP	DRB1*11:65	O75767	TRP2	223	237	48.12
5173	VISCKLIKRATTRQP	DRB1*11:74	O75767	TRP2	223	237	33.86
5174	VISCKLIKRATTRQP	DRB1*13:01	O75767	TRP2	223	237	48.12
5175	VISCKLIKRATTRQP	DRB1*13:05	O75767	TRP2	223	237	33.86
5176	VISCKLIKRATTRQP	DRB1*13:11	O75767	TRP2	223	237	20.64
5177	VISCKLIKRATTRQP	DRB1*13:14	O75767	TRP2	223	237	33.86
5178	VISCKLIKRATTRQP	DRB1*13:50	O75767	TRP2	223	237	33.86
5179	VITKKVADLVGFLLL	DRB1*01:18	P43355	MAGE1	101	115	33.71
5180	VITKKVADLVGFLLL	DRB1*01:20	P43355	MAGE1	101	115	36.19
5181	VIWVNNTIINGSQVW	DRB1*11:14	P40967	PMEL17	102	116	31.07
5182	VIWVNNTIINGSQVW	DRB1*13:02	P40967	PMEL17	102	116	31.07
5183	VIWVNNTIINGSQVW	DRB1*13:23	P40967	PMEL17	102	116	31.07
5184	VIWVNNTIINGSQVW	DRB1*13:96	P40967	PMEL17	102	116	41.76
5185	VIWVNNTIINGSQVW	DRB1*13:97	P40967	PMEL17	102	116	31.07
5186	VKVLEHVVRVNARVR	DRB1*08:04	P43358	MAGE4	285	299	34.31
5187	VKVLEHVVRVNARVR	DRB1*11:01	P43358	MAGE4	285	299	30.17
5188	VKVLEHVVRVNARVR	DRB1*11:02	P43358	MAGE4	285	299	27.4
5189	VKVLEHVVRVNARVR	DRB1*11:03	P43358	MAGE4	285	299	25.51
5190	VKVLEHVVRVNARVR	DRB1*11:04	P43358	MAGE4	285	299	13.92
5191	VKVLEHVVRVNARVR	DRB1*11:10	P43358	MAGE4	285	299	30.17
5192	VKVLEHVVRVNARVR	DRB1*11:12	P43358	MAGE4	285	299	30.17
5193	VKVLEHVVRVNARVR	DRB1*11:13	P43358	MAGE4	285	299	24.4
5194	VKVLEHVVRVNARVR	DRB1*11:28	P43358	MAGE4	285	299	30.17
5195	VKVLEHVVRVNARVR	DRB1*11:29	P43358	MAGE4	285	299	30.17
5196	VKVLEHVVRVNARVR	DRB1*11:42	P43358	MAGE4	285	299	16.03

5197	VKVLEHVVRVNARVR	DRB1*11:46	P43358	MAGE4	285	299	13.92
5198	VKVLEHVVRVNARVR	DRB1*11:49	P43358	MAGE4	285	299	30.17
5199	VKVLEHVVRVNARVR	DRB1*11:58	P43358	MAGE4	285	299	13.92
5200	VKVLEHVVRVNARVR	DRB1*11:62	P43358	MAGE4	285	299	30.17
5201	VKVLEHVVRVNARVR	DRB1*11:65	P43358	MAGE4	285	299	27.4
5202	VKVLEHVVRVNARVR	DRB1*11:74	P43358	MAGE4	285	299	30.17
5203	VKVLEHVVRVNARVR	DRB1*11:84	P43358	MAGE4	285	299	34.22
5204	VKVLEHVVRVNARVR	DRB1*13:01	P43358	MAGE4	285	299	27.4
5205	VKVLEHVVRVNARVR	DRB1*13:05	P43358	MAGE4	285	299	30.17
5206	VKVLEHVVRVNARVR	DRB1*13:11	P43358	MAGE4	285	299	13.92
5207	VKVLEHVVRVNARVR	DRB1*13:14	P43358	MAGE4	285	299	30.17
5208	VKVLEHVVRVNARVR	DRB1*13:50	P43358	MAGE4	285	299	30.17
5209	VKVLEHVVRVNARVR	DRB1*14:06	P43358	MAGE4	285	299	42.9
5210	VKVLEHVVRVNARVR	DRB1*14:32	P43358	MAGE4	285	299	46.12
5211	VKVLEYVIKVSARVR	DRB1*01:01	P43355	MAGE1	277	291	13.87
5212	VKVLEYVIKVSARVR	DRB1*01:11	P43355	MAGE1	277	291	26.54
5213	VKVLEYVIKVSARVR	DRB1*01:18	P43355	MAGE1	277	291	9.9
5214	VKVLEYVIKVSARVR	DRB1*01:20	P43355	MAGE1	277	291	29.48
5215	VKVLEYVIKVSARVR	DRB1*01:24	P43355	MAGE1	277	291	28.97
5216	VKVLEYVIKVSARVR	DRB1*01:29	P43355	MAGE1	277	291	19.83
5217	VKVLEYVIKVSARVR	DRB1*07:01	P43355	MAGE1	277	291	44.91
5218	VKVLEYVIKVSARVR	DRB1*08:04	P43355	MAGE1	277	291	37.55
5219	VKVLEYVIKVSARVR	DRB1*10:01	P43355	MAGE1	277	291	46.73
5220	VKVLEYVIKVSARVR	DRB1*11:01	P43355	MAGE1	277	291	29.38
5221	VKVLEYVIKVSARVR	DRB1*11:02	P43355	MAGE1	277	291	24.8
5222	VKVLEYVIKVSARVR	DRB1*11:03	P43355	MAGE1	277	291	36.03
5223	VKVLEYVIKVSARVR	DRB1*11:04	P43355	MAGE1	277	291	21.36
5224	VKVLEYVIKVSARVR	DRB1*11:08	P43355	MAGE1	277	291	30.97
5225	VKVLEYVIKVSARVR	DRB1*11:10	P43355	MAGE1	277	291	29.38
5226	VKVLEYVIKVSARVR	DRB1*11:12	P43355	MAGE1	277	291	29.38
5227	VKVLEYVIKVSARVR	DRB1*11:13	P43355	MAGE1	277	291	20.29
5228	VKVLEYVIKVSARVR	DRB1*11:14	P43355	MAGE1	277	291	46.25
5229	VKVLEYVIKVSARVR	DRB1*11:28	P43355	MAGE1	277	291	29.38
5230	VKVLEYVIKVSARVR	DRB1*11:29	P43355	MAGE1	277	291	29.38
5231	VKVLEYVIKVSARVR	DRB1*11:42	P43355	MAGE1	277	291	15.96
5232	VKVLEYVIKVSARVR	DRB1*11:46	P43355	MAGE1	277	291	21.36
5233	VKVLEYVIKVSARVR	DRB1*11:49	P43355	MAGE1	277	291	29.38
5234	VKVLEYVIKVSARVR	DRB1*11:58	P43355	MAGE1	277	291	21.36
5235	VKVLEYVIKVSARVR	DRB1*11:62	P43355	MAGE1	277	291	29.38
5236	VKVLEYVIKVSARVR	DRB1*11:65	P43355	MAGE1	277	291	24.8

5237	VKVLEYVIKVSARVR	DRB1*11:74	P43355	MAGE1	277	291	29.38
5238	VKVLEYVIKVSARVR	DRB1*11:84	P43355	MAGE1	277	291	39.08
5239	VKVLEYVIKVSARVR	DRB1*13:01	P43355	MAGE1	277	291	24.8
5240	VKVLEYVIKVSARVR	DRB1*13:02	P43355	MAGE1	277	291	46.25
5241	VKVLEYVIKVSARVR	DRB1*13:05	P43355	MAGE1	277	291	29.38
5242	VKVLEYVIKVSARVR	DRB1*13:11	P43355	MAGE1	277	291	21.36
5243	VKVLEYVIKVSARVR	DRB1*13:14	P43355	MAGE1	277	291	29.38
5244	VKVLEYVIKVSARVR	DRB1*13:23	P43355	MAGE1	277	291	46.25
5245	VKVLEYVIKVSARVR	DRB1*13:50	P43355	MAGE1	277	291	29.38
5246	VKVLEYVIKVSARVR	DRB1*13:97	P43355	MAGE1	277	291	46.25
5247	VKVLEYVIKVSARVR	DRB1*14:06	P43355	MAGE1	277	291	40.43
5248	VKVLEYVIKVSARVR	DRB1*14:32	P43355	MAGE1	277	291	36.1
5249	VKVLEYVIKVSARVR	DRB1*16:02	P43355	MAGE1	277	291	43.03
5250	VKVLEYVIKVSARVR	DRB1*16:09	P43355	MAGE1	277	291	42.99
5251	VLASLIYRRRLMKQD	DRB1*11:02	P40967	PMEL17	610	624	18.87
5252	VLASLIYRRRLMKQD	DRB1*11:03	P40967	PMEL17	610	624	13.43
5253	VLASLIYRRRLMKQD	DRB1*11:04	P40967	PMEL17	610	624	22.87
5254	VLASLIYRRRLMKQD	DRB1*11:13	P40967	PMEL17	610	624	40.36
5255	VLASLIYRRRLMKQD	DRB1*11:42	P40967	PMEL17	610	624	22.78
5256	VLASLIYRRRLMKQD	DRB1*11:46	P40967	PMEL17	610	624	22.87
5257	VLASLIYRRRLMKQD	DRB1*11:58	P40967	PMEL17	610	624	22.87
5258	VLASLIYRRRLMKQD	DRB1*11:65	P40967	PMEL17	610	624	18.87
5259	VLASLIYRRRLMKQD	DRB1*11:84	P40967	PMEL17	610	624	45.75
5260	VLASLIYRRRLMKQD	DRB1*13:01	P40967	PMEL17	610	624	18.87
5261	VLASLIYRRRLMKQD	DRB1*13:11	P40967	PMEL17	610	624	22.87
5262	VLASLIYRRRLMKQD	DRB1*13:21	P40967	PMEL17	610	624	46.93
5263	VLEHVVRVNARVRIA	DRB1*01:18	P43358	MAGE4	287	301	47.38
5264	VLEHVVRVNARVRIA	DRB1*01:20	P43358	MAGE4	287	301	36.74
5265	VLEHVVRVNARVRIA	DRB1*03:11	P43358	MAGE4	287	301	29.41
5266	VLEHVVRVNARVRIA	DRB1*08:04	P43358	MAGE4	287	301	43.42
5267	VLEHVVRVNARVRIA	DRB1*11:02	P43358	MAGE4	287	301	12.31
5268	VLEHVVRVNARVRIA	DRB1*11:03	P43358	MAGE4	287	301	17.08
5269	VLEHVVRVNARVRIA	DRB1*11:04	P43358	MAGE4	287	301	20.88
5270	VLEHVVRVNARVRIA	DRB1*11:08	P43358	MAGE4	287	301	49.56
5271	VLEHVVRVNARVRIA	DRB1*11:13	P43358	MAGE4	287	301	16.61
5272	VLEHVVRVNARVRIA	DRB1*11:14	P43358	MAGE4	287	301	20.72
5273	VLEHVVRVNARVRIA	DRB1*11:42	P43358	MAGE4	287	301	14.07
5274	VLEHVVRVNARVRIA	DRB1*11:46	P43358	MAGE4	287	301	20.88
5275	VLEHVVRVNARVRIA	DRB1*11:58	P43358	MAGE4	287	301	20.88
5276	VLEHVVRVNARVRIA	DRB1*11:65	P43358	MAGE4	287	301	12.31

5277	VLEHVVRVNARVRIA	DRB1*11:84	P43358	MAGE4	287	301	47.19
5278	VLEHVVRVNARVRIA	DRB1*13:01	P43358	MAGE4	287	301	12.31
5279	VLEHVVRVNARVRIA	DRB1*13:02	P43358	MAGE4	287	301	20.72
5280	VLEHVVRVNARVRIA	DRB1*13:11	P43358	MAGE4	287	301	20.88
5281	VLEHVVRVNARVRIA	DRB1*13:23	P43358	MAGE4	287	301	20.72
5282	VLEHVVRVNARVRIA	DRB1*13:61	P43358	MAGE4	287	301	20.85
5283	VLEHVVRVNARVRIA	DRB1*13:96	P43358	MAGE4	287	301	28.34
5284	VLEHVVRVNARVRIA	DRB1*13:97	P43358	MAGE4	287	301	20.72
5285	VLEHVVRVNARVRIA	DRB1*14:06	P43358	MAGE4	287	301	25.83
5286	VLEHVVRVNARVRIA	DRB1*14:12	P43358	MAGE4	287	301	42.41
5287	VLEHVVRVNARVRIA	DRB1*14:32	P43358	MAGE4	287	301	24.5
5288	VLEYVIKVSARVRFF	DRB1*01:01	P43355	MAGE1	279	293	11.83
5289	VLEYVIKVSARVRFF	DRB1*01:11	P43355	MAGE1	279	293	18.7
5290	VLEYVIKVSARVRFF	DRB1*01:18	P43355	MAGE1	279	293	7.97
5291	VLEYVIKVSARVRFF	DRB1*01:20	P43355	MAGE1	279	293	19.43
5292	VLEYVIKVSARVRFF	DRB1*01:24	P43355	MAGE1	279	293	18.49
5293	VLEYVIKVSARVRFF	DRB1*01:29	P43355	MAGE1	279	293	17.09
5294	VLEYVIKVSARVRFF	DRB1*03:01	P43355	MAGE1	279	293	43.72
5295	VLEYVIKVSARVRFF	DRB1*03:04	P43355	MAGE1	279	293	43.72
5296	VLEYVIKVSARVRFF	DRB1*03:11	P43355	MAGE1	279	293	20.27
5297	VLEYVIKVSARVRFF	DRB1*03:13	P43355	MAGE1	279	293	43.72
5298	VLEYVIKVSARVRFF	DRB1*03:15	P43355	MAGE1	279	293	35.85
5299	VLEYVIKVSARVRFF	DRB1*07:01	P43355	MAGE1	279	293	38.63
5300	VLEYVIKVSARVRFF	DRB1*08:04	P43355	MAGE1	279	293	33.8
5301	VLEYVIKVSARVRFF	DRB1*08:30	P43355	MAGE1	279	293	40.81
5302	VLEYVIKVSARVRFF	DRB1*10:01	P43355	MAGE1	279	293	43.12
5303	VLEYVIKVSARVRFF	DRB1*11:01	P43355	MAGE1	279	293	31.77
5304	VLEYVIKVSARVRFF	DRB1*11:02	P43355	MAGE1	279	293	9.97
5305	VLEYVIKVSARVRFF	DRB1*11:03	P43355	MAGE1	279	293	17.85
5306	VLEYVIKVSARVRFF	DRB1*11:04	P43355	MAGE1	279	293	16.18
5307	VLEYVIKVSARVRFF	DRB1*11:08	P43355	MAGE1	279	293	27.01
5308	VLEYVIKVSARVRFF	DRB1*11:10	P43355	MAGE1	279	293	31.77
5309	VLEYVIKVSARVRFF	DRB1*11:12	P43355	MAGE1	279	293	31.77
5310	VLEYVIKVSARVRFF	DRB1*11:13	P43355	MAGE1	279	293	10.92
5311	VLEYVIKVSARVRFF	DRB1*11:14	P43355	MAGE1	279	293	25.89
5312	VLEYVIKVSARVRFF	DRB1*11:19	P43355	MAGE1	279	293	39.25
5313	VLEYVIKVSARVRFF	DRB1*11:28	P43355	MAGE1	279	293	31.77
5314	VLEYVIKVSARVRFF	DRB1*11:29	P43355	MAGE1	279	293	31.77
5315	VLEYVIKVSARVRFF	DRB1*11:42	P43355	MAGE1	279	293	10.57
5316	VLEYVIKVSARVRFF	DRB1*11:46	P43355	MAGE1	279	293	16.18

5317	VLEYVIKVSARVRFF	DRB1*11:49	P43355	MAGE1	279	293	31.77
5318	VLEYVIKVSARVRFF	DRB1*11:58	P43355	MAGE1	279	293	16.18
5319	VLEYVIKVSARVRFF	DRB1*11:62	P43355	MAGE1	279	293	31.77
5320	VLEYVIKVSARVRFF	DRB1*11:65	P43355	MAGE1	279	293	9.97
5321	VLEYVIKVSARVRFF	DRB1*11:74	P43355	MAGE1	279	293	31.77
5322	VLEYVIKVSARVRFF	DRB1*11:84	P43355	MAGE1	279	293	27.42
5323	VLEYVIKVSARVRFF	DRB1*12:03	P43355	MAGE1	279	293	39.35
5324	VLEYVIKVSARVRFF	DRB1*13:01	P43355	MAGE1	279	293	9.97
5325	VLEYVIKVSARVRFF	DRB1*13:02	P43355	MAGE1	279	293	25.89
5326	VLEYVIKVSARVRFF	DRB1*13:05	P43355	MAGE1	279	293	31.77
5327	VLEYVIKVSARVRFF	DRB1*13:11	P43355	MAGE1	279	293	16.18
5328	VLEYVIKVSARVRFF	DRB1*13:14	P43355	MAGE1	279	293	31.77
5329	VLEYVIKVSARVRFF	DRB1*13:21	P43355	MAGE1	279	293	39.34
5330	VLEYVIKVSARVRFF	DRB1*13:23	P43355	MAGE1	279	293	25.89
5331	VLEYVIKVSARVRFF	DRB1*13:50	P43355	MAGE1	279	293	31.77
5332	VLEYVIKVSARVRFF	DRB1*13:61	P43355	MAGE1	279	293	20.95
5333	VLEYVIKVSARVRFF	DRB1*13:66	P43355	MAGE1	279	293	49.8
5334	VLEYVIKVSARVRFF	DRB1*13:96	P43355	MAGE1	279	293	33.05
5335	VLEYVIKVSARVRFF	DRB1*13:97	P43355	MAGE1	279	293	25.89
5336	VLEYVIKVSARVRFF	DRB1*14:01	P43355	MAGE1	279	293	21.81
5337	VLEYVIKVSARVRFF	DRB1*14:04	P43355	MAGE1	279	293	39.3
5338	VLEYVIKVSARVRFF	DRB1*14:05	P43355	MAGE1	279	293	43.45
5339	VLEYVIKVSARVRFF	DRB1*14:06	P43355	MAGE1	279	293	24.13
5340	VLEYVIKVSARVRFF	DRB1*14:12	P43355	MAGE1	279	293	31.56
5341	VLEYVIKVSARVRFF	DRB1*14:23	P43355	MAGE1	279	293	43.45
5342	VLEYVIKVSARVRFF	DRB1*14:32	P43355	MAGE1	279	293	14.14
5343	VLEYVIKVSARVRFF	DRB1*14:38	P43355	MAGE1	279	293	40.51
5344	VLEYVIKVSARVRFF	DRB1*14:54	P43355	MAGE1	279	293	21.81
5345	VLEYVIKVSARVRFF	DRB1*15:01	P43355	MAGE1	279	293	43.36
5346	VLEYVIKVSARVRFF	DRB1*15:02	P43355	MAGE1	279	293	47.33
5347	VLEYVIKVSARVRFF	DRB1*15:06	P43355	MAGE1	279	293	43.36
5348	VLEYVIKVSARVRFF	DRB1*15:15	P43355	MAGE1	279	293	45.8
5349	VLEYVIKVSARVRFF	DRB1*16:02	P43355	MAGE1	279	293	39.58
5350	VLEYVIKVSARVRFF	DRB1*16:09	P43355	MAGE1	279	293	34.95
5351	VLHQILKGGSGTYCL	DRB1*11:04	P40967	PMEL17	553	567	49.95
5352	VLHQILKGGSGTYCL	DRB1*11:46	P40967	PMEL17	553	567	49.95
5353	VLHQILKGGSGTYCL	DRB1*11:58	P40967	PMEL17	553	567	49.95
5354	VLHQILKGGSGTYCL	DRB1*13:11	P40967	PMEL17	553	567	49.95
5355	VLKRCLLHLAVIGAL	DRB1*01:01	P40967	PMEL17	4	18	35.25
5356	VLKRCLLHLAVIGAL	DRB1*01:18	P40967	PMEL17	4	18	23.95

5357	VLKRCLLHLAVIGAL	DRB1*01:20	P40967	PMEL17	4	18	31.22
5358	VLKRCLLHLAVIGAL	DRB1*01:24	P40967	PMEL17	4	18	45.78
5359	VLKRCLLHLAVIGAL	DRB1*01:29	P40967	PMEL17	4	18	48.64
5360	VLLKEFTVSGNILTI	DRB1*01:01	P78358	NY-ESO-1	121	135	23.95
5361	VLLKEFTVSGNILTI	DRB1*01:11	P78358	NY-ESO-1	121	135	48
5362	VLLKEFTVSGNILTI	DRB1*01:18	P78358	NY-ESO-1	121	135	22.94
5363	VLLKEFTVSGNILTI	DRB1*01:24	P78358	NY-ESO-1	121	135	36.91
5364	VLLKEFTVSGNILTI	DRB1*04:01	P78358	NY-ESO-1	121	135	48.48
5365	VLLKEFTVSGNILTI	DRB1*04:08	P78358	NY-ESO-1	121	135	43.81
5366	VLLKEFTVSGNILTI	DRB1*07:01	P78358	NY-ESO-1	121	135	31.61
5367	VLLKEFTVSGNILTI	DRB1*10:01	P78358	NY-ESO-1	121	135	46.32
5368	VLLKEFTVSGNILTI	DRB1*11:14	P78358	NY-ESO-1	121	135	49.15
5369	VLLKEFTVSGNILTI	DRB1*13:02	P78358	NY-ESO-1	121	135	49.15
5370	VLLKEFTVSGNILTI	DRB1*13:23	P78358	NY-ESO-1	121	135	49.15
5371	VLLKEFTVSGNILTI	DRB1*13:97	P78358	NY-ESO-1	121	135	49.15
5372	VLMAVVLASLIYRRR	DRB1*01:18	P40967	PMEL17	605	619	38.07
5373	VLMAVVLASLIYRRR	DRB1*01:20	P40967	PMEL17	605	619	35.51
5374	VLMAVVLASLIYRRR	DRB1*11:02	P40967	PMEL17	605	619	37.73
5375	VLMAVVLASLIYRRR	DRB1*11:13	P40967	PMEL17	605	619	33.79
5376	VLMAVVLASLIYRRR	DRB1*11:42	P40967	PMEL17	605	619	34.16
5377	VLMAVVLASLIYRRR	DRB1*11:65	P40967	PMEL17	605	619	37.73
5378	VLMAVVLASLIYRRR	DRB1*13:01	P40967	PMEL17	605	619	37.73
5379	VLMAVVLASLIYRRR	DRB1*14:06	P40967	PMEL17	605	619	45.12
5380	VLTALLAGLVSLLCR	DRB1*01:01	P14679	Tyrosinase	487	501	30.19
5381	VLTALLAGLVSLLCR	DRB1*01:18	P14679	Tyrosinase	487	501	22.16
5382	VLTALLAGLVSLLCR	DRB1*01:20	P14679	Tyrosinase	487	501	27.38
5383	VLTALLAGLVSLLCR	DRB1*01:24	P14679	Tyrosinase	487	501	31.75
5384	VLTALLAGLVSLLCR	DRB1*01:29	P14679	Tyrosinase	487	501	46.6
5385	VLVMIAMEGGHAPEE	DRB1*01:01	P43355	MAGE1	198	212	16.47
5386	VLVMIAMEGGHAPEE	DRB1*01:02	P43355	MAGE1	198	212	37.74
5387	VLVMIAMEGGHAPEE	DRB1*01:18	P43355	MAGE1	198	212	17.79
5388	VLVMIAMEGGHAPEE	DRB1*01:20	P43355	MAGE1	198	212	14.12
5389	VLVMIAMEGGHAPEE	DRB1*01:29	P43355	MAGE1	198	212	41.18
5390	VLYCLLWSFQTSAGH	DRB1*01:01	P14679	Tyrosinase	5	19	40.64
5391	VLYCLLWSFQTSAGH	DRB1*01:18	P14679	Tyrosinase	5	19	36.26
5392	VLYRYGSFSVTLDIV	DPB1*33:01	P40967	PMEL17	476	490	26.84
5393	VLYRYGSFSVTLDIV	DPB1*71:01	P40967	PMEL17	476	490	26.84
5394	VLYRYGSFSVTLDIV	DRB1*04:05	P40967	PMEL17	476	490	26.64
5395	VLYRYGSFSVTLDIV	DRB1*10:01	P40967	PMEL17	476	490	39.11
5396	VLYRYGSFSVTLDIV	DRB1*15:01	P40967	PMEL17	476	490	22.92

5397	VLYRYGSFSVTLDIV	DRB1*15:02	P40967	PMEL17	476	490	48.99
5398	VLYRYGSFSVTLDIV	DRB1*15:06	P40967	PMEL17	476	490	22.92
5399	VMIAMEGGHAPEEEI	DRB1*01:01	P43355	MAGE1	200	214	47.22
5400	VMIAMEGGHAPEEEI	DRB1*01:20	P43355	MAGE1	200	214	35.4
5401	VNARVRIAYPSLREA	DRB1*01:20	P43358	MAGE4	294	308	35.36
5402	VNARVRIAYPSLREA	DRB1*03:11	P43358	MAGE4	294	308	44.53
5403	VNARVRIAYPSLREA	DRB1*11:02	P43358	MAGE4	294	308	37.07
5404	VNARVRIAYPSLREA	DRB1*11:13	P43358	MAGE4	294	308	47.15
5405	VNARVRIAYPSLREA	DRB1*11:42	P43358	MAGE4	294	308	33.84
5406	VNARVRIAYPSLREA	DRB1*11:65	P43358	MAGE4	294	308	37.07
5407	VNARVRIAYPSLREA	DRB1*13:01	P43358	MAGE4	294	308	37.07
5408	VPFIPLYRNGDFFIS	DRB1*08:01	P14679	Tyrosinase	427	441	48.28
5409	VPFIPLYRNGDFFIS	DRB1*11:01	P14679	Tyrosinase	427	441	41.52
5410	VPFIPLYRNGDFFIS	DRB1*11:10	P14679	Tyrosinase	427	441	41.52
5411	VPFIPLYRNGDFFIS	DRB1*11:12	P14679	Tyrosinase	427	441	41.52
5412	VPFIPLYRNGDFFIS	DRB1*11:28	P14679	Tyrosinase	427	441	41.52
5413	VPFIPLYRNGDFFIS	DRB1*11:29	P14679	Tyrosinase	427	441	41.52
5414	VPFIPLYRNGDFFIS	DRB1*11:49	P14679	Tyrosinase	427	441	41.52
5415	VPFIPLYRNGDFFIS	DRB1*11:62	P14679	Tyrosinase	427	441	41.52
5416	VPFIPLYRNGDFFIS	DRB1*11:74	P14679	Tyrosinase	427	441	41.52
5417	VPFIPLYRNGDFFIS	DRB1*13:05	P14679	Tyrosinase	427	441	41.52
5418	VPFIPLYRNGDFFIS	DRB1*13:14	P14679	Tyrosinase	427	441	41.52
5419	VPFIPLYRNGDFFIS	DRB1*13:21	P14679	Tyrosinase	427	441	15.28
5420	VPFIPLYRNGDFFIS	DRB1*13:50	P14679	Tyrosinase	427	441	41.52
5421	VPLDCVLYRYGSFSV	DRB1*15:01	P40967	PMEL17	471	485	42.2
5422	VPLDCVLYRYGSFSV	DRB1*15:06	P40967	PMEL17	471	485	42.2
5423	VRALDMAKRTTHPLF	DRB1*11:03	P17643	TRP1	145	159	47.66
5424	VRALDMAKRTTHPLF	DRB1*11:04	P17643	TRP1	145	159	31.74
5425	VRALDMAKRTTHPLF	DRB1*11:42	P17643	TRP1	145	159	38.2
5426	VRALDMAKRTTHPLF	DRB1*11:46	P17643	TRP1	145	159	31.74
5427	VRALDMAKRTTHPLF	DRB1*11:58	P17643	TRP1	145	159	31.74
5428	VRALDMAKRTTHPLF	DRB1*13:11	P17643	TRP1	145	159	31.74
5429	VRDTLLGGFFPWLKV	DPB1*33:01	O75767	TRP2	193	207	22.03
5430	VRDTLLGGFFPWLKV	DPB1*71:01	O75767	TRP2	193	207	22.03
5431	VRFFFPSLREAALRE	DRB1*01:01	P43355	MAGE1	290	304	39.1
5432	VRFFFPSLREAALRE	DRB1*01:18	P43355	MAGE1	290	304	35.95
5433	VRFFFPSLREAALRE	DRB1*08:24	P43355	MAGE1	290	304	48.96
5434	VRFFFPSLREAALRE	DRB1*10:01	P43355	MAGE1	290	304	37.99
5435	VRFFFPSLREAALRE	DRB1*11:01	P43355	MAGE1	290	304	14.6
5436	VRFFFPSLREAALRE	DRB1*11:04	P43355	MAGE1	290	304	38.38

5437	VRFFFPSLREAALRE	DRB1*11:08	P43355	MAGE1	290	304	33.35
5438	VRFFFPSLREAALRE	DRB1*11:10	P43355	MAGE1	290	304	14.6
5439	VRFFFPSLREAALRE	DRB1*11:12	P43355	MAGE1	290	304	14.6
5440	VRFFFPSLREAALRE	DRB1*11:27	P43355	MAGE1	290	304	44.98
5441	VRFFFPSLREAALRE	DRB1*11:28	P43355	MAGE1	290	304	14.6
5442	VRFFFPSLREAALRE	DRB1*11:29	P43355	MAGE1	290	304	14.6
5443	VRFFFPSLREAALRE	DRB1*11:37	P43355	MAGE1	290	304	23.67
5444	VRFFFPSLREAALRE	DRB1*11:42	P43355	MAGE1	290	304	48.96
5445	VRFFFPSLREAALRE	DRB1*11:46	P43355	MAGE1	290	304	38.38
5446	VRFFFPSLREAALRE	DRB1*11:49	P43355	MAGE1	290	304	14.6
5447	VRFFFPSLREAALRE	DRB1*11:58	P43355	MAGE1	290	304	38.38
5448	VRFFFPSLREAALRE	DRB1*11:62	P43355	MAGE1	290	304	14.6
5449	VRFFFPSLREAALRE	DRB1*11:74	P43355	MAGE1	290	304	14.6
5450	VRFFFPSLREAALRE	DRB1*13:05	P43355	MAGE1	290	304	14.6
5451	VRFFFPSLREAALRE	DRB1*13:07	P43355	MAGE1	290	304	23.67
5452	VRFFFPSLREAALRE	DRB1*13:11	P43355	MAGE1	290	304	38.38
5453	VRFFFPSLREAALRE	DRB1*13:14	P43355	MAGE1	290	304	14.6
5454	VRFFFPSLREAALRE	DRB1*13:21	P43355	MAGE1	290	304	19.25
5455	VRFFFPSLREAALRE	DRB1*13:50	P43355	MAGE1	290	304	14.6
5456	VRSLHNLAHLFLNGT	DRB1*01:18	P17643	TRP1	373	387	48.47
5457	VRSLHNLAHLFLNGT	DRB1*01:20	P17643	TRP1	373	387	47.37
5458	VRVNARVRIAYPSLR	DRB1*11:02	P43358	MAGE4	292	306	24.23
5459	VRVNARVRIAYPSLR	DRB1*11:03	P43358	MAGE4	292	306	31.25
5460	VRVNARVRIAYPSLR	DRB1*11:13	P43358	MAGE4	292	306	36.81
5461	VRVNARVRIAYPSLR	DRB1*11:42	P43358	MAGE4	292	306	36
5462	VRVNARVRIAYPSLR	DRB1*11:65	P43358	MAGE4	292	306	24.23
5463	VRVNARVRIAYPSLR	DRB1*13:01	P43358	MAGE4	292	306	24.23
5464	VSARVRFFFPSLREA	DPB1*33:01	P43355	MAGE1	286	300	34.91
5465	VSARVRFFFPSLREA	DPB1*71:01	P43355	MAGE1	286	300	34.91
5466	VSARVRFFFPSLREA	DRB1*01:20	P43355	MAGE1	286	300	39.61
5467	VSARVRFFFPSLREA	DRB1*15:01	P43355	MAGE1	286	300	42.26
5468	VSARVRFFFPSLREA	DRB1*15:03	P43355	MAGE1	286	300	44.58
5469	VSARVRFFFPSLREA	DRB1*15:06	P43355	MAGE1	286	300	42.26
5470	VSGLSIGTGRAMLGT	DRB1*01:01	P40967	PMEL17	167	181	31.26
5471	VSGLSIGTGRAMLGT	DRB1*01:18	P40967	PMEL17	167	181	34.18
5472	VSGLSIGTGRAMLGT	DRB1*01:20	P40967	PMEL17	167	181	21.94
5473	VSGNILTIRLTAADH	DRB1*01:20	P78358	NY-ESO-1	128	142	47.44
5474	VSGNILTIRLTAADH	DRB1*11:42	P78358	NY-ESO-1	128	142	48.71
5475	VSIVVLSGTTAAQVT	DRB1*01:01	P40967	PMEL17	405	419	11.23
5476	VSIVVLSGTTAAQVT	DRB1*01:02	P40967	PMEL17	405	419	45.22

5477	VSIVVLSGTTAAQVT	DRB1*01:11	P40967	PMEL17	405	419	21.57
5478	VSIVVLSGTTAAQVT	DRB1*01:18	P40967	PMEL17	405	419	10.11
5479	VSIVVLSGTTAAQVT	DRB1*01:20	P40967	PMEL17	405	419	11.75
5480	VSIVVLSGTTAAQVT	DRB1*01:24	P40967	PMEL17	405	419	18.37
5481	VSIVVLSGTTAAQVT	DRB1*01:29	P40967	PMEL17	405	419	21.95
5482	VSIVVLSGTTAAQVT	DRB1*04:04	P40967	PMEL17	405	419	25.56
5483	VSIVVLSGTTAAQVT	DRB1*04:08	P40967	PMEL17	405	419	45.64
5484	VSIVVLSGTTAAQVT	DRB1*04:44	P40967	PMEL17	405	419	47.75
5485	VSIVVLSGTTAAQVT	DRB1*07:01	P40967	PMEL17	405	419	29.66
5486	VSIVVLSGTTAAQVT	DRB1*10:01	P40967	PMEL17	405	419	40.57
5487	VSIVVLSGTTAAQVT	DRB1*11:14	P40967	PMEL17	405	419	43.21
5488	VSIVVLSGTTAAQVT	DRB1*13:02	P40967	PMEL17	405	419	43.21
5489	VSIVVLSGTTAAQVT	DRB1*13:23	P40967	PMEL17	405	419	43.21
5490	VSIVVLSGTTAAQVT	DRB1*13:97	P40967	PMEL17	405	419	43.21
5491	VSLLCRHKRKQLPEE	DRB1*11:03	P14679	Tyrosinase	496	510	34.76
5492	VSVSQLRALDGGNKH	DRB1*01:20	P40967	PMEL17	217	231	31.55
5493	VTAQVVLQAAIPLTS	DRB1*01:01	P40967	PMEL17	286	300	6.37
5494	VTAQVVLQAAIPLTS	DRB1*01:02	P40967	PMEL17	286	300	15.58
5495	VTAQVVLQAAIPLTS	DRB1*01:11	P40967	PMEL17	286	300	13.13
5496	VTAQVVLQAAIPLTS	DRB1*01:18	P40967	PMEL17	286	300	6.37
5497	VTAQVVLQAAIPLTS	DRB1*01:20	P40967	PMEL17	286	300	5.84
5498	VTAQVVLQAAIPLTS	DRB1*01:24	P40967	PMEL17	286	300	12.6
5499	VTAQVVLQAAIPLTS	DRB1*01:29	P40967	PMEL17	286	300	12.98
5500	VTAQVVLQAAIPLTS	DRB1*10:01	P40967	PMEL17	286	300	22.84
5501	VTAQVVLQAAIPLTS	DRB1*11:02	P40967	PMEL17	286	300	39.64
5502	VTAQVVLQAAIPLTS	DRB1*11:13	P40967	PMEL17	286	300	38.25
5503	VTAQVVLQAAIPLTS	DRB1*11:14	P40967	PMEL17	286	300	20.55
5504	VTAQVVLQAAIPLTS	DRB1*11:42	P40967	PMEL17	286	300	47.48
5505	VTAQVVLQAAIPLTS	DRB1*11:65	P40967	PMEL17	286	300	39.64
5506	VTAQVVLQAAIPLTS	DRB1*12:03	P40967	PMEL17	286	300	48.5
5507	VTAQVVLQAAIPLTS	DRB1*13:01	P40967	PMEL17	286	300	39.64
5508	VTAQVVLQAAIPLTS	DRB1*13:02	P40967	PMEL17	286	300	20.55
5509	VTAQVVLQAAIPLTS	DRB1*13:23	P40967	PMEL17	286	300	20.55
5510	VTAQVVLQAAIPLTS	DRB1*13:96	P40967	PMEL17	286	300	39.8
5511	VTAQVVLQAAIPLTS	DRB1*13:97	P40967	PMEL17	286	300	20.55
5512	VTAQVVLQAAIPLTS	DRB1*14:32	P40967	PMEL17	286	300	34.94
5513	VTAQVVLQAAIPLTS	DRB1*15:01	P40967	PMEL17	286	300	35.4
5514	VTAQVVLQAAIPLTS	DRB1*15:06	P40967	PMEL17	286	300	35.4
5515	VTKAEMLERVIKNYK	DRB1*11:42	P43358	MAGE4	131	145	48.8
5516	VTVYHRRGSRSYVPL	DRB1*11:03	P40967	PMEL17	186	200	48.46

5517	VVLASLIYRRRLMKQ	DRB1*01:20	P40967	PMEL17	609	623	46.02
5518	VVLASLIYRRRLMKQ	DRB1*11:02	P40967	PMEL17	609	623	15.16
5519	VVLASLIYRRRLMKQ	DRB1*11:03	P40967	PMEL17	609	623	12.36
5520	VVLASLIYRRRLMKQ	DRB1*11:04	P40967	PMEL17	609	623	19.21
5521	VVLASLIYRRRLMKQ	DRB1*11:13	P40967	PMEL17	609	623	28.73
5522	VVLASLIYRRRLMKQ	DRB1*11:42	P40967	PMEL17	609	623	18.47
5523	VVLASLIYRRRLMKQ	DRB1*11:46	P40967	PMEL17	609	623	19.21
5524	VVLASLIYRRRLMKQ	DRB1*11:58	P40967	PMEL17	609	623	19.21
5525	VVLASLIYRRRLMKQ	DRB1*11:65	P40967	PMEL17	609	623	15.16
5526	VVLASLIYRRRLMKQ	DRB1*11:84	P40967	PMEL17	609	623	35.36
5527	VVLASLIYRRRLMKQ	DRB1*13:01	P40967	PMEL17	609	623	15.16
5528	VVLASLIYRRRLMKQ	DRB1*13:11	P40967	PMEL17	609	623	19.21
5529	VVLASLIYRRRLMKQ	DRB1*13:21	P40967	PMEL17	609	623	38.83
5530	VVLASLIYRRRLMKQ	DRB1*13:61	P40967	PMEL17	609	623	45.4
5531	VVLASLIYRRRLMKQ	DRB1*14:06	P40967	PMEL17	609	623	38.33
5532	VVLASLIYRRRLMKQ	DRB1*14:12	P40967	PMEL17	609	623	47.51
5533	VVLQAAIPLTSCGSS	DRB1*01:01	P40967	PMEL17	290	304	36.79
5534	VVLQAAIPLTSCGSS	DRB1*01:18	P40967	PMEL17	290	304	34.52
5535	VVLQAAIPLTSCGSS	DRB1*01:20	P40967	PMEL17	290	304	25.6
5536	VVLQAAIPLTSCGSS	DRB1*10:01	P40967	PMEL17	290	304	36.16
5537	VVRVNARVRIAYPSL	DRB1*11:02	P43358	MAGE4	291	305	16.81
5538	VVRVNARVRIAYPSL	DRB1*11:03	P43358	MAGE4	291	305	22.84
5539	VVRVNARVRIAYPSL	DRB1*11:13	P43358	MAGE4	291	305	25.05
5540	VVRVNARVRIAYPSL	DRB1*11:14	P43358	MAGE4	291	305	32.87
5541	VVRVNARVRIAYPSL	DRB1*11:42	P43358	MAGE4	291	305	25.48
5542	VVRVNARVRIAYPSL	DRB1*11:65	P43358	MAGE4	291	305	16.81
5543	VVRVNARVRIAYPSL	DRB1*13:01	P43358	MAGE4	291	305	16.81
5544	VVRVNARVRIAYPSL	DRB1*13:02	P43358	MAGE4	291	305	32.87
5545	VVRVNARVRIAYPSL	DRB1*13:23	P43358	MAGE4	291	305	32.87
5546	VVRVNARVRIAYPSL	DRB1*13:61	P43358	MAGE4	291	305	30.1
5547	VVRVNARVRIAYPSL	DRB1*13:96	P43358	MAGE4	291	305	44.43
5548	VVRVNARVRIAYPSL	DRB1*13:97	P43358	MAGE4	291	305	32.87
5549	VVRVNARVRIAYPSL	DRB1*14:06	P43358	MAGE4	291	305	48.86
5550	VVRVNARVRIAYPSL	DRB1*14:32	P43358	MAGE4	291	305	37.95
5551	VWLHYYSVRDTLLGG	DRB1*01:01	O75767	TRP2	186	200	46.44
5552	VWLHYYSVRDTLLGG	DRB1*01:18	O75767	TRP2	186	200	43.62
5553	VWMHYYVSMDALLGG	DRB1*01:01	P14679	Tyrosinase	177	191	6.98
5554	VWMHYYVSMDALLGG	DRB1*01:11	P14679	Tyrosinase	177	191	21.13
5555	VWMHYYVSMDALLGG	DRB1*01:18	P14679	Tyrosinase	177	191	7.47
5556	VWMHYYVSMDALLGG	DRB1*01:20	P14679	Tyrosinase	177	191	30.03

5557	VWMHYYVSMDALLGG	DRB1*01:24	P14679	Tyrosinase	177	191	15.9
5558	VWMHYYVSMDALLGG	DRB1*01:29	P14679	Tyrosinase	177	191	15.4
5559	VWMHYYVSMDALLGG	DRB1*04:01	P14679	Tyrosinase	177	191	38.16
5560	VWMHYYVSMDALLGG	DRB1*04:05	P14679	Tyrosinase	177	191	38.24
5561	VWMHYYVSMDALLGG	DRB1*04:08	P14679	Tyrosinase	177	191	29.69
5562	VWMHYYVSMDALLGG	DRB1*10:01	P14679	Tyrosinase	177	191	10.07
5563	VWMHYYVSMDALLGG	DRB1*16:02	P14679	Tyrosinase	177	191	41.31
5564	VWPLRFFNRTCHCNG	DRB1*11:03	P17643	TRP1	89	103	45.96
5565	VWTHYYSVKKTFLGV	DRB1*01:01	P17643	TRP1	189	203	46.38
5566	VWTHYYSVKKTFLGV	DRB1*01:18	P17643	TRP1	189	203	36.71
5567	VWTHYYSVKKTFLGV	DRB1*11:01	P17643	TRP1	189	203	31.01
5568	VWTHYYSVKKTFLGV	DRB1*11:10	P17643	TRP1	189	203	31.01
5569	VWTHYYSVKKTFLGV	DRB1*11:12	P17643	TRP1	189	203	31.01
5570	VWTHYYSVKKTFLGV	DRB1*11:28	P17643	TRP1	189	203	31.01
5571	VWTHYYSVKKTFLGV	DRB1*11:29	P17643	TRP1	189	203	31.01
5572	VWTHYYSVKKTFLGV	DRB1*11:49	P17643	TRP1	189	203	31.01
5573	VWTHYYSVKKTFLGV	DRB1*11:62	P17643	TRP1	189	203	31.01
5574	VWTHYYSVKKTFLGV	DRB1*11:74	P17643	TRP1	189	203	31.01
5575	VWTHYYSVKKTFLGV	DRB1*13:05	P17643	TRP1	189	203	31.01
5576	VWTHYYSVKKTFLGV	DRB1*13:14	P17643	TRP1	189	203	31.01
5577	VWTHYYSVKKTFLGV	DRB1*13:21	P17643	TRP1	189	203	30.57
5578	VWTHYYSVKKTFLGV	DRB1*13:50	P17643	TRP1	189	203	31.01
5579	VYDFFVWLHYYSVRD	DPB1*01:01	O75767	TRP2	181	195	49.56
5580	VYDFFVWLHYYSVRD	DPB1*02:01	O75767	TRP2	181	195	13.62
5581	VYDFFVWLHYYSVRD	DPB1*02:02	O75767	TRP2	181	195	14.25
5582	VYDFFVWLHYYSVRD	DPB1*04:01	O75767	TRP2	181	195	17.12
5583	VYDFFVWLHYYSVRD	DPB1*126:01	O75767	TRP2	181	195	17.12
5584	VYDFFVWLHYYSVRD	DPB1*15:01	O75767	TRP2	181	195	31.55
5585	VYDFFVWLHYYSVRD	DPB1*23:01	O75767	TRP2	181	195	17.12
5586	VYDFFVWLHYYSVRD	DPB1*33:01	O75767	TRP2	181	195	6.9
5587	VYDFFVWLHYYSVRD	DPB1*39:01	O75767	TRP2	181	195	17.12
5588	VYDFFVWLHYYSVRD	DPB1*46:01	O75767	TRP2	181	195	13.62
5589	VYDFFVWLHYYSVRD	DPB1*47:01	O75767	TRP2	181	195	14.25
5590	VYDFFVWLHYYSVRD	DPB1*71:01	O75767	TRP2	181	195	6.9
5591	VYDFFVWLHYYSVRD	DPB1*72:01	O75767	TRP2	181	195	22.94
5592	VYDFFVWLHYYSVRD	DPB1*81:01	O75767	TRP2	181	195	13.62
5593	VYDFFVWLHYYSVRD	DRB1*01:18	O75767	TRP2	181	195	32.05
5594	VYYYRFVIGLRVWQW	DRB1*01:01	O75767	TRP2	207	221	8.56
5595	VYYYRFVIGLRVWQW	DRB1*01:11	O75767	TRP2	207	221	11.19
5596	VYYYRFVIGLRVWQW	DRB1*01:18	O75767	TRP2	207	221	6.24

5597	VYYYRFVIGLRVWQW	DRB1*01:20	O75767	TRP2	207	221	29.94
5598	VYYYRFVIGLRVWQW	DRB1*01:24	O75767	TRP2	207	221	7.21
5599	VYYYRFVIGLRVWQW	DRB1*01:29	O75767	TRP2	207	221	11.62
5600	VYYYRFVIGLRVWQW	DRB1*07:01	O75767	TRP2	207	221	17.44
5601	VYYYRFVIGLRVWQW	DRB1*10:01	O75767	TRP2	207	221	24.36
5602	VYYYRFVIGLRVWQW	DRB1*15:02	O75767	TRP2	207	221	29.64
5603	VYYYRFVIGLRVWQW	DRB1*15:15	O75767	TRP2	207	221	43.09
5604	VYYYRFVIGLRVWQW	DRB1*16:01	O75767	TRP2	207	221	38.89
5605	VYYYRFVIGLRVWQW	DRB1*16:02	O75767	TRP2	207	221	29.7
5606	VYYYRFVIGLRVWQW	DRB1*16:04	O75767	TRP2	207	221	47.58
5607	VYYYRFVIGLRVWQW	DRB1*16:05	O75767	TRP2	207	221	26.4
5608	VYYYRFVIGLRVWQW	DRB1*16:09	O75767	TRP2	207	221	25.57
5609	WEKMKASEKIFYVYM	DRB1*03:11	Q16385	SSX2	37	51	42.21
5610	WEKMKASEKIFYVYM	DRB1*11:02	Q16385	SSX2	37	51	45.94
5611	WEKMKASEKIFYVYM	DRB1*11:13	Q16385	SSX2	37	51	31.7
5612	WEKMKASEKIFYVYM	DRB1*11:42	Q16385	SSX2	37	51	34.7
5613	WEKMKASEKIFYVYM	DRB1*11:65	Q16385	SSX2	37	51	45.94
5614	WEKMKASEKIFYVYM	DRB1*13:01	Q16385	SSX2	37	51	45.94
5615	WEKMKASEKIFYVYM	DRB1*14:01	Q16385	SSX2	37	51	47.73
5616	WEKMKASEKIFYVYM	DRB1*14:32	Q16385	SSX2	37	51	34.22
5617	WEKMKASEKIFYVYM	DRB1*14:54	Q16385	SSX2	37	51	47.73
5618	WEVISCKLIKRATTR	DRB1*08:04	O75767	TRP2	221	235	38.83
5619	WEVISCKLIKRATTR	DRB1*11:01	O75767	TRP2	221	235	36.89
5620	WEVISCKLIKRATTR	DRB1*11:02	O75767	TRP2	221	235	39.15
5621	WEVISCKLIKRATTR	DRB1*11:03	O75767	TRP2	221	235	27.69
5622	WEVISCKLIKRATTR	DRB1*11:04	O75767	TRP2	221	235	22.06
5623	WEVISCKLIKRATTR	DRB1*11:10	O75767	TRP2	221	235	36.89
5624	WEVISCKLIKRATTR	DRB1*11:12	O75767	TRP2	221	235	36.89
5625	WEVISCKLIKRATTR	DRB1*11:13	O75767	TRP2	221	235	44.69
5626	WEVISCKLIKRATTR	DRB1*11:28	O75767	TRP2	221	235	36.89
5627	WEVISCKLIKRATTR	DRB1*11:29	O75767	TRP2	221	235	36.89
5628	WEVISCKLIKRATTR	DRB1*11:42	O75767	TRP2	221	235	23.84
5629	WEVISCKLIKRATTR	DRB1*11:46	O75767	TRP2	221	235	22.06
5630	WEVISCKLIKRATTR	DRB1*11:49	O75767	TRP2	221	235	36.89
5631	WEVISCKLIKRATTR	DRB1*11:58	O75767	TRP2	221	235	22.06
5632	WEVISCKLIKRATTR	DRB1*11:62	O75767	TRP2	221	235	36.89
5633	WEVISCKLIKRATTR	DRB1*11:65	O75767	TRP2	221	235	39.15
5634	WEVISCKLIKRATTR	DRB1*11:74	O75767	TRP2	221	235	36.89
5635	WEVISCKLIKRATTR	DRB1*13:01	O75767	TRP2	221	235	39.15
5636	WEVISCKLIKRATTR	DRB1*13:05	O75767	TRP2	221	235	36.89

5637	WEVISCKLIKRATTR	DRB1*13:11	O75767	TRP2	221	235	22.06
5638	WEVISCKLIKRATTR	DRB1*13:14	O75767	TRP2	221	235	36.89
5639	WEVISCKLIKRATTR	DRB1*13:50	O75767	TRP2	221	235	36.89
5640	WEVISCKLIKRATTR	DRB1*14:06	O75767	TRP2	221	235	48.94
5641	WGPRALAETSYVKVL	DPB1*33:01	P43355	MAGE1	266	280	23.48
5642	WGPRALAETSYVKVL	DPB1*33:01	P43358	MAGE4	274	288	23.48
5643	WGPRALAETSYVKVL	DPB1*71:01	P43355	MAGE1	266	280	23.48
5644	WGPRALAETSYVKVL	DPB1*71:01	P43358	MAGE4	274	288	23.48
5645	WGQYWQVLGGPVSGL	DRB1*01:01	P40967	PMEL17	156	170	7.45
5646	WGQYWQVLGGPVSGL	DRB1*01:11	P40967	PMEL17	156	170	27.26
5647	WGQYWQVLGGPVSGL	DRB1*01:18	P40967	PMEL17	156	170	7.87
5648	WGQYWQVLGGPVSGL	DRB1*01:20	P40967	PMEL17	156	170	25.56
5649	WGQYWQVLGGPVSGL	DRB1*01:24	P40967	PMEL17	156	170	19.21
5650	WGQYWQVLGGPVSGL	DRB1*01:29	P40967	PMEL17	156	170	17.78
5651	WGQYWQVLGGPVSGL	DRB1*07:01	P40967	PMEL17	156	170	40.66
5652	WGQYWQVLGGPVSGL	DRB1*09:01	P40967	PMEL17	156	170	48.18
5653	WGQYWQVLGGPVSGL	DRB1*10:01	P40967	PMEL17	156	170	20.64
5654	WHRLFLLRWEQEIQK	DPB1*33:01	P14679	Tyrosinase	210	224	46.08
5655	WHRLFLLRWEQEIQK	DPB1*71:01	P14679	Tyrosinase	210	224	46.08
5656	WHRYHLLRLEKDMQE	DRB1*10:01	P17643	TRP1	223	237	42.51
5657	WITQCFLPVFLAQPP	DPB1*33:01	P78358	NY-ESO-1	161	175	26.21
5658	WITQCFLPVFLAQPP	DPB1*71:01	P78358	NY-ESO-1	161	175	26.21
5659	WLGVSRQLRTKAWNR	DRB1*11:03	P40967	PMEL17	32	46	33.51
5660	WLGVSRQLRTKAWNR	DRB1*11:04	P40967	PMEL17	32	46	35.36
5661	WLGVSRQLRTKAWNR	DRB1*11:42	P40967	PMEL17	32	46	37.33
5662	WLGVSRQLRTKAWNR	DRB1*11:46	P40967	PMEL17	32	46	35.36
5663	WLGVSRQLRTKAWNR	DRB1*11:58	P40967	PMEL17	32	46	35.36
5664	WLGVSRQLRTKAWNR	DRB1*13:11	P40967	PMEL17	32	46	35.36
5665	WLHYYSVRDTLLGGF	DRB1*01:01	O75767	TRP2	187	201	36.3
5666	WLHYYSVRDTLLGGF	DRB1*01:18	O75767	TRP2	187	201	35.91
5667	WLKVYYYRFVIGLRV	DPB1*33:01	O75767	TRP2	204	218	41.22
5668	WLKVYYYRFVIGLRV	DPB1*71:01	O75767	TRP2	204	218	41.22
5669	WLKVYYYRFVIGLRV	DRB1*01:01	O75767	TRP2	204	218	9.93
5670	WLKVYYYRFVIGLRV	DRB1*01:11	O75767	TRP2	204	218	16.89
5671	WLKVYYYRFVIGLRV	DRB1*01:18	O75767	TRP2	204	218	7.51
5672	WLKVYYYRFVIGLRV	DRB1*01:20	O75767	TRP2	204	218	40.86
5673	WLKVYYYRFVIGLRV	DRB1*01:24	O75767	TRP2	204	218	10.06
5674	WLKVYYYRFVIGLRV	DRB1*01:29	O75767	TRP2	204	218	16.28
5675	WLKVYYYRFVIGLRV	DRB1*07:01	O75767	TRP2	204	218	9.46
5676	WLKVYYYRFVIGLRV	DRB1*10:01	O75767	TRP2	204	218	25.68

5677	WLKVYYYRFVIGLRV	DRB1*15:02	O75767	TRP2	204	218	33.96
5678	WLKVYYYRFVIGLRV	DRB1*15:15	O75767	TRP2	204	218	40.86
5679	WLKVYYYRFVIGLRV	DRB1*16:01	O75767	TRP2	204	218	45.54
5680	WLKVYYYRFVIGLRV	DRB1*16:02	O75767	TRP2	204	218	31.11
5681	WLKVYYYRFVIGLRV	DRB1*16:05	O75767	TRP2	204	218	32.26
5682	WLKVYYYRFVIGLRV	DRB1*16:09	O75767	TRP2	204	218	26.84
5683	WMHYYVSMDALLGGS	DRB1*01:01	P14679	Tyrosinase	178	192	6.14
5684	WMHYYVSMDALLGGS	DRB1*01:11	P14679	Tyrosinase	178	192	17.88
5685	WMHYYVSMDALLGGS	DRB1*01:18	P14679	Tyrosinase	178	192	6.47
5686	WMHYYVSMDALLGGS	DRB1*01:20	P14679	Tyrosinase	178	192	23.91
5687	WMHYYVSMDALLGGS	DRB1*01:24	P14679	Tyrosinase	178	192	13.67
5688	WMHYYVSMDALLGGS	DRB1*01:29	P14679	Tyrosinase	178	192	12.32
5689	WMHYYVSMDALLGGS	DRB1*04:01	P14679	Tyrosinase	178	192	32.52
5690	WMHYYVSMDALLGGS	DRB1*04:05	P14679	Tyrosinase	178	192	40.42
5691	WMHYYVSMDALLGGS	DRB1*04:08	P14679	Tyrosinase	178	192	26.42
5692	WMHYYVSMDALLGGS	DRB1*10:01	P14679	Tyrosinase	178	192	9.19
5693	WMHYYVSMDALLGGS	DRB1*16:01	P14679	Tyrosinase	178	192	49.25
5694	WMHYYVSMDALLGGS	DRB1*16:02	P14679	Tyrosinase	178	192	34.8
5695	WMHYYVSMDALLGGS	DRB1*16:09	P14679	Tyrosinase	178	192	46.83
5696	WQWEVISCKLIKRAT	DRB1*11:42	O75767	TRP2	219	233	47.17
5697	WRDIDFAHEAPAFLP	DRB1*01:01	P14679	Tyrosinase	195	209	44.47
5698	WSQKRSFVYVWKTWG	DRB1*11:01	P40967	PMEL17	143	157	41.25
5699	WSQKRSFVYVWKTWG	DRB1*11:10	P40967	PMEL17	143	157	41.25
5700	WSQKRSFVYVWKTWG	DRB1*11:12	P40967	PMEL17	143	157	41.25
5701	WSQKRSFVYVWKTWG	DRB1*11:28	P40967	PMEL17	143	157	41.25
5702	WSQKRSFVYVWKTWG	DRB1*11:29	P40967	PMEL17	143	157	41.25
5703	WSQKRSFVYVWKTWG	DRB1*11:49	P40967	PMEL17	143	157	41.25
5704	WSQKRSFVYVWKTWG	DRB1*11:62	P40967	PMEL17	143	157	41.25
5705	WSQKRSFVYVWKTWG	DRB1*11:74	P40967	PMEL17	143	157	41.25
5706	WSQKRSFVYVWKTWG	DRB1*13:05	P40967	PMEL17	143	157	41.25
5707	WSQKRSFVYVWKTWG	DRB1*13:14	P40967	PMEL17	143	157	41.25
5708	WSQKRSFVYVWKTWG	DRB1*13:21	P40967	PMEL17	143	157	41.02
5709	WSQKRSFVYVWKTWG	DRB1*13:50	P40967	PMEL17	143	157	41.25
5710	WSWLLGAAMVGAVLT	DRB1*01:01	P14679	Tyrosinase	475	489	44.08
5711	WSWLLGAAMVGAVLT	DRB1*01:18	P14679	Tyrosinase	475	489	33.19
5712	WTHYYSVKKTFLGVG	DRB1*01:18	P17643	TRP1	190	204	38.39
5713	WTHYYSVKKTFLGVG	DRB1*11:01	P17643	TRP1	190	204	32.76
5714	WTHYYSVKKTFLGVG	DRB1*11:10	P17643	TRP1	190	204	32.76
5715	WTHYYSVKKTFLGVG	DRB1*11:12	P17643	TRP1	190	204	32.76
5716	WTHYYSVKKTFLGVG	DRB1*11:28	P17643	TRP1	190	204	32.76

5717	WTHYYSVKKTFLGVG	DRB1*11:29	P17643	TRP1	190	204	32.76
5718	WTHYYSVKKTFLGVG	DRB1*11:49	P17643	TRP1	190	204	32.76
5719	WTHYYSVKKTFLGVG	DRB1*11:62	P17643	TRP1	190	204	32.76
5720	WTHYYSVKKTFLGVG	DRB1*11:74	P17643	TRP1	190	204	32.76
5721	WTHYYSVKKTFLGVG	DRB1*13:05	P17643	TRP1	190	204	32.76
5722	WTHYYSVKKTFLGVG	DRB1*13:14	P17643	TRP1	190	204	32.76
5723	WTHYYSVKKTFLGVG	DRB1*13:21	P17643	TRP1	190	204	35.86
5724	WTHYYSVKKTFLGVG	DRB1*13:50	P17643	TRP1	190	204	32.76
5725	YCLLWSFQTSAGHFP	DRB1*10:01	P14679	Tyrosinase	7	21	49.84
5726	YDFFVWLHYYSVRDT	DPB1*02:01	O75767	TRP2	182	196	13.64
5727	YDFFVWLHYYSVRDT	DPB1*02:02	O75767	TRP2	182	196	15
5728	YDFFVWLHYYSVRDT	DPB1*04:01	O75767	TRP2	182	196	18.02
5729	YDFFVWLHYYSVRDT	DPB1*126:01	O75767	TRP2	182	196	18.02
5730	YDFFVWLHYYSVRDT	DPB1*15:01	O75767	TRP2	182	196	31.94
5731	YDFFVWLHYYSVRDT	DPB1*23:01	O75767	TRP2	182	196	18.02
5732	YDFFVWLHYYSVRDT	DPB1*33:01	O75767	TRP2	182	196	7.06
5733	YDFFVWLHYYSVRDT	DPB1*39:01	O75767	TRP2	182	196	18.02
5734	YDFFVWLHYYSVRDT	DPB1*46:01	O75767	TRP2	182	196	13.64
5735	YDFFVWLHYYSVRDT	DPB1*47:01	O75767	TRP2	182	196	15
5736	YDFFVWLHYYSVRDT	DPB1*71:01	O75767	TRP2	182	196	7.06
5737	YDFFVWLHYYSVRDT	DPB1*72:01	O75767	TRP2	182	196	23.67
5738	YDFFVWLHYYSVRDT	DPB1*81:01	O75767	TRP2	182	196	13.64
5739	YDFFVWLHYYSVRDT	DRB1*01:01	O75767	TRP2	182	196	42.66
5740	YDFFVWLHYYSVRDT	DRB1*01:18	O75767	TRP2	182	196	27.59
5741	YDLFVWMHYYVSMDA	DPB1*02:02	P14679	Tyrosinase	173	187	45.78
5742	YDLFVWMHYYVSMDA	DPB1*33:01	P14679	Tyrosinase	173	187	17.24
5743	YDLFVWMHYYVSMDA	DPB1*47:01	P14679	Tyrosinase	173	187	45.78
5744	YDLFVWMHYYVSMDA	DPB1*71:01	P14679	Tyrosinase	173	187	17.24
5745	YDPAVRSLHNLAHLF	DRB1*01:18	P17643	TRP1	369	383	46.22
5746	YDPAVRSLHNLAHLF	DRB1*01:20	P17643	TRP1	369	383	31.57
5747	YDPAVRSLHNLAHLF	DRB1*04:04	P17643	TRP1	369	383	30.79
5748	YDPAVRSLHNLAHLF	DRB1*11:42	P17643	TRP1	369	383	46.98
5749	YEFLWGPRALAETSY	DRB1*01:01	P43355	MAGE1	262	276	46.25
5750	YEFLWGPRALAETSY	DRB1*01:01	P43358	MAGE4	270	284	46.25
5751	YEFLWGPRALAETSY	DRB1*01:18	P43355	MAGE1	262	276	39.13
5752	YEFLWGPRALAETSY	DRB1*01:18	P43358	MAGE4	270	284	39.13
5753	YFVWTHYYSVKKTFL	DPB1*01:01	P17643	TRP1	187	201	32.8
5754	YFVWTHYYSVKKTFL	DPB1*02:01	P17643	TRP1	187	201	7.2
5755	YFVWTHYYSVKKTFL	DPB1*02:02	P17643	TRP1	187	201	8.43
5756	YFVWTHYYSVKKTFL	DPB1*04:01	P17643	TRP1	187	201	9.08

5757	YFVWTHYYSVKKTFL	DPB1*04:02	P17643	TRP1	187	201	32.03
5758	YFVWTHYYSVKKTFL	DPB1*105:01	P17643	TRP1	187	201	32.03
5759	YFVWTHYYSVKKTFL	DPB1*126:01	P17643	TRP1	187	201	9.08
5760	YFVWTHYYSVKKTFL	DPB1*15:01	P17643	TRP1	187	201	32.01
5761	YFVWTHYYSVKKTFL	DPB1*16:01	P17643	TRP1	187	201	25.97
5762	YFVWTHYYSVKKTFL	DPB1*23:01	P17643	TRP1	187	201	9.08
5763	YFVWTHYYSVKKTFL	DPB1*33:01	P17643	TRP1	187	201	5.02
5764	YFVWTHYYSVKKTFL	DPB1*34:01	P17643	TRP1	187	201	41.02
5765	YFVWTHYYSVKKTFL	DPB1*39:01	P17643	TRP1	187	201	9.08
5766	YFVWTHYYSVKKTFL	DPB1*40:01	P17643	TRP1	187	201	39.52
5767	YFVWTHYYSVKKTFL	DPB1*41:01	P17643	TRP1	187	201	22.39
5768	YFVWTHYYSVKKTFL	DPB1*46:01	P17643	TRP1	187	201	7.2
5769	YFVWTHYYSVKKTFL	DPB1*47:01	P17643	TRP1	187	201	8.43
5770	YFVWTHYYSVKKTFL	DPB1*49:01	P17643	TRP1	187	201	32.03
5771	YFVWTHYYSVKKTFL	DPB1*71:01	P17643	TRP1	187	201	5.02
5772	YFVWTHYYSVKKTFL	DPB1*72:01	P17643	TRP1	187	201	10.89
5773	YFVWTHYYSVKKTFL	DPB1*81:01	P17643	TRP1	187	201	7.2
5774	YFVWTHYYSVKKTFL	DRB1*01:01	P17643	TRP1	187	201	48.52
5775	YFVWTHYYSVKKTFL	DRB1*01:18	P17643	TRP1	187	201	38.5
5776	YFVWTHYYSVKKTFL	DRB1*07:01	P17643	TRP1	187	201	29.71
5777	YFVWTHYYSVKKTFL	DRB1*13:21	P17643	TRP1	187	201	36.31
5778	YFVWTHYYSVKKTFL	DRB1*15:15	P17643	TRP1	187	201	34.18
5779	YIKSYLEQASRIWSW	DRB1*01:01	P14679	Tyrosinase	463	477	27.34
5780	YIKSYLEQASRIWSW	DRB1*01:18	P14679	Tyrosinase	463	477	23.97
5781	YIKSYLEQASRIWSW	DRB1*01:20	P14679	Tyrosinase	463	477	38.8
5782	YLIRARRSMDEANQP	DRB1*13:21	P17643	TRP1	499	513	45.73
5783	YMKRKYEAMTKLGFK	DRB1*13:21	Q16385	SSX2	50	64	41.62
5784	YMVPFIPLYRNGDFF	DRB1*11:01	P14679	Tyrosinase	425	439	42.2
5785	YMVPFIPLYRNGDFF	DRB1*11:10	P14679	Tyrosinase	425	439	42.2
5786	YMVPFIPLYRNGDFF	DRB1*11:12	P14679	Tyrosinase	425	439	42.2
5787	YMVPFIPLYRNGDFF	DRB1*11:28	P14679	Tyrosinase	425	439	42.2
5788	YMVPFIPLYRNGDFF	DRB1*11:29	P14679	Tyrosinase	425	439	42.2
5789	YMVPFIPLYRNGDFF	DRB1*11:49	P14679	Tyrosinase	425	439	42.2
5790	YMVPFIPLYRNGDFF	DRB1*11:62	P14679	Tyrosinase	425	439	42.2
5791	YMVPFIPLYRNGDFF	DRB1*11:74	P14679	Tyrosinase	425	439	42.2
5792	YMVPFIPLYRNGDFF	DRB1*13:05	P14679	Tyrosinase	425	439	42.2
5793	YMVPFIPLYRNGDFF	DRB1*13:14	P14679	Tyrosinase	425	439	42.2
5794	YMVPFIPLYRNGDFF	DRB1*13:21	P14679	Tyrosinase	425	439	16.43
5795	YMVPFIPLYRNGDFF	DRB1*13:50	P14679	Tyrosinase	425	439	42.2
5796	YNYFVWTHYYSVKKT	DPB1*01:01	P17643	TRP1	185	199	16.47

5797	YNYFVWTHYYSVKKT	DPB1*02:01	P17643	TRP1	185	199	4.64
5798	YNYFVWTHYYSVKKT	DPB1*02:02	P17643	TRP1	185	199	5.48
5799	YNYFVWTHYYSVKKT	DPB1*04:01	P17643	TRP1	185	199	4.79
5800	YNYFVWTHYYSVKKT	DPB1*04:02	P17643	TRP1	185	199	14.45
5801	YNYFVWTHYYSVKKT	DPB1*105:01	P17643	TRP1	185	199	14.45
5802	YNYFVWTHYYSVKKT	DPB1*126:01	P17643	TRP1	185	199	4.79
5803	YNYFVWTHYYSVKKT	DPB1*15:01	P17643	TRP1	185	199	14.8
5804	YNYFVWTHYYSVKKT	DPB1*16:01	P17643	TRP1	185	199	13.47
5805	YNYFVWTHYYSVKKT	DPB1*19:01	P17643	TRP1	185	199	25.99
5806	YNYFVWTHYYSVKKT	DPB1*23:01	P17643	TRP1	185	199	4.79
5807	YNYFVWTHYYSVKKT	DPB1*33:01	P17643	TRP1	185	199	3.63
5808	YNYFVWTHYYSVKKT	DPB1*34:01	P17643	TRP1	185	199	19.84
5809	YNYFVWTHYYSVKKT	DPB1*39:01	P17643	TRP1	185	199	4.79
5810	YNYFVWTHYYSVKKT	DPB1*40:01	P17643	TRP1	185	199	18.64
5811	YNYFVWTHYYSVKKT	DPB1*41:01	P17643	TRP1	185	199	11.48
5812	YNYFVWTHYYSVKKT	DPB1*46:01	P17643	TRP1	185	199	4.64
5813	YNYFVWTHYYSVKKT	DPB1*47:01	P17643	TRP1	185	199	5.48
5814	YNYFVWTHYYSVKKT	DPB1*49:01	P17643	TRP1	185	199	14.45
5815	YNYFVWTHYYSVKKT	DPB1*55:01	P17643	TRP1	185	199	43.38
5816	YNYFVWTHYYSVKKT	DPB1*71:01	P17643	TRP1	185	199	3.63
5817	YNYFVWTHYYSVKKT	DPB1*72:01	P17643	TRP1	185	199	5.37
5818	YNYFVWTHYYSVKKT	DPB1*81:01	P17643	TRP1	185	199	4.64
5819	YNYFVWTHYYSVKKT	DRB1*01:01	P17643	TRP1	185	199	40.38
5820	YNYFVWTHYYSVKKT	DRB1*01:18	P17643	TRP1	185	199	30.68
5821	YNYFVWTHYYSVKKT	DRB1*07:01	P17643	TRP1	185	199	21.34
5822	YNYFVWTHYYSVKKT	DRB1*10:01	P17643	TRP1	185	199	49.68
5823	YNYFVWTHYYSVKKT	DRB1*15:02	P17643	TRP1	185	199	41.6
5824	YNYFVWTHYYSVKKT	DRB1*15:15	P17643	TRP1	185	199	27.93
5825	YNYFVWTHYYSVKKT	DRB1*16:09	P17643	TRP1	185	199	43.18
5826	YRFVIGLRVWQWEVI	DPB1*33:01	O75767	TRP2	210	224	30.93
5827	YRFVIGLRVWQWEVI	DPB1*71:01	O75767	TRP2	210	224	30.93
5828	YRFVIGLRVWQWEVI	DRB1*01:01	O75767	TRP2	210	224	28.72
5829	YRFVIGLRVWQWEVI	DRB1*01:18	O75767	TRP2	210	224	21.41
5830	YRFVIGLRVWQWEVI	DRB1*01:24	O75767	TRP2	210	224	33.28
5831	YRFVIGLRVWQWEVI	DRB1*01:29	O75767	TRP2	210	224	49.96
5832	YRYGSFSVTLDIVQG	DRB1*04:05	P40967	PMEL17	478	492	35.35
5833	YVIKVSARVRFFFPS	DRB1*01:01	P43355	MAGE1	282	296	33.79
5834	YVIKVSARVRFFFPS	DRB1*01:18	P43355	MAGE1	282	296	23.34
5835	YVIKVSARVRFFFPS	DRB1*01:20	P43355	MAGE1	282	296	33.79
5836	YVIKVSARVRFFFPS	DRB1*03:11	P43355	MAGE1	282	296	23.05

5837	YVIKVSARVRFFFPS	DRB1*03:15	P43355	MAGE1	282	296	45.46
5838	YVIKVSARVRFFFPS	DRB1*11:02	P43355	MAGE1	282	296	12.43
5839	YVIKVSARVRFFFPS	DRB1*11:03	P43355	MAGE1	282	296	24.8
5840	YVIKVSARVRFFFPS	DRB1*11:04	P43355	MAGE1	282	296	23.47
5841	YVIKVSARVRFFFPS	DRB1*11:13	P43355	MAGE1	282	296	12.91
5842	YVIKVSARVRFFFPS	DRB1*11:42	P43355	MAGE1	282	296	14.54
5843	YVIKVSARVRFFFPS	DRB1*11:46	P43355	MAGE1	282	296	23.47
5844	YVIKVSARVRFFFPS	DRB1*11:58	P43355	MAGE1	282	296	23.47
5845	YVIKVSARVRFFFPS	DRB1*11:65	P43355	MAGE1	282	296	12.43
5846	YVIKVSARVRFFFPS	DRB1*11:84	P43355	MAGE1	282	296	37.73
5847	YVIKVSARVRFFFPS	DRB1*12:03	P43355	MAGE1	282	296	49.92
5848	YVIKVSARVRFFFPS	DRB1*13:01	P43355	MAGE1	282	296	12.43
5849	YVIKVSARVRFFFPS	DRB1*13:11	P43355	MAGE1	282	296	23.47
5850	YVIKVSARVRFFFPS	DRB1*13:61	P43355	MAGE1	282	296	25.8
5851	YVIKVSARVRFFFPS	DRB1*14:01	P43355	MAGE1	282	296	25.1
5852	YVIKVSARVRFFFPS	DRB1*14:04	P43355	MAGE1	282	296	48.9
5853	YVIKVSARVRFFFPS	DRB1*14:06	P43355	MAGE1	282	296	32.54
5854	YVIKVSARVRFFFPS	DRB1*14:12	P43355	MAGE1	282	296	44.84
5855	YVIKVSARVRFFFPS	DRB1*14:32	P43355	MAGE1	282	296	16.15
5856	YVIKVSARVRFFFPS	DRB1*14:38	P43355	MAGE1	282	296	44.16
5857	YVIKVSARVRFFFPS	DRB1*14:54	P43355	MAGE1	282	296	25.1
5858	YVKVLEHVVRVNARV	DRB1*08:04	P43358	MAGE4	284	298	44.75
5859	YVKVLEHVVRVNARV	DRB1*11:01	P43358	MAGE4	284	298	40.41
5860	YVKVLEHVVRVNARV	DRB1*11:03	P43358	MAGE4	284	298	45.05
5861	YVKVLEHVVRVNARV	DRB1*11:04	P43358	MAGE4	284	298	19.77
5862	YVKVLEHVVRVNARV	DRB1*11:10	P43358	MAGE4	284	298	40.41
5863	YVKVLEHVVRVNARV	DRB1*11:12	P43358	MAGE4	284	298	40.41
5864	YVKVLEHVVRVNARV	DRB1*11:13	P43358	MAGE4	284	298	29.8
5865	YVKVLEHVVRVNARV	DRB1*11:28	P43358	MAGE4	284	298	40.41
5866	YVKVLEHVVRVNARV	DRB1*11:29	P43358	MAGE4	284	298	40.41
5867	YVKVLEHVVRVNARV	DRB1*11:42	P43358	MAGE4	284	298	22.47
5868	YVKVLEHVVRVNARV	DRB1*11:46	P43358	MAGE4	284	298	19.77
5869	YVKVLEHVVRVNARV	DRB1*11:49	P43358	MAGE4	284	298	40.41
5870	YVKVLEHVVRVNARV	DRB1*11:58	P43358	MAGE4	284	298	19.77
5871	YVKVLEHVVRVNARV	DRB1*11:62	P43358	MAGE4	284	298	40.41
5872	YVKVLEHVVRVNARV	DRB1*11:74	P43358	MAGE4	284	298	40.41
5873	YVKVLEHVVRVNARV	DRB1*13:05	P43358	MAGE4	284	298	40.41
5874	YVKVLEHVVRVNARV	DRB1*13:11	P43358	MAGE4	284	298	19.77
5875	YVKVLEHVVRVNARV	DRB1*13:14	P43358	MAGE4	284	298	40.41
5876	YVKVLEHVVRVNARV	DRB1*13:50	P43358	MAGE4	284	298	40.41

5877	YVKVLEYVIKVSARV	DRB1*01:01	P43355	MAGE1	276	290	25.86
5878	YVKVLEYVIKVSARV	DRB1*01:18	P43355	MAGE1	276	290	17.68
5879	YVKVLEYVIKVSARV	DRB1*01:29	P43355	MAGE1	276	290	43.11
5880	YVKVLEYVIKVSARV	DRB1*11:13	P43355	MAGE1	276	290	34.69
5881	YVKVLEYVIKVSARV	DRB1*11:42	P43355	MAGE1	276	290	36.14
5882	YVPLAHSSSAFTITD	DRB1*01:01	P40967	PMEL17	197	211	37.85
5883	YVPLAHSSSAFTITD	DRB1*01:18	P40967	PMEL17	197	211	45.1
5884	YVPLAHSSSAFTITD	DRB1*01:20	P40967	PMEL17	197	211	42.06
5885	YVPLAHSSSAFTITD	DRB1*07:01	P40967	PMEL17	197	211	48.73
5886	YVPLAHSSSAFTITD	DRB1*09:01	P40967	PMEL17	197	211	39.68
5887	YVSMDALLGGSEIWR	DRB1*01:01	P14679	Tyrosinase	182	196	28.17
5888	YVSMDALLGGSEIWR	DRB1*01:18	P14679	Tyrosinase	182	196	32.68
5889	YVSMDALLGGSEIWR	DRB1*10:01	P14679	Tyrosinase	182	196	42.74
5890	YVYMKRKYEAMTKLG	DRB1*11:03	Q16385	SSX2	48	62	27.78
5891	YVYMKRKYEAMTKLG	DRB1*11:04	Q16385	SSX2	48	62	35.29
5892	YVYMKRKYEAMTKLG	DRB1*11:46	Q16385	SSX2	48	62	35.29
5893	YVYMKRKYEAMTKLG	DRB1*11:58	Q16385	SSX2	48	62	35.29
5894	YVYMKRKYEAMTKLG	DRB1*13:11	Q16385	SSX2	48	62	35.29
5895	YVYMKRKYEAMTKLG	DRB1*13:21	Q16385	SSX2	48	62	43.16
5896	YWQVLGGPVSGLSIG	DRB1*01:01	P40967	PMEL17	159	173	16.82
5897	YWQVLGGPVSGLSIG	DRB1*01:18	P40967	PMEL17	159	173	20.35
5898	YYRFVIGLRVWQWEV	DRB1*01:01	O75767	TRP2	209	223	9.39
5899	YYRFVIGLRVWQWEV	DRB1*01:11	O75767	TRP2	209	223	16.56
5900	YYRFVIGLRVWQWEV	DRB1*01:18	O75767	TRP2	209	223	7.14
5901	YYRFVIGLRVWQWEV	DRB1*01:20	O75767	TRP2	209	223	31.31
5902	YYRFVIGLRVWQWEV	DRB1*01:24	O75767	TRP2	209	223	9.55
5903	YYRFVIGLRVWQWEV	DRB1*01:29	O75767	TRP2	209	223	14.91
5904	YYRFVIGLRVWQWEV	DRB1*07:01	O75767	TRP2	209	223	21.28
5905	YYRFVIGLRVWQWEV	DRB1*10:01	O75767	TRP2	209	223	30.56
5906	YYRFVIGLRVWQWEV	DRB1*16:02	O75767	TRP2	209	223	45.76
5907	YYRFVIGLRVWQWEV	DRB1*16:05	O75767	TRP2	209	223	46.5
5908	YYRFVIGLRVWQWEV	DRB1*16:09	O75767	TRP2	209	223	31.95
5909	YYVSMDALLGGSEIW	DRB1*01:01	P14679	Tyrosinase	181	195	9.22
5910	YYVSMDALLGGSEIW	DRB1*01:11	P14679	Tyrosinase	181	195	34.27
5911	YYVSMDALLGGSEIW	DRB1*01:18	P14679	Tyrosinase	181	195	10.14
5912	YYVSMDALLGGSEIW	DRB1*01:20	P14679	Tyrosinase	181	195	34.9
5913	YYVSMDALLGGSEIW	DRB1*01:24	P14679	Tyrosinase	181	195	24.46
5914	YYVSMDALLGGSEIW	DRB1*01:29	P14679	Tyrosinase	181	195	22.18
5915	YYVSMDALLGGSEIW	DRB1*10:01	P14679	Tyrosinase	181	195	12.51
5916	YYYRFVIGLRVWQWE	DRB1*01:01	O75767	TRP2	208	222	7.56

5917	YYYRFVIGLRVWQWE	DRB1*01:11	O75767	TRP2	208	222	11.36
5918	YYYRFVIGLRVWQWE	DRB1*01:18	O75767	TRP2	208	222	5.78
5919	YYYRFVIGLRVWQWE	DRB1*01:20	O75767	TRP2	208	222	24.68
5920	YYYRFVIGLRVWQWE	DRB1*01:24	O75767	TRP2	208	222	6.88
5921	YYYRFVIGLRVWQWE	DRB1*01:29	O75767	TRP2	208	222	10.94
5922	YYYRFVIGLRVWQWE	DRB1*07:01	O75767	TRP2	208	222	15.65
5923	YYYRFVIGLRVWQWE	DRB1*10:01	O75767	TRP2	208	222	21.78
5924	YYYRFVIGLRVWQWE	DRB1*11:01	O75767	TRP2	208	222	49.95
5925	YYYRFVIGLRVWQWE	DRB1*11:10	O75767	TRP2	208	222	49.95
5926	YYYRFVIGLRVWQWE	DRB1*11:12	O75767	TRP2	208	222	49.95
5927	YYYRFVIGLRVWQWE	DRB1*11:28	O75767	TRP2	208	222	49.95
5928	YYYRFVIGLRVWQWE	DRB1*11:29	O75767	TRP2	208	222	49.95
5929	YYYRFVIGLRVWQWE	DRB1*11:49	O75767	TRP2	208	222	49.95
5930	YYYRFVIGLRVWQWE	DRB1*11:62	O75767	TRP2	208	222	49.95
5931	YYYRFVIGLRVWQWE	DRB1*11:74	O75767	TRP2	208	222	49.95
5932	YYYRFVIGLRVWQWE	DRB1*13:05	O75767	TRP2	208	222	49.95
5933	YYYRFVIGLRVWQWE	DRB1*13:14	O75767	TRP2	208	222	49.95
5934	YYYRFVIGLRVWQWE	DRB1*13:50	O75767	TRP2	208	222	49.95
5935	YYYRFVIGLRVWQWE	DRB1*15:02	O75767	TRP2	208	222	30.94
5936	YYYRFVIGLRVWQWE	DRB1*15:15	O75767	TRP2	208	222	41.32
5937	YYYRFVIGLRVWQWE	DRB1*16:01	O75767	TRP2	208	222	36.45
5938	YYYRFVIGLRVWQWE	DRB1*16:02	O75767	TRP2	208	222	28.76
5939	YYYRFVIGLRVWQWE	DRB1*16:04	O75767	TRP2	208	222	43.5
5940	YYYRFVIGLRVWQWE	DRB1*16:05	O75767	TRP2	208	222	28.04
5941	YYYRFVIGLRVWQWE	DRB1*16:09	O75767	TRP2	208	222	22.89

Table S6. Frequency of occurrence of the 679 unique 15-AA peptides with strong PBBAs. Both of the two strongest binders (N = 63) were from antigen MAGE1.

Index	Peptide	Frequency
1	LEYVIKVSARVRFFF	63
2	VLEYVIKVSARVRFF	63
3	KVLEYVIKVSARVRF	57
4	EYVIKVSARVRFFFP	55
5	RAVFLALSAQLLQAR	47
6	ARYEFLWGPRALAET	46
7	PARYEFLWGPRALAE	46
8	VKVLEYVIKVSARVR	40
9	HPLFVIATRRSEEIL	38
10	ILESLFRAVITKKVA	38
11	RYEFLWGPRALAETS	38
12	THPLFVIATRRSEEI	36
13	KVLEHVVRVNARVRI	35
14	ARVRFFFPSLREAAL	34
15	ESLFRAVITKKVADL	34
16	KFFAYLTLAKHTISS	34
17	SLFRAVITKKVADLV	34
18	NASFSIALNFPGSQK	33
19	ASFSIALNFPGSQKV	32
20	EKIFYVYMKRKYEAM	32
21	KDKFFAYLTLAKHTI	32
22	LESLFRAVITKKVAD	32
23	KIFYVYMKRKYEAMT	31
24	TTHPLFVIATRRSEE	31
25	IYNYFVWTHYYSVKK	30
26	LAHFLLRKYRAKELV	30
27	YNYFVWTHYYSVKKT	30
28	ADLVGFLLLKYRARE	29
29	AHFLLRKYRAKELVT	29
30	DGTATLRLVKRQVPL	29
31	DKFFAYLTLAKHTIS	29
32	DLVGFLLLKYRAREP	29
33	IFYVYMKRKYEAMTK	29
34	RVRFFFPSLREAALR	29
35	CILESLFRAVITKKV	28
36	GTATLRLVKRQVPLD	28
37	PLFVIATRRSEEILG	28

38	SEKIFYVYMKRKYEA	28
39	ANASFSIALNFPGSQ	27
40	ELAHFLLRKYRAKEL	27
41	LVGFLLLKYRAREPV	27
42	NKHFLRNQPLTFALQ	27
43	NYFVWTHYYSVKKTF	27
44	SRLLEFYLAMPFATP	27
45	VGFLLLKYRAREPVT	27
46	FPLLLFQQARAQFPR	26
47	LEHVVRVNARVRIAY	26
48	RTTHPLFVIATRRSE	26
49	SARVRFFFPSLREAA	26
50	TATLRLVKRQVPLDC	26
51	YFVWTHYYSVKKTFL	26
52	YYYRFVIGLRVWQWE	26
53	ARAVFLALSAQLLQA	25
54	FFAYLTLAKHTISSD	25
55	VKVLEHVVRVNARVR	25
56	VLEHVVRVNARVRIA	25
57	VRFFFPSLREAALRE	25
58	YVIKVSARVRFFFPS	25
59	GFLLLKYRAREPVTK	24
60	SIYNYFVWTHYYSVK	24
61	AARAVFLALSAQLLQ	23
62	ATLRLVKRQVPLDCV	23
63	EVISCKLIKRATTRQ	23
64	FAYLTLAKHTISSDY	23
65	GNKHFLRNQPLTFAL	23
66	VALIFGTASYLIRAR	23
67	WEVISCKLIKRATTR	23
68	FYVYMKRKYEAMTKL	22
69	ISIYNYFVWTHYYSV	22
70	VTAQVVLQAAIPLTS	22
71	EHVVRVNARVRIAYP	21
72	FVWTHYYSVKKTFLG	21
73	QRVLIVRRNLLDLSK	21
74	VISCKLIKRATTRQP	21
75	AVFLALSAQLLQARL	20
76	ESLFREALSNKVDEL	20
77	KHFLRNQPLTFALQL	20

78	KRSFVYVWKTWGQYW	20
79	QKRSFVYVWKTWGQY	20
80	SYVKVLEHVVRVNAR	20
81	DQRVLIVRRNLLDLS	19
82	FFPLLLFQQARAQFP	19
83	HVVRVNARVRIAYPS	19
84	LKEFTVSGNILTIRL	19
85	PLLLFQQARAQFPRQ	19
86	TASYLIRARRSMDEA	19
87	VADLVGFLLLKYRAR	19
88	VDELAHFLLRKYRAK	19
89	YVKVLEHVVRVNARV	19
90	ASEKIFYVYMKRKYE	18
91	ASYLIRARRSMDEAN	18
92	CDQRVLIVRRNLLDL	18
93	CQLVLHQILKGGSGT	18
94	DELAHFLLRKYRAKE	18
95	GPAFLTWHRYHLLRL	18
96	HFLLRKYRAKELVTK	18
97	KVYYYRFVIGLRVWQ	18
98	LFVIATRRSEEILGP	18
99	LLKEFTVSGNILTIR	18
100	NRESYMVPFIPLYRN	18
101	QLVLHQILKGGSGTY	18
102	RLLEFYLAMPFATPM	18
103	RSYVPLAHSSSAFTI	18
104	SQKRSFVYVWKTWGQ	18
105	AVVLASLIYRRRLMK	17
106	DFFVWLHYYSVRDTL	17
107	MAARAVFLALSAQLL	17
108	PVTAQVVLQAAIPLT	17
109	RFFFPSLREAALREE	17
110	VFLALSAQLLQARLM	17
111	ALIFGTASYLIRARR	16
112	KEFTVSGNILTIRLT	16
113	LFRAVITKKVADLVG	16
114	TAQVVLQAAIPLTSC	16
115	VSIVVLSGTTAAQVT	16
116	VVLASLIYRRRLMKQ	16
117	WLKVYYYRFVIGLRV	16

118	AACDQRVLIVRRNLL	15
119	ACDQRVLIVRRNLLD	15
120	AFLTWHRYHLLRLEK	15
121	EKDKFFAYLTLAKHT	15
122	MAVVLASLIYRRRLM	15
123	PAFLTWHRYHLLRLE	15
124	VIKVSARVRFFFPSL	15
125	VYDFFVWLHYYSVRD	15
126	VYYYRFVIGLRVWQW	15
127	YDFFVWLHYYSVRDT	15
128	DHRQLQLSISSCLQQ	14
129	EVSIVVLSGTTAAQV	14
130	GANASFSIALNFPGS	14
131	GSRSYVPLAHSSSAF	14
132	HRQLQLSISSCLQQL	14
133	LKVYYYRFVIGLRVW	14
134	LMAVVLASLIYRRRL	14
135	LMWITQCFLPVFLAQ	14
136	LSLLMWITQCFLPVF	14
137	MWITQCFLPVFLAQP	14
138	QLSLLMWITQCFLPV	14
139	RVLIVRRNLLDLSKE	14
140	SRSYVPLAHSSSAFT	14
141	SVYDFFVWLHYYSVR	14
142	VVRVNARVRIAYPSL	14
143	VWTHYYSVKKTFLGV	14
144	AVIGALLAVGATKVP	13
145	GGNKHFLRNQPLTFA	13
146	GPVTAQVVLQAAIPL	13
147	HHAFVDSIFEQWLRR	13
148	HNRESYMVPFIPLYR	13
149	IGALLAVGATKVPRN	13
150	LIFGTASYLIRARRS	13
151	LLEFYLAMPFATPME	13
152	LLMWITQCFLPVFLA	13
153	MVPFIPLYRNGDFFI	13
154	SFSIALNFPGSQKVL	13
155	SLFREALSNKVDELA	13
156	THYYSVKKTFLGVGQ	13
157	TSCILESLFRAVITK	13

158	VIGALLAVGATKVPR	13
159	VPFIPLYRNGDFFIS	13
160	WMHYYVSMDALLGGS	13
161	WTHYYSVKKTFLGVG	13
162	ALLAGLVSLLCRHKR	12
163	AQVVLQAAIPLTSCG	12
164	DPIFLLHHAFVDSIF	12
165	EGPAFLTWHRYHLLR	12
166	FLLHHAFVDSIFEQW	12
167	HAFVDSIFEQWLRRH	12
168	KRKYEAMTKLGFKAT	12
169	MKRKYEAMTKLGFKA	12
170	PIFLLHHAFVDSIFE	12
171	VLASLIYRRRLMKQD	12
172	VLLKEFTVSGNILTI	12
173	WSQKRSFVYVWKTWG	12
174	YMVPFIPLYRNGDFF	12
175	AESLFREALSNKVDE	11
176	AGLVSLLCRHKRKQL	11
177	CSVYDFFVWLHYYSV	11
178	DPARYEFLWGPRALA	11
179	ESRLLEFYLAMPFAT	11
180	FFFPSLREAALREEE	11
181	FVWMHYYVSMDALLG	11
182	HNALHIYMNGTMSQV	11
183	IFFPLLLFQQARAQF	11
184	IFGTASYLIRARRSM	11
185	LDGTATLRLVKRQVP	11
186	LLAGLVSLLCRHKRK	11
187	LVALIFGTASYLIRA	11
188	MHYYVSMDALLGGSE	11
189	NALHIYMNGTMSQVQ	11
190	NDPIFLLHHAFVDSI	11
191	NPARYEFLWGPRALA	11
192	RKFFHRTCKCTGNFA	11
193	RSFVYVWKTWGQYWQ	11
194	SESLKMIFGIDVKEV	11
195	VWMHYYVSMDALLGG	11
196	YYRFVIGLRVWQWEV	11
197	ALHIYMNGTMSQVQG	10

198	DAESLFREALSNKVD	10
199	EFYLAMPFATPMEAE	10
200	FFVWLHYYSVRDTLL	10
201	FLLLKYRAREPVTKA	10
202	KLGFKATLPPFMCNK	10
203	LEFYLAMPFATPMEA	10
204	LFREALSNKVDELAH	10
205	PDAESLFREALSNKV	10
206	SCILESLFRAVITKK	10
207	SIVVLSGTTAAQVTT	10
208	AEMLERVIKNYKRCF	9
209	AEVSIVVLSGTTAAQ	9
210	ASESLKMIFGIDVKE	9
211	ASLIYRRRLMKQDFS	9
212	FLLRKYRAKELVTKA	9
213	FMAFVAMVTTACHEF	9
214	GLVSLLCRHKRKQLP	9
215	GPTLIGANASFSIAL	9
216	HYYVSMDALLGGSEI	9
217	KRTTHPLFVIATRRS	9
218	LAGLVSLLCRHKRKQ	9
219	LASLIYRRRLMKQDF	9
220	LAVIGALLAVGATKV	9
221	LVSLLCRHKRKQLPE	9
222	MAFVAMVTTACHEFF	9
223	NARVRIAYPSLREAA	9
224	PEKDKFFAYLTLAKH	9
225	PTLIGANASFSIALN	9
226	RESYMVPFIPLYRNG	9
227	TKLGFKATLPPFMCN	9
228	TLRLVKRQVPLDCVL	9
229	WEKMKASEKIFYVYM	9
230	WGQYWQVLGGPVSGL	9
231	ALLAVGATKVPRNQD	8
232	ANDPIFLLHHAFVDS	8
233	ARVRIAYPSLREAAL	8
234	DGGNKHFLRNQPLTF	8
235	DPIFVLLHTFTDAVF	8
236	EFMAFVAMVTTACHE	8
237	FLALSAQLLQARLMK	8

238	FLTWHRYHLLRLEKD	8
239	GALLAVGATKVPRNQ	8
240	HFLRNQPLTFALQLH	8
241	HLAVIGALLAVGATK	8
242	IFLLHHAFVDSIFEQ	8
243	KHTISSDYVIPIGTY	8
244	KKVADLVGFLLLKYR	8
245	LGFKATLPPFMCNKR	8
246	MVGAVLTALLAGLVS	8
247	NDPIFVLLHTFTDAV	8
248	PESRLLEFYLAMPFA	8
249	PIFVLLHTFTDAVFD	8
250	QVVLQAAIPLTSCGS	8
251	TKKVADLVGFLLLKY	8
252	TSYVKVLEHVVRVNA	8
253	VGAVLTALLAGLVSL	8
254	VLMAVVLASLIYRRR	8
255	ACQLVLHQILKGGSG	7
256	AFVDSIFEQWLRRHR	7
257	ASRIWSWLLGAAMVG	7
258	CQNILLSNAPLGPQF	7
259	DCVLYRYGSFSVTLD	7
260	DGPTLIGANASFSIA	7
261	DPAVRSLHNLAHLFL	7
262	ELKELINNELSHFLE	7
263	ESLKMIFGIDVKEVD	7
264	FGTASYLIRARRSMD	7
265	GAVLTALLAGLVSLL	7
266	GPSTSCILESLFRAV	7
267	GQYWQVLGGPVSGLS	7
268	GTASYLIRARRSMDE	7
269	HEGPAFLTWHRYHLL	7
270	IGANASFSIALNFPG	7
271	IKSYLEQASRIWSWL	7
272	ILTIRLTAADHRQLQ	7
273	KASESLKMIFGIDVK	7
274	KSYLEQASRIWSWLL	7
275	LHHAFVDSIFEQWLR	7
276	LLLFQQARAQFPRQC	7
277	LLVALIFGTASYLIR	7

278	MTKLGFKATLPPFMC	7
279	NILLSNAPLGPQFPF	7
280	NILTIRLTAADHRQL	7
281	PAVRSLHNLAHLFLN	7
282	PSTSCILESLFRAVI	7
283	QASRIWSWLLGAAMV	7
284	QNILLSNAPLGPQFP	7
285	QYWQVLGGPVSGLSI	7
286	RIWSWLLGAAMVGAV	7
287	RVNARVRIAYPSLRE	7
288	SCQNILLSNAPLGPQ	7
289	SDPARYEFLWGPRAL	7
290	SELKELINNELSHFL	7
291	SGNILTIRLTAADHR	7
292	SNPARYEFLWGPRAL	7
293	SRIWSWLLGAAMVGA	7
294	STSCILESLFRAVIT	7
295	TWGQYWQVLGGPVSG	7
296	VLYRYGSFSVTLDIV	7
297	VNARVRIAYPSLREA	7
298	YYVSMDALLGGSEIW	7
299	AVLTALLAGLVSLLC	6
300	DWLGVSRQLRTKAWN	6
301	FLGALDLAKKRVHPD	6
302	FVRALDMAKRTTHPL	6
303	FVWLHYYSVRDTLLG	6
304	IVLVMIAMEGGHAPE	6
305	KTWGQYWQVLGGPVS	6
306	KVDELAHFLLRKYRA	6
307	LGALDLAKKRVHPDY	6
308	LGVSRQLRTKAWNRQ	6
309	LHIYMNGTMSQVQGS	6
310	LLHHAFVDSIFEQWL	6
311	MHNALHIYMNGTMSQ	6
312	MTFGRLQGISPKIMP	6
313	PAEVSIVVLSGTTAA	6
314	QDWLGVSRQLRTKAW	6
315	QEFMAFVAMVTTACH	6
316	QFLGALDLAKKRVHP	6
317	QMTFGRLQGISPKIM	6

318	QWEVISCKLIKRATT	6
319	RQLQLSISSCLQQLS	6
320	SLKMIFGIDVKEVDP	6
321	SLLMWITQCFLPVFL	6
322	SYLIRARRSMDEANQ	6
323	SYVPLAHSSSAFTIT	6
324	TALLAGLVSLLCRHK	6
325	TLIGANASFSIALNF	6
326	VRALDMAKRTTHPLF	6
327	VRVNARVRIAYPSLR	6
328	VSARVRFFFPSLREA	6
329	WLGVSRQLRTKAWNR	6
330	YRFVIGLRVWQWEVI	6
331	YVYMKRKYEAMTKLG	6
332	ADHRQLQLSISSCLQ	5
333	AMTKLGFKATLPPFM	5
334	AMVGAVLTALLAGLV	5
335	CVLYRYGSFSVTLDI	5
336	DGQVIWVNNTIINGS	5
337	DTPPFYSNSTNSFRN	5
338	EKNHFVRALDMAKRT	5
339	EMLERVIKNYKRCFP	5
340	ERVIKNYKRCFPVIF	5
341	ESYMVPFIPLYRNGD	5
342	GQTHLSPNDPIFVLL	5
343	GQVIWVNNTIINGSQ	5
344	IIVLVMIAMEGGHAP	5
345	IKVSARVRFFFPSLR	5
346	KNHFVRALDMAKRTT	5
347	KRCLLHLAVIGALLA	5
348	KVADLVGFLLLKYRA	5
349	LALSAQLLQARLMKE	5
350	LERVIKNYKRCFPVI	5
351	LHLAVIGALLAVGAT	5
352	LKRCLLHLAVIGALL	5
353	LPDGQVIWVNNTIIN	5
354	LVLHQILKGGSGTYC	5
355	MLERVIKNYKRCFPV	5
356	NQIMPKTGFLIIVLV	5
357	PACQLVLHQILKGGS	5

358	PDGQVIWVNNTIING	5
359	PLDCVLYRYGSFSVT	5
360	PNDPIFVLLHTFTDA	5
361	PQMTFGRLQGISPKI	5
362	QTHLSPNDPIFVLLH	5
363	QVIWVNNTIINGSQV	5
364	SESLQLVFGIDVKEA	5
365	SGTLISRALVVTHTY	5
366	TKAEMLERVIKNYKR	5
367	TPPFYSNSTNSFRNT	5
368	VIWVNNTIINGSQVW	5
369	VLKRCLLHLAVIGAL	5
370	VLTALLAGLVSLLCR	5
371	VLVMIAMEGGHAPEE	5
372	YVKVLEYVIKVSARV	5
373	YVPLAHSSSAFTITD	5
374	AFVAMVTTACHEFFE	4
375	AKHTISSDYVIPIGT	4
376	ALGLVCVQAATSSSS	4
377	ALSAQLLQARLMKEE	4
378	DFQEFMAFVAMVTTA	4
379	DGPIRRNPAGNVARP	4
380	DSSGTLISRALVVTH	4
381	EALGLVCVQAATSSS	4
382	EDGPIRRNPAGNVAR	4
383	EKAMVALIDVFHQYS	4
384	EKMKASEKIFYVYMK	4
385	EQASRIWSWLLGAAM	4
386	ETSYVKVLEHVVRVN	4
387	EVTVYHRRGSRSYVP	4
388	EWEKMKASEKIFYVY	4
389	FDTPPFYSNSTNSFR	4
390	FLPVFLAQPPSGQRR	4
391	FSIALNFPGSQKVLP	4
392	GDSSGTLISRALVVT	4
393	GFKATLPPFMCNKRA	4
394	GPESRLLEFYLAMPF	4
395	GPIRRNPAGNVARPM	4
396	GPRALAETSYVKVLE	4
397	GTLISRALVVTHTYL	4

398	IFVLLHTFTDAVFDE	4
399	IVVLSGTTAAQVTTT	4
400	IWSWLLGAAMVGAVL	4
401	KAMVALIDVFHQYSG	4
402	KKPPVIRQNIHSLSP	4
403	KPPVIRQNIHSLSPQ	4
404	LDCVLYRYGSFSVTL	4
405	LEKAMVALIDVFHQY	4
406	LGLVCVQAATSSSSP	4
407	LIGANASFSIALNFP	4
408	LKELINNELSHFLEE	4
409	LLHLAVIGALLAVGA	4
410	LTALLAGLVSLLCRH	4
411	LTIRLTAADHRQLQL	4
412	LWGPRALAETSYVKV	4
413	LYRYGSFSVTLDIVQ	4
414	MEVTVYHRRGSRSYV	4
415	NDGPTLIGANASFSI	4
416	PFYSNSTNSFRNTVE	4
417	PIRRNPAGNVARPMV	4
418	PPFYSNSTNSFRNTV	4
419	PRALAETSYVKVLEY	4
420	QEALGLVCVQAATSS	4
421	RALAETSYVKVLEYV	4
422	RDTLLGGFFPWLKVY	4
423	RKKPPVIRQNIHSLS	4
424	RNGYRALMDKSLHVG	4
425	RRNGYRALMDKSLHV	4
426	SLIYRRRLMKQDFSV	4
427	SSGTLISRALVVTHT	4
428	TEDGPIRRNPAGNVA	4
429	TFGRLQGISPKIMPK	4
430	THLSPNDPIFVLLHT	4
431	TMEVTVYHRRGSRSY	4
432	VLHQILKGGSGTYCL	4
433	VVLQAAIPLTSCGSS	4
434	WGPRALAETSYVKVL	4
435	YDLFVWMHYYVSMDA	4
436	YDPAVRSLHNLAHLF	4
437	YEFLWGPRALAETSY	4

438	ASESLQLVFGIDVKE	3
439	AVLYCLLWSFQTSAG	3
440	CFLPVFLAQPPSGQR	3
441	CFPVIFGKASESLKM	3
442	CIFFPLLLFQQARAQ	3
443	DNQIMPKTGFLIIVL	3
444	DYIKSYLEQASRIWS	3
445	ESLQLVFGIDVKEAD	3
446	FGRLQGISPKIMPKK	3
447	FPVIFGKASESLKMI	3
448	FQEFMAFVAMVTTAC	3
449	FRAVITKKVADLVGF	3
450	FSLPYWNFATGKNVC	3
451	FYLAMPFATPMEAEL	3
452	GDNQIMPKTGFLIIV	3
453	GNILTIRLTAADHRQ	3
454	GNNQIFPKTGLLIIV	3
455	GSCQNILLSNAPLGP	3
456	GYRALMDKSLHVGTQ	3
457	IDFAHEAPAFLPWHR	3
458	ITKKVADLVGFLLLK	3
459	KASESLQLVFGIDVK	3
460	KKSELKELINNELSH	3
461	KSELKELINNELSHF	3
462	LIIVLVMIAMEGGHA	3
463	LLFQQARAQFPRQCA	3
464	LLRKYRAKELVTKAE	3
465	LLWSFQTSAGHFPRA	3
466	LPYWNFATGKNVCDI	3
467	LSAQLLQARLMKEES	3
468	LTWHRYHLLRLEKDM	3
469	LVLKRCLLHLAVIGA	3
470	LVMIAMEGGHAPEEE	3
471	NGYRALMDKSLHVGT	3
472	NNQIFPKTGLLIIVL	3
473	NQIFPKTGLLIIVLG	3
474	PVSGLSIGTGRAMLG	3
475	RPQMTFGRLQGISPK	3
476	SAQLLQARLMKEESP	3
477	SYVKVLEYVIKVSAR	3

478	TEMFVTAPDNLGYTY	3
479	TPAEVSIVVLSGTTA	3
480	TTQHWVGLLGPNGTQ	3
481	VSGLSIGTGRAMLGT	3
482	YIKSYLEQASRIWSW	3
483	YVSMDALLGGSEIWR	3
484	AETSYVKVLEHVVRV	2
485	ALAETSYVKVLEYVI	2
486	APEKDKFFAYLTLAK	2
487	APPAYEKLSAEQSPP	2
488	AVITKKVADLVGFLL	2
489	AVRSLHNLAHLFLNG	2
490	DIDFAHEAPAFLPWH	2
491	DLFVWMHYYVSMDAL	2
492	DSDPARYEFLWGPRA	2
493	DTLLGGFFPWLKVYY	2
494	EAPAFLPWHRLFLLR	2
495	EEKNHFVRALDMAKR	2
496	EEWEKMKASEKIFYV	2
497	EFTVSGNILTIRLTA	2
498	EMFVTAPDNLGYTYE	2
499	EREQFLGALDLAKKR	2
500	EVDFSHEGPAFLTWH	2
501	FFPWLKVYYYRFVIG	2
502	FREALSNKVDELAHF	2
503	FVIGLRVWQWEVISC	2
504	GGFFPWLKVYYYRFV	2
505	GHNRESYMVPFIPLY	2
506	GKASESLKMIFGIDV	2
507	GLRVWQWEVISCKLI	2
508	GLVCVQAATSSSSPL	2
509	GPLLDGTATLRLVKR	2
510	GPVSGLSIGTGRAML	2
511	GSNPARYEFLWGPRA	2
512	GVLLKEFTVSGNILT	2
513	IGHNRESYMVPFIPL	2
514	IGLRVWQWEVISCKL	2
515	IKNYKRCFPVIFGKA	2
516	INIYDLFVWMHYYVS	2
517	ITTQHWVGLLGPNGT	2

518	IYDLFVWMHYYVSMD	2
519	KASEKIFYVYMKRKY	2
520	KLLSLGCIFFPLLLF	2
521	KNYKRCFPVIFGKAS	2
522	KVSARVRFFFPSLRE	2
523	LAVLYCLLWSFQTSA	2
524	LFVWMHYYVSMDALL	2
525	LGGFFPWLKVYYYRF	2
526	LGNNQIFPKTGLLII	2
527	LHYYSVRDTLLGGFF	2
528	LLLVALIFGTASYLI	2
529	LSAPEKDKFFAYLTL	2
530	LSLGCIFFPLLLFQQ	2
531	LYCLLWSFQTSAGHF	2
532	MFVTAPDNLGYTYEI	2
533	NIYDLFVWMHYYVSM	2
534	NLLSPASFFSSWQIV	2
535	NQDWLGVSRQLRTKA	2
536	NTEMFVTAPDNLGYT	2
537	PRALAETSYVKVLEH	2
538	PWLKVYYYRFVIGLR	2
539	PYWNFATGKNVCDIC	2
540	QDLVQEKYLEYRQVP	2
541	QDYIKSYLEQASRIW	2
542	QHWVGLLGPNGTQPQ	2
543	QIMPKTGFLIIVLVM	2
544	RALAETSYVKVLEHV	2
545	RCLLHLAVIGALLAV	2
546	RDIDFAHEAPAFLPW	2
547	RFVIGLRVWQWEVIS	2
548	RGPESRLLEFYLAMP	2
549	RLRERKQLVIYEEIS	2
550	RRRNGYRALMDKSLH	2
551	SAPEKDKFFAYLTLA	2
552	SGLSIGTGRAMLGTH	2
553	SLGCIFFPLLLFQQA	2
554	SLPYWNFATGKNVCD	2
555	SMHNALHIYMNGTMS	2
556	SPASFFSSWQIVCSR	2
557	SPNDPIFVLLHTFTD	2

558	SVRDTLLGGFFPWLK	2
559	SVSVSQLRALDGGNK	2
560	SWLLGAAMVGAVLTA	2
561	SYMVPFIPLYRNGDF	2
562	TLLGGFFPWLKVYYY	2
563	TNTEMFVTAPDNLGY	2
564	TQDLVQEKYLEYRQV	2
565	TQDWVQENYLEYRQV	2
566	TQHWVGLLGPNGTQP	2
567	TWHRYHLLRLEKDMQ	2
568	VIGLRVWQWEVISCK	2
569	VIKNYKRCFPVIFGK	2
570	VITKKVADLVGFLLL	2
571	VLYCLLWSFQTSAGH	2
572	VMIAMEGGHAPEEEI	2
573	VPLDCVLYRYGSFSV	2
574	VRDTLLGGFFPWLKV	2
575	VRSLHNLAHLFLNGT	2
576	VSGNILTIRLTAADH	2
577	VWLHYYSVRDTLLGG	2
578	WHRLFLLRWEQEIQK	2
579	WITQCFLPVFLAQPP	2
580	WLHYYSVRDTLLGGF	2
581	WSWLLGAAMVGAVLT	2
582	YWQVLGGPVSGLSIG	2
583	AADHRQLQLSISSCL	1
584	AEMLESVIKNYKHCF	1
585	AETSYVKVLEYVIKV	1
586	AFTITDQVPFSVSVS	1
587	AHLFLNGTGGQTHLS	1
588	ARLMKEESPVVSWRL	1
589	ASFFSSWQIVCSRLE	1
590	ATPMEAELARRSLAQ	1
591	CDFQEFMAFVAMVTT	1
592	CLLHLAVIGALLAVG	1
593	CLLWSFQTSAGHFPR	1
594	CQPVLPSPACQLVLH	1
595	CTEVRADTRPWSGPY	1
596	DMQEMLQEPSFSLPY	1
597	DQVPFSVSVSQLRAL	1

598	ECDFQEFMAFVAMVT	1
599	ELEKAMVALIDVFHQ	1
600	ETSYVKVLEYVIKVS	1
601	EVRADTRPWSGPYIL	1
602	EVWPLRFFNRTCHCN	1
603	FATPMEAELARRSLA	1
604	FEQWLRRHRPLQEVY	1
605	FGDSSGTLISRALVV	1
606	FQDYIKSYLEQASRI	1
607	FSVSVSQLRALDGGN	1
608	FSVTLDIVQGIESAE	1
609	GEVDFSHEGPAFLTW	1
610	GLSIGTGRAMLGTHT	1
611	GQCTEVRADTRPWSG	1
612	GRAMLGTHTMEVTVY	1
613	GRLQGISPKIMPKKP	1
614	GSFSVTLDIVQGIES	1
615	HFVRALDMAKRTTHP	1
616	HTISSDYVIPIGTYG	1
617	HTMEVTVYHRRGSRS	1
618	IFEQWLRRHRPLQEV	1
619	KAEMLERVIKNYKRC	1
620	LAETSYVKVLEHVVR	1
621	LAETSYVKVLEYVIK	1
622	LAHLFLNGTGGQTHL	1
623	LAKHTISSDYVIPIG	1
624	LGDNQIMPKTGFLII	1
625	LGPLLDGTATLRLVK	1
626	LGYTYEIQWPSREFS	1
627	LKKSELKELINNELS	1
628	LLLKYRAREPVTKAE	1
629	MQEMLQEPSFSLPYW	1
630	NAPPAYEKLSAEQSP	1
631	NHFVRALDMAKRTTH	1
632	PFIPLYRNGDFFISS	1
633	PFSVSVSQLRALDGG	1
634	PLLDGTATLRLVKRQ	1
635	PVIFGKASESLKMIF	1
636	QARLMKEESPVVSWR	1
637	QCFLPVFLAQPPSGQ	1

638	QCTEVRADTRPWSGP	1
639	QEREQFLGALDLAKK	1
640	QPVLPSPACQLVLHQ	1
641	QVTTTEWVETTAREL	1
642	RAMLGTHTMEVTVYH	1
643	RCFPVIFGKASESLK	1
644	REQFLGALDLAKKRV	1
645	RGQCTEVRADTRPWS	1
646	RGSCQNILLSNAPLG	1
647	RGSRSYVPLAHSSSA	1
648	RKYEAMTKLGFKATL	1
649	RSNFDSTLISPNSVF	1
650	RVIKNYKRCFPVIFG	1
651	RVRIAYPSLREAALL	1
652	SFSLPYWNFATGKNV	1
653	SFSVTLDIVQGIESA	1
654	SIFEQWLRRHRPLQE	1
655	SLQLVFGIDVKEADP	1
656	SRSNFDSTLISPNSV	1
657	SVSQLRALDGGNKHF	1
658	TERRLLVRRNIFDLS	1
659	TEVRADTRPWSGPYI	1
660	TGRAMLGTHTMEVTV	1
661	THTMEVTVYHRRGSR	1
662	TIRLTAADHRQLQLS	1
663	TLISRALVVTHTYLE	1
664	TSYVKVLEYVIKVSA	1
665	TTEWVETTARELPIP	1
666	TTTEWVETTARELPI	1
667	VDFSHEGPAFLTWHR	1
668	VSLLCRHKRKQLPEE	1
669	VSVSQLRALDGGNKH	1
670	VTKAEMLERVIKNYK	1
671	VTVYHRRGSRSYVPL	1
672	VWPLRFFNRTCHCNG	1
673	WHRYHLLRLEKDMQE	1
674	WQWEVISCKLIKRAT	1
675	WRDIDFAHEAPAFLP	1
676	YCLLWSFQTSAGHFP	1
677	YLIRARRSMDEANQP	1

678	YMKRKYEAMTKLGFK	1
679	YRYGSFSVTLDIVQG	1

Table S7. Number of strong binders (PBBA IC_{50} < 50 nM) per antigen and allele. Data are shown in ascending order for protein and allele.

Protein	Antigen	Allele	N strong
O75767	TRP2	DPB1*01:01	1
O75767	TRP2	DPB1*02:01	6
O75767	TRP2	DPB1*02:02	7
O75767	TRP2	DPB1*04:01	5
O75767	TRP2	DPB1*126:01	5
O75767	TRP2	DPB1*15:01	4
O75767	TRP2	DPB1*23:01	5
O75767	TRP2	DPB1*33:01	24
O75767	TRP2	DPB1*39:01	5
O75767	TRP2	DPB1*46:01	6
O75767	TRP2	DPB1*47:01	7
O75767	TRP2	DPB1*71:01	24
O75767	TRP2	DPB1*72:01	4
O75767	TRP2	DPB1*81:01	6
O75767	TRP2	DRB1*01:01	16
O75767	TRP2	DRB1*01:11	6
O75767	TRP2	DRB1*01:18	23
O75767	TRP2	DRB1*01:20	7
O75767	TRP2	DRB1*01:24	7
O75767	TRP2	DRB1*01:29	7
O75767	TRP2	DRB1*03:11	6
O75767	TRP2	DRB1*07:01	6
O75767	TRP2	DRB1*08:04	3
O75767	TRP2	DRB1*10:01	7
O75767	TRP2	DRB1*11:01	5
O75767	TRP2	DRB1*11:02	3
O75767	TRP2	DRB1*11:03	7
O75767	TRP2	DRB1*11:04	7
O75767	TRP2	DRB1*11:08	1
O75767	TRP2	DRB1*11:10	5
O75767	TRP2	DRB1*11:12	5
O75767	TRP2	DRB1*11:13	2
O75767	TRP2	DRB1*11:14	3
O75767	TRP2	DRB1*11:28	5
O75767	TRP2	DRB1*11:29	5
O75767	TRP2	DRB1*11:42	8

O75767	TRP2	DRB1*11:46	7
O75767	TRP2	DRB1*11:49	5
O75767	TRP2	DRB1*11:58	7
O75767	TRP2	DRB1*11:62	5
O75767	TRP2	DRB1*11:65	3
O75767	TRP2	DRB1*11:74	5
O75767	TRP2	DRB1*11:84	1
O75767	TRP2	DRB1*13:01	3
O75767	TRP2	DRB1*13:02	3
O75767	TRP2	DRB1*13:05	5
O75767	TRP2	DRB1*13:11	7
O75767	TRP2	DRB1*13:14	5
O75767	TRP2	DRB1*13:23	3
O75767	TRP2	DRB1*13:50	5
O75767	TRP2	DRB1*13:97	3
O75767	TRP2	DRB1*14:06	1
O75767	TRP2	DRB1*15:01	3
O75767	TRP2	DRB1*15:02	5
O75767	TRP2	DRB1*15:06	3
O75767	TRP2	DRB1*15:15	6
O75767	TRP2	DRB1*16:01	5
O75767	TRP2	DRB1*16:02	6
O75767	TRP2	DRB1*16:04	3
O75767	TRP2	DRB1*16:05	6
O75767	TRP2	DRB1*16:09	6
P04271	S100	DRB1*01:01	9
P04271	S100	DRB1*01:11	4
P04271	S100	DRB1*01:18	13
P04271	S100	DRB1*01:20	11
P04271	S100	DRB1*01:24	5
P04271	S100	DRB1*01:29	4
P04271	S100	DRB1*04:04	7
P04271	S100	DRB1*04:08	3
P04271	S100	DRB1*04:10	3
P04271	S100	DRB1*10:01	10
P04271	S100	DRB1*11:14	3
P04271	S100	DRB1*13:02	3
P04271	S100	DRB1*13:23	3
P04271	S100	DRB1*13:96	2
P04271	S100	DRB1*13:97	3

P14679	Tyrosinase	DPB1*02:01	12
P14679	Tyrosinase	DPB1*02:02	14
P14679	Tyrosinase	DPB1*04:01	8
P14679	Tyrosinase	DPB1*126:01	8
P14679	Tyrosinase	DPB1*15:01	4
P14679	Tyrosinase	DPB1*23:01	8
P14679	Tyrosinase	DPB1*33:01	36
P14679	Tyrosinase	DPB1*39:01	8
P14679	Tyrosinase	DPB1*46:01	12
P14679	Tyrosinase	DPB1*47:01	14
P14679	Tyrosinase	DPB1*71:01	36
P14679	Tyrosinase	DPB1*72:01	7
P14679	Tyrosinase	DPB1*81:01	12
P14679	Tyrosinase	DRB1*01:01	56
P14679	Tyrosinase	DRB1*01:02	3
P14679	Tyrosinase	DRB1*01:11	15
P14679	Tyrosinase	DRB1*01:18	59
P14679	Tyrosinase	DRB1*01:20	39
P14679	Tyrosinase	DRB1*01:24	24
P14679	Tyrosinase	DRB1*01:29	23
P14679	Tyrosinase	DRB1*03:01	2
P14679	Tyrosinase	DRB1*03:04	2
P14679	Tyrosinase	DRB1*03:11	4
P14679	Tyrosinase	DRB1*03:13	2
P14679	Tyrosinase	DRB1*04:01	4
P14679	Tyrosinase	DRB1*04:05	4
P14679	Tyrosinase	DRB1*04:08	5
P14679	Tyrosinase	DRB1*07:01	3
P14679	Tyrosinase	DRB1*08:01	2
P14679	Tyrosinase	DRB1*08:04	3
P14679	Tyrosinase	DRB1*09:01	2
P14679	Tyrosinase	DRB1*10:01	32
P14679	Tyrosinase	DRB1*11:01	8
P14679	Tyrosinase	DRB1*11:02	8
P14679	Tyrosinase	DRB1*11:03	11
P14679	Tyrosinase	DRB1*11:04	12
P14679	Tyrosinase	DRB1*11:08	4
P14679	Tyrosinase	DRB1*11:10	8
P14679	Tyrosinase	DRB1*11:12	8
P14679	Tyrosinase	DRB1*11:13	6

P14679	Tyrosinase	DRB1*11:14	10
P14679	Tyrosinase	DRB1*11:28	8
P14679	Tyrosinase	DRB1*11:29	8
P14679	Tyrosinase	DRB1*11:37	1
P14679	Tyrosinase	DRB1*11:42	16
P14679	Tyrosinase	DRB1*11:46	12
P14679	Tyrosinase	DRB1*11:49	8
P14679	Tyrosinase	DRB1*11:58	12
P14679	Tyrosinase	DRB1*11:62	8
P14679	Tyrosinase	DRB1*11:65	8
P14679	Tyrosinase	DRB1*11:74	8
P14679	Tyrosinase	DRB1*11:84	1
P14679	Tyrosinase	DRB1*12:16	3
P14679	Tyrosinase	DRB1*13:01	8
P14679	Tyrosinase	DRB1*13:02	10
P14679	Tyrosinase	DRB1*13:05	8
P14679	Tyrosinase	DRB1*13:07	1
P14679	Tyrosinase	DRB1*13:11	12
P14679	Tyrosinase	DRB1*13:14	8
P14679	Tyrosinase	DRB1*13:21	12
P14679	Tyrosinase	DRB1*13:23	10
P14679	Tyrosinase	DRB1*13:50	8
P14679	Tyrosinase	DRB1*13:96	3
P14679	Tyrosinase	DRB1*13:97	10
P14679	Tyrosinase	DRB1*14:32	1
P14679	Tyrosinase	DRB1*15:01	5
P14679	Tyrosinase	DRB1*15:02	1
P14679	Tyrosinase	DRB1*15:06	5
P14679	Tyrosinase	DRB1*15:07	1
P14679	Tyrosinase	DRB1*15:15	5
P14679	Tyrosinase	DRB1*16:01	2
P14679	Tyrosinase	DRB1*16:02	4
P14679	Tyrosinase	DRB1*16:09	6
P17643	TRP1	DPB1*01:01	6
P17643	TRP1	DPB1*02:01	13
P17643	TRP1	DPB1*02:02	13
P17643	TRP1	DPB1*04:01	6
P17643	TRP1	DPB1*04:02	6
P17643	TRP1	DPB1*105:01	6
P17643	TRP1	DPB1*126:01	6

P17643	TRP1	DPB1*15:01	6
P17643	TRP1	DPB1*16:01	9
P17643	TRP1	DPB1*19:01	4
P17643	TRP1	DPB1*23:01	6
P17643	TRP1	DPB1*33:01	24
P17643	TRP1	DPB1*34:01	6
P17643	TRP1	DPB1*39:01	6
P17643	TRP1	DPB1*40:01	6
P17643	TRP1	DPB1*41:01	11
P17643	TRP1	DPB1*46:01	13
P17643	TRP1	DPB1*47:01	13
P17643	TRP1	DPB1*49:01	6
P17643	TRP1	DPB1*55:01	2
P17643	TRP1	DPB1*71:01	24
P17643	TRP1	DPB1*72:01	6
P17643	TRP1	DPB1*81:01	13
P17643	TRP1	DRB1*01:01	36
P17643	TRP1	DRB1*01:02	3
P17643	TRP1	DRB1*01:11	6
P17643	TRP1	DRB1*01:18	45
P17643	TRP1	DRB1*01:20	28
P17643	TRP1	DRB1*01:24	7
P17643	TRP1	DRB1*01:29	8
P17643	TRP1	DRB1*04:01	5
P17643	TRP1	DRB1*04:04	11
P17643	TRP1	DRB1*04:05	6
P17643	TRP1	DRB1*04:08	9
P17643	TRP1	DRB1*04:10	5
P17643	TRP1	DRB1*04:72	1
P17643	TRP1	DRB1*07:01	12
P17643	TRP1	DRB1*08:01	7
P17643	TRP1	DRB1*08:02	2
P17643	TRP1	DRB1*08:04	9
P17643	TRP1	DRB1*08:24	3
P17643	TRP1	DRB1*09:01	7
P17643	TRP1	DRB1*10:01	31
P17643	TRP1	DRB1*11:01	12
P17643	TRP1	DRB1*11:02	17
P17643	TRP1	DRB1*11:03	27
P17643	TRP1	DRB1*11:04	26

P17643	TRP1	DRB1*11:08	5
P17643	TRP1	DRB1*11:10	12
P17643	TRP1	DRB1*11:11	2
P17643	TRP1	DRB1*11:12	12
P17643	TRP1	DRB1*11:13	14
P17643	TRP1	DRB1*11:14	14
P17643	TRP1	DRB1*11:28	12
P17643	TRP1	DRB1*11:29	12
P17643	TRP1	DRB1*11:37	4
P17643	TRP1	DRB1*11:42	35
P17643	TRP1	DRB1*11:46	26
P17643	TRP1	DRB1*11:49	12
P17643	TRP1	DRB1*11:58	26
P17643	TRP1	DRB1*11:62	12
P17643	TRP1	DRB1*11:65	17
P17643	TRP1	DRB1*11:74	12
P17643	TRP1	DRB1*11:84	6
P17643	TRP1	DRB1*13:01	17
P17643	TRP1	DRB1*13:02	14
P17643	TRP1	DRB1*13:05	12
P17643	TRP1	DRB1*13:07	4
P17643	TRP1	DRB1*13:11	26
P17643	TRP1	DRB1*13:14	12
P17643	TRP1	DRB1*13:21	25
P17643	TRP1	DRB1*13:23	14
P17643	TRP1	DRB1*13:50	12
P17643	TRP1	DRB1*13:61	2
P17643	TRP1	DRB1*13:66	2
P17643	TRP1	DRB1*13:96	4
P17643	TRP1	DRB1*13:97	14
P17643	TRP1	DRB1*14:01	6
P17643	TRP1	DRB1*14:04	5
P17643	TRP1	DRB1*14:06	5
P17643	TRP1	DRB1*14:12	1
P17643	TRP1	DRB1*14:32	11
P17643	TRP1	DRB1*14:38	1
P17643	TRP1	DRB1*14:54	6
P17643	TRP1	DRB1*15:01	7
P17643	TRP1	DRB1*15:02	4
P17643	TRP1	DRB1*15:03	5

P17643	TRP1	DRB1*15:06	7
P17643	TRP1	DRB1*15:07	2
P17643	TRP1	DRB1*15:15	8
P17643	TRP1	DRB1*15:37	1
P17643	TRP1	DRB1*16:02	1
P17643	TRP1	DRB1*16:09	3
P40967	PMEL17	DPB1*33:01	6
P40967	PMEL17	DPB1*71:01	6
P40967	PMEL17	DRB1*01:01	62
P40967	PMEL17	DRB1*01:02	17
P40967	PMEL17	DRB1*01:11	29
P40967	PMEL17	DRB1*01:18	66
P40967	PMEL17	DRB1*01:20	73
P40967	PMEL17	DRB1*01:24	37
P40967	PMEL17	DRB1*01:29	36
P40967	PMEL17	DRB1*03:11	4
P40967	PMEL17	DRB1*04:01	6
P40967	PMEL17	DRB1*04:04	8
P40967	PMEL17	DRB1*04:05	9
P40967	PMEL17	DRB1*04:08	7
P40967	PMEL17	DRB1*04:44	1
P40967	PMEL17	DRB1*04:72	2
P40967	PMEL17	DRB1*07:01	32
P40967	PMEL17	DRB1*08:04	7
P40967	PMEL17	DRB1*09:01	9
P40967	PMEL17	DRB1*10:01	41
P40967	PMEL17	DRB1*11:01	17
P40967	PMEL17	DRB1*11:02	22
P40967	PMEL17	DRB1*11:03	28
P40967	PMEL17	DRB1*11:04	26
P40967	PMEL17	DRB1*11:06	2
P40967	PMEL17	DRB1*11:08	10
P40967	PMEL17	DRB1*11:10	17
P40967	PMEL17	DRB1*11:12	17
P40967	PMEL17	DRB1*11:13	15
P40967	PMEL17	DRB1*11:14	32
P40967	PMEL17	DRB1*11:19	3
P40967	PMEL17	DRB1*11:28	17
P40967	PMEL17	DRB1*11:29	17
P40967	PMEL17	DRB1*11:37	3

P40967	PMEL17	DRB1*11:42	28
P40967	PMEL17	DRB1*11:46	26
P40967	PMEL17	DRB1*11:49	17
P40967	PMEL17	DRB1*11:54	1
P40967	PMEL17	DRB1*11:58	26
P40967	PMEL17	DRB1*11:62	17
P40967	PMEL17	DRB1*11:65	22
P40967	PMEL17	DRB1*11:74	17
P40967	PMEL17	DRB1*11:84	6
P40967	PMEL17	DRB1*12:03	3
P40967	PMEL17	DRB1*13:01	22
P40967	PMEL17	DRB1*13:02	32
P40967	PMEL17	DRB1*13:05	17
P40967	PMEL17	DRB1*13:07	3
P40967	PMEL17	DRB1*13:11	26
P40967	PMEL17	DRB1*13:14	17
P40967	PMEL17	DRB1*13:21	12
P40967	PMEL17	DRB1*13:23	32
P40967	PMEL17	DRB1*13:33	1
P40967	PMEL17	DRB1*13:50	17
P40967	PMEL17	DRB1*13:61	3
P40967	PMEL17	DRB1*13:66	5
P40967	PMEL17	DRB1*13:96	19
P40967	PMEL17	DRB1*13:97	32
P40967	PMEL17	DRB1*14:02	4
P40967	PMEL17	DRB1*14:06	9
P40967	PMEL17	DRB1*14:07	2
P40967	PMEL17	DRB1*14:12	2
P40967	PMEL17	DRB1*14:32	11
P40967	PMEL17	DRB1*15:01	9
P40967	PMEL17	DRB1*15:02	2
P40967	PMEL17	DRB1*15:06	9
P40967	PMEL17	DRB1*15:07	2
P40967	PMEL17	DRB1*15:15	3
P40967	PMEL17	DRB1*15:37	1
P40967	PMEL17	DRB1*16:01	4
P40967	PMEL17	DRB1*16:02	4
P40967	PMEL17	DRB1*16:09	4
P43355	MAGE1	DPB1*02:01	7
P43355	MAGE1	DPB1*02:02	10

P43355	MAGE1	DPB1*04:01	1
P43355	MAGE1	DPB1*126:01	1
P43355	MAGE1	DPB1*15:01	6
P43355	MAGE1	DPB1*23:01	1
P43355	MAGE1	DPB1*33:01	27
P43355	MAGE1	DPB1*39:01	1
P43355	MAGE1	DPB1*40:01	1
P43355	MAGE1	DPB1*46:01	7
P43355	MAGE1	DPB1*47:01	10
P43355	MAGE1	DPB1*71:01	27
P43355	MAGE1	DPB1*72:01	1
P43355	MAGE1	DPB1*81:01	7
P43355	MAGE1	DRB1*01:01	46
P43355	MAGE1	DRB1*01:02	3
P43355	MAGE1	DRB1*01:11	16
P43355	MAGE1	DRB1*01:18	59
P43355	MAGE1	DRB1*01:20	53
P43355	MAGE1	DRB1*01:24	19
P43355	MAGE1	DRB1*01:29	22
P43355	MAGE1	DRB1*03:01	3
P43355	MAGE1	DRB1*03:04	3
P43355	MAGE1	DRB1*03:11	6
P43355	MAGE1	DRB1*03:13	3
P43355	MAGE1	DRB1*03:15	5
P43355	MAGE1	DRB1*07:01	11
P43355	MAGE1	DRB1*08:02	1
P43355	MAGE1	DRB1*08:04	6
P43355	MAGE1	DRB1*08:24	2
P43355	MAGE1	DRB1*08:30	3
P43355	MAGE1	DRB1*09:01	6
P43355	MAGE1	DRB1*10:01	23
P43355	MAGE1	DRB1*11:01	23
P43355	MAGE1	DRB1*11:02	16
P43355	MAGE1	DRB1*11:03	17
P43355	MAGE1	DRB1*11:04	22
P43355	MAGE1	DRB1*11:07	2
P43355	MAGE1	DRB1*11:08	13
P43355	MAGE1	DRB1*11:10	23
P43355	MAGE1	DRB1*11:12	23
P43355	MAGE1	DRB1*11:13	20

P43355	MAGE1	DRB1*11:14	5
P43355	MAGE1	DRB1*11:19	3
P43355	MAGE1	DRB1*11:27	2
P43355	MAGE1	DRB1*11:28	23
P43355	MAGE1	DRB1*11:29	23
P43355	MAGE1	DRB1*11:37	4
P43355	MAGE1	DRB1*11:42	28
P43355	MAGE1	DRB1*11:46	22
P43355	MAGE1	DRB1*11:49	23
P43355	MAGE1	DRB1*11:58	22
P43355	MAGE1	DRB1*11:62	23
P43355	MAGE1	DRB1*11:65	16
P43355	MAGE1	DRB1*11:74	23
P43355	MAGE1	DRB1*11:84	9
P43355	MAGE1	DRB1*12:03	8
P43355	MAGE1	DRB1*12:16	7
P43355	MAGE1	DRB1*13:01	16
P43355	MAGE1	DRB1*13:02	5
P43355	MAGE1	DRB1*13:05	23
P43355	MAGE1	DRB1*13:07	4
P43355	MAGE1	DRB1*13:11	22
P43355	MAGE1	DRB1*13:14	23
P43355	MAGE1	DRB1*13:21	25
P43355	MAGE1	DRB1*13:23	5
P43355	MAGE1	DRB1*13:50	23
P43355	MAGE1	DRB1*13:61	6
P43355	MAGE1	DRB1*13:66	1
P43355	MAGE1	DRB1*13:96	4
P43355	MAGE1	DRB1*13:97	5
P43355	MAGE1	DRB1*14:01	6
P43355	MAGE1	DRB1*14:04	5
P43355	MAGE1	DRB1*14:05	4
P43355	MAGE1	DRB1*14:06	6
P43355	MAGE1	DRB1*14:12	5
P43355	MAGE1	DRB1*14:23	4
P43355	MAGE1	DRB1*14:32	10
P43355	MAGE1	DRB1*14:38	5
P43355	MAGE1	DRB1*14:54	6
P43355	MAGE1	DRB1*15:01	8
P43355	MAGE1	DRB1*15:02	2

P43355	MAGE1	DRB1*15:03	3
P43355	MAGE1	DRB1*15:06	8
P43355	MAGE1	DRB1*15:15	12
P43355	MAGE1	DRB1*16:01	9
P43355	MAGE1	DRB1*16:02	13
P43355	MAGE1	DRB1*16:04	5
P43355	MAGE1	DRB1*16:05	3
P43355	MAGE1	DRB1*16:09	14
P43358	MAGE4	DPB1*33:01	6
P43358	MAGE4	DPB1*71:01	6
P43358	MAGE4	DRB1*01:01	31
P43358	MAGE4	DRB1*01:02	5
P43358	MAGE4	DRB1*01:11	15
P43358	MAGE4	DRB1*01:18	41
P43358	MAGE4	DRB1*01:20	38
P43358	MAGE4	DRB1*01:24	16
P43358	MAGE4	DRB1*01:29	16
P43358	MAGE4	DRB1*03:11	8
P43358	MAGE4	DRB1*04:01	1
P43358	MAGE4	DRB1*07:01	7
P43358	MAGE4	DRB1*08:04	5
P43358	MAGE4	DRB1*09:01	6
P43358	MAGE4	DRB1*10:01	12
P43358	MAGE4	DRB1*11:01	12
P43358	MAGE4	DRB1*11:02	21
P43358	MAGE4	DRB1*11:03	25
P43358	MAGE4	DRB1*11:04	18
P43358	MAGE4	DRB1*11:08	3
P43358	MAGE4	DRB1*11:10	12
P43358	MAGE4	DRB1*11:11	2
P43358	MAGE4	DRB1*11:12	12
P43358	MAGE4	DRB1*11:13	17
P43358	MAGE4	DRB1*11:14	7
P43358	MAGE4	DRB1*11:28	12
P43358	MAGE4	DRB1*11:29	12
P43358	MAGE4	DRB1*11:42	31
P43358	MAGE4	DRB1*11:46	18
P43358	MAGE4	DRB1*11:49	12
P43358	MAGE4	DRB1*11:58	18
P43358	MAGE4	DRB1*11:62	12

P43358	MAGE4	DRB1*11:65	21
P43358	MAGE4	DRB1*11:74	12
P43358	MAGE4	DRB1*11:84	4
P43358	MAGE4	DRB1*12:02	2
P43358	MAGE4	DRB1*13:01	21
P43358	MAGE4	DRB1*13:02	7
P43358	MAGE4	DRB1*13:05	12
P43358	MAGE4	DRB1*13:11	18
P43358	MAGE4	DRB1*13:14	12
P43358	MAGE4	DRB1*13:21	9
P43358	MAGE4	DRB1*13:23	7
P43358	MAGE4	DRB1*13:50	12
P43358	MAGE4	DRB1*13:61	6
P43358	MAGE4	DRB1*13:96	6
P43358	MAGE4	DRB1*13:97	7
P43358	MAGE4	DRB1*14:06	9
P43358	MAGE4	DRB1*14:12	4
P43358	MAGE4	DRB1*14:32	7
P43358	MAGE4	DRB1*15:01	8
P43358	MAGE4	DRB1*15:02	1
P43358	MAGE4	DRB1*15:03	6
P43358	MAGE4	DRB1*15:06	8
P43358	MAGE4	DRB1*15:07	4
P43358	MAGE4	DRB1*15:15	10
P43358	MAGE4	DRB1*15:37	4
P43358	MAGE4	DRB1*16:01	6
P43358	MAGE4	DRB1*16:02	9
P43358	MAGE4	DRB1*16:05	2
P43358	MAGE4	DRB1*16:09	4
P78358	NY-ESO-1	DPB1*01:01	8
P78358	NY-ESO-1	DPB1*02:01	7
P78358	NY-ESO-1	DPB1*02:02	7
P78358	NY-ESO-1	DPB1*04:01	7
P78358	NY-ESO-1	DPB1*126:01	7
P78358	NY-ESO-1	DPB1*15:01	10
P78358	NY-ESO-1	DPB1*23:01	7
P78358	NY-ESO-1	DPB1*33:01	16
P78358	NY-ESO-1	DPB1*39:01	7
P78358	NY-ESO-1	DPB1*46:01	7
P78358	NY-ESO-1	DPB1*47:01	7

P78358	NY-ESO-1	DPB1*71:01	16
P78358	NY-ESO-1	DPB1*72:01	7
P78358	NY-ESO-1	DPB1*81:01	7
P78358	NY-ESO-1	DRB1*01:01	18
P78358	NY-ESO-1	DRB1*01:02	2
P78358	NY-ESO-1	DRB1*01:11	11
P78358	NY-ESO-1	DRB1*01:18	18
P78358	NY-ESO-1	DRB1*01:20	22
P78358	NY-ESO-1	DRB1*01:24	13
P78358	NY-ESO-1	DRB1*01:29	12
P78358	NY-ESO-1	DRB1*03:11	3
P78358	NY-ESO-1	DRB1*04:01	4
P78358	NY-ESO-1	DRB1*04:08	4
P78358	NY-ESO-1	DRB1*04:72	3
P78358	NY-ESO-1	DRB1*07:01	12
P78358	NY-ESO-1	DRB1*09:01	8
P78358	NY-ESO-1	DRB1*10:01	14
P78358	NY-ESO-1	DRB1*11:02	2
P78358	NY-ESO-1	DRB1*11:04	1
P78358	NY-ESO-1	DRB1*11:13	5
P78358	NY-ESO-1	DRB1*11:14	6
P78358	NY-ESO-1	DRB1*11:42	6
P78358	NY-ESO-1	DRB1*11:46	1
P78358	NY-ESO-1	DRB1*11:58	1
P78358	NY-ESO-1	DRB1*11:65	2
P78358	NY-ESO-1	DRB1*13:01	2
P78358	NY-ESO-1	DRB1*13:02	6
P78358	NY-ESO-1	DRB1*13:11	1
P78358	NY-ESO-1	DRB1*13:23	6
P78358	NY-ESO-1	DRB1*13:96	2
P78358	NY-ESO-1	DRB1*13:97	6
P78358	NY-ESO-1	DRB1*14:06	1
P78358	NY-ESO-1	DRB1*14:32	2
P78358	NY-ESO-1	DRB1*15:01	4
P78358	NY-ESO-1	DRB1*15:03	1
P78358	NY-ESO-1	DRB1*15:06	4
P78358	NY-ESO-1	DRB1*15:37	1
P78358	NY-ESO-1	DRB1*16:01	3
P78358	NY-ESO-1	DRB1*16:02	3
P78358	NY-ESO-1	DRB1*16:05	1

P78358	NY-ESO-1	DRB1*16:09	2
Q13072	BAGE	DPB1*33:01	5
Q13072	BAGE	DPB1*71:01	5
Q13072	BAGE	DRB1*01:01	11
Q13072	BAGE	DRB1*01:02	8
Q13072	BAGE	DRB1*01:03	5
Q13072	BAGE	DRB1*01:11	7
Q13072	BAGE	DRB1*01:18	11
Q13072	BAGE	DRB1*01:20	13
Q13072	BAGE	DRB1*01:24	7
Q13072	BAGE	DRB1*01:29	9
Q13072	BAGE	DRB1*04:08	1
Q13072	BAGE	DRB1*07:01	6
Q13072	BAGE	DRB1*08:30	1
Q13072	BAGE	DRB1*09:01	3
Q13072	BAGE	DRB1*10:01	7
Q13072	BAGE	DRB1*11:01	1
Q13072	BAGE	DRB1*11:08	2
Q13072	BAGE	DRB1*11:10	1
Q13072	BAGE	DRB1*11:12	1
Q13072	BAGE	DRB1*11:13	2
Q13072	BAGE	DRB1*11:14	1
Q13072	BAGE	DRB1*11:19	1
Q13072	BAGE	DRB1*11:28	1
Q13072	BAGE	DRB1*11:29	1
Q13072	BAGE	DRB1*11:42	1
Q13072	BAGE	DRB1*11:49	1
Q13072	BAGE	DRB1*11:62	1
Q13072	BAGE	DRB1*11:74	1
Q13072	BAGE	DRB1*12:16	3
Q13072	BAGE	DRB1*13:02	1
Q13072	BAGE	DRB1*13:05	1
Q13072	BAGE	DRB1*13:14	1
Q13072	BAGE	DRB1*13:23	1
Q13072	BAGE	DRB1*13:50	1
Q13072	BAGE	DRB1*13:97	1
Q13072	BAGE	DRB1*14:32	2
Q13072	BAGE	DRB1*15:01	3
Q13072	BAGE	DRB1*15:02	6
Q13072	BAGE	DRB1*15:03	1

Q13072	BAGE	DRB1*15:06	3
Q13072	BAGE	DRB1*15:07	1
Q13072	BAGE	DRB1*15:15	6
Q13072	BAGE	DRB1*16:01	6
Q13072	BAGE	DRB1*16:02	6
Q13072	BAGE	DRB1*16:04	5
Q13072	BAGE	DRB1*16:05	6
Q13072	BAGE	DRB1*16:09	6
Q16385	GAGE	DPB1*33:01	1
Q16385	GAGE	DPB1*71:01	1
Q16385	GAGE	DRB1*01:01	12
Q16385	GAGE	DRB1*01:11	3
Q16385	GAGE	DRB1*01:18	12
Q16385	GAGE	DRB1*01:20	8
Q16385	GAGE	DRB1*01:24	7
Q16385	GAGE	DRB1*01:29	6
Q16385	GAGE	DRB1*03:11	3
Q16385	GAGE	DRB1*07:01	6
Q16385	GAGE	DRB1*08:01	4
Q16385	GAGE	DRB1*08:04	3
Q16385	GAGE	DRB1*08:24	2
Q16385	GAGE	DRB1*09:01	5
Q16385	GAGE	DRB1*10:01	11
Q16385	GAGE	DRB1*11:01	8
Q16385	GAGE	DRB1*11:02	6
Q16385	GAGE	DRB1*11:03	8
Q16385	GAGE	DRB1*11:04	7
Q16385	GAGE	DRB1*11:08	4
Q16385	GAGE	DRB1*11:10	8
Q16385	GAGE	DRB1*11:11	5
Q16385	GAGE	DRB1*11:12	8
Q16385	GAGE	DRB1*11:13	8
Q16385	GAGE	DRB1*11:27	2
Q16385	GAGE	DRB1*11:28	8
Q16385	GAGE	DRB1*11:29	8
Q16385	GAGE	DRB1*11:37	4
Q16385	GAGE	DRB1*11:42	10
Q16385	GAGE	DRB1*11:46	7
Q16385	GAGE	DRB1*11:49	8
Q16385	GAGE	DRB1*11:54	1

Q16385	GAGE	DRB1*11:58	7
Q16385	GAGE	DRB1*11:62	8
Q16385	GAGE	DRB1*11:65	6
Q16385	GAGE	DRB1*11:74	8
Q16385	GAGE	DRB1*11:84	4
Q16385	GAGE	DRB1*13:01	6
Q16385	GAGE	DRB1*13:05	8
Q16385	GAGE	DRB1*13:07	4
Q16385	GAGE	DRB1*13:11	7
Q16385	GAGE	DRB1*13:14	8
Q16385	GAGE	DRB1*13:21	11
Q16385	GAGE	DRB1*13:50	8
Q16385	GAGE	DRB1*14:01	1
Q16385	GAGE	DRB1*14:32	4
Q16385	GAGE	DRB1*14:54	1
Q16385	GAGE	DRB1*16:02	1
Q16655	MELAN	DRB1*01:01	6
Q16655	MELAN	DRB1*01:18	5
Q16655	MELAN	DRB1*01:24	2
Q16655	MELAN	DRB1*01:29	2
Q16655	MELAN	DRB1*03:11	2
Q16655	MELAN	DRB1*10:01	2

Table S8. Counts (N) and percentages (%) of hits across all 11 antigens tested. Number of alleles = 117 alleles. Total number of hits = 5941. Percent (%) = 100N/5941.

	Allele	N	%
1	DRB1*01:18	352	5.925
2	DRB1*01:01	303	5.100
3	DRB1*01:20	292	4.915
4	DRB1*10:01	190	3.198
5	DRB1*11:42	163	2.740
6	DPB1*33:01	145	2.441
7	DPB1*71:01	145	2.441
8	DRB1*01:29	145	2.441
9	DRB1*01:24	144	2.424
10	DRB1*11:03	123	2.070
11	DRB1*11:04	119	2.003
12	DRB1*11:46	119	2.000
13	DRB1*11:58	119	2.000
14	DRB1*13:11	119	2.000
15	DRB1*01:11	112	1.885
16	DRB1*07:01	95	1.599
17	DRB1*11:02	95	1.599
18	DRB1*11:65	95	1.600
19	DRB1*13:01	95	1.600
20	DRB1*13:21	94	1.580
21	DRB1*11:13	89	1.500
22	DRB1*11:01	86	1.448
23	DRB1*11:10	86	1.450
24	DRB1*11:12	86	1.450
25	DRB1*11:28	86	1.450
26	DRB1*11:29	86	1.450
27	DRB1*11:49	86	1.450
28	DRB1*11:62	86	1.450
29	DRB1*11:74	86	1.450
30	DRB1*13:05	86	1.450
31	DRB1*13:14	86	1.450
32	DRB1*13:50	86	1.448
33	DRB1*11:14	81	1.360
34	DRB1*13:02	81	1.360
35	DRB1*13:23	81	1.363
36	DRB1*13:97	81	1.363
37	DPB1*02:02	51	0.858

38	DPB1*47:01	51	0.858
39	DRB1*15:15	50	0.842
40	DRB1*14:32	48	0.808
41	DRB1*15:01	47	0.791
42	DRB1*15:06	47	0.791
43	DRB1*16:02	47	0.791
44	DRB1*09:01	46	0.774
45	DPB1*02:01	45	0.757
46	DPB1*46:01	45	0.757
47	DPB1*81:01	45	0.757
48	DRB1*16:09	45	0.757
49	DRB1*11:08	42	0.707
50	DRB1*01:02	41	0.690
51	DRB1*13:96	40	0.673
52	DRB1*03:11	36	0.606
53	DRB1*08:04	36	0.606
54	DRB1*16:01	35	0.589
55	DRB1*11:84	31	0.520
56	DRB1*14:06	31	0.522
57	DPB1*15:01	30	0.505
58	DRB1*04:08	29	0.488
59	DPB1*04:01	27	0.454
60	DPB1*126:0	27	0.454
61	DPB1*23:01	27	0.454
62	DPB1*39:01	27	0.454
63	DRB1*04:04	26	0.438
64	DPB1*72:01	25	0.421
65	DRB1*15:02	21	0.353
66	DRB1*04:01	20	0.337
67	DRB1*04:05	19	0.320
68	DRB1*16:05	18	0.303
69	DRB1*13:61	17	0.286
70	DRB1*11:37	16	0.270
71	DRB1*13:07	16	0.270
72	DRB1*15:03	16	0.269
73	DPB1*01:01	15	0.252
74	DRB1*08:01	13	0.219
75	DRB1*12:16	13	0.220
76	DRB1*14:01	13	0.219
77	DRB1*14:54	13	0.219

78	DRB1*16:04	13	0.219
79	DRB1*14:12	12	0.202
80	DPB1*41:01	11	0.185
81	DRB1*12:03	11	0.190
82	DRB1*14:04	10	0.168
83	DRB1*15:07	10	0.168
84	DPB1*16:01	9	0.151
85	DRB1*11:11	9	0.150
86	DRB1*04:10	8	0.135
87	DRB1*13:66	8	0.135
88	DPB1*40:01	7	0.118
89	DRB1*08:24	7	0.118
90	DRB1*11:19	7	0.120
91	DRB1*15:37	7	0.118
92	DPB1*04:02	6	0.101
93	DPB1*105:0	6	0.101
94	DPB1*34:01	6	0.101
95	DPB1*49:01	6	0.101
96	DRB1*04:72	6	0.101
97	DRB1*14:38	6	0.101
98	DRB1*01:03	5	0.084
99	DRB1*03:01	5	0.084
100	DRB1*03:04	5	0.084
101	DRB1*03:13	5	0.084
102	DRB1*03:15	5	0.084
103	DPB1*19:01	4	0.067
104	DRB1*08:30	4	0.067
105	DRB1*11:27	4	0.070
106	DRB1*14:02	4	0.067
107	DRB1*14:05	4	0.067
108	DRB1*14:23	4	0.067
109	DRB1*08:02	3	0.050
110	DPB1*55:01	2	0.034
111	DRB1*11:06	2	0.034
112	DRB1*11:07	2	0.034
113	DRB1*11:54	2	0.030
114	DRB1*12:02	2	0.030
115	DRB1*14:07	2	0.034
116	DRB1*04:44	1	0.017
117	DRB1*13:33	1	0.017

Table S9. Relative frequencies of the alleles binding strongly to peptides, across the 7 ethnic population groups. AFA (African/African American), API (Asian/Pacific Islands), EURO (European/European descent), MENA (Middle East/North Coast of Africa), HIS (South or Central America/Hispanic/Latino), NAM (Native American populations) and UNK (unknown/not asked/multiple ancestries/other). Total is the overall population i.e., all groups combined. Data are from [52].

	Allele	AFA	API	EURO	MENA	HIS	NAM	UNK	Total counts
1	DPB1*01:01	0.12729	0.05721	0.68094	0.00520	0.03929	0.00589	0.08418	755389
2	DPB1*02:01	0.02179	0.10976	0.73299	0.02865	0.03479	0.00385	0.06817	2042484
3	DPB1*02:02	0.00839	0.22479	0.58460	0.00667	0.07592	0.00554	0.09410	71863
4	DPB1*04:01	0.00665	0.05499	0.82035	0.02133	0.02897	0.00293	0.06478	5524116
5	DPB1*04:02	0.02434	0.03539	0.74822	0.02411	0.08985	0.00764	0.07045	1791265
6	DPB1*105:01	0.41455	0.02730	0.27608	0.02438	0.11616	0.01900	0.12253	29046
7	DPB1*126:01	0.00571	0.01910	0.83503	0.07641	0.04292	0.00273	0.01811	4031
8	DPB1*15:01	0.01823	0.06718	0.78955	0.02488	0.02778	0.00266	0.06971	101323
9	DPB1*16:01	0.00578	0.06799	0.83606	0.00498	0.02133	0.00229	0.06157	73041
10	DPB1*19:01	0.01116	0.03518	0.85079	0.00721	0.02674	0.00319	0.06573	86386
11	DPB1*23:01	0.00334	0.01239	0.89421	0.02311	0.02030	0.00171	0.04495	94258
12	DPB1*33:01	0.00384	0.12860	0.76392	0.02879	0.02495	0.00192	0.04798	521
13	DPB1*34:01	0.08400	0.02598	0.68500	0.05820	0.04983	0.00481	0.09219	5619
14	DPB1*39:01	0.67751	0.01071	0.05437	0.00659	0.08526	0.01153	0.15404	2428
15	DPB1*40:01	0.70481	0.00117	0.02539	0.00317	0.08169	0.01520	0.16856	5986
16	DPB1*41:01	0.00199	0.37188	0.47557	0.03290	0.01795	0.00199	0.09771	1003
17	DPB1*46:01	0.06766	0.30046	0.54989	0.01835	0.01433	0.00229	0.04702	1744
18	DPB1*47:01	0.03529	0.14771	0.56209	0.17124	0.00000	0.00000	0.08366	765
19	DPB1*49:01	0.22917	0.01442	0.10897	0.01603	0.49199	0.02404	0.11538	624
20	DPB1*55:01	0.61483	0.02712	0.10850	0.02170	0.11212	0.00181	0.11392	553
21	DPB1*71:01	0.02875	0.10144	0.73083	0.03435	0.03514	0.00240	0.06709	1252
22	DPB1*72:01	0.03810	0.20000	0.56190	0.08571	0.01905	0.00000	0.09524	105
23	DPB1*81:01	0.00597	0.06826	0.80034	0.05034	0.01962	0.00256	0.05290	1172
24	DRB1*01:01	0.00748	0.03041	0.86108	0.01046	0.02440	0.00251	0.06365	1257941
25	DRB1*01:02	0.07315	0.00923	0.62704	0.04355	0.10071	0.00767	0.13865	218126
26	DRB1*01:03	0.01092	0.00223	0.79875	0.00273	0.06429	0.00514	0.11594	83126
27	DRB1*01:11	0.12500	0.00000	0.75000	0.00000	0.04167	0.00000	0.08333	24
28	DRB1*01:18	0.00000	0.00000	1.00000	0.00000	0.00000	0.00000	0.00000	17
29	DRB1*01:20	0.00000	0.08333	0.83333	0.00000	0.00000	0.00000	0.08333	24
30	DRB1*01:24	0.00000	0.00000	1.00000	0.00000	0.00000	0.00000	0.00000	14
31	DRB1*01:29	0.00000	0.00000	1.00000	0.00000	0.00000	0.00000	0.00000	5
32	DRB1*03:01	0.01597	0.04991	0.80381	0.02328	0.02905	0.00287	0.07512	1566657
33	DRB1*03:04	0.00185	0.00508	0.94134	0.00185	0.01570	0.00046	0.03372	2165
34	DRB1*03:11	0.00000	0.00000	0.53333	0.13333	0.20000	0.00000	0.13333	15
35	DRB1*03:13	0.00621	0.00000	0.90683	0.00000	0.02484	0.01242	0.04969	161

36	DRB1*03:15	0.02432	0.01520	0.41945	0.01216	0.42249	0.02736	0.07903	329
37	DRB1*04:01	0.00673	0.01474	0.88444	0.00571	0.01405	0.00226	0.07207	997223
38	DRB1*04:04	0.00765	0.03720	0.76639	0.01587	0.07360	0.00567	0.09363	420915
39	DRB1*04:05	0.00538	0.05217	0.83288	0.01746	0.02439	0.00179	0.06592	66336
40	DRB1*04:08	0.01254	0.48624	0.12068	0.03872	0.21140	0.02094	0.10947	8212
41	DRB1*04:10	0.02206	0.00673	0.08692	0.00174	0.65215	0.07525	0.15516	21262
42	DRB1*04:44	0.00000	0.10000	0.90000	0.00000	0.00000	0.00000	0.00000	10
43	DRB1*04:72	0.00000	0.00000	0.84058	0.00000	0.01449	0.00000	0.14493	69
44	DRB1*07:01	0.01916	0.08778	0.75330	0.02221	0.03533	0.00346	0.07877	2025356
45	DRB1*08:01	0.00385	0.00926	0.88561	0.00700	0.02198	0.00216	0.07013	352415
46	DRB1*08:02	0.00847	0.09208	0.15205	0.00503	0.55427	0.03387	0.15424	87404
47	DRB1*08:04	0.32148	0.01555	0.40686	0.04291	0.08601	0.01151	0.11568	64840
48	DRB1*08:24	0.00000	0.00000	0.50000	0.25000	0.25000	0.00000	0.00000	4
49	DRB1*08:30	0.00000	0.50000	0.00000	0.00000	0.00000	0.00000	0.50000	2
50	DRB1*09:01	0.05211	0.23641	0.54872	0.01413	0.04427	0.00564	0.09872	199400
51	DRB1*10:01	0.03447	0.31078	0.46135	0.05443	0.04459	0.00547	0.08891	225172
52	DRB1*11:01	0.02754	0.06142	0.78232	0.02827	0.02518	0.00256	0.07272	1056770
53	DRB1*11:02	0.23015	0.00349	0.48504	0.02092	0.12693	0.01626	0.11721	61946
54	DRB1*11:03	0.00247	0.00449	0.88409	0.02333	0.02581	0.00155	0.05826	120462
55	DRB1*11:04	0.00591	0.02166	0.74949	0.07158	0.03644	0.00261	0.11232	563051
56	DRB1*11:06	0.00552	0.86713	0.06067	0.01203	0.00301	0.00877	0.04287	3989
57	DRB1*11:07	0.05941	0.00000	0.72277	0.00000	0.07921	0.00990	0.12871	101
58	DRB1*11:08	0.00590	0.72345	0.22566	0.01622	0.00074	0.00369	0.02434	1356
59	DRB1*11:10	0.73607	0.00147	0.04399	0.00147	0.05572	0.00587	0.15543	682
60	DRB1*11:11	0.00606	0.50187	0.35028	0.01866	0.00840	0.00187	0.11287	2144
61	DRB1*11:12	0.00271	0.03742	0.79035	0.11542	0.01353	0.00045	0.04013	2218
62	DRB1*11:13	0.00520	0.00312	0.89189	0.03534	0.02599	0.00000	0.03846	962
63	DRB1*11:14	0.03437	0.00503	0.81811	0.00251	0.05197	0.00503	0.08298	1193
64	DRB1*11:19	0.00267	0.00933	0.87067	0.00267	0.06133	0.00133	0.05200	750
65	DRB1*11:27	0.00202	0.01210	0.93548	0.00202	0.02419	0.00000	0.02419	496
66	DRB1*11:28	0.00916	0.03664	0.89160	0.00763	0.01527	0.00000	0.03969	655
67	DRB1*11:29	0.01247	0.08728	0.82793	0.02494	0.00998	0.00000	0.03741	401
68	DRB1*11:37	0.01053	0.07368	0.84211	0.02105	0.01053	0.00000	0.04211	95
69	DRB1*11:42	0.00000	0.00000	0.60870	0.14130	0.09783	0.00000	0.15217	92
70	DRB1*11:46	0.00000	0.00000	0.50000	0.00000	0.50000	0.00000	0.00000	2
71	DRB1*11:49	0.08696	0.04348	0.78261	0.00000	0.00000	0.00000	0.08696	23
72	DRB1*11:54	0.00000	0.33333	0.33333	0.00000	0.00000	0.00000	0.33333	3
73	DRB1*11:58	0.00000	0.00000	0.89091	0.05455	0.00000	0.00000	0.05455	55
74	DRB1*11:62	0.00000	0.71429	0.28571	0.00000	0.00000	0.00000	0.00000	14
75	DRB1*11:65	0.75000	0.00000	0.00000	0.00000	0.00000	0.00000	0.25000	12
76	DRB1*11:74	0.00000	0.00000	1.00000	0.00000	0.00000	0.00000	0.00000	1
77	DRB1*11:84	0.00000	0.00000	0.61765	0.00000	0.26471	0.00000	0.11765	34
78	DRB1*12:02	0.01211	0.85610	0.03797	0.00797	0.00859	0.00165	0.07561	82560

79	DRB1*12:03	0.00000	0.00613	0.76687	0.00000	0.00000	0.00613	0.22086	163
80	DRB1*12:16	0.00000	1.00000	0.00000	0.00000	0.00000	0.00000	0.00000	1
81	DRB1*13:01	0.02067	0.06838	0.78492	0.02042	0.03064	0.00331	0.07166	993642
82	DRB1*13:02	0.04008	0.06933	0.72399	0.02697	0.03871	0.00421	0.09670	632158
83	DRB1*13:05	0.00476	0.00433	0.67712	0.05590	0.06918	0.00351	0.18520	35081
84	DRB1*13:07	0.00746	0.59701	0.11940	0.20896	0.00000	0.00000	0.06716	134
85	DRB1*13:11	0.00546	0.08197	0.56557	0.06831	0.15847	0.01093	0.10929	366
86	DRB1*13:14	0.06383	0.05167	0.72036	0.07295	0.03647	0.00000	0.05471	329
87	DRB1*13:21	0.00000	0.00000	0.90843	0.04337	0.01446	0.00000	0.03373	415
88	DRB1*13:23	0.16667	0.00000	0.16667	0.00000	0.16667	0.00000	0.50000	6
89	DRB1*13:33	0.00000	0.00000	0.96774	0.00000	0.00000	0.00000	0.03226	31
90	DRB1*13:50	0.18182	0.22727	0.27273	0.09091	0.00000	0.00000	0.22727	22
91	DRB1*13:61	0.00000	0.06944	0.90278	0.00000	0.01389	0.00000	0.01389	72
92	DRB1*13:66	0.20000	0.00000	0.40000	0.40000	0.00000	0.00000	0.00000	5
93	DRB1*13:96	0.07692	0.23077	0.69231	0.00000	0.00000	0.00000	0.00000	13
94	DRB1*13:97	0.20000	0.20000	0.40000	0.20000	0.00000	0.00000	0.00000	5
95	DRB1*14:01	0.01720	0.05188	0.76219	0.04284	0.03188	0.00292	0.09110	386529
96	DRB1*14:02	0.01571	0.02845	0.15786	0.00239	0.52827	0.07052	0.19681	27240
97	DRB1*14:04	0.00296	0.82575	0.11257	0.02111	0.00515	0.00241	0.03005	100454
98	DRB1*14:05	0.00117	0.78490	0.08021	0.02917	0.00273	0.00026	0.10156	7680
99	DRB1*14:06	0.00811	0.03570	0.07871	0.01423	0.69667	0.03618	0.13040	26641
100	DRB1*14:07	0.00499	0.26508	0.45826	0.07737	0.11356	0.00434	0.07640	6217
101	DRB1*14:12	0.00000	0.26689	0.28041	0.39865	0.01014	0.00000	0.04392	296
102	DRB1*14:23	0.01042	0.04167	0.80208	0.04167	0.03125	0.01042	0.06250	96
103	DRB1*14:32	0.00000	0.00000	0.66667	0.08333	0.00000	0.00000	0.25000	12
104	DRB1*14:38	0.00000	0.00000	0.25000	0.75000	0.00000	0.00000	0.00000	4
105	DRB1*14:54								N/A
106	DRB1*15:01	0.00534	0.07031	0.81935	0.01301	0.02256	0.00220	0.06724	1839872
107	DRB1*15:02	0.00401	0.47434	0.34897	0.04867	0.02750	0.00285	0.09365	284679
108	DRB1*15:03	0.64205	0.00458	0.06162	0.02701	0.09568	0.01815	0.15092	70079
109	DRB1*15:06	0.00123	0.92168	0.02204	0.02930	0.00254	0.00203	0.02117	13790
110	DRB1*15:07	0.02646	0.03704	0.79365	0.06349	0.01587	0.00000	0.06349	189
111	DRB1*15:15	0.00000	0.50000	0.50000	0.00000	0.00000	0.00000	0.00000	10
112	DRB1*15:37	0.00000	0.00000	0.96296	0.03704	0.00000	0.00000	0.00000	27
113	DRB1*16:01	0.00255	0.00407	0.88760	0.03743	0.01898	0.00159	0.04779	368934
114	DRB1*16:02	0.07278	0.20915	0.32862	0.05262	0.20262	0.01845	0.11577	73775
115	DRB1*16:04	0.00000	0.00000	0.79365	0.01587	0.01587	0.00000	0.17460	63
116	DRB1*16:05	0.00000	0.01963	0.72382	0.22251	0.00524	0.00000	0.02880	764
117	DRB1*16:09	0.00000	0.01250	0.86250	0.07500	0.02500	0.00000	0.02500	80
	Mean	0.06627	0.13629	0.58743	0.04617	0.07113	0.00530	0.08741	(1.000)
